# Multilevel sex-influenced neurobiological signatures of early life adversity

**DOI:** 10.1073/pnas.2603982123

**Published:** 2026-08-27

**Authors:** Sowmya Narayan, Christina Beer, Veronika Kovarova, Carlo Castoldi, Tibor Stark, Beatrice Dal Bianco, Simone Röh, Susann Sauer, Joeri Bordes, Stoyo Karamihalev, Shiladitya Mitra, Patricia Maidana Miguel, Bonnie Alberry, Darina Czamara, Patricia P. Silveira, Michael Czisch, Bianca Silva, Elisabeth B. Binder, Mathias V. Schmidt

**Affiliations:** ^a^ Research Group Neurobiology of Stress Resilience, Max Planck Institute of Psychiatry, Munich 80804, Germany; ^b^ Department Genes and Environment, Max Planck Institute of Psychiatry, Munich 80804, Germany; ^c^International Max Planck Research School for Translational Psychiatry, Munich 80804, Germany; ^d^ Neural Circuits of Emotional Memory, CNRS UMR7275, INSERM U1318, Institute of Molecular and Cellular Pharmacology, Université Côte d’Azur, Valbonne P789+P5, France; ^e^ Neuroimaging Core Unit, Max Planck Institute of Psychiatry, Munich 80804, Germany; ^f^ Emotion Research Department, Max Planck Institute of Psychiatry, Munich 80804, Germany; ^g^ Douglas Research Centre, McGill University, Montreal, QC CCV8+G2, Canada

**Keywords:** early life stress, sex differences, resilience, transcriptomics, behavioral phenotyping

## Abstract

Stress exposure early in life is an established risk factor for adult psychiatric illness, yet these disorders—including anxiety disorders and depression—show significant sex differences in prevalence, symptomatology, and treatment response. The biology underlying these differences remains largely unexplored and may contribute to the clinical heterogeneity in anxiety and depression. Here, we characterize the lasting impact of developmental stress on adulthood neurobiology and behavior in mice by combining analyses of multiple levels of brain function, including whole-brain c-Fos mapping, manganese-enhanced MRI, and transcriptomics with advanced behavioral phenotyping. Across levels of investigation, we find distinct and often opposite effects of developmental stress based on sex. These results together showcase the strong influence of sex on how early life adversity affects the onset of stress-related disorders. This work emphasizes the necessity of considering sex when investigating developmental and neurobiological underpinnings of stress-related disorders and displays a vast range of lasting effects of developmental stress on the brain, which provides a valuable resource for future studies aiming to improve psychiatric treatments.

## Significance

Early life adversity is a risk factor for psychiatric disorders, yet the biological mechanisms driving these conditions-and their distinct prevalence in men and women-remain poorly understood. By integrating robust behavioral phenotyping, whole-brain mapping combining MRI and cFos, and transcriptomics in a translational mouse model, we demonstrate that developmental stress induces divergent neurobiological signatures in males and females. These findings reveal that sex plays a fundamental role in how early life stress interacts with the developing brain. This multilevel resource provides a framework for human comparison, especially through the use of cross-species modalities as MRI or deep behavioral phenotyping, and for developing hypotheses for future investigations into sex-specific biomarkers and mechanisms, promoting improved, targeted therapeutic interventions for stress-related mental illness.

Stress exposure during neurodevelopmental time periods is an established risk factor for psychiatric disorders, such as depression and anxiety disorders (1–3). Exposure to prenatal and early life stress are unfortunately commonplace, and psychopathology is seen to be more challenging and resistant to treatment when it is associated with developmental exposure to adversity (1, 4). While it is known that stress during these time periods incites biological changes that directly affect the developing brain and influence later psychopathology, the specific neurobiological mechanisms that link these developmental stressors to adult psychopathology remain to be fully understood.

A critical, yet often neglected, factor is sex (5, 6). There are well-documented sex differences in the incidence of stress-related disorders, with women and sexual- and gender- minority groups often experiencing higher rates of adversity and psychiatric illness (7–9). The brain exhibits natural sexual differentiation during developmental processes in regions which have important functional roles in processing stress, such as the amygdala, hippocampus, hypothalamus, and BNST (10), and there is evidence of differing interactions of male and female gonadal hormones with the sustained hormonal stress response, the hypothalamic–pituitary–adrenal (HPA) axis (11, 12). To this end, many studies show sexually divergent effects of developmental stressors in the brain, which converge onto behavioral phenotypes (13–27). However, a holistic characterization of the lasting, sex-influenced effects of developmental stress on the brain and behavior is still needed to fully understand risk mechanisms for mental illness.

**Fig. 1.**
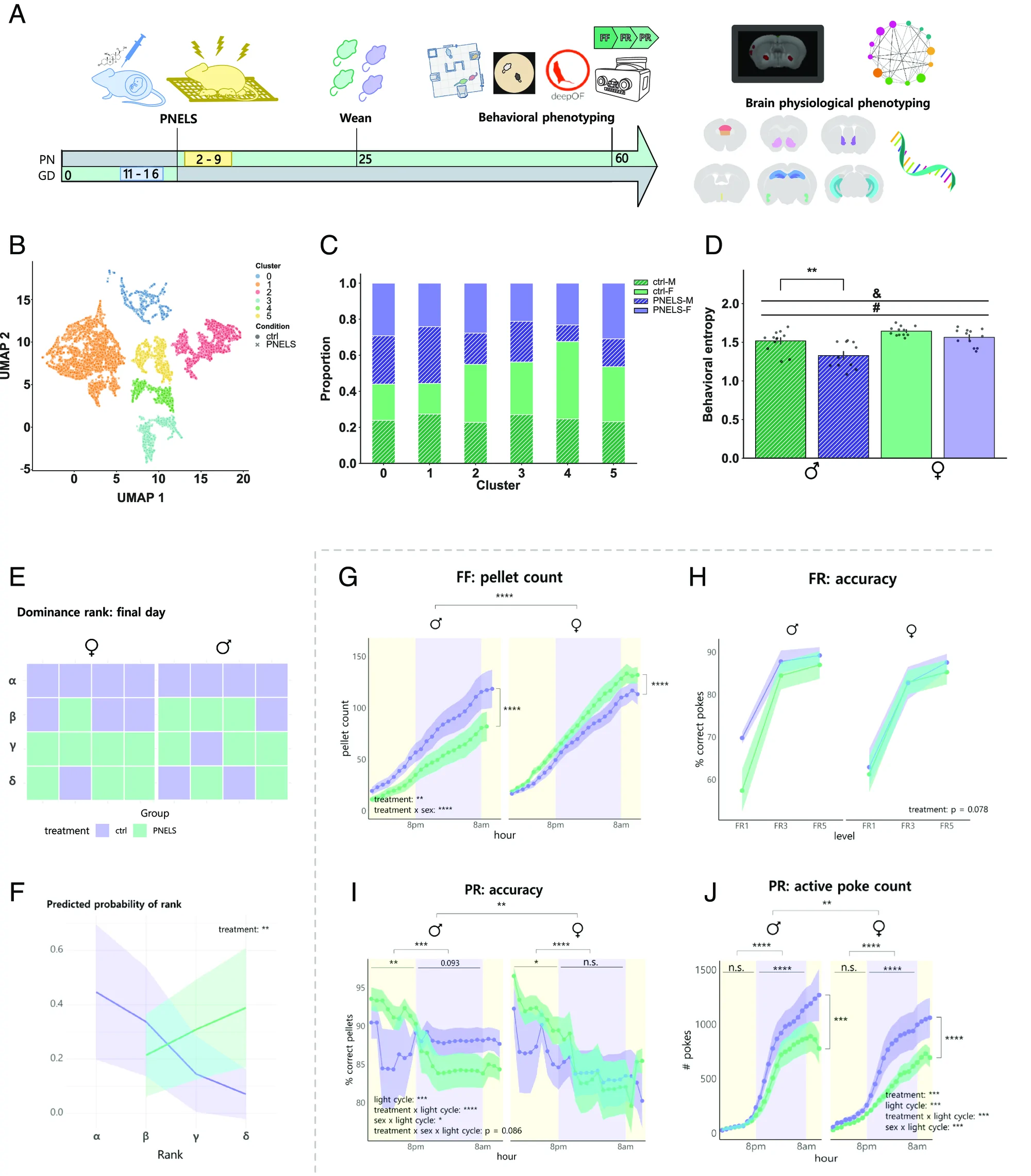
Developmental stress leads to alterations in adulthood behaviors related to psychiatric disorders. Timeline of PNELS experiments; all animals were first exposed to prenatal stress (CORT injection during GD 11-16) and early life stress (limited bedding and nesting during PN 2-9) (*A*). From the OFSI test, 6 stable behavioral clusters were identified (*B*; silhouette score = 0.462, Davies–Bouldin index = 0.703, Calinski–Harabasz index = 16,126), with effects on cluster occupancy (*C*, PNELS: χ^2^_(5)_ = 279.28, *P* < 0.0001, Cramér’s V = 0.144; sex: χ^2^_(5)_ = 471.67, *P* < 0.0001, Cramér’s V = 0.187). Stress and sex affected behavioral entropy, indicating more stereotyped, less flexible behavioral transitions in PNELS males (*D*, two-way ANOVA: sex: *F*_(1,43)_ = 24.17, *P* < 0.001; condition: *F*_(1,43)_ = 13.35, *P* < 0.001, interaction: *F*_(1,43)_ = 2.26, *P* = 0.140). In the semi-naturalistic social box paradigm, PNELS mice were always in subordinate positions in the social dominance hierarchy (*E*), making PNELS treatment predictive of rank (*F*, estimate = −2.1281, SE = 0.7519, z = −2.83, *P* = 0.00465). In the FED3 paradigm, free feeding pellet differed between sexes in the effect of PNELS [*G*, ANOVA: treatment: F(1, 992) = 9.151, *P* = 0.0026; sex: F(1, 992) = 72.139, *P* < 0.0001; time: F(23, 992) = 50.474, *P* < 0.0001; treatment x sex: F(1, 992) = 95.751, *P* < 0.0001, Post hoc Tukey: M ctrl vs. PNELS: *P* < 0.0001; F ctrl vs. PNELS: *P* < 0.0001], while percentage of active pokes in the fixed ratio stages was similar between sexes with only a trend toward lower accuracy in the PNELS group [*H*, ANOVA: treatment: F(1, 114) =3.156, *P* = 0.0783; Post hoc Tukey: all ns]. Task accuracy is higher in all PNELS animals during the daytime but switches at nighttime to reduced accuracy in PNELS males and no differences in females [*I*, ANOVA: treatment: F(1, 956) = 3.272, *P* = 0.0708; sex: F(1, 956) = 5.145, *P* = 0.0235; light cycle: F(1, 956) = 54.396, *P* < 0.0001; treatment x light cycle: F(1, 956) = 25.902, *P* < 0.0001; sex x light cycle: F(1, 956) = 5.851, *P* = 0.0158; treatment x sex x light cycle: F(1, 956) = 2.946, *P* = 0.0864]. Post hoc Tukey: day ctrl vs. PNELS: *P* < 0.0001, night ctrl vs. PNELS: *P* = 0.3128; day M vs. F: *P* = 0.9573, night M vs. F: *P* = 0.0062). Amount of active pokes is lower in PNELS animals for both sexes [*J*, ANOVA: treatment: F(1, 958) = 53.668, *P* < 0.0001; sex: F(1, 958) = 9.185, *P* = 0.0025; light cycle: F(1, 958) = 538.219, *P* < 0.0001; treatment x light cycle: F(1, 958) = 23.096, *P* < 0.0001; sex x light cycle: F(1, 958) = 13.587, *P* = 0.0002]. Post hoc Tukey: F ctrl vs. PNELS: *P* < 0.0001, M ctrl vs. PNELS: *P* = 0.0003; day ctrl vs. PNELS: *P* = 0.8807, night ctrl vs. PNELS: *P* < 0.0001; day M vs. F: *P* = 0.7325, night M vs. F: *P* < 0.0001). Stars represent *P*-values as follows: *<0.05, **<0.01, ***<0.001, ****<0.0001.

To address this, we developed a mouse model which combines prenatal and early life stress exposures (PNELS) to conduct a comprehensive sex-specific analysis of developmental stress. This model better captures the wider timelines of neurodevelopmental processes that play determining roles in the onset of psychopathology, leading to improved translatability of the cumulative burden of chronic adversity that characterizes the human condition (28). By combining deep learning-based behavioral phenotyping with complementing, multilevel neurobiological profiling—including sustained neural activity, acute functional circuit activation, and transcriptomics (see Fig. 1*A* for summary of study design)—we uncover distinct, sex-influenced pathways by which early adversity impacts adult behavior and brain function. We show how sex strongly shapes neurobiological and behavioral responses to developmental stress, and we identify molecular and circuit-level signatures associated with a greater risk of depression-like behavior in adulthood.

With this study, we provide an expansive foundational resource for a more translational, sex-informed approach to understanding psychiatric illness, and re-emphasize the critical need to consider sex as a biological variable in both preclinical research and the development of targeted therapies for stress-related disorders.

## Results

### Developmental Stress Leads to Alterations in Adulthood Behaviors Related to Psychiatric Disorders.

We first assessed whether developmental stress leads to enduring, sex-influenced alterations in behaviors relevant to psychiatric disorders. To achieve this, we employed an open field and social interaction (OFSI) test (29) along with a deep behavioral phenotyping pipeline utilizing marker-less pose estimation [DeepLabCut (30)] and an unsupervised annotation framework via DeepOF (29, 31) (Fig. 1*A* and *SI Appendix*, Figs. S1 *A*–*C*, S2, and S3). All behavioral differences in the DeepOF behavioral classifiers are depicted in *SI Appendix*, Figs. S1–S3, with clear effects of early life adversity as well as sex on individual behaviors such as nose-to-body interaction or stationary passive behavior. Using a data-driven, unsupervised machine learning framework combining UMAP dimensionality reduction and HDBSCAN clustering on the automated behavioral tracking data, 6 clusters representing experimentally meaningful and interpretable behaviors were identified (Fig. 1*B* and *SI Appendix*, Fig. S2 *A* and *B*, cluster 0: baseline locomotion, cluster 1: social investigation, cluster 2: arena exploration, cluster 3 and 4: passive/immobile, cluster 5: static active close to the arena), with strong separation (silhouette score = 0.462, Davies–Bouldin index = 0.703, Calinski–Harabasz index = 16126). Chi-square analysis confirmed that cluster membership was nonrandomly distributed across both condition (χ^2^_(5)_ = 279.28, *P* < 0.0001, Cramér’s V = 0.144) and sex (χ^2^_(5)_ = 471.67, *P* < 0.0001, Cramér’s V = 0.187), with sex emerging as the stronger organizer of behavioral state usage. Multinomial logistic regression confirmed that control animals showed significantly higher log-odds of occupying arena exploration (Cluster 2: β = 0.52, *z* = 5.29, *P* < 0.001; OR = 1.69), passive and immobile states (Cluster 3: β = 0.69, *z* = 6.00, *P* < 0.001, OR = 2.00; Cluster 4: β = 0.99, *z* = 8.53, *P* < 0.001, OR = 2.69), and the static active state (Cluster 5: β = 0.36, *z* = 3.31, *P* = 0.001, OR = 1.43) relative to PNELS animals. Notably, social investigation (Cluster 1)—the dominant behavioral state overall—was equally occupied by PNELS and control animals (*z* = 0.23, *P* = 0.818), indicating that PNELS does not suppress social engagement per se. Instead, PNELS appears to reduce the accessible behavioral range: Controls distribute themselves across a broader set of structured states, while PNELS animals default to undifferentiated locomotion. Sex was a stronger organizer of behavioral state usage than condition, with males predominantly occupying the social investigation state (Fig. 1*C*; OR = 1.42, *z* = 3.96, *P* < 0.001). A significant condition × sex interaction for Cluster 3 (OR = 0.67, *z* = −2.46, *P* = 0.014) indicated that the PNELS-associated suppression of this passive state was driven predominantly by females. Furthermore, in males, the dominant consequence of PNELS was a reduction in behavioral entropy and networks (*SI Appendix*, Fig. S2)—a more stereotyped, less flexible sequence of state transitions, while PNELS females remained comparably variable in their behavioral dynamics (Fig. 1*D* and *SI Appendix*, Fig. S2*C*; two-way ANOVA: sex: *F*_(1,43)_ = 24.17, *P* < 0.001; condition: *F*_(1,43)_ = 13.35, *P* < 0.001, interaction: *F*_(1,43)_ = 2.26, *P* = 0.140). To further characterize the effects of PNELS on social behavior, we assessed social dominance using the social box paradigm (32) in a separate cohort of mice, where both male and female PNELS animals adopted exclusively subordinate positions of the social dominance hierarchy (Fig. 1*E*). Developmental stress (PNELS) was found to be a significant predictor of rank in the hierarchy (Fig. 1*F*, *P* = 0.0047).

In a sequential set of home-cage paradigms using sucrose pellet rewards to assess depression-related behaviors (33) (*SI Appendix*, Fig. S1*C*, n = 12 for each group), PNELS males displayed reduced passive reward consumption (*P* < 0.0001), consistent with classical reports of anhedonia-like behavior. In contrast, PNELS females consumed more than controls (*P* < 0.0001), suggesting divergent effects of stress on reward sensitivity between sexes (Fig. 1*G*). In the fixed ratio task stages (33) FR1, FR3, and FR5 (*SI Appendix*, Fig. S1*C*), task accuracy did not differ due to PNELS or sex, but PNELS animals showed a trend toward lower task accuracy which was driven by the male FR1 stage (Fig. 1*H*; *P* = 0.0780), suggesting possible PNELS-linked learning impairments in males alone. In the progressive ratio (PR) task, PNELS animals showed higher accuracy during daytime which decreased at night—more so in males, with no difference in females (Fig. 1*I*). Active pokes followed a similar pattern, with PNELS animals exhibiting lower activity which was most significant during nighttime (Fig. 1*J*, *P* < 0.0001). In males particularly, reduced night time accuracy despite sustained activity suggests increased impulsivity due to PNELS.

Together, these findings support that developmental stress has long-term, sex-influenced effects on depression- and anxiety-like behaviors. Our assessments uncovered robust sex differences in behavioral patterns, which are often missed in classical tests (*SI Appendix*, Fig. S4). In particular, behavioral flexibility as well as social and reward-motivated behaviors are affected by developmental stress in a sex-influenced manner.

### Baseline Brain Activity in Regions Relevant for Stress Processing Is Altered in Adulthood in a Sex-Influenced Manner.

To understand brain-wide sustained activity differences in adulthood induced by developmental stress, we employed manganese-enhanced MRI (MEMRI) following sustained Mn^2+^ uptake via osmotic pump infusion (*SI Appendix*, Fig. S1*C*; ctrl M: n = 9; PNELS M: n = 8; ctrl F: n = 12; PNELS F: n = 11). Here, MEMRI contrast differences reflect activity-dependent accumulation of Mn2+ averaged over a whole week (p_FDR,cluster_ < 0.05).

**Fig. 2.**
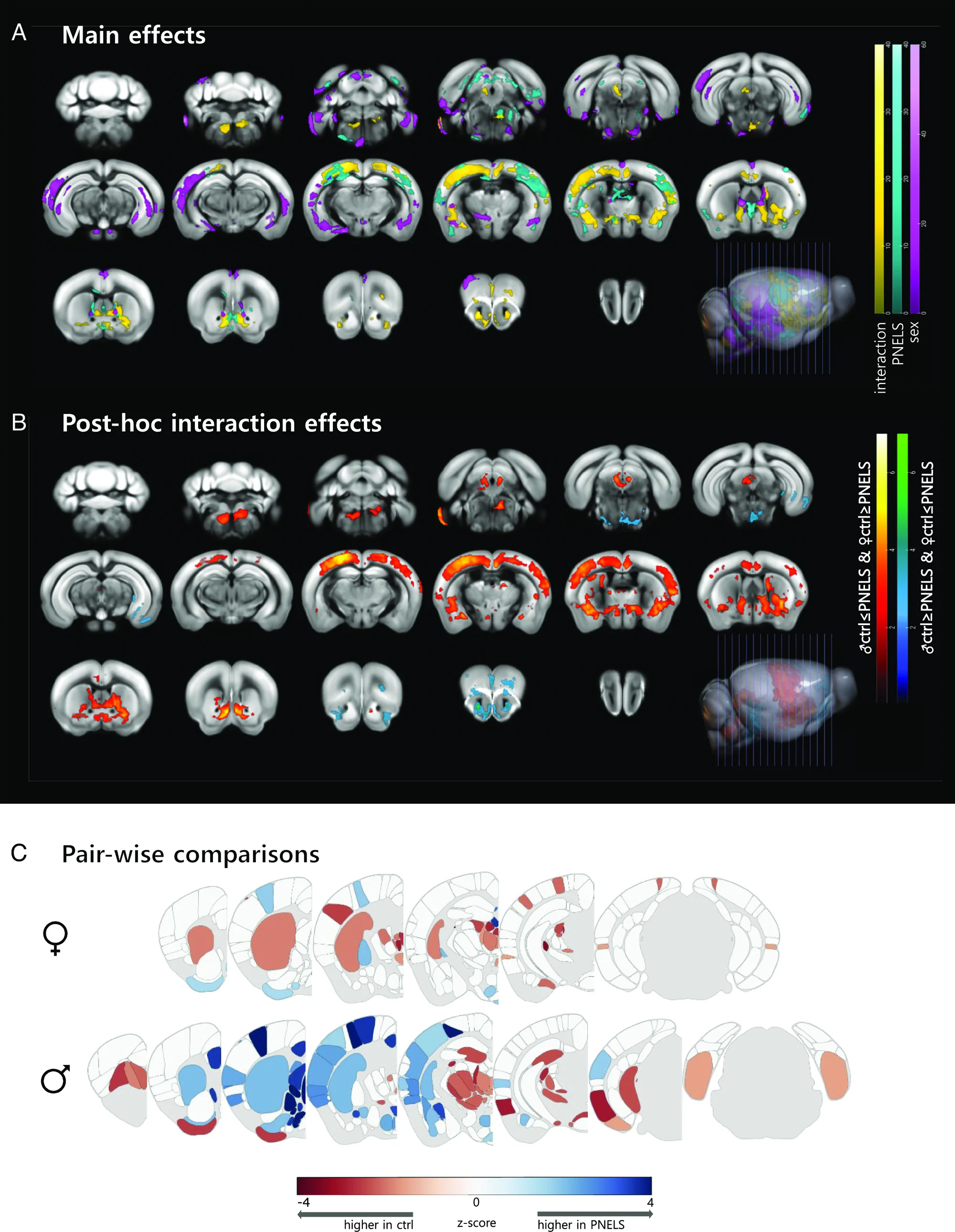
Baseline brain activity in regions relevant for stress processing are altered in adulthood in a sex-influenced manner. Main effects (*A*) of PNELS, sex, and their interaction in brain activity evaluated by voxel-wise analysis of MEMRI found significant (F-test, p_FDR,cluster_ < 0.05) changes in common stress related regions including amygdala, BNST, NAc, hypothalamus, thalamic structures, and cortex (*B*). Pairwise comparisons of voxel intensities between ctrl and PNELS groups of each sex indicate opposing direction of effect of PNELS in stress related regions (*t* test, p_FDR,cluster_ < 0.05) (*C*). Statistical maps are presented with clusters surviving FDR cluster correction (pFDR, cluster < 0.05), using a collection threshold of uncorrected *P* < 0.005.

Voxel-wise analysis revealed a predominant main effect of PNELS in increasing Mn^2+^-enhanced contrast mainly in Superior Colliculus, Entorhinal areas, Lateral Septum, Medial Preoptic Area, cortices (primary somatosensory, Posterolateral visual area, Agranular Insula posterior part), Endopiriform nucleus ventral part, and central amygdala (Fig. 2*A*, sex: pink, PNELS: turquoise, interaction: yellow; *SI Appendix*, Fig. S5*A*), as well as wide range of sex-influenced stress effects in a number of different brain regions including cortical, subcortical, and brainstem areas, with mainly intensity increases for stressed males and decreases for stressed females (Fig. 2 *B* and *C*). Common stress-related regions, including the amygdala, bed nucleus of the stria terminalis (BNST), nucleus accumbens (NAc), and hypothalamic, thalamic, and cortical structures, were found in clusters with significant interaction effects and in pairwise comparisons between groups, indicating broad sex-influenced effects of PNELS on sustained brain activity that mirror the distinct behavioral patterns.

### Developmental Stress Has Sex-Specific Effects on Brain Functional Networks Underlying Behaviors Relevant for Psychiatric Disorders.

**Fig. 3.**
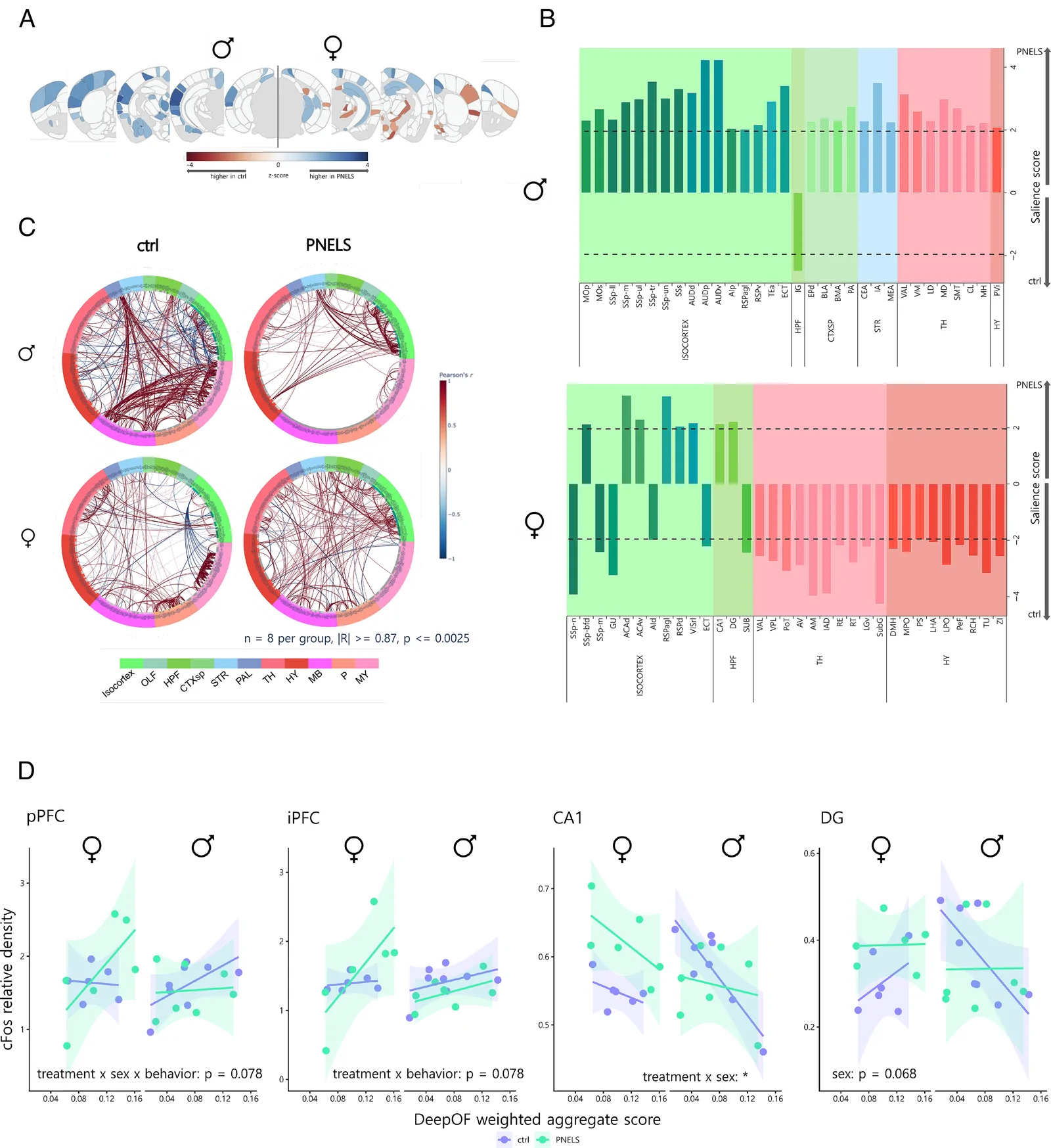
Developmental stress has sex-specific effects on brain functional networks underlying behaviors relevant for psychiatric disorders. Direction of contribution of cFos activity to stress in brain subregions, in males (global *P*-value = 0.227) and females (global *P*-value = 0.075) (*A* and *B*; *P* < 0.05) and functional connectome of each group (*C*; R ≥ 0.87, *P* < 0.0025) showed that males and females showed many opposing patterns of brain network connectivity in adulthood after PNELS. Linear regression to show whether cFos relative density values from select brain regions, sex, and a DeepOF behavioral composite score can predict cFos relative density [*D*, lm(region ~ sex * treatment * DeepOF score); pPFC: sexM:treatmentPNELS, *P* = 0.078; iPFC: treatmentPNELS:score, *P* = 0.078; CA1: treatmentPNELS:sexM, *P* = 0.047; vDG: sexM: *P* = 0.0368].

To further probe the neural correlates of behavioral alterations induced by developmental stress, we next performed whole-brain c-Fos mapping following the OFSI paradigm (*SI Appendix*, Figs. S1*B* and S6, n = 8 for each group and sex). We again observed sex-influenced effects of developmental stress on global c-Fos expression. A region-by region analysis revealed that in males, PNELS increased c-Fos expression relative to the control condition in regions where control females showed higher baseline activity, such as in thalamic, hypothalamic, and some isocortical areas (Fig. 3 *A* and *B* and *SI Appendix*, Fig. S7) suggesting sex-influenced activity reorganization following stress during early development. Functional connectome analyses further supported this dissociation. Although PNELS males showed a less prominent global activity shift, they showed a marked reduction in interregional connectivity relative to controls, whereas PNELS females exhibited the opposite pattern, with enhanced connectivity strength and density (Fig. 3*C*, R ≥ 0.87; *P* ≤ 0.0025).

To identify associations between the behavioral and brain network alterations following PNELS, we focused on regions relevant to stress processing and used linear regression to associate c-Fos relative density values with a weighted aggregate score calculated from the 3 top most informative behavioral clusters found from the OFSI test. The prelimbic and infralimbic prefrontal cortex and the dentate gyrus (DG) and CA1 of the hippocampus stood out as regions where predictive relationships between c-Fos activity and behavioral scores were altered by both sex and developmental stress (Fig. 3*D*, *P* < 0.05).

Together, these results reveal that developmental stress reshapes circuit activation and functional connectivity in a sex-influenced manner, with implications for divergent behavioral outcomes.

### Diverse Changes in the Brain Transcriptomic Responses Indicate Sex-Influenced Effects of Developmental Stress.

**Fig. 4.**
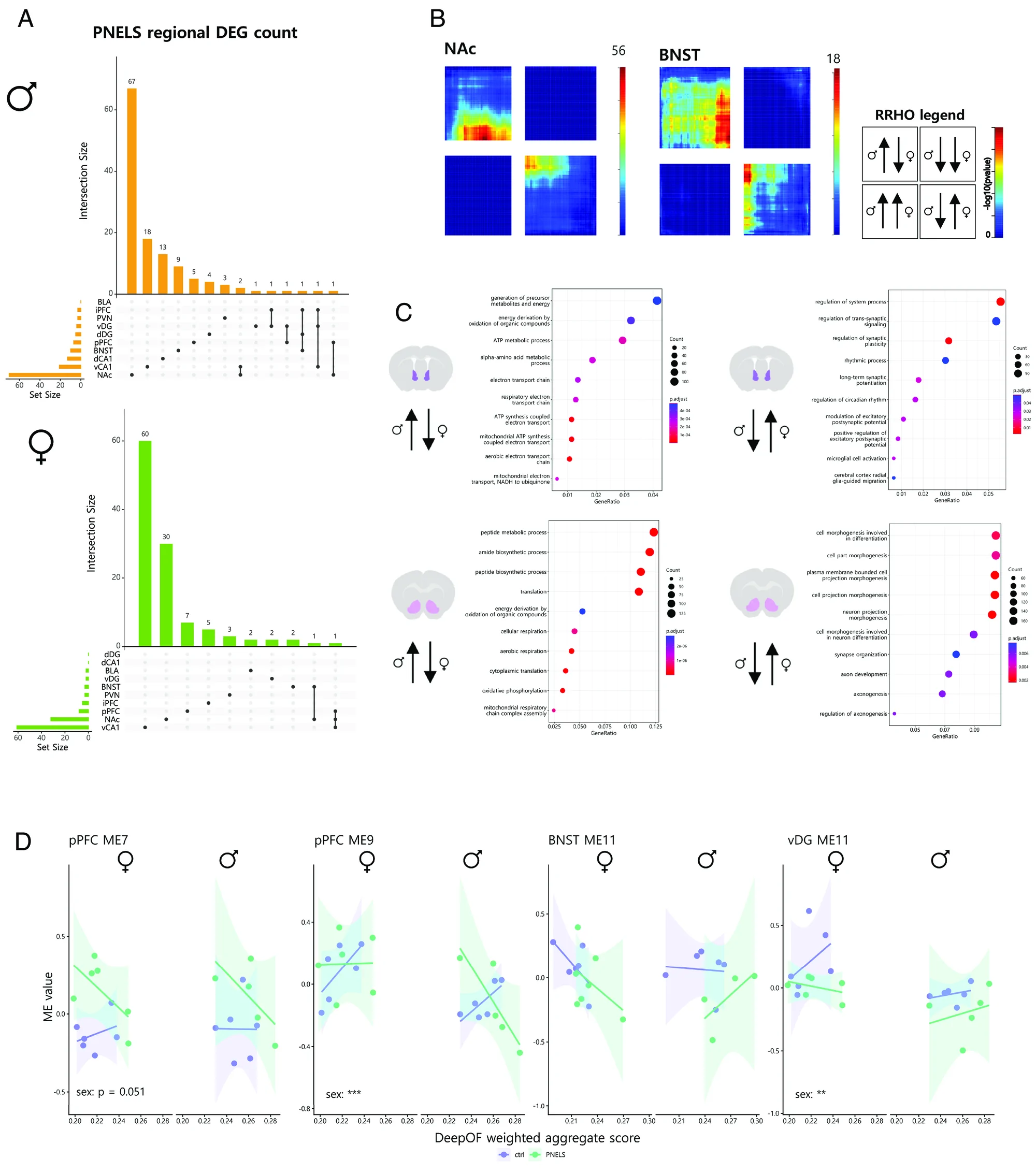
Diverse changes in the brain transcriptomic responses indicate sex-influenced effects of developmental stress. The majority genes differentially expressed (*P* < 0.1) due to PNELS were not shared across brain regions (*A*). Threshold-free overlap of commonly expressed male and female gene lists found discordance in NAc and BNST (*B*). Gene ontology enrichment for the lists discordant between sexes indicate opposite direction of regulation of neuronal signaling and cell metabolic process (*C*). WGCNA revealed four modules strongly associated to PNELS, sex, and DeepOF score (R > 0.5; *P* < 0.05) of which the eigenvalues of three had predictive relationships with behavior which were dependent on sex and treatment [*D*, lm(DeepOF score ~ sex * treatment * ME eigenvalues); pPFC ME7: sexM, *P* = 0.052; pPFC ME9: sexM: *P* = 0.0008; BNST ME11: all ns; vDG ME11: sexM: *P* = 0.0085].

To investigate the molecular basis of these differential brain activity and behavioral patterns, we next performed bulk RNA sequencing (RNA-seq) in 10 selected regions of interest (*SI Appendix*, Fig. S1*A*), selected for their known roles in stress-related psychopathology. Differential gene expression analysis from bulk RNA-seq revealed region- and sex-influenced transcriptional responses to PNELS [FDR-adjusted *P* (*P*adj) < 0.1]. The majority of differentially expressed genes (DEGs, 93.7% in males and 98.2% in females) were unique to a single region, and the amount of DEGs per region varied markedly by sex (Fig. 4*A* and *SI Appendix*, Table S1) with the highest number of DEGs in males identified in the NAc (N = 67) and the ventral CA1 (N = 60) in females. These findings underscore the distinct biological roles and transcriptional architectures of each brain region in the context of developmental stress.

As very few DEGs were shared between sexes at the FDR cut-off (FET *P* > 0.5), we applied a threshold-free rank–rank hypergeometric overlap (RRHO) analysis to examine transcriptomic convergence or divergence between sexes, allowing for a prioritization of the significance of rank-consistency over the absolute magnitude of change for individual variables. While most regions displayed largely concordant expression changes across sexes after PNELS (*SI Appendix*, Fig. S8*A*), the NAc, and BNST showed divergence in direction of gene regulation between sexes (Fig. 4*B*; ρ < 0, CI < 0.5). Conversely, exclusive concordance between sexes was seen in all when comparing the transcriptomic overlap for adulthood acute restraint stress (*SI Appendix*, Fig. S8*B*). The NAc and BNST regions, implicated in reward processing and anxiety, may therefore serve as sex-specific hubs mediating observed behavioral responses to developmental stress.

Functional enrichment of genes regulated in opposite directions between sexes in the NAc and BNST (*SI Appendix*, Table S2) further supported this dissociation. In males, upregulated pathways included energy metabolism and cellular homeostasis, while in females, opposing changes were enriched for synaptic signaling, plasticity, and neurodevelopmental processes (Fig. 4*C*).

To identify coordinated transcriptional networks associated with PNELS, we performed weighted gene coexpression network analysis (WGCNA) in regions of interest where previous data implicated sexual dimorphism in developmental stress outcomes (*SI Appendix*, Fig. S9 and Table S3). Of four modules in the pPFC, BNST, and vDG found to be correlated to PNELS, three showed significant correlations and predictive relationships with sex (Fig. 4*D*, *P* < 0.05) revealing candidate gene networks that may underlie sex-specific vulnerability to developmental stress.

### Sex Differences in Associations of Developmental Stress to Major Depressive Disorder in Humans.

**Fig. 5.**
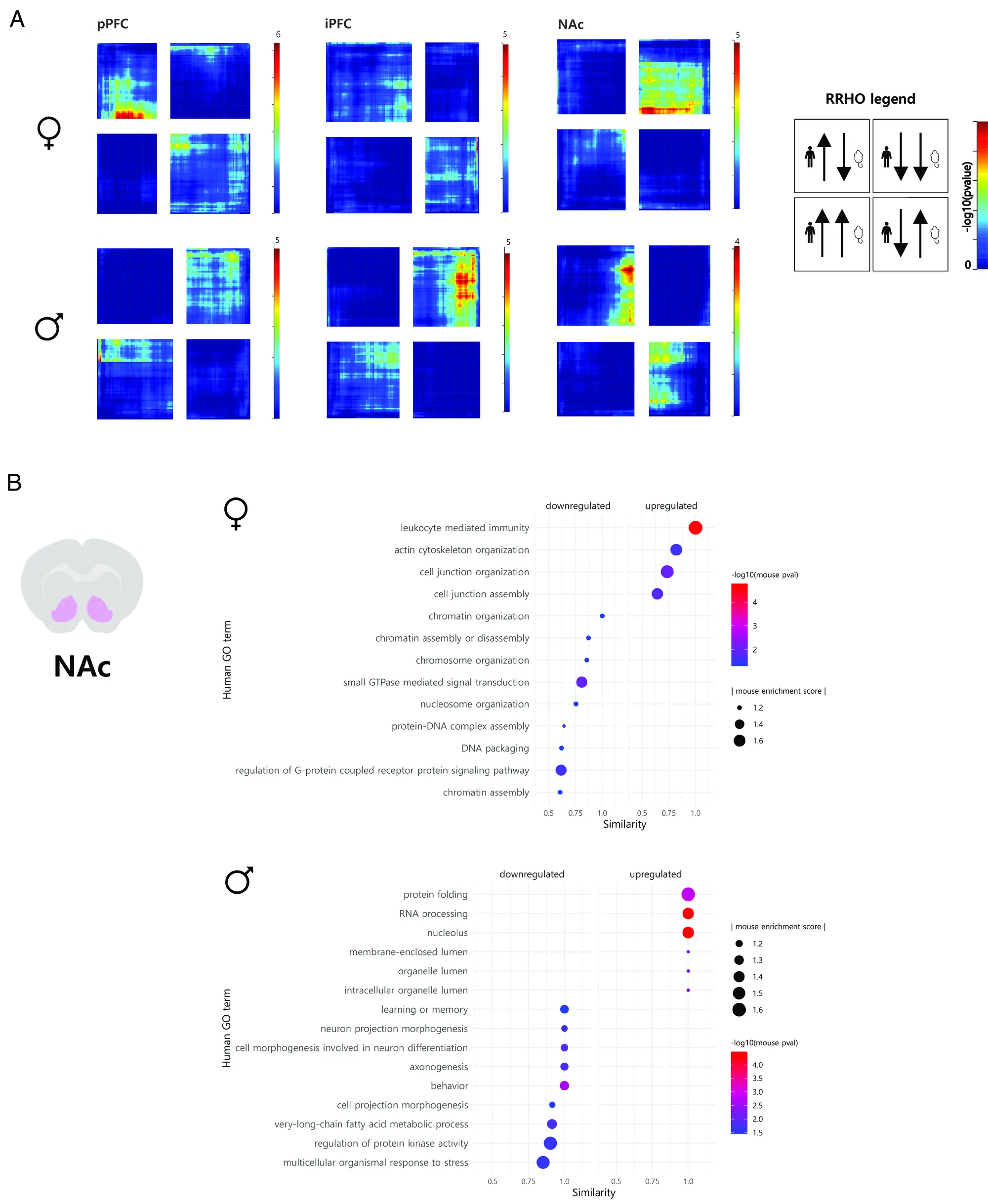
Sex differences in associations of developmental stress to human major depressive disorder. RRHO for male and female MDD profiles against PNELS profiles in pPFC, iPFC, and NAc showed an opposite pattern of direction of regulation between sexes in overlapping genes per region (*A*). Semantic similarity analysis indicates sex-influencedterms overlapping across PNELS in mice and MDD in humans (*B*, *P* < 0.05, similarity > 0.5), with processes related to cellular and DNA organization more conserved in females and processes more related to neuronal function in males.

To assess the translational relevance of transcriptomic alterations observed in the PNELS model, we compared our mouse data to a publicly available human dataset profiling gene expression in the postmortem pPFC, iPFC, and NAc of individuals with major depressive disorder (MDD) (34). Interestingly, though concordance was low between species (ρ ≈ 0, CI ≈ 0.5), we observed opposing patterns of direction of gene regulation between sexes across each region (Fig. 5*A*), suggesting that developmental stress may engage distinct transcriptional programs in males and females that converge on divergent risk trajectories for depression.

Furthermore, using available human data for the NAc, we assessed semantic similarity of gene ontology enrichment for developmental stress and major depressive disorder, finding distinct biological processes between species for male and female lists (*SI Appendix*, Table S4). Males had more overlap between species and upregulated terms related to nuclear processes downregulated to neuronal and behavioral terms, while females had upregulated terms related to cellular assembly and downregulated terms related to DNA organization (Fig. 5*B*).

These findings underscore the importance of considering sex-influenced molecular pathways in the etiology of stress-related disorders and suggest that developmental stress can prime the brain for depression through distinct, region- and sex-dependent mechanisms.

## Discussion

In this study, we characterized sex-influenced effects of developmental stress exposure on adulthood behavior and neurobiology, in a translationally relevant mouse model which better encompasses neurodevelopmental processes relevant to the onset of human psychiatric disorders. By combining deep behavioral phenotyping with multimodal investigation, including baseline brain-wide activity, circuit-level function, and bulk transcriptomic profiling, we offer a comprehensive view of how developmental stress shapes the brain and behavior across multiple levels of organization in males and females.

A central finding of our work is the profound sexual dimorphism in how early life adversity impacts the brain and behavior. This observation builds on and significantly extends previous literature documenting substantial sex differences in response to stress and adversity (14, 35–37). Our data reveal convergent and distinct effects of developmental stress across behavioral domains, which could largely explain the sex differences in prevalence and symptomatology of stress-related psychopathology reported in humans (22, 38). This work challenges classical, male-centric interpretations of stress-related behavior and indicates the importance of including sex in investigations in stress and psychiatric research, especially in a developmental or life-history context. It offers insight into how stress responses may reflect distinct adaptive or maladaptive strategies in males and females, crucial for hypothesis generation for future mechanistic studies.

At the neurobiological level, we found that PNELS induces widespread, sex-influenced alterations in brain activity patterns. Using MEMRI to investigate brain activity over the course of a week, we showed that PNELS recruits a coherent network for stress response, with heightened basal activity in regions involved in hypervigilance, anticipatory anxiety, and autonomic tone. Interaction effects between sex and PNELS are showing mainly increases for males after PNELS, of which some activity difference may be linked to the observed behavioral differences, such as higher impulsivity in the sucrose reward task to elevated hypothalamic (arousal) and NAc activity in stressed male mice (39).

Functional c-Fos mapping of task-evoked activity revealed different and often opposing network-level strategies for stress adaptation in males vs. females, potentially speaking to the interplay of gonadal hormones and sex-chromosomal effects on modulating neuroplasticity, connectivity, and neurotransmitter signaling (40). In males, predominantly cortical and limbic brain areas showed hyperactivation following early adversity, while in females thalamic and hypothalamic nuclei displayed hypoactivation. Collectively, these findings suggest that even similar behavioral consequences of early life adversity, such as lower social dominance (41) and suppressed reward behavior, are governed by sex-distinct changes in brain circuit activity.

By using different methodologies to characterize not only sustained brain activity in baseline conditions but also acute responses after a specialized task, we showcase the profound complexities between male and female brain outcomes in the context of developmental stress, and provide a neuroimaging resource that can be compared to future human studies investigating mechanisms of early life adversity. These modalities capture distinct temporal and spatial scales: MEMRI provides a brain-wide, temporally integrated measure of “tonic” activity over a week, reflecting a stable baseline state, whereas cFos mapping provides a cellular-level “phasic” snapshot of task-evoked recruitment. By employing both, we intended to distinguish between stable neurobiological traits and state-dependent circuit reactivity, in order to provide a more nuanced understanding of stress adaptations. Converging effects in specific brain regions across modalities highlight potential strong mediators to focus on in future studies, while diverging effects showcase the context-specificity of stress effects.

Our transcriptomic analysis further supports this divergence, revealing sometimes opposing directions of gene regulation due to developmental stress in key brain regions—particularly the NAc and bed nucleus of the stria terminalis (BNST). These regions are known to mediate reward and anxiety-related behaviors and are strongly interconnected (42, 43). Notably, enrichment analyses suggest that PNELS affects synaptic signaling, neurodevelopment, and metabolic pathways in a sex-influenced manner. These data align with previous works (44, 45) and expand upon them, suggesting that sex differences in molecular response to early stress, particularly in regions related to reward-processing and anxiety, may play a central role in shaping long-term vulnerability or resilience to psychiatric disorders.

These neurobiological insights are valuable due to the comparability of our rich animal dataset to past and future studies of psychiatric disorders and early life adversity. A cross-species comparison using a currently available dataset of postmortem human brain tissue from individuals with major depressive disorder (MDD) (34) indicate only slight overlap but in a sex-influenced and region-specific manner, suggesting that MDD transcriptomic profiles reflect changes including but also going beyond developmental-stress-related effects. Enriched biological processes between PNELS and MDD were also distinct between sexes, and future comparisons with ELA-focused human datasets can be essential for the development of sex-sensitive interventions for patients with history of early life adversity.

This study was not without limitations. Phenotypes were only measured in young adulthood, but manifestations of psychopathology fluctuate throughout life and are worthy of detailed investigation for more enhanced translatability. Furthermore, while our RRHO approach provides powerful, threshold-free insights into global dataset concordance, it operates as a rank-based metric that prioritizes systems-level alignment over the absolute magnitude of individual gene changes. Similarly, comparing overlap of bulk sequencing datasets provides insights only into transcriptomic patterns and potential associated mechanistic differences but do not consider specific mediating genes, differing magnitudes of changes between regions, or mechanistic roles of nonoverlapping genes which were profiled. Finally, our experiments were not designed to allow conclusions on which aspect of sex, i.e., hormones, sex chromosomes, or lived experience, underlies the influence of sex on the observed genetic, functional, and behavioral phenotypes. Despite this lack of specificity in the current methodology, the overarching study clearly indicates sexually dimorphic effects, and future studies can use the provided data as a resource for hypothesis-generation and interspecies comparisons.

In conclusion, this study’s translational approach, complementing multimodal neurobiological analysis, and rigorous sex-specific investigation reveal insights into the lasting consequences of early life adversity. By laying a foundation for more inclusive and precise models of psychiatric vulnerability, this work encourages more effective and equitable strategies for prevention and treatment.

## Materials and Methods

### Mouse Model.

#### Housing conditions.

All protocols were approved by the committee for the Care and Use of Laboratory Animals of the government of Upper Bavaria, Germany, and all procedures were in accordance with the European Communities Council directive 2010/63/UE.

Adult CD1 mice aged 8 to 10 wk were obtained from Charles River and housed in standard conditions (12 h/12 h light/dark cycle with dark phase starting at 8 pm, temperature 23 ± 2 °C, 55% humidity) at the Max Planck Institute for Psychiatry, with laboratory chow diet and water available ad libitum. Animals were acclimated for 1 wk in the facility before beginning procedures.

#### Breeding.

Male and female mice were pair-housed and females checked daily for vaginal plugs to estimate date of conception. Males and females were separated after 5 d of breeding.

#### Developmental stress treatment.

A combination of prenatal and postnatal early life stressors was used, to increase translational value.

The protocol for prenatal stress exposure (PNS) was derived from Astiz et al. (46). Solid 98% corticosterone (CORT) was dissolved in polyethylene glycol-400 (*Sigma-Aldrich*) at a concentration of 20 mg/mL and stored at room temperature. Pregnant dams were injected subcutaneously with CORT daily at a concentration of 50 mg/mL from gestational day (GD) 11 through GD16 within the first hour of the lights turning on in the animal facility, to offset natural corticosterone rhythms.

The established limited bedding and nesting (LBN) paradigm (47) was introduced as an early life stressor (ELS). On postnatal day 2 (D2), litters were culled to a maximum of 12 pups, balancing as much as possible for sex. Dams and pups were transferred to a cage a metal grid inlayed on the floor, which contained half of a cotton nestlet and 50 mL of bedding material. Control litters were transferred to a cage with the standard amount of bedding material and 2 cotton nestlets. At D9, pups were weighed and transferred back to standard housing conditions.

Each experimental cohort included two experimental groups: nonstressed controls (ctrl) and a combination of prenatal and early life stress (PNELS) with an equal number of animals of each sex in each group.

#### Weaning.

Between postnatal days 24 to 26, animals which were to be used for social behavioral phenotyping were weaned into nonsibling social groups containing two PNELS animals and 2 ctrl animals. Animals were weighed on the weaning day and all behavioral phenotyping began in young adulthood, from 8 wk of age.

### Behavioral Phenotyping.

#### OFSI test.

OFSI tasks were performed in an acute novel context as previously described (29). Experimental animals were placed in a round open field (diameter 38 cm) with bedding material on the apparatus floor. Exploration activity of the single animal was recorded for 10 min, then an unfamiliar C57BL6 of similar age was introduced into the apparatus as a novel social partner and pair activity was recorded for another 10 min.

#### Social box.

The social box was used to test group social dynamics and dominance hierarchy in a seminaturalistic setting. The protocol and setup were implemented as previously published (32). Mice were marked with distinguishable colors and placed in groups of four in a 60 × 60 cm square arena, where their activity was recorded for 4 days and 4 nights.

#### FED3 home-cage operant reward task.

Feeding Experiment Devices version 3.0 (33) (FED3) were used to assess behaviors related to motivation, anhedonia, and reward learning. Each FED3 has a programmed active and inactive nose port and a mechanism to contain and dispel 20 mg sucrose pellets (*5TUT*). Mice were first habituated with single housing and 15 sucrose pellets scattered throughout the home cage. On the first experimental day, a FED3 was added to the home cage in free feeding (FF) mode, in which sucrose pellets were readily retrievable. Animals were checked once daily and progressed to the fixed-ratio (FR) stages once they retrieved a minimum of 100 pellets in the FF stage. For the stages FR1, FR3, and FR5, the animal needed to perform one, three, or five nose-pokes, respectively, in the active port in order to receive a sucrose pellet. Animals were progressed fromFR1 to FR3 after receiving at least 75 pellets in FR1 and to FR5 after 60 pellets in FR3. After 60 pellets in FR5, animals were food-restricted for 24 h and proceeded to the final progressive ration (PR) stage. During the PR, the number of nose pokes required to receive a reward increased after each reward was retrieved. Analysis of FED3 data was implemented with the aid of python package FED3_Viz-0.5.1 and R package FED3.

### MEMRI.

#### Subject preparation.

Animals were sedated with 4% isoflurane, secured in a prone stance on a platform, and maintained at 1.5 to 2.5% isoflurane anesthesia (100% oxygen, airflow 1.5 L/min) throughout the scanning procedure. Bepanthen® cream was applied to the eyes to prevent drying. Body temperature was monitored via a rectal temperature and maintained at 36 to 38 °C, and respiration was monitored with a pressure sensor set under the animal’s thorax. Isoflurane flow was adjusted accordingly as needed to maintain a respiration rate of 80 to 120 breaths per minute. Animals were killed after the scanning procedure.

#### Manganese infusion.

Manganese was delivered continuously via osmotic mini pumps as previously described (48) to minimize systemic side effects and to reduce stress associated with repeated injections. To monitor for potential side effects attributable to MnCl_2_ toxicity or the implantation procedure (49), we applied a strict welfare scoring system with exclusion criteria encompassing evaluation of the surgical wound, body weight, food and water intake, behavior, mobility, and overall appearance. Manganese(II) chloride tetrahydrate (MnCl2 x 4H2O, *Sigma Aldrich*) was dissolved in Tris buffer at a dosage of 60 mg/kg for each animal injected into an osmotic minipump (*Alzet*) of 100 µL volume. Pumps were primed in a water bath at 37 °C overnight before surgical implantation to ensure their activation. Vetalgin (200 mg/kg) was given subcutaneously as an analgesic 30 min prior to the surgery and mice were anesthetized with 4% isoflurane and secured to a stereotaxic frame. Immediately prior to the surgery, Meloxicam (0.5 mg/kg) was administered as an analgesic and eye ointment was applied to prevent drying of the eyes. Pumps were implanted subcutaneously under the skin of the left flank of the animal, while maintained under anesthesia with 2% isoflurane. Animals received Metacam in drinking water 3 d postoperation and were monitored for 7 d prior to imaging.

#### MRI.

MEMRI was performed on a 9.4T Bruker Biospec 94/20 system using a cross-coil configuration: Radio-frequency transmission was achieved with a volume resonator, and signal detection was performed using room temperature 2 × 2 surface array coils. Six short consecutive 3D T1-weighted FLASH images were acquired with the following parameters: TR = 20.1 ms, TE = 5.86 ms, flip angle = 30°, matrix size = 200 × 150 × 100 voxels, field of view = 20 × 15 × 10 mm^3^, bandwidth = 25 kHz, two averages, and RF spoiling. Images were acquired along the rostral–caudal axis, with each 3D acquisition lasting 10 min 50 s. The total imaging session, including localizer scans and system adjustments, required approximately 75 min.

Data were converted using the Python Bru2nii function, in which the size of the voxels in the images was increased by 10-fold, in order to be comparable with default values for human sized brains used by SPM12 in the following analyses. The six individual runs were coregistered, and a mean image calculated.

The steps for MEMRI image preprocessing are found in *SI Appendix*, *Supplemental Methods*.

### c-Fos Mapping.

#### Tissue extraction for c-Fos.

c-Fos expression was induced before killing the animal using the aforementioned open field & social interaction paradigm as a mild social challenge 60 min prior to perfusion. Animals (n = 32; divided equally by developmental stress treatment and sex) were then anesthetized with isoflurane and perfused transcardially with 4% paraformaldehyde (PFA). Brains were dissected and fixed overnight in PFA, then for the following 48 h in phosphate-buffered saline (PBS)-30% sucrose, and then in PBS at 4 °C.

#### Brain sectioning.

Whole brains were placed in a container, bathed with optimal cutting temperature compound (OCT) and frozen, then stored at −80 °C. Brains were sliced at 40 μm using a cryotome (*LEICA CM1900*) and stored in PBS or antifreeze solution.

#### Immunohistochemistry.

In order to maintain specificity of c-Fos detection, tissue slices underwent blocking and permeabilization using bovine serum albumin and Triton X-100 respectively. Slices were then incubated with recombinant rabbit anti-c-Fos as a primary antibody to bind to the c-Fos protein, and donkey anti-rabbit Alexa Fluor-647 (AF647) as a secondary antibody to bind to the primary and therefore mark c-Fos molecules with a red fluorescent tag. Following incubation, slices were mounted onto slides and covered with DAPI, a nuclear stain.

#### Image acquisition and analysis.

Images were acquired with slide scanner system Axioscan by Zeiss, using channels for DAPI and AF647. The tool Aligning Big Brain Atlases (ABBA) (50) was used to align images to the Allen Brain Atlas in order to have an accurate per-region quantification of c-Fos-expressing neurons. BraiAn’s extension for QuPath (51) v0.4.4 was used to segment c-Fos+ cells in the aligned images, as well as to localize image artifacts. Exemplary images from each experimental group are provided in *SI Appendix*, Fig. S6.

### Bulk RNA-seq.

#### Euthanasia.

After behavioral testing was finished, animals were left undisturbed for over 24 h before being euthanized under baseline conditions. A subset of animals, balanced for sex and developmental condition, underwent restraint stress 4 h prior to euthanization. Animals were anesthetized with isoflurane and sacrificed by decapitation. Brains were dissected out and snap-frozen using methyl-butane on dry ice and stored at −80 °C.

#### Sample preparation and sequencing.

Frozen brains were sectioned using a cryostat microtome at 250 μM and a 0.8 mm-diameter punching needle was used to extract tissue from the following brain regions: 1. prelimbic and 2. infralimbic prefrontal cortex (pPFC & iPFC), 3. NAc, 4. anterodorsal bed nucleus of the stria terminalis (BNST), 5. paraventricular nucleus of the hypothalamus (PVN), 6. basolateral amygdala (BLA), 7. dorsal DG and 8. dorsal CA1 of the hippocampus (dDG & dCA1), and 9. ventral DG and 10. ventral CA1 (vDG & vCA1).

Prior to preparation, samples were randomized to reduce experimental batches using R package Omixer and subdivided into 5 plates each, including 2 brain regions; each brain region was treated as an individual experiment. RNA isolation was performed using the Zymo Direct-Zol kit and quality measured using the Agilent Tape Station 2000, small RNA kit and Bioanalyzer, Agilent RNA ScreenTape and High Sensitivity RNA ScreenTape. cDNA libraries were prepared using the NEBNext Ultra II Direction RNA Library Prep Kit for Illumina, and sequencing was preformed using a NovaSeq6000 and Helmholtz Munich in two separate runs, using an S4 flow cell for the first four plates and S1 for the fifth plate.

### Statistical Analysis.

#### DeepOF analysis.

Videos were analyzed using automated pose estimation software DeepLabCut v2.2.2 (30) and annotated motion tracking program DeepOF v0.8 (29).

The detailed pipeline for the behavioral analysis is found in *SI Appendix*, *Supplemental Methods*.

#### Social box analysis.

Interactions between mice were analyzed using DeepLabCut tracking and a previously published pipeline (52), which calculated a David Score as a metric based on chasing behavior as a readout of social dominance rank.

#### Behavioral phenotyping statistics.

For behavioral tests, R version 4.3.3 was used to assess statistical significance and create plots. For OFSI and FED3, normality was assessed with Shapiro–Wilk tests, and homogeneity of variance by Levene’s tests. When assumptions of normality and homogeneity were met, ANOVA was used for one, two, or three-variable designs. Significant ANOVAs were followed up by post hoc Tukey tests for mean comparisons. A repeated-measured ANOVA was used to compare experimental groups when measurements were taken from multiple timepoints, and a mixed effects model was implemented in case of any missing values. Significance was set at *P* < 0.05 and outliers were determined via the interquartile range method. For the social box, ordinal logistic regression was used to model the effect of stress on rank in the social dominance hierarchy.

#### MEMRI analysis.

Statistical analysis was performed in SPM12, using a 2 × 2 factorial design with factors “sex” and “stress,” assuming independence and unequal variance between groups (male, ctrl: n = 9; male, PNELS: n = 8; female, ctrl: n = 12; female, PNELS: n = 11). As an explicit mask, we used the ABA mask image excluding the cerebellum and the olfactory bulb. Global normalization was considered by introducing global signal intensity as a nuisance regressor in the model. Statistical maps are presented with clusters surviving FDR cluster correction (p_FDR,cluster_ < 0.05), using a collection threshold of uncorrected *P* < 0.005.

We also tested if potential morphological differences between groups may coalign with the MEMRI findings. For this, the deformation matrices were converted to logarithmic jacobian determinants and again compared in the 2 × 2 factorial model (*SI Appendix*, Fig. S4*B*).

#### c-Fos mapping analysis.

For the whole-brain c-Fos analysis, mean-centered task partial least squares analysis (PLS) was conducted in python with BraiAn (50). The analysis was done over 176 “summary structures” [a list of regions defined by CCFv3, (53)], within Isocortex, CTXsp, HPF, STR, PAL, TH, and HY. The PLS was performed over the c-Fos density of each brain structure, and the resulting contrasts and salience scores were generalized and normalized through permutation and bootstrap testing sampled 10,000 times each. In order to circumvent possible biases introduced by performing the immunochemistry staining at different time points—thus, with different solutions, we performed a batch normalization. All region-quantifications were normalized on an additional staining that was performed the same day with the same solution throughout all 32 brains, 5 sections each.

PLS is presented for regions where *P* < 0.05 for the comparison between ctrl and PNELS for each sex. For connectivity analysis, Pearson correlation was calculated for cFos density across regions and presented for correlations where R ≥ 0.87 and *P* ≤ 0.0025. Linear regression was used to predict effects of relative density of cFos for individual brain regions of interest on the weighted aggregate score of DeepOF behaviors. Regions were chosen from those selected for bulk RNA-seq due to known stress-related roles. Linear model formulas were formatted as lm(region ~ sex * treatment * DeepOF score).

#### Bulk RNA-seq analysis.

For RNA-seq, raw data were processed using the nf-core bioinformatic pipeline *rnaseq* [version 3.5; (54)], in which *STAR* was used for alignment to the reference genome mm10 for mouse (refGenie), and *salmon* for gene quantification. Samples with an alignment rate of less than 5% were excluded and gene expression was filtered to a minimum of 10 counts for subsequent analysis. Principal component analysis was used to further identify outliers and exclude 36 of the 469 samples.

Differential gene expression was used to define transcripts significantly altered. DEGs were determined using the DESeq2 R package, with a sex-stratified approach using likelihood ratio tests, which captured the individual effects of PNELS and adulthood stress, as well as the interaction between them. The threshold used to define differential expression was *P*adj < 0.1. Full DESeq2 results are found in *SI Appendix*, Tables S1–S4.

Rank-Rank hypergeometric overlap (RRHO) analysis, a threshold-free method to compare genetic signatures across studies (55), was used to compare transcriptomic profiles across studies. RRHO was conducted using the RRHO2 R package (56). Lists were filtered to include only genes overlapping between studies and the significance was mapped using −log10(*P*adj) adjusted for direction of change in expression, using data from Labonté et al. (34) for the interspecies comparison. Fisher’s exact tests (FET) were used to assess the significance of overlapping differentially expressed genes (DEGs) between datasets for each quadrant of concordant and discordant regulation, using the intersection of tested genes as background. For the sex-specific comparisons, global concordance and discordance of gene expression changes between males and females were quantified using the RRHO correlation (ρ), the proportion of genes changing in the same direction (sign concordance), and 95% CI.

Gene ontology enrichment analysis was performed with the R package clusterProfiler. For mouse RRHO, discordant genes in the BNST and NAc were set against a background of all genes overlapping between sexes for each region for gene ontology enrichment (enrichGO, cutoff = 0.1). For the interspecies comparison in the NAc, gene set enrichment analysis (gseGO) was used to account for significance and directionality (*P* cutoff = 0.05). R package GoSemSim was used to determine semantic similarity between significantly enriched (*P* < 0.05) human and mouse GO terms from NAc data. Dotplots include top human terms with highest similarity to a mouse term (>0.6), with the point color and size for each term determined by them mouse term’s *P*-value and enrichment score respectively.

Weighted gene coexpression network analysis (57) via the WGCNA package was used to identify patterns of gene coexpression and determine functionally relevant networks of genes in an unsupervised manner. Samples were stratified by region, as well as adulthood condition to avoid skewing of module detection. Pearson correlation of module eigengene (ME) values to traits was used to identify modules associated with PNELS, weighed aggregate score of DeepOF behaviors, and sex. For select modules with associations (R > 0.5, *P* < 0.1) to any of the traits, linear regression was used to assess how well sex, PNELS, and ME value predict the behavioral score.

## Supplementary Material

Appendix 01 (PDF)

## Data Availability

All data are included in the article and/or *SI Appendix*.
