# Supplementary material for: Multilevel sex-influenced neurobiological signatures of early life adversity
Source: Proc Natl Acad Sci U S A. 2026 Aug 27;123(35):e2603982123. doi: 10.1073/pnas.2603982123 (PMC13535199; doi:10.1073/pnas.2603982123)
Supplement: Supplementary file 1 [file pnas.2603982123.sapp.pdf]

Supplement

Supplemental methods

MEMRI image preprocessing

As the MEMRI data were acquired with a surface coil, correction of intensity inhomogeneities was essential. To ensure that bias field estimation was restricted to brain tissue, we generated a brain mask in native space. Because strong Mn^{2+} accumulation occurs in specific regions (e.g., cerebellum, olfactory bulb, and colliculi) and may distort bias field estimation, these regions were excluded from the mask.

The preprocessing pipeline was implemented in two passes. In the first round, images were only coarsely bias corrected, and the resulting masks were used to refine the second round of corrections. Preprocessing steps were performed using custom scripts and MATLAB R2024b and SPM12 (www.fil.ion.ucl.ac.uk/spm), if not otherwise stated.

a) Native mean MEMRI images were first coregistered to Hikishima space, followed by N4 bias correction (using 3D Slicer (vers. 5.8.1, www.slicer.org)) without a mask.
b) A preliminary brain mask was generated by segmenting the MEMRI images in SPM with the Hikishima template, summing the GM and WM compartments, and binarizing the result.
c) Individual masks were combined across subjects to create a group mask. Voxels were retained if they were included in at least two subjects.
d) This group mask was applied for brain extraction. A second segmentation was then performed using modified Hikishima templates in which ventricular and cortical CSF were separated, and brain extraction was repeated with this refined mask.
e) Brain-extracted images were normalized to the Allen Brain Atlas (ABA; AVGT.nii) using the "old normalization" function in SPM12.
f) The deformation fields were inverted to enable registration of ABA images into individual native space. Using this approach, we generated subject-specific ABA masks excluding the cerebellum, olfactory bulb, and inferior colliculi (defined via their ROI identifiers in ANO.nii).

In the second run of the pipeline, the original MEMRI images were bias corrected again (3D Slicer), this time using the refined ABA-derived brain masks in subject's native space. Steps (d) and (e) were then repeated on these newly corrected images. Finally, data were smoothed using a kernel of 2 mm.

DeepOF behavioral analysis

Statistical Analysis

The behavioral clustering associated analyses were performed in Python (v3.10) using NumPy, pandas, scikit-learn, SciPy, UMAP-learn, HDBSCAN, scikit-posthocs, statsmodels, NetworkX, matplotlib, and seaborn libraries.

Data Preprocessing

Raw behavioral tracking data were binned into 2-second intervals and aggregated per animal bz computing the mean within each bin. Eleven behavioral features from DeepOF were retained for further analysis: "nose2body", "nose2tail", "sniffing", "moving", "stat-passive", "stat-active", "stat-lookaround", "immobility", "sniff-arena", "climb-arena", "following". The dataset was filtered to the 10-minute session duration, sorted by animal identifier and interval, and confirmed to contain no missing values after zero-imputation of residual gaps.

Dimensionality Reduction and Clustering

Features were standardized to zero mean and unit variance prior to dimensionality reduction. UMAP(umap-learn) was applied to produce a 2-dimensional embedding using Euclidean distance (random seed=42). Optimal hyperparameters for UMAP and HDBSCAN were selected by exhaustive grid search over 504 combinations. Each combination was scored on four metrics: the Density-Based Clustering Validation index (DBCV; weight 0.40), silhouette score (o.25), noise fraction penalty (0.20), and set function preference for biologically interpretable solutions in the range of 4–10 clusters (0.15). The parameters were normalized to [0,1] and combined into a composite score. UMAP embeddings were cached across HDBSCAN evaluations, scores were computed on subsamples of 10 000 points. The top ranked parameter set (UMAP: n_neighbors = 10, min_dist = 0.05; HDBSCAN: min_cluster_size = 700, min_samples = 20) yielded 6 behavioral clusters with a noise fraction of 4.3%. Noise-labeled observations were excluded from all subsequent analyses.

Cluster Validation

Internal validity was assessed on the final 2-dimensional embedding: DBCV = 0.058, silhouette score = 0.462, Davies–Bouldin index = 0.703, and Calinski–Harabasz index = 16,125.8. Mean and median HDBSCAN membership probabilities were 0.812 and 0.869, respectively.

Cluster Composition and Behavioral Profiles

The distribution of experimental condition and sex across clusters was tested with Pearson chi-square tests of independence, with effect sizes quantified by Cramér's V. Cluster membership differed significantly by condition ($\chi^2 = 279.28$, $p = 2.84 \times 10^{-58}$, V = 0.144) and sex ($\chi^2 = 471.67$, $p = 1.04 \times 10^{-99}$, V = 0.187). A multinomial logistic regression model was fitted with cluster membership as the outcome and condition, sex, and their interaction as predictors.

Differences in each behavioral feature across clusters were assessed with Kruskal–Wallis H-tests, with p-values corrected using the Benjamini–Hochberg FDR procedure ($\alpha = 0.05$). Effect sizes were estimated as $\eta^2 = (H - k + 1)/(n - k)$. All eleven features showed significant differences after FDR correction (η^2 range: 0.147–0.738). Pairwise post-hoc comparisons used Dunn's test with FDR-BH correction.

Within each cluster, the effects of condition and sex on the multivariate behavioral profile were tested with PERMANOVA (999 permutations, Euclidean distance; skbio), with p-values FDR-corrected across clusters and factors. Both condition and sex were significant within all six clusters (pseudo-F range: 4.49–23.55).

Behavioral Entropy

Shannon entropy was computed per animal over cluster occupancy proportions. Group differences were evaluated by two-way ANOVA and, for within-sex condition comparisons, by Mann–Whitney U test. A Kruskal–Wallis omnibus test across all four condition × sex groups was significant (H = 22.51, p < 0.00) followed by Dunn post-hoc comparisons with FDR-BH correction.

Individual-Level Behavioral Scoring

To link behavioral state structure to downstream biological readouts, clusters were first ranked by a composite cluster k distinctiveness score $D_{(k)}$ integrating: (a) behavioral separation, defined as fraction of significant pairwise Dunn comparisons ($p < 0.05$); (b) effect size, representing normalized mean absolute deviation of cluster feature means from the grand mean; (c) PERMANOVA sensitivity, calculated as normalized sum of pseudo-F statistics for significant condition and sex effects; and

optionally (d) biomarker linkage, represented as mean |ρ| to external biomarker data (set to 0 in the current analysis). Components were normalized to [0, 1] and combined with weights of 0.35, 0.30, 0.20, and 0.15, respectively. The top three clusters D values were: Cluster 3 = 0.608, Cluster 1 = 0.552, Cluster 2 = 0.508. All three were designated as the most experimentally informative behavioral states.

Per-animal behavioral scores were then derived in two complementary forms. First, raw cluster occupancy proportions:

$$p(i,k) = \frac{n(i,k)}{N(i)}$$

were computed for each animal i and cluster k, where $n(i,k)$ is the number of intervals in cluster k and $N(i)$ is the total non-noise intervals. These proportions represent the fraction of the session an animal spent in each behavioral state. Second, cluster-weighted scores were computed as:

$$S(i,k) = p(i,k) \times D(k)$$

weighting occupancy by each cluster's experimental relevance in a manner analogous to principal component loadings. An aggregate score was computed according to following formula:

$$S\_agg(i) = \frac{\sum_{k \in \mathrm{top}-3} p(i,k) \times D(k)}{\sum_{k \in \mathrm{top}-3} D(k)}$$

as a single normalized summary variable across the three most informative clusters, serving as the primary behavioral predictor for downstream correlation analyses and as the closest direct analogue to a PC1 score. The aggregate score was employed as a composite predictor when cluster scores were collinear.

Significance Thresholds and Reporting Conventions

Throughout the analysis, statistical significance was defined at $\alpha = 0.05$. Significance symbols follow the convention: *** p < 0.001, ** p < 0.01, * p < 0.05, # p < 0.10, ns = not significant. All reported p-values for tests involving multiple comparisons reflect FDR-BH correction unless otherwise stated.

Supplementary Figure 1. Cohort timelines

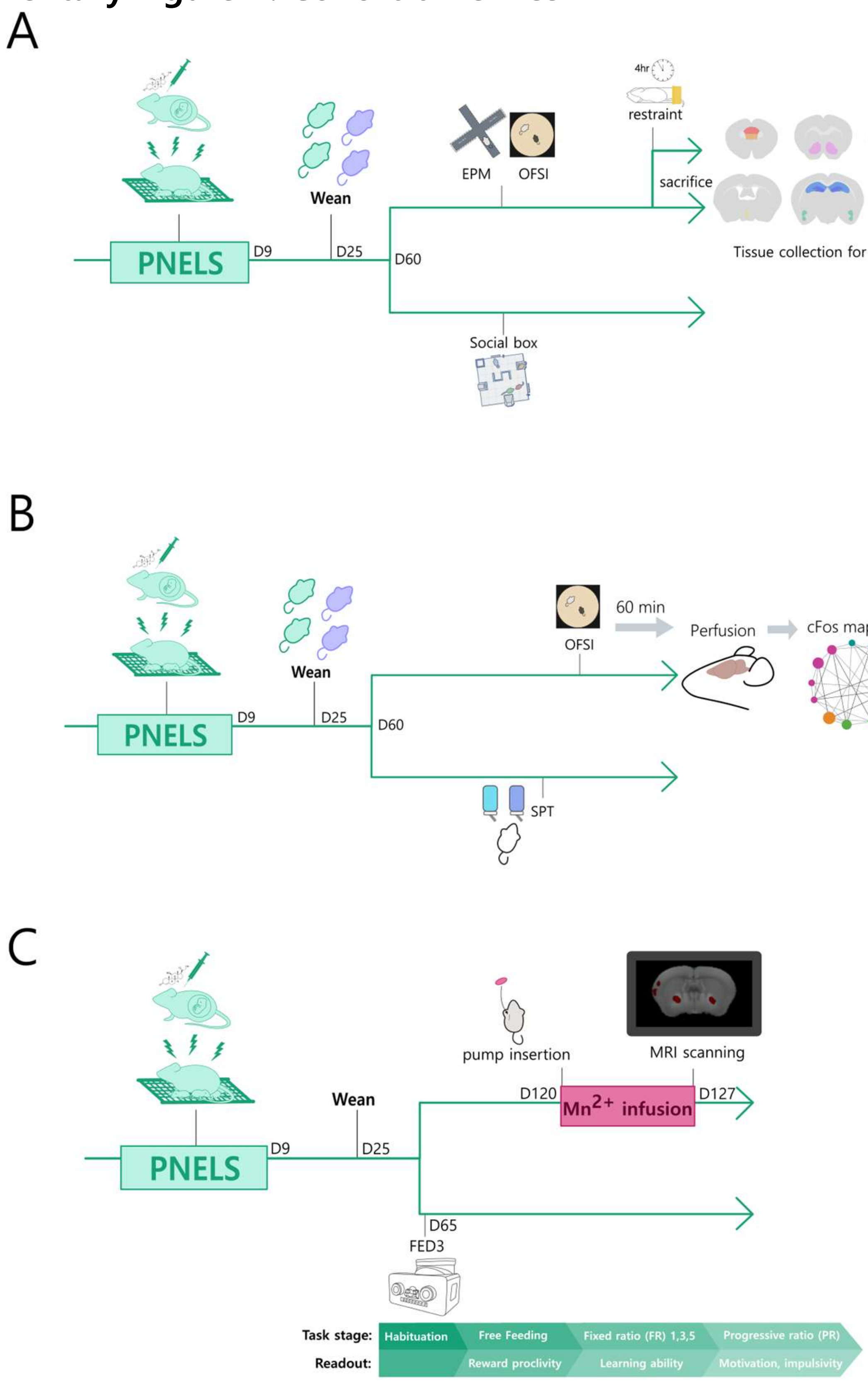

Supplementary Figure 1. Experimental timelines for each cohort. Behavioral phenotyping and bulk RNA sequencing were done in the first cohort (**A**), behavior and cFos mapping in the second (**B**), and MRI in the 3rd (**C**). Each timeline bifurcation represents a different subset of animals.

Supplementary Figure 2. Clustering of DeepOF behaviors

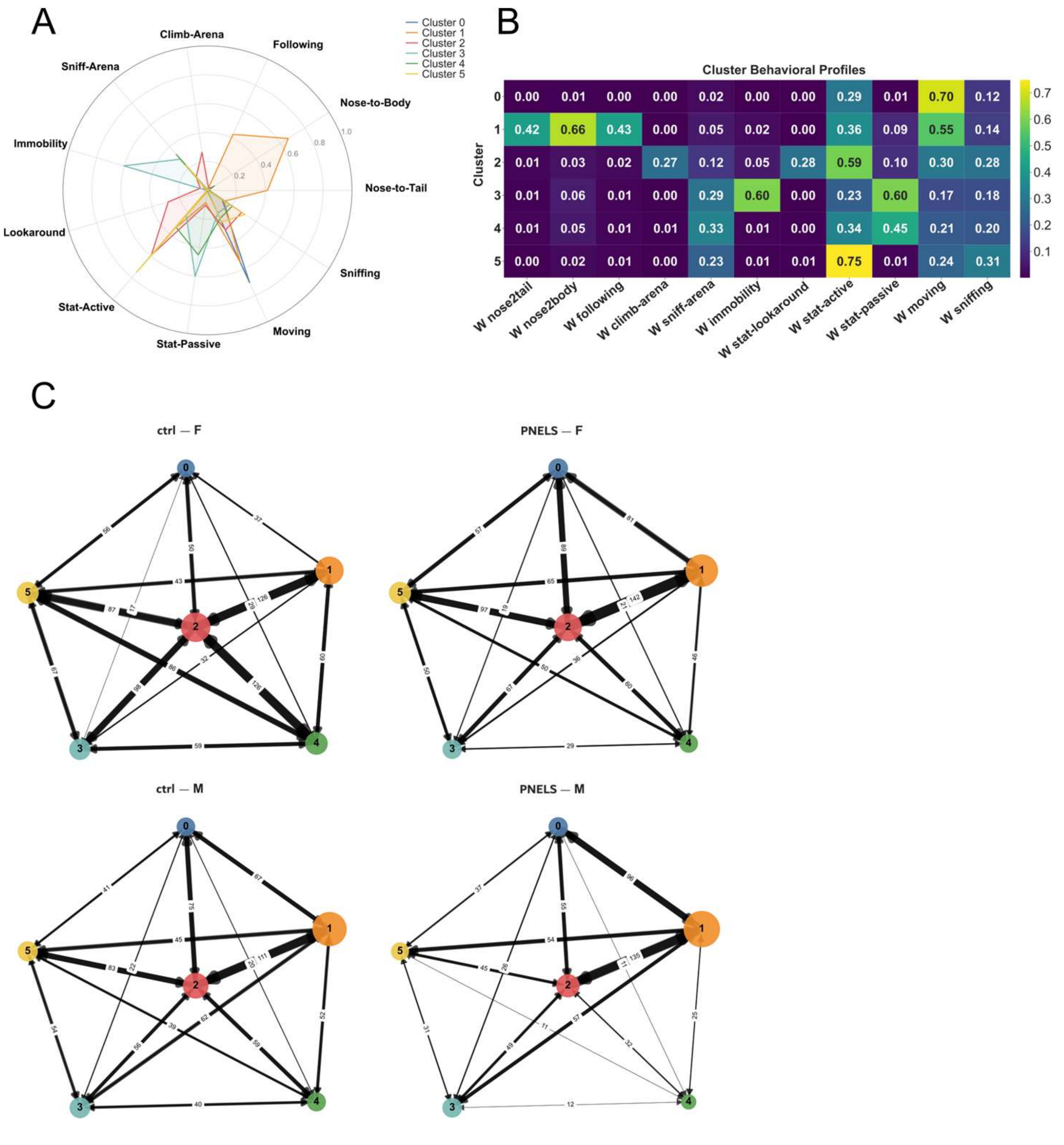

Supplementary Figure 2. Radar plot showing the mean normalized proportion of time spent in each behavioural state of the "W" animal for each of the 6 (0-5) clusters identified by UMAP dimensionality reduction followed by HDBSCAN density-based clustering (**A**). Heatmap of mean behavioral state proportions per cluster (rows: clusters 0-5; columns: behavioural features), with cell values reporting the mean time fraction and color intensity reflecting the relative engagement (yellow =high, purple = low) (**B**). Cluster Transition Network for each experimental group (ctrl-F, PNELS-F, ctrl-M, PNELS-M). Nodes represent behavioural clusters (colored and sized by cluster identity), and directed edges represent observed sequential transitions between behavioural states across consecutive 2-scond intervals. Edge weights reflect transition probabilities. Node size is proportional to cluster occupancy within each group (**C**).

Supplementary Figure 3. Individual and social behavioral variables from the DeepOF analysis in control and PNELS cohorts

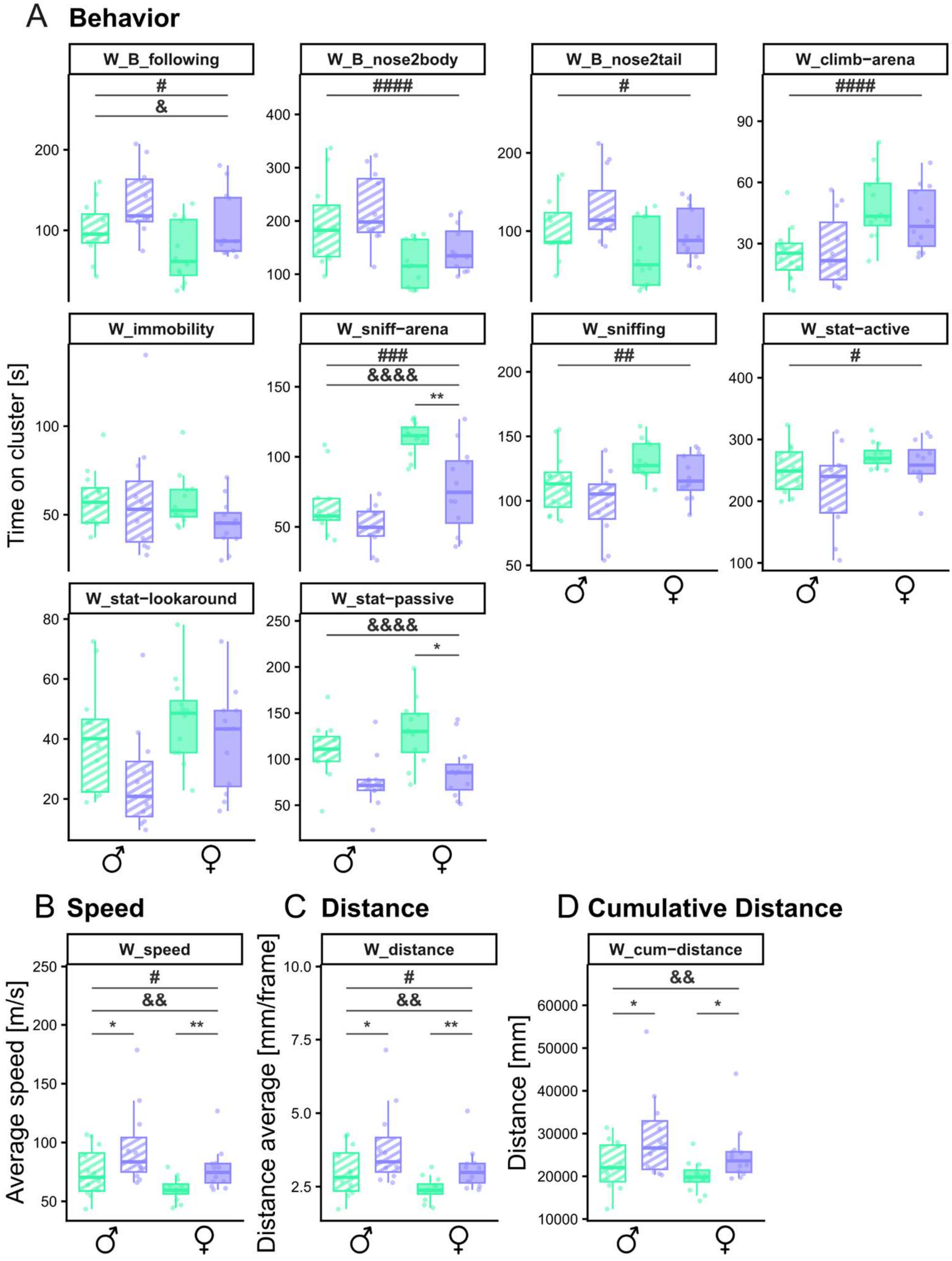

Supplementary Figure 3. Box plots represent DeepOF behavioural variables per group and highlight main effects of sex (#) and condition (&). "W_B_following" showed significant main effects of both condition ($F(1,43) = 9.20$, $p = 0.014$) and sex ($F(1,43) = 6.80$, $p = 0.021$), with no significant interaction ($F(1,43) = 0.025$, $p = 0.998$). No post-hoc pairwise comparisons reached significance after FDR correction. "W_B_nose2body" showed a highly significant main effect of sex ($F(1,43) = 18.66$, $p < 0.0001$), but no significant effect of condition ($F(1,43) = 2.88$, $p = 0.134$), no interaction ($F(1,43) = 7.15 \times 10{-}6$, $p = 0.998$), and no significant post-hoc pairwise comparisons **(A)**. "W_B_nose2tail" showed a significant main effect of sex ($F(1,43) = 7.85$, $p = 0.015$), with a trend toward a condition effect ($F(1,43) = 4.65$, $p = 0.073$), no interaction ($F(1,43) = 1.59 \times 10{-}3$, $p = 0.998$), and no post-hoc pairwise comparisons significance. "W_climb-arena" showed a significant main effect of sex ($F(1,43) = 17.20$, $p < 0.0001$) but no effect of condition ($F(1,43) = 0.32$, $p = 0.574$) and no interaction ($F(1,43) = 0.797$, $p = 0.998$). "W_immobility" showed no significant main effects of (condition: $F(1,43) = 1.16$, $p = 0.319$; sex: $F(1,43) = 1.29$, $p = 0.263$; interaction: $F(1,43) = 1.24$, $p = 0.998$). "W_sniff-arena" showed highly significant main effects of both condition ($F(1,43) = 17.50$, $p < 0.0001$) and sex ($F(1,43) = 37.37$, $p < 0.001$), with no significant interaction ($F(1,43) = 3.31$, $p = 0.758$). Post-hoc testing revealed that ctrl_F showed significantly less arena sniffing than PNELS_F ($p = 0.006$). "W_sniffing" showed trending main effect of condition ($F(1,43) = 5.28$, $p = 0.066$) and significant effect of sex ($F(1,43) = 10.00$, $p = 0.007$), with no interaction ($F(1,43) = 0.042$, $p = 0.998$). No post-hoc significance was detected. "W_stat-active" showed a significant main effect of sex ($F(1,43) = 5.80$, $p = 0.029$) but no significant effect of condition ($F(1,43) = 2.71$, $p = 0.134$) and no interaction ($F(1,43) = 0.420$, $p = 0.998$). "W_stat-lookaround" showed only a trend toward significance for both condition ($F(1,43) = 4.29$, $p = 0.074$) and sex ($F(1,43) = 4.11$, $p = 0.061$), with no interaction ($F(1,43) = 0.479$, $p = 0.998$). "W_stat-passive" showed a highly significant main effect of condition ($F(1,43) = 18.83$, $p < 0.001$) but no significant effect of sex ($F(1,43) = 3.11$, $p = 0.094$) and no interaction ($F(1,43) = 0.202$, $p = 0.998$). Post-hoc testing revealed that PNELS_F showed significantly less passive stationary behavior than ctrl_F (Tukey HSD, $p = 0.037$). "W_speed" showed significant main effects of condition (Kruskal–Wallis, $H(1) = 9.00$, $p = 0.003$) and sex ($H(1) = 4.99$, $p = 0.025$). Post-hoc Dunn tests revealed that ctrl_M moved faster than PNELS_M ($p = 0.024$), ctrl_F moved faster than PNELS_F ($p = 0.010$) (**B**). "W_distance" showed identical effects to W_speed as expected from their mathematical relationship: significant main effects of condition ($H(1) = 9.00$, $p = 0.003$) and sex ($H(1) = 4.99$, $p = 0.025$), with the same post-hoc pattern (ctrl_M vs. PNELS_M: $p = 0.024$; ctrl_F vs. PNELS_F: $p = 0.010$) (**C**). "W_cum-distance" showed a significant main effect of condition ($H(1) = 7.89$, $p = 0.005$) but not sex ($H(1) = 2.28$, $p = 0.131$). Post-hoc Dunn tests revealed that PNELS animals accumulated greater total distance than controls in both males (ctrl_M vs. PNELS_M: $p = 0.036$) and females (ctrl_F vs. PNELS_F: $p = 0.013$) (**D**).

Supplementary Figure 4. Classical behavioral assessments

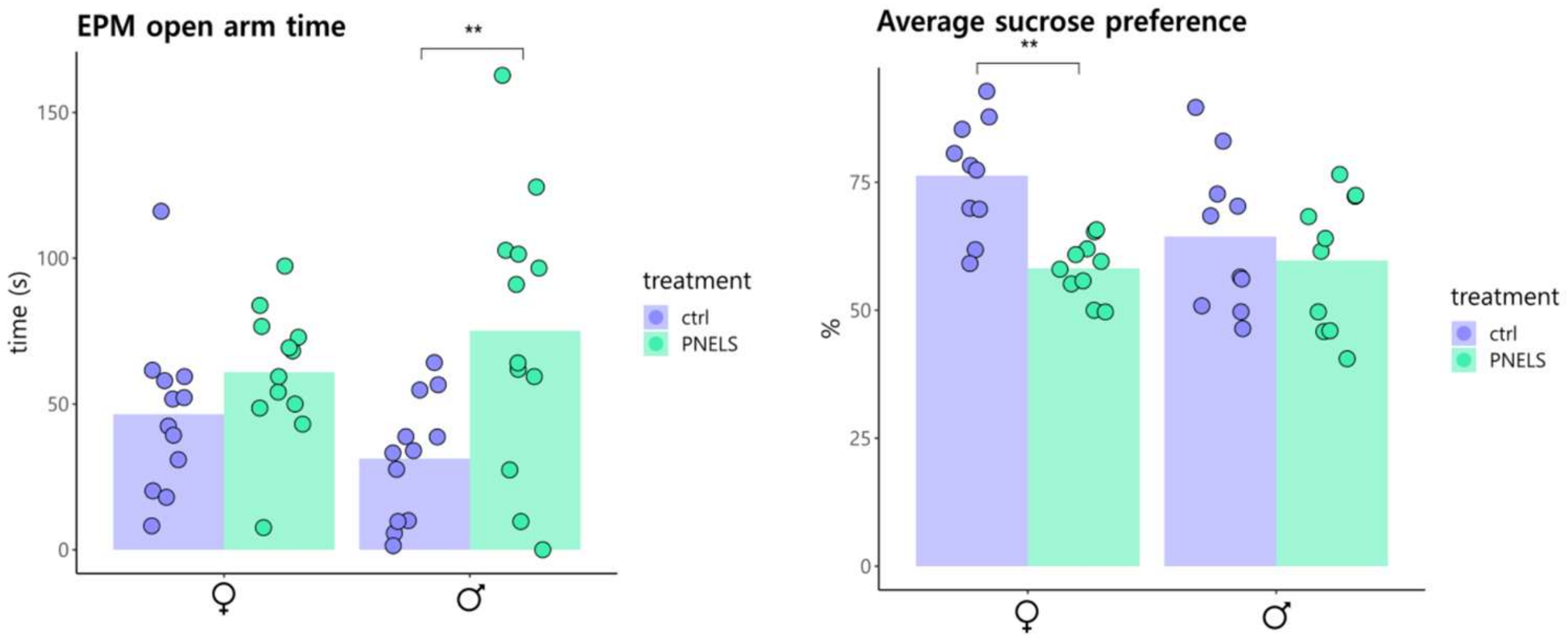

Supplementary Figure 4. Classical assessments of stress-related behaviors did not capture nuanced and sex specific effects of developmental stress (**EPM**: treatment: $F(1, 44) = 10.093$, $p = 0.00272$; sex: ns; sex:PNELS: ns; Post-hoc Tukey: M ctrl vs PNELS: $p = 0.00798$. **SPT**: treatment: $F(1, 36) = 9.526$, $p = 0.00388$; sex: ns; sex:PNELS: ns; Post-hoc Tukey: F ctrl vs PNELS: $p = 0.00717$).

Supplementary Figure 5. MEMRI stress effects and validation of lack of structural confounds

A

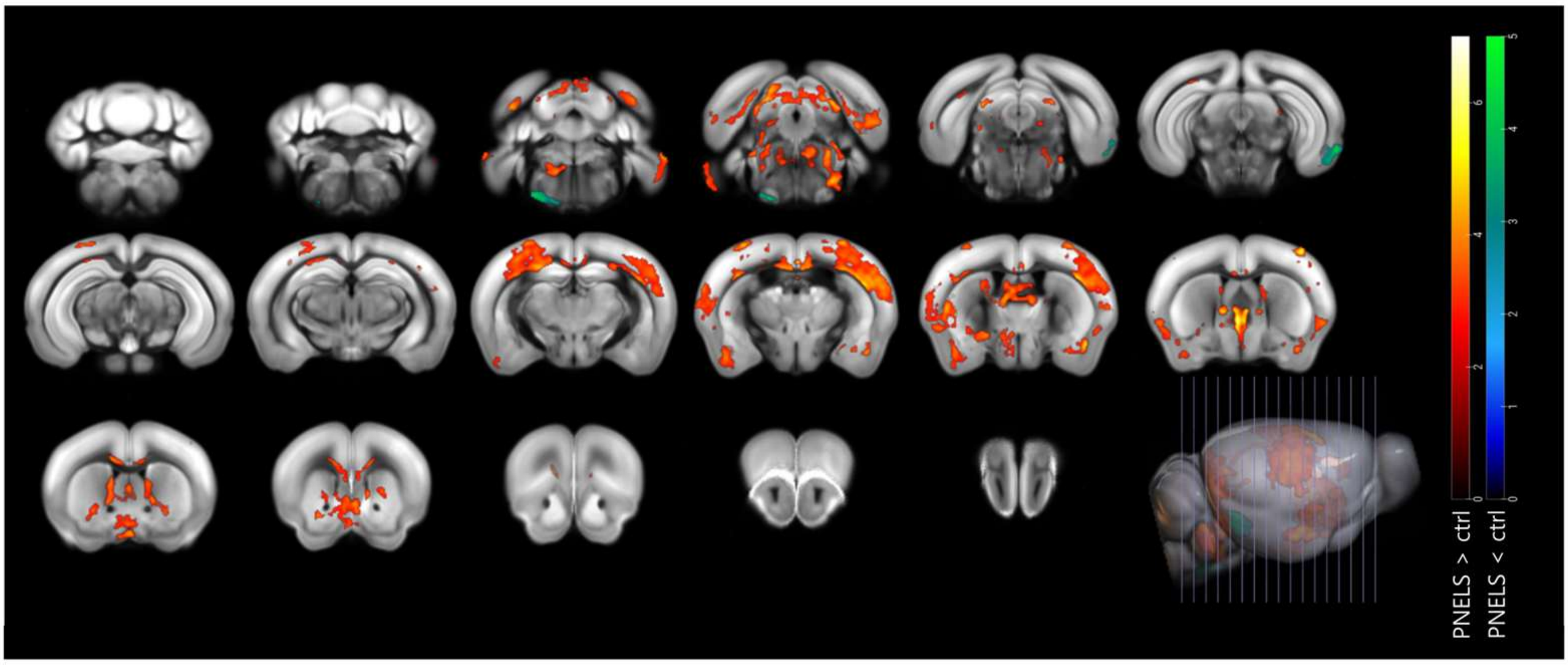

B

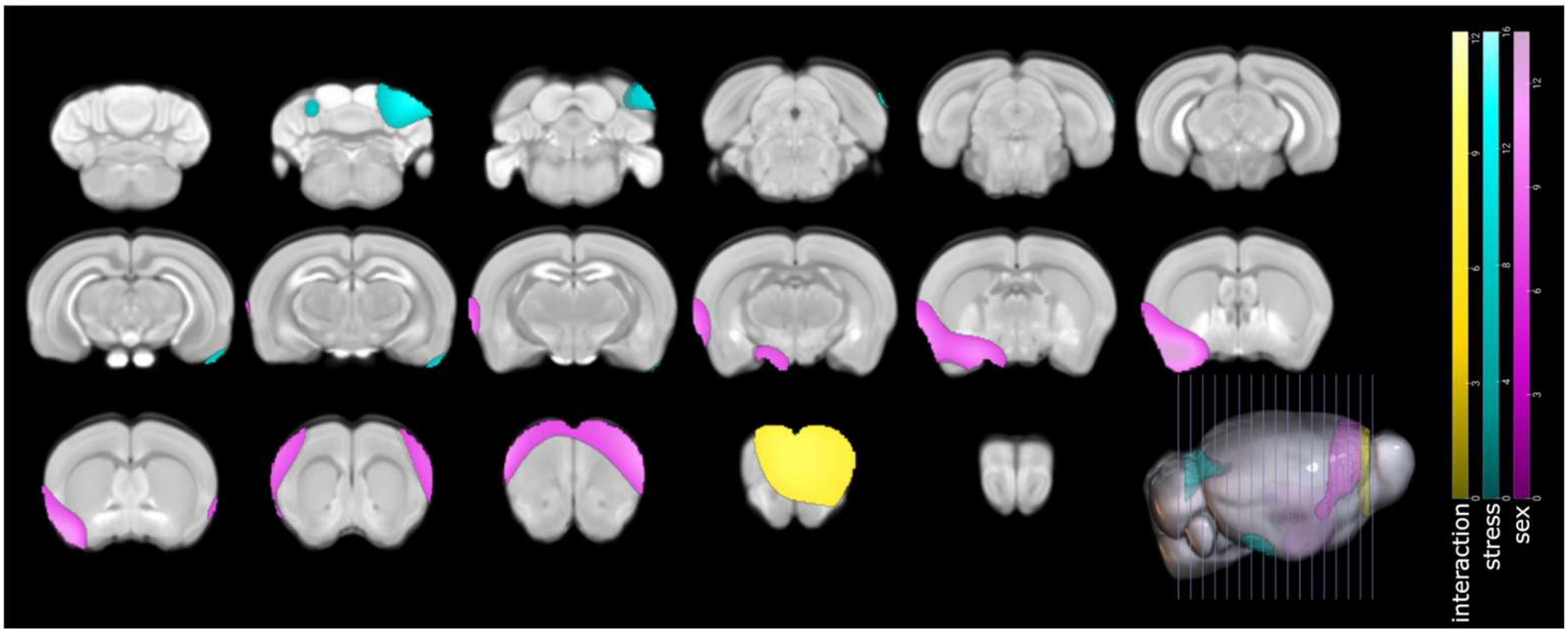

Supplementary Figure 5. The main effect of stress complements interaction in many regions, such as Superior Colliculus, Entorhinal areas, Lateral Septum, Medial Preoptic Area, cortices (primary somatosensory, Posterolateral visual area, Agranular Insula posterior part), Endopiriform nucleus ventral part, and central amygdala. Stress predominantly increased Mn^{2+} intensity. Statistical maps are presented with clusters surviving FDR cluster correction ($p_{FDR,cluster} < 0.05$), using a collection threshold of uncorrected $p < 0.005$ (**A**).
Comparison of the logarithmic jacobian determinants (JD) in a 2 x 2 factorial design with factors “sex” and “stress”, with global JD intensity per subject added as a nuisance regressor. Main effects and interaction are shown as F-maps. At the very lenient statistical threshold of uncorrected $p < 0.01$, we found sex specific differences in the rostral primary and secondary motor cortex and in left ventrolateral brain regions including the piriform area and olfactory tubercle. In comparison with the MEMRI results, only a small cluster in the left frontal motor cortex would show difference in both analyses. The lack of colocalized difference for DBM and MEMRI analyses might suggest that the observed intensity difference for MEMRI is not causally linked to morphological differences (**B**).

Supplementary Figure 6. Exemplary photomicrographs of cFos stainings

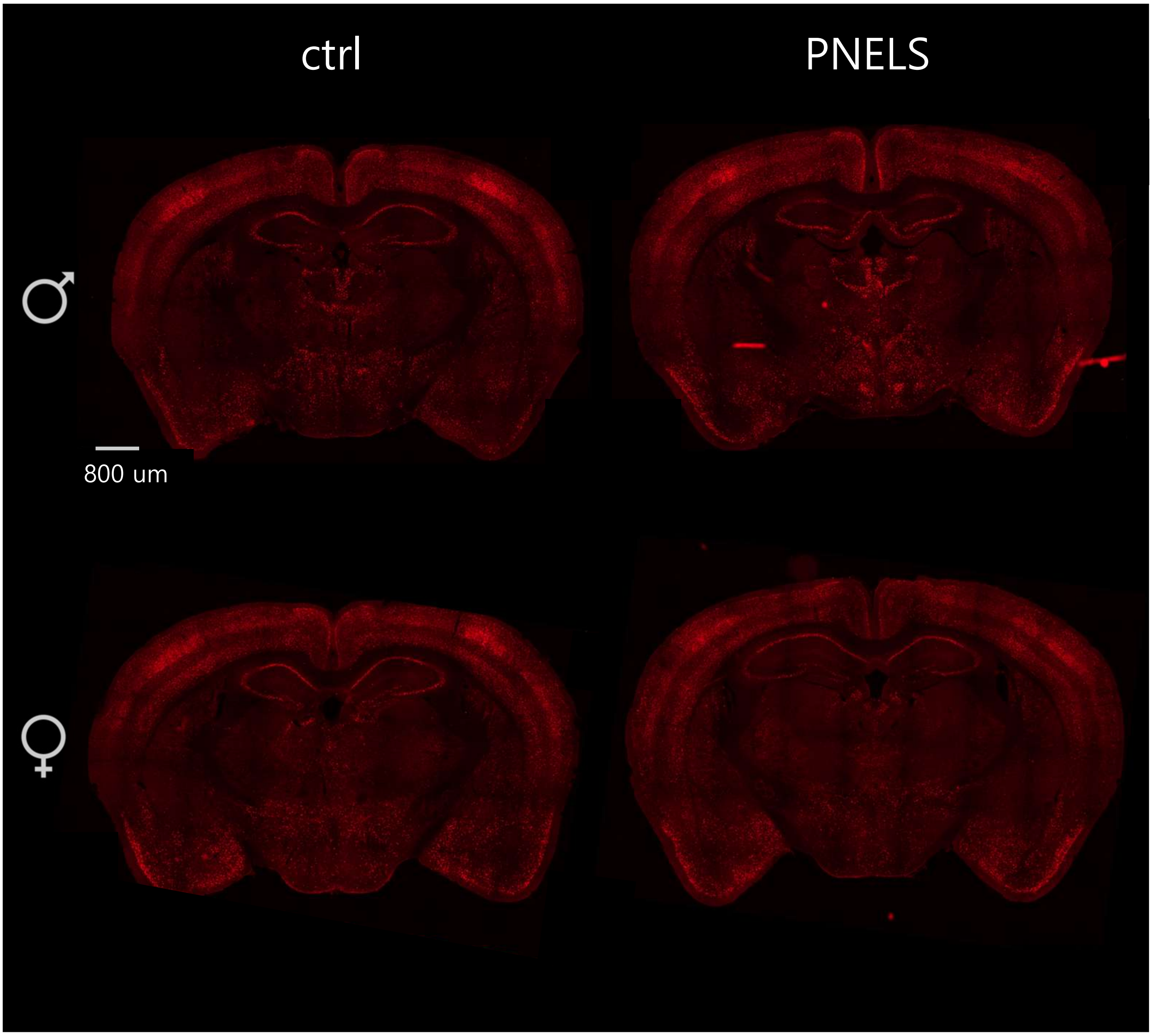

Supplementary Figure 6. Images of sections stained for cFos, showcasing density in dorsal hippocampal and amygdalar sections for an individual each from group: ctrl M (**A**), ctrl F (**B**), PNELS M (**C**), and PNELS F (**D**)

Supplementary Figure 7A: cFos relative density
ctrl vs PNELS
Females

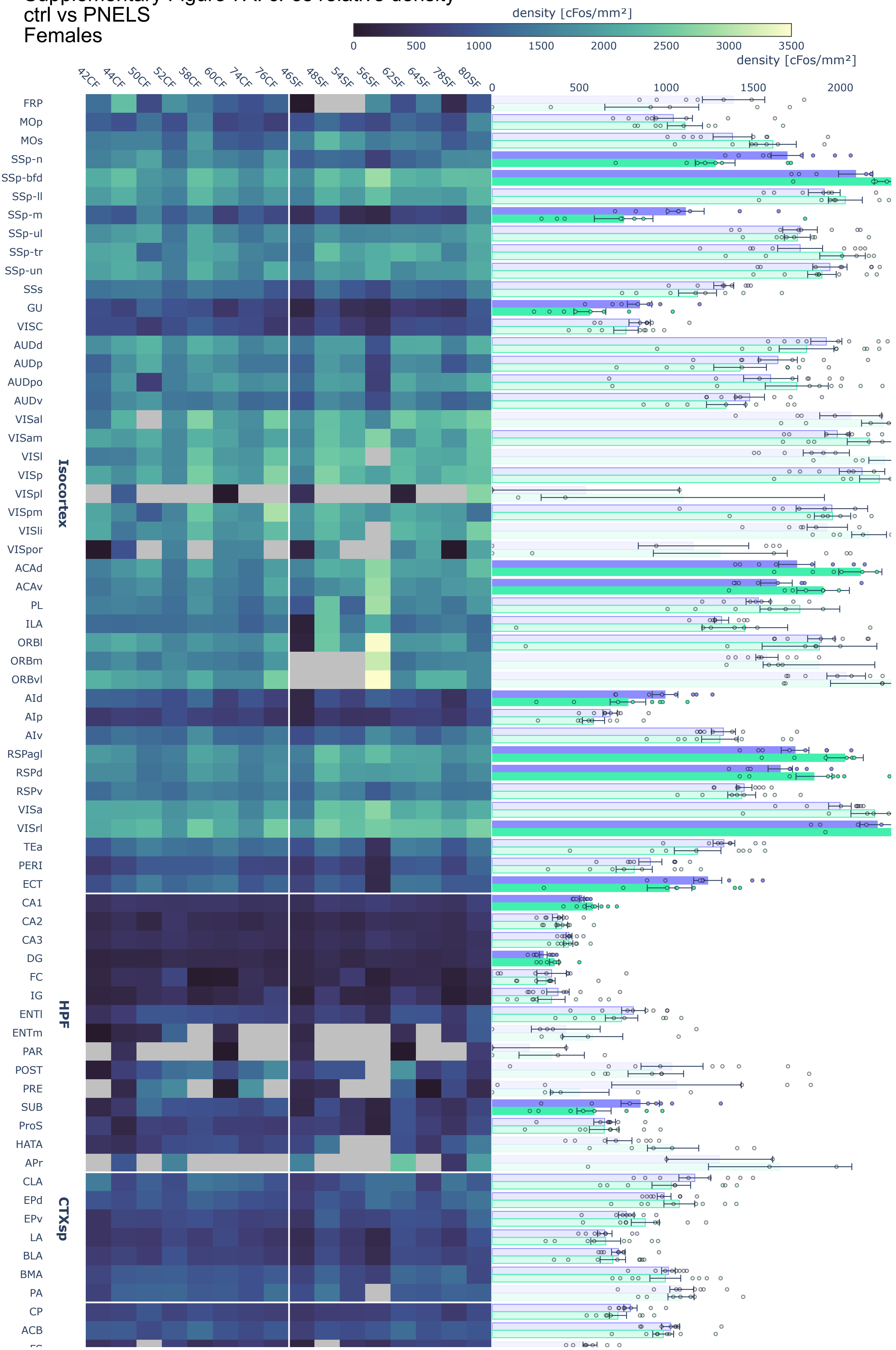

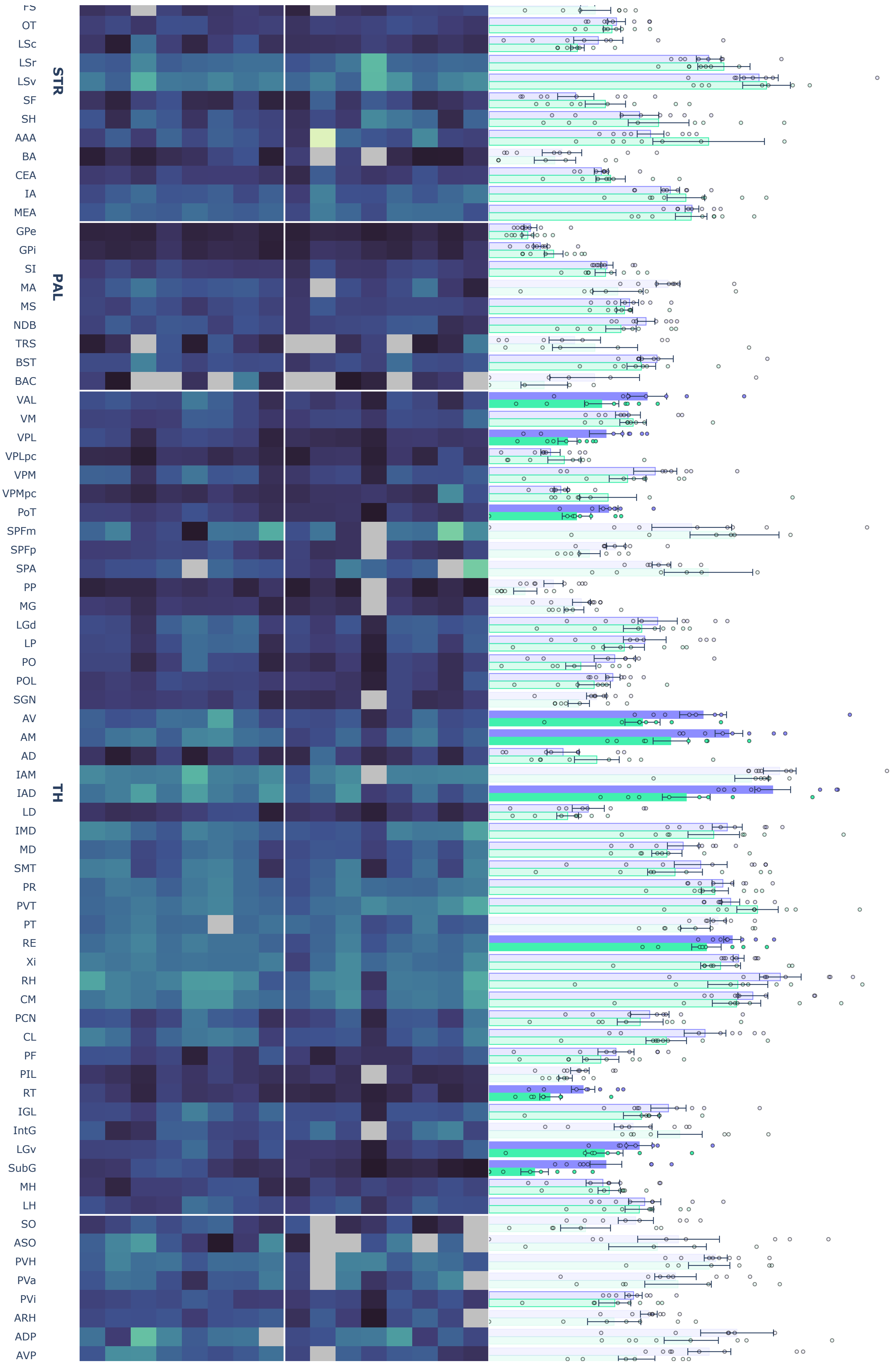

FS
OT
LSc
LSr
LSv
SF
SH
AAA
BA
CEA
IA
MEA
STR
GPe
GPi
SI
MA
MS
NDB
TRS
BST
BAC
PAL
VAL
VM
VPL
VPLpc
VPM
VPMpc
PoT
SPFm
SPFp
SPA
PP
MG
LGd
LP
PO
POL
SGN
AV
AM
AD
IAM
IAD
LD
IMD
MD
SMT
PR
PVT
PT
RE
Xi
RH
CM
PCN
CL
PF
PIL
RT
IGL
IntG
LGv
SubG
MH
LH
TH
SO
ASO
PVH
PVa
PVi
ARH
ADP
AVP

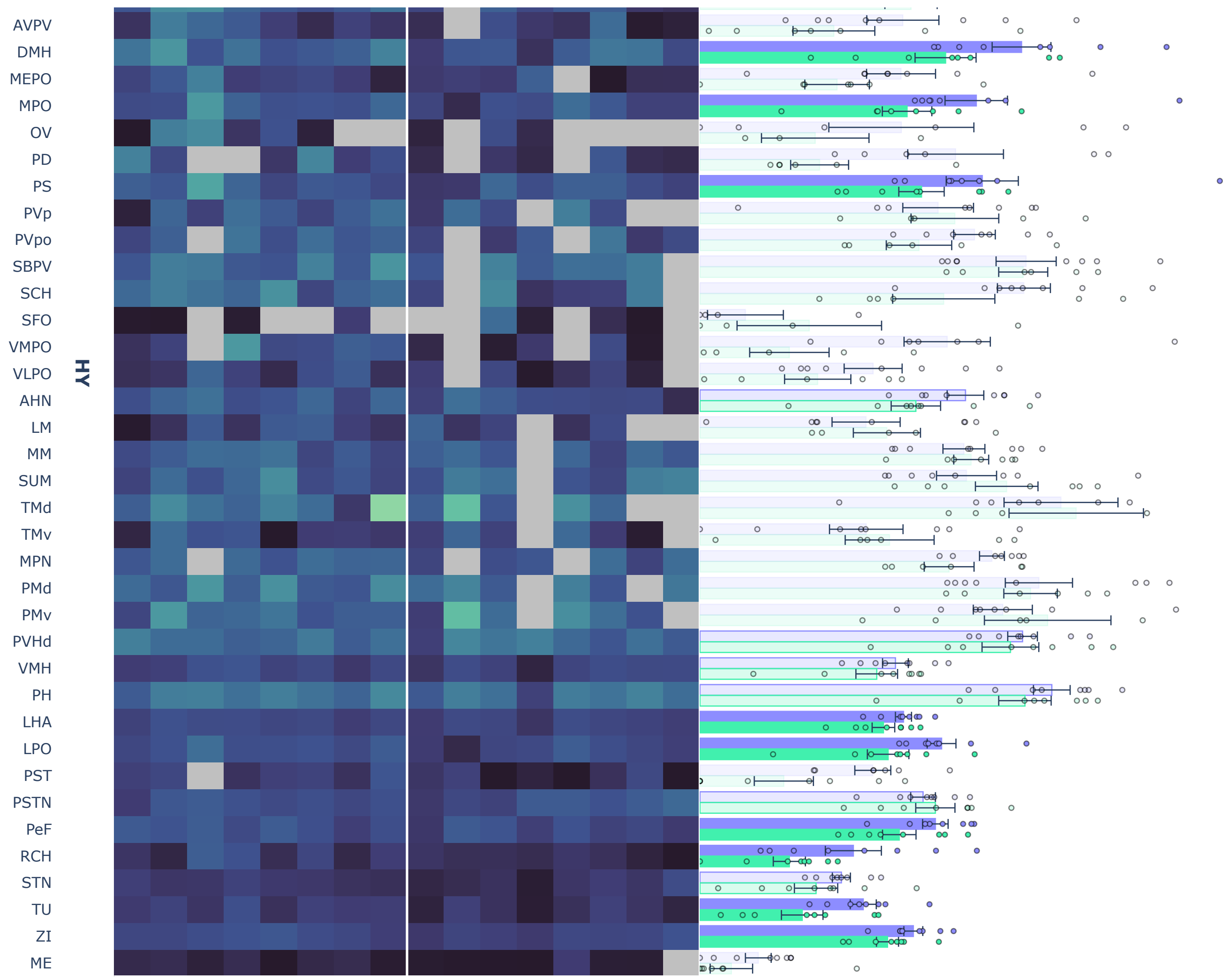
AVPV
DMH
MEPO
MPO
OV
PD
PS
PVp
PVpo
SBPV
SCH
SFO
VMPO
VLPO
AHN
LM
MM
SUM
TMd
TMv
MPN
PMd
PMv
PVHd
VMH
PH
LHA
LPO
PST
PSTN
PeF
RCH
STN
TU
ZI
ME
HY

Supplementary Figure 7B: cFos relative density
ctrl vs PNELS
Males

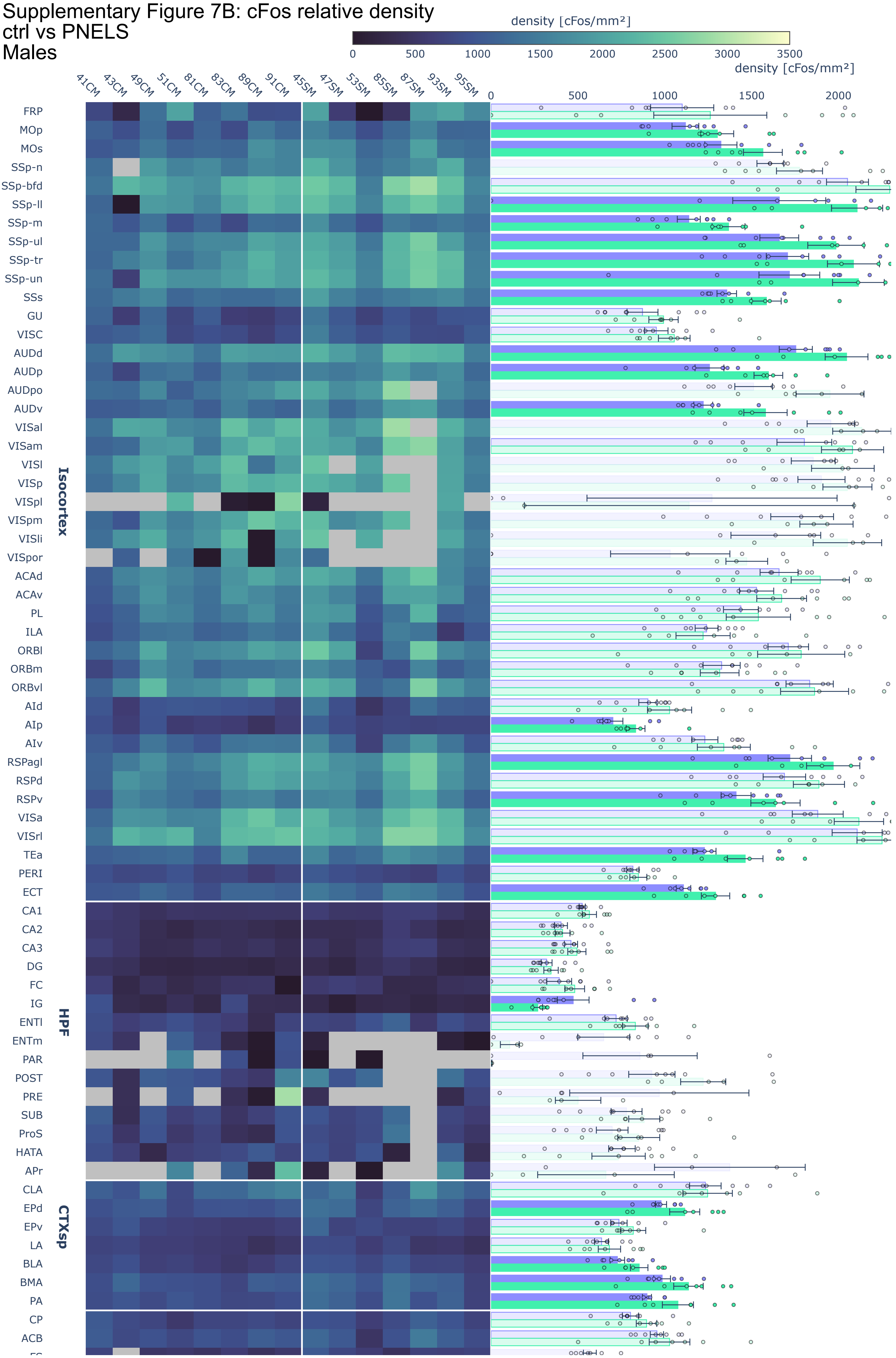

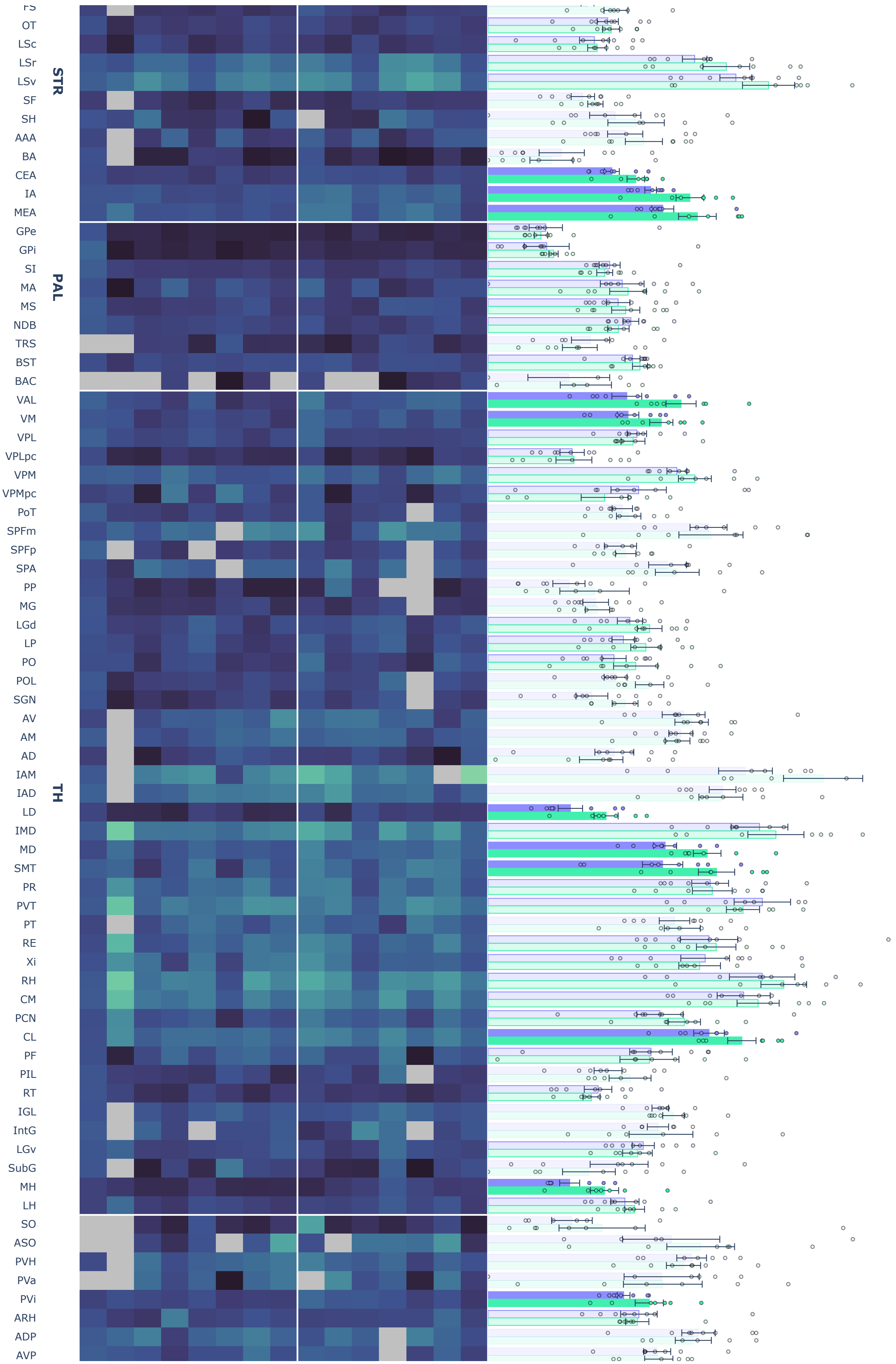
STR
PAL
TH
FS
OT
LSc
LSr
LSv
SF
SH
AAA
BA
CEA
IA
MEA
GPe
GPi
SI
MA
MS
NDB
TRS
BST
BAC
VAL
VM
VPL
VPLpc
VPM
VPMpc
PoT
SPFm
SPFp
SPA
PP
MG
LGd
LP
PO
POL
SGN
AV
AM
AD
IAM
IAD
LD
IMD
MD
SMT
PR
PVT
PT
RE
Xi
RH
CM
PCN
CL
PF
PIL
RT
IGL
IntG
LGv
SubG
MH
LH
SO
ASO
PVH
PVa
PVi
ARH
ADP
AVP

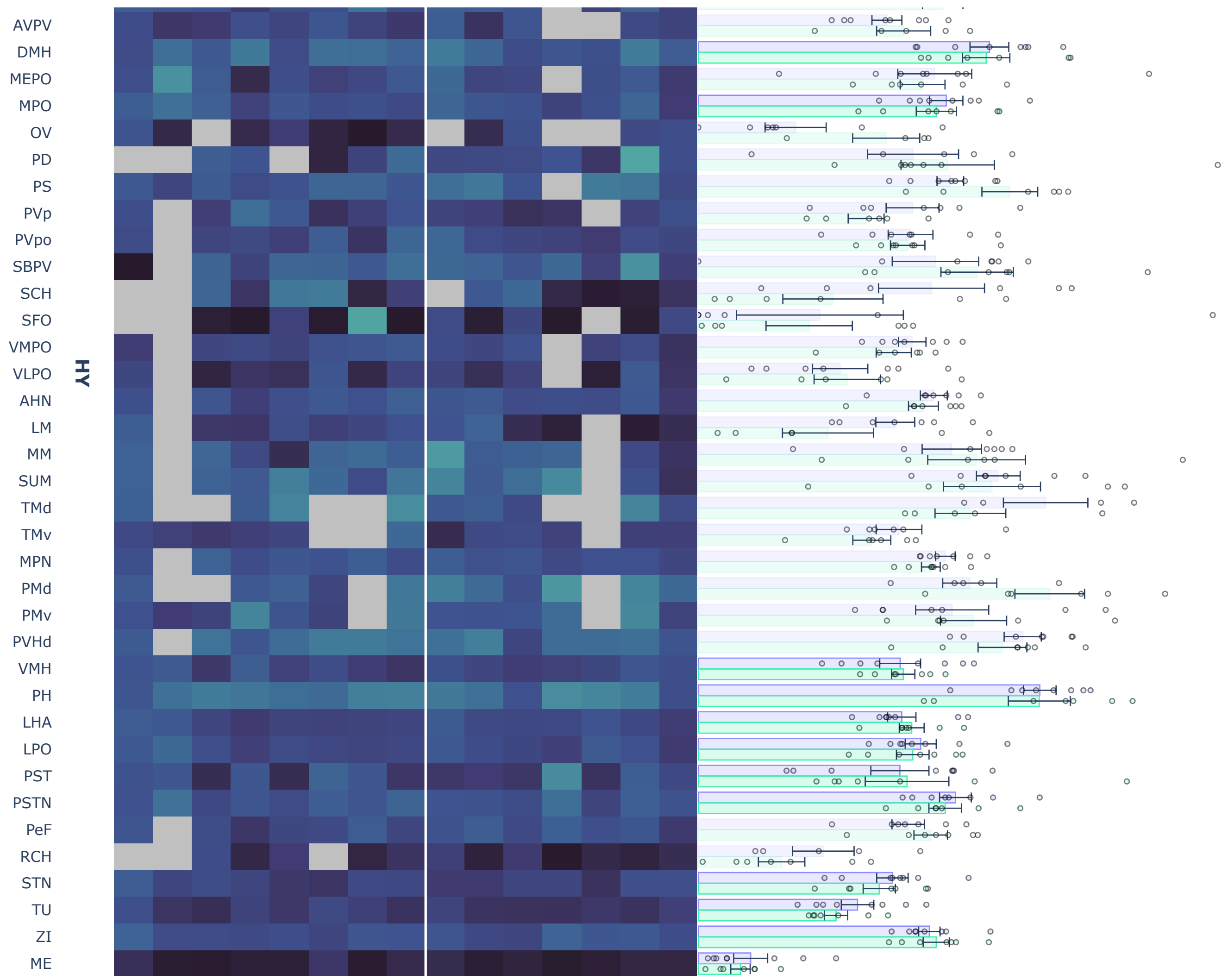

HY
AVPV
DMH
MEPO
MPO
OV
PD
PS
PVp
PVpo
SBPV
SCH
SFO
VMPO
VLPO
AHN
LM
MM
SUM
TMd
TMv
MPN
PMd
PMv
PVHd
VMH
PH
LHA
LPO
PST
PSTN
PeF
RCH
STN
TU
ZI
ME

Supplementary Figure 8. RRHO plots for all collected regions

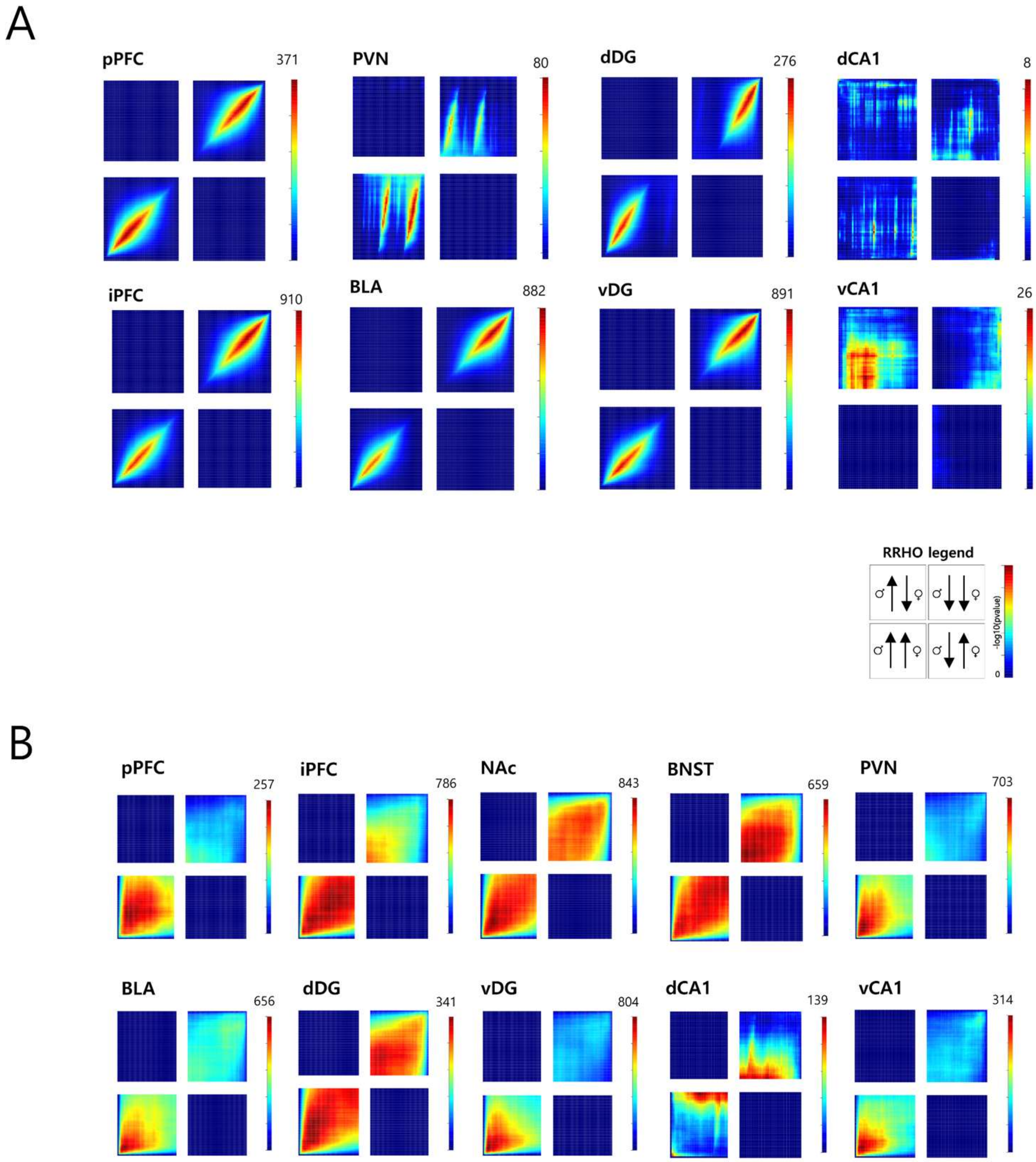

Supplementary Figure 8. RRHO comparing male and female PNELS results show mainly concordance of transcriptional signatures for all collected regions (**A**) except NAc and BNST (Fig. 4B). RRHO for restraint results show exclusive concordance between sexes, highlighting the unique sex-specificity associated with PNELS in select regions (**B**).

Supplementary Figure 9. WGCNA modules

region	condition	ME #	PNELS association		sex association		DeepOF PC1 association	
			R-value	pval	R-value	pval	R-value	pval
**BNST**	baseline	none						
	restrained	**11**					**-0.65**	**0.0006**
**NAc**	baseline	none						
	restrained	none						
**pPFC**	baseline	**7**			**0.57**	**0.004**		
		9	0.54	0.008				
	restrained	none						
**iPFC**	baseline	none						
	restrained	none						
**dDG**	baseline	none						
	restrained	none						
**vDG**	baseline	**11**	**0.52**	**0.01**				
	restrained	none						

Supplementary Figure 9. Modules found with strong and significant ($R > 0.5$, $p < 0.05$) associated to PNELS, sex, or DeepOF PC1 for regions chosen for WGCNA. Modules highlighted in green were analyzed for predictive effects on behavior (Fig. 4D).

Supplementary Table 1. genes differentially expressed in each region and for each sex (p < 0.1) due to PNELS.

region	sex	ENSEMBLID	gene name	baseMean	log2FC	lfcSE	stat	pval	padj
pPFC	F	ENSMUSG00000022013	Dnajc15	582.31698	-0.644589	0.1338069	22.43491	2.17E-06	0.0141975
pPFC	F	ENSMUSG00000036986	Pml	189.460124	-0.645894	0.1347235	23.42368	1.30E-06	0.0141975
pPFC	F	ENSMUSG00000036893	Ehmt1	980.375795	-0.596408	0.1311071	20.1488	7.16E-06	0.0311943
pPFC	F	ENSMUSG00000038351	Sgsm2	2577.44136	0.207925	0.0483727	18.43016	1.76E-05	0.0575531
pPFC	F	ENSMUSG00000027956	Tmem144	60.3503663	-0.9244	0.2267009	16.85625	4.03E-05	0.0768519
pPFC	F	ENSMUSG00000036225	Kctd1	1046.38249	0.2435067	0.0585765	17.3841	3.05E-05	0.0768519
pPFC	F	ENSMUSG00000040365	Trim41	914.687407	0.3557334	0.087224	16.81596	4.12E-05	0.0768519
pPFC	F	ENSMUSG00000045608	Dbx2	131.162582	0.6872522	0.1678705	16.10943	5.98E-05	0.0976147
pPFC	M	ENSMUSG00000004113	Cacna1b	2728.15776	-0.469881	0.0844669	32.18426	1.40E-08	0.0001755
pPFC	M	ENSMUSG00000103332	Pcdhga2	104.956429	0.7791199	0.1545868	26.21727	3.05E-07	0.0019092
pPFC	M	ENSMUSG00000030423	Pop4	109.164253	-0.966184	0.1933494	24.02725	9.50E-07	0.0039627
pPFC	M	ENSMUSG00000036893	Ehmt1	803.026573	-0.692335	0.152185	21.56065	3.43E-06	0.0085813
pPFC	M	ENSMUSG00000039637	Coro7	1012.84212	0.3810303	0.0827372	21.61166	3.34E-06	0.0085813
pPFC	M	ENSMUSG00000021990	Spata13	278.492119	-0.605794	0.1318985	20.24295	6.82E-06	0.0142274
pPFC	M	ENSMUSG00000021238	Aldh6a1	383.683069	-0.322572	0.0762614	17.82502	2.42E-05	0.0433013
iPFC	F	ENSMUSG00000015085	Entpd2	260.729268	-0.550871	0.1118147	24.78231	6.42E-07	0.0082547
iPFC	F	ENSMUSG00000024507	Hsd17b4	1042.42573	-0.314626	0.0699273	20.21116	6.93E-06	0.0445937
iPFC	F	ENSMUSG00000045917	Tmem268	194.113418	0.4846966	0.1120335	18.90428	1.37E-05	0.0589223
iPFC	F	ENSMUSG00000039307	Hexdc	238.395448	-0.561574	0.129801	17.57769	2.76E-05	0.0886781
iPFC	F	ENSMUSG00000036880	Acaa2	270.990995	-0.295888	0.0721219	17.06352	3.62E-05	0.0929851
iPFC	M	ENSMUSG00000035964	Tmem59l	1668.63544	0.5126664	0.0858398	35.93895	2.04E-09	2.55E-05
iPFC	M	ENSMUSG00000034845	Plvap	76.1215596	-1.210172	0.2050457	33.71697	6.37E-09	3.99E-05
iPFC	M	ENSMUSG00000000792	Slc5a5	177.617925	1.2646295	0.2549005	25.60239	4.20E-07	0.0017498
NAc	F	ENSMUSG00000026452	Syt2	1769.9602	0.8399179	0.1421939	34.01583	5.47E-09	7.06E-05
NAc	F	ENSMUSG00000089997	1810020O05Rik	159.874879	-1.237001	0.2184399	31.22009	2.30E-08	0.0001489
NAc	F	ENSMUSG00000056870	Gulp1	139.994183	-0.768811	0.1496003	26.4059	2.77E-07	0.0011919
NAc	F	ENSMUSG00000005716	Pvalb	378.891109	2.2230393	0.4069525	24.92249	5.97E-07	0.0015425
NAc	F	ENSMUSG00000030352	Tspan9	1076.4287	0.4128883	0.0825274	24.9501	5.88E-07	0.0015425
NAc	F	ENSMUSG00000027168	Pax6	73.4739927	1.3016866	0.2709053	22.27415	2.36E-06	0.003982
NAc	F	ENSMUSG00000053930	Shisa6	180.758453	1.878805	0.376505	22.1935	2.47E-06	0.003982
NAc	F	ENSMUSG00000095407	Tmem200c	188.085231	0.9851204	0.2052685	22.44654	2.16E-06	0.003982
NAc	F	ENSMUSG00000039747	Orai2	1516.84252	0.3357659	0.0761471	19.36552	1.08E-05	0.0154987
NAc	F	ENSMUSG00000000420	Galnt1	625.040028	-0.405542	0.096804	17.46643	2.92E-05	0.0343547
NAc	F	ENSMUSG00000031158	Timm17b	238.61162	-0.75146	0.1790108	17.52268	2.84E-05	0.0343547
NAc	F	ENSMUSG00000025425	St8sia5	260.324972	0.8740495	0.2072034	17.27775	3.23E-05	0.0347787
NAc	F	ENSMUSG00000058153	Sez6l	2089.4294	0.4845698	0.1170559	16.97994	3.78E-05	0.037553
NAc	F	ENSMUSG00000065251	Gm23971	8606.48094	-0.638222	0.1553331	16.81124	4.13E-05	0.0381119
NAc	F	ENSMUSG00000021730	Hcn1	716.122141	0.4101856	0.1024448	15.94225	6.53E-05	0.0517849
NAc	F	ENSMUSG00000029661	Col1a2	222.5398	0.775	0.1952269	15.5549	8.01E-05	0.0517849
NAc	F	ENSMUSG00000032280	Tle3	522.605503	0.4373625	0.1106084	15.59633	7.84E-05	0.0517849
NAc	F	ENSMUSG00000040649	Rimklb	519.835084	-0.546556	0.1358746	15.97578	6.42E-05	0.0517849
NAc	F	ENSMUSG00000041261	Car8	204.097024	1.5978028	0.3719801	15.64646	7.64E-05	0.0517849
NAc	F	ENSMUSG00000079109	Pms2	281.542064	-0.512976	0.1297577	15.58666	7.88E-05	0.0517849
NAc	F	ENSMUSG00000048978	Nrsn1	3006.74839	-0.462063	0.1176009	15.31035	9.12E-05	0.0561323
NAc	F	ENSMUSG00000032368	Zic1	386.426494	1.1617559	0.2867227	15.20132	9.66E-05	0.0567649
NAc	F	ENSMUSG00000026837	Col5a1	81.8124649	1.1858058	0.301449	14.84174	0.000117	0.0656933
NAc	F	ENSMUSG00000028339	Col15a1	70.3692787	1.5028918	0.3800094	14.57595	0.000135	0.0695886
NAc	F	ENSMUSG00000055489	Ano5	56.7119826	0.8965386	0.2347439	14.60254	0.000133	0.0695886
NAc	F	ENSMUSG00000031543	Ank1	1316.66119	0.4898443	0.1292842	14.18291	0.000166	0.0824437
NAc	F	ENSMUSG00000059003	Grin2a	4578.21948	0.3979879	0.1056865	14.08583	0.000175	0.0835948
NAc	F	ENSMUSG00000007594	Hapln4	741.950719	0.8889225	0.2322442	13.99457	0.000183	0.0836168
NAc	F	ENSMUSG00000031654	Cbln1	128.991755	2.5000718	0.5803308	13.95097	0.000188	0.0836168
NAc	F	ENSMUSG00000003279	Dlgap1	3698.62425	0.2931646	0.0791082	13.65148	0.00022	0.0917398
NAc	F	ENSMUSG00000032332	Col12a1	149.907783	1.0139586	0.2709499	13.70443	0.000214	0.0917398
NAc	F	ENSMUSG00000036893	Ehmt1	1046.16622	-0.652065	0.1758108	13.51543	0.000237	0.0955533
NAc	M	ENSMUSG00000087968	Gm25395	2561.7487	0.3705404	0.0549338	45.2153	1.77E-11	1.15E-07
NAc	M	ENSMUSG00000025351	Cd63	657.111191	0.3951033	0.0655784	36.1389	1.84E-09	6.01E-06
NAc	M	ENSMUSG00000003575	Crtc1	5877.86601	-0.135548	0.027078	25.07621	5.51E-07	0.0012014
NAc	M	ENSMUSG00000036309	Skp1a	3770.70849	0.1691621	0.0357364	22.21531	2.44E-06	0.0039849
NAc	M	ENSMUSG00000025066	Sfr1	880.499534	0.1891677	0.0422466	20.19596	6.99E-06	0.009143
NAc	M	ENSMUSG00000059291	Rpl11	4017.05462	0.1872448	0.0425392	19.68685	9.12E-06	0.0099438
BNST	F	ENSMUSG00000028284	Map3k7	368.369463	-0.469901	0.0940563	25.04682	5.60E-07	0.0066284
BNST	F	ENSMUSG00000113621	2900060N12Rik	45.1143967	-1.153402	0.2591425	20.29445	6.64E-06	0.0393246
BNST	F	ENSMUSG00000040649	Rimklb	440.341855	-0.656405	0.1517974	18.09782	2.10E-05	0.0828594
BNST	M	ENSMUSG00000034463	Scara3	305.46746	-0.53622	0.099525	29.08103	6.94E-08	0.0009145
BNST	M	ENSMUSG00000069895	Atxn1l	803.65079	-0.631928	0.122287	26.76012	2.30E-07	0.0015173

BNST	M	ENSMUSG00000029920	Smarcad1	437.563383	-0.426003	0.0907686	21.82305	2.99E-06	0.0098471
BNST	M	ENSMUSG00000068798	Rap1a	261.882413	0.714356	0.1513192	21.95719	2.79E-06	0.0098471
BNST	M	ENSMUSG00000071516	Hist1h2ai	98.2890785	1.1000126	0.2415155	21.12479	4.30E-06	0.0113382
BNST	M	ENSMUSG00000029577	Ube3b	1096.99536	-0.375285	0.0817174	20.56363	5.77E-06	0.012665
BNST	M	ENSMUSG00000034845	Plvap	103.27128	-1.149754	0.2702724	17.51838	2.85E-05	0.0468572
BNST	M	ENSMUSG00000073418	C4b	1007.61086	-0.669843	0.1578022	17.56797	2.77E-05	0.0468572
BNST	M	ENSMUSG00000055707	Klhl26	787.623328	-0.291175	0.0700988	17.17247	3.41E-05	0.0499657
BNST	M	ENSMUSG00000027203	Dut	225.954262	-0.450491	0.112129	15.87791	6.76E-05	0.0890073
PVN	F	ENSMUSG00000015013	Trappc2l	562.375635	-0.459713	0.0995642	21.33554	3.86E-06	0.0438117
PVN	F	ENSMUSG00000029561	Oasl2	80.116899	-1.464433	0.3378496	19.7993	8.60E-06	0.0438117
PVN	F	ENSMUSG00000071531	Gprin2	642.760374	-0.848694	0.1954949	19.61406	9.48E-06	0.0438117
PVN	M	ENSMUSG00000024330	Col11a2	266.402239	-1.120144	0.1928203	34.77055	3.71E-09	5.05E-05
PVN	M	ENSMUSG00000054728	Phactr1	2972.40805	-0.340198	0.0727266	22.40364	2.21E-06	0.0150503
PVN	M	ENSMUSG00000030452	Nipa2	418.072196	0.4872626	0.1091426	19.14043	1.21E-05	0.0551482
BLA	F	ENSMUSG00000022211	Carmil3	564.719615	-0.414297	0.0823644	25.85304	3.68E-07	0.0028392
BLA	F	ENSMUSG00000069170	Adgrv1	79.2947237	1.1262936	0.2272368	25.54148	4.33E-07	0.0028392
dDG	M	ENSMUSG00000043091	Tuba1c	165.834207	-3.032147	0.607312	22.24832	2.40E-06	0.0332017
dDG	M	ENSMUSG00000007888	Crlf1	496.226782	0.4806736	0.1155826	17.139	3.47E-05	0.0963496
dDG	M	ENSMUSG00000012296	Tjap1	334.535019	-0.388592	0.09051	18.41949	1.77E-05	0.0963496
dDG	M	ENSMUSG00000024240	Epc1	907.820594	-0.245586	0.0586619	17.56451	2.78E-05	0.0963496
dDG	M	ENSMUSG00000034845	Plvap	116.638882	-0.789715	0.1902196	17.13796	3.48E-05	0.0963496
vDG	F	ENSMUSG00000071708	Sms	2020.60108	-0.246762	0.0514948	22.73323	1.86E-06	0.0123026
vDG	F	ENSMUSG00000094777	Hist1h2ap	96.8245626	0.9535367	0.2030442	22.81153	1.79E-06	0.0123026
vDG	M	ENSMUSG00000000792	Slc5a5	118.626977	1.4244888	0.2549612	32.14573	1.43E-08	0.0001937
vDG	M	ENSMUSG00000035964	Tmem59l	3947.26702	0.414804	0.0886827	22.2725	2.37E-06	0.0160229
vDG	M	ENSMUSG00000030423	Pop4	183.594421	-0.673838	0.15095	19.9124	8.11E-06	0.036607
vDG	M	ENSMUSG00000028467	Gba2	1616.01733	0.2111666	0.0499741	17.84309	2.40E-05	0.0812381
dCA1	M	ENSMUSG00000064220	Hist2h2aa1	206.227187	0.8329596	0.1577938	25.99067	3.43E-07	0.0028736
dCA1	M	ENSMUSG00000029822	Osbpl3	94.2875786	-0.668603	0.148344	19.89716	8.17E-06	0.0256061
dCA1	M	ENSMUSG00000095098	Ccdc85b	1076.2691	0.3625556	0.0821456	19.67671	9.17E-06	0.0256061
dCA1	M	ENSMUSG00000038642	Ctss	243.898533	0.3880535	0.0913881	17.76973	2.49E-05	0.0417659
dCA1	M	ENSMUSG00000060032	H2afj	300.959108	0.3562581	0.0836879	17.96887	2.25E-05	0.0417659
dCA1	M	ENSMUSG00000047604	Frat2	88.2725694	0.4905573	0.1172262	17.28813	3.21E-05	0.0448376
dCA1	M	ENSMUSG00000032172	Olfm2	148.506025	-0.400513	0.1002687	16.10983	5.98E-05	0.0625823
dCA1	M	ENSMUSG00000050379	Sept6	242.110298	-0.356701	0.0880106	16.23388	5.60E-05	0.0625823
dCA1	M	ENSMUSG00000030592	Ryr1	138.231194	0.6985395	0.1744866	15.60622	7.80E-05	0.0725898
dCA1	M	ENSMUSG00000003680	Taf6l	69.0860831	0.4872524	0.1234578	15.39906	8.70E-05	0.0728977
dCA1	M	ENSMUSG00000022377	Asap1	370.406653	-0.312085	0.0802048	15.15327	9.91E-05	0.0754802
dCA1	M	ENSMUSG00000110344	Gm45716	70.9235326	0.8981835	0.2310631	14.74416	0.000123	0.0859437
dCA1	M	ENSMUSG00000021708	Rasgrf2	317.569768	0.3850838	0.1013799	14.31612	0.000155	0.0995684
vCA1	M	ENSMUSG00000035964	Tmem59l	928.698226	0.5171456	0.1039463	24.58047	7.13E-07	0.0018865
vCA1	M	ENSMUSG00000072235	Tuba1a	3718.83364	0.1522991	0.0305254	24.82213	6.29E-07	0.0018865
vCA1	M	ENSMUSG00000029547	Ints1	932.878705	-0.357975	0.0765676	21.91255	2.85E-06	0.0050357
vCA1	M	ENSMUSG00000007610	Gtpbp3	194.189577	-0.321355	0.075524	18.19428	1.99E-05	0.0264008
vCA1	M	ENSMUSG00000026568	Mpc2	378.8253	0.2880572	0.069537	17.09974	3.55E-05	0.0312938
vCA1	M	ENSMUSG00000048583	Igf2	269.383342	-1.104942	0.2616234	17.18223	3.40E-05	0.0312938
vCA1	M	ENSMUSG00000037894	H2afz	315.752006	0.4012399	0.0980088	16.52203	4.81E-05	0.0363683
vCA1	M	ENSMUSG00000022884	Eif4a2	1379.53525	0.1461137	0.036229	16.24928	5.55E-05	0.0367474
vCA1	M	ENSMUSG00000073616	Cops9	407.470578	0.253077	0.0660978	14.63963	0.00013	0.0765565
vCA1	M	ENSMUSG00000027581	Stmn3	3288.62006	0.2109514	0.0560111	14.10859	0.000173	0.0766252
vCA1	M	ENSMUSG00000034566	Atp5h	815.783034	0.2194589	0.0580251	14.25848	0.000159	0.0766252
vCA1	M	ENSMUSG00000069306	Hist1h4m	251.156097	0.3993095	0.1054558	14.09627	0.000174	0.0766252
vCA1	M	ENSMUSG00000040481	Bptf	544.581716	-0.278116	0.0756974	13.53807	0.000234	0.0951996
vCA1	M	ENSMUSG00000000738	Spg7	182.169451	-0.254661	0.0714901	12.67318	0.000371	0.0981251
vCA1	M	ENSMUSG00000014956	Ppp1cb	834.465208	0.1792483	0.0503262	12.62165	0.000381	0.0981251
vCA1	M	ENSMUSG00000022099	Dmtn	1992.43226	0.2204539	0.0619237	12.59311	0.000387	0.0981251
vCA1	M	ENSMUSG00000022425	Enpp2	867.62533	-1.896025	0.5155521	12.58316	0.000389	0.0981251
vCA1	M	ENSMUSG00000036398	Ppp1r11	246.812493	0.2517131	0.0705779	12.75364	0.000355	0.0981251
vCA1	M	ENSMUSG00000037601	Nme1	501.00354	0.3180039	0.0889112	12.6611	0.000373	0.0981251
vCA1	M	ENSMUSG00000038368	Focad	310.623407	-0.482784	0.1343591	12.93382	0.000323	0.0981251
vCA1	M	ENSMUSG00000039323	Igfbp2	164.61587	-0.94575	0.2625389	12.89668	0.000329	0.0981251
vCA1	F	ENSMUSG00000062908	Acadm	124.757226	-0.575861	0.1086675	29.24425	6.38E-08	0.0005536
vCA1	F	ENSMUSG00000024074	Crim1	1311.68189	-0.259289	0.0524776	24.30347	8.23E-07	0.0035702
vCA1	F	ENSMUSG00000057522	Spop	750.181438	-0.275163	0.0613814	19.78861	8.65E-06	0.0250176
vCA1	F	ENSMUSG00000006494	Pdk1	247.957269	0.3596786	0.0829321	18.3365	1.85E-05	0.0321061
vCA1	F	ENSMUSG00000028797	Tmem234	311.146399	-0.347444	0.0818856	18.01682	2.19E-05	0.0321061
vCA1	F	ENSMUSG00000032849	Abcc4	78.3507429	-0.664031	0.1564485	17.88402	2.35E-05	0.0321061
vCA1	F	ENSMUSG00000036112	Metap2	226.976321	-0.419741	0.1010775	17.69719	2.59E-05	0.0321061
vCA1	F	ENSMUSG00000018412	Kansl1	527.70703	0.3409291	0.0828872	16.95257	3.83E-05	0.03695
vCA1	F	ENSMUSG00000025245	Lztfl1	95.4585204	-0.750862	0.1740604	17.13115	3.49E-05	0.03695
vCA1	F	ENSMUSG00000024201	Kdm4b	745.019503	0.2948686	0.0725652	16.59595	4.62E-05	0.0401308
vCA1	F	ENSMUSG00000036893	Ehmt1	345.973475	-0.564643	0.141437	16.24815	5.56E-05	0.0438297

vCA1	F	ENSMUSG00000026571	Dcaf6	298.90347	0.4688993	0.1189303	15.79407	7.06E-05	0.0471384
vCA1	F	ENSMUSG00000040177	2310057M21Rik	114.37498	0.5237408	0.131284	15.80552	7.02E-05	0.0471384
vCA1	F	ENSMUSG00000013663	Pten	1005.46716	0.2001228	0.0512996	15.2828	9.26E-05	0.050194
vCA1	F	ENSMUSG00000028809	Srrm1	272.699542	0.3963599	0.1022169	15.31528	9.10E-05	0.050194
vCA1	F	ENSMUSG00000059810	Rgs3	132.226094	-0.617778	0.1558515	15.39824	8.71E-05	0.050194
vCA1	F	ENSMUSG00000008604	Ubqln4	718.397554	-0.170425	0.0444199	14.74415	0.000123	0.0511418
vCA1	F	ENSMUSG00000027253	Lrp4	677.199619	-0.426971	0.1083869	15.02554	0.000106	0.0511418
vCA1	F	ENSMUSG00000032518	Rpsa	2832.27802	-0.365279	0.0941519	14.83926	0.000117	0.0511418
vCA1	F	ENSMUSG00000035401	Emsy	205.700708	0.4077376	0.1050741	14.73431	0.000124	0.0511418
vCA1	F	ENSMUSG00000037416	Dmxl1	346.968514	0.300168	0.0773818	15.025	0.000106	0.0511418
vCA1	F	ENSMUSG00000049092	Gpr137c	81.3137299	-0.650176	0.1753157	14.36246	0.000151	0.0594684
vCA1	F	ENSMUSG00000032579	Hemk1	79.2533058	-0.926004	0.2515826	14.258	0.000159	0.0601285
vCA1	F	ENSMUSG00000007039	Ddah2	89.182566	-0.55597	0.1455929	14.01115	0.000182	0.0615332
vCA1	F	ENSMUSG00000020257	Wdr82	590.670448	0.3194562	0.085252	13.98393	0.000184	0.0615332
vCA1	F	ENSMUSG00000093674	Rpl41	2032.33481	-0.210438	0.0563136	14.06639	0.000176	0.0615332
vCA1	F	ENSMUSG00000023007	Prpf40b	284.374885	-0.316983	0.0857952	13.82132	0.000201	0.0623213
vCA1	F	ENSMUSG00000026163	Sphkap	1628.69074	0.3628768	0.0997219	13.59254	0.000227	0.0623213
vCA1	F	ENSMUSG00000026796	Fam129b	185.312096	-0.508763	0.1381969	13.5213	0.000236	0.0623213
vCA1	F	ENSMUSG00000029725	Ppp1r35	81.1051123	-0.511475	0.138657	13.67257	0.000218	0.0623213
vCA1	F	ENSMUSG00000033569	Adgrb3	805.539982	0.2229481	0.0607385	13.5122	0.000237	0.0623213
vCA1	F	ENSMUSG00000036062	Phf24	872.358316	0.3175217	0.0854541	13.77547	0.000206	0.0623213
vCA1	F	ENSMUSG00000042613	Pbxip1	562.032042	-0.34285	0.0932251	13.6465	0.000221	0.0623213
vCA1	F	ENSMUSG00000003731	Kpna6	651.244411	0.1952655	0.0538539	13.22758	0.000276	0.0654048
vCA1	F	ENSMUSG00000032307	Ube2q2	126.451057	0.3798691	0.1037962	13.20707	0.000279	0.0654048
vCA1	F	ENSMUSG00000049086	Bmyc	367.41424	-0.228922	0.0628116	13.22277	0.000277	0.0654048
vCA1	F	ENSMUSG00000051166	Eml5	291.589823	0.3027524	0.0837999	13.28955	0.000267	0.0654048
vCA1	F	ENSMUSG00000006920	Ezh1	406.665761	0.2284273	0.0634079	12.93951	0.000322	0.0719283
vCA1	F	ENSMUSG00000027993	Trim2	2839.16189	0.2544127	0.0709212	12.84053	0.000339	0.0719283
vCA1	F	ENSMUSG00000028560	Usp1	92.4244883	0.8348708	0.2290951	12.83679	0.00034	0.0719283
vCA1	F	ENSMUSG00000033991	Ttc37	130.27002	-0.570688	0.1548554	12.84666	0.000338	0.0719283
vCA1	F	ENSMUSG00000031778	Cx3cl1	2933.83643	0.192129	0.0535419	12.78847	0.000349	0.0720527
vCA1	F	ENSMUSG00000022914	Brwd1	733.311301	0.2249682	0.0627108	12.6031	0.000385	0.075945
vCA1	F	ENSMUSG00000047213	Ythdf3	199.353132	0.3419191	0.0969911	12.63531	0.000379	0.075945
vCA1	F	ENSMUSG00000028106	Rprd2	393.760585	0.2491469	0.0712743	12.25561	0.000464	0.0804985
vCA1	F	ENSMUSG00000029059	Fam213b	280.092128	-0.31464	0.0897703	12.25902	0.000463	0.0804985
vCA1	F	ENSMUSG00000036199	Ndufa13	537.163443	-0.267216	0.0758036	12.4178	0.000425	0.0804985
vCA1	F	ENSMUSG00000039323	Igfbp2	281.833739	-1.255135	0.3582815	12.27903	0.000458	0.0804985
vCA1	F	ENSMUSG00000051285	Pcmtd1	251.737384	0.4427842	0.1243311	12.38001	0.000434	0.0804985
vCA1	F	ENSMUSG00000053646	Plxnb1	1444.80348	-0.274636	0.077786	12.3339	0.000445	0.0804985
vCA1	F	ENSMUSG00000031715	Smarca5	219.326366	0.2722078	0.0784527	12.09287	0.000506	0.0849516
vCA1	F	ENSMUSG00000033965	Slc16a2	266.455371	-0.596713	0.1676755	12.04651	0.000519	0.0849516
vCA1	F	ENSMUSG00000068551	Zfp467	115.400734	-0.399853	0.1156953	12.0732	0.000512	0.0849516
vCA1	F	ENSMUSG00000034248	Slc25a37	87.9272601	0.4638312	0.1339926	11.82044	0.000586	0.0924245
vCA1	F	ENSMUSG00000109536	9330162G02Rik	208.411454	0.3663078	0.1067479	11.8266	0.000584	0.0924245
vCA1	F	ENSMUSG00000092607	Scnm1	81.1769042	-0.408296	0.1194777	11.77668	0.0006	0.092933
vCA1	F	ENSMUSG00000032030	Cul5	274.474959	0.2693376	0.0791037	11.66329	0.000637	0.0970387
vCA1	F	ENSMUSG00000019437	Tlcd1	81.4834803	-0.502838	0.1470428	11.61367	0.000655	0.0979436
vCA1	F	ENSMUSG00000028467	Gba2	538.73438	0.2728088	0.0801703	11.5062	0.000694	0.0991719
vCA1	F	ENSMUSG00000052727	Map1b	9061.44051	0.1656356	0.0489775	11.53294	0.000684	0.0991719
vCA1	F	ENSMUSG00000059291	Rpl11	1243.6921	-0.206513	0.0609397	11.49673	0.000697	0.0991719

Supplementary Table 2. lists of genes discordantly expressed between sexes and background genes in the NAc and BNST

BNST_FemaleUp_MaleDown

ENSEMBL_ID	gene_name
ENSMUSG00000015377	Dennd6b
ENSMUSG00000015461	Atf6b
ENSMUSG00000015476	Prrt1
ENSMUSG00000015599	Ttbk1
ENSMUSG00000015605	Srf
ENSMUSG00000015653	Steap2
ENSMUSG00000015766	Eps8
ENSMUSG00000015879	Fam184b
ENSMUSG00000015944	Gatsl2
ENSMUSG00000015961	Adss
ENSMUSG00000015971	Actr8
ENSMUSG00000016028	Celsr1
ENSMUSG00000016239	Lonrf3
ENSMUSG00000016262	Sertad4
ENSMUSG00000016349	Eef1a2
ENSMUSG00000016386	Mpped2
ENSMUSG00000016427	Ndufa1
ENSMUSG00000016481	Cr1l
ENSMUSG00000016619	Nup50
ENSMUSG00000016637	Ift27
ENSMUSG00000016831	Tox4
ENSMUSG00000017167	Cntnap1
ENSMUSG00000017176	Nt5c3b
ENSMUSG00000017264	Exosc10
ENSMUSG00000017286	Glod4
ENSMUSG00000017288	Vps53
ENSMUSG00000017291	Taok1
ENSMUSG00000017299	Dnttip1
ENSMUSG00000017417	Plxdc1
ENSMUSG00000017466	Timp2
ENSMUSG00000017548	Suz12
ENSMUSG00000017639	Rab11fip4
ENSMUSG00000017686	Rhot1
ENSMUSG00000017692	Rhbdl3
ENSMUSG00000017715	Pgs1
ENSMUSG00000017740	Slc12a5
ENSMUSG00000017754	Pltp
ENSMUSG00000017764	Zswim1
ENSMUSG00000017774	Myo1c
ENSMUSG00000017843	Ppp2r5c
ENSMUSG00000017999	Ddx27
ENSMUSG00000018076	Med13l
ENSMUSG00000018102	Hist1h2bc
ENSMUSG00000018196	Glrx2
ENSMUSG00000018209	Stk4
ENSMUSG00000018287	Spag7
ENSMUSG00000018334	Ksr1
ENSMUSG00000018340	Anxa6

ENSMUSG00000018347	Zkscan6
ENSMUSG00000018401	Mtmr4
ENSMUSG00000018425	Dhx40
ENSMUSG00000018427	Ypel2
ENSMUSG00000018428	Akap1
ENSMUSG00000018433	Nol11
ENSMUSG00000018446	C1qbp
ENSMUSG00000018476	Kdm6b
ENSMUSG00000018509	Cenpv
ENSMUSG00000018547	Pip4k2b
ENSMUSG00000018548	Trim37
ENSMUSG00000018648	Dusp14
ENSMUSG00000018651	Tada2a
ENSMUSG00000018666	Cbx1
ENSMUSG00000018678	Sp2
ENSMUSG00000018697	Aatf
ENSMUSG00000018750	Zbtb4
ENSMUSG00000018849	Wwc1
ENSMUSG00000019082	Slc25a22
ENSMUSG00000019132	BC005537
ENSMUSG00000019146	Cacng2
ENSMUSG00000019173	Rab5c
ENSMUSG00000019194	Scn1b
ENSMUSG00000019297	Nop9
ENSMUSG00000019302	Atp6v0a1
ENSMUSG00000019437	Tlcd1
ENSMUSG00000019478	Rab4a
ENSMUSG00000019494	Cops6
ENSMUSG00000019558	Slc6a8
ENSMUSG00000019699	Akt3
ENSMUSG00000019718	L3hypdh
ENSMUSG00000019796	Lrp11
ENSMUSG00000019802	Sec63
ENSMUSG00000019831	Wasf1
ENSMUSG00000019841	Rev3l
ENSMUSG00000019849	Prep
ENSMUSG00000019861	Gopc
ENSMUSG00000019868	Vta1
ENSMUSG00000019890	Nts
ENSMUSG00000019894	Slc6a15
ENSMUSG00000019927	Ube2d1
ENSMUSG00000019943	Atp2b1
ENSMUSG00000019969	Psen1
ENSMUSG00000019975	Ikbip
ENSMUSG00000019984	Med23
ENSMUSG00000019996	Map7
ENSMUSG00000019998	Stx7
ENSMUSG00000020015	Cdk17
ENSMUSG00000020021	Fgd6
ENSMUSG00000020022	Ndufa12
ENSMUSG00000020027	Socs2
ENSMUSG00000020038	Cry1
ENSMUSG00000020069	Hnrnph3

ENSMUSG00000020074	Ccar1
ENSMUSG00000020075	Ddx21
ENSMUSG00000020091	Eif4ebp2
ENSMUSG00000020105	Lrig3
ENSMUSG00000020109	Dnajb12
ENSMUSG00000020134	Peli1
ENSMUSG00000020149	Rab1
ENSMUSG00000020160	Meis1
ENSMUSG00000020167	Tcf3
ENSMUSG00000020173	Cobl
ENSMUSG00000020175	Rab36
ENSMUSG00000020186	Csrp2
ENSMUSG00000020211	Sf3a2
ENSMUSG00000020224	Llph
ENSMUSG00000020225	Tmbim4
ENSMUSG00000020232	Hmg20b
ENSMUSG00000020238	Ncln
ENSMUSG00000020250	Txnrd1
ENSMUSG00000020257	Wdr82
ENSMUSG00000020261	Slc36a1
ENSMUSG00000020263	Appl2
ENSMUSG00000020271	Fbxw11
ENSMUSG00000020272	Stk10
ENSMUSG00000020273	Papolg
ENSMUSG00000020280	Pus10
ENSMUSG00000020287	Mpg
ENSMUSG00000020290	Xpo1
ENSMUSG00000020297	Nsg2
ENSMUSG00000020305	Asb3
ENSMUSG00000020315	Sptbn1
ENSMUSG00000020329	Polrmt
ENSMUSG00000020331	Hcn2
ENSMUSG00000020333	Acsl6
ENSMUSG00000020334	Slc22a4
ENSMUSG00000020340	Cyfip2
ENSMUSG00000020358	Hnrnpab
ENSMUSG00000020362	Cnot6
ENSMUSG00000020380	Rad50
ENSMUSG00000020387	Jade2
ENSMUSG00000020393	Kremen1
ENSMUSG00000020397	Med7
ENSMUSG00000020430	Pes1
ENSMUSG00000020431	Adcy1
ENSMUSG00000020444	Guk1
ENSMUSG00000020453	Patz1
ENSMUSG00000020458	Rtn4
ENSMUSG00000020462	Cfap36
ENSMUSG00000020473	Aebp1
ENSMUSG00000020491	2810021J22Rik
ENSMUSG00000020523	Fam114a2
ENSMUSG00000020536	Llgl1
ENSMUSG00000020541	Tom1l1
ENSMUSG00000020564	Atxn7l1

ENSMUSG00000020572	Nampt
ENSMUSG00000020580	Rock2
ENSMUSG00000020585	Laptm4a
ENSMUSG00000020589	Fam49a
ENSMUSG00000020594	Pum2
ENSMUSG00000020605	Hs1bp3
ENSMUSG00000020610	Amz2
ENSMUSG00000020623	Map2k6
ENSMUSG00000020634	Ubxn2a
ENSMUSG00000020639	Pfn4
ENSMUSG00000020640	Itsn2
ENSMUSG00000020644	Id2
ENSMUSG00000020658	Efr3b
ENSMUSG00000020659	Cbll1
ENSMUSG00000020668	Kif3c
ENSMUSG00000020671	Rab10
ENSMUSG00000020674	Pxdn
ENSMUSG00000020687	Cdc27
ENSMUSG00000020691	Mettl2
ENSMUSG00000020694	Tlk2
ENSMUSG00000020697	Lig3
ENSMUSG00000020704	Asic2
ENSMUSG00000020715	Ern1
ENSMUSG00000020723	Cacng4
ENSMUSG00000020733	Slc9a3r1
ENSMUSG00000020734	Grin2c
ENSMUSG00000020740	Gga3
ENSMUSG00000020745	Pafah1b1
ENSMUSG00000020747	Tmem94
ENSMUSG00000020777	Acox1
ENSMUSG00000020780	Srp68
ENSMUSG00000020790	Ankfy1
ENSMUSG00000020799	Tekt1
ENSMUSG00000020817	Rabep1
ENSMUSG00000020823	Sec14l1
ENSMUSG00000020827	Mink1
ENSMUSG00000020841	Cpd
ENSMUSG00000020848	Doc2b
ENSMUSG00000020849	Ywhae
ENSMUSG00000020882	Cacnb1
ENSMUSG00000020893	Per1
ENSMUSG00000020900	Myh10
ENSMUSG00000020923	Ubtf
ENSMUSG00000020926	Adam11
ENSMUSG00000020932	Gfap
ENSMUSG00000020950	Foxg1
ENSMUSG00000020952	Scfd1
ENSMUSG00000020955	Ap4s1
ENSMUSG00000020964	Sel1l
ENSMUSG00000021036	Sptlc2
ENSMUSG00000021039	Snw1
ENSMUSG00000021061	Sptb
ENSMUSG00000021062	Rab15

ENSMUSG00000021068	Nin
ENSMUSG00000021069	Pygl
ENSMUSG00000021071	Trim9
ENSMUSG00000021094	Dhrs7
ENSMUSG00000021096	Ppm1a
ENSMUSG00000021111	Papola
ENSMUSG00000021131	Erh
ENSMUSG00000021149	Gtpbp4
ENSMUSG00000021180	Rps6ka5
ENSMUSG00000021182	Ccdc88c
ENSMUSG00000021185	Dglucy
ENSMUSG00000021196	Pfkp
ENSMUSG00000021198	Unc79
ENSMUSG00000021203	Otub2
ENSMUSG00000021215	Net1
ENSMUSG00000021224	Numb
ENSMUSG00000021234	Fam161b
ENSMUSG00000021256	Vash1
ENSMUSG00000021258	Ccnk
ENSMUSG00000021259	Cyp46a1
ENSMUSG00000021260	Hhipl1
ENSMUSG00000021266	Wars
ENSMUSG00000021285	Ppp1r13b
ENSMUSG00000021318	Gli3
ENSMUSG00000021338	Carmil1
ENSMUSG00000021360	Gcnt2
ENSMUSG00000021361	Tmem14c
ENSMUSG00000021385	Ippk
ENSMUSG00000021392	Nol8
ENSMUSG00000021427	Ssr1
ENSMUSG00000021448	Shc3
ENSMUSG00000021481	Zfp346
ENSMUSG00000021483	Cdk20
ENSMUSG00000021484	Lman2
ENSMUSG00000021494	Ddx41
ENSMUSG00000021532	Fastkd3
ENSMUSG00000021537	Cetn3
ENSMUSG00000021540	Smad5
ENSMUSG00000021546	Hnrnpk
ENSMUSG00000021552	Gkap1
ENSMUSG00000021597	Slf1
ENSMUSG00000021645	Smn1
ENSMUSG00000021650	Ptcd2
ENSMUSG00000021660	Btf3
ENSMUSG00000021665	Hexb
ENSMUSG00000021666	Gfm2
ENSMUSG00000021684	Pde8b
ENSMUSG00000021686	Ap3b1
ENSMUSG00000021694	Ercc8
ENSMUSG00000021702	Thbs4
ENSMUSG00000021712	Trim23
ENSMUSG00000021720	Rnf180
ENSMUSG00000021725	Parp8

ENSMUSG00000021731	Mrps30
ENSMUSG00000021774	Ube2e1
ENSMUSG00000021775	Nr1d2
ENSMUSG00000021786	Oxsm
ENSMUSG00000021810	Ecd
ENSMUSG00000021814	Anxa7
ENSMUSG00000021819	Zswim8
ENSMUSG00000021866	Anxa11
ENSMUSG00000021900	Btd
ENSMUSG00000021901	Bap1
ENSMUSG00000021906	Oxnad1
ENSMUSG00000021943	Gdf10
ENSMUSG00000021945	Zmym2
ENSMUSG00000021973	Micu2
ENSMUSG00000021978	Extl3
ENSMUSG00000021993	Mipep
ENSMUSG00000022023	Wbp4
ENSMUSG00000022040	Ephx2
ENSMUSG00000022054	Nefm
ENSMUSG00000022075	Rhobtb2
ENSMUSG00000022095	Fam160b2
ENSMUSG00000022100	Xpo7
ENSMUSG00000022109	Med4
ENSMUSG00000022119	Rbm26
ENSMUSG00000022131	Gpr180
ENSMUSG00000022139	Mbnl2
ENSMUSG00000022141	Nipbl
ENSMUSG00000022142	Nup155
ENSMUSG00000022180	Slc7a8
ENSMUSG00000022186	Oxct1
ENSMUSG00000022194	Pabpn1
ENSMUSG00000022208	Jph4
ENSMUSG00000022244	Amacr
ENSMUSG00000022262	Dnah5
ENSMUSG00000022283	Pabpc1
ENSMUSG00000022297	Fzd6
ENSMUSG00000022359	Wdyhv1
ENSMUSG00000022361	Zhx1
ENSMUSG00000022364	Tbc1d31
ENSMUSG00000022378	Fam49b
ENSMUSG00000022389	Tef
ENSMUSG00000022390	Zc3h7b
ENSMUSG00000022394	L3mbtl2
ENSMUSG00000022416	Cacna1i
ENSMUSG00000022420	Dnal4
ENSMUSG00000022433	Csnk1e
ENSMUSG00000022437	Samm50
ENSMUSG00000022443	Myh9
ENSMUSG00000022449	Adamts20
ENSMUSG00000022454	Nell2
ENSMUSG00000022475	Hdac7
ENSMUSG00000022490	Ppp1r1a
ENSMUSG00000022494	Shisa9

ENSMUSG00000022505	Emp2
ENSMUSG00000022521	Crebbp
ENSMUSG00000022528	Hes1
ENSMUSG00000022529	Zfp263
ENSMUSG00000022537	Tmem44
ENSMUSG00000022552	Sharpin
ENSMUSG00000022557	Bop1
ENSMUSG00000022560	Slc52a2
ENSMUSG00000022564	Grina
ENSMUSG00000022565	Plec
ENSMUSG00000022580	Rhpn1
ENSMUSG00000022594	Lynx1
ENSMUSG00000022620	Arsa
ENSMUSG00000022621	Rabl2
ENSMUSG00000022623	Shank3
ENSMUSG00000022636	Alcam
ENSMUSG00000022639	Dubr
ENSMUSG00000022665	Ccdc80
ENSMUSG00000022668	Gtpbp8
ENSMUSG00000022673	Mcm4
ENSMUSG00000022681	Ntan1
ENSMUSG00000022706	Mrpl40
ENSMUSG00000022707	Gbe1
ENSMUSG00000022744	Cldnd1
ENSMUSG00000022752	Tomm70a
ENSMUSG00000022757	Tfg
ENSMUSG00000022759	Lrrc74b
ENSMUSG00000022770	Dlg1
ENSMUSG00000022771	Ppil2
ENSMUSG00000022773	Ypel1
ENSMUSG00000022790	Igsf11
ENSMUSG00000022807	Osbpl11
ENSMUSG00000022812	Gsk3b
ENSMUSG00000022814	Umps
ENSMUSG00000022829	Stxbp5l
ENSMUSG00000022836	Mylk
ENSMUSG00000022855	Senp2
ENSMUSG00000022856	Tmem41a
ENSMUSG00000022858	Tra2b
ENSMUSG00000022860	Chodl
ENSMUSG00000022863	Btg3
ENSMUSG00000022889	Mrpl39
ENSMUSG00000022890	Atp5j
ENSMUSG00000022892	App
ENSMUSG00000022905	Kpna1
ENSMUSG00000022947	Cbr3
ENSMUSG00000022961	Son
ENSMUSG00000022962	Gart
ENSMUSG00000022965	Ifngr2
ENSMUSG00000022974	Paxbp1
ENSMUSG00000022978	Mis18a
ENSMUSG00000023027	Atf1
ENSMUSG00000023033	Scn8a

ENSMUSG00000023034	Nr4a1
ENSMUSG00000023055	Calcoco1
ENSMUSG00000023066	Rttn
ENSMUSG00000023068	Nus1
ENSMUSG00000023079	Gtf2ird1
ENSMUSG00000023087	Noct
ENSMUSG00000023150	Ivns1abp
ENSMUSG00000023169	Slc38a1
ENSMUSG00000023192	Grm2
ENSMUSG00000023348	Trip6
ENSMUSG00000023473	Celsr3
ENSMUSG00000023571	C1qtnf12
ENSMUSG00000023791	Pigx
ENSMUSG00000023826	Park2
ENSMUSG00000023909	Paqr4
ENSMUSG00000023912	Slc25a27
ENSMUSG00000023927	Satb1
ENSMUSG00000023952	Gtpbp2
ENSMUSG00000023960	Enpp5
ENSMUSG00000023961	Enpp4
ENSMUSG00000023972	Ptk7
ENSMUSG00000023977	Ubr2
ENSMUSG00000024013	Fgd2
ENSMUSG00000024033	Rsph1
ENSMUSG00000024052	Lpin2
ENSMUSG00000024054	Smchd1
ENSMUSG00000024065	Ehd3
ENSMUSG00000024076	Vit
ENSMUSG00000024098	Twsg1
ENSMUSG00000024122	Pdpk1
ENSMUSG00000024137	E4f1
ENSMUSG00000024169	Ift140
ENSMUSG00000024186	Rgs11
ENSMUSG00000024201	Kdm4b
ENSMUSG00000024206	Rfx2
ENSMUSG00000024218	Taf11
ENSMUSG00000024228	Nudt12
ENSMUSG00000024238	Zeb1
ENSMUSG00000024298	Zfp871
ENSMUSG00000024302	Dtna
ENSMUSG00000024304	Cdh2
ENSMUSG00000024325	Ring1
ENSMUSG00000024327	Slc39a7
ENSMUSG00000024350	Dnajc18
ENSMUSG00000024370	Cdc23
ENSMUSG00000024384	Iws1
ENSMUSG00000024393	Prrc2a
ENSMUSG00000024400	Wdr33
ENSMUSG00000024426	Atat1
ENSMUSG00000024493	Lars
ENSMUSG00000024502	Jakmip2
ENSMUSG00000024534	Sncaip
ENSMUSG00000024539	Ptpn2

ENSMUSG00000024544	Ldlrad4
ENSMUSG00000024558	Mapk4
ENSMUSG00000024576	Csnk1a1
ENSMUSG00000024617	Camk2a
ENSMUSG00000024620	Pdgfrb
ENSMUSG00000024622	Hmgxb3
ENSMUSG00000024743	Syt7
ENSMUSG00000024750	Zfand5
ENSMUSG00000024764	Naa40
ENSMUSG00000024769	Cdc42bpg
ENSMUSG00000024789	Jak2
ENSMUSG00000024827	Gldc
ENSMUSG00000024835	Coro1b
ENSMUSG00000024853	Sf3b2
ENSMUSG00000024855	Pacs1
ENSMUSG00000024897	Apba1
ENSMUSG00000024900	Cpt1a
ENSMUSG00000024913	Lrp5
ENSMUSG00000024921	Smarca2
ENSMUSG00000024935	Slc1a1
ENSMUSG00000024939	Fam89b
ENSMUSG00000024949	Sf1
ENSMUSG00000024978	Gpam
ENSMUSG00000024981	Acsl5
ENSMUSG00000024991	Eif3a
ENSMUSG00000025008	Tctn3
ENSMUSG00000025024	Smndc1
ENSMUSG00000025026	Add3
ENSMUSG00000025027	Xpnpep1
ENSMUSG00000025035	Arl3
ENSMUSG00000025060	Slk
ENSMUSG00000025078	Nhlrc2
ENSMUSG00000025092	Hspa12a
ENSMUSG00000025103	Btbd1
ENSMUSG00000025104	Hdgfl3
ENSMUSG00000025130	P4hb
ENSMUSG00000025134	Alyref
ENSMUSG00000025144	Cenpx
ENSMUSG00000025145	Lrrc45
ENSMUSG00000025147	Mob2
ENSMUSG00000025157	Zdhhc16
ENSMUSG00000025170	Rab40b
ENSMUSG00000025173	Wdr45b
ENSMUSG00000025184	R3hcc1l
ENSMUSG00000025193	Cutc
ENSMUSG00000025195	Dnmbp
ENSMUSG00000025212	Sfxn3
ENSMUSG00000025220	Mgea5
ENSMUSG00000025231	Sufu
ENSMUSG00000025260	Hsd17b10
ENSMUSG00000025265	Fgd1
ENSMUSG00000025278	Flnb
ENSMUSG00000025314	Ptprj

ENSMUSG00000025323	Sp4
ENSMUSG00000025332	Kdm5c
ENSMUSG00000025348	Itga7
ENSMUSG00000025366	Esyt1
ENSMUSG00000025369	Smarcc2
ENSMUSG00000025372	Baiap2
ENSMUSG00000025402	Nab2
ENSMUSG00000025409	Mbd6
ENSMUSG00000025413	Ttc4
ENSMUSG00000025422	Agap2
ENSMUSG00000025427	Rnf165
ENSMUSG00000025470	Zfp511
ENSMUSG00000025474	Tubgcp2
ENSMUSG00000025478	Dpysl4
ENSMUSG00000025537	Phkg1
ENSMUSG00000025571	Tnrc6c
ENSMUSG00000025576	Rbfox3
ENSMUSG00000025586	Cpeb1
ENSMUSG00000025616	Usp16
ENSMUSG00000025658	Ksr2
ENSMUSG00000025737	Wdr24
ENSMUSG00000025739	Gng13
ENSMUSG00000025759	Mfsd8
ENSMUSG00000025780	Itih5
ENSMUSG00000025781	Atp5c1
ENSMUSG00000025867	Cplx2
ENSMUSG00000025889	Snca
ENSMUSG00000025898	Cwf19l2
ENSMUSG00000025903	Lypla1
ENSMUSG00000025907	Rb1cc1
ENSMUSG00000025937	Lactb2
ENSMUSG00000025949	Pikfyve
ENSMUSG00000025964	Adam23
ENSMUSG00000025981	Coq10b
ENSMUSG00000026000	Lancl1
ENSMUSG00000026014	Raph1
ENSMUSG00000026077	Npas2
ENSMUSG00000026082	Rev1
ENSMUSG00000026087	Mrpl30
ENSMUSG00000026102	Inpp1
ENSMUSG00000026104	Stat1
ENSMUSG00000026111	Unc50
ENSMUSG00000026121	Sema4c
ENSMUSG00000026131	Dst
ENSMUSG00000026142	Rhbdd1
ENSMUSG00000026185	Igfbp5
ENSMUSG00000026198	Abcb6
ENSMUSG00000026207	Speg
ENSMUSG00000026213	Stk11ip
ENSMUSG00000026220	Slc16a14
ENSMUSG00000026227	2810459M11Rik
ENSMUSG00000026229	Psmd1
ENSMUSG00000026254	Eif4e2

ENSMUSG00000026259	Ngef
ENSMUSG00000026269	Rnpepl1
ENSMUSG00000026277	Stk25
ENSMUSG00000026281	Dtymk
ENSMUSG00000026301	Iqca
ENSMUSG00000026353	Ubxn4
ENSMUSG00000026355	Mcm6
ENSMUSG00000026365	Cfh
ENSMUSG00000026425	Srgap2
ENSMUSG00000026436	Elk4
ENSMUSG00000026452	Syt2
ENSMUSG00000026469	Xpr1
ENSMUSG00000026470	Stx6
ENSMUSG00000026478	Lamc1
ENSMUSG00000026482	Rgl1
ENSMUSG00000026491	Ahctf1
ENSMUSG00000026496	Parp1
ENSMUSG00000026502	Desi2
ENSMUSG00000026504	Sdccag8
ENSMUSG00000026509	Capn2
ENSMUSG00000026514	Cnih3
ENSMUSG00000026516	Nvl
ENSMUSG00000026546	Cfap45
ENSMUSG00000026547	Tagln2
ENSMUSG00000026554	Dcaf8
ENSMUSG00000026565	Pou2f1
ENSMUSG00000026584	Scyl3
ENSMUSG00000026600	Soat1
ENSMUSG00000026615	Eprs
ENSMUSG00000026634	Angel2
ENSMUSG00000026643	Nmt2
ENSMUSG00000026649	Cfap126
ENSMUSG00000026663	Atf6
ENSMUSG00000026675	Hsd17b7
ENSMUSG00000026678	Rgs5
ENSMUSG00000026701	Prdx6
ENSMUSG00000026709	Dars2
ENSMUSG00000026721	Rabgap1l
ENSMUSG00000026728	Vim
ENSMUSG00000026740	Dnajc1
ENSMUSG00000026761	Orc4
ENSMUSG00000026775	Yme1l1
ENSMUSG00000026790	Odf2
ENSMUSG00000026796	Fam129b
ENSMUSG00000026799	Med27
ENSMUSG00000026807	Ak8
ENSMUSG00000026843	Fubp3
ENSMUSG00000026883	Dab2ip
ENSMUSG00000026885	Ttll11
ENSMUSG00000026926	Pmpca
ENSMUSG00000026927	Sdccag3
ENSMUSG00000026933	Camsap1
ENSMUSG00000026959	Grin1

ENSMUSG00000026972	Arrdc1
ENSMUSG00000026987	Baz2b
ENSMUSG00000027080	Med19
ENSMUSG00000027087	Itgav
ENSMUSG00000027109	Sp3
ENSMUSG00000027134	Lpcat4
ENSMUSG00000027195	Hsd17b12
ENSMUSG00000027206	Cops2
ENSMUSG00000027227	Sord
ENSMUSG00000027239	Mdk
ENSMUSG00000027254	Map1a
ENSMUSG00000027270	Lamp5
ENSMUSG00000027273	Snap25
ENSMUSG00000027284	Cdan1
ENSMUSG00000027312	Atrn
ENSMUSG00000027324	Rpusd2
ENSMUSG00000027360	Hdc
ENSMUSG00000027361	Gabpb1
ENSMUSG00000027386	Fbln7
ENSMUSG00000027422	Rrbp1
ENSMUSG00000027425	Kat14
ENSMUSG00000027427	Polr3f
ENSMUSG00000027457	Snph
ENSMUSG00000027502	Rtf2
ENSMUSG00000027522	Stx16
ENSMUSG00000027533	Fabp5
ENSMUSG00000027575	Arfgap1
ENSMUSG00000027577	Chrna4
ENSMUSG00000027580	Helz2
ENSMUSG00000027598	Itch
ENSMUSG00000027634	Ndrg3
ENSMUSG00000027646	Src
ENSMUSG00000027652	Ralgapb
ENSMUSG00000027674	Pex5l
ENSMUSG00000027677	Ttc14
ENSMUSG00000027782	Kpna4
ENSMUSG00000027796	Smad9
ENSMUSG00000027800	Tm4sf1
ENSMUSG00000027803	Wwtr1
ENSMUSG00000027806	Tsc22d2
ENSMUSG00000027808	Serp1
ENSMUSG00000027828	Ssr3
ENSMUSG00000027829	Ccnl1
ENSMUSG00000027878	Notch2
ENSMUSG00000027981	Rnpc3
ENSMUSG00000028019	Pdgfc
ENSMUSG00000028034	Fubp1
ENSMUSG00000028060	2810403A07Rik
ENSMUSG00000028093	Acp6
ENSMUSG00000028106	Rprd2
ENSMUSG00000028121	Bcar3
ENSMUSG00000028133	Rwdd3
ENSMUSG00000028137	Celf3

ENSMUSG00000028152	Tspan5
ENSMUSG00000028161	Ppp3ca
ENSMUSG00000028176	Lrrc7
ENSMUSG00000028195	Cyr61
ENSMUSG00000028247	Coq3
ENSMUSG00000028291	Akirin2
ENSMUSG00000028294	Cfap206
ENSMUSG00000028328	Tmod1
ENSMUSG00000028341	Nr4a3
ENSMUSG00000028385	Snx30
ENSMUSG00000028403	Zdhhc21
ENSMUSG00000028405	Aco1
ENSMUSG00000028409	Smu1
ENSMUSG00000028410	Dnaja1
ENSMUSG00000028411	Aptx
ENSMUSG00000028443	Nudt2
ENSMUSG00000028458	Tesk1
ENSMUSG00000028470	Hint2
ENSMUSG00000028517	Plpp3
ENSMUSG00000028525	Pde4b
ENSMUSG00000028568	Btf3l4
ENSMUSG00000028580	Pum1
ENSMUSG00000028581	Laptm5
ENSMUSG00000028622	Mrpl37
ENSMUSG00000028643	Svbp
ENSMUSG00000028656	Cap1
ENSMUSG00000028661	Epha8
ENSMUSG00000028693	Nasp
ENSMUSG00000028757	Ddost
ENSMUSG00000028760	Eif4g3
ENSMUSG00000028763	Hspg2
ENSMUSG00000028793	Rnf19b
ENSMUSG00000028799	Zfp362
ENSMUSG00000028809	Srrm1
ENSMUSG00000028820	Sfpq
ENSMUSG00000028822	Tmem50a
ENSMUSG00000028833	Ncdn
ENSMUSG00000028838	Extl1
ENSMUSG00000028868	Wasf2
ENSMUSG00000028879	Stx12
ENSMUSG00000028890	Mtf1
ENSMUSG00000028894	Inpp5b
ENSMUSG00000028901	Gmeb1
ENSMUSG00000028906	Epb41
ENSMUSG00000028907	Utp11
ENSMUSG00000028919	Arhgef19
ENSMUSG00000028936	Rpl22
ENSMUSG00000028957	Per3
ENSMUSG00000028960	Ube4b
ENSMUSG00000028978	Nos3
ENSMUSG00000029009	Mthfr
ENSMUSG00000029016	Clcn6
ENSMUSG00000029022	Miip

ENSMUSG00000029026	Trp73
ENSMUSG00000029030	Tprgl
ENSMUSG00000029033	Acap3
ENSMUSG00000029049	Morn1
ENSMUSG00000029050	Ski
ENSMUSG00000029055	Plch2
ENSMUSG00000029062	Cdk11b
ENSMUSG00000029093	Sorcs2
ENSMUSG00000029094	Afap1
ENSMUSG00000029098	Acox3
ENSMUSG00000029104	Htt
ENSMUSG00000029120	Ppp2r2c
ENSMUSG00000029131	Dnajb6
ENSMUSG00000029156	Sgcb
ENSMUSG00000029191	Rfc1
ENSMUSG00000029208	Guf1
ENSMUSG00000029229	Chic2
ENSMUSG00000029238	Clock
ENSMUSG00000029253	Cenpc1
ENSMUSG00000029265	Dr1
ENSMUSG00000029279	Brdt
ENSMUSG00000029334	Prkg2
ENSMUSG00000029346	Srrd
ENSMUSG00000029381	Shroom3
ENSMUSG00000029385	Ccng2
ENSMUSG00000029386	Tctn2
ENSMUSG00000029387	Gtf2h3
ENSMUSG00000029389	Ddx55
ENSMUSG00000029394	Cdk2ap1
ENSMUSG00000029406	Pitpnm2
ENSMUSG00000029415	Sdad1
ENSMUSG00000029416	Slc15a4
ENSMUSG00000029446	Psph
ENSMUSG00000029455	Aldh2
ENSMUSG00000029461	Fam168a
ENSMUSG00000029463	Fam216a
ENSMUSG00000029470	P2rx4
ENSMUSG00000029471	Camkk2
ENSMUSG00000029501	Ankle2
ENSMUSG00000029536	Gatc
ENSMUSG00000029550	Sppl3
ENSMUSG00000029563	Foxp2
ENSMUSG00000029569	Tmem168
ENSMUSG00000029608	Rph3a
ENSMUSG00000029610	Aimp2
ENSMUSG00000029621	Arpc1a
ENSMUSG00000029625	Cpsf4
ENSMUSG00000029640	Usp12
ENSMUSG00000029648	Flt1
ENSMUSG00000029673	Auts2
ENSMUSG00000029674	Limk1
ENSMUSG00000029687	Ezh2
ENSMUSG00000029703	Lrwd1

ENSMUSG00000029705	Cux1
ENSMUSG00000029710	Ephb4
ENSMUSG00000029712	Actl6b
ENSMUSG00000029722	Agfg2
ENSMUSG00000029754	Dlx6
ENSMUSG00000029761	Cald1
ENSMUSG00000029790	Cep41
ENSMUSG00000029819	Npy
ENSMUSG00000029823	Luc7l2
ENSMUSG00000029833	Trim24
ENSMUSG00000029924	Slc37a3
ENSMUSG00000030016	Zfp638
ENSMUSG00000030020	Prickle2
ENSMUSG00000030034	Ino80b
ENSMUSG00000030060	Hmces
ENSMUSG00000030098	Grip2
ENSMUSG00000030103	Bhlhe40
ENSMUSG00000030104	Edem1
ENSMUSG00000030105	Arl8b
ENSMUSG00000030120	Mlf2
ENSMUSG00000030125	Lrrc23
ENSMUSG00000030138	Bms1
ENSMUSG00000030166	Rad52
ENSMUSG00000030201	Lrp6
ENSMUSG00000030203	Dusp16
ENSMUSG00000030216	Wbp11
ENSMUSG00000030231	Plekha5
ENSMUSG00000030237	Slco1a4
ENSMUSG00000030249	Abcc9
ENSMUSG00000030271	Ogg1
ENSMUSG00000030275	Etnk1
ENSMUSG00000030302	Atp2b2
ENSMUSG00000030315	Vgll4
ENSMUSG00000030339	Ltbr
ENSMUSG00000030340	Scnn1a
ENSMUSG00000030376	Slc8a2
ENSMUSG00000030380	Mzf1
ENSMUSG00000030386	Zfp606
ENSMUSG00000030397	Mark4
ENSMUSG00000030410	Dmwd
ENSMUSG00000030411	Nova2
ENSMUSG00000030471	Zdhhc13
ENSMUSG00000030522	Mtmr10
ENSMUSG00000030525	Chrna7
ENSMUSG00000030527	Crtc3
ENSMUSG00000030532	Hddc3
ENSMUSG00000030533	Unc45a
ENSMUSG00000030583	Sipa1l3
ENSMUSG00000030591	Psmd8
ENSMUSG00000030613	Ccdc90b
ENSMUSG00000030630	Fah
ENSMUSG00000030659	Nucb2
ENSMUSG00000030671	Pde3b

ENSMUSG00000030678	Maz
ENSMUSG00000030704	Rab6a
ENSMUSG00000030737	Slco2b1
ENSMUSG00000030747	Dgat2
ENSMUSG00000030759	Far1
ENSMUSG00000030760	Acer3
ENSMUSG00000030772	Dkk3
ENSMUSG00000030782	Tgfb1i1
ENSMUSG00000030786	Itgam
ENSMUSG00000030806	Stx1b
ENSMUSG00000030826	Bcat2
ENSMUSG00000030868	Dctn5
ENSMUSG00000030872	Gga2
ENSMUSG00000030876	Mettl9
ENSMUSG00000030881	Arfip2
ENSMUSG00000030882	Dnhd1
ENSMUSG00000030889	Vwa3a
ENSMUSG00000030890	Ilk
ENSMUSG00000030898	Cckbr
ENSMUSG00000030930	Chst15
ENSMUSG00000030960	Eef1akmt2
ENSMUSG00000030982	9030624J02Rik
ENSMUSG00000031016	Wee1
ENSMUSG00000031024	St5
ENSMUSG00000031095	Cul4b
ENSMUSG00000031109	Enox2
ENSMUSG00000031134	Rbmx
ENSMUSG00000031148	Gpkow
ENSMUSG00000031153	Gripap1
ENSMUSG00000031166	Wdr13
ENSMUSG00000031167	Rbm3
ENSMUSG00000031176	Dynlt3
ENSMUSG00000031216	Stard8
ENSMUSG00000031217	Efnb1
ENSMUSG00000031285	Dcx
ENSMUSG00000031302	Nlgn3
ENSMUSG00000031311	Nono
ENSMUSG00000031328	Flna
ENSMUSG00000031333	Abcb7
ENSMUSG00000031353	Rbbp7
ENSMUSG00000031375	Bgn
ENSMUSG00000031386	Hcfc1
ENSMUSG00000031391	L1cam
ENSMUSG00000031442	Mcf2l
ENSMUSG00000031483	Erlin2
ENSMUSG00000031485	Plpbp
ENSMUSG00000031511	Arhgef7
ENSMUSG00000031529	Tnks
ENSMUSG00000031539	Ap3m2
ENSMUSG00000031548	Sfrp1
ENSMUSG00000031601	Cnot7
ENSMUSG00000031605	Klhl2
ENSMUSG00000031617	Tmem184c

ENSMUSG00000031626	Sorbs2
ENSMUSG00000031642	Sh3rf1
ENSMUSG00000031661	Nkd1
ENSMUSG00000031668	Eif2ak3
ENSMUSG00000031703	Itfg1
ENSMUSG00000031706	Rfx1
ENSMUSG00000031748	Gnao1
ENSMUSG00000031765	Mt1
ENSMUSG00000031812	Map1lc3b
ENSMUSG00000031843	Mphosph6
ENSMUSG00000031860	Pbx4
ENSMUSG00000031889	D230025D16Rik
ENSMUSG00000031925	Maml2
ENSMUSG00000031938	4931406C07Rik
ENSMUSG00000031939	Taf1d
ENSMUSG00000031948	Kars
ENSMUSG00000031971	Ccsap
ENSMUSG00000031987	Egln1
ENSMUSG00000031988	Vps26b
ENSMUSG00000031996	Aplp2
ENSMUSG00000032014	Oaf
ENSMUSG00000032020	Ubash3b
ENSMUSG00000032035	Ets1
ENSMUSG00000032036	Kirrel3
ENSMUSG00000032040	Dcps
ENSMUSG00000032058	Ppp2r1b
ENSMUSG00000032067	Pts
ENSMUSG00000032097	Ddx6
ENSMUSG00000032109	Nlrx1
ENSMUSG00000032120	C2cd2l
ENSMUSG00000032125	Robo4
ENSMUSG00000032174	Icam5
ENSMUSG00000032192	Gnb5
ENSMUSG00000032217	Rnf111
ENSMUSG00000032221	Mns1
ENSMUSG00000032231	Anxa2
ENSMUSG00000032238	Rora
ENSMUSG00000032239	Rp9
ENSMUSG00000032265	Fam46a
ENSMUSG00000032280	Tle3
ENSMUSG00000032295	Man2c1
ENSMUSG00000032328	Tmem30a
ENSMUSG00000032336	Nptn
ENSMUSG00000032340	Neo1
ENSMUSG00000032342	Mto1
ENSMUSG00000032355	Mlip
ENSMUSG00000032359	Ctsh
ENSMUSG00000032370	Lactb
ENSMUSG00000032382	Snx1
ENSMUSG00000032399	Rpl4
ENSMUSG00000032402	Smad3
ENSMUSG00000032405	Pias1
ENSMUSG00000032407	U2surp

ENSMUSG00000032409	Atr
ENSMUSG00000032418	Me1
ENSMUSG00000032420	Nt5e
ENSMUSG00000032435	Dync1li1
ENSMUSG00000032437	Stt3b
ENSMUSG00000032463	Faim
ENSMUSG00000032478	Nme6
ENSMUSG00000032481	Smarcc1
ENSMUSG00000032485	Scap
ENSMUSG00000032489	Kif9
ENSMUSG00000032514	Ttc21a
ENSMUSG00000032523	Hhatl
ENSMUSG00000032531	Amotl2
ENSMUSG00000032549	Rab6b
ENSMUSG00000032564	Cpne4
ENSMUSG00000032565	Nudt16
ENSMUSG00000032570	Atp2c1
ENSMUSG00000032571	Pik3r4
ENSMUSG00000032580	Rbm5
ENSMUSG00000032595	Cdhr4
ENSMUSG00000032624	Eml4
ENSMUSG00000032625	Thsd7a
ENSMUSG00000032637	Atxn2l
ENSMUSG00000032641	Gpr19
ENSMUSG00000032667	Pon2
ENSMUSG00000032688	Malt1
ENSMUSG00000032712	2810474O19Rik
ENSMUSG00000032737	Inppl1
ENSMUSG00000032741	Tpcn1
ENSMUSG00000032743	D430042O09Rik
ENSMUSG00000032773	Chrm1
ENSMUSG00000032803	Cdv3
ENSMUSG00000032827	Ppp1r9a
ENSMUSG00000032839	Trpc1
ENSMUSG00000032846	Zswim6
ENSMUSG00000032870	Smap2
ENSMUSG00000032883	Acsl3
ENSMUSG00000032902	Slc16a1
ENSMUSG00000032936	Camkv
ENSMUSG00000032965	Ift57
ENSMUSG00000032966	Fkbp1a
ENSMUSG00000032968	Inha
ENSMUSG00000033004	Mycbp2
ENSMUSG00000033055	Ankrd54
ENSMUSG00000033088	Triobp
ENSMUSG00000033099	Nol12
ENSMUSG00000033106	Slc7a6os
ENSMUSG00000033111	3830406C13Rik
ENSMUSG00000033128	Gga1
ENSMUSG00000033161	Atp1a1
ENSMUSG00000033184	Tmed7
ENSMUSG00000033209	Ttc28
ENSMUSG00000033228	Scaf11

ENSMUSG00000033237	Arid2
ENSMUSG00000033253	Szt2
ENSMUSG00000033272	Slc35a4
ENSMUSG00000033276	Stk36
ENSMUSG00000033287	Kctd17
ENSMUSG00000033306	Lpp
ENSMUSG00000033342	Plppr5
ENSMUSG00000033365	Ipo13
ENSMUSG00000033382	Trappc8
ENSMUSG00000033417	Cacul1
ENSMUSG00000033419	Snap91
ENSMUSG00000033429	Mcee
ENSMUSG00000033454	Zbtb1
ENSMUSG00000033502	Cdc14a
ENSMUSG00000033545	Znrf1
ENSMUSG00000033618	Map3k13
ENSMUSG00000033701	Acbd6
ENSMUSG00000033720	Sfxn5
ENSMUSG00000033739	Fkbpl
ENSMUSG00000033767	Tmem131l
ENSMUSG00000033773	Rpap2
ENSMUSG00000033790	Tubgcp5
ENSMUSG00000033805	Ephx4
ENSMUSG00000033863	Klf9
ENSMUSG00000033885	Pxk
ENSMUSG00000033904	Ccp110
ENSMUSG00000033917	Gde1
ENSMUSG00000033931	Rbm34
ENSMUSG00000033940	Brk1
ENSMUSG00000033965	Slc16a2
ENSMUSG00000033970	Rfc3
ENSMUSG00000033983	Coil
ENSMUSG00000034066	Farp2
ENSMUSG00000034075	Zdhhc5
ENSMUSG00000034109	Golim4
ENSMUSG00000034135	Sik3
ENSMUSG00000034154	Ino80
ENSMUSG00000034160	Ogt
ENSMUSG00000034163	Zfc3h1
ENSMUSG00000034168	Irf2bpl
ENSMUSG00000034211	Mrps17
ENSMUSG00000034216	Vps18
ENSMUSG00000034255	Arhgap27
ENSMUSG00000034259	Exosc4
ENSMUSG00000034265	Zdhhc14
ENSMUSG00000034269	Setd5
ENSMUSG00000034290	Nek9
ENSMUSG00000034300	Fam53c
ENSMUSG00000034312	Iqsec1
ENSMUSG00000034320	Slc26a2
ENSMUSG00000034336	Ina
ENSMUSG00000034341	Wbp2
ENSMUSG00000034353	Ramp1

ENSMUSG00000034413	Neurl1b
ENSMUSG00000034442	Trmt5
ENSMUSG00000034467	Dynlrb2
ENSMUSG00000034471	Caskin2
ENSMUSG00000034480	Diaph2
ENSMUSG00000034518	Hmgxb4
ENSMUSG00000034525	Ice1
ENSMUSG00000034573	Ptpn13
ENSMUSG00000034593	Myo5a
ENSMUSG00000034612	Chst11
ENSMUSG00000034620	Tmem5
ENSMUSG00000034621	Gpatch8
ENSMUSG00000034647	Ankrd12
ENSMUSG00000034686	Prr7
ENSMUSG00000034706	Dnaic2
ENSMUSG00000034771	Tle2
ENSMUSG00000034796	Cpne7
ENSMUSG00000034799	Unc13a
ENSMUSG00000034818	Celf5
ENSMUSG00000034850	Tmem127
ENSMUSG00000034854	Mfsd12
ENSMUSG00000034892	Rps29
ENSMUSG00000034902	Pip5k1c
ENSMUSG00000034928	Rnf44
ENSMUSG00000034997	Htr2a
ENSMUSG00000034998	Foxn2
ENSMUSG00000035007	Rundc1
ENSMUSG00000035011	Zbtb7a
ENSMUSG00000035049	Rrp12
ENSMUSG00000035109	Shc4
ENSMUSG00000035126	Wdr78
ENSMUSG00000035179	Ppp1r32
ENSMUSG00000035199	Arl6ip5
ENSMUSG00000035268	Pkig
ENSMUSG00000035277	Arx
ENSMUSG00000035283	Adrb1
ENSMUSG00000035314	Gdpd5
ENSMUSG00000035325	Sec31a
ENSMUSG00000035357	Pdzrn3
ENSMUSG00000035372	1810055G02Rik
ENSMUSG00000035397	Klf16
ENSMUSG00000035486	Plk5
ENSMUSG00000035504	Reep6
ENSMUSG00000035509	Fbxl21
ENSMUSG00000035539	Ccdc180
ENSMUSG00000035561	Aldh1b1
ENSMUSG00000035569	Ankrd11
ENSMUSG00000035575	Utp6
ENSMUSG00000035620	Ric8b
ENSMUSG00000035735	Dagla
ENSMUSG00000035762	Tmem161b
ENSMUSG00000035765	Dym
ENSMUSG00000035790	Cep19

ENSMUSG00000035851	Ythdc1
ENSMUSG00000035860	Cdhr3
ENSMUSG00000035898	Uba6
ENSMUSG00000035967	Ints6l
ENSMUSG00000036054	Sugp2
ENSMUSG00000036062	Phf24
ENSMUSG00000036086	Zranb3
ENSMUSG00000036087	Slain2
ENSMUSG00000036095	Dgkb
ENSMUSG00000036099	Vezt
ENSMUSG00000036103	Colec12
ENSMUSG00000036192	Rorb
ENSMUSG00000036206	Sh3bp4
ENSMUSG00000036225	Kctd1
ENSMUSG00000036273	Lrrk2
ENSMUSG00000036278	Macrod1
ENSMUSG00000036282	Naa30
ENSMUSG00000036285	Noa1
ENSMUSG00000036298	Slc2a13
ENSMUSG00000036323	Srp72
ENSMUSG00000036333	Kidins220
ENSMUSG00000036353	P2ry12
ENSMUSG00000036499	Eea1
ENSMUSG00000036568	Bicral
ENSMUSG00000036591	Arhgap21
ENSMUSG00000036598	Ccdc113
ENSMUSG00000036611	Eepd1
ENSMUSG00000036672	Cenpt
ENSMUSG00000036712	Cyld
ENSMUSG00000036748	Cuedc2
ENSMUSG00000036766	Dner
ENSMUSG00000036775	Decr2
ENSMUSG00000036782	Klhl13
ENSMUSG00000036817	Sun1
ENSMUSG00000036882	Arhgap33
ENSMUSG00000036887	C1qa
ENSMUSG00000036894	Rap2b
ENSMUSG00000036896	C1qc
ENSMUSG00000036902	Neto2
ENSMUSG00000036908	Unc93b1
ENSMUSG00000036916	Zfp280c
ENSMUSG00000036959	Bcorl1
ENSMUSG00000036989	Trim3
ENSMUSG00000036990	Otud4
ENSMUSG00000037007	Zfp113
ENSMUSG00000037017	Zscan21
ENSMUSG00000037022	Mmaa
ENSMUSG00000037029	Zfp146
ENSMUSG00000037035	Inhbb
ENSMUSG00000037098	Rab11fip3
ENSMUSG00000037112	Sik2
ENSMUSG00000037126	Psd
ENSMUSG00000037149	Ddx1

ENSMUSG00000037172	E330009J07Rik
ENSMUSG00000037217	Syn1
ENSMUSG00000037275	Gemin5
ENSMUSG00000037306	Man1c1
ENSMUSG00000037355	Uvssa
ENSMUSG00000037364	Srrt
ENSMUSG00000037386	Rims2
ENSMUSG00000037410	Tbc1d2b
ENSMUSG00000037455	Slc18b1
ENSMUSG00000037461	Ints7
ENSMUSG00000037503	Fam168b
ENSMUSG00000037514	Pank2
ENSMUSG00000037541	Shank2
ENSMUSG00000037552	Plekhg2
ENSMUSG00000037553	Zdhhc18
ENSMUSG00000037570	Mcrs1
ENSMUSG00000037579	Kcnh3
ENSMUSG00000037608	Bclaf1
ENSMUSG00000037617	Spag1
ENSMUSG00000037622	Wdtc1
ENSMUSG00000037652	Phc3
ENSMUSG00000037716	Ccdc33
ENSMUSG00000037720	Tmem33
ENSMUSG00000037791	Phf12
ENSMUSG00000037820	Tgm2
ENSMUSG00000037852	Cpe
ENSMUSG00000037855	Zfp365
ENSMUSG00000037887	Dusp8
ENSMUSG00000037890	Wdr19
ENSMUSG00000037896	Rcor1
ENSMUSG00000037902	Sirpa
ENSMUSG00000037907	Ankrd13b
ENSMUSG00000037935	Smarce1
ENSMUSG00000037965	Zc3h7a
ENSMUSG00000037989	Wnk2
ENSMUSG00000037990	Sh3rf3
ENSMUSG00000037993	Dhx38
ENSMUSG00000037995	Igsf9
ENSMUSG00000037996	Slc24a2
ENSMUSG00000037999	Arap2
ENSMUSG00000038005	Hpf1
ENSMUSG00000038011	Dnah10
ENSMUSG00000038014	Fam120a
ENSMUSG00000038025	Phf2
ENSMUSG00000038070	Cntln
ENSMUSG00000038084	Opa1
ENSMUSG00000038121	Fam210a
ENSMUSG00000038143	Stox2
ENSMUSG00000038170	Pde4dip
ENSMUSG00000038208	Pgap3
ENSMUSG00000038244	Mical2
ENSMUSG00000038264	Sema7a
ENSMUSG00000038267	Slc22a23

ENSMUSG00000038296	Galnt18
ENSMUSG00000038302	Afg1l
ENSMUSG00000038332	Sesn1
ENSMUSG00000038346	Zfp384
ENSMUSG00000038369	Ncoa6
ENSMUSG00000038375	Trp53inp2
ENSMUSG00000038388	Mpp6
ENSMUSG00000038393	Txnip
ENSMUSG00000038406	Scaf1
ENSMUSG00000038416	Cdc16
ENSMUSG00000038417	Fig4
ENSMUSG00000038418	Egr1
ENSMUSG00000038437	Mllt6
ENSMUSG00000038453	Srcin1
ENSMUSG00000038459	Abhd17c
ENSMUSG00000038518	Jarid2
ENSMUSG00000038530	Rgs4
ENSMUSG00000038552	Fndc4
ENSMUSG00000038563	Efl1
ENSMUSG00000038564	Ift172
ENSMUSG00000038602	Slc35f1
ENSMUSG00000038611	Phrf1
ENSMUSG00000038642	Ctss
ENSMUSG00000038679	Trps1
ENSMUSG00000038683	Pak1ip1
ENSMUSG00000038690	Atp5j2
ENSMUSG00000038705	Gmeb2
ENSMUSG00000038718	Pbx3
ENSMUSG00000038759	Nup205
ENSMUSG00000038773	Kdm3b
ENSMUSG00000038807	Rap1gap2
ENSMUSG00000038822	Hace1
ENSMUSG00000038831	Ralgps1
ENSMUSG00000038838	Vars2
ENSMUSG00000038866	Zcchc2
ENSMUSG00000038872	Zfhx3
ENSMUSG00000038886	Man2a2
ENSMUSG00000038895	Zfp653
ENSMUSG00000038902	Pogz
ENSMUSG00000038916	Soga3
ENSMUSG00000038949	Cnst
ENSMUSG00000038976	Ppp1r9b
ENSMUSG00000038984	Tspyl5
ENSMUSG00000039004	Bmp6
ENSMUSG00000039048	Foxred1
ENSMUSG00000039059	Hrh3
ENSMUSG00000039081	Zfp503
ENSMUSG00000039086	Ss18l1
ENSMUSG00000039100	March6
ENSMUSG00000039108	Lsm14b
ENSMUSG00000039110	Mycbpap
ENSMUSG00000039117	Taf4
ENSMUSG00000039126	Prune2

ENSMUSG00000039157	Fam102a
ENSMUSG00000039194	Rlbp1
ENSMUSG00000039197	Adk
ENSMUSG00000039202	Abhd2
ENSMUSG00000039234	Sec24d
ENSMUSG00000039252	Lgi2
ENSMUSG00000039275	Foxk2
ENSMUSG00000039308	Ndst2
ENSMUSG00000039361	Picalm
ENSMUSG00000039372	March4
ENSMUSG00000039405	Prss23
ENSMUSG00000039477	Tnrc18
ENSMUSG00000039478	Micu3
ENSMUSG00000039509	Nup133
ENSMUSG00000039530	Tusc3
ENSMUSG00000039533	Mmd2
ENSMUSG00000039536	Stau1
ENSMUSG00000039552	Rsph4a
ENSMUSG00000039568	Ubald1
ENSMUSG00000039585	Myo9a
ENSMUSG00000039633	Lonrf1
ENSMUSG00000039637	Coro7
ENSMUSG00000039660	Spout1
ENSMUSG00000039697	Ncoa7
ENSMUSG00000039716	Dock3
ENSMUSG00000039741	Bahcc1
ENSMUSG00000039754	Alkbh4
ENSMUSG00000039781	Cep131
ENSMUSG00000039841	Zfp800
ENSMUSG00000039842	Mcph1
ENSMUSG00000039844	Rapgef1
ENSMUSG00000039852	Rere
ENSMUSG00000039860	Srrm3
ENSMUSG00000039886	Tmem120a
ENSMUSG00000039952	Dag1
ENSMUSG00000039954	Stk32a
ENSMUSG00000039963	Ccdc40
ENSMUSG00000039994	Timeless
ENSMUSG00000040022	Rab11fip2
ENSMUSG00000040025	Ythdf2
ENSMUSG00000040033	Stat2
ENSMUSG00000040118	Cacna2d1
ENSMUSG00000040123	Zmym5
ENSMUSG00000040125	Gpr26
ENSMUSG00000040128	Pnrc1
ENSMUSG00000040147	Maob
ENSMUSG00000040174	Alkbh3
ENSMUSG00000040183	Ankrd6
ENSMUSG00000040195	Nemp1
ENSMUSG00000040209	Zfp704
ENSMUSG00000040219	Ttc12
ENSMUSG00000040220	Gas8
ENSMUSG00000040225	Prrc2c

ENSMUSG00000040359	Ufl1
ENSMUSG00000040365	Trim41
ENSMUSG00000040390	Map3k10
ENSMUSG00000040410	Fbxl4
ENSMUSG00000040414	Slc25a28
ENSMUSG00000040433	Zbtb38
ENSMUSG00000040459	Arglu1
ENSMUSG00000040502	March9
ENSMUSG00000040524	Zfp609
ENSMUSG00000040543	Pitpnm3
ENSMUSG00000040612	Ildr2
ENSMUSG00000040620	Dhx33
ENSMUSG00000040713	Creg1
ENSMUSG00000040721	Zfhx2
ENSMUSG00000040725	Hnrnpul1
ENSMUSG00000040729	Cep126
ENSMUSG00000040738	Ints8
ENSMUSG00000040749	Siah1b
ENSMUSG00000040820	Hlcs
ENSMUSG00000040848	Sft2d2
ENSMUSG00000040852	Plekhh2
ENSMUSG00000040857	Erf
ENSMUSG00000040860	Crocc
ENSMUSG00000040896	Kcnd3
ENSMUSG00000040928	S100pbp
ENSMUSG00000040936	Ulk4
ENSMUSG00000040945	Rcc2
ENSMUSG00000041025	Iffo2
ENSMUSG00000041040	Fam117b
ENSMUSG00000041046	Ramp3
ENSMUSG00000041057	Wdr43
ENSMUSG00000041073	Nacad
ENSMUSG00000041078	Grid1
ENSMUSG00000041084	Ostc
ENSMUSG00000041112	Elmo1
ENSMUSG00000041117	Ccdc8
ENSMUSG00000041126	H2afv
ENSMUSG00000041164	Zmiz2
ENSMUSG00000041205	Map6d1
ENSMUSG00000041220	Elovl6
ENSMUSG00000041225	Arhgap12
ENSMUSG00000041235	Chd7
ENSMUSG00000041297	Cdk13
ENSMUSG00000041308	Sntb2
ENSMUSG00000041309	Nkx6-2
ENSMUSG00000041329	Atp1b2
ENSMUSG00000041351	Rap1gap
ENSMUSG00000041360	Pum3
ENSMUSG00000041408	Wapl
ENSMUSG00000041415	Dicer1
ENSMUSG00000041417	Pik3r1
ENSMUSG00000041438	Utp4
ENSMUSG00000041444	Arhgap32

ENSMUSG00000041445	Mmrn2
ENSMUSG00000041453	Rpl21
ENSMUSG00000041488	Stx3
ENSMUSG00000041491	Cep78
ENSMUSG00000041530	Ago1
ENSMUSG00000041540	Sox5
ENSMUSG00000041556	Fbxo2
ENSMUSG00000041571	Selenow
ENSMUSG00000041577	Prelp
ENSMUSG00000041592	Sdk2
ENSMUSG00000041608	Entpd3
ENSMUSG00000041617	Ccdc74a
ENSMUSG00000041670	Rims1
ENSMUSG00000041685	Fcho2
ENSMUSG00000041729	Coro2b
ENSMUSG00000041762	Gpr155
ENSMUSG00000041771	Slc24a4
ENSMUSG00000041781	Cpsf2
ENSMUSG00000041797	Abca9
ENSMUSG00000041836	Ptpre
ENSMUSG00000041890	Git2
ENSMUSG00000041895	Wipi1
ENSMUSG00000041912	Tdrkh
ENSMUSG00000041930	Fam222a
ENSMUSG00000041936	Agrn
ENSMUSG00000041939	Mvk
ENSMUSG00000041975	Mettl8
ENSMUSG00000042029	Ncapg2
ENSMUSG00000042032	Mat2b
ENSMUSG00000042055	Wdr11
ENSMUSG00000042111	Ccdc115
ENSMUSG00000042121	Ssh1
ENSMUSG00000042202	Slc35e2
ENSMUSG00000042207	Kdm5b
ENSMUSG00000042256	Ptchd4
ENSMUSG00000042308	Setd1a
ENSMUSG00000042348	Arl15
ENSMUSG00000042350	Arel1
ENSMUSG00000042364	Snx18
ENSMUSG00000042369	Rbm45
ENSMUSG00000042389	Tsen2
ENSMUSG00000042390	Gatad2b
ENSMUSG00000042410	Agps
ENSMUSG00000042423	Fbrs
ENSMUSG00000042460	C1galt1
ENSMUSG00000042500	Ago4
ENSMUSG00000042502	Cd2bp2
ENSMUSG00000042507	Elmsan1
ENSMUSG00000042520	Ubap2l
ENSMUSG00000042523	Dnal1
ENSMUSG00000042524	Sun2
ENSMUSG00000042535	Gtpbp1
ENSMUSG00000042548	Asxl1

ENSMUSG00000042564	Fam227a
ENSMUSG00000042570	Mier2
ENSMUSG00000042589	Cux2
ENSMUSG00000042594	Sh2b3
ENSMUSG00000042604	Kcna4
ENSMUSG00000042616	Oscp1
ENSMUSG00000042632	Pla2g6
ENSMUSG00000042659	Arrdc4
ENSMUSG00000042680	Garem1
ENSMUSG00000042686	Jph1
ENSMUSG00000042688	Mapk6
ENSMUSG00000042694	Stn1
ENSMUSG00000042699	Dhx9
ENSMUSG00000042700	Sipa1l1
ENSMUSG00000042726	Trafd1
ENSMUSG00000042729	Wdr74
ENSMUSG00000042734	Ttc9
ENSMUSG00000042742	Bmt2
ENSMUSG00000042745	Id1
ENSMUSG00000042772	Smg7
ENSMUSG00000042826	Fgf11
ENSMUSG00000042846	Lrrtm3
ENSMUSG00000042978	Sbk1
ENSMUSG00000043079	Synpo
ENSMUSG00000043164	Tmem212
ENSMUSG00000043279	Trim56
ENSMUSG00000043313	Pcdhb19
ENSMUSG00000043323	Fbrsl1
ENSMUSG00000043391	2510009E07Rik
ENSMUSG00000043411	Usp48
ENSMUSG00000043439	Epop
ENSMUSG00000043487	Acot6
ENSMUSG00000043602	Zfp3
ENSMUSG00000043621	Ubxn10
ENSMUSG00000043635	Adamts3
ENSMUSG00000043668	Tox3
ENSMUSG00000043671	Dpy19l3
ENSMUSG00000043683	Fem1a
ENSMUSG00000043831	Lysmd4
ENSMUSG00000043857	Mgat5b
ENSMUSG00000043929	Klhl15
ENSMUSG00000044005	Gls2
ENSMUSG00000044167	Foxo1
ENSMUSG00000044340	Phlpp1
ENSMUSG00000044352	Sowaha
ENSMUSG00000044393	Dsg2
ENSMUSG00000044447	Dock5
ENSMUSG00000044477	Zfand3
ENSMUSG00000044499	Hs3st5
ENSMUSG00000044600	Smim7
ENSMUSG00000044636	Csrnp2
ENSMUSG00000044641	Pard6b
ENSMUSG00000044667	Plppr4

ENSMUSG00000044674	Fzd1
ENSMUSG00000044730	9930104L06Rik
ENSMUSG00000044763	Trmt10c
ENSMUSG00000044791	Setd2
ENSMUSG00000044835	Ankrd45
ENSMUSG00000044906	4930503L19Rik
ENSMUSG00000045045	Lrfn4
ENSMUSG00000045062	Pcdhb7
ENSMUSG00000045092	S1pr1
ENSMUSG00000045098	Kmt5b
ENSMUSG00000045114	Prrt2
ENSMUSG00000045180	Shroom2
ENSMUSG00000045210	Vcpip1
ENSMUSG00000045216	Hs6st1
ENSMUSG00000045251	Zfp688
ENSMUSG00000045259	Klhdc9
ENSMUSG00000045302	Preb
ENSMUSG00000045409	Trim39
ENSMUSG00000045411	2410002F23Rik
ENSMUSG00000045427	Hnrnph2
ENSMUSG00000045482	Trrap
ENSMUSG00000045498	Pcdhb3
ENSMUSG00000045515	Pou3f3
ENSMUSG00000045534	Kcna5
ENSMUSG00000045573	Penk
ENSMUSG00000045576	St7l
ENSMUSG00000045608	Dbx2
ENSMUSG00000045648	Vwc2l
ENSMUSG00000045655	Fam216b
ENSMUSG00000045659	Plekha7
ENSMUSG00000045672	Col27a1
ENSMUSG00000045733	Sprn
ENSMUSG00000045767	B230219D22Rik
ENSMUSG00000045817	Zfp36l2
ENSMUSG00000045838	A430105I19Rik
ENSMUSG00000045967	Gpr158
ENSMUSG00000045983	Eif4g1
ENSMUSG00000046032	Snx12
ENSMUSG00000046111	Cep295
ENSMUSG00000046139	Patl1
ENSMUSG00000046191	Pcdhb20
ENSMUSG00000046192	Iqub
ENSMUSG00000046201	Scaf8
ENSMUSG00000046230	Vps13a
ENSMUSG00000046240	Hepacam
ENSMUSG00000046324	Ermp1
ENSMUSG00000046329	Slc25a23
ENSMUSG00000046447	Camk2n1
ENSMUSG00000046541	Zfp526
ENSMUSG00000046607	Hrk
ENSMUSG00000046637	Ttc34
ENSMUSG00000046667	Rbm12b1
ENSMUSG00000046668	Cxxc5

ENSMUSG00000046805	Mpeg1
ENSMUSG00000046822	Slc39a3
ENSMUSG00000046982	Tshz1
ENSMUSG00000047021	Cfap65
ENSMUSG00000047090	Tmem198b
ENSMUSG00000047187	Rab2a
ENSMUSG00000047215	Rpl9
ENSMUSG00000047242	Taf9b
ENSMUSG00000047361	Gm973
ENSMUSG00000047394	Odf3b
ENSMUSG00000047409	Ctdspl
ENSMUSG00000047446	Arl4a
ENSMUSG00000047454	Gphn
ENSMUSG00000047495	Dlgap2
ENSMUSG00000047635	2810006K23Rik
ENSMUSG00000047648	Fbxo30
ENSMUSG00000047656	Trpt1
ENSMUSG00000047658	Gal3st3
ENSMUSG00000047669	Msl3l2
ENSMUSG00000047694	Yipf6
ENSMUSG00000047721	Bola2
ENSMUSG00000047747	Rnf150
ENSMUSG00000047766	Lrrc49
ENSMUSG00000047787	Flrt1
ENSMUSG00000047797	Gjb1
ENSMUSG00000047804	Akap10
ENSMUSG00000047866	Lonp2
ENSMUSG00000048029	Eno4
ENSMUSG00000048038	Ccdc187
ENSMUSG00000048039	Isg20l2
ENSMUSG00000048076	Arf1
ENSMUSG00000048120	Entpd1
ENSMUSG00000048154	Kmt2d
ENSMUSG00000048163	Selplg
ENSMUSG00000048216	Gpr85
ENSMUSG00000048232	Fbxo10
ENSMUSG00000048279	Sacs
ENSMUSG00000048307	Ankrd46
ENSMUSG00000048310	Pskh1
ENSMUSG00000048388	Fam171b
ENSMUSG00000048478	Spata33
ENSMUSG00000048490	Nrip1
ENSMUSG00000048756	Foxo3
ENSMUSG00000048758	Rpl29
ENSMUSG00000048832	Vps37c
ENSMUSG00000048895	Cdk5r1
ENSMUSG00000048897	Zfp710
ENSMUSG00000048921	Zfp689
ENSMUSG00000049001	Ndnf
ENSMUSG00000049076	Acap2
ENSMUSG00000049184	Purg
ENSMUSG00000049252	Lrp1b
ENSMUSG00000049300	Prmt6

ENSMUSG00000049313	Sorl1
ENSMUSG00000049421	Zfp260
ENSMUSG00000049488	Tmem67
ENSMUSG00000049493	Pls1
ENSMUSG00000049504	Proser1
ENSMUSG00000049556	Lingo1
ENSMUSG00000049571	Cfap46
ENSMUSG00000049577	Zfpm1
ENSMUSG00000049606	Zfp644
ENSMUSG00000049612	Omg
ENSMUSG00000049630	C1ql3
ENSMUSG00000049672	Zbtb14
ENSMUSG00000049686	Orai1
ENSMUSG00000049728	Zfp668
ENSMUSG00000049878	Rlf
ENSMUSG00000049932	H2afx
ENSMUSG00000049939	Lrrc4
ENSMUSG00000049971	Glt1d1
ENSMUSG00000050064	Zfp697
ENSMUSG00000050121	Opalin
ENSMUSG00000050138	Kcnk12
ENSMUSG00000050148	Ubqln2
ENSMUSG00000050229	Pigm
ENSMUSG00000050271	Prag1
ENSMUSG00000050310	Rictor
ENSMUSG00000050312	Nsun3
ENSMUSG00000050382	Kif7
ENSMUSG00000050511	Oprd1
ENSMUSG00000050530	Fam171a1
ENSMUSG00000050545	Fam228b
ENSMUSG00000050556	Kcnb1
ENSMUSG00000050628	Ubald2
ENSMUSG00000050751	Pgbd5
ENSMUSG00000050930	Map10
ENSMUSG00000050953	Gja1
ENSMUSG00000050954	Zfp169
ENSMUSG00000050973	Gdpgp1
ENSMUSG00000051007	Gatd1
ENSMUSG00000051034	Zfp11
ENSMUSG00000051065	Mb21d2
ENSMUSG00000051067	Lingo3
ENSMUSG00000051177	Plcb1
ENSMUSG00000051285	Pcmtd1
ENSMUSG00000051331	Cacna1c
ENSMUSG00000051335	Gfod1
ENSMUSG00000051375	Pcdh1
ENSMUSG00000051527	Usp29
ENSMUSG00000051650	B3gnt2
ENSMUSG00000051652	Lrrc3
ENSMUSG00000051695	Pcbp1
ENSMUSG00000051721	Wdcp
ENSMUSG00000051730	Mettl5
ENSMUSG00000051768	Xrcc1

ENSMUSG00000051817	Sox12
ENSMUSG00000051950	B3glct
ENSMUSG00000052040	Klf13
ENSMUSG00000052087	Rgs14
ENSMUSG00000052102	Gnpda1
ENSMUSG00000052105	Mtcl1
ENSMUSG00000052133	Sema5b
ENSMUSG00000052135	Foxo6
ENSMUSG00000052146	Rps10
ENSMUSG00000052273	Dnah3
ENSMUSG00000052293	Taf9
ENSMUSG00000052299	Ltn1
ENSMUSG00000052310	Slc39a1
ENSMUSG00000052336	Cx3cr1
ENSMUSG00000052369	Tmem106c
ENSMUSG00000052373	Mpp3
ENSMUSG00000052374	Actn2
ENSMUSG00000052397	Ezr
ENSMUSG00000052407	Ccdc171
ENSMUSG00000052488	Cherp
ENSMUSG00000052539	Magi3
ENSMUSG00000052565	Hist1h1d
ENSMUSG00000052632	Asap2
ENSMUSG00000052713	Zfp608
ENSMUSG00000052748	Swt1
ENSMUSG00000052798	Nup107
ENSMUSG00000052812	Atad2b
ENSMUSG00000052833	Sae1
ENSMUSG00000052861	Dnah6
ENSMUSG00000052917	Senp7
ENSMUSG00000052920	Prkg1
ENSMUSG00000052934	Fbxo31
ENSMUSG00000053025	Sv2b
ENSMUSG00000053134	Supt7l
ENSMUSG00000053137	Mapk11
ENSMUSG00000053199	Arhgap20
ENSMUSG00000053253	Ndfip2
ENSMUSG00000053390	Zfp952
ENSMUSG00000053436	Mapk14
ENSMUSG00000053460	Ggcx
ENSMUSG00000053536	Cstf2t
ENSMUSG00000053580	Tanc2
ENSMUSG00000053644	Aldh7a1
ENSMUSG00000053716	Dusp7
ENSMUSG00000053838	Nudcd3
ENSMUSG00000053877	Srcap
ENSMUSG00000053916	Nanp
ENSMUSG00000053931	Cnn3
ENSMUSG00000054021	Sirt5
ENSMUSG00000054034	Tceal5
ENSMUSG00000054162	Spock3
ENSMUSG00000054226	Tprkb
ENSMUSG00000054237	Fra10ac1

ENSMUSG00000054280	Prr14l
ENSMUSG00000054302	Eapp
ENSMUSG00000054309	Cpsf3
ENSMUSG00000054414	Slc30a7
ENSMUSG00000054517	Trim65
ENSMUSG00000054675	Tmem119
ENSMUSG00000054693	Adam10
ENSMUSG00000054733	Msra
ENSMUSG00000054766	Set
ENSMUSG00000054770	Kctd18
ENSMUSG00000054808	Actn4
ENSMUSG00000054823	Nsd3
ENSMUSG00000054843	Atrnl1
ENSMUSG00000055013	Agap1
ENSMUSG00000055053	Nfic
ENSMUSG00000055065	Ddx17
ENSMUSG00000055296	Tmem245
ENSMUSG00000055322	Tns1
ENSMUSG00000055421	Pcdh9
ENSMUSG00000055435	Maf
ENSMUSG00000055553	Kxd1
ENSMUSG00000055612	Cdca7
ENSMUSG00000055782	Abcd2
ENSMUSG00000055805	Fmnl1
ENSMUSG00000055866	Per2
ENSMUSG00000055900	Tmem69
ENSMUSG00000055991	Zkscan5
ENSMUSG00000056014	A430033K04Rik
ENSMUSG00000056050	Mia3
ENSMUSG00000056076	Eif3b
ENSMUSG00000056091	St3gal5
ENSMUSG00000056124	B4galt6
ENSMUSG00000056211	R3hdm1
ENSMUSG00000056215	Lrguk
ENSMUSG00000056305	Usp39
ENSMUSG00000056342	Usp34
ENSMUSG00000056427	Slit3
ENSMUSG00000056486	Chn1
ENSMUSG00000056493	Foxk1
ENSMUSG00000056592	Zfp658
ENSMUSG00000056692	D17Wsu92e
ENSMUSG00000056698	Elmod3
ENSMUSG00000056708	Ier5
ENSMUSG00000056752	Dnah9
ENSMUSG00000056755	Grm7
ENSMUSG00000056895	Hist3h2ba
ENSMUSG00000056952	Tatdn2
ENSMUSG00000056999	Ide
ENSMUSG00000057177	Gsk3a
ENSMUSG00000057182	Scn3a
ENSMUSG00000057230	Aak1
ENSMUSG00000057329	Bcl2
ENSMUSG00000057335	Cep170

ENSMUSG00000057359	Gm17494
ENSMUSG00000057367	Birc2
ENSMUSG00000057396	Zfp759
ENSMUSG00000057530	Ece1
ENSMUSG00000057554	Lgals8
ENSMUSG00000057738	Sptan1
ENSMUSG00000057897	Camk2b
ENSMUSG00000057914	Cacnb2
ENSMUSG00000058013	Sept11
ENSMUSG00000058135	Gstm1
ENSMUSG00000058152	Chsy3
ENSMUSG00000058192	Zfp846
ENSMUSG00000058254	Tspan7
ENSMUSG00000058317	Ube2e2
ENSMUSG00000058325	Dock1
ENSMUSG00000058388	Phtf1
ENSMUSG00000058589	Anks1b
ENSMUSG00000058600	Rpl30
ENSMUSG00000058740	Kcnt1
ENSMUSG00000058799	Nap1l1
ENSMUSG00000058966	Fam57b
ENSMUSG00000059003	Grin2a
ENSMUSG00000059173	Pde1a
ENSMUSG00000059273	Zc3h4
ENSMUSG00000059327	Eda
ENSMUSG00000059363	Fxn
ENSMUSG00000059401	Mamld1
ENSMUSG00000059552	Trp53
ENSMUSG00000059586	Nsmce2
ENSMUSG00000059602	Syn3
ENSMUSG00000059708	Akap17b
ENSMUSG00000059796	Eif4a1
ENSMUSG00000059834	Sclt1
ENSMUSG00000059854	Hydin
ENSMUSG00000059890	Ube4a
ENSMUSG00000059895	Ptp4a3
ENSMUSG00000059920	4930453N24Rik
ENSMUSG00000059970	Hspa2
ENSMUSG00000059975	Zfp74
ENSMUSG00000059991	Nptx2
ENSMUSG00000060090	Rp2
ENSMUSG00000060147	Serpinb6a
ENSMUSG00000060166	Zdhhc8
ENSMUSG00000060176	Kif27
ENSMUSG00000060227	Casc4
ENSMUSG00000060257	Scrt2
ENSMUSG00000060275	Nrg2
ENSMUSG00000060371	Caln1
ENSMUSG00000060487	Samd5
ENSMUSG00000060534	Dcc
ENSMUSG00000060639	Hist1h4i
ENSMUSG00000060671	Atp8b2
ENSMUSG00000060733	Ipmk

ENSMUSG00000060862	Zbtb40
ENSMUSG00000060882	Kcnd2
ENSMUSG00000060935	Tmem263
ENSMUSG00000060988	Galnt13
ENSMUSG00000061313	Ddhd2
ENSMUSG00000061374	Fiz1
ENSMUSG00000061410	Zcchc14
ENSMUSG00000061411	Nol4l
ENSMUSG00000061451	Tmem151a
ENSMUSG00000061458	Nol10
ENSMUSG00000061462	Obscn
ENSMUSG00000061479	Snrpa
ENSMUSG00000061533	Cep128
ENSMUSG00000061576	Dpp6
ENSMUSG00000061601	Pclo
ENSMUSG00000061689	Dlgap4
ENSMUSG00000061751	Kalrn
ENSMUSG00000061887	Ssbp3
ENSMUSG00000061898	Rbak
ENSMUSG00000062006	Rpl34
ENSMUSG00000062031	Pgghg
ENSMUSG00000062110	Scfd2
ENSMUSG00000062203	Gspt1
ENSMUSG00000062232	Rapgef2
ENSMUSG00000062257	Opcml
ENSMUSG00000062312	Erbb2
ENSMUSG00000062353	Gm15772
ENSMUSG00000062785	Kcnc3
ENSMUSG00000062901	Klhl24
ENSMUSG00000062906	Hdac10
ENSMUSG00000062944	9130023H24Rik
ENSMUSG00000062960	Kdr
ENSMUSG00000062981	Mrpl42
ENSMUSG00000062991	Nrg1
ENSMUSG00000063047	Zfp780b
ENSMUSG00000063063	Ctnna2
ENSMUSG00000063065	Mapk3
ENSMUSG00000063077	Kif1b
ENSMUSG00000063142	Kcnma1
ENSMUSG00000063316	Rpl27
ENSMUSG00000063358	Mapk1
ENSMUSG00000063564	Col23a1
ENSMUSG00000063600	Egfem1
ENSMUSG00000063646	Jakmip1
ENSMUSG00000063659	Zbtb18
ENSMUSG00000063714	Sp3os
ENSMUSG00000063760	Rnf217
ENSMUSG00000063765	Chadl
ENSMUSG00000063785	Utp14a
ENSMUSG00000063796	Slc22a8
ENSMUSG00000063801	Ap3s2
ENSMUSG00000063887	Nlgn1
ENSMUSG00000063902	Gm7964

ENSMUSG00000063919	Srrm4
ENSMUSG00000063972	Nr6a1
ENSMUSG00000064043	Trerf1
ENSMUSG00000064080	Fbln2
ENSMUSG00000064120	Mocs1
ENSMUSG00000064210	Ano6
ENSMUSG00000064220	Hist2h2aa1
ENSMUSG00000064225	Paqr9
ENSMUSG00000064302	Clasp1
ENSMUSG00000064329	Scn1a
ENSMUSG00000064451	Snora23
ENSMUSG00000064702	Gm24950
ENSMUSG00000064853	Gm23442
ENSMUSG00000064856	Gm23444
ENSMUSG00000065701	Rny1
ENSMUSG00000065767	Gm23849
ENSMUSG00000065944	Rnu2-10
ENSMUSG00000065990	Aurkaip1
ENSMUSG00000066037	Hnrnpr
ENSMUSG00000066151	Fkbp15
ENSMUSG00000066189	Cacng3
ENSMUSG00000066406	Akap13
ENSMUSG00000066613	Zfp932
ENSMUSG00000066621	Tecpr1
ENSMUSG00000066640	Fbxl18
ENSMUSG00000066643	Wdr35
ENSMUSG00000066877	Nck2
ENSMUSG00000066900	Suds3
ENSMUSG00000067194	Eif1ax
ENSMUSG00000067199	Frat1
ENSMUSG00000067336	Bmpr2
ENSMUSG00000067851	Arfgef1
ENSMUSG00000067889	Sptbn2
ENSMUSG00000067931	Zfp948
ENSMUSG00000068205	Macrod2
ENSMUSG00000068263	Efcc1
ENSMUSG00000068290	Ddrgk1
ENSMUSG00000068373	D430041D05Rik
ENSMUSG00000068523	Gng5
ENSMUSG00000068551	Zfp467
ENSMUSG00000068663	Clec16a
ENSMUSG00000068742	Cry2
ENSMUSG00000068747	Sort1
ENSMUSG00000068859	Sp9
ENSMUSG00000069020	Urm1
ENSMUSG00000069089	Cdk7
ENSMUSG00000069255	Dusp22
ENSMUSG00000069495	Epc2
ENSMUSG00000069631	Strada
ENSMUSG00000069662	Marcks
ENSMUSG00000069678	Pcgf1
ENSMUSG00000069729	Arid1b
ENSMUSG00000069814	Ccdc92b

ENSMUSG00000069833	Ahnak
ENSMUSG00000070000	Fcho1
ENSMUSG00000070462	Tlnrd1
ENSMUSG00000070565	Rasal2
ENSMUSG00000070570	Slc17a7
ENSMUSG00000070576	Mn1
ENSMUSG00000070808	Bicra
ENSMUSG00000070866	Zfp804a
ENSMUSG00000070923	Klhl9
ENSMUSG00000071226	Cecr2
ENSMUSG00000071265	1700086L19Rik
ENSMUSG00000071267	Zfp942
ENSMUSG00000071341	Egr4
ENSMUSG00000071369	Map3k5
ENSMUSG00000071454	Dtnb
ENSMUSG00000071456	1110002L01Rik
ENSMUSG00000071533	Pcnp
ENSMUSG00000071550	Cfap44
ENSMUSG00000071636	Rimbp3
ENSMUSG00000071645	Tut1
ENSMUSG00000071659	Hnrnpul2
ENSMUSG00000071660	Ttc9c
ENSMUSG00000071753	C230004F18Rik
ENSMUSG00000071866	Ppia
ENSMUSG00000072494	Ppp1r3e
ENSMUSG00000072501	Phf20l1
ENSMUSG00000072568	Fam84b
ENSMUSG00000072663	Spef2
ENSMUSG00000072769	Gm10419
ENSMUSG00000072949	Acot1
ENSMUSG00000073079	Srp54
ENSMUSG00000073102	Drc1
ENSMUSG00000073139	Tmem185a
ENSMUSG00000073295	Nudt11
ENSMUSG00000073424	Cyp4f15
ENSMUSG00000073434	Wdr90
ENSMUSG00000073514	Dok6
ENSMUSG00000073702	Rpl31
ENSMUSG00000073779	Lrp8os2
ENSMUSG00000073968	Trim68
ENSMUSG00000074165	Zfp788
ENSMUSG00000074212	Dnajb14
ENSMUSG00000074238	Ap1ar
ENSMUSG00000074259	Gramd2
ENSMUSG00000074283	Zfp109
ENSMUSG00000074305	Peak1
ENSMUSG00000074364	Ehd2
ENSMUSG00000074403	Hist2h3b
ENSMUSG00000074405	Zfp865
ENSMUSG00000074406	Zfp628
ENSMUSG00000074457	S100a16
ENSMUSG00000074664	A830092H15Rik
ENSMUSG00000074746	Pdzd8

ENSMUSG00000074749	Kiz
ENSMUSG00000074797	Itpa
ENSMUSG00000074994	Qser1
ENSMUSG00000075376	Rc3h2
ENSMUSG00000075389	2810410L24Rik
ENSMUSG00000075470	Deaf1
ENSMUSG00000075590	Nrbp2
ENSMUSG00000075700	Selenot
ENSMUSG00000076435	Acsf2
ENSMUSG00000077450	Rab11b
ENSMUSG00000078429	Ctdsp2
ENSMUSG00000078515	Ddi2
ENSMUSG00000078591	Hs3st4
ENSMUSG00000078656	Vps25
ENSMUSG00000078671	Chd2
ENSMUSG00000078816	Prkcg
ENSMUSG00000078897	Gm4724
ENSMUSG00000078908	Mon1b
ENSMUSG00000079003	Samd1
ENSMUSG00000079018	Ly6c1
ENSMUSG00000079056	Kcnip3
ENSMUSG00000079108	Srp54b
ENSMUSG00000079144	A130010J15Rik
ENSMUSG00000079184	Mphosph8
ENSMUSG00000079283	2310009B15Rik
ENSMUSG00000079477	Rab7
ENSMUSG00000079523	Tmsb10
ENSMUSG00000081058	Hist2h3c2
ENSMUSG00000081788	Gm5898
ENSMUSG00000084349	Rpl3-ps1
ENSMUSG00000084708	Gm22988
ENSMUSG00000084957	Bbip1
ENSMUSG00000084989	Crocc2
ENSMUSG00000085438	1700020I14Rik
ENSMUSG00000085492	Trmt61b
ENSMUSG00000085795	Zfp703
ENSMUSG00000086158	Ccpg1os
ENSMUSG00000086448	9330162012Rik
ENSMUSG00000086968	4933431E20Rik
ENSMUSG00000087370	Tmem170b
ENSMUSG00000087679	Tmem250-ps
ENSMUSG00000088529	Gm26083
ENSMUSG00000088835	Gm23547
ENSMUSG00000089704	Galnt2
ENSMUSG00000089715	Cbx6
ENSMUSG00000089809	Rasgef1b
ENSMUSG00000089832	Shkbp1
ENSMUSG00000089917	Uckl1
ENSMUSG00000089945	Pakap
ENSMUSG00000090000	Ier3ip1
ENSMUSG00000090015	Gm15446
ENSMUSG00000090053	Palm2
ENSMUSG00000090071	Cdk5r2

ENSMUSG00000090137	Uba52
ENSMUSG00000090213	Tmem189
ENSMUSG00000090266	Mettl23
ENSMUSG00000090330	9130221H12Rik
ENSMUSG00000090799	Klhl33
ENSMUSG00000091228	Gm20390
ENSMUSG00000091264	Smim13
ENSMUSG00000091415	Ak9
ENSMUSG00000091474	2610021A01Rik
ENSMUSG00000092274	Neat1
ENSMUSG00000092341	Malat1
ENSMUSG00000092558	Med20
ENSMUSG00000093452	Zfhx2os
ENSMUSG00000093843	Gm25939
ENSMUSG00000093880	Tmem181c-ps
ENSMUSG00000094050	Gm23472
ENSMUSG00000094483	Purb
ENSMUSG00000094626	Cecr6
ENSMUSG00000094627	Gm10182
ENSMUSG00000094910	D430019H16Rik
ENSMUSG00000095139	Pou3f2
ENSMUSG00000095253	Zfp799
ENSMUSG00000096010	Hist4h4
ENSMUSG00000096054	Syne1
ENSMUSG00000096659	Gm25679
ENSMUSG00000096768	Gm47283
ENSMUSG00000097061	9330151L19Rik
ENSMUSG00000097164	Cep83os
ENSMUSG00000097333	Zfp87
ENSMUSG00000097571	Jpx
ENSMUSG00000097583	6430590A07Rik
ENSMUSG00000097589	Dleu2
ENSMUSG00000097736	9530059O14Rik
ENSMUSG00000098051	Gm27032
ENSMUSG00000098134	Rnf113a2
ENSMUSG00000098234	Snhg6
ENSMUSG00000099681	1700052K11Rik
ENSMUSG00000100252	Mir124-2hg
ENSMUSG00000101013	A630072M18Rik
ENSMUSG00000101609	Kcnq1ot1
ENSMUSG00000102206	Pcdha11
ENSMUSG00000102222	Pcdhga10
ENSMUSG00000102312	Pcdha3
ENSMUSG00000102336	Gm37233
ENSMUSG00000102428	Pcdhga12
ENSMUSG00000102543	Pcdhgc5
ENSMUSG00000102692	Dchs2
ENSMUSG00000102697	Pcdhac2
ENSMUSG00000102742	Pcdhga11
ENSMUSG00000102918	Pcdhgc3
ENSMUSG00000103088	Pcdhgb6
ENSMUSG00000103144	Pcdhga1
ENSMUSG00000103214	Gm38286

ENSMUSG00000103332	Pcdhga2
ENSMUSG00000103749	Pcdhgb5
ENSMUSG00000103793	Pcdhga6
ENSMUSG00000103897	Pcdhga8
ENSMUSG00000104063	Pcdhgb7
ENSMUSG00000104148	Pcdha2
ENSMUSG00000104178	Gm9916
ENSMUSG00000104318	Pcdha7
ENSMUSG00000104696	Gm42946
ENSMUSG00000105827	Hist2h2bb
ENSMUSG00000106579	Gm42771
ENSMUSG00000106826	Gm42583
ENSMUSG00000106864	Gtf3c2
ENSMUSG00000107096	Gm43597
ENSMUSG00000107858	Gm43879
ENSMUSG00000108049	Gm44168
ENSMUSG00000108616	Gm35040
ENSMUSG00000109122	A230103L15Rik
ENSMUSG00000109321	A330076H08Rik
ENSMUSG00000109398	Gm3854
ENSMUSG00000109946	Brd3os
ENSMUSG00000110195	Pde2a
ENSMUSG00000113528	Gm32699
ENSMUSG00000114970	AC164978.1
ENSMUSG00000116766	NA
ENSMUSG00000116875	NA
ENSMUSG00000116908	NA
ENSMUSG00000117679	NA
ENSMUSG00000117814	NA
ENSMUSG00000118125	NA
ENSMUSG00000118202	NA
ENSMUSG00000001025	S100a6
ENSMUSG00000027002	Nckap1
ENSMUSG00000027799	Nbea
ENSMUSG00000073542	Cep76
ENSMUSG00000079469	Pigb
ENSMUSG00000027848	Olfml3
ENSMUSG00000029413	Naaa
ENSMUSG00000030629	Zfand6
ENSMUSG00000101355	Hist1h3h
ENSMUSG00000048402	Gli2
ENSMUSG00000021116	Eif2s1
ENSMUSG00000021730	Hcn1
ENSMUSG00000024810	Il33
ENSMUSG00000025656	Arhgef9
ENSMUSG00000026893	Gca
ENSMUSG00000047205	Dusp18
ENSMUSG00000063021	Hist1h2ak
ENSMUSG00000020354	Sgcd
ENSMUSG00000032826	Ank2
ENSMUSG00000041000	Trim62
ENSMUSG00000020492	Ska2
ENSMUSG00000022052	Ppp2r2a

ENSMUSG00000025066	Sfr1
ENSMUSG00000046865	Fbl
ENSMUSG00000059824	Dbp
ENSMUSG00000027597	Ahcy
ENSMUSG00000040943	Tet2
ENSMUSG00000043415	Otud1
ENSMUSG00000001052	Sec24b
ENSMUSG00000025188	Hps1
ENSMUSG00000060212	Pcnx2
ENSMUSG00000059486	Kbtbd2
ENSMUSG00000059839	Zfp874b
ENSMUSG00000006369	Fbln1
ENSMUSG00000026553	Copa
ENSMUSG00000034201	Gas2l1
ENSMUSG00000028184	Adgrl2
ENSMUSG00000036435	Exoc1
ENSMUSG00000052085	Dock8
ENSMUSG00000031154	Otud5
ENSMUSG00000063450	Syne2
ENSMUSG00000065954	Tacc1
ENSMUSG00000017670	Elmo2
ENSMUSG00000023460	Rab12
ENSMUSG00000037808	Fam76b
ENSMUSG00000005698	Ctcf
ENSMUSG00000007836	Hnrnpa0
ENSMUSG00000015942	Gtf2ird2
ENSMUSG00000027714	Exosc9
ENSMUSG00000029918	Mrps33
ENSMUSG00000031639	Tlr3
ENSMUSG00000031828	Klhl36
ENSMUSG00000031833	Mast3
ENSMUSG00000035064	Eef2k
ENSMUSG00000035382	Pcsk7
ENSMUSG00000060336	Zfp937
ENSMUSG00000094777	Hist1h2ap
ENSMUSG00000006456	Rbm14
ENSMUSG00000018999	Slc35b4
ENSMUSG00000030801	Kat8
ENSMUSG00000041649	Klf8
ENSMUSG00000056602	Fry
ENSMUSG00000056204	Pgpep1
ENSMUSG00000059923	Grb2
ENSMUSG00000052512	Nav2
ENSMUSG00000002985	Apoe
ENSMUSG00000022091	Sorbs3
ENSMUSG00000025223	Ldb1
ENSMUSG00000026764	Kif5c
ENSMUSG00000028164	Manba
ENSMUSG00000031207	Msn
ENSMUSG00000031666	Rbl2
ENSMUSG00000032551	1110059G10Rik
ENSMUSG00000038828	Tmem214
ENSMUSG00000039747	Orai2

ENSMUSG00000062151	Unc13c
ENSMUSG00000109562	Gm45178
ENSMUSG00000032589	Bsn
ENSMUSG00000038384	Setd1b
ENSMUSG00000024663	Rab3il1
ENSMUSG00000027498	Cstf1
ENSMUSG00000037224	Zfyve28
ENSMUSG00000039976	Tbc1d16
ENSMUSG00000005397	Nid1
ENSMUSG00000015468	Notch4
ENSMUSG00000020647	Ncoa1
ENSMUSG00000020883	Fbxl20
ENSMUSG00000031816	Mthfsd
ENSMUSG00000064115	Cadm2
ENSMUSG00000003037	Rab8a
ENSMUSG00000030649	Anapc15
ENSMUSG00000037001	Zfp39
ENSMUSG00000040761	Spen
ENSMUSG00000027433	Xrn2
ENSMUSG00000038604	Ripor1
ENSMUSG00000038893	Fam117a
ENSMUSG00000039989	Cbx4
ENSMUSG00000040699	Limd2
ENSMUSG00000026341	Actr3
ENSMUSG00000036550	Cnot1
ENSMUSG00000053141	Ptprt
ENSMUSG00000024251	Thada
ENSMUSG00000019139	Isyna1
ENSMUSG00000020889	Nr1d1
ENSMUSG00000034832	Tet3
ENSMUSG00000006567	Atp7b
ENSMUSG00000046603	Tcaim
ENSMUSG00000091345	Col6a5
ENSMUSG00000073418	C4b
ENSMUSG00000069895	Atxn1l
ENSMUSG00000034463	Scara3

BNST_MaleUp_FemaleDown

ENSEMBL_ID	gene_name
ENSMUSG00000071516	Hist1h2ai
ENSMUSG00000078786	BC024978
ENSMUSG00000036707	Cab39
ENSMUSG00000062908	Acadm
ENSMUSG00000002845	Tmem39a
ENSMUSG00000021466	Ptch1
ENSMUSG00000039704	Lmbrd2
ENSMUSG00000087598	Zfp111
ENSMUSG00000039640	Mrpl12
ENSMUSG00000024099	Ndufv2
ENSMUSG00000034566	Atp5h
ENSMUSG00000029167	Ppargc1a
ENSMUSG00000027651	Rprd1b
ENSMUSG00000005510	Ndufs3
ENSMUSG00000021277	Traf3

ENSMUSG00000022551	Cyc1
ENSMUSG00000028016	Ints12
ENSMUSG00000037174	Elf2
ENSMUSG00000040383	Aqr
ENSMUSG00000053929	Cyhr1
ENSMUSG00000035964	Tmem59l
ENSMUSG00000008348	Ubc
ENSMUSG00000063882	Uqcrh
ENSMUSG00000025289	Prdx4
ENSMUSG00000026311	Asb1
ENSMUSG00000039089	L3mbtl3
ENSMUSG00000048915	Efna5
ENSMUSG00000031782	Coq9
ENSMUSG00000074872	Ctxn2
ENSMUSG00000014856	Tmem208
ENSMUSG00000000563	Atp5f1
ENSMUSG00000026566	Mpzl1
ENSMUSG00000033208	S100b
ENSMUSG00000034602	Mon2
ENSMUSG00000019505	Ubb
ENSMUSG00000031344	Gabrq
ENSMUSG00000031667	Aktip
ENSMUSG00000047003	Zfp41
ENSMUSG00000067365	Tmem128
ENSMUSG00000070287	Slc35g2
ENSMUSG00000072889	Nfxl1
ENSMUSG00000035934	Pknox2
ENSMUSG00000024598	Fbn2
ENSMUSG00000032412	Atp1b3
ENSMUSG00000049760	2410015M20Rik
ENSMUSG00000025613	Cct8
ENSMUSG00000001518	Itfg2
ENSMUSG00000002345	Borcs8
ENSMUSG00000017428	Psmd11
ENSMUSG00000020076	Ddx50
ENSMUSG00000021290	2010107E04Rik
ENSMUSG00000032479	Map4
ENSMUSG00000035431	Sstr1
ENSMUSG00000038542	Pcid2
ENSMUSG00000040451	Sgms1
ENSMUSG00000057766	Ankrd29
ENSMUSG00000100862	Gm10925
ENSMUSG00000103677	Pcdhga4
ENSMUSG00000021615	Xrcc4
ENSMUSG00000020922	Lsm12
ENSMUSG00000030695	Aldoa
ENSMUSG00000038489	Polr2l
ENSMUSG00000050608	Minos1
ENSMUSG00000046330	Rpl37a
ENSMUSG00000001674	Ddx18
ENSMUSG00000002661	Alkbh7
ENSMUSG00000004815	Dgkq
ENSMUSG00000014606	Slc25a11

ENSMUSG00000018820	Zfyve27
ENSMUSG00000020570	Sypl
ENSMUSG00000022019	Tdrd3
ENSMUSG00000029176	Anapc4
ENSMUSG00000029777	Gars
ENSMUSG00000038301	Snx10
ENSMUSG00000038702	Dsel
ENSMUSG00000041035	Gm17018
ENSMUSG00000048000	Gigyf2
ENSMUSG00000054676	1600014C10Rik
ENSMUSG00000061414	Cracr2a
ENSMUSG00000061724	Gm2423
ENSMUSG00000002250	Ppard
ENSMUSG00000031990	Jam3
ENSMUSG00000063694	Cycs
ENSMUSG00000034880	Mrpl34
ENSMUSG00000071655	Ubxn1
ENSMUSG00000027257	Pacsin3
ENSMUSG00000031848	Lsm4
ENSMUSG00000035242	Oaz1
ENSMUSG00000070709	Zfp974
ENSMUSG00000028284	Map3k7
ENSMUSG00000017421	Zfp207
ENSMUSG00000033233	Trim45
ENSMUSG00000110523	C230057M02Rik
ENSMUSG00000003934	Efnb3
ENSMUSG00000032313	Tmem266
ENSMUSG00000024943	Smc5
ENSMUSG00000027170	Eif3m
ENSMUSG00000029162	Khk
ENSMUSG00000027581	Stmn3
ENSMUSG00000030357	Fkbp4
ENSMUSG00000042462	Dctpp1
ENSMUSG00000049659	Aftph
ENSMUSG00000056201	Cfl1
ENSMUSG00000062270	Morf4l1
ENSMUSG00000047603	Zfp235
ENSMUSG00000005357	Slc1a6
ENSMUSG00000026867	Gapvd1
ENSMUSG00000029126	Nsg1
ENSMUSG00000028546	Elavl4
ENSMUSG00000011148	Adssl1
ENSMUSG00000028937	Acot7
ENSMUSG00000059540	Tcea2
ENSMUSG00000073792	Alg6
ENSMUSG00000010406	Mrpl52
ENSMUSG00000022425	Enpp2
ENSMUSG00000035161	Ints6
ENSMUSG00000022471	Xrcc6
ENSMUSG00000090671	Gm5067
ENSMUSG00000020132	Rab21
ENSMUSG00000024816	Frmd8
ENSMUSG00000001774	Chordc1

ENSMUSG00000020994	Pnn
ENSMUSG00000021832	Psmc6
ENSMUSG00000027998	Plrg1
ENSMUSG00000051359	Ncald
ENSMUSG00000014303	Glis2
ENSMUSG00000025321	Itgb8
ENSMUSG00000028393	Alad
ENSMUSG00000050002	Idnk
ENSMUSG00000005378	Bud23
ENSMUSG00000022438	Parvb
ENSMUSG00000026753	Ppp6c
ENSMUSG00000031347	Cetn2
ENSMUSG00000042104	Uggt2
ENSMUSG00000066551	Hmgb1
ENSMUSG00000038909	Kat7
ENSMUSG00000070348	Ccnd1
ENSMUSG00000000078	Klf6
ENSMUSG00000000085	Scmh1
ENSMUSG00000000088	Cox5a
ENSMUSG00000000131	Xpo6
ENSMUSG00000000142	Axin2
ENSMUSG00000000168	Dlat
ENSMUSG00000000171	Sdhd
ENSMUSG00000000194	Gpr107
ENSMUSG00000000266	Mid2
ENSMUSG00000000275	Trim25
ENSMUSG00000000295	Hddc2
ENSMUSG00000000325	Arvcf
ENSMUSG00000000355	Mcts1
ENSMUSG00000000420	Galnt1
ENSMUSG00000000538	Tom1l2
ENSMUSG00000000594	Gm2a
ENSMUSG00000000605	Clcn4
ENSMUSG00000000627	Sema4f
ENSMUSG00000000804	Usp32
ENSMUSG00000000827	Tpd52l2
ENSMUSG00000000876	Pxmp4
ENSMUSG00000000934	Top1mt
ENSMUSG00000000948	Gm38393
ENSMUSG00000001056	Nhp2
ENSMUSG00000001100	Poldip2
ENSMUSG00000001105	Ift20
ENSMUSG00000001127	Araf
ENSMUSG00000001260	Gabrg1
ENSMUSG00000001285	Myg1
ENSMUSG00000001289	Pfdn5
ENSMUSG00000001366	Fbxo9
ENSMUSG00000001383	Zmat2
ENSMUSG00000001415	Smg5
ENSMUSG00000001424	Snd1
ENSMUSG00000001435	Col18a1
ENSMUSG00000001467	Cyp51
ENSMUSG00000001482	Def8

ENSMUSG00000001506	Col1a1
ENSMUSG00000001520	Nrip2
ENSMUSG00000001700	Gramd3
ENSMUSG00000001755	Ppat
ENSMUSG00000001767	Crnkl1
ENSMUSG00000001768	Rin2
ENSMUSG00000001794	Capns1
ENSMUSG00000001802	Lrp3
ENSMUSG00000001870	Ltbp1
ENSMUSG00000001962	Fam50a
ENSMUSG00000001964	Emd
ENSMUSG00000001998	Ap4e1
ENSMUSG00000002012	Pnck
ENSMUSG00000002015	Bcap31
ENSMUSG00000002017	Fam98a
ENSMUSG00000002058	Unc119
ENSMUSG00000002064	Sdf2
ENSMUSG00000002233	Rhoc
ENSMUSG00000002265	Peg3
ENSMUSG00000002266	Zim1
ENSMUSG00000002274	Metrn
ENSMUSG00000002289	Angptl4
ENSMUSG00000002320	Tm9sf1
ENSMUSG00000002326	Gmpr2
ENSMUSG00000002329	Mdp1
ENSMUSG00000002332	Dhrs1
ENSMUSG00000002343	Armc6
ENSMUSG00000002365	Snx9
ENSMUSG00000002372	Ranbp3
ENSMUSG00000002379	Ndufa11
ENSMUSG00000002395	Use1
ENSMUSG00000002458	Rgs19
ENSMUSG00000002475	Abhd3
ENSMUSG00000002504	Slc9a3r2
ENSMUSG00000002602	Axl
ENSMUSG00000002617	Zfp40
ENSMUSG00000002660	Clpp
ENSMUSG00000002731	Prkra
ENSMUSG00000002768	Mea1
ENSMUSG00000002771	Grin2d
ENSMUSG00000002778	Kdelr1
ENSMUSG00000002804	Nudt14
ENSMUSG00000002820	Atg4d
ENSMUSG00000002881	Nab1
ENSMUSG00000002908	Kcnn1
ENSMUSG00000002910	Arrdc2
ENSMUSG00000002980	Bcam
ENSMUSG00000003062	Stard3nl
ENSMUSG00000003070	Efna2
ENSMUSG00000003072	Atp5d
ENSMUSG00000003099	Ppp5c
ENSMUSG00000003184	Irf3
ENSMUSG00000003234	Abcf3

ENSMUSG00000003235	Eif2b5
ENSMUSG00000003316	Glg1
ENSMUSG00000003380	Rabac1
ENSMUSG00000003382	Etv3
ENSMUSG00000003402	Prkcsh
ENSMUSG00000003411	Rab3b
ENSMUSG00000003418	St8sia6
ENSMUSG00000003476	Crhr2
ENSMUSG00000003531	Dgcr6
ENSMUSG00000003549	Ercc1
ENSMUSG00000003581	Rnf215
ENSMUSG00000003657	Calb2
ENSMUSG00000003731	Kpna6
ENSMUSG00000003812	Dnase2a
ENSMUSG00000003813	Rad23a
ENSMUSG00000003848	Nob1
ENSMUSG00000003868	Ruvbl2
ENSMUSG00000003872	Lin7b
ENSMUSG00000003923	Tfam
ENSMUSG00000003948	Mmd
ENSMUSG00000003970	Rpl8
ENSMUSG00000004018	Fancl
ENSMUSG00000004035	Gstm7
ENSMUSG00000004040	Stat3
ENSMUSG00000004069	Dnaja3
ENSMUSG00000004070	Hmox2
ENSMUSG00000004071	Cdip1
ENSMUSG00000004207	Psap
ENSMUSG00000004267	Eno2
ENSMUSG00000004268	Emg1
ENSMUSG00000004285	Atp6v1f
ENSMUSG00000004347	Pde1c
ENSMUSG00000004356	Utp20
ENSMUSG00000004364	Cul3
ENSMUSG00000004561	Mettl17
ENSMUSG00000004591	Pkn2
ENSMUSG00000004633	Chn2
ENSMUSG00000004642	Slbp
ENSMUSG00000004748	Mtfp1
ENSMUSG00000004788	Eif2b2
ENSMUSG00000004880	Lbr
ENSMUSG00000004896	Rrnad1
ENSMUSG00000004902	Slc25a18
ENSMUSG00000004934	Pias4
ENSMUSG00000004961	Syt5
ENSMUSG00000005034	Prkacb
ENSMUSG00000005043	Sgsh
ENSMUSG00000005103	Wdr1
ENSMUSG00000005150	Wdr83
ENSMUSG00000005161	Prdx2
ENSMUSG00000005268	Prlr
ENSMUSG00000005354	Txn2
ENSMUSG00000005469	Prkaca

ENSMUSG00000005481	Ddx39
ENSMUSG00000005566	Trim28
ENSMUSG00000005610	Eif4g2
ENSMUSG00000005656	Snx6
ENSMUSG00000005683	Cs
ENSMUSG00000005779	Psmb4
ENSMUSG00000005813	Metap1
ENSMUSG00000005873	Reep5
ENSMUSG00000005882	Uqcc1
ENSMUSG00000005897	Nr2c1
ENSMUSG00000006021	Kptn
ENSMUSG00000006050	Sra1
ENSMUSG00000006057	Atp5g1
ENSMUSG00000006270	Vax1
ENSMUSG00000006273	Atp6v1b2
ENSMUSG00000006281	Tep1
ENSMUSG00000006333	Rps9
ENSMUSG00000006335	Tfpt
ENSMUSG00000006395	Hyi
ENSMUSG00000006418	Rnf114
ENSMUSG00000006423	C330007P06Rik
ENSMUSG00000006463	Zdhhc24
ENSMUSG00000006464	Bbs1
ENSMUSG00000006527	Sfmbt1
ENSMUSG00000006576	Slc4a3
ENSMUSG00000006651	Aplp1
ENSMUSG00000006699	Cdc42
ENSMUSG00000006782	Cnp
ENSMUSG00000006800	Sulf2
ENSMUSG00000006906	Stambp
ENSMUSG00000006941	Eif1b
ENSMUSG00000007021	Syngr3
ENSMUSG00000007038	Neu1
ENSMUSG00000007097	Atp1a2
ENSMUSG00000007458	M6pr
ENSMUSG00000007564	Ppp2r1a
ENSMUSG00000007570	Fance
ENSMUSG00000007603	Dus3l
ENSMUSG00000007653	Gabrb2
ENSMUSG00000007659	Bcl2l1
ENSMUSG00000007682	Dio2
ENSMUSG00000007739	Cct4
ENSMUSG00000007815	Rhoa
ENSMUSG00000007987	Ift22
ENSMUSG00000008318	Relt
ENSMUSG00000008435	Rdh13
ENSMUSG00000008489	Elavl2
ENSMUSG00000008540	Mgst1
ENSMUSG00000008575	Nfib
ENSMUSG00000008682	Rpl10
ENSMUSG00000008892	Vdac3
ENSMUSG00000008999	Bmp7
ENSMUSG00000009535	Rnmt

ENSMUSG00000009549	Srp14
ENSMUSG00000009566	Fpgs
ENSMUSG00000009614	Sardh
ENSMUSG00000009647	Mcu
ENSMUSG00000009733	Tfcp2
ENSMUSG00000009863	Sdhb
ENSMUSG00000009894	Snap47
ENSMUSG00000009905	Kdsr
ENSMUSG00000010025	Aldh3a2
ENSMUSG00000010044	Zmynd10
ENSMUSG00000010045	Tmem115
ENSMUSG00000010047	Hyal2
ENSMUSG00000010057	Nprl2
ENSMUSG00000010066	Cacna2d2
ENSMUSG00000010067	Rassf1
ENSMUSG00000010154	Spire2
ENSMUSG00000010290	AI597479
ENSMUSG00000010362	Rdm1
ENSMUSG00000010376	Nedd8
ENSMUSG00000010453	Kansl3
ENSMUSG00000010609	Psen2
ENSMUSG00000010755	Cars
ENSMUSG00000010911	Apip
ENSMUSG00000010936	Vac14
ENSMUSG00000011752	Pgam1
ENSMUSG00000012483	Rpa3
ENSMUSG00000012609	Ttll5
ENSMUSG00000012819	Cdh23
ENSMUSG00000012848	Rps5
ENSMUSG00000013160	Atp6v0d1
ENSMUSG00000013367	Iglon5
ENSMUSG00000013539	Tango2
ENSMUSG00000013593	Ndufs2
ENSMUSG00000013646	Sh3bp5l
ENSMUSG00000013662	Atad1
ENSMUSG00000013698	Pea15a
ENSMUSG00000013701	Timm23
ENSMUSG00000013787	Ehmt2
ENSMUSG00000013822	Elof1
ENSMUSG00000013878	Rnf170
ENSMUSG00000014039	Prdm15
ENSMUSG00000014294	Ndufa2
ENSMUSG00000014547	Wdfy2
ENSMUSG00000014550	Rbsn
ENSMUSG00000014668	Chfr
ENSMUSG00000014846	Tppp3
ENSMUSG00000014867	Surf4
ENSMUSG00000014956	Ppp1cb
ENSMUSG00000015013	Trappc2l
ENSMUSG00000015023	Ddx19a
ENSMUSG00000015027	Galns
ENSMUSG00000015092	Edf1
ENSMUSG00000015214	Mtmr1

ENSMUSG00000015222	Map2
ENSMUSG00000015484	Fam163a
ENSMUSG00000015522	Arnt
ENSMUSG00000015575	Atp6v0e
ENSMUSG00000015597	Zfp318
ENSMUSG00000015656	Hspa8
ENSMUSG00000015668	Pdzd11
ENSMUSG00000015697	Setdb1
ENSMUSG00000015711	Prune1
ENSMUSG00000015750	Aph1a
ENSMUSG00000015776	Med22
ENSMUSG00000015804	Med28
ENSMUSG00000015806	Qdpr
ENSMUSG00000015882	Lcorl
ENSMUSG00000015932	Dstn
ENSMUSG00000015937	H2afy
ENSMUSG00000015994	Fnta
ENSMUSG00000016150	Tenm1
ENSMUSG00000016252	Atp5e
ENSMUSG00000016257	Prelid3b
ENSMUSG00000016382	Pls3
ENSMUSG00000016495	Plgrkt
ENSMUSG00000016510	Mtif3
ENSMUSG00000016541	Atxn10
ENSMUSG00000016664	Pacsin2
ENSMUSG00000016757	Ttll12
ENSMUSG00000016940	Kctd2
ENSMUSG00000017132	Cyth1
ENSMUSG00000017188	Coa3
ENSMUSG00000017314	Mpp2
ENSMUSG00000017386	Traf4
ENSMUSG00000017631	Abr
ENSMUSG00000017667	Zfp334
ENSMUSG00000017679	Ttpal
ENSMUSG00000017713	Tha1
ENSMUSG00000017724	Etv4
ENSMUSG00000017734	Dbndd2
ENSMUSG00000017776	Crk
ENSMUSG00000017778	Cox7c
ENSMUSG00000017929	B4galt5
ENSMUSG00000017943	Gdap1l1
ENSMUSG00000017978	Cadps2
ENSMUSG00000018001	Cyth3
ENSMUSG00000018012	Rac3
ENSMUSG00000018040	Rrp7a
ENSMUSG00000018068	Ints2
ENSMUSG00000018143	Mafk
ENSMUSG00000018171	Vmp1
ENSMUSG00000018189	Uchl5
ENSMUSG00000018286	Psmb6
ENSMUSG00000018322	Tomm34
ENSMUSG00000018326	Ywhab
ENSMUSG00000018339	Gpx3

ENSMUSG00000018378	Cuedc1
ENSMUSG00000018379	Srsf1
ENSMUSG00000018405	Mrm1
ENSMUSG00000018411	Mapt
ENSMUSG00000018412	Kansl1
ENSMUSG00000018417	Myo1b
ENSMUSG00000018449	Rpain
ENSMUSG00000018459	Slc13a3
ENSMUSG00000018583	G3bp1
ENSMUSG00000018589	Glra2
ENSMUSG00000018593	Sparc
ENSMUSG00000018599	Mief2
ENSMUSG00000018659	Pnpo
ENSMUSG00000018661	Cog1
ENSMUSG00000018677	Slc25a39
ENSMUSG00000018770	Atp5g3
ENSMUSG00000018848	Rars
ENSMUSG00000018861	Fdxr
ENSMUSG00000018865	Sult4a1
ENSMUSG00000018882	Mrpl45
ENSMUSG00000018900	Slc22a5
ENSMUSG00000018906	P4ha2
ENSMUSG00000018932	Map2k3
ENSMUSG00000018995	Nars2
ENSMUSG00000019027	Dnah1
ENSMUSG00000019054	Fis1
ENSMUSG00000019087	Atp6ap1
ENSMUSG00000019124	Scrn1
ENSMUSG00000019143	Hars2
ENSMUSG00000019158	Tmem160
ENSMUSG00000019188	H13
ENSMUSG00000019232	Etnppl
ENSMUSG00000019261	Map1s
ENSMUSG00000019295	Tmem129
ENSMUSG00000019338	Zfp687
ENSMUSG00000019362	D8Ertd738e
ENSMUSG00000019370	Calm3
ENSMUSG00000019433	Gipc1
ENSMUSG00000019467	Arhgef25
ENSMUSG00000019518	Ap4m1
ENSMUSG00000019528	Gyg
ENSMUSG00000019577	Pdk4
ENSMUSG00000019590	Cyb561
ENSMUSG00000019689	Fmc1
ENSMUSG00000019710	Mrpl24
ENSMUSG00000019726	Lyst
ENSMUSG00000019791	Hint3
ENSMUSG00000019795	Pcmt1
ENSMUSG00000019803	Nr2e1
ENSMUSG00000019809	Pex3
ENSMUSG00000019810	Fuca2
ENSMUSG00000019854	Reps1
ENSMUSG00000019877	Serinc1

ENSMUSG00000019889	Ptprk
ENSMUSG00000019892	Lrriq1
ENSMUSG00000019920	Lims1
ENSMUSG00000019923	Zwint
ENSMUSG00000019947	Arid5b
ENSMUSG00000019951	Uhrf1bp1l
ENSMUSG00000019970	Sgk1
ENSMUSG00000019971	Cep290
ENSMUSG00000019979	Apaf1
ENSMUSG00000019986	Ahi1
ENSMUSG00000020019	Ntn4
ENSMUSG00000020023	Tmcc3
ENSMUSG00000020070	Rufy2
ENSMUSG00000020085	Aifm2
ENSMUSG00000020086	H2afy2
ENSMUSG00000020087	Tysnd1
ENSMUSG00000020089	Ppa1
ENSMUSG00000020090	Npffr1
ENSMUSG00000020107	Anapc16
ENSMUSG00000020108	Ddit4
ENSMUSG00000020111	Micu1
ENSMUSG00000020114	Cand1
ENSMUSG00000020115	Tbk1
ENSMUSG00000020122	Egfr
ENSMUSG00000020130	Tbc1d15
ENSMUSG00000020133	2310011J03Rik
ENSMUSG00000020135	Apc2
ENSMUSG00000020137	Thap2
ENSMUSG00000020142	Slc1a4
ENSMUSG00000020151	Ptprr
ENSMUSG00000020153	Ndufs7
ENSMUSG00000020170	Frs2
ENSMUSG00000020171	Yeats4
ENSMUSG00000020182	Ddc
ENSMUSG00000020183	Cpm
ENSMUSG00000020184	Mdm2
ENSMUSG00000020230	Prmt2
ENSMUSG00000020231	Dip2a
ENSMUSG00000020235	Fzr1
ENSMUSG00000020260	Pofut2
ENSMUSG00000020267	Hint1
ENSMUSG00000020289	Nprl3
ENSMUSG00000020311	Erlec1
ENSMUSG00000020328	Nudcd2
ENSMUSG00000020359	Phykpl
ENSMUSG00000020375	Rufy1
ENSMUSG00000020381	Mrnip
ENSMUSG00000020386	Sar1b
ENSMUSG00000020402	Vdac1
ENSMUSG00000020436	Gabrg2
ENSMUSG00000020440	Arf5
ENSMUSG00000020448	Rnf185
ENSMUSG00000020460	Rps27a

ENSMUSG00000020474	Polm
ENSMUSG00000020477	Mrps24
ENSMUSG00000020482	Ccdc117
ENSMUSG00000020484	Xbp1
ENSMUSG00000020485	Supt4a
ENSMUSG00000020486	Sept4
ENSMUSG00000020520	Galnt10
ENSMUSG00000020525	Ppm1d
ENSMUSG00000020526	Znhit3
ENSMUSG00000020528	Prpsap2
ENSMUSG00000020532	Acaca
ENSMUSG00000020546	Stxbp4
ENSMUSG00000020576	Nbas
ENSMUSG00000020598	Nrcam
ENSMUSG00000020604	Arsg
ENSMUSG00000020612	Prkar1a
ENSMUSG00000020629	Adi1
ENSMUSG00000020646	Mboat2
ENSMUSG00000020650	Bcap29
ENSMUSG00000020656	Grhl1
ENSMUSG00000020664	Dld
ENSMUSG00000020681	Ace
ENSMUSG00000020692	Nle1
ENSMUSG00000020706	Ftsj3
ENSMUSG00000020716	Nf1
ENSMUSG00000020720	Psmd12
ENSMUSG00000020736	Nt5c
ENSMUSG00000020737	Hn1
ENSMUSG00000020739	Nup85
ENSMUSG00000020755	Sap30bp
ENSMUSG00000020770	Unk
ENSMUSG00000020781	Tsen54
ENSMUSG00000020805	Slc13a5
ENSMUSG00000020807	4933427D14Rik
ENSMUSG00000020811	Wscd1
ENSMUSG00000020818	Mfsd11
ENSMUSG00000020821	Kif1c
ENSMUSG00000020834	Dhrs13
ENSMUSG00000020843	Timm22
ENSMUSG00000020857	Nme2
ENSMUSG00000020866	Cacna1g
ENSMUSG00000020877	Scrn2
ENSMUSG00000020892	Aloxe3
ENSMUSG00000020899	Pfas
ENSMUSG00000020902	Ntn1
ENSMUSG00000020903	Stx8
ENSMUSG00000020919	Stat5b
ENSMUSG00000020925	Ccdc43
ENSMUSG00000020929	Eftud2
ENSMUSG00000020936	Nmt1
ENSMUSG00000020948	Klhl28
ENSMUSG00000020962	Gtf2a1
ENSMUSG00000020978	Klhdc2

ENSMUSG00000020982	Nemf
ENSMUSG00000021013	Ttc8
ENSMUSG00000021018	Polr2h
ENSMUSG00000021023	1110008L16Rik
ENSMUSG00000021024	Psma6
ENSMUSG00000021027	Ralgapa1
ENSMUSG00000021033	Gstz1
ENSMUSG00000021067	Sav1
ENSMUSG00000021076	Actr10
ENSMUSG00000021136	Smoc1
ENSMUSG00000021178	Psmc1
ENSMUSG00000021217	Tshz3
ENSMUSG00000021218	Gdi2
ENSMUSG00000021235	Coq6
ENSMUSG00000021236	Entpd5
ENSMUSG00000021241	Isca2
ENSMUSG00000021242	Npc2
ENSMUSG00000021254	Gpatch2l
ENSMUSG00000021257	Angel1
ENSMUSG00000021270	Hsp90aa1
ENSMUSG00000021279	Cdc42bpb
ENSMUSG00000021282	Eif5
ENSMUSG00000021340	Gpld1
ENSMUSG00000021365	Nedd9
ENSMUSG00000021375	Kif13a
ENSMUSG00000021377	Dek
ENSMUSG00000021411	Pxdc1
ENSMUSG00000021428	Riok1
ENSMUSG00000021432	Slc35b3
ENSMUSG00000021451	Sema4d
ENSMUSG00000021458	2010111I01Rik
ENSMUSG00000021460	Auh
ENSMUSG00000021477	Ctsl
ENSMUSG00000021482	Aaed1
ENSMUSG00000021486	Prelid1
ENSMUSG00000021501	Caml
ENSMUSG00000021504	B4galt7
ENSMUSG00000021519	Mterf3
ENSMUSG00000021520	Uqcrb
ENSMUSG00000021534	1700001L19Rik
ENSMUSG00000021548	Ccnh
ENSMUSG00000021550	2210016F16Rik
ENSMUSG00000021555	Naa35
ENSMUSG00000021573	Tppp
ENSMUSG00000021578	Ccdc127
ENSMUSG00000021583	Erap1
ENSMUSG00000021587	Pcsk1
ENSMUSG00000021591	Glrx
ENSMUSG00000021606	Ndufs6
ENSMUSG00000021607	Mrpl36
ENSMUSG00000021613	Hapln1
ENSMUSG00000021614	Vcan
ENSMUSG00000021621	Zcchc9

ENSMUSG00000021629	Slc30a5
ENSMUSG00000021635	Rad17
ENSMUSG00000021647	Cartpt
ENSMUSG00000021662	Arhgef28
ENSMUSG00000021670	Hmgcr
ENSMUSG00000021671	Poc5
ENSMUSG00000021693	Kif2a
ENSMUSG00000021703	Serinc5
ENSMUSG00000021706	Zfyve16
ENSMUSG00000021711	Trappc13
ENSMUSG00000021721	Htr1a
ENSMUSG00000021737	Psmd6
ENSMUSG00000021754	Map3k1
ENSMUSG00000021771	Vdac2
ENSMUSG00000021772	Nkiras1
ENSMUSG00000021773	Comtd1
ENSMUSG00000021785	Ngly1
ENSMUSG00000021792	Fam213a
ENSMUSG00000021794	Glud1
ENSMUSG00000021806	Nid2
ENSMUSG00000021831	Ero1l
ENSMUSG00000021877	Arf4
ENSMUSG00000021929	Kpna3
ENSMUSG00000021952	Xpo4
ENSMUSG00000021959	Lats2
ENSMUSG00000021969	Zdhhc20
ENSMUSG00000021981	Cab39l
ENSMUSG00000021982	Cdadc1
ENSMUSG00000021987	Mtmr6
ENSMUSG00000021996	Esd
ENSMUSG00000022010	Tsc22d1
ENSMUSG00000022024	Sugt1
ENSMUSG00000022035	Ccdc25
ENSMUSG00000022051	Bnip3l
ENSMUSG00000022055	Nefl
ENSMUSG00000022076	Klhl1
ENSMUSG00000022090	Pdlim2
ENSMUSG00000022092	Ppp3cc
ENSMUSG00000022096	Hr
ENSMUSG00000022110	Sucla2
ENSMUSG00000022122	Ednrb
ENSMUSG00000022151	Ttc33
ENSMUSG00000022159	Rab2b
ENSMUSG00000022179	4931414P19Rik
ENSMUSG00000022193	Psmb5
ENSMUSG00000022199	Slc22a17
ENSMUSG00000022205	Sub1
ENSMUSG00000022212	Cpne6
ENSMUSG00000022217	Emc9
ENSMUSG00000022234	Cct5
ENSMUSG00000022248	Rad1
ENSMUSG00000022257	Laptm4b
ENSMUSG00000022263	Trio

ENSMUSG00000022272	Myo10
ENSMUSG00000022285	Ywhaz
ENSMUSG00000022296	Baalc
ENSMUSG00000022313	Utp23
ENSMUSG00000022329	Stk3
ENSMUSG00000022337	Emc2
ENSMUSG00000022340	Sybu
ENSMUSG00000022354	Ndufb9
ENSMUSG00000022360	Atad2
ENSMUSG00000022370	Mrpl13
ENSMUSG00000022376	Adcy8
ENSMUSG00000022391	Rangap1
ENSMUSG00000022400	Rbx1
ENSMUSG00000022404	Slc25a17
ENSMUSG00000022407	Adsl
ENSMUSG00000022419	Deptor
ENSMUSG00000022428	Cby1
ENSMUSG00000022436	Sh3bp1
ENSMUSG00000022452	Smdt1
ENSMUSG00000022456	Sept3
ENSMUSG00000022466	Rpap3
ENSMUSG00000022469	Rapgef3
ENSMUSG00000022474	Pmm1
ENSMUSG00000022477	Aco2
ENSMUSG00000022507	1810013L24Rik
ENSMUSG00000022516	Nudt16l1
ENSMUSG00000022525	Hrasls
ENSMUSG00000022536	Glyr1
ENSMUSG00000022538	Lsg1
ENSMUSG00000022546	Gpt
ENSMUSG00000022548	Apod
ENSMUSG00000022555	Dgat1
ENSMUSG00000022558	Mroh1
ENSMUSG00000022571	Pycrl
ENSMUSG00000022574	Naprt
ENSMUSG00000022577	Ly6h
ENSMUSG00000022587	Ly6e
ENSMUSG00000022607	Ptk2
ENSMUSG00000022629	Kif21a
ENSMUSG00000022677	Fopnl
ENSMUSG00000022682	Rrn3
ENSMUSG00000022684	Bfar
ENSMUSG00000022696	Sidt1
ENSMUSG00000022710	Usp7
ENSMUSG00000022721	Trmt2a
ENSMUSG00000022724	Riox2
ENSMUSG00000022748	Cmss1
ENSMUSG00000022751	Nit2
ENSMUSG00000022763	Aifm3
ENSMUSG00000022774	Ncbp2
ENSMUSG00000022783	Spag6
ENSMUSG00000022787	Wdr53
ENSMUSG00000022800	Fyttd1

ENSMUSG00000022801	Lrch3
ENSMUSG00000022808	Snx4
ENSMUSG00000022820	Ndufb4
ENSMUSG00000022822	Abcc5
ENSMUSG00000022841	Ap2m1
ENSMUSG00000022842	Ece2
ENSMUSG00000022861	Dgkg
ENSMUSG00000022865	Cxadr
ENSMUSG00000022884	Eif4a2
ENSMUSG00000022893	Adamts1
ENSMUSG00000022899	Slc15a2
ENSMUSG00000022911	Arl13b
ENSMUSG00000022913	Psmg1
ENSMUSG00000022914	Brwd1
ENSMUSG00000022935	Grik1
ENSMUSG00000022946	Dopey2
ENSMUSG00000022949	Clic6
ENSMUSG00000022951	Rcan1
ENSMUSG00000022957	Itsn1
ENSMUSG00000022964	Tmem50b
ENSMUSG00000022982	Sod1
ENSMUSG00000022987	Zfp641
ENSMUSG00000022994	Adcy6
ENSMUSG00000023004	Tuba1b
ENSMUSG00000023010	Tmbim6
ENSMUSG00000023017	Asic1
ENSMUSG00000023020	Cox14
ENSMUSG00000023021	Cers5
ENSMUSG00000023025	Larp4
ENSMUSG00000023030	Slc11a2
ENSMUSG00000023048	Prr13
ENSMUSG00000023050	Map3k12
ENSMUSG00000023051	Tarbp2
ENSMUSG00000023067	Cdkn1a
ENSMUSG00000023094	Msrb2
ENSMUSG00000023104	Rfc2
ENSMUSG00000023175	Bsg
ENSMUSG00000023191	P3h3
ENSMUSG00000023235	Ccl25
ENSMUSG00000023236	Scg5
ENSMUSG00000023266	Frs3
ENSMUSG00000023328	Ache
ENSMUSG00000023707	Ogfod2
ENSMUSG00000023723	Mrps23
ENSMUSG00000023809	Rps6ka2
ENSMUSG00000023827	Agpat4
ENSMUSG00000023852	Chd1
ENSMUSG00000023885	Thbs2
ENSMUSG00000023904	Hcfc1r1
ENSMUSG00000023911	Flywch2
ENSMUSG00000023932	Cdc5l
ENSMUSG00000023938	Aars2
ENSMUSG00000023953	Polh

ENSMUSG00000023966	Rsph9
ENSMUSG00000023973	Cnpy3
ENSMUSG00000023984	Gm20517
ENSMUSG00000024007	Ppil1
ENSMUSG00000024011	Pi16
ENSMUSG00000024012	Mtch1
ENSMUSG00000024038	Ndufv3
ENSMUSG00000024042	Sik1
ENSMUSG00000024044	Epb41l3
ENSMUSG00000024068	Spast
ENSMUSG00000024069	Slc30a6
ENSMUSG00000024082	Ndufaf7
ENSMUSG00000024084	Qpct
ENSMUSG00000024091	Vapa
ENSMUSG00000024096	Ralbp1
ENSMUSG00000024121	Atp6v0c
ENSMUSG00000024127	Prepl
ENSMUSG00000024140	Epas1
ENSMUSG00000024146	Cript
ENSMUSG00000024150	Mcfd2
ENSMUSG00000024170	Telo2
ENSMUSG00000024181	Mrpl28
ENSMUSG00000024193	Phf1
ENSMUSG00000024194	Cuta
ENSMUSG00000024211	Grm8
ENSMUSG00000024222	Fkbp5
ENSMUSG00000024241	Sos1
ENSMUSG00000024247	Pkdcc
ENSMUSG00000024248	Cox7a2l
ENSMUSG00000024253	Dync2li1
ENSMUSG00000024261	Syt4
ENSMUSG00000024269	Tpgs2
ENSMUSG00000024271	Elp2
ENSMUSG00000024276	Zfp397
ENSMUSG00000024287	Thoc1
ENSMUSG00000024299	Adamts10
ENSMUSG00000024309	Pfdn6
ENSMUSG00000024357	Sil1
ENSMUSG00000024381	Bin1
ENSMUSG00000024404	Riok3
ENSMUSG00000024423	Impact
ENSMUSG00000024424	Ttc39c
ENSMUSG00000024425	Ndfip1
ENSMUSG00000024429	Gnl1
ENSMUSG00000024457	Trim26
ENSMUSG00000024480	Ap3s1
ENSMUSG00000024487	Yipf5
ENSMUSG00000024507	Hsd17b4
ENSMUSG00000024542	Cep192
ENSMUSG00000024563	Smad2
ENSMUSG00000024565	Sall3
ENSMUSG00000024566	Atp9b
ENSMUSG00000024608	Rps14

ENSMUSG00000024614	Tmx3
ENSMUSG00000024647	Cbln2
ENSMUSG00000024664	Fads3
ENSMUSG00000024725	Ostf1
ENSMUSG00000024732	Ccdc86
ENSMUSG00000024735	Prpf19
ENSMUSG00000024754	Tmem2
ENSMUSG00000024767	Otub1
ENSMUSG00000024772	Ehd1
ENSMUSG00000024776	Stambpl1
ENSMUSG00000024781	Lipa
ENSMUSG00000024798	Htr7
ENSMUSG00000024800	Rpp30
ENSMUSG00000024805	Pcgf5
ENSMUSG00000024817	Uhrf2
ENSMUSG00000024826	Dpf2
ENSMUSG00000024829	Mrpl21
ENSMUSG00000024843	Chka
ENSMUSG00000024851	Pitpnm1
ENSMUSG00000024862	Klc2
ENSMUSG00000024873	Cnih2
ENSMUSG00000024875	Yif1a
ENSMUSG00000024891	Slc29a2
ENSMUSG00000024896	Minpp1
ENSMUSG00000024901	Peli3
ENSMUSG00000024902	Mrpl11
ENSMUSG00000024907	Gal
ENSMUSG00000024914	Drap1
ENSMUSG00000024925	Rnaseh2c
ENSMUSG00000024944	Arl2
ENSMUSG00000024947	Men1
ENSMUSG00000024953	Prdx5
ENSMUSG00000024963	Dnajc4
ENSMUSG00000024966	Stip1
ENSMUSG00000025020	Slit1
ENSMUSG00000025037	Maoa
ENSMUSG00000025040	Fundc1
ENSMUSG00000025049	Taf5
ENSMUSG00000025068	Gsto1
ENSMUSG00000025077	Dclre1a
ENSMUSG00000025086	Trub1
ENSMUSG00000025094	Slc18a2
ENSMUSG00000025133	Ints4
ENSMUSG00000025139	Tollip
ENSMUSG00000025151	Maged1
ENSMUSG00000025154	Arhgap19
ENSMUSG00000025175	Fn3k
ENSMUSG00000025190	Got1
ENSMUSG00000025200	Cwf19l1
ENSMUSG00000025204	Ndufb8
ENSMUSG00000025208	Mrpl43
ENSMUSG00000025217	Btrc
ENSMUSG00000025218	Poll

ENSMUSG00000025236	Adpgk
ENSMUSG00000025255	Zfhx4
ENSMUSG00000025264	Tsr2
ENSMUSG00000025268	Maged2
ENSMUSG00000025326	Ube3a
ENSMUSG00000025351	Cd63
ENSMUSG00000025362	Rps26
ENSMUSG00000025375	Aatk
ENSMUSG00000025377	Tepsin
ENSMUSG00000025407	Gli1
ENSMUSG00000025408	Ddit3
ENSMUSG00000025421	Hdhd2
ENSMUSG00000025425	St8sia5
ENSMUSG00000025436	Atp23
ENSMUSG00000025437	Usp33
ENSMUSG00000025451	Paip1
ENSMUSG00000025465	Echs1
ENSMUSG00000025466	Fuom
ENSMUSG00000025468	Caly
ENSMUSG00000025485	Ric8a
ENSMUSG00000025499	Hras
ENSMUSG00000025508	Rplp2
ENSMUSG00000025510	Cd151
ENSMUSG00000025511	Tspan4
ENSMUSG00000025512	Chid1
ENSMUSG00000025529	Zfp711
ENSMUSG00000025534	Gusb
ENSMUSG00000025538	Sumf2
ENSMUSG00000025558	Dock9
ENSMUSG00000025597	Klhl4
ENSMUSG00000025602	Zfp202
ENSMUSG00000025608	Podxl
ENSMUSG00000025630	Hprt
ENSMUSG00000025647	Shisa5
ENSMUSG00000025666	Tmem47
ENSMUSG00000025702	March8
ENSMUSG00000025723	Nmb
ENSMUSG00000025728	Pigq
ENSMUSG00000025731	Mettl26
ENSMUSG00000025733	Rhot2
ENSMUSG00000025743	Sdc3
ENSMUSG00000025757	Hspa4l
ENSMUSG00000025766	D3Ertd751e
ENSMUSG00000025777	Gdap1
ENSMUSG00000025785	Exosc7
ENSMUSG00000025786	Zdhhc3
ENSMUSG00000025789	St8sia2
ENSMUSG00000025790	Slco3a1
ENSMUSG00000025791	Pgm2
ENSMUSG00000025792	Slc25a10
ENSMUSG00000025794	Rpl14
ENSMUSG00000025810	Nrp1
ENSMUSG00000025813	Homer2

ENSMUSG00000025855	Prkar1b
ENSMUSG00000025856	Pdgfa
ENSMUSG00000025869	Nop16
ENSMUSG00000025873	Faf2
ENSMUSG00000025878	Uimc1
ENSMUSG00000025885	Myo5b
ENSMUSG00000025905	Oprk1
ENSMUSG00000025909	Sntg1
ENSMUSG00000025915	Sgk3
ENSMUSG00000025920	Stau2
ENSMUSG00000025925	Terf1
ENSMUSG00000025940	Tmem70
ENSMUSG00000025950	Idh1
ENSMUSG00000025956	Mettl21a
ENSMUSG00000025959	Klf7
ENSMUSG00000025968	Ndufs1
ENSMUSG00000025969	Nrp2
ENSMUSG00000025982	Sf3b1
ENSMUSG00000026003	Acadl
ENSMUSG00000026005	Rpe
ENSMUSG00000026019	Wdr12
ENSMUSG00000026020	Nop58
ENSMUSG00000026021	Sumo1
ENSMUSG00000026027	Stradb
ENSMUSG00000026028	Trak2
ENSMUSG00000026031	Cflar
ENSMUSG00000026034	Clk1
ENSMUSG00000026035	Ppil3
ENSMUSG00000026043	Col3a1
ENSMUSG00000026078	Pdcl3
ENSMUSG00000026095	Asnsd1
ENSMUSG00000026098	Pms1
ENSMUSG00000026109	Tmeff2
ENSMUSG00000026110	Mgat4a
ENSMUSG00000026123	Plekhb2
ENSMUSG00000026154	Sdhaf4
ENSMUSG00000026156	B3gat2
ENSMUSG00000026163	Sphkap
ENSMUSG00000026179	Pnkd
ENSMUSG00000026187	Xrcc5
ENSMUSG00000026193	Fn1
ENSMUSG00000026199	Ankzf1
ENSMUSG00000026200	Glb1l
ENSMUSG00000026201	Stk16
ENSMUSG00000026223	Itm2c
ENSMUSG00000026224	4933407L21Rik
ENSMUSG00000026247	Ecel1
ENSMUSG00000026273	Mterf4
ENSMUSG00000026275	Ppp1r7
ENSMUSG00000026279	Thap4
ENSMUSG00000026305	Lrrfip1
ENSMUSG00000026307	Scly
ENSMUSG00000026309	Ilkap

ENSMUSG00000026317	Cln8
ENSMUSG00000026344	Lypd1
ENSMUSG00000026360	Rgs2
ENSMUSG00000026377	Nifk
ENSMUSG00000026421	Csrp1
ENSMUSG00000026427	Eif2d
ENSMUSG00000026437	Cdk18
ENSMUSG00000026455	Klhl12
ENSMUSG00000026473	Glul
ENSMUSG00000026490	Cdc42bpa
ENSMUSG00000026499	Acbd3
ENSMUSG00000026526	Fh1
ENSMUSG00000026558	Uck2
ENSMUSG00000026563	Tada1
ENSMUSG00000026576	Atp1b1
ENSMUSG00000026577	Blzf1
ENSMUSG00000026578	Ccdc181
ENSMUSG00000026587	Astn1
ENSMUSG00000026594	Ralgps2
ENSMUSG00000026610	Esrrg
ENSMUSG00000026617	Bpnt1
ENSMUSG00000026627	Tmem206
ENSMUSG00000026641	Usf1
ENSMUSG00000026648	Dclre1c
ENSMUSG00000026662	Sephs1
ENSMUSG00000026667	Uhmk1
ENSMUSG00000026672	Optn
ENSMUSG00000026698	Pigc
ENSMUSG00000026705	Klhl20
ENSMUSG00000026727	Rsu1
ENSMUSG00000026754	Golga1
ENSMUSG00000026755	Arpc5l
ENSMUSG00000026766	Mmadhc
ENSMUSG00000026767	Mindy3
ENSMUSG00000026781	Acbd5
ENSMUSG00000026787	Gad2
ENSMUSG00000026792	Lrsam1
ENSMUSG00000026803	Ttf1
ENSMUSG00000026817	Ak1
ENSMUSG00000026820	Ptges2
ENSMUSG00000026841	Fibcd1
ENSMUSG00000026842	Abl1
ENSMUSG00000026853	Crat
ENSMUSG00000026857	Ntmt1
ENSMUSG00000026880	Stom
ENSMUSG00000026889	Rbm18
ENSMUSG00000026895	Ndufa8
ENSMUSG00000026914	Psmd14
ENSMUSG00000026944	Abca2
ENSMUSG00000026956	Uap1l1
ENSMUSG00000026966	Ssna1
ENSMUSG00000026988	Wdsub1
ENSMUSG00000026991	Pkp4

ENSMUSG00000027006	Dnajc10
ENSMUSG00000027012	Dync1i2
ENSMUSG00000027079	Clp1
ENSMUSG00000027091	Zc3h15
ENSMUSG00000027104	Atf2
ENSMUSG00000027163	Commd9
ENSMUSG00000027166	Dnajc24
ENSMUSG00000027168	Pax6
ENSMUSG00000027175	Tcp11l1
ENSMUSG00000027176	Cstf3
ENSMUSG00000027193	Api5
ENSMUSG00000027207	Galk2
ENSMUSG00000027217	Tspan18
ENSMUSG00000027220	Syt13
ENSMUSG00000027236	Eif3j1
ENSMUSG00000027255	Arfgap2
ENSMUSG00000027263	Tubgcp4
ENSMUSG00000027272	Ubr1
ENSMUSG00000027282	Mtch2
ENSMUSG00000027286	Lrrc57
ENSMUSG00000027303	Ptpra
ENSMUSG00000027304	Rtf1
ENSMUSG00000027309	4930402H24Rik
ENSMUSG00000027313	Chac1
ENSMUSG00000027315	Spint1
ENSMUSG00000027316	Gfra4
ENSMUSG00000027342	Pcna
ENSMUSG00000027359	Slc27a2
ENSMUSG00000027365	Trpm7
ENSMUSG00000027367	Stard7
ENSMUSG00000027423	Snx5
ENSMUSG00000027430	Dtd1
ENSMUSG00000027434	Nkx2-2
ENSMUSG00000027455	Nsfl1c
ENSMUSG00000027459	Fam110a
ENSMUSG00000027472	Pdrg1
ENSMUSG00000027489	Necab3
ENSMUSG00000027499	Pkia
ENSMUSG00000027500	Stmn2
ENSMUSG00000027506	Tpd52
ENSMUSG00000027508	Pag1
ENSMUSG00000027523	Gnas
ENSMUSG00000027525	Phactr3
ENSMUSG00000027550	Lrrcc1
ENSMUSG00000027583	Zbtb46
ENSMUSG00000027584	Oprl1
ENSMUSG00000027589	Pcmtd2
ENSMUSG00000027602	Map1lc3a
ENSMUSG00000027603	Ggt7
ENSMUSG00000027605	Acss2
ENSMUSG00000027613	Eif6
ENSMUSG00000027618	Nfs1
ENSMUSG00000027628	Aar2

ENSMUSG00000027649	Ctnnbl1
ENSMUSG00000027655	Dhx35
ENSMUSG00000027660	Skil
ENSMUSG00000027663	Zmat3
ENSMUSG00000027667	Zfp639
ENSMUSG00000027668	Mfn1
ENSMUSG00000027671	Actl6a
ENSMUSG00000027673	Ndufb5
ENSMUSG00000027680	Fxr1
ENSMUSG00000027694	Gm8325
ENSMUSG00000027706	Sec62
ENSMUSG00000027708	Dcun1d1
ENSMUSG00000027710	Acad9
ENSMUSG00000027746	Ufm1
ENSMUSG00000027748	Trpc4
ENSMUSG00000027770	Dhx36
ENSMUSG00000027787	Nmd3
ENSMUSG00000027797	Dclk1
ENSMUSG00000027809	Etfdh
ENSMUSG00000027822	Slc33a1
ENSMUSG00000027823	Gmps
ENSMUSG00000027854	Sike1
ENSMUSG00000027893	Ahcyl1
ENSMUSG00000027895	Kcnc4
ENSMUSG00000027932	Slc27a3
ENSMUSG00000027937	Jtb
ENSMUSG00000027950	Chrnb2
ENSMUSG00000027953	Slc50a1
ENSMUSG00000027956	Tmem144
ENSMUSG00000027963	Extl2
ENSMUSG00000027971	Ndst4
ENSMUSG00000027977	Ndst3
ENSMUSG00000028004	Npy2r
ENSMUSG00000028013	Ppa2
ENSMUSG00000028018	Gstcd
ENSMUSG00000028020	Glrb
ENSMUSG00000028029	Aimp1
ENSMUSG00000028035	Dnajb4
ENSMUSG00000028057	Rit1
ENSMUSG00000028063	Lmna
ENSMUSG00000028081	Rps3a1
ENSMUSG00000028098	Rnf115
ENSMUSG00000028099	Polr3c
ENSMUSG00000028104	Polr3gl
ENSMUSG00000028107	Tars2
ENSMUSG00000028134	Ptbp2
ENSMUSG00000028138	Adh5
ENSMUSG00000028218	Fam92a
ENSMUSG00000028223	Decr1
ENSMUSG00000028233	Tgs1
ENSMUSG00000028252	Ccnc
ENSMUSG00000028277	Ube2j1
ENSMUSG00000028292	Rars2

ENSMUSG00000028293	Slc35a1
ENSMUSG00000028309	Rnf20
ENSMUSG00000028322	Exosc3
ENSMUSG00000028337	Coro2a
ENSMUSG00000028343	Erp44
ENSMUSG00000028345	Tex10
ENSMUSG00000028354	Fmn2
ENSMUSG00000028373	Astn2
ENSMUSG00000028381	Ugcg
ENSMUSG00000028389	Zfp37
ENSMUSG00000028431	Ikbkap
ENSMUSG00000028445	Enho
ENSMUSG00000028453	Fancg
ENSMUSG00000028454	Pigo
ENSMUSG00000028461	Ccdc107
ENSMUSG00000028468	Rgp1
ENSMUSG00000028476	Reck
ENSMUSG00000028479	Gne
ENSMUSG00000028488	Sh3gl2
ENSMUSG00000028495	Rps6
ENSMUSG00000028514	Usp24
ENSMUSG00000028538	St3gal3
ENSMUSG00000028540	Dph2
ENSMUSG00000028560	Usp1
ENSMUSG00000028578	Caap1
ENSMUSG00000028603	Scp2
ENSMUSG00000028618	Tmem59
ENSMUSG00000028619	Tceanc2
ENSMUSG00000028633	Ctps
ENSMUSG00000028636	Ppcs
ENSMUSG00000028645	Slc2a1
ENSMUSG00000028646	Rragc
ENSMUSG00000028647	Mycbp
ENSMUSG00000028653	Trit1
ENSMUSG00000028655	Mfsd2a
ENSMUSG00000028664	Ephb2
ENSMUSG00000028668	Eloa
ENSMUSG00000028677	Rnf220
ENSMUSG00000028680	Plk3
ENSMUSG00000028683	Eif2b3
ENSMUSG00000028684	Urod
ENSMUSG00000028688	Toe1
ENSMUSG00000028690	Mmachc
ENSMUSG00000028691	Prdx1
ENSMUSG00000028692	Akr1a1
ENSMUSG00000028698	Pik3r3
ENSMUSG00000028700	Pomgnt1
ENSMUSG00000028708	Mknk1
ENSMUSG00000028719	Cmpk1
ENSMUSG00000028729	Ebna1bp2
ENSMUSG00000028737	Aldh4a1
ENSMUSG00000028741	Mrto4
ENSMUSG00000028745	Capzb

ENSMUSG00000028753	Vwa5b1
ENSMUSG00000028756	Pink1
ENSMUSG00000028759	Hp1bp3
ENSMUSG00000028766	Alpl
ENSMUSG00000028772	Zcchc17
ENSMUSG00000028779	Pef1
ENSMUSG00000028792	Ak2
ENSMUSG00000028795	Ccdc28b
ENSMUSG00000028796	Phc2
ENSMUSG00000028803	Nipal3
ENSMUSG00000028832	Stmn1
ENSMUSG00000028837	Psmb2
ENSMUSG00000028840	Zfp593
ENSMUSG00000028843	Sh3bgrl3
ENSMUSG00000028845	Tekt2
ENSMUSG00000028847	Trappc3
ENSMUSG00000028850	Gpatch3
ENSMUSG00000028857	Tmem222
ENSMUSG00000028882	Ppp1r8
ENSMUSG00000028893	Sesn2
ENSMUSG00000028896	Rcc1
ENSMUSG00000028914	Casp9
ENSMUSG00000028932	Psmc2
ENSMUSG00000028953	Abcf2
ENSMUSG00000028959	Fastk
ENSMUSG00000028962	Slc4a2
ENSMUSG00000028964	Park7
ENSMUSG00000028973	Abcb8
ENSMUSG00000028986	Klhl7
ENSMUSG00000028992	Nmnat1
ENSMUSG00000028998	Tomm7
ENSMUSG00000029001	Fbxo44
ENSMUSG00000029036	Atad3a
ENSMUSG00000029038	Ssu72
ENSMUSG00000029047	Pex10
ENSMUSG00000029064	Gnb1
ENSMUSG00000029066	Mrpl20
ENSMUSG00000029068	Ccnl2
ENSMUSG00000029071	Dvl1
ENSMUSG00000029076	Sdf4
ENSMUSG00000029084	Cd38
ENSMUSG00000029088	Kcnip4
ENSMUSG00000029090	Adgra3
ENSMUSG00000029103	Lrpap1
ENSMUSG00000029123	Stk32b
ENSMUSG00000029125	Stx18
ENSMUSG00000029145	Eif2b4
ENSMUSG00000029147	Ppm1g
ENSMUSG00000029166	Mapre3
ENSMUSG00000029196	Tada2b
ENSMUSG00000029201	Ugdh
ENSMUSG00000029203	Ube2k
ENSMUSG00000029206	Nsun7

ENSMUSG00000029213	Commd8
ENSMUSG00000029219	Slc10a4
ENSMUSG00000029223	Uchl1
ENSMUSG00000029227	Fip1l1
ENSMUSG00000029228	Lnx1
ENSMUSG00000029231	Pdgfra
ENSMUSG00000029233	Srd5a3
ENSMUSG00000029234	Tmem165
ENSMUSG00000029246	Ppat
ENSMUSG00000029247	Paics
ENSMUSG00000029250	Polr2b
ENSMUSG00000029263	Pigg
ENSMUSG00000029267	Mtf2
ENSMUSG00000029270	Fam69a
ENSMUSG00000029276	Glmn
ENSMUSG00000029313	Aff1
ENSMUSG00000029319	Coq2
ENSMUSG00000029348	Asphd2
ENSMUSG00000029364	Wsb2
ENSMUSG00000029366	Dck
ENSMUSG00000029376	Mthfd2l
ENSMUSG00000029401	Rilpl2
ENSMUSG00000029408	Abcb9
ENSMUSG00000029422	Rsrc2
ENSMUSG00000029427	Zcchc8
ENSMUSG00000029430	Ran
ENSMUSG00000029438	Bcl7a
ENSMUSG00000029440	Psmd9
ENSMUSG00000029447	Cct6a
ENSMUSG00000029462	Vps29
ENSMUSG00000029464	Gpn3
ENSMUSG00000029466	Anapc7
ENSMUSG00000029468	P2rx7
ENSMUSG00000029469	Ift81
ENSMUSG00000029480	Dhx37
ENSMUSG00000029500	Pgam5
ENSMUSG00000029504	Ddx51
ENSMUSG00000029507	Pus1
ENSMUSG00000029518	Rab35
ENSMUSG00000029524	Sirt4
ENSMUSG00000029545	Acads
ENSMUSG00000029557	Mrm2
ENSMUSG00000029559	2210016L21Rik
ENSMUSG00000029560	Snx8
ENSMUSG00000029614	Rpl6
ENSMUSG00000029616	Erp29
ENSMUSG00000029622	Arpc1b
ENSMUSG00000029627	Zkscan14
ENSMUSG00000029632	Ndufa4
ENSMUSG00000029635	Cdk8
ENSMUSG00000029647	Pan3
ENSMUSG00000029649	Pomp
ENSMUSG00000029669	Tspan12

ENSMUSG00000029672	Fam3c
ENSMUSG00000029676	Pot1a
ENSMUSG00000029681	Bcl7b
ENSMUSG00000029701	Rbm28
ENSMUSG00000029708	Gcc1
ENSMUSG00000029714	Gigyf1
ENSMUSG00000029715	Pop7
ENSMUSG00000029725	Ppp1r35
ENSMUSG00000029752	Asns
ENSMUSG00000029776	Hibadh
ENSMUSG00000029787	Avl9
ENSMUSG00000029810	Tmem176b
ENSMUSG00000029817	Tra2a
ENSMUSG00000029863	Casp2
ENSMUSG00000030007	Cct7
ENSMUSG00000030029	Lrig1
ENSMUSG00000030030	1700003E16Rik
ENSMUSG00000030032	Wdr54
ENSMUSG00000030035	Wbp1
ENSMUSG00000030042	Pole4
ENSMUSG00000030059	Tmf1
ENSMUSG00000030062	Rpn1
ENSMUSG00000030077	Chl1
ENSMUSG00000030092	Cntn6
ENSMUSG00000030095	Tmem43
ENSMUSG00000030110	Ret
ENSMUSG00000030145	Zfp248
ENSMUSG00000030161	Gabarapl1
ENSMUSG00000030204	Ddx47
ENSMUSG00000030209	Grin2b
ENSMUSG00000030254	Rad18
ENSMUSG00000030268	Bcat1
ENSMUSG00000030282	Cmas
ENSMUSG00000030283	St8sia1
ENSMUSG00000030307	Slc6a11
ENSMUSG00000030309	Caprin2
ENSMUSG00000030310	Slc6a1
ENSMUSG00000030313	Dennd5b
ENSMUSG00000030317	Timp4
ENSMUSG00000030335	Mrpl51
ENSMUSG00000030407	Qpctl
ENSMUSG00000030409	Dmpk
ENSMUSG00000030428	Ttyh1
ENSMUSG00000030447	Cyfip1
ENSMUSG00000030469	Zfp719
ENSMUSG00000030495	Slc7a10
ENSMUSG00000030521	Mphosph10
ENSMUSG00000030534	Vps33b
ENSMUSG00000030539	Sema4b
ENSMUSG00000030554	Synm
ENSMUSG00000030556	Lrrc28
ENSMUSG00000030584	Dpf1
ENSMUSG00000030592	Ryr1

ENSMUSG00000030595	Nfkbib
ENSMUSG00000030603	Psmc4
ENSMUSG00000030611	Mrps11
ENSMUSG00000030612	Mrpl46
ENSMUSG00000030616	Sytl2
ENSMUSG00000030638	Sh3gl3
ENSMUSG00000030647	Ndufc2
ENSMUSG00000030660	Pik3c2a
ENSMUSG00000030662	Ipo5
ENSMUSG00000030683	Sez6l2
ENSMUSG00000030685	Kctd13
ENSMUSG00000030688	Stard10
ENSMUSG00000030691	Fchsd2
ENSMUSG00000030701	Plekhb1
ENSMUSG00000030706	Mrpl48
ENSMUSG00000030707	Coro1a
ENSMUSG00000030714	Sgf29
ENSMUSG00000030720	Cln3
ENSMUSG00000030744	Rps3
ENSMUSG00000030754	Copb1
ENSMUSG00000030757	Zkscan2
ENSMUSG00000030768	Disp1
ENSMUSG00000030774	Pak1
ENSMUSG00000030780	BC017158
ENSMUSG00000030788	Rnf141
ENSMUSG00000030802	Bckdk
ENSMUSG00000030815	Phkg2
ENSMUSG00000030823	9130019O22Rik
ENSMUSG00000030824	Nucb1
ENSMUSG00000030839	Sergef
ENSMUSG00000030842	Lamtor1
ENSMUSG00000030844	Rgs10
ENSMUSG00000030852	Tacc2
ENSMUSG00000030861	Acadsb
ENSMUSG00000030871	Ears2
ENSMUSG00000030880	Polr3e
ENSMUSG00000030888	Rrp8
ENSMUSG00000030934	Oat
ENSMUSG00000030956	Fam53b
ENSMUSG00000030986	Dhx32
ENSMUSG00000030987	Stim1
ENSMUSG00000031021	Tmem9b
ENSMUSG00000031028	Tub
ENSMUSG00000031059	Ndufb11
ENSMUSG00000031060	Rbm10
ENSMUSG00000031066	Usp11
ENSMUSG00000031075	Ano1
ENSMUSG00000031078	Cttn
ENSMUSG00000031111	Igsf1
ENSMUSG00000031119	Gpc4
ENSMUSG00000031137	Fgf13
ENSMUSG00000031144	Syp
ENSMUSG00000031149	Praf2

ENSMUSG00000031150	Ccdc120
ENSMUSG00000031158	Timm17b
ENSMUSG00000031161	Hdac6
ENSMUSG00000031169	Porcn
ENSMUSG00000031171	Ftsj1
ENSMUSG00000031197	Vbp1
ENSMUSG00000031198	Fundc2
ENSMUSG00000031201	Brcc3
ENSMUSG00000031210	Gpr165
ENSMUSG00000031214	Ophn1
ENSMUSG00000031224	Magee2
ENSMUSG00000031227	Magee1
ENSMUSG00000031239	Itm2a
ENSMUSG00000031242	2610002M06Rik
ENSMUSG00000031246	Sh3bgrl
ENSMUSG00000031255	Sytl4
ENSMUSG00000031256	Cstf2
ENSMUSG00000031297	Slc7a3
ENSMUSG00000031299	Pdha1
ENSMUSG00000031327	Chic1
ENSMUSG00000031337	Mtm1
ENSMUSG00000031343	Gabra3
ENSMUSG00000031349	Nsdhl
ENSMUSG00000031355	Arhgap6
ENSMUSG00000031358	Msl3
ENSMUSG00000031360	Ctps2
ENSMUSG00000031402	Mpp1
ENSMUSG00000031403	Dkc1
ENSMUSG00000031409	Tceal6
ENSMUSG00000031422	Morf4l2
ENSMUSG00000031428	Zcchc18
ENSMUSG00000031453	Rasa3
ENSMUSG00000031458	Coprs
ENSMUSG00000031467	Agpat5
ENSMUSG00000031479	Vps36
ENSMUSG00000031482	Slc25a15
ENSMUSG00000031502	Col4a1
ENSMUSG00000031505	Naxd
ENSMUSG00000031508	Ankrd10
ENSMUSG00000031516	Dctn6
ENSMUSG00000031532	Saraf
ENSMUSG00000031543	Ank1
ENSMUSG00000031556	Tm2d2
ENSMUSG00000031575	Ash2l
ENSMUSG00000031585	Gtf2e2
ENSMUSG00000031604	Msmo1
ENSMUSG00000031608	Galnt7
ENSMUSG00000031622	Sin3b
ENSMUSG00000031673	Cdh11
ENSMUSG00000031700	Gpt2
ENSMUSG00000031729	Ist1
ENSMUSG00000031730	Dhodh
ENSMUSG00000031751	Amfr

ENSMUSG00000031753	Cog4
ENSMUSG00000031760	Mt3
ENSMUSG00000031776	Arl2bp
ENSMUSG00000031783	Polr2c
ENSMUSG00000031813	Mvb12a
ENSMUSG00000031818	Cox4i1
ENSMUSG00000031823	Zdhhc7
ENSMUSG00000031824	6430548M08Rik
ENSMUSG00000031827	Cotl1
ENSMUSG00000031837	Necab2
ENSMUSG00000031864	Ints10
ENSMUSG00000031897	Psmb10
ENSMUSG00000031903	Pla2g15
ENSMUSG00000031913	Vps4a
ENSMUSG00000031916	Cog8
ENSMUSG00000031917	Nip7
ENSMUSG00000031921	Terf2
ENSMUSG00000031930	Wwp2
ENSMUSG00000031950	Gabarapl2
ENSMUSG00000031955	Bcar1
ENSMUSG00000031969	Acad8
ENSMUSG00000031974	Abcb10
ENSMUSG00000031984	2810004N23Rik
ENSMUSG00000031993	Snx19
ENSMUSG00000032026	Rexo2
ENSMUSG00000032038	St3gal4
ENSMUSG00000032047	Acat1
ENSMUSG00000032051	Fdx1
ENSMUSG00000032060	Cryab
ENSMUSG00000032077	Bud13
ENSMUSG00000032078	Zpr1
ENSMUSG00000032101	Ddx25
ENSMUSG00000032115	Hyou1
ENSMUSG00000032123	Dpagt1
ENSMUSG00000032127	Vps11
ENSMUSG00000032171	Pin1
ENSMUSG00000032182	Yipf2
ENSMUSG00000032193	Ldlr
ENSMUSG00000032244	Fem1b
ENSMUSG00000032245	Cln6
ENSMUSG00000032253	Phip
ENSMUSG00000032262	Elovl4
ENSMUSG00000032264	Zw10
ENSMUSG00000032279	Idh3a
ENSMUSG00000032289	Thsd4
ENSMUSG00000032297	Celf6
ENSMUSG00000032298	Neil1
ENSMUSG00000032312	Csk
ENSMUSG00000032324	Tspan3
ENSMUSG00000032350	Gclc
ENSMUSG00000032353	Tmed3
ENSMUSG00000032374	Plod2
ENSMUSG00000032413	Rasa2

ENSMUSG00000032417	Rwdd2a
ENSMUSG00000032458	Copb2
ENSMUSG00000032469	Dbr1
ENSMUSG00000032475	Nck1
ENSMUSG00000032497	Lrrfip2
ENSMUSG00000032502	Stac
ENSMUSG00000032504	Pdcd6ip
ENSMUSG00000032507	Fbxl2
ENSMUSG00000032511	Scn5a
ENSMUSG00000032518	Rpsa
ENSMUSG00000032526	Ss18l2
ENSMUSG00000032553	Srprb
ENSMUSG00000032554	Trf
ENSMUSG00000032560	Dnajc13
ENSMUSG00000032563	Mrpl3
ENSMUSG00000032578	Cish
ENSMUSG00000032582	Rbm6
ENSMUSG00000032586	Traip
ENSMUSG00000032599	Ip6k2
ENSMUSG00000032602	Slc25a20
ENSMUSG00000032607	Amt
ENSMUSG00000032615	Nt5m
ENSMUSG00000032633	Flcn
ENSMUSG00000032649	Colgalt2
ENSMUSG00000032666	1700025G04Rik
ENSMUSG00000032702	Kank1
ENSMUSG00000032724	Abtb2
ENSMUSG00000032733	Snx33
ENSMUSG00000032744	Heyl
ENSMUSG00000032777	Gtf3c1
ENSMUSG00000032812	Arap1
ENSMUSG00000032816	Igdcc4
ENSMUSG00000032840	2410131K14Rik
ENSMUSG00000032850	Rnft2
ENSMUSG00000032878	Ccdc85a
ENSMUSG00000032890	Rims3
ENSMUSG00000032905	Atg12
ENSMUSG00000032932	Hspa13
ENSMUSG00000032952	Ap4b1
ENSMUSG00000032959	Pebp1
ENSMUSG00000032977	Fam207a
ENSMUSG00000032998	Foxj3
ENSMUSG00000033020	Polr2f
ENSMUSG00000033022	Cdo1
ENSMUSG00000033047	Eif3l
ENSMUSG00000033061	Resp18
ENSMUSG00000033096	Apmap
ENSMUSG00000033159	Cnppd1
ENSMUSG00000033166	Dis3
ENSMUSG00000033191	Tie1
ENSMUSG00000033214	Slitrk5
ENSMUSG00000033216	Eefsec
ENSMUSG00000033282	Rpgrip1l

ENSMUSG00000033285	Wdr3
ENSMUSG00000033323	Ctdp1
ENSMUSG00000033326	Kdm4a
ENSMUSG00000033377	Palmd
ENSMUSG00000033379	Atp6v0b
ENSMUSG00000033392	Clasp2
ENSMUSG00000033396	Spg11
ENSMUSG00000033400	Agl
ENSMUSG00000033420	Antxr1
ENSMUSG00000033430	Terf2ip
ENSMUSG00000033434	Gtpbp6
ENSMUSG00000033453	Adamts15
ENSMUSG00000033460	Armcx1
ENSMUSG00000033467	Crlf2
ENSMUSG00000033478	Fam160b1
ENSMUSG00000033487	Fndc3a
ENSMUSG00000033510	Otud7a
ENSMUSG00000033554	Dph5
ENSMUSG00000033557	Fam20b
ENSMUSG00000033578	Tmem35a
ENSMUSG00000033579	Fa2h
ENSMUSG00000033585	Ndn
ENSMUSG00000033595	Lgi3
ENSMUSG00000033610	Pank1
ENSMUSG00000033615	Cplx1
ENSMUSG00000033629	Hacd3
ENSMUSG00000033684	Qsox1
ENSMUSG00000033685	Ucp2
ENSMUSG00000033697	Arhgap39
ENSMUSG00000033707	Lrrc24
ENSMUSG00000033717	Adra2a
ENSMUSG00000033781	Asb13
ENSMUSG00000033792	Atp7a
ENSMUSG00000033808	Tmem87a
ENSMUSG00000033819	Ppp1r16a
ENSMUSG00000033849	B3galt2
ENSMUSG00000033880	Lgals3bp
ENSMUSG00000033916	Chmp2a
ENSMUSG00000033933	Vhl
ENSMUSG00000033938	Ndufb7
ENSMUSG00000033943	Mga
ENSMUSG00000033955	Tnks1bp1
ENSMUSG00000033964	Zbtb41
ENSMUSG00000033972	Zfp944
ENSMUSG00000033998	Kcnk1
ENSMUSG00000034000	Neu4
ENSMUSG00000034006	Pqlc1
ENSMUSG00000034021	Pds5b
ENSMUSG00000034024	Cct2
ENSMUSG00000034037	Fgd5
ENSMUSG00000034055	Phka1
ENSMUSG00000034083	Ccdc174
ENSMUSG00000034098	Fstl5

ENSMUSG00000034120	Srsf2
ENSMUSG00000034145	Tmem63c
ENSMUSG00000034171	Faah
ENSMUSG00000034173	Zbed5
ENSMUSG00000034175	Rhbdd3
ENSMUSG00000034192	Lsm3
ENSMUSG00000034203	Chchd4
ENSMUSG00000034218	Atm
ENSMUSG00000034235	Usp54
ENSMUSG00000034245	Hdac11
ENSMUSG00000034254	Agpat1
ENSMUSG00000034263	Ints14
ENSMUSG00000034271	Jdp2
ENSMUSG00000034274	Thoc5
ENSMUSG00000034278	Dnajc17
ENSMUSG00000034282	Evpl
ENSMUSG00000034295	Fhod3
ENSMUSG00000034308	Sdr42e1
ENSMUSG00000034310	Tmem132d
ENSMUSG00000034324	Tmem132c
ENSMUSG00000034343	Ube2f
ENSMUSG00000034345	Gtf2h5
ENSMUSG00000034354	Mtmr3
ENSMUSG00000034371	Tkfc
ENSMUSG00000034377	Tulp4
ENSMUSG00000034424	Gcsh
ENSMUSG00000034429	Zfp707
ENSMUSG00000034445	Cyb561a3
ENSMUSG00000034473	Sec22a
ENSMUSG00000034488	Edil3
ENSMUSG00000034532	Fbxo16
ENSMUSG00000034543	Morc2a
ENSMUSG00000034551	Hdx
ENSMUSG00000034563	Ccpg1
ENSMUSG00000034586	Hid1
ENSMUSG00000034617	Mtrr
ENSMUSG00000034648	Lrrn1
ENSMUSG00000034653	Ythdc2
ENSMUSG00000034673	Pbx2
ENSMUSG00000034744	Nagk
ENSMUSG00000034748	Sirt6
ENSMUSG00000034751	Mast4
ENSMUSG00000034793	G6pc3
ENSMUSG00000034800	Zfp661
ENSMUSG00000034825	Nrip3
ENSMUSG00000034826	Nup54
ENSMUSG00000034848	Ttc21b
ENSMUSG00000034910	Pygo1
ENSMUSG00000034926	Dhcr24
ENSMUSG00000034930	Rtkn
ENSMUSG00000034949	Zfr2
ENSMUSG00000034958	Atcay
ENSMUSG00000034993	Vat1

ENSMUSG00000035045	Zc3h12b
ENSMUSG00000035047	Kri1
ENSMUSG00000035062	Zc4h2
ENSMUSG00000035086	Becn1
ENSMUSG00000035104	Eva1a
ENSMUSG00000035133	Arhgap5
ENSMUSG00000035164	Zc3h12c
ENSMUSG00000035173	Ccdc186
ENSMUSG00000035206	Sppl2b
ENSMUSG00000035215	Lsm7
ENSMUSG00000035227	Spcs2
ENSMUSG00000035235	Trim13
ENSMUSG00000035245	Eogt
ENSMUSG00000035285	Nat14
ENSMUSG00000035297	Cops4
ENSMUSG00000035329	Fbxo33
ENSMUSG00000035342	Lzts2
ENSMUSG00000035367	Rmi1
ENSMUSG00000035378	Shq1
ENSMUSG00000035407	Kank4
ENSMUSG00000035437	Rabgap1
ENSMUSG00000035439	Haus8
ENSMUSG00000035476	Tab3
ENSMUSG00000035495	Tstd2
ENSMUSG00000035521	Gnptg
ENSMUSG00000035547	Capn5
ENSMUSG00000035596	Mboat7
ENSMUSG00000035601	Trmt10b
ENSMUSG00000035615	Frmpd1
ENSMUSG00000035621	Midn
ENSMUSG00000035632	Cnot3
ENSMUSG00000035637	Grhpr
ENSMUSG00000035642	Aamdc
ENSMUSG00000035653	Lrfn5
ENSMUSG00000035666	Gtf3c4
ENSMUSG00000035686	Thrsp
ENSMUSG00000035713	Usp35
ENSMUSG00000035776	Cd99l2
ENSMUSG00000035781	R3hdm4
ENSMUSG00000035783	Acta2
ENSMUSG00000035805	Mlc1
ENSMUSG00000035829	Ppp1r26
ENSMUSG00000035835	Plppr3
ENSMUSG00000035845	Alg12
ENSMUSG00000035868	Zfp983
ENSMUSG00000035877	Zhx3
ENSMUSG00000035910	Dcdc2a
ENSMUSG00000035919	Bbs9
ENSMUSG00000035936	Aldh5a1
ENSMUSG00000035949	Fbxw2
ENSMUSG00000035958	Tdp2
ENSMUSG00000035960	Apex1
ENSMUSG00000036002	Fam214b

ENSMUSG00000036006	Ripor2
ENSMUSG00000036009	Mettl25
ENSMUSG00000036026	Tmem63b
ENSMUSG00000036036	Zfp57
ENSMUSG00000036067	Slc2a6
ENSMUSG00000036073	Galt
ENSMUSG00000036093	Arl5a
ENSMUSG00000036098	Myrf
ENSMUSG00000036111	Lmo1
ENSMUSG00000036114	Rpp25l
ENSMUSG00000036138	Acaa1a
ENSMUSG00000036180	Gatad2a
ENSMUSG00000036186	Fam69b
ENSMUSG00000036188	Ankmy2
ENSMUSG00000036198	Arhgap36
ENSMUSG00000036199	Ndufa13
ENSMUSG00000036242	3632451O06Rik
ENSMUSG00000036246	Gmip
ENSMUSG00000036249	Rbm43
ENSMUSG00000036257	Pnpla8
ENSMUSG00000036270	Edc4
ENSMUSG00000036281	Snapc4
ENSMUSG00000036299	BC031181
ENSMUSG00000036306	Lzts1
ENSMUSG00000036309	Skp1a
ENSMUSG00000036339	Tmem260
ENSMUSG00000036357	Gpr101
ENSMUSG00000036376	Abt1
ENSMUSG00000036377	C530008M17Rik
ENSMUSG00000036398	Ppp1r11
ENSMUSG00000036427	Gpi1
ENSMUSG00000036442	Thap11
ENSMUSG00000036459	Wtip
ENSMUSG00000036469	March1
ENSMUSG00000036478	Btg1
ENSMUSG00000036510	Cdh8
ENSMUSG00000036528	Ppfibp2
ENSMUSG00000036534	Slc38a7
ENSMUSG00000036537	Rnf113a1
ENSMUSG00000036561	Ppp6r2
ENSMUSG00000036578	Fxyd7
ENSMUSG00000036613	Eipr1
ENSMUSG00000036620	Mgat4b
ENSMUSG00000036622	Atp13a2
ENSMUSG00000036632	Alg5
ENSMUSG00000036676	Tmtc3
ENSMUSG00000036699	Zcchc12
ENSMUSG00000036733	Rbm42
ENSMUSG00000036751	Cox6b1
ENSMUSG00000036764	Dnajc12
ENSMUSG00000036800	Fam135b
ENSMUSG00000036815	Dpp10
ENSMUSG00000036819	Jmjd4

ENSMUSG00000036820	Amdhd2
ENSMUSG00000036850	Mrpl41
ENSMUSG00000036879	Phkb
ENSMUSG00000036941	Elac1
ENSMUSG00000036943	Rab8b
ENSMUSG00000036949	Slc39a12
ENSMUSG00000036975	Tmem177
ENSMUSG00000036980	Taf6
ENSMUSG00000037014	Sstr4
ENSMUSG00000037031	Tspan15
ENSMUSG00000037032	Apbb1
ENSMUSG00000037049	Smpd1
ENSMUSG00000037072	Selenof
ENSMUSG00000037119	Fam91a1
ENSMUSG00000037190	Cyb561d2
ENSMUSG00000037197	Rbm17
ENSMUSG00000037214	Thap1
ENSMUSG00000037234	Hook3
ENSMUSG00000037235	Mxd4
ENSMUSG00000037236	Matr3
ENSMUSG00000037257	Aagab
ENSMUSG00000037259	Dzank1
ENSMUSG00000037260	Hgsnat
ENSMUSG00000037262	Kin
ENSMUSG00000037278	Tmem97
ENSMUSG00000037280	Galnt6
ENSMUSG00000037286	Stag1
ENSMUSG00000037287	Tbcel
ENSMUSG00000037296	Lsm1
ENSMUSG00000037313	Tacc3
ENSMUSG00000037353	Letmd1
ENSMUSG00000037370	Enpp1
ENSMUSG00000037373	Ctbp1
ENSMUSG00000037408	Cnnm4
ENSMUSG00000037419	Endod1
ENSMUSG00000037475	Thoc2
ENSMUSG00000037492	Zmat4
ENSMUSG00000037493	Cib2
ENSMUSG00000037523	Mavs
ENSMUSG00000037525	Bcdin3d
ENSMUSG00000037526	Atg14
ENSMUSG00000037573	Tob1
ENSMUSG00000037601	Nme1
ENSMUSG00000037625	Cldn11
ENSMUSG00000037685	Atp8a1
ENSMUSG00000037706	Cd81
ENSMUSG00000037710	Cisd1
ENSMUSG00000037712	Fermt2
ENSMUSG00000037730	Mynn
ENSMUSG00000037735	2810032G03Rik
ENSMUSG00000037736	Limch1
ENSMUSG00000037740	Mrps26
ENSMUSG00000037752	Xkr8

ENSMUSG00000037773	Pced1a
ENSMUSG00000037787	Apopt1
ENSMUSG00000037805	Rpl10a
ENSMUSG00000037816	Fbxw17
ENSMUSG00000037822	Smim14
ENSMUSG00000037857	Nufip2
ENSMUSG00000037894	H2afz
ENSMUSG00000037904	Ankrd9
ENSMUSG00000037905	Bri3bp
ENSMUSG00000037916	Ndufv1
ENSMUSG00000037926	Ssh2
ENSMUSG00000037938	Chchd5
ENSMUSG00000037966	Ninj1
ENSMUSG00000037971	1110032A03Rik
ENSMUSG00000037977	6430571L13Rik
ENSMUSG00000037997	Parp11
ENSMUSG00000038023	Atp6v0a2
ENSMUSG00000038028	Tigar
ENSMUSG00000038039	Gcc2
ENSMUSG00000038042	Ptpdc1
ENSMUSG00000038058	Nod1
ENSMUSG00000038072	Galnt11
ENSMUSG00000038077	Kcna6
ENSMUSG00000038079	Tmem237
ENSMUSG00000038080	Kdm1b
ENSMUSG00000038112	AW551984
ENSMUSG00000038127	Ccdc50
ENSMUSG00000038146	Notch3
ENSMUSG00000038174	Fam126b
ENSMUSG00000038187	Btbd10
ENSMUSG00000038215	Cep44
ENSMUSG00000038240	Pdss2
ENSMUSG00000038252	Ncapd2
ENSMUSG00000038271	Iffo1
ENSMUSG00000038274	Fau
ENSMUSG00000038279	Nop2
ENSMUSG00000038290	Smg6
ENSMUSG00000038291	Snx25
ENSMUSG00000038305	Spats2l
ENSMUSG00000038342	Mlxip
ENSMUSG00000038344	Txlng
ENSMUSG00000038351	Sgsm2
ENSMUSG00000038354	Ankrd35
ENSMUSG00000038365	Fbxo25
ENSMUSG00000038412	Higd1a
ENSMUSG00000038422	Hdhd3
ENSMUSG00000038429	Usp5
ENSMUSG00000038482	Tfdp1
ENSMUSG00000038486	Sv2a
ENSMUSG00000038495	Otud7b
ENSMUSG00000038502	Ptov1
ENSMUSG00000038503	Mesd
ENSMUSG00000038515	Grtp1

ENSMUSG00000038520	Tbc1d17
ENSMUSG00000038524	Fchsd1
ENSMUSG00000038533	Cbfa2t2
ENSMUSG00000038545	Cul7
ENSMUSG00000038607	Gng10
ENSMUSG00000038608	Dock10
ENSMUSG00000038628	Polr3k
ENSMUSG00000038630	Zkscan16
ENSMUSG00000038637	Lrrc56
ENSMUSG00000038650	Rnh1
ENSMUSG00000038717	Atp5l
ENSMUSG00000038742	Angptl6
ENSMUSG00000038805	Six3
ENSMUSG00000038812	Trmt112
ENSMUSG00000038816	Ctnnal1
ENSMUSG00000038836	Agbl3
ENSMUSG00000038861	Pi4kb
ENSMUSG00000038876	Rnf146
ENSMUSG00000038900	Rpl12
ENSMUSG00000038914	Dido1
ENSMUSG00000038954	Supt3
ENSMUSG00000038957	Edc3
ENSMUSG00000038965	Ube2l3
ENSMUSG00000039007	Cpq
ENSMUSG00000039016	Timm8b
ENSMUSG00000039018	Mtg1
ENSMUSG00000039033	Tasp1
ENSMUSG00000039041	Adrm1
ENSMUSG00000039065	Fam173b
ENSMUSG00000039105	Atp6v1g1
ENSMUSG00000039137	Whrn
ENSMUSG00000039154	Shd
ENSMUSG00000039164	Naif1
ENSMUSG00000039167	Adgrl4
ENSMUSG00000039182	AW209491
ENSMUSG00000039195	1110008P14Rik
ENSMUSG00000039210	Gpatch2
ENSMUSG00000039221	Rpl22l1
ENSMUSG00000039230	Tbcd
ENSMUSG00000039231	Suv39h1
ENSMUSG00000039233	Tbce
ENSMUSG00000039257	Vstm2b
ENSMUSG00000039294	BC017643
ENSMUSG00000039313	AF529169
ENSMUSG00000039316	Rftn1
ENSMUSG00000039345	Mettl22
ENSMUSG00000039347	Atp6v0e2
ENSMUSG00000039349	C130074G19Rik
ENSMUSG00000039356	Exosc2
ENSMUSG00000039428	Tmem135
ENSMUSG00000039452	Snx22
ENSMUSG00000039456	Morc3
ENSMUSG00000039483	Asb6

ENSMUSG00000039485	Tspyl4
ENSMUSG00000039515	Ptpa
ENSMUSG00000039539	Sgcz
ENSMUSG00000039542	Ncam1
ENSMUSG00000039556	Ppp1r3f
ENSMUSG00000039578	Ccser1
ENSMUSG00000039579	Grin3a
ENSMUSG00000039634	Zfp189
ENSMUSG00000039648	Kyat1
ENSMUSG00000039652	Cpeb3
ENSMUSG00000039661	Dusp26
ENSMUSG00000039662	Icmt
ENSMUSG00000039693	Msantd3
ENSMUSG00000039703	Nploc4
ENSMUSG00000039706	Ldb2
ENSMUSG00000039717	Ralyl
ENSMUSG00000039740	Alg2
ENSMUSG00000039759	Thap3
ENSMUSG00000039771	Polr2j
ENSMUSG00000039795	Zfand1
ENSMUSG00000039828	Wdr70
ENSMUSG00000039850	Endov
ENSMUSG00000039887	Alg14
ENSMUSG00000039904	Gpr37
ENSMUSG00000039910	Cited2
ENSMUSG00000039929	Urb1
ENSMUSG00000039943	Plcb4
ENSMUSG00000039953	Clstn1
ENSMUSG00000039960	Rhou
ENSMUSG00000039967	Zfp292
ENSMUSG00000039983	Ccdc32
ENSMUSG00000039987	Phtf2
ENSMUSG00000040003	Magi2
ENSMUSG00000040018	Cox15
ENSMUSG00000040035	Disp2
ENSMUSG00000040037	Negr1
ENSMUSG00000040112	Mrps35
ENSMUSG00000040242	Fgfr1op2
ENSMUSG00000040254	Sema3d
ENSMUSG00000040270	Bach2
ENSMUSG00000040274	Cdk6
ENSMUSG00000040302	Rbm48
ENSMUSG00000040321	Zfp770
ENSMUSG00000040331	Nsmce4a
ENSMUSG00000040351	Ankib1
ENSMUSG00000040387	Klhl32
ENSMUSG00000040447	Spns2
ENSMUSG00000040455	Usp45
ENSMUSG00000040462	Os9
ENSMUSG00000040463	Mybbp1a
ENSMUSG00000040479	Dgkz
ENSMUSG00000040488	Ltbp4
ENSMUSG00000040532	Abhd11

ENSMUSG00000040586	Ofd1
ENSMUSG00000040591	1110051M20Rik
ENSMUSG00000040596	Pogk
ENSMUSG00000040599	Mis12
ENSMUSG00000040616	Tmem51
ENSMUSG00000040618	Pck2
ENSMUSG00000040649	Rimklb
ENSMUSG00000040651	Fam208a
ENSMUSG00000040652	Oaz2
ENSMUSG00000040667	Nup88
ENSMUSG00000040675	Mthfd1l
ENSMUSG00000040697	Dnajc16
ENSMUSG00000040740	Slc25a34
ENSMUSG00000040759	Cmtm5
ENSMUSG00000040822	1700123O20Rik
ENSMUSG00000040824	Snrpd2
ENSMUSG00000040843	Tiprl
ENSMUSG00000040850	Psme4
ENSMUSG00000040856	Dlk1
ENSMUSG00000040859	Bsdc1
ENSMUSG00000040888	Gfer
ENSMUSG00000040913	Fbxw4
ENSMUSG00000040940	Arhgef1
ENSMUSG00000040964	Arhgef10l
ENSMUSG00000040997	Abhd4
ENSMUSG00000041014	Nrg3
ENSMUSG00000041096	Tspyl2
ENSMUSG00000041124	Msantd4
ENSMUSG00000041141	Pnmal1
ENSMUSG00000041144	Dnah7b
ENSMUSG00000041180	Hectd2
ENSMUSG00000041231	Ublcp1
ENSMUSG00000041258	Zfp236
ENSMUSG00000041264	Uspl1
ENSMUSG00000041380	Htr2c
ENSMUSG00000041544	Disp3
ENSMUSG00000041629	Fam104a
ENSMUSG00000041658	Rragb
ENSMUSG00000041679	Lrrc29
ENSMUSG00000041710	Trpc5
ENSMUSG00000041733	Coq5
ENSMUSG00000041740	Rnf10
ENSMUSG00000041763	Tpp2
ENSMUSG00000041777	Cir1
ENSMUSG00000041798	Gck
ENSMUSG00000041801	Phlda3
ENSMUSG00000041870	Ankrd13a
ENSMUSG00000041881	Ndufa7
ENSMUSG00000041907	Gpr45
ENSMUSG00000041911	Dlx1
ENSMUSG00000041935	AW549877
ENSMUSG00000041977	Arhgef11
ENSMUSG00000041992	Rapgef5

ENSMUSG00000042015	Wdr41
ENSMUSG00000042078	Svop
ENSMUSG00000042099	Kank3
ENSMUSG00000042105	Inpp5f
ENSMUSG00000042115	Klhdc8a
ENSMUSG00000042116	Vwa1
ENSMUSG00000042156	Dzip1
ENSMUSG00000042167	Papd4
ENSMUSG00000042208	0610010F05Rik
ENSMUSG00000042211	Fbxo38
ENSMUSG00000042229	Rabif
ENSMUSG00000042249	Grk3
ENSMUSG00000042271	Nxt2
ENSMUSG00000042275	Pelo
ENSMUSG00000042284	Itga1
ENSMUSG00000042286	Stab1
ENSMUSG00000042293	Gm5617
ENSMUSG00000042305	Tmem183a
ENSMUSG00000042331	Specc1
ENSMUSG00000042359	Osbpl6
ENSMUSG00000042363	Lgalsl
ENSMUSG00000042380	Smim12
ENSMUSG00000042404	Dennd4b
ENSMUSG00000042406	Atf4
ENSMUSG00000042425	Frmpd3
ENSMUSG00000042446	Zmym4
ENSMUSG00000042472	Zfp410
ENSMUSG00000042473	Tbc1d8b
ENSMUSG00000042492	Tbc1d10b
ENSMUSG00000042515	Mum1l1
ENSMUSG00000042541	Sem1
ENSMUSG00000042557	Sin3a
ENSMUSG00000042558	Adprhl2
ENSMUSG00000042606	Hirip3
ENSMUSG00000042607	Asb4
ENSMUSG00000042628	Zfyve1
ENSMUSG00000042662	Dusp15
ENSMUSG00000042712	Tceal9
ENSMUSG00000042737	Dpm3
ENSMUSG00000042761	Mrap2
ENSMUSG00000042766	Trim46
ENSMUSG00000042770	Hebp1
ENSMUSG00000042787	Exog
ENSMUSG00000042790	Rnf214
ENSMUSG00000042804	Gpr153
ENSMUSG00000042903	Foxo4
ENSMUSG00000042942	Greb1l
ENSMUSG00000042997	Nhlrc3
ENSMUSG00000043004	Gng2
ENSMUSG00000043019	Edem3
ENSMUSG00000043061	Tmem18
ENSMUSG00000043154	Ppp2r3a
ENSMUSG00000043190	Rfesd

ENSMUSG00000043259	Fam13c
ENSMUSG00000043284	Tmem11
ENSMUSG00000043300	B3galnt1
ENSMUSG00000043311	D17H6S53E
ENSMUSG00000043384	Gprasp1
ENSMUSG00000043388	Tmem130
ENSMUSG00000043419	Chd3os
ENSMUSG00000043448	Gjc2
ENSMUSG00000043456	Zfp536
ENSMUSG00000043496	Tril
ENSMUSG00000043587	Pxylp1
ENSMUSG00000043614	Vps37d
ENSMUSG00000043629	1700019D03Rik
ENSMUSG00000043648	Pld6
ENSMUSG00000043702	Pde12
ENSMUSG00000043716	Rpl7
ENSMUSG00000043801	Oaz1-ps
ENSMUSG00000043909	Trp53bp1
ENSMUSG00000043987	Cep164
ENSMUSG00000043991	Pura
ENSMUSG00000043998	Mgat2
ENSMUSG00000043999	Gpr75
ENSMUSG00000044024	Rell2
ENSMUSG00000044033	Ccdc141
ENSMUSG00000044067	Gpr22
ENSMUSG00000044071	Fam19a2
ENSMUSG00000044072	Eml6
ENSMUSG00000044117	2900011O08Rik
ENSMUSG00000044231	Nhlrc1
ENSMUSG00000044250	Pced1b
ENSMUSG00000044285	Gm1821
ENSMUSG00000044349	Snhg11
ENSMUSG00000044365	Cxxc4
ENSMUSG00000044367	Slc16a13
ENSMUSG00000044433	Camsap3
ENSMUSG00000044471	Lncpint
ENSMUSG00000044528	Tram1l1
ENSMUSG00000044550	Tceal3
ENSMUSG00000044627	Swi5
ENSMUSG00000044647	Csrnp3
ENSMUSG00000044712	Slc38a6
ENSMUSG00000044716	Dok7
ENSMUSG00000044768	D1Ertd622e
ENSMUSG00000044792	Isca1
ENSMUSG00000044847	Lsm11
ENSMUSG00000044857	Lemd2
ENSMUSG00000044894	Uqcrq
ENSMUSG00000044933	Sstr3
ENSMUSG00000044934	Zfp367
ENSMUSG00000044952	Kctd21
ENSMUSG00000044968	Napepld
ENSMUSG00000044986	Tst
ENSMUSG00000044991	1110034G24Rik

ENSMUSG00000045034	Ankrd34b
ENSMUSG00000045078	Rnf216
ENSMUSG00000045100	Slc25a26
ENSMUSG00000045103	Dmd
ENSMUSG00000045128	Rpl18a
ENSMUSG00000045179	Sox3
ENSMUSG00000045211	Nudt18
ENSMUSG00000045215	Asxl3
ENSMUSG00000045237	1110012L19Rik
ENSMUSG00000045333	Zfp423
ENSMUSG00000045414	1190002N15Rik
ENSMUSG00000045435	Tmem60
ENSMUSG00000045466	Zfp956
ENSMUSG00000045532	C1ql1
ENSMUSG00000045538	Ddx28
ENSMUSG00000045594	Glb1
ENSMUSG00000045598	Zfp553
ENSMUSG00000045639	Zfp629
ENSMUSG00000045664	Cdc42ep2
ENSMUSG00000045665	Mfsd5
ENSMUSG00000045777	Ifitm10
ENSMUSG00000045822	Zswim3
ENSMUSG00000045917	Tmem268
ENSMUSG00000045948	Mrps12
ENSMUSG00000045954	Cavin2
ENSMUSG00000045969	Ing1
ENSMUSG00000045980	Tmem104
ENSMUSG00000045991	Onecut2
ENSMUSG00000045994	B3gat1
ENSMUSG00000046010	Zfp830
ENSMUSG00000046020	Pofut1
ENSMUSG00000046152	Fut10
ENSMUSG00000046160	Olig1
ENSMUSG00000046178	Nxph1
ENSMUSG00000046185	Zfp84
ENSMUSG00000046312	AI464131
ENSMUSG00000046378	Asphd1
ENSMUSG00000046402	Rbp1
ENSMUSG00000046434	Hnrnpa1
ENSMUSG00000046442	Ppm1e
ENSMUSG00000046519	Golph3l
ENSMUSG00000046532	Ar
ENSMUSG00000046567	4930430F08Rik
ENSMUSG00000046593	Tmem215
ENSMUSG00000046605	B3gntl1
ENSMUSG00000046709	Mapk10
ENSMUSG00000046722	Cdc42se1
ENSMUSG00000046727	Cystm1
ENSMUSG00000046756	Mrps7
ENSMUSG00000046785	Epm2aip1
ENSMUSG00000046807	Lrrc75b
ENSMUSG00000046862	Pramef8
ENSMUSG00000046876	Atxn1

ENSMUSG00000047084	Ngrn
ENSMUSG00000047141	Zfp654
ENSMUSG00000047181	Samd14
ENSMUSG00000047193	Dync2h1
ENSMUSG00000047213	Ythdf3
ENSMUSG00000047246	Hist1h2be
ENSMUSG00000047261	Gap43
ENSMUSG00000047264	Zfp358
ENSMUSG00000047284	Neurl4
ENSMUSG00000047344	Lancl3
ENSMUSG00000047368	Abhd17b
ENSMUSG00000047371	Zfp768
ENSMUSG00000047379	B4gat1
ENSMUSG00000047388	Atmin
ENSMUSG00000047423	AI837181
ENSMUSG00000047496	Rnf152
ENSMUSG00000047514	Tspyl1
ENSMUSG00000047539	Fbxo28
ENSMUSG00000047547	Cltb
ENSMUSG00000047613	A430005L14Rik
ENSMUSG00000047710	Champ1
ENSMUSG00000047777	Phf13
ENSMUSG00000047844	Bex4
ENSMUSG00000047879	Usp14
ENSMUSG00000047888	Tnrc6b
ENSMUSG00000047907	Tshz2
ENSMUSG00000047945	Marcksl1
ENSMUSG00000048001	Hes5
ENSMUSG00000048004	Tmem196
ENSMUSG00000048040	Arxes2
ENSMUSG00000048047	Zbtb33
ENSMUSG00000048100	Taf13
ENSMUSG00000048126	Col6a3
ENSMUSG00000048218	Amigo2
ENSMUSG00000048249	Crebrf
ENSMUSG00000048264	Dip2c
ENSMUSG00000048280	Zfp738
ENSMUSG00000048351	Coa7
ENSMUSG00000048376	F2r
ENSMUSG00000048379	Socs4
ENSMUSG00000048439	Nupl2
ENSMUSG00000048486	Fitm2
ENSMUSG00000048537	Phldb1
ENSMUSG00000048562	Sp8
ENSMUSG00000048581	E130311K13Rik
ENSMUSG00000048696	Mex3d
ENSMUSG00000048706	Lurap1l
ENSMUSG00000048707	Tprn
ENSMUSG00000048732	Klhl11
ENSMUSG00000048787	Dcun1d3
ENSMUSG00000048807	Slc35e4
ENSMUSG00000048833	Slc39a9
ENSMUSG00000048856	Slc25a47

ENSMUSG00000048930	Tada3
ENSMUSG00000048965	Mrgpre
ENSMUSG00000048970	C1galt1c1
ENSMUSG00000049090	Zadh2
ENSMUSG00000049091	Sephs2
ENSMUSG00000049164	Zfp518a
ENSMUSG00000049232	Tigd2
ENSMUSG00000049299	Trappc1
ENSMUSG00000049303	Syt12
ENSMUSG00000049327	Kmt5a
ENSMUSG00000049339	Retreg2
ENSMUSG00000049420	Tmem200a
ENSMUSG00000049439	Cyp20a1
ENSMUSG00000049489	Fam58b
ENSMUSG00000049517	Rps23
ENSMUSG00000049536	Tceal1
ENSMUSG00000049553	Polr1a
ENSMUSG00000049555	Tmie
ENSMUSG00000049600	Zbtb45
ENSMUSG00000049624	Slc17a5
ENSMUSG00000049643	2310022A10Rik
ENSMUSG00000049717	Lig4
ENSMUSG00000049721	Gal3st1
ENSMUSG00000049791	Fzd4
ENSMUSG00000049796	Crh
ENSMUSG00000049882	Vcpkmt
ENSMUSG00000049946	BC030500
ENSMUSG00000049957	Ccdc137
ENSMUSG00000049960	Mrps16
ENSMUSG00000049985	Ankrd55
ENSMUSG00000050017	Pitpnb
ENSMUSG00000050029	Rap2c
ENSMUSG00000050052	Tdrp
ENSMUSG00000050071	Bex1
ENSMUSG00000050164	Mchr1
ENSMUSG00000050188	Lsm10
ENSMUSG00000050192	Eif5a2
ENSMUSG00000050248	Evc2
ENSMUSG00000050272	Dscam
ENSMUSG00000050334	C130071C03Rik
ENSMUSG00000050357	Carmil2
ENSMUSG00000050373	Snx21
ENSMUSG00000050394	Armcx6
ENSMUSG00000050538	B230217C12Rik
ENSMUSG00000050565	Tor1aip2
ENSMUSG00000050587	Lrrc4c
ENSMUSG00000050627	Gpd1l
ENSMUSG00000050640	Tmem150c
ENSMUSG00000050730	Arhgap42
ENSMUSG00000050732	Vamp8
ENSMUSG00000050821	Fam131a
ENSMUSG00000050822	Slc29a4
ENSMUSG00000050830	Vwc2

ENSMUSG00000050840	Cdh20
ENSMUSG00000050846	Zfp623
ENSMUSG00000050856	Atp5k
ENSMUSG00000050890	Pdik1l
ENSMUSG00000050891	Tatdn1
ENSMUSG00000050910	Cdr2l
ENSMUSG00000050912	Tmem123
ENSMUSG00000050963	Kcns2
ENSMUSG00000050965	Prkca
ENSMUSG00000050989	Selenon
ENSMUSG00000051041	Olfml1
ENSMUSG00000051062	Fbll1
ENSMUSG00000051098	Mblac2
ENSMUSG00000051154	Commd3
ENSMUSG00000051169	Rpusd3
ENSMUSG00000051223	Bzw1
ENSMUSG00000051234	Rnf7
ENSMUSG00000051238	Swsap1
ENSMUSG00000051256	Jagn1
ENSMUSG00000051319	1500011K16Rik
ENSMUSG00000051355	Commd1
ENSMUSG00000051373	Plpp7
ENSMUSG00000051412	Vamp7
ENSMUSG00000051427	Ccdc157
ENSMUSG00000051483	Cbr1
ENSMUSG00000051499	Zfp786
ENSMUSG00000051557	Pusl1
ENSMUSG00000051579	Tceal8
ENSMUSG00000051615	Rap2a
ENSMUSG00000051705	Senp8
ENSMUSG00000051747	Ttn
ENSMUSG00000051851	Rtl8c
ENSMUSG00000052026	Slc6a7
ENSMUSG00000052031	Tagap1
ENSMUSG00000052033	Pfdn4
ENSMUSG00000052137	Rbm12b2
ENSMUSG00000052151	Plpp2
ENSMUSG00000052214	Opa3
ENSMUSG00000052296	Ppp6r1
ENSMUSG00000052301	Doc2a
ENSMUSG00000052395	Rft1
ENSMUSG00000052423	B4galt3
ENSMUSG00000052456	Asna1
ENSMUSG00000052459	Atp6v1a
ENSMUSG00000052557	Gan
ENSMUSG00000052560	Cpne8
ENSMUSG00000052566	Hook2
ENSMUSG00000052584	Serp2
ENSMUSG00000052593	Adam17
ENSMUSG00000052613	Pcdh15
ENSMUSG00000052676	Zmat1
ENSMUSG00000052726	Kcnt2
ENSMUSG00000052738	Suclg1

ENSMUSG00000052763	Zfp212
ENSMUSG00000052783	Grk4
ENSMUSG00000052928	Ctif
ENSMUSG00000052981	Ube2ql1
ENSMUSG00000052997	Uba2
ENSMUSG00000053012	Krcc1
ENSMUSG00000053062	Jam2
ENSMUSG00000053070	9230110C19Rik
ENSMUSG00000053093	Myh7
ENSMUSG00000053119	Chmp3
ENSMUSG00000053128	Rnf26
ENSMUSG00000053192	Mllt11
ENSMUSG00000053291	Rab4b
ENSMUSG00000053297	AI854703
ENSMUSG00000053329	D10Jhu81e
ENSMUSG00000053398	Phgdh
ENSMUSG00000053411	Cbx7
ENSMUSG00000053414	Hunk
ENSMUSG00000053470	Kdm3a
ENSMUSG00000053483	Usp21
ENSMUSG00000053510	Nrd1
ENSMUSG00000053641	Dennd4a
ENSMUSG00000053693	Mast1
ENSMUSG00000053769	Lysmd1
ENSMUSG00000053825	Ppfia2
ENSMUSG00000053870	Fpgt
ENSMUSG00000053898	Ech1
ENSMUSG00000053985	Zfp14
ENSMUSG00000054027	Nt5dc3
ENSMUSG00000054091	1810037I17Rik
ENSMUSG00000054252	Fgfr3
ENSMUSG00000054277	Arfgap3
ENSMUSG00000054381	Zfp747
ENSMUSG00000054408	Spcs3
ENSMUSG00000054409	Tmem74
ENSMUSG00000054452	Aes
ENSMUSG00000054469	Lclat1
ENSMUSG00000054499	Dedd2
ENSMUSG00000054640	Slc8a1
ENSMUSG00000054667	Irs4
ENSMUSG00000054836	Elp6
ENSMUSG00000055026	Gabrg3
ENSMUSG00000055041	Commd5
ENSMUSG00000055240	Zfp101
ENSMUSG00000055334	Snupn
ENSMUSG00000055371	Stam2
ENSMUSG00000055407	Map6
ENSMUSG00000055430	Nap1l5
ENSMUSG00000055480	Zfp458
ENSMUSG00000055652	Klhl25
ENSMUSG00000055653	Gpc3
ENSMUSG00000055670	Zzef1
ENSMUSG00000055681	Cope

ENSMUSG00000055720	Ubl7
ENSMUSG00000055733	Nap1l3
ENSMUSG00000055737	Ghr
ENSMUSG00000055762	Eef1d
ENSMUSG00000055817	Mta3
ENSMUSG00000055862	Izumo4
ENSMUSG00000055884	Fancm
ENSMUSG00000055943	Emc7
ENSMUSG00000055945	Prr18
ENSMUSG00000056145	AI504432
ENSMUSG00000056185	Snx32
ENSMUSG00000056260	Lrif1
ENSMUSG00000056394	Lig1
ENSMUSG00000056429	Tgoln1
ENSMUSG00000056476	Med12l
ENSMUSG00000056537	Rlim
ENSMUSG00000056553	Ptprn2
ENSMUSG00000056579	Tug1
ENSMUSG00000056608	Chd9
ENSMUSG00000056665	Them6
ENSMUSG00000056679	Gpr173
ENSMUSG00000056770	Setd3
ENSMUSG00000056820	Tsnax
ENSMUSG00000056870	Gulp1
ENSMUSG00000056941	Commd7
ENSMUSG00000056972	Magel2
ENSMUSG00000057113	Npm1
ENSMUSG00000057193	Slc44a2
ENSMUSG00000057229	Dmac2
ENSMUSG00000057236	Rbbp4
ENSMUSG00000057315	Arhgap24
ENSMUSG00000057322	Rpl38
ENSMUSG00000057375	Yipf1
ENSMUSG00000057440	Mpp7
ENSMUSG00000057541	Pus7
ENSMUSG00000057551	Zfp317
ENSMUSG00000057561	Eif1a
ENSMUSG00000057614	Gnai1
ENSMUSG00000057751	Megf6
ENSMUSG00000057788	Ddx49
ENSMUSG00000057895	Zfp105
ENSMUSG00000057982	Zfp809
ENSMUSG00000058006	Mdn1
ENSMUSG00000058076	Sdhc
ENSMUSG00000058153	Sez6l
ENSMUSG00000058230	Arhgap35
ENSMUSG00000058240	Cryzl1
ENSMUSG00000058258	Idi1
ENSMUSG00000058267	Mrps14
ENSMUSG00000058298	Mcm9
ENSMUSG00000058392	Rrp1b
ENSMUSG00000058407	Txndc9
ENSMUSG00000058488	Kl

ENSMUSG00000058503	Fam133b
ENSMUSG00000058546	Rpl23a
ENSMUSG00000058624	Gda
ENSMUSG00000058793	Cds2
ENSMUSG00000058886	Deaf1
ENSMUSG00000058997	Vwa8
ENSMUSG00000059005	Hnrnpa3
ENSMUSG00000059040	Eno1b
ENSMUSG00000059070	Rpl18
ENSMUSG00000059183	Mtfmt
ENSMUSG00000059263	Usp47
ENSMUSG00000059277	R74862
ENSMUSG00000059278	Naa38
ENSMUSG00000059291	Rpl11
ENSMUSG00000059316	Slc27a4
ENSMUSG00000059355	Wdr83os
ENSMUSG00000059361	Nrsn2
ENSMUSG00000059423	Zfp933
ENSMUSG00000059474	Mbtd1
ENSMUSG00000059534	Uqcr10
ENSMUSG00000059713	Rcan3
ENSMUSG00000059734	Ndufs8
ENSMUSG00000059743	Fdps
ENSMUSG00000059852	Kcng2
ENSMUSG00000059878	Zfp422
ENSMUSG00000059921	Unc5c
ENSMUSG00000060143	Gm10076
ENSMUSG00000060240	Cend1
ENSMUSG00000060373	Hnrnpc
ENSMUSG00000060376	Bckdha
ENSMUSG00000060380	C030014I23Rik
ENSMUSG00000060450	Rnf14
ENSMUSG00000060538	Tmem219
ENSMUSG00000060657	Marf1
ENSMUSG00000060678	Hist1h4c
ENSMUSG00000060681	Slc9a6
ENSMUSG00000060716	Plekhh1
ENSMUSG00000060938	Rpl26
ENSMUSG00000060950	Trmt61a
ENSMUSG00000060981	Hist1h4h
ENSMUSG00000060992	Copz1
ENSMUSG00000061024	Rrs1
ENSMUSG00000061111	Mcrip1
ENSMUSG00000061130	Ppm1b
ENSMUSG00000061136	Prpf40a
ENSMUSG00000061175	Fnip2
ENSMUSG00000061186	Sfmbt2
ENSMUSG00000061207	Stk19
ENSMUSG00000061286	Exosc5
ENSMUSG00000061298	Agbl4
ENSMUSG00000061360	Phf5a
ENSMUSG00000061393	Acvr2b
ENSMUSG00000061436	Hipk2

ENSMUSG00000061461	Smim20
ENSMUSG00000061482	Hist1h4d
ENSMUSG00000061524	Zic2
ENSMUSG00000061559	Wdr61
ENSMUSG00000061578	Ksr2
ENSMUSG00000061589	Dot1l
ENSMUSG00000061603	Akap6
ENSMUSG00000061607	Mdc1
ENSMUSG00000061702	Tmem91
ENSMUSG00000061758	Akr1b10
ENSMUSG00000061778	Mospd2
ENSMUSG00000061950	Ppp4r1
ENSMUSG00000061979	Rcc1l
ENSMUSG00000061981	Flot2
ENSMUSG00000061983	Rps12
ENSMUSG00000062014	Gmfb
ENSMUSG00000062054	Iah1
ENSMUSG00000062252	Lhfpl5
ENSMUSG00000062380	Tubb3
ENSMUSG00000062393	Dgkk
ENSMUSG00000062519	Zfp398
ENSMUSG00000062580	Timm17a
ENSMUSG00000062590	Armc9
ENSMUSG00000062604	Srpk2
ENSMUSG00000062691	Cebpzos
ENSMUSG00000062760	1810041L15Rik
ENSMUSG00000062762	Ei24
ENSMUSG00000062825	Actg1
ENSMUSG00000062861	Zfp28
ENSMUSG00000062866	Phactr2
ENSMUSG00000062867	Impdh2
ENSMUSG00000062963	Ufc1
ENSMUSG00000062997	Rpl35
ENSMUSG00000063018	2010204K13Rik
ENSMUSG00000063108	Zfp26
ENSMUSG00000063229	Ldha
ENSMUSG00000063235	Ptpmt1
ENSMUSG00000063273	Naa15
ENSMUSG00000063410	Stk24
ENSMUSG00000063480	Snu13
ENSMUSG00000063506	Arhgap22
ENSMUSG00000063511	Snrnp70
ENSMUSG00000063524	Eno1
ENSMUSG00000063568	Jazf1
ENSMUSG00000063687	Pcdhb5
ENSMUSG00000063787	Chchd1
ENSMUSG00000063800	Prpf38a
ENSMUSG00000063802	Hspbp1
ENSMUSG00000063804	Lin28b
ENSMUSG00000063873	Slc24a3
ENSMUSG00000064030	Pym1
ENSMUSG00000064138	Fam172a
ENSMUSG00000064284	Cdpf1

ENSMUSG00000064373	Selenop
ENSMUSG00000065251	Gm23971
ENSMUSG00000065820	Gm26316
ENSMUSG00000066042	Med18
ENSMUSG00000066113	Adamtsl1
ENSMUSG00000066129	Kndc1
ENSMUSG00000066150	Slc31a1
ENSMUSG00000066232	Ipo7
ENSMUSG00000066235	Pomgnt2
ENSMUSG00000066357	Wdr6
ENSMUSG00000066440	Zfyve26
ENSMUSG00000066441	Rdh11
ENSMUSG00000066553	Gm6969
ENSMUSG00000066687	Zbtb16
ENSMUSG00000066721	Zfp575
ENSMUSG00000066829	Zfp810
ENSMUSG00000066839	Ecsit
ENSMUSG00000066842	Hmcn1
ENSMUSG00000066892	Fbxl12
ENSMUSG00000067150	Xpo5
ENSMUSG00000067274	Rplp0
ENSMUSG00000067279	Ppp1r3c
ENSMUSG00000067787	Blcap
ENSMUSG00000067825	Pex26
ENSMUSG00000067847	Romo1
ENSMUSG00000067924	Rtl8b
ENSMUSG00000068039	Tcp1
ENSMUSG00000068099	1500009C09Rik
ENSMUSG00000068250	Amn1
ENSMUSG00000068270	Shroom4
ENSMUSG00000068299	Nat8f4
ENSMUSG00000068329	Htra2
ENSMUSG00000068394	Cep152
ENSMUSG00000068735	Trp53i11
ENSMUSG00000068739	Sars
ENSMUSG00000068749	Psma5
ENSMUSG00000068922	Msto1
ENSMUSG00000069094	Pde7a
ENSMUSG00000069135	Fgfr1op
ENSMUSG00000069206	Zfp874a
ENSMUSG00000069227	Gprin1
ENSMUSG00000069266	Hist1h4b
ENSMUSG00000069274	Hist1h4f
ENSMUSG00000069520	Tmem19
ENSMUSG00000069808	Fam57a
ENSMUSG00000069917	Hba-a2
ENSMUSG00000070002	Ell
ENSMUSG00000070047	Fat1
ENSMUSG00000070319	Eif3g
ENSMUSG00000070394	Tmem256
ENSMUSG00000070420	Zscan25
ENSMUSG00000070461	9230112E08Rik
ENSMUSG00000070697	Utp3

ENSMUSG00000070699	Sars2
ENSMUSG00000070733	Fryl
ENSMUSG00000070880	Gad1
ENSMUSG00000071037	Camkmt
ENSMUSG00000071064	Zfp827
ENSMUSG00000071072	Ptges3
ENSMUSG00000071074	Yipf3
ENSMUSG00000071176	Arhgef10
ENSMUSG00000071302	2610044O15Rik8
ENSMUSG00000071337	Tia1
ENSMUSG00000071424	Grid2
ENSMUSG00000071477	Zfp777
ENSMUSG00000071528	Usmg5
ENSMUSG00000071547	Nt5dc2
ENSMUSG00000071632	2510002D24Rik
ENSMUSG00000071648	Rom1
ENSMUSG00000071649	B3gat3
ENSMUSG00000071652	Ints5
ENSMUSG00000071654	Uqcc3
ENSMUSG00000071723	Gspt2
ENSMUSG00000071855	Ccdc112
ENSMUSG00000071862	Lrrtm2
ENSMUSG00000072582	Ptrh2
ENSMUSG00000072694	1500011B03Rik
ENSMUSG00000072893	4933439C10Rik
ENSMUSG00000072915	Gm12258
ENSMUSG00000072964	Bhlhb9
ENSMUSG00000072966	Gprasp2
ENSMUSG00000073096	Lrrc61
ENSMUSG00000073147	5031425E22Rik
ENSMUSG00000073155	1810058I24Rik
ENSMUSG00000073433	Arhgdig
ENSMUSG00000073481	March2
ENSMUSG00000073600	Prob1
ENSMUSG00000073609	D2hgdh
ENSMUSG00000073616	Cops9
ENSMUSG00000073643	Wdfy1
ENSMUSG00000073680	Tmem88b
ENSMUSG00000073684	Faap20
ENSMUSG00000073700	Klhl21
ENSMUSG00000073725	Lmbrd1
ENSMUSG00000073805	Fam196a
ENSMUSG00000074102	Rbm15b
ENSMUSG00000074129	Rpl13a
ENSMUSG00000074182	Znhit6
ENSMUSG00000074211	Sdhaf1
ENSMUSG00000074220	Zfp382
ENSMUSG00000074221	Zfp568
ENSMUSG00000074227	Spint2
ENSMUSG00000074461	Gm10699
ENSMUSG00000074575	Kcng1
ENSMUSG00000074576	Mocs3
ENSMUSG00000074578	Zfas1

ENSMUSG00000074656	Eif2s2
ENSMUSG00000074733	Zfp950
ENSMUSG00000074781	Ube2n
ENSMUSG00000074793	Hspa12b
ENSMUSG00000074794	Arrdc3
ENSMUSG00000074811	Hps6
ENSMUSG00000074892	B3galt5
ENSMUSG00000074918	Inafm2
ENSMUSG00000074925	Ptar1
ENSMUSG00000075033	Nxpe3
ENSMUSG00000075224	Lrrc55
ENSMUSG00000075296	Aldh3b2
ENSMUSG00000075316	Scn9a
ENSMUSG00000075327	Zbtb2
ENSMUSG00000075419	Dolk
ENSMUSG00000075478	Slitrk1
ENSMUSG00000075514	Gm13375
ENSMUSG00000075595	Zfp652
ENSMUSG00000075600	Zc3h3
ENSMUSG00000075702	Selenom
ENSMUSG00000076432	Ywhaq
ENSMUSG00000076437	Selenoh
ENSMUSG00000076439	Mog
ENSMUSG00000076441	Ass1
ENSMUSG00000078185	Chml
ENSMUSG00000078202	Nrarp
ENSMUSG00000078234	Klhdc7a
ENSMUSG00000078348	Sf3b5
ENSMUSG00000078532	Nkain1
ENSMUSG00000078566	Bnip3
ENSMUSG00000078572	Ndufaf8
ENSMUSG00000078619	Smarcd2
ENSMUSG00000078636	Gm7336
ENSMUSG00000078695	Cisd3
ENSMUSG00000078713	Tomm5
ENSMUSG00000078779	Zfp59
ENSMUSG00000078812	Eif5a
ENSMUSG00000078813	Leng1
ENSMUSG00000078851	Hist3h2a
ENSMUSG00000078862	Gm14326
ENSMUSG00000078923	Ube2v1
ENSMUSG00000079083	Jrkl
ENSMUSG00000079104	Prps1l3
ENSMUSG00000079111	Kdelr2
ENSMUSG00000079157	Fam155a
ENSMUSG00000079316	Rab9
ENSMUSG00000079317	Trappc2
ENSMUSG00000079334	Nat6
ENSMUSG00000079435	Rpl36a
ENSMUSG00000079478	Sssca1
ENSMUSG00000079562	Maea
ENSMUSG00000079592	C1qtnf5
ENSMUSG00000079598	Clec2l

ENSMUSG00000079605	Zbtb9
ENSMUSG00000079610	Ankrd39
ENSMUSG00000079641	Rpl39
ENSMUSG00000079677	Fdx1l
ENSMUSG00000080316	Spaca6
ENSMUSG00000080365	Gm25776
ENSMUSG00000081534	Slc48a1
ENSMUSG00000082229	Nap1l2
ENSMUSG00000083327	Vcp-rs
ENSMUSG00000083380	Gm3244
ENSMUSG00000084530	Mir1897
ENSMUSG00000084842	Pabpc1l2b-ps
ENSMUSG00000084880	Tomm6os
ENSMUSG00000084910	C630043F03Rik
ENSMUSG00000085069	Gm13111
ENSMUSG00000085208	Brip1os
ENSMUSG00000085282	Gm15663
ENSMUSG00000085334	Gm12940
ENSMUSG00000085408	C530005A16Rik
ENSMUSG00000085793	Lin52
ENSMUSG00000085925	Rtl1
ENSMUSG00000086370	Ftx
ENSMUSG00000086841	2410006H16Rik
ENSMUSG00000087075	Lbhd2
ENSMUSG00000087396	4933407K13Rik
ENSMUSG00000087403	Kantr
ENSMUSG00000087497	2810001G20Rik
ENSMUSG00000087620	5330434G04Rik
ENSMUSG00000088789	Scarna13
ENSMUSG00000089617	Scarna10
ENSMUSG00000089682	Bcl2l2
ENSMUSG00000089810	Gm16536
ENSMUSG00000089857	Zfp882
ENSMUSG00000089872	Rps6kc1
ENSMUSG00000090083	Rnf8
ENSMUSG00000090247	Bloc1s1
ENSMUSG00000090553	Snrpe
ENSMUSG00000090841	Myl6
ENSMUSG00000090935	Synj2bp
ENSMUSG00000091405	Hist2h4
ENSMUSG00000091712	Sec14l5
ENSMUSG00000091735	Gpr62
ENSMUSG00000092083	Kcnb2
ENSMUSG00000092329	Gm20388
ENSMUSG00000092746	Rn7s6
ENSMUSG00000093445	Lrch4
ENSMUSG00000093536	Smim17
ENSMUSG00000093661	Eif4e3
ENSMUSG00000093674	Rpl41
ENSMUSG00000093752	Gm20716
ENSMUSG00000094724	Rnaset2b
ENSMUSG00000094936	Rbm4
ENSMUSG00000095123	Gm21781

ENSMUSG00000095325	Zfp870
ENSMUSG00000095334	Gm21984
ENSMUSG00000095362	Gm14325
ENSMUSG00000095407	Tmem200c
ENSMUSG00000095526	Gm10243
ENSMUSG00000096145	Vkorc1
ENSMUSG00000096188	Cmtm4
ENSMUSG00000096225	Lhx8
ENSMUSG00000096370	Gm21992
ENSMUSG00000096403	Gm9825
ENSMUSG00000096458	Moap1
ENSMUSG00000096472	Cdkn2d
ENSMUSG00000096847	Tmem151b
ENSMUSG00000096929	A330023F24Rik
ENSMUSG00000097141	Gm10524
ENSMUSG00000097392	D930016D06Rik
ENSMUSG00000097428	AW047730
ENSMUSG00000097440	Gm6277
ENSMUSG00000097545	Mir124a-1hg
ENSMUSG00000097767	Miat
ENSMUSG00000097785	B230217O12Rik
ENSMUSG00000098176	Ccdc166
ENSMUSG00000099021	Rn7s1
ENSMUSG00000099583	Hist1h3d
ENSMUSG00000099689	Zfp383
ENSMUSG00000100131	Gm28439
ENSMUSG00000102070	Gm28661
ENSMUSG00000102252	Snrpn
ENSMUSG00000102465	Gm38198
ENSMUSG00000102533	Gm37226
ENSMUSG00000102627	Snurf
ENSMUSG00000103041	Gm37305
ENSMUSG00000103103	4833445I07Rik
ENSMUSG00000103472	Pcdhga7
ENSMUSG00000103509	Gm38372
ENSMUSG00000103585	Pcdhgb4
ENSMUSG00000103800	Pcdha8
ENSMUSG00000103924	Gm37895
ENSMUSG00000104132	Gm36952
ENSMUSG00000104856	Rnu3b3
ENSMUSG00000105176	Gm43668
ENSMUSG00000105895	Gm42829
ENSMUSG00000106847	Peg13
ENSMUSG00000107000	Gm43481
ENSMUSG00000107023	Gm42715
ENSMUSG00000107068	Gm42742
ENSMUSG00000107331	Gm42732
ENSMUSG00000107470	Gm3375
ENSMUSG00000107879	9330102E08Rik
ENSMUSG00000108005	Gm44435
ENSMUSG00000108228	6430584L05Rik
ENSMUSG00000108621	Gm37494
ENSMUSG00000108880	Gm44560

ENSMUSG00000109394	A230057D06Rik
ENSMUSG00000109574	B230206I08Rik
ENSMUSG00000109901	Chmp1b
ENSMUSG00000110234	Gm45799
ENSMUSG00000110841	Gpx4-ps2
ENSMUSG00000110980	Gm47204
ENSMUSG00000111375	Btbd8
ENSMUSG00000111394	AC160637.1
ENSMUSG00000111977	Gm47163
ENSMUSG00000112280	A830082N09Rik
ENSMUSG00000112343	Sfta3-ps
ENSMUSG00000113061	Gm11361
ENSMUSG00000113184	AC125351.1
ENSMUSG00000114153	Gm48692

NAc_FemaleUp_MaleDown

ENSEMBL_ID	gene_name
ENSMUSG00000020422	Tns3
ENSMUSG00000020451	Limk2
ENSMUSG00000025190	Got1
ENSMUSG00000031078	Cttn
ENSMUSG00000033419	Snap91
ENSMUSG00000044791	Setd2
ENSMUSG00000053774	Ubxn7
ENSMUSG00000038072	Galnt11
ENSMUSG00000027221	Chst1
ENSMUSG00000018395	Kif3a
ENSMUSG00000040383	Aqr
ENSMUSG00000027340	Slc23a2
ENSMUSG00000052373	Mpp3
ENSMUSG00000001445	Mrpl10
ENSMUSG00000018001	Cyth3
ENSMUSG00000021196	Pfkp
ENSMUSG00000022253	Nadk2
ENSMUSG00000022750	Klhl22
ENSMUSG00000024617	Camk2a
ENSMUSG00000032480	Dhx30
ENSMUSG00000035311	Gnptab
ENSMUSG00000035933	Cog5
ENSMUSG00000037344	Slc12a9
ENSMUSG00000045441	Gprin3
ENSMUSG00000053693	Mast1
ENSMUSG00000035247	Hectd1
ENSMUSG00000020810	Cygb
ENSMUSG00000022214	Dcaf11
ENSMUSG00000022672	Prkdc
ENSMUSG00000024044	Epb41l3
ENSMUSG00000028035	Dnajb4
ENSMUSG00000032181	Scg3
ENSMUSG00000037210	Fam193a
ENSMUSG00000053199	Arhgap20
ENSMUSG00000041624	Gucy1a2
ENSMUSG00000000881	Dlg3
ENSMUSG00000007036	Abhd16a

ENSMUSG00000009207	Lnpk
ENSMUSG00000030733	Sh2b1
ENSMUSG00000036181	Hist1h1c
ENSMUSG00000040054	Baz2a
ENSMUSG00000042078	Svop
ENSMUSG00000030806	Stx1b
ENSMUSG00000031824	6430548M08Rik
ENSMUSG00000004895	Prcc
ENSMUSG00000020598	Nrcam
ENSMUSG00000027575	Arfgap1
ENSMUSG00000027678	Ncoa3
ENSMUSG00000029227	Fip1l1
ENSMUSG00000031732	Phlpp2
ENSMUSG00000032386	Trip4
ENSMUSG00000034958	Atcay
ENSMUSG00000036282	Naa30
ENSMUSG00000036779	Papd5
ENSMUSG00000038128	Camk4
ENSMUSG00000039318	Rab3gap2
ENSMUSG00000039372	March4
ENSMUSG00000039578	Ccser1
ENSMUSG00000040725	Hnrnpul1
ENSMUSG00000044667	Plppr4
ENSMUSG00000046691	Chtf8
ENSMUSG00000052397	Ezr
ENSMUSG00000022961	Son
ENSMUSG00000020745	Pafah1b1
ENSMUSG00000037032	Apbb1
ENSMUSG00000017412	Cacnb4
ENSMUSG00000029202	Pds5a
ENSMUSG00000029592	Usp30
ENSMUSG00000030592	Ryr1
ENSMUSG00000034353	Ramp1
ENSMUSG00000039117	Taf4
ENSMUSG00000026853	Crat
ENSMUSG00000028277	Ube2j1
ENSMUSG00000008301	Phax
ENSMUSG00000023965	Fbxl17
ENSMUSG00000031812	Map1lc3b
ENSMUSG00000047284	Neurl4
ENSMUSG00000033436	Armcx2
ENSMUSG00000027298	Tyro3
ENSMUSG00000003153	Slc2a3
ENSMUSG00000008489	Elavl2
ENSMUSG00000010097	Nxf1
ENSMUSG00000015461	Atf6b
ENSMUSG00000015536	Mocs2
ENSMUSG00000017943	Gdap1l1
ENSMUSG00000018865	Sult4a1
ENSMUSG00000020178	Adora2a
ENSMUSG00000022914	Brwd1
ENSMUSG00000024283	Wac
ENSMUSG00000024787	Snx15

ENSMUSG00000026452	Syt2
ENSMUSG00000026657	Frmd4a
ENSMUSG00000027438	Napb
ENSMUSG00000028086	Fbxw7
ENSMUSG00000029472	Anapc5
ENSMUSG00000030059	Tmf1
ENSMUSG00000030956	Fam53b
ENSMUSG00000031161	Hdac6
ENSMUSG00000032042	Srpr
ENSMUSG00000032743	D430042O09Rik
ENSMUSG00000032786	Alas1
ENSMUSG00000033499	Larp4b
ENSMUSG00000034265	Zdhhc14
ENSMUSG00000034297	Med13
ENSMUSG00000034687	Fras1
ENSMUSG00000034751	Mast4
ENSMUSG00000034761	Map4k5
ENSMUSG00000035246	Pcyt1b
ENSMUSG00000035754	Wdr18
ENSMUSG00000035949	Fbxw2
ENSMUSG00000036158	Prickle1
ENSMUSG00000036887	C1qa
ENSMUSG00000038026	Kcnj9
ENSMUSG00000038332	Sesn1
ENSMUSG00000039159	Ube2h
ENSMUSG00000040118	Cacna2d1
ENSMUSG00000041241	Mul1
ENSMUSG00000041408	Wapl
ENSMUSG00000042364	Snx18
ENSMUSG00000045411	2410002F23Rik
ENSMUSG00000046442	Ppm1e
ENSMUSG00000046556	Zfp319
ENSMUSG00000046722	Cdc42se1
ENSMUSG00000047030	Spata2
ENSMUSG00000054263	Lifr
ENSMUSG00000001995	Sipa1l2
ENSMUSG00000037876	Jmjd1c
ENSMUSG00000028669	Pithd1
ENSMUSG00000053768	Chchd3
ENSMUSG00000040097	Flywch1
ENSMUSG00000032212	Sltm
ENSMUSG00000006169	Clint1
ENSMUSG00000019726	Lyst
ENSMUSG00000019971	Cep290
ENSMUSG00000020936	Nmt1
ENSMUSG00000021003	Galc
ENSMUSG00000021596	Mctp1
ENSMUSG00000021846	Peli2
ENSMUSG00000024304	Cdh2
ENSMUSG00000024347	Psd2
ENSMUSG00000024940	Ltbp3
ENSMUSG00000025855	Prkar1b
ENSMUSG00000026743	Mllt10

ENSMUSG00000028565	Nfia
ENSMUSG00000034673	Pbx2
ENSMUSG00000036104	Rab3gap1
ENSMUSG00000038291	Snx25
ENSMUSG00000038299	Wdr36
ENSMUSG00000042444	Mindy2
ENSMUSG00000045752	Tssc4
ENSMUSG00000016253	Nelfcd
ENSMUSG00000022055	Nefl
ENSMUSG00000022340	Sybu
ENSMUSG00000052298	Cdc42se2
ENSMUSG00000026185	Igfbp5
ENSMUSG00000053333	Dis3l2
ENSMUSG00000024222	Fkbp5
ENSMUSG00000038576	Susd4
ENSMUSG00000029167	Ppargc1a
ENSMUSG00000002028	Kmt2a
ENSMUSG00000020794	Ube2g1
ENSMUSG00000037852	Cpe
ENSMUSG00000056367	Actr3b
ENSMUSG00000025871	4833439L19Rik
ENSMUSG00000037266	Rsrp1
ENSMUSG00000040234	Tm7sf3
ENSMUSG00000024941	Scyl1
ENSMUSG00000054871	Tmem158
ENSMUSG00000028005	Gucy1b1
ENSMUSG00000037062	Sh3glb1
ENSMUSG00000022992	Kansl2
ENSMUSG00000032570	Atp2c1
ENSMUSG00000051451	Crebzf
ENSMUSG00000009995	Taz
ENSMUSG00000029054	Gabrd
ENSMUSG00000032174	Icam5
ENSMUSG00000033720	Sfxn5
ENSMUSG00000039252	Lgi2
ENSMUSG00000009905	Kdsr
ENSMUSG00000024286	Ccny
ENSMUSG00000003464	Pex19
ENSMUSG00000017119	Nbr1
ENSMUSG00000021144	Mta1
ENSMUSG00000034269	Setd5
ENSMUSG00000040699	Limd2
ENSMUSG00000006362	Cbfa2t3
ENSMUSG00000021892	Sh3bp5
ENSMUSG00000022489	Pde1b
ENSMUSG00000024172	St6gal2
ENSMUSG00000030772	Dkk3
ENSMUSG00000053253	Ndfip2
ENSMUSG00000025060	Slk
ENSMUSG00000050043	Tmx2
ENSMUSG00000013846	St3gal1
ENSMUSG00000054675	Tmem119
ENSMUSG00000021260	Hhipl1

ENSMUSG00000025195	Dnmbp
ENSMUSG00000019790	Stxbp5
ENSMUSG00000036529	Sbf1
ENSMUSG00000040712	Camta2
ENSMUSG00000056665	Them6
ENSMUSG00000063659	Zbtb18
ENSMUSG00000017009	Sdc4
ENSMUSG00000033350	Chst2
ENSMUSG00000005069	Pex5
ENSMUSG00000038481	Cdk19
ENSMUSG00000050530	Fam171a1
ENSMUSG00000040459	Arglu1
ENSMUSG00000044788	Fads6
ENSMUSG00000024975	Pdcd4
ENSMUSG00000020409	Slu7
ENSMUSG00000038884	A230050P20Rik
ENSMUSG00000040697	Dnajc16
ENSMUSG00000020585	Laptm4a
ENSMUSG00000009647	Mcu
ENSMUSG00000033713	Foxn3
ENSMUSG00000030084	Plxna1
ENSMUSG00000023973	Cnpy3
ENSMUSG00000018209	Stk4
ENSMUSG00000056050	Mia3
ENSMUSG00000024335	Brd2
ENSMUSG00000024736	Tmem132a
ENSMUSG00000024908	Ppp6r3
ENSMUSG00000034799	Unc13a
ENSMUSG00000039197	Adk
ENSMUSG00000023009	Nckap5l
ENSMUSG00000031214	Ophn1
ENSMUSG00000055409	Nell1
ENSMUSG00000029461	Fam168a
ENSMUSG00000021963	Sap18
ENSMUSG00000022962	Gart
ENSMUSG00000022557	Bop1
ENSMUSG00000048895	Cdk5r1
ENSMUSG00000024382	Ercc3
ENSMUSG00000045534	Kcna5
ENSMUSG00000042766	Trim46
ENSMUSG00000042810	Krba1
ENSMUSG00000009216	Fam163b
ENSMUSG00000021484	Lman2
ENSMUSG00000022054	Nefm
ENSMUSG00000032096	Arcn1
ENSMUSG00000030127	Cops7a
ENSMUSG00000001632	Brpf1
ENSMUSG00000007950	Abhd8
ENSMUSG00000022141	Nipbl
ENSMUSG00000022200	Golph3
ENSMUSG00000034731	Dgkh
ENSMUSG00000042109	Csdc2
ENSMUSG00000042202	Slc35e2

ENSMUSG00000050192	Eif5a2
ENSMUSG00000053716	Dusp7
ENSMUSG00000021895	Arhgef3
ENSMUSG00000023018	Smarcd1
ENSMUSG00000028414	Fktn
ENSMUSG00000032867	Fbxw8
ENSMUSG00000024524	Gnal
ENSMUSG00000025571	Tnrc6c
ENSMUSG00000028289	Epha7
ENSMUSG00000028757	Ddost
ENSMUSG00000037885	Stk35
ENSMUSG00000000902	Smarcb1
ENSMUSG00000022307	Oxr1
ENSMUSG00000038429	Usp5
ENSMUSG00000024533	Spire1
ENSMUSG00000030374	Strn4
ENSMUSG00000039041	Adrm1
ENSMUSG00000031393	Mecp2
ENSMUSG00000030854	Ptpn5
ENSMUSG00000040007	Bahd1
ENSMUSG00000020151	Ptprr
ENSMUSG00000022412	Mief1
ENSMUSG00000029392	Rilpl1
ENSMUSG00000031987	Egln1
ENSMUSG00000039801	2410089E03Rik
ENSMUSG00000053329	D10Jhu81e
ENSMUSG00000034040	Galnt17
ENSMUSG00000034612	Chst11
ENSMUSG00000026587	Astn1
ENSMUSG00000035632	Cnot3
ENSMUSG00000021816	Ppp3cb
ENSMUSG00000047694	Yipf6
ENSMUSG00000022044	Stmn4
ENSMUSG00000024955	Esrra
ENSMUSG00000019470	Xab2
ENSMUSG00000003410	Elavl3
ENSMUSG00000022296	Baalc
ENSMUSG00000028613	Lrp8
ENSMUSG00000034145	Tmem63c
ENSMUSG00000030102	Itpr1
ENSMUSG00000030045	Mrpl19
ENSMUSG00000032281	Acsbg1
ENSMUSG00000023868	Pde10a
ENSMUSG00000030447	Cyfip1
ENSMUSG00000038546	Ranbp9
ENSMUSG00000019810	Fuca2
ENSMUSG00000023068	Nus1
ENSMUSG00000046324	Ermp1
ENSMUSG00000028078	Dclk2
ENSMUSG00000022801	Lrch3
ENSMUSG00000021684	Pde8b
ENSMUSG00000002820	Atg4d
ENSMUSG00000056812	St8sia3

ENSMUSG00000002504	Slc9a3r2
ENSMUSG00000017288	Vps53
ENSMUSG00000026489	Coq8a
ENSMUSG00000050812	AI314180
ENSMUSG00000037503	Fam168b
ENSMUSG00000002844	Adprh
ENSMUSG00000041773	Enc1
ENSMUSG00000046032	Snx12
ENSMUSG00000027351	Spred1
ENSMUSG00000028243	Ubxn2b
ENSMUSG00000021133	Susd6
ENSMUSG00000032320	Rcn2
ENSMUSG00000020108	Ddit4
ENSMUSG00000022194	Pabpn1
ENSMUSG00000054720	Lrrc8c
ENSMUSG00000006599	Gtf2h1
ENSMUSG00000030275	Etnk1
ENSMUSG00000032485	Scap
ENSMUSG00000039478	Micu3
ENSMUSG00000030103	Bhlhe40
ENSMUSG00000051243	Islr2
ENSMUSG00000020902	Ntn1
ENSMUSG00000021068	Nin
ENSMUSG00000022974	Paxbp1
ENSMUSG00000027963	Extl2
ENSMUSG00000035206	Sppl2b
ENSMUSG00000047414	Flrt2
ENSMUSG00000031508	Ankrd10
ENSMUSG00000022548	Apod
ENSMUSG00000038115	Ano2
ENSMUSG00000020484	Xbp1
ENSMUSG00000025730	Rab40c
ENSMUSG00000037126	Psd
ENSMUSG00000047935	Sox1ot
ENSMUSG00000033256	Shf
ENSMUSG00000038582	Pptc7
ENSMUSG00000021730	Hcn1
ENSMUSG00000029385	Ccng2
ENSMUSG00000040433	Zbtb38
ENSMUSG00000052488	Cherp
ENSMUSG00000029420	Rimbp2
ENSMUSG00000038612	Mcl1
ENSMUSG00000048920	Fkrp
ENSMUSG00000007783	Cpt1c
ENSMUSG00000028785	Hpca
ENSMUSG00000046215	Rprml
ENSMUSG00000034101	Ctnnd1
ENSMUSG00000041747	Utp15
ENSMUSG00000055540	Epha6
ENSMUSG00000015605	Srf
ENSMUSG00000022100	Xpo7
ENSMUSG00000029364	Wsb2
ENSMUSG00000030530	Furin

ENSMUSG00000038290	Smg6
ENSMUSG00000029145	Eif2b4
ENSMUSG00000032624	Eml4
ENSMUSG00000027603	Ggt7
ENSMUSG00000033478	Fam160b1
ENSMUSG00000018379	Srsf1
ENSMUSG00000006476	Nsmf
ENSMUSG00000021662	Arhgef28
ENSMUSG00000029804	Herc3
ENSMUSG00000042520	Ubap2l
ENSMUSG00000028639	Ybx1
ENSMUSG00000019467	Arhgef25
ENSMUSG00000021893	Capn7
ENSMUSG00000022619	Mapk8ip2
ENSMUSG00000026790	Odf2
ENSMUSG00000028426	Rad23b
ENSMUSG00000029312	Klhl8
ENSMUSG00000030352	Tspan9
ENSMUSG00000031788	Kifc3
ENSMUSG00000033365	Ipo13
ENSMUSG00000033965	Slc16a2
ENSMUSG00000037275	Gemin5
ENSMUSG00000038602	Slc35f1
ENSMUSG00000040260	Daam2
ENSMUSG00000044216	Kcnj4
ENSMUSG00000051786	Tubgcp6
ENSMUSG00000025237	Parp6
ENSMUSG00000027519	Rab22a
ENSMUSG00000020362	Cnot6
ENSMUSG00000027500	Stmn2
ENSMUSG00000016921	Srsf6
ENSMUSG00000040123	Zmym5
ENSMUSG00000022124	Fbxl3
ENSMUSG00000024740	Ddb1
ENSMUSG00000027272	Ubr1
ENSMUSG00000035064	Eef2k
ENSMUSG00000037306	Man1c1
ENSMUSG00000022463	Srebf2
ENSMUSG00000020536	Llgl1
ENSMUSG00000020964	Sel1l
ENSMUSG00000027660	Skil
ENSMUSG00000042671	Rgs8
ENSMUSG00000042743	Sgtb
ENSMUSG00000052565	Hist1h1d
ENSMUSG00000030224	Strap
ENSMUSG00000027950	Chrnb2
ENSMUSG00000042613	Pbxip1
ENSMUSG00000047446	Arl4a
ENSMUSG00000070047	Fat1
ENSMUSG00000025931	Paqr8
ENSMUSG00000052684	Jun
ENSMUSG00000005882	Uqcc1
ENSMUSG00000045287	Rtn4rl1

ENSMUSG00000033671	Cep350
ENSMUSG00000044465	Fam160a2
ENSMUSG00000021870	Slmap
ENSMUSG00000022526	Zfp251
ENSMUSG00000026596	Abl2
ENSMUSG00000031555	Adam9
ENSMUSG00000035649	Zcchc7
ENSMUSG00000044477	Zfand3
ENSMUSG00000019261	Map1s
ENSMUSG00000021699	Pde4d
ENSMUSG00000039477	Tnrc18
ENSMUSG00000001143	Lman2l
ENSMUSG00000038170	Pde4dip
ENSMUSG00000028496	Mllt3
ENSMUSG00000020590	Snx13
ENSMUSG00000036949	Slc39a12
ENSMUSG00000020329	Polrmt
ENSMUSG00000004069	Dnaja3
ENSMUSG00000024242	Map4k3
ENSMUSG00000034574	Daam1
ENSMUSG00000048307	Ankrd46
ENSMUSG00000050908	Tvp23a
ENSMUSG00000008575	Nfib
ENSMUSG00000024019	Cmtr1
ENSMUSG00000031706	Rfx1
ENSMUSG00000033009	Ogfod1
ENSMUSG00000022443	Myh9
ENSMUSG00000022552	Sharpin
ENSMUSG00000027291	Vps39
ENSMUSG00000002900	Lamb1
ENSMUSG00000028423	Nfx1
ENSMUSG00000043557	Mdga1
ENSMUSG00000051703	Tmem198
ENSMUSG00000001211	Agpat3
ENSMUSG00000018412	Kansl1
ENSMUSG00000026930	Gpsm1
ENSMUSG00000027472	Pdrg1
ENSMUSG00000054728	Phactr1
ENSMUSG00000027777	Schip1
ENSMUSG00000020326	Ccng1
ENSMUSG00000031985	Gnpat
ENSMUSG00000027247	Arhgap1
ENSMUSG00000034432	Cops8
ENSMUSG00000022476	Polr3h
ENSMUSG00000048232	Fbxo10
ENSMUSG00000007207	Stx1a
ENSMUSG00000021549	Rasa1
ENSMUSG00000027597	Ahcy
ENSMUSG00000041444	Arhgap32
ENSMUSG00000041815	Poldip3
ENSMUSG00000034854	Mfsd12
ENSMUSG00000035007	Rundc1
ENSMUSG00000019370	Calm3

ENSMUSG00000025421	Hdhd2
ENSMUSG00000030729	Pgm2l1
ENSMUSG00000043542	Zc2hc1a
ENSMUSG00000040612	Ildr2
ENSMUSG00000044864	Ankrd50
ENSMUSG00000038467	Chmp4b
ENSMUSG00000054611	Kdm2a
ENSMUSG00000040640	Erc2
ENSMUSG00000029056	Pank4
ENSMUSG00000039156	Stim2
ENSMUSG00000021259	Cyp46a1
ENSMUSG00000020015	Cdk17
ENSMUSG00000003948	Mmd
ENSMUSG00000049119	Fam110b
ENSMUSG00000022957	Itsn1
ENSMUSG00000024772	Ehd1
ENSMUSG00000026571	Dcaf6
ENSMUSG00000027677	Ttc14
ENSMUSG00000037487	Ubr5
ENSMUSG00000040850	Psme4
ENSMUSG00000042249	Grk3
ENSMUSG00000047714	Ppp1r2
ENSMUSG00000048481	Mypop
ENSMUSG00000000134	Tfe3
ENSMUSG00000006675	P4htm
ENSMUSG00000015002	Efr3a
ENSMUSG00000015214	Mtmr1
ENSMUSG00000016534	Lamp2
ENSMUSG00000024853	Sf3b2
ENSMUSG00000028152	Tspan5
ENSMUSG00000035890	Rnf126
ENSMUSG00000043259	Fam13c
ENSMUSG00000022816	Fstl1
ENSMUSG00000026159	Agfg1
ENSMUSG00000022947	Cbr3
ENSMUSG00000025393	Atp5b
ENSMUSG00000022433	Csnk1e
ENSMUSG00000031540	Kat6a
ENSMUSG00000028645	Slc2a1
ENSMUSG00000029126	Nsg1
ENSMUSG00000042428	Mgat3
ENSMUSG00000052105	Mtcl1
ENSMUSG00000056211	R3hdm1
ENSMUSG00000035045	Zc3h12b
ENSMUSG00000000346	Dazap2
ENSMUSG00000002222	Rmnd5a
ENSMUSG00000006205	Htra1
ENSMUSG00000028060	2810403A07Rik
ENSMUSG00000043323	Fbrsl1
ENSMUSG00000048175	Asb8
ENSMUSG00000050965	Prkca
ENSMUSG00000021771	Vdac2
ENSMUSG00000029578	Wipi2

ENSMUSG00000035248	Zcchc6
ENSMUSG00000043445	Pgp
ENSMUSG00000054027	Nt5dc3
ENSMUSG00000010110	Stx5a
ENSMUSG00000032802	Srxn1
ENSMUSG00000054976	Nyap2
ENSMUSG00000053963	Stum
ENSMUSG00000031754	Nudt21
ENSMUSG00000021036	Sptlc2
ENSMUSG00000019947	Arid5b
ENSMUSG00000033569	Adgrb3
ENSMUSG00000035992	Fnip1
ENSMUSG00000022185	Acin1
ENSMUSG00000034171	Faah
ENSMUSG00000000326	Comt
ENSMUSG00000052387	Trpm3
ENSMUSG00000039206	Daglb
ENSMUSG00000006522	Itih3
ENSMUSG00000020255	D10Wsu102e
ENSMUSG00000032855	Pkd1
ENSMUSG00000034997	Htr2a
ENSMUSG00000042751	Nmnat2
ENSMUSG00000028246	Faxc
ENSMUSG00000038555	Reep2
ENSMUSG00000048304	Slitrk3
ENSMUSG00000017929	B4galt5
ENSMUSG00000020376	Rnf130
ENSMUSG00000031292	Cdkl5
ENSMUSG00000032411	Tfdp2
ENSMUSG00000032840	2410131K14Rik
ENSMUSG00000036478	Btg1
ENSMUSG00000028926	Cdk14
ENSMUSG00000029544	Cabp1
ENSMUSG00000031827	Cotl1
ENSMUSG00000032939	Nup93
ENSMUSG00000035798	Zdhhc17
ENSMUSG00000020205	Phlda1
ENSMUSG00000026163	Sphkap
ENSMUSG00000040867	Begain
ENSMUSG00000029924	Slc37a3
ENSMUSG00000030223	Ptpro
ENSMUSG00000033184	Tmed7
ENSMUSG00000042401	Crtac1
ENSMUSG00000042423	Fbrs
ENSMUSG00000039278	Pcsk1n
ENSMUSG00000053580	Tanc2
ENSMUSG00000037172	E330009J07Rik
ENSMUSG00000048429	Timm29
ENSMUSG00000003435	Supt5
ENSMUSG00000004056	Akt2
ENSMUSG00000017390	Aldoc
ENSMUSG00000020101	Vsir
ENSMUSG00000024008	Cpne5

ENSMUSG00000031169	Porcn
ENSMUSG00000041695	Kcnj2
ENSMUSG00000005672	Kit
ENSMUSG00000009293	Ube2g2
ENSMUSG00000036180	Gatad2a
ENSMUSG00000030257	Srgap3
ENSMUSG00000032883	Acsl3
ENSMUSG00000001150	Mcm3ap
ENSMUSG00000022710	Usp7
ENSMUSG00000034863	Ano8
ENSMUSG00000024054	Smchd1
ENSMUSG00000041609	Bicdl1
ENSMUSG00000021686	Ap3b1
ENSMUSG00000029467	Atp2a2
ENSMUSG00000000787	Ddx3x
ENSMUSG00000017670	Elmo2
ENSMUSG00000029861	Fam131b
ENSMUSG00000030286	Emc3
ENSMUSG00000030643	Rab30
ENSMUSG00000033510	Otud7a
ENSMUSG00000039057	Myo16
ENSMUSG00000049755	Zfp672
ENSMUSG00000057530	Ece1
ENSMUSG00000001687	Ubl3
ENSMUSG00000027605	Acss2
ENSMUSG00000029121	Crmp1
ENSMUSG00000033161	Atp1a1
ENSMUSG00000037679	Inf2
ENSMUSG00000040606	Kazn
ENSMUSG00000045318	Adra2c
ENSMUSG00000018574	Acadvl
ENSMUSG00000018076	Med13l
ENSMUSG00000021273	Fdft1
ENSMUSG00000023075	Akirin1
ENSMUSG00000024384	Iws1
ENSMUSG00000025223	Ldb1
ENSMUSG00000027782	Kpna4
ENSMUSG00000028974	Dffa
ENSMUSG00000030138	Bms1
ENSMUSG00000031748	Gnao1
ENSMUSG00000034248	Slc25a37
ENSMUSG00000039270	Megf9
ENSMUSG00000052889	Prkcb
ENSMUSG00000036450	Hif1an
ENSMUSG00000020530	Ggnbp2
ENSMUSG00000034210	Efcab14
ENSMUSG00000021288	Klc1
ENSMUSG00000022895	Ets2
ENSMUSG00000038729	Akap2
ENSMUSG00000014074	Rnf168
ENSMUSG00000017686	Rhot1
ENSMUSG00000026469	Xpr1
ENSMUSG00000039202	Abhd2

ENSMUSG00000034893	Cog3
ENSMUSG00000038412	Higd1a
ENSMUSG00000021130	Galnt16
ENSMUSG00000044857	Lemd2
ENSMUSG00000053617	Sh3pxd2a
ENSMUSG00000024782	Ak3
ENSMUSG00000031916	Cog8
ENSMUSG00000026924	Sec16a
ENSMUSG00000026473	Glul
ENSMUSG00000026932	Nacc2
ENSMUSG00000034684	Sema3f
ENSMUSG00000035168	Tanc1
ENSMUSG00000037287	Tbcel
ENSMUSG00000038013	Wipf2
ENSMUSG00000039145	Camk1d
ENSMUSG00000025034	Trim8
ENSMUSG00000022829	Stxbp5l
ENSMUSG00000027425	Kat14
ENSMUSG00000044352	Sowaha
ENSMUSG00000048814	Lonrf2
ENSMUSG00000030621	Me3
ENSMUSG00000031816	Mthfsd
ENSMUSG00000038181	Chpf2
ENSMUSG00000020848	Doc2b
ENSMUSG00000003813	Rad23a
ENSMUSG00000004929	Thop1
ENSMUSG00000017167	Cntnap1
ENSMUSG00000022211	Carmil3
ENSMUSG00000026024	Als2
ENSMUSG00000028541	B4galt2
ENSMUSG00000034931	Dhx8
ENSMUSG00000035173	Ccdc186
ENSMUSG00000015971	Actr8
ENSMUSG00000017760	Ctsa
ENSMUSG00000020899	Pfas
ENSMUSG00000036894	Rap2b
ENSMUSG00000031618	Nr3c2
ENSMUSG00000038708	Golga4
ENSMUSG00000017802	Retreg3
ENSMUSG00000021814	Anxa7
ENSMUSG00000022799	Arhgap31
ENSMUSG00000024743	Syt7
ENSMUSG00000026305	Lrrfip1
ENSMUSG00000026510	Trp53bp2
ENSMUSG00000028525	Pde4b
ENSMUSG00000031776	Arl2bp
ENSMUSG00000034042	Gpbp1l1
ENSMUSG00000048109	Rbm15
ENSMUSG00000097993	Ptprv
ENSMUSG00000014361	Mertk
ENSMUSG00000026361	Cdc73
ENSMUSG00000033732	Sf3b3
ENSMUSG00000040183	Ankrd6

ENSMUSG00000024483	Ankhd1
ENSMUSG00000039176	Polg
ENSMUSG00000025047	Pdcd11
ENSMUSG00000028868	Wasf2
ENSMUSG00000039976	Tbc1d16
ENSMUSG00000042303	Sgsm3
ENSMUSG00000002550	Uck1
ENSMUSG00000015501	Hivep2
ENSMUSG00000020704	Asic2
ENSMUSG00000025373	Rnf41
ENSMUSG00000031431	Tsc22d3
ENSMUSG00000031446	Cul4a
ENSMUSG00000008305	Tle1
ENSMUSG00000041702	Btbd7
ENSMUSG00000043962	Thrap3
ENSMUSG00000039716	Dock3
ENSMUSG00000056413	Adap1
ENSMUSG00000014592	Camta1
ENSMUSG00000023277	Twf2
ENSMUSG00000030120	Mlf2
ENSMUSG00000036989	Trim3
ENSMUSG00000024188	Luc7l
ENSMUSG00000028328	Tmod1
ENSMUSG00000049553	Polr1a
ENSMUSG00000053477	Tcf4
ENSMUSG00000017314	Mpp2
ENSMUSG00000026458	Ppfia4
ENSMUSG00000030256	Bhlhe41
ENSMUSG00000033790	Tubgcp5
ENSMUSG00000033863	Klf9
ENSMUSG00000036686	Cc2d1a
ENSMUSG00000037112	Sik2
ENSMUSG00000018501	Ncor1
ENSMUSG00000020463	Ppp4r3b
ENSMUSG00000022342	Kcnv1
ENSMUSG00000032554	Trf
ENSMUSG00000042772	Smg7
ENSMUSG00000021266	Wars
ENSMUSG00000023087	Noct
ENSMUSG00000030207	Fam234b
ENSMUSG00000038733	Wdr26
ENSMUSG00000041417	Pik3r1
ENSMUSG00000046329	Slc25a23
ENSMUSG00000000374	Trappc10
ENSMUSG00000028838	Extl1
ENSMUSG00000048874	Phf3
ENSMUSG00000053046	Brsk2
ENSMUSG00000053093	Myh7
ENSMUSG00000019818	Cd164
ENSMUSG00000029920	Smarcad1
ENSMUSG00000028849	Map7d1
ENSMUSG00000029207	Apbb2
ENSMUSG00000045174	Amer3

ENSMUSG00000021313	Ryr2
ENSMUSG00000025872	Thoc3
ENSMUSG00000035049	Rrp12
ENSMUSG00000048027	Rgmb
ENSMUSG00000020894	Vamp2
ENSMUSG00000025221	Kcnip2
ENSMUSG00000040855	Reps2
ENSMUSG00000000627	Sema4f
ENSMUSG00000031137	Fgf13
ENSMUSG00000024081	Cebpz
ENSMUSG00000037851	Iars
ENSMUSG00000052752	Traf7
ENSMUSG00000097767	Miat
ENSMUSG00000031903	Pla2g15
ENSMUSG00000027628	Aar2
ENSMUSG00000026384	Ptpn4
ENSMUSG00000029432	Nipsnap2
ENSMUSG00000020032	Nuak1
ENSMUSG00000047842	Diras2
ENSMUSG00000005802	Slc30a4
ENSMUSG00000054814	Usp46
ENSMUSG00000015757	Ppil4
ENSMUSG00000026312	Cdh7
ENSMUSG00000040560	Wdr7
ENSMUSG00000040055	Gjb6
ENSMUSG00000024251	Thada
ENSMUSG00000024826	Dpf2
ENSMUSG00000026833	Olfm1
ENSMUSG00000027708	Dcun1d1
ENSMUSG00000029608	Rph3a
ENSMUSG00000037855	Zfp365
ENSMUSG00000038456	Dennd2a
ENSMUSG00000038838	Vars2
ENSMUSG00000022462	Slc38a2
ENSMUSG00000049176	Frmpd4
ENSMUSG00000038482	Tfdp1
ENSMUSG00000045983	Eif4g1
ENSMUSG00000003352	Cacnb3
ENSMUSG00000019969	Psen1
ENSMUSG00000022265	Ank
ENSMUSG00000035478	Mbd3
ENSMUSG00000030306	Tmtc1
ENSMUSG00000003233	Dvl3
ENSMUSG00000004221	Ikbkg
ENSMUSG00000034560	Washc4
ENSMUSG00000034781	Gna11
ENSMUSG00000045038	Prkce
ENSMUSG00000050357	Carmil2
ENSMUSG00000021044	Adck1
ENSMUSG00000032252	Glce
ENSMUSG00000034994	Eef2
ENSMUSG00000042331	Specc1
ENSMUSG00000051375	Pcdh1

ENSMUSG00000012117	Dhdds
ENSMUSG00000004110	Cacna1e
ENSMUSG00000028351	Brinp1
ENSMUSG00000040430	Pitpnc1
ENSMUSG00000003500	Impdh1
ENSMUSG00000054364	Rhob
ENSMUSG00000003154	Foxj2
ENSMUSG00000044072	Eml6
ENSMUSG00000033615	Cplx1
ENSMUSG00000036306	Lzts1
ENSMUSG00000036766	Dner
ENSMUSG00000033228	Scaf11
ENSMUSG00000029651	Mtus2
ENSMUSG00000038244	Mical2
ENSMUSG00000021745	Ptprg
ENSMUSG00000032826	Ank2
ENSMUSG00000040396	Abhd13
ENSMUSG00000049076	Acap2
ENSMUSG00000028488	Sh3gl2
ENSMUSG00000041781	Cpsf2
ENSMUSG00000042978	Sbk1
ENSMUSG00000048148	Nwd1
ENSMUSG00000027422	Rrbp1
ENSMUSG00000020238	Ncln
ENSMUSG00000035877	Zhx3
ENSMUSG00000032382	Snx1
ENSMUSG00000049739	Zfp646
ENSMUSG00000032329	Hmg20a
ENSMUSG00000032462	Pik3cb
ENSMUSG00000031392	Irak1
ENSMUSG00000048897	Zfp710
ENSMUSG00000015981	Stk32c
ENSMUSG00000029787	Avl9
ENSMUSG00000034940	Synrg
ENSMUSG00000046480	Scn4b
ENSMUSG00000026192	Atic
ENSMUSG00000027035	Cers6
ENSMUSG00000032280	Tle3
ENSMUSG00000014402	Tsg101
ENSMUSG00000022095	Fam160b2
ENSMUSG00000031906	Smpd3
ENSMUSG00000034413	Neurl1b
ENSMUSG00000039671	Zmynd8
ENSMUSG00000048142	Nat8l
ENSMUSG00000049422	Chchd10
ENSMUSG00000031834	Pik3r2
ENSMUSG00000001227	Sema6b
ENSMUSG00000003346	Abhd17a
ENSMUSG00000025782	Taf3
ENSMUSG00000031996	Aplp2
ENSMUSG00000042532	Golga7b
ENSMUSG00000024576	Csnk1a1
ENSMUSG00000030376	Slc8a2

ENSMUSG00000032376	Usp3
ENSMUSG00000025212	Sfxn3
ENSMUSG00000040794	C1qtnf4
ENSMUSG00000020886	Dlg4
ENSMUSG00000021775	Nr1d2
ENSMUSG00000025607	Copg2
ENSMUSG00000040389	Wdr47
ENSMUSG00000009555	Cdk9
ENSMUSG00000017291	Taok1
ENSMUSG00000035232	Pdk3
ENSMUSG00000036452	Arhgap26
ENSMUSG00000037012	Hk1
ENSMUSG00000039474	Wfs1
ENSMUSG00000041439	Mfsd6
ENSMUSG00000005534	Insr
ENSMUSG00000055745	Rtl6
ENSMUSG00000028354	Fmn2
ENSMUSG00000050022	Amz1
ENSMUSG00000009470	Tnpo1
ENSMUSG00000035078	Mtmr9
ENSMUSG00000041712	Ubr7
ENSMUSG00000034120	Srsf2
ENSMUSG00000009681	Bcr
ENSMUSG00000013236	Ptprs
ENSMUSG00000018411	Mapt
ENSMUSG00000022052	Ppp2r2a
ENSMUSG00000025228	Actr1a
ENSMUSG00000026608	Kctd3
ENSMUSG00000030533	Unc45a
ENSMUSG00000031539	Ap3m2
ENSMUSG00000036270	Edc4
ENSMUSG00000037426	Depdc5
ENSMUSG00000038976	Ppp1r9b
ENSMUSG00000039838	Slc45a1
ENSMUSG00000045733	Sprn
ENSMUSG00000047085	Lrrc4b
ENSMUSG00000002968	Med25
ENSMUSG00000013663	Pten
ENSMUSG00000022094	Slc39a14
ENSMUSG00000030678	Maz
ENSMUSG00000022545	Ercc4
ENSMUSG00000025280	Polr3a
ENSMUSG00000019831	Wasf1
ENSMUSG00000034730	Adgrb1
ENSMUSG00000039068	Zzz3
ENSMUSG00000039809	Gabbr2
ENSMUSG00000030279	C2cd5
ENSMUSG00000007594	Hapln4
ENSMUSG00000024883	Rin1
ENSMUSG00000024589	Nedd4l
ENSMUSG00000029613	Eif2ak1
ENSMUSG00000051675	Trim32
ENSMUSG00000050132	Sarm1

ENSMUSG00000034602	Mon2
ENSMUSG00000022197	Pdzd2
ENSMUSG00000021770	Samd8
ENSMUSG00000024059	Clip4
ENSMUSG00000030870	Ubfd1
ENSMUSG00000001415	Smg5
ENSMUSG00000032437	Stt3b
ENSMUSG00000056427	Slit3
ENSMUSG00000018334	Ksr1
ENSMUSG00000033697	Arhgap39
ENSMUSG00000040563	Plppr2
ENSMUSG00000001334	Fndc5
ENSMUSG00000045639	Zfp629
ENSMUSG00000025417	Pip4k2c
ENSMUSG00000021062	Rab15
ENSMUSG00000052911	Lamb2
ENSMUSG00000001270	Ckb
ENSMUSG00000024843	Chka
ENSMUSG00000024319	Vps52
ENSMUSG00000025159	Mms19
ENSMUSG00000029602	Rasal1
ENSMUSG00000033335	Dnm2
ENSMUSG00000033955	Tnks1bp1
ENSMUSG00000038894	Irs2
ENSMUSG00000043460	Elfn2
ENSMUSG00000051855	Mest
ENSMUSG00000047658	Gal3st3
ENSMUSG00000017639	Rab11fip4
ENSMUSG00000034211	Mrps17
ENSMUSG00000051695	Pcbp1
ENSMUSG00000051934	Spats2
ENSMUSG00000006673	Qrich1
ENSMUSG00000020882	Cacnb1
ENSMUSG00000026090	2010300C02Rik
ENSMUSG00000030747	Dgat2
ENSMUSG00000034247	Plekhm1
ENSMUSG00000029408	Abcb9
ENSMUSG00000022594	Lynx1
ENSMUSG00000041889	Shisa4
ENSMUSG00000004568	Arhgef18
ENSMUSG00000022139	Mbnl2
ENSMUSG00000026213	Stk11ip
ENSMUSG00000029361	Nos1
ENSMUSG00000030216	Wbp11
ENSMUSG00000039473	Ubn1
ENSMUSG00000041303	Gtf3c3
ENSMUSG00000044433	Camsap3
ENSMUSG00000022237	Ankrd33b
ENSMUSG00000001036	Epn2
ENSMUSG00000003031	Cdkn1b
ENSMUSG00000026207	Speg
ENSMUSG00000027223	Mapk8ip1
ENSMUSG00000027296	Itpka

ENSMUSG00000027881	Prpf38b
ENSMUSG00000031065	Cdk16
ENSMUSG00000031376	Atp2b3
ENSMUSG00000034686	Prr7
ENSMUSG00000040125	Gpr26
ENSMUSG00000040455	Usp45
ENSMUSG00000040651	Fam208a
ENSMUSG00000042675	Ypel3
ENSMUSG00000059003	Grin2a
ENSMUSG00000079157	Fam155a
ENSMUSG00000021460	Auh
ENSMUSG00000022240	Ctnnd2
ENSMUSG00000026470	Stx6
ENSMUSG00000032657	Fam189b
ENSMUSG00000032435	Dync1li1
ENSMUSG00000046314	Stxbp6
ENSMUSG00000020250	Txnrd1
ENSMUSG00000020524	Gria1
ENSMUSG00000020564	Atxn7l1
ENSMUSG00000021314	Amph
ENSMUSG00000025323	Sp4
ENSMUSG00000031666	Rbl2
ENSMUSG00000031819	Emc8
ENSMUSG00000039621	Prex1
ENSMUSG00000030201	Lrp6
ENSMUSG00000037754	Ppp1r16b
ENSMUSG00000020926	Adam11
ENSMUSG00000006920	Ezh1
ENSMUSG00000025158	Rfng
ENSMUSG00000052512	Nav2
ENSMUSG00000027193	Api5
ENSMUSG00000030770	Parva
ENSMUSG00000033128	Gga1
ENSMUSG00000034472	Rasd2
ENSMUSG00000052299	Ltn1
ENSMUSG00000052302	Tbc1d30
ENSMUSG00000023800	Tiam2
ENSMUSG00000032773	Chrm1
ENSMUSG00000019039	Dalrd3
ENSMUSG00000055373	Fut9
ENSMUSG00000037526	Atg14
ENSMUSG00000001482	Def8
ENSMUSG00000027350	Chgb
ENSMUSG00000030020	Prickle2
ENSMUSG00000017400	Stac2
ENSMUSG00000019254	Ppp1r12c
ENSMUSG00000028890	Mtf1
ENSMUSG00000029501	Ankle2
ENSMUSG00000031657	Heatr3
ENSMUSG00000033595	Lgi3
ENSMUSG00000034850	Tmem127
ENSMUSG00000036057	Ptpn23
ENSMUSG00000037887	Dusp8

ENSMUSG00000055675	Kbtbd11
ENSMUSG00000014349	Ube2z
ENSMUSG00000020674	Pxdn
ENSMUSG00000021189	Atxn3
ENSMUSG00000005312	Ubqln1
ENSMUSG00000027582	Zgpat
ENSMUSG00000023495	Pcbp4
ENSMUSG00000026811	St6galnac6
ENSMUSG00000041040	Fam117b
ENSMUSG00000039678	Tbc1d13
ENSMUSG00000003863	Ppfia3
ENSMUSG00000005148	Klf5
ENSMUSG00000008730	Hipk1
ENSMUSG00000018401	Mtmr4
ENSMUSG00000021038	Vipas39
ENSMUSG00000021991	Cacna2d3
ENSMUSG00000025314	Ptprj
ENSMUSG00000030657	Xylt1
ENSMUSG00000037224	Zfyve28
ENSMUSG00000037351	Actr1b
ENSMUSG00000037902	Sirpa
ENSMUSG00000041268	Dmxl2
ENSMUSG00000047412	Zbtb44
ENSMUSG00000024370	Cdc23
ENSMUSG00000023026	Dip2b
ENSMUSG00000025745	Hadha
ENSMUSG00000031523	Dlc1
ENSMUSG00000046962	Zbtb21
ENSMUSG00000038485	Socs7
ENSMUSG00000002546	Golga2
ENSMUSG00000041977	Arhgef11
ENSMUSG00000022421	Nptxr
ENSMUSG00000055567	Unc80
ENSMUSG00000033287	Kctd17
ENSMUSG00000003458	Ncstn
ENSMUSG00000024949	Sf1
ENSMUSG00000025170	Rab40b
ENSMUSG00000035765	Dym
ENSMUSG00000027746	Ufm1
ENSMUSG00000022540	Rogdi
ENSMUSG00000034825	Nrip3
ENSMUSG00000040964	Arhgef10l
ENSMUSG00000038762	Abcf1
ENSMUSG00000051344	Plekhm3
ENSMUSG00000029765	Plxna4
ENSMUSG00000041846	Ppp4r3a
ENSMUSG00000054693	Adam10
ENSMUSG00000014550	Rbsn
ENSMUSG00000032356	Rasgrf1
ENSMUSG00000025544	Tm9sf2
ENSMUSG00000026277	Stk25
ENSMUSG00000040414	Slc25a28
ENSMUSG00000038178	Slc43a2

ENSMUSG00000040111	Gramd1b
ENSMUSG00000042185	Nfrkb
ENSMUSG00000024491	Rbm27
ENSMUSG00000040481	Bptf
ENSMUSG00000002393	Nr2f6
ENSMUSG00000025786	Zdhhc3
ENSMUSG00000035392	Dennd1a
ENSMUSG00000017740	Slc12a5
ENSMUSG00000034156	Tspoap1
ENSMUSG00000034636	Zyg11b
ENSMUSG00000037579	Kcnh3
ENSMUSG00000001802	Lrp3
ENSMUSG00000025085	Ablim1
ENSMUSG00000026202	Tuba4a
ENSMUSG00000096910	Zfp955b
ENSMUSG00000046607	Hrk
ENSMUSG00000004031	Brinp2
ENSMUSG00000033998	Kcnk1
ENSMUSG00000035011	Zbtb7a
ENSMUSG00000038473	Nos1ap
ENSMUSG00000042066	Tmcc2
ENSMUSG00000031932	Gpr83
ENSMUSG00000002010	Idh3g
ENSMUSG00000041199	Rpusd1
ENSMUSG00000029064	Gnb1
ENSMUSG00000045967	Gpr158
ENSMUSG00000022553	Maf1
ENSMUSG00000026821	Ralgds
ENSMUSG00000037341	Slc9a7
ENSMUSG00000040720	Virma
ENSMUSG00000049336	Tenm2
ENSMUSG00000042688	Mapk6
ENSMUSG00000056258	Kcnq3
ENSMUSG00000020777	Acox1
ENSMUSG00000036225	Kctd1
ENSMUSG00000021820	Camk2g
ENSMUSG00000042846	Lrrtm3
ENSMUSG00000017831	Rab5a
ENSMUSG00000018427	Ypel2
ENSMUSG00000026792	Lrsam1
ENSMUSG00000054423	Cadps
ENSMUSG00000021087	Rtn1
ENSMUSG00000018417	Myo1b
ENSMUSG00000031391	L1cam
ENSMUSG00000037098	Rab11fip3
ENSMUSG00000048799	Cep120
ENSMUSG00000056222	Spock1
ENSMUSG00000000276	Dgke
ENSMUSG00000026235	Epha4
ENSMUSG00000026466	Tor1aip1
ENSMUSG00000027273	Snap25
ENSMUSG00000028454	Pigo
ENSMUSG00000034275	Igsf9b

ENSMUSG00000038205	Prkab2
ENSMUSG00000040102	Klhl42
ENSMUSG00000040356	Skiv2l
ENSMUSG00000042155	Klhl23
ENSMUSG00000042388	Dlgap3
ENSMUSG00000048458	Fam212b
ENSMUSG00000049550	Clip1
ENSMUSG00000062353	Gm15772
ENSMUSG00000000632	Sez6
ENSMUSG00000006307	Kmt2b
ENSMUSG00000027612	Mmp24
ENSMUSG00000042350	Arel1
ENSMUSG00000087141	Plcxd2
ENSMUSG00000032336	Nptn
ENSMUSG00000016831	Tox4
ENSMUSG00000027016	Zfp385b
ENSMUSG00000029772	Ahcyl2
ENSMUSG00000030123	Plxnd1
ENSMUSG00000031971	Ccsap
ENSMUSG00000032560	Dnajc13
ENSMUSG00000050272	Dscam
ENSMUSG00000036026	Tmem63b
ENSMUSG00000037049	Smpd1
ENSMUSG00000047013	Fbxo41
ENSMUSG00000033174	Mgll
ENSMUSG00000040495	Chrm4
ENSMUSG00000036264	Fstl4
ENSMUSG00000004567	Mcoln1
ENSMUSG00000028479	Gne
ENSMUSG00000018820	Zfyve27
ENSMUSG00000036819	Jmjd4
ENSMUSG00000050751	Pgbd5
ENSMUSG00000025728	Pigq
ENSMUSG00000030986	Dhx32
ENSMUSG00000003068	Stk11
ENSMUSG00000018921	Pelp1
ENSMUSG00000021936	Mapk8
ENSMUSG00000032009	Sesn3
ENSMUSG00000037316	Bag4
ENSMUSG00000002957	Ap2a2
ENSMUSG00000026885	Ttll11
ENSMUSG00000037933	Bicd2
ENSMUSG00000025092	Hspa12a
ENSMUSG00000044066	Cep68
ENSMUSG00000009569	Mkl2
ENSMUSG00000022228	Zscan26
ENSMUSG00000027933	Ints3
ENSMUSG00000034430	Zxdc
ENSMUSG00000039697	Ncoa7
ENSMUSG00000000223	Drp2
ENSMUSG00000009291	Pttg1ip
ENSMUSG00000011096	Akt1s1
ENSMUSG00000026491	Ahctf1

ENSMUSG00000036606	Plxnb2
ENSMUSG00000025582	Nptx1
ENSMUSG00000014355	Anapc1
ENSMUSG00000022454	Nell2
ENSMUSG00000036473	Tbc1d24
ENSMUSG00000046157	Tmem229b
ENSMUSG00000030741	Spns1
ENSMUSG00000031960	Aars
ENSMUSG00000025575	Cant1
ENSMUSG00000028796	Phc2
ENSMUSG00000013419	Zfp651
ENSMUSG00000037110	Ralgapa2
ENSMUSG00000047213	Ythdf3
ENSMUSG00000027411	Vps16
ENSMUSG00000044030	Irf2bp1
ENSMUSG00000013275	Slc41a1
ENSMUSG00000022024	Sugt1
ENSMUSG00000045114	Prrt2
ENSMUSG00000021690	Jmy
ENSMUSG00000023169	Slc38a1
ENSMUSG00000041961	Znrf3
ENSMUSG00000037536	Fbxo34
ENSMUSG00000048279	Sacs
ENSMUSG00000020315	Sptbn1
ENSMUSG00000022883	Robo1
ENSMUSG00000024851	Pitpnm1
ENSMUSG00000032036	Kirrel3
ENSMUSG00000037541	Shank2
ENSMUSG00000038264	Sema7a
ENSMUSG00000038535	Zfp280d
ENSMUSG00000039354	Smarcal1
ENSMUSG00000040782	Cop1
ENSMUSG00000011306	Sugp1
ENSMUSG00000003660	Snrnp200
ENSMUSG00000045216	Hs6st1
ENSMUSG00000028701	Lurap1
ENSMUSG00000024240	Epc1
ENSMUSG00000031605	Klhl2
ENSMUSG00000051864	Tbc1d22a
ENSMUSG00000000631	Myo18a
ENSMUSG00000004798	Ulk2
ENSMUSG00000053062	Jam2
ENSMUSG00000022840	Adcy5
ENSMUSG00000039546	Ajap1
ENSMUSG00000044071	Fam19a2
ENSMUSG00000040479	Dgkz
ENSMUSG00000025139	Tollip
ENSMUSG00000027806	Tsc22d2
ENSMUSG00000035203	Epn1
ENSMUSG00000018446	C1qbp
ENSMUSG00000024897	Apba1
ENSMUSG00000028906	Epb41
ENSMUSG00000032850	Rnft2

ENSMUSG00000033530	Ttc7b
ENSMUSG00000039952	Dag1
ENSMUSG00000045007	Tubg2
ENSMUSG00000055932	Fto
ENSMUSG00000001419	Mef2d
ENSMUSG00000024603	Dctn4
ENSMUSG00000030930	Chst15
ENSMUSG00000029726	Mepce
ENSMUSG00000047888	Tnrc6b
ENSMUSG00000032295	Man2c1
ENSMUSG00000030518	Fam189a1
ENSMUSG00000006731	B4galnt1
ENSMUSG00000020496	Rnf187
ENSMUSG00000021819	Zswim8
ENSMUSG00000026496	Parp1
ENSMUSG00000035226	Rims4
ENSMUSG00000028124	Gclm
ENSMUSG00000032549	Rab6b
ENSMUSG00000031993	Snx19
ENSMUSG00000032262	Elovl4
ENSMUSG00000025020	Slit1
ENSMUSG00000034032	Rpap1
ENSMUSG00000041000	Trim62
ENSMUSG00000041570	Camsap2
ENSMUSG00000021719	Rgs7bp
ENSMUSG00000025477	Inpp5a
ENSMUSG00000027194	Ttc17
ENSMUSG00000040724	Kcna2
ENSMUSG00000041297	Cdk13
ENSMUSG00000053024	Cntn2
ENSMUSG00000048385	Scrt1
ENSMUSG00000000594	Gm2a
ENSMUSG00000021750	Fam107a
ENSMUSG00000026158	Ogfrl1
ENSMUSG00000043940	Wdfy3
ENSMUSG00000018547	Pip4k2b
ENSMUSG00000034656	Cacna1a
ENSMUSG00000038014	Fam120a
ENSMUSG00000041308	Sntb2
ENSMUSG00000052296	Ppp6r1
ENSMUSG00000034336	Ina
ENSMUSG00000038271	Iffo1
ENSMUSG00000037151	Lrrc20
ENSMUSG00000040620	Dhx33
ENSMUSG00000000552	Zfp385a
ENSMUSG00000022263	Trio
ENSMUSG00000022757	Tfg
ENSMUSG00000020658	Efr3b
ENSMUSG00000002489	Tiam1
ENSMUSG00000025780	Itih5
ENSMUSG00000038784	Cnot4
ENSMUSG00000020950	Foxg1
ENSMUSG00000032131	Abcg4

ENSMUSG00000032571	Pik3r4
ENSMUSG00000028820	Sfpq
ENSMUSG00000026663	Atf6
ENSMUSG00000005198	Polr2a
ENSMUSG00000020790	Ankfy1
ENSMUSG00000026576	Atp1b1
ENSMUSG00000031837	Necab2
ENSMUSG00000050947	Amigo1
ENSMUSG00000039686	Zer1
ENSMUSG00000025422	Agap2
ENSMUSG00000070570	Slc17a7
ENSMUSG00000035401	Emsy
ENSMUSG00000038679	Trps1
ENSMUSG00000025551	Fgf14
ENSMUSG00000029055	Plch2
ENSMUSG00000038296	Galnt18
ENSMUSG00000030105	Arl8b
ENSMUSG00000057133	Chd6
ENSMUSG00000009406	Elk1
ENSMUSG00000020537	Drg2
ENSMUSG00000021978	Extl3
ENSMUSG00000026443	Lrrn2
ENSMUSG00000027799	Nbea
ENSMUSG00000034154	Ino80
ENSMUSG00000037993	Dhx38
ENSMUSG00000040225	Prrc2c
ENSMUSG00000053166	Cdh22
ENSMUSG00000070738	Dgkd
ENSMUSG00000050511	Oprd1
ENSMUSG00000034402	Kcnh5
ENSMUSG00000013858	Tmem259
ENSMUSG00000024777	Ppp2r5b
ENSMUSG00000042599	Kdm7a
ENSMUSG00000050600	Zfp831
ENSMUSG00000048388	Fam171b
ENSMUSG00000011751	Sptbn4
ENSMUSG00000038390	Gpr162
ENSMUSG00000045009	Prrt3
ENSMUSG00000022892	App
ENSMUSG00000037907	Ankrd13b
ENSMUSG00000042207	Kdm5b
ENSMUSG00000022718	Dgcr8
ENSMUSG00000029166	Mapre3
ENSMUSG00000028782	Adgrb2
ENSMUSG00000028176	Lrrc7
ENSMUSG00000024030	Abcg1
ENSMUSG00000029434	Vps33a
ENSMUSG00000001054	Rmnd5b
ENSMUSG00000031770	Herpud1
ENSMUSG00000038172	Ttc39b
ENSMUSG00000042121	Ssh1
ENSMUSG00000042650	Alkbh5
ENSMUSG00000030180	Kdm5a

ENSMUSG00000022000	Zc3h13
ENSMUSG00000001089	Luzp1
ENSMUSG00000027198	Ext2
ENSMUSG00000028830	AU040320
ENSMUSG00000003166	Dgcr2
ENSMUSG00000022195	6030458C11Rik
ENSMUSG00000023852	Chd1
ENSMUSG00000028106	Rprd2
ENSMUSG00000029419	Gm996
ENSMUSG00000031565	Fgfr1
ENSMUSG00000033618	Map3k13
ENSMUSG00000035891	Cerk
ENSMUSG00000039738	Slx4
ENSMUSG00000042524	Sun2
ENSMUSG00000051403	Ppp1r37
ENSMUSG00000005575	Ube2m
ENSMUSG00000024892	Pcx
ENSMUSG00000026116	Tmem131
ENSMUSG00000026753	Ppp6c
ENSMUSG00000027312	Atrn
ENSMUSG00000035314	Gdpd5
ENSMUSG00000025576	Rbfox3
ENSMUSG00000027634	Ndrg3
ENSMUSG00000032393	Dpp8
ENSMUSG00000045374	Wdr81
ENSMUSG00000024862	Klc2
ENSMUSG00000020453	Patz1
ENSMUSG00000042508	Dmtf1
ENSMUSG00000038738	Shank1
ENSMUSG00000036817	Sun1
ENSMUSG00000039959	Hip1
ENSMUSG00000024065	Ehd3
ENSMUSG00000035735	Dagla
ENSMUSG00000060924	Csmd1
ENSMUSG00000002496	Tsc2
ENSMUSG00000022043	Trim35
ENSMUSG00000029120	Ppp2r2c
ENSMUSG00000034858	Fam214a
ENSMUSG00000056608	Chd9
ENSMUSG00000033684	Qsox1
ENSMUSG00000036561	Ppp6r2
ENSMUSG00000032637	Atxn2l
ENSMUSG00000038141	Tmem181a
ENSMUSG00000020671	Rab10
ENSMUSG00000021179	Nrde2
ENSMUSG00000021360	Gcnt2
ENSMUSG00000026447	Pik3c2b
ENSMUSG00000029438	Bcl7a
ENSMUSG00000040687	Madd
ENSMUSG00000052934	Fbxo31
ENSMUSG00000031823	Zdhhc7
ENSMUSG00000021051	Ppp2r5e
ENSMUSG00000024270	Slc39a6

ENSMUSG00000030731	Syt3
ENSMUSG00000015290	Ubl4a
ENSMUSG00000042686	Jph1
ENSMUSG00000029345	Tfip11
ENSMUSG00000039860	Srrm3
ENSMUSG00000041986	Elmod1
ENSMUSG00000020823	Sec14l1
ENSMUSG00000027014	Cwc22
ENSMUSG00000031955	Bcar1
ENSMUSG00000049092	Gpr137c
ENSMUSG00000001552	Jup
ENSMUSG00000000131	Xpo6
ENSMUSG00000005580	Adcy9
ENSMUSG00000036760	Kcnk9
ENSMUSG00000002129	Sf3a1
ENSMUSG00000029705	Cux1
ENSMUSG00000045078	Rnf216
ENSMUSG00000029598	Plbd2
ENSMUSG00000034908	Sidt2
ENSMUSG00000047921	Trappc9
ENSMUSG00000058975	Kcnc1
ENSMUSG00000029655	N4bp2l2
ENSMUSG00000054226	Tprkb
ENSMUSG00000001248	Gramd1a
ENSMUSG00000021823	Vcl
ENSMUSG00000028931	Kcnab2
ENSMUSG00000017376	Nlk
ENSMUSG00000006215	Zbtb17
ENSMUSG00000021279	Cdc42bpb
ENSMUSG00000025876	Unc5a
ENSMUSG00000051331	Cacna1c
ENSMUSG00000021488	Nsd1
ENSMUSG00000016349	Eef1a2
ENSMUSG00000002107	Celf2
ENSMUSG00000038533	Cbfa2t2
ENSMUSG00000039713	Plekhg5
ENSMUSG00000042724	Map3k9
ENSMUSG00000039568	Ubald1
ENSMUSG00000032046	Abhd12
ENSMUSG00000022848	Dirc2
ENSMUSG00000040009	Gnaz
ENSMUSG00000023830	Igf2r
ENSMUSG00000049577	Zfpm1
ENSMUSG00000034160	Ogt
ENSMUSG00000008153	Clstn3
ENSMUSG00000020734	Grin2c
ENSMUSG00000025189	Cnnm1
ENSMUSG00000027332	Ivd
ENSMUSG00000030655	Smg1
ENSMUSG00000043079	Synpo
ENSMUSG00000043683	Fem1a
ENSMUSG00000074102	Rbm15b
ENSMUSG00000024002	Brd4

ENSMUSG00000025558	Dock9
ENSMUSG00000027784	Ppm1l
ENSMUSG00000030554	Synm
ENSMUSG00000042046	Dstyk
ENSMUSG00000024613	Tcof1
ENSMUSG00000024807	Syvn1
ENSMUSG00000037703	Lzts3
ENSMUSG00000040390	Map3k10
ENSMUSG00000051067	Lingo3
ENSMUSG00000052727	Map1b
ENSMUSG00000028991	Mtor
ENSMUSG00000032340	Neo1
ENSMUSG00000001924	Uba1
ENSMUSG00000019194	Scn1b
ENSMUSG00000027522	Stx16
ENSMUSG00000031778	Cx3cl1
ENSMUSG00000038324	Trpc4ap
ENSMUSG00000054843	Atrnl1
ENSMUSG00000032217	Rnf111
ENSMUSG00000030894	Tpp1
ENSMUSG00000025512	Chid1
ENSMUSG00000033917	Gde1
ENSMUSG00000039770	Ypel5
ENSMUSG00000004383	Large1
ENSMUSG00000056091	St3gal5
ENSMUSG00000045636	Mtus1
ENSMUSG00000034613	Ppm1h
ENSMUSG00000047804	Akap10
ENSMUSG00000011158	Brf1
ENSMUSG00000034570	Inpp5j
ENSMUSG00000024109	Nrxn1
ENSMUSG00000058966	Fam57b
ENSMUSG00000020741	Cluh
ENSMUSG00000022416	Cacna1i
ENSMUSG00000024462	Gabbr1
ENSMUSG00000047824	Pygo2
ENSMUSG00000051335	Gfod1
ENSMUSG00000052040	Klf13
ENSMUSG00000032598	Nckipsd
ENSMUSG00000033004	Mycbp2
ENSMUSG00000008429	Herpud2
ENSMUSG00000005609	Ctr9
ENSMUSG00000042292	Mkl1
ENSMUSG00000001120	Pcbp3
ENSMUSG00000034254	Agpat1
ENSMUSG00000030209	Grin2b
ENSMUSG00000037217	Syn1
ENSMUSG00000027646	Src
ENSMUSG00000045589	Frrs1l
ENSMUSG00000020198	Ap3d1
ENSMUSG00000032261	Sh3bgrl2
ENSMUSG00000025372	Baiap2
ENSMUSG00000028519	Dab1

ENSMUSG00000026179	Pnkd
ENSMUSG00000041037	Irgq
ENSMUSG00000041729	Coro2b
ENSMUSG00000027589	Pcmtd2
ENSMUSG00000029512	Ulk1
ENSMUSG00000039367	Sec24c
ENSMUSG00000029050	Ski
ENSMUSG00000027134	Lpcat4
ENSMUSG00000048756	Foxo3
ENSMUSG00000034216	Vps18
ENSMUSG00000029406	Pitpnm2
ENSMUSG00000020827	Mink1
ENSMUSG00000026509	Capn2
ENSMUSG00000040276	Pacsin1
ENSMUSG00000053877	Srcap
ENSMUSG00000021913	Ogdhl
ENSMUSG00000039840	Epg5
ENSMUSG00000031626	Sorbs2
ENSMUSG00000021071	Trim9
ENSMUSG00000005338	Cadm3
ENSMUSG00000002346	Slc25a42
ENSMUSG00000008690	Ncaph2
ENSMUSG00000021027	Ralgapa1
ENSMUSG00000020262	Adarb1
ENSMUSG00000028096	Gpr89
ENSMUSG00000026825	Dnm1
ENSMUSG00000032238	Rora
ENSMUSG00000027457	Snph
ENSMUSG00000063952	Brpf3
ENSMUSG00000015776	Med22
ENSMUSG00000025277	Abhd6
ENSMUSG00000028556	Dock7
ENSMUSG00000029330	Cds1
ENSMUSG00000031865	Dctn1
ENSMUSG00000034187	Nsf
ENSMUSG00000034949	Zfr2
ENSMUSG00000047417	Rexo1
ENSMUSG00000050148	Ubqln2
ENSMUSG00000025907	Rb1cc1
ENSMUSG00000028809	Srrm1
ENSMUSG00000030583	Sipa1l3
ENSMUSG00000027087	Itgav
ENSMUSG00000007038	Neu1
ENSMUSG00000040907	Atp1a3
ENSMUSG00000021275	Tecpr2
ENSMUSG00000024773	Atg2a
ENSMUSG00000025427	Rnf165
ENSMUSG00000039834	Zfp335
ENSMUSG00000012609	Ttll5
ENSMUSG00000038970	Lmtk2
ENSMUSG00000022564	Grina
ENSMUSG00000022761	Lztr1
ENSMUSG00000050556	Kcnb1

ENSMUSG00000031822	Gse1
ENSMUSG00000041720	Pi4ka
ENSMUSG00000046822	Slc39a3
ENSMUSG00000022779	Top3b
ENSMUSG00000010277	2610507B11Rik
ENSMUSG00000020220	Vps13d
ENSMUSG00000024947	Men1
ENSMUSG00000026640	Plxna2
ENSMUSG00000029020	Mfn2
ENSMUSG00000034201	Gas2l1
ENSMUSG00000039804	Ncoa5
ENSMUSG00000041329	Atp1b2
ENSMUSG00000044308	Ubr3
ENSMUSG00000049728	Zfp668
ENSMUSG00000063239	Grm4
ENSMUSG00000030302	Atp2b2
ENSMUSG00000025738	Fbxl16
ENSMUSG00000038020	Rapgefl1
ENSMUSG00000036155	Mgat5
ENSMUSG00000025006	Sorbs1
ENSMUSG00000051149	Adnp
ENSMUSG00000013646	Sh3bp5l
ENSMUSG00000022426	Josd1
ENSMUSG00000019796	Lrp11
ENSMUSG00000034310	Tmem132d
ENSMUSG00000003378	Grik5
ENSMUSG00000042700	Sipa1l1
ENSMUSG00000030519	Apba2
ENSMUSG00000014426	Map3k4
ENSMUSG00000024797	Vps51
ENSMUSG00000029471	Camkk2
ENSMUSG00000033902	Mapkbp1
ENSMUSG00000040856	Dlk1
ENSMUSG00000040506	Ambra1
ENSMUSG00000039953	Clstn1
ENSMUSG00000047976	Kcna1
ENSMUSG00000036957	Lrfn3
ENSMUSG00000023977	Ubr2
ENSMUSG00000039523	Cep104
ENSMUSG00000025332	Kdm5c
ENSMUSG00000025658	Ksr2
ENSMUSG00000014786	Slc9a5
ENSMUSG00000027254	Map1a
ENSMUSG00000029449	Rhof
ENSMUSG00000053395	Cacng8
ENSMUSG00000020435	Osbp2
ENSMUSG00000023033	Scn8a
ENSMUSG00000025964	Adam23
ENSMUSG00000027347	Rasgrp1
ENSMUSG00000022587	Ly6e
ENSMUSG00000032589	Bsn
ENSMUSG00000026883	Dab2ip
ENSMUSG00000002052	Supt6

ENSMUSG00000013921	Clip3
ENSMUSG00000031833	Mast3
ENSMUSG00000025220	Mgea5
ENSMUSG00000024096	Ralbp1
ENSMUSG00000027894	Slc6a17
ENSMUSG00000053550	Shisa7
ENSMUSG00000032010	Usp2
ENSMUSG00000037791	Phf12
ENSMUSG00000026080	Chst10
ENSMUSG00000049511	Htr1b
ENSMUSG00000020400	Tnip1
ENSMUSG00000022558	Mroh1
ENSMUSG00000038861	Pi4kb
ENSMUSG00000032741	Tpcn1
ENSMUSG00000003344	Btbd2
ENSMUSG00000038967	Pdk2
ENSMUSG00000015476	Prrt1
ENSMUSG00000026858	Miga2
ENSMUSG00000009112	Bcl2l13
ENSMUSG00000029053	Prkcz
ENSMUSG00000033389	Arhgap44
ENSMUSG00000032087	Dscaml1
ENSMUSG00000047434	Xxylt1
ENSMUSG00000024104	Washc2
ENSMUSG00000024750	Zfand5
ENSMUSG00000037415	Ranbp10
ENSMUSG00000032402	Smad3
ENSMUSG00000024163	Mapk8ip3
ENSMUSG00000030739	Myh14
ENSMUSG00000009035	Tmem184b
ENSMUSG00000027951	Adar
ENSMUSG00000021061	Sptb
ENSMUSG00000022756	Slc7a4
ENSMUSG00000026959	Grin1
ENSMUSG00000020261	Slc36a1
ENSMUSG00000036591	Arhgap21
ENSMUSG00000001229	Dpp9
ENSMUSG00000005886	Ncoa2
ENSMUSG00000042763	Maneal
ENSMUSG00000045039	Megf8
ENSMUSG00000056602	Fry
ENSMUSG00000063430	Wscd2
ENSMUSG00000032875	Arhgef17
ENSMUSG00000001829	Clpb
ENSMUSG00000039126	Prune2
ENSMUSG00000037989	Wnk2
ENSMUSG00000053293	Pom121
ENSMUSG00000002409	Dyrk1b
ENSMUSG00000026113	Inpp4a
ENSMUSG00000042625	Safb2
ENSMUSG00000029577	Ube3b
ENSMUSG00000029581	Fscn1
ENSMUSG00000034902	Pip5k1c

ENSMUSG00000023032	Slc4a8
ENSMUSG00000029550	Sppl3
ENSMUSG00000062785	Kcnc3
ENSMUSG00000049313	Sorl1
ENSMUSG00000024393	Prrc2a
ENSMUSG00000056493	Foxk1
ENSMUSG00000032187	Smarca4
ENSMUSG00000034891	Sncb
ENSMUSG00000031511	Arhgef7
ENSMUSG00000006464	Bbs1
ENSMUSG00000034928	Rnf44
ENSMUSG00000048826	Dact2
ENSMUSG00000038256	Bcl9
ENSMUSG00000039747	Orai2
ENSMUSG00000020747	Tmem94
ENSMUSG00000022946	Dopey2
ENSMUSG00000026425	Srgap2
ENSMUSG00000044288	Cnr1
ENSMUSG00000029104	Htt
ENSMUSG00000029033	Acap3
ENSMUSG00000020889	Nr1d1
ENSMUSG00000030852	Tacc2
ENSMUSG00000045973	Slc25a51
ENSMUSG00000047248	C2cd3
ENSMUSG00000003575	Crtc1

NAc_MaleUp_FemaleDown

ENSEMBL_ID	gene_name
ENSMUSG00000025351	Cd63
ENSMUSG00000036309	Skp1a
ENSMUSG00000059291	Rpl11
ENSMUSG00000037601	Nme1
ENSMUSG00000024608	Rps14
ENSMUSG00000060073	Psma3
ENSMUSG00000042712	Tceal9
ENSMUSG00000057863	Rpl36
ENSMUSG00000049517	Rps23
ENSMUSG00000071415	Rpl23
ENSMUSG00000071528	Usmg5
ENSMUSG00000053398	Phgdh
ENSMUSG00000031320	Rps4x
ENSMUSG00000014294	Ndufa2
ENSMUSG00000045128	Rpl18a
ENSMUSG00000058318	Phf21a
ENSMUSG00000073616	Cops9
ENSMUSG00000026750	Psmb7
ENSMUSG00000059070	Rpl18
ENSMUSG00000037286	Stag1
ENSMUSG00000042750	Bex2
ENSMUSG00000057322	Rpl38
ENSMUSG00000008373	Prpf31
ENSMUSG00000026311	Asb1
ENSMUSG00000028347	Tmeff1
ENSMUSG00000028495	Rps6

ENSMUSG00000031848	Lsm4
ENSMUSG00000039530	Tusc3
ENSMUSG00000029833	Trim24
ENSMUSG00000049751	Rpl36al
ENSMUSG00000025340	Rabgef1
ENSMUSG00000026238	Ptma
ENSMUSG00000057858	Fam204a
ENSMUSG00000014769	Psmb1
ENSMUSG00000020460	Rps27a
ENSMUSG00000017778	Cox7c
ENSMUSG00000098274	Rpl24
ENSMUSG00000014956	Ppp1cb
ENSMUSG00000058325	Dock1
ENSMUSG00000020267	Hint1
ENSMUSG00000028410	Dnaja1
ENSMUSG00000027581	Stmn3
ENSMUSG00000054091	1810037I17Rik
ENSMUSG00000018567	Gabarap
ENSMUSG00000022674	Ube2v2
ENSMUSG00000039470	Zdhhc2
ENSMUSG00000061983	Rps12
ENSMUSG00000026032	Ndufb3
ENSMUSG00000034345	Gtf2h5
ENSMUSG00000034566	Atp5h
ENSMUSG00000048249	Crebrf
ENSMUSG00000058546	Rpl23a
ENSMUSG00000062997	Rpl35
ENSMUSG00000020857	Nme2
ENSMUSG00000021606	Ndufs6
ENSMUSG00000035726	Supt16
ENSMUSG00000023089	Ndufa5
ENSMUSG00000061130	Ppm1b
ENSMUSG00000004945	Tmem242
ENSMUSG00000050071	Bex1
ENSMUSG00000021794	Glud1
ENSMUSG00000022498	Txndc11
ENSMUSG00000032112	Trappc4
ENSMUSG00000024976	Shoc2
ENSMUSG00000006333	Rps9
ENSMUSG00000036422	Pcdh8
ENSMUSG00000093674	Rpl41
ENSMUSG00000030744	Rps3
ENSMUSG00000025967	Eef1b2
ENSMUSG00000022205	Sub1
ENSMUSG00000024953	Prdx5
ENSMUSG00000024099	Ndufv2
ENSMUSG00000089417	Gm22009
ENSMUSG00000020180	Snrpd3
ENSMUSG00000028029	Aimp1
ENSMUSG00000038690	Atp5j2
ENSMUSG00000079509	Zfx
ENSMUSG00000037563	Rps16
ENSMUSG00000031299	Pdha1

ENSMUSG00000006412	Pfdn2
ENSMUSG00000020538	Srebf1
ENSMUSG00000021831	Ero1l
ENSMUSG00000030647	Ndufc2
ENSMUSG00000061315	Naca
ENSMUSG00000046330	Rpl37a
ENSMUSG00000022311	Csmd3
ENSMUSG00000079435	Rpl36a
ENSMUSG00000008668	Rps18
ENSMUSG00000090247	Bloc1s1
ENSMUSG00000025980	Hspd1
ENSMUSG00000028759	Hp1bp3
ENSMUSG00000024074	Crim1
ENSMUSG00000002768	Mea1
ENSMUSG00000043067	Dpy19l1
ENSMUSG00000021520	Uqcrb
ENSMUSG00000055733	Nap1l3
ENSMUSG00000055762	Eef1d
ENSMUSG00000068823	Csde1
ENSMUSG00000096472	Cdkn2d
ENSMUSG00000020124	Usp15
ENSMUSG00000029131	Dnajb6
ENSMUSG00000022744	Cldnd1
ENSMUSG00000038607	Gng10
ENSMUSG00000050334	C130071C03Rik
ENSMUSG00000023236	Scg5
ENSMUSG00000021400	Wrnip1
ENSMUSG00000023147	Wrb
ENSMUSG00000062270	Morf4l1
ENSMUSG00000024012	Mtch1
ENSMUSG00000109324	Prmt1
ENSMUSG00000031839	Hsbp1
ENSMUSG00000047215	Rpl9
ENSMUSG00000005779	Psmb4
ENSMUSG00000030842	Lamtor1
ENSMUSG00000019923	Zwint
ENSMUSG00000021660	Btf3
ENSMUSG00000021711	Trappc13
ENSMUSG00000022452	Smdt1
ENSMUSG00000025104	Hdgfl3
ENSMUSG00000028102	Pex11b
ENSMUSG00000001289	Pfdn5
ENSMUSG00000038274	Fau
ENSMUSG00000019471	Cdc37
ENSMUSG00000036879	Phkb
ENSMUSG00000019775	Rgs17
ENSMUSG00000039682	Lap3
ENSMUSG00000003062	Stard3nl
ENSMUSG00000025889	Snca
ENSMUSG00000027170	Eif3m
ENSMUSG00000032527	Pccb
ENSMUSG00000042541	Sem1
ENSMUSG00000079108	Srp54b

ENSMUSG00000004285	Atp6v1f
ENSMUSG00000028419	Chmp5
ENSMUSG00000028648	Ndufs5
ENSMUSG00000034203	Chchd4
ENSMUSG00000020368	Canx
ENSMUSG00000020849	Ywhae
ENSMUSG00000026914	Psmd14
ENSMUSG00000068039	Tcp1
ENSMUSG00000000184	Ccnd2
ENSMUSG00000006932	Ctnnb1
ENSMUSG00000022403	St13
ENSMUSG00000028081	Rps3a1
ENSMUSG00000033624	Pdpr
ENSMUSG00000018286	Psmb6
ENSMUSG00000021114	Atp6v1d
ENSMUSG00000027447	Cst3
ENSMUSG00000033938	Ndufb7
ENSMUSG00000058558	Rpl5
ENSMUSG00000034024	Cct2
ENSMUSG00000046434	Hnrnpa1
ENSMUSG00000069117	Gm10260
ENSMUSG00000020390	Ube2b
ENSMUSG00000070493	Chchd2
ENSMUSG00000016252	Atp5e
ENSMUSG00000046985	Tapt1
ENSMUSG00000032350	Gclc
ENSMUSG00000021102	Glrx5
ENSMUSG00000022354	Ndufb9
ENSMUSG00000015092	Edf1
ENSMUSG00000026915	Strbp
ENSMUSG00000028062	Lamtor2
ENSMUSG00000031954	Cfdp1
ENSMUSG00000039016	Timm8b
ENSMUSG00000046432	Bex3
ENSMUSG00000052837	Junb
ENSMUSG00000053799	Exoc6
ENSMUSG00000074884	Serf2
ENSMUSG00000053565	Eif3k
ENSMUSG00000028437	Ubap1
ENSMUSG00000030282	Cmas
ENSMUSG00000038717	Atp5l
ENSMUSG00000001783	Rtcb
ENSMUSG00000022151	Ttc33
ENSMUSG00000079179	Rab10os
ENSMUSG00000072772	Grcc10
ENSMUSG00000088025	Rprl3
ENSMUSG00000062929	Cfl2
ENSMUSG00000034484	Snx2
ENSMUSG00000025613	Cct8
ENSMUSG00000035242	Oaz1
ENSMUSG00000037706	Cd81
ENSMUSG00000031422	Morf4l2
ENSMUSG00000036966	Spryd3

ENSMUSG00000016554	Eif3d
ENSMUSG00000022811	Zfp148
ENSMUSG00000030007	Cct7
ENSMUSG00000044647	Csrnp3
ENSMUSG00000044715	Gskip
ENSMUSG00000048000	Gigyf2
ENSMUSG00000049191	Rtl5
ENSMUSG00000057335	Cep170
ENSMUSG00000057561	Eif1a
ENSMUSG00000062797	Hikeshi
ENSMUSG00000078812	Eif5a
ENSMUSG00000079641	Rpl39
ENSMUSG00000020608	Smc6
ENSMUSG00000060126	Tpt1
ENSMUSG00000097451	Rian
ENSMUSG00000060675	Pla2g16
ENSMUSG00000031333	Abcb7
ENSMUSG00000020694	Tlk2
ENSMUSG00000028522	Mier1
ENSMUSG00000020311	Erlec1
ENSMUSG00000024423	Impact
ENSMUSG00000051674	Dcun1d4
ENSMUSG00000038301	Snx10
ENSMUSG00000037058	Paip2
ENSMUSG00000047261	Gap43
ENSMUSG00000020646	Mboat2
ENSMUSG00000020022	Ndufa12
ENSMUSG00000019647	Sema6a
ENSMUSG00000038900	Rpl12
ENSMUSG00000001833	Sept7
ENSMUSG00000083327	Vcp-rs
ENSMUSG00000036699	Zcchc12
ENSMUSG00000022338	Eny2
ENSMUSG00000032854	Ugt8a
ENSMUSG00000030754	Copb1
ENSMUSG00000034647	Ankrd12
ENSMUSG00000024921	Smarca2
ENSMUSG00000033808	Tmem87a
ENSMUSG00000074909	Ranbp6
ENSMUSG00000060373	Hnrnpc
ENSMUSG00000055430	Nap1l5
ENSMUSG00000091537	Tma7
ENSMUSG00000025920	Stau2
ENSMUSG00000029038	Ssu72
ENSMUSG00000078941	Ak6
ENSMUSG00000009535	Rnmt
ENSMUSG00000030839	Sergef
ENSMUSG00000031708	Tecr
ENSMUSG00000029993	Nfu1
ENSMUSG00000038374	Rbm8a
ENSMUSG00000031818	Cox4i1
ENSMUSG00000028878	Fam76a
ENSMUSG00000029614	Rpl6

ENSMUSG00000020612	Prkar1a
ENSMUSG00000033916	Chmp2a
ENSMUSG00000027852	Nras
ENSMUSG00000019054	Fis1
ENSMUSG00000032601	Prkar2a
ENSMUSG00000036398	Ppp1r11
ENSMUSG00000049775	Tmsb4x
ENSMUSG00000076432	Ywhaq
ENSMUSG00000036550	Cnot1
ENSMUSG00000031760	Mt3
ENSMUSG00000037072	Selenof
ENSMUSG00000000563	Atp5f1
ENSMUSG00000026421	Csrp1
ENSMUSG00000032513	Gorasp1
ENSMUSG00000030335	Mrpl51
ENSMUSG00000032557	Uba5
ENSMUSG00000035960	Apex1
ENSMUSG00000039033	Tasp1
ENSMUSG00000020919	Stat5b
ENSMUSG00000016427	Ndufa1
ENSMUSG00000031409	Tceal6
ENSMUSG00000021555	Naa35
ENSMUSG00000029153	Ociad2
ENSMUSG00000043388	Tmem130
ENSMUSG00000042699	Dhx9
ENSMUSG00000019590	Cyb561
ENSMUSG00000028156	Eif4e
ENSMUSG00000025666	Tmem47
ENSMUSG00000026851	BC005624
ENSMUSG00000028234	Rps20
ENSMUSG00000022698	Naa50
ENSMUSG00000047675	Rps8
ENSMUSG00000021124	Vti1b
ENSMUSG00000035354	Uvrag
ENSMUSG00000041912	Tdrkh
ENSMUSG00000062352	Itgb1bp1
ENSMUSG00000052459	Atp6v1a
ENSMUSG00000006699	Cdc42
ENSMUSG00000029152	Ociad1
ENSMUSG00000022708	Zbtb20
ENSMUSG00000027998	Plrg1
ENSMUSG00000031950	Gabarapl2
ENSMUSG00000044627	Swi5
ENSMUSG00000051483	Cbr1
ENSMUSG00000052146	Rps10
ENSMUSG00000025290	Rps24
ENSMUSG00000032301	Psma4
ENSMUSG00000025630	Hprt
ENSMUSG00000003531	Dgcr6
ENSMUSG00000032215	Rsl24d1
ENSMUSG00000027944	Hax1
ENSMUSG00000035227	Spcs2
ENSMUSG00000039221	Rpl22l1

ENSMUSG00000071644	Eef1g
ENSMUSG00000025255	Zfhx4
ENSMUSG00000040785	Ttc3
ENSMUSG00000020075	Ddx21
ENSMUSG00000041236	Vps41
ENSMUSG00000070319	Eif3g
ENSMUSG00000026991	Pkp4
ENSMUSG00000037257	Aagab
ENSMUSG00000063410	Stk24
ENSMUSG00000067873	Htatsf1
ENSMUSG00000071655	Ubxn1
ENSMUSG00000019966	Kitl
ENSMUSG00000046364	Rpl27a
ENSMUSG00000050856	Atp5k
ENSMUSG00000074129	Rpl13a
ENSMUSG00000079658	Eloc
ENSMUSG00000039428	Tmem135
ENSMUSG00000010376	Nedd8
ENSMUSG00000018736	Ndel1
ENSMUSG00000023883	Phf10
ENSMUSG00000025439	Clns1a
ENSMUSG00000030638	Sh3gl3
ENSMUSG00000028367	Txn1
ENSMUSG00000042901	Aida
ENSMUSG00000028274	Rngtt
ENSMUSG00000062580	Timm17a
ENSMUSG00000029141	Slc4a1ap
ENSMUSG00000039105	Atp6v1g1
ENSMUSG00000027804	Ppid
ENSMUSG00000002015	Bcap31
ENSMUSG00000027007	Ssfa2
ENSMUSG00000043004	Gng2
ENSMUSG00000019868	Vta1
ENSMUSG00000021497	Txndc15
ENSMUSG00000031584	Gsr
ENSMUSG00000021669	Col4a3bp
ENSMUSG00000034820	Cpsf7
ENSMUSG00000002341	Ncan
ENSMUSG00000036199	Ndufa13
ENSMUSG00000044550	Tceal3
ENSMUSG00000051234	Rnf7
ENSMUSG00000024182	Axin1
ENSMUSG00000021420	Fars2
ENSMUSG00000024997	Prdx3
ENSMUSG00000025362	Rps26
ENSMUSG00000025103	Btbd1
ENSMUSG00000028692	Akr1a1
ENSMUSG00000035274	Tpbg
ENSMUSG00000072946	Ptgr2
ENSMUSG00000020720	Psmd12
ENSMUSG00000021218	Gdi2
ENSMUSG00000043716	Rpl7
ENSMUSG00000029404	Arl6ip4

ENSMUSG00000031231	Cox7b
ENSMUSG00000001056	Nhp2
ENSMUSG00000005846	Rsl1d1
ENSMUSG00000043419	Chd3os
ENSMUSG00000072294	Klf12
ENSMUSG00000074968	Ano3
ENSMUSG00000057841	Rpl32
ENSMUSG00000029466	Anapc7
ENSMUSG00000002602	Axl
ENSMUSG00000014195	Dnajc7
ENSMUSG00000020088	Sar1a
ENSMUSG00000024261	Syt4
ENSMUSG00000027282	Mtch2
ENSMUSG00000027423	Snx5
ENSMUSG00000046449	Nexmif
ENSMUSG00000045136	Tubb2b
ENSMUSG00000041459	Tardbp
ENSMUSG00000043384	Gprasp1
ENSMUSG00000088789	Scarna13
ENSMUSG00000040667	Nup88
ENSMUSG00000036112	Metap2
ENSMUSG00000029465	Arpc3
ENSMUSG00000034152	Exoc3
ENSMUSG00000030298	Sec13
ENSMUSG00000039128	Cdc123
ENSMUSG00000060743	H3f3a
ENSMUSG00000020922	Lsm12
ENSMUSG00000033904	Ccp110
ENSMUSG00000037434	Slc30a1
ENSMUSG00000042642	Flad1
ENSMUSG00000044528	Tram1l1
ENSMUSG00000054428	Atpif1
ENSMUSG00000020708	Psmc5
ENSMUSG00000036585	Fgf1
ENSMUSG00000066643	Wdr35
ENSMUSG00000021096	Ppm1a
ENSMUSG00000058897	Col25a1
ENSMUSG00000073702	Rpl31
ENSMUSG00000037710	Cisd1
ENSMUSG00000025135	Anapc11
ENSMUSG00000025508	Rplp2
ENSMUSG00000020070	Rufy2
ENSMUSG00000023827	Agpat4
ENSMUSG00000026150	Mff
ENSMUSG00000029992	Gfpt1
ENSMUSG00000031059	Ndufb11
ENSMUSG00000032118	Fez1
ENSMUSG00000051527	Usp29
ENSMUSG00000021131	Erh
ENSMUSG00000032412	Atp1b3
ENSMUSG00000045098	Kmt5b
ENSMUSG00000028954	Nub1
ENSMUSG00000058317	Ube2e2

ENSMUSG00000022982	Sod1
ENSMUSG00000005732	Ranbp1
ENSMUSG00000034377	Tulp4
ENSMUSG00000099250	Rn7s2
ENSMUSG00000024425	Ndfip1
ENSMUSG00000034973	Dopey1
ENSMUSG00000057455	Rit2
ENSMUSG00000060860	Ube2s
ENSMUSG00000102252	Snrpn
ENSMUSG00000020547	Bzw2
ENSMUSG00000035228	Ccdc106
ENSMUSG00000045205	Dpy19l4
ENSMUSG00000023952	Gtpbp2
ENSMUSG00000026434	Nucks1
ENSMUSG00000062006	Rpl34
ENSMUSG00000051851	Rtl8c
ENSMUSG00000001260	Gabrg1
ENSMUSG00000021764	Ndufs4
ENSMUSG00000026721	Rabgap1l
ENSMUSG00000029623	Pdap1
ENSMUSG00000020811	Wscd1
ENSMUSG00000032482	Cspg5
ENSMUSG00000044080	S100a1
ENSMUSG00000057531	Dtnbp1
ENSMUSG00000060938	Rpl26
ENSMUSG00000032316	Clk3
ENSMUSG00000045095	Magi1
ENSMUSG00000089872	Rps6kc1
ENSMUSG00000026319	2310035C23Rik
ENSMUSG00000036438	Calm2
ENSMUSG00000041923	Nol4
ENSMUSG00000046516	Cox17
ENSMUSG00000068396	Rpl34-ps1
ENSMUSG00000031157	Pqbp1
ENSMUSG00000058799	Nap1l1
ENSMUSG00000017404	Rpl19
ENSMUSG00000021709	Erbin
ENSMUSG00000026895	Ndufa8
ENSMUSG00000029918	Mrps33
ENSMUSG00000034681	Rnps1
ENSMUSG00000036580	Spg20
ENSMUSG00000040524	Zfp609
ENSMUSG00000049106	Dcaf5
ENSMUSG00000067847	Romo1
ENSMUSG00000044221	Grsf1
ENSMUSG00000019877	Serinc1
ENSMUSG00000033186	Mzt1
ENSMUSG00000091228	Gm20390
ENSMUSG00000115783	AC161763.1
ENSMUSG00000030654	Arl6ip1
ENSMUSG00000019699	Akt3
ENSMUSG00000031402	Mpp1
ENSMUSG00000068739	Sars

ENSMUSG00000025404	R3hdm2
ENSMUSG00000039286	Fndc3b
ENSMUSG00000021413	Prpf4b
ENSMUSG00000040681	Hmgn1
ENSMUSG00000013701	Timm23
ENSMUSG00000031502	Col4a1
ENSMUSG00000047547	Cltb
ENSMUSG00000059263	Usp47
ENSMUSG00000058672	Tubb2a
ENSMUSG00000033059	Pygb
ENSMUSG00000003380	Rabac1
ENSMUSG00000003970	Rpl8
ENSMUSG00000020436	Gabrg2
ENSMUSG00000051223	Bzw1
ENSMUSG00000022956	Atp5o
ENSMUSG00000026830	Ermn
ENSMUSG00000020265	Sumo3
ENSMUSG00000040128	Pnrc1
ENSMUSG00000015806	Qdpr
ENSMUSG00000021037	Ahsa1
ENSMUSG00000025364	Pa2g4
ENSMUSG00000106864	Gtf3c2
ENSMUSG00000035674	Ndufa3
ENSMUSG00000044117	2900011O08Rik
ENSMUSG00000024083	Pja2
ENSMUSG00000002395	Use1
ENSMUSG00000028945	Rheb
ENSMUSG00000062647	Rpl7a
ENSMUSG00000024208	Uqcc2
ENSMUSG00000067786	Nnat
ENSMUSG00000031974	Abcb10
ENSMUSG00000039735	Fnbp1l
ENSMUSG00000062328	Rpl17
ENSMUSG00000028484	Psip1
ENSMUSG00000039485	Tspyl4
ENSMUSG00000038619	Ensa
ENSMUSG00000028821	Syf2
ENSMUSG00000020130	Tbc1d15
ENSMUSG00000027680	Fxr1
ENSMUSG00000062761	Zfp512
ENSMUSG00000024359	Hspa9
ENSMUSG00000090112	Shprh
ENSMUSG00000022400	Rbx1
ENSMUSG00000022577	Ly6h
ENSMUSG00000040652	Oaz2
ENSMUSG00000029017	Pmpcb
ENSMUSG00000040746	Rnf167
ENSMUSG00000039067	Psmd7
ENSMUSG00000029147	Ppm1g
ENSMUSG00000050608	Minos1
ENSMUSG00000006373	Pgrmc1
ENSMUSG00000032434	Cmtm6
ENSMUSG00000037826	Ppm1k

ENSMUSG00000056537	Rlim
ENSMUSG00000020993	Trappc6b
ENSMUSG00000027942	4933434E20Rik
ENSMUSG00000038437	Mllt6
ENSMUSG00000024309	Pfdn6
ENSMUSG00000026335	Pam
ENSMUSG00000040570	Rundc3b
ENSMUSG00000008036	Ap2s1
ENSMUSG00000026526	Fh1
ENSMUSG00000020458	Rtn4
ENSMUSG00000032309	Fbxo22
ENSMUSG00000000168	Dlat
ENSMUSG00000039100	March6
ENSMUSG00000021076	Actr10
ENSMUSG00000003778	Brd8
ENSMUSG00000006288	Ttc5
ENSMUSG00000030204	Ddx47
ENSMUSG00000030868	Dctn5
ENSMUSG00000031148	Gpkow
ENSMUSG00000031631	Cfap97
ENSMUSG00000031683	Lsm6
ENSMUSG00000062078	Qk
ENSMUSG00000074457	S100a16
ENSMUSG00000001376	Vps50
ENSMUSG00000025794	Rpl14
ENSMUSG00000026360	Rgs2
ENSMUSG00000033578	Tmem35a
ENSMUSG00000068921	Dap3
ENSMUSG00000001313	Rnd2
ENSMUSG00000018770	Atp5g3
ENSMUSG00000027651	Rprd1b
ENSMUSG00000032458	Copb2
ENSMUSG00000028245	Nsmaf
ENSMUSG00000029723	Tsc22d4
ENSMUSG00000031918	Mtmr2
ENSMUSG00000115987	Vps28
ENSMUSG00000020986	Sec23a
ENSMUSG00000020954	Strn3
ENSMUSG00000038127	Ccdc50
ENSMUSG00000025156	Gps1
ENSMUSG00000026019	Wdr12
ENSMUSG00000034403	Pja1
ENSMUSG00000036299	BC031181
ENSMUSG00000040649	Rimklb
ENSMUSG00000052738	Suclg1
ENSMUSG00000062151	Unc13c
ENSMUSG00000078676	Casc3
ENSMUSG00000072694	1500011B03Rik
ENSMUSG00000024276	Zfp397
ENSMUSG00000025268	Maged2
ENSMUSG00000026034	Clk1
ENSMUSG00000027858	Tspan2
ENSMUSG00000044068	Zrsr1

ENSMUSG00000029571	Tmem106b
ENSMUSG00000029014	Dnajc2
ENSMUSG00000015790	Surf1
ENSMUSG00000071172	Srsf3
ENSMUSG00000020361	Hspa4
ENSMUSG00000043991	Pura
ENSMUSG00000020738	Sumo2
ENSMUSG00000024213	Nudt3
ENSMUSG00000025264	Tsr2
ENSMUSG00000041225	Arhgap12
ENSMUSG00000033712	Ccar2
ENSMUSG00000024248	Cox7a2l
ENSMUSG00000033577	Myo6
ENSMUSG00000090841	Myl6
ENSMUSG00000036902	Neto2
ENSMUSG00000024493	Lars
ENSMUSG00000031432	Prps1
ENSMUSG00000021576	Pdcd6
ENSMUSG00000023004	Tuba1b
ENSMUSG00000029328	Hnrnpdl
ENSMUSG00000021917	Spcs1
ENSMUSG00000022016	Akap11
ENSMUSG00000028670	Lypla2
ENSMUSG00000032076	Cadm1
ENSMUSG00000033883	D3Ertd254e
ENSMUSG00000034755	Pcdh11x
ENSMUSG00000008859	Rala
ENSMUSG00000067279	Ppp1r3c
ENSMUSG00000001380	Hars
ENSMUSG00000028988	Ctnnbip1
ENSMUSG00000055067	Smyd3
ENSMUSG00000071014	Ndufb6
ENSMUSG00000009575	Cbx5
ENSMUSG00000037514	Pank2
ENSMUSG00000018293	Pfn1
ENSMUSG00000030706	Mrpl48
ENSMUSG00000021670	Hmgcr
ENSMUSG00000015149	Sirt2
ENSMUSG00000031425	Plp1
ENSMUSG00000045294	Insig1
ENSMUSG00000036751	Cox6b1
ENSMUSG00000048537	Phldb1
ENSMUSG00000018377	Vezf1
ENSMUSG00000021792	Fam213a
ENSMUSG00000014226	Cacybp
ENSMUSG00000029638	Glcci1
ENSMUSG00000005899	Smpd4
ENSMUSG00000020949	Fkbp3
ENSMUSG00000027176	Cstf3
ENSMUSG00000027349	Fam98b
ENSMUSG00000027430	Dtd1
ENSMUSG00000030541	Idh2
ENSMUSG00000031660	Brd7

ENSMUSG00000060988	Galnt13
ENSMUSG00000074656	Eif2s2
ENSMUSG00000026344	Lypd1
ENSMUSG00000024560	Cxxc1
ENSMUSG00000009927	Rps25
ENSMUSG00000019362	D8Ertd738e
ENSMUSG00000024121	Atp6v0c
ENSMUSG00000026234	Ncl
ENSMUSG00000028233	Tgs1
ENSMUSG00000052364	B630019K06Rik
ENSMUSG00000056296	Synpr
ENSMUSG00000075706	Gpx4
ENSMUSG00000041355	Ssr2
ENSMUSG00000024038	Ndufv3
ENSMUSG00000029632	Ndufa4
ENSMUSG00000031367	Ap1s2
ENSMUSG00000032330	Cox7a2
ENSMUSG00000040363	Bcor
ENSMUSG00000057411	Fam173a
ENSMUSG00000032932	Hspa13
ENSMUSG00000005973	Rcn1
ENSMUSG00000025982	Sf3b1
ENSMUSG00000053644	Aldh7a1
ENSMUSG00000083773	Gm13394
ENSMUSG00000047187	Rab2a
ENSMUSG00000034724	Cnot6l
ENSMUSG00000021156	Zmynd11
ENSMUSG00000020260	Pofut2
ENSMUSG00000021996	Esd
ENSMUSG00000000948	Gm38393
ENSMUSG00000066705	Fxyd6
ENSMUSG00000078899	Gm4631
ENSMUSG00000080465	Gm22486
ENSMUSG00000031149	Praf2
ENSMUSG00000052712	BC004004
ENSMUSG00000025130	P4hb
ENSMUSG00000025204	Ndufb8
ENSMUSG00000028779	Pef1
ENSMUSG00000050711	Scg2
ENSMUSG00000002332	Dhrs1
ENSMUSG00000039660	Spout1
ENSMUSG00000061904	Slc25a3
ENSMUSG00000046844	Vat1l
ENSMUSG00000032518	Rpsa
ENSMUSG00000025968	Ndufs1
ENSMUSG00000028998	Tomm7
ENSMUSG00000047879	Usp14
ENSMUSG00000060981	Hist1h4h
ENSMUSG00000078440	Dohh
ENSMUSG00000012848	Rps5
ENSMUSG00000018451	6330403K07Rik
ENSMUSG00000022193	Psmb5
ENSMUSG00000044876	Zfp444

ENSMUSG00000053080	2700081O15Rik
ENSMUSG00000040824	Snrpd2
ENSMUSG00000041141	Pnmal1
ENSMUSG00000003038	Hmgn2
ENSMUSG00000027180	Fbxo3
ENSMUSG00000072235	Tuba1a
ENSMUSG00000077714	Snord17
ENSMUSG00000033585	Ndn
ENSMUSG00000073888	Ccl27a
ENSMUSG00000005371	Fbxo11
ENSMUSG00000000088	Cox5a
ENSMUSG00000028226	Mmp16
ENSMUSG00000020821	Kif1c
ENSMUSG00000009549	Srp14
ENSMUSG00000078903	Gm14391
ENSMUSG00000022234	Cct5
ENSMUSG00000042616	Oscp1
ENSMUSG00000047098	Rnf31
ENSMUSG00000024218	Taf11
ENSMUSG00000041220	Elovl6
ENSMUSG00000014606	Slc25a11
ENSMUSG00000026020	Nop58
ENSMUSG00000005034	Prkacb
ENSMUSG00000027620	Rbm39
ENSMUSG00000022420	Dnal4
ENSMUSG00000030763	Lcmt1
ENSMUSG00000031099	Smarca1
ENSMUSG00000067288	Rps28
ENSMUSG00000020346	Mgat1
ENSMUSG00000032060	Cryab
ENSMUSG00000022884	Eif4a2
ENSMUSG00000027303	Ptpra
ENSMUSG00000026229	Psmd1
ENSMUSG00000061702	Tmem91
ENSMUSG00000029878	Dbpht2
ENSMUSG00000040351	Ankib1
ENSMUSG00000061482	Hist1h4d
ENSMUSG00000090935	Synj2bp
ENSMUSG00000117286	NA
ENSMUSG00000002580	Mien1
ENSMUSG00000011179	Odc1
ENSMUSG00000015291	Gdi1
ENSMUSG00000022280	Rnf19a
ENSMUSG00000024500	Ppp2r2b
ENSMUSG00000024665	Fads2
ENSMUSG00000025978	Rftn2
ENSMUSG00000028633	Ctps
ENSMUSG00000028691	Prdx1
ENSMUSG00000030246	Ldhb
ENSMUSG00000031066	Usp11
ENSMUSG00000033940	Brk1
ENSMUSG00000071533	Pcnp
ENSMUSG00000072640	Lyrm9

ENSMUSG00000021024	Psma6
ENSMUSG00000073838	Tufm
ENSMUSG00000025041	Nt5c2
ENSMUSG00000006315	Tmem147
ENSMUSG00000022723	Crybg3
ENSMUSG00000004892	Bcan
ENSMUSG00000019977	Hbs1l
ENSMUSG00000020277	Pfkl
ENSMUSG00000020783	Ncbp3
ENSMUSG00000021149	Gtpbp4
ENSMUSG00000021194	Chga
ENSMUSG00000022661	Cd200
ENSMUSG00000024158	Hagh
ENSMUSG00000024271	Elp2
ENSMUSG00000027566	Psma7
ENSMUSG00000029390	Tmed2
ENSMUSG00000029518	Rab35
ENSMUSG00000030056	Isy1
ENSMUSG00000030788	Rnf141
ENSMUSG00000032297	Celf6
ENSMUSG00000033475	Tomm6
ENSMUSG00000048701	Ccdc6
ENSMUSG00000063882	Uqcrh
ENSMUSG00000066357	Wdr6
ENSMUSG00000073678	Pgap1
ENSMUSG00000016541	Atxn10
ENSMUSG00000063511	Snrnp70
ENSMUSG00000027162	Lin7c
ENSMUSG00000027200	Sema6d
ENSMUSG00000036560	Lgi4
ENSMUSG00000040669	Phc1
ENSMUSG00000022261	Sdc2
ENSMUSG00000028851	Nudc
ENSMUSG00000036712	Cyld
ENSMUSG00000038702	Dsel
ENSMUSG00000039615	Stub1
ENSMUSG00000037916	Ndufv1
ENSMUSG00000041841	Rpl37
ENSMUSG00000021779	Thrb
ENSMUSG00000043154	Ppp2r3a
ENSMUSG00000006930	Hap1
ENSMUSG00000020386	Sar1b
ENSMUSG00000020444	Guk1
ENSMUSG00000025203	Scd2
ENSMUSG00000030161	Gabarapl1
ENSMUSG00000033460	Armcx1
ENSMUSG00000033629	Hacd3
ENSMUSG00000000266	Mid2
ENSMUSG00000006095	Tbcb
ENSMUSG00000023175	Bsg
ENSMUSG00000030086	Chchd6
ENSMUSG00000038489	Polr2l
ENSMUSG00000053291	Rab4b

ENSMUSG00000027199	Gatm
ENSMUSG00000038388	Mpp6
ENSMUSG00000008683	Rps15a
ENSMUSG00000026869	Psmd5
ENSMUSG00000031202	Rab39b
ENSMUSG00000049305	Ccdc71
ENSMUSG00000024231	Cul2
ENSMUSG00000000740	Rpl13
ENSMUSG00000049804	Armcx4
ENSMUSG00000061353	Cxcl12
ENSMUSG00000018196	Glrx2
ENSMUSG00000021969	Zdhhc20
ENSMUSG00000025764	Jade1
ENSMUSG00000026028	Trak2
ENSMUSG00000032959	Pebp1
ENSMUSG00000041592	Sdk2
ENSMUSG00000029559	2210016L21Rik
ENSMUSG00000034563	Ccpg1
ENSMUSG00000021290	2010107E04Rik
ENSMUSG00000061666	Gdpd1
ENSMUSG00000106379	Lhfpl3
ENSMUSG00000058240	Cryzl1
ENSMUSG00000005102	Eif2ak4
ENSMUSG00000020114	Cand1
ENSMUSG00000023904	Hcfc1r1
ENSMUSG00000063480	Snu13
ENSMUSG00000005505	Kbtbd4
ENSMUSG00000020115	Tbk1
ENSMUSG00000079562	Maea
ENSMUSG00000002416	Ndufb2
ENSMUSG00000014077	Chp1
ENSMUSG00000046876	Atxn1
ENSMUSG00000061859	Patj
ENSMUSG00000020640	Itsn2
ENSMUSG00000035824	Tk2
ENSMUSG00000058761	Rnf169
ENSMUSG00000020297	Nsg2
ENSMUSG00000020664	Dld
ENSMUSG00000020740	Gga3
ENSMUSG00000024926	Kat5
ENSMUSG00000027849	Syt6
ENSMUSG00000028811	Yars
ENSMUSG00000031167	Rbm3
ENSMUSG00000033342	Plppr5
ENSMUSG00000033793	Atp6v1h
ENSMUSG00000046204	Pnma2
ENSMUSG00000053483	Usp21
ENSMUSG00000054648	Zfp869
ENSMUSG00000059981	Taok2
ENSMUSG00000061477	Rps7
ENSMUSG00000051615	Rap2a
ENSMUSG00000029475	Kdm2b
ENSMUSG00000062421	Arf2

ENSMUSG00000025474	Tubgcp2
ENSMUSG00000030779	Rbbp6
ENSMUSG00000002658	Gtf2f1
ENSMUSG00000031913	Vps4a
ENSMUSG00000042439	Zfp532
ENSMUSG00000024966	Stip1
ENSMUSG00000026112	Coa5
ENSMUSG00000027195	Hsd17b12
ENSMUSG00000032705	Exd2
ENSMUSG00000037685	Atp8a1
ENSMUSG00000043467	Zbtb37
ENSMUSG00000012535	Tnpo3
ENSMUSG00000020572	Nampt
ENSMUSG00000020716	Nf1
ENSMUSG00000030307	Slc6a11
ENSMUSG00000053436	Mapk14
ENSMUSG00000086119	Gm2415
ENSMUSG00000034789	Rab24
ENSMUSG00000036275	9530068E07Rik
ENSMUSG00000034801	Sos2
ENSMUSG00000002625	Akap8l
ENSMUSG00000000420	Galnt1
ENSMUSG00000006418	Rnf114
ENSMUSG00000027466	Rbck1
ENSMUSG00000029135	Fosl2
ENSMUSG00000040548	Tex2
ENSMUSG00000042873	Lhfpl4
ENSMUSG00000047786	Lix1
ENSMUSG00000054920	Klhl5
ENSMUSG00000013523	Bcas1
ENSMUSG00000021703	Serinc5
ENSMUSG00000026411	Tmem9
ENSMUSG00000026426	Arl8a
ENSMUSG00000026904	Slc4a10
ENSMUSG00000029048	Rer1
ENSMUSG00000030016	Zfp638
ENSMUSG00000030701	Plekhb1
ENSMUSG00000035285	Nat14
ENSMUSG00000060450	Rnf14
ENSMUSG00000062070	Pgk1
ENSMUSG00000021983	Atp8a2
ENSMUSG00000036435	Exoc1
ENSMUSG00000031428	Zcchc18
ENSMUSG00000020483	Dynll2
ENSMUSG00000020307	Cdc34
ENSMUSG00000061787	Rps17
ENSMUSG00000043929	Klhl15
ENSMUSG00000057406	Nsd2
ENSMUSG00000034813	Grip1
ENSMUSG00000044912	Syt16
ENSMUSG00000022629	Kif21a
ENSMUSG00000041415	Dicer1
ENSMUSG00000079477	Rab7

ENSMUSG00000036053	Fmnl2
ENSMUSG00000023832	Acat2
ENSMUSG00000006941	Eif1b
ENSMUSG00000008035	Mid1ip1
ENSMUSG00000020982	Nemf
ENSMUSG00000033981	Gria2
ENSMUSG00000043909	Trp53bp1
ENSMUSG00000044502	Bod1
ENSMUSG00000024642	Tle4
ENSMUSG00000002012	Pnck
ENSMUSG00000031105	Slc25a14
ENSMUSG00000037239	Spred3
ENSMUSG00000022791	Tnk2
ENSMUSG00000037788	Vopp1
ENSMUSG00000036667	Tcaf1
ENSMUSG00000069520	Tmem19
ENSMUSG00000006782	Cnp
ENSMUSG00000012422	Tmem167
ENSMUSG00000038991	Txndc5
ENSMUSG00000093752	Gm20716
ENSMUSG00000005813	Metap1
ENSMUSG00000033900	Map9
ENSMUSG00000021276	Cinp
ENSMUSG00000026249	Serpine2
ENSMUSG00000054408	Spcs3
ENSMUSG00000030605	Mfge8
ENSMUSG00000022858	Tra2b
ENSMUSG00000025134	Alyref
ENSMUSG00000033444	Specc1l
ENSMUSG00000050697	Prkaa1
ENSMUSG00000015994	Fnta
ENSMUSG00000002660	Clpp
ENSMUSG00000026014	Raph1
ENSMUSG00000028826	Tmem57
ENSMUSG00000038132	Rbm24
ENSMUSG00000028937	Acot7
ENSMUSG00000031667	Aktip
ENSMUSG00000046160	Olig1
ENSMUSG00000022037	Clu
ENSMUSG00000036167	Pphln1
ENSMUSG00000072966	Gprasp2
ENSMUSG00000007892	Rplp1
ENSMUSG00000009418	Nav1
ENSMUSG00000026109	Tmeff2
ENSMUSG00000026617	Bpnt1
ENSMUSG00000027455	Nsfl1c
ENSMUSG00000028869	Gnl2
ENSMUSG00000035093	Secisbp2l
ENSMUSG00000038462	Uqcrfs1
ENSMUSG00000095969	Rnu1a1
ENSMUSG00000015671	Psma2
ENSMUSG00000028798	Eif3i
ENSMUSG00000026356	Dars

ENSMUSG00000030062	Rpn1
ENSMUSG00000040025	Ythdf2
ENSMUSG00000020132	Rab21
ENSMUSG00000000759	Tubgcp3
ENSMUSG00000037643	Prkci
ENSMUSG00000022255	Mtdh
ENSMUSG00000024420	Zfp521
ENSMUSG00000027210	Meis2
ENSMUSG00000024429	Gnl1
ENSMUSG00000015013	Trappc2l
ENSMUSG00000031622	Sin3b
ENSMUSG00000021796	Bmpr1a
ENSMUSG00000026773	Pfkfb3
ENSMUSG00000049670	Morn4
ENSMUSG00000055850	Rnf181
ENSMUSG00000069670	Nkain2
ENSMUSG00000006010	BC003331
ENSMUSG00000001525	Tubb5
ENSMUSG00000020308	Tpgs1
ENSMUSG00000029148	Nrbp1
ENSMUSG00000024357	Sil1
ENSMUSG00000024404	Riok3
ENSMUSG00000052656	Rnf103
ENSMUSG00000035653	Lrfn5
ENSMUSG00000020893	Per1
ENSMUSG00000026275	Ppp1r7
ENSMUSG00000031575	Ash2l
ENSMUSG00000018659	Pnpo
ENSMUSG00000031967	Afg3l1
ENSMUSG00000089911	Mfsd14a
ENSMUSG00000102070	Gm28661
ENSMUSG00000028542	Slc6a9
ENSMUSG00000027865	Gdap2
ENSMUSG00000039219	Arid4b
ENSMUSG00000021911	Parg
ENSMUSG00000022781	Pak2
ENSMUSG00000024501	Dpysl3
ENSMUSG00000025465	Echs1
ENSMUSG00000041997	Tlk1
ENSMUSG00000044365	Cxxc4
ENSMUSG00000060093	Hist1h4a
ENSMUSG00000062190	Lancl2
ENSMUSG00000031765	Mt1
ENSMUSG00000032410	Xrn1
ENSMUSG00000045193	Cirbp
ENSMUSG00000060475	Wtap
ENSMUSG00000000711	Rab5b
ENSMUSG00000020076	Ddx50
ENSMUSG00000036501	Fam13b
ENSMUSG00000032294	Pkm
ENSMUSG00000033487	Fndc3a
ENSMUSG00000005161	Prdx2
ENSMUSG00000040289	Hey1

ENSMUSG00000027533	Fabp5
ENSMUSG00000051177	Plcb1
ENSMUSG00000059208	Hnrnpm
ENSMUSG00000063856	Gpx1
ENSMUSG00000071662	Polr2g
ENSMUSG00000021466	Ptch1
ENSMUSG00000025035	Arl3
ENSMUSG00000094050	Gm23472
ENSMUSG00000003746	Man1a
ENSMUSG00000004562	Arhgef40
ENSMUSG00000021573	Tppp
ENSMUSG00000028249	Sdcbp
ENSMUSG00000052613	Pcdh15
ENSMUSG00000028284	Map3k7
ENSMUSG00000003934	Efnb3
ENSMUSG00000019338	Zfp687
ENSMUSG00000028397	Kdm4c
ENSMUSG00000089862	Umad1
ENSMUSG00000009630	Ppp2cb
ENSMUSG00000020918	Kat2a
ENSMUSG00000020978	Klhdc2
ENSMUSG00000021379	Id4
ENSMUSG00000022677	Fopnl
ENSMUSG00000024063	Lbh
ENSMUSG00000025208	Mrpl43
ENSMUSG00000027327	1700037H04Rik
ENSMUSG00000041697	Cox6a1
ENSMUSG00000078671	Chd2
ENSMUSG00000004961	Syt5
ENSMUSG00000003429	Rps11
ENSMUSG00000015804	Med28
ENSMUSG00000017734	Dbndd2
ENSMUSG00000031633	Slc25a4
ENSMUSG00000054942	Miga1
ENSMUSG00000029777	Gars
ENSMUSG00000002459	Rgs20
ENSMUSG00000022607	Ptk2
ENSMUSG00000028879	Stx12
ENSMUSG00000060739	Nsa2
ENSMUSG00000029836	Cbx3
ENSMUSG00000033237	Arid2
ENSMUSG00000034471	Caskin2
ENSMUSG00000038630	Zkscan16
ENSMUSG00000005510	Ndufs3
ENSMUSG00000020149	Rab1
ENSMUSG00000022568	Scrib
ENSMUSG00000022820	Ndufb4
ENSMUSG00000008348	Ubc
ENSMUSG00000030849	Fgfr2
ENSMUSG00000036555	Iqce
ENSMUSG00000010608	Rbm25
ENSMUSG00000029616	Erp29
ENSMUSG00000034488	Edil3

ENSMUSG00000036323	Srp72
ENSMUSG00000059474	Mbtd1
ENSMUSG00000030987	Stim1
ENSMUSG00000030942	Thumpd1
ENSMUSG00000005674	Tomm40l
ENSMUSG00000029722	Agfg2
ENSMUSG00000029776	Hibadh
ENSMUSG00000030265	Kras
ENSMUSG00000006356	Crip2
ENSMUSG00000046836	Brox
ENSMUSG00000024901	Peli3
ENSMUSG00000035150	Eif2s3x
ENSMUSG00000091337	Eid1
ENSMUSG00000018593	Sparc
ENSMUSG00000021111	Papola
ENSMUSG00000027669	Gnb4
ENSMUSG00000029672	Fam3c
ENSMUSG00000030815	Phkg2
ENSMUSG00000034245	Hdac11
ENSMUSG00000038416	Cdc16
ENSMUSG00000047037	Nipa1
ENSMUSG00000056201	Cfl1
ENSMUSG00000058076	Sdhc
ENSMUSG00000062963	Ufc1
ENSMUSG00000095526	Gm10243
ENSMUSG00000037646	Vps13b
ENSMUSG00000001891	Ugp2
ENSMUSG00000030095	Tmem43
ENSMUSG00000042523	Dnal1
ENSMUSG00000049090	Zadh2
ENSMUSG00000000600	Krit1
ENSMUSG00000036634	Mag
ENSMUSG00000020792	Exoc7
ENSMUSG00000033396	Spg11
ENSMUSG00000022890	Atp5j
ENSMUSG00000025337	Sbds
ENSMUSG00000039904	Gpr37
ENSMUSG00000015478	Rnf5
ENSMUSG00000031714	Gab1
ENSMUSG00000041571	Selenow
ENSMUSG00000033039	Micall1
ENSMUSG00000037148	Arhgap10
ENSMUSG00000002455	Prpf6
ENSMUSG00000021748	Pdhb
ENSMUSG00000022752	Tomm70a
ENSMUSG00000027854	Sike1
ENSMUSG00000031256	Cstf2

**BNST_background ENSEMBL_ID**	**NAc_background ENSEMBL_ID**
ENSMUSG00000000001	ENSMUSG00000000056
ENSMUSG00000000056	ENSMUSG00000000085
ENSMUSG00000000078	ENSMUSG00000000088
ENSMUSG00000000085	ENSMUSG00000000131

ENSMUSG00000000088	ENSMUSG00000000134
ENSMUSG00000000127	ENSMUSG00000000142
ENSMUSG00000000131	ENSMUSG00000000149
ENSMUSG00000000134	ENSMUSG00000000168
ENSMUSG00000000142	ENSMUSG00000000171
ENSMUSG00000000148	ENSMUSG00000000184
ENSMUSG00000000149	ENSMUSG00000000194
ENSMUSG00000000168	ENSMUSG00000000197
ENSMUSG00000000171	ENSMUSG00000000223
ENSMUSG00000000184	ENSMUSG00000000253
ENSMUSG00000000194	ENSMUSG00000000266
ENSMUSG00000000197	ENSMUSG00000000276
ENSMUSG00000000202	ENSMUSG00000000282
ENSMUSG00000000223	ENSMUSG00000000305
ENSMUSG00000000247	ENSMUSG00000000308
ENSMUSG00000000253	ENSMUSG00000000325
ENSMUSG00000000266	ENSMUSG00000000326
ENSMUSG00000000275	ENSMUSG00000000339
ENSMUSG00000000276	ENSMUSG00000000346
ENSMUSG00000000278	ENSMUSG00000000374
ENSMUSG00000000282	ENSMUSG00000000378
ENSMUSG00000000295	ENSMUSG00000000399
ENSMUSG00000000296	ENSMUSG00000000416
ENSMUSG00000000305	ENSMUSG00000000420
ENSMUSG00000000308	ENSMUSG00000000439
ENSMUSG00000000325	ENSMUSG00000000441
ENSMUSG00000000326	ENSMUSG00000000531
ENSMUSG00000000339	ENSMUSG00000000532
ENSMUSG00000000340	ENSMUSG00000000538
ENSMUSG00000000346	ENSMUSG00000000552
ENSMUSG00000000355	ENSMUSG00000000560
ENSMUSG00000000374	ENSMUSG00000000561
ENSMUSG00000000378	ENSMUSG00000000563
ENSMUSG00000000384	ENSMUSG00000000568
ENSMUSG00000000399	ENSMUSG00000000594
ENSMUSG00000000416	ENSMUSG00000000600
ENSMUSG00000000420	ENSMUSG00000000605
ENSMUSG00000000439	ENSMUSG00000000627
ENSMUSG00000000441	ENSMUSG00000000631
ENSMUSG00000000489	ENSMUSG00000000632
ENSMUSG00000000530	ENSMUSG00000000711
ENSMUSG00000000531	ENSMUSG00000000738
ENSMUSG00000000532	ENSMUSG00000000740
ENSMUSG00000000538	ENSMUSG00000000743
ENSMUSG00000000552	ENSMUSG00000000751
ENSMUSG00000000560	ENSMUSG00000000759
ENSMUSG00000000561	ENSMUSG00000000787
ENSMUSG00000000563	ENSMUSG00000000794
ENSMUSG00000000567	ENSMUSG00000000804
ENSMUSG00000000568	ENSMUSG00000000823
ENSMUSG00000000579	ENSMUSG00000000826
ENSMUSG00000000581	ENSMUSG00000000827
ENSMUSG00000000594	ENSMUSG00000000838

ENSMUSG00000000600	ENSMUSG00000000861
ENSMUSG00000000605	ENSMUSG00000000881
ENSMUSG00000000627	ENSMUSG00000000902
ENSMUSG00000000631	ENSMUSG00000000915
ENSMUSG00000000632	ENSMUSG00000000948
ENSMUSG00000000693	ENSMUSG00000000959
ENSMUSG00000000708	ENSMUSG00000001016
ENSMUSG00000000711	ENSMUSG00000001017
ENSMUSG00000000738	ENSMUSG00000001036
ENSMUSG00000000740	ENSMUSG00000001052
ENSMUSG00000000743	ENSMUSG00000001054
ENSMUSG00000000751	ENSMUSG00000001056
ENSMUSG00000000759	ENSMUSG00000001062
ENSMUSG00000000766	ENSMUSG00000001089
ENSMUSG00000000776	ENSMUSG00000001098
ENSMUSG00000000787	ENSMUSG00000001100
ENSMUSG00000000794	ENSMUSG00000001119
ENSMUSG00000000804	ENSMUSG00000001120
ENSMUSG00000000805	ENSMUSG00000001127
ENSMUSG00000000823	ENSMUSG00000001138
ENSMUSG00000000826	ENSMUSG00000001143
ENSMUSG00000000827	ENSMUSG00000001150
ENSMUSG00000000838	ENSMUSG00000001151
ENSMUSG00000000861	ENSMUSG00000001156
ENSMUSG00000000876	ENSMUSG00000001173
ENSMUSG00000000881	ENSMUSG00000001175
ENSMUSG00000000902	ENSMUSG00000001211
ENSMUSG00000000915	ENSMUSG00000001227
ENSMUSG00000000916	ENSMUSG00000001229
ENSMUSG00000000934	ENSMUSG00000001248
ENSMUSG00000000948	ENSMUSG00000001260
ENSMUSG00000000957	ENSMUSG00000001270
ENSMUSG00000000959	ENSMUSG00000001280
ENSMUSG00000000976	ENSMUSG00000001289
ENSMUSG00000001016	ENSMUSG00000001300
ENSMUSG00000001017	ENSMUSG00000001313
ENSMUSG00000001018	ENSMUSG00000001323
ENSMUSG00000001025	ENSMUSG00000001334
ENSMUSG00000001034	ENSMUSG00000001366
ENSMUSG00000001036	ENSMUSG00000001376
ENSMUSG00000001039	ENSMUSG00000001380
ENSMUSG00000001052	ENSMUSG00000001383
ENSMUSG00000001053	ENSMUSG00000001415
ENSMUSG00000001054	ENSMUSG00000001416
ENSMUSG00000001056	ENSMUSG00000001419
ENSMUSG00000001062	ENSMUSG00000001424
ENSMUSG00000001065	ENSMUSG00000001440
ENSMUSG00000001082	ENSMUSG00000001441
ENSMUSG00000001089	ENSMUSG00000001445
ENSMUSG00000001098	ENSMUSG00000001467
ENSMUSG00000001100	ENSMUSG00000001472
ENSMUSG00000001105	ENSMUSG00000001482
ENSMUSG00000001119	ENSMUSG00000001518

ENSMUSG00000001120	ENSMUSG00000001525
ENSMUSG00000001127	ENSMUSG00000001552
ENSMUSG00000001138	ENSMUSG00000001569
ENSMUSG00000001143	ENSMUSG00000001576
ENSMUSG00000001150	ENSMUSG00000001632
ENSMUSG00000001151	ENSMUSG00000001687
ENSMUSG00000001156	ENSMUSG00000001729
ENSMUSG00000001157	ENSMUSG00000001768
ENSMUSG00000001158	ENSMUSG00000001774
ENSMUSG00000001173	ENSMUSG00000001783
ENSMUSG00000001175	ENSMUSG00000001794
ENSMUSG00000001211	ENSMUSG00000001802
ENSMUSG00000001227	ENSMUSG00000001829
ENSMUSG00000001229	ENSMUSG00000001833
ENSMUSG00000001240	ENSMUSG00000001847
ENSMUSG00000001247	ENSMUSG00000001855
ENSMUSG00000001248	ENSMUSG00000001891
ENSMUSG00000001260	ENSMUSG00000001909
ENSMUSG00000001270	ENSMUSG00000001910
ENSMUSG00000001280	ENSMUSG00000001911
ENSMUSG00000001285	ENSMUSG00000001924
ENSMUSG00000001288	ENSMUSG00000001985
ENSMUSG00000001289	ENSMUSG00000001986
ENSMUSG00000001300	ENSMUSG00000001995
ENSMUSG00000001305	ENSMUSG00000002006
ENSMUSG00000001313	ENSMUSG00000002010
ENSMUSG00000001323	ENSMUSG00000002012
ENSMUSG00000001334	ENSMUSG00000002015
ENSMUSG00000001366	ENSMUSG00000002028
ENSMUSG00000001376	ENSMUSG00000002052
ENSMUSG00000001380	ENSMUSG00000002064
ENSMUSG00000001383	ENSMUSG00000002102
ENSMUSG00000001415	ENSMUSG00000002103
ENSMUSG00000001416	ENSMUSG00000002107
ENSMUSG00000001418	ENSMUSG00000002129
ENSMUSG00000001419	ENSMUSG00000002221
ENSMUSG00000001424	ENSMUSG00000002222
ENSMUSG00000001435	ENSMUSG00000002265
ENSMUSG00000001436	ENSMUSG00000002274
ENSMUSG00000001440	ENSMUSG00000002319
ENSMUSG00000001441	ENSMUSG00000002320
ENSMUSG00000001445	ENSMUSG00000002332
ENSMUSG00000001467	ENSMUSG00000002341
ENSMUSG00000001472	ENSMUSG00000002346
ENSMUSG00000001482	ENSMUSG00000002372
ENSMUSG00000001496	ENSMUSG00000002379
ENSMUSG00000001506	ENSMUSG00000002393
ENSMUSG00000001507	ENSMUSG00000002395
ENSMUSG00000001518	ENSMUSG00000002409
ENSMUSG00000001520	ENSMUSG00000002413
ENSMUSG00000001521	ENSMUSG00000002416
ENSMUSG00000001524	ENSMUSG00000002455
ENSMUSG00000001525	ENSMUSG00000002459

ENSMUSG00000001542	ENSMUSG00000002475
ENSMUSG00000001552	ENSMUSG00000002489
ENSMUSG00000001555	ENSMUSG00000002496
ENSMUSG00000001569	ENSMUSG00000002504
ENSMUSG00000001576	ENSMUSG00000002524
ENSMUSG00000001627	ENSMUSG00000002546
ENSMUSG00000001630	ENSMUSG00000002550
ENSMUSG00000001632	ENSMUSG00000002580
ENSMUSG00000001642	ENSMUSG00000002602
ENSMUSG00000001666	ENSMUSG00000002608
ENSMUSG00000001674	ENSMUSG00000002625
ENSMUSG00000001687	ENSMUSG00000002658
ENSMUSG00000001700	ENSMUSG00000002660
ENSMUSG00000001707	ENSMUSG00000002718
ENSMUSG00000001729	ENSMUSG00000002741
ENSMUSG00000001750	ENSMUSG00000002748
ENSMUSG00000001751	ENSMUSG00000002763
ENSMUSG00000001755	ENSMUSG00000002767
ENSMUSG00000001761	ENSMUSG00000002768
ENSMUSG00000001763	ENSMUSG00000002778
ENSMUSG00000001767	ENSMUSG00000002799
ENSMUSG00000001768	ENSMUSG00000002803
ENSMUSG00000001773	ENSMUSG00000002808
ENSMUSG00000001774	ENSMUSG00000002812
ENSMUSG00000001783	ENSMUSG00000002820
ENSMUSG00000001785	ENSMUSG00000002833
ENSMUSG00000001786	ENSMUSG00000002844
ENSMUSG00000001794	ENSMUSG00000002900
ENSMUSG00000001802	ENSMUSG00000002948
ENSMUSG00000001829	ENSMUSG00000002949
ENSMUSG00000001833	ENSMUSG00000002957
ENSMUSG00000001844	ENSMUSG00000002968
ENSMUSG00000001847	ENSMUSG00000002981
ENSMUSG00000001855	ENSMUSG00000002984
ENSMUSG00000001864	ENSMUSG00000002985
ENSMUSG00000001870	ENSMUSG00000002997
ENSMUSG00000001891	ENSMUSG00000003031
ENSMUSG00000001901	ENSMUSG00000003033
ENSMUSG00000001909	ENSMUSG00000003037
ENSMUSG00000001910	ENSMUSG00000003038
ENSMUSG00000001911	ENSMUSG00000003039
ENSMUSG00000001924	ENSMUSG00000003062
ENSMUSG00000001930	ENSMUSG00000003068
ENSMUSG00000001942	ENSMUSG00000003072
ENSMUSG00000001946	ENSMUSG00000003099
ENSMUSG00000001962	ENSMUSG00000003119
ENSMUSG00000001964	ENSMUSG00000003131
ENSMUSG00000001983	ENSMUSG00000003134
ENSMUSG00000001985	ENSMUSG00000003153
ENSMUSG00000001986	ENSMUSG00000003154
ENSMUSG00000001995	ENSMUSG00000003166
ENSMUSG00000001998	ENSMUSG00000003199
ENSMUSG00000001999	ENSMUSG00000003200

ENSMUSG00000002006	ENSMUSG00000003226
ENSMUSG00000002010	ENSMUSG00000003228
ENSMUSG00000002012	ENSMUSG00000003233
ENSMUSG00000002014	ENSMUSG00000003234
ENSMUSG00000002015	ENSMUSG00000003235
ENSMUSG00000002017	ENSMUSG00000003269
ENSMUSG00000002028	ENSMUSG00000003273
ENSMUSG00000002031	ENSMUSG00000003279
ENSMUSG00000002032	ENSMUSG00000003299
ENSMUSG00000002043	ENSMUSG00000003308
ENSMUSG00000002052	ENSMUSG00000003316
ENSMUSG00000002058	ENSMUSG00000003344
ENSMUSG00000002059	ENSMUSG00000003345
ENSMUSG00000002064	ENSMUSG00000003346
ENSMUSG00000002083	ENSMUSG00000003352
ENSMUSG00000002102	ENSMUSG00000003360
ENSMUSG00000002103	ENSMUSG00000003363
ENSMUSG00000002105	ENSMUSG00000003378
ENSMUSG00000002107	ENSMUSG00000003380
ENSMUSG00000002109	ENSMUSG00000003382
ENSMUSG00000002129	ENSMUSG00000003402
ENSMUSG00000002205	ENSMUSG00000003410
ENSMUSG00000002210	ENSMUSG00000003421
ENSMUSG00000002221	ENSMUSG00000003429
ENSMUSG00000002222	ENSMUSG00000003435
ENSMUSG00000002227	ENSMUSG00000003437
ENSMUSG00000002233	ENSMUSG00000003452
ENSMUSG00000002250	ENSMUSG00000003458
ENSMUSG00000002265	ENSMUSG00000003464
ENSMUSG00000002266	ENSMUSG00000003469
ENSMUSG00000002274	ENSMUSG00000003500
ENSMUSG00000002279	ENSMUSG00000003518
ENSMUSG00000002280	ENSMUSG00000003528
ENSMUSG00000002289	ENSMUSG00000003531
ENSMUSG00000002307	ENSMUSG00000003534
ENSMUSG00000002308	ENSMUSG00000003575
ENSMUSG00000002319	ENSMUSG00000003660
ENSMUSG00000002320	ENSMUSG00000003662
ENSMUSG00000002325	ENSMUSG00000003721
ENSMUSG00000002326	ENSMUSG00000003731
ENSMUSG00000002329	ENSMUSG00000003746
ENSMUSG00000002332	ENSMUSG00000003778
ENSMUSG00000002341	ENSMUSG00000003808
ENSMUSG00000002342	ENSMUSG00000003810
ENSMUSG00000002343	ENSMUSG00000003813
ENSMUSG00000002345	ENSMUSG00000003814
ENSMUSG00000002346	ENSMUSG00000003847
ENSMUSG00000002365	ENSMUSG00000003863
ENSMUSG00000002372	ENSMUSG00000003872
ENSMUSG00000002379	ENSMUSG00000003923
ENSMUSG00000002393	ENSMUSG00000003934
ENSMUSG00000002395	ENSMUSG00000003948
ENSMUSG00000002396	ENSMUSG00000003949

ENSMUSG00000002409	ENSMUSG00000003970
ENSMUSG00000002413	ENSMUSG00000003974
ENSMUSG00000002416	ENSMUSG00000003992
ENSMUSG00000002428	ENSMUSG00000004031
ENSMUSG00000002455	ENSMUSG00000004032
ENSMUSG00000002458	ENSMUSG00000004040
ENSMUSG00000002459	ENSMUSG00000004054
ENSMUSG00000002475	ENSMUSG00000004056
ENSMUSG00000002477	ENSMUSG00000004069
ENSMUSG00000002486	ENSMUSG00000004070
ENSMUSG00000002489	ENSMUSG00000004071
ENSMUSG00000002496	ENSMUSG00000004096
ENSMUSG00000002504	ENSMUSG00000004099
ENSMUSG00000002524	ENSMUSG00000004110
ENSMUSG00000002546	ENSMUSG00000004113
ENSMUSG00000002550	ENSMUSG00000004151
ENSMUSG00000002578	ENSMUSG00000004187
ENSMUSG00000002580	ENSMUSG00000004207
ENSMUSG00000002602	ENSMUSG00000004221
ENSMUSG00000002603	ENSMUSG00000004263
ENSMUSG00000002608	ENSMUSG00000004264
ENSMUSG00000002617	ENSMUSG00000004267
ENSMUSG00000002625	ENSMUSG00000004285
ENSMUSG00000002635	ENSMUSG00000004319
ENSMUSG00000002658	ENSMUSG00000004347
ENSMUSG00000002660	ENSMUSG00000004356
ENSMUSG00000002661	ENSMUSG00000004360
ENSMUSG00000002679	ENSMUSG00000004364
ENSMUSG00000002688	ENSMUSG00000004366
ENSMUSG00000002718	ENSMUSG00000004383
ENSMUSG00000002728	ENSMUSG00000004393
ENSMUSG00000002731	ENSMUSG00000004394
ENSMUSG00000002733	ENSMUSG00000004455
ENSMUSG00000002741	ENSMUSG00000004460
ENSMUSG00000002748	ENSMUSG00000004508
ENSMUSG00000002763	ENSMUSG00000004530
ENSMUSG00000002767	ENSMUSG00000004535
ENSMUSG00000002768	ENSMUSG00000004558
ENSMUSG00000002771	ENSMUSG00000004562
ENSMUSG00000002778	ENSMUSG00000004565
ENSMUSG00000002781	ENSMUSG00000004567
ENSMUSG00000002797	ENSMUSG00000004568
ENSMUSG00000002799	ENSMUSG00000004591
ENSMUSG00000002803	ENSMUSG00000004610
ENSMUSG00000002804	ENSMUSG00000004633
ENSMUSG00000002808	ENSMUSG00000004667
ENSMUSG00000002812	ENSMUSG00000004677
ENSMUSG00000002814	ENSMUSG00000004748
ENSMUSG00000002820	ENSMUSG00000004768
ENSMUSG00000002825	ENSMUSG00000004771
ENSMUSG00000002833	ENSMUSG00000004788
ENSMUSG00000002835	ENSMUSG00000004789
ENSMUSG00000002844	ENSMUSG00000004798

ENSMUSG00000002845	ENSMUSG00000004815
ENSMUSG00000002846	ENSMUSG00000004843
ENSMUSG00000002870	ENSMUSG00000004846
ENSMUSG00000002871	ENSMUSG00000004849
ENSMUSG00000002881	ENSMUSG00000004865
ENSMUSG00000002885	ENSMUSG00000004892
ENSMUSG00000002900	ENSMUSG00000004895
ENSMUSG00000002908	ENSMUSG00000004897
ENSMUSG00000002910	ENSMUSG00000004929
ENSMUSG00000002948	ENSMUSG00000004933
ENSMUSG00000002949	ENSMUSG00000004936
ENSMUSG00000002957	ENSMUSG00000004937
ENSMUSG00000002963	ENSMUSG00000004945
ENSMUSG00000002968	ENSMUSG00000004961
ENSMUSG00000002980	ENSMUSG00000004980
ENSMUSG00000002981	ENSMUSG00000005034
ENSMUSG00000002983	ENSMUSG00000005045
ENSMUSG00000002984	ENSMUSG00000005069
ENSMUSG00000002985	ENSMUSG00000005089
ENSMUSG00000002996	ENSMUSG00000005102
ENSMUSG00000002997	ENSMUSG00000005103
ENSMUSG00000003031	ENSMUSG00000005125
ENSMUSG00000003033	ENSMUSG00000005142
ENSMUSG00000003037	ENSMUSG00000005148
ENSMUSG00000003038	ENSMUSG00000005161
ENSMUSG00000003039	ENSMUSG00000005198
ENSMUSG00000003062	ENSMUSG00000005204
ENSMUSG00000003068	ENSMUSG00000005262
ENSMUSG00000003070	ENSMUSG00000005299
ENSMUSG00000003072	ENSMUSG00000005312
ENSMUSG00000003099	ENSMUSG00000005338
ENSMUSG00000003119	ENSMUSG00000005354
ENSMUSG00000003123	ENSMUSG00000005360
ENSMUSG00000003131	ENSMUSG00000005362
ENSMUSG00000003134	ENSMUSG00000005371
ENSMUSG00000003135	ENSMUSG00000005374
ENSMUSG00000003153	ENSMUSG00000005417
ENSMUSG00000003154	ENSMUSG00000005442
ENSMUSG00000003161	ENSMUSG00000005469
ENSMUSG00000003166	ENSMUSG00000005483
ENSMUSG00000003184	ENSMUSG00000005505
ENSMUSG00000003199	ENSMUSG00000005506
ENSMUSG00000003200	ENSMUSG00000005510
ENSMUSG00000003208	ENSMUSG00000005514
ENSMUSG00000003226	ENSMUSG00000005533
ENSMUSG00000003228	ENSMUSG00000005534
ENSMUSG00000003233	ENSMUSG00000005566
ENSMUSG00000003234	ENSMUSG00000005575
ENSMUSG00000003235	ENSMUSG00000005580
ENSMUSG00000003269	ENSMUSG00000005583
ENSMUSG00000003273	ENSMUSG00000005609
ENSMUSG00000003279	ENSMUSG00000005610
ENSMUSG00000003299	ENSMUSG00000005615

ENSMUSG00000003308	ENSMUSG00000005621
ENSMUSG00000003316	ENSMUSG00000005625
ENSMUSG00000003344	ENSMUSG00000005672
ENSMUSG00000003345	ENSMUSG00000005674
ENSMUSG00000003346	ENSMUSG00000005683
ENSMUSG00000003348	ENSMUSG00000005686
ENSMUSG00000003352	ENSMUSG00000005687
ENSMUSG00000003354	ENSMUSG00000005698
ENSMUSG00000003360	ENSMUSG00000005732
ENSMUSG00000003363	ENSMUSG00000005779
ENSMUSG00000003378	ENSMUSG00000005802
ENSMUSG00000003380	ENSMUSG00000005804
ENSMUSG00000003382	ENSMUSG00000005813
ENSMUSG00000003402	ENSMUSG00000005846
ENSMUSG00000003410	ENSMUSG00000005871
ENSMUSG00000003411	ENSMUSG00000005873
ENSMUSG00000003418	ENSMUSG00000005881
ENSMUSG00000003420	ENSMUSG00000005882
ENSMUSG00000003421	ENSMUSG00000005886
ENSMUSG00000003423	ENSMUSG00000005893
ENSMUSG00000003429	ENSMUSG00000005899
ENSMUSG00000003435	ENSMUSG00000005936
ENSMUSG00000003437	ENSMUSG00000005973
ENSMUSG00000003438	ENSMUSG00000005981
ENSMUSG00000003444	ENSMUSG00000005982
ENSMUSG00000003452	ENSMUSG00000005986
ENSMUSG00000003458	ENSMUSG00000006005
ENSMUSG00000003464	ENSMUSG00000006010
ENSMUSG00000003469	ENSMUSG00000006021
ENSMUSG00000003476	ENSMUSG00000006024
ENSMUSG00000003500	ENSMUSG00000006057
ENSMUSG00000003518	ENSMUSG00000006058
ENSMUSG00000003526	ENSMUSG00000006095
ENSMUSG00000003527	ENSMUSG00000006134
ENSMUSG00000003528	ENSMUSG00000006169
ENSMUSG00000003531	ENSMUSG00000006205
ENSMUSG00000003534	ENSMUSG00000006215
ENSMUSG00000003545	ENSMUSG00000006273
ENSMUSG00000003546	ENSMUSG00000006276
ENSMUSG00000003549	ENSMUSG00000006288
ENSMUSG00000003559	ENSMUSG00000006299
ENSMUSG00000003573	ENSMUSG00000006304
ENSMUSG00000003575	ENSMUSG00000006307
ENSMUSG00000003581	ENSMUSG00000006315
ENSMUSG00000003585	ENSMUSG00000006333
ENSMUSG00000003617	ENSMUSG00000006356
ENSMUSG00000003623	ENSMUSG00000006362
ENSMUSG00000003644	ENSMUSG00000006373
ENSMUSG00000003657	ENSMUSG00000006412
ENSMUSG00000003660	ENSMUSG00000006418
ENSMUSG00000003662	ENSMUSG00000006423
ENSMUSG00000003680	ENSMUSG00000006435
ENSMUSG00000003721	ENSMUSG00000006442

ENSMUSG00000003731	ENSMUSG00000006456
ENSMUSG00000003746	ENSMUSG00000006464
ENSMUSG00000003752	ENSMUSG00000006476
ENSMUSG00000003762	ENSMUSG00000006494
ENSMUSG00000003778	ENSMUSG00000006522
ENSMUSG00000003808	ENSMUSG00000006527
ENSMUSG00000003809	ENSMUSG00000006575
ENSMUSG00000003810	ENSMUSG00000006576
ENSMUSG00000003812	ENSMUSG00000006586
ENSMUSG00000003813	ENSMUSG00000006599
ENSMUSG00000003814	ENSMUSG00000006651
ENSMUSG00000003847	ENSMUSG00000006673
ENSMUSG00000003848	ENSMUSG00000006675
ENSMUSG00000003863	ENSMUSG00000006676
ENSMUSG00000003865	ENSMUSG00000006699
ENSMUSG00000003868	ENSMUSG00000006711
ENSMUSG00000003872	ENSMUSG00000006728
ENSMUSG00000003873	ENSMUSG00000006731
ENSMUSG00000003923	ENSMUSG00000006736
ENSMUSG00000003929	ENSMUSG00000006740
ENSMUSG00000003934	ENSMUSG00000006782
ENSMUSG00000003948	ENSMUSG00000006800
ENSMUSG00000003949	ENSMUSG00000006818
ENSMUSG00000003955	ENSMUSG00000006920
ENSMUSG00000003970	ENSMUSG00000006930
ENSMUSG00000003974	ENSMUSG00000006932
ENSMUSG00000003992	ENSMUSG00000006941
ENSMUSG00000004018	ENSMUSG00000006998
ENSMUSG00000004031	ENSMUSG00000007021
ENSMUSG00000004032	ENSMUSG00000007029
ENSMUSG00000004035	ENSMUSG00000007036
ENSMUSG00000004040	ENSMUSG00000007038
ENSMUSG00000004043	ENSMUSG00000007097
ENSMUSG00000004044	ENSMUSG00000007207
ENSMUSG00000004054	ENSMUSG00000007338
ENSMUSG00000004056	ENSMUSG00000007411
ENSMUSG00000004069	ENSMUSG00000007415
ENSMUSG00000004070	ENSMUSG00000007458
ENSMUSG00000004071	ENSMUSG00000007476
ENSMUSG00000004085	ENSMUSG00000007564
ENSMUSG00000004096	ENSMUSG00000007594
ENSMUSG00000004098	ENSMUSG00000007603
ENSMUSG00000004099	ENSMUSG00000007617
ENSMUSG00000004100	ENSMUSG00000007653
ENSMUSG00000004110	ENSMUSG00000007656
ENSMUSG00000004113	ENSMUSG00000007659
ENSMUSG00000004127	ENSMUSG00000007670
ENSMUSG00000004151	ENSMUSG00000007682
ENSMUSG00000004187	ENSMUSG00000007721
ENSMUSG00000004207	ENSMUSG00000007739
ENSMUSG00000004221	ENSMUSG00000007783
ENSMUSG00000004233	ENSMUSG00000007815
ENSMUSG00000004263	ENSMUSG00000007817

ENSMUSG00000004264	ENSMUSG00000007836
ENSMUSG00000004267	ENSMUSG00000007850
ENSMUSG00000004268	ENSMUSG00000007880
ENSMUSG00000004270	ENSMUSG00000007891
ENSMUSG00000004285	ENSMUSG00000007892
ENSMUSG00000004317	ENSMUSG00000007944
ENSMUSG00000004319	ENSMUSG00000007950
ENSMUSG00000004347	ENSMUSG00000007987
ENSMUSG00000004356	ENSMUSG00000007989
ENSMUSG00000004360	ENSMUSG00000008035
ENSMUSG00000004364	ENSMUSG00000008036
ENSMUSG00000004366	ENSMUSG00000008140
ENSMUSG00000004383	ENSMUSG00000008153
ENSMUSG00000004393	ENSMUSG00000008200
ENSMUSG00000004394	ENSMUSG00000008206
ENSMUSG00000004446	ENSMUSG00000008301
ENSMUSG00000004451	ENSMUSG00000008305
ENSMUSG00000004455	ENSMUSG00000008348
ENSMUSG00000004460	ENSMUSG00000008373
ENSMUSG00000004462	ENSMUSG00000008393
ENSMUSG00000004500	ENSMUSG00000008429
ENSMUSG00000004508	ENSMUSG00000008475
ENSMUSG00000004530	ENSMUSG00000008489
ENSMUSG00000004535	ENSMUSG00000008575
ENSMUSG00000004558	ENSMUSG00000008604
ENSMUSG00000004561	ENSMUSG00000008658
ENSMUSG00000004562	ENSMUSG00000008668
ENSMUSG00000004565	ENSMUSG00000008682
ENSMUSG00000004567	ENSMUSG00000008683
ENSMUSG00000004568	ENSMUSG00000008690
ENSMUSG00000004591	ENSMUSG00000008730
ENSMUSG00000004610	ENSMUSG00000008734
ENSMUSG00000004626	ENSMUSG00000008763
ENSMUSG00000004631	ENSMUSG00000008855
ENSMUSG00000004633	ENSMUSG00000008859
ENSMUSG00000004637	ENSMUSG00000008892
ENSMUSG00000004642	ENSMUSG00000009013
ENSMUSG00000004661	ENSMUSG00000009035
ENSMUSG00000004667	ENSMUSG00000009073
ENSMUSG00000004677	ENSMUSG00000009079
ENSMUSG00000004698	ENSMUSG00000009090
ENSMUSG00000004748	ENSMUSG00000009112
ENSMUSG00000004768	ENSMUSG00000009207
ENSMUSG00000004771	ENSMUSG00000009216
ENSMUSG00000004788	ENSMUSG00000009291
ENSMUSG00000004789	ENSMUSG00000009292
ENSMUSG00000004798	ENSMUSG00000009293
ENSMUSG00000004815	ENSMUSG00000009394
ENSMUSG00000004843	ENSMUSG00000009406
ENSMUSG00000004846	ENSMUSG00000009418
ENSMUSG00000004849	ENSMUSG00000009470
ENSMUSG00000004865	ENSMUSG00000009535
ENSMUSG00000004880	ENSMUSG00000009549

ENSMUSG00000004891	ENSMUSG00000009555
ENSMUSG00000004892	ENSMUSG00000009569
ENSMUSG00000004894	ENSMUSG00000009575
ENSMUSG00000004895	ENSMUSG00000009630
ENSMUSG00000004896	ENSMUSG00000009647
ENSMUSG00000004897	ENSMUSG00000009681
ENSMUSG00000004902	ENSMUSG00000009739
ENSMUSG00000004929	ENSMUSG00000009741
ENSMUSG00000004931	ENSMUSG00000009863
ENSMUSG00000004933	ENSMUSG00000009894
ENSMUSG00000004934	ENSMUSG00000009905
ENSMUSG00000004936	ENSMUSG00000009907
ENSMUSG00000004937	ENSMUSG00000009927
ENSMUSG00000004945	ENSMUSG00000009995
ENSMUSG00000004947	ENSMUSG00000010025
ENSMUSG00000004952	ENSMUSG00000010045
ENSMUSG00000004961	ENSMUSG00000010054
ENSMUSG00000004980	ENSMUSG00000010064
ENSMUSG00000004994	ENSMUSG00000010066
ENSMUSG00000004996	ENSMUSG00000010086
ENSMUSG00000005034	ENSMUSG00000010095
ENSMUSG00000005043	ENSMUSG00000010097
ENSMUSG00000005045	ENSMUSG00000010110
ENSMUSG00000005054	ENSMUSG00000010205
ENSMUSG00000005057	ENSMUSG00000010277
ENSMUSG00000005069	ENSMUSG00000010376
ENSMUSG00000005078	ENSMUSG00000010392
ENSMUSG00000005089	ENSMUSG00000010453
ENSMUSG00000005102	ENSMUSG00000010517
ENSMUSG00000005103	ENSMUSG00000010554
ENSMUSG00000005125	ENSMUSG00000010608
ENSMUSG00000005142	ENSMUSG00000010663
ENSMUSG00000005148	ENSMUSG00000010721
ENSMUSG00000005150	ENSMUSG00000010803
ENSMUSG00000005161	ENSMUSG00000010914
ENSMUSG00000005198	ENSMUSG00000010936
ENSMUSG00000005204	ENSMUSG00000011096
ENSMUSG00000005225	ENSMUSG00000011114
ENSMUSG00000005237	ENSMUSG00000011158
ENSMUSG00000005251	ENSMUSG00000011179
ENSMUSG00000005262	ENSMUSG00000011257
ENSMUSG00000005267	ENSMUSG00000011306
ENSMUSG00000005268	ENSMUSG00000011751
ENSMUSG00000005299	ENSMUSG00000011752
ENSMUSG00000005312	ENSMUSG00000011831
ENSMUSG00000005338	ENSMUSG00000011832
ENSMUSG00000005354	ENSMUSG00000011877
ENSMUSG00000005357	ENSMUSG00000011960
ENSMUSG00000005360	ENSMUSG00000012076
ENSMUSG00000005362	ENSMUSG00000012114
ENSMUSG00000005370	ENSMUSG00000012117
ENSMUSG00000005371	ENSMUSG00000012405
ENSMUSG00000005374	ENSMUSG00000012422

ENSMUSG00000005378	ENSMUSG00000012535
ENSMUSG00000005397	ENSMUSG00000012609
ENSMUSG00000005410	ENSMUSG00000012848
ENSMUSG00000005417	ENSMUSG00000013033
ENSMUSG00000005442	ENSMUSG00000013076
ENSMUSG00000005447	ENSMUSG00000013089
ENSMUSG00000005469	ENSMUSG00000013160
ENSMUSG00000005481	ENSMUSG00000013236
ENSMUSG00000005483	ENSMUSG00000013275
ENSMUSG00000005501	ENSMUSG00000013419
ENSMUSG00000005505	ENSMUSG00000013465
ENSMUSG00000005506	ENSMUSG00000013523
ENSMUSG00000005510	ENSMUSG00000013539
ENSMUSG00000005514	ENSMUSG00000013593
ENSMUSG00000005533	ENSMUSG00000013622
ENSMUSG00000005534	ENSMUSG00000013629
ENSMUSG00000005566	ENSMUSG00000013646
ENSMUSG00000005575	ENSMUSG00000013662
ENSMUSG00000005580	ENSMUSG00000013663
ENSMUSG00000005583	ENSMUSG00000013698
ENSMUSG00000005609	ENSMUSG00000013701
ENSMUSG00000005610	ENSMUSG00000013787
ENSMUSG00000005615	ENSMUSG00000013833
ENSMUSG00000005621	ENSMUSG00000013846
ENSMUSG00000005625	ENSMUSG00000013858
ENSMUSG00000005656	ENSMUSG00000013921
ENSMUSG00000005667	ENSMUSG00000014074
ENSMUSG00000005672	ENSMUSG00000014077
ENSMUSG00000005674	ENSMUSG00000014164
ENSMUSG00000005682	ENSMUSG00000014195
ENSMUSG00000005683	ENSMUSG00000014226
ENSMUSG00000005686	ENSMUSG00000014294
ENSMUSG00000005687	ENSMUSG00000014313
ENSMUSG00000005698	ENSMUSG00000014349
ENSMUSG00000005699	ENSMUSG00000014353
ENSMUSG00000005718	ENSMUSG00000014355
ENSMUSG00000005732	ENSMUSG00000014361
ENSMUSG00000005774	ENSMUSG00000014402
ENSMUSG00000005779	ENSMUSG00000014426
ENSMUSG00000005802	ENSMUSG00000014470
ENSMUSG00000005803	ENSMUSG00000014496
ENSMUSG00000005804	ENSMUSG00000014498
ENSMUSG00000005813	ENSMUSG00000014550
ENSMUSG00000005823	ENSMUSG00000014551
ENSMUSG00000005846	ENSMUSG00000014592
ENSMUSG00000005871	ENSMUSG00000014601
ENSMUSG00000005873	ENSMUSG00000014602
ENSMUSG00000005881	ENSMUSG00000014606
ENSMUSG00000005882	ENSMUSG00000014748
ENSMUSG00000005886	ENSMUSG00000014763
ENSMUSG00000005893	ENSMUSG00000014769
ENSMUSG00000005897	ENSMUSG00000014786
ENSMUSG00000005899	ENSMUSG00000014850

ENSMUSG00000005907	ENSMUSG00000014867
ENSMUSG00000005936	ENSMUSG00000014956
ENSMUSG00000005949	ENSMUSG00000014959
ENSMUSG00000005958	ENSMUSG00000015002
ENSMUSG00000005973	ENSMUSG00000015013
ENSMUSG00000005981	ENSMUSG00000015087
ENSMUSG00000005982	ENSMUSG00000015090
ENSMUSG00000005986	ENSMUSG00000015092
ENSMUSG00000006005	ENSMUSG00000015094
ENSMUSG00000006010	ENSMUSG00000015095
ENSMUSG00000006019	ENSMUSG00000015120
ENSMUSG00000006021	ENSMUSG00000015127
ENSMUSG00000006024	ENSMUSG00000015143
ENSMUSG00000006050	ENSMUSG00000015149
ENSMUSG00000006057	ENSMUSG00000015165
ENSMUSG00000006058	ENSMUSG00000015176
ENSMUSG00000006095	ENSMUSG00000015189
ENSMUSG00000006127	ENSMUSG00000015214
ENSMUSG00000006134	ENSMUSG00000015222
ENSMUSG00000006169	ENSMUSG00000015243
ENSMUSG00000006191	ENSMUSG00000015290
ENSMUSG00000006205	ENSMUSG00000015291
ENSMUSG00000006215	ENSMUSG00000015305
ENSMUSG00000006235	ENSMUSG00000015341
ENSMUSG00000006262	ENSMUSG00000015357
ENSMUSG00000006270	ENSMUSG00000015377
ENSMUSG00000006273	ENSMUSG00000015461
ENSMUSG00000006276	ENSMUSG00000015476
ENSMUSG00000006281	ENSMUSG00000015478
ENSMUSG00000006288	ENSMUSG00000015488
ENSMUSG00000006289	ENSMUSG00000015501
ENSMUSG00000006299	ENSMUSG00000015536
ENSMUSG00000006301	ENSMUSG00000015568
ENSMUSG00000006304	ENSMUSG00000015597
ENSMUSG00000006307	ENSMUSG00000015599
ENSMUSG00000006315	ENSMUSG00000015605
ENSMUSG00000006333	ENSMUSG00000015656
ENSMUSG00000006335	ENSMUSG00000015671
ENSMUSG00000006342	ENSMUSG00000015697
ENSMUSG00000006356	ENSMUSG00000015709
ENSMUSG00000006362	ENSMUSG00000015714
ENSMUSG00000006369	ENSMUSG00000015733
ENSMUSG00000006373	ENSMUSG00000015747
ENSMUSG00000006386	ENSMUSG00000015749
ENSMUSG00000006390	ENSMUSG00000015750
ENSMUSG00000006392	ENSMUSG00000015755
ENSMUSG00000006395	ENSMUSG00000015757
ENSMUSG00000006403	ENSMUSG00000015759
ENSMUSG00000006412	ENSMUSG00000015766
ENSMUSG00000006418	ENSMUSG00000015776
ENSMUSG00000006423	ENSMUSG00000015790
ENSMUSG00000006435	ENSMUSG00000015804
ENSMUSG00000006442	ENSMUSG00000015806

ENSMUSG00000006456	ENSMUSG00000015829
ENSMUSG00000006463	ENSMUSG00000015837
ENSMUSG00000006464	ENSMUSG00000015843
ENSMUSG00000006471	ENSMUSG00000015846
ENSMUSG00000006476	ENSMUSG00000015879
ENSMUSG00000006494	ENSMUSG00000015932
ENSMUSG00000006498	ENSMUSG00000015937
ENSMUSG00000006517	ENSMUSG00000015944
ENSMUSG00000006519	ENSMUSG00000015961
ENSMUSG00000006522	ENSMUSG00000015968
ENSMUSG00000006526	ENSMUSG00000015971
ENSMUSG00000006527	ENSMUSG00000015981
ENSMUSG00000006567	ENSMUSG00000015994
ENSMUSG00000006575	ENSMUSG00000016018
ENSMUSG00000006576	ENSMUSG00000016150
ENSMUSG00000006586	ENSMUSG00000016179
ENSMUSG00000006589	ENSMUSG00000016200
ENSMUSG00000006599	ENSMUSG00000016252
ENSMUSG00000006638	ENSMUSG00000016253
ENSMUSG00000006641	ENSMUSG00000016256
ENSMUSG00000006651	ENSMUSG00000016262
ENSMUSG00000006673	ENSMUSG00000016319
ENSMUSG00000006675	ENSMUSG00000016344
ENSMUSG00000006676	ENSMUSG00000016346
ENSMUSG00000006678	ENSMUSG00000016349
ENSMUSG00000006699	ENSMUSG00000016382
ENSMUSG00000006705	ENSMUSG00000016386
ENSMUSG00000006711	ENSMUSG00000016427
ENSMUSG00000006717	ENSMUSG00000016487
ENSMUSG00000006728	ENSMUSG00000016534
ENSMUSG00000006731	ENSMUSG00000016541
ENSMUSG00000006732	ENSMUSG00000016554
ENSMUSG00000006736	ENSMUSG00000016559
ENSMUSG00000006740	ENSMUSG00000016619
ENSMUSG00000006763	ENSMUSG00000016664
ENSMUSG00000006782	ENSMUSG00000016831
ENSMUSG00000006784	ENSMUSG00000016921
ENSMUSG00000006800	ENSMUSG00000016933
ENSMUSG00000006818	ENSMUSG00000016940
ENSMUSG00000006850	ENSMUSG00000017009
ENSMUSG00000006906	ENSMUSG00000017119
ENSMUSG00000006920	ENSMUSG00000017132
ENSMUSG00000006930	ENSMUSG00000017167
ENSMUSG00000006931	ENSMUSG00000017188
ENSMUSG00000006932	ENSMUSG00000017210
ENSMUSG00000006941	ENSMUSG00000017221
ENSMUSG00000006958	ENSMUSG00000017264
ENSMUSG00000006998	ENSMUSG00000017286
ENSMUSG00000007021	ENSMUSG00000017288
ENSMUSG00000007029	ENSMUSG00000017291
ENSMUSG00000007036	ENSMUSG00000017314
ENSMUSG00000007038	ENSMUSG00000017344
ENSMUSG00000007039	ENSMUSG00000017376

ENSMUSG00000007041	ENSMUSG00000017390
ENSMUSG00000007097	ENSMUSG00000017400
ENSMUSG00000007207	ENSMUSG00000017404
ENSMUSG00000007216	ENSMUSG00000017412
ENSMUSG00000007338	ENSMUSG00000017421
ENSMUSG00000007411	ENSMUSG00000017428
ENSMUSG00000007415	ENSMUSG00000017466
ENSMUSG00000007440	ENSMUSG00000017478
ENSMUSG00000007458	ENSMUSG00000017485
ENSMUSG00000007476	ENSMUSG00000017491
ENSMUSG00000007564	ENSMUSG00000017493
ENSMUSG00000007570	ENSMUSG00000017548
ENSMUSG00000007589	ENSMUSG00000017615
ENSMUSG00000007594	ENSMUSG00000017631
ENSMUSG00000007603	ENSMUSG00000017639
ENSMUSG00000007610	ENSMUSG00000017664
ENSMUSG00000007613	ENSMUSG00000017667
ENSMUSG00000007617	ENSMUSG00000017670
ENSMUSG00000007653	ENSMUSG00000017677
ENSMUSG00000007656	ENSMUSG00000017679
ENSMUSG00000007659	ENSMUSG00000017686
ENSMUSG00000007670	ENSMUSG00000017692
ENSMUSG00000007682	ENSMUSG00000017707
ENSMUSG00000007721	ENSMUSG00000017715
ENSMUSG00000007739	ENSMUSG00000017721
ENSMUSG00000007777	ENSMUSG00000017734
ENSMUSG00000007783	ENSMUSG00000017740
ENSMUSG00000007812	ENSMUSG00000017754
ENSMUSG00000007815	ENSMUSG00000017760
ENSMUSG00000007817	ENSMUSG00000017776
ENSMUSG00000007827	ENSMUSG00000017778
ENSMUSG00000007833	ENSMUSG00000017781
ENSMUSG00000007836	ENSMUSG00000017802
ENSMUSG00000007850	ENSMUSG00000017831
ENSMUSG00000007867	ENSMUSG00000017843
ENSMUSG00000007872	ENSMUSG00000017929
ENSMUSG00000007880	ENSMUSG00000017943
ENSMUSG00000007891	ENSMUSG00000018001
ENSMUSG00000007892	ENSMUSG00000018040
ENSMUSG00000007908	ENSMUSG00000018042
ENSMUSG00000007944	ENSMUSG00000018068
ENSMUSG00000007950	ENSMUSG00000018076
ENSMUSG00000007987	ENSMUSG00000018160
ENSMUSG00000007989	ENSMUSG00000018167
ENSMUSG00000008035	ENSMUSG00000018171
ENSMUSG00000008036	ENSMUSG00000018196
ENSMUSG00000008090	ENSMUSG00000018199
ENSMUSG00000008140	ENSMUSG00000018209
ENSMUSG00000008153	ENSMUSG00000018286
ENSMUSG00000008167	ENSMUSG00000018293
ENSMUSG00000008200	ENSMUSG00000018322
ENSMUSG00000008206	ENSMUSG00000018326
ENSMUSG00000008226	ENSMUSG00000018334

ENSMUSG00000008301	ENSMUSG00000018340
ENSMUSG00000008305	ENSMUSG00000018363
ENSMUSG00000008318	ENSMUSG00000018377
ENSMUSG00000008333	ENSMUSG00000018378
ENSMUSG00000008348	ENSMUSG00000018379
ENSMUSG00000008373	ENSMUSG00000018395
ENSMUSG00000008384	ENSMUSG00000018398
ENSMUSG00000008393	ENSMUSG00000018401
ENSMUSG00000008429	ENSMUSG00000018411
ENSMUSG00000008435	ENSMUSG00000018412
ENSMUSG00000008450	ENSMUSG00000018415
ENSMUSG00000008475	ENSMUSG00000018417
ENSMUSG00000008489	ENSMUSG00000018425
ENSMUSG00000008496	ENSMUSG00000018427
ENSMUSG00000008540	ENSMUSG00000018428
ENSMUSG00000008575	ENSMUSG00000018446
ENSMUSG00000008604	ENSMUSG00000018451
ENSMUSG00000008658	ENSMUSG00000018474
ENSMUSG00000008668	ENSMUSG00000018476
ENSMUSG00000008682	ENSMUSG00000018481
ENSMUSG00000008683	ENSMUSG00000018501
ENSMUSG00000008690	ENSMUSG00000018547
ENSMUSG00000008730	ENSMUSG00000018548
ENSMUSG00000008734	ENSMUSG00000018559
ENSMUSG00000008763	ENSMUSG00000018565
ENSMUSG00000008822	ENSMUSG00000018567
ENSMUSG00000008855	ENSMUSG00000018572
ENSMUSG00000008859	ENSMUSG00000018574
ENSMUSG00000008892	ENSMUSG00000018583
ENSMUSG00000008958	ENSMUSG00000018593
ENSMUSG00000008976	ENSMUSG00000018659
ENSMUSG00000008999	ENSMUSG00000018661
ENSMUSG00000009013	ENSMUSG00000018666
ENSMUSG00000009030	ENSMUSG00000018669
ENSMUSG00000009035	ENSMUSG00000018677
ENSMUSG00000009073	ENSMUSG00000018707
ENSMUSG00000009075	ENSMUSG00000018736
ENSMUSG00000009076	ENSMUSG00000018750
ENSMUSG00000009079	ENSMUSG00000018765
ENSMUSG00000009090	ENSMUSG00000018770
ENSMUSG00000009112	ENSMUSG00000018796
ENSMUSG00000009207	ENSMUSG00000018800
ENSMUSG00000009216	ENSMUSG00000018820
ENSMUSG00000009281	ENSMUSG00000018846
ENSMUSG00000009291	ENSMUSG00000018848
ENSMUSG00000009292	ENSMUSG00000018849
ENSMUSG00000009293	ENSMUSG00000018865
ENSMUSG00000009394	ENSMUSG00000018909
ENSMUSG00000009406	ENSMUSG00000018921
ENSMUSG00000009418	ENSMUSG00000018965
ENSMUSG00000009470	ENSMUSG00000018974
ENSMUSG00000009535	ENSMUSG00000018999
ENSMUSG00000009549	ENSMUSG00000019039

ENSMUSG00000009555	ENSMUSG00000019054
ENSMUSG00000009563	ENSMUSG00000019082
ENSMUSG00000009566	ENSMUSG00000019087
ENSMUSG00000009569	ENSMUSG00000019124
ENSMUSG00000009575	ENSMUSG00000019132
ENSMUSG00000009614	ENSMUSG00000019139
ENSMUSG00000009621	ENSMUSG00000019146
ENSMUSG00000009630	ENSMUSG00000019173
ENSMUSG00000009640	ENSMUSG00000019179
ENSMUSG00000009647	ENSMUSG00000019188
ENSMUSG00000009681	ENSMUSG00000019189
ENSMUSG00000009731	ENSMUSG00000019194
ENSMUSG00000009733	ENSMUSG00000019210
ENSMUSG00000009739	ENSMUSG00000019235
ENSMUSG00000009741	ENSMUSG00000019254
ENSMUSG00000009828	ENSMUSG00000019261
ENSMUSG00000009863	ENSMUSG00000019302
ENSMUSG00000009894	ENSMUSG00000019338
ENSMUSG00000009905	ENSMUSG00000019362
ENSMUSG00000009907	ENSMUSG00000019370
ENSMUSG00000009927	ENSMUSG00000019373
ENSMUSG00000009995	ENSMUSG00000019428
ENSMUSG00000010021	ENSMUSG00000019432
ENSMUSG00000010025	ENSMUSG00000019433
ENSMUSG00000010044	ENSMUSG00000019467
ENSMUSG00000010045	ENSMUSG00000019470
ENSMUSG00000010047	ENSMUSG00000019471
ENSMUSG00000010048	ENSMUSG00000019494
ENSMUSG00000010054	ENSMUSG00000019505
ENSMUSG00000010057	ENSMUSG00000019558
ENSMUSG00000010064	ENSMUSG00000019578
ENSMUSG00000010066	ENSMUSG00000019590
ENSMUSG00000010067	ENSMUSG00000019647
ENSMUSG00000010086	ENSMUSG00000019699
ENSMUSG00000010095	ENSMUSG00000019710
ENSMUSG00000010097	ENSMUSG00000019715
ENSMUSG00000010110	ENSMUSG00000019726
ENSMUSG00000010154	ENSMUSG00000019731
ENSMUSG00000010175	ENSMUSG00000019775
ENSMUSG00000010205	ENSMUSG00000019777
ENSMUSG00000010277	ENSMUSG00000019785
ENSMUSG00000010290	ENSMUSG00000019790
ENSMUSG00000010307	ENSMUSG00000019795
ENSMUSG00000010362	ENSMUSG00000019796
ENSMUSG00000010376	ENSMUSG00000019802
ENSMUSG00000010392	ENSMUSG00000019804
ENSMUSG00000010406	ENSMUSG00000019806
ENSMUSG00000010453	ENSMUSG00000019810
ENSMUSG00000010505	ENSMUSG00000019818
ENSMUSG00000010517	ENSMUSG00000019820
ENSMUSG00000010554	ENSMUSG00000019828
ENSMUSG00000010607	ENSMUSG00000019831
ENSMUSG00000010608	ENSMUSG00000019841

ENSMUSG00000010609	ENSMUSG00000019843
ENSMUSG00000010660	ENSMUSG00000019849
ENSMUSG00000010663	ENSMUSG00000019852
ENSMUSG00000010721	ENSMUSG00000019854
ENSMUSG00000010755	ENSMUSG00000019856
ENSMUSG00000010803	ENSMUSG00000019861
ENSMUSG00000010911	ENSMUSG00000019868
ENSMUSG00000010914	ENSMUSG00000019877
ENSMUSG00000010936	ENSMUSG00000019894
ENSMUSG00000011096	ENSMUSG00000019906
ENSMUSG00000011114	ENSMUSG00000019907
ENSMUSG00000011148	ENSMUSG00000019916
ENSMUSG00000011154	ENSMUSG00000019923
ENSMUSG00000011158	ENSMUSG00000019943
ENSMUSG00000011179	ENSMUSG00000019947
ENSMUSG00000011254	ENSMUSG00000019951
ENSMUSG00000011256	ENSMUSG00000019966
ENSMUSG00000011257	ENSMUSG00000019969
ENSMUSG00000011305	ENSMUSG00000019970
ENSMUSG00000011306	ENSMUSG00000019971
ENSMUSG00000011382	ENSMUSG00000019977
ENSMUSG00000011427	ENSMUSG00000019978
ENSMUSG00000011589	ENSMUSG00000019984
ENSMUSG00000011658	ENSMUSG00000019986
ENSMUSG00000011751	ENSMUSG00000019990
ENSMUSG00000011752	ENSMUSG00000019996
ENSMUSG00000011831	ENSMUSG00000019998
ENSMUSG00000011832	ENSMUSG00000020014
ENSMUSG00000011837	ENSMUSG00000020015
ENSMUSG00000011877	ENSMUSG00000020022
ENSMUSG00000011884	ENSMUSG00000020023
ENSMUSG00000011958	ENSMUSG00000020024
ENSMUSG00000011960	ENSMUSG00000020029
ENSMUSG00000012017	ENSMUSG00000020032
ENSMUSG00000012076	ENSMUSG00000020042
ENSMUSG00000012114	ENSMUSG00000020044
ENSMUSG00000012117	ENSMUSG00000020048
ENSMUSG00000012126	ENSMUSG00000020070
ENSMUSG00000012296	ENSMUSG00000020074
ENSMUSG00000012405	ENSMUSG00000020075
ENSMUSG00000012422	ENSMUSG00000020076
ENSMUSG00000012429	ENSMUSG00000020088
ENSMUSG00000012483	ENSMUSG00000020089
ENSMUSG00000012535	ENSMUSG00000020091
ENSMUSG00000012609	ENSMUSG00000020099
ENSMUSG00000012640	ENSMUSG00000020101
ENSMUSG00000012819	ENSMUSG00000020107
ENSMUSG00000012848	ENSMUSG00000020108
ENSMUSG00000013033	ENSMUSG00000020109
ENSMUSG00000013076	ENSMUSG00000020111
ENSMUSG00000013089	ENSMUSG00000020114
ENSMUSG00000013150	ENSMUSG00000020115
ENSMUSG00000013155	ENSMUSG00000020121

ENSMUSG00000013160	ENSMUSG00000020124
ENSMUSG00000013236	ENSMUSG00000020130
ENSMUSG00000013275	ENSMUSG00000020132
ENSMUSG00000013367	ENSMUSG00000020134
ENSMUSG00000013419	ENSMUSG00000020135
ENSMUSG00000013465	ENSMUSG00000020142
ENSMUSG00000013495	ENSMUSG00000020149
ENSMUSG00000013523	ENSMUSG00000020151
ENSMUSG00000013539	ENSMUSG00000020152
ENSMUSG00000013593	ENSMUSG00000020153
ENSMUSG00000013622	ENSMUSG00000020154
ENSMUSG00000013629	ENSMUSG00000020156
ENSMUSG00000013646	ENSMUSG00000020163
ENSMUSG00000013662	ENSMUSG00000020166
ENSMUSG00000013663	ENSMUSG00000020170
ENSMUSG00000013698	ENSMUSG00000020173
ENSMUSG00000013701	ENSMUSG00000020175
ENSMUSG00000013736	ENSMUSG00000020176
ENSMUSG00000013787	ENSMUSG00000020178
ENSMUSG00000013822	ENSMUSG00000020180
ENSMUSG00000013833	ENSMUSG00000020181
ENSMUSG00000013846	ENSMUSG00000020184
ENSMUSG00000013858	ENSMUSG00000020189
ENSMUSG00000013878	ENSMUSG00000020190
ENSMUSG00000013921	ENSMUSG00000020196
ENSMUSG00000013973	ENSMUSG00000020198
ENSMUSG00000013997	ENSMUSG00000020205
ENSMUSG00000014039	ENSMUSG00000020211
ENSMUSG00000014074	ENSMUSG00000020219
ENSMUSG00000014075	ENSMUSG00000020220
ENSMUSG00000014077	ENSMUSG00000020230
ENSMUSG00000014164	ENSMUSG00000020231
ENSMUSG00000014177	ENSMUSG00000020235
ENSMUSG00000014195	ENSMUSG00000020238
ENSMUSG00000014226	ENSMUSG00000020250
ENSMUSG00000014232	ENSMUSG00000020255
ENSMUSG00000014245	ENSMUSG00000020257
ENSMUSG00000014294	ENSMUSG00000020260
ENSMUSG00000014301	ENSMUSG00000020261
ENSMUSG00000014303	ENSMUSG00000020262
ENSMUSG00000014313	ENSMUSG00000020263
ENSMUSG00000014329	ENSMUSG00000020265
ENSMUSG00000014349	ENSMUSG00000020267
ENSMUSG00000014353	ENSMUSG00000020271
ENSMUSG00000014355	ENSMUSG00000020277
ENSMUSG00000014361	ENSMUSG00000020284
ENSMUSG00000014402	ENSMUSG00000020290
ENSMUSG00000014418	ENSMUSG00000020297
ENSMUSG00000014426	ENSMUSG00000020300
ENSMUSG00000014444	ENSMUSG00000020307
ENSMUSG00000014470	ENSMUSG00000020308
ENSMUSG00000014496	ENSMUSG00000020311
ENSMUSG00000014498	ENSMUSG00000020312

ENSMUSG00000014504	ENSMUSG00000020315
ENSMUSG00000014547	ENSMUSG00000020321
ENSMUSG00000014550	ENSMUSG00000020326
ENSMUSG00000014551	ENSMUSG00000020329
ENSMUSG00000014554	ENSMUSG00000020331
ENSMUSG00000014592	ENSMUSG00000020333
ENSMUSG00000014599	ENSMUSG00000020340
ENSMUSG00000014601	ENSMUSG00000020346
ENSMUSG00000014602	ENSMUSG00000020349
ENSMUSG00000014606	ENSMUSG00000020358
ENSMUSG00000014633	ENSMUSG00000020361
ENSMUSG00000014668	ENSMUSG00000020362
ENSMUSG00000014748	ENSMUSG00000020366
ENSMUSG00000014763	ENSMUSG00000020368
ENSMUSG00000014767	ENSMUSG00000020372
ENSMUSG00000014769	ENSMUSG00000020376
ENSMUSG00000014771	ENSMUSG00000020385
ENSMUSG00000014773	ENSMUSG00000020386
ENSMUSG00000014776	ENSMUSG00000020387
ENSMUSG00000014778	ENSMUSG00000020390
ENSMUSG00000014786	ENSMUSG00000020396
ENSMUSG00000014813	ENSMUSG00000020400
ENSMUSG00000014837	ENSMUSG00000020402
ENSMUSG00000014846	ENSMUSG00000020409
ENSMUSG00000014850	ENSMUSG00000020422
ENSMUSG00000014856	ENSMUSG00000020430
ENSMUSG00000014859	ENSMUSG00000020431
ENSMUSG00000014867	ENSMUSG00000020435
ENSMUSG00000014873	ENSMUSG00000020436
ENSMUSG00000014905	ENSMUSG00000020440
ENSMUSG00000014907	ENSMUSG00000020444
ENSMUSG00000014932	ENSMUSG00000020448
ENSMUSG00000014956	ENSMUSG00000020451
ENSMUSG00000014959	ENSMUSG00000020453
ENSMUSG00000014980	ENSMUSG00000020454
ENSMUSG00000015002	ENSMUSG00000020456
ENSMUSG00000015013	ENSMUSG00000020457
ENSMUSG00000015016	ENSMUSG00000020458
ENSMUSG00000015023	ENSMUSG00000020460
ENSMUSG00000015027	ENSMUSG00000020462
ENSMUSG00000015085	ENSMUSG00000020463
ENSMUSG00000015087	ENSMUSG00000020476
ENSMUSG00000015090	ENSMUSG00000020483
ENSMUSG00000015092	ENSMUSG00000020484
ENSMUSG00000015094	ENSMUSG00000020486
ENSMUSG00000015095	ENSMUSG00000020496
ENSMUSG00000015120	ENSMUSG00000020515
ENSMUSG00000015126	ENSMUSG00000020516
ENSMUSG00000015127	ENSMUSG00000020524
ENSMUSG00000015143	ENSMUSG00000020528
ENSMUSG00000015149	ENSMUSG00000020530
ENSMUSG00000015165	ENSMUSG00000020532
ENSMUSG00000015176	ENSMUSG00000020536

ENSMUSG00000015189	ENSMUSG00000020537
ENSMUSG00000015202	ENSMUSG00000020538
ENSMUSG00000015214	ENSMUSG00000020547
ENSMUSG00000015217	ENSMUSG00000020564
ENSMUSG00000015222	ENSMUSG00000020571
ENSMUSG00000015224	ENSMUSG00000020572
ENSMUSG00000015243	ENSMUSG00000020576
ENSMUSG00000015247	ENSMUSG00000020577
ENSMUSG00000015289	ENSMUSG00000020580
ENSMUSG00000015290	ENSMUSG00000020585
ENSMUSG00000015291	ENSMUSG00000020589
ENSMUSG00000015305	ENSMUSG00000020590
ENSMUSG00000015337	ENSMUSG00000020591
ENSMUSG00000015341	ENSMUSG00000020593
ENSMUSG00000015342	ENSMUSG00000020594
ENSMUSG00000015357	ENSMUSG00000020598
ENSMUSG00000015363	ENSMUSG00000020599
ENSMUSG00000015377	ENSMUSG00000020601
ENSMUSG00000015396	ENSMUSG00000020607
ENSMUSG00000015461	ENSMUSG00000020608
ENSMUSG00000015468	ENSMUSG00000020610
ENSMUSG00000015474	ENSMUSG00000020611
ENSMUSG00000015476	ENSMUSG00000020612
ENSMUSG00000015478	ENSMUSG00000020620
ENSMUSG00000015484	ENSMUSG00000020627
ENSMUSG00000015488	ENSMUSG00000020628
ENSMUSG00000015501	ENSMUSG00000020629
ENSMUSG00000015522	ENSMUSG00000020640
ENSMUSG00000015536	ENSMUSG00000020646
ENSMUSG00000015542	ENSMUSG00000020647
ENSMUSG00000015568	ENSMUSG00000020654
ENSMUSG00000015575	ENSMUSG00000020657
ENSMUSG00000015597	ENSMUSG00000020658
ENSMUSG00000015599	ENSMUSG00000020661
ENSMUSG00000015605	ENSMUSG00000020664
ENSMUSG00000015647	ENSMUSG00000020668
ENSMUSG00000015653	ENSMUSG00000020671
ENSMUSG00000015656	ENSMUSG00000020674
ENSMUSG00000015659	ENSMUSG00000020684
ENSMUSG00000015668	ENSMUSG00000020687
ENSMUSG00000015671	ENSMUSG00000020694
ENSMUSG00000015672	ENSMUSG00000020697
ENSMUSG00000015697	ENSMUSG00000020701
ENSMUSG00000015709	ENSMUSG00000020704
ENSMUSG00000015711	ENSMUSG00000020705
ENSMUSG00000015714	ENSMUSG00000020706
ENSMUSG00000015733	ENSMUSG00000020708
ENSMUSG00000015745	ENSMUSG00000020716
ENSMUSG00000015747	ENSMUSG00000020719
ENSMUSG00000015748	ENSMUSG00000020720
ENSMUSG00000015749	ENSMUSG00000020721
ENSMUSG00000015750	ENSMUSG00000020723
ENSMUSG00000015755	ENSMUSG00000020733

ENSMUSG00000015757	ENSMUSG00000020734
ENSMUSG00000015759	ENSMUSG00000020737
ENSMUSG00000015766	ENSMUSG00000020738
ENSMUSG00000015776	ENSMUSG00000020740
ENSMUSG00000015790	ENSMUSG00000020741
ENSMUSG00000015804	ENSMUSG00000020745
ENSMUSG00000015806	ENSMUSG00000020747
ENSMUSG00000015829	ENSMUSG00000020755
ENSMUSG00000015837	ENSMUSG00000020775
ENSMUSG00000015839	ENSMUSG00000020776
ENSMUSG00000015843	ENSMUSG00000020777
ENSMUSG00000015846	ENSMUSG00000020780
ENSMUSG00000015850	ENSMUSG00000020783
ENSMUSG00000015852	ENSMUSG00000020785
ENSMUSG00000015869	ENSMUSG00000020790
ENSMUSG00000015879	ENSMUSG00000020792
ENSMUSG00000015882	ENSMUSG00000020794
ENSMUSG00000015889	ENSMUSG00000020802
ENSMUSG00000015932	ENSMUSG00000020810
ENSMUSG00000015937	ENSMUSG00000020811
ENSMUSG00000015942	ENSMUSG00000020817
ENSMUSG00000015943	ENSMUSG00000020818
ENSMUSG00000015944	ENSMUSG00000020821
ENSMUSG00000015950	ENSMUSG00000020823
ENSMUSG00000015961	ENSMUSG00000020827
ENSMUSG00000015968	ENSMUSG00000020840
ENSMUSG00000015970	ENSMUSG00000020841
ENSMUSG00000015971	ENSMUSG00000020843
ENSMUSG00000015981	ENSMUSG00000020848
ENSMUSG00000015994	ENSMUSG00000020849
ENSMUSG00000016018	ENSMUSG00000020850
ENSMUSG00000016024	ENSMUSG00000020857
ENSMUSG00000016028	ENSMUSG00000020859
ENSMUSG00000016128	ENSMUSG00000020863
ENSMUSG00000016150	ENSMUSG00000020864
ENSMUSG00000016179	ENSMUSG00000020866
ENSMUSG00000016181	ENSMUSG00000020869
ENSMUSG00000016200	ENSMUSG00000020882
ENSMUSG00000016239	ENSMUSG00000020883
ENSMUSG00000016252	ENSMUSG00000020886
ENSMUSG00000016253	ENSMUSG00000020889
ENSMUSG00000016256	ENSMUSG00000020893
ENSMUSG00000016257	ENSMUSG00000020894
ENSMUSG00000016262	ENSMUSG00000020898
ENSMUSG00000016308	ENSMUSG00000020899
ENSMUSG00000016319	ENSMUSG00000020900
ENSMUSG00000016344	ENSMUSG00000020902
ENSMUSG00000016346	ENSMUSG00000020917
ENSMUSG00000016349	ENSMUSG00000020918
ENSMUSG00000016382	ENSMUSG00000020919
ENSMUSG00000016386	ENSMUSG00000020922
ENSMUSG00000016409	ENSMUSG00000020923
ENSMUSG00000016427	ENSMUSG00000020926

ENSMUSG00000016477	ENSMUSG00000020929
ENSMUSG00000016481	ENSMUSG00000020932
ENSMUSG00000016487	ENSMUSG00000020936
ENSMUSG00000016494	ENSMUSG00000020946
ENSMUSG00000016495	ENSMUSG00000020949
ENSMUSG00000016503	ENSMUSG00000020950
ENSMUSG00000016510	ENSMUSG00000020954
ENSMUSG00000016520	ENSMUSG00000020961
ENSMUSG00000016526	ENSMUSG00000020962
ENSMUSG00000016528	ENSMUSG00000020964
ENSMUSG00000016534	ENSMUSG00000020978
ENSMUSG00000016541	ENSMUSG00000020982
ENSMUSG00000016552	ENSMUSG00000020986
ENSMUSG00000016554	ENSMUSG00000020993
ENSMUSG00000016559	ENSMUSG00000020994
ENSMUSG00000016619	ENSMUSG00000021003
ENSMUSG00000016624	ENSMUSG00000021012
ENSMUSG00000016637	ENSMUSG00000021024
ENSMUSG00000016664	ENSMUSG00000021027
ENSMUSG00000016757	ENSMUSG00000021036
ENSMUSG00000016763	ENSMUSG00000021037
ENSMUSG00000016831	ENSMUSG00000021038
ENSMUSG00000016833	ENSMUSG00000021039
ENSMUSG00000016918	ENSMUSG00000021044
ENSMUSG00000016921	ENSMUSG00000021047
ENSMUSG00000016933	ENSMUSG00000021048
ENSMUSG00000016940	ENSMUSG00000021051
ENSMUSG00000016946	ENSMUSG00000021054
ENSMUSG00000016995	ENSMUSG00000021057
ENSMUSG00000017009	ENSMUSG00000021061
ENSMUSG00000017057	ENSMUSG00000021062
ENSMUSG00000017119	ENSMUSG00000021065
ENSMUSG00000017132	ENSMUSG00000021066
ENSMUSG00000017144	ENSMUSG00000021068
ENSMUSG00000017167	ENSMUSG00000021071
ENSMUSG00000017176	ENSMUSG00000021072
ENSMUSG00000017188	ENSMUSG00000021076
ENSMUSG00000017210	ENSMUSG00000021087
ENSMUSG00000017221	ENSMUSG00000021096
ENSMUSG00000017264	ENSMUSG00000021097
ENSMUSG00000017286	ENSMUSG00000021102
ENSMUSG00000017288	ENSMUSG00000021108
ENSMUSG00000017291	ENSMUSG00000021109
ENSMUSG00000017299	ENSMUSG00000021111
ENSMUSG00000017307	ENSMUSG00000021112
ENSMUSG00000017314	ENSMUSG00000021113
ENSMUSG00000017344	ENSMUSG00000021114
ENSMUSG00000017376	ENSMUSG00000021124
ENSMUSG00000017386	ENSMUSG00000021130
ENSMUSG00000017390	ENSMUSG00000021131
ENSMUSG00000017400	ENSMUSG00000021133
ENSMUSG00000017404	ENSMUSG00000021134
ENSMUSG00000017412	ENSMUSG00000021140

ENSMUSG00000017417	ENSMUSG00000021143
ENSMUSG00000017418	ENSMUSG00000021144
ENSMUSG00000017421	ENSMUSG00000021147
ENSMUSG00000017428	ENSMUSG00000021149
ENSMUSG00000017466	ENSMUSG00000021156
ENSMUSG00000017478	ENSMUSG00000021171
ENSMUSG00000017485	ENSMUSG00000021178
ENSMUSG00000017491	ENSMUSG00000021179
ENSMUSG00000017493	ENSMUSG00000021180
ENSMUSG00000017548	ENSMUSG00000021182
ENSMUSG00000017550	ENSMUSG00000021188
ENSMUSG00000017561	ENSMUSG00000021189
ENSMUSG00000017615	ENSMUSG00000021190
ENSMUSG00000017631	ENSMUSG00000021193
ENSMUSG00000017639	ENSMUSG00000021194
ENSMUSG00000017664	ENSMUSG00000021196
ENSMUSG00000017667	ENSMUSG00000021198
ENSMUSG00000017670	ENSMUSG00000021209
ENSMUSG00000017677	ENSMUSG00000021218
ENSMUSG00000017679	ENSMUSG00000021238
ENSMUSG00000017686	ENSMUSG00000021242
ENSMUSG00000017692	ENSMUSG00000021244
ENSMUSG00000017707	ENSMUSG00000021245
ENSMUSG00000017713	ENSMUSG00000021248
ENSMUSG00000017715	ENSMUSG00000021254
ENSMUSG00000017721	ENSMUSG00000021259
ENSMUSG00000017724	ENSMUSG00000021260
ENSMUSG00000017734	ENSMUSG00000021262
ENSMUSG00000017740	ENSMUSG00000021264
ENSMUSG00000017747	ENSMUSG00000021266
ENSMUSG00000017754	ENSMUSG00000021268
ENSMUSG00000017756	ENSMUSG00000021270
ENSMUSG00000017760	ENSMUSG00000021273
ENSMUSG00000017764	ENSMUSG00000021275
ENSMUSG00000017765	ENSMUSG00000021276
ENSMUSG00000017774	ENSMUSG00000021277
ENSMUSG00000017776	ENSMUSG00000021279
ENSMUSG00000017778	ENSMUSG00000021282
ENSMUSG00000017781	ENSMUSG00000021285
ENSMUSG00000017801	ENSMUSG00000021288
ENSMUSG00000017802	ENSMUSG00000021290
ENSMUSG00000017831	ENSMUSG00000021301
ENSMUSG00000017837	ENSMUSG00000021302
ENSMUSG00000017843	ENSMUSG00000021311
ENSMUSG00000017858	ENSMUSG00000021313
ENSMUSG00000017929	ENSMUSG00000021314
ENSMUSG00000017943	ENSMUSG00000021327
ENSMUSG00000017978	ENSMUSG00000021338
ENSMUSG00000017999	ENSMUSG00000021339
ENSMUSG00000018001	ENSMUSG00000021340
ENSMUSG00000018008	ENSMUSG00000021357
ENSMUSG00000018012	ENSMUSG00000021360
ENSMUSG00000018040	ENSMUSG00000021366

ENSMUSG00000018042	ENSMUSG00000021373
ENSMUSG00000018068	ENSMUSG00000021374
ENSMUSG00000018076	ENSMUSG00000021375
ENSMUSG00000018102	ENSMUSG00000021379
ENSMUSG00000018143	ENSMUSG00000021385
ENSMUSG00000018160	ENSMUSG00000021395
ENSMUSG00000018166	ENSMUSG00000021400
ENSMUSG00000018167	ENSMUSG00000021413
ENSMUSG00000018171	ENSMUSG00000021420
ENSMUSG00000018189	ENSMUSG00000021427
ENSMUSG00000018196	ENSMUSG00000021448
ENSMUSG00000018199	ENSMUSG00000021451
ENSMUSG00000018209	ENSMUSG00000021458
ENSMUSG00000018217	ENSMUSG00000021460
ENSMUSG00000018286	ENSMUSG00000021466
ENSMUSG00000018287	ENSMUSG00000021474
ENSMUSG00000018293	ENSMUSG00000021476
ENSMUSG00000018322	ENSMUSG00000021477
ENSMUSG00000018326	ENSMUSG00000021478
ENSMUSG00000018334	ENSMUSG00000021481
ENSMUSG00000018339	ENSMUSG00000021484
ENSMUSG00000018340	ENSMUSG00000021486
ENSMUSG00000018347	ENSMUSG00000021488
ENSMUSG00000018362	ENSMUSG00000021495
ENSMUSG00000018363	ENSMUSG00000021497
ENSMUSG00000018377	ENSMUSG00000021500
ENSMUSG00000018378	ENSMUSG00000021508
ENSMUSG00000018379	ENSMUSG00000021518
ENSMUSG00000018395	ENSMUSG00000021520
ENSMUSG00000018398	ENSMUSG00000021536
ENSMUSG00000018401	ENSMUSG00000021540
ENSMUSG00000018405	ENSMUSG00000021546
ENSMUSG00000018411	ENSMUSG00000021549
ENSMUSG00000018412	ENSMUSG00000021555
ENSMUSG00000018415	ENSMUSG00000021557
ENSMUSG00000018417	ENSMUSG00000021559
ENSMUSG00000018425	ENSMUSG00000021573
ENSMUSG00000018427	ENSMUSG00000021576
ENSMUSG00000018428	ENSMUSG00000021577
ENSMUSG00000018433	ENSMUSG00000021578
ENSMUSG00000018442	ENSMUSG00000021595
ENSMUSG00000018446	ENSMUSG00000021596
ENSMUSG00000018449	ENSMUSG00000021597
ENSMUSG00000018451	ENSMUSG00000021606
ENSMUSG00000018459	ENSMUSG00000021608
ENSMUSG00000018474	ENSMUSG00000021610
ENSMUSG00000018476	ENSMUSG00000021614
ENSMUSG00000018481	ENSMUSG00000021629
ENSMUSG00000018500	ENSMUSG00000021646
ENSMUSG00000018501	ENSMUSG00000021647
ENSMUSG00000018507	ENSMUSG00000021660
ENSMUSG00000018509	ENSMUSG00000021662
ENSMUSG00000018537	ENSMUSG00000021665

ENSMUSG00000018541	ENSMUSG00000021669
ENSMUSG00000018547	ENSMUSG00000021670
ENSMUSG00000018548	ENSMUSG00000021671
ENSMUSG00000018559	ENSMUSG00000021681
ENSMUSG00000018565	ENSMUSG00000021684
ENSMUSG00000018567	ENSMUSG00000021686
ENSMUSG00000018572	ENSMUSG00000021687
ENSMUSG00000018574	ENSMUSG00000021690
ENSMUSG00000018581	ENSMUSG00000021693
ENSMUSG00000018583	ENSMUSG00000021699
ENSMUSG00000018585	ENSMUSG00000021700
ENSMUSG00000018589	ENSMUSG00000021701
ENSMUSG00000018593	ENSMUSG00000021703
ENSMUSG00000018599	ENSMUSG00000021704
ENSMUSG00000018634	ENSMUSG00000021708
ENSMUSG00000018648	ENSMUSG00000021709
ENSMUSG00000018651	ENSMUSG00000021710
ENSMUSG00000018659	ENSMUSG00000021711
ENSMUSG00000018661	ENSMUSG00000021712
ENSMUSG00000018666	ENSMUSG00000021719
ENSMUSG00000018669	ENSMUSG00000021730
ENSMUSG00000018677	ENSMUSG00000021737
ENSMUSG00000018678	ENSMUSG00000021745
ENSMUSG00000018697	ENSMUSG00000021748
ENSMUSG00000018707	ENSMUSG00000021750
ENSMUSG00000018733	ENSMUSG00000021756
ENSMUSG00000018736	ENSMUSG00000021764
ENSMUSG00000018750	ENSMUSG00000021767
ENSMUSG00000018761	ENSMUSG00000021770
ENSMUSG00000018765	ENSMUSG00000021771
ENSMUSG00000018770	ENSMUSG00000021772
ENSMUSG00000018796	ENSMUSG00000021775
ENSMUSG00000018800	ENSMUSG00000021779
ENSMUSG00000018809	ENSMUSG00000021782
ENSMUSG00000018820	ENSMUSG00000021792
ENSMUSG00000018821	ENSMUSG00000021794
ENSMUSG00000018841	ENSMUSG00000021796
ENSMUSG00000018846	ENSMUSG00000021810
ENSMUSG00000018848	ENSMUSG00000021814
ENSMUSG00000018849	ENSMUSG00000021816
ENSMUSG00000018858	ENSMUSG00000021819
ENSMUSG00000018861	ENSMUSG00000021820
ENSMUSG00000018865	ENSMUSG00000021823
ENSMUSG00000018882	ENSMUSG00000021824
ENSMUSG00000018900	ENSMUSG00000021830
ENSMUSG00000018906	ENSMUSG00000021831
ENSMUSG00000018909	ENSMUSG00000021832
ENSMUSG00000018921	ENSMUSG00000021838
ENSMUSG00000018923	ENSMUSG00000021840
ENSMUSG00000018931	ENSMUSG00000021843
ENSMUSG00000018932	ENSMUSG00000021846
ENSMUSG00000018965	ENSMUSG00000021870
ENSMUSG00000018974	ENSMUSG00000021877

ENSMUSG00000018995	ENSMUSG00000021892
ENSMUSG00000018999	ENSMUSG00000021893
ENSMUSG00000019027	ENSMUSG00000021895
ENSMUSG00000019039	ENSMUSG00000021901
ENSMUSG00000019054	ENSMUSG00000021910
ENSMUSG00000019055	ENSMUSG00000021911
ENSMUSG00000019080	ENSMUSG00000021913
ENSMUSG00000019082	ENSMUSG00000021917
ENSMUSG00000019087	ENSMUSG00000021929
ENSMUSG00000019124	ENSMUSG00000021930
ENSMUSG00000019132	ENSMUSG00000021936
ENSMUSG00000019139	ENSMUSG00000021938
ENSMUSG00000019143	ENSMUSG00000021939
ENSMUSG00000019146	ENSMUSG00000021945
ENSMUSG00000019158	ENSMUSG00000021957
ENSMUSG00000019173	ENSMUSG00000021963
ENSMUSG00000019179	ENSMUSG00000021969
ENSMUSG00000019188	ENSMUSG00000021972
ENSMUSG00000019189	ENSMUSG00000021978
ENSMUSG00000019194	ENSMUSG00000021982
ENSMUSG00000019210	ENSMUSG00000021983
ENSMUSG00000019232	ENSMUSG00000021986
ENSMUSG00000019235	ENSMUSG00000021987
ENSMUSG00000019254	ENSMUSG00000021990
ENSMUSG00000019261	ENSMUSG00000021991
ENSMUSG00000019295	ENSMUSG00000021996
ENSMUSG00000019297	ENSMUSG00000022000
ENSMUSG00000019302	ENSMUSG00000022008
ENSMUSG00000019338	ENSMUSG00000022010
ENSMUSG00000019359	ENSMUSG00000022012
ENSMUSG00000019362	ENSMUSG00000022013
ENSMUSG00000019370	ENSMUSG00000022016
ENSMUSG00000019373	ENSMUSG00000022019
ENSMUSG00000019428	ENSMUSG00000022023
ENSMUSG00000019432	ENSMUSG00000022024
ENSMUSG00000019433	ENSMUSG00000022031
ENSMUSG00000019437	ENSMUSG00000022037
ENSMUSG00000019461	ENSMUSG00000022043
ENSMUSG00000019467	ENSMUSG00000022044
ENSMUSG00000019470	ENSMUSG00000022048
ENSMUSG00000019471	ENSMUSG00000022051
ENSMUSG00000019478	ENSMUSG00000022052
ENSMUSG00000019494	ENSMUSG00000022054
ENSMUSG00000019505	ENSMUSG00000022055
ENSMUSG00000019518	ENSMUSG00000022066
ENSMUSG00000019528	ENSMUSG00000022075
ENSMUSG00000019558	ENSMUSG00000022094
ENSMUSG00000019564	ENSMUSG00000022095
ENSMUSG00000019577	ENSMUSG00000022099
ENSMUSG00000019578	ENSMUSG00000022100
ENSMUSG00000019579	ENSMUSG00000022108
ENSMUSG00000019590	ENSMUSG00000022110
ENSMUSG00000019647	ENSMUSG00000022112

ENSMUSG00000019659	ENSMUSG00000022114
ENSMUSG00000019689	ENSMUSG00000022119
ENSMUSG00000019699	ENSMUSG00000022122
ENSMUSG00000019710	ENSMUSG00000022124
ENSMUSG00000019715	ENSMUSG00000022136
ENSMUSG00000019718	ENSMUSG00000022139
ENSMUSG00000019726	ENSMUSG00000022141
ENSMUSG00000019731	ENSMUSG00000022142
ENSMUSG00000019738	ENSMUSG00000022151
ENSMUSG00000019763	ENSMUSG00000022174
ENSMUSG00000019767	ENSMUSG00000022175
ENSMUSG00000019768	ENSMUSG00000022176
ENSMUSG00000019775	ENSMUSG00000022180
ENSMUSG00000019777	ENSMUSG00000022185
ENSMUSG00000019782	ENSMUSG00000022186
ENSMUSG00000019785	ENSMUSG00000022191
ENSMUSG00000019790	ENSMUSG00000022193
ENSMUSG00000019791	ENSMUSG00000022194
ENSMUSG00000019794	ENSMUSG00000022195
ENSMUSG00000019795	ENSMUSG00000022197
ENSMUSG00000019796	ENSMUSG00000022199
ENSMUSG00000019797	ENSMUSG00000022200
ENSMUSG00000019802	ENSMUSG00000022201
ENSMUSG00000019803	ENSMUSG00000022205
ENSMUSG00000019804	ENSMUSG00000022208
ENSMUSG00000019806	ENSMUSG00000022211
ENSMUSG00000019809	ENSMUSG00000022212
ENSMUSG00000019810	ENSMUSG00000022214
ENSMUSG00000019814	ENSMUSG00000022223
ENSMUSG00000019817	ENSMUSG00000022228
ENSMUSG00000019818	ENSMUSG00000022231
ENSMUSG00000019820	ENSMUSG00000022234
ENSMUSG00000019822	ENSMUSG00000022237
ENSMUSG00000019823	ENSMUSG00000022240
ENSMUSG00000019826	ENSMUSG00000022253
ENSMUSG00000019828	ENSMUSG00000022255
ENSMUSG00000019831	ENSMUSG00000022257
ENSMUSG00000019837	ENSMUSG00000022261
ENSMUSG00000019841	ENSMUSG00000022263
ENSMUSG00000019843	ENSMUSG00000022265
ENSMUSG00000019849	ENSMUSG00000022270
ENSMUSG00000019852	ENSMUSG00000022272
ENSMUSG00000019854	ENSMUSG00000022280
ENSMUSG00000019856	ENSMUSG00000022283
ENSMUSG00000019857	ENSMUSG00000022285
ENSMUSG00000019861	ENSMUSG00000022292
ENSMUSG00000019863	ENSMUSG00000022295
ENSMUSG00000019864	ENSMUSG00000022296
ENSMUSG00000019868	ENSMUSG00000022305
ENSMUSG00000019872	ENSMUSG00000022307
ENSMUSG00000019873	ENSMUSG00000022311
ENSMUSG00000019874	ENSMUSG00000022312
ENSMUSG00000019877	ENSMUSG00000022314

ENSMUSG00000019878	ENSMUSG00000022321
ENSMUSG00000019883	ENSMUSG00000022324
ENSMUSG00000019888	ENSMUSG00000022332
ENSMUSG00000019889	ENSMUSG00000022336
ENSMUSG00000019890	ENSMUSG00000022338
ENSMUSG00000019891	ENSMUSG00000022340
ENSMUSG00000019892	ENSMUSG00000022342
ENSMUSG00000019894	ENSMUSG00000022350
ENSMUSG00000019897	ENSMUSG00000022351
ENSMUSG00000019899	ENSMUSG00000022353
ENSMUSG00000019906	ENSMUSG00000022354
ENSMUSG00000019907	ENSMUSG00000022358
ENSMUSG00000019916	ENSMUSG00000022361
ENSMUSG00000019920	ENSMUSG00000022365
ENSMUSG00000019923	ENSMUSG00000022377
ENSMUSG00000019927	ENSMUSG00000022378
ENSMUSG00000019943	ENSMUSG00000022387
ENSMUSG00000019947	ENSMUSG00000022389
ENSMUSG00000019948	ENSMUSG00000022390
ENSMUSG00000019951	ENSMUSG00000022391
ENSMUSG00000019952	ENSMUSG00000022394
ENSMUSG00000019960	ENSMUSG00000022400
ENSMUSG00000019961	ENSMUSG00000022403
ENSMUSG00000019966	ENSMUSG00000022412
ENSMUSG00000019969	ENSMUSG00000022415
ENSMUSG00000019970	ENSMUSG00000022416
ENSMUSG00000019971	ENSMUSG00000022420
ENSMUSG00000019975	ENSMUSG00000022421
ENSMUSG00000019977	ENSMUSG00000022425
ENSMUSG00000019978	ENSMUSG00000022426
ENSMUSG00000019979	ENSMUSG00000022427
ENSMUSG00000019984	ENSMUSG00000022428
ENSMUSG00000019986	ENSMUSG00000022433
ENSMUSG00000019990	ENSMUSG00000022437
ENSMUSG00000019996	ENSMUSG00000022442
ENSMUSG00000019998	ENSMUSG00000022443
ENSMUSG00000020000	ENSMUSG00000022450
ENSMUSG00000020003	ENSMUSG00000022451
ENSMUSG00000020009	ENSMUSG00000022452
ENSMUSG00000020014	ENSMUSG00000022454
ENSMUSG00000020015	ENSMUSG00000022456
ENSMUSG00000020019	ENSMUSG00000022462
ENSMUSG00000020021	ENSMUSG00000022463
ENSMUSG00000020022	ENSMUSG00000022469
ENSMUSG00000020023	ENSMUSG00000022472
ENSMUSG00000020024	ENSMUSG00000022474
ENSMUSG00000020027	ENSMUSG00000022476
ENSMUSG00000020029	ENSMUSG00000022477
ENSMUSG00000020032	ENSMUSG00000022489
ENSMUSG00000020034	ENSMUSG00000022490
ENSMUSG00000020037	ENSMUSG00000022494
ENSMUSG00000020038	ENSMUSG00000022498
ENSMUSG00000020042	ENSMUSG00000022507

ENSMUSG00000020044	ENSMUSG00000022515
ENSMUSG00000020048	ENSMUSG00000022516
ENSMUSG00000020056	ENSMUSG00000022517
ENSMUSG00000020063	ENSMUSG00000022521
ENSMUSG00000020064	ENSMUSG00000022523
ENSMUSG00000020069	ENSMUSG00000022526
ENSMUSG00000020070	ENSMUSG00000022533
ENSMUSG00000020074	ENSMUSG00000022536
ENSMUSG00000020075	ENSMUSG00000022537
ENSMUSG00000020076	ENSMUSG00000022540
ENSMUSG00000020078	ENSMUSG00000022545
ENSMUSG00000020079	ENSMUSG00000022548
ENSMUSG00000020083	ENSMUSG00000022551
ENSMUSG00000020085	ENSMUSG00000022552
ENSMUSG00000020086	ENSMUSG00000022553
ENSMUSG00000020087	ENSMUSG00000022556
ENSMUSG00000020088	ENSMUSG00000022557
ENSMUSG00000020089	ENSMUSG00000022558
ENSMUSG00000020090	ENSMUSG00000022561
ENSMUSG00000020091	ENSMUSG00000022564
ENSMUSG00000020092	ENSMUSG00000022565
ENSMUSG00000020097	ENSMUSG00000022568
ENSMUSG00000020098	ENSMUSG00000022577
ENSMUSG00000020099	ENSMUSG00000022587
ENSMUSG00000020100	ENSMUSG00000022594
ENSMUSG00000020101	ENSMUSG00000022601
ENSMUSG00000020102	ENSMUSG00000022602
ENSMUSG00000020105	ENSMUSG00000022607
ENSMUSG00000020107	ENSMUSG00000022619
ENSMUSG00000020108	ENSMUSG00000022620
ENSMUSG00000020109	ENSMUSG00000022623
ENSMUSG00000020111	ENSMUSG00000022629
ENSMUSG00000020114	ENSMUSG00000022634
ENSMUSG00000020115	ENSMUSG00000022636
ENSMUSG00000020116	ENSMUSG00000022641
ENSMUSG00000020120	ENSMUSG00000022658
ENSMUSG00000020121	ENSMUSG00000022661
ENSMUSG00000020122	ENSMUSG00000022672
ENSMUSG00000020124	ENSMUSG00000022674
ENSMUSG00000020128	ENSMUSG00000022677
ENSMUSG00000020130	ENSMUSG00000022679
ENSMUSG00000020132	ENSMUSG00000022680
ENSMUSG00000020133	ENSMUSG00000022682
ENSMUSG00000020134	ENSMUSG00000022684
ENSMUSG00000020135	ENSMUSG00000022698
ENSMUSG00000020137	ENSMUSG00000022702
ENSMUSG00000020142	ENSMUSG00000022708
ENSMUSG00000020149	ENSMUSG00000022710
ENSMUSG00000020150	ENSMUSG00000022718
ENSMUSG00000020151	ENSMUSG00000022721
ENSMUSG00000020152	ENSMUSG00000022723
ENSMUSG00000020153	ENSMUSG00000022742
ENSMUSG00000020154	ENSMUSG00000022744

ENSMUSG00000020156	ENSMUSG00000022749
ENSMUSG00000020160	ENSMUSG00000022750
ENSMUSG00000020163	ENSMUSG00000022752
ENSMUSG00000020166	ENSMUSG00000022756
ENSMUSG00000020167	ENSMUSG00000022757
ENSMUSG00000020170	ENSMUSG00000022761
ENSMUSG00000020171	ENSMUSG00000022762
ENSMUSG00000020173	ENSMUSG00000022763
ENSMUSG00000020175	ENSMUSG00000022770
ENSMUSG00000020176	ENSMUSG00000022771
ENSMUSG00000020178	ENSMUSG00000022772
ENSMUSG00000020180	ENSMUSG00000022774
ENSMUSG00000020181	ENSMUSG00000022779
ENSMUSG00000020182	ENSMUSG00000022781
ENSMUSG00000020183	ENSMUSG00000022788
ENSMUSG00000020184	ENSMUSG00000022789
ENSMUSG00000020186	ENSMUSG00000022790
ENSMUSG00000020189	ENSMUSG00000022791
ENSMUSG00000020190	ENSMUSG00000022797
ENSMUSG00000020196	ENSMUSG00000022799
ENSMUSG00000020198	ENSMUSG00000022800
ENSMUSG00000020205	ENSMUSG00000022801
ENSMUSG00000020211	ENSMUSG00000022811
ENSMUSG00000020212	ENSMUSG00000022812
ENSMUSG00000020219	ENSMUSG00000022816
ENSMUSG00000020220	ENSMUSG00000022817
ENSMUSG00000020224	ENSMUSG00000022820
ENSMUSG00000020225	ENSMUSG00000022822
ENSMUSG00000020228	ENSMUSG00000022829
ENSMUSG00000020230	ENSMUSG00000022840
ENSMUSG00000020231	ENSMUSG00000022841
ENSMUSG00000020232	ENSMUSG00000022842
ENSMUSG00000020235	ENSMUSG00000022843
ENSMUSG00000020238	ENSMUSG00000022848
ENSMUSG00000020241	ENSMUSG00000022855
ENSMUSG00000020246	ENSMUSG00000022858
ENSMUSG00000020248	ENSMUSG00000022861
ENSMUSG00000020250	ENSMUSG00000022883
ENSMUSG00000020253	ENSMUSG00000022884
ENSMUSG00000020255	ENSMUSG00000022890
ENSMUSG00000020257	ENSMUSG00000022892
ENSMUSG00000020258	ENSMUSG00000022895
ENSMUSG00000020260	ENSMUSG00000022897
ENSMUSG00000020261	ENSMUSG00000022905
ENSMUSG00000020262	ENSMUSG00000022914
ENSMUSG00000020263	ENSMUSG00000022946
ENSMUSG00000020265	ENSMUSG00000022947
ENSMUSG00000020267	ENSMUSG00000022951
ENSMUSG00000020268	ENSMUSG00000022956
ENSMUSG00000020271	ENSMUSG00000022957
ENSMUSG00000020272	ENSMUSG00000022961
ENSMUSG00000020273	ENSMUSG00000022962
ENSMUSG00000020277	ENSMUSG00000022964

ENSMUSG00000020280	ENSMUSG00000022967
ENSMUSG00000020282	ENSMUSG00000022973
ENSMUSG00000020283	ENSMUSG00000022974
ENSMUSG00000020284	ENSMUSG00000022982
ENSMUSG00000020287	ENSMUSG00000022983
ENSMUSG00000020288	ENSMUSG00000022992
ENSMUSG00000020289	ENSMUSG00000022995
ENSMUSG00000020290	ENSMUSG00000023004
ENSMUSG00000020297	ENSMUSG00000023007
ENSMUSG00000020300	ENSMUSG00000023009
ENSMUSG00000020305	ENSMUSG00000023010
ENSMUSG00000020307	ENSMUSG00000023011
ENSMUSG00000020308	ENSMUSG00000023017
ENSMUSG00000020311	ENSMUSG00000023018
ENSMUSG00000020312	ENSMUSG00000023019
ENSMUSG00000020315	ENSMUSG00000023020
ENSMUSG00000020319	ENSMUSG00000023025
ENSMUSG00000020321	ENSMUSG00000023026
ENSMUSG00000020326	ENSMUSG00000023030
ENSMUSG00000020328	ENSMUSG00000023032
ENSMUSG00000020329	ENSMUSG00000023033
ENSMUSG00000020331	ENSMUSG00000023034
ENSMUSG00000020333	ENSMUSG00000023036
ENSMUSG00000020334	ENSMUSG00000023050
ENSMUSG00000020340	ENSMUSG00000023055
ENSMUSG00000020346	ENSMUSG00000023067
ENSMUSG00000020349	ENSMUSG00000023068
ENSMUSG00000020354	ENSMUSG00000023075
ENSMUSG00000020358	ENSMUSG00000023079
ENSMUSG00000020359	ENSMUSG00000023087
ENSMUSG00000020361	ENSMUSG00000023089
ENSMUSG00000020362	ENSMUSG00000023092
ENSMUSG00000020364	ENSMUSG00000023110
ENSMUSG00000020366	ENSMUSG00000023118
ENSMUSG00000020368	ENSMUSG00000023147
ENSMUSG00000020372	ENSMUSG00000023150
ENSMUSG00000020374	ENSMUSG00000023169
ENSMUSG00000020375	ENSMUSG00000023175
ENSMUSG00000020376	ENSMUSG00000023236
ENSMUSG00000020380	ENSMUSG00000023277
ENSMUSG00000020381	ENSMUSG00000023307
ENSMUSG00000020385	ENSMUSG00000023328
ENSMUSG00000020386	ENSMUSG00000023353
ENSMUSG00000020387	ENSMUSG00000023452
ENSMUSG00000020388	ENSMUSG00000023456
ENSMUSG00000020389	ENSMUSG00000023460
ENSMUSG00000020390	ENSMUSG00000023473
ENSMUSG00000020392	ENSMUSG00000023495
ENSMUSG00000020393	ENSMUSG00000023572
ENSMUSG00000020396	ENSMUSG00000023800
ENSMUSG00000020397	ENSMUSG00000023805
ENSMUSG00000020400	ENSMUSG00000023809
ENSMUSG00000020402	ENSMUSG00000023827

ENSMUSG00000020409	ENSMUSG00000023830
ENSMUSG00000020412	ENSMUSG00000023832
ENSMUSG00000020413	ENSMUSG00000023845
ENSMUSG00000020422	ENSMUSG00000023852
ENSMUSG00000020423	ENSMUSG00000023861
ENSMUSG00000020427	ENSMUSG00000023868
ENSMUSG00000020430	ENSMUSG00000023883
ENSMUSG00000020431	ENSMUSG00000023904
ENSMUSG00000020432	ENSMUSG00000023909
ENSMUSG00000020435	ENSMUSG00000023913
ENSMUSG00000020436	ENSMUSG00000023915
ENSMUSG00000020440	ENSMUSG00000023921
ENSMUSG00000020441	ENSMUSG00000023923
ENSMUSG00000020444	ENSMUSG00000023932
ENSMUSG00000020448	ENSMUSG00000023944
ENSMUSG00000020451	ENSMUSG00000023945
ENSMUSG00000020453	ENSMUSG00000023951
ENSMUSG00000020454	ENSMUSG00000023952
ENSMUSG00000020455	ENSMUSG00000023960
ENSMUSG00000020456	ENSMUSG00000023965
ENSMUSG00000020457	ENSMUSG00000023967
ENSMUSG00000020458	ENSMUSG00000023973
ENSMUSG00000020459	ENSMUSG00000023977
ENSMUSG00000020460	ENSMUSG00000023991
ENSMUSG00000020462	ENSMUSG00000024002
ENSMUSG00000020463	ENSMUSG00000024008
ENSMUSG00000020464	ENSMUSG00000024012
ENSMUSG00000020471	ENSMUSG00000024019
ENSMUSG00000020472	ENSMUSG00000024026
ENSMUSG00000020473	ENSMUSG00000024030
ENSMUSG00000020474	ENSMUSG00000024038
ENSMUSG00000020476	ENSMUSG00000024042
ENSMUSG00000020477	ENSMUSG00000024044
ENSMUSG00000020482	ENSMUSG00000024045
ENSMUSG00000020483	ENSMUSG00000024050
ENSMUSG00000020484	ENSMUSG00000024052
ENSMUSG00000020485	ENSMUSG00000024054
ENSMUSG00000020486	ENSMUSG00000024059
ENSMUSG00000020491	ENSMUSG00000024063
ENSMUSG00000020492	ENSMUSG00000024065
ENSMUSG00000020495	ENSMUSG00000024068
ENSMUSG00000020496	ENSMUSG00000024072
ENSMUSG00000020514	ENSMUSG00000024073
ENSMUSG00000020515	ENSMUSG00000024074
ENSMUSG00000020516	ENSMUSG00000024077
ENSMUSG00000020519	ENSMUSG00000024081
ENSMUSG00000020520	ENSMUSG00000024083
ENSMUSG00000020521	ENSMUSG00000024085
ENSMUSG00000020522	ENSMUSG00000024091
ENSMUSG00000020523	ENSMUSG00000024095
ENSMUSG00000020524	ENSMUSG00000024096
ENSMUSG00000020525	ENSMUSG00000024097
ENSMUSG00000020526	ENSMUSG00000024098

ENSMUSG00000020528	ENSMUSG00000024099
ENSMUSG00000020530	ENSMUSG00000024101
ENSMUSG00000020532	ENSMUSG00000024104
ENSMUSG00000020536	ENSMUSG00000024109
ENSMUSG00000020537	ENSMUSG00000024112
ENSMUSG00000020538	ENSMUSG00000024120
ENSMUSG00000020541	ENSMUSG00000024121
ENSMUSG00000020544	ENSMUSG00000024122
ENSMUSG00000020546	ENSMUSG00000024127
ENSMUSG00000020547	ENSMUSG00000024130
ENSMUSG00000020549	ENSMUSG00000024140
ENSMUSG00000020561	ENSMUSG00000024142
ENSMUSG00000020564	ENSMUSG00000024143
ENSMUSG00000020566	ENSMUSG00000024150
ENSMUSG00000020570	ENSMUSG00000024151
ENSMUSG00000020571	ENSMUSG00000024158
ENSMUSG00000020572	ENSMUSG00000024163
ENSMUSG00000020576	ENSMUSG00000024169
ENSMUSG00000020577	ENSMUSG00000024172
ENSMUSG00000020580	ENSMUSG00000024176
ENSMUSG00000020585	ENSMUSG00000024181
ENSMUSG00000020589	ENSMUSG00000024182
ENSMUSG00000020590	ENSMUSG00000024188
ENSMUSG00000020591	ENSMUSG00000024193
ENSMUSG00000020593	ENSMUSG00000024201
ENSMUSG00000020594	ENSMUSG00000024208
ENSMUSG00000020598	ENSMUSG00000024212
ENSMUSG00000020599	ENSMUSG00000024213
ENSMUSG00000020601	ENSMUSG00000024218
ENSMUSG00000020604	ENSMUSG00000024219
ENSMUSG00000020605	ENSMUSG00000024220
ENSMUSG00000020607	ENSMUSG00000024222
ENSMUSG00000020608	ENSMUSG00000024231
ENSMUSG00000020610	ENSMUSG00000024238
ENSMUSG00000020611	ENSMUSG00000024240
ENSMUSG00000020612	ENSMUSG00000024241
ENSMUSG00000020614	ENSMUSG00000024242
ENSMUSG00000020620	ENSMUSG00000024248
ENSMUSG00000020621	ENSMUSG00000024251
ENSMUSG00000020623	ENSMUSG00000024259
ENSMUSG00000020627	ENSMUSG00000024260
ENSMUSG00000020628	ENSMUSG00000024261
ENSMUSG00000020629	ENSMUSG00000024268
ENSMUSG00000020630	ENSMUSG00000024269
ENSMUSG00000020634	ENSMUSG00000024270
ENSMUSG00000020638	ENSMUSG00000024271
ENSMUSG00000020639	ENSMUSG00000024276
ENSMUSG00000020640	ENSMUSG00000024277
ENSMUSG00000020642	ENSMUSG00000024283
ENSMUSG00000020644	ENSMUSG00000024286
ENSMUSG00000020646	ENSMUSG00000024290
ENSMUSG00000020647	ENSMUSG00000024294
ENSMUSG00000020650	ENSMUSG00000024298

ENSMUSG00000020653	ENSMUSG00000024302
ENSMUSG00000020654	ENSMUSG00000024304
ENSMUSG00000020656	ENSMUSG00000024308
ENSMUSG00000020657	ENSMUSG00000024309
ENSMUSG00000020658	ENSMUSG00000024319
ENSMUSG00000020659	ENSMUSG00000024327
ENSMUSG00000020661	ENSMUSG00000024335
ENSMUSG00000020664	ENSMUSG00000024347
ENSMUSG00000020668	ENSMUSG00000024350
ENSMUSG00000020669	ENSMUSG00000024357
ENSMUSG00000020671	ENSMUSG00000024359
ENSMUSG00000020672	ENSMUSG00000024360
ENSMUSG00000020674	ENSMUSG00000024370
ENSMUSG00000020677	ENSMUSG00000024381
ENSMUSG00000020680	ENSMUSG00000024382
ENSMUSG00000020681	ENSMUSG00000024383
ENSMUSG00000020684	ENSMUSG00000024384
ENSMUSG00000020687	ENSMUSG00000024387
ENSMUSG00000020691	ENSMUSG00000024392
ENSMUSG00000020692	ENSMUSG00000024393
ENSMUSG00000020694	ENSMUSG00000024400
ENSMUSG00000020696	ENSMUSG00000024403
ENSMUSG00000020697	ENSMUSG00000024404
ENSMUSG00000020700	ENSMUSG00000024411
ENSMUSG00000020701	ENSMUSG00000024413
ENSMUSG00000020704	ENSMUSG00000024420
ENSMUSG00000020705	ENSMUSG00000024423
ENSMUSG00000020706	ENSMUSG00000024424
ENSMUSG00000020708	ENSMUSG00000024425
ENSMUSG00000020715	ENSMUSG00000024426
ENSMUSG00000020716	ENSMUSG00000024429
ENSMUSG00000020717	ENSMUSG00000024431
ENSMUSG00000020719	ENSMUSG00000024442
ENSMUSG00000020720	ENSMUSG00000024454
ENSMUSG00000020721	ENSMUSG00000024456
ENSMUSG00000020723	ENSMUSG00000024462
ENSMUSG00000020728	ENSMUSG00000024472
ENSMUSG00000020733	ENSMUSG00000024474
ENSMUSG00000020734	ENSMUSG00000024479
ENSMUSG00000020736	ENSMUSG00000024483
ENSMUSG00000020737	ENSMUSG00000024487
ENSMUSG00000020738	ENSMUSG00000024491
ENSMUSG00000020739	ENSMUSG00000024493
ENSMUSG00000020740	ENSMUSG00000024498
ENSMUSG00000020741	ENSMUSG00000024500
ENSMUSG00000020743	ENSMUSG00000024501
ENSMUSG00000020744	ENSMUSG00000024502
ENSMUSG00000020745	ENSMUSG00000024507
ENSMUSG00000020747	ENSMUSG00000024511
ENSMUSG00000020752	ENSMUSG00000024515
ENSMUSG00000020755	ENSMUSG00000024524
ENSMUSG00000020758	ENSMUSG00000024527
ENSMUSG00000020766	ENSMUSG00000024533

ENSMUSG00000020770	ENSMUSG00000024548
ENSMUSG00000020775	ENSMUSG00000024558
ENSMUSG00000020776	ENSMUSG00000024560
ENSMUSG00000020777	ENSMUSG00000024561
ENSMUSG00000020778	ENSMUSG00000024563
ENSMUSG00000020780	ENSMUSG00000024566
ENSMUSG00000020781	ENSMUSG00000024571
ENSMUSG00000020783	ENSMUSG00000024576
ENSMUSG00000020785	ENSMUSG00000024581
ENSMUSG00000020788	ENSMUSG00000024583
ENSMUSG00000020790	ENSMUSG00000024587
ENSMUSG00000020792	ENSMUSG00000024588
ENSMUSG00000020794	ENSMUSG00000024589
ENSMUSG00000020799	ENSMUSG00000024593
ENSMUSG00000020802	ENSMUSG00000024597
ENSMUSG00000020803	ENSMUSG00000024601
ENSMUSG00000020805	ENSMUSG00000024603
ENSMUSG00000020807	ENSMUSG00000024608
ENSMUSG00000020810	ENSMUSG00000024613
ENSMUSG00000020811	ENSMUSG00000024614
ENSMUSG00000020812	ENSMUSG00000024617
ENSMUSG00000020814	ENSMUSG00000024621
ENSMUSG00000020817	ENSMUSG00000024622
ENSMUSG00000020818	ENSMUSG00000024639
ENSMUSG00000020821	ENSMUSG00000024640
ENSMUSG00000020823	ENSMUSG00000024642
ENSMUSG00000020827	ENSMUSG00000024644
ENSMUSG00000020828	ENSMUSG00000024654
ENSMUSG00000020829	ENSMUSG00000024661
ENSMUSG00000020831	ENSMUSG00000024665
ENSMUSG00000020832	ENSMUSG00000024687
ENSMUSG00000020834	ENSMUSG00000024695
ENSMUSG00000020840	ENSMUSG00000024712
ENSMUSG00000020841	ENSMUSG00000024735
ENSMUSG00000020843	ENSMUSG00000024736
ENSMUSG00000020844	ENSMUSG00000024740
ENSMUSG00000020846	ENSMUSG00000024743
ENSMUSG00000020848	ENSMUSG00000024750
ENSMUSG00000020849	ENSMUSG00000024758
ENSMUSG00000020850	ENSMUSG00000024759
ENSMUSG00000020857	ENSMUSG00000024764
ENSMUSG00000020859	ENSMUSG00000024767
ENSMUSG00000020863	ENSMUSG00000024772
ENSMUSG00000020864	ENSMUSG00000024773
ENSMUSG00000020866	ENSMUSG00000024777
ENSMUSG00000020868	ENSMUSG00000024780
ENSMUSG00000020869	ENSMUSG00000024782
ENSMUSG00000020873	ENSMUSG00000024787
ENSMUSG00000020876	ENSMUSG00000024789
ENSMUSG00000020877	ENSMUSG00000024797
ENSMUSG00000020882	ENSMUSG00000024807
ENSMUSG00000020883	ENSMUSG00000024811
ENSMUSG00000020886	ENSMUSG00000024812

ENSMUSG00000020888	ENSMUSG00000024817
ENSMUSG00000020889	ENSMUSG00000024826
ENSMUSG00000020892	ENSMUSG00000024835
ENSMUSG00000020893	ENSMUSG00000024843
ENSMUSG00000020894	ENSMUSG00000024847
ENSMUSG00000020895	ENSMUSG00000024851
ENSMUSG00000020898	ENSMUSG00000024853
ENSMUSG00000020899	ENSMUSG00000024855
ENSMUSG00000020900	ENSMUSG00000024858
ENSMUSG00000020902	ENSMUSG00000024862
ENSMUSG00000020903	ENSMUSG00000024870
ENSMUSG00000020904	ENSMUSG00000024873
ENSMUSG00000020910	ENSMUSG00000024883
ENSMUSG00000020917	ENSMUSG00000024892
ENSMUSG00000020918	ENSMUSG00000024896
ENSMUSG00000020919	ENSMUSG00000024897
ENSMUSG00000020921	ENSMUSG00000024901
ENSMUSG00000020922	ENSMUSG00000024908
ENSMUSG00000020923	ENSMUSG00000024911
ENSMUSG00000020925	ENSMUSG00000024914
ENSMUSG00000020926	ENSMUSG00000024921
ENSMUSG00000020929	ENSMUSG00000024924
ENSMUSG00000020932	ENSMUSG00000024926
ENSMUSG00000020935	ENSMUSG00000024935
ENSMUSG00000020936	ENSMUSG00000024937
ENSMUSG00000020937	ENSMUSG00000024940
ENSMUSG00000020941	ENSMUSG00000024941
ENSMUSG00000020946	ENSMUSG00000024943
ENSMUSG00000020948	ENSMUSG00000024944
ENSMUSG00000020949	ENSMUSG00000024947
ENSMUSG00000020950	ENSMUSG00000024948
ENSMUSG00000020952	ENSMUSG00000024949
ENSMUSG00000020953	ENSMUSG00000024952
ENSMUSG00000020954	ENSMUSG00000024953
ENSMUSG00000020955	ENSMUSG00000024955
ENSMUSG00000020956	ENSMUSG00000024958
ENSMUSG00000020961	ENSMUSG00000024962
ENSMUSG00000020962	ENSMUSG00000024966
ENSMUSG00000020964	ENSMUSG00000024969
ENSMUSG00000020973	ENSMUSG00000024974
ENSMUSG00000020978	ENSMUSG00000024975
ENSMUSG00000020982	ENSMUSG00000024976
ENSMUSG00000020986	ENSMUSG00000024978
ENSMUSG00000020988	ENSMUSG00000024983
ENSMUSG00000020990	ENSMUSG00000024991
ENSMUSG00000020993	ENSMUSG00000024997
ENSMUSG00000020994	ENSMUSG00000024998
ENSMUSG00000021000	ENSMUSG00000025006
ENSMUSG00000021003	ENSMUSG00000025007
ENSMUSG00000021007	ENSMUSG00000025016
ENSMUSG00000021009	ENSMUSG00000025019
ENSMUSG00000021010	ENSMUSG00000025020
ENSMUSG00000021012	ENSMUSG00000025025

ENSMUSG00000021013	ENSMUSG00000025026
ENSMUSG00000021018	ENSMUSG00000025027
ENSMUSG00000021022	ENSMUSG00000025034
ENSMUSG00000021023	ENSMUSG00000025035
ENSMUSG00000021024	ENSMUSG00000025041
ENSMUSG00000021025	ENSMUSG00000025047
ENSMUSG00000021027	ENSMUSG00000025060
ENSMUSG00000021028	ENSMUSG00000025066
ENSMUSG00000021032	ENSMUSG00000025078
ENSMUSG00000021033	ENSMUSG00000025085
ENSMUSG00000021036	ENSMUSG00000025089
ENSMUSG00000021037	ENSMUSG00000025092
ENSMUSG00000021038	ENSMUSG00000025103
ENSMUSG00000021039	ENSMUSG00000025104
ENSMUSG00000021040	ENSMUSG00000025130
ENSMUSG00000021044	ENSMUSG00000025132
ENSMUSG00000021047	ENSMUSG00000025133
ENSMUSG00000021048	ENSMUSG00000025134
ENSMUSG00000021051	ENSMUSG00000025135
ENSMUSG00000021054	ENSMUSG00000025137
ENSMUSG00000021057	ENSMUSG00000025139
ENSMUSG00000021061	ENSMUSG00000025142
ENSMUSG00000021062	ENSMUSG00000025151
ENSMUSG00000021065	ENSMUSG00000025153
ENSMUSG00000021066	ENSMUSG00000025156
ENSMUSG00000021067	ENSMUSG00000025158
ENSMUSG00000021068	ENSMUSG00000025159
ENSMUSG00000021069	ENSMUSG00000025162
ENSMUSG00000021071	ENSMUSG00000025170
ENSMUSG00000021072	ENSMUSG00000025173
ENSMUSG00000021076	ENSMUSG00000025178
ENSMUSG00000021079	ENSMUSG00000025189
ENSMUSG00000021087	ENSMUSG00000025190
ENSMUSG00000021090	ENSMUSG00000025195
ENSMUSG00000021091	ENSMUSG00000025198
ENSMUSG00000021094	ENSMUSG00000025203
ENSMUSG00000021096	ENSMUSG00000025204
ENSMUSG00000021097	ENSMUSG00000025208
ENSMUSG00000021102	ENSMUSG00000025212
ENSMUSG00000021103	ENSMUSG00000025217
ENSMUSG00000021108	ENSMUSG00000025220
ENSMUSG00000021109	ENSMUSG00000025221
ENSMUSG00000021111	ENSMUSG00000025223
ENSMUSG00000021112	ENSMUSG00000025224
ENSMUSG00000021113	ENSMUSG00000025227
ENSMUSG00000021114	ENSMUSG00000025228
ENSMUSG00000021115	ENSMUSG00000025232
ENSMUSG00000021116	ENSMUSG00000025234
ENSMUSG00000021124	ENSMUSG00000025235
ENSMUSG00000021127	ENSMUSG00000025237
ENSMUSG00000021130	ENSMUSG00000025240
ENSMUSG00000021131	ENSMUSG00000025241
ENSMUSG00000021133	ENSMUSG00000025245

ENSMUSG00000021134	ENSMUSG00000025246
ENSMUSG00000021136	ENSMUSG00000025255
ENSMUSG00000021139	ENSMUSG00000025261
ENSMUSG00000021140	ENSMUSG00000025262
ENSMUSG00000021143	ENSMUSG00000025264
ENSMUSG00000021144	ENSMUSG00000025266
ENSMUSG00000021147	ENSMUSG00000025268
ENSMUSG00000021149	ENSMUSG00000025272
ENSMUSG00000021156	ENSMUSG00000025277
ENSMUSG00000021171	ENSMUSG00000025278
ENSMUSG00000021177	ENSMUSG00000025280
ENSMUSG00000021178	ENSMUSG00000025290
ENSMUSG00000021179	ENSMUSG00000025314
ENSMUSG00000021180	ENSMUSG00000025318
ENSMUSG00000021182	ENSMUSG00000025321
ENSMUSG00000021185	ENSMUSG00000025323
ENSMUSG00000021186	ENSMUSG00000025326
ENSMUSG00000021188	ENSMUSG00000025332
ENSMUSG00000021189	ENSMUSG00000025337
ENSMUSG00000021190	ENSMUSG00000025340
ENSMUSG00000021192	ENSMUSG00000025351
ENSMUSG00000021193	ENSMUSG00000025354
ENSMUSG00000021194	ENSMUSG00000025362
ENSMUSG00000021196	ENSMUSG00000025364
ENSMUSG00000021198	ENSMUSG00000025369
ENSMUSG00000021203	ENSMUSG00000025372
ENSMUSG00000021209	ENSMUSG00000025373
ENSMUSG00000021215	ENSMUSG00000025374
ENSMUSG00000021217	ENSMUSG00000025375
ENSMUSG00000021218	ENSMUSG00000025381
ENSMUSG00000021219	ENSMUSG00000025393
ENSMUSG00000021222	ENSMUSG00000025402
ENSMUSG00000021223	ENSMUSG00000025404
ENSMUSG00000021224	ENSMUSG00000025410
ENSMUSG00000021226	ENSMUSG00000025413
ENSMUSG00000021234	ENSMUSG00000025417
ENSMUSG00000021235	ENSMUSG00000025421
ENSMUSG00000021236	ENSMUSG00000025422
ENSMUSG00000021238	ENSMUSG00000025423
ENSMUSG00000021240	ENSMUSG00000025427
ENSMUSG00000021241	ENSMUSG00000025428
ENSMUSG00000021242	ENSMUSG00000025437
ENSMUSG00000021243	ENSMUSG00000025439
ENSMUSG00000021244	ENSMUSG00000025451
ENSMUSG00000021245	ENSMUSG00000025465
ENSMUSG00000021248	ENSMUSG00000025468
ENSMUSG00000021250	ENSMUSG00000025474
ENSMUSG00000021252	ENSMUSG00000025475
ENSMUSG00000021253	ENSMUSG00000025477
ENSMUSG00000021254	ENSMUSG00000025478
ENSMUSG00000021256	ENSMUSG00000025485
ENSMUSG00000021257	ENSMUSG00000025486
ENSMUSG00000021258	ENSMUSG00000025487

ENSMUSG00000021259	ENSMUSG00000025495
ENSMUSG00000021260	ENSMUSG00000025499
ENSMUSG00000021262	ENSMUSG00000025503
ENSMUSG00000021264	ENSMUSG00000025508
ENSMUSG00000021265	ENSMUSG00000025512
ENSMUSG00000021266	ENSMUSG00000025531
ENSMUSG00000021268	ENSMUSG00000025544
ENSMUSG00000021270	ENSMUSG00000025551
ENSMUSG00000021271	ENSMUSG00000025555
ENSMUSG00000021273	ENSMUSG00000025558
ENSMUSG00000021275	ENSMUSG00000025571
ENSMUSG00000021276	ENSMUSG00000025575
ENSMUSG00000021277	ENSMUSG00000025576
ENSMUSG00000021279	ENSMUSG00000025579
ENSMUSG00000021282	ENSMUSG00000025580
ENSMUSG00000021285	ENSMUSG00000025582
ENSMUSG00000021286	ENSMUSG00000025583
ENSMUSG00000021287	ENSMUSG00000025607
ENSMUSG00000021288	ENSMUSG00000025608
ENSMUSG00000021290	ENSMUSG00000025609
ENSMUSG00000021294	ENSMUSG00000025613
ENSMUSG00000021301	ENSMUSG00000025630
ENSMUSG00000021302	ENSMUSG00000025646
ENSMUSG00000021303	ENSMUSG00000025651
ENSMUSG00000021306	ENSMUSG00000025656
ENSMUSG00000021311	ENSMUSG00000025658
ENSMUSG00000021313	ENSMUSG00000025666
ENSMUSG00000021314	ENSMUSG00000025702
ENSMUSG00000021318	ENSMUSG00000025728
ENSMUSG00000021326	ENSMUSG00000025730
ENSMUSG00000021327	ENSMUSG00000025733
ENSMUSG00000021338	ENSMUSG00000025735
ENSMUSG00000021339	ENSMUSG00000025737
ENSMUSG00000021340	ENSMUSG00000025738
ENSMUSG00000021357	ENSMUSG00000025743
ENSMUSG00000021360	ENSMUSG00000025745
ENSMUSG00000021361	ENSMUSG00000025757
ENSMUSG00000021363	ENSMUSG00000025764
ENSMUSG00000021364	ENSMUSG00000025777
ENSMUSG00000021365	ENSMUSG00000025780
ENSMUSG00000021366	ENSMUSG00000025781
ENSMUSG00000021368	ENSMUSG00000025782
ENSMUSG00000021371	ENSMUSG00000025786
ENSMUSG00000021373	ENSMUSG00000025790
ENSMUSG00000021374	ENSMUSG00000025791
ENSMUSG00000021375	ENSMUSG00000025793
ENSMUSG00000021377	ENSMUSG00000025794
ENSMUSG00000021379	ENSMUSG00000025809
ENSMUSG00000021385	ENSMUSG00000025812
ENSMUSG00000021392	ENSMUSG00000025816
ENSMUSG00000021395	ENSMUSG00000025823
ENSMUSG00000021400	ENSMUSG00000025825
ENSMUSG00000021411	ENSMUSG00000025854

ENSMUSG00000021413	ENSMUSG00000025855
ENSMUSG00000021417	ENSMUSG00000025858
ENSMUSG00000021420	ENSMUSG00000025860
ENSMUSG00000021427	ENSMUSG00000025862
ENSMUSG00000021428	ENSMUSG00000025867
ENSMUSG00000021431	ENSMUSG00000025868
ENSMUSG00000021432	ENSMUSG00000025871
ENSMUSG00000021448	ENSMUSG00000025872
ENSMUSG00000021451	ENSMUSG00000025873
ENSMUSG00000021453	ENSMUSG00000025876
ENSMUSG00000021458	ENSMUSG00000025880
ENSMUSG00000021460	ENSMUSG00000025885
ENSMUSG00000021461	ENSMUSG00000025889
ENSMUSG00000021466	ENSMUSG00000025892
ENSMUSG00000021468	ENSMUSG00000025899
ENSMUSG00000021470	ENSMUSG00000025905
ENSMUSG00000021474	ENSMUSG00000025907
ENSMUSG00000021476	ENSMUSG00000025909
ENSMUSG00000021477	ENSMUSG00000025917
ENSMUSG00000021478	ENSMUSG00000025920
ENSMUSG00000021481	ENSMUSG00000025931
ENSMUSG00000021482	ENSMUSG00000025939
ENSMUSG00000021483	ENSMUSG00000025949
ENSMUSG00000021484	ENSMUSG00000025950
ENSMUSG00000021486	ENSMUSG00000025958
ENSMUSG00000021488	ENSMUSG00000025959
ENSMUSG00000021493	ENSMUSG00000025964
ENSMUSG00000021494	ENSMUSG00000025967
ENSMUSG00000021495	ENSMUSG00000025968
ENSMUSG00000021497	ENSMUSG00000025969
ENSMUSG00000021500	ENSMUSG00000025978
ENSMUSG00000021501	ENSMUSG00000025980
ENSMUSG00000021504	ENSMUSG00000025982
ENSMUSG00000021508	ENSMUSG00000025986
ENSMUSG00000021510	ENSMUSG00000026000
ENSMUSG00000021514	ENSMUSG00000026014
ENSMUSG00000021518	ENSMUSG00000026018
ENSMUSG00000021519	ENSMUSG00000026019
ENSMUSG00000021520	ENSMUSG00000026020
ENSMUSG00000021532	ENSMUSG00000026021
ENSMUSG00000021534	ENSMUSG00000026024
ENSMUSG00000021536	ENSMUSG00000026028
ENSMUSG00000021537	ENSMUSG00000026032
ENSMUSG00000021540	ENSMUSG00000026034
ENSMUSG00000021541	ENSMUSG00000026074
ENSMUSG00000021546	ENSMUSG00000026077
ENSMUSG00000021548	ENSMUSG00000026080
ENSMUSG00000021549	ENSMUSG00000026082
ENSMUSG00000021550	ENSMUSG00000026083
ENSMUSG00000021552	ENSMUSG00000026090
ENSMUSG00000021555	ENSMUSG00000026103
ENSMUSG00000021556	ENSMUSG00000026109
ENSMUSG00000021557	ENSMUSG00000026110

ENSMUSG00000021559	ENSMUSG00000026112
ENSMUSG00000021573	ENSMUSG00000026113
ENSMUSG00000021576	ENSMUSG00000026116
ENSMUSG00000021577	ENSMUSG00000026123
ENSMUSG00000021578	ENSMUSG00000026127
ENSMUSG00000021583	ENSMUSG00000026131
ENSMUSG00000021587	ENSMUSG00000026135
ENSMUSG00000021589	ENSMUSG00000026150
ENSMUSG00000021591	ENSMUSG00000026155
ENSMUSG00000021594	ENSMUSG00000026158
ENSMUSG00000021595	ENSMUSG00000026159
ENSMUSG00000021596	ENSMUSG00000026163
ENSMUSG00000021597	ENSMUSG00000026179
ENSMUSG00000021598	ENSMUSG00000026181
ENSMUSG00000021606	ENSMUSG00000026185
ENSMUSG00000021607	ENSMUSG00000026192
ENSMUSG00000021608	ENSMUSG00000026193
ENSMUSG00000021610	ENSMUSG00000026201
ENSMUSG00000021613	ENSMUSG00000026202
ENSMUSG00000021614	ENSMUSG00000026203
ENSMUSG00000021615	ENSMUSG00000026204
ENSMUSG00000021621	ENSMUSG00000026207
ENSMUSG00000021629	ENSMUSG00000026209
ENSMUSG00000021635	ENSMUSG00000026213
ENSMUSG00000021638	ENSMUSG00000026219
ENSMUSG00000021639	ENSMUSG00000026223
ENSMUSG00000021645	ENSMUSG00000026229
ENSMUSG00000021646	ENSMUSG00000026234
ENSMUSG00000021647	ENSMUSG00000026235
ENSMUSG00000021650	ENSMUSG00000026238
ENSMUSG00000021660	ENSMUSG00000026239
ENSMUSG00000021661	ENSMUSG00000026245
ENSMUSG00000021662	ENSMUSG00000026247
ENSMUSG00000021665	ENSMUSG00000026249
ENSMUSG00000021666	ENSMUSG00000026254
ENSMUSG00000021668	ENSMUSG00000026259
ENSMUSG00000021669	ENSMUSG00000026260
ENSMUSG00000021670	ENSMUSG00000026270
ENSMUSG00000021671	ENSMUSG00000026275
ENSMUSG00000021676	ENSMUSG00000026277
ENSMUSG00000021681	ENSMUSG00000026280
ENSMUSG00000021684	ENSMUSG00000026289
ENSMUSG00000021686	ENSMUSG00000026305
ENSMUSG00000021687	ENSMUSG00000026309
ENSMUSG00000021690	ENSMUSG00000026311
ENSMUSG00000021692	ENSMUSG00000026312
ENSMUSG00000021693	ENSMUSG00000026313
ENSMUSG00000021694	ENSMUSG00000026317
ENSMUSG00000021699	ENSMUSG00000026319
ENSMUSG00000021700	ENSMUSG00000026335
ENSMUSG00000021701	ENSMUSG00000026339
ENSMUSG00000021702	ENSMUSG00000026341
ENSMUSG00000021703	ENSMUSG00000026344

ENSMUSG00000021704	ENSMUSG00000026349
ENSMUSG00000021706	ENSMUSG00000026353
ENSMUSG00000021708	ENSMUSG00000026356
ENSMUSG00000021709	ENSMUSG00000026360
ENSMUSG00000021710	ENSMUSG00000026361
ENSMUSG00000021711	ENSMUSG00000026374
ENSMUSG00000021712	ENSMUSG00000026384
ENSMUSG00000021715	ENSMUSG00000026385
ENSMUSG00000021716	ENSMUSG00000026393
ENSMUSG00000021719	ENSMUSG00000026409
ENSMUSG00000021720	ENSMUSG00000026411
ENSMUSG00000021721	ENSMUSG00000026421
ENSMUSG00000021725	ENSMUSG00000026424
ENSMUSG00000021728	ENSMUSG00000026425
ENSMUSG00000021730	ENSMUSG00000026426
ENSMUSG00000021731	ENSMUSG00000026434
ENSMUSG00000021733	ENSMUSG00000026436
ENSMUSG00000021737	ENSMUSG00000026439
ENSMUSG00000021738	ENSMUSG00000026442
ENSMUSG00000021745	ENSMUSG00000026443
ENSMUSG00000021748	ENSMUSG00000026447
ENSMUSG00000021750	ENSMUSG00000026452
ENSMUSG00000021752	ENSMUSG00000026455
ENSMUSG00000021754	ENSMUSG00000026457
ENSMUSG00000021756	ENSMUSG00000026458
ENSMUSG00000021759	ENSMUSG00000026463
ENSMUSG00000021763	ENSMUSG00000026466
ENSMUSG00000021764	ENSMUSG00000026469
ENSMUSG00000021767	ENSMUSG00000026470
ENSMUSG00000021770	ENSMUSG00000026473
ENSMUSG00000021771	ENSMUSG00000026478
ENSMUSG00000021772	ENSMUSG00000026482
ENSMUSG00000021773	ENSMUSG00000026489
ENSMUSG00000021774	ENSMUSG00000026490
ENSMUSG00000021775	ENSMUSG00000026491
ENSMUSG00000021779	ENSMUSG00000026494
ENSMUSG00000021782	ENSMUSG00000026496
ENSMUSG00000021785	ENSMUSG00000026499
ENSMUSG00000021786	ENSMUSG00000026509
ENSMUSG00000021792	ENSMUSG00000026510
ENSMUSG00000021794	ENSMUSG00000026516
ENSMUSG00000021796	ENSMUSG00000026526
ENSMUSG00000021806	ENSMUSG00000026527
ENSMUSG00000021807	ENSMUSG00000026553
ENSMUSG00000021809	ENSMUSG00000026554
ENSMUSG00000021810	ENSMUSG00000026558
ENSMUSG00000021811	ENSMUSG00000026563
ENSMUSG00000021814	ENSMUSG00000026565
ENSMUSG00000021816	ENSMUSG00000026568
ENSMUSG00000021819	ENSMUSG00000026571
ENSMUSG00000021820	ENSMUSG00000026576
ENSMUSG00000021823	ENSMUSG00000026585
ENSMUSG00000021824	ENSMUSG00000026587

ENSMUSG00000021830	ENSMUSG00000026594
ENSMUSG00000021831	ENSMUSG00000026596
ENSMUSG00000021832	ENSMUSG00000026600
ENSMUSG00000021838	ENSMUSG00000026608
ENSMUSG00000021840	ENSMUSG00000026615
ENSMUSG00000021843	ENSMUSG00000026617
ENSMUSG00000021846	ENSMUSG00000026618
ENSMUSG00000021848	ENSMUSG00000026620
ENSMUSG00000021852	ENSMUSG00000026623
ENSMUSG00000021866	ENSMUSG00000026626
ENSMUSG00000021868	ENSMUSG00000026634
ENSMUSG00000021870	ENSMUSG00000026640
ENSMUSG00000021877	ENSMUSG00000026641
ENSMUSG00000021879	ENSMUSG00000026643
ENSMUSG00000021890	ENSMUSG00000026657
ENSMUSG00000021891	ENSMUSG00000026663
ENSMUSG00000021892	ENSMUSG00000026667
ENSMUSG00000021893	ENSMUSG00000026670
ENSMUSG00000021895	ENSMUSG00000026678
ENSMUSG00000021900	ENSMUSG00000026696
ENSMUSG00000021901	ENSMUSG00000026701
ENSMUSG00000021904	ENSMUSG00000026718
ENSMUSG00000021905	ENSMUSG00000026721
ENSMUSG00000021906	ENSMUSG00000026737
ENSMUSG00000021910	ENSMUSG00000026739
ENSMUSG00000021911	ENSMUSG00000026740
ENSMUSG00000021913	ENSMUSG00000026743
ENSMUSG00000021916	ENSMUSG00000026748
ENSMUSG00000021917	ENSMUSG00000026750
ENSMUSG00000021918	ENSMUSG00000026753
ENSMUSG00000021928	ENSMUSG00000026755
ENSMUSG00000021929	ENSMUSG00000026764
ENSMUSG00000021930	ENSMUSG00000026771
ENSMUSG00000021936	ENSMUSG00000026773
ENSMUSG00000021938	ENSMUSG00000026775
ENSMUSG00000021939	ENSMUSG00000026781
ENSMUSG00000021943	ENSMUSG00000026782
ENSMUSG00000021945	ENSMUSG00000026787
ENSMUSG00000021947	ENSMUSG00000026788
ENSMUSG00000021948	ENSMUSG00000026790
ENSMUSG00000021951	ENSMUSG00000026792
ENSMUSG00000021952	ENSMUSG00000026797
ENSMUSG00000021957	ENSMUSG00000026811
ENSMUSG00000021959	ENSMUSG00000026812
ENSMUSG00000021962	ENSMUSG00000026817
ENSMUSG00000021963	ENSMUSG00000026819
ENSMUSG00000021967	ENSMUSG00000026821
ENSMUSG00000021969	ENSMUSG00000026825
ENSMUSG00000021972	ENSMUSG00000026827
ENSMUSG00000021973	ENSMUSG00000026830
ENSMUSG00000021974	ENSMUSG00000026833
ENSMUSG00000021975	ENSMUSG00000026842
ENSMUSG00000021978	ENSMUSG00000026843

ENSMUSG00000021981	ENSMUSG00000026848
ENSMUSG00000021982	ENSMUSG00000026851
ENSMUSG00000021983	ENSMUSG00000026853
ENSMUSG00000021986	ENSMUSG00000026854
ENSMUSG00000021987	ENSMUSG00000026858
ENSMUSG00000021990	ENSMUSG00000026860
ENSMUSG00000021991	ENSMUSG00000026864
ENSMUSG00000021993	ENSMUSG00000026867
ENSMUSG00000021994	ENSMUSG00000026869
ENSMUSG00000021996	ENSMUSG00000026872
ENSMUSG00000022000	ENSMUSG00000026878
ENSMUSG00000022003	ENSMUSG00000026879
ENSMUSG00000022008	ENSMUSG00000026883
ENSMUSG00000022009	ENSMUSG00000026885
ENSMUSG00000022010	ENSMUSG00000026889
ENSMUSG00000022012	ENSMUSG00000026890
ENSMUSG00000022013	ENSMUSG00000026895
ENSMUSG00000022016	ENSMUSG00000026904
ENSMUSG00000022018	ENSMUSG00000026914
ENSMUSG00000022019	ENSMUSG00000026915
ENSMUSG00000022020	ENSMUSG00000026918
ENSMUSG00000022023	ENSMUSG00000026923
ENSMUSG00000022024	ENSMUSG00000026924
ENSMUSG00000022031	ENSMUSG00000026926
ENSMUSG00000022035	ENSMUSG00000026927
ENSMUSG00000022037	ENSMUSG00000026930
ENSMUSG00000022040	ENSMUSG00000026932
ENSMUSG00000022043	ENSMUSG00000026933
ENSMUSG00000022044	ENSMUSG00000026944
ENSMUSG00000022048	ENSMUSG00000026959
ENSMUSG00000022051	ENSMUSG00000026965
ENSMUSG00000022052	ENSMUSG00000026977
ENSMUSG00000022054	ENSMUSG00000026987
ENSMUSG00000022055	ENSMUSG00000026991
ENSMUSG00000022064	ENSMUSG00000027002
ENSMUSG00000022066	ENSMUSG00000027006
ENSMUSG00000022075	ENSMUSG00000027007
ENSMUSG00000022076	ENSMUSG00000027010
ENSMUSG00000022089	ENSMUSG00000027011
ENSMUSG00000022090	ENSMUSG00000027012
ENSMUSG00000022091	ENSMUSG00000027014
ENSMUSG00000022092	ENSMUSG00000027016
ENSMUSG00000022094	ENSMUSG00000027030
ENSMUSG00000022095	ENSMUSG00000027035
ENSMUSG00000022096	ENSMUSG00000027067
ENSMUSG00000022098	ENSMUSG00000027087
ENSMUSG00000022099	ENSMUSG00000027091
ENSMUSG00000022100	ENSMUSG00000027104
ENSMUSG00000022103	ENSMUSG00000027108
ENSMUSG00000022105	ENSMUSG00000027109
ENSMUSG00000022106	ENSMUSG00000027122
ENSMUSG00000022108	ENSMUSG00000027130
ENSMUSG00000022109	ENSMUSG00000027131

ENSMUSG00000022110	ENSMUSG00000027134
ENSMUSG00000022111	ENSMUSG00000027162
ENSMUSG00000022112	ENSMUSG00000027170
ENSMUSG00000022114	ENSMUSG00000027175
ENSMUSG00000022119	ENSMUSG00000027176
ENSMUSG00000022120	ENSMUSG00000027177
ENSMUSG00000022122	ENSMUSG00000027180
ENSMUSG00000022124	ENSMUSG00000027184
ENSMUSG00000022131	ENSMUSG00000027185
ENSMUSG00000022132	ENSMUSG00000027189
ENSMUSG00000022136	ENSMUSG00000027193
ENSMUSG00000022139	ENSMUSG00000027194
ENSMUSG00000022141	ENSMUSG00000027195
ENSMUSG00000022142	ENSMUSG00000027198
ENSMUSG00000022150	ENSMUSG00000027199
ENSMUSG00000022151	ENSMUSG00000027200
ENSMUSG00000022159	ENSMUSG00000027201
ENSMUSG00000022160	ENSMUSG00000027206
ENSMUSG00000022174	ENSMUSG00000027210
ENSMUSG00000022175	ENSMUSG00000027220
ENSMUSG00000022176	ENSMUSG00000027221
ENSMUSG00000022179	ENSMUSG00000027223
ENSMUSG00000022180	ENSMUSG00000027244
ENSMUSG00000022185	ENSMUSG00000027247
ENSMUSG00000022186	ENSMUSG00000027248
ENSMUSG00000022191	ENSMUSG00000027253
ENSMUSG00000022193	ENSMUSG00000027254
ENSMUSG00000022194	ENSMUSG00000027255
ENSMUSG00000022195	ENSMUSG00000027270
ENSMUSG00000022197	ENSMUSG00000027272
ENSMUSG00000022199	ENSMUSG00000027273
ENSMUSG00000022200	ENSMUSG00000027282
ENSMUSG00000022201	ENSMUSG00000027288
ENSMUSG00000022203	ENSMUSG00000027291
ENSMUSG00000022204	ENSMUSG00000027296
ENSMUSG00000022205	ENSMUSG00000027298
ENSMUSG00000022208	ENSMUSG00000027303
ENSMUSG00000022210	ENSMUSG00000027304
ENSMUSG00000022211	ENSMUSG00000027309
ENSMUSG00000022212	ENSMUSG00000027312
ENSMUSG00000022214	ENSMUSG00000027316
ENSMUSG00000022216	ENSMUSG00000027327
ENSMUSG00000022217	ENSMUSG00000027332
ENSMUSG00000022223	ENSMUSG00000027333
ENSMUSG00000022228	ENSMUSG00000027339
ENSMUSG00000022231	ENSMUSG00000027340
ENSMUSG00000022234	ENSMUSG00000027346
ENSMUSG00000022235	ENSMUSG00000027347
ENSMUSG00000022237	ENSMUSG00000027349
ENSMUSG00000022240	ENSMUSG00000027350
ENSMUSG00000022241	ENSMUSG00000027351
ENSMUSG00000022244	ENSMUSG00000027363
ENSMUSG00000022246	ENSMUSG00000027365

ENSMUSG00000022247	ENSMUSG00000027366
ENSMUSG00000022248	ENSMUSG00000027367
ENSMUSG00000022253	ENSMUSG00000027374
ENSMUSG00000022255	ENSMUSG00000027375
ENSMUSG00000022257	ENSMUSG00000027394
ENSMUSG00000022261	ENSMUSG00000027397
ENSMUSG00000022262	ENSMUSG00000027400
ENSMUSG00000022263	ENSMUSG00000027404
ENSMUSG00000022265	ENSMUSG00000027405
ENSMUSG00000022270	ENSMUSG00000027406
ENSMUSG00000022272	ENSMUSG00000027411
ENSMUSG00000022280	ENSMUSG00000027419
ENSMUSG00000022283	ENSMUSG00000027422
ENSMUSG00000022285	ENSMUSG00000027423
ENSMUSG00000022292	ENSMUSG00000027425
ENSMUSG00000022295	ENSMUSG00000027429
ENSMUSG00000022296	ENSMUSG00000027430
ENSMUSG00000022297	ENSMUSG00000027433
ENSMUSG00000022299	ENSMUSG00000027438
ENSMUSG00000022300	ENSMUSG00000027439
ENSMUSG00000022305	ENSMUSG00000027447
ENSMUSG00000022306	ENSMUSG00000027455
ENSMUSG00000022307	ENSMUSG00000027457
ENSMUSG00000022311	ENSMUSG00000027465
ENSMUSG00000022312	ENSMUSG00000027466
ENSMUSG00000022313	ENSMUSG00000027472
ENSMUSG00000022314	ENSMUSG00000027475
ENSMUSG00000022321	ENSMUSG00000027479
ENSMUSG00000022323	ENSMUSG00000027495
ENSMUSG00000022324	ENSMUSG00000027499
ENSMUSG00000022329	ENSMUSG00000027500
ENSMUSG00000022332	ENSMUSG00000027502
ENSMUSG00000022335	ENSMUSG00000027506
ENSMUSG00000022336	ENSMUSG00000027508
ENSMUSG00000022337	ENSMUSG00000027519
ENSMUSG00000022338	ENSMUSG00000027520
ENSMUSG00000022339	ENSMUSG00000027522
ENSMUSG00000022340	ENSMUSG00000027523
ENSMUSG00000022342	ENSMUSG00000027525
ENSMUSG00000022350	ENSMUSG00000027531
ENSMUSG00000022351	ENSMUSG00000027533
ENSMUSG00000022353	ENSMUSG00000027540
ENSMUSG00000022354	ENSMUSG00000027544
ENSMUSG00000022358	ENSMUSG00000027546
ENSMUSG00000022359	ENSMUSG00000027562
ENSMUSG00000022360	ENSMUSG00000027566
ENSMUSG00000022361	ENSMUSG00000027573
ENSMUSG00000022364	ENSMUSG00000027575
ENSMUSG00000022365	ENSMUSG00000027581
ENSMUSG00000022370	ENSMUSG00000027582
ENSMUSG00000022376	ENSMUSG00000027584
ENSMUSG00000022377	ENSMUSG00000027589
ENSMUSG00000022378	ENSMUSG00000027593

ENSMUSG00000022382	ENSMUSG00000027597
ENSMUSG00000022386	ENSMUSG00000027598
ENSMUSG00000022387	ENSMUSG00000027599
ENSMUSG00000022389	ENSMUSG00000027602
ENSMUSG00000022390	ENSMUSG00000027603
ENSMUSG00000022391	ENSMUSG00000027605
ENSMUSG00000022394	ENSMUSG00000027612
ENSMUSG00000022400	ENSMUSG00000027613
ENSMUSG00000022401	ENSMUSG00000027618
ENSMUSG00000022403	ENSMUSG00000027620
ENSMUSG00000022404	ENSMUSG00000027624
ENSMUSG00000022407	ENSMUSG00000027628
ENSMUSG00000022412	ENSMUSG00000027630
ENSMUSG00000022414	ENSMUSG00000027634
ENSMUSG00000022415	ENSMUSG00000027642
ENSMUSG00000022416	ENSMUSG00000027646
ENSMUSG00000022419	ENSMUSG00000027649
ENSMUSG00000022420	ENSMUSG00000027651
ENSMUSG00000022421	ENSMUSG00000027652
ENSMUSG00000022425	ENSMUSG00000027660
ENSMUSG00000022426	ENSMUSG00000027663
ENSMUSG00000022427	ENSMUSG00000027665
ENSMUSG00000022428	ENSMUSG00000027668
ENSMUSG00000022433	ENSMUSG00000027669
ENSMUSG00000022434	ENSMUSG00000027673
ENSMUSG00000022436	ENSMUSG00000027674
ENSMUSG00000022437	ENSMUSG00000027677
ENSMUSG00000022438	ENSMUSG00000027678
ENSMUSG00000022441	ENSMUSG00000027680
ENSMUSG00000022442	ENSMUSG00000027692
ENSMUSG00000022443	ENSMUSG00000027698
ENSMUSG00000022449	ENSMUSG00000027706
ENSMUSG00000022450	ENSMUSG00000027708
ENSMUSG00000022451	ENSMUSG00000027710
ENSMUSG00000022452	ENSMUSG00000027742
ENSMUSG00000022453	ENSMUSG00000027746
ENSMUSG00000022454	ENSMUSG00000027763
ENSMUSG00000022456	ENSMUSG00000027765
ENSMUSG00000022462	ENSMUSG00000027770
ENSMUSG00000022463	ENSMUSG00000027774
ENSMUSG00000022466	ENSMUSG00000027777
ENSMUSG00000022469	ENSMUSG00000027782
ENSMUSG00000022471	ENSMUSG00000027784
ENSMUSG00000022472	ENSMUSG00000027797
ENSMUSG00000022474	ENSMUSG00000027799
ENSMUSG00000022475	ENSMUSG00000027804
ENSMUSG00000022476	ENSMUSG00000027805
ENSMUSG00000022477	ENSMUSG00000027806
ENSMUSG00000022488	ENSMUSG00000027810
ENSMUSG00000022489	ENSMUSG00000027823
ENSMUSG00000022490	ENSMUSG00000027827
ENSMUSG00000022494	ENSMUSG00000027828
ENSMUSG00000022498	ENSMUSG00000027834

ENSMUSG00000022500	ENSMUSG00000027849
ENSMUSG00000022503	ENSMUSG00000027852
ENSMUSG00000022505	ENSMUSG00000027854
ENSMUSG00000022507	ENSMUSG00000027858
ENSMUSG00000022508	ENSMUSG00000027864
ENSMUSG00000022514	ENSMUSG00000027865
ENSMUSG00000022515	ENSMUSG00000027878
ENSMUSG00000022516	ENSMUSG00000027879
ENSMUSG00000022517	ENSMUSG00000027881
ENSMUSG00000022519	ENSMUSG00000027889
ENSMUSG00000022521	ENSMUSG00000027893
ENSMUSG00000022523	ENSMUSG00000027894
ENSMUSG00000022525	ENSMUSG00000027933
ENSMUSG00000022526	ENSMUSG00000027940
ENSMUSG00000022528	ENSMUSG00000027942
ENSMUSG00000022529	ENSMUSG00000027944
ENSMUSG00000022533	ENSMUSG00000027950
ENSMUSG00000022536	ENSMUSG00000027951
ENSMUSG00000022537	ENSMUSG00000027963
ENSMUSG00000022538	ENSMUSG00000027965
ENSMUSG00000022540	ENSMUSG00000027993
ENSMUSG00000022545	ENSMUSG00000027998
ENSMUSG00000022546	ENSMUSG00000028004
ENSMUSG00000022548	ENSMUSG00000028005
ENSMUSG00000022550	ENSMUSG00000028020
ENSMUSG00000022551	ENSMUSG00000028029
ENSMUSG00000022552	ENSMUSG00000028030
ENSMUSG00000022553	ENSMUSG00000028032
ENSMUSG00000022554	ENSMUSG00000028033
ENSMUSG00000022555	ENSMUSG00000028034
ENSMUSG00000022556	ENSMUSG00000028035
ENSMUSG00000022557	ENSMUSG00000028039
ENSMUSG00000022558	ENSMUSG00000028041
ENSMUSG00000022559	ENSMUSG00000028049
ENSMUSG00000022560	ENSMUSG00000028053
ENSMUSG00000022561	ENSMUSG00000028059
ENSMUSG00000022562	ENSMUSG00000028060
ENSMUSG00000022564	ENSMUSG00000028062
ENSMUSG00000022565	ENSMUSG00000028064
ENSMUSG00000022568	ENSMUSG00000028078
ENSMUSG00000022570	ENSMUSG00000028080
ENSMUSG00000022571	ENSMUSG00000028081
ENSMUSG00000022574	ENSMUSG00000028086
ENSMUSG00000022577	ENSMUSG00000028096
ENSMUSG00000022580	ENSMUSG00000028102
ENSMUSG00000022587	ENSMUSG00000028106
ENSMUSG00000022594	ENSMUSG00000028107
ENSMUSG00000022601	ENSMUSG00000028124
ENSMUSG00000022602	ENSMUSG00000028126
ENSMUSG00000022604	ENSMUSG00000028127
ENSMUSG00000022607	ENSMUSG00000028132
ENSMUSG00000022614	ENSMUSG00000028134
ENSMUSG00000022617	ENSMUSG00000028136

ENSMUSG00000022619	ENSMUSG00000028137
ENSMUSG00000022620	ENSMUSG00000028138
ENSMUSG00000022621	ENSMUSG00000028140
ENSMUSG00000022623	ENSMUSG00000028149
ENSMUSG00000022629	ENSMUSG00000028152
ENSMUSG00000022634	ENSMUSG00000028156
ENSMUSG00000022635	ENSMUSG00000028161
ENSMUSG00000022636	ENSMUSG00000028176
ENSMUSG00000022637	ENSMUSG00000028180
ENSMUSG00000022639	ENSMUSG00000028184
ENSMUSG00000022641	ENSMUSG00000028194
ENSMUSG00000022656	ENSMUSG00000028207
ENSMUSG00000022658	ENSMUSG00000028222
ENSMUSG00000022661	ENSMUSG00000028226
ENSMUSG00000022663	ENSMUSG00000028233
ENSMUSG00000022664	ENSMUSG00000028234
ENSMUSG00000022665	ENSMUSG00000028243
ENSMUSG00000022668	ENSMUSG00000028245
ENSMUSG00000022671	ENSMUSG00000028246
ENSMUSG00000022672	ENSMUSG00000028248
ENSMUSG00000022673	ENSMUSG00000028249
ENSMUSG00000022674	ENSMUSG00000028266
ENSMUSG00000022677	ENSMUSG00000028274
ENSMUSG00000022678	ENSMUSG00000028277
ENSMUSG00000022679	ENSMUSG00000028278
ENSMUSG00000022680	ENSMUSG00000028284
ENSMUSG00000022681	ENSMUSG00000028289
ENSMUSG00000022682	ENSMUSG00000028309
ENSMUSG00000022684	ENSMUSG00000028328
ENSMUSG00000022685	ENSMUSG00000028330
ENSMUSG00000022687	ENSMUSG00000028333
ENSMUSG00000022696	ENSMUSG00000028347
ENSMUSG00000022698	ENSMUSG00000028351
ENSMUSG00000022702	ENSMUSG00000028354
ENSMUSG00000022704	ENSMUSG00000028367
ENSMUSG00000022706	ENSMUSG00000028381
ENSMUSG00000022707	ENSMUSG00000028385
ENSMUSG00000022708	ENSMUSG00000028393
ENSMUSG00000022710	ENSMUSG00000028397
ENSMUSG00000022711	ENSMUSG00000028399
ENSMUSG00000022718	ENSMUSG00000028402
ENSMUSG00000022721	ENSMUSG00000028403
ENSMUSG00000022722	ENSMUSG00000028405
ENSMUSG00000022723	ENSMUSG00000028409
ENSMUSG00000022724	ENSMUSG00000028410
ENSMUSG00000022742	ENSMUSG00000028412
ENSMUSG00000022744	ENSMUSG00000028414
ENSMUSG00000022748	ENSMUSG00000028416
ENSMUSG00000022749	ENSMUSG00000028419
ENSMUSG00000022750	ENSMUSG00000028423
ENSMUSG00000022751	ENSMUSG00000028426
ENSMUSG00000022752	ENSMUSG00000028430
ENSMUSG00000022756	ENSMUSG00000028431

ENSMUSG00000022757	ENSMUSG00000028433
ENSMUSG00000022759	ENSMUSG00000028434
ENSMUSG00000022760	ENSMUSG00000028436
ENSMUSG00000022761	ENSMUSG00000028437
ENSMUSG00000022762	ENSMUSG00000028439
ENSMUSG00000022763	ENSMUSG00000028444
ENSMUSG00000022765	ENSMUSG00000028445
ENSMUSG00000022769	ENSMUSG00000028447
ENSMUSG00000022770	ENSMUSG00000028452
ENSMUSG00000022771	ENSMUSG00000028454
ENSMUSG00000022772	ENSMUSG00000028455
ENSMUSG00000022773	ENSMUSG00000028458
ENSMUSG00000022774	ENSMUSG00000028465
ENSMUSG00000022779	ENSMUSG00000028466
ENSMUSG00000022781	ENSMUSG00000028467
ENSMUSG00000022783	ENSMUSG00000028468
ENSMUSG00000022787	ENSMUSG00000028469
ENSMUSG00000022788	ENSMUSG00000028478
ENSMUSG00000022789	ENSMUSG00000028479
ENSMUSG00000022790	ENSMUSG00000028484
ENSMUSG00000022791	ENSMUSG00000028488
ENSMUSG00000022792	ENSMUSG00000028495
ENSMUSG00000022793	ENSMUSG00000028496
ENSMUSG00000022797	ENSMUSG00000028514
ENSMUSG00000022799	ENSMUSG00000028517
ENSMUSG00000022800	ENSMUSG00000028518
ENSMUSG00000022801	ENSMUSG00000028519
ENSMUSG00000022802	ENSMUSG00000028522
ENSMUSG00000022805	ENSMUSG00000028524
ENSMUSG00000022807	ENSMUSG00000028525
ENSMUSG00000022808	ENSMUSG00000028528
ENSMUSG00000022811	ENSMUSG00000028530
ENSMUSG00000022812	ENSMUSG00000028532
ENSMUSG00000022814	ENSMUSG00000028541
ENSMUSG00000022816	ENSMUSG00000028542
ENSMUSG00000022817	ENSMUSG00000028552
ENSMUSG00000022820	ENSMUSG00000028556
ENSMUSG00000022822	ENSMUSG00000028557
ENSMUSG00000022827	ENSMUSG00000028559
ENSMUSG00000022828	ENSMUSG00000028565
ENSMUSG00000022829	ENSMUSG00000028568
ENSMUSG00000022836	ENSMUSG00000028572
ENSMUSG00000022837	ENSMUSG00000028577
ENSMUSG00000022840	ENSMUSG00000028580
ENSMUSG00000022841	ENSMUSG00000028582
ENSMUSG00000022842	ENSMUSG00000028603
ENSMUSG00000022843	ENSMUSG00000028613
ENSMUSG00000022848	ENSMUSG00000028618
ENSMUSG00000022855	ENSMUSG00000028622
ENSMUSG00000022856	ENSMUSG00000028633
ENSMUSG00000022858	ENSMUSG00000028634
ENSMUSG00000022860	ENSMUSG00000028639
ENSMUSG00000022861	ENSMUSG00000028645

ENSMUSG00000022863	ENSMUSG00000028646
ENSMUSG00000022864	ENSMUSG00000028648
ENSMUSG00000022865	ENSMUSG00000028649
ENSMUSG00000022867	ENSMUSG00000028656
ENSMUSG00000022883	ENSMUSG00000028657
ENSMUSG00000022884	ENSMUSG00000028668
ENSMUSG00000022885	ENSMUSG00000028669
ENSMUSG00000022887	ENSMUSG00000028670
ENSMUSG00000022889	ENSMUSG00000028676
ENSMUSG00000022890	ENSMUSG00000028677
ENSMUSG00000022892	ENSMUSG00000028684
ENSMUSG00000022893	ENSMUSG00000028691
ENSMUSG00000022895	ENSMUSG00000028692
ENSMUSG00000022897	ENSMUSG00000028698
ENSMUSG00000022898	ENSMUSG00000028700
ENSMUSG00000022899	ENSMUSG00000028701
ENSMUSG00000022905	ENSMUSG00000028703
ENSMUSG00000022911	ENSMUSG00000028710
ENSMUSG00000022912	ENSMUSG00000028719
ENSMUSG00000022913	ENSMUSG00000028729
ENSMUSG00000022914	ENSMUSG00000028737
ENSMUSG00000022935	ENSMUSG00000028745
ENSMUSG00000022940	ENSMUSG00000028756
ENSMUSG00000022946	ENSMUSG00000028757
ENSMUSG00000022947	ENSMUSG00000028758
ENSMUSG00000022948	ENSMUSG00000028759
ENSMUSG00000022949	ENSMUSG00000028760
ENSMUSG00000022951	ENSMUSG00000028772
ENSMUSG00000022956	ENSMUSG00000028779
ENSMUSG00000022957	ENSMUSG00000028782
ENSMUSG00000022960	ENSMUSG00000028785
ENSMUSG00000022961	ENSMUSG00000028788
ENSMUSG00000022962	ENSMUSG00000028789
ENSMUSG00000022964	ENSMUSG00000028790
ENSMUSG00000022965	ENSMUSG00000028793
ENSMUSG00000022967	ENSMUSG00000028796
ENSMUSG00000022971	ENSMUSG00000028797
ENSMUSG00000022972	ENSMUSG00000028798
ENSMUSG00000022973	ENSMUSG00000028803
ENSMUSG00000022974	ENSMUSG00000028804
ENSMUSG00000022978	ENSMUSG00000028809
ENSMUSG00000022982	ENSMUSG00000028811
ENSMUSG00000022983	ENSMUSG00000028820
ENSMUSG00000022987	ENSMUSG00000028821
ENSMUSG00000022992	ENSMUSG00000028822
ENSMUSG00000022994	ENSMUSG00000028826
ENSMUSG00000022995	ENSMUSG00000028830
ENSMUSG00000022999	ENSMUSG00000028832
ENSMUSG00000023004	ENSMUSG00000028833
ENSMUSG00000023007	ENSMUSG00000028838
ENSMUSG00000023008	ENSMUSG00000028842
ENSMUSG00000023009	ENSMUSG00000028843
ENSMUSG00000023010	ENSMUSG00000028847

ENSMUSG00000023011	ENSMUSG00000028849
ENSMUSG00000023015	ENSMUSG00000028851
ENSMUSG00000023017	ENSMUSG00000028854
ENSMUSG00000023018	ENSMUSG00000028857
ENSMUSG00000023019	ENSMUSG00000028863
ENSMUSG00000023020	ENSMUSG00000028868
ENSMUSG00000023021	ENSMUSG00000028869
ENSMUSG00000023022	ENSMUSG00000028878
ENSMUSG00000023025	ENSMUSG00000028879
ENSMUSG00000023026	ENSMUSG00000028882
ENSMUSG00000023027	ENSMUSG00000028886
ENSMUSG00000023030	ENSMUSG00000028890
ENSMUSG00000023032	ENSMUSG00000028906
ENSMUSG00000023033	ENSMUSG00000028911
ENSMUSG00000023034	ENSMUSG00000028917
ENSMUSG00000023036	ENSMUSG00000028920
ENSMUSG00000023044	ENSMUSG00000028926
ENSMUSG00000023046	ENSMUSG00000028927
ENSMUSG00000023048	ENSMUSG00000028931
ENSMUSG00000023050	ENSMUSG00000028932
ENSMUSG00000023051	ENSMUSG00000028936
ENSMUSG00000023055	ENSMUSG00000028937
ENSMUSG00000023066	ENSMUSG00000028944
ENSMUSG00000023067	ENSMUSG00000028945
ENSMUSG00000023068	ENSMUSG00000028949
ENSMUSG00000023072	ENSMUSG00000028953
ENSMUSG00000023075	ENSMUSG00000028954
ENSMUSG00000023079	ENSMUSG00000028957
ENSMUSG00000023087	ENSMUSG00000028959
ENSMUSG00000023088	ENSMUSG00000028960
ENSMUSG00000023089	ENSMUSG00000028961
ENSMUSG00000023092	ENSMUSG00000028962
ENSMUSG00000023094	ENSMUSG00000028964
ENSMUSG00000023104	ENSMUSG00000028969
ENSMUSG00000023106	ENSMUSG00000028973
ENSMUSG00000023110	ENSMUSG00000028974
ENSMUSG00000023118	ENSMUSG00000028975
ENSMUSG00000023143	ENSMUSG00000028986
ENSMUSG00000023147	ENSMUSG00000028988
ENSMUSG00000023150	ENSMUSG00000028991
ENSMUSG00000023156	ENSMUSG00000028995
ENSMUSG00000023169	ENSMUSG00000028998
ENSMUSG00000023170	ENSMUSG00000029001
ENSMUSG00000023175	ENSMUSG00000029004
ENSMUSG00000023186	ENSMUSG00000029009
ENSMUSG00000023191	ENSMUSG00000029014
ENSMUSG00000023192	ENSMUSG00000029016
ENSMUSG00000023235	ENSMUSG00000029017
ENSMUSG00000023236	ENSMUSG00000029020
ENSMUSG00000023259	ENSMUSG00000029028
ENSMUSG00000023262	ENSMUSG00000029030
ENSMUSG00000023266	ENSMUSG00000029033
ENSMUSG00000023272	ENSMUSG00000029036

ENSMUSG00000023277	ENSMUSG00000029038
ENSMUSG00000023284	ENSMUSG00000029048
ENSMUSG00000023286	ENSMUSG00000029050
ENSMUSG00000023307	ENSMUSG00000029053
ENSMUSG00000023328	ENSMUSG00000029054
ENSMUSG00000023330	ENSMUSG00000029055
ENSMUSG00000023348	ENSMUSG00000029056
ENSMUSG00000023353	ENSMUSG00000029059
ENSMUSG00000023367	ENSMUSG00000029060
ENSMUSG00000023391	ENSMUSG00000029062
ENSMUSG00000023452	ENSMUSG00000029063
ENSMUSG00000023456	ENSMUSG00000029064
ENSMUSG00000023460	ENSMUSG00000029068
ENSMUSG00000023473	ENSMUSG00000029071
ENSMUSG00000023495	ENSMUSG00000029076
ENSMUSG00000023571	ENSMUSG00000029088
ENSMUSG00000023572	ENSMUSG00000029093
ENSMUSG00000023707	ENSMUSG00000029095
ENSMUSG00000023723	ENSMUSG00000029101
ENSMUSG00000023764	ENSMUSG00000029103
ENSMUSG00000023791	ENSMUSG00000029104
ENSMUSG00000023800	ENSMUSG00000029106
ENSMUSG00000023805	ENSMUSG00000029108
ENSMUSG00000023806	ENSMUSG00000029110
ENSMUSG00000023809	ENSMUSG00000029119
ENSMUSG00000023826	ENSMUSG00000029120
ENSMUSG00000023827	ENSMUSG00000029121
ENSMUSG00000023830	ENSMUSG00000029126
ENSMUSG00000023832	ENSMUSG00000029128
ENSMUSG00000023845	ENSMUSG00000029131
ENSMUSG00000023852	ENSMUSG00000029135
ENSMUSG00000023861	ENSMUSG00000029141
ENSMUSG00000023868	ENSMUSG00000029145
ENSMUSG00000023883	ENSMUSG00000029146
ENSMUSG00000023885	ENSMUSG00000029147
ENSMUSG00000023904	ENSMUSG00000029148
ENSMUSG00000023909	ENSMUSG00000029152
ENSMUSG00000023911	ENSMUSG00000029153
ENSMUSG00000023912	ENSMUSG00000029156
ENSMUSG00000023913	ENSMUSG00000029166
ENSMUSG00000023915	ENSMUSG00000029167
ENSMUSG00000023921	ENSMUSG00000029168
ENSMUSG00000023923	ENSMUSG00000029169
ENSMUSG00000023927	ENSMUSG00000029175
ENSMUSG00000023932	ENSMUSG00000029176
ENSMUSG00000023938	ENSMUSG00000029190
ENSMUSG00000023939	ENSMUSG00000029191
ENSMUSG00000023942	ENSMUSG00000029192
ENSMUSG00000023944	ENSMUSG00000029196
ENSMUSG00000023951	ENSMUSG00000029198
ENSMUSG00000023952	ENSMUSG00000029202
ENSMUSG00000023953	ENSMUSG00000029203
ENSMUSG00000023960	ENSMUSG00000029207

ENSMUSG00000023961	ENSMUSG00000029211
ENSMUSG00000023964	ENSMUSG00000029212
ENSMUSG00000023965	ENSMUSG00000029213
ENSMUSG00000023966	ENSMUSG00000029221
ENSMUSG00000023967	ENSMUSG00000029223
ENSMUSG00000023971	ENSMUSG00000029227
ENSMUSG00000023972	ENSMUSG00000029231
ENSMUSG00000023973	ENSMUSG00000029238
ENSMUSG00000023977	ENSMUSG00000029245
ENSMUSG00000023980	ENSMUSG00000029247
ENSMUSG00000023984	ENSMUSG00000029250
ENSMUSG00000023988	ENSMUSG00000029267
ENSMUSG00000023990	ENSMUSG00000029291
ENSMUSG00000023991	ENSMUSG00000029309
ENSMUSG00000023994	ENSMUSG00000029310
ENSMUSG00000023999	ENSMUSG00000029312
ENSMUSG00000024002	ENSMUSG00000029319
ENSMUSG00000024006	ENSMUSG00000029326
ENSMUSG00000024007	ENSMUSG00000029328
ENSMUSG00000024008	ENSMUSG00000029330
ENSMUSG00000024011	ENSMUSG00000029345
ENSMUSG00000024012	ENSMUSG00000029348
ENSMUSG00000024013	ENSMUSG00000029359
ENSMUSG00000024018	ENSMUSG00000029361
ENSMUSG00000024019	ENSMUSG00000029364
ENSMUSG00000024026	ENSMUSG00000029385
ENSMUSG00000024027	ENSMUSG00000029390
ENSMUSG00000024030	ENSMUSG00000029392
ENSMUSG00000024033	ENSMUSG00000029403
ENSMUSG00000024036	ENSMUSG00000029404
ENSMUSG00000024037	ENSMUSG00000029405
ENSMUSG00000024038	ENSMUSG00000029406
ENSMUSG00000024039	ENSMUSG00000029407
ENSMUSG00000024042	ENSMUSG00000029408
ENSMUSG00000024044	ENSMUSG00000029419
ENSMUSG00000024045	ENSMUSG00000029420
ENSMUSG00000024048	ENSMUSG00000029422
ENSMUSG00000024050	ENSMUSG00000029426
ENSMUSG00000024052	ENSMUSG00000029430
ENSMUSG00000024054	ENSMUSG00000029432
ENSMUSG00000024055	ENSMUSG00000029434
ENSMUSG00000024059	ENSMUSG00000029436
ENSMUSG00000024063	ENSMUSG00000029438
ENSMUSG00000024064	ENSMUSG00000029439
ENSMUSG00000024065	ENSMUSG00000029447
ENSMUSG00000024066	ENSMUSG00000029449
ENSMUSG00000024067	ENSMUSG00000029455
ENSMUSG00000024068	ENSMUSG00000029458
ENSMUSG00000024069	ENSMUSG00000029461
ENSMUSG00000024070	ENSMUSG00000029462
ENSMUSG00000024072	ENSMUSG00000029463
ENSMUSG00000024073	ENSMUSG00000029465
ENSMUSG00000024074	ENSMUSG00000029466

ENSMUSG00000024076	ENSMUSG00000029467
ENSMUSG00000024077	ENSMUSG00000029471
ENSMUSG00000024078	ENSMUSG00000029472
ENSMUSG00000024079	ENSMUSG00000029474
ENSMUSG00000024081	ENSMUSG00000029475
ENSMUSG00000024082	ENSMUSG00000029478
ENSMUSG00000024083	ENSMUSG00000029482
ENSMUSG00000024084	ENSMUSG00000029500
ENSMUSG00000024085	ENSMUSG00000029501
ENSMUSG00000024091	ENSMUSG00000029502
ENSMUSG00000024095	ENSMUSG00000029505
ENSMUSG00000024096	ENSMUSG00000029512
ENSMUSG00000024097	ENSMUSG00000029516
ENSMUSG00000024098	ENSMUSG00000029518
ENSMUSG00000024099	ENSMUSG00000029538
ENSMUSG00000024101	ENSMUSG00000029544
ENSMUSG00000024104	ENSMUSG00000029547
ENSMUSG00000024109	ENSMUSG00000029550
ENSMUSG00000024112	ENSMUSG00000029559
ENSMUSG00000024120	ENSMUSG00000029563
ENSMUSG00000024121	ENSMUSG00000029571
ENSMUSG00000024122	ENSMUSG00000029577
ENSMUSG00000024127	ENSMUSG00000029578
ENSMUSG00000024130	ENSMUSG00000029580
ENSMUSG00000024132	ENSMUSG00000029581
ENSMUSG00000024135	ENSMUSG00000029587
ENSMUSG00000024137	ENSMUSG00000029592
ENSMUSG00000024140	ENSMUSG00000029598
ENSMUSG00000024142	ENSMUSG00000029599
ENSMUSG00000024143	ENSMUSG00000029602
ENSMUSG00000024146	ENSMUSG00000029603
ENSMUSG00000024150	ENSMUSG00000029608
ENSMUSG00000024151	ENSMUSG00000029613
ENSMUSG00000024158	ENSMUSG00000029614
ENSMUSG00000024160	ENSMUSG00000029616
ENSMUSG00000024163	ENSMUSG00000029621
ENSMUSG00000024165	ENSMUSG00000029623
ENSMUSG00000024169	ENSMUSG00000029624
ENSMUSG00000024170	ENSMUSG00000029629
ENSMUSG00000024172	ENSMUSG00000029632
ENSMUSG00000024175	ENSMUSG00000029634
ENSMUSG00000024176	ENSMUSG00000029635
ENSMUSG00000024180	ENSMUSG00000029636
ENSMUSG00000024181	ENSMUSG00000029638
ENSMUSG00000024182	ENSMUSG00000029640
ENSMUSG00000024186	ENSMUSG00000029642
ENSMUSG00000024187	ENSMUSG00000029647
ENSMUSG00000024188	ENSMUSG00000029648
ENSMUSG00000024190	ENSMUSG00000029649
ENSMUSG00000024191	ENSMUSG00000029651
ENSMUSG00000024193	ENSMUSG00000029655
ENSMUSG00000024194	ENSMUSG00000029657
ENSMUSG00000024197	ENSMUSG00000029672

ENSMUSG00000024201	ENSMUSG00000029673
ENSMUSG00000024206	ENSMUSG00000029674
ENSMUSG00000024208	ENSMUSG00000029681
ENSMUSG00000024211	ENSMUSG00000029684
ENSMUSG00000024212	ENSMUSG00000029686
ENSMUSG00000024213	ENSMUSG00000029701
ENSMUSG00000024217	ENSMUSG00000029705
ENSMUSG00000024218	ENSMUSG00000029708
ENSMUSG00000024219	ENSMUSG00000029712
ENSMUSG00000024220	ENSMUSG00000029713
ENSMUSG00000024222	ENSMUSG00000029714
ENSMUSG00000024228	ENSMUSG00000029722
ENSMUSG00000024231	ENSMUSG00000029723
ENSMUSG00000024234	ENSMUSG00000029726
ENSMUSG00000024236	ENSMUSG00000029729
ENSMUSG00000024238	ENSMUSG00000029752
ENSMUSG00000024240	ENSMUSG00000029757
ENSMUSG00000024241	ENSMUSG00000029763
ENSMUSG00000024242	ENSMUSG00000029765
ENSMUSG00000024245	ENSMUSG00000029767
ENSMUSG00000024247	ENSMUSG00000029769
ENSMUSG00000024248	ENSMUSG00000029772
ENSMUSG00000024251	ENSMUSG00000029775
ENSMUSG00000024253	ENSMUSG00000029776
ENSMUSG00000024258	ENSMUSG00000029777
ENSMUSG00000024259	ENSMUSG00000029778
ENSMUSG00000024260	ENSMUSG00000029781
ENSMUSG00000024261	ENSMUSG00000029787
ENSMUSG00000024268	ENSMUSG00000029804
ENSMUSG00000024269	ENSMUSG00000029817
ENSMUSG00000024270	ENSMUSG00000029819
ENSMUSG00000024271	ENSMUSG00000029823
ENSMUSG00000024273	ENSMUSG00000029833
ENSMUSG00000024276	ENSMUSG00000029836
ENSMUSG00000024277	ENSMUSG00000029838
ENSMUSG00000024283	ENSMUSG00000029840
ENSMUSG00000024286	ENSMUSG00000029861
ENSMUSG00000024287	ENSMUSG00000029869
ENSMUSG00000024290	ENSMUSG00000029878
ENSMUSG00000024292	ENSMUSG00000029916
ENSMUSG00000024293	ENSMUSG00000029918
ENSMUSG00000024294	ENSMUSG00000029920
ENSMUSG00000024298	ENSMUSG00000029922
ENSMUSG00000024299	ENSMUSG00000029924
ENSMUSG00000024302	ENSMUSG00000029992
ENSMUSG00000024304	ENSMUSG00000029993
ENSMUSG00000024308	ENSMUSG00000029998
ENSMUSG00000024309	ENSMUSG00000029999
ENSMUSG00000024312	ENSMUSG00000030000
ENSMUSG00000024317	ENSMUSG00000030002
ENSMUSG00000024319	ENSMUSG00000030007
ENSMUSG00000024325	ENSMUSG00000030016
ENSMUSG00000024327	ENSMUSG00000030019

ENSMUSG00000024330	ENSMUSG00000030020
ENSMUSG00000024335	ENSMUSG00000030029
ENSMUSG00000024339	ENSMUSG00000030043
ENSMUSG00000024346	ENSMUSG00000030045
ENSMUSG00000024347	ENSMUSG00000030056
ENSMUSG00000024350	ENSMUSG00000030057
ENSMUSG00000024357	ENSMUSG00000030058
ENSMUSG00000024359	ENSMUSG00000030059
ENSMUSG00000024360	ENSMUSG00000030061
ENSMUSG00000024369	ENSMUSG00000030062
ENSMUSG00000024370	ENSMUSG00000030067
ENSMUSG00000024376	ENSMUSG00000030075
ENSMUSG00000024378	ENSMUSG00000030077
ENSMUSG00000024381	ENSMUSG00000030079
ENSMUSG00000024382	ENSMUSG00000030082
ENSMUSG00000024383	ENSMUSG00000030083
ENSMUSG00000024384	ENSMUSG00000030084
ENSMUSG00000024387	ENSMUSG00000030086
ENSMUSG00000024392	ENSMUSG00000030088
ENSMUSG00000024393	ENSMUSG00000030091
ENSMUSG00000024395	ENSMUSG00000030093
ENSMUSG00000024400	ENSMUSG00000030095
ENSMUSG00000024403	ENSMUSG00000030096
ENSMUSG00000024404	ENSMUSG00000030102
ENSMUSG00000024410	ENSMUSG00000030103
ENSMUSG00000024411	ENSMUSG00000030105
ENSMUSG00000024413	ENSMUSG00000030120
ENSMUSG00000024414	ENSMUSG00000030122
ENSMUSG00000024420	ENSMUSG00000030123
ENSMUSG00000024421	ENSMUSG00000030126
ENSMUSG00000024422	ENSMUSG00000030127
ENSMUSG00000024423	ENSMUSG00000030134
ENSMUSG00000024424	ENSMUSG00000030138
ENSMUSG00000024425	ENSMUSG00000030161
ENSMUSG00000024426	ENSMUSG00000030168
ENSMUSG00000024427	ENSMUSG00000030172
ENSMUSG00000024429	ENSMUSG00000030180
ENSMUSG00000024431	ENSMUSG00000030201
ENSMUSG00000024436	ENSMUSG00000030204
ENSMUSG00000024437	ENSMUSG00000030207
ENSMUSG00000024442	ENSMUSG00000030209
ENSMUSG00000024446	ENSMUSG00000030213
ENSMUSG00000024451	ENSMUSG00000030216
ENSMUSG00000024454	ENSMUSG00000030223
ENSMUSG00000024456	ENSMUSG00000030224
ENSMUSG00000024457	ENSMUSG00000030226
ENSMUSG00000024462	ENSMUSG00000030231
ENSMUSG00000024472	ENSMUSG00000030232
ENSMUSG00000024474	ENSMUSG00000030235
ENSMUSG00000024477	ENSMUSG00000030246
ENSMUSG00000024479	ENSMUSG00000030256
ENSMUSG00000024480	ENSMUSG00000030257
ENSMUSG00000024483	ENSMUSG00000030265

ENSMUSG00000024486	ENSMUSG00000030268
ENSMUSG00000024487	ENSMUSG00000030275
ENSMUSG00000024491	ENSMUSG00000030279
ENSMUSG00000024493	ENSMUSG00000030282
ENSMUSG00000024498	ENSMUSG00000030283
ENSMUSG00000024500	ENSMUSG00000030284
ENSMUSG00000024501	ENSMUSG00000030286
ENSMUSG00000024502	ENSMUSG00000030287
ENSMUSG00000024507	ENSMUSG00000030298
ENSMUSG00000024511	ENSMUSG00000030301
ENSMUSG00000024513	ENSMUSG00000030302
ENSMUSG00000024515	ENSMUSG00000030306
ENSMUSG00000024516	ENSMUSG00000030307
ENSMUSG00000024524	ENSMUSG00000030310
ENSMUSG00000024525	ENSMUSG00000030313
ENSMUSG00000024527	ENSMUSG00000030323
ENSMUSG00000024528	ENSMUSG00000030327
ENSMUSG00000024530	ENSMUSG00000030329
ENSMUSG00000024533	ENSMUSG00000030330
ENSMUSG00000024534	ENSMUSG00000030335
ENSMUSG00000024535	ENSMUSG00000030337
ENSMUSG00000024539	ENSMUSG00000030350
ENSMUSG00000024542	ENSMUSG00000030352
ENSMUSG00000024544	ENSMUSG00000030357
ENSMUSG00000024548	ENSMUSG00000030374
ENSMUSG00000024553	ENSMUSG00000030376
ENSMUSG00000024556	ENSMUSG00000030397
ENSMUSG00000024558	ENSMUSG00000030401
ENSMUSG00000024560	ENSMUSG00000030410
ENSMUSG00000024561	ENSMUSG00000030411
ENSMUSG00000024563	ENSMUSG00000030421
ENSMUSG00000024565	ENSMUSG00000030428
ENSMUSG00000024566	ENSMUSG00000030432
ENSMUSG00000024570	ENSMUSG00000030435
ENSMUSG00000024571	ENSMUSG00000030447
ENSMUSG00000024576	ENSMUSG00000030451
ENSMUSG00000024579	ENSMUSG00000030452
ENSMUSG00000024580	ENSMUSG00000030465
ENSMUSG00000024581	ENSMUSG00000030515
ENSMUSG00000024583	ENSMUSG00000030516
ENSMUSG00000024587	ENSMUSG00000030518
ENSMUSG00000024588	ENSMUSG00000030519
ENSMUSG00000024589	ENSMUSG00000030530
ENSMUSG00000024590	ENSMUSG00000030533
ENSMUSG00000024592	ENSMUSG00000030541
ENSMUSG00000024593	ENSMUSG00000030554
ENSMUSG00000024594	ENSMUSG00000030557
ENSMUSG00000024597	ENSMUSG00000030583
ENSMUSG00000024598	ENSMUSG00000030584
ENSMUSG00000024601	ENSMUSG00000030591
ENSMUSG00000024603	ENSMUSG00000030592
ENSMUSG00000024604	ENSMUSG00000030600
ENSMUSG00000024608	ENSMUSG00000030603

ENSMUSG00000024613	ENSMUSG00000030605
ENSMUSG00000024614	ENSMUSG00000030621
ENSMUSG00000024617	ENSMUSG00000030638
ENSMUSG00000024620	ENSMUSG00000030643
ENSMUSG00000024621	ENSMUSG00000030647
ENSMUSG00000024622	ENSMUSG00000030654
ENSMUSG00000024639	ENSMUSG00000030655
ENSMUSG00000024640	ENSMUSG00000030657
ENSMUSG00000024642	ENSMUSG00000030660
ENSMUSG00000024644	ENSMUSG00000030662
ENSMUSG00000024646	ENSMUSG00000030663
ENSMUSG00000024647	ENSMUSG00000030678
ENSMUSG00000024654	ENSMUSG00000030682
ENSMUSG00000024660	ENSMUSG00000030683
ENSMUSG00000024661	ENSMUSG00000030685
ENSMUSG00000024663	ENSMUSG00000030688
ENSMUSG00000024664	ENSMUSG00000030689
ENSMUSG00000024665	ENSMUSG00000030691
ENSMUSG00000024666	ENSMUSG00000030695
ENSMUSG00000024668	ENSMUSG00000030697
ENSMUSG00000024683	ENSMUSG00000030701
ENSMUSG00000024687	ENSMUSG00000030704
ENSMUSG00000024695	ENSMUSG00000030706
ENSMUSG00000024712	ENSMUSG00000030707
ENSMUSG00000024713	ENSMUSG00000030718
ENSMUSG00000024725	ENSMUSG00000030729
ENSMUSG00000024726	ENSMUSG00000030731
ENSMUSG00000024732	ENSMUSG00000030733
ENSMUSG00000024735	ENSMUSG00000030738
ENSMUSG00000024736	ENSMUSG00000030739
ENSMUSG00000024740	ENSMUSG00000030741
ENSMUSG00000024742	ENSMUSG00000030744
ENSMUSG00000024743	ENSMUSG00000030747
ENSMUSG00000024750	ENSMUSG00000030751
ENSMUSG00000024754	ENSMUSG00000030753
ENSMUSG00000024758	ENSMUSG00000030754
ENSMUSG00000024759	ENSMUSG00000030757
ENSMUSG00000024764	ENSMUSG00000030759
ENSMUSG00000024766	ENSMUSG00000030763
ENSMUSG00000024767	ENSMUSG00000030770
ENSMUSG00000024769	ENSMUSG00000030772
ENSMUSG00000024772	ENSMUSG00000030774
ENSMUSG00000024773	ENSMUSG00000030779
ENSMUSG00000024776	ENSMUSG00000030788
ENSMUSG00000024777	ENSMUSG00000030795
ENSMUSG00000024780	ENSMUSG00000030802
ENSMUSG00000024781	ENSMUSG00000030805
ENSMUSG00000024782	ENSMUSG00000030806
ENSMUSG00000024785	ENSMUSG00000030811
ENSMUSG00000024787	ENSMUSG00000030815
ENSMUSG00000024789	ENSMUSG00000030816
ENSMUSG00000024790	ENSMUSG00000030824
ENSMUSG00000024792	ENSMUSG00000030835

ENSMUSG00000024797	ENSMUSG00000030839
ENSMUSG00000024798	ENSMUSG00000030842
ENSMUSG00000024799	ENSMUSG00000030844
ENSMUSG00000024800	ENSMUSG00000030846
ENSMUSG00000024805	ENSMUSG00000030849
ENSMUSG00000024807	ENSMUSG00000030850
ENSMUSG00000024810	ENSMUSG00000030852
ENSMUSG00000024811	ENSMUSG00000030854
ENSMUSG00000024812	ENSMUSG00000030861
ENSMUSG00000024816	ENSMUSG00000030868
ENSMUSG00000024817	ENSMUSG00000030869
ENSMUSG00000024824	ENSMUSG00000030870
ENSMUSG00000024826	ENSMUSG00000030872
ENSMUSG00000024827	ENSMUSG00000030876
ENSMUSG00000024829	ENSMUSG00000030879
ENSMUSG00000024830	ENSMUSG00000030880
ENSMUSG00000024831	ENSMUSG00000030881
ENSMUSG00000024835	ENSMUSG00000030884
ENSMUSG00000024841	ENSMUSG00000030890
ENSMUSG00000024843	ENSMUSG00000030894
ENSMUSG00000024844	ENSMUSG00000030905
ENSMUSG00000024845	ENSMUSG00000030930
ENSMUSG00000024847	ENSMUSG00000030934
ENSMUSG00000024851	ENSMUSG00000030942
ENSMUSG00000024853	ENSMUSG00000030956
ENSMUSG00000024854	ENSMUSG00000030967
ENSMUSG00000024855	ENSMUSG00000030982
ENSMUSG00000024856	ENSMUSG00000030986
ENSMUSG00000024858	ENSMUSG00000030987
ENSMUSG00000024862	ENSMUSG00000031007
ENSMUSG00000024867	ENSMUSG00000031010
ENSMUSG00000024870	ENSMUSG00000031012
ENSMUSG00000024873	ENSMUSG00000031021
ENSMUSG00000024875	ENSMUSG00000031026
ENSMUSG00000024883	ENSMUSG00000031028
ENSMUSG00000024885	ENSMUSG00000031029
ENSMUSG00000024887	ENSMUSG00000031059
ENSMUSG00000024889	ENSMUSG00000031060
ENSMUSG00000024891	ENSMUSG00000031065
ENSMUSG00000024892	ENSMUSG00000031066
ENSMUSG00000024896	ENSMUSG00000031068
ENSMUSG00000024897	ENSMUSG00000031078
ENSMUSG00000024899	ENSMUSG00000031095
ENSMUSG00000024900	ENSMUSG00000031099
ENSMUSG00000024901	ENSMUSG00000031105
ENSMUSG00000024902	ENSMUSG00000031134
ENSMUSG00000024906	ENSMUSG00000031137
ENSMUSG00000024907	ENSMUSG00000031144
ENSMUSG00000024908	ENSMUSG00000031148
ENSMUSG00000024909	ENSMUSG00000031149
ENSMUSG00000024911	ENSMUSG00000031153
ENSMUSG00000024913	ENSMUSG00000031154
ENSMUSG00000024914	ENSMUSG00000031155

ENSMUSG00000024921	ENSMUSG00000031157
ENSMUSG00000024924	ENSMUSG00000031161
ENSMUSG00000024925	ENSMUSG00000031166
ENSMUSG00000024926	ENSMUSG00000031167
ENSMUSG00000024927	ENSMUSG00000031169
ENSMUSG00000024935	ENSMUSG00000031176
ENSMUSG00000024937	ENSMUSG00000031202
ENSMUSG00000024939	ENSMUSG00000031214
ENSMUSG00000024940	ENSMUSG00000031227
ENSMUSG00000024941	ENSMUSG00000031229
ENSMUSG00000024942	ENSMUSG00000031231
ENSMUSG00000024943	ENSMUSG00000031242
ENSMUSG00000024944	ENSMUSG00000031246
ENSMUSG00000024947	ENSMUSG00000031256
ENSMUSG00000024948	ENSMUSG00000031278
ENSMUSG00000024949	ENSMUSG00000031284
ENSMUSG00000024952	ENSMUSG00000031292
ENSMUSG00000024953	ENSMUSG00000031299
ENSMUSG00000024955	ENSMUSG00000031302
ENSMUSG00000024958	ENSMUSG00000031309
ENSMUSG00000024959	ENSMUSG00000031310
ENSMUSG00000024960	ENSMUSG00000031311
ENSMUSG00000024962	ENSMUSG00000031314
ENSMUSG00000024963	ENSMUSG00000031320
ENSMUSG00000024966	ENSMUSG00000031327
ENSMUSG00000024969	ENSMUSG00000031328
ENSMUSG00000024970	ENSMUSG00000031333
ENSMUSG00000024974	ENSMUSG00000031342
ENSMUSG00000024975	ENSMUSG00000031343
ENSMUSG00000024976	ENSMUSG00000031353
ENSMUSG00000024978	ENSMUSG00000031355
ENSMUSG00000024981	ENSMUSG00000031365
ENSMUSG00000024982	ENSMUSG00000031367
ENSMUSG00000024983	ENSMUSG00000031370
ENSMUSG00000024985	ENSMUSG00000031376
ENSMUSG00000024990	ENSMUSG00000031386
ENSMUSG00000024991	ENSMUSG00000031391
ENSMUSG00000024993	ENSMUSG00000031392
ENSMUSG00000024997	ENSMUSG00000031393
ENSMUSG00000024998	ENSMUSG00000031400
ENSMUSG00000024999	ENSMUSG00000031402
ENSMUSG00000025006	ENSMUSG00000031409
ENSMUSG00000025007	ENSMUSG00000031422
ENSMUSG00000025008	ENSMUSG00000031425
ENSMUSG00000025010	ENSMUSG00000031428
ENSMUSG00000025016	ENSMUSG00000031431
ENSMUSG00000025019	ENSMUSG00000031432
ENSMUSG00000025020	ENSMUSG00000031441
ENSMUSG00000025024	ENSMUSG00000031442
ENSMUSG00000025025	ENSMUSG00000031446
ENSMUSG00000025026	ENSMUSG00000031447
ENSMUSG00000025027	ENSMUSG00000031451
ENSMUSG00000025034	ENSMUSG00000031453

ENSMUSG00000025035	ENSMUSG00000031467
ENSMUSG00000025036	ENSMUSG00000031483
ENSMUSG00000025037	ENSMUSG00000031485
ENSMUSG00000025040	ENSMUSG00000031502
ENSMUSG00000025041	ENSMUSG00000031503
ENSMUSG00000025047	ENSMUSG00000031508
ENSMUSG00000025049	ENSMUSG00000031511
ENSMUSG00000025050	ENSMUSG00000031513
ENSMUSG00000025059	ENSMUSG00000031516
ENSMUSG00000025060	ENSMUSG00000031517
ENSMUSG00000025066	ENSMUSG00000031523
ENSMUSG00000025068	ENSMUSG00000031529
ENSMUSG00000025077	ENSMUSG00000031532
ENSMUSG00000025078	ENSMUSG00000031537
ENSMUSG00000025083	ENSMUSG00000031539
ENSMUSG00000025085	ENSMUSG00000031540
ENSMUSG00000025086	ENSMUSG00000031543
ENSMUSG00000025089	ENSMUSG00000031545
ENSMUSG00000025092	ENSMUSG00000031555
ENSMUSG00000025094	ENSMUSG00000031556
ENSMUSG00000025102	ENSMUSG00000031558
ENSMUSG00000025103	ENSMUSG00000031561
ENSMUSG00000025104	ENSMUSG00000031563
ENSMUSG00000025130	ENSMUSG00000031565
ENSMUSG00000025132	ENSMUSG00000031575
ENSMUSG00000025133	ENSMUSG00000031584
ENSMUSG00000025134	ENSMUSG00000031592
ENSMUSG00000025135	ENSMUSG00000031600
ENSMUSG00000025137	ENSMUSG00000031601
ENSMUSG00000025138	ENSMUSG00000031604
ENSMUSG00000025139	ENSMUSG00000031605
ENSMUSG00000025142	ENSMUSG00000031617
ENSMUSG00000025144	ENSMUSG00000031618
ENSMUSG00000025145	ENSMUSG00000031622
ENSMUSG00000025147	ENSMUSG00000031626
ENSMUSG00000025151	ENSMUSG00000031631
ENSMUSG00000025153	ENSMUSG00000031633
ENSMUSG00000025154	ENSMUSG00000031642
ENSMUSG00000025155	ENSMUSG00000031644
ENSMUSG00000025156	ENSMUSG00000031647
ENSMUSG00000025157	ENSMUSG00000031652
ENSMUSG00000025158	ENSMUSG00000031657
ENSMUSG00000025159	ENSMUSG00000031660
ENSMUSG00000025162	ENSMUSG00000031666
ENSMUSG00000025169	ENSMUSG00000031667
ENSMUSG00000025170	ENSMUSG00000031672
ENSMUSG00000025173	ENSMUSG00000031673
ENSMUSG00000025175	ENSMUSG00000031683
ENSMUSG00000025178	ENSMUSG00000031691
ENSMUSG00000025184	ENSMUSG00000031696
ENSMUSG00000025188	ENSMUSG00000031700
ENSMUSG00000025189	ENSMUSG00000031701
ENSMUSG00000025190	ENSMUSG00000031703

ENSMUSG00000025192	ENSMUSG00000031706
ENSMUSG00000025193	ENSMUSG00000031708
ENSMUSG00000025195	ENSMUSG00000031709
ENSMUSG00000025198	ENSMUSG00000031714
ENSMUSG00000025199	ENSMUSG00000031715
ENSMUSG00000025200	ENSMUSG00000031728
ENSMUSG00000025203	ENSMUSG00000031729
ENSMUSG00000025204	ENSMUSG00000031731
ENSMUSG00000025207	ENSMUSG00000031732
ENSMUSG00000025208	ENSMUSG00000031748
ENSMUSG00000025209	ENSMUSG00000031749
ENSMUSG00000025212	ENSMUSG00000031750
ENSMUSG00000025217	ENSMUSG00000031751
ENSMUSG00000025218	ENSMUSG00000031753
ENSMUSG00000025220	ENSMUSG00000031754
ENSMUSG00000025221	ENSMUSG00000031760
ENSMUSG00000025223	ENSMUSG00000031762
ENSMUSG00000025224	ENSMUSG00000031765
ENSMUSG00000025225	ENSMUSG00000031770
ENSMUSG00000025226	ENSMUSG00000031774
ENSMUSG00000025227	ENSMUSG00000031776
ENSMUSG00000025228	ENSMUSG00000031778
ENSMUSG00000025231	ENSMUSG00000031781
ENSMUSG00000025232	ENSMUSG00000031782
ENSMUSG00000025234	ENSMUSG00000031783
ENSMUSG00000025235	ENSMUSG00000031785
ENSMUSG00000025236	ENSMUSG00000031787
ENSMUSG00000025237	ENSMUSG00000031788
ENSMUSG00000025239	ENSMUSG00000031790
ENSMUSG00000025240	ENSMUSG00000031791
ENSMUSG00000025241	ENSMUSG00000031796
ENSMUSG00000025245	ENSMUSG00000031808
ENSMUSG00000025246	ENSMUSG00000031812
ENSMUSG00000025255	ENSMUSG00000031816
ENSMUSG00000025260	ENSMUSG00000031818
ENSMUSG00000025261	ENSMUSG00000031819
ENSMUSG00000025262	ENSMUSG00000031820
ENSMUSG00000025264	ENSMUSG00000031822
ENSMUSG00000025265	ENSMUSG00000031823
ENSMUSG00000025266	ENSMUSG00000031824
ENSMUSG00000025268	ENSMUSG00000031826
ENSMUSG00000025269	ENSMUSG00000031827
ENSMUSG00000025272	ENSMUSG00000031833
ENSMUSG00000025277	ENSMUSG00000031834
ENSMUSG00000025278	ENSMUSG00000031835
ENSMUSG00000025280	ENSMUSG00000031837
ENSMUSG00000025283	ENSMUSG00000031839
ENSMUSG00000025287	ENSMUSG00000031840
ENSMUSG00000025289	ENSMUSG00000031841
ENSMUSG00000025290	ENSMUSG00000031848
ENSMUSG00000025314	ENSMUSG00000031858
ENSMUSG00000025316	ENSMUSG00000031862
ENSMUSG00000025318	ENSMUSG00000031864

ENSMUSG00000025321	ENSMUSG00000031865
ENSMUSG00000025323	ENSMUSG00000031889
ENSMUSG00000025324	ENSMUSG00000031903
ENSMUSG00000025326	ENSMUSG00000031906
ENSMUSG00000025332	ENSMUSG00000031913
ENSMUSG00000025337	ENSMUSG00000031916
ENSMUSG00000025340	ENSMUSG00000031917
ENSMUSG00000025348	ENSMUSG00000031918
ENSMUSG00000025350	ENSMUSG00000031921
ENSMUSG00000025351	ENSMUSG00000031924
ENSMUSG00000025352	ENSMUSG00000031930
ENSMUSG00000025354	ENSMUSG00000031932
ENSMUSG00000025357	ENSMUSG00000031948
ENSMUSG00000025362	ENSMUSG00000031950
ENSMUSG00000025364	ENSMUSG00000031954
ENSMUSG00000025366	ENSMUSG00000031955
ENSMUSG00000025369	ENSMUSG00000031959
ENSMUSG00000025370	ENSMUSG00000031960
ENSMUSG00000025371	ENSMUSG00000031967
ENSMUSG00000025372	ENSMUSG00000031971
ENSMUSG00000025373	ENSMUSG00000031974
ENSMUSG00000025374	ENSMUSG00000031983
ENSMUSG00000025375	ENSMUSG00000031985
ENSMUSG00000025377	ENSMUSG00000031987
ENSMUSG00000025381	ENSMUSG00000031988
ENSMUSG00000025384	ENSMUSG00000031993
ENSMUSG00000025393	ENSMUSG00000031996
ENSMUSG00000025400	ENSMUSG00000032009
ENSMUSG00000025402	ENSMUSG00000032010
ENSMUSG00000025403	ENSMUSG00000032011
ENSMUSG00000025404	ENSMUSG00000032012
ENSMUSG00000025407	ENSMUSG00000032018
ENSMUSG00000025408	ENSMUSG00000032026
ENSMUSG00000025409	ENSMUSG00000032030
ENSMUSG00000025410	ENSMUSG00000032036
ENSMUSG00000025413	ENSMUSG00000032042
ENSMUSG00000025417	ENSMUSG00000032046
ENSMUSG00000025420	ENSMUSG00000032047
ENSMUSG00000025421	ENSMUSG00000032050
ENSMUSG00000025422	ENSMUSG00000032060
ENSMUSG00000025423	ENSMUSG00000032064
ENSMUSG00000025425	ENSMUSG00000032076
ENSMUSG00000025427	ENSMUSG00000032086
ENSMUSG00000025428	ENSMUSG00000032087
ENSMUSG00000025436	ENSMUSG00000032096
ENSMUSG00000025437	ENSMUSG00000032097
ENSMUSG00000025439	ENSMUSG00000032101
ENSMUSG00000025451	ENSMUSG00000032112
ENSMUSG00000025453	ENSMUSG00000032115
ENSMUSG00000025464	ENSMUSG00000032116
ENSMUSG00000025465	ENSMUSG00000032118
ENSMUSG00000025466	ENSMUSG00000032120
ENSMUSG00000025468	ENSMUSG00000032127

ENSMUSG00000025470	ENSMUSG00000032131
ENSMUSG00000025474	ENSMUSG00000032171
ENSMUSG00000025475	ENSMUSG00000032172
ENSMUSG00000025477	ENSMUSG00000032174
ENSMUSG00000025478	ENSMUSG00000032177
ENSMUSG00000025484	ENSMUSG00000032178
ENSMUSG00000025485	ENSMUSG00000032181
ENSMUSG00000025486	ENSMUSG00000032185
ENSMUSG00000025487	ENSMUSG00000032186
ENSMUSG00000025492	ENSMUSG00000032187
ENSMUSG00000025495	ENSMUSG00000032192
ENSMUSG00000025499	ENSMUSG00000032199
ENSMUSG00000025503	ENSMUSG00000032212
ENSMUSG00000025505	ENSMUSG00000032215
ENSMUSG00000025508	ENSMUSG00000032216
ENSMUSG00000025509	ENSMUSG00000032217
ENSMUSG00000025510	ENSMUSG00000032224
ENSMUSG00000025511	ENSMUSG00000032228
ENSMUSG00000025512	ENSMUSG00000032238
ENSMUSG00000025521	ENSMUSG00000032244
ENSMUSG00000025529	ENSMUSG00000032249
ENSMUSG00000025531	ENSMUSG00000032252
ENSMUSG00000025532	ENSMUSG00000032253
ENSMUSG00000025533	ENSMUSG00000032259
ENSMUSG00000025534	ENSMUSG00000032261
ENSMUSG00000025537	ENSMUSG00000032262
ENSMUSG00000025538	ENSMUSG00000032279
ENSMUSG00000025544	ENSMUSG00000032280
ENSMUSG00000025545	ENSMUSG00000032281
ENSMUSG00000025551	ENSMUSG00000032285
ENSMUSG00000025555	ENSMUSG00000032290
ENSMUSG00000025558	ENSMUSG00000032293
ENSMUSG00000025571	ENSMUSG00000032294
ENSMUSG00000025575	ENSMUSG00000032295
ENSMUSG00000025576	ENSMUSG00000032297
ENSMUSG00000025578	ENSMUSG00000032299
ENSMUSG00000025579	ENSMUSG00000032301
ENSMUSG00000025580	ENSMUSG00000032306
ENSMUSG00000025582	ENSMUSG00000032307
ENSMUSG00000025583	ENSMUSG00000032309
ENSMUSG00000025584	ENSMUSG00000032312
ENSMUSG00000025586	ENSMUSG00000032316
ENSMUSG00000025591	ENSMUSG00000032320
ENSMUSG00000025597	ENSMUSG00000032324
ENSMUSG00000025602	ENSMUSG00000032328
ENSMUSG00000025607	ENSMUSG00000032329
ENSMUSG00000025608	ENSMUSG00000032330
ENSMUSG00000025609	ENSMUSG00000032333
ENSMUSG00000025612	ENSMUSG00000032336
ENSMUSG00000025613	ENSMUSG00000032340
ENSMUSG00000025616	ENSMUSG00000032349
ENSMUSG00000025626	ENSMUSG00000032350
ENSMUSG00000025630	ENSMUSG00000032356

ENSMUSG00000025645	ENSMUSG00000032366
ENSMUSG00000025646	ENSMUSG00000032373
ENSMUSG00000025647	ENSMUSG00000032376
ENSMUSG00000025648	ENSMUSG00000032382
ENSMUSG00000025651	ENSMUSG00000032383
ENSMUSG00000025656	ENSMUSG00000032384
ENSMUSG00000025658	ENSMUSG00000032386
ENSMUSG00000025665	ENSMUSG00000032388
ENSMUSG00000025666	ENSMUSG00000032393
ENSMUSG00000025702	ENSMUSG00000032396
ENSMUSG00000025722	ENSMUSG00000032399
ENSMUSG00000025723	ENSMUSG00000032402
ENSMUSG00000025724	ENSMUSG00000032405
ENSMUSG00000025728	ENSMUSG00000032407
ENSMUSG00000025730	ENSMUSG00000032410
ENSMUSG00000025731	ENSMUSG00000032411
ENSMUSG00000025732	ENSMUSG00000032412
ENSMUSG00000025733	ENSMUSG00000032413
ENSMUSG00000025735	ENSMUSG00000032422
ENSMUSG00000025736	ENSMUSG00000032423
ENSMUSG00000025737	ENSMUSG00000032425
ENSMUSG00000025738	ENSMUSG00000032434
ENSMUSG00000025739	ENSMUSG00000032435
ENSMUSG00000025742	ENSMUSG00000032437
ENSMUSG00000025743	ENSMUSG00000032449
ENSMUSG00000025745	ENSMUSG00000032458
ENSMUSG00000025757	ENSMUSG00000032462
ENSMUSG00000025759	ENSMUSG00000032468
ENSMUSG00000025762	ENSMUSG00000032470
ENSMUSG00000025764	ENSMUSG00000032479
ENSMUSG00000025766	ENSMUSG00000032480
ENSMUSG00000025777	ENSMUSG00000032481
ENSMUSG00000025780	ENSMUSG00000032482
ENSMUSG00000025781	ENSMUSG00000032485
ENSMUSG00000025782	ENSMUSG00000032500
ENSMUSG00000025785	ENSMUSG00000032503
ENSMUSG00000025786	ENSMUSG00000032504
ENSMUSG00000025789	ENSMUSG00000032507
ENSMUSG00000025790	ENSMUSG00000032512
ENSMUSG00000025791	ENSMUSG00000032513
ENSMUSG00000025792	ENSMUSG00000032517
ENSMUSG00000025793	ENSMUSG00000032518
ENSMUSG00000025794	ENSMUSG00000032525
ENSMUSG00000025795	ENSMUSG00000032527
ENSMUSG00000025809	ENSMUSG00000032536
ENSMUSG00000025810	ENSMUSG00000032537
ENSMUSG00000025812	ENSMUSG00000032549
ENSMUSG00000025813	ENSMUSG00000032554
ENSMUSG00000025815	ENSMUSG00000032557
ENSMUSG00000025816	ENSMUSG00000032560
ENSMUSG00000025817	ENSMUSG00000032562
ENSMUSG00000025821	ENSMUSG00000032563
ENSMUSG00000025823	ENSMUSG00000032570

ENSMUSG00000025825	ENSMUSG00000032571
ENSMUSG00000025854	ENSMUSG00000032575
ENSMUSG00000025855	ENSMUSG00000032580
ENSMUSG00000025856	ENSMUSG00000032582
ENSMUSG00000025857	ENSMUSG00000032589
ENSMUSG00000025858	ENSMUSG00000032590
ENSMUSG00000025860	ENSMUSG00000032594
ENSMUSG00000025862	ENSMUSG00000032598
ENSMUSG00000025867	ENSMUSG00000032599
ENSMUSG00000025868	ENSMUSG00000032601
ENSMUSG00000025869	ENSMUSG00000032604
ENSMUSG00000025870	ENSMUSG00000032606
ENSMUSG00000025871	ENSMUSG00000032612
ENSMUSG00000025872	ENSMUSG00000032621
ENSMUSG00000025873	ENSMUSG00000032624
ENSMUSG00000025875	ENSMUSG00000032625
ENSMUSG00000025876	ENSMUSG00000032633
ENSMUSG00000025878	ENSMUSG00000032637
ENSMUSG00000025880	ENSMUSG00000032652
ENSMUSG00000025885	ENSMUSG00000032657
ENSMUSG00000025889	ENSMUSG00000032666
ENSMUSG00000025892	ENSMUSG00000032705
ENSMUSG00000025894	ENSMUSG00000032735
ENSMUSG00000025898	ENSMUSG00000032737
ENSMUSG00000025899	ENSMUSG00000032740
ENSMUSG00000025903	ENSMUSG00000032741
ENSMUSG00000025905	ENSMUSG00000032743
ENSMUSG00000025907	ENSMUSG00000032745
ENSMUSG00000025909	ENSMUSG00000032773
ENSMUSG00000025911	ENSMUSG00000032777
ENSMUSG00000025915	ENSMUSG00000032786
ENSMUSG00000025917	ENSMUSG00000032788
ENSMUSG00000025920	ENSMUSG00000032802
ENSMUSG00000025921	ENSMUSG00000032803
ENSMUSG00000025925	ENSMUSG00000032816
ENSMUSG00000025931	ENSMUSG00000032826
ENSMUSG00000025933	ENSMUSG00000032827
ENSMUSG00000025935	ENSMUSG00000032834
ENSMUSG00000025937	ENSMUSG00000032839
ENSMUSG00000025939	ENSMUSG00000032840
ENSMUSG00000025940	ENSMUSG00000032846
ENSMUSG00000025949	ENSMUSG00000032850
ENSMUSG00000025950	ENSMUSG00000032854
ENSMUSG00000025956	ENSMUSG00000032855
ENSMUSG00000025958	ENSMUSG00000032867
ENSMUSG00000025959	ENSMUSG00000032870
ENSMUSG00000025962	ENSMUSG00000032875
ENSMUSG00000025964	ENSMUSG00000032878
ENSMUSG00000025967	ENSMUSG00000032883
ENSMUSG00000025968	ENSMUSG00000032890
ENSMUSG00000025969	ENSMUSG00000032898
ENSMUSG00000025971	ENSMUSG00000032905
ENSMUSG00000025978	ENSMUSG00000032911

ENSMUSG00000025979	ENSMUSG00000032913
ENSMUSG00000025980	ENSMUSG00000032932
ENSMUSG00000025981	ENSMUSG00000032936
ENSMUSG00000025982	ENSMUSG00000032939
ENSMUSG00000025986	ENSMUSG00000032946
ENSMUSG00000025993	ENSMUSG00000032959
ENSMUSG00000025995	ENSMUSG00000032966
ENSMUSG00000026000	ENSMUSG00000032977
ENSMUSG00000026003	ENSMUSG00000032997
ENSMUSG00000026004	ENSMUSG00000032998
ENSMUSG00000026005	ENSMUSG00000033004
ENSMUSG00000026014	ENSMUSG00000033006
ENSMUSG00000026017	ENSMUSG00000033007
ENSMUSG00000026018	ENSMUSG00000033009
ENSMUSG00000026019	ENSMUSG00000033014
ENSMUSG00000026020	ENSMUSG00000033022
ENSMUSG00000026021	ENSMUSG00000033039
ENSMUSG00000026024	ENSMUSG00000033047
ENSMUSG00000026027	ENSMUSG00000033054
ENSMUSG00000026028	ENSMUSG00000033059
ENSMUSG00000026031	ENSMUSG00000033060
ENSMUSG00000026032	ENSMUSG00000033063
ENSMUSG00000026034	ENSMUSG00000033065
ENSMUSG00000026035	ENSMUSG00000033066
ENSMUSG00000026036	ENSMUSG00000033068
ENSMUSG00000026037	ENSMUSG00000033096
ENSMUSG00000026042	ENSMUSG00000033105
ENSMUSG00000026043	ENSMUSG00000033124
ENSMUSG00000026048	ENSMUSG00000033128
ENSMUSG00000026058	ENSMUSG00000033152
ENSMUSG00000026074	ENSMUSG00000033161
ENSMUSG00000026077	ENSMUSG00000033174
ENSMUSG00000026078	ENSMUSG00000033184
ENSMUSG00000026080	ENSMUSG00000033186
ENSMUSG00000026082	ENSMUSG00000033208
ENSMUSG00000026083	ENSMUSG00000033214
ENSMUSG00000026087	ENSMUSG00000033228
ENSMUSG00000026090	ENSMUSG00000033237
ENSMUSG00000026095	ENSMUSG00000033253
ENSMUSG00000026096	ENSMUSG00000033256
ENSMUSG00000026098	ENSMUSG00000033272
ENSMUSG00000026102	ENSMUSG00000033278
ENSMUSG00000026103	ENSMUSG00000033282
ENSMUSG00000026104	ENSMUSG00000033287
ENSMUSG00000026107	ENSMUSG00000033295
ENSMUSG00000026109	ENSMUSG00000033307
ENSMUSG00000026110	ENSMUSG00000033319
ENSMUSG00000026111	ENSMUSG00000033326
ENSMUSG00000026112	ENSMUSG00000033335
ENSMUSG00000026113	ENSMUSG00000033342
ENSMUSG00000026116	ENSMUSG00000033350
ENSMUSG00000026121	ENSMUSG00000033352
ENSMUSG00000026123	ENSMUSG00000033364

ENSMUSG00000026127	ENSMUSG00000033365
ENSMUSG00000026131	ENSMUSG00000033373
ENSMUSG00000026134	ENSMUSG00000033379
ENSMUSG00000026135	ENSMUSG00000033382
ENSMUSG00000026142	ENSMUSG00000033389
ENSMUSG00000026150	ENSMUSG00000033392
ENSMUSG00000026153	ENSMUSG00000033396
ENSMUSG00000026154	ENSMUSG00000033400
ENSMUSG00000026155	ENSMUSG00000033417
ENSMUSG00000026156	ENSMUSG00000033419
ENSMUSG00000026158	ENSMUSG00000033423
ENSMUSG00000026159	ENSMUSG00000033430
ENSMUSG00000026163	ENSMUSG00000033436
ENSMUSG00000026171	ENSMUSG00000033444
ENSMUSG00000026172	ENSMUSG00000033460
ENSMUSG00000026173	ENSMUSG00000033475
ENSMUSG00000026174	ENSMUSG00000033478
ENSMUSG00000026176	ENSMUSG00000033487
ENSMUSG00000026179	ENSMUSG00000033499
ENSMUSG00000026181	ENSMUSG00000033510
ENSMUSG00000026185	ENSMUSG00000033526
ENSMUSG00000026187	ENSMUSG00000033530
ENSMUSG00000026188	ENSMUSG00000033545
ENSMUSG00000026192	ENSMUSG00000033557
ENSMUSG00000026193	ENSMUSG00000033565
ENSMUSG00000026197	ENSMUSG00000033569
ENSMUSG00000026198	ENSMUSG00000033577
ENSMUSG00000026199	ENSMUSG00000033578
ENSMUSG00000026200	ENSMUSG00000033585
ENSMUSG00000026201	ENSMUSG00000033594
ENSMUSG00000026202	ENSMUSG00000033595
ENSMUSG00000026203	ENSMUSG00000033596
ENSMUSG00000026204	ENSMUSG00000033597
ENSMUSG00000026207	ENSMUSG00000033615
ENSMUSG00000026209	ENSMUSG00000033618
ENSMUSG00000026211	ENSMUSG00000033623
ENSMUSG00000026213	ENSMUSG00000033624
ENSMUSG00000026219	ENSMUSG00000033628
ENSMUSG00000026220	ENSMUSG00000033629
ENSMUSG00000026223	ENSMUSG00000033653
ENSMUSG00000026224	ENSMUSG00000033658
ENSMUSG00000026227	ENSMUSG00000033671
ENSMUSG00000026229	ENSMUSG00000033676
ENSMUSG00000026234	ENSMUSG00000033684
ENSMUSG00000026235	ENSMUSG00000033697
ENSMUSG00000026238	ENSMUSG00000033701
ENSMUSG00000026239	ENSMUSG00000033706
ENSMUSG00000026240	ENSMUSG00000033707
ENSMUSG00000026245	ENSMUSG00000033712
ENSMUSG00000026247	ENSMUSG00000033713
ENSMUSG00000026248	ENSMUSG00000033720
ENSMUSG00000026249	ENSMUSG00000033728
ENSMUSG00000026254	ENSMUSG00000033730

ENSMUSG00000026255	ENSMUSG00000033732
ENSMUSG00000026259	ENSMUSG00000033763
ENSMUSG00000026260	ENSMUSG00000033767
ENSMUSG00000026269	ENSMUSG00000033768
ENSMUSG00000026270	ENSMUSG00000033769
ENSMUSG00000026273	ENSMUSG00000033781
ENSMUSG00000026274	ENSMUSG00000033790
ENSMUSG00000026275	ENSMUSG00000033793
ENSMUSG00000026276	ENSMUSG00000033799
ENSMUSG00000026277	ENSMUSG00000033808
ENSMUSG00000026278	ENSMUSG00000033813
ENSMUSG00000026279	ENSMUSG00000033819
ENSMUSG00000026280	ENSMUSG00000033862
ENSMUSG00000026281	ENSMUSG00000033863
ENSMUSG00000026283	ENSMUSG00000033871
ENSMUSG00000026288	ENSMUSG00000033883
ENSMUSG00000026289	ENSMUSG00000033900
ENSMUSG00000026301	ENSMUSG00000033902
ENSMUSG00000026305	ENSMUSG00000033904
ENSMUSG00000026307	ENSMUSG00000033909
ENSMUSG00000026309	ENSMUSG00000033910
ENSMUSG00000026311	ENSMUSG00000033916
ENSMUSG00000026312	ENSMUSG00000033917
ENSMUSG00000026313	ENSMUSG00000033933
ENSMUSG00000026317	ENSMUSG00000033938
ENSMUSG00000026319	ENSMUSG00000033940
ENSMUSG00000026322	ENSMUSG00000033943
ENSMUSG00000026335	ENSMUSG00000033948
ENSMUSG00000026339	ENSMUSG00000033953
ENSMUSG00000026341	ENSMUSG00000033955
ENSMUSG00000026342	ENSMUSG00000033960
ENSMUSG00000026344	ENSMUSG00000033964
ENSMUSG00000026347	ENSMUSG00000033965
ENSMUSG00000026349	ENSMUSG00000033981
ENSMUSG00000026353	ENSMUSG00000033991
ENSMUSG00000026355	ENSMUSG00000033998
ENSMUSG00000026356	ENSMUSG00000034007
ENSMUSG00000026360	ENSMUSG00000034021
ENSMUSG00000026361	ENSMUSG00000034022
ENSMUSG00000026365	ENSMUSG00000034024
ENSMUSG00000026374	ENSMUSG00000034032
ENSMUSG00000026377	ENSMUSG00000034040
ENSMUSG00000026383	ENSMUSG00000034042
ENSMUSG00000026384	ENSMUSG00000034064
ENSMUSG00000026385	ENSMUSG00000034075
ENSMUSG00000026389	ENSMUSG00000034088
ENSMUSG00000026393	ENSMUSG00000034101
ENSMUSG00000026404	ENSMUSG00000034111
ENSMUSG00000026409	ENSMUSG00000034118
ENSMUSG00000026411	ENSMUSG00000034120
ENSMUSG00000026421	ENSMUSG00000034135
ENSMUSG00000026424	ENSMUSG00000034145
ENSMUSG00000026425	ENSMUSG00000034152

ENSMUSG00000026426	ENSMUSG00000034154
ENSMUSG00000026427	ENSMUSG00000034156
ENSMUSG00000026430	ENSMUSG00000034157
ENSMUSG00000026434	ENSMUSG00000034158
ENSMUSG00000026436	ENSMUSG00000034160
ENSMUSG00000026437	ENSMUSG00000034163
ENSMUSG00000026439	ENSMUSG00000034168
ENSMUSG00000026442	ENSMUSG00000034171
ENSMUSG00000026443	ENSMUSG00000034187
ENSMUSG00000026447	ENSMUSG00000034189
ENSMUSG00000026452	ENSMUSG00000034190
ENSMUSG00000026455	ENSMUSG00000034201
ENSMUSG00000026456	ENSMUSG00000034203
ENSMUSG00000026457	ENSMUSG00000034210
ENSMUSG00000026458	ENSMUSG00000034211
ENSMUSG00000026463	ENSMUSG00000034216
ENSMUSG00000026466	ENSMUSG00000034220
ENSMUSG00000026469	ENSMUSG00000034235
ENSMUSG00000026470	ENSMUSG00000034243
ENSMUSG00000026473	ENSMUSG00000034245
ENSMUSG00000026475	ENSMUSG00000034247
ENSMUSG00000026478	ENSMUSG00000034248
ENSMUSG00000026482	ENSMUSG00000034252
ENSMUSG00000026484	ENSMUSG00000034254
ENSMUSG00000026489	ENSMUSG00000034265
ENSMUSG00000026490	ENSMUSG00000034269
ENSMUSG00000026491	ENSMUSG00000034274
ENSMUSG00000026492	ENSMUSG00000034275
ENSMUSG00000026494	ENSMUSG00000034285
ENSMUSG00000026495	ENSMUSG00000034290
ENSMUSG00000026496	ENSMUSG00000034297
ENSMUSG00000026499	ENSMUSG00000034300
ENSMUSG00000026502	ENSMUSG00000034310
ENSMUSG00000026504	ENSMUSG00000034312
ENSMUSG00000026509	ENSMUSG00000034336
ENSMUSG00000026510	ENSMUSG00000034341
ENSMUSG00000026511	ENSMUSG00000034342
ENSMUSG00000026514	ENSMUSG00000034343
ENSMUSG00000026516	ENSMUSG00000034345
ENSMUSG00000026519	ENSMUSG00000034353
ENSMUSG00000026520	ENSMUSG00000034354
ENSMUSG00000026526	ENSMUSG00000034361
ENSMUSG00000026527	ENSMUSG00000034377
ENSMUSG00000026546	ENSMUSG00000034390
ENSMUSG00000026547	ENSMUSG00000034402
ENSMUSG00000026553	ENSMUSG00000034403
ENSMUSG00000026554	ENSMUSG00000034413
ENSMUSG00000026556	ENSMUSG00000034424
ENSMUSG00000026558	ENSMUSG00000034430
ENSMUSG00000026563	ENSMUSG00000034432
ENSMUSG00000026565	ENSMUSG00000034462
ENSMUSG00000026566	ENSMUSG00000034471
ENSMUSG00000026568	ENSMUSG00000034472

ENSMUSG00000026571	ENSMUSG00000034480
ENSMUSG00000026575	ENSMUSG00000034484
ENSMUSG00000026576	ENSMUSG00000034488
ENSMUSG00000026577	ENSMUSG00000034501
ENSMUSG00000026578	ENSMUSG00000034525
ENSMUSG00000026584	ENSMUSG00000034543
ENSMUSG00000026585	ENSMUSG00000034557
ENSMUSG00000026586	ENSMUSG00000034560
ENSMUSG00000026587	ENSMUSG00000034563
ENSMUSG00000026594	ENSMUSG00000034566
ENSMUSG00000026596	ENSMUSG00000034570
ENSMUSG00000026600	ENSMUSG00000034574
ENSMUSG00000026603	ENSMUSG00000034575
ENSMUSG00000026604	ENSMUSG00000034586
ENSMUSG00000026608	ENSMUSG00000034593
ENSMUSG00000026610	ENSMUSG00000034602
ENSMUSG00000026614	ENSMUSG00000034610
ENSMUSG00000026615	ENSMUSG00000034612
ENSMUSG00000026617	ENSMUSG00000034613
ENSMUSG00000026618	ENSMUSG00000034614
ENSMUSG00000026620	ENSMUSG00000034621
ENSMUSG00000026623	ENSMUSG00000034636
ENSMUSG00000026626	ENSMUSG00000034647
ENSMUSG00000026627	ENSMUSG00000034648
ENSMUSG00000026632	ENSMUSG00000034653
ENSMUSG00000026634	ENSMUSG00000034656
ENSMUSG00000026640	ENSMUSG00000034659
ENSMUSG00000026641	ENSMUSG00000034667
ENSMUSG00000026643	ENSMUSG00000034673
ENSMUSG00000026648	ENSMUSG00000034675
ENSMUSG00000026649	ENSMUSG00000034681
ENSMUSG00000026655	ENSMUSG00000034684
ENSMUSG00000026657	ENSMUSG00000034685
ENSMUSG00000026659	ENSMUSG00000034686
ENSMUSG00000026662	ENSMUSG00000034687
ENSMUSG00000026663	ENSMUSG00000034707
ENSMUSG00000026664	ENSMUSG00000034708
ENSMUSG00000026667	ENSMUSG00000034709
ENSMUSG00000026670	ENSMUSG00000034714
ENSMUSG00000026672	ENSMUSG00000034723
ENSMUSG00000026674	ENSMUSG00000034724
ENSMUSG00000026675	ENSMUSG00000034730
ENSMUSG00000026678	ENSMUSG00000034731
ENSMUSG00000026687	ENSMUSG00000034751
ENSMUSG00000026688	ENSMUSG00000034755
ENSMUSG00000026694	ENSMUSG00000034757
ENSMUSG00000026696	ENSMUSG00000034761
ENSMUSG00000026698	ENSMUSG00000034780
ENSMUSG00000026701	ENSMUSG00000034781
ENSMUSG00000026705	ENSMUSG00000034789
ENSMUSG00000026709	ENSMUSG00000034799
ENSMUSG00000026718	ENSMUSG00000034801
ENSMUSG00000026721	ENSMUSG00000034807

ENSMUSG00000026727	ENSMUSG00000034813
ENSMUSG00000026728	ENSMUSG00000034818
ENSMUSG00000026737	ENSMUSG00000034820
ENSMUSG00000026739	ENSMUSG00000034825
ENSMUSG00000026740	ENSMUSG00000034832
ENSMUSG00000026743	ENSMUSG00000034839
ENSMUSG00000026748	ENSMUSG00000034850
ENSMUSG00000026749	ENSMUSG00000034853
ENSMUSG00000026750	ENSMUSG00000034854
ENSMUSG00000026753	ENSMUSG00000034858
ENSMUSG00000026754	ENSMUSG00000034863
ENSMUSG00000026755	ENSMUSG00000034868
ENSMUSG00000026761	ENSMUSG00000034889
ENSMUSG00000026764	ENSMUSG00000034891
ENSMUSG00000026766	ENSMUSG00000034892
ENSMUSG00000026767	ENSMUSG00000034893
ENSMUSG00000026771	ENSMUSG00000034898
ENSMUSG00000026773	ENSMUSG00000034902
ENSMUSG00000026775	ENSMUSG00000034908
ENSMUSG00000026778	ENSMUSG00000034910
ENSMUSG00000026781	ENSMUSG00000034912
ENSMUSG00000026782	ENSMUSG00000034926
ENSMUSG00000026787	ENSMUSG00000034928
ENSMUSG00000026788	ENSMUSG00000034930
ENSMUSG00000026790	ENSMUSG00000034931
ENSMUSG00000026791	ENSMUSG00000034940
ENSMUSG00000026792	ENSMUSG00000034949
ENSMUSG00000026796	ENSMUSG00000034951
ENSMUSG00000026797	ENSMUSG00000034958
ENSMUSG00000026798	ENSMUSG00000034973
ENSMUSG00000026799	ENSMUSG00000034974
ENSMUSG00000026803	ENSMUSG00000034981
ENSMUSG00000026806	ENSMUSG00000034993
ENSMUSG00000026807	ENSMUSG00000034994
ENSMUSG00000026810	ENSMUSG00000034997
ENSMUSG00000026811	ENSMUSG00000035007
ENSMUSG00000026812	ENSMUSG00000035011
ENSMUSG00000026814	ENSMUSG00000035027
ENSMUSG00000026816	ENSMUSG00000035045
ENSMUSG00000026817	ENSMUSG00000035049
ENSMUSG00000026819	ENSMUSG00000035051
ENSMUSG00000026820	ENSMUSG00000035064
ENSMUSG00000026821	ENSMUSG00000035078
ENSMUSG00000026824	ENSMUSG00000035086
ENSMUSG00000026825	ENSMUSG00000035093
ENSMUSG00000026827	ENSMUSG00000035133
ENSMUSG00000026830	ENSMUSG00000035150
ENSMUSG00000026833	ENSMUSG00000035152
ENSMUSG00000026834	ENSMUSG00000035161
ENSMUSG00000026836	ENSMUSG00000035164
ENSMUSG00000026839	ENSMUSG00000035168
ENSMUSG00000026840	ENSMUSG00000035173
ENSMUSG00000026841	ENSMUSG00000035199

ENSMUSG00000026842	ENSMUSG00000035202
ENSMUSG00000026843	ENSMUSG00000035203
ENSMUSG00000026848	ENSMUSG00000035206
ENSMUSG00000026849	ENSMUSG00000035226
ENSMUSG00000026851	ENSMUSG00000035227
ENSMUSG00000026853	ENSMUSG00000035228
ENSMUSG00000026854	ENSMUSG00000035232
ENSMUSG00000026856	ENSMUSG00000035236
ENSMUSG00000026857	ENSMUSG00000035242
ENSMUSG00000026858	ENSMUSG00000035246
ENSMUSG00000026860	ENSMUSG00000035247
ENSMUSG00000026864	ENSMUSG00000035248
ENSMUSG00000026867	ENSMUSG00000035268
ENSMUSG00000026869	ENSMUSG00000035274
ENSMUSG00000026872	ENSMUSG00000035284
ENSMUSG00000026878	ENSMUSG00000035285
ENSMUSG00000026879	ENSMUSG00000035297
ENSMUSG00000026880	ENSMUSG00000035311
ENSMUSG00000026883	ENSMUSG00000035314
ENSMUSG00000026885	ENSMUSG00000035325
ENSMUSG00000026887	ENSMUSG00000035342
ENSMUSG00000026888	ENSMUSG00000035354
ENSMUSG00000026889	ENSMUSG00000035390
ENSMUSG00000026890	ENSMUSG00000035392
ENSMUSG00000026893	ENSMUSG00000035397
ENSMUSG00000026895	ENSMUSG00000035401
ENSMUSG00000026904	ENSMUSG00000035437
ENSMUSG00000026914	ENSMUSG00000035469
ENSMUSG00000026915	ENSMUSG00000035478
ENSMUSG00000026917	ENSMUSG00000035517
ENSMUSG00000026918	ENSMUSG00000035521
ENSMUSG00000026921	ENSMUSG00000035530
ENSMUSG00000026923	ENSMUSG00000035545
ENSMUSG00000026924	ENSMUSG00000035547
ENSMUSG00000026925	ENSMUSG00000035566
ENSMUSG00000026926	ENSMUSG00000035569
ENSMUSG00000026927	ENSMUSG00000035575
ENSMUSG00000026930	ENSMUSG00000035585
ENSMUSG00000026932	ENSMUSG00000035596
ENSMUSG00000026933	ENSMUSG00000035614
ENSMUSG00000026942	ENSMUSG00000035620
ENSMUSG00000026944	ENSMUSG00000035621
ENSMUSG00000026956	ENSMUSG00000035623
ENSMUSG00000026958	ENSMUSG00000035629
ENSMUSG00000026959	ENSMUSG00000035632
ENSMUSG00000026960	ENSMUSG00000035640
ENSMUSG00000026965	ENSMUSG00000035649
ENSMUSG00000026966	ENSMUSG00000035653
ENSMUSG00000026970	ENSMUSG00000035666
ENSMUSG00000026972	ENSMUSG00000035674
ENSMUSG00000026974	ENSMUSG00000035681
ENSMUSG00000026975	ENSMUSG00000035726
ENSMUSG00000026977	ENSMUSG00000035735

ENSMUSG00000026986	ENSMUSG00000035754
ENSMUSG00000026987	ENSMUSG00000035764
ENSMUSG00000026988	ENSMUSG00000035765
ENSMUSG00000026991	ENSMUSG00000035770
ENSMUSG00000026999	ENSMUSG00000035776
ENSMUSG00000027001	ENSMUSG00000035781
ENSMUSG00000027002	ENSMUSG00000035790
ENSMUSG00000027006	ENSMUSG00000035798
ENSMUSG00000027007	ENSMUSG00000035805
ENSMUSG00000027010	ENSMUSG00000035824
ENSMUSG00000027011	ENSMUSG00000035828
ENSMUSG00000027012	ENSMUSG00000035835
ENSMUSG00000027014	ENSMUSG00000035847
ENSMUSG00000027016	ENSMUSG00000035851
ENSMUSG00000027018	ENSMUSG00000035863
ENSMUSG00000027030	ENSMUSG00000035864
ENSMUSG00000027035	ENSMUSG00000035877
ENSMUSG00000027067	ENSMUSG00000035885
ENSMUSG00000027076	ENSMUSG00000035890
ENSMUSG00000027079	ENSMUSG00000035891
ENSMUSG00000027080	ENSMUSG00000035898
ENSMUSG00000027086	ENSMUSG00000035900
ENSMUSG00000027087	ENSMUSG00000035901
ENSMUSG00000027088	ENSMUSG00000035919
ENSMUSG00000027091	ENSMUSG00000035933
ENSMUSG00000027099	ENSMUSG00000035934
ENSMUSG00000027104	ENSMUSG00000035936
ENSMUSG00000027108	ENSMUSG00000035941
ENSMUSG00000027109	ENSMUSG00000035949
ENSMUSG00000027111	ENSMUSG00000035953
ENSMUSG00000027122	ENSMUSG00000035954
ENSMUSG00000027130	ENSMUSG00000035960
ENSMUSG00000027131	ENSMUSG00000035964
ENSMUSG00000027133	ENSMUSG00000035967
ENSMUSG00000027134	ENSMUSG00000035969
ENSMUSG00000027160	ENSMUSG00000035992
ENSMUSG00000027162	ENSMUSG00000036006
ENSMUSG00000027163	ENSMUSG00000036026
ENSMUSG00000027164	ENSMUSG00000036046
ENSMUSG00000027165	ENSMUSG00000036052
ENSMUSG00000027166	ENSMUSG00000036053
ENSMUSG00000027167	ENSMUSG00000036054
ENSMUSG00000027168	ENSMUSG00000036057
ENSMUSG00000027170	ENSMUSG00000036062
ENSMUSG00000027175	ENSMUSG00000036093
ENSMUSG00000027176	ENSMUSG00000036095
ENSMUSG00000027177	ENSMUSG00000036097
ENSMUSG00000027180	ENSMUSG00000036098
ENSMUSG00000027184	ENSMUSG00000036099
ENSMUSG00000027185	ENSMUSG00000036104
ENSMUSG00000027187	ENSMUSG00000036112
ENSMUSG00000027188	ENSMUSG00000036138
ENSMUSG00000027189	ENSMUSG00000036155

ENSMUSG00000027193	ENSMUSG00000036158
ENSMUSG00000027194	ENSMUSG00000036167
ENSMUSG00000027195	ENSMUSG00000036180
ENSMUSG00000027198	ENSMUSG00000036181
ENSMUSG00000027199	ENSMUSG00000036186
ENSMUSG00000027200	ENSMUSG00000036188
ENSMUSG00000027201	ENSMUSG00000036192
ENSMUSG00000027203	ENSMUSG00000036199
ENSMUSG00000027204	ENSMUSG00000036202
ENSMUSG00000027206	ENSMUSG00000036206
ENSMUSG00000027207	ENSMUSG00000036225
ENSMUSG00000027210	ENSMUSG00000036241
ENSMUSG00000027215	ENSMUSG00000036242
ENSMUSG00000027217	ENSMUSG00000036257
ENSMUSG00000027220	ENSMUSG00000036264
ENSMUSG00000027221	ENSMUSG00000036270
ENSMUSG00000027222	ENSMUSG00000036273
ENSMUSG00000027223	ENSMUSG00000036275
ENSMUSG00000027227	ENSMUSG00000036282
ENSMUSG00000027236	ENSMUSG00000036295
ENSMUSG00000027238	ENSMUSG00000036298
ENSMUSG00000027239	ENSMUSG00000036299
ENSMUSG00000027242	ENSMUSG00000036306
ENSMUSG00000027243	ENSMUSG00000036309
ENSMUSG00000027244	ENSMUSG00000036323
ENSMUSG00000027245	ENSMUSG00000036327
ENSMUSG00000027247	ENSMUSG00000036333
ENSMUSG00000027248	ENSMUSG00000036352
ENSMUSG00000027253	ENSMUSG00000036357
ENSMUSG00000027254	ENSMUSG00000036371
ENSMUSG00000027255	ENSMUSG00000036377
ENSMUSG00000027257	ENSMUSG00000036398
ENSMUSG00000027259	ENSMUSG00000036402
ENSMUSG00000027263	ENSMUSG00000036422
ENSMUSG00000027270	ENSMUSG00000036427
ENSMUSG00000027272	ENSMUSG00000036435
ENSMUSG00000027273	ENSMUSG00000036438
ENSMUSG00000027274	ENSMUSG00000036450
ENSMUSG00000027276	ENSMUSG00000036452
ENSMUSG00000027281	ENSMUSG00000036469
ENSMUSG00000027282	ENSMUSG00000036473
ENSMUSG00000027284	ENSMUSG00000036478
ENSMUSG00000027285	ENSMUSG00000036499
ENSMUSG00000027286	ENSMUSG00000036501
ENSMUSG00000027287	ENSMUSG00000036503
ENSMUSG00000027288	ENSMUSG00000036510
ENSMUSG00000027291	ENSMUSG00000036529
ENSMUSG00000027293	ENSMUSG00000036550
ENSMUSG00000027296	ENSMUSG00000036555
ENSMUSG00000027298	ENSMUSG00000036560
ENSMUSG00000027300	ENSMUSG00000036561
ENSMUSG00000027303	ENSMUSG00000036564
ENSMUSG00000027304	ENSMUSG00000036565

ENSMUSG00000027305	ENSMUSG00000036568
ENSMUSG00000027309	ENSMUSG00000036580
ENSMUSG00000027312	ENSMUSG00000036585
ENSMUSG00000027313	ENSMUSG00000036591
ENSMUSG00000027315	ENSMUSG00000036606
ENSMUSG00000027316	ENSMUSG00000036613
ENSMUSG00000027324	ENSMUSG00000036617
ENSMUSG00000027327	ENSMUSG00000036620
ENSMUSG00000027329	ENSMUSG00000036622
ENSMUSG00000027330	ENSMUSG00000036634
ENSMUSG00000027332	ENSMUSG00000036636
ENSMUSG00000027333	ENSMUSG00000036644
ENSMUSG00000027339	ENSMUSG00000036646
ENSMUSG00000027340	ENSMUSG00000036667
ENSMUSG00000027341	ENSMUSG00000036686
ENSMUSG00000027342	ENSMUSG00000036698
ENSMUSG00000027346	ENSMUSG00000036699
ENSMUSG00000027347	ENSMUSG00000036707
ENSMUSG00000027349	ENSMUSG00000036712
ENSMUSG00000027350	ENSMUSG00000036733
ENSMUSG00000027351	ENSMUSG00000036737
ENSMUSG00000027357	ENSMUSG00000036745
ENSMUSG00000027359	ENSMUSG00000036748
ENSMUSG00000027360	ENSMUSG00000036751
ENSMUSG00000027361	ENSMUSG00000036752
ENSMUSG00000027363	ENSMUSG00000036760
ENSMUSG00000027365	ENSMUSG00000036766
ENSMUSG00000027366	ENSMUSG00000036769
ENSMUSG00000027367	ENSMUSG00000036779
ENSMUSG00000027371	ENSMUSG00000036782
ENSMUSG00000027374	ENSMUSG00000036790
ENSMUSG00000027375	ENSMUSG00000036792
ENSMUSG00000027378	ENSMUSG00000036800
ENSMUSG00000027384	ENSMUSG00000036815
ENSMUSG00000027386	ENSMUSG00000036817
ENSMUSG00000027394	ENSMUSG00000036819
ENSMUSG00000027395	ENSMUSG00000036825
ENSMUSG00000027397	ENSMUSG00000036862
ENSMUSG00000027400	ENSMUSG00000036879
ENSMUSG00000027404	ENSMUSG00000036882
ENSMUSG00000027405	ENSMUSG00000036885
ENSMUSG00000027406	ENSMUSG00000036887
ENSMUSG00000027408	ENSMUSG00000036893
ENSMUSG00000027411	ENSMUSG00000036894
ENSMUSG00000027419	ENSMUSG00000036902
ENSMUSG00000027422	ENSMUSG00000036940
ENSMUSG00000027423	ENSMUSG00000036941
ENSMUSG00000027425	ENSMUSG00000036943
ENSMUSG00000027427	ENSMUSG00000036948
ENSMUSG00000027428	ENSMUSG00000036949
ENSMUSG00000027429	ENSMUSG00000036955
ENSMUSG00000027430	ENSMUSG00000036957
ENSMUSG00000027433	ENSMUSG00000036959

ENSMUSG00000027434	ENSMUSG00000036966
ENSMUSG00000027435	ENSMUSG00000036985
ENSMUSG00000027438	ENSMUSG00000036989
ENSMUSG00000027439	ENSMUSG00000036990
ENSMUSG00000027447	ENSMUSG00000037012
ENSMUSG00000027452	ENSMUSG00000037029
ENSMUSG00000027455	ENSMUSG00000037032
ENSMUSG00000027457	ENSMUSG00000037049
ENSMUSG00000027459	ENSMUSG00000037058
ENSMUSG00000027465	ENSMUSG00000037062
ENSMUSG00000027466	ENSMUSG00000037070
ENSMUSG00000027472	ENSMUSG00000037071
ENSMUSG00000027475	ENSMUSG00000037072
ENSMUSG00000027479	ENSMUSG00000037098
ENSMUSG00000027487	ENSMUSG00000037104
ENSMUSG00000027488	ENSMUSG00000037110
ENSMUSG00000027489	ENSMUSG00000037111
ENSMUSG00000027495	ENSMUSG00000037112
ENSMUSG00000027498	ENSMUSG00000037119
ENSMUSG00000027499	ENSMUSG00000037126
ENSMUSG00000027500	ENSMUSG00000037138
ENSMUSG00000027502	ENSMUSG00000037148
ENSMUSG00000027506	ENSMUSG00000037149
ENSMUSG00000027508	ENSMUSG00000037151
ENSMUSG00000027509	ENSMUSG00000037172
ENSMUSG00000027519	ENSMUSG00000037174
ENSMUSG00000027520	ENSMUSG00000037210
ENSMUSG00000027522	ENSMUSG00000037217
ENSMUSG00000027523	ENSMUSG00000037224
ENSMUSG00000027525	ENSMUSG00000037234
ENSMUSG00000027531	ENSMUSG00000037235
ENSMUSG00000027533	ENSMUSG00000037236
ENSMUSG00000027534	ENSMUSG00000037239
ENSMUSG00000027540	ENSMUSG00000037242
ENSMUSG00000027544	ENSMUSG00000037251
ENSMUSG00000027546	ENSMUSG00000037253
ENSMUSG00000027550	ENSMUSG00000037257
ENSMUSG00000027551	ENSMUSG00000037259
ENSMUSG00000027560	ENSMUSG00000037260
ENSMUSG00000027562	ENSMUSG00000037266
ENSMUSG00000027566	ENSMUSG00000037270
ENSMUSG00000027568	ENSMUSG00000037275
ENSMUSG00000027569	ENSMUSG00000037286
ENSMUSG00000027570	ENSMUSG00000037287
ENSMUSG00000027573	ENSMUSG00000037306
ENSMUSG00000027574	ENSMUSG00000037316
ENSMUSG00000027575	ENSMUSG00000037326
ENSMUSG00000027577	ENSMUSG00000037331
ENSMUSG00000027580	ENSMUSG00000037341
ENSMUSG00000027581	ENSMUSG00000037343
ENSMUSG00000027582	ENSMUSG00000037344
ENSMUSG00000027583	ENSMUSG00000037348
ENSMUSG00000027584	ENSMUSG00000037351

ENSMUSG00000027589	ENSMUSG00000037353
ENSMUSG00000027593	ENSMUSG00000037364
ENSMUSG00000027597	ENSMUSG00000037369
ENSMUSG00000027598	ENSMUSG00000037373
ENSMUSG00000027599	ENSMUSG00000037386
ENSMUSG00000027601	ENSMUSG00000037400
ENSMUSG00000027602	ENSMUSG00000037410
ENSMUSG00000027603	ENSMUSG00000037415
ENSMUSG00000027605	ENSMUSG00000037416
ENSMUSG00000027610	ENSMUSG00000037419
ENSMUSG00000027612	ENSMUSG00000037426
ENSMUSG00000027613	ENSMUSG00000037428
ENSMUSG00000027615	ENSMUSG00000037434
ENSMUSG00000027618	ENSMUSG00000037458
ENSMUSG00000027620	ENSMUSG00000037461
ENSMUSG00000027624	ENSMUSG00000037470
ENSMUSG00000027628	ENSMUSG00000037475
ENSMUSG00000027630	ENSMUSG00000037486
ENSMUSG00000027634	ENSMUSG00000037487
ENSMUSG00000027637	ENSMUSG00000037503
ENSMUSG00000027639	ENSMUSG00000037509
ENSMUSG00000027642	ENSMUSG00000037514
ENSMUSG00000027646	ENSMUSG00000037519
ENSMUSG00000027649	ENSMUSG00000037526
ENSMUSG00000027650	ENSMUSG00000037533
ENSMUSG00000027651	ENSMUSG00000037536
ENSMUSG00000027652	ENSMUSG00000037541
ENSMUSG00000027655	ENSMUSG00000037553
ENSMUSG00000027660	ENSMUSG00000037563
ENSMUSG00000027663	ENSMUSG00000037570
ENSMUSG00000027665	ENSMUSG00000037573
ENSMUSG00000027667	ENSMUSG00000037579
ENSMUSG00000027668	ENSMUSG00000037601
ENSMUSG00000027669	ENSMUSG00000037605
ENSMUSG00000027671	ENSMUSG00000037608
ENSMUSG00000027673	ENSMUSG00000037622
ENSMUSG00000027674	ENSMUSG00000037624
ENSMUSG00000027676	ENSMUSG00000037625
ENSMUSG00000027677	ENSMUSG00000037640
ENSMUSG00000027678	ENSMUSG00000037643
ENSMUSG00000027679	ENSMUSG00000037646
ENSMUSG00000027680	ENSMUSG00000037652
ENSMUSG00000027692	ENSMUSG00000037653
ENSMUSG00000027694	ENSMUSG00000037656
ENSMUSG00000027695	ENSMUSG00000037669
ENSMUSG00000027698	ENSMUSG00000037674
ENSMUSG00000027706	ENSMUSG00000037679
ENSMUSG00000027708	ENSMUSG00000037685
ENSMUSG00000027709	ENSMUSG00000037692
ENSMUSG00000027710	ENSMUSG00000037697
ENSMUSG00000027712	ENSMUSG00000037703
ENSMUSG00000027714	ENSMUSG00000037706
ENSMUSG00000027716	ENSMUSG00000037710

ENSMUSG00000027722	ENSMUSG00000037712
ENSMUSG00000027737	ENSMUSG00000037720
ENSMUSG00000027739	ENSMUSG00000037736
ENSMUSG00000027742	ENSMUSG00000037740
ENSMUSG00000027744	ENSMUSG00000037742
ENSMUSG00000027746	ENSMUSG00000037747
ENSMUSG00000027748	ENSMUSG00000037754
ENSMUSG00000027751	ENSMUSG00000037771
ENSMUSG00000027752	ENSMUSG00000037784
ENSMUSG00000027763	ENSMUSG00000037788
ENSMUSG00000027765	ENSMUSG00000037791
ENSMUSG00000027770	ENSMUSG00000037795
ENSMUSG00000027774	ENSMUSG00000037805
ENSMUSG00000027775	ENSMUSG00000037815
ENSMUSG00000027777	ENSMUSG00000037822
ENSMUSG00000027778	ENSMUSG00000037826
ENSMUSG00000027782	ENSMUSG00000037843
ENSMUSG00000027784	ENSMUSG00000037851
ENSMUSG00000027787	ENSMUSG00000037852
ENSMUSG00000027796	ENSMUSG00000037855
ENSMUSG00000027797	ENSMUSG00000037857
ENSMUSG00000027799	ENSMUSG00000037876
ENSMUSG00000027800	ENSMUSG00000037885
ENSMUSG00000027803	ENSMUSG00000037887
ENSMUSG00000027804	ENSMUSG00000037894
ENSMUSG00000027805	ENSMUSG00000037902
ENSMUSG00000027806	ENSMUSG00000037905
ENSMUSG00000027808	ENSMUSG00000037907
ENSMUSG00000027809	ENSMUSG00000037916
ENSMUSG00000027810	ENSMUSG00000037926
ENSMUSG00000027822	ENSMUSG00000037933
ENSMUSG00000027823	ENSMUSG00000037935
ENSMUSG00000027827	ENSMUSG00000037972
ENSMUSG00000027828	ENSMUSG00000037979
ENSMUSG00000027829	ENSMUSG00000037989
ENSMUSG00000027834	ENSMUSG00000037990
ENSMUSG00000027835	ENSMUSG00000037993
ENSMUSG00000027845	ENSMUSG00000037996
ENSMUSG00000027848	ENSMUSG00000037999
ENSMUSG00000027849	ENSMUSG00000038002
ENSMUSG00000027852	ENSMUSG00000038013
ENSMUSG00000027854	ENSMUSG00000038014
ENSMUSG00000027858	ENSMUSG00000038020
ENSMUSG00000027860	ENSMUSG00000038023
ENSMUSG00000027864	ENSMUSG00000038024
ENSMUSG00000027865	ENSMUSG00000038025
ENSMUSG00000027875	ENSMUSG00000038026
ENSMUSG00000027878	ENSMUSG00000038034
ENSMUSG00000027879	ENSMUSG00000038039
ENSMUSG00000027881	ENSMUSG00000038042
ENSMUSG00000027882	ENSMUSG00000038056
ENSMUSG00000027883	ENSMUSG00000038065
ENSMUSG00000027884	ENSMUSG00000038072

ENSMUSG00000027889	ENSMUSG00000038077
ENSMUSG00000027890	ENSMUSG00000038084
ENSMUSG00000027893	ENSMUSG00000038095
ENSMUSG00000027894	ENSMUSG00000038102
ENSMUSG00000027895	ENSMUSG00000038115
ENSMUSG00000027900	ENSMUSG00000038116
ENSMUSG00000027905	ENSMUSG00000038121
ENSMUSG00000027932	ENSMUSG00000038127
ENSMUSG00000027933	ENSMUSG00000038128
ENSMUSG00000027936	ENSMUSG00000038132
ENSMUSG00000027937	ENSMUSG00000038141
ENSMUSG00000027940	ENSMUSG00000038143
ENSMUSG00000027942	ENSMUSG00000038145
ENSMUSG00000027944	ENSMUSG00000038146
ENSMUSG00000027947	ENSMUSG00000038150
ENSMUSG00000027950	ENSMUSG00000038156
ENSMUSG00000027951	ENSMUSG00000038170
ENSMUSG00000027952	ENSMUSG00000038172
ENSMUSG00000027953	ENSMUSG00000038174
ENSMUSG00000027956	ENSMUSG00000038178
ENSMUSG00000027957	ENSMUSG00000038181
ENSMUSG00000027962	ENSMUSG00000038187
ENSMUSG00000027963	ENSMUSG00000038205
ENSMUSG00000027965	ENSMUSG00000038212
ENSMUSG00000027966	ENSMUSG00000038241
ENSMUSG00000027968	ENSMUSG00000038244
ENSMUSG00000027971	ENSMUSG00000038248
ENSMUSG00000027977	ENSMUSG00000038250
ENSMUSG00000027981	ENSMUSG00000038256
ENSMUSG00000027983	ENSMUSG00000038264
ENSMUSG00000027984	ENSMUSG00000038267
ENSMUSG00000027985	ENSMUSG00000038271
ENSMUSG00000027993	ENSMUSG00000038274
ENSMUSG00000027998	ENSMUSG00000038279
ENSMUSG00000027999	ENSMUSG00000038280
ENSMUSG00000028004	ENSMUSG00000038290
ENSMUSG00000028005	ENSMUSG00000038291
ENSMUSG00000028007	ENSMUSG00000038296
ENSMUSG00000028013	ENSMUSG00000038299
ENSMUSG00000028015	ENSMUSG00000038301
ENSMUSG00000028016	ENSMUSG00000038319
ENSMUSG00000028018	ENSMUSG00000038324
ENSMUSG00000028019	ENSMUSG00000038332
ENSMUSG00000028020	ENSMUSG00000038335
ENSMUSG00000028029	ENSMUSG00000038349
ENSMUSG00000028030	ENSMUSG00000038351
ENSMUSG00000028032	ENSMUSG00000038365
ENSMUSG00000028033	ENSMUSG00000038366
ENSMUSG00000028034	ENSMUSG00000038368
ENSMUSG00000028035	ENSMUSG00000038369
ENSMUSG00000028039	ENSMUSG00000038370
ENSMUSG00000028041	ENSMUSG00000038371
ENSMUSG00000028042	ENSMUSG00000038374

ENSMUSG00000028047	ENSMUSG00000038375
ENSMUSG00000028048	ENSMUSG00000038384
ENSMUSG00000028049	ENSMUSG00000038388
ENSMUSG00000028051	ENSMUSG00000038390
ENSMUSG00000028053	ENSMUSG00000038400
ENSMUSG00000028057	ENSMUSG00000038406
ENSMUSG00000028059	ENSMUSG00000038412
ENSMUSG00000028060	ENSMUSG00000038416
ENSMUSG00000028062	ENSMUSG00000038417
ENSMUSG00000028063	ENSMUSG00000038418
ENSMUSG00000028064	ENSMUSG00000038429
ENSMUSG00000028069	ENSMUSG00000038437
ENSMUSG00000028070	ENSMUSG00000038446
ENSMUSG00000028078	ENSMUSG00000038453
ENSMUSG00000028080	ENSMUSG00000038456
ENSMUSG00000028081	ENSMUSG00000038462
ENSMUSG00000028082	ENSMUSG00000038467
ENSMUSG00000028085	ENSMUSG00000038473
ENSMUSG00000028086	ENSMUSG00000038481
ENSMUSG00000028089	ENSMUSG00000038482
ENSMUSG00000028093	ENSMUSG00000038485
ENSMUSG00000028096	ENSMUSG00000038486
ENSMUSG00000028098	ENSMUSG00000038489
ENSMUSG00000028099	ENSMUSG00000038495
ENSMUSG00000028101	ENSMUSG00000038497
ENSMUSG00000028102	ENSMUSG00000038502
ENSMUSG00000028104	ENSMUSG00000038503
ENSMUSG00000028106	ENSMUSG00000038506
ENSMUSG00000028107	ENSMUSG00000038517
ENSMUSG00000028108	ENSMUSG00000038518
ENSMUSG00000028114	ENSMUSG00000038520
ENSMUSG00000028121	ENSMUSG00000038530
ENSMUSG00000028124	ENSMUSG00000038533
ENSMUSG00000028126	ENSMUSG00000038535
ENSMUSG00000028127	ENSMUSG00000038538
ENSMUSG00000028128	ENSMUSG00000038545
ENSMUSG00000028132	ENSMUSG00000038546
ENSMUSG00000028133	ENSMUSG00000038552
ENSMUSG00000028134	ENSMUSG00000038555
ENSMUSG00000028136	ENSMUSG00000038564
ENSMUSG00000028137	ENSMUSG00000038576
ENSMUSG00000028138	ENSMUSG00000038582
ENSMUSG00000028140	ENSMUSG00000038587
ENSMUSG00000028145	ENSMUSG00000038602
ENSMUSG00000028149	ENSMUSG00000038604
ENSMUSG00000028152	ENSMUSG00000038607
ENSMUSG00000028156	ENSMUSG00000038608
ENSMUSG00000028158	ENSMUSG00000038611
ENSMUSG00000028161	ENSMUSG00000038612
ENSMUSG00000028163	ENSMUSG00000038615
ENSMUSG00000028164	ENSMUSG00000038619
ENSMUSG00000028165	ENSMUSG00000038630
ENSMUSG00000028172	ENSMUSG00000038633

ENSMUSG00000028173	ENSMUSG00000038642
ENSMUSG00000028176	ENSMUSG00000038650
ENSMUSG00000028180	ENSMUSG00000038658
ENSMUSG00000028184	ENSMUSG00000038664
ENSMUSG00000028187	ENSMUSG00000038665
ENSMUSG00000028189	ENSMUSG00000038677
ENSMUSG00000028191	ENSMUSG00000038679
ENSMUSG00000028194	ENSMUSG00000038690
ENSMUSG00000028195	ENSMUSG00000038696
ENSMUSG00000028199	ENSMUSG00000038702
ENSMUSG00000028207	ENSMUSG00000038708
ENSMUSG00000028211	ENSMUSG00000038717
ENSMUSG00000028218	ENSMUSG00000038718
ENSMUSG00000028221	ENSMUSG00000038729
ENSMUSG00000028222	ENSMUSG00000038733
ENSMUSG00000028223	ENSMUSG00000038738
ENSMUSG00000028224	ENSMUSG00000038740
ENSMUSG00000028226	ENSMUSG00000038759
ENSMUSG00000028228	ENSMUSG00000038762
ENSMUSG00000028229	ENSMUSG00000038773
ENSMUSG00000028232	ENSMUSG00000038774
ENSMUSG00000028233	ENSMUSG00000038780
ENSMUSG00000028234	ENSMUSG00000038784
ENSMUSG00000028243	ENSMUSG00000038805
ENSMUSG00000028245	ENSMUSG00000038806
ENSMUSG00000028246	ENSMUSG00000038807
ENSMUSG00000028247	ENSMUSG00000038822
ENSMUSG00000028248	ENSMUSG00000038828
ENSMUSG00000028249	ENSMUSG00000038831
ENSMUSG00000028251	ENSMUSG00000038838
ENSMUSG00000028252	ENSMUSG00000038845
ENSMUSG00000028256	ENSMUSG00000038848
ENSMUSG00000028261	ENSMUSG00000038855
ENSMUSG00000028266	ENSMUSG00000038860
ENSMUSG00000028271	ENSMUSG00000038861
ENSMUSG00000028273	ENSMUSG00000038871
ENSMUSG00000028274	ENSMUSG00000038872
ENSMUSG00000028277	ENSMUSG00000038876
ENSMUSG00000028278	ENSMUSG00000038884
ENSMUSG00000028282	ENSMUSG00000038886
ENSMUSG00000028284	ENSMUSG00000038894
ENSMUSG00000028289	ENSMUSG00000038900
ENSMUSG00000028291	ENSMUSG00000038902
ENSMUSG00000028292	ENSMUSG00000038909
ENSMUSG00000028293	ENSMUSG00000038910
ENSMUSG00000028294	ENSMUSG00000038914
ENSMUSG00000028300	ENSMUSG00000038916
ENSMUSG00000028309	ENSMUSG00000038936
ENSMUSG00000028312	ENSMUSG00000038949
ENSMUSG00000028322	ENSMUSG00000038957
ENSMUSG00000028328	ENSMUSG00000038965
ENSMUSG00000028329	ENSMUSG00000038967
ENSMUSG00000028330	ENSMUSG00000038970

ENSMUSG00000028333	ENSMUSG00000038975
ENSMUSG00000028334	ENSMUSG00000038976
ENSMUSG00000028337	ENSMUSG00000038984
ENSMUSG00000028339	ENSMUSG00000038991
ENSMUSG00000028341	ENSMUSG00000039000
ENSMUSG00000028343	ENSMUSG00000039001
ENSMUSG00000028344	ENSMUSG00000039016
ENSMUSG00000028345	ENSMUSG00000039033
ENSMUSG00000028347	ENSMUSG00000039037
ENSMUSG00000028351	ENSMUSG00000039041
ENSMUSG00000028354	ENSMUSG00000039047
ENSMUSG00000028358	ENSMUSG00000039050
ENSMUSG00000028360	ENSMUSG00000039057
ENSMUSG00000028364	ENSMUSG00000039058
ENSMUSG00000028367	ENSMUSG00000039059
ENSMUSG00000028373	ENSMUSG00000039067
ENSMUSG00000028381	ENSMUSG00000039068
ENSMUSG00000028382	ENSMUSG00000039081
ENSMUSG00000028383	ENSMUSG00000039086
ENSMUSG00000028385	ENSMUSG00000039087
ENSMUSG00000028389	ENSMUSG00000039100
ENSMUSG00000028391	ENSMUSG00000039105
ENSMUSG00000028393	ENSMUSG00000039108
ENSMUSG00000028394	ENSMUSG00000039117
ENSMUSG00000028397	ENSMUSG00000039126
ENSMUSG00000028398	ENSMUSG00000039128
ENSMUSG00000028399	ENSMUSG00000039145
ENSMUSG00000028402	ENSMUSG00000039148
ENSMUSG00000028403	ENSMUSG00000039156
ENSMUSG00000028405	ENSMUSG00000039157
ENSMUSG00000028409	ENSMUSG00000039159
ENSMUSG00000028410	ENSMUSG00000039176
ENSMUSG00000028411	ENSMUSG00000039197
ENSMUSG00000028412	ENSMUSG00000039202
ENSMUSG00000028414	ENSMUSG00000039206
ENSMUSG00000028416	ENSMUSG00000039210
ENSMUSG00000028419	ENSMUSG00000039218
ENSMUSG00000028420	ENSMUSG00000039219
ENSMUSG00000028423	ENSMUSG00000039220
ENSMUSG00000028426	ENSMUSG00000039221
ENSMUSG00000028430	ENSMUSG00000039230
ENSMUSG00000028431	ENSMUSG00000039252
ENSMUSG00000028433	ENSMUSG00000039253
ENSMUSG00000028434	ENSMUSG00000039262
ENSMUSG00000028436	ENSMUSG00000039270
ENSMUSG00000028437	ENSMUSG00000039275
ENSMUSG00000028439	ENSMUSG00000039278
ENSMUSG00000028441	ENSMUSG00000039285
ENSMUSG00000028443	ENSMUSG00000039286
ENSMUSG00000028444	ENSMUSG00000039318
ENSMUSG00000028445	ENSMUSG00000039347
ENSMUSG00000028447	ENSMUSG00000039354
ENSMUSG00000028452	ENSMUSG00000039361

ENSMUSG00000028453	ENSMUSG00000039367
ENSMUSG00000028454	ENSMUSG00000039372
ENSMUSG00000028455	ENSMUSG00000039375
ENSMUSG00000028456	ENSMUSG00000039414
ENSMUSG00000028458	ENSMUSG00000039419
ENSMUSG00000028461	ENSMUSG00000039428
ENSMUSG00000028465	ENSMUSG00000039431
ENSMUSG00000028466	ENSMUSG00000039449
ENSMUSG00000028467	ENSMUSG00000039463
ENSMUSG00000028468	ENSMUSG00000039470
ENSMUSG00000028469	ENSMUSG00000039473
ENSMUSG00000028470	ENSMUSG00000039474
ENSMUSG00000028476	ENSMUSG00000039477
ENSMUSG00000028478	ENSMUSG00000039478
ENSMUSG00000028479	ENSMUSG00000039483
ENSMUSG00000028483	ENSMUSG00000039485
ENSMUSG00000028484	ENSMUSG00000039501
ENSMUSG00000028488	ENSMUSG00000039509
ENSMUSG00000028494	ENSMUSG00000039512
ENSMUSG00000028495	ENSMUSG00000039515
ENSMUSG00000028496	ENSMUSG00000039523
ENSMUSG00000028514	ENSMUSG00000039530
ENSMUSG00000028517	ENSMUSG00000039533
ENSMUSG00000028518	ENSMUSG00000039536
ENSMUSG00000028519	ENSMUSG00000039542
ENSMUSG00000028521	ENSMUSG00000039546
ENSMUSG00000028522	ENSMUSG00000039556
ENSMUSG00000028524	ENSMUSG00000039568
ENSMUSG00000028525	ENSMUSG00000039578
ENSMUSG00000028527	ENSMUSG00000039579
ENSMUSG00000028528	ENSMUSG00000039585
ENSMUSG00000028530	ENSMUSG00000039601
ENSMUSG00000028532	ENSMUSG00000039607
ENSMUSG00000028538	ENSMUSG00000039611
ENSMUSG00000028540	ENSMUSG00000039615
ENSMUSG00000028541	ENSMUSG00000039621
ENSMUSG00000028542	ENSMUSG00000039629
ENSMUSG00000028545	ENSMUSG00000039630
ENSMUSG00000028546	ENSMUSG00000039637
ENSMUSG00000028550	ENSMUSG00000039640
ENSMUSG00000028552	ENSMUSG00000039652
ENSMUSG00000028556	ENSMUSG00000039656
ENSMUSG00000028557	ENSMUSG00000039660
ENSMUSG00000028559	ENSMUSG00000039661
ENSMUSG00000028560	ENSMUSG00000039662
ENSMUSG00000028563	ENSMUSG00000039671
ENSMUSG00000028565	ENSMUSG00000039678
ENSMUSG00000028567	ENSMUSG00000039682
ENSMUSG00000028568	ENSMUSG00000039686
ENSMUSG00000028572	ENSMUSG00000039697
ENSMUSG00000028576	ENSMUSG00000039703
ENSMUSG00000028577	ENSMUSG00000039704
ENSMUSG00000028578	ENSMUSG00000039713

ENSMUSG00000028580	ENSMUSG00000039716
ENSMUSG00000028581	ENSMUSG00000039717
ENSMUSG00000028582	ENSMUSG00000039735
ENSMUSG00000028583	ENSMUSG00000039738
ENSMUSG00000028601	ENSMUSG00000039740
ENSMUSG00000028603	ENSMUSG00000039741
ENSMUSG00000028607	ENSMUSG00000039747
ENSMUSG00000028608	ENSMUSG00000039753
ENSMUSG00000028609	ENSMUSG00000039768
ENSMUSG00000028613	ENSMUSG00000039770
ENSMUSG00000028614	ENSMUSG00000039782
ENSMUSG00000028617	ENSMUSG00000039801
ENSMUSG00000028618	ENSMUSG00000039804
ENSMUSG00000028619	ENSMUSG00000039809
ENSMUSG00000028621	ENSMUSG00000039834
ENSMUSG00000028622	ENSMUSG00000039835
ENSMUSG00000028626	ENSMUSG00000039838
ENSMUSG00000028629	ENSMUSG00000039840
ENSMUSG00000028630	ENSMUSG00000039844
ENSMUSG00000028633	ENSMUSG00000039849
ENSMUSG00000028634	ENSMUSG00000039850
ENSMUSG00000028636	ENSMUSG00000039852
ENSMUSG00000028637	ENSMUSG00000039860
ENSMUSG00000028639	ENSMUSG00000039904
ENSMUSG00000028641	ENSMUSG00000039917
ENSMUSG00000028643	ENSMUSG00000039952
ENSMUSG00000028645	ENSMUSG00000039953
ENSMUSG00000028646	ENSMUSG00000039954
ENSMUSG00000028647	ENSMUSG00000039959
ENSMUSG00000028648	ENSMUSG00000039967
ENSMUSG00000028649	ENSMUSG00000039976
ENSMUSG00000028651	ENSMUSG00000039983
ENSMUSG00000028653	ENSMUSG00000039988
ENSMUSG00000028654	ENSMUSG00000039989
ENSMUSG00000028655	ENSMUSG00000040003
ENSMUSG00000028656	ENSMUSG00000040007
ENSMUSG00000028657	ENSMUSG00000040009
ENSMUSG00000028661	ENSMUSG00000040010
ENSMUSG00000028664	ENSMUSG00000040021
ENSMUSG00000028668	ENSMUSG00000040022
ENSMUSG00000028669	ENSMUSG00000040025
ENSMUSG00000028670	ENSMUSG00000040028
ENSMUSG00000028671	ENSMUSG00000040029
ENSMUSG00000028672	ENSMUSG00000040035
ENSMUSG00000028673	ENSMUSG00000040037
ENSMUSG00000028675	ENSMUSG00000040048
ENSMUSG00000028676	ENSMUSG00000040054
ENSMUSG00000028677	ENSMUSG00000040055
ENSMUSG00000028680	ENSMUSG00000040097
ENSMUSG00000028681	ENSMUSG00000040102
ENSMUSG00000028683	ENSMUSG00000040105
ENSMUSG00000028684	ENSMUSG00000040111
ENSMUSG00000028688	ENSMUSG00000040118

ENSMUSG00000028690	ENSMUSG00000040123
ENSMUSG00000028691	ENSMUSG00000040125
ENSMUSG00000028692	ENSMUSG00000040128
ENSMUSG00000028693	ENSMUSG00000040151
ENSMUSG00000028696	ENSMUSG00000040183
ENSMUSG00000028698	ENSMUSG00000040209
ENSMUSG00000028700	ENSMUSG00000040225
ENSMUSG00000028701	ENSMUSG00000040234
ENSMUSG00000028703	ENSMUSG00000040236
ENSMUSG00000028706	ENSMUSG00000040242
ENSMUSG00000028708	ENSMUSG00000040249
ENSMUSG00000028710	ENSMUSG00000040260
ENSMUSG00000028719	ENSMUSG00000040265
ENSMUSG00000028729	ENSMUSG00000040268
ENSMUSG00000028737	ENSMUSG00000040276
ENSMUSG00000028741	ENSMUSG00000040289
ENSMUSG00000028743	ENSMUSG00000040297
ENSMUSG00000028744	ENSMUSG00000040325
ENSMUSG00000028745	ENSMUSG00000040327
ENSMUSG00000028753	ENSMUSG00000040339
ENSMUSG00000028756	ENSMUSG00000040351
ENSMUSG00000028757	ENSMUSG00000040354
ENSMUSG00000028758	ENSMUSG00000040356
ENSMUSG00000028759	ENSMUSG00000040359
ENSMUSG00000028760	ENSMUSG00000040363
ENSMUSG00000028763	ENSMUSG00000040365
ENSMUSG00000028766	ENSMUSG00000040383
ENSMUSG00000028771	ENSMUSG00000040385
ENSMUSG00000028772	ENSMUSG00000040389
ENSMUSG00000028773	ENSMUSG00000040390
ENSMUSG00000028779	ENSMUSG00000040396
ENSMUSG00000028780	ENSMUSG00000040407
ENSMUSG00000028782	ENSMUSG00000040412
ENSMUSG00000028785	ENSMUSG00000040414
ENSMUSG00000028788	ENSMUSG00000040415
ENSMUSG00000028789	ENSMUSG00000040423
ENSMUSG00000028790	ENSMUSG00000040430
ENSMUSG00000028792	ENSMUSG00000040433
ENSMUSG00000028793	ENSMUSG00000040446
ENSMUSG00000028795	ENSMUSG00000040451
ENSMUSG00000028796	ENSMUSG00000040455
ENSMUSG00000028797	ENSMUSG00000040459
ENSMUSG00000028798	ENSMUSG00000040462
ENSMUSG00000028799	ENSMUSG00000040463
ENSMUSG00000028800	ENSMUSG00000040472
ENSMUSG00000028803	ENSMUSG00000040479
ENSMUSG00000028804	ENSMUSG00000040481
ENSMUSG00000028809	ENSMUSG00000040488
ENSMUSG00000028811	ENSMUSG00000040495
ENSMUSG00000028820	ENSMUSG00000040506
ENSMUSG00000028821	ENSMUSG00000040524
ENSMUSG00000028822	ENSMUSG00000040536
ENSMUSG00000028826	ENSMUSG00000040537

ENSMUSG00000028830	ENSMUSG00000040543
ENSMUSG00000028832	ENSMUSG00000040548
ENSMUSG00000028833	ENSMUSG00000040549
ENSMUSG00000028837	ENSMUSG00000040550
ENSMUSG00000028838	ENSMUSG00000040560
ENSMUSG00000028840	ENSMUSG00000040563
ENSMUSG00000028842	ENSMUSG00000040565
ENSMUSG00000028843	ENSMUSG00000040570
ENSMUSG00000028845	ENSMUSG00000040591
ENSMUSG00000028847	ENSMUSG00000040596
ENSMUSG00000028848	ENSMUSG00000040606
ENSMUSG00000028849	ENSMUSG00000040612
ENSMUSG00000028850	ENSMUSG00000040620
ENSMUSG00000028851	ENSMUSG00000040631
ENSMUSG00000028854	ENSMUSG00000040640
ENSMUSG00000028857	ENSMUSG00000040648
ENSMUSG00000028861	ENSMUSG00000040649
ENSMUSG00000028863	ENSMUSG00000040651
ENSMUSG00000028868	ENSMUSG00000040652
ENSMUSG00000028869	ENSMUSG00000040659
ENSMUSG00000028876	ENSMUSG00000040661
ENSMUSG00000028878	ENSMUSG00000040667
ENSMUSG00000028879	ENSMUSG00000040669
ENSMUSG00000028882	ENSMUSG00000040681
ENSMUSG00000028883	ENSMUSG00000040687
ENSMUSG00000028884	ENSMUSG00000040697
ENSMUSG00000028886	ENSMUSG00000040699
ENSMUSG00000028889	ENSMUSG00000040712
ENSMUSG00000028890	ENSMUSG00000040720
ENSMUSG00000028893	ENSMUSG00000040721
ENSMUSG00000028894	ENSMUSG00000040722
ENSMUSG00000028896	ENSMUSG00000040724
ENSMUSG00000028898	ENSMUSG00000040725
ENSMUSG00000028899	ENSMUSG00000040731
ENSMUSG00000028901	ENSMUSG00000040746
ENSMUSG00000028902	ENSMUSG00000040760
ENSMUSG00000028906	ENSMUSG00000040761
ENSMUSG00000028907	ENSMUSG00000040782
ENSMUSG00000028909	ENSMUSG00000040785
ENSMUSG00000028910	ENSMUSG00000040794
ENSMUSG00000028911	ENSMUSG00000040797
ENSMUSG00000028914	ENSMUSG00000040811
ENSMUSG00000028917	ENSMUSG00000040813
ENSMUSG00000028919	ENSMUSG00000040824
ENSMUSG00000028920	ENSMUSG00000040842
ENSMUSG00000028923	ENSMUSG00000040843
ENSMUSG00000028926	ENSMUSG00000040848
ENSMUSG00000028927	ENSMUSG00000040850
ENSMUSG00000028931	ENSMUSG00000040855
ENSMUSG00000028932	ENSMUSG00000040856
ENSMUSG00000028933	ENSMUSG00000040857
ENSMUSG00000028936	ENSMUSG00000040859
ENSMUSG00000028937	ENSMUSG00000040860

ENSMUSG00000028944	ENSMUSG00000040865
ENSMUSG00000028945	ENSMUSG00000040867
ENSMUSG00000028948	ENSMUSG00000040875
ENSMUSG00000028949	ENSMUSG00000040896
ENSMUSG00000028952	ENSMUSG00000040907
ENSMUSG00000028953	ENSMUSG00000040928
ENSMUSG00000028954	ENSMUSG00000040929
ENSMUSG00000028955	ENSMUSG00000040943
ENSMUSG00000028957	ENSMUSG00000040945
ENSMUSG00000028958	ENSMUSG00000040952
ENSMUSG00000028959	ENSMUSG00000040957
ENSMUSG00000028960	ENSMUSG00000040964
ENSMUSG00000028961	ENSMUSG00000040990
ENSMUSG00000028962	ENSMUSG00000040997
ENSMUSG00000028964	ENSMUSG00000041000
ENSMUSG00000028967	ENSMUSG00000041020
ENSMUSG00000028969	ENSMUSG00000041028
ENSMUSG00000028973	ENSMUSG00000041035
ENSMUSG00000028974	ENSMUSG00000041037
ENSMUSG00000028975	ENSMUSG00000041040
ENSMUSG00000028976	ENSMUSG00000041058
ENSMUSG00000028977	ENSMUSG00000041073
ENSMUSG00000028978	ENSMUSG00000041078
ENSMUSG00000028980	ENSMUSG00000041096
ENSMUSG00000028982	ENSMUSG00000041112
ENSMUSG00000028986	ENSMUSG00000041115
ENSMUSG00000028988	ENSMUSG00000041124
ENSMUSG00000028990	ENSMUSG00000041130
ENSMUSG00000028991	ENSMUSG00000041133
ENSMUSG00000028992	ENSMUSG00000041141
ENSMUSG00000028995	ENSMUSG00000041164
ENSMUSG00000028998	ENSMUSG00000041168
ENSMUSG00000028999	ENSMUSG00000041199
ENSMUSG00000029001	ENSMUSG00000041203
ENSMUSG00000029003	ENSMUSG00000041215
ENSMUSG00000029004	ENSMUSG00000041216
ENSMUSG00000029007	ENSMUSG00000041220
ENSMUSG00000029009	ENSMUSG00000041225
ENSMUSG00000029012	ENSMUSG00000041229
ENSMUSG00000029014	ENSMUSG00000041236
ENSMUSG00000029016	ENSMUSG00000041241
ENSMUSG00000029017	ENSMUSG00000041245
ENSMUSG00000029020	ENSMUSG00000041258
ENSMUSG00000029022	ENSMUSG00000041263
ENSMUSG00000029026	ENSMUSG00000041264
ENSMUSG00000029028	ENSMUSG00000041268
ENSMUSG00000029029	ENSMUSG00000041278
ENSMUSG00000029030	ENSMUSG00000041297
ENSMUSG00000029033	ENSMUSG00000041298
ENSMUSG00000029034	ENSMUSG00000041303
ENSMUSG00000029036	ENSMUSG00000041308
ENSMUSG00000029038	ENSMUSG00000041313
ENSMUSG00000029047	ENSMUSG00000041328

ENSMUSG00000029048	ENSMUSG00000041329
ENSMUSG00000029049	ENSMUSG00000041341
ENSMUSG00000029050	ENSMUSG00000041351
ENSMUSG00000029053	ENSMUSG00000041355
ENSMUSG00000029054	ENSMUSG00000041375
ENSMUSG00000029055	ENSMUSG00000041380
ENSMUSG00000029056	ENSMUSG00000041408
ENSMUSG00000029059	ENSMUSG00000041415
ENSMUSG00000029060	ENSMUSG00000041417
ENSMUSG00000029062	ENSMUSG00000041439
ENSMUSG00000029063	ENSMUSG00000041444
ENSMUSG00000029064	ENSMUSG00000041453
ENSMUSG00000029066	ENSMUSG00000041459
ENSMUSG00000029068	ENSMUSG00000041483
ENSMUSG00000029070	ENSMUSG00000041528
ENSMUSG00000029071	ENSMUSG00000041530
ENSMUSG00000029073	ENSMUSG00000041556
ENSMUSG00000029076	ENSMUSG00000041560
ENSMUSG00000029084	ENSMUSG00000041570
ENSMUSG00000029086	ENSMUSG00000041571
ENSMUSG00000029088	ENSMUSG00000041592
ENSMUSG00000029089	ENSMUSG00000041598
ENSMUSG00000029090	ENSMUSG00000041607
ENSMUSG00000029093	ENSMUSG00000041609
ENSMUSG00000029094	ENSMUSG00000041624
ENSMUSG00000029095	ENSMUSG00000041638
ENSMUSG00000029096	ENSMUSG00000041642
ENSMUSG00000029097	ENSMUSG00000041645
ENSMUSG00000029098	ENSMUSG00000041658
ENSMUSG00000029101	ENSMUSG00000041670
ENSMUSG00000029103	ENSMUSG00000041688
ENSMUSG00000029104	ENSMUSG00000041695
ENSMUSG00000029106	ENSMUSG00000041697
ENSMUSG00000029108	ENSMUSG00000041702
ENSMUSG00000029110	ENSMUSG00000041708
ENSMUSG00000029111	ENSMUSG00000041712
ENSMUSG00000029119	ENSMUSG00000041720
ENSMUSG00000029120	ENSMUSG00000041729
ENSMUSG00000029121	ENSMUSG00000041733
ENSMUSG00000029122	ENSMUSG00000041740
ENSMUSG00000029123	ENSMUSG00000041747
ENSMUSG00000029125	ENSMUSG00000041757
ENSMUSG00000029126	ENSMUSG00000041762
ENSMUSG00000029127	ENSMUSG00000041763
ENSMUSG00000029128	ENSMUSG00000041765
ENSMUSG00000029130	ENSMUSG00000041769
ENSMUSG00000029131	ENSMUSG00000041771
ENSMUSG00000029135	ENSMUSG00000041773
ENSMUSG00000029141	ENSMUSG00000041781
ENSMUSG00000029145	ENSMUSG00000041794
ENSMUSG00000029146	ENSMUSG00000041815
ENSMUSG00000029147	ENSMUSG00000041817
ENSMUSG00000029148	ENSMUSG00000041836

ENSMUSG00000029152	ENSMUSG00000041841
ENSMUSG00000029153	ENSMUSG00000041846
ENSMUSG00000029155	ENSMUSG00000041852
ENSMUSG00000029156	ENSMUSG00000041870
ENSMUSG00000029161	ENSMUSG00000041879
ENSMUSG00000029162	ENSMUSG00000041881
ENSMUSG00000029165	ENSMUSG00000041889
ENSMUSG00000029166	ENSMUSG00000041890
ENSMUSG00000029167	ENSMUSG00000041891
ENSMUSG00000029168	ENSMUSG00000041912
ENSMUSG00000029169	ENSMUSG00000041923
ENSMUSG00000029171	ENSMUSG00000041926
ENSMUSG00000029173	ENSMUSG00000041935
ENSMUSG00000029174	ENSMUSG00000041936
ENSMUSG00000029175	ENSMUSG00000041958
ENSMUSG00000029176	ENSMUSG00000041961
ENSMUSG00000029178	ENSMUSG00000041977
ENSMUSG00000029179	ENSMUSG00000041986
ENSMUSG00000029189	ENSMUSG00000041992
ENSMUSG00000029190	ENSMUSG00000041997
ENSMUSG00000029191	ENSMUSG00000042032
ENSMUSG00000029192	ENSMUSG00000042035
ENSMUSG00000029196	ENSMUSG00000042046
ENSMUSG00000029198	ENSMUSG00000042055
ENSMUSG00000029199	ENSMUSG00000042066
ENSMUSG00000029201	ENSMUSG00000042078
ENSMUSG00000029202	ENSMUSG00000042079
ENSMUSG00000029203	ENSMUSG00000042082
ENSMUSG00000029206	ENSMUSG00000042097
ENSMUSG00000029207	ENSMUSG00000042105
ENSMUSG00000029208	ENSMUSG00000042109
ENSMUSG00000029209	ENSMUSG00000042121
ENSMUSG00000029211	ENSMUSG00000042133
ENSMUSG00000029212	ENSMUSG00000042155
ENSMUSG00000029213	ENSMUSG00000042156
ENSMUSG00000029219	ENSMUSG00000042182
ENSMUSG00000029221	ENSMUSG00000042185
ENSMUSG00000029223	ENSMUSG00000042197
ENSMUSG00000029227	ENSMUSG00000042202
ENSMUSG00000029228	ENSMUSG00000042203
ENSMUSG00000029229	ENSMUSG00000042207
ENSMUSG00000029231	ENSMUSG00000042211
ENSMUSG00000029233	ENSMUSG00000042216
ENSMUSG00000029234	ENSMUSG00000042249
ENSMUSG00000029238	ENSMUSG00000042272
ENSMUSG00000029245	ENSMUSG00000042292
ENSMUSG00000029246	ENSMUSG00000042298
ENSMUSG00000029247	ENSMUSG00000042302
ENSMUSG00000029249	ENSMUSG00000042303
ENSMUSG00000029250	ENSMUSG00000042305
ENSMUSG00000029253	ENSMUSG00000042308
ENSMUSG00000029263	ENSMUSG00000042323
ENSMUSG00000029265	ENSMUSG00000042331

ENSMUSG00000029267	ENSMUSG00000042350
ENSMUSG00000029270	ENSMUSG00000042359
ENSMUSG00000029276	ENSMUSG00000042363
ENSMUSG00000029279	ENSMUSG00000042364
ENSMUSG00000029283	ENSMUSG00000042388
ENSMUSG00000029287	ENSMUSG00000042390
ENSMUSG00000029290	ENSMUSG00000042401
ENSMUSG00000029291	ENSMUSG00000042404
ENSMUSG00000029309	ENSMUSG00000042406
ENSMUSG00000029310	ENSMUSG00000042408
ENSMUSG00000029311	ENSMUSG00000042410
ENSMUSG00000029312	ENSMUSG00000042423
ENSMUSG00000029313	ENSMUSG00000042425
ENSMUSG00000029319	ENSMUSG00000042426
ENSMUSG00000029326	ENSMUSG00000042428
ENSMUSG00000029328	ENSMUSG00000042429
ENSMUSG00000029330	ENSMUSG00000042439
ENSMUSG00000029334	ENSMUSG00000042444
ENSMUSG00000029344	ENSMUSG00000042446
ENSMUSG00000029345	ENSMUSG00000042447
ENSMUSG00000029346	ENSMUSG00000042453
ENSMUSG00000029348	ENSMUSG00000042492
ENSMUSG00000029359	ENSMUSG00000042502
ENSMUSG00000029361	ENSMUSG00000042506
ENSMUSG00000029364	ENSMUSG00000042507
ENSMUSG00000029366	ENSMUSG00000042508
ENSMUSG00000029376	ENSMUSG00000042520
ENSMUSG00000029381	ENSMUSG00000042523
ENSMUSG00000029385	ENSMUSG00000042524
ENSMUSG00000029386	ENSMUSG00000042532
ENSMUSG00000029387	ENSMUSG00000042535
ENSMUSG00000029388	ENSMUSG00000042541
ENSMUSG00000029389	ENSMUSG00000042548
ENSMUSG00000029390	ENSMUSG00000042557
ENSMUSG00000029392	ENSMUSG00000042570
ENSMUSG00000029394	ENSMUSG00000042572
ENSMUSG00000029397	ENSMUSG00000042589
ENSMUSG00000029401	ENSMUSG00000042590
ENSMUSG00000029402	ENSMUSG00000042599
ENSMUSG00000029403	ENSMUSG00000042604
ENSMUSG00000029404	ENSMUSG00000042605
ENSMUSG00000029405	ENSMUSG00000042613
ENSMUSG00000029406	ENSMUSG00000042616
ENSMUSG00000029407	ENSMUSG00000042625
ENSMUSG00000029408	ENSMUSG00000042628
ENSMUSG00000029413	ENSMUSG00000042642
ENSMUSG00000029415	ENSMUSG00000042650
ENSMUSG00000029416	ENSMUSG00000042671
ENSMUSG00000029419	ENSMUSG00000042675
ENSMUSG00000029420	ENSMUSG00000042680
ENSMUSG00000029422	ENSMUSG00000042682
ENSMUSG00000029426	ENSMUSG00000042686
ENSMUSG00000029427	ENSMUSG00000042688

ENSMUSG00000029428	ENSMUSG00000042699
ENSMUSG00000029430	ENSMUSG00000042700
ENSMUSG00000029432	ENSMUSG00000042712
ENSMUSG00000029433	ENSMUSG00000042719
ENSMUSG00000029434	ENSMUSG00000042724
ENSMUSG00000029436	ENSMUSG00000042726
ENSMUSG00000029438	ENSMUSG00000042734
ENSMUSG00000029439	ENSMUSG00000042743
ENSMUSG00000029440	ENSMUSG00000042744
ENSMUSG00000029442	ENSMUSG00000042750
ENSMUSG00000029446	ENSMUSG00000042751
ENSMUSG00000029447	ENSMUSG00000042763
ENSMUSG00000029449	ENSMUSG00000042766
ENSMUSG00000029454	ENSMUSG00000042772
ENSMUSG00000029455	ENSMUSG00000042790
ENSMUSG00000029458	ENSMUSG00000042804
ENSMUSG00000029461	ENSMUSG00000042807
ENSMUSG00000029462	ENSMUSG00000042810
ENSMUSG00000029463	ENSMUSG00000042826
ENSMUSG00000029464	ENSMUSG00000042846
ENSMUSG00000029465	ENSMUSG00000042873
ENSMUSG00000029466	ENSMUSG00000042901
ENSMUSG00000029467	ENSMUSG00000042978
ENSMUSG00000029468	ENSMUSG00000043004
ENSMUSG00000029469	ENSMUSG00000043019
ENSMUSG00000029470	ENSMUSG00000043067
ENSMUSG00000029471	ENSMUSG00000043079
ENSMUSG00000029472	ENSMUSG00000043090
ENSMUSG00000029474	ENSMUSG00000043154
ENSMUSG00000029475	ENSMUSG00000043207
ENSMUSG00000029478	ENSMUSG00000043241
ENSMUSG00000029480	ENSMUSG00000043252
ENSMUSG00000029482	ENSMUSG00000043259
ENSMUSG00000029486	ENSMUSG00000043323
ENSMUSG00000029500	ENSMUSG00000043384
ENSMUSG00000029501	ENSMUSG00000043388
ENSMUSG00000029502	ENSMUSG00000043391
ENSMUSG00000029504	ENSMUSG00000043411
ENSMUSG00000029505	ENSMUSG00000043419
ENSMUSG00000029507	ENSMUSG00000043445
ENSMUSG00000029512	ENSMUSG00000043456
ENSMUSG00000029513	ENSMUSG00000043460
ENSMUSG00000029516	ENSMUSG00000043463
ENSMUSG00000029518	ENSMUSG00000043467
ENSMUSG00000029524	ENSMUSG00000043496
ENSMUSG00000029528	ENSMUSG00000043531
ENSMUSG00000029534	ENSMUSG00000043535
ENSMUSG00000029535	ENSMUSG00000043542
ENSMUSG00000029536	ENSMUSG00000043557
ENSMUSG00000029538	ENSMUSG00000043635
ENSMUSG00000029544	ENSMUSG00000043670
ENSMUSG00000029545	ENSMUSG00000043671
ENSMUSG00000029547	ENSMUSG00000043683

ENSMUSG00000029550	ENSMUSG00000043716
ENSMUSG00000029551	ENSMUSG00000043733
ENSMUSG00000029554	ENSMUSG00000043857
ENSMUSG00000029557	ENSMUSG00000043866
ENSMUSG00000029559	ENSMUSG00000043881
ENSMUSG00000029560	ENSMUSG00000043885
ENSMUSG00000029563	ENSMUSG00000043909
ENSMUSG00000029569	ENSMUSG00000043929
ENSMUSG00000029570	ENSMUSG00000043940
ENSMUSG00000029571	ENSMUSG00000043962
ENSMUSG00000029575	ENSMUSG00000043991
ENSMUSG00000029576	ENSMUSG00000044024
ENSMUSG00000029577	ENSMUSG00000044030
ENSMUSG00000029578	ENSMUSG00000044066
ENSMUSG00000029580	ENSMUSG00000044068
ENSMUSG00000029581	ENSMUSG00000044071
ENSMUSG00000029587	ENSMUSG00000044072
ENSMUSG00000029592	ENSMUSG00000044080
ENSMUSG00000029594	ENSMUSG00000044098
ENSMUSG00000029598	ENSMUSG00000044117
ENSMUSG00000029599	ENSMUSG00000044147
ENSMUSG00000029600	ENSMUSG00000044167
ENSMUSG00000029602	ENSMUSG00000044216
ENSMUSG00000029603	ENSMUSG00000044221
ENSMUSG00000029608	ENSMUSG00000044252
ENSMUSG00000029610	ENSMUSG00000044285
ENSMUSG00000029613	ENSMUSG00000044288
ENSMUSG00000029614	ENSMUSG00000044308
ENSMUSG00000029616	ENSMUSG00000044340
ENSMUSG00000029617	ENSMUSG00000044349
ENSMUSG00000029621	ENSMUSG00000044352
ENSMUSG00000029622	ENSMUSG00000044365
ENSMUSG00000029623	ENSMUSG00000044433
ENSMUSG00000029624	ENSMUSG00000044452
ENSMUSG00000029625	ENSMUSG00000044465
ENSMUSG00000029627	ENSMUSG00000044471
ENSMUSG00000029629	ENSMUSG00000044477
ENSMUSG00000029632	ENSMUSG00000044502
ENSMUSG00000029634	ENSMUSG00000044528
ENSMUSG00000029635	ENSMUSG00000044533
ENSMUSG00000029636	ENSMUSG00000044550
ENSMUSG00000029638	ENSMUSG00000044600
ENSMUSG00000029640	ENSMUSG00000044627
ENSMUSG00000029642	ENSMUSG00000044628
ENSMUSG00000029647	ENSMUSG00000044636
ENSMUSG00000029648	ENSMUSG00000044647
ENSMUSG00000029649	ENSMUSG00000044667
ENSMUSG00000029651	ENSMUSG00000044676
ENSMUSG00000029655	ENSMUSG00000044700
ENSMUSG00000029657	ENSMUSG00000044708
ENSMUSG00000029661	ENSMUSG00000044715
ENSMUSG00000029669	ENSMUSG00000044788
ENSMUSG00000029670	ENSMUSG00000044791

ENSMUSG00000029672	ENSMUSG00000044792
ENSMUSG00000029673	ENSMUSG00000044807
ENSMUSG00000029674	ENSMUSG00000044835
ENSMUSG00000029675	ENSMUSG00000044857
ENSMUSG00000029676	ENSMUSG00000044864
ENSMUSG00000029681	ENSMUSG00000044876
ENSMUSG00000029684	ENSMUSG00000044894
ENSMUSG00000029686	ENSMUSG00000044912
ENSMUSG00000029687	ENSMUSG00000044950
ENSMUSG00000029701	ENSMUSG00000045007
ENSMUSG00000029703	ENSMUSG00000045009
ENSMUSG00000029705	ENSMUSG00000045034
ENSMUSG00000029708	ENSMUSG00000045038
ENSMUSG00000029710	ENSMUSG00000045039
ENSMUSG00000029712	ENSMUSG00000045045
ENSMUSG00000029713	ENSMUSG00000045078
ENSMUSG00000029714	ENSMUSG00000045083
ENSMUSG00000029715	ENSMUSG00000045092
ENSMUSG00000029720	ENSMUSG00000045095
ENSMUSG00000029722	ENSMUSG00000045098
ENSMUSG00000029723	ENSMUSG00000045103
ENSMUSG00000029725	ENSMUSG00000045114
ENSMUSG00000029726	ENSMUSG00000045128
ENSMUSG00000029729	ENSMUSG00000045136
ENSMUSG00000029730	ENSMUSG00000045174
ENSMUSG00000029752	ENSMUSG00000045180
ENSMUSG00000029754	ENSMUSG00000045193
ENSMUSG00000029755	ENSMUSG00000045205
ENSMUSG00000029757	ENSMUSG00000045210
ENSMUSG00000029761	ENSMUSG00000045211
ENSMUSG00000029763	ENSMUSG00000045216
ENSMUSG00000029765	ENSMUSG00000045252
ENSMUSG00000029767	ENSMUSG00000045284
ENSMUSG00000029769	ENSMUSG00000045287
ENSMUSG00000029772	ENSMUSG00000045294
ENSMUSG00000029775	ENSMUSG00000045302
ENSMUSG00000029776	ENSMUSG00000045318
ENSMUSG00000029777	ENSMUSG00000045348
ENSMUSG00000029778	ENSMUSG00000045349
ENSMUSG00000029780	ENSMUSG00000045374
ENSMUSG00000029781	ENSMUSG00000045411
ENSMUSG00000029782	ENSMUSG00000045427
ENSMUSG00000029787	ENSMUSG00000045441
ENSMUSG00000029790	ENSMUSG00000045482
ENSMUSG00000029802	ENSMUSG00000045503
ENSMUSG00000029804	ENSMUSG00000045515
ENSMUSG00000029810	ENSMUSG00000045534
ENSMUSG00000029815	ENSMUSG00000045573
ENSMUSG00000029817	ENSMUSG00000045576
ENSMUSG00000029819	ENSMUSG00000045589
ENSMUSG00000029821	ENSMUSG00000045636
ENSMUSG00000029822	ENSMUSG00000045639
ENSMUSG00000029823	ENSMUSG00000045658

ENSMUSG00000029833	ENSMUSG00000045671
ENSMUSG00000029836	ENSMUSG00000045733
ENSMUSG00000029838	ENSMUSG00000045752
ENSMUSG00000029840	ENSMUSG00000045763
ENSMUSG00000029860	ENSMUSG00000045767
ENSMUSG00000029861	ENSMUSG00000045790
ENSMUSG00000029863	ENSMUSG00000045896
ENSMUSG00000029864	ENSMUSG00000045962
ENSMUSG00000029869	ENSMUSG00000045967
ENSMUSG00000029875	ENSMUSG00000045973
ENSMUSG00000029878	ENSMUSG00000045983
ENSMUSG00000029910	ENSMUSG00000045994
ENSMUSG00000029911	ENSMUSG00000046020
ENSMUSG00000029913	ENSMUSG00000046032
ENSMUSG00000029916	ENSMUSG00000046062
ENSMUSG00000029918	ENSMUSG00000046079
ENSMUSG00000029920	ENSMUSG00000046093
ENSMUSG00000029922	ENSMUSG00000046096
ENSMUSG00000029924	ENSMUSG00000046111
ENSMUSG00000029992	ENSMUSG00000046138
ENSMUSG00000029993	ENSMUSG00000046157
ENSMUSG00000029998	ENSMUSG00000046160
ENSMUSG00000029999	ENSMUSG00000046178
ENSMUSG00000030000	ENSMUSG00000046182
ENSMUSG00000030002	ENSMUSG00000046201
ENSMUSG00000030007	ENSMUSG00000046204
ENSMUSG00000030016	ENSMUSG00000046215
ENSMUSG00000030019	ENSMUSG00000046230
ENSMUSG00000030020	ENSMUSG00000046240
ENSMUSG00000030022	ENSMUSG00000046311
ENSMUSG00000030029	ENSMUSG00000046312
ENSMUSG00000030030	ENSMUSG00000046314
ENSMUSG00000030032	ENSMUSG00000046324
ENSMUSG00000030034	ENSMUSG00000046329
ENSMUSG00000030035	ENSMUSG00000046330
ENSMUSG00000030036	ENSMUSG00000046364
ENSMUSG00000030037	ENSMUSG00000046378
ENSMUSG00000030042	ENSMUSG00000046432
ENSMUSG00000030043	ENSMUSG00000046434
ENSMUSG00000030045	ENSMUSG00000046442
ENSMUSG00000030055	ENSMUSG00000046447
ENSMUSG00000030056	ENSMUSG00000046449
ENSMUSG00000030057	ENSMUSG00000046480
ENSMUSG00000030058	ENSMUSG00000046516
ENSMUSG00000030059	ENSMUSG00000046532
ENSMUSG00000030060	ENSMUSG00000046556
ENSMUSG00000030061	ENSMUSG00000046562
ENSMUSG00000030062	ENSMUSG00000046574
ENSMUSG00000030064	ENSMUSG00000046598
ENSMUSG00000030067	ENSMUSG00000046607
ENSMUSG00000030075	ENSMUSG00000046658
ENSMUSG00000030077	ENSMUSG00000046671
ENSMUSG00000030079	ENSMUSG00000046691

ENSMUSG00000030082	ENSMUSG00000046699
ENSMUSG00000030083	ENSMUSG00000046707
ENSMUSG00000030084	ENSMUSG00000046709
ENSMUSG00000030086	ENSMUSG00000046711
ENSMUSG00000030087	ENSMUSG00000046722
ENSMUSG00000030088	ENSMUSG00000046727
ENSMUSG00000030091	ENSMUSG00000046785
ENSMUSG00000030092	ENSMUSG00000046798
ENSMUSG00000030093	ENSMUSG00000046822
ENSMUSG00000030094	ENSMUSG00000046836
ENSMUSG00000030095	ENSMUSG00000046844
ENSMUSG00000030096	ENSMUSG00000046861
ENSMUSG00000030098	ENSMUSG00000046876
ENSMUSG00000030101	ENSMUSG00000046897
ENSMUSG00000030102	ENSMUSG00000046922
ENSMUSG00000030103	ENSMUSG00000046962
ENSMUSG00000030104	ENSMUSG00000046982
ENSMUSG00000030105	ENSMUSG00000046985
ENSMUSG00000030110	ENSMUSG00000047013
ENSMUSG00000030120	ENSMUSG00000047030
ENSMUSG00000030122	ENSMUSG00000047036
ENSMUSG00000030123	ENSMUSG00000047037
ENSMUSG00000030125	ENSMUSG00000047045
ENSMUSG00000030126	ENSMUSG00000047084
ENSMUSG00000030127	ENSMUSG00000047085
ENSMUSG00000030134	ENSMUSG00000047098
ENSMUSG00000030138	ENSMUSG00000047126
ENSMUSG00000030145	ENSMUSG00000047181
ENSMUSG00000030161	ENSMUSG00000047187
ENSMUSG00000030166	ENSMUSG00000047193
ENSMUSG00000030168	ENSMUSG00000047205
ENSMUSG00000030172	ENSMUSG00000047213
ENSMUSG00000030180	ENSMUSG00000047215
ENSMUSG00000030189	ENSMUSG00000047248
ENSMUSG00000030199	ENSMUSG00000047261
ENSMUSG00000030201	ENSMUSG00000047264
ENSMUSG00000030203	ENSMUSG00000047284
ENSMUSG00000030204	ENSMUSG00000047379
ENSMUSG00000030207	ENSMUSG00000047388
ENSMUSG00000030209	ENSMUSG00000047409
ENSMUSG00000030213	ENSMUSG00000047412
ENSMUSG00000030216	ENSMUSG00000047414
ENSMUSG00000030222	ENSMUSG00000047417
ENSMUSG00000030223	ENSMUSG00000047423
ENSMUSG00000030224	ENSMUSG00000047434
ENSMUSG00000030226	ENSMUSG00000047446
ENSMUSG00000030231	ENSMUSG00000047454
ENSMUSG00000030232	ENSMUSG00000047459
ENSMUSG00000030235	ENSMUSG00000047466
ENSMUSG00000030237	ENSMUSG00000047495
ENSMUSG00000030243	ENSMUSG00000047507
ENSMUSG00000030245	ENSMUSG00000047514
ENSMUSG00000030246	ENSMUSG00000047539

ENSMUSG00000030249	ENSMUSG00000047547
ENSMUSG00000030254	ENSMUSG00000047658
ENSMUSG00000030255	ENSMUSG00000047675
ENSMUSG00000030256	ENSMUSG00000047676
ENSMUSG00000030257	ENSMUSG00000047694
ENSMUSG00000030259	ENSMUSG00000047710
ENSMUSG00000030264	ENSMUSG00000047712
ENSMUSG00000030265	ENSMUSG00000047714
ENSMUSG00000030268	ENSMUSG00000047731
ENSMUSG00000030269	ENSMUSG00000047747
ENSMUSG00000030271	ENSMUSG00000047766
ENSMUSG00000030272	ENSMUSG00000047786
ENSMUSG00000030275	ENSMUSG00000047787
ENSMUSG00000030276	ENSMUSG00000047804
ENSMUSG00000030279	ENSMUSG00000047824
ENSMUSG00000030282	ENSMUSG00000047842
ENSMUSG00000030283	ENSMUSG00000047866
ENSMUSG00000030284	ENSMUSG00000047879
ENSMUSG00000030286	ENSMUSG00000047888
ENSMUSG00000030287	ENSMUSG00000047907
ENSMUSG00000030291	ENSMUSG00000047921
ENSMUSG00000030298	ENSMUSG00000047935
ENSMUSG00000030301	ENSMUSG00000047945
ENSMUSG00000030302	ENSMUSG00000047976
ENSMUSG00000030303	ENSMUSG00000048000
ENSMUSG00000030304	ENSMUSG00000048022
ENSMUSG00000030306	ENSMUSG00000048027
ENSMUSG00000030307	ENSMUSG00000048038
ENSMUSG00000030309	ENSMUSG00000048040
ENSMUSG00000030310	ENSMUSG00000048076
ENSMUSG00000030313	ENSMUSG00000048078
ENSMUSG00000030314	ENSMUSG00000048109
ENSMUSG00000030315	ENSMUSG00000048118
ENSMUSG00000030316	ENSMUSG00000048142
ENSMUSG00000030317	ENSMUSG00000048148
ENSMUSG00000030319	ENSMUSG00000048154
ENSMUSG00000030321	ENSMUSG00000048163
ENSMUSG00000030322	ENSMUSG00000048175
ENSMUSG00000030323	ENSMUSG00000048216
ENSMUSG00000030327	ENSMUSG00000048222
ENSMUSG00000030329	ENSMUSG00000048232
ENSMUSG00000030330	ENSMUSG00000048240
ENSMUSG00000030335	ENSMUSG00000048249
ENSMUSG00000030337	ENSMUSG00000048251
ENSMUSG00000030339	ENSMUSG00000048264
ENSMUSG00000030340	ENSMUSG00000048271
ENSMUSG00000030341	ENSMUSG00000048279
ENSMUSG00000030342	ENSMUSG00000048285
ENSMUSG00000030347	ENSMUSG00000048304
ENSMUSG00000030350	ENSMUSG00000048307
ENSMUSG00000030352	ENSMUSG00000048330
ENSMUSG00000030357	ENSMUSG00000048385
ENSMUSG00000030374	ENSMUSG00000048388

ENSMUSG00000030376	ENSMUSG00000048410
ENSMUSG00000030380	ENSMUSG00000048429
ENSMUSG00000030386	ENSMUSG00000048458
ENSMUSG00000030393	ENSMUSG00000048481
ENSMUSG00000030397	ENSMUSG00000048483
ENSMUSG00000030400	ENSMUSG00000048490
ENSMUSG00000030401	ENSMUSG00000048537
ENSMUSG00000030403	ENSMUSG00000048546
ENSMUSG00000030407	ENSMUSG00000048578
ENSMUSG00000030409	ENSMUSG00000048644
ENSMUSG00000030410	ENSMUSG00000048661
ENSMUSG00000030411	ENSMUSG00000048701
ENSMUSG00000030417	ENSMUSG00000048732
ENSMUSG00000030421	ENSMUSG00000048756
ENSMUSG00000030423	ENSMUSG00000048799
ENSMUSG00000030428	ENSMUSG00000048807
ENSMUSG00000030432	ENSMUSG00000048814
ENSMUSG00000030435	ENSMUSG00000048826
ENSMUSG00000030447	ENSMUSG00000048833
ENSMUSG00000030451	ENSMUSG00000048834
ENSMUSG00000030452	ENSMUSG00000048874
ENSMUSG00000030465	ENSMUSG00000048878
ENSMUSG00000030469	ENSMUSG00000048895
ENSMUSG00000030471	ENSMUSG00000048897
ENSMUSG00000030486	ENSMUSG00000048911
ENSMUSG00000030494	ENSMUSG00000048920
ENSMUSG00000030495	ENSMUSG00000048930
ENSMUSG00000030498	ENSMUSG00000048960
ENSMUSG00000030499	ENSMUSG00000048978
ENSMUSG00000030500	ENSMUSG00000048988
ENSMUSG00000030505	ENSMUSG00000049044
ENSMUSG00000030509	ENSMUSG00000049047
ENSMUSG00000030512	ENSMUSG00000049076
ENSMUSG00000030513	ENSMUSG00000049086
ENSMUSG00000030515	ENSMUSG00000049090
ENSMUSG00000030516	ENSMUSG00000049091
ENSMUSG00000030518	ENSMUSG00000049092
ENSMUSG00000030519	ENSMUSG00000049097
ENSMUSG00000030521	ENSMUSG00000049100
ENSMUSG00000030522	ENSMUSG00000049106
ENSMUSG00000030525	ENSMUSG00000049119
ENSMUSG00000030527	ENSMUSG00000049176
ENSMUSG00000030530	ENSMUSG00000049191
ENSMUSG00000030532	ENSMUSG00000049225
ENSMUSG00000030533	ENSMUSG00000049252
ENSMUSG00000030534	ENSMUSG00000049281
ENSMUSG00000030536	ENSMUSG00000049295
ENSMUSG00000030538	ENSMUSG00000049303
ENSMUSG00000030539	ENSMUSG00000049305
ENSMUSG00000030541	ENSMUSG00000049313
ENSMUSG00000030545	ENSMUSG00000049323
ENSMUSG00000030551	ENSMUSG00000049327
ENSMUSG00000030554	ENSMUSG00000049336

ENSMUSG00000030556	ENSMUSG00000049339
ENSMUSG00000030557	ENSMUSG00000049354
ENSMUSG00000030560	ENSMUSG00000049401
ENSMUSG00000030579	ENSMUSG00000049421
ENSMUSG00000030583	ENSMUSG00000049422
ENSMUSG00000030584	ENSMUSG00000049470
ENSMUSG00000030588	ENSMUSG00000049511
ENSMUSG00000030590	ENSMUSG00000049517
ENSMUSG00000030591	ENSMUSG00000049532
ENSMUSG00000030592	ENSMUSG00000049550
ENSMUSG00000030595	ENSMUSG00000049553
ENSMUSG00000030600	ENSMUSG00000049556
ENSMUSG00000030602	ENSMUSG00000049577
ENSMUSG00000030603	ENSMUSG00000049583
ENSMUSG00000030604	ENSMUSG00000049606
ENSMUSG00000030605	ENSMUSG00000049612
ENSMUSG00000030609	ENSMUSG00000049658
ENSMUSG00000030610	ENSMUSG00000049659
ENSMUSG00000030611	ENSMUSG00000049670
ENSMUSG00000030612	ENSMUSG00000049672
ENSMUSG00000030613	ENSMUSG00000049680
ENSMUSG00000030614	ENSMUSG00000049728
ENSMUSG00000030616	ENSMUSG00000049739
ENSMUSG00000030619	ENSMUSG00000049751
ENSMUSG00000030621	ENSMUSG00000049755
ENSMUSG00000030629	ENSMUSG00000049760
ENSMUSG00000030630	ENSMUSG00000049764
ENSMUSG00000030638	ENSMUSG00000049775
ENSMUSG00000030643	ENSMUSG00000049792
ENSMUSG00000030647	ENSMUSG00000049800
ENSMUSG00000030649	ENSMUSG00000049804
ENSMUSG00000030652	ENSMUSG00000049807
ENSMUSG00000030654	ENSMUSG00000049866
ENSMUSG00000030655	ENSMUSG00000049878
ENSMUSG00000030657	ENSMUSG00000049932
ENSMUSG00000030659	ENSMUSG00000049939
ENSMUSG00000030660	ENSMUSG00000049940
ENSMUSG00000030662	ENSMUSG00000050017
ENSMUSG00000030663	ENSMUSG00000050022
ENSMUSG00000030671	ENSMUSG00000050043
ENSMUSG00000030678	ENSMUSG00000050069
ENSMUSG00000030681	ENSMUSG00000050071
ENSMUSG00000030682	ENSMUSG00000050079
ENSMUSG00000030683	ENSMUSG00000050132
ENSMUSG00000030685	ENSMUSG00000050144
ENSMUSG00000030688	ENSMUSG00000050148
ENSMUSG00000030689	ENSMUSG00000050164
ENSMUSG00000030691	ENSMUSG00000050192
ENSMUSG00000030695	ENSMUSG00000050244
ENSMUSG00000030697	ENSMUSG00000050271
ENSMUSG00000030701	ENSMUSG00000050272
ENSMUSG00000030704	ENSMUSG00000050310
ENSMUSG00000030706	ENSMUSG00000050321

ENSMUSG00000030707	ENSMUSG00000050334
ENSMUSG00000030714	ENSMUSG00000050357
ENSMUSG00000030718	ENSMUSG00000050379
ENSMUSG00000030720	ENSMUSG00000050473
ENSMUSG00000030726	ENSMUSG00000050511
ENSMUSG00000030727	ENSMUSG00000050530
ENSMUSG00000030729	ENSMUSG00000050556
ENSMUSG00000030731	ENSMUSG00000050565
ENSMUSG00000030733	ENSMUSG00000050587
ENSMUSG00000030737	ENSMUSG00000050600
ENSMUSG00000030738	ENSMUSG00000050608
ENSMUSG00000030739	ENSMUSG00000050627
ENSMUSG00000030741	ENSMUSG00000050640
ENSMUSG00000030744	ENSMUSG00000050668
ENSMUSG00000030747	ENSMUSG00000050697
ENSMUSG00000030748	ENSMUSG00000050708
ENSMUSG00000030750	ENSMUSG00000050711
ENSMUSG00000030751	ENSMUSG00000050751
ENSMUSG00000030753	ENSMUSG00000050812
ENSMUSG00000030754	ENSMUSG00000050821
ENSMUSG00000030757	ENSMUSG00000050856
ENSMUSG00000030759	ENSMUSG00000050875
ENSMUSG00000030760	ENSMUSG00000050908
ENSMUSG00000030761	ENSMUSG00000050947
ENSMUSG00000030763	ENSMUSG00000050953
ENSMUSG00000030766	ENSMUSG00000050963
ENSMUSG00000030768	ENSMUSG00000050965
ENSMUSG00000030770	ENSMUSG00000050967
ENSMUSG00000030772	ENSMUSG00000051062
ENSMUSG00000030774	ENSMUSG00000051065
ENSMUSG00000030779	ENSMUSG00000051067
ENSMUSG00000030780	ENSMUSG00000051111
ENSMUSG00000030782	ENSMUSG00000051146
ENSMUSG00000030786	ENSMUSG00000051149
ENSMUSG00000030788	ENSMUSG00000051166
ENSMUSG00000030795	ENSMUSG00000051177
ENSMUSG00000030801	ENSMUSG00000051223
ENSMUSG00000030802	ENSMUSG00000051234
ENSMUSG00000030805	ENSMUSG00000051243
ENSMUSG00000030806	ENSMUSG00000051285
ENSMUSG00000030811	ENSMUSG00000051323
ENSMUSG00000030814	ENSMUSG00000051331
ENSMUSG00000030815	ENSMUSG00000051335
ENSMUSG00000030816	ENSMUSG00000051339
ENSMUSG00000030822	ENSMUSG00000051343
ENSMUSG00000030823	ENSMUSG00000051344
ENSMUSG00000030824	ENSMUSG00000051351
ENSMUSG00000030826	ENSMUSG00000051359
ENSMUSG00000030835	ENSMUSG00000051375
ENSMUSG00000030839	ENSMUSG00000051379
ENSMUSG00000030842	ENSMUSG00000051390
ENSMUSG00000030844	ENSMUSG00000051391
ENSMUSG00000030846	ENSMUSG00000051401

ENSMUSG00000030847	ENSMUSG00000051403
ENSMUSG00000030849	ENSMUSG00000051451
ENSMUSG00000030850	ENSMUSG00000051469
ENSMUSG00000030852	ENSMUSG00000051483
ENSMUSG00000030854	ENSMUSG00000051495
ENSMUSG00000030861	ENSMUSG00000051510
ENSMUSG00000030868	ENSMUSG00000051527
ENSMUSG00000030869	ENSMUSG00000051537
ENSMUSG00000030870	ENSMUSG00000051586
ENSMUSG00000030871	ENSMUSG00000051615
ENSMUSG00000030872	ENSMUSG00000051627
ENSMUSG00000030876	ENSMUSG00000051674
ENSMUSG00000030878	ENSMUSG00000051675
ENSMUSG00000030879	ENSMUSG00000051695
ENSMUSG00000030880	ENSMUSG00000051703
ENSMUSG00000030881	ENSMUSG00000051726
ENSMUSG00000030882	ENSMUSG00000051786
ENSMUSG00000030884	ENSMUSG00000051790
ENSMUSG00000030888	ENSMUSG00000051851
ENSMUSG00000030889	ENSMUSG00000051853
ENSMUSG00000030890	ENSMUSG00000051855
ENSMUSG00000030894	ENSMUSG00000051864
ENSMUSG00000030898	ENSMUSG00000051910
ENSMUSG00000030905	ENSMUSG00000051934
ENSMUSG00000030917	ENSMUSG00000051951
ENSMUSG00000030929	ENSMUSG00000052040
ENSMUSG00000030930	ENSMUSG00000052087
ENSMUSG00000030934	ENSMUSG00000052105
ENSMUSG00000030942	ENSMUSG00000052144
ENSMUSG00000030946	ENSMUSG00000052146
ENSMUSG00000030956	ENSMUSG00000052155
ENSMUSG00000030960	ENSMUSG00000052214
ENSMUSG00000030965	ENSMUSG00000052229
ENSMUSG00000030967	ENSMUSG00000052253
ENSMUSG00000030970	ENSMUSG00000052296
ENSMUSG00000030978	ENSMUSG00000052298
ENSMUSG00000030979	ENSMUSG00000052299
ENSMUSG00000030980	ENSMUSG00000052302
ENSMUSG00000030982	ENSMUSG00000052305
ENSMUSG00000030983	ENSMUSG00000052310
ENSMUSG00000030986	ENSMUSG00000052336
ENSMUSG00000030987	ENSMUSG00000052337
ENSMUSG00000030990	ENSMUSG00000052364
ENSMUSG00000031007	ENSMUSG00000052372
ENSMUSG00000031010	ENSMUSG00000052373
ENSMUSG00000031012	ENSMUSG00000052374
ENSMUSG00000031015	ENSMUSG00000052387
ENSMUSG00000031016	ENSMUSG00000052397
ENSMUSG00000031021	ENSMUSG00000052406
ENSMUSG00000031024	ENSMUSG00000052423
ENSMUSG00000031026	ENSMUSG00000052456
ENSMUSG00000031028	ENSMUSG00000052459
ENSMUSG00000031029	ENSMUSG00000052488

ENSMUSG00000031059	ENSMUSG00000052512
ENSMUSG00000031060	ENSMUSG00000052516
ENSMUSG00000031065	ENSMUSG00000052533
ENSMUSG00000031066	ENSMUSG00000052534
ENSMUSG00000031068	ENSMUSG00000052539
ENSMUSG00000031072	ENSMUSG00000052551
ENSMUSG00000031075	ENSMUSG00000052557
ENSMUSG00000031077	ENSMUSG00000052565
ENSMUSG00000031078	ENSMUSG00000052572
ENSMUSG00000031093	ENSMUSG00000052581
ENSMUSG00000031095	ENSMUSG00000052584
ENSMUSG00000031099	ENSMUSG00000052613
ENSMUSG00000031104	ENSMUSG00000052656
ENSMUSG00000031105	ENSMUSG00000052684
ENSMUSG00000031109	ENSMUSG00000052698
ENSMUSG00000031111	ENSMUSG00000052707
ENSMUSG00000031112	ENSMUSG00000052712
ENSMUSG00000031119	ENSMUSG00000052713
ENSMUSG00000031129	ENSMUSG00000052727
ENSMUSG00000031133	ENSMUSG00000052738
ENSMUSG00000031134	ENSMUSG00000052751
ENSMUSG00000031137	ENSMUSG00000052752
ENSMUSG00000031143	ENSMUSG00000052812
ENSMUSG00000031144	ENSMUSG00000052833
ENSMUSG00000031148	ENSMUSG00000052837
ENSMUSG00000031149	ENSMUSG00000052852
ENSMUSG00000031150	ENSMUSG00000052889
ENSMUSG00000031153	ENSMUSG00000052911
ENSMUSG00000031154	ENSMUSG00000052915
ENSMUSG00000031155	ENSMUSG00000052917
ENSMUSG00000031156	ENSMUSG00000052928
ENSMUSG00000031157	ENSMUSG00000052934
ENSMUSG00000031158	ENSMUSG00000052949
ENSMUSG00000031161	ENSMUSG00000052981
ENSMUSG00000031166	ENSMUSG00000052997
ENSMUSG00000031167	ENSMUSG00000053024
ENSMUSG00000031168	ENSMUSG00000053025
ENSMUSG00000031169	ENSMUSG00000053046
ENSMUSG00000031171	ENSMUSG00000053062
ENSMUSG00000031174	ENSMUSG00000053080
ENSMUSG00000031176	ENSMUSG00000053093
ENSMUSG00000031189	ENSMUSG00000053094
ENSMUSG00000031197	ENSMUSG00000053119
ENSMUSG00000031198	ENSMUSG00000053128
ENSMUSG00000031201	ENSMUSG00000053141
ENSMUSG00000031202	ENSMUSG00000053166
ENSMUSG00000031207	ENSMUSG00000053192
ENSMUSG00000031209	ENSMUSG00000053199
ENSMUSG00000031210	ENSMUSG00000053253
ENSMUSG00000031214	ENSMUSG00000053291
ENSMUSG00000031216	ENSMUSG00000053293
ENSMUSG00000031217	ENSMUSG00000053310
ENSMUSG00000031221	ENSMUSG00000053329

ENSMUSG00000031224	ENSMUSG00000053332
ENSMUSG00000031226	ENSMUSG00000053333
ENSMUSG00000031227	ENSMUSG00000053395
ENSMUSG00000031229	ENSMUSG00000053398
ENSMUSG00000031231	ENSMUSG00000053411
ENSMUSG00000031232	ENSMUSG00000053436
ENSMUSG00000031239	ENSMUSG00000053470
ENSMUSG00000031242	ENSMUSG00000053477
ENSMUSG00000031246	ENSMUSG00000053483
ENSMUSG00000031255	ENSMUSG00000053510
ENSMUSG00000031256	ENSMUSG00000053536
ENSMUSG00000031266	ENSMUSG00000053550
ENSMUSG00000031274	ENSMUSG00000053565
ENSMUSG00000031278	ENSMUSG00000053580
ENSMUSG00000031284	ENSMUSG00000053581
ENSMUSG00000031285	ENSMUSG00000053617
ENSMUSG00000031290	ENSMUSG00000053641
ENSMUSG00000031292	ENSMUSG00000053644
ENSMUSG00000031295	ENSMUSG00000053646
ENSMUSG00000031297	ENSMUSG00000053693
ENSMUSG00000031299	ENSMUSG00000053702
ENSMUSG00000031302	ENSMUSG00000053716
ENSMUSG00000031309	ENSMUSG00000053754
ENSMUSG00000031310	ENSMUSG00000053768
ENSMUSG00000031311	ENSMUSG00000053774
ENSMUSG00000031314	ENSMUSG00000053799
ENSMUSG00000031320	ENSMUSG00000053819
ENSMUSG00000031323	ENSMUSG00000053825
ENSMUSG00000031327	ENSMUSG00000053838
ENSMUSG00000031328	ENSMUSG00000053841
ENSMUSG00000031333	ENSMUSG00000053877
ENSMUSG00000031337	ENSMUSG00000053907
ENSMUSG00000031340	ENSMUSG00000053929
ENSMUSG00000031342	ENSMUSG00000053950
ENSMUSG00000031343	ENSMUSG00000053963
ENSMUSG00000031344	ENSMUSG00000054008
ENSMUSG00000031347	ENSMUSG00000054013
ENSMUSG00000031349	ENSMUSG00000054027
ENSMUSG00000031352	ENSMUSG00000054034
ENSMUSG00000031353	ENSMUSG00000054051
ENSMUSG00000031355	ENSMUSG00000054091
ENSMUSG00000031357	ENSMUSG00000054162
ENSMUSG00000031358	ENSMUSG00000054199
ENSMUSG00000031360	ENSMUSG00000054226
ENSMUSG00000031365	ENSMUSG00000054252
ENSMUSG00000031367	ENSMUSG00000054256
ENSMUSG00000031370	ENSMUSG00000054263
ENSMUSG00000031371	ENSMUSG00000054280
ENSMUSG00000031374	ENSMUSG00000054302
ENSMUSG00000031375	ENSMUSG00000054309
ENSMUSG00000031376	ENSMUSG00000054364
ENSMUSG00000031378	ENSMUSG00000054387
ENSMUSG00000031381	ENSMUSG00000054405

ENSMUSG00000031385	ENSMUSG00000054408
ENSMUSG00000031386	ENSMUSG00000054414
ENSMUSG00000031388	ENSMUSG00000054423
ENSMUSG00000031389	ENSMUSG00000054428
ENSMUSG00000031391	ENSMUSG00000054452
ENSMUSG00000031392	ENSMUSG00000054455
ENSMUSG00000031393	ENSMUSG00000054459
ENSMUSG00000031398	ENSMUSG00000054469
ENSMUSG00000031399	ENSMUSG00000054499
ENSMUSG00000031400	ENSMUSG00000054604
ENSMUSG00000031402	ENSMUSG00000054611
ENSMUSG00000031403	ENSMUSG00000054640
ENSMUSG00000031409	ENSMUSG00000054648
ENSMUSG00000031422	ENSMUSG00000054675
ENSMUSG00000031425	ENSMUSG00000054693
ENSMUSG00000031428	ENSMUSG00000054720
ENSMUSG00000031429	ENSMUSG00000054728
ENSMUSG00000031431	ENSMUSG00000054766
ENSMUSG00000031432	ENSMUSG00000054792
ENSMUSG00000031434	ENSMUSG00000054793
ENSMUSG00000031438	ENSMUSG00000054808
ENSMUSG00000031441	ENSMUSG00000054814
ENSMUSG00000031442	ENSMUSG00000054823
ENSMUSG00000031446	ENSMUSG00000054843
ENSMUSG00000031447	ENSMUSG00000054863
ENSMUSG00000031451	ENSMUSG00000054871
ENSMUSG00000031453	ENSMUSG00000054874
ENSMUSG00000031458	ENSMUSG00000054920
ENSMUSG00000031467	ENSMUSG00000054934
ENSMUSG00000031479	ENSMUSG00000054942
ENSMUSG00000031482	ENSMUSG00000054976
ENSMUSG00000031483	ENSMUSG00000055003
ENSMUSG00000031485	ENSMUSG00000055013
ENSMUSG00000031486	ENSMUSG00000055022
ENSMUSG00000031487	ENSMUSG00000055024
ENSMUSG00000031502	ENSMUSG00000055026
ENSMUSG00000031503	ENSMUSG00000055053
ENSMUSG00000031505	ENSMUSG00000055065
ENSMUSG00000031508	ENSMUSG00000055067
ENSMUSG00000031511	ENSMUSG00000055204
ENSMUSG00000031513	ENSMUSG00000055239
ENSMUSG00000031516	ENSMUSG00000055254
ENSMUSG00000031517	ENSMUSG00000055296
ENSMUSG00000031523	ENSMUSG00000055302
ENSMUSG00000031527	ENSMUSG00000055319
ENSMUSG00000031529	ENSMUSG00000055322
ENSMUSG00000031530	ENSMUSG00000055371
ENSMUSG00000031532	ENSMUSG00000055373
ENSMUSG00000031533	ENSMUSG00000055407
ENSMUSG00000031534	ENSMUSG00000055409
ENSMUSG00000031536	ENSMUSG00000055421
ENSMUSG00000031537	ENSMUSG00000055430
ENSMUSG00000031538	ENSMUSG00000055436

ENSMUSG00000031539	ENSMUSG00000055447
ENSMUSG00000031540	ENSMUSG00000055471
ENSMUSG00000031543	ENSMUSG00000055485
ENSMUSG00000031545	ENSMUSG00000055493
ENSMUSG00000031546	ENSMUSG00000055531
ENSMUSG00000031548	ENSMUSG00000055538
ENSMUSG00000031555	ENSMUSG00000055540
ENSMUSG00000031556	ENSMUSG00000055567
ENSMUSG00000031557	ENSMUSG00000055629
ENSMUSG00000031558	ENSMUSG00000055639
ENSMUSG00000031561	ENSMUSG00000055670
ENSMUSG00000031562	ENSMUSG00000055675
ENSMUSG00000031563	ENSMUSG00000055681
ENSMUSG00000031565	ENSMUSG00000055692
ENSMUSG00000031568	ENSMUSG00000055707
ENSMUSG00000031570	ENSMUSG00000055717
ENSMUSG00000031575	ENSMUSG00000055733
ENSMUSG00000031577	ENSMUSG00000055745
ENSMUSG00000031578	ENSMUSG00000055762
ENSMUSG00000031583	ENSMUSG00000055805
ENSMUSG00000031584	ENSMUSG00000055817
ENSMUSG00000031585	ENSMUSG00000055839
ENSMUSG00000031590	ENSMUSG00000055850
ENSMUSG00000031591	ENSMUSG00000055866
ENSMUSG00000031592	ENSMUSG00000055932
ENSMUSG00000031596	ENSMUSG00000055943
ENSMUSG00000031600	ENSMUSG00000055945
ENSMUSG00000031601	ENSMUSG00000056004
ENSMUSG00000031604	ENSMUSG00000056050
ENSMUSG00000031605	ENSMUSG00000056073
ENSMUSG00000031608	ENSMUSG00000056076
ENSMUSG00000031610	ENSMUSG00000056091
ENSMUSG00000031617	ENSMUSG00000056124
ENSMUSG00000031618	ENSMUSG00000056167
ENSMUSG00000031622	ENSMUSG00000056185
ENSMUSG00000031626	ENSMUSG00000056201
ENSMUSG00000031627	ENSMUSG00000056211
ENSMUSG00000031628	ENSMUSG00000056222
ENSMUSG00000031631	ENSMUSG00000056234
ENSMUSG00000031633	ENSMUSG00000056258
ENSMUSG00000031634	ENSMUSG00000056268
ENSMUSG00000031639	ENSMUSG00000056296
ENSMUSG00000031641	ENSMUSG00000056342
ENSMUSG00000031642	ENSMUSG00000056367
ENSMUSG00000031644	ENSMUSG00000056413
ENSMUSG00000031647	ENSMUSG00000056427
ENSMUSG00000031652	ENSMUSG00000056429
ENSMUSG00000031654	ENSMUSG00000056476
ENSMUSG00000031657	ENSMUSG00000056486
ENSMUSG00000031659	ENSMUSG00000056493
ENSMUSG00000031660	ENSMUSG00000056515
ENSMUSG00000031661	ENSMUSG00000056537
ENSMUSG00000031665	ENSMUSG00000056553

ENSMUSG00000031666	ENSMUSG00000056579
ENSMUSG00000031667	ENSMUSG00000056596
ENSMUSG00000031668	ENSMUSG00000056602
ENSMUSG00000031671	ENSMUSG00000056608
ENSMUSG00000031672	ENSMUSG00000056629
ENSMUSG00000031673	ENSMUSG00000056665
ENSMUSG00000031681	ENSMUSG00000056692
ENSMUSG00000031683	ENSMUSG00000056755
ENSMUSG00000031691	ENSMUSG00000056770
ENSMUSG00000031696	ENSMUSG00000056812
ENSMUSG00000031697	ENSMUSG00000056820
ENSMUSG00000031700	ENSMUSG00000056851
ENSMUSG00000031701	ENSMUSG00000056854
ENSMUSG00000031703	ENSMUSG00000056856
ENSMUSG00000031706	ENSMUSG00000056900
ENSMUSG00000031708	ENSMUSG00000056952
ENSMUSG00000031709	ENSMUSG00000056999
ENSMUSG00000031711	ENSMUSG00000057060
ENSMUSG00000031714	ENSMUSG00000057101
ENSMUSG00000031715	ENSMUSG00000057113
ENSMUSG00000031723	ENSMUSG00000057133
ENSMUSG00000031728	ENSMUSG00000057134
ENSMUSG00000031729	ENSMUSG00000057177
ENSMUSG00000031730	ENSMUSG00000057181
ENSMUSG00000031731	ENSMUSG00000057182
ENSMUSG00000031732	ENSMUSG00000057193
ENSMUSG00000031748	ENSMUSG00000057229
ENSMUSG00000031749	ENSMUSG00000057230
ENSMUSG00000031750	ENSMUSG00000057236
ENSMUSG00000031751	ENSMUSG00000057322
ENSMUSG00000031753	ENSMUSG00000057329
ENSMUSG00000031754	ENSMUSG00000057335
ENSMUSG00000031755	ENSMUSG00000057342
ENSMUSG00000031758	ENSMUSG00000057363
ENSMUSG00000031760	ENSMUSG00000057367
ENSMUSG00000031762	ENSMUSG00000057378
ENSMUSG00000031765	ENSMUSG00000057406
ENSMUSG00000031770	ENSMUSG00000057411
ENSMUSG00000031772	ENSMUSG00000057421
ENSMUSG00000031774	ENSMUSG00000057455
ENSMUSG00000031775	ENSMUSG00000057522
ENSMUSG00000031776	ENSMUSG00000057530
ENSMUSG00000031778	ENSMUSG00000057531
ENSMUSG00000031781	ENSMUSG00000057561
ENSMUSG00000031782	ENSMUSG00000057614
ENSMUSG00000031783	ENSMUSG00000057637
ENSMUSG00000031785	ENSMUSG00000057649
ENSMUSG00000031786	ENSMUSG00000057666
ENSMUSG00000031787	ENSMUSG00000057672
ENSMUSG00000031788	ENSMUSG00000057691
ENSMUSG00000031790	ENSMUSG00000057715
ENSMUSG00000031791	ENSMUSG00000057716
ENSMUSG00000031792	ENSMUSG00000057719

ENSMUSG00000031796	ENSMUSG00000057738
ENSMUSG00000031799	ENSMUSG00000057841
ENSMUSG00000031807	ENSMUSG00000057858
ENSMUSG00000031808	ENSMUSG00000057863
ENSMUSG00000031812	ENSMUSG00000057880
ENSMUSG00000031813	ENSMUSG00000057894
ENSMUSG00000031816	ENSMUSG00000057897
ENSMUSG00000031818	ENSMUSG00000057914
ENSMUSG00000031819	ENSMUSG00000057963
ENSMUSG00000031820	ENSMUSG00000058006
ENSMUSG00000031822	ENSMUSG00000058013
ENSMUSG00000031823	ENSMUSG00000058070
ENSMUSG00000031824	ENSMUSG00000058076
ENSMUSG00000031826	ENSMUSG00000058126
ENSMUSG00000031827	ENSMUSG00000058135
ENSMUSG00000031828	ENSMUSG00000058152
ENSMUSG00000031832	ENSMUSG00000058153
ENSMUSG00000031833	ENSMUSG00000058230
ENSMUSG00000031834	ENSMUSG00000058239
ENSMUSG00000031835	ENSMUSG00000058240
ENSMUSG00000031837	ENSMUSG00000058248
ENSMUSG00000031839	ENSMUSG00000058254
ENSMUSG00000031840	ENSMUSG00000058260
ENSMUSG00000031841	ENSMUSG00000058291
ENSMUSG00000031843	ENSMUSG00000058297
ENSMUSG00000031847	ENSMUSG00000058301
ENSMUSG00000031848	ENSMUSG00000058317
ENSMUSG00000031851	ENSMUSG00000058318
ENSMUSG00000031858	ENSMUSG00000058325
ENSMUSG00000031860	ENSMUSG00000058355
ENSMUSG00000031862	ENSMUSG00000058388
ENSMUSG00000031864	ENSMUSG00000058407
ENSMUSG00000031865	ENSMUSG00000058441
ENSMUSG00000031870	ENSMUSG00000058454
ENSMUSG00000031871	ENSMUSG00000058546
ENSMUSG00000031872	ENSMUSG00000058558
ENSMUSG00000031875	ENSMUSG00000058569
ENSMUSG00000031878	ENSMUSG00000058589
ENSMUSG00000031879	ENSMUSG00000058594
ENSMUSG00000031885	ENSMUSG00000058600
ENSMUSG00000031889	ENSMUSG00000058624
ENSMUSG00000031897	ENSMUSG00000058655
ENSMUSG00000031901	ENSMUSG00000058656
ENSMUSG00000031902	ENSMUSG00000058672
ENSMUSG00000031903	ENSMUSG00000058690
ENSMUSG00000031904	ENSMUSG00000058709
ENSMUSG00000031906	ENSMUSG00000058740
ENSMUSG00000031907	ENSMUSG00000058756
ENSMUSG00000031913	ENSMUSG00000058761
ENSMUSG00000031916	ENSMUSG00000058793
ENSMUSG00000031917	ENSMUSG00000058799
ENSMUSG00000031918	ENSMUSG00000058833
ENSMUSG00000031921	ENSMUSG00000058835

ENSMUSG00000031922	ENSMUSG00000058881
ENSMUSG00000031924	ENSMUSG00000058886
ENSMUSG00000031925	ENSMUSG00000058897
ENSMUSG00000031928	ENSMUSG00000058966
ENSMUSG00000031930	ENSMUSG00000058975
ENSMUSG00000031931	ENSMUSG00000058997
ENSMUSG00000031932	ENSMUSG00000059003
ENSMUSG00000031934	ENSMUSG00000059005
ENSMUSG00000031935	ENSMUSG00000059040
ENSMUSG00000031937	ENSMUSG00000059070
ENSMUSG00000031938	ENSMUSG00000059119
ENSMUSG00000031939	ENSMUSG00000059146
ENSMUSG00000031948	ENSMUSG00000059173
ENSMUSG00000031949	ENSMUSG00000059208
ENSMUSG00000031950	ENSMUSG00000059213
ENSMUSG00000031951	ENSMUSG00000059248
ENSMUSG00000031954	ENSMUSG00000059263
ENSMUSG00000031955	ENSMUSG00000059273
ENSMUSG00000031958	ENSMUSG00000059288
ENSMUSG00000031959	ENSMUSG00000059291
ENSMUSG00000031960	ENSMUSG00000059316
ENSMUSG00000031963	ENSMUSG00000059361
ENSMUSG00000031967	ENSMUSG00000059409
ENSMUSG00000031969	ENSMUSG00000059436
ENSMUSG00000031970	ENSMUSG00000059439
ENSMUSG00000031971	ENSMUSG00000059447
ENSMUSG00000031974	ENSMUSG00000059456
ENSMUSG00000031976	ENSMUSG00000059474
ENSMUSG00000031979	ENSMUSG00000059486
ENSMUSG00000031980	ENSMUSG00000059493
ENSMUSG00000031982	ENSMUSG00000059495
ENSMUSG00000031983	ENSMUSG00000059518
ENSMUSG00000031984	ENSMUSG00000059534
ENSMUSG00000031985	ENSMUSG00000059602
ENSMUSG00000031986	ENSMUSG00000059713
ENSMUSG00000031987	ENSMUSG00000059714
ENSMUSG00000031988	ENSMUSG00000059734
ENSMUSG00000031990	ENSMUSG00000059743
ENSMUSG00000031993	ENSMUSG00000059796
ENSMUSG00000031996	ENSMUSG00000059811
ENSMUSG00000031997	ENSMUSG00000059824
ENSMUSG00000032002	ENSMUSG00000059890
ENSMUSG00000032009	ENSMUSG00000059895
ENSMUSG00000032010	ENSMUSG00000059921
ENSMUSG00000032011	ENSMUSG00000059923
ENSMUSG00000032012	ENSMUSG00000059970
ENSMUSG00000032014	ENSMUSG00000059974
ENSMUSG00000032017	ENSMUSG00000059981
ENSMUSG00000032018	ENSMUSG00000059995
ENSMUSG00000032020	ENSMUSG00000060002
ENSMUSG00000032024	ENSMUSG00000060012
ENSMUSG00000032026	ENSMUSG00000060032
ENSMUSG00000032030	ENSMUSG00000060036

ENSMUSG00000032034	ENSMUSG00000060073
ENSMUSG00000032035	ENSMUSG00000060093
ENSMUSG00000032036	ENSMUSG00000060098
ENSMUSG00000032038	ENSMUSG00000060126
ENSMUSG00000032040	ENSMUSG00000060166
ENSMUSG00000032042	ENSMUSG00000060206
ENSMUSG00000032044	ENSMUSG00000060212
ENSMUSG00000032046	ENSMUSG00000060216
ENSMUSG00000032047	ENSMUSG00000060227
ENSMUSG00000032050	ENSMUSG00000060240
ENSMUSG00000032051	ENSMUSG00000060261
ENSMUSG00000032058	ENSMUSG00000060279
ENSMUSG00000032059	ENSMUSG00000060301
ENSMUSG00000032060	ENSMUSG00000060314
ENSMUSG00000032064	ENSMUSG00000060371
ENSMUSG00000032067	ENSMUSG00000060373
ENSMUSG00000032076	ENSMUSG00000060376
ENSMUSG00000032077	ENSMUSG00000060450
ENSMUSG00000032078	ENSMUSG00000060475
ENSMUSG00000032086	ENSMUSG00000060510
ENSMUSG00000032087	ENSMUSG00000060534
ENSMUSG00000032089	ENSMUSG00000060548
ENSMUSG00000032096	ENSMUSG00000060568
ENSMUSG00000032097	ENSMUSG00000060601
ENSMUSG00000032101	ENSMUSG00000060636
ENSMUSG00000032109	ENSMUSG00000060639
ENSMUSG00000032112	ENSMUSG00000060657
ENSMUSG00000032114	ENSMUSG00000060671
ENSMUSG00000032115	ENSMUSG00000060675
ENSMUSG00000032116	ENSMUSG00000060678
ENSMUSG00000032118	ENSMUSG00000060681
ENSMUSG00000032119	ENSMUSG00000060716
ENSMUSG00000032120	ENSMUSG00000060733
ENSMUSG00000032121	ENSMUSG00000060739
ENSMUSG00000032123	ENSMUSG00000060743
ENSMUSG00000032125	ENSMUSG00000060780
ENSMUSG00000032126	ENSMUSG00000060803
ENSMUSG00000032127	ENSMUSG00000060860
ENSMUSG00000032131	ENSMUSG00000060882
ENSMUSG00000032135	ENSMUSG00000060904
ENSMUSG00000032171	ENSMUSG00000060924
ENSMUSG00000032172	ENSMUSG00000060935
ENSMUSG00000032174	ENSMUSG00000060938
ENSMUSG00000032175	ENSMUSG00000060961
ENSMUSG00000032177	ENSMUSG00000060981
ENSMUSG00000032178	ENSMUSG00000060988
ENSMUSG00000032180	ENSMUSG00000060992
ENSMUSG00000032181	ENSMUSG00000061028
ENSMUSG00000032182	ENSMUSG00000061032
ENSMUSG00000032184	ENSMUSG00000061046
ENSMUSG00000032185	ENSMUSG00000061080
ENSMUSG00000032186	ENSMUSG00000061111
ENSMUSG00000032187	ENSMUSG00000061130

ENSMUSG00000032192	ENSMUSG00000061136
ENSMUSG00000032193	ENSMUSG00000061175
ENSMUSG00000032194	ENSMUSG00000061244
ENSMUSG00000032198	ENSMUSG00000061288
ENSMUSG00000032199	ENSMUSG00000061306
ENSMUSG00000032202	ENSMUSG00000061313
ENSMUSG00000032212	ENSMUSG00000061315
ENSMUSG00000032215	ENSMUSG00000061353
ENSMUSG00000032216	ENSMUSG00000061410
ENSMUSG00000032217	ENSMUSG00000061411
ENSMUSG00000032220	ENSMUSG00000061436
ENSMUSG00000032221	ENSMUSG00000061451
ENSMUSG00000032224	ENSMUSG00000061477
ENSMUSG00000032228	ENSMUSG00000061479
ENSMUSG00000032231	ENSMUSG00000061482
ENSMUSG00000032232	ENSMUSG00000061518
ENSMUSG00000032235	ENSMUSG00000061536
ENSMUSG00000032238	ENSMUSG00000061576
ENSMUSG00000032239	ENSMUSG00000061578
ENSMUSG00000032244	ENSMUSG00000061589
ENSMUSG00000032245	ENSMUSG00000061601
ENSMUSG00000032249	ENSMUSG00000061603
ENSMUSG00000032251	ENSMUSG00000061650
ENSMUSG00000032252	ENSMUSG00000061666
ENSMUSG00000032253	ENSMUSG00000061689
ENSMUSG00000032258	ENSMUSG00000061702
ENSMUSG00000032259	ENSMUSG00000061718
ENSMUSG00000032261	ENSMUSG00000061731
ENSMUSG00000032262	ENSMUSG00000061740
ENSMUSG00000032263	ENSMUSG00000061751
ENSMUSG00000032264	ENSMUSG00000061755
ENSMUSG00000032265	ENSMUSG00000061762
ENSMUSG00000032267	ENSMUSG00000061787
ENSMUSG00000032279	ENSMUSG00000061859
ENSMUSG00000032280	ENSMUSG00000061887
ENSMUSG00000032281	ENSMUSG00000061904
ENSMUSG00000032285	ENSMUSG00000061911
ENSMUSG00000032288	ENSMUSG00000061981
ENSMUSG00000032289	ENSMUSG00000061983
ENSMUSG00000032290	ENSMUSG00000062006
ENSMUSG00000032293	ENSMUSG00000062014
ENSMUSG00000032294	ENSMUSG00000062044
ENSMUSG00000032295	ENSMUSG00000062070
ENSMUSG00000032297	ENSMUSG00000062078
ENSMUSG00000032298	ENSMUSG00000062098
ENSMUSG00000032299	ENSMUSG00000062115
ENSMUSG00000032300	ENSMUSG00000062127
ENSMUSG00000032301	ENSMUSG00000062151
ENSMUSG00000032305	ENSMUSG00000062184
ENSMUSG00000032306	ENSMUSG00000062190
ENSMUSG00000032307	ENSMUSG00000062202
ENSMUSG00000032308	ENSMUSG00000062203
ENSMUSG00000032309	ENSMUSG00000062209

ENSMUSG00000032312	ENSMUSG00000062232
ENSMUSG00000032313	ENSMUSG00000062234
ENSMUSG00000032314	ENSMUSG00000062257
ENSMUSG00000032316	ENSMUSG00000062270
ENSMUSG00000032320	ENSMUSG00000062296
ENSMUSG00000032324	ENSMUSG00000062328
ENSMUSG00000032328	ENSMUSG00000062352
ENSMUSG00000032329	ENSMUSG00000062353
ENSMUSG00000032330	ENSMUSG00000062372
ENSMUSG00000032332	ENSMUSG00000062373
ENSMUSG00000032333	ENSMUSG00000062380
ENSMUSG00000032334	ENSMUSG00000062397
ENSMUSG00000032336	ENSMUSG00000062421
ENSMUSG00000032338	ENSMUSG00000062444
ENSMUSG00000032340	ENSMUSG00000062519
ENSMUSG00000032342	ENSMUSG00000062580
ENSMUSG00000032348	ENSMUSG00000062590
ENSMUSG00000032349	ENSMUSG00000062591
ENSMUSG00000032350	ENSMUSG00000062604
ENSMUSG00000032352	ENSMUSG00000062627
ENSMUSG00000032353	ENSMUSG00000062647
ENSMUSG00000032355	ENSMUSG00000062661
ENSMUSG00000032356	ENSMUSG00000062683
ENSMUSG00000032359	ENSMUSG00000062760
ENSMUSG00000032366	ENSMUSG00000062761
ENSMUSG00000032368	ENSMUSG00000062762
ENSMUSG00000032370	ENSMUSG00000062785
ENSMUSG00000032373	ENSMUSG00000062797
ENSMUSG00000032374	ENSMUSG00000062825
ENSMUSG00000032375	ENSMUSG00000062867
ENSMUSG00000032376	ENSMUSG00000062901
ENSMUSG00000032381	ENSMUSG00000062929
ENSMUSG00000032382	ENSMUSG00000062963
ENSMUSG00000032383	ENSMUSG00000062995
ENSMUSG00000032384	ENSMUSG00000062997
ENSMUSG00000032386	ENSMUSG00000063015
ENSMUSG00000032388	ENSMUSG00000063019
ENSMUSG00000032393	ENSMUSG00000063063
ENSMUSG00000032396	ENSMUSG00000063065
ENSMUSG00000032399	ENSMUSG00000063077
ENSMUSG00000032402	ENSMUSG00000063108
ENSMUSG00000032405	ENSMUSG00000063142
ENSMUSG00000032407	ENSMUSG00000063146
ENSMUSG00000032409	ENSMUSG00000063160
ENSMUSG00000032410	ENSMUSG00000063229
ENSMUSG00000032411	ENSMUSG00000063239
ENSMUSG00000032412	ENSMUSG00000063253
ENSMUSG00000032413	ENSMUSG00000063260
ENSMUSG00000032415	ENSMUSG00000063273
ENSMUSG00000032417	ENSMUSG00000063297
ENSMUSG00000032418	ENSMUSG00000063316
ENSMUSG00000032420	ENSMUSG00000063317
ENSMUSG00000032422	ENSMUSG00000063334

ENSMUSG00000032423	ENSMUSG00000063358
ENSMUSG00000032425	ENSMUSG00000063362
ENSMUSG00000032434	ENSMUSG00000063382
ENSMUSG00000032435	ENSMUSG00000063410
ENSMUSG00000032437	ENSMUSG00000063430
ENSMUSG00000032440	ENSMUSG00000063434
ENSMUSG00000032449	ENSMUSG00000063446
ENSMUSG00000032452	ENSMUSG00000063455
ENSMUSG00000032458	ENSMUSG00000063457
ENSMUSG00000032459	ENSMUSG00000063480
ENSMUSG00000032462	ENSMUSG00000063511
ENSMUSG00000032463	ENSMUSG00000063524
ENSMUSG00000032468	ENSMUSG00000063531
ENSMUSG00000032469	ENSMUSG00000063550
ENSMUSG00000032470	ENSMUSG00000063576
ENSMUSG00000032475	ENSMUSG00000063626
ENSMUSG00000032477	ENSMUSG00000063632
ENSMUSG00000032478	ENSMUSG00000063646
ENSMUSG00000032479	ENSMUSG00000063659
ENSMUSG00000032480	ENSMUSG00000063694
ENSMUSG00000032481	ENSMUSG00000063801
ENSMUSG00000032482	ENSMUSG00000063802
ENSMUSG00000032485	ENSMUSG00000063856
ENSMUSG00000032489	ENSMUSG00000063870
ENSMUSG00000032497	ENSMUSG00000063873
ENSMUSG00000032498	ENSMUSG00000063882
ENSMUSG00000032500	ENSMUSG00000063884
ENSMUSG00000032501	ENSMUSG00000063887
ENSMUSG00000032502	ENSMUSG00000063894
ENSMUSG00000032503	ENSMUSG00000063904
ENSMUSG00000032504	ENSMUSG00000063919
ENSMUSG00000032507	ENSMUSG00000063952
ENSMUSG00000032511	ENSMUSG00000064043
ENSMUSG00000032512	ENSMUSG00000064061
ENSMUSG00000032513	ENSMUSG00000064105
ENSMUSG00000032514	ENSMUSG00000064115
ENSMUSG00000032515	ENSMUSG00000064127
ENSMUSG00000032517	ENSMUSG00000064138
ENSMUSG00000032518	ENSMUSG00000064145
ENSMUSG00000032519	ENSMUSG00000064193
ENSMUSG00000032523	ENSMUSG00000064225
ENSMUSG00000032525	ENSMUSG00000064264
ENSMUSG00000032526	ENSMUSG00000064288
ENSMUSG00000032527	ENSMUSG00000064302
ENSMUSG00000032531	ENSMUSG00000064329
ENSMUSG00000032532	ENSMUSG00000064373
ENSMUSG00000032534	ENSMUSG00000064387
ENSMUSG00000032536	ENSMUSG00000064702
ENSMUSG00000032537	ENSMUSG00000064856
ENSMUSG00000032540	ENSMUSG00000065037
ENSMUSG00000032547	ENSMUSG00000065251
ENSMUSG00000032549	ENSMUSG00000065353
ENSMUSG00000032551	ENSMUSG00000065649

ENSMUSG00000032553	ENSMUSG00000065701
ENSMUSG00000032554	ENSMUSG00000065767
ENSMUSG00000032555	ENSMUSG00000065944
ENSMUSG00000032557	ENSMUSG00000065954
ENSMUSG00000032558	ENSMUSG00000065979
ENSMUSG00000032560	ENSMUSG00000065990
ENSMUSG00000032562	ENSMUSG00000066036
ENSMUSG00000032563	ENSMUSG00000066037
ENSMUSG00000032564	ENSMUSG00000066129
ENSMUSG00000032565	ENSMUSG00000066151
ENSMUSG00000032570	ENSMUSG00000066189
ENSMUSG00000032571	ENSMUSG00000066232
ENSMUSG00000032575	ENSMUSG00000066235
ENSMUSG00000032578	ENSMUSG00000066306
ENSMUSG00000032579	ENSMUSG00000066324
ENSMUSG00000032580	ENSMUSG00000066357
ENSMUSG00000032582	ENSMUSG00000066392
ENSMUSG00000032583	ENSMUSG00000066406
ENSMUSG00000032586	ENSMUSG00000066415
ENSMUSG00000032589	ENSMUSG00000066440
ENSMUSG00000032590	ENSMUSG00000066551
ENSMUSG00000032594	ENSMUSG00000066568
ENSMUSG00000032595	ENSMUSG00000066571
ENSMUSG00000032598	ENSMUSG00000066607
ENSMUSG00000032599	ENSMUSG00000066621
ENSMUSG00000032601	ENSMUSG00000066640
ENSMUSG00000032602	ENSMUSG00000066643
ENSMUSG00000032604	ENSMUSG00000066647
ENSMUSG00000032606	ENSMUSG00000066687
ENSMUSG00000032607	ENSMUSG00000066705
ENSMUSG00000032609	ENSMUSG00000066735
ENSMUSG00000032612	ENSMUSG00000066839
ENSMUSG00000032615	ENSMUSG00000066900
ENSMUSG00000032621	ENSMUSG00000066979
ENSMUSG00000032624	ENSMUSG00000067028
ENSMUSG00000032625	ENSMUSG00000067150
ENSMUSG00000032633	ENSMUSG00000067194
ENSMUSG00000032637	ENSMUSG00000067242
ENSMUSG00000032640	ENSMUSG00000067274
ENSMUSG00000032641	ENSMUSG00000067279
ENSMUSG00000032648	ENSMUSG00000067288
ENSMUSG00000032649	ENSMUSG00000067336
ENSMUSG00000032652	ENSMUSG00000067629
ENSMUSG00000032656	ENSMUSG00000067713
ENSMUSG00000032657	ENSMUSG00000067736
ENSMUSG00000032666	ENSMUSG00000067786
ENSMUSG00000032667	ENSMUSG00000067787
ENSMUSG00000032679	ENSMUSG00000067847
ENSMUSG00000032688	ENSMUSG00000067851
ENSMUSG00000032698	ENSMUSG00000067873
ENSMUSG00000032702	ENSMUSG00000067889
ENSMUSG00000032705	ENSMUSG00000067924
ENSMUSG00000032712	ENSMUSG00000067925

ENSMUSG00000032714	ENSMUSG00000068036
ENSMUSG00000032724	ENSMUSG00000068039
ENSMUSG00000032727	ENSMUSG00000068040
ENSMUSG00000032733	ENSMUSG00000068099
ENSMUSG00000032735	ENSMUSG00000068205
ENSMUSG00000032737	ENSMUSG00000068206
ENSMUSG00000032740	ENSMUSG00000068267
ENSMUSG00000032741	ENSMUSG00000068284
ENSMUSG00000032743	ENSMUSG00000068290
ENSMUSG00000032744	ENSMUSG00000068328
ENSMUSG00000032745	ENSMUSG00000068373
ENSMUSG00000032754	ENSMUSG00000068396
ENSMUSG00000032757	ENSMUSG00000068479
ENSMUSG00000032763	ENSMUSG00000068551
ENSMUSG00000032766	ENSMUSG00000068663
ENSMUSG00000032773	ENSMUSG00000068696
ENSMUSG00000032777	ENSMUSG00000068732
ENSMUSG00000032782	ENSMUSG00000068735
ENSMUSG00000032786	ENSMUSG00000068739
ENSMUSG00000032788	ENSMUSG00000068740
ENSMUSG00000032796	ENSMUSG00000068742
ENSMUSG00000032802	ENSMUSG00000068747
ENSMUSG00000032803	ENSMUSG00000068748
ENSMUSG00000032812	ENSMUSG00000068749
ENSMUSG00000032816	ENSMUSG00000068823
ENSMUSG00000032826	ENSMUSG00000068856
ENSMUSG00000032827	ENSMUSG00000068882
ENSMUSG00000032834	ENSMUSG00000068921
ENSMUSG00000032839	ENSMUSG00000068923
ENSMUSG00000032840	ENSMUSG00000069020
ENSMUSG00000032841	ENSMUSG00000069072
ENSMUSG00000032842	ENSMUSG00000069117
ENSMUSG00000032846	ENSMUSG00000069227
ENSMUSG00000032849	ENSMUSG00000069237
ENSMUSG00000032850	ENSMUSG00000069305
ENSMUSG00000032854	ENSMUSG00000069495
ENSMUSG00000032855	ENSMUSG00000069520
ENSMUSG00000032867	ENSMUSG00000069565
ENSMUSG00000032869	ENSMUSG00000069601
ENSMUSG00000032870	ENSMUSG00000069662
ENSMUSG00000032872	ENSMUSG00000069670
ENSMUSG00000032875	ENSMUSG00000069729
ENSMUSG00000032878	ENSMUSG00000069744
ENSMUSG00000032883	ENSMUSG00000069769
ENSMUSG00000032890	ENSMUSG00000069806
ENSMUSG00000032892	ENSMUSG00000069814
ENSMUSG00000032897	ENSMUSG00000069833
ENSMUSG00000032898	ENSMUSG00000069895
ENSMUSG00000032902	ENSMUSG00000069919
ENSMUSG00000032905	ENSMUSG00000070000
ENSMUSG00000032908	ENSMUSG00000070003
ENSMUSG00000032911	ENSMUSG00000070047
ENSMUSG00000032913	ENSMUSG00000070056

ENSMUSG00000032932	ENSMUSG00000070304
ENSMUSG00000032936	ENSMUSG00000070319
ENSMUSG00000032939	ENSMUSG00000070426
ENSMUSG00000032946	ENSMUSG00000070462
ENSMUSG00000032952	ENSMUSG00000070476
ENSMUSG00000032959	ENSMUSG00000070493
ENSMUSG00000032965	ENSMUSG00000070498
ENSMUSG00000032966	ENSMUSG00000070509
ENSMUSG00000032968	ENSMUSG00000070544
ENSMUSG00000032977	ENSMUSG00000070565
ENSMUSG00000032997	ENSMUSG00000070570
ENSMUSG00000032998	ENSMUSG00000070576
ENSMUSG00000033004	ENSMUSG00000070639
ENSMUSG00000033006	ENSMUSG00000070695
ENSMUSG00000033007	ENSMUSG00000070730
ENSMUSG00000033009	ENSMUSG00000070733
ENSMUSG00000033014	ENSMUSG00000070738
ENSMUSG00000033016	ENSMUSG00000070802
ENSMUSG00000033020	ENSMUSG00000070822
ENSMUSG00000033021	ENSMUSG00000070880
ENSMUSG00000033022	ENSMUSG00000070923
ENSMUSG00000033032	ENSMUSG00000070934
ENSMUSG00000033039	ENSMUSG00000070939
ENSMUSG00000033047	ENSMUSG00000071014
ENSMUSG00000033054	ENSMUSG00000071054
ENSMUSG00000033055	ENSMUSG00000071064
ENSMUSG00000033059	ENSMUSG00000071072
ENSMUSG00000033060	ENSMUSG00000071074
ENSMUSG00000033061	ENSMUSG00000071076
ENSMUSG00000033063	ENSMUSG00000071172
ENSMUSG00000033065	ENSMUSG00000071176
ENSMUSG00000033066	ENSMUSG00000071234
ENSMUSG00000033068	ENSMUSG00000071337
ENSMUSG00000033075	ENSMUSG00000071359
ENSMUSG00000033083	ENSMUSG00000071369
ENSMUSG00000033088	ENSMUSG00000071379
ENSMUSG00000033096	ENSMUSG00000071415
ENSMUSG00000033099	ENSMUSG00000071424
ENSMUSG00000033102	ENSMUSG00000071454
ENSMUSG00000033105	ENSMUSG00000071477
ENSMUSG00000033106	ENSMUSG00000071528
ENSMUSG00000033111	ENSMUSG00000071533
ENSMUSG00000033124	ENSMUSG00000071644
ENSMUSG00000033126	ENSMUSG00000071645
ENSMUSG00000033128	ENSMUSG00000071646
ENSMUSG00000033147	ENSMUSG00000071649
ENSMUSG00000033149	ENSMUSG00000071650
ENSMUSG00000033152	ENSMUSG00000071652
ENSMUSG00000033157	ENSMUSG00000071655
ENSMUSG00000033159	ENSMUSG00000071657
ENSMUSG00000033161	ENSMUSG00000071658
ENSMUSG00000033166	ENSMUSG00000071659
ENSMUSG00000033170	ENSMUSG00000071662

ENSMUSG00000033174	ENSMUSG00000071708
ENSMUSG00000033184	ENSMUSG00000071719
ENSMUSG00000033186	ENSMUSG00000071723
ENSMUSG00000033191	ENSMUSG00000071753
ENSMUSG00000033192	ENSMUSG00000071793
ENSMUSG00000033207	ENSMUSG00000071796
ENSMUSG00000033208	ENSMUSG00000071862
ENSMUSG00000033209	ENSMUSG00000071866
ENSMUSG00000033214	ENSMUSG00000072214
ENSMUSG00000033216	ENSMUSG00000072235
ENSMUSG00000033228	ENSMUSG00000072294
ENSMUSG00000033233	ENSMUSG00000072437
ENSMUSG00000033237	ENSMUSG00000072501
ENSMUSG00000033249	ENSMUSG00000072640
ENSMUSG00000033253	ENSMUSG00000072694
ENSMUSG00000033256	ENSMUSG00000072704
ENSMUSG00000033257	ENSMUSG00000072769
ENSMUSG00000033272	ENSMUSG00000072772
ENSMUSG00000033276	ENSMUSG00000072825
ENSMUSG00000033278	ENSMUSG00000072872
ENSMUSG00000033282	ENSMUSG00000072875
ENSMUSG00000033285	ENSMUSG00000072946
ENSMUSG00000033287	ENSMUSG00000072964
ENSMUSG00000033294	ENSMUSG00000072966
ENSMUSG00000033295	ENSMUSG00000073079
ENSMUSG00000033306	ENSMUSG00000073096
ENSMUSG00000033307	ENSMUSG00000073139
ENSMUSG00000033308	ENSMUSG00000073295
ENSMUSG00000033316	ENSMUSG00000073411
ENSMUSG00000033319	ENSMUSG00000073436
ENSMUSG00000033323	ENSMUSG00000073481
ENSMUSG00000033326	ENSMUSG00000073514
ENSMUSG00000033327	ENSMUSG00000073557
ENSMUSG00000033335	ENSMUSG00000073563
ENSMUSG00000033342	ENSMUSG00000073616
ENSMUSG00000033350	ENSMUSG00000073639
ENSMUSG00000033352	ENSMUSG00000073643
ENSMUSG00000033361	ENSMUSG00000073664
ENSMUSG00000033364	ENSMUSG00000073678
ENSMUSG00000033365	ENSMUSG00000073680
ENSMUSG00000033373	ENSMUSG00000073702
ENSMUSG00000033377	ENSMUSG00000073725
ENSMUSG00000033379	ENSMUSG00000073755
ENSMUSG00000033382	ENSMUSG00000073838
ENSMUSG00000033389	ENSMUSG00000073888
ENSMUSG00000033392	ENSMUSG00000074024
ENSMUSG00000033396	ENSMUSG00000074030
ENSMUSG00000033400	ENSMUSG00000074093
ENSMUSG00000033411	ENSMUSG00000074102
ENSMUSG00000033416	ENSMUSG00000074129
ENSMUSG00000033417	ENSMUSG00000074165
ENSMUSG00000033419	ENSMUSG00000074212
ENSMUSG00000033420	ENSMUSG00000074238

ENSMUSG00000033423	ENSMUSG00000074247
ENSMUSG00000033429	ENSMUSG00000074305
ENSMUSG00000033430	ENSMUSG00000074405
ENSMUSG00000033434	ENSMUSG00000074415
ENSMUSG00000033436	ENSMUSG00000074457
ENSMUSG00000033439	ENSMUSG00000074505
ENSMUSG00000033444	ENSMUSG00000074582
ENSMUSG00000033453	ENSMUSG00000074637
ENSMUSG00000033454	ENSMUSG00000074643
ENSMUSG00000033458	ENSMUSG00000074649
ENSMUSG00000033460	ENSMUSG00000074656
ENSMUSG00000033467	ENSMUSG00000074657
ENSMUSG00000033475	ENSMUSG00000074671
ENSMUSG00000033478	ENSMUSG00000074698
ENSMUSG00000033487	ENSMUSG00000074736
ENSMUSG00000033488	ENSMUSG00000074746
ENSMUSG00000033499	ENSMUSG00000074748
ENSMUSG00000033502	ENSMUSG00000074781
ENSMUSG00000033510	ENSMUSG00000074785
ENSMUSG00000033526	ENSMUSG00000074794
ENSMUSG00000033530	ENSMUSG00000074797
ENSMUSG00000033540	ENSMUSG00000074884
ENSMUSG00000033543	ENSMUSG00000074886
ENSMUSG00000033545	ENSMUSG00000074892
ENSMUSG00000033554	ENSMUSG00000074909
ENSMUSG00000033557	ENSMUSG00000074918
ENSMUSG00000033565	ENSMUSG00000074923
ENSMUSG00000033569	ENSMUSG00000074925
ENSMUSG00000033577	ENSMUSG00000074968
ENSMUSG00000033578	ENSMUSG00000075012
ENSMUSG00000033579	ENSMUSG00000075028
ENSMUSG00000033585	ENSMUSG00000075054
ENSMUSG00000033594	ENSMUSG00000075232
ENSMUSG00000033595	ENSMUSG00000075254
ENSMUSG00000033596	ENSMUSG00000075318
ENSMUSG00000033597	ENSMUSG00000075376
ENSMUSG00000033610	ENSMUSG00000075415
ENSMUSG00000033615	ENSMUSG00000075467
ENSMUSG00000033618	ENSMUSG00000075470
ENSMUSG00000033623	ENSMUSG00000075478
ENSMUSG00000033624	ENSMUSG00000075528
ENSMUSG00000033628	ENSMUSG00000075590
ENSMUSG00000033629	ENSMUSG00000075595
ENSMUSG00000033632	ENSMUSG00000075700
ENSMUSG00000033653	ENSMUSG00000075702
ENSMUSG00000033658	ENSMUSG00000075703
ENSMUSG00000033669	ENSMUSG00000075706
ENSMUSG00000033671	ENSMUSG00000076432
ENSMUSG00000033676	ENSMUSG00000076439
ENSMUSG00000033684	ENSMUSG00000076441
ENSMUSG00000033685	ENSMUSG00000077450
ENSMUSG00000033697	ENSMUSG00000077714
ENSMUSG00000033701	ENSMUSG00000078137

ENSMUSG00000033703	ENSMUSG00000078161
ENSMUSG00000033705	ENSMUSG00000078234
ENSMUSG00000033706	ENSMUSG00000078307
ENSMUSG00000033707	ENSMUSG00000078429
ENSMUSG00000033712	ENSMUSG00000078440
ENSMUSG00000033713	ENSMUSG00000078485
ENSMUSG00000033717	ENSMUSG00000078515
ENSMUSG00000033720	ENSMUSG00000078517
ENSMUSG00000033722	ENSMUSG00000078566
ENSMUSG00000033728	ENSMUSG00000078578
ENSMUSG00000033730	ENSMUSG00000078584
ENSMUSG00000033732	ENSMUSG00000078591
ENSMUSG00000033735	ENSMUSG00000078622
ENSMUSG00000033739	ENSMUSG00000078636
ENSMUSG00000033740	ENSMUSG00000078652
ENSMUSG00000033751	ENSMUSG00000078671
ENSMUSG00000033760	ENSMUSG00000078676
ENSMUSG00000033763	ENSMUSG00000078716
ENSMUSG00000033767	ENSMUSG00000078794
ENSMUSG00000033768	ENSMUSG00000078812
ENSMUSG00000033769	ENSMUSG00000078816
ENSMUSG00000033773	ENSMUSG00000078851
ENSMUSG00000033781	ENSMUSG00000078899
ENSMUSG00000033790	ENSMUSG00000078903
ENSMUSG00000033792	ENSMUSG00000078908
ENSMUSG00000033793	ENSMUSG00000078923
ENSMUSG00000033799	ENSMUSG00000078941
ENSMUSG00000033805	ENSMUSG00000079003
ENSMUSG00000033808	ENSMUSG00000079020
ENSMUSG00000033809	ENSMUSG00000079037
ENSMUSG00000033813	ENSMUSG00000079055
ENSMUSG00000033819	ENSMUSG00000079056
ENSMUSG00000033845	ENSMUSG00000079108
ENSMUSG00000033849	ENSMUSG00000079157
ENSMUSG00000033854	ENSMUSG00000079179
ENSMUSG00000033855	ENSMUSG00000079184
ENSMUSG00000033857	ENSMUSG00000079215
ENSMUSG00000033862	ENSMUSG00000079426
ENSMUSG00000033863	ENSMUSG00000079435
ENSMUSG00000033871	ENSMUSG00000079470
ENSMUSG00000033880	ENSMUSG00000079477
ENSMUSG00000033883	ENSMUSG00000079481
ENSMUSG00000033885	ENSMUSG00000079509
ENSMUSG00000033900	ENSMUSG00000079523
ENSMUSG00000033902	ENSMUSG00000079557
ENSMUSG00000033904	ENSMUSG00000079562
ENSMUSG00000033906	ENSMUSG00000079641
ENSMUSG00000033909	ENSMUSG00000079658
ENSMUSG00000033910	ENSMUSG00000080242
ENSMUSG00000033916	ENSMUSG00000080465
ENSMUSG00000033917	ENSMUSG00000081534
ENSMUSG00000033918	ENSMUSG00000082229
ENSMUSG00000033931	ENSMUSG00000083282

ENSMUSG00000033933	ENSMUSG00000083327
ENSMUSG00000033938	ENSMUSG00000083773
ENSMUSG00000033940	ENSMUSG00000084349
ENSMUSG00000033943	ENSMUSG00000084708
ENSMUSG00000033948	ENSMUSG00000084786
ENSMUSG00000033953	ENSMUSG00000084842
ENSMUSG00000033955	ENSMUSG00000084845
ENSMUSG00000033960	ENSMUSG00000084883
ENSMUSG00000033961	ENSMUSG00000085133
ENSMUSG00000033964	ENSMUSG00000085396
ENSMUSG00000033965	ENSMUSG00000085438
ENSMUSG00000033966	ENSMUSG00000086040
ENSMUSG00000033970	ENSMUSG00000086119
ENSMUSG00000033972	ENSMUSG00000086370
ENSMUSG00000033981	ENSMUSG00000086496
ENSMUSG00000033983	ENSMUSG00000086968
ENSMUSG00000033991	ENSMUSG00000087006
ENSMUSG00000033998	ENSMUSG00000087141
ENSMUSG00000034000	ENSMUSG00000087370
ENSMUSG00000034006	ENSMUSG00000087408
ENSMUSG00000034007	ENSMUSG00000087620
ENSMUSG00000034021	ENSMUSG00000087679
ENSMUSG00000034022	ENSMUSG00000087968
ENSMUSG00000034024	ENSMUSG00000088025
ENSMUSG00000034032	ENSMUSG00000088088
ENSMUSG00000034037	ENSMUSG00000088185
ENSMUSG00000034040	ENSMUSG00000088789
ENSMUSG00000034042	ENSMUSG00000089281
ENSMUSG00000034055	ENSMUSG00000089417
ENSMUSG00000034059	ENSMUSG00000089617
ENSMUSG00000034064	ENSMUSG00000089682
ENSMUSG00000034066	ENSMUSG00000089704
ENSMUSG00000034071	ENSMUSG00000089715
ENSMUSG00000034075	ENSMUSG00000089774
ENSMUSG00000034083	ENSMUSG00000089809
ENSMUSG00000034088	ENSMUSG00000089824
ENSMUSG00000034098	ENSMUSG00000089862
ENSMUSG00000034101	ENSMUSG00000089872
ENSMUSG00000034105	ENSMUSG00000089911
ENSMUSG00000034108	ENSMUSG00000089945
ENSMUSG00000034109	ENSMUSG00000090063
ENSMUSG00000034110	ENSMUSG00000090071
ENSMUSG00000034111	ENSMUSG00000090083
ENSMUSG00000034118	ENSMUSG00000090100
ENSMUSG00000034120	ENSMUSG00000090112
ENSMUSG00000034121	ENSMUSG00000090125
ENSMUSG00000034126	ENSMUSG00000090137
ENSMUSG00000034135	ENSMUSG00000090223
ENSMUSG00000034145	ENSMUSG00000090247
ENSMUSG00000034152	ENSMUSG00000090291
ENSMUSG00000034154	ENSMUSG00000090733
ENSMUSG00000034156	ENSMUSG00000090841
ENSMUSG00000034157	ENSMUSG00000090862

ENSMUSG00000034158	ENSMUSG00000090935
ENSMUSG00000034160	ENSMUSG00000091228
ENSMUSG00000034163	ENSMUSG00000091264
ENSMUSG00000034165	ENSMUSG00000091337
ENSMUSG00000034168	ENSMUSG00000091405
ENSMUSG00000034171	ENSMUSG00000091537
ENSMUSG00000034173	ENSMUSG00000091811
ENSMUSG00000034175	ENSMUSG00000091896
ENSMUSG00000034187	ENSMUSG00000091957
ENSMUSG00000034189	ENSMUSG00000092035
ENSMUSG00000034190	ENSMUSG00000092274
ENSMUSG00000034192	ENSMUSG00000092329
ENSMUSG00000034194	ENSMUSG00000092341
ENSMUSG00000034201	ENSMUSG00000092837
ENSMUSG00000034203	ENSMUSG00000093674
ENSMUSG00000034210	ENSMUSG00000093752
ENSMUSG00000034211	ENSMUSG00000093815
ENSMUSG00000034216	ENSMUSG00000093843
ENSMUSG00000034218	ENSMUSG00000093904
ENSMUSG00000034220	ENSMUSG00000093930
ENSMUSG00000034226	ENSMUSG00000093989
ENSMUSG00000034227	ENSMUSG00000094050
ENSMUSG00000034235	ENSMUSG00000094377
ENSMUSG00000034243	ENSMUSG00000094410
ENSMUSG00000034245	ENSMUSG00000094483
ENSMUSG00000034247	ENSMUSG00000094626
ENSMUSG00000034248	ENSMUSG00000094655
ENSMUSG00000034252	ENSMUSG00000094826
ENSMUSG00000034254	ENSMUSG00000094910
ENSMUSG00000034255	ENSMUSG00000094936
ENSMUSG00000034259	ENSMUSG00000095041
ENSMUSG00000034263	ENSMUSG00000095098
ENSMUSG00000034265	ENSMUSG00000095253
ENSMUSG00000034269	ENSMUSG00000095463
ENSMUSG00000034271	ENSMUSG00000095526
ENSMUSG00000034274	ENSMUSG00000095567
ENSMUSG00000034275	ENSMUSG00000095969
ENSMUSG00000034278	ENSMUSG00000096014
ENSMUSG00000034282	ENSMUSG00000096054
ENSMUSG00000034285	ENSMUSG00000096146
ENSMUSG00000034290	ENSMUSG00000096188
ENSMUSG00000034292	ENSMUSG00000096205
ENSMUSG00000034295	ENSMUSG00000096206
ENSMUSG00000034297	ENSMUSG00000096210
ENSMUSG00000034300	ENSMUSG00000096214
ENSMUSG00000034308	ENSMUSG00000096243
ENSMUSG00000034310	ENSMUSG00000096472
ENSMUSG00000034312	ENSMUSG00000096659
ENSMUSG00000034320	ENSMUSG00000096768
ENSMUSG00000034321	ENSMUSG00000096780
ENSMUSG00000034324	ENSMUSG00000096847
ENSMUSG00000034327	ENSMUSG00000096910
ENSMUSG00000034333	ENSMUSG00000096929

ENSMUSG00000034336	ENSMUSG00000097391
ENSMUSG00000034341	ENSMUSG00000097451
ENSMUSG00000034342	ENSMUSG00000097545
ENSMUSG00000034343	ENSMUSG00000097736
ENSMUSG00000034345	ENSMUSG00000097767
ENSMUSG00000034353	ENSMUSG00000097971
ENSMUSG00000034354	ENSMUSG00000097993
ENSMUSG00000034361	ENSMUSG00000098178
ENSMUSG00000034371	ENSMUSG00000098274
ENSMUSG00000034377	ENSMUSG00000098557
ENSMUSG00000034390	ENSMUSG00000099021
ENSMUSG00000034402	ENSMUSG00000099250
ENSMUSG00000034403	ENSMUSG00000099696
ENSMUSG00000034412	ENSMUSG00000100252
ENSMUSG00000034413	ENSMUSG00000100801
ENSMUSG00000034424	ENSMUSG00000100826
ENSMUSG00000034429	ENSMUSG00000100862
ENSMUSG00000034430	ENSMUSG00000101111
ENSMUSG00000034432	ENSMUSG00000101609
ENSMUSG00000034442	ENSMUSG00000102070
ENSMUSG00000034445	ENSMUSG00000102222
ENSMUSG00000034453	ENSMUSG00000102252
ENSMUSG00000034462	ENSMUSG00000102428
ENSMUSG00000034463	ENSMUSG00000102440
ENSMUSG00000034467	ENSMUSG00000102543
ENSMUSG00000034471	ENSMUSG00000102692
ENSMUSG00000034472	ENSMUSG00000102697
ENSMUSG00000034473	ENSMUSG00000102742
ENSMUSG00000034480	ENSMUSG00000102869
ENSMUSG00000034484	ENSMUSG00000102918
ENSMUSG00000034485	ENSMUSG00000102976
ENSMUSG00000034488	ENSMUSG00000103034
ENSMUSG00000034501	ENSMUSG00000103088
ENSMUSG00000034509	ENSMUSG00000104063
ENSMUSG00000034518	ENSMUSG00000104222
ENSMUSG00000034522	ENSMUSG00000104597
ENSMUSG00000034525	ENSMUSG00000105025
ENSMUSG00000034532	ENSMUSG00000105867
ENSMUSG00000034538	ENSMUSG00000106379
ENSMUSG00000034543	ENSMUSG00000106847
ENSMUSG00000034544	ENSMUSG00000106864
ENSMUSG00000034551	ENSMUSG00000107068
ENSMUSG00000034557	ENSMUSG00000107877
ENSMUSG00000034560	ENSMUSG00000108348
ENSMUSG00000034563	ENSMUSG00000109125
ENSMUSG00000034566	ENSMUSG00000109324
ENSMUSG00000034570	ENSMUSG00000109336
ENSMUSG00000034573	ENSMUSG00000109536
ENSMUSG00000034574	ENSMUSG00000110185
ENSMUSG00000034575	ENSMUSG00000110195
ENSMUSG00000034586	ENSMUSG00000110710
ENSMUSG00000034591	ENSMUSG00000111375
ENSMUSG00000034593	ENSMUSG00000113949

ENSMUSG00000034595
ENSMUSG00000034601
ENSMUSG00000034602
ENSMUSG00000034610
ENSMUSG00000034612
ENSMUSG00000034613
ENSMUSG00000034614
ENSMUSG00000034616
ENSMUSG00000034617
ENSMUSG00000034620
ENSMUSG00000034621
ENSMUSG00000034636
ENSMUSG00000034640
ENSMUSG00000034647
ENSMUSG00000034648
ENSMUSG00000034653
ENSMUSG00000034656
ENSMUSG00000034659
ENSMUSG00000034663
ENSMUSG00000034667
ENSMUSG00000034673
ENSMUSG00000034674
ENSMUSG00000034675
ENSMUSG00000034681
ENSMUSG00000034684
ENSMUSG00000034685
ENSMUSG00000034686
ENSMUSG00000034687
ENSMUSG00000034706
ENSMUSG00000034707
ENSMUSG00000034708
ENSMUSG00000034709
ENSMUSG00000034714
ENSMUSG00000034723
ENSMUSG00000034724
ENSMUSG00000034729
ENSMUSG00000034730
ENSMUSG00000034731
ENSMUSG00000034744
ENSMUSG00000034748
ENSMUSG00000034751
ENSMUSG00000034755
ENSMUSG00000034757
ENSMUSG00000034761
ENSMUSG00000034765
ENSMUSG00000034771
ENSMUSG00000034780
ENSMUSG00000034781
ENSMUSG00000034789
ENSMUSG00000034793
ENSMUSG00000034796
ENSMUSG00000034799
ENSMUSG00000034800
ENSMUSG00000114797
ENSMUSG00000115420
ENSMUSG00000115783
ENSMUSG00000115987
ENSMUSG00000116048
ENSMUSG00000116165
ENSMUSG00000116275
ENSMUSG00000116594
ENSMUSG00000117286
ENSMUSG00000117310
ENSMUSG00000117679
ENSMUSG00000118401

ENSMUSG00000034801
ENSMUSG00000034807
ENSMUSG00000034813
ENSMUSG00000034818
ENSMUSG00000034820
ENSMUSG00000034825
ENSMUSG00000034826
ENSMUSG00000034832
ENSMUSG00000034839
ENSMUSG00000034845
ENSMUSG00000034848
ENSMUSG00000034850
ENSMUSG00000034853
ENSMUSG00000034854
ENSMUSG00000034858
ENSMUSG00000034863
ENSMUSG00000034867
ENSMUSG00000034868
ENSMUSG00000034875
ENSMUSG00000034880
ENSMUSG00000034889
ENSMUSG00000034891
ENSMUSG00000034892
ENSMUSG00000034893
ENSMUSG00000034898
ENSMUSG00000034902
ENSMUSG00000034903
ENSMUSG00000034908
ENSMUSG00000034910
ENSMUSG00000034912
ENSMUSG00000034926
ENSMUSG00000034928
ENSMUSG00000034930
ENSMUSG00000034931
ENSMUSG00000034932
ENSMUSG00000034936
ENSMUSG00000034940
ENSMUSG00000034949
ENSMUSG00000034951
ENSMUSG00000034957
ENSMUSG00000034958
ENSMUSG00000034973
ENSMUSG00000034974
ENSMUSG00000034981
ENSMUSG00000034993
ENSMUSG00000034994
ENSMUSG00000034997
ENSMUSG00000034998
ENSMUSG00000035007
ENSMUSG00000035011
ENSMUSG00000035024
ENSMUSG00000035027
ENSMUSG00000035045

ENSMUSG00000035047
ENSMUSG00000035048
ENSMUSG00000035049
ENSMUSG00000035051
ENSMUSG00000035062
ENSMUSG00000035064
ENSMUSG00000035067
ENSMUSG00000035078
ENSMUSG00000035086
ENSMUSG00000035093
ENSMUSG00000035095
ENSMUSG00000035104
ENSMUSG00000035107
ENSMUSG00000035109
ENSMUSG00000035125
ENSMUSG00000035126
ENSMUSG00000035131
ENSMUSG00000035133
ENSMUSG00000035139
ENSMUSG00000035142
ENSMUSG00000035150
ENSMUSG00000035151
ENSMUSG00000035152
ENSMUSG00000035161
ENSMUSG00000035164
ENSMUSG00000035168
ENSMUSG00000035171
ENSMUSG00000035172
ENSMUSG00000035173
ENSMUSG00000035179
ENSMUSG00000035181
ENSMUSG00000035184
ENSMUSG00000035189
ENSMUSG00000035198
ENSMUSG00000035199
ENSMUSG00000035202
ENSMUSG00000035203
ENSMUSG00000035206
ENSMUSG00000035212
ENSMUSG00000035215
ENSMUSG00000035226
ENSMUSG00000035227
ENSMUSG00000035228
ENSMUSG00000035232
ENSMUSG00000035235
ENSMUSG00000035236
ENSMUSG00000035237
ENSMUSG00000035242
ENSMUSG00000035245
ENSMUSG00000035246
ENSMUSG00000035247
ENSMUSG00000035248
ENSMUSG00000035266

ENSMUSG00000035268
ENSMUSG00000035274
ENSMUSG00000035275
ENSMUSG00000035277
ENSMUSG00000035278
ENSMUSG00000035283
ENSMUSG00000035284
ENSMUSG00000035285
ENSMUSG00000035293
ENSMUSG00000035297
ENSMUSG00000035299
ENSMUSG00000035310
ENSMUSG00000035311
ENSMUSG00000035314
ENSMUSG00000035325
ENSMUSG00000035329
ENSMUSG00000035342
ENSMUSG00000035354
ENSMUSG00000035355
ENSMUSG00000035357
ENSMUSG00000035367
ENSMUSG00000035372
ENSMUSG00000035376
ENSMUSG00000035378
ENSMUSG00000035382
ENSMUSG00000035390
ENSMUSG00000035392
ENSMUSG00000035397
ENSMUSG00000035399
ENSMUSG00000035401
ENSMUSG00000035403
ENSMUSG00000035407
ENSMUSG00000035413
ENSMUSG00000035431
ENSMUSG00000035437
ENSMUSG00000035439
ENSMUSG00000035441
ENSMUSG00000035443
ENSMUSG00000035469
ENSMUSG00000035476
ENSMUSG00000035478
ENSMUSG00000035486
ENSMUSG00000035495
ENSMUSG00000035504
ENSMUSG00000035505
ENSMUSG00000035509
ENSMUSG00000035517
ENSMUSG00000035521
ENSMUSG00000035529
ENSMUSG00000035530
ENSMUSG00000035539
ENSMUSG00000035545
ENSMUSG00000035547

ENSMUSG00000035551
ENSMUSG00000035559
ENSMUSG00000035561
ENSMUSG00000035566
ENSMUSG00000035569
ENSMUSG00000035572
ENSMUSG00000035575
ENSMUSG00000035576
ENSMUSG00000035578
ENSMUSG00000035585
ENSMUSG00000035596
ENSMUSG00000035597
ENSMUSG00000035601
ENSMUSG00000035606
ENSMUSG00000035614
ENSMUSG00000035615
ENSMUSG00000035620
ENSMUSG00000035621
ENSMUSG00000035623
ENSMUSG00000035629
ENSMUSG00000035632
ENSMUSG00000035637
ENSMUSG00000035640
ENSMUSG00000035642
ENSMUSG00000035649
ENSMUSG00000035653
ENSMUSG00000035666
ENSMUSG00000035671
ENSMUSG00000035673
ENSMUSG00000035674
ENSMUSG00000035681
ENSMUSG00000035686
ENSMUSG00000035696
ENSMUSG00000035713
ENSMUSG00000035722
ENSMUSG00000035725
ENSMUSG00000035726
ENSMUSG00000035735
ENSMUSG00000035754
ENSMUSG00000035757
ENSMUSG00000035762
ENSMUSG00000035764
ENSMUSG00000035765
ENSMUSG00000035769
ENSMUSG00000035770
ENSMUSG00000035772
ENSMUSG00000035773
ENSMUSG00000035776
ENSMUSG00000035781
ENSMUSG00000035783
ENSMUSG00000035790
ENSMUSG00000035798
ENSMUSG00000035805

ENSMUSG00000035824
ENSMUSG00000035828
ENSMUSG00000035829
ENSMUSG00000035835
ENSMUSG00000035840
ENSMUSG00000035845
ENSMUSG00000035847
ENSMUSG00000035849
ENSMUSG00000035851
ENSMUSG00000035860
ENSMUSG00000035863
ENSMUSG00000035864
ENSMUSG00000035868
ENSMUSG00000035877
ENSMUSG00000035878
ENSMUSG00000035885
ENSMUSG00000035890
ENSMUSG00000035891
ENSMUSG00000035898
ENSMUSG00000035900
ENSMUSG00000035901
ENSMUSG00000035910
ENSMUSG00000035914
ENSMUSG00000035919
ENSMUSG00000035933
ENSMUSG00000035934
ENSMUSG00000035936
ENSMUSG00000035941
ENSMUSG00000035944
ENSMUSG00000035949
ENSMUSG00000035953
ENSMUSG00000035954
ENSMUSG00000035958
ENSMUSG00000035960
ENSMUSG00000035964
ENSMUSG00000035967
ENSMUSG00000035969
ENSMUSG00000035984
ENSMUSG00000035992
ENSMUSG00000036002
ENSMUSG00000036006
ENSMUSG00000036009
ENSMUSG00000036019
ENSMUSG00000036023
ENSMUSG00000036026
ENSMUSG00000036036
ENSMUSG00000036040
ENSMUSG00000036046
ENSMUSG00000036052
ENSMUSG00000036053
ENSMUSG00000036054
ENSMUSG00000036057
ENSMUSG00000036061

ENSMUSG00000036062
ENSMUSG00000036067
ENSMUSG00000036073
ENSMUSG00000036078
ENSMUSG00000036086
ENSMUSG00000036087
ENSMUSG00000036093
ENSMUSG00000036095
ENSMUSG00000036097
ENSMUSG00000036098
ENSMUSG00000036099
ENSMUSG00000036103
ENSMUSG00000036104
ENSMUSG00000036106
ENSMUSG00000036111
ENSMUSG00000036112
ENSMUSG00000036114
ENSMUSG00000036120
ENSMUSG00000036138
ENSMUSG00000036155
ENSMUSG00000036158
ENSMUSG00000036160
ENSMUSG00000036167
ENSMUSG00000036180
ENSMUSG00000036181
ENSMUSG00000036186
ENSMUSG00000036188
ENSMUSG00000036192
ENSMUSG00000036197
ENSMUSG00000036198
ENSMUSG00000036199
ENSMUSG00000036202
ENSMUSG00000036206
ENSMUSG00000036218
ENSMUSG00000036225
ENSMUSG00000036241
ENSMUSG00000036242
ENSMUSG00000036246
ENSMUSG00000036249
ENSMUSG00000036256
ENSMUSG00000036257
ENSMUSG00000036264
ENSMUSG00000036270
ENSMUSG00000036273
ENSMUSG00000036275
ENSMUSG00000036278
ENSMUSG00000036281
ENSMUSG00000036282
ENSMUSG00000036285
ENSMUSG00000036291
ENSMUSG00000036295
ENSMUSG00000036298
ENSMUSG00000036299

ENSMUSG00000036304
ENSMUSG00000036306
ENSMUSG00000036309
ENSMUSG00000036315
ENSMUSG00000036323
ENSMUSG00000036327
ENSMUSG00000036333
ENSMUSG00000036334
ENSMUSG00000036339
ENSMUSG00000036352
ENSMUSG00000036353
ENSMUSG00000036356
ENSMUSG00000036357
ENSMUSG00000036368
ENSMUSG00000036371
ENSMUSG00000036372
ENSMUSG00000036376
ENSMUSG00000036377
ENSMUSG00000036391
ENSMUSG00000036398
ENSMUSG00000036402
ENSMUSG00000036403
ENSMUSG00000036411
ENSMUSG00000036422
ENSMUSG00000036427
ENSMUSG00000036430
ENSMUSG00000036432
ENSMUSG00000036435
ENSMUSG00000036437
ENSMUSG00000036438
ENSMUSG00000036442
ENSMUSG00000036450
ENSMUSG00000036452
ENSMUSG00000036459
ENSMUSG00000036466
ENSMUSG00000036469
ENSMUSG00000036473
ENSMUSG00000036478
ENSMUSG00000036499
ENSMUSG00000036501
ENSMUSG00000036502
ENSMUSG00000036503
ENSMUSG00000036504
ENSMUSG00000036510
ENSMUSG00000036513
ENSMUSG00000036528
ENSMUSG00000036529
ENSMUSG00000036533
ENSMUSG00000036534
ENSMUSG00000036537
ENSMUSG00000036545
ENSMUSG00000036550
ENSMUSG00000036552

ENSMUSG00000036555
ENSMUSG00000036560
ENSMUSG00000036561
ENSMUSG00000036564
ENSMUSG00000036565
ENSMUSG00000036568
ENSMUSG00000036570
ENSMUSG00000036572
ENSMUSG00000036578
ENSMUSG00000036580
ENSMUSG00000036585
ENSMUSG00000036591
ENSMUSG00000036598
ENSMUSG00000036599
ENSMUSG00000036606
ENSMUSG00000036611
ENSMUSG00000036613
ENSMUSG00000036615
ENSMUSG00000036617
ENSMUSG00000036620
ENSMUSG00000036622
ENSMUSG00000036632
ENSMUSG00000036634
ENSMUSG00000036636
ENSMUSG00000036639
ENSMUSG00000036641
ENSMUSG00000036644
ENSMUSG00000036646
ENSMUSG00000036661
ENSMUSG00000036667
ENSMUSG00000036672
ENSMUSG00000036676
ENSMUSG00000036678
ENSMUSG00000036686
ENSMUSG00000036693
ENSMUSG00000036698
ENSMUSG00000036699
ENSMUSG00000036707
ENSMUSG00000036712
ENSMUSG00000036721
ENSMUSG00000036733
ENSMUSG00000036737
ENSMUSG00000036745
ENSMUSG00000036748
ENSMUSG00000036751
ENSMUSG00000036752
ENSMUSG00000036760
ENSMUSG00000036764
ENSMUSG00000036766
ENSMUSG00000036769
ENSMUSG00000036775
ENSMUSG00000036777
ENSMUSG00000036779

ENSMUSG00000036782
ENSMUSG00000036790
ENSMUSG00000036792
ENSMUSG00000036800
ENSMUSG00000036810
ENSMUSG00000036815
ENSMUSG00000036817
ENSMUSG00000036819
ENSMUSG00000036820
ENSMUSG00000036822
ENSMUSG00000036825
ENSMUSG00000036833
ENSMUSG00000036834
ENSMUSG00000036835
ENSMUSG00000036840
ENSMUSG00000036845
ENSMUSG00000036850
ENSMUSG00000036856
ENSMUSG00000036860
ENSMUSG00000036862
ENSMUSG00000036863
ENSMUSG00000036873
ENSMUSG00000036879
ENSMUSG00000036880
ENSMUSG00000036882
ENSMUSG00000036885
ENSMUSG00000036887
ENSMUSG00000036890
ENSMUSG00000036893
ENSMUSG00000036894
ENSMUSG00000036896
ENSMUSG00000036898
ENSMUSG00000036902
ENSMUSG00000036904
ENSMUSG00000036905
ENSMUSG00000036908
ENSMUSG00000036913
ENSMUSG00000036916
ENSMUSG00000036918
ENSMUSG00000036923
ENSMUSG00000036932
ENSMUSG00000036940
ENSMUSG00000036941
ENSMUSG00000036943
ENSMUSG00000036948
ENSMUSG00000036949
ENSMUSG00000036955
ENSMUSG00000036957
ENSMUSG00000036959
ENSMUSG00000036964
ENSMUSG00000036966
ENSMUSG00000036968
ENSMUSG00000036972

ENSMUSG00000036975
ENSMUSG00000036980
ENSMUSG00000036985
ENSMUSG00000036986
ENSMUSG00000036989
ENSMUSG00000036990
ENSMUSG00000037001
ENSMUSG00000037003
ENSMUSG00000037007
ENSMUSG00000037010
ENSMUSG00000037012
ENSMUSG00000037013
ENSMUSG00000037014
ENSMUSG00000037016
ENSMUSG00000037017
ENSMUSG00000037022
ENSMUSG00000037029
ENSMUSG00000037031
ENSMUSG00000037032
ENSMUSG00000037035
ENSMUSG00000037049
ENSMUSG00000037058
ENSMUSG00000037060
ENSMUSG00000037062
ENSMUSG00000037070
ENSMUSG00000037071
ENSMUSG00000037072
ENSMUSG00000037075
ENSMUSG00000037085
ENSMUSG00000037089
ENSMUSG00000037098
ENSMUSG00000037103
ENSMUSG00000037104
ENSMUSG00000037108
ENSMUSG00000037110
ENSMUSG00000037111
ENSMUSG00000037112
ENSMUSG00000037119
ENSMUSG00000037126
ENSMUSG00000037134
ENSMUSG00000037138
ENSMUSG00000037143
ENSMUSG00000037148
ENSMUSG00000037149
ENSMUSG00000037151
ENSMUSG00000037152
ENSMUSG00000037166
ENSMUSG00000037169
ENSMUSG00000037172
ENSMUSG00000037174
ENSMUSG00000037190
ENSMUSG00000037196
ENSMUSG00000037197

ENSMUSG00000037204
ENSMUSG00000037206
ENSMUSG00000037210
ENSMUSG00000037211
ENSMUSG00000037214
ENSMUSG00000037217
ENSMUSG00000037221
ENSMUSG00000037224
ENSMUSG00000037225
ENSMUSG00000037234
ENSMUSG00000037235
ENSMUSG00000037236
ENSMUSG00000037239
ENSMUSG00000037242
ENSMUSG00000037243
ENSMUSG00000037251
ENSMUSG00000037253
ENSMUSG00000037257
ENSMUSG00000037259
ENSMUSG00000037260
ENSMUSG00000037262
ENSMUSG00000037266
ENSMUSG00000037270
ENSMUSG00000037275
ENSMUSG00000037278
ENSMUSG00000037280
ENSMUSG00000037286
ENSMUSG00000037287
ENSMUSG00000037295
ENSMUSG00000037296
ENSMUSG00000037300
ENSMUSG00000037306
ENSMUSG00000037313
ENSMUSG00000037315
ENSMUSG00000037316
ENSMUSG00000037325
ENSMUSG00000037326
ENSMUSG00000037331
ENSMUSG00000037339
ENSMUSG00000037341
ENSMUSG00000037343
ENSMUSG00000037344
ENSMUSG00000037348
ENSMUSG00000037351
ENSMUSG00000037353
ENSMUSG00000037355
ENSMUSG00000037361
ENSMUSG00000037363
ENSMUSG00000037364
ENSMUSG00000037369
ENSMUSG00000037370
ENSMUSG00000037373
ENSMUSG00000037376

ENSMUSG00000037386
ENSMUSG00000037395
ENSMUSG00000037400
ENSMUSG00000037408
ENSMUSG00000037410
ENSMUSG00000037415
ENSMUSG00000037416
ENSMUSG00000037419
ENSMUSG00000037426
ENSMUSG00000037428
ENSMUSG00000037434
ENSMUSG00000037443
ENSMUSG00000037447
ENSMUSG00000037455
ENSMUSG00000037458
ENSMUSG00000037461
ENSMUSG00000037465
ENSMUSG00000037470
ENSMUSG00000037475
ENSMUSG00000037486
ENSMUSG00000037487
ENSMUSG00000037490
ENSMUSG00000037492
ENSMUSG00000037493
ENSMUSG00000037499
ENSMUSG00000037503
ENSMUSG00000037509
ENSMUSG00000037514
ENSMUSG00000037519
ENSMUSG00000037523
ENSMUSG00000037525
ENSMUSG00000037526
ENSMUSG00000037531
ENSMUSG00000037533
ENSMUSG00000037536
ENSMUSG00000037541
ENSMUSG00000037552
ENSMUSG00000037553
ENSMUSG00000037563
ENSMUSG00000037570
ENSMUSG00000037573
ENSMUSG00000037579
ENSMUSG00000037594
ENSMUSG00000037601
ENSMUSG00000037605
ENSMUSG00000037606
ENSMUSG00000037608
ENSMUSG00000037617
ENSMUSG00000037622
ENSMUSG00000037624
ENSMUSG00000037625
ENSMUSG00000037627
ENSMUSG00000037640

ENSMUSG00000037643
ENSMUSG00000037646
ENSMUSG00000037649
ENSMUSG00000037652
ENSMUSG00000037653
ENSMUSG00000037656
ENSMUSG00000037669
ENSMUSG00000037674
ENSMUSG00000037679
ENSMUSG00000037681
ENSMUSG00000037683
ENSMUSG00000037685
ENSMUSG00000037692
ENSMUSG00000037697
ENSMUSG00000037703
ENSMUSG00000037706
ENSMUSG00000037709
ENSMUSG00000037710
ENSMUSG00000037712
ENSMUSG00000037716
ENSMUSG00000037720
ENSMUSG00000037730
ENSMUSG00000037735
ENSMUSG00000037736
ENSMUSG00000037740
ENSMUSG00000037742
ENSMUSG00000037747
ENSMUSG00000037750
ENSMUSG00000037752
ENSMUSG00000037754
ENSMUSG00000037761
ENSMUSG00000037771
ENSMUSG00000037772
ENSMUSG00000037773
ENSMUSG00000037784
ENSMUSG00000037787
ENSMUSG00000037788
ENSMUSG00000037791
ENSMUSG00000037795
ENSMUSG00000037805
ENSMUSG00000037808
ENSMUSG00000037813
ENSMUSG00000037815
ENSMUSG00000037816
ENSMUSG00000037818
ENSMUSG00000037820
ENSMUSG00000037822
ENSMUSG00000037824
ENSMUSG00000037826
ENSMUSG00000037843
ENSMUSG00000037845
ENSMUSG00000037851
ENSMUSG00000037852

ENSMUSG00000037855
ENSMUSG00000037857
ENSMUSG00000037872
ENSMUSG00000037876
ENSMUSG00000037885
ENSMUSG00000037887
ENSMUSG00000037890
ENSMUSG00000037892
ENSMUSG00000037894
ENSMUSG00000037896
ENSMUSG00000037902
ENSMUSG00000037904
ENSMUSG00000037905
ENSMUSG00000037907
ENSMUSG00000037916
ENSMUSG00000037926
ENSMUSG00000037933
ENSMUSG00000037935
ENSMUSG00000037936
ENSMUSG00000037938
ENSMUSG00000037940
ENSMUSG00000037946
ENSMUSG00000037949
ENSMUSG00000037957
ENSMUSG00000037958
ENSMUSG00000037965
ENSMUSG00000037966
ENSMUSG00000037971
ENSMUSG00000037972
ENSMUSG00000037977
ENSMUSG00000037979
ENSMUSG00000037989
ENSMUSG00000037990
ENSMUSG00000037992
ENSMUSG00000037993
ENSMUSG00000037995
ENSMUSG00000037996
ENSMUSG00000037997
ENSMUSG00000037999
ENSMUSG00000038000
ENSMUSG00000038002
ENSMUSG00000038005
ENSMUSG00000038007
ENSMUSG00000038011
ENSMUSG00000038013
ENSMUSG00000038014
ENSMUSG00000038020
ENSMUSG00000038022
ENSMUSG00000038023
ENSMUSG00000038024
ENSMUSG00000038025
ENSMUSG00000038026
ENSMUSG00000038028

ENSMUSG00000038034
ENSMUSG00000038039
ENSMUSG00000038042
ENSMUSG00000038046
ENSMUSG00000038047
ENSMUSG00000038048
ENSMUSG00000038055
ENSMUSG00000038056
ENSMUSG00000038058
ENSMUSG00000038060
ENSMUSG00000038065
ENSMUSG00000038068
ENSMUSG00000038069
ENSMUSG00000038070
ENSMUSG00000038072
ENSMUSG00000038074
ENSMUSG00000038077
ENSMUSG00000038079
ENSMUSG00000038080
ENSMUSG00000038084
ENSMUSG00000038094
ENSMUSG00000038095
ENSMUSG00000038102
ENSMUSG00000038112
ENSMUSG00000038115
ENSMUSG00000038116
ENSMUSG00000038119
ENSMUSG00000038121
ENSMUSG00000038122
ENSMUSG00000038126
ENSMUSG00000038127
ENSMUSG00000038128
ENSMUSG00000038132
ENSMUSG00000038141
ENSMUSG00000038143
ENSMUSG00000038145
ENSMUSG00000038146
ENSMUSG00000038150
ENSMUSG00000038156
ENSMUSG00000038160
ENSMUSG00000038170
ENSMUSG00000038172
ENSMUSG00000038174
ENSMUSG00000038175
ENSMUSG00000038178
ENSMUSG00000038181
ENSMUSG00000038187
ENSMUSG00000038205
ENSMUSG00000038206
ENSMUSG00000038208
ENSMUSG00000038212
ENSMUSG00000038213
ENSMUSG00000038214

ENSMUSG00000038215
ENSMUSG00000038233
ENSMUSG00000038240
ENSMUSG00000038241
ENSMUSG00000038244
ENSMUSG00000038248
ENSMUSG00000038250
ENSMUSG00000038252
ENSMUSG00000038256
ENSMUSG00000038257
ENSMUSG00000038260
ENSMUSG00000038264
ENSMUSG00000038267
ENSMUSG00000038268
ENSMUSG00000038271
ENSMUSG00000038274
ENSMUSG00000038279
ENSMUSG00000038280
ENSMUSG00000038286
ENSMUSG00000038290
ENSMUSG00000038291
ENSMUSG00000038295
ENSMUSG00000038296
ENSMUSG00000038299
ENSMUSG00000038301
ENSMUSG00000038302
ENSMUSG00000038305
ENSMUSG00000038312
ENSMUSG00000038319
ENSMUSG00000038323
ENSMUSG00000038324
ENSMUSG00000038332
ENSMUSG00000038335
ENSMUSG00000038342
ENSMUSG00000038344
ENSMUSG00000038346
ENSMUSG00000038349
ENSMUSG00000038351
ENSMUSG00000038354
ENSMUSG00000038365
ENSMUSG00000038366
ENSMUSG00000038368
ENSMUSG00000038369
ENSMUSG00000038370
ENSMUSG00000038371
ENSMUSG00000038372
ENSMUSG00000038374
ENSMUSG00000038375
ENSMUSG00000038383
ENSMUSG00000038384
ENSMUSG00000038387
ENSMUSG00000038388
ENSMUSG00000038390

ENSMUSG00000038393
ENSMUSG00000038398
ENSMUSG00000038400
ENSMUSG00000038406
ENSMUSG00000038412
ENSMUSG00000038416
ENSMUSG00000038417
ENSMUSG00000038418
ENSMUSG00000038422
ENSMUSG00000038425
ENSMUSG00000038429
ENSMUSG00000038437
ENSMUSG00000038446
ENSMUSG00000038453
ENSMUSG00000038456
ENSMUSG00000038459
ENSMUSG00000038462
ENSMUSG00000038467
ENSMUSG00000038473
ENSMUSG00000038481
ENSMUSG00000038482
ENSMUSG00000038485
ENSMUSG00000038486
ENSMUSG00000038489
ENSMUSG00000038495
ENSMUSG00000038497
ENSMUSG00000038500
ENSMUSG00000038502
ENSMUSG00000038503
ENSMUSG00000038506
ENSMUSG00000038507
ENSMUSG00000038510
ENSMUSG00000038515
ENSMUSG00000038517
ENSMUSG00000038518
ENSMUSG00000038520
ENSMUSG00000038524
ENSMUSG00000038525
ENSMUSG00000038526
ENSMUSG00000038530
ENSMUSG00000038533
ENSMUSG00000038534
ENSMUSG00000038535
ENSMUSG00000038538
ENSMUSG00000038539
ENSMUSG00000038542
ENSMUSG00000038544
ENSMUSG00000038545
ENSMUSG00000038546
ENSMUSG00000038550
ENSMUSG00000038552
ENSMUSG00000038555
ENSMUSG00000038563

ENSMUSG00000038564
ENSMUSG00000038569
ENSMUSG00000038576
ENSMUSG00000038578
ENSMUSG00000038582
ENSMUSG00000038587
ENSMUSG00000038593
ENSMUSG00000038594
ENSMUSG00000038602
ENSMUSG00000038604
ENSMUSG00000038605
ENSMUSG00000038607
ENSMUSG00000038608
ENSMUSG00000038611
ENSMUSG00000038612
ENSMUSG00000038615
ENSMUSG00000038619
ENSMUSG00000038623
ENSMUSG00000038628
ENSMUSG00000038630
ENSMUSG00000038633
ENSMUSG00000038637
ENSMUSG00000038642
ENSMUSG00000038644
ENSMUSG00000038646
ENSMUSG00000038648
ENSMUSG00000038650
ENSMUSG00000038658
ENSMUSG00000038664
ENSMUSG00000038665
ENSMUSG00000038668
ENSMUSG00000038671
ENSMUSG00000038677
ENSMUSG00000038679
ENSMUSG00000038683
ENSMUSG00000038685
ENSMUSG00000038690
ENSMUSG00000038695
ENSMUSG00000038696
ENSMUSG00000038697
ENSMUSG00000038702
ENSMUSG00000038705
ENSMUSG00000038708
ENSMUSG00000038712
ENSMUSG00000038717
ENSMUSG00000038718
ENSMUSG00000038722
ENSMUSG00000038729
ENSMUSG00000038733
ENSMUSG00000038736
ENSMUSG00000038738
ENSMUSG00000038740
ENSMUSG00000038742

ENSMUSG00000038759
ENSMUSG00000038760
ENSMUSG00000038762
ENSMUSG00000038764
ENSMUSG00000038766
ENSMUSG00000038773
ENSMUSG00000038774
ENSMUSG00000038776
ENSMUSG00000038777
ENSMUSG00000038780
ENSMUSG00000038784
ENSMUSG00000038797
ENSMUSG00000038803
ENSMUSG00000038805
ENSMUSG00000038806
ENSMUSG00000038807
ENSMUSG00000038812
ENSMUSG00000038816
ENSMUSG00000038822
ENSMUSG00000038827
ENSMUSG00000038828
ENSMUSG00000038831
ENSMUSG00000038836
ENSMUSG00000038838
ENSMUSG00000038844
ENSMUSG00000038845
ENSMUSG00000038848
ENSMUSG00000038855
ENSMUSG00000038860
ENSMUSG00000038861
ENSMUSG00000038866
ENSMUSG00000038871
ENSMUSG00000038872
ENSMUSG00000038876
ENSMUSG00000038879
ENSMUSG00000038880
ENSMUSG00000038884
ENSMUSG00000038886
ENSMUSG00000038888
ENSMUSG00000038893
ENSMUSG00000038894
ENSMUSG00000038895
ENSMUSG00000038900
ENSMUSG00000038902
ENSMUSG00000038909
ENSMUSG00000038910
ENSMUSG00000038914
ENSMUSG00000038916
ENSMUSG00000038930
ENSMUSG00000038936
ENSMUSG00000038949
ENSMUSG00000038954
ENSMUSG00000038957

ENSMUSG00000038965
ENSMUSG00000038967
ENSMUSG00000038970
ENSMUSG00000038975
ENSMUSG00000038976
ENSMUSG00000038982
ENSMUSG00000038984
ENSMUSG00000038990
ENSMUSG00000038991
ENSMUSG00000039000
ENSMUSG00000039001
ENSMUSG00000039004
ENSMUSG00000039007
ENSMUSG00000039016
ENSMUSG00000039018
ENSMUSG00000039031
ENSMUSG00000039033
ENSMUSG00000039037
ENSMUSG00000039041
ENSMUSG00000039043
ENSMUSG00000039046
ENSMUSG00000039047
ENSMUSG00000039048
ENSMUSG00000039050
ENSMUSG00000039057
ENSMUSG00000039058
ENSMUSG00000039059
ENSMUSG00000039065
ENSMUSG00000039067
ENSMUSG00000039068
ENSMUSG00000039069
ENSMUSG00000039081
ENSMUSG00000039086
ENSMUSG00000039087
ENSMUSG00000039089
ENSMUSG00000039096
ENSMUSG00000039100
ENSMUSG00000039105
ENSMUSG00000039106
ENSMUSG00000039108
ENSMUSG00000039110
ENSMUSG00000039115
ENSMUSG00000039117
ENSMUSG00000039126
ENSMUSG00000039128
ENSMUSG00000039130
ENSMUSG00000039137
ENSMUSG00000039145
ENSMUSG00000039148
ENSMUSG00000039154
ENSMUSG00000039156
ENSMUSG00000039157
ENSMUSG00000039158

ENSMUSG00000039159
ENSMUSG00000039164
ENSMUSG00000039166
ENSMUSG00000039167
ENSMUSG00000039176
ENSMUSG00000039178
ENSMUSG00000039179
ENSMUSG00000039182
ENSMUSG00000039183
ENSMUSG00000039191
ENSMUSG00000039194
ENSMUSG00000039195
ENSMUSG00000039197
ENSMUSG00000039199
ENSMUSG00000039201
ENSMUSG00000039202
ENSMUSG00000039205
ENSMUSG00000039206
ENSMUSG00000039210
ENSMUSG00000039218
ENSMUSG00000039219
ENSMUSG00000039220
ENSMUSG00000039221
ENSMUSG00000039230
ENSMUSG00000039231
ENSMUSG00000039233
ENSMUSG00000039234
ENSMUSG00000039239
ENSMUSG00000039242
ENSMUSG00000039244
ENSMUSG00000039252
ENSMUSG00000039253
ENSMUSG00000039254
ENSMUSG00000039257
ENSMUSG00000039262
ENSMUSG00000039263
ENSMUSG00000039270
ENSMUSG00000039275
ENSMUSG00000039278
ENSMUSG00000039285
ENSMUSG00000039286
ENSMUSG00000039294
ENSMUSG00000039298
ENSMUSG00000039307
ENSMUSG00000039308
ENSMUSG00000039313
ENSMUSG00000039316
ENSMUSG00000039318
ENSMUSG00000039323
ENSMUSG00000039328
ENSMUSG00000039345
ENSMUSG00000039347
ENSMUSG00000039349

ENSMUSG00000039354
ENSMUSG00000039356
ENSMUSG00000039357
ENSMUSG00000039361
ENSMUSG00000039367
ENSMUSG00000039372
ENSMUSG00000039375
ENSMUSG00000039382
ENSMUSG00000039385
ENSMUSG00000039405
ENSMUSG00000039410
ENSMUSG00000039414
ENSMUSG00000039419
ENSMUSG00000039427
ENSMUSG00000039428
ENSMUSG00000039431
ENSMUSG00000039449
ENSMUSG00000039450
ENSMUSG00000039452
ENSMUSG00000039456
ENSMUSG00000039458
ENSMUSG00000039461
ENSMUSG00000039463
ENSMUSG00000039470
ENSMUSG00000039473
ENSMUSG00000039474
ENSMUSG00000039477
ENSMUSG00000039478
ENSMUSG00000039483
ENSMUSG00000039485
ENSMUSG00000039488
ENSMUSG00000039501
ENSMUSG00000039509
ENSMUSG00000039512
ENSMUSG00000039515
ENSMUSG00000039519
ENSMUSG00000039523
ENSMUSG00000039530
ENSMUSG00000039531
ENSMUSG00000039533
ENSMUSG00000039536
ENSMUSG00000039539
ENSMUSG00000039542
ENSMUSG00000039546
ENSMUSG00000039552
ENSMUSG00000039556
ENSMUSG00000039568
ENSMUSG00000039577
ENSMUSG00000039578
ENSMUSG00000039579
ENSMUSG00000039585
ENSMUSG00000039599
ENSMUSG00000039601

ENSMUSG00000039607
ENSMUSG00000039611
ENSMUSG00000039615
ENSMUSG00000039620
ENSMUSG00000039621
ENSMUSG00000039623
ENSMUSG00000039629
ENSMUSG00000039630
ENSMUSG00000039632
ENSMUSG00000039633
ENSMUSG00000039634
ENSMUSG00000039637
ENSMUSG00000039640
ENSMUSG00000039646
ENSMUSG00000039648
ENSMUSG00000039652
ENSMUSG00000039656
ENSMUSG00000039660
ENSMUSG00000039661
ENSMUSG00000039662
ENSMUSG00000039671
ENSMUSG00000039676
ENSMUSG00000039678
ENSMUSG00000039680
ENSMUSG00000039682
ENSMUSG00000039683
ENSMUSG00000039686
ENSMUSG00000039693
ENSMUSG00000039697
ENSMUSG00000039701
ENSMUSG00000039703
ENSMUSG00000039704
ENSMUSG00000039706
ENSMUSG00000039713
ENSMUSG00000039715
ENSMUSG00000039716
ENSMUSG00000039717
ENSMUSG00000039725
ENSMUSG00000039735
ENSMUSG00000039737
ENSMUSG00000039738
ENSMUSG00000039740
ENSMUSG00000039741
ENSMUSG00000039747
ENSMUSG00000039753
ENSMUSG00000039754
ENSMUSG00000039756
ENSMUSG00000039759
ENSMUSG00000039763
ENSMUSG00000039765
ENSMUSG00000039768
ENSMUSG00000039770
ENSMUSG00000039771

ENSMUSG00000039781
ENSMUSG00000039782
ENSMUSG00000039787
ENSMUSG00000039789
ENSMUSG00000039795
ENSMUSG00000039801
ENSMUSG00000039804
ENSMUSG00000039809
ENSMUSG00000039810
ENSMUSG00000039824
ENSMUSG00000039826
ENSMUSG00000039828
ENSMUSG00000039830
ENSMUSG00000039831
ENSMUSG00000039834
ENSMUSG00000039835
ENSMUSG00000039838
ENSMUSG00000039840
ENSMUSG00000039841
ENSMUSG00000039842
ENSMUSG00000039844
ENSMUSG00000039849
ENSMUSG00000039850
ENSMUSG00000039852
ENSMUSG00000039860
ENSMUSG00000039879
ENSMUSG00000039886
ENSMUSG00000039887
ENSMUSG00000039901
ENSMUSG00000039904
ENSMUSG00000039910
ENSMUSG00000039911
ENSMUSG00000039913
ENSMUSG00000039914
ENSMUSG00000039917
ENSMUSG00000039929
ENSMUSG00000039936
ENSMUSG00000039943
ENSMUSG00000039952
ENSMUSG00000039953
ENSMUSG00000039954
ENSMUSG00000039959
ENSMUSG00000039960
ENSMUSG00000039963
ENSMUSG00000039967
ENSMUSG00000039968
ENSMUSG00000039976
ENSMUSG00000039982
ENSMUSG00000039983
ENSMUSG00000039987
ENSMUSG00000039988
ENSMUSG00000039989
ENSMUSG00000039990

ENSMUSG00000039994
ENSMUSG00000040003
ENSMUSG00000040006
ENSMUSG00000040007
ENSMUSG00000040009
ENSMUSG00000040010
ENSMUSG00000040018
ENSMUSG00000040021
ENSMUSG00000040022
ENSMUSG00000040025
ENSMUSG00000040028
ENSMUSG00000040029
ENSMUSG00000040033
ENSMUSG00000040035
ENSMUSG00000040037
ENSMUSG00000040040
ENSMUSG00000040043
ENSMUSG00000040044
ENSMUSG00000040048
ENSMUSG00000040054
ENSMUSG00000040055
ENSMUSG00000040097
ENSMUSG00000040102
ENSMUSG00000040105
ENSMUSG00000040111
ENSMUSG00000040112
ENSMUSG00000040118
ENSMUSG00000040123
ENSMUSG00000040124
ENSMUSG00000040125
ENSMUSG00000040128
ENSMUSG00000040133
ENSMUSG00000040136
ENSMUSG00000040147
ENSMUSG00000040151
ENSMUSG00000040152
ENSMUSG00000040167
ENSMUSG00000040174
ENSMUSG00000040177
ENSMUSG00000040181
ENSMUSG00000040183
ENSMUSG00000040188
ENSMUSG00000040195
ENSMUSG00000040209
ENSMUSG00000040213
ENSMUSG00000040219
ENSMUSG00000040220
ENSMUSG00000040225
ENSMUSG00000040234
ENSMUSG00000040236
ENSMUSG00000040242
ENSMUSG00000040249
ENSMUSG00000040250

ENSMUSG00000040254
ENSMUSG00000040260
ENSMUSG00000040263
ENSMUSG00000040265
ENSMUSG00000040268
ENSMUSG00000040269
ENSMUSG00000040270
ENSMUSG00000040274
ENSMUSG00000040276
ENSMUSG00000040282
ENSMUSG00000040289
ENSMUSG00000040297
ENSMUSG00000040302
ENSMUSG00000040321
ENSMUSG00000040325
ENSMUSG00000040327
ENSMUSG00000040331
ENSMUSG00000040339
ENSMUSG00000040351
ENSMUSG00000040354
ENSMUSG00000040356
ENSMUSG00000040359
ENSMUSG00000040363
ENSMUSG00000040365
ENSMUSG00000040370
ENSMUSG00000040372
ENSMUSG00000040373
ENSMUSG00000040374
ENSMUSG00000040383
ENSMUSG00000040385
ENSMUSG00000040387
ENSMUSG00000040389
ENSMUSG00000040390
ENSMUSG00000040396
ENSMUSG00000040407
ENSMUSG00000040410
ENSMUSG00000040412
ENSMUSG00000040414
ENSMUSG00000040415
ENSMUSG00000040420
ENSMUSG00000040423
ENSMUSG00000040430
ENSMUSG00000040433
ENSMUSG00000040435
ENSMUSG00000040446
ENSMUSG00000040447
ENSMUSG00000040451
ENSMUSG00000040455
ENSMUSG00000040459
ENSMUSG00000040462
ENSMUSG00000040463
ENSMUSG00000040464
ENSMUSG00000040466

ENSMUSG00000040472
ENSMUSG00000040473
ENSMUSG00000040479
ENSMUSG00000040481
ENSMUSG00000040482
ENSMUSG00000040488
ENSMUSG00000040490
ENSMUSG00000040495
ENSMUSG00000040502
ENSMUSG00000040506
ENSMUSG00000040520
ENSMUSG00000040521
ENSMUSG00000040524
ENSMUSG00000040532
ENSMUSG00000040536
ENSMUSG00000040537
ENSMUSG00000040543
ENSMUSG00000040548
ENSMUSG00000040549
ENSMUSG00000040550
ENSMUSG00000040560
ENSMUSG00000040563
ENSMUSG00000040565
ENSMUSG00000040570
ENSMUSG00000040584
ENSMUSG00000040586
ENSMUSG00000040591
ENSMUSG00000040594
ENSMUSG00000040596
ENSMUSG00000040599
ENSMUSG00000040606
ENSMUSG00000040612
ENSMUSG00000040616
ENSMUSG00000040618
ENSMUSG00000040620
ENSMUSG00000040621
ENSMUSG00000040624
ENSMUSG00000040631
ENSMUSG00000040640
ENSMUSG00000040648
ENSMUSG00000040649
ENSMUSG00000040651
ENSMUSG00000040652
ENSMUSG00000040653
ENSMUSG00000040659
ENSMUSG00000040661
ENSMUSG00000040667
ENSMUSG00000040669
ENSMUSG00000040675
ENSMUSG00000040681
ENSMUSG00000040687
ENSMUSG00000040688
ENSMUSG00000040690

ENSMUSG00000040697
ENSMUSG00000040699
ENSMUSG00000040703
ENSMUSG00000040710
ENSMUSG00000040711
ENSMUSG00000040712
ENSMUSG00000040713
ENSMUSG00000040717
ENSMUSG00000040720
ENSMUSG00000040721
ENSMUSG00000040722
ENSMUSG00000040724
ENSMUSG00000040725
ENSMUSG00000040729
ENSMUSG00000040731
ENSMUSG00000040738
ENSMUSG00000040740
ENSMUSG00000040746
ENSMUSG00000040749
ENSMUSG00000040752
ENSMUSG00000040759
ENSMUSG00000040760
ENSMUSG00000040761
ENSMUSG00000040767
ENSMUSG00000040771
ENSMUSG00000040774
ENSMUSG00000040782
ENSMUSG00000040785
ENSMUSG00000040794
ENSMUSG00000040795
ENSMUSG00000040797
ENSMUSG00000040811
ENSMUSG00000040813
ENSMUSG00000040818
ENSMUSG00000040820
ENSMUSG00000040822
ENSMUSG00000040824
ENSMUSG00000040836
ENSMUSG00000040842
ENSMUSG00000040843
ENSMUSG00000040848
ENSMUSG00000040850
ENSMUSG00000040852
ENSMUSG00000040855
ENSMUSG00000040856
ENSMUSG00000040857
ENSMUSG00000040859
ENSMUSG00000040860
ENSMUSG00000040865
ENSMUSG00000040867
ENSMUSG00000040875
ENSMUSG00000040877
ENSMUSG00000040883

ENSMUSG00000040888
ENSMUSG00000040896
ENSMUSG00000040907
ENSMUSG00000040913
ENSMUSG00000040918
ENSMUSG00000040928
ENSMUSG00000040929
ENSMUSG00000040936
ENSMUSG00000040938
ENSMUSG00000040940
ENSMUSG00000040943
ENSMUSG00000040945
ENSMUSG00000040952
ENSMUSG00000040957
ENSMUSG00000040964
ENSMUSG00000040972
ENSMUSG00000040978
ENSMUSG00000040990
ENSMUSG00000040997
ENSMUSG00000040998
ENSMUSG00000041000
ENSMUSG00000041014
ENSMUSG00000041020
ENSMUSG00000041025
ENSMUSG00000041028
ENSMUSG00000041035
ENSMUSG00000041037
ENSMUSG00000041040
ENSMUSG00000041046
ENSMUSG00000041057
ENSMUSG00000041058
ENSMUSG00000041073
ENSMUSG00000041075
ENSMUSG00000041078
ENSMUSG00000041079
ENSMUSG00000041084
ENSMUSG00000041096
ENSMUSG00000041112
ENSMUSG00000041115
ENSMUSG00000041117
ENSMUSG00000041119
ENSMUSG00000041120
ENSMUSG00000041124
ENSMUSG00000041126
ENSMUSG00000041130
ENSMUSG00000041132
ENSMUSG00000041133
ENSMUSG00000041141
ENSMUSG00000041144
ENSMUSG00000041147
ENSMUSG00000041153
ENSMUSG00000041164
ENSMUSG00000041168

ENSMUSG00000041180
ENSMUSG00000041199
ENSMUSG00000041203
ENSMUSG00000041205
ENSMUSG00000041215
ENSMUSG00000041216
ENSMUSG00000041220
ENSMUSG00000041225
ENSMUSG00000041229
ENSMUSG00000041231
ENSMUSG00000041235
ENSMUSG00000041236
ENSMUSG00000041241
ENSMUSG00000041245
ENSMUSG00000041258
ENSMUSG00000041261
ENSMUSG00000041263
ENSMUSG00000041264
ENSMUSG00000041268
ENSMUSG00000041272
ENSMUSG00000041278
ENSMUSG00000041297
ENSMUSG00000041298
ENSMUSG00000041303
ENSMUSG00000041308
ENSMUSG00000041309
ENSMUSG00000041313
ENSMUSG00000041319
ENSMUSG00000041323
ENSMUSG00000041328
ENSMUSG00000041329
ENSMUSG00000041341
ENSMUSG00000041343
ENSMUSG00000041346
ENSMUSG00000041351
ENSMUSG00000041353
ENSMUSG00000041354
ENSMUSG00000041355
ENSMUSG00000041360
ENSMUSG00000041362
ENSMUSG00000041375
ENSMUSG00000041378
ENSMUSG00000041380
ENSMUSG00000041396
ENSMUSG00000041408
ENSMUSG00000041415
ENSMUSG00000041417
ENSMUSG00000041420
ENSMUSG00000041423
ENSMUSG00000041426
ENSMUSG00000041438
ENSMUSG00000041439
ENSMUSG00000041444

ENSMUSG00000041445
ENSMUSG00000041453
ENSMUSG00000041459
ENSMUSG00000041468
ENSMUSG00000041471
ENSMUSG00000041477
ENSMUSG00000041483
ENSMUSG00000041488
ENSMUSG00000041491
ENSMUSG00000041506
ENSMUSG00000041528
ENSMUSG00000041530
ENSMUSG00000041540
ENSMUSG00000041544
ENSMUSG00000041548
ENSMUSG00000041552
ENSMUSG00000041556
ENSMUSG00000041560
ENSMUSG00000041570
ENSMUSG00000041571
ENSMUSG00000041577
ENSMUSG00000041592
ENSMUSG00000041594
ENSMUSG00000041598
ENSMUSG00000041607
ENSMUSG00000041608
ENSMUSG00000041609
ENSMUSG00000041617
ENSMUSG00000041623
ENSMUSG00000041624
ENSMUSG00000041625
ENSMUSG00000041629
ENSMUSG00000041632
ENSMUSG00000041638
ENSMUSG00000041642
ENSMUSG00000041645
ENSMUSG00000041649
ENSMUSG00000041650
ENSMUSG00000041654
ENSMUSG00000041658
ENSMUSG00000041660
ENSMUSG00000041670
ENSMUSG00000041671
ENSMUSG00000041679
ENSMUSG00000041684
ENSMUSG00000041685
ENSMUSG00000041688
ENSMUSG00000041695
ENSMUSG00000041697
ENSMUSG00000041702
ENSMUSG00000041703
ENSMUSG00000041708
ENSMUSG00000041710

ENSMUSG00000041712
ENSMUSG00000041716
ENSMUSG00000041720
ENSMUSG00000041729
ENSMUSG00000041731
ENSMUSG00000041733
ENSMUSG00000041734
ENSMUSG00000041740
ENSMUSG00000041741
ENSMUSG00000041747
ENSMUSG00000041757
ENSMUSG00000041762
ENSMUSG00000041763
ENSMUSG00000041765
ENSMUSG00000041769
ENSMUSG00000041771
ENSMUSG00000041773
ENSMUSG00000041774
ENSMUSG00000041775
ENSMUSG00000041777
ENSMUSG00000041781
ENSMUSG00000041794
ENSMUSG00000041797
ENSMUSG00000041798
ENSMUSG00000041801
ENSMUSG00000041809
ENSMUSG00000041815
ENSMUSG00000041817
ENSMUSG00000041828
ENSMUSG00000041836
ENSMUSG00000041837
ENSMUSG00000041841
ENSMUSG00000041842
ENSMUSG00000041846
ENSMUSG00000041852
ENSMUSG00000041870
ENSMUSG00000041879
ENSMUSG00000041881
ENSMUSG00000041889
ENSMUSG00000041890
ENSMUSG00000041891
ENSMUSG00000041895
ENSMUSG00000041907
ENSMUSG00000041911
ENSMUSG00000041912
ENSMUSG00000041915
ENSMUSG00000041920
ENSMUSG00000041921
ENSMUSG00000041923
ENSMUSG00000041926
ENSMUSG00000041930
ENSMUSG00000041935
ENSMUSG00000041936

ENSMUSG00000041939
ENSMUSG00000041949
ENSMUSG00000041957
ENSMUSG00000041958
ENSMUSG00000041961
ENSMUSG00000041966
ENSMUSG00000041975
ENSMUSG00000041977
ENSMUSG00000041986
ENSMUSG00000041992
ENSMUSG00000041995
ENSMUSG00000041997
ENSMUSG00000042010
ENSMUSG00000042015
ENSMUSG00000042029
ENSMUSG00000042032
ENSMUSG00000042035
ENSMUSG00000042042
ENSMUSG00000042043
ENSMUSG00000042046
ENSMUSG00000042050
ENSMUSG00000042055
ENSMUSG00000042063
ENSMUSG00000042066
ENSMUSG00000042073
ENSMUSG00000042078
ENSMUSG00000042079
ENSMUSG00000042082
ENSMUSG00000042097
ENSMUSG00000042099
ENSMUSG00000042104
ENSMUSG00000042105
ENSMUSG00000042109
ENSMUSG00000042111
ENSMUSG00000042115
ENSMUSG00000042116
ENSMUSG00000042121
ENSMUSG00000042129
ENSMUSG00000042133
ENSMUSG00000042138
ENSMUSG00000042148
ENSMUSG00000042155
ENSMUSG00000042156
ENSMUSG00000042167
ENSMUSG00000042178
ENSMUSG00000042182
ENSMUSG00000042185
ENSMUSG00000042197
ENSMUSG00000042198
ENSMUSG00000042202
ENSMUSG00000042203
ENSMUSG00000042207
ENSMUSG00000042208

ENSMUSG00000042210
ENSMUSG00000042211
ENSMUSG00000042215
ENSMUSG00000042216
ENSMUSG00000042229
ENSMUSG00000042246
ENSMUSG00000042249
ENSMUSG00000042256
ENSMUSG00000042258
ENSMUSG00000042271
ENSMUSG00000042272
ENSMUSG00000042275
ENSMUSG00000042284
ENSMUSG00000042286
ENSMUSG00000042292
ENSMUSG00000042293
ENSMUSG00000042298
ENSMUSG00000042302
ENSMUSG00000042303
ENSMUSG00000042305
ENSMUSG00000042308
ENSMUSG00000042312
ENSMUSG00000042323
ENSMUSG00000042328
ENSMUSG00000042331
ENSMUSG00000042348
ENSMUSG00000042350
ENSMUSG00000042353
ENSMUSG00000042354
ENSMUSG00000042359
ENSMUSG00000042363
ENSMUSG00000042364
ENSMUSG00000042369
ENSMUSG00000042380
ENSMUSG00000042388
ENSMUSG00000042389
ENSMUSG00000042390
ENSMUSG00000042396
ENSMUSG00000042401
ENSMUSG00000042404
ENSMUSG00000042406
ENSMUSG00000042408
ENSMUSG00000042410
ENSMUSG00000042419
ENSMUSG00000042423
ENSMUSG00000042425
ENSMUSG00000042426
ENSMUSG00000042428
ENSMUSG00000042429
ENSMUSG00000042439
ENSMUSG00000042444
ENSMUSG00000042446
ENSMUSG00000042447

ENSMUSG00000042453
ENSMUSG00000042460
ENSMUSG00000042462
ENSMUSG00000042472
ENSMUSG00000042473
ENSMUSG00000042487
ENSMUSG00000042492
ENSMUSG00000042496
ENSMUSG00000042500
ENSMUSG00000042502
ENSMUSG00000042506
ENSMUSG00000042507
ENSMUSG00000042508
ENSMUSG00000042515
ENSMUSG00000042520
ENSMUSG00000042523
ENSMUSG00000042524
ENSMUSG00000042529
ENSMUSG00000042532
ENSMUSG00000042535
ENSMUSG00000042541
ENSMUSG00000042548
ENSMUSG00000042557
ENSMUSG00000042558
ENSMUSG00000042564
ENSMUSG00000042567
ENSMUSG00000042569
ENSMUSG00000042570
ENSMUSG00000042572
ENSMUSG00000042579
ENSMUSG00000042581
ENSMUSG00000042589
ENSMUSG00000042590
ENSMUSG00000042594
ENSMUSG00000042595
ENSMUSG00000042599
ENSMUSG00000042604
ENSMUSG00000042605
ENSMUSG00000042606
ENSMUSG00000042607
ENSMUSG00000042608
ENSMUSG00000042613
ENSMUSG00000042616
ENSMUSG00000042625
ENSMUSG00000042626
ENSMUSG00000042628
ENSMUSG00000042631
ENSMUSG00000042632
ENSMUSG00000042642
ENSMUSG00000042650
ENSMUSG00000042659
ENSMUSG00000042660
ENSMUSG00000042662

ENSMUSG00000042670
ENSMUSG00000042671
ENSMUSG00000042675
ENSMUSG00000042680
ENSMUSG00000042682
ENSMUSG00000042686
ENSMUSG00000042688
ENSMUSG00000042694
ENSMUSG00000042699
ENSMUSG00000042700
ENSMUSG00000042705
ENSMUSG00000042707
ENSMUSG00000042709
ENSMUSG00000042712
ENSMUSG00000042719
ENSMUSG00000042724
ENSMUSG00000042726
ENSMUSG00000042729
ENSMUSG00000042734
ENSMUSG00000042737
ENSMUSG00000042742
ENSMUSG00000042743
ENSMUSG00000042744
ENSMUSG00000042745
ENSMUSG00000042747
ENSMUSG00000042750
ENSMUSG00000042751
ENSMUSG00000042757
ENSMUSG00000042761
ENSMUSG00000042763
ENSMUSG00000042766
ENSMUSG00000042770
ENSMUSG00000042772
ENSMUSG00000042787
ENSMUSG00000042790
ENSMUSG00000042797
ENSMUSG00000042804
ENSMUSG00000042807
ENSMUSG00000042810
ENSMUSG00000042826
ENSMUSG00000042831
ENSMUSG00000042834
ENSMUSG00000042846
ENSMUSG00000042851
ENSMUSG00000042854
ENSMUSG00000042870
ENSMUSG00000042873
ENSMUSG00000042901
ENSMUSG00000042903
ENSMUSG00000042942
ENSMUSG00000042978
ENSMUSG00000042992
ENSMUSG00000042997

ENSMUSG00000043004
ENSMUSG00000043019
ENSMUSG00000043059
ENSMUSG00000043061
ENSMUSG00000043065
ENSMUSG00000043067
ENSMUSG00000043079
ENSMUSG00000043090
ENSMUSG00000043131
ENSMUSG00000043140
ENSMUSG00000043153
ENSMUSG00000043154
ENSMUSG00000043162
ENSMUSG00000043164
ENSMUSG00000043183
ENSMUSG00000043190
ENSMUSG00000043207
ENSMUSG00000043241
ENSMUSG00000043252
ENSMUSG00000043257
ENSMUSG00000043259
ENSMUSG00000043262
ENSMUSG00000043279
ENSMUSG00000043284
ENSMUSG00000043290
ENSMUSG00000043300
ENSMUSG00000043301
ENSMUSG00000043311
ENSMUSG00000043313
ENSMUSG00000043323
ENSMUSG00000043372
ENSMUSG00000043384
ENSMUSG00000043388
ENSMUSG00000043391
ENSMUSG00000043398
ENSMUSG00000043411
ENSMUSG00000043415
ENSMUSG00000043419
ENSMUSG00000043424
ENSMUSG00000043432
ENSMUSG00000043439
ENSMUSG00000043441
ENSMUSG00000043445
ENSMUSG00000043448
ENSMUSG00000043456
ENSMUSG00000043458
ENSMUSG00000043460
ENSMUSG00000043463
ENSMUSG00000043467
ENSMUSG00000043487
ENSMUSG00000043496
ENSMUSG00000043518
ENSMUSG00000043531

ENSMUSG00000043535
ENSMUSG00000043541
ENSMUSG00000043542
ENSMUSG00000043557
ENSMUSG00000043572
ENSMUSG00000043587
ENSMUSG00000043602
ENSMUSG00000043614
ENSMUSG00000043621
ENSMUSG00000043629
ENSMUSG00000043635
ENSMUSG00000043639
ENSMUSG00000043648
ENSMUSG00000043668
ENSMUSG00000043670
ENSMUSG00000043671
ENSMUSG00000043683
ENSMUSG00000043687
ENSMUSG00000043702
ENSMUSG00000043716
ENSMUSG00000043733
ENSMUSG00000043773
ENSMUSG00000043801
ENSMUSG00000043811
ENSMUSG00000043831
ENSMUSG00000043843
ENSMUSG00000043857
ENSMUSG00000043866
ENSMUSG00000043872
ENSMUSG00000043881
ENSMUSG00000043885
ENSMUSG00000043903
ENSMUSG00000043909
ENSMUSG00000043923
ENSMUSG00000043929
ENSMUSG00000043940
ENSMUSG00000043962
ENSMUSG00000043964
ENSMUSG00000043987
ENSMUSG00000043991
ENSMUSG00000043993
ENSMUSG00000043998
ENSMUSG00000043999
ENSMUSG00000044005
ENSMUSG00000044018
ENSMUSG00000044024
ENSMUSG00000044030
ENSMUSG00000044033
ENSMUSG00000044037
ENSMUSG00000044042
ENSMUSG00000044043
ENSMUSG00000044066
ENSMUSG00000044067

ENSMUSG00000044068
ENSMUSG00000044071
ENSMUSG00000044072
ENSMUSG00000044080
ENSMUSG00000044098
ENSMUSG00000044117
ENSMUSG00000044134
ENSMUSG00000044147
ENSMUSG00000044148
ENSMUSG00000044149
ENSMUSG00000044155
ENSMUSG00000044164
ENSMUSG00000044167
ENSMUSG00000044197
ENSMUSG00000044216
ENSMUSG00000044221
ENSMUSG00000044224
ENSMUSG00000044229
ENSMUSG00000044231
ENSMUSG00000044250
ENSMUSG00000044252
ENSMUSG00000044285
ENSMUSG00000044288
ENSMUSG00000044308
ENSMUSG00000044328
ENSMUSG00000044337
ENSMUSG00000044340
ENSMUSG00000044345
ENSMUSG00000044348
ENSMUSG00000044349
ENSMUSG00000044352
ENSMUSG00000044364
ENSMUSG00000044365
ENSMUSG00000044367
ENSMUSG00000044390
ENSMUSG00000044393
ENSMUSG00000044408
ENSMUSG00000044433
ENSMUSG00000044442
ENSMUSG00000044447
ENSMUSG00000044452
ENSMUSG00000044465
ENSMUSG00000044468
ENSMUSG00000044469
ENSMUSG00000044471
ENSMUSG00000044475
ENSMUSG00000044477
ENSMUSG00000044496
ENSMUSG00000044499
ENSMUSG00000044502
ENSMUSG00000044528
ENSMUSG00000044533
ENSMUSG00000044548

ENSMUSG00000044550
ENSMUSG00000044562
ENSMUSG00000044566
ENSMUSG00000044573
ENSMUSG00000044576
ENSMUSG00000044600
ENSMUSG00000044617
ENSMUSG00000044627
ENSMUSG00000044628
ENSMUSG00000044629
ENSMUSG00000044636
ENSMUSG00000044641
ENSMUSG00000044645
ENSMUSG00000044646
ENSMUSG00000044647
ENSMUSG00000044667
ENSMUSG00000044674
ENSMUSG00000044676
ENSMUSG00000044700
ENSMUSG00000044708
ENSMUSG00000044709
ENSMUSG00000044712
ENSMUSG00000044715
ENSMUSG00000044716
ENSMUSG00000044730
ENSMUSG00000044763
ENSMUSG00000044768
ENSMUSG00000044770
ENSMUSG00000044783
ENSMUSG00000044788
ENSMUSG00000044791
ENSMUSG00000044792
ENSMUSG00000044795
ENSMUSG00000044807
ENSMUSG00000044813
ENSMUSG00000044835
ENSMUSG00000044847
ENSMUSG00000044857
ENSMUSG00000044864
ENSMUSG00000044876
ENSMUSG00000044894
ENSMUSG00000044906
ENSMUSG00000044912
ENSMUSG00000044921
ENSMUSG00000044927
ENSMUSG00000044933
ENSMUSG00000044934
ENSMUSG00000044948
ENSMUSG00000044949
ENSMUSG00000044950
ENSMUSG00000044952
ENSMUSG00000044968
ENSMUSG00000044982

ENSMUSG00000044986
ENSMUSG00000044991
ENSMUSG00000045005
ENSMUSG00000045007
ENSMUSG00000045009
ENSMUSG00000045034
ENSMUSG00000045038
ENSMUSG00000045039
ENSMUSG00000045045
ENSMUSG00000045053
ENSMUSG00000045062
ENSMUSG00000045064
ENSMUSG00000045071
ENSMUSG00000045078
ENSMUSG00000045083
ENSMUSG00000045087
ENSMUSG00000045092
ENSMUSG00000045095
ENSMUSG00000045098
ENSMUSG00000045100
ENSMUSG00000045103
ENSMUSG00000045107
ENSMUSG00000045114
ENSMUSG00000045128
ENSMUSG00000045136
ENSMUSG00000045160
ENSMUSG00000045174
ENSMUSG00000045176
ENSMUSG00000045179
ENSMUSG00000045180
ENSMUSG00000045193
ENSMUSG00000045201
ENSMUSG00000045205
ENSMUSG00000045210
ENSMUSG00000045211
ENSMUSG00000045215
ENSMUSG00000045216
ENSMUSG00000045237
ENSMUSG00000045248
ENSMUSG00000045251
ENSMUSG00000045252
ENSMUSG00000045257
ENSMUSG00000045259
ENSMUSG00000045284
ENSMUSG00000045287
ENSMUSG00000045294
ENSMUSG00000045302
ENSMUSG00000045312
ENSMUSG00000045316
ENSMUSG00000045318
ENSMUSG00000045333
ENSMUSG00000045348
ENSMUSG00000045349

ENSMUSG00000045374
ENSMUSG00000045382
ENSMUSG00000045404
ENSMUSG00000045409
ENSMUSG00000045411
ENSMUSG00000045414
ENSMUSG00000045427
ENSMUSG00000045435
ENSMUSG00000045438
ENSMUSG00000045441
ENSMUSG00000045466
ENSMUSG00000045482
ENSMUSG00000045498
ENSMUSG00000045503
ENSMUSG00000045515
ENSMUSG00000045519
ENSMUSG00000045532
ENSMUSG00000045534
ENSMUSG00000045538
ENSMUSG00000045573
ENSMUSG00000045576
ENSMUSG00000045589
ENSMUSG00000045594
ENSMUSG00000045598
ENSMUSG00000045608
ENSMUSG00000045613
ENSMUSG00000045624
ENSMUSG00000045636
ENSMUSG00000045639
ENSMUSG00000045648
ENSMUSG00000045655
ENSMUSG00000045658
ENSMUSG00000045659
ENSMUSG00000045664
ENSMUSG00000045665
ENSMUSG00000045671
ENSMUSG00000045672
ENSMUSG00000045691
ENSMUSG00000045731
ENSMUSG00000045733
ENSMUSG00000045752
ENSMUSG00000045761
ENSMUSG00000045763
ENSMUSG00000045767
ENSMUSG00000045777
ENSMUSG00000045790
ENSMUSG00000045795
ENSMUSG00000045813
ENSMUSG00000045817
ENSMUSG00000045822
ENSMUSG00000045838
ENSMUSG00000045867
ENSMUSG00000045875

ENSMUSG00000045896
ENSMUSG00000045903
ENSMUSG00000045912
ENSMUSG00000045917
ENSMUSG00000045948
ENSMUSG00000045954
ENSMUSG00000045962
ENSMUSG00000045967
ENSMUSG00000045969
ENSMUSG00000045973
ENSMUSG00000045975
ENSMUSG00000045980
ENSMUSG00000045983
ENSMUSG00000045991
ENSMUSG00000045994
ENSMUSG00000045996
ENSMUSG00000046010
ENSMUSG00000046020
ENSMUSG00000046027
ENSMUSG00000046032
ENSMUSG00000046034
ENSMUSG00000046058
ENSMUSG00000046062
ENSMUSG00000046079
ENSMUSG00000046093
ENSMUSG00000046096
ENSMUSG00000046111
ENSMUSG00000046138
ENSMUSG00000046139
ENSMUSG00000046152
ENSMUSG00000046157
ENSMUSG00000046159
ENSMUSG00000046160
ENSMUSG00000046169
ENSMUSG00000046178
ENSMUSG00000046182
ENSMUSG00000046185
ENSMUSG00000046191
ENSMUSG00000046192
ENSMUSG00000046201
ENSMUSG00000046204
ENSMUSG00000046215
ENSMUSG00000046229
ENSMUSG00000046230
ENSMUSG00000046240
ENSMUSG00000046269
ENSMUSG00000046287
ENSMUSG00000046311
ENSMUSG00000046312
ENSMUSG00000046314
ENSMUSG00000046321
ENSMUSG00000046324
ENSMUSG00000046329

ENSMUSG00000046330
ENSMUSG00000046351
ENSMUSG00000046364
ENSMUSG00000046378
ENSMUSG00000046380
ENSMUSG00000046387
ENSMUSG00000046402
ENSMUSG00000046404
ENSMUSG00000046432
ENSMUSG00000046434
ENSMUSG00000046441
ENSMUSG00000046442
ENSMUSG00000046447
ENSMUSG00000046449
ENSMUSG00000046480
ENSMUSG00000046516
ENSMUSG00000046519
ENSMUSG00000046523
ENSMUSG00000046532
ENSMUSG00000046541
ENSMUSG00000046546
ENSMUSG00000046556
ENSMUSG00000046562
ENSMUSG00000046567
ENSMUSG00000046572
ENSMUSG00000046573
ENSMUSG00000046574
ENSMUSG00000046593
ENSMUSG00000046598
ENSMUSG00000046603
ENSMUSG00000046605
ENSMUSG00000046607
ENSMUSG00000046613
ENSMUSG00000046637
ENSMUSG00000046658
ENSMUSG00000046667
ENSMUSG00000046668
ENSMUSG00000046671
ENSMUSG00000046675
ENSMUSG00000046679
ENSMUSG00000046691
ENSMUSG00000046699
ENSMUSG00000046707
ENSMUSG00000046709
ENSMUSG00000046711
ENSMUSG00000046722
ENSMUSG00000046727
ENSMUSG00000046743
ENSMUSG00000046753
ENSMUSG00000046756
ENSMUSG00000046761
ENSMUSG00000046785
ENSMUSG00000046791

ENSMUSG00000046792
ENSMUSG00000046793
ENSMUSG00000046798
ENSMUSG00000046805
ENSMUSG00000046807
ENSMUSG00000046822
ENSMUSG00000046836
ENSMUSG00000046841
ENSMUSG00000046844
ENSMUSG00000046861
ENSMUSG00000046862
ENSMUSG00000046865
ENSMUSG00000046873
ENSMUSG00000046876
ENSMUSG00000046897
ENSMUSG00000046922
ENSMUSG00000046947
ENSMUSG00000046949
ENSMUSG00000046962
ENSMUSG00000046982
ENSMUSG00000046985
ENSMUSG00000046994
ENSMUSG00000046997
ENSMUSG00000047003
ENSMUSG00000047013
ENSMUSG00000047021
ENSMUSG00000047030
ENSMUSG00000047036
ENSMUSG00000047037
ENSMUSG00000047044
ENSMUSG00000047045
ENSMUSG00000047067
ENSMUSG00000047084
ENSMUSG00000047085
ENSMUSG00000047090
ENSMUSG00000047098
ENSMUSG00000047123
ENSMUSG00000047126
ENSMUSG00000047139
ENSMUSG00000047141
ENSMUSG00000047146
ENSMUSG00000047153
ENSMUSG00000047155
ENSMUSG00000047181
ENSMUSG00000047187
ENSMUSG00000047193
ENSMUSG00000047205
ENSMUSG00000047213
ENSMUSG00000047215
ENSMUSG00000047221
ENSMUSG00000047238
ENSMUSG00000047242
ENSMUSG00000047246

ENSMUSG00000047248
ENSMUSG00000047250
ENSMUSG00000047260
ENSMUSG00000047261
ENSMUSG00000047264
ENSMUSG00000047284
ENSMUSG00000047342
ENSMUSG00000047344
ENSMUSG00000047361
ENSMUSG00000047368
ENSMUSG00000047371
ENSMUSG00000047379
ENSMUSG00000047388
ENSMUSG00000047394
ENSMUSG00000047409
ENSMUSG00000047412
ENSMUSG00000047414
ENSMUSG00000047415
ENSMUSG00000047417
ENSMUSG00000047419
ENSMUSG00000047423
ENSMUSG00000047428
ENSMUSG00000047434
ENSMUSG00000047446
ENSMUSG00000047454
ENSMUSG00000047459
ENSMUSG00000047466
ENSMUSG00000047473
ENSMUSG00000047495
ENSMUSG00000047496
ENSMUSG00000047502
ENSMUSG00000047507
ENSMUSG00000047514
ENSMUSG00000047539
ENSMUSG00000047547
ENSMUSG00000047554
ENSMUSG00000047557
ENSMUSG00000047583
ENSMUSG00000047603
ENSMUSG00000047604
ENSMUSG00000047613
ENSMUSG00000047617
ENSMUSG00000047635
ENSMUSG00000047648
ENSMUSG00000047649
ENSMUSG00000047656
ENSMUSG00000047658
ENSMUSG00000047669
ENSMUSG00000047675
ENSMUSG00000047694
ENSMUSG00000047710
ENSMUSG00000047712
ENSMUSG00000047714

ENSMUSG00000047719
ENSMUSG00000047721
ENSMUSG00000047731
ENSMUSG00000047747
ENSMUSG00000047749
ENSMUSG00000047766
ENSMUSG00000047767
ENSMUSG00000047773
ENSMUSG00000047777
ENSMUSG00000047786
ENSMUSG00000047787
ENSMUSG00000047789
ENSMUSG00000047793
ENSMUSG00000047797
ENSMUSG00000047804
ENSMUSG00000047824
ENSMUSG00000047832
ENSMUSG00000047842
ENSMUSG00000047843
ENSMUSG00000047844
ENSMUSG00000047866
ENSMUSG00000047879
ENSMUSG00000047888
ENSMUSG00000047904
ENSMUSG00000047907
ENSMUSG00000047909
ENSMUSG00000047910
ENSMUSG00000047921
ENSMUSG00000047935
ENSMUSG00000047945
ENSMUSG00000047959
ENSMUSG00000047976
ENSMUSG00000047989
ENSMUSG00000047992
ENSMUSG00000048000
ENSMUSG00000048001
ENSMUSG00000048004
ENSMUSG00000048022
ENSMUSG00000048027
ENSMUSG00000048029
ENSMUSG00000048038
ENSMUSG00000048039
ENSMUSG00000048040
ENSMUSG00000048047
ENSMUSG00000048058
ENSMUSG00000048076
ENSMUSG00000048078
ENSMUSG00000048100
ENSMUSG00000048106
ENSMUSG00000048109
ENSMUSG00000048118
ENSMUSG00000048120
ENSMUSG00000048126

ENSMUSG00000048142
ENSMUSG00000048148
ENSMUSG00000048154
ENSMUSG00000048163
ENSMUSG00000048170
ENSMUSG00000048175
ENSMUSG00000048216
ENSMUSG00000048218
ENSMUSG00000048222
ENSMUSG00000048232
ENSMUSG00000048234
ENSMUSG00000048240
ENSMUSG00000048249
ENSMUSG00000048251
ENSMUSG00000048264
ENSMUSG00000048271
ENSMUSG00000048277
ENSMUSG00000048279
ENSMUSG00000048280
ENSMUSG00000048281
ENSMUSG00000048285
ENSMUSG00000048304
ENSMUSG00000048307
ENSMUSG00000048310
ENSMUSG00000048330
ENSMUSG00000048332
ENSMUSG00000048347
ENSMUSG00000048351
ENSMUSG00000048355
ENSMUSG00000048371
ENSMUSG00000048376
ENSMUSG00000048379
ENSMUSG00000048385
ENSMUSG00000048388
ENSMUSG00000048402
ENSMUSG00000048410
ENSMUSG00000048429
ENSMUSG00000048439
ENSMUSG00000048445
ENSMUSG00000048458
ENSMUSG00000048478
ENSMUSG00000048481
ENSMUSG00000048483
ENSMUSG00000048486
ENSMUSG00000048490
ENSMUSG00000048503
ENSMUSG00000048537
ENSMUSG00000048546
ENSMUSG00000048550
ENSMUSG00000048562
ENSMUSG00000048578
ENSMUSG00000048581
ENSMUSG00000048583

ENSMUSG00000048616
ENSMUSG00000048644
ENSMUSG00000048661
ENSMUSG00000048668
ENSMUSG00000048696
ENSMUSG00000048701
ENSMUSG00000048706
ENSMUSG00000048707
ENSMUSG00000048720
ENSMUSG00000048721
ENSMUSG00000048732
ENSMUSG00000048755
ENSMUSG00000048756
ENSMUSG00000048758
ENSMUSG00000048787
ENSMUSG00000048796
ENSMUSG00000048799
ENSMUSG00000048807
ENSMUSG00000048814
ENSMUSG00000048826
ENSMUSG00000048832
ENSMUSG00000048833
ENSMUSG00000048834
ENSMUSG00000048856
ENSMUSG00000048874
ENSMUSG00000048878
ENSMUSG00000048895
ENSMUSG00000048897
ENSMUSG00000048899
ENSMUSG00000048905
ENSMUSG00000048911
ENSMUSG00000048915
ENSMUSG00000048920
ENSMUSG00000048921
ENSMUSG00000048930
ENSMUSG00000048939
ENSMUSG00000048960
ENSMUSG00000048965
ENSMUSG00000048967
ENSMUSG00000048970
ENSMUSG00000048978
ENSMUSG00000048988
ENSMUSG00000048997
ENSMUSG00000049001
ENSMUSG00000049036
ENSMUSG00000049038
ENSMUSG00000049044
ENSMUSG00000049047
ENSMUSG00000049076
ENSMUSG00000049086
ENSMUSG00000049090
ENSMUSG00000049091
ENSMUSG00000049092

ENSMUSG00000049097
ENSMUSG00000049100
ENSMUSG00000049106
ENSMUSG00000049112
ENSMUSG00000049119
ENSMUSG00000049122
ENSMUSG00000049148
ENSMUSG00000049164
ENSMUSG00000049176
ENSMUSG00000049184
ENSMUSG00000049191
ENSMUSG00000049225
ENSMUSG00000049232
ENSMUSG00000049252
ENSMUSG00000049265
ENSMUSG00000049281
ENSMUSG00000049285
ENSMUSG00000049287
ENSMUSG00000049288
ENSMUSG00000049295
ENSMUSG00000049299
ENSMUSG00000049300
ENSMUSG00000049303
ENSMUSG00000049305
ENSMUSG00000049313
ENSMUSG00000049321
ENSMUSG00000049323
ENSMUSG00000049327
ENSMUSG00000049336
ENSMUSG00000049339
ENSMUSG00000049354
ENSMUSG00000049396
ENSMUSG00000049401
ENSMUSG00000049411
ENSMUSG00000049420
ENSMUSG00000049421
ENSMUSG00000049422
ENSMUSG00000049439
ENSMUSG00000049470
ENSMUSG00000049482
ENSMUSG00000049488
ENSMUSG00000049489
ENSMUSG00000049493
ENSMUSG00000049504
ENSMUSG00000049511
ENSMUSG00000049516
ENSMUSG00000049517
ENSMUSG00000049521
ENSMUSG00000049532
ENSMUSG00000049536
ENSMUSG00000049538
ENSMUSG00000049539
ENSMUSG00000049550

ENSMUSG00000049551
ENSMUSG00000049553
ENSMUSG00000049555
ENSMUSG00000049556
ENSMUSG00000049562
ENSMUSG00000049571
ENSMUSG00000049577
ENSMUSG00000049580
ENSMUSG00000049583
ENSMUSG00000049600
ENSMUSG00000049606
ENSMUSG00000049612
ENSMUSG00000049624
ENSMUSG00000049630
ENSMUSG00000049643
ENSMUSG00000049657
ENSMUSG00000049658
ENSMUSG00000049659
ENSMUSG00000049670
ENSMUSG00000049672
ENSMUSG00000049680
ENSMUSG00000049686
ENSMUSG00000049690
ENSMUSG00000049717
ENSMUSG00000049721
ENSMUSG00000049728
ENSMUSG00000049739
ENSMUSG00000049751
ENSMUSG00000049755
ENSMUSG00000049760
ENSMUSG00000049764
ENSMUSG00000049775
ENSMUSG00000049791
ENSMUSG00000049792
ENSMUSG00000049796
ENSMUSG00000049800
ENSMUSG00000049804
ENSMUSG00000049807
ENSMUSG00000049811
ENSMUSG00000049823
ENSMUSG00000049858
ENSMUSG00000049866
ENSMUSG00000049878
ENSMUSG00000049881
ENSMUSG00000049882
ENSMUSG00000049892
ENSMUSG00000049904
ENSMUSG00000049907
ENSMUSG00000049922
ENSMUSG00000049932
ENSMUSG00000049939
ENSMUSG00000049940
ENSMUSG00000049946

ENSMUSG00000049957
ENSMUSG00000049960
ENSMUSG00000049971
ENSMUSG00000049985
ENSMUSG00000049999
ENSMUSG00000050002
ENSMUSG00000050017
ENSMUSG00000050022
ENSMUSG00000050029
ENSMUSG00000050043
ENSMUSG00000050052
ENSMUSG00000050064
ENSMUSG00000050069
ENSMUSG00000050071
ENSMUSG00000050079
ENSMUSG00000050088
ENSMUSG00000050121
ENSMUSG00000050132
ENSMUSG00000050138
ENSMUSG00000050144
ENSMUSG00000050148
ENSMUSG00000050164
ENSMUSG00000050188
ENSMUSG00000050192
ENSMUSG00000050199
ENSMUSG00000050211
ENSMUSG00000050213
ENSMUSG00000050229
ENSMUSG00000050240
ENSMUSG00000050244
ENSMUSG00000050248
ENSMUSG00000050271
ENSMUSG00000050272
ENSMUSG00000050288
ENSMUSG00000050310
ENSMUSG00000050312
ENSMUSG00000050315
ENSMUSG00000050321
ENSMUSG00000050332
ENSMUSG00000050334
ENSMUSG00000050357
ENSMUSG00000050373
ENSMUSG00000050379
ENSMUSG00000050382
ENSMUSG00000050390
ENSMUSG00000050394
ENSMUSG00000050423
ENSMUSG00000050428
ENSMUSG00000050447
ENSMUSG00000050471
ENSMUSG00000050473
ENSMUSG00000050505
ENSMUSG00000050511

ENSMUSG00000050530
ENSMUSG00000050538
ENSMUSG00000050545
ENSMUSG00000050552
ENSMUSG00000050556
ENSMUSG00000050565
ENSMUSG00000050567
ENSMUSG00000050587
ENSMUSG00000050600
ENSMUSG00000050605
ENSMUSG00000050608
ENSMUSG00000050619
ENSMUSG00000050627
ENSMUSG00000050628
ENSMUSG00000050640
ENSMUSG00000050663
ENSMUSG00000050668
ENSMUSG00000050697
ENSMUSG00000050705
ENSMUSG00000050708
ENSMUSG00000050711
ENSMUSG00000050714
ENSMUSG00000050721
ENSMUSG00000050730
ENSMUSG00000050732
ENSMUSG00000050751
ENSMUSG00000050783
ENSMUSG00000050786
ENSMUSG00000050796
ENSMUSG00000050812
ENSMUSG00000050821
ENSMUSG00000050822
ENSMUSG00000050824
ENSMUSG00000050830
ENSMUSG00000050840
ENSMUSG00000050846
ENSMUSG00000050854
ENSMUSG00000050855
ENSMUSG00000050856
ENSMUSG00000050860
ENSMUSG00000050875
ENSMUSG00000050890
ENSMUSG00000050891
ENSMUSG00000050908
ENSMUSG00000050910
ENSMUSG00000050912
ENSMUSG00000050930
ENSMUSG00000050936
ENSMUSG00000050945
ENSMUSG00000050947
ENSMUSG00000050953
ENSMUSG00000050954
ENSMUSG00000050963

ENSMUSG00000050965
ENSMUSG00000050967
ENSMUSG00000050973
ENSMUSG00000050989
ENSMUSG00000051007
ENSMUSG00000051022
ENSMUSG00000051034
ENSMUSG00000051041
ENSMUSG00000051048
ENSMUSG00000051062
ENSMUSG00000051065
ENSMUSG00000051067
ENSMUSG00000051098
ENSMUSG00000051111
ENSMUSG00000051113
ENSMUSG00000051146
ENSMUSG00000051149
ENSMUSG00000051154
ENSMUSG00000051166
ENSMUSG00000051169
ENSMUSG00000051177
ENSMUSG00000051185
ENSMUSG00000051223
ENSMUSG00000051232
ENSMUSG00000051234
ENSMUSG00000051238
ENSMUSG00000051242
ENSMUSG00000051243
ENSMUSG00000051246
ENSMUSG00000051256
ENSMUSG00000051285
ENSMUSG00000051306
ENSMUSG00000051316
ENSMUSG00000051319
ENSMUSG00000051323
ENSMUSG00000051329
ENSMUSG00000051331
ENSMUSG00000051335
ENSMUSG00000051339
ENSMUSG00000051343
ENSMUSG00000051344
ENSMUSG00000051346
ENSMUSG00000051351
ENSMUSG00000051355
ENSMUSG00000051359
ENSMUSG00000051373
ENSMUSG00000051375
ENSMUSG00000051379
ENSMUSG00000051390
ENSMUSG00000051391
ENSMUSG00000051396
ENSMUSG00000051401
ENSMUSG00000051403

ENSMUSG00000051412
ENSMUSG00000051413
ENSMUSG00000051427
ENSMUSG00000051435
ENSMUSG00000051451
ENSMUSG00000051469
ENSMUSG00000051483
ENSMUSG00000051495
ENSMUSG00000051497
ENSMUSG00000051499
ENSMUSG00000051502
ENSMUSG00000051504
ENSMUSG00000051510
ENSMUSG00000051515
ENSMUSG00000051518
ENSMUSG00000051527
ENSMUSG00000051537
ENSMUSG00000051550
ENSMUSG00000051557
ENSMUSG00000051579
ENSMUSG00000051586
ENSMUSG00000051590
ENSMUSG00000051615
ENSMUSG00000051627
ENSMUSG00000051650
ENSMUSG00000051652
ENSMUSG00000051671
ENSMUSG00000051674
ENSMUSG00000051675
ENSMUSG00000051695
ENSMUSG00000051703
ENSMUSG00000051705
ENSMUSG00000051721
ENSMUSG00000051726
ENSMUSG00000051730
ENSMUSG00000051747
ENSMUSG00000051768
ENSMUSG00000051786
ENSMUSG00000051790
ENSMUSG00000051817
ENSMUSG00000051851
ENSMUSG00000051853
ENSMUSG00000051855
ENSMUSG00000051864
ENSMUSG00000051890
ENSMUSG00000051910
ENSMUSG00000051934
ENSMUSG00000051950
ENSMUSG00000051951
ENSMUSG00000051989
ENSMUSG00000052026
ENSMUSG00000052031
ENSMUSG00000052033

ENSMUSG00000052040
ENSMUSG00000052056
ENSMUSG00000052062
ENSMUSG00000052085
ENSMUSG00000052087
ENSMUSG00000052102
ENSMUSG00000052105
ENSMUSG00000052133
ENSMUSG00000052135
ENSMUSG00000052137
ENSMUSG00000052139
ENSMUSG00000052144
ENSMUSG00000052146
ENSMUSG00000052151
ENSMUSG00000052155
ENSMUSG00000052214
ENSMUSG00000052229
ENSMUSG00000052253
ENSMUSG00000052273
ENSMUSG00000052293
ENSMUSG00000052296
ENSMUSG00000052298
ENSMUSG00000052299
ENSMUSG00000052301
ENSMUSG00000052302
ENSMUSG00000052305
ENSMUSG00000052310
ENSMUSG00000052331
ENSMUSG00000052336
ENSMUSG00000052337
ENSMUSG00000052353
ENSMUSG00000052364
ENSMUSG00000052369
ENSMUSG00000052372
ENSMUSG00000052373
ENSMUSG00000052374
ENSMUSG00000052387
ENSMUSG00000052395
ENSMUSG00000052397
ENSMUSG00000052406
ENSMUSG00000052407
ENSMUSG00000052419
ENSMUSG00000052423
ENSMUSG00000052428
ENSMUSG00000052430
ENSMUSG00000052446
ENSMUSG00000052456
ENSMUSG00000052459
ENSMUSG00000052488
ENSMUSG00000052504
ENSMUSG00000052512
ENSMUSG00000052516
ENSMUSG00000052533

ENSMUSG00000052534
ENSMUSG00000052539
ENSMUSG00000052544
ENSMUSG00000052551
ENSMUSG00000052557
ENSMUSG00000052560
ENSMUSG00000052565
ENSMUSG00000052566
ENSMUSG00000052572
ENSMUSG00000052581
ENSMUSG00000052584
ENSMUSG00000052593
ENSMUSG00000052609
ENSMUSG00000052613
ENSMUSG00000052632
ENSMUSG00000052656
ENSMUSG00000052675
ENSMUSG00000052676
ENSMUSG00000052681
ENSMUSG00000052684
ENSMUSG00000052698
ENSMUSG00000052707
ENSMUSG00000052712
ENSMUSG00000052713
ENSMUSG00000052726
ENSMUSG00000052727
ENSMUSG00000052738
ENSMUSG00000052748
ENSMUSG00000052751
ENSMUSG00000052752
ENSMUSG00000052763
ENSMUSG00000052783
ENSMUSG00000052794
ENSMUSG00000052798
ENSMUSG00000052812
ENSMUSG00000052833
ENSMUSG00000052837
ENSMUSG00000052848
ENSMUSG00000052852
ENSMUSG00000052861
ENSMUSG00000052889
ENSMUSG00000052906
ENSMUSG00000052911
ENSMUSG00000052914
ENSMUSG00000052915
ENSMUSG00000052917
ENSMUSG00000052920
ENSMUSG00000052921
ENSMUSG00000052926
ENSMUSG00000052928
ENSMUSG00000052934
ENSMUSG00000052942
ENSMUSG00000052949

ENSMUSG00000052957
ENSMUSG00000052962
ENSMUSG00000052981
ENSMUSG00000052997
ENSMUSG00000053004
ENSMUSG00000053007
ENSMUSG00000053012
ENSMUSG00000053024
ENSMUSG00000053025
ENSMUSG00000053040
ENSMUSG00000053046
ENSMUSG00000053062
ENSMUSG00000053070
ENSMUSG00000053080
ENSMUSG00000053091
ENSMUSG00000053093
ENSMUSG00000053094
ENSMUSG00000053110
ENSMUSG00000053111
ENSMUSG00000053119
ENSMUSG00000053128
ENSMUSG00000053134
ENSMUSG00000053137
ENSMUSG00000053141
ENSMUSG00000053166
ENSMUSG00000053192
ENSMUSG00000053199
ENSMUSG00000053205
ENSMUSG00000053214
ENSMUSG00000053253
ENSMUSG00000053286
ENSMUSG00000053289
ENSMUSG00000053291
ENSMUSG00000053293
ENSMUSG00000053297
ENSMUSG00000053310
ENSMUSG00000053317
ENSMUSG00000053329
ENSMUSG00000053332
ENSMUSG00000053333
ENSMUSG00000053334
ENSMUSG00000053390
ENSMUSG00000053395
ENSMUSG00000053398
ENSMUSG00000053411
ENSMUSG00000053414
ENSMUSG00000053436
ENSMUSG00000053453
ENSMUSG00000053460
ENSMUSG00000053465
ENSMUSG00000053470
ENSMUSG00000053477
ENSMUSG00000053483

ENSMUSG00000053510
ENSMUSG00000053519
ENSMUSG00000053536
ENSMUSG00000053550
ENSMUSG00000053552
ENSMUSG00000053553
ENSMUSG00000053565
ENSMUSG00000053580
ENSMUSG00000053581
ENSMUSG00000053604
ENSMUSG00000053617
ENSMUSG00000053641
ENSMUSG00000053644
ENSMUSG00000053646
ENSMUSG00000053684
ENSMUSG00000053693
ENSMUSG00000053702
ENSMUSG00000053716
ENSMUSG00000053730
ENSMUSG00000053754
ENSMUSG00000053768
ENSMUSG00000053769
ENSMUSG00000053774
ENSMUSG00000053799
ENSMUSG00000053801
ENSMUSG00000053819
ENSMUSG00000053825
ENSMUSG00000053838
ENSMUSG00000053841
ENSMUSG00000053870
ENSMUSG00000053877
ENSMUSG00000053898
ENSMUSG00000053907
ENSMUSG00000053916
ENSMUSG00000053929
ENSMUSG00000053930
ENSMUSG00000053931
ENSMUSG00000053950
ENSMUSG00000053963
ENSMUSG00000053985
ENSMUSG00000054000
ENSMUSG00000054008
ENSMUSG00000054013
ENSMUSG00000054021
ENSMUSG00000054027
ENSMUSG00000054034
ENSMUSG00000054051
ENSMUSG00000054079
ENSMUSG00000054091
ENSMUSG00000054099
ENSMUSG00000054115
ENSMUSG00000054162
ENSMUSG00000054199

ENSMUSG00000054226
ENSMUSG00000054237
ENSMUSG00000054252
ENSMUSG00000054256
ENSMUSG00000054263
ENSMUSG00000054277
ENSMUSG00000054280
ENSMUSG00000054302
ENSMUSG00000054309
ENSMUSG00000054312
ENSMUSG00000054364
ENSMUSG00000054381
ENSMUSG00000054383
ENSMUSG00000054387
ENSMUSG00000054405
ENSMUSG00000054408
ENSMUSG00000054409
ENSMUSG00000054414
ENSMUSG00000054423
ENSMUSG00000054426
ENSMUSG00000054428
ENSMUSG00000054434
ENSMUSG00000054452
ENSMUSG00000054453
ENSMUSG00000054455
ENSMUSG00000054459
ENSMUSG00000054469
ENSMUSG00000054477
ENSMUSG00000054484
ENSMUSG00000054499
ENSMUSG00000054509
ENSMUSG00000054517
ENSMUSG00000054604
ENSMUSG00000054611
ENSMUSG00000054619
ENSMUSG00000054640
ENSMUSG00000054648
ENSMUSG00000054667
ENSMUSG00000054675
ENSMUSG00000054676
ENSMUSG00000054679
ENSMUSG00000054693
ENSMUSG00000054708
ENSMUSG00000054715
ENSMUSG00000054716
ENSMUSG00000054717
ENSMUSG00000054720
ENSMUSG00000054723
ENSMUSG00000054728
ENSMUSG00000054733
ENSMUSG00000054752
ENSMUSG00000054766
ENSMUSG00000054770

ENSMUSG00000054792
ENSMUSG00000054793
ENSMUSG00000054808
ENSMUSG00000054814
ENSMUSG00000054823
ENSMUSG00000054836
ENSMUSG00000054843
ENSMUSG00000054850
ENSMUSG00000054855
ENSMUSG00000054863
ENSMUSG00000054871
ENSMUSG00000054874
ENSMUSG00000054893
ENSMUSG00000054894
ENSMUSG00000054920
ENSMUSG00000054931
ENSMUSG00000054934
ENSMUSG00000054939
ENSMUSG00000054942
ENSMUSG00000054976
ENSMUSG00000055003
ENSMUSG00000055013
ENSMUSG00000055022
ENSMUSG00000055024
ENSMUSG00000055026
ENSMUSG00000055041
ENSMUSG00000055053
ENSMUSG00000055065
ENSMUSG00000055067
ENSMUSG00000055078
ENSMUSG00000055116
ENSMUSG00000055128
ENSMUSG00000055202
ENSMUSG00000055204
ENSMUSG00000055214
ENSMUSG00000055239
ENSMUSG00000055240
ENSMUSG00000055254
ENSMUSG00000055296
ENSMUSG00000055302
ENSMUSG00000055305
ENSMUSG00000055319
ENSMUSG00000055320
ENSMUSG00000055322
ENSMUSG00000055334
ENSMUSG00000055371
ENSMUSG00000055373
ENSMUSG00000055401
ENSMUSG00000055407
ENSMUSG00000055409
ENSMUSG00000055421
ENSMUSG00000055430
ENSMUSG00000055435

ENSMUSG00000055436
ENSMUSG00000055447
ENSMUSG00000055471
ENSMUSG00000055480
ENSMUSG00000055485
ENSMUSG00000055491
ENSMUSG00000055493
ENSMUSG00000055531
ENSMUSG00000055538
ENSMUSG00000055540
ENSMUSG00000055553
ENSMUSG00000055567
ENSMUSG00000055612
ENSMUSG00000055629
ENSMUSG00000055633
ENSMUSG00000055639
ENSMUSG00000055652
ENSMUSG00000055653
ENSMUSG00000055660
ENSMUSG00000055670
ENSMUSG00000055675
ENSMUSG00000055681
ENSMUSG00000055692
ENSMUSG00000055707
ENSMUSG00000055717
ENSMUSG00000055720
ENSMUSG00000055723
ENSMUSG00000055725
ENSMUSG00000055733
ENSMUSG00000055737
ENSMUSG00000055745
ENSMUSG00000055761
ENSMUSG00000055762
ENSMUSG00000055782
ENSMUSG00000055805
ENSMUSG00000055809
ENSMUSG00000055817
ENSMUSG00000055835
ENSMUSG00000055839
ENSMUSG00000055850
ENSMUSG00000055862
ENSMUSG00000055866
ENSMUSG00000055884
ENSMUSG00000055897
ENSMUSG00000055900
ENSMUSG00000055917
ENSMUSG00000055923
ENSMUSG00000055932
ENSMUSG00000055943
ENSMUSG00000055945
ENSMUSG00000055963
ENSMUSG00000055980
ENSMUSG00000055991

ENSMUSG00000056004
ENSMUSG00000056014
ENSMUSG00000056019
ENSMUSG00000056050
ENSMUSG00000056069
ENSMUSG00000056073
ENSMUSG00000056076
ENSMUSG00000056091
ENSMUSG00000056121
ENSMUSG00000056124
ENSMUSG00000056131
ENSMUSG00000056145
ENSMUSG00000056153
ENSMUSG00000056158
ENSMUSG00000056167
ENSMUSG00000056174
ENSMUSG00000056185
ENSMUSG00000056201
ENSMUSG00000056204
ENSMUSG00000056211
ENSMUSG00000056214
ENSMUSG00000056215
ENSMUSG00000056216
ENSMUSG00000056222
ENSMUSG00000056228
ENSMUSG00000056234
ENSMUSG00000056258
ENSMUSG00000056260
ENSMUSG00000056267
ENSMUSG00000056268
ENSMUSG00000056296
ENSMUSG00000056305
ENSMUSG00000056306
ENSMUSG00000056310
ENSMUSG00000056342
ENSMUSG00000056367
ENSMUSG00000056383
ENSMUSG00000056394
ENSMUSG00000056413
ENSMUSG00000056427
ENSMUSG00000056429
ENSMUSG00000056458
ENSMUSG00000056459
ENSMUSG00000056476
ENSMUSG00000056486
ENSMUSG00000056492
ENSMUSG00000056493
ENSMUSG00000056515
ENSMUSG00000056536
ENSMUSG00000056537
ENSMUSG00000056553
ENSMUSG00000056579
ENSMUSG00000056592

ENSMUSG00000056596
ENSMUSG00000056598
ENSMUSG00000056602
ENSMUSG00000056608
ENSMUSG00000056612
ENSMUSG00000056629
ENSMUSG00000056665
ENSMUSG00000056666
ENSMUSG00000056679
ENSMUSG00000056692
ENSMUSG00000056698
ENSMUSG00000056708
ENSMUSG00000056724
ENSMUSG00000056749
ENSMUSG00000056752
ENSMUSG00000056755
ENSMUSG00000056763
ENSMUSG00000056770
ENSMUSG00000056812
ENSMUSG00000056820
ENSMUSG00000056832
ENSMUSG00000056851
ENSMUSG00000056854
ENSMUSG00000056856
ENSMUSG00000056870
ENSMUSG00000056895
ENSMUSG00000056900
ENSMUSG00000056917
ENSMUSG00000056919
ENSMUSG00000056938
ENSMUSG00000056941
ENSMUSG00000056952
ENSMUSG00000056962
ENSMUSG00000056966
ENSMUSG00000056972
ENSMUSG00000056999
ENSMUSG00000057060
ENSMUSG00000057069
ENSMUSG00000057093
ENSMUSG00000057098
ENSMUSG00000057101
ENSMUSG00000057103
ENSMUSG00000057110
ENSMUSG00000057113
ENSMUSG00000057130
ENSMUSG00000057133
ENSMUSG00000057134
ENSMUSG00000057147
ENSMUSG00000057156
ENSMUSG00000057176
ENSMUSG00000057177
ENSMUSG00000057181
ENSMUSG00000057182

ENSMUSG00000057193
ENSMUSG00000057219
ENSMUSG00000057229
ENSMUSG00000057230
ENSMUSG00000057236
ENSMUSG00000057278
ENSMUSG00000057315
ENSMUSG00000057322
ENSMUSG00000057329
ENSMUSG00000057335
ENSMUSG00000057337
ENSMUSG00000057342
ENSMUSG00000057359
ENSMUSG00000057363
ENSMUSG00000057367
ENSMUSG00000057375
ENSMUSG00000057378
ENSMUSG00000057388
ENSMUSG00000057396
ENSMUSG00000057406
ENSMUSG00000057409
ENSMUSG00000057411
ENSMUSG00000057421
ENSMUSG00000057440
ENSMUSG00000057455
ENSMUSG00000057469
ENSMUSG00000057497
ENSMUSG00000057506
ENSMUSG00000057522
ENSMUSG00000057530
ENSMUSG00000057531
ENSMUSG00000057541
ENSMUSG00000057551
ENSMUSG00000057554
ENSMUSG00000057561
ENSMUSG00000057572
ENSMUSG00000057594
ENSMUSG00000057614
ENSMUSG00000057637
ENSMUSG00000057649
ENSMUSG00000057666
ENSMUSG00000057667
ENSMUSG00000057672
ENSMUSG00000057691
ENSMUSG00000057706
ENSMUSG00000057715
ENSMUSG00000057716
ENSMUSG00000057738
ENSMUSG00000057751
ENSMUSG00000057766
ENSMUSG00000057778
ENSMUSG00000057788
ENSMUSG00000057789

ENSMUSG00000057841
ENSMUSG00000057858
ENSMUSG00000057863
ENSMUSG00000057880
ENSMUSG00000057894
ENSMUSG00000057895
ENSMUSG00000057897
ENSMUSG00000057914
ENSMUSG00000057963
ENSMUSG00000057982
ENSMUSG00000058006
ENSMUSG00000058013
ENSMUSG00000058056
ENSMUSG00000058070
ENSMUSG00000058076
ENSMUSG00000058135
ENSMUSG00000058145
ENSMUSG00000058152
ENSMUSG00000058153
ENSMUSG00000058192
ENSMUSG00000058230
ENSMUSG00000058239
ENSMUSG00000058240
ENSMUSG00000058248
ENSMUSG00000058254
ENSMUSG00000058258
ENSMUSG00000058260
ENSMUSG00000058267
ENSMUSG00000058291
ENSMUSG00000058297
ENSMUSG00000058298
ENSMUSG00000058301
ENSMUSG00000058317
ENSMUSG00000058318
ENSMUSG00000058325
ENSMUSG00000058355
ENSMUSG00000058385
ENSMUSG00000058388
ENSMUSG00000058392
ENSMUSG00000058402
ENSMUSG00000058407
ENSMUSG00000058420
ENSMUSG00000058440
ENSMUSG00000058441
ENSMUSG00000058444
ENSMUSG00000058446
ENSMUSG00000058454
ENSMUSG00000058486
ENSMUSG00000058488
ENSMUSG00000058503
ENSMUSG00000058546
ENSMUSG00000058558
ENSMUSG00000058569

ENSMUSG00000058571
ENSMUSG00000058587
ENSMUSG00000058589
ENSMUSG00000058594
ENSMUSG00000058600
ENSMUSG00000058620
ENSMUSG00000058624
ENSMUSG00000058638
ENSMUSG00000058655
ENSMUSG00000058656
ENSMUSG00000058672
ENSMUSG00000058690
ENSMUSG00000058704
ENSMUSG00000058706
ENSMUSG00000058709
ENSMUSG00000058740
ENSMUSG00000058748
ENSMUSG00000058756
ENSMUSG00000058761
ENSMUSG00000058793
ENSMUSG00000058799
ENSMUSG00000058833
ENSMUSG00000058835
ENSMUSG00000058881
ENSMUSG00000058886
ENSMUSG00000058897
ENSMUSG00000058966
ENSMUSG00000058975
ENSMUSG00000058979
ENSMUSG00000058997
ENSMUSG00000059003
ENSMUSG00000059005
ENSMUSG00000059013
ENSMUSG00000059040
ENSMUSG00000059070
ENSMUSG00000059119
ENSMUSG00000059142
ENSMUSG00000059146
ENSMUSG00000059149
ENSMUSG00000059173
ENSMUSG00000059182
ENSMUSG00000059183
ENSMUSG00000059187
ENSMUSG00000059203
ENSMUSG00000059208
ENSMUSG00000059213
ENSMUSG00000059248
ENSMUSG00000059263
ENSMUSG00000059273
ENSMUSG00000059277
ENSMUSG00000059278
ENSMUSG00000059288
ENSMUSG00000059291

ENSMUSG00000059316
ENSMUSG00000059323
ENSMUSG00000059325
ENSMUSG00000059326
ENSMUSG00000059327
ENSMUSG00000059336
ENSMUSG00000059355
ENSMUSG00000059361
ENSMUSG00000059363
ENSMUSG00000059401
ENSMUSG00000059409
ENSMUSG00000059423
ENSMUSG00000059436
ENSMUSG00000059439
ENSMUSG00000059447
ENSMUSG00000059456
ENSMUSG00000059474
ENSMUSG00000059475
ENSMUSG00000059482
ENSMUSG00000059486
ENSMUSG00000059493
ENSMUSG00000059495
ENSMUSG00000059518
ENSMUSG00000059534
ENSMUSG00000059540
ENSMUSG00000059552
ENSMUSG00000059554
ENSMUSG00000059586
ENSMUSG00000059602
ENSMUSG00000059669
ENSMUSG00000059674
ENSMUSG00000059689
ENSMUSG00000059708
ENSMUSG00000059713
ENSMUSG00000059714
ENSMUSG00000059734
ENSMUSG00000059742
ENSMUSG00000059743
ENSMUSG00000059772
ENSMUSG00000059796
ENSMUSG00000059810
ENSMUSG00000059811
ENSMUSG00000059820
ENSMUSG00000059824
ENSMUSG00000059834
ENSMUSG00000059839
ENSMUSG00000059842
ENSMUSG00000059851
ENSMUSG00000059852
ENSMUSG00000059854
ENSMUSG00000059857
ENSMUSG00000059866
ENSMUSG00000059878

ENSMUSG00000059890
ENSMUSG00000059895
ENSMUSG00000059897
ENSMUSG00000059920
ENSMUSG00000059921
ENSMUSG00000059923
ENSMUSG00000059939
ENSMUSG00000059970
ENSMUSG00000059974
ENSMUSG00000059975
ENSMUSG00000059981
ENSMUSG00000059991
ENSMUSG00000059995
ENSMUSG00000060002
ENSMUSG00000060012
ENSMUSG00000060032
ENSMUSG00000060036
ENSMUSG00000060038
ENSMUSG00000060073
ENSMUSG00000060090
ENSMUSG00000060093
ENSMUSG00000060098
ENSMUSG00000060121
ENSMUSG00000060126
ENSMUSG00000060143
ENSMUSG00000060147
ENSMUSG00000060152
ENSMUSG00000060166
ENSMUSG00000060176
ENSMUSG00000060181
ENSMUSG00000060206
ENSMUSG00000060212
ENSMUSG00000060216
ENSMUSG00000060227
ENSMUSG00000060240
ENSMUSG00000060257
ENSMUSG00000060260
ENSMUSG00000060261
ENSMUSG00000060275
ENSMUSG00000060279
ENSMUSG00000060301
ENSMUSG00000060314
ENSMUSG00000060336
ENSMUSG00000060371
ENSMUSG00000060373
ENSMUSG00000060376
ENSMUSG00000060380
ENSMUSG00000060397
ENSMUSG00000060424
ENSMUSG00000060427
ENSMUSG00000060429
ENSMUSG00000060450
ENSMUSG00000060475

ENSMUSG00000060477
ENSMUSG00000060487
ENSMUSG00000060510
ENSMUSG00000060519
ENSMUSG00000060530
ENSMUSG00000060534
ENSMUSG00000060538
ENSMUSG00000060548
ENSMUSG00000060568
ENSMUSG00000060601
ENSMUSG00000060636
ENSMUSG00000060639
ENSMUSG00000060657
ENSMUSG00000060671
ENSMUSG00000060675
ENSMUSG00000060678
ENSMUSG00000060679
ENSMUSG00000060681
ENSMUSG00000060703
ENSMUSG00000060708
ENSMUSG00000060716
ENSMUSG00000060733
ENSMUSG00000060735
ENSMUSG00000060739
ENSMUSG00000060743
ENSMUSG00000060771
ENSMUSG00000060780
ENSMUSG00000060798
ENSMUSG00000060802
ENSMUSG00000060803
ENSMUSG00000060860
ENSMUSG00000060862
ENSMUSG00000060882
ENSMUSG00000060904
ENSMUSG00000060924
ENSMUSG00000060935
ENSMUSG00000060938
ENSMUSG00000060950
ENSMUSG00000060961
ENSMUSG00000060981
ENSMUSG00000060988
ENSMUSG00000060992
ENSMUSG00000061024
ENSMUSG00000061028
ENSMUSG00000061032
ENSMUSG00000061046
ENSMUSG00000061080
ENSMUSG00000061111
ENSMUSG00000061118
ENSMUSG00000061119
ENSMUSG00000061130
ENSMUSG00000061136
ENSMUSG00000061143

ENSMUSG00000061175
ENSMUSG00000061186
ENSMUSG00000061207
ENSMUSG00000061232
ENSMUSG00000061244
ENSMUSG00000061273
ENSMUSG00000061286
ENSMUSG00000061288
ENSMUSG00000061298
ENSMUSG00000061306
ENSMUSG00000061313
ENSMUSG00000061315
ENSMUSG00000061322
ENSMUSG00000061353
ENSMUSG00000061360
ENSMUSG00000061374
ENSMUSG00000061393
ENSMUSG00000061410
ENSMUSG00000061411
ENSMUSG00000061414
ENSMUSG00000061436
ENSMUSG00000061451
ENSMUSG00000061455
ENSMUSG00000061458
ENSMUSG00000061461
ENSMUSG00000061462
ENSMUSG00000061474
ENSMUSG00000061477
ENSMUSG00000061479
ENSMUSG00000061482
ENSMUSG00000061517
ENSMUSG00000061518
ENSMUSG00000061524
ENSMUSG00000061533
ENSMUSG00000061536
ENSMUSG00000061559
ENSMUSG00000061576
ENSMUSG00000061578
ENSMUSG00000061589
ENSMUSG00000061601
ENSMUSG00000061603
ENSMUSG00000061607
ENSMUSG00000061613
ENSMUSG00000061650
ENSMUSG00000061654
ENSMUSG00000061665
ENSMUSG00000061666
ENSMUSG00000061689
ENSMUSG00000061702
ENSMUSG00000061718
ENSMUSG00000061724
ENSMUSG00000061731
ENSMUSG00000061740

ENSMUSG00000061751
ENSMUSG00000061755
ENSMUSG00000061758
ENSMUSG00000061759
ENSMUSG00000061762
ENSMUSG00000061778
ENSMUSG00000061787
ENSMUSG00000061838
ENSMUSG00000061859
ENSMUSG00000061887
ENSMUSG00000061894
ENSMUSG00000061898
ENSMUSG00000061904
ENSMUSG00000061911
ENSMUSG00000061950
ENSMUSG00000061979
ENSMUSG00000061981
ENSMUSG00000061983
ENSMUSG00000062006
ENSMUSG00000062012
ENSMUSG00000062014
ENSMUSG00000062031
ENSMUSG00000062040
ENSMUSG00000062044
ENSMUSG00000062054
ENSMUSG00000062070
ENSMUSG00000062075
ENSMUSG00000062078
ENSMUSG00000062098
ENSMUSG00000062110
ENSMUSG00000062115
ENSMUSG00000062116
ENSMUSG00000062127
ENSMUSG00000062151
ENSMUSG00000062169
ENSMUSG00000062184
ENSMUSG00000062190
ENSMUSG00000062202
ENSMUSG00000062203
ENSMUSG00000062209
ENSMUSG00000062232
ENSMUSG00000062234
ENSMUSG00000062252
ENSMUSG00000062257
ENSMUSG00000062270
ENSMUSG00000062296
ENSMUSG00000062300
ENSMUSG00000062309
ENSMUSG00000062312
ENSMUSG00000062328
ENSMUSG00000062352
ENSMUSG00000062353
ENSMUSG00000062372

ENSMUSG00000062373
ENSMUSG00000062376
ENSMUSG00000062380
ENSMUSG00000062393
ENSMUSG00000062397
ENSMUSG00000062421
ENSMUSG00000062444
ENSMUSG00000062480
ENSMUSG00000062519
ENSMUSG00000062526
ENSMUSG00000062542
ENSMUSG00000062580
ENSMUSG00000062590
ENSMUSG00000062591
ENSMUSG00000062604
ENSMUSG00000062627
ENSMUSG00000062646
ENSMUSG00000062647
ENSMUSG00000062661
ENSMUSG00000062683
ENSMUSG00000062691
ENSMUSG00000062729
ENSMUSG00000062753
ENSMUSG00000062760
ENSMUSG00000062761
ENSMUSG00000062762
ENSMUSG00000062785
ENSMUSG00000062797
ENSMUSG00000062822
ENSMUSG00000062825
ENSMUSG00000062861
ENSMUSG00000062866
ENSMUSG00000062867
ENSMUSG00000062901
ENSMUSG00000062906
ENSMUSG00000062908
ENSMUSG00000062929
ENSMUSG00000062931
ENSMUSG00000062937
ENSMUSG00000062944
ENSMUSG00000062949
ENSMUSG00000062960
ENSMUSG00000062961
ENSMUSG00000062963
ENSMUSG00000062981
ENSMUSG00000062991
ENSMUSG00000062995
ENSMUSG00000062997
ENSMUSG00000063015
ENSMUSG00000063018
ENSMUSG00000063019
ENSMUSG00000063021
ENSMUSG00000063047

ENSMUSG00000063049
ENSMUSG00000063052
ENSMUSG00000063063
ENSMUSG00000063065
ENSMUSG00000063077
ENSMUSG00000063087
ENSMUSG00000063108
ENSMUSG00000063142
ENSMUSG00000063145
ENSMUSG00000063146
ENSMUSG00000063160
ENSMUSG00000063171
ENSMUSG00000063179
ENSMUSG00000063200
ENSMUSG00000063229
ENSMUSG00000063235
ENSMUSG00000063236
ENSMUSG00000063239
ENSMUSG00000063253
ENSMUSG00000063260
ENSMUSG00000063273
ENSMUSG00000063281
ENSMUSG00000063296
ENSMUSG00000063297
ENSMUSG00000063316
ENSMUSG00000063317
ENSMUSG00000063334
ENSMUSG00000063358
ENSMUSG00000063362
ENSMUSG00000063382
ENSMUSG00000063406
ENSMUSG00000063410
ENSMUSG00000063415
ENSMUSG00000063428
ENSMUSG00000063430
ENSMUSG00000063434
ENSMUSG00000063439
ENSMUSG00000063445
ENSMUSG00000063446
ENSMUSG00000063450
ENSMUSG00000063455
ENSMUSG00000063457
ENSMUSG00000063480
ENSMUSG00000063488
ENSMUSG00000063506
ENSMUSG00000063511
ENSMUSG00000063524
ENSMUSG00000063531
ENSMUSG00000063550
ENSMUSG00000063564
ENSMUSG00000063568
ENSMUSG00000063576
ENSMUSG00000063600

ENSMUSG00000063626
ENSMUSG00000063632
ENSMUSG00000063646
ENSMUSG00000063659
ENSMUSG00000063663
ENSMUSG00000063681
ENSMUSG00000063687
ENSMUSG00000063694
ENSMUSG00000063698
ENSMUSG00000063704
ENSMUSG00000063714
ENSMUSG00000063760
ENSMUSG00000063765
ENSMUSG00000063785
ENSMUSG00000063787
ENSMUSG00000063796
ENSMUSG00000063800
ENSMUSG00000063801
ENSMUSG00000063802
ENSMUSG00000063804
ENSMUSG00000063808
ENSMUSG00000063810
ENSMUSG00000063849
ENSMUSG00000063856
ENSMUSG00000063870
ENSMUSG00000063873
ENSMUSG00000063882
ENSMUSG00000063884
ENSMUSG00000063887
ENSMUSG00000063888
ENSMUSG00000063889
ENSMUSG00000063894
ENSMUSG00000063897
ENSMUSG00000063902
ENSMUSG00000063904
ENSMUSG00000063919
ENSMUSG00000063931
ENSMUSG00000063952
ENSMUSG00000063972
ENSMUSG00000064030
ENSMUSG00000064036
ENSMUSG00000064037
ENSMUSG00000064043
ENSMUSG00000064061
ENSMUSG00000064065
ENSMUSG00000064068
ENSMUSG00000064080
ENSMUSG00000064105
ENSMUSG00000064115
ENSMUSG00000064120
ENSMUSG00000064125
ENSMUSG00000064127
ENSMUSG00000064138

ENSMUSG00000064145
ENSMUSG00000064181
ENSMUSG00000064193
ENSMUSG00000064210
ENSMUSG00000064215
ENSMUSG00000064220
ENSMUSG00000064225
ENSMUSG00000064246
ENSMUSG00000064247
ENSMUSG00000064254
ENSMUSG00000064264
ENSMUSG00000064284
ENSMUSG00000064288
ENSMUSG00000064289
ENSMUSG00000064293
ENSMUSG00000064302
ENSMUSG00000064326
ENSMUSG00000064329
ENSMUSG00000064373
ENSMUSG00000064380
ENSMUSG00000064382
ENSMUSG00000064387
ENSMUSG00000064451
ENSMUSG00000064702
ENSMUSG00000064853
ENSMUSG00000064856
ENSMUSG00000064945
ENSMUSG00000064966
ENSMUSG00000065037
ENSMUSG00000065087
ENSMUSG00000065176
ENSMUSG00000065251
ENSMUSG00000065353
ENSMUSG00000065649
ENSMUSG00000065701
ENSMUSG00000065767
ENSMUSG00000065820
ENSMUSG00000065944
ENSMUSG00000065954
ENSMUSG00000065979
ENSMUSG00000065990
ENSMUSG00000066026
ENSMUSG00000066036
ENSMUSG00000066037
ENSMUSG00000066042
ENSMUSG00000066043
ENSMUSG00000066057
ENSMUSG00000066113
ENSMUSG00000066129
ENSMUSG00000066148
ENSMUSG00000066149
ENSMUSG00000066150
ENSMUSG00000066151

ENSMUSG00000066152
ENSMUSG00000066189
ENSMUSG00000066191
ENSMUSG00000066232
ENSMUSG00000066235
ENSMUSG00000066278
ENSMUSG00000066306
ENSMUSG00000066324
ENSMUSG00000066357
ENSMUSG00000066392
ENSMUSG00000066406
ENSMUSG00000066415
ENSMUSG00000066438
ENSMUSG00000066440
ENSMUSG00000066441
ENSMUSG00000066456
ENSMUSG00000066510
ENSMUSG00000066551
ENSMUSG00000066553
ENSMUSG00000066568
ENSMUSG00000066571
ENSMUSG00000066595
ENSMUSG00000066607
ENSMUSG00000066613
ENSMUSG00000066621
ENSMUSG00000066640
ENSMUSG00000066643
ENSMUSG00000066647
ENSMUSG00000066687
ENSMUSG00000066705
ENSMUSG00000066721
ENSMUSG00000066735
ENSMUSG00000066798
ENSMUSG00000066829
ENSMUSG00000066839
ENSMUSG00000066842
ENSMUSG00000066877
ENSMUSG00000066880
ENSMUSG00000066892
ENSMUSG00000066900
ENSMUSG00000066979
ENSMUSG00000067028
ENSMUSG00000067071
ENSMUSG00000067148
ENSMUSG00000067150
ENSMUSG00000067194
ENSMUSG00000067199
ENSMUSG00000067215
ENSMUSG00000067242
ENSMUSG00000067274
ENSMUSG00000067279
ENSMUSG00000067288
ENSMUSG00000067336

ENSMUSG00000067365
ENSMUSG00000067369
ENSMUSG00000067377
ENSMUSG00000067424
ENSMUSG00000067430
ENSMUSG00000067455
ENSMUSG00000067567
ENSMUSG00000067578
ENSMUSG00000067586
ENSMUSG00000067594
ENSMUSG00000067629
ENSMUSG00000067713
ENSMUSG00000067722
ENSMUSG00000067786
ENSMUSG00000067787
ENSMUSG00000067818
ENSMUSG00000067825
ENSMUSG00000067847
ENSMUSG00000067851
ENSMUSG00000067860
ENSMUSG00000067873
ENSMUSG00000067879
ENSMUSG00000067889
ENSMUSG00000067924
ENSMUSG00000067925
ENSMUSG00000067928
ENSMUSG00000067931
ENSMUSG00000067942
ENSMUSG00000067995
ENSMUSG00000068015
ENSMUSG00000068036
ENSMUSG00000068039
ENSMUSG00000068040
ENSMUSG00000068099
ENSMUSG00000068114
ENSMUSG00000068134
ENSMUSG00000068154
ENSMUSG00000068205
ENSMUSG00000068206
ENSMUSG00000068250
ENSMUSG00000068263
ENSMUSG00000068264
ENSMUSG00000068267
ENSMUSG00000068270
ENSMUSG00000068284
ENSMUSG00000068290
ENSMUSG00000068299
ENSMUSG00000068328
ENSMUSG00000068329
ENSMUSG00000068373
ENSMUSG00000068391
ENSMUSG00000068394
ENSMUSG00000068396

ENSMUSG00000068479
ENSMUSG00000068523
ENSMUSG00000068551
ENSMUSG00000068566
ENSMUSG00000068580
ENSMUSG00000068617
ENSMUSG00000068663
ENSMUSG00000068696
ENSMUSG00000068732
ENSMUSG00000068735
ENSMUSG00000068739
ENSMUSG00000068740
ENSMUSG00000068742
ENSMUSG00000068747
ENSMUSG00000068748
ENSMUSG00000068749
ENSMUSG00000068798
ENSMUSG00000068823
ENSMUSG00000068854
ENSMUSG00000068855
ENSMUSG00000068856
ENSMUSG00000068859
ENSMUSG00000068874
ENSMUSG00000068876
ENSMUSG00000068882
ENSMUSG00000068917
ENSMUSG00000068921
ENSMUSG00000068922
ENSMUSG00000068923
ENSMUSG00000068959
ENSMUSG00000068966
ENSMUSG00000069020
ENSMUSG00000069072
ENSMUSG00000069089
ENSMUSG00000069094
ENSMUSG00000069114
ENSMUSG00000069117
ENSMUSG00000069135
ENSMUSG00000069170
ENSMUSG00000069171
ENSMUSG00000069206
ENSMUSG00000069227
ENSMUSG00000069237
ENSMUSG00000069255
ENSMUSG00000069266
ENSMUSG00000069273
ENSMUSG00000069274
ENSMUSG00000069302
ENSMUSG00000069305
ENSMUSG00000069306
ENSMUSG00000069307
ENSMUSG00000069495
ENSMUSG00000069520

ENSMUSG00000069539
ENSMUSG00000069565
ENSMUSG00000069601
ENSMUSG00000069631
ENSMUSG00000069662
ENSMUSG00000069670
ENSMUSG00000069678
ENSMUSG00000069729
ENSMUSG00000069744
ENSMUSG00000069763
ENSMUSG00000069769
ENSMUSG00000069806
ENSMUSG00000069808
ENSMUSG00000069814
ENSMUSG00000069833
ENSMUSG00000069844
ENSMUSG00000069895
ENSMUSG00000069911
ENSMUSG00000069917
ENSMUSG00000069919
ENSMUSG00000070000
ENSMUSG00000070002
ENSMUSG00000070003
ENSMUSG00000070044
ENSMUSG00000070047
ENSMUSG00000070056
ENSMUSG00000070283
ENSMUSG00000070284
ENSMUSG00000070287
ENSMUSG00000070304
ENSMUSG00000070306
ENSMUSG00000070319
ENSMUSG00000070327
ENSMUSG00000070348
ENSMUSG00000070372
ENSMUSG00000070392
ENSMUSG00000070394
ENSMUSG00000070420
ENSMUSG00000070426
ENSMUSG00000070436
ENSMUSG00000070461
ENSMUSG00000070462
ENSMUSG00000070476
ENSMUSG00000070493
ENSMUSG00000070498
ENSMUSG00000070509
ENSMUSG00000070520
ENSMUSG00000070532
ENSMUSG00000070544
ENSMUSG00000070565
ENSMUSG00000070570
ENSMUSG00000070576
ENSMUSG00000070583

ENSMUSG00000070639
ENSMUSG00000070643
ENSMUSG00000070644
ENSMUSG00000070695
ENSMUSG00000070697
ENSMUSG00000070699
ENSMUSG00000070705
ENSMUSG00000070709
ENSMUSG00000070713
ENSMUSG00000070730
ENSMUSG00000070733
ENSMUSG00000070738
ENSMUSG00000070802
ENSMUSG00000070808
ENSMUSG00000070814
ENSMUSG00000070822
ENSMUSG00000070858
ENSMUSG00000070866
ENSMUSG00000070871
ENSMUSG00000070880
ENSMUSG00000070923
ENSMUSG00000070934
ENSMUSG00000070939
ENSMUSG00000070953
ENSMUSG00000071014
ENSMUSG00000071037
ENSMUSG00000071042
ENSMUSG00000071054
ENSMUSG00000071064
ENSMUSG00000071072
ENSMUSG00000071073
ENSMUSG00000071074
ENSMUSG00000071076
ENSMUSG00000071078
ENSMUSG00000071172
ENSMUSG00000071176
ENSMUSG00000071180
ENSMUSG00000071226
ENSMUSG00000071234
ENSMUSG00000071252
ENSMUSG00000071253
ENSMUSG00000071256
ENSMUSG00000071265
ENSMUSG00000071266
ENSMUSG00000071267
ENSMUSG00000071281
ENSMUSG00000071302
ENSMUSG00000071324
ENSMUSG00000071337
ENSMUSG00000071341
ENSMUSG00000071359
ENSMUSG00000071369
ENSMUSG00000071379

ENSMUSG00000071398
ENSMUSG00000071415
ENSMUSG00000071424
ENSMUSG00000071454
ENSMUSG00000071456
ENSMUSG00000071477
ENSMUSG00000071516
ENSMUSG00000071528
ENSMUSG00000071531
ENSMUSG00000071533
ENSMUSG00000071547
ENSMUSG00000071550
ENSMUSG00000071604
ENSMUSG00000071632
ENSMUSG00000071636
ENSMUSG00000071644
ENSMUSG00000071645
ENSMUSG00000071646
ENSMUSG00000071647
ENSMUSG00000071648
ENSMUSG00000071649
ENSMUSG00000071650
ENSMUSG00000071652
ENSMUSG00000071654
ENSMUSG00000071655
ENSMUSG00000071657
ENSMUSG00000071658
ENSMUSG00000071659
ENSMUSG00000071660
ENSMUSG00000071662
ENSMUSG00000071669
ENSMUSG00000071708
ENSMUSG00000071711
ENSMUSG00000071719
ENSMUSG00000071723
ENSMUSG00000071753
ENSMUSG00000071757
ENSMUSG00000071793
ENSMUSG00000071796
ENSMUSG00000071847
ENSMUSG00000071855
ENSMUSG00000071856
ENSMUSG00000071862
ENSMUSG00000071866
ENSMUSG00000071984
ENSMUSG00000072214
ENSMUSG00000072235
ENSMUSG00000072258
ENSMUSG00000072294
ENSMUSG00000072437
ENSMUSG00000072494
ENSMUSG00000072501
ENSMUSG00000072568

ENSMUSG00000072582
ENSMUSG00000072623
ENSMUSG00000072640
ENSMUSG00000072647
ENSMUSG00000072653
ENSMUSG00000072663
ENSMUSG00000072674
ENSMUSG00000072694
ENSMUSG00000072704
ENSMUSG00000072769
ENSMUSG00000072772
ENSMUSG00000072825
ENSMUSG00000072872
ENSMUSG00000072875
ENSMUSG00000072889
ENSMUSG00000072893
ENSMUSG00000072915
ENSMUSG00000072941
ENSMUSG00000072946
ENSMUSG00000072949
ENSMUSG00000072964
ENSMUSG00000072966
ENSMUSG00000072969
ENSMUSG00000073016
ENSMUSG00000073060
ENSMUSG00000073062
ENSMUSG00000073079
ENSMUSG00000073096
ENSMUSG00000073102
ENSMUSG00000073131
ENSMUSG00000073139
ENSMUSG00000073147
ENSMUSG00000073155
ENSMUSG00000073176
ENSMUSG00000073274
ENSMUSG00000073293
ENSMUSG00000073295
ENSMUSG00000073411
ENSMUSG00000073418
ENSMUSG00000073422
ENSMUSG00000073423
ENSMUSG00000073424
ENSMUSG00000073427
ENSMUSG00000073433
ENSMUSG00000073434
ENSMUSG00000073435
ENSMUSG00000073436
ENSMUSG00000073468
ENSMUSG00000073471
ENSMUSG00000073481
ENSMUSG00000073514
ENSMUSG00000073542
ENSMUSG00000073557

ENSMUSG00000073563
ENSMUSG00000073565
ENSMUSG00000073591
ENSMUSG00000073600
ENSMUSG00000073609
ENSMUSG00000073616
ENSMUSG00000073639
ENSMUSG00000073643
ENSMUSG00000073664
ENSMUSG00000073676
ENSMUSG00000073678
ENSMUSG00000073680
ENSMUSG00000073684
ENSMUSG00000073700
ENSMUSG00000073702
ENSMUSG00000073725
ENSMUSG00000073755
ENSMUSG00000073771
ENSMUSG00000073775
ENSMUSG00000073779
ENSMUSG00000073792
ENSMUSG00000073805
ENSMUSG00000073838
ENSMUSG00000073856
ENSMUSG00000073888
ENSMUSG00000073889
ENSMUSG00000073910
ENSMUSG00000073940
ENSMUSG00000073968
ENSMUSG00000073982
ENSMUSG00000073987
ENSMUSG00000074024
ENSMUSG00000074030
ENSMUSG00000074064
ENSMUSG00000074088
ENSMUSG00000074093
ENSMUSG00000074102
ENSMUSG00000074129
ENSMUSG00000074165
ENSMUSG00000074182
ENSMUSG00000074211
ENSMUSG00000074212
ENSMUSG00000074220
ENSMUSG00000074221
ENSMUSG00000074227
ENSMUSG00000074238
ENSMUSG00000074247
ENSMUSG00000074259
ENSMUSG00000074264
ENSMUSG00000074282
ENSMUSG00000074283
ENSMUSG00000074305
ENSMUSG00000074345

ENSMUSG00000074358
ENSMUSG00000074364
ENSMUSG00000074403
ENSMUSG00000074405
ENSMUSG00000074406
ENSMUSG00000074415
ENSMUSG00000074457
ENSMUSG00000074461
ENSMUSG00000074480
ENSMUSG00000074505
ENSMUSG00000074513
ENSMUSG00000074517
ENSMUSG00000074519
ENSMUSG00000074527
ENSMUSG00000074575
ENSMUSG00000074576
ENSMUSG00000074578
ENSMUSG00000074582
ENSMUSG00000074607
ENSMUSG00000074622
ENSMUSG00000074634
ENSMUSG00000074637
ENSMUSG00000074643
ENSMUSG00000074649
ENSMUSG00000074656
ENSMUSG00000074657
ENSMUSG00000074664
ENSMUSG00000074671
ENSMUSG00000074682
ENSMUSG00000074698
ENSMUSG00000074733
ENSMUSG00000074736
ENSMUSG00000074738
ENSMUSG00000074746
ENSMUSG00000074748
ENSMUSG00000074749
ENSMUSG00000074754
ENSMUSG00000074771
ENSMUSG00000074781
ENSMUSG00000074785
ENSMUSG00000074793
ENSMUSG00000074794
ENSMUSG00000074797
ENSMUSG00000074811
ENSMUSG00000074872
ENSMUSG00000074876
ENSMUSG00000074884
ENSMUSG00000074886
ENSMUSG00000074890
ENSMUSG00000074892
ENSMUSG00000074909
ENSMUSG00000074918
ENSMUSG00000074923

ENSMUSG00000074925
ENSMUSG00000074968
ENSMUSG00000074994
ENSMUSG00000075000
ENSMUSG00000075012
ENSMUSG00000075028
ENSMUSG00000075031
ENSMUSG00000075033
ENSMUSG00000075040
ENSMUSG00000075054
ENSMUSG00000075224
ENSMUSG00000075225
ENSMUSG00000075227
ENSMUSG00000075232
ENSMUSG00000075254
ENSMUSG00000075270
ENSMUSG00000075271
ENSMUSG00000075273
ENSMUSG00000075284
ENSMUSG00000075289
ENSMUSG00000075296
ENSMUSG00000075316
ENSMUSG00000075318
ENSMUSG00000075327
ENSMUSG00000075334
ENSMUSG00000075376
ENSMUSG00000075389
ENSMUSG00000075415
ENSMUSG00000075419
ENSMUSG00000075467
ENSMUSG00000075470
ENSMUSG00000075478
ENSMUSG00000075486
ENSMUSG00000075514
ENSMUSG00000075528
ENSMUSG00000075569
ENSMUSG00000075585
ENSMUSG00000075590
ENSMUSG00000075592
ENSMUSG00000075595
ENSMUSG00000075600
ENSMUSG00000075602
ENSMUSG00000075700
ENSMUSG00000075701
ENSMUSG00000075702
ENSMUSG00000075703
ENSMUSG00000075704
ENSMUSG00000075705
ENSMUSG00000075706
ENSMUSG00000076431
ENSMUSG00000076432
ENSMUSG00000076435
ENSMUSG00000076437

ENSMUSG00000076439
ENSMUSG00000076441
ENSMUSG00000077167
ENSMUSG00000077254
ENSMUSG00000077450
ENSMUSG00000077714
ENSMUSG00000078117
ENSMUSG00000078137
ENSMUSG00000078161
ENSMUSG00000078185
ENSMUSG00000078201
ENSMUSG00000078202
ENSMUSG00000078234
ENSMUSG00000078235
ENSMUSG00000078307
ENSMUSG00000078317
ENSMUSG00000078348
ENSMUSG00000078349
ENSMUSG00000078350
ENSMUSG00000078427
ENSMUSG00000078429
ENSMUSG00000078440
ENSMUSG00000078485
ENSMUSG00000078490
ENSMUSG00000078515
ENSMUSG00000078517
ENSMUSG00000078532
ENSMUSG00000078566
ENSMUSG00000078570
ENSMUSG00000078572
ENSMUSG00000078578
ENSMUSG00000078580
ENSMUSG00000078584
ENSMUSG00000078591
ENSMUSG00000078619
ENSMUSG00000078622
ENSMUSG00000078636
ENSMUSG00000078652
ENSMUSG00000078656
ENSMUSG00000078670
ENSMUSG00000078671
ENSMUSG00000078676
ENSMUSG00000078681
ENSMUSG00000078684
ENSMUSG00000078695
ENSMUSG00000078713
ENSMUSG00000078716
ENSMUSG00000078747
ENSMUSG00000078765
ENSMUSG00000078771
ENSMUSG00000078779
ENSMUSG00000078786
ENSMUSG00000078789

ENSMUSG00000078794
ENSMUSG00000078812
ENSMUSG00000078813
ENSMUSG00000078816
ENSMUSG00000078851
ENSMUSG00000078862
ENSMUSG00000078870
ENSMUSG00000078878
ENSMUSG00000078879
ENSMUSG00000078897
ENSMUSG00000078899
ENSMUSG00000078903
ENSMUSG00000078908
ENSMUSG00000078919
ENSMUSG00000078923
ENSMUSG00000078931
ENSMUSG00000078941
ENSMUSG00000078958
ENSMUSG00000078970
ENSMUSG00000078974
ENSMUSG00000079003
ENSMUSG00000079018
ENSMUSG00000079020
ENSMUSG00000079036
ENSMUSG00000079037
ENSMUSG00000079038
ENSMUSG00000079043
ENSMUSG00000079055
ENSMUSG00000079056
ENSMUSG00000079057
ENSMUSG00000079065
ENSMUSG00000079083
ENSMUSG00000079084
ENSMUSG00000079104
ENSMUSG00000079108
ENSMUSG00000079109
ENSMUSG00000079111
ENSMUSG00000079144
ENSMUSG00000079157
ENSMUSG00000079179
ENSMUSG00000079184
ENSMUSG00000079197
ENSMUSG00000079215
ENSMUSG00000079235
ENSMUSG00000079260
ENSMUSG00000079283
ENSMUSG00000079316
ENSMUSG00000079317
ENSMUSG00000079334
ENSMUSG00000079426
ENSMUSG00000079427
ENSMUSG00000079435
ENSMUSG00000079442

ENSMUSG00000079469
ENSMUSG00000079470
ENSMUSG00000079477
ENSMUSG00000079478
ENSMUSG00000079481
ENSMUSG00000079484
ENSMUSG00000079487
ENSMUSG00000079508
ENSMUSG00000079509
ENSMUSG00000079523
ENSMUSG00000079557
ENSMUSG00000079562
ENSMUSG00000079592
ENSMUSG00000079598
ENSMUSG00000079605
ENSMUSG00000079610
ENSMUSG00000079614
ENSMUSG00000079641
ENSMUSG00000079654
ENSMUSG00000079657
ENSMUSG00000079658
ENSMUSG00000079677
ENSMUSG00000079710
ENSMUSG00000080058
ENSMUSG00000080242
ENSMUSG00000080268
ENSMUSG00000080316
ENSMUSG00000080365
ENSMUSG00000080465
ENSMUSG00000080717
ENSMUSG00000081058
ENSMUSG00000081272
ENSMUSG00000081534
ENSMUSG00000081788
ENSMUSG00000082229
ENSMUSG00000083282
ENSMUSG00000083307
ENSMUSG00000083327
ENSMUSG00000083380
ENSMUSG00000083405
ENSMUSG00000083679
ENSMUSG00000083773
ENSMUSG00000083844
ENSMUSG00000084349
ENSMUSG00000084530
ENSMUSG00000084708
ENSMUSG00000084786
ENSMUSG00000084842
ENSMUSG00000084845
ENSMUSG00000084880
ENSMUSG00000084883
ENSMUSG00000084910
ENSMUSG00000084957

ENSMUSG00000084989
ENSMUSG00000085037
ENSMUSG00000085069
ENSMUSG00000085084
ENSMUSG00000085133
ENSMUSG00000085148
ENSMUSG00000085208
ENSMUSG00000085282
ENSMUSG00000085334
ENSMUSG00000085385
ENSMUSG00000085396
ENSMUSG00000085408
ENSMUSG00000085438
ENSMUSG00000085492
ENSMUSG00000085566
ENSMUSG00000085586
ENSMUSG00000085741
ENSMUSG00000085793
ENSMUSG00000085795
ENSMUSG00000085875
ENSMUSG00000085925
ENSMUSG00000086040
ENSMUSG00000086119
ENSMUSG00000086158
ENSMUSG00000086290
ENSMUSG00000086316
ENSMUSG00000086370
ENSMUSG00000086448
ENSMUSG00000086496
ENSMUSG00000086502
ENSMUSG00000086583
ENSMUSG00000086596
ENSMUSG00000086784
ENSMUSG00000086841
ENSMUSG00000086859
ENSMUSG00000086968
ENSMUSG00000087006
ENSMUSG00000087026
ENSMUSG00000087075
ENSMUSG00000087088
ENSMUSG00000087141
ENSMUSG00000087143
ENSMUSG00000087174
ENSMUSG00000087177
ENSMUSG00000087259
ENSMUSG00000087260
ENSMUSG00000087370
ENSMUSG00000087380
ENSMUSG00000087385
ENSMUSG00000087396
ENSMUSG00000087403
ENSMUSG00000087408
ENSMUSG00000087497

ENSMUSG00000087574
ENSMUSG00000087598
ENSMUSG00000087620
ENSMUSG00000087651
ENSMUSG00000087679
ENSMUSG00000087687
ENSMUSG00000087968
ENSMUSG00000088025
ENSMUSG00000088088
ENSMUSG00000088185
ENSMUSG00000088529
ENSMUSG00000088789
ENSMUSG00000088835
ENSMUSG00000088948
ENSMUSG00000089281
ENSMUSG00000089417
ENSMUSG00000089542
ENSMUSG00000089617
ENSMUSG00000089661
ENSMUSG00000089682
ENSMUSG00000089704
ENSMUSG00000089715
ENSMUSG00000089739
ENSMUSG00000089762
ENSMUSG00000089774
ENSMUSG00000089809
ENSMUSG00000089810
ENSMUSG00000089824
ENSMUSG00000089832
ENSMUSG00000089847
ENSMUSG00000089857
ENSMUSG00000089862
ENSMUSG00000089872
ENSMUSG00000089911
ENSMUSG00000089917
ENSMUSG00000089922
ENSMUSG00000089945
ENSMUSG00000089997
ENSMUSG00000090000
ENSMUSG00000090015
ENSMUSG00000090031
ENSMUSG00000090053
ENSMUSG00000090061
ENSMUSG00000090063
ENSMUSG00000090069
ENSMUSG00000090071
ENSMUSG00000090077
ENSMUSG00000090083
ENSMUSG00000090086
ENSMUSG00000090100
ENSMUSG00000090112
ENSMUSG00000090115
ENSMUSG00000090122

ENSMUSG00000090125
ENSMUSG00000090137
ENSMUSG00000090150
ENSMUSG00000090213
ENSMUSG00000090223
ENSMUSG00000090247
ENSMUSG00000090258
ENSMUSG00000090266
ENSMUSG00000090290
ENSMUSG00000090291
ENSMUSG00000090330
ENSMUSG00000090386
ENSMUSG00000090553
ENSMUSG00000090626
ENSMUSG00000090671
ENSMUSG00000090733
ENSMUSG00000090799
ENSMUSG00000090841
ENSMUSG00000090862
ENSMUSG00000090877
ENSMUSG00000090935
ENSMUSG00000090946
ENSMUSG00000091002
ENSMUSG00000091228
ENSMUSG00000091264
ENSMUSG00000091337
ENSMUSG00000091345
ENSMUSG00000091405
ENSMUSG00000091415
ENSMUSG00000091474
ENSMUSG00000091475
ENSMUSG00000091476
ENSMUSG00000091512
ENSMUSG00000091537
ENSMUSG00000091542
ENSMUSG00000091586
ENSMUSG00000091712
ENSMUSG00000091722
ENSMUSG00000091735
ENSMUSG00000091780
ENSMUSG00000091803
ENSMUSG00000091811
ENSMUSG00000091890
ENSMUSG00000091896
ENSMUSG00000091957
ENSMUSG00000091971
ENSMUSG00000091994
ENSMUSG00000092035
ENSMUSG00000092060
ENSMUSG00000092074
ENSMUSG00000092083
ENSMUSG00000092274
ENSMUSG00000092329

ENSMUSG00000092341
ENSMUSG00000092416
ENSMUSG00000092417
ENSMUSG00000092558
ENSMUSG00000092607
ENSMUSG00000092746
ENSMUSG00000092805
ENSMUSG00000092837
ENSMUSG00000093445
ENSMUSG00000093452
ENSMUSG00000093460
ENSMUSG00000093536
ENSMUSG00000093593
ENSMUSG00000093661
ENSMUSG00000093674
ENSMUSG00000093752
ENSMUSG00000093803
ENSMUSG00000093815
ENSMUSG00000093843
ENSMUSG00000093880
ENSMUSG00000093904
ENSMUSG00000093910
ENSMUSG00000093930
ENSMUSG00000093989
ENSMUSG00000094002
ENSMUSG00000094050
ENSMUSG00000094377
ENSMUSG00000094410
ENSMUSG00000094441
ENSMUSG00000094483
ENSMUSG00000094626
ENSMUSG00000094627
ENSMUSG00000094655
ENSMUSG00000094724
ENSMUSG00000094777
ENSMUSG00000094826
ENSMUSG00000094870
ENSMUSG00000094910
ENSMUSG00000094936
ENSMUSG00000095041
ENSMUSG00000095098
ENSMUSG00000095123
ENSMUSG00000095139
ENSMUSG00000095199
ENSMUSG00000095253
ENSMUSG00000095325
ENSMUSG00000095334
ENSMUSG00000095362
ENSMUSG00000095407
ENSMUSG00000095432
ENSMUSG00000095440
ENSMUSG00000095463
ENSMUSG00000095526

ENSMUSG00000095567
ENSMUSG00000095597
ENSMUSG00000095676
ENSMUSG00000095677
ENSMUSG00000095687
ENSMUSG00000095930
ENSMUSG00000095969
ENSMUSG00000096010
ENSMUSG00000096014
ENSMUSG00000096054
ENSMUSG00000096141
ENSMUSG00000096145
ENSMUSG00000096146
ENSMUSG00000096188
ENSMUSG00000096199
ENSMUSG00000096205
ENSMUSG00000096206
ENSMUSG00000096210
ENSMUSG00000096214
ENSMUSG00000096225
ENSMUSG00000096243
ENSMUSG00000096370
ENSMUSG00000096403
ENSMUSG00000096433
ENSMUSG00000096458
ENSMUSG00000096472
ENSMUSG00000096606
ENSMUSG00000096659
ENSMUSG00000096687
ENSMUSG00000096718
ENSMUSG00000096768
ENSMUSG00000096780
ENSMUSG00000096847
ENSMUSG00000096856
ENSMUSG00000096910
ENSMUSG00000096914
ENSMUSG00000096929
ENSMUSG00000097023
ENSMUSG00000097039
ENSMUSG00000097048
ENSMUSG00000097061
ENSMUSG00000097119
ENSMUSG00000097141
ENSMUSG00000097156
ENSMUSG00000097164
ENSMUSG00000097195
ENSMUSG00000097216
ENSMUSG00000097230
ENSMUSG00000097287
ENSMUSG00000097311
ENSMUSG00000097320
ENSMUSG00000097328
ENSMUSG00000097333

ENSMUSG00000097375
ENSMUSG00000097391
ENSMUSG00000097392
ENSMUSG00000097428
ENSMUSG00000097440
ENSMUSG00000097451
ENSMUSG00000097545
ENSMUSG00000097571
ENSMUSG00000097583
ENSMUSG00000097589
ENSMUSG00000097649
ENSMUSG00000097715
ENSMUSG00000097729
ENSMUSG00000097736
ENSMUSG00000097750
ENSMUSG00000097767
ENSMUSG00000097772
ENSMUSG00000097785
ENSMUSG00000097787
ENSMUSG00000097789
ENSMUSG00000097820
ENSMUSG00000097908
ENSMUSG00000097929
ENSMUSG00000097960
ENSMUSG00000097961
ENSMUSG00000097971
ENSMUSG00000097994
ENSMUSG00000098051
ENSMUSG00000098132
ENSMUSG00000098134
ENSMUSG00000098176
ENSMUSG00000098178
ENSMUSG00000098188
ENSMUSG00000098234
ENSMUSG00000098274
ENSMUSG00000098332
ENSMUSG00000098374
ENSMUSG00000098557
ENSMUSG00000098641
ENSMUSG00000098702
ENSMUSG00000098923
ENSMUSG00000098925
ENSMUSG00000099021
ENSMUSG00000099041
ENSMUSG00000099083
ENSMUSG00000099250
ENSMUSG00000099481
ENSMUSG00000099583
ENSMUSG00000099681
ENSMUSG00000099689
ENSMUSG00000099696
ENSMUSG00000099881
ENSMUSG00000100017

ENSMUSG00000100131
ENSMUSG00000100252
ENSMUSG00000100455
ENSMUSG00000100691
ENSMUSG00000100801
ENSMUSG00000100826
ENSMUSG00000100862
ENSMUSG00000101013
ENSMUSG00000101111
ENSMUSG00000101355
ENSMUSG00000101599
ENSMUSG00000101609
ENSMUSG00000101892
ENSMUSG00000101970
ENSMUSG00000101972
ENSMUSG00000102070
ENSMUSG00000102101
ENSMUSG00000102206
ENSMUSG00000102222
ENSMUSG00000102252
ENSMUSG00000102312
ENSMUSG00000102317
ENSMUSG00000102336
ENSMUSG00000102428
ENSMUSG00000102440
ENSMUSG00000102465
ENSMUSG00000102533
ENSMUSG00000102543
ENSMUSG00000102555
ENSMUSG00000102602
ENSMUSG00000102627
ENSMUSG00000102657
ENSMUSG00000102659
ENSMUSG00000102692
ENSMUSG00000102697
ENSMUSG00000102742
ENSMUSG00000102747
ENSMUSG00000102748
ENSMUSG00000102752
ENSMUSG00000102869
ENSMUSG00000102918
ENSMUSG00000102976
ENSMUSG00000103037
ENSMUSG00000103041
ENSMUSG00000103088
ENSMUSG00000103092
ENSMUSG00000103103
ENSMUSG00000103144
ENSMUSG00000103214
ENSMUSG00000103310
ENSMUSG00000103332
ENSMUSG00000103472
ENSMUSG00000103509

ENSMUSG00000103544
ENSMUSG00000103567
ENSMUSG00000103585
ENSMUSG00000103677
ENSMUSG00000103697
ENSMUSG00000103707
ENSMUSG00000103749
ENSMUSG00000103770
ENSMUSG00000103793
ENSMUSG00000103800
ENSMUSG00000103897
ENSMUSG00000103898
ENSMUSG00000103906
ENSMUSG00000103924
ENSMUSG00000104030
ENSMUSG00000104063
ENSMUSG00000104132
ENSMUSG00000104148
ENSMUSG00000104178
ENSMUSG00000104222
ENSMUSG00000104252
ENSMUSG00000104318
ENSMUSG00000104346
ENSMUSG00000104362
ENSMUSG00000104525
ENSMUSG00000104597
ENSMUSG00000104655
ENSMUSG00000104696
ENSMUSG00000104856
ENSMUSG00000104960
ENSMUSG00000105025
ENSMUSG00000105176
ENSMUSG00000105263
ENSMUSG00000105265
ENSMUSG00000105771
ENSMUSG00000105827
ENSMUSG00000105867
ENSMUSG00000105895
ENSMUSG00000105974
ENSMUSG00000106099
ENSMUSG00000106379
ENSMUSG00000106579
ENSMUSG00000106826
ENSMUSG00000106847
ENSMUSG00000106864
ENSMUSG00000106918
ENSMUSG00000106951
ENSMUSG00000106981
ENSMUSG00000107000
ENSMUSG00000107002
ENSMUSG00000107023
ENSMUSG00000107068
ENSMUSG00000107092

ENSMUSG00000107096
ENSMUSG00000107182
ENSMUSG00000107283
ENSMUSG00000107331
ENSMUSG00000107383
ENSMUSG00000107470
ENSMUSG00000107476
ENSMUSG00000107512
ENSMUSG00000107741
ENSMUSG00000107858
ENSMUSG00000107877
ENSMUSG00000107879
ENSMUSG00000107947
ENSMUSG00000108005
ENSMUSG00000108049
ENSMUSG00000108070
ENSMUSG00000108228
ENSMUSG00000108348
ENSMUSG00000108350
ENSMUSG00000108414
ENSMUSG00000108591
ENSMUSG00000108616
ENSMUSG00000108621
ENSMUSG00000108815
ENSMUSG00000108841
ENSMUSG00000108880
ENSMUSG00000109006
ENSMUSG00000109121
ENSMUSG00000109122
ENSMUSG00000109125
ENSMUSG00000109239
ENSMUSG00000109321
ENSMUSG00000109324
ENSMUSG00000109336
ENSMUSG00000109372
ENSMUSG00000109394
ENSMUSG00000109398
ENSMUSG00000109511
ENSMUSG00000109523
ENSMUSG00000109536
ENSMUSG00000109562
ENSMUSG00000109574
ENSMUSG00000109715
ENSMUSG00000109865
ENSMUSG00000109901
ENSMUSG00000109946
ENSMUSG00000110080
ENSMUSG00000110185
ENSMUSG00000110195
ENSMUSG00000110234
ENSMUSG00000110246
ENSMUSG00000110332
ENSMUSG00000110357

ENSMUSG00000110444
ENSMUSG00000110523
ENSMUSG00000110558
ENSMUSG00000110710
ENSMUSG00000110841
ENSMUSG00000110945
ENSMUSG00000110980
ENSMUSG00000111080
ENSMUSG00000111128
ENSMUSG00000111375
ENSMUSG00000111394
ENSMUSG00000111977
ENSMUSG00000112117
ENSMUSG00000112280
ENSMUSG00000112302
ENSMUSG00000112343
ENSMUSG00000112449
ENSMUSG00000112941
ENSMUSG00000113061
ENSMUSG00000113184
ENSMUSG00000113204
ENSMUSG00000113528
ENSMUSG00000113621
ENSMUSG00000113902
ENSMUSG00000113949
ENSMUSG00000114019
ENSMUSG00000114153
ENSMUSG00000114584
ENSMUSG00000114797
ENSMUSG00000114970
ENSMUSG00000115338
ENSMUSG00000115388
ENSMUSG00000115420
ENSMUSG00000115423
ENSMUSG00000115783
ENSMUSG00000115987
ENSMUSG00000116048
ENSMUSG00000116138
ENSMUSG00000116165
ENSMUSG00000116180
ENSMUSG00000116275
ENSMUSG00000116305
ENSMUSG00000116564
ENSMUSG00000116594
ENSMUSG00000116760
ENSMUSG00000116766
ENSMUSG00000116858
ENSMUSG00000116875
ENSMUSG00000116908
ENSMUSG00000116995
ENSMUSG00000117098
ENSMUSG00000117171
ENSMUSG00000117172

ENSMUSG00000117278
ENSMUSG00000117286
ENSMUSG00000117310
ENSMUSG00000117406
ENSMUSG00000117499
ENSMUSG00000117679
ENSMUSG00000117725
ENSMUSG00000117789
ENSMUSG00000117814
ENSMUSG00000117901
ENSMUSG00000117924
ENSMUSG00000117943
ENSMUSG00000117992
ENSMUSG00000118012
ENSMUSG00000118018
ENSMUSG00000118125
ENSMUSG00000118202
ENSMUSG00000118332
ENSMUSG00000118401

Supplementary Table 3. background genes and lists of genes found in WGCNA modules with an association to PNELS, sex, or DeepOF behavioral component ($R > 0.5$, $p < 0.05$)

pPFC ME7	pPFC ME9	BNST ME11	vDG ME11
Narf	Drp2	Cacna2d2	Mmp14
Pdgfb	Tamalin	Ntn4	Map2k7
Rmnd5b	Gmpr2	Npffr1	Tbc1d8
Mef2d	Dgcr6	Per1	Gys1
Ipo4	Mmd	Gng4	Col26a1
Slc25a42	Akt2	Cxcl14	Arhgef18
Relb	Ap1s1	Prkcd	Otx1
Cacnb3	Rasa4	Myo10	Kctd20
Prkcsh	Chd5	Arc	Tep1
Itpkc	Por	Dgkg	Lrrc8a
Nob1	Pvalb	Dlx2	Fgfrl1
Brinp2	Ephb3	Glp1r	Rdh13
Bud23	Slc4a3	Gfra1	Trpm2
Hmox1	Hapln4	Ddr2	Vav2
Dnajb1	Prrg2	Pax6	Kcnd1
Kctd20	Met	Sema6d	Slc38a3
Cbfa2t3	Pou6f1	Zbtb46	Tjap1
P4htm	Slc9a5	Trpc3	Lrrk1
Abhd8	Plekho1	Ccdc30	Rxra
Nf2	Scube1	Fosl2	Castor2
Spire2	Cntnap1	Sel1l3	C1qtnf1
Kansl3	Nlk	Rhof	Mmp9
Zfp790	Pitpna	Atp2a2	Pltp
Slc41a1	Pip4k2b	Ret	Myo1c
Ehmt2	Crhr1	Ano1	Zbtb4
Klhl3	Wwc1	Dlc1	Isyna1
Rnf166	Sult4a1	Zic1	Utrn
Surf4	Scn1b	Adra2a	Aebp1
Srf	Rps6kl1	BC034090	Cenpo
Serac1	Hkdc1	Fgd5	Klf11
Camk1g	Cobl	Dusp5	Mmp28
Mapkapk2	Rasgef1c	Kank4	Cacng4
Ttll12	Dynll2	Ripor2	Ntn1
Mpp2	Lratd1	Lmo1	Plcd3
Zfp334	Rnf144a	Lzts1	Mthfd1
Stard3	Sntg2	Zic4	Smoc1
Abi3	Mxra7	Whrn	Exoc3l4
Mtmr4	Cacna1g	C130074G19Rik	Mrs2
Dusp14	Rgs6	Prdm16	Sema4d
Ppp1r12c	Isca2	Cacng5	Ccdc191
P4ha1	Cyp46a1	Nbl1	Dip2b
Nuak1	Meg3	Chd7	Dlx2
Jade2	Gcnt2	Zic5	Rhebl1
Osbp2	Plpp1	Hs3st2	Vegfa
Pdia6	Anxa11	Cxxc5	Tmem204
Trib2	Extl3	Col6a3	Svil
Klhl29	Slc39a14	Sp8	Pcx

Camkk1	Gfra2	Rasd1	Rela
Cacnb1	Marchf11	Prag1	Ltbp3
Per1	Cacna1i	Trhde	Map4k2
Tmem101	Plec	Klf13	Gpam
Ccdc88c	Zcrb1	Hunk	Dnmbp
Otub2	Boc	Shisa6	Sema4g
Tgfb3	Sidt1	Irs1	Gaa
Yy1	Smarcd1	Zic2	Pfkfb4
Rhobtb2	Igfbp6	Zic3	Sdc3
Mettl3	Cdkn1a	Sp9	Smad7
Acin1	Nagpa	Kcng1	Nfasc
Ankrd33b	Grm2	Inafm2	Slc25a25
Pop1	Bysl	Prdm16os	Inpp5e
Josd1	Cpne5	Lbhd2	Lrp4
Sh3bp1	Ldlrad4	Tcerg1l	Dnaaf9
Polr3h	Mapk4	Sox1	Edn3
Hsf1	Tcof1		Hcn3
Scrib	Camk2a		Nipal3
Sdf2l1	Acsl5		Wasf2
Arhgap31	Kcnip2		Mtf1
Cbr3	Pip4k2c		Plekhm2
Rcan1	Rbfox3		Clcn6
Asic1	Cbx8		Htra3
Creld2	Rassf3		Wasf3
Ralbp1	Prkar1b		Irf5
Mllt1	Tspan17		Slc6a13
Myo1f	Tmem169		Mtmr14
Cxxc1	Epha4		Slc6a1
Pcyox1l	Bok		Acan
Syvn1	Asb1		Bag3
Uhrf2	Pam		Abcd1
Dpf2	Stxbp1		Atp11a
Chka	Acvr1		Eif2ak3
Fam89b	Dab2ip		Mmp15
Men1	Frzb		Ldhd
Afap1l2	Lamp5		Snx19
Mfsd13a	Itpka		Ldlr
Banp	Adra1d		Pstpip1
Tepsin	Bmp2		Marchf3
Ark2c	Idh3b		Heyl
Rab40c	Pcsk2		Afap1l1
Pdia4	Ppm1l		Micall1
Fam20c	Kcnab1		Senp1
Unc5a	Ptgfrn		Slc22a15
Ppm1f	Slc6a17		Stk36
Capn10	Bcar3		Ptprf
Rassf5	Tspan5		Ppip5k1
Pycr2	Wls		Idua
Usf1	Brinp1		BC034090
Pfkfb3	B4galt2		Foxj1
Slc25a25	Epha8		Igsf9b
Nr4a2	Gale		Nek9
Crat	Hpca		Tmem132d

Hspa5	Extl1	Caskin2
Pag1	Plch2	Inpp5j
Ptpn1	Slc30a3	Mrps10
Mmp24	Cdk2ap1	Asb16
Src	Cabp1	Acot11
Khdc4	Mtus2	Rims4
Sema4a	Cux1	Midn
Nr4a3	Cpne9	Ppp1r26
Cntfr	Prmt8	Aldh5a1
Ak4	Apba2	Tmtc2
Hivep3	Ppme1	Sh3bp4
Ccdc28b	Dkkl1	Adamts2
Prkag2	Stx1b	Micall2
Ube4b	Cpxm2	Asap3
Pgd	Stard8	Tns2
Ski	Atp2b3	Sik2
Trmt44	Mylk3	Tbc1d2b
Fosl2	6430548M08Rik	Uggt1
Tbc1d1	Mast3	Vstm2l
Slc15a4	Pin1	Fgd3
Ncor2	Hcn4	Slc43a2
Aacs	Gsta4	Mindy1
Ulk1	Dclk3	Bmp6
Zfp12	Cck	Zdhhc1
Dtx1	Arhgef17	Npepl1
Prickle2	Rims3	Ndst2
Lrig1	Phldb2	Rgl3
Plxna1	S100b	Accs
Bhlhe40	Kctd17	Tmem51
Furin	Crlf2	Sh3pxd2b
Sipa1l3	Emx1	Slc25a34
Dpf1	Egr3	Kirrel1
Aen	Asb13	Dlx1
Bcat2	Zswim5	Map3k9
Fam53b	Galnt17	Tril
Dennd2b	Igsf9b	Gpr146
Pim2	Fhod3	Macir
Slc35a2	Kcnh5	Myorg
Efnb1	Rasd2	Xxylt1
Dusp4	Rab24	Baiap3
Ist1	Unc13a	Gpr157
Gse1	Pip5k1c	Zbtb8b
Taf1c	Arl6ip5	Igf2
Smpd3	Prdm8	Cfap46
Ppp2r1b	Dnajb5	Arhgap23
Abcg4	Mgat5	Adam17
Cgnl1	Kctd1	Arhgef15
Tle3	Fstl4	Cdh22
Ptpn9	Cdh8	Gm9899
Celf6	Sun1	Rpia
Slc25a38	Rskr	Ankrd24
Cish	Spag6	Nfic
Pwp2	2810032G03Rik	Alk

Fbxw8
Fhip2a
Znrf1
Spata2l
Caskin1
Arhgap39
Irf2bpl
Rasl10a
Plekhm1
Zdhhc14
Neurl1b
Tent4a
Chst11
Tmem109
Gna11
Mfsd12
Dhcr24
Rundc1
Plekhj1
Pcsk7
Midn
Rubcn
Zswim4
Gramd4
Mgat4b
Lin37
Wnt4
Sik2
Mxd4
Man1c1
Paqr7
Osbpl5
Ahdc1
Egr2
Ankrd13b
Sh3rf3
Rara
Dexi
Stox2
Neurod2
Sgsm2
Setd1b
Egr1
Socs7
Ripor1
Smurf1
Irs2
Polg
Ints15
Fndc3b
Fut11
Tnrc18
Ap5z1
Vstm2l
Kcnj9
Tigar
Tapbpl
Trpm4
Sema7a
Pmepa1
Rgs4
Arfrp1
Lmtk2
Nrn1
Naif1
Bbln
Metrnl
C130074G19Rik
Znfx1
Htatip2
Gpr26
Bach2
Hey1
Cchcr1
Dgkz
Iqsec3
Plekhh2
Npnt
Plekha6
Slc16a6
Nol4
Igsf3
Kank3
Golga7b
Rgs8
Tmem108
Rtn4r
Mgat5b
Dnajc21
Osbpl1a
Alkbh2
Lsm11
Ubtd2
Kcng3
Sowahb
Pnma3
Asphd1
Kcnk6
Camk2n1
Rbm15
Nat8l
Lhfpl6
Inka2
Bdnf
Mmgt2
Dact2
Tmem150a
Apol8
Ece1
Panx2
Kcnc1
Scrt2
Atp8b2
Acvr2b
Zfp696
Syne2
Vsig10
4930581F22Rik
Zswim9
Gprin2
D730003I15Rik
Prob1
Thbd
Nrbp2
Nynrin
Klhdc7a
Cfap74
Slc45a4
Zfp664
Tomm6os
Tmem200c
Jpx
Gm43597
Gm49338
Gm40841

Lonrf1	Ptgr3
Dusp26	Pdp1
Slx4	Lingo1
Bahcc1	Zfpm1
Gnaz	Nhsl3
Tax1bp3	Adra1b
Bcor	Tmem198
Spns2	Mtcl1
Lrfn2	Doc2a
Chrm4	Mpp3
Dok4	A330008L17Rik
Spen	Pcdh15
Osbpl10	Nrgn
Zfp598	Pnma1
Mul1	Smim10l2a
Cdk13	Rnd1
Sntb2	Lrtm2
Nthl1	Tns1
Rrp9	Gucy2g
Mrps27	Tmem191
Kif21b	Otulinl
Myrip	Car10
Mvk	Kcnq3
Sgsm1	Ilrun
Hps4	Kcnh1
Kcnj12	Nptx2
Trim46	Trmt61a
Sbk1	Ing2
Synpo	Tacc1
Elfn2	Cacng3
Fem1a	Zbtb16
Rsbn1	Fam149a
Sowaha	Gmppb
Rin3	Mpzl3
Zfand3	Etnk2
Rtn4rl1	Map3k5
Spred2	Septin5
Cxxc5	Lrrc61
Fbxo41	Mtarc2
Gpr68	Syndig1
Pygo2	Hps6
Bri3	Heg1
Mlec	Dnlz
Hexim1	6330403L08Rik
Lix1l	Fam174b
Kmt5a	Zfp703
Ogfr	Gm2415
Urgcp	Susd5
Slc25a44	Gcnt4
Il17d	Tnnc1
Oprd1	Tmem200c
Lingo3	A830009L08Rik
Gfod1	Miat

2900026A02Rik
Pcdh1
Mafg
Mical3
Kcnf1
Slc6a7
Foxo6
Gpr17
Zfta
Gatd3a
Dusp7
Txlna
Kdm2a
Tmem119
Klhl18
Per2
Irs1
Ier5
Jmjd6
Slc35f3
Armc7
Upf1
Pop5
Pwwp2b
Ipmk
Lrrtm1
Zbtb40
Lmnb2
Rai1
Cys1
Numbl
Grm4
Bcl9l
Fbxl18
Cry2
Psrc1
Ccdc92b
Mn1
Dgkd
Egr4
Nalf2
Ccnf
Nanos1
Acot1
Zfp865
Kcng1
Inafm2
Nrbf2
Fam43b
F8a
Ddi2
Chd2
St6galnac4
Gm29100
Gm43481
Particl
Gm35161
Gm47155
Gm49037

Pabpc1l2b
Ier5l
Pou3f1
Tmem121b
Erdr1x
Tmem151b
Mir124a-1hg
2900093K20Rik
Pdxp
Gm54798

pPFC background	BNST background	vDG background
Gnai3	Gnai3	Gnai3
Narf	Narf	Narf
Cav2	Cav2	Cav2
Klf6	Klf6	Klf6
Scmh1	Scmh1	Scmh1
Cox5a	Cox5a	Cox5a
Wnt9a	Ngfr	Wnt3
Fer	Wnt3	Wnt9a
Xpo6	Fer	Fer
Tfe3	Xpo6	Xpo6
Axin2	Tfe3	Tfe3
Brat1	Axin2	Axin2
Gna12	Brat1	Brat1
Dlat	Gna12	Gna12
Sdhd	Dlat	Dlat
Ccnd2	Sdhd	Sdhd
Gpr107	Ccnd2	Ccnd2
Nalcn	Gpr107	Gpr107
Btbd17	Nalcn	Nalcn
Th	Btbd17	Btbd17
Drp2	Drp2	Drp2
Lhx2	Lhx2	Lhx2
Gmpr	Gmpr	Gmpr
Mid2	Mid2	Mid2
Trim25	Trim25	Trim25
Dgke	Dgke	Dgke
Scpep1	Scpep1	Scpep1
Mnt	Mnt	Mnt
Itgb2	Itgb2	Itgb2
Hddc2	Hddc2	Hddc2
Tpd52l1	Tpd52l1	Tpd52l1
Cdh4	Cdh4	Cdh4
Ckmt1	Ckmt1	Ckmt1
Comt	Comt	Comt
Rtca	Rtca	Rtca
Dbt	Dbt	Dbt
Dazap2	Dazap2	Dazap2
Mcts1	Mcts1	Mcts1
Trappc10	Trappc10	Trappc10
Ccm2	Ccm2	Ccm2
Tbrg4	Tbrg4	Tbrg4
Ndufa9	Ndufa9	Ndufa9

Cttnbp2	Cttnbp2	Cttnbp2
Galnt1	Galnt1	Galnt1
Mkrn2	Mkrn2	Mkrn2
Pparg	Raf1	Raf1
Raf1	Pdgfb	Pdgfb
Pdgfb	Acvrl1	Acvrl1
Acvrl1	Tamalin	Tamalin
Tamalin	Acvr1b	Acvr1b
Acvr1b	Tom1l2	Tom1l2
Tom1l2	Zfp385a	Zfp385a
Zfp385a	Gabra2	Gabra2
Gabra2	Wdr77	Wdr77
Wdr77	Atp5pb	Atp5pb
Atp5pb	Sox9	Sox9
Sox9	Hnrnpd	Hnrnpd
Hnrnpd	Dynlt1c	Dynlt1c
Dynlt1c	C1d	C1d
C1d	Gm2a	Gm2a
Gm2a	Krit1	Krit1
Krit1	Clcn4	Clcn4
Clcn4	Sema4f	Sema4f
Sema4f	Hk2	Hk2
Hk2	Myo18a	Myo18a
Myo18a	Sez6	Sez6
Sez6	Abhd15	Loxl3
Loxl3	Loxl3	Kat2b
Kat2b	Kat2b	Rab5b
Rab5b	Rab5b	Icosl
Spg7	Icosl	Spg7
Rpl13	Spg7	Rpl13
Chmp1a	Rpl13	Chmp1a
Rpa1	Chmp1a	Rpa1
Serpinf1	Rpa1	Serpinf1
Tubgcp3	Serpinf1	Tubgcp3
Oprm1	Tubgcp3	Oprm1
Polr3d	Oprm1	Polr3d
Ddx3x	Polr3d	Ddx3x
Slc5a5	Ddx3x	Slc5a5
Kcnn3	Slc5a5	Kcnn3
Usp32	Kcnn3	Usp32
Car4	Usp32	Car4
Txnrd3	Car4	Txnrd3
Zfp512b	Txnrd3	Zfp512b
Dnajc5	Zfp512b	Dnajc5
Tpd52l2	Dnajc5	Tpd52l2
Fmr1	Tpd52l2	Fmr1
Bcl11a	Fmr1	Bcl11a
Pxmp4	Bcl11a	Pxmp4
Dlg3	Pxmp4	Dlg3
Gnb1l	Dlg3	Gnb1l
Smarcb1	Smarcb1	Smarcb1
Hip1r	Hip1r	Hip1r
Nsun5	Nsun5	Nsun5

Top1mt	Top1mt	Top1mt
Gm38393	Gm38393	Gm38393
Mmp14	Mmp14	Mmp14
Oxa1l	Oxa1l	Oxa1l
Heatr6	Heatr6	Heatr6
Ilf2	Ilf2	Ilf2
Chtop	Chtop	Chtop
Snapin	Snapin	Snapin
S100a6	S100a6	S100a6
Mapk7	Mapk7	Mapk7
Epn2	Epn2	Epn2
B9d1	B9d1	B9d1
Sec24b	Sec24b	Sec24b
N4bp3	N4bp3	N4bp3
Rmnd5b	Rmnd5b	Rmnd5b
Nhp2	Nhp2	Nhp2
Vps9d1	Vps9d1	Vps9d1
Zfp276	Zfp276	Zfp276
Mfsd10	Mfsd10	Mfsd10
Luzp1	Luzp1	Luzp1
Kctd10	Kctd10	Kctd10
Poldip2	Poldip2	Poldip2
Sebox	Ift20	Sebox
Ift20	Col6a1	Ift20
Col6a1	Pcbp3	Col6a1
Pcbp3	Araf	Pcbp3
Lgals9	Cfp	Lgals9
Araf	Cnnm3	Araf
Cfp	Lman2l	Cfp
Uxt	Mcm3ap	Uxt
Cnnm3	Pcnt	Cnnm3
Lman2l	Mxd1	Lman2l
Mcm3ap	Gmcl1	Mcm3ap
Pcnt	Snrnp27	Pcnt
Mxd1	Ocrl	Mxd1
Gmcl1	Calm1	Gmcl1
Snrnp27	Agpat3	Snrnp27
Ocrl	Sema6b	Ocrl
Calm1	Dpp9	Calm1
Agpat3	Ramp2	Agpat3
Sema6b	Lsr	Sema6b
Dpp9	Gramd1a	Dpp9
Ramp2	Gabrg1	Ramp2
Lsr	Ckb	Lsr
Gramd1a	Sp1	Gramd1a
Gabrg1	Myg1	Gabrg1
Ckb	Rarg	Ckb
Sp1	Pfdn5	Sp1
Myg1	Efnb2	Myg1
Rarg	Rrp15	Rarg
Pfdn5	Rnd2	Pfdn5
Efnb2	Srr	Efnb2
Rrp15	Fndc5	Rrp15

Rnd2	Fbxo9	Rnd2
Srr	Vps50	Srr
Sync	Hars1	Fndc5
Fndc5	Zmat2	Fbxo9
Fbxo9	Smg5	Vps50
Vps50	Cct3	Hars1
Hars1	Glmp	Zmat2
Zmat2	Mef2d	Smg5
Smg5	Snd1	Cct3
Cct3	Col18a1	Glmp
Glmp	Slc19a1	Mef2d
Mef2d	Kpnb1	Snd1
Snd1	Npepps	Col18a1
Slc19a1	Mrpl10	Slc19a1
Kpnb1	Cyp51	Kpnb1
Npepps	Tcf25	Npepps
Mrpl10	Def8	Mrpl10
Cyp51	Nkx2-1	Cyp51
Tcf25	Col1a1	Tcf25
Def8	Itga3	Def8
Col1a1	Itfg2	Col1a1
Itga3	Nrip2	Itga3
Itfg2	Tulp3	Itfg2
Nrip2	Gtf2h4	Nrip2
Tulp3	Tubb5	Tulp3
Gtf2h4	Ell2	Gtf2h4
Tubb5	Jup	Tubb5
Ell2	Fkbp10	Ell2
Jup	Nom1	Jup
Fkbp10	Ergic1	Fkbp10
Nom1	Ifrd1	Nom1
Ergic1	Stk38l	Ergic1
Ifrd1	Brpf1	Ifrd1
Stk38l	Akr1b1	Stk38l
Brpf1	Gstt1	Brpf1
Akr1b1	Gstt3	Akr1b1
Gstt1	Ddt	Gstt1
Ddt	Ddx18	Gstt3
Ddx18	Ubl3	Ddt
Ubl3	Gramd2b	Ddx18
Gramd2b	Eef1e1	Ubl3
Eef1e1	Akt1	Gramd2b
Akt1	Tcirg1	Eef1e1
Il16	Naglu	Akt1
Tcirg1	Coasy	Il16
Naglu	Smo	Tcirg1
Coasy	Tspan33	Naglu
Smo	Crnkl1	Coasy
Tspan33	Rin2	Smo
Crnkl1	Folh1	Tspan33
Rin2	Chordc1	Crnkl1
Folh1	Rtcb	Rin2
Chordc1	Pwp1	Folh1

Rtcb	Fbxo7	Chordc1
Pwp1	Capns1	Rtcb
Fbxo7	Lrp3	Pwp1
Capns1	Clpb	Fbxo7
Lrp3	Septin7	Capns1
Clpb	Zdhhc4	Lrp3
Septin7	Rac1	Clpb
Zdhhc4	Nup214	Septin7
Rac1	Aif1l	Zdhhc4
Nup214	Ltbp1	Rac1
Aif1l	Ugp2	Nup214
Ltbp1	Kcnh6	Aif1l
Ugp2	Trmt1	Ltbp1
Kcnh6	Nacc1	Ugp2
Trmt1	Nfix	Trmt1
Nacc1	Uba1	Nacc1
Nfix	Vwf	Nfix
Uba1	Siae	Uba1
Vwf	Esam	Vwf
Siae	Fam50a	Siae
Esam	Emd	Esam
Fam50a	Taco1	Fam50a
Emd	Grik3	Emd
Taco1	Gria3	Taco1
Grik3	Sipa1l2	Grik3
Gria3	Ap4e1	Gria3
Npas1	Blvra	Npas1
Sipa1l2	Pdzd4	Sipa1l2
Ap4e1	Idh3g	Ap4e1
Blvra	Pnck	Blvra
Pdzd4	Ssr4	Pdzd4
Srpk3	Bcap31	Idh3g
Idh3g	Fam98a	Pnck
Pnck	Kmt2a	Ssr4
Ssr4	Ift46	Bcap31
Bcap31	Tmem25	Fam98a
Fam98a	Trappc6a	Kmt2a
Kmt2a	Supt6	Ift46
Ift46	Unc119	Tmem25
Tmem25	Rab34	Trappc6a
Trappc6a	Sdf2	Supt6
Supt6	Ccne1	Spag5
Spag5	Bbc3	Unc119
Unc119	Psmc3	Rab34
Rab34	Acp2	Sdf2
Sdf2	Slc39a13	Ccne1
Ccne1	Celf2	Bbc3
Bbc3	Ddb2	Psmc3
Psmc3	Sf3a1	Acp2
Acp2	Stat6	Slc39a13
Slc39a13	Vrk3	Celf2
Celf2	Smg9	Nr1h3
Ddb2	Paxip1	Ddb2

Spi1	Rmnd5a	Spi1
Sf3a1	Mov10	Sf3a1
Stat6	Rhoc	Stat6
Clgn	Ppard	Clgn
Vrk3	Peg3	Vrk3
Smg9	Zim1	Smg9
Paxip1	Metrn	Paxip1
Rmnd5a	Lmf1	Rmnd5a
Mov10	Ciao3	Mov10
Rhoc	Angptl4	Rhoc
Ppard	Daxx	Ppard
Peg3	Cd320	Peg3
Metrn	Ipo4	Zim1
Lmf1	Tm9sf1	Metrn
Ciao3	Irf9	Lmf1
Angptl4	Gmpr2	Ciao3
Daxx	Mdp1	Daxx
Cd320	Dhrs1	Cd320
Ipo4	Ncan	Ipo4
Tm9sf1	Tmem161a	Tm9sf1
Irf9	Armc6	Irf9
Gmpr2	Borcs8	Gmpr2
Mdp1	Slc25a42	Mdp1
Dhrs1	Snx9	Dhrs1
Ncan	Ranbp3	Ncan
Tmem161a	Ndufa11	Tmem161a
Armc6	Nr2f6	Armc6
Borcs8	Use1	Borcs8
Slc25a42	Ocel1	Slc25a42
Snx9	Dyrk1b	Snx9
Ranbp3	Braf	Ranbp3
Ndufa11	Ndufb2	Ndufa11
Nr2f6	Hltf	Nr2f6
Use1	Prpf6	Use1
Ocel1	Rgs19	Ocel1
Dyrk1b	Rgs20	Dyrk1b
Braf	Abhd3	Braf
Ndufb2	Snrpd1	Ndufb2
Hltf	Tchp	Hltf
Prpf6	Tiam1	Prpf6
Rgs19	Tsc2	Rgs19
Rgs20	Nherf2	Rgs20
Abhd3	Puf60	Abhd3
Snrpd1	Golga2	Snrpd1
Tchp	Uck1	Tchp
Tiam1	Ikzf4	Tiam1
Tsc2	Mien1	Tsc2
Nherf2	Axl	Nherf2
Puf60	Tgfb1	Puf60
Golga2	Ccdc97	Golga2
Uck1	Zfp40	Uck1
Ikzf4	Akap8l	Ikzf4
Mien1	Pdcd2l	Mien1

Axl	Gtf2f1	Axl
Tgfb1	Clpp	Tgfb1
Ccdc97	Alkbh7	Ccdc97
Zfp40	Med6	Zfp40
Akap8l	Prkd1	Akap8l
Shh	Cse1l	Pdcd2l
Pdcd2l	Naa20	Gtf2f1
Gtf2f1	Prkra	Clpp
Clpp	Plekha3	Alkbh7
Alkbh7	Ykt6	Med6
Med6	Baz1b	Prkd1
Prkd1	Pex6	Lcp2
Lcp2	Mrpl2	Cse1l
Cse1l	Mea1	Naa20
Naa20	Grin2d	Prkra
Prkra	Kdelr1	Plekha3
Plekha3	Tmem143	Ykt6
Ykt6	Ggct	Baz1b
Baz1b	Jag2	Pex6
Pex6	Btbd6	Mrpl2
Mrpl2	Nudt14	Mea1
Mea1	Epdr1	Grin2d
Grin2d	Flii	Kdelr1
Kdelr1	Top3a	Tmem143
Tmem143	Atg4d	Ggct
Ggct	Qtrt1	Jag2
Jag2	Plin4	Btbd6
Btbd6	Hdgfl2	Nudt14
Nudt14	Chaf1a	Epdr1
Epdr1	Adprh	Flii
Flii	Tmem39a	Top3a
Top3a	Timmdc1	Atg4d
Atg4d	Mcm2	Qtrt1
Qtrt1	Tpra1	Hdgfl2
Hdgfl2	Nab1	Chaf1a
Chaf1a	Adgre5	Adprh
Adprh	Il17ra	Tmem39a
Tmem39a	Lamb1	Timmdc1
Timmdc1	Kcnn1	Mcm2
Mcm2	Arrdc2	Tpra1
Tpra1	Map2k7	Nab1
Nab1	Timm44	Adgre5
Adgre5	Ap2a2	Il17ra
Il17ra	Pnkp	Lamb1
Lamb1	Med25	Kcnn1
Kcnn1	Bcam	Arrdc2
Arrdc2	Clptm1	Map2k7
Map2k7	Relb	Timm44
Timm44	Tomm40	Ap2a2
Ap2a2	Apoe	Pnkp
Pnkp	Hbp1	Med25
Med25	Prkar2b	Bcam
Bcam	Cdkn1b	Clptm1

Clptm1	Klf4	Relb
Relb	Ap1m1	Tomm40
Tomm40	Rab8a	Apoe
Apoe	Hmgn2	Hbp1
Hbp1	Fam32a	Prkar2b
Prkar2b	Stard3nl	Cdkn1b
Cdkn1b	Stk11	Ap1m1
Klf4	Efna2	Rab8a
Ap1m1	Atp5f1d	Hmgn2
Rab8a	Ppp5c	Fam32a
Hmgn2	Cdk12	Stard3nl
Fam32a	Lipe	Stk11
Stard3nl	Pafah1b2	Efna2
Stk11	Tbc1d8	Atp5f1d
Efna2	Cnot11	Ppp5c
Atp5f1d	Slc2a3	Cdk12
Ppp5c	Foxj2	Lipe
Cdk12	Sri	Pafah1b2
Lipe	Dgcr2	Tbc1d8
Pafah1b2	Irf3	Cnot11
Tbc1d8	Mpnd	Slc2a3
Cnot11	Sh3gl1	Foxj2
Slc2a3	Yju2	Sri
Foxj2	Ranbp2	Dgcr2
Sri	Grk5	Irf3
Dgcr2	Dvl3	Mpnd
Irf3	Abcf3	Sh3gl1
Zfp959	Eif2b5	Yju2
Mpnd	Cyth2	Ranbp2
Sh3gl1	Car11	Grk5
Yju2	Dlgap1	Dvl3
Ranbp2	Mrpl4	Abcf3
Grk5	Keap1	Eif2b5
Dvl3	Glg1	Cyth2
Abcf3	Btbd2	Sult2b1
Eif2b5	Csnk1g2	Car11
Cyth2	Abhd17a	Dlgap1
Sult2b1	Mob3a	Plag1
Car11	Cacnb3	Mrpl4
Dlgap1	Ccdc65	Keap1
Plag1	Ddx23	Glg1
Mrpl4	Pld3	Btbd2
Keap1	Grik5	Csnk1g2
Glg1	Rabac1	Abhd17a
Btbd2	Etv3	Mob3a
Csnk1g2	Prkcsh	Cacnb3
Abhd17a	Elavl3	Ccdc65
Mob3a	Rab3b	Ddx23
Cacnb3	St8sia6	Pld3
Ccdc65	Fcgrt	Grik5
Ddx23	Nosip	Rabac1
Pld3	Pih1d1	Etv3
Grik5	Rps11	Prkcsh

Rabac1
Etv3
Prkcsh
Elavl3
Rab3b
Fcgrt
Nosip
Pih1d1
Rps11
Supt5
Paf1
Timm50
Med29
Bicd1
Ncstn
Pex19
Phyhip
Impdh1
Dusp3
Prodh
Ess2
Slc25a1
Dgcr6
Ddr1
Ier3
Fosb
Klc4
Ercc1
As3mt
Homer3
Crtc1
Rnf215
Sec14l2
Aven
Cp
Crot
Rps6ka1
Calb2
Snrnp200
Ciao1
Taf6l
Insig2
Kpna6
Man1a
Itpkc
Coq8b
Brd8
Farsa
Gcdh
Mast2
Dnase2a
Rad23a
Calr

Supt5
Paf1
Timm50
Med29
Bicd1
Ncstn
Pex19
Phyhip
Crhr2
Impdh1
Dusp3
Prodh
Ess2
Slc25a1
Dgcr6
Ddr1
Fosb
Klc4
Ercc1
As3mt
Homer3
Crtc1
Rnf215
Sec14l2
Aven
Cp
Crot
Rps6ka1
Calb2
Snrnp200
Ciao1
Taf6l
Insig2
Kpna6
Man1a
Itpkc
Coq8b
Brd8
Farsa
Gcdh
Mast2
Dnase2a
Rad23a
Calr
Nfat5
Nob1
Ppfia3
Gys1
Ruvbl2
Lin7b
Bax
Tfam
Zfp81

Elavl3
Rab3b
St8sia6
Fcgrt
Nosip
Pih1d1
Rps11
Supt5
Paf1
Timm50
Med29
Bicd1
Ncstn
Pex19
Phyhip
Impdh1
Dusp3
Prodh
Ess2
Slc25a1
Dgcr6
Ddr1
Fosb
Klc4
Ercc1
As3mt
Homer3
Crtc1
Rnf215
Sec14l2
Aven
Cp
Crot
Rps6ka1
Calb2
Snrnp200
Ciao1
Taf6l
Insig2
Kpna6
Man1a
Itpkc
Coq8b
Brd8
Farsa
Gcdh
Mast2
Dnase2a
Rad23a
Calr
Nfat5
Nob1
Nqo1

Nfat5	Efnb3	Ppfia3
Nob1	Mmd	Gys1
Ppfia3	Hlf	Ruvbl2
Gys1	Fam162a	Lin7b
Ruvbl2	Rpl8	Bax
Lin7b	Grm3	Tfam
Bax	Ssbp2	Zfp81
Tfam	Fancl	Efnb3
Zfp81	Brinp2	Mmd
Efnb3	Gstm5	Hlf
Mmd	Gstm7	Fam162a
Hlf	Stat3	Rpl8
Fam162a	Stat5a	Grm3
Rpl8	Cavin1	Ssbp2
Grm3	Map3k11	Fancl
Ssbp2	Akt2	Brinp2
Fancl	Dnaja3	Gstm5
Brinp2	Hmox2	Gstm7
Gstm5	Cdip1	Stat3
Gstm7	Map3k20	Cavin1
Stat3	Cwc15	Map3k11
Cavin1	Col5a3	Akt2
Map3k11	Dnmt1	Dnaja3
Akt2	Ppan	Hmox2
Dnaja3	Cacna1e	Cdip1
Hmox2	Cacna1b	Map3k20
Cdip1	Trmt10a	Cwc15
Map3k20	Etv1	Col5a3
Cwc15	Kifc2	Dnmt1
Dnmt1	Psap	Ppan
Ppan	Ikbkg	Cacna1e
Cacna1e	Wars2	Cacna1b
Cacna1b	Atn1	Trmt10a
Trmt10a	Phb2	Etv1
Etv1	Eno2	Kifc2
Kifc2	Emg1	Psap
Psap	Lpcat3	Ikbkg
Ikbkg	Atp6v1f	Wars2
Wars2	Clcn5	Atn1
Atn1	Clcn3	Phb2
Phb2	Hif3a	Eno2
Ptpn6	Pde1c	Emg1
Eno2	Utp20	Lpcat3
Emg1	9330159F19Rik	Atp6v1f
Lpcat3	Cul3	Clcn5
Atp6v1f	Sst	Clcn3
Clcn5	Large1	Pde1c
Clcn3	Ddx56	Utp20
Pde1c	Tmed4	9330159F19Rik
Utp20	Bid	Cul3
9330159F19Rik	Ralb	Sst
Cul3	Ppp1cc	Large1
Sst	Dnajb11	Ddx56

Large1	Tbccd1	Tmed4
Ddx56	Zfp324	Col26a1
Tmed4	Gab2	Bid
Col26a1	Coro1c	Ralb
Bid	Tax1bp1	Ppp1cc
Ralb	Ndrg2	Dnajb11
Ppp1cc	Mettl17	Tbccd1
Dnajb11	Arhgef40	Clec11a
Tbccd1	Pnpla6	Zfp324
Clec11a	Mcoln1	Gab2
Zfp324	Arhgef18	Coro1c
Gab2	Pkn2	Tax1bp1
Coro1c	Etfb	Ndrg2
Tax1bp1	Stxbp2	Mettl17
Ndrg2	Sgce	Arhgef40
Mettl17	Chn2	Pnpla6
Arhgef40	Wwox	Mcoln1
Pnpla6	Slbp	Arhgef18
Mcoln1	Arid3b	Pkn2
Arhgef18	Cnn2	Etfb
Pkn2	Polr2e	Stxbp2
Cd33	Myo9b	Sgce
Etfb	Hdac9	Chn2
Stxbp2	Mtfp1	Wwox
Sgce	Rab23	Slbp
Chn2	Rab11a	Arid3b
Wwox	Eif2b2	Cnn2
Slbp	Dlst	Polr2e
Arid3b	Ulk2	Myo9b
Cnn2	Dgkq	Hdac9
Polr2e	Chmp2b	Adgre1
Myo9b	Plod3	Mtfp1
Hdac9	Ap1s1	Rab23
Adgre1	Srpk1	Rab11a
Mtfp1	Lbr	Eif2b2
Rab23	Nes	Dlst
Rab11a	Bcan	Pgf
Eif2b2	Hapln2	Ulk2
Dlst	Prcc	Dgkq
Pgf	Mettl25b	Grap
Ulk2	Hdgf	Chmp2b
Dgkq	Slc25a18	Plod3
Grap	Thop1	Ap1s1
Chmp2b	Apba3	Srpk1
Plod3	Matk	Lbr
Ap1s1	Pias4	Nes
Srpk1	Map2k1	Bcan
Lbr	Sgta	Hapln2
Nes	Tmem242	Prcc
Bcan	Dtx2	Mettl25b
Hapln2	Rasa4	Hdgf
Prcc	Syt5	Slc25a18
Mettl25b	Hnrnpa2b1	Thop1

Hdgf	Yju2b	Apba3
Slc25a18	Mri1	Matk
Thop1	Prkacb	Pias4
Apba3	Sgsh	Map2k1
Matk	Chd5	Sgta
Pias4	Cstb	Tmem242
Map2k1	Sh2b2	Dtx2
Sgta	Pex5	Hspb1
Tmem242	Jkamp	Rasa4
Dtx2	Slc1a2	Syt5
Hspb1	Eif2ak4	Hnrnpa2b1
Rasa4	Wdr1	Yju2b
Syt5	Ndrg1	Mri1
Hnrnpa2b1	Man2b1	Prkacb
Yju2b	Klf5	Sgsh
Mri1	Wdr83	Chd5
Prkacb	Prdx2	Cstb
Sgsh	Polr2a	Sh2b2
Chd5	Senp3	Pex5
Cstb	Plekha8	Jkamp
Sh2b2	Dnah2	Slc1a2
Pex5	Ripk4	Eif2ak4
Jkamp	Ufd1	Wdr1
Cd44	Zfp287	Slc2a9
Slc1a2	Prlr	Ndrg1
Eif2ak4	Letm1	Man2b1
Wdr1	Ubqln1	Klf5
Ccn4	Cadm3	Wdr83
Ndrg1	Txn2	Prdx2
Man2b1	Slc1a6	Polr2a
Klf5	Slc1a3	Senp3
Wdr83	Crbn	Plekha8
Prdx2	Msh6	Ufd1
Polr2a	Fbxo11	Zfp287
Senp3	Tbl2	Prlr
Plekha8	Bud23	Letm1
Ufd1	Nid1	Ubqln1
Zfp287	Mprip	Cadm3
Letm1	Cic	Txn2
Ubqln1	Pafah1b3	Slc1a6
Cadm3	Prkaca	Slc1a3
Txn2	Ddx39a	Crbn
Slc1a6	Dnajb1	Msh6
Slc1a3	Usp40	Fbxo11
Crbn	Kbtbd4	Tbl2
Msh6	Celf1	Bud23
Fbxo11	Ndufs3	Nid1
Tbl2	Por	Mcm5
Bud23	Igf1r	Hmox1
Nid1	Insr	Mprip
Mcm5	Trim28	Cic
Hmox1	Ube2m	Pafah1b3
Mprip	Adcy9	Prkaca

Cic	Mef2c	Ddx39a
Pafah1b3	Ctr9	Dnajb1
Prkaca	Eif4g2	Usp40
Ddx39a	Pcyt1a	Kbtbd4
Dnajb1	Zfp592	Celf1
Usp40	Psmd4	Ndufs3
Kbtbd4	Snx6	Por
Celf1	Mthfd2	Igf1r
Ndufs3	Kit	Insr
Por	Tomm40l	Trim28
Igf1r	Pan2	Ube2m
Insr	Cs	Adcy9
Trim28	Ampd3	Mef2c
Ube2m	Bcas2	Ctr9
Adcy9	Ctcf	Eif4g2
Mef2c	Pard6a	Irag1
Ctr9	Tfap4	Pcyt1a
Eif4g2	Ranbp1	Zfp592
Pcyt1a	Rfx5	Psmd4
Zfp592	Psmb4	Snx6
Psmd4	Slc30a4	Mthfd2
Snx6	Sqor	Kit
Mthfd2	Bloc1s6	Tomm40l
Kit	Metap1	Pan2
Tomm40l	Gpr108	Cs
Pan2	Rsl1d1	Ampd3
Cs	Apc	Bcas2
Ampd3	Reep5	Ctcf
Bcas2	Ergic3	Pard6a
Ctcf	Uqcc1	Pvalb
Pard6a	Ncoa2	Tfap4
Pvalb	Trh	Ranbp1
Tfap4	Nr2c2	Rfx5
Ranbp1	Nr2c1	Psmb4
Rfx5	Smpd4	Slc30a4
Psmb4	Pex1	Sqor
Slc30a4	Kctd20	Bloc1s6
Sqor	Ctns	Metap1
Bloc1s6	Shpk	Gpr108
Metap1	Ephb3	Rsl1d1
Gpr108	Rcn1	Apc
Rsl1d1	Trap1	Reep5
Apc	Naa60	Ergic3
Reep5	1700037C18Rik	Uqcc1
Ergic3	Ankrd13d	Ncoa2
Uqcc1	Tpr	Nr2c2
Ncoa2	Odr4	Nr2c1
Trh	Dhx34	Smpd4
Nr2c2	Kptn	Pex1
Nr2c1	Napa	Otx1
Smpd4	Sra1	Kctd20
Pex1	Atp5mc1	Ctns
Otx1	Snf8	P2rx5

Kctd20	Tbcb	Shpk
Ctns	Inpp5k	Ephb3
Shpk	Crkl	Tuft1
Ephb3	Clint1	Rcn1
Rcn1	Cdkal1	Trap1
Trap1	Htra1	Naa60
Naa60	Zbtb17	1700037C18Rik
1700037C18Rik	Epor	Ankrd13d
Ankrd13d	Ccdc159	Tpr
Tpr	Mob1b	Odr4
Odr4	Vax1	Dhx34
Dhx34	Atp6v1b2	Kptn
Kptn	Eps15l1	Napa
Napa	Tep1	Sra1
Sra1	Ttc5	Atp5mc1
Atp5mc1	Osgep	Snf8
Snf8	Aamp	Tbcb
Tbcb	Tmbim1	Inpp5k
Inpp5k	Arpc2	Crkl
Crkl	Kmt2b	Clint1
Clint1	Tmem147	Cdkal1
Cdkal1	Rps9	Htra1
Htra1	Tfpt	Zbtb17
Zbtb17	Susd2	Epor
Fam131c	Crip2	Ccdc159
Epor	Crip1	Mob1b
Ccdc159	Cbfa2t3	Atp6v1b2
Mob1b	Fbln1	Eps15l1
Atp6v1b2	Pgrmc1	Tep1
Eps15l1	Gcat	Ttc5
Tep1	Tek	Osgep
Ttc5	Elovl1	Aamp
Osgep	Med8	Tmbim1
Aamp	Hyi	Arpc2
Tmbim1	Adamts4	Kmt2b
Arpc2	Pfdn2	Tmem147
Kmt2b	Rnf114	Rps9
Tmem147	Steep1	Tfpt
Rps9	Neurl1a	Susd2
Tfpt	Srm	Crip2
Susd2	Rbm14	Cbfa2t3
Crip2	Zdhhc24	Fbln1
Cbfa2t3	Bbs1	Pgrmc1
Fbln1	Ndor1	Gcat
Pgrmc1	Nsmf	Tek
Gcat	Pdk1	Elovl1
Tek	Ptbp1	Med8
Elovl1	Mvd	Adamts4
Med8	Cyba	Nectin4
Hyi	Itih3	Pfdn2
Adamts4	Stimate	Rnf114
Nectin4	Sfmbt1	Steep1
Pfdn2	Atp7b	Neurl1a

Rnf114
Steep1
Neurl1a
Srm
Rbm14
Zdhhc24
Bbs1
Ndor1
Nsmf
Pdk1
Ptbp1
Mvd
Cyba
Itih3
Stimate
Sfmbt1
Rundc3a
Slc4a3
Cdt1
Runx1t1
Aprt
Gtf2h1
Abhd1
Slc5a6
Aplp1
Qrich1
P4htm
Usp19
Pola1
Cdc42
Pknox1
D130043K22Rik
Acot13
Zfp184
Cdk4
B4galnt1
Mettl1
Tspan31
Kif5b
Saal1
Cnp
Sulf2
Sod2
Tmco6
Stambp
Ezh1
Hap1
P3h4
Ctnnb1
Eif1b
Chrd
Psmd2
Syngr3
Rundc3a
Slc4a3
Runx1t1
Aprt
Gtf2h1
Abhd1
Slc5a6
Aplp1
Qrich1
P4htm
Usp19
Pola1
Cdc42
Pknox1
D130043K22Rik
Acot13
Cdk4
B4galnt1
Mettl1
Tspan31
Kif5b
Saal1
Cnp
Odad4
Sulf2
Sod2
Tmco6
Stambp
Ezh1
Hap1
P3h4
Ctnnb1
Eif1b
Chrd
Psmd2
Syngr3
Vars1
Abhd16a
Neu1
Ddah2
Clic1
Atp1a2
Stx1a
Zfp775
Mrpl49
Mark3
Gatad1
Pcdha10
M6pr
Lrrc8a
Ppp2r1a
Fance
Tinf2
Srm
Rbm14
Zdhhc24
Bbs1
Ndor1
Nsmf
Pdk1
Ptbp1
Mvd
Cyba
Itih3
Stimate
Sfmbt1
Atp7b
Rundc3a
Slc4a3
Runx1t1
Aprt
Gtf2h1
Hfe
Abhd1
Slc5a6
Aplp1
Qrich1
P4htm
Usp19
Pola1
Cdc42
Pknox1
D130043K22Rik
Acot13
Cdk4
B4galnt1
Mettl1
Tspan31
Kif5b
Saal1
Cnp
Sulf2
Sod2
Tmco6
Stambp
Ezh1
Hap1
P3h4
Ctnnb1
Eif1b
Chrd
Psmd2
Syngr3
Vars1
Abhd16a
Neu1

Vars1	Hapln4	Ddah2
Abhd16a	Dus3l	Clic1
Neu1	Gtpbp3	Lsm2
Ddah2	Tgfbr1	Atp1a2
Clic1	Homer1	Stx1a
Lsm2	Gabrb2	Zfp775
Atp1a2	Arpp19	Scube2
Stx1a	Bcl2l1	Mrpl49
Zfp775	Khsrp	Mark3
Mrpl49	Dio2	Gatad1
Mark3	Ccdc124	Pcdha10
Gatad1	Cct4	M6pr
Pcdha10	Trappc2b	Lrrc8a
M6pr	Cpt1c	Ppp2r1a
Lrrc8a	Zfp655	Fance
Ppp2r1a	Rhoa	Tinf2
Fance	Zmiz1	Hapln4
Tinf2	Ankrd26	Dus3l
Hapln4	Aldh16a1	Gtpbp3
Dus3l	Hnrnpa0	Tgfbr1
Gtpbp3	Hnrnph1	Homer1
Tgfbr1	Ift43	Rad51c
Homer1	Id3	Gabrb2
Rad51c	Arid1a	Cav1
Gabrb2	Ctsd	Arpp19
Cav1	Rplp1	Bcl2l1
Arpp19	Hmgcll1	Khsrp
Bcl2l1	Ttc9b	Dio2
Khsrp	Abhd8	Ccdc124
Dio2	Ift22	Cct4
Ccdc124	Fzd3	Trappc2b
Cct4	Mid1ip1	Cpt1c
Trappc2b	Ap2s1	Zfp655
Cpt1c	Fgfrl1	Rhoa
Zfp655	Emc10	Zmiz1
Rhoa	Clstn3	Ankrd26
Zmiz1	Fbxw9	Aldh16a1
Ankrd26	Fnbp4	Hnrnpa0
Aldh16a1	Cers4	Prrg2
Hnrnpa0	Scrn3	Hnrnph1
Prrg2	Phax	Ift43
Hnrnph1	Tle1	Id3
Ift43	Relt	Arid1a
Id3	Snrpb2	Crlf1
Tcap	Ubc	Ctsd
Arid1a	Prpf31	Rplp1
Ctsd	Sertad1	Hmgcll1
Rplp1	Carhsp1	Ttc9b
Hmgcll1	Elk3	Abhd8
Ttc9b	Herpud2	Ift22
Abhd8	Rdh13	Fzd3
Ift22	Nutf2	Mid1ip1
Fzd3	Arpc5	Ap2s1

Mid1ip1	Elavl2	Fgfrl1
Ap2s1	Pou2f2	Brme1
Fgfrl1	Mgst1	Fhl2
Brme1	Nfib	Emc10
Fhl2	Ubqln4	Clstn3
Emc10	Rbfox1	Fbxw9
Clstn3	Rps18	Fnbp4
Fbxw9	Rpl10	Cers4
Fnbp4	Rps15a	Scrn3
Cers4	Ncaph2	Phax
Scrn3	Hipk1	Tle1
Phax	Gprc5b	Relt
Tle1	Man1a2	Snrpb2
Relt	Acyp1	Ubc
Snrpb2	Hdac5	Prpf31
Ubc	Rala	Sertad1
Prpf31	Vdac3	Carhsp1
Sertad1	Vps72	Elk3
Carhsp1	Gabpa	Herpud2
Elk3	Bmp7	Rdh13
Herpud2	Dynll1	Nutf2
Rdh13	Pdcl	Arpc5
Nutf2	Tmem184b	Elavl2
Arpc5	Nf2	Pou2f2
Elavl2	Cabp7	Nfib
Pou2f2	Zmat5	Ubqln4
Nfib	Ewsr1	Rbfox1
Ubqln4	Ap1b1	Rps18
Rbfox1	Bcl2l13	Rpl10
Rps18	Lnpk	Rps15a
Rpl10	Fam163b	Ncaph2
Rps15a	Rarres2	Hipk1
Ncaph2	Pttg1ip	Gprc5b
Hipk1	Trpm2	Man1a2
Gprc5b	Ube2g2	Acyp1
Man1a2	Syn2	Hdac5
Acyp1	Elk1	Rala
Hdac5	Nav1	Vdac3
Rala	Tnpo1	Vps72
Vdac3	Rnmt	Gabpa
Vps72	Srp14	Bmp7
Gabpa	Cdk9	Dynll1
Bmp7	Tor2a	Pdcl
Dynll1	Fpgs	Tmem184b
Pdcl	Mrtfb	Nf2
Tmem184b	Cbx5	Cabp7
Nf2	Sardh	Zmat5
Cabp7	Vav2	Ewsr1
Zmat5	Ppp2cb	Ap1b1
Ewsr1	Dmap1	Bcl2l13
Ap1b1	Mcu	Lnpk
Bcl2l13	Bcr	Fam163b
Lnpk	Kcnd1	Rarres2

Fam163b	Tfcp2	Pttg1ip
Pttg1ip	Pou6f1	Trpm2
Trpm2	Ubp1	Ube2g2
Ube2g2	Cilk1	Syn2
Met	Sdhb	Elk1
Syn2	Snap47	Nav1
Elk1	Kdsr	Tnpo1
Nav1	Vps4b	Rnmt
Tnpo1	Rps25	Srp14
Rnmt	Tafazzin	Cdk9
Srp14	Kif19a	Tor2a
Cdk9	Aldh3a2	Fpgs
Tor2a	Zmynd10	Mrtfb
Fpgs	Tmem115	Cbx5
Mrtfb	Hyal2	Apobec3
Cbx5	Ifrd2	Sardh
Sardh	Hyal1	Vav2
Vav2	Tusc2	Tex15
Ppp2cb	Nprl2	Ppp2cb
G0s2	Slc38a3	G0s2
Dmap1	Cacna2d2	Dmap1
Mcu	Rassf1	Mcu
Bcr	Rnf112	Bcr
Fxyd5	Slc3a2	Kcnd1
Kcnd1	Nxf1	Tfcp2
Tfcp2	Stx5a	Pou6f2
Pou6f1	Spire2	Pou6f1
Ubp1	Prox1	Ubp1
Cilk1	Raver1	Cilk1
Sdhb	Bltp2	Sdhb
Snap47	AI597479	Snap47
Kdsr	Tmem86a	Kdsr
Vps4b	Rdm1	Vps4b
Rps25	Nedd8	Rps25
Tafazzin	Gosr1	Tafazzin
Kif19a	Mrpl52	Kif19a
Aldh3a2	Kansl3	Aldh3a2
Zmynd10	Myt1	Tmem115
Tmem115	Faf1	Hyal2
Hyal2	Mettl16	Ifrd2
Ifrd2	Pigyl	Hyal1
Hyal1	Rbm25	Tusc2
Tusc2	Psen2	Nprl2
Nprl2	Plcd1	Slc38a3
Slc38a3	Fads1	Cacna2d2
Cacna2d2	Lmbr1	Rassf1
Rassf1	Cars1	Epn3
Rnf112	Gabra1	Rnf112
Slc3a2	Apip	Slc3a2
Nxf1	Pdhx	Nxf1
Stx5a	Vac14	Stx5a
Spire2	Akt1s1	Spire2
Prox1	Tbrg1	Prox1

Raver1	Adss1	Raver1
Bltp2	Cfap161	Bltp2
AI597479	Brf1	AI597479
Tmem86a	Odc1	Tmem86a
Rdm1	Thg1l	Ifi35
Nedd8	Adam19	Rdm1
Gosr1	Pabpc4	Nedd8
Mrpl52	Plin5	Gosr1
Kansl3	Sugp1	Mrpl52
Myt1	Dhdh	Kansl3
Faf1	Zfp790	Myt1
Gm266	Fsd1	Faf1
Mettl16	Fuz	Mettl16
Pigyl	Sptbn4	Pigyl
Rbm25	Pgam1	Rbm25
Psen2	Evi5	Psen2
Plcd1	Evi5l	Plcd1
Fads1	Snapc2	Fads1
Lmbr1	Git1	Lmbr1
Cars1	Gltp	Cars1
Wnt2	Bnip2	Wnt2
Gabra1	Ccnt1	Gabra1
Grid2ip	Scarf2	Grid2ip
Apip	Brms1l	Apip
Pdhx	Med15	Pdhx
Vac14	Dhdds	Vac14
Akt1s1	Ubxn11	Akt1s1
Tbrg1	Tjap1	Tbrg1
Adss1	Rpl15	Adss1
Brf1	Tmem167	Brf1
Odc1	Mplkip	Vipr2
Thg1l	Rpa3	Odc1
Adam19	Tnpo3	Thg1l
Pabpc4	Ttll5	Adam19
Zfp296	Zfp715	Pabpc4
Sugp1	Cdh23	Sugp1
Dhdh	Rps5	Dhdh
Zfp790	Adgrl1	Zfp790
Fsd1	Amotl1	Fsd1
Fuz	Etv5	Fuz
Sptbn4	Gfod2	Sptbn4
Pgam1	Enkd1	Pgam1
Evi5	Atp6v0d1	Evi5
Evi5l	Ptprs	Evi5l
Snapc2	Slc41a1	Snapc2
Git1	Iglon5	Git1
Gltp	Zbtb47	Gltp
Bnip2	Nelfb	Bnip2
Ccnt1	Tmem175	Ccnt1
Scarf2	Bcas1	Brms1l
Brms1l	Tango2	Med15
Med15	Ndufs2	Dhdds
Dhdds	Atraid	Ubxn11

Crybg2	Cad	Tjap1
Ubxn11	Sh3bp5l	Rpl15
Tjap1	Atad1	Tmem167
Rpl15	Pten	Mplkip
Tmem167	Pea15a	Rpa3
Mplkip	Timm23	Tnpo3
Rpa3	Trnt1	Ttll5
Tnpo3	Ehmt2	Zfp715
Ttll5	Elof1	Cdh23
Zfp715	Med16	Rps5
Rps5	St3gal1	Adgrl1
Adgrl1	Tmem259	Amotl1
Amotl1	Rnf170	Etv5
Etv5	Clip3	Gfod2
Gfod2	Dedd	Enkd1
Enkd1	Nit1	Atp6v0d1
Atp6v0d1	Prdm15	Ptprs
Ptprs	Rnf168	Slc41a1
Slc41a1	Dynlt2b	Iglon5
Iglon5	Chp1	Zbtb47
Zbtb47	Klhl3	Nelfb
Nelfb	Tvp23b	Tmem175
Tmem175	Dnajc7	Bcas1
Bcas1	Cacybp	Tango2
Tango2	Cluap1	Aldh1a2
Aldh1a2	Zswim7	Ndufs2
Ndufs2	Pigl	Atraid
Atraid	Ndufa2	Cad
Cad	Pam16	Sh3bp5l
Sh3bp5l	Glis2	Atad1
Atad1	Cox6c	Pten
Pten	Bicc1	Pea15a
Pea15a	Ube2z	Timm23
Timm23	Tmem87b	Trnt1
Trnt1	Anapc1	Ehmt2
Ehmt2	Mertk	Elof1
Elof1	Tsg101	Med16
Med16	Hps5	St3gal1
St3gal1	Map3k4	Tmem259
Tmem259	Piezo1	Rnf170
Rnf170	Rnf166	Clip3
Clip3	Ankrd28	Dedd
Dedd	Ankrd52	Nit1
Nit1	Srp19	Prdm15
Prdm15	Wdfy2	Rnf168
Rnf168	Rbsn	Dynlt2b
Dynlt2b	Mrps25	Chp1
Chp1	Dguok	Klhl3
Klhl3	Camta1	Tvp23b
Tvp23b	Csf1	Dnajc7
Dnajc7	Strip1	Cacybp
Cacybp	Kif1a	Cluap1
Cluap1	Slc25a11	Zswim7

Zswim7	Cmc2	Pigl
Pigl	Chfr	Ndufa2
Ndufa2	Tex261	Pam16
Pam16	Fam120b	Glis2
Glis2	Tbp	Cox6c
Cox6c	Psmb1	Bicc1
Bicc1	Pdcd2	Ube2z
Ube2z	Dll1	Tmem87b
Tmem87b	Nol3	Anapc1
Anapc1	Fhod1	Mertk
Mertk	Slc9a5	Tsg101
Tsg101	Stc1	Hps5
Hps5	Matcap1	Map3k4
Map3k4	Tppp3	Piezo1
Piezo1	Msh3	Rnf166
Rnf166	Tmem208	Ankrd28
Ankrd28	E2f4	Ankrd52
Ankrd52	Surf4	Srp19
Srp19	Surf2	Wdfy2
Wdfy2	Dnajb9	Rbsn
Rbsn	Naf1	Mrps25
Mrps25	Yes1	Dguok
Dguok	Ppp1cb	Camta1
Camta1	Gorasp2	Csf1
Csf1	Tsen15	Strip1
Strip1	Efr3a	Kif1a
Kif1a	Trappc2l	Slc25a11
Slc25a11	Acsf3	Cmc2
Cmc2	Ddx19a	Chfr
Chfr	Galns	Tex261
Tex261	Gata2	Fam120b
Fam120b	Entpd2	Tbp
Tbp	Rabl6	Psmb1
Psmb1	Ptgds	Pdcd2
Pdcd2	Edf1	Dll1
Dll1	Npdc1	Nol3
Nol3	Fbxw5	Fhod1
Slc9a5	Slc25a13	Slc9a5
Matcap1	Ube2i	Stc1
Tppp3	Tsr3	Matcap1
Msh3	Unkl	Tppp3
Adamts13	Lrrk1	Msh3
Tmem208	Actn1	Tmem208
E2f4	Sirt2	E2f4
Surf4	Hnrnpl	Surf4
Surf2	Nolc1	Surf2
Dnajb9	Casd1	Dnajb9
Naf1	Cnksr3	Naf1
Yes1	Mtmr1	Yes1
Ppp1cb	Hmgb3	Ppp1cb
Gorasp2	Map2	Gorasp2
Tsen15	Cyp2j9	Tsen15
Efr3a	Abca1	Efr3a

Trappc2l	Nipsnap3b	Trappc2l
Acsf3	Lage3	Acsf3
Ddx19a	Ubl4a	Ddx19a
Galns	Gdi1	Galns
Gata2	Sash1	Gata2
Entpd2	Gadd45b	Entpd2
Rabl6	Zdhhc12	Rabl6
Ptgds	Endog	Ptgds
Edf1	Golga7	Edf1
Npdc1	Xk	Npdc1
Fbxw5	Pcolce2	Fbxw5
Ube2i	Clpx	Ube2i
Tsr3	Trabd	Tsr3
Unkl	Dennd6b	Unkl
Lrrk1	Cd83	Lrrk1
Aldh1a3	Atf6b	Actn1
Actn1	Notch4	Sirt2
Sirt2	Ppt2	Hnrnpl
Hnrnpl	Prrt1	Nolc1
Nolc1	Rnf5	Casd1
Casd1	Fam163a	Cnksr3
Cnksr3	Cacfd1	Mtmr1
Mtmr1	Hivep2	Hmgb3
Hmgb3	Arnt	Map2
Map2	Mocs2	Cyp2j9
Cyp2j9	Nat9	Abca1
Abca1	Lpl	Nipsnap3b
Nipsnap3b	Atp6v0e	Lage3
Lage3	Zfp318	Ubl4a
Ubl4a	Ttbk1	Gdi1
Gdi1	Srf	Sash1
Sash1	Lama5	Gadd45b
Gadd45b	Steap2	Zdhhc12
Zdhhc12	Hspa8	Endog
Endog	Serac1	Golga7
Golga7	Pdzd11	Xk
Xk	Psma2	Pcolce2
Clpx	Mrpl32	Clpx
Trabd	Setdb1	Trabd
Dennd6b	Arnt2	Dennd6b
Cd83	Prune1	Cd83
Atf6b	Cers2	Atf6b
Notch4	Capza2	Notch4
Ppt2	Plekho1	Ppt2
Prrt1	Vps45	Prrt1
Rnf5	Prpf3	Rnf5
Fam163a	Anp32e	Fam163a
Cacfd1	Aph1a	Cacfd1
Hivep2	Tab2	Hivep2
Arnt	Ppil4	Arnt
Mocs2	Cnih1	Mocs2
Nat9	Eps8	Nat9
Lpl	Med22	Lpl

Atp6v0e	Surf1	Atp6v0e
Zfp318	Med28	Zfp318
Ttbk1	Qdpr	Ttbk1
Srf	Tnr	Srf
Lama5	Sqstm1	Lama5
Steap2	Nfe2l2	Steap2
Hspa8	Rxrg	Hspa8
Serac1	Rxra	Serac1
Pdzd11	Adamtsl4	Pdzd11
Psma2	Fcrl2	Psma2
Mrpl32	Prpsap1	Mrpl32
Setdb1	Fam184b	Setdb1
Arnt2	Lcorl	Arnt2
Prune1	Lta4h	Prune1
Cers2	Dstn	Cers2
Capza2	Macroh2a1	Capza2
Plekho1	Gtf2ird2	Plekho1
Vps45	Bola1	Vps45
Prpf3	Castor2	Prpf3
Anp32e	Ncf1	Anp32e
Aph1a	Adss2	Aph1a
Tab2	Cacna1d	Tab2
Ppil4	Chdh	Ppil4
Cnih1	Actr8	Cnih1
Eps8	Lrrc27	Eps8
Med22	Stk32c	Med22
Surf1	Fnta	Surf1
Med28	Mtrex	Med28
Qdpr	Lbp	Qdpr
Tnr	Celsr1	Tnr
Sqstm1	Stard13	Sqstm1
Nfe2l2	Tenm1	Nfe2l2
Rxrg	Camk1g	Rxrg
Rxra	Utp25	Rxra
Adamtsl4	Syt14	Adamtsl4
Fcrl2	Lonrf3	Fcrl2
Prpsap1	Atp5f1e	Prpsap1
Fam184b	Nelfcd	Fam184b
Lcorl	Ctsz	Lcorl
Lta4h	Prelid3b	Lta4h
Dstn	Sertad4	Dstn
Macroh2a1	Ube2a	Macroh2a1
Gtf2ird2	Slc25a5	Gtf2ird2
Bola1	Ppdpf	Bola1
Castor2	Kcnq2	Castor2
Fcgr1	Eef1a2	Ncf1
Ncf1	Pls3	Adss2
Adss2	Mpped2	Cacna1d
Cacna1d	Nkap	Chdh
Chdh	Ndufa1	Actr8
Actr8	E2f3	Lrrc27
Lrrc27	Cr1l	Stk32c
Stk32c	Ppfibp1	Fnta

Fnta	Cd34	Mtrex
Mtrex	Plgrkt	Celsr1
Celsr1	Gtf3a	Fli1
Fli1	Mtif3	Stard13
Stard13	Lnx2	Tenm1
Tenm1	Dyrk3	Camk1g
Camk1g	Mapkapk2	Utp25
Utp25	Lamp2	Hsd11b1
Hsd11b1	Atxn10	Syt14
Syt14	Foxred2	Lonrf3
Lonrf3	Eif3d	Atp5f1e
Atp5f1e	H3f3b	Nelfcd
Nelfcd	Nup50	Ctsz
Ctsz	Phf21b	Prelid3b
Prelid3b	Ift27	Sertad4
Sertad4	Pacsin2	Ube2a
Ube2a	Ttll12	Slc25a5
Slc25a5	Scube1	Ppdpf
Ppdpf	Tox4	Kcnq2
Kcnq2	Mrps18c	Eef1a2
Eef1a2	Sulf1	Col20a1
Pls3	Srsf6	Pls3
Mpped2	Plcg1	Mpped2
Nkap	Kctd2	Nkap
Ndufa1	Kctd5	Ndufa1
E2f3	Etaa1	E2f3
Cr1l	Matn4	Cr1l
Ppfibp1	Sdc4	Ppfibp1
Cd34	Il13ra1	Cd46
Plgrkt	Nbr1	Cd34
Gtf3a	Cyth1	Plgrkt
Mtif3	Rnd3	Gtf3a
Lnx2	Cntnap1	Mtif3
Dyrk3	Nt5c3b	Lnx2
Mapkapk2	Coa3	Dyrk3
Lamp2	Med24	Mapkapk2
Atxn10	Psmd3	Lamp2
Foxred2	Exosc10	Atxn10
Eif3d	Glod4	Foxred2
H3f3b	Vps53	Eif3d
Nup50	Taok1	H3f3b
Phf21b	Dnttip1	Nup50
Ift27	Acot8	Phf21b
Pacsin2	Mpp2	Ift27
Ttll12	Vtn	Pacsin2
Scube1	Nlk	Ttll12
Tox4	Traf4	Scube1
Mrps18c	Aldoc	Tox4
Sulf1	Stac2	Mrps18c
Srsf6	Rpl19	Srsf6
Plcg1	Cacnb4	Plcg1
Kctd2	Plxdc1	Kctd2
Kctd5	Arl5b	Kctd5

Etaa1	Zfp207	Etaa1
Matn4	Psmd11	Matn4
Sdc4	C1qtnf1	Sdc4
Il13ra1	Timp2	Nbr1
Nbr1	Zc3h18	Cyth1
Cyth1	Top2b	Rnd3
Rnd3	Rarb	Cntnap1
Cntnap1	Igfbp4	Nt5c3b
Nt5c3b	Suz12	Coa3
Coa3	Atad5	Med24
Med24	Crlf3	Psmd3
Psmd3	Tnfaip1	Exosc10
Exosc10	Abr	Glod4
Glod4	Rab11fip4	Vps53
Vps53	Slc35c2	Taok1
Taok1	Zfp334	Dnttip1
Dnttip1	Elmo2	Acot8
Acot8	Wsb1	Mpp2
Mpp2	Ttpal	Vtn
Vtn	Rhot1	Nlk
Nlk	Rhbdl3	Traf4
Traf4	Serinc3	Aldoc
Aldoc	Tha1	Stac2
Stac2	Pgs1	Rpl19
Rpl19	Pigt	Nek8
Nek8	Etv4	Cacnb4
Cacnb4	Dbndd2	Plxdc1
Plxdc1	Slc12a5	Arl5b
Arl5b	Ghdc	Zfp207
Zfp207	Pltp	Psmd11
Psmd11	Slc12a7	C1qtnf1
C1qtnf1	Ctsa	Timp2
Timp2	Zswim1	Zc3h18
Zc3h18	Slc12a4	Top2b
Top2b	Myo1c	Rarb
Rarb	Crk	Igfbp4
Igfbp4	Cox7c	Suz12
Suz12	Pitpna	Atad5
Atad5	Mlx	Crlf3
Crlf3	Retreg3	Tnfaip1
Tnfaip1	Rab5a	Abr
Abr	Nkiras2	Rab11fip4
Rab11fip4	Ppp2r5c	Slc35c2
Slc35c2	Ift52	Zfp334
Zfp334	Eya2	Elmo2
Elmo2	B4galt5	Wsb1
Wsb1	Gdap1l1	Ttpal
Ttpal	Cadps2	Rhot1
Rhot1	Ddx27	Rhbdl3
Rhbdl3	Cyth3	Serinc3
Serinc3	Cyth4	Tha1
Tha1	Rac3	Pgs1
Pgs1	Rrp7a	Pigt

Afmid	Cyb5r3	Etv4
Pigt	Ints2	Dbndd2
Etv4	Med13l	Mmp9
Dbndd2	H2bc4	Slc12a5
Slc12a5	Mafk	Ghdc
Ghdc	Med1	Pltp
Pltp	Erbb3	Slc12a7
Slc12a7	Stard3	Ctsa
Ctsa	Mfng	Zswim1
Zswim1	Vmp1	Slc12a4
Slc12a4	Uchl5	Myo1c
Myo1c	Glrx2	Crk
Crk	Ro60	Cox7c
Cox7c	Stk4	Pitpna
Pitpna	Pmp22	Mlx
Mlx	Psmb6	Retreg3
Retreg3	Spag7	Rab5a
Rab5a	Pfn1	Nkiras2
Nkiras2	Tomm34	Ppp2r5c
Ppp2r5c	Ywhab	Ift52
Ift52	Ksr1	B4galt5
B4galt5	Gpx3	Gdap1l1
Gdap1l1	Anxa6	Cadps2
Cadps2	Zkscan6	Ddx27
Ddx27	Kpna2	Cyth3
Cyth3	Smurf2	Cyth4
Cyth4	Cep95	Rac3
Rac3	Vezf1	Rrp7a
Rrp7a	Cuedc1	Cyb5r3
Cyb5r3	Srsf1	Ints2
Ints2	Shroom1	Med13l
Med13l	Kif3a	H2bc4
H2bc4	Septin8	Mafk
Baiap2l2	Mtmr4	Med1
Mafk	Mrm1	Erbb3
Med1	Mapt	Stard3
Erbb3	Kansl1	Mfng
Stard3	Gid4	Vmp1
Mfng	Myo1b	Uchl5
Vmp1	Dhx40	Glrx2
Uchl5	Ypel2	Ro60
Glrx2	Akap1	Stk4
Ro60	Nol11	Pmp22
Stk4	Derl2	Zcchc10
Pmp22	C1qbp	Psmb6
Zcchc10	Rpain	Spag7
Psmb6	6330403K07Rik	Pfn1
Spag7	Slc13a3	Tomm34
Pfn1	Kcnab3	Ywhab
Tomm34	Chd3	Ksr1
Ywhab	Kdm6b	Anxa6
Ksr1	Appbp2	Zkscan6
Gpx3	Adora2b	Kpna2

Anxa6	Ncor1	Smurf2
Zkscan6	Trpv2	Cep95
Kpna2	Cenpv	Vezf1
Smurf2	Pcgf2	Cuedc1
Cep95	Cwc25	Srsf1
Vezf1	Pip4k2b	Abi3
Cuedc1	Trim37	Shroom1
Srsf1	Ctdnep1	Kif3a
Abi3	Elp5	Septin8
Shroom1	Gabarap	Mtmr4
Kif3a	Phf23	Mrm1
Septin8	Acadvl	Mapt
Mtmr4	Dnah11	Kansl1
Mrm1	G3bp1	Gid4
Mapt	Atox1	Myo1b
Kansl1	Glra2	Dhx40
Gid4	Sparc	Ypel2
Myo1b	Mief2	Akap1
Dhx40	Crhr1	Nol11
Ypel2	Dusp14	Derl2
Akap1	Tada2a	C1qbp
Nol11	Pnpo	Rpain
Derl2	Cog1	6330403K07Rik
C1qbp	Cbx1	Slc13a3
Rpain	Cdk5rap3	Kcnab3
6330403K07Rik	Slc25a39	Chd3
Slc13a3	Sp2	Kdm6b
Kcnab3	Aatf	Appbp2
Chd3	Dync1h1	Wnt9b
Kdm6b	Pex12	Adora2b
Appbp2	Ndel1	Ncor1
Adora2b	Zbtb4	Trpv2
Ncor1	Mpdu1	Cenpv
Trpv2	Fxr2	Pcgf2
Cenpv	Atp5mc3	Cwc25
Pcgf2	Acsl1	Pip4k2b
Cwc25	Abca5	Trim37
Pip4k2b	Smyd4	Ctdnep1
Trim37	Zfyve27	Elp5
Ctdnep1	Avpi1	Gabarap
Elp5	Myh11	Phf23
Gabarap	Rad51d	Acadvl
Phf23	Pank3	G3bp1
Acadvl	Rars1	Atox1
G3bp1	Wwc1	Glra2
Atox1	Mrpl58	Sparc
Glra2	Fdxr	Mief2
Sparc	Sult4a1	Tbx3
Mief2	Mrpl45	Crhr1
Tbx3	Irf1	Dusp14
Crhr1	Slc22a5	Tada2a
Dusp14	P4ha2	Ikzf1
Tada2a	Arrb1	Pnpo

Ikzf1	Pelp1	Cog1
Pnpo	Med11	Cbx1
Cog1	Natd1	Cdk5rap3
Cbx1	Map2k3	Copz2
Cdk5rap3	Ywhah	Slc25a39
Copz2	Sart3	Sp2
Slc25a39	Nars2	Aatf
Sp2	Slc35b4	Dync1h1
Aatf	Dnah1	Pex12
Dync1h1	Dalrd3	Ndel1
Pex12	Fis1	Slc25a35
Ndel1	Plod1	Zbtb4
Slc25a35	Mfsd3	Mpdu1
Zbtb4	Slc25a22	Fxr2
Mpdu1	Atp6ap1	Atp5mc3
Fxr2	Scrn1	Acsl1
Atp5mc3	BC005537	Abca5
Cd68	Isyna1	Smyd4
Acsl1	Hars2	Zfyve27
Abca5	Cacng2	Avpi1
Smyd4	Tmem160	Myh11
Lsp1	Rab5c	Rad51d
Zfyve27	Mdh2	Pank3
Avpi1	H13	Rars1
Myh11	Rnf145	Wwc1
Rad51d	Scn1b	Mrpl58
Pank3	Atp6v1e1	Fdxr
Rars1	Etnppl	Sult4a1
Wwc1	Rps6kl1	Mrpl45
Mrpl58	Ppp1r12c	Irf1
Fdxr	Ahr	Slc22a5
Sult4a1	Map1s	P4ha2
Mrpl45	Tmem129	Arrb1
Irf1	Nop9	Pelp1
Slc22a5	Atp6v0a1	Med11
P4ha2	Zfp687	Natd1
Arrb1	Gdpd2	Map2k3
Pelp1	D8Ertd738e	Ywhah
Med11	Calm3	Sart3
Natd1	Cops3	Nars2
Map2k3	Fkbp8	Slc35b4
Ywhah	Ddx39b	Dnah1
Sart3	Gipc1	Dalrd3
Nars2	Tlcd1	Fis1
Slc35b4	Plscr3	Plod1
Dalrd3	Arhgef25	Rab3d
Fis1	Xab2	Mfsd3
Plod1	Cdc37	Slc25a22
Rab3d	Rab4a	Atp6ap1
Mfsd3	Cops6	Scrn1
Slc25a22	Ubb	BC005537
Atp6ap1	Ap4m1	Isyna1
Scrn1	Gyg1	Hars2

BC005537
Isyna1
Hars2
Cacng2
Tmem160
Rab5c
Mdh2
H13
Rnf145
Scn1b
Atp6v1e1
Chtf18
Rps6kl1
Ppp1r12c
Ahr
Map1s
Tmem129
Nop9
Atp6v0a1
Zfp687
Gdpd2
D8Ertd738e
Calm3
Cops3
Fkbp8
Ddx39b
Gipc1
Tlcd1
Plscr3
Arhgef25
Xab2
Cdc37
Rab4a
Trip10
Cops6
Ubb
Ap4m1
Gyg1
Rcn3
Slc6a8
Arid3a
Pdk4
Ubxn6
Mydgf
Cyb561
Sema6a
Ccdc12
Fmc1
Akt3
Mrpl24
Gle1
L3hypdh
Lyst
Rcn3
Slc6a8
Arid3a
Pdk4
Ubxn6
Mydgf
Cyb561
Sema6a
Ccdc12
Fmc1
Akt3
Mrpl24
Gle1
L3hypdh
Lyst
Slc35e1
Polr2i
Rmnd1
Ccdc170
Esr1
Mtrf1l
Rgs17
Hdac2
Rwdd1
Clvs2
Hey2
Stxbp5
Hint3
Katna1
Pcmt1
Lrp11
Mtres1
Sec63
Nr2e1
Snx3
Aig1
Pex3
Fuca2
Cep57l1
Ltv1
Plagl1
Cd164
Utrn
Smpd2
Mical1
Zbtb24
Grm1
Wasf1
Gtf3c6
Rev3l
Fyn
Lama4
Prep
Cacng2
Tmem160
Rab5c
Mdh2
H13
Rnf145
Scn1b
Atp6v1e1
Lhx9
Rps6kl1
Ppp1r12c
Ahr
Map1s
Tmem129
Nop9
Atp6v0a1
Zfp687
Gdpd2
D8Ertd738e
Calm3
Cops3
Fkbp8
Ddx39b
Gipc1
Tlcd1
Plscr3
Arhgef25
Xab2
Cdc37
Rab4a
Trip10
Cops6
Ubb
Ap4m1
Gyg1
Rcn3
Slc6a8
Arid3a
Pdk4
Ubxn6
Mydgf
Cyb561
Sema6a
Ccdc12
Fmc1
Akt3
Mrpl24
Gle1
L3hypdh
Lyst
Slc35e1
Polr2i
Krt10

Slc35e1
Polr2i
Rmnd1
Esr1
Vip
Mtrf1l
Rgs17
Hdac2
Rwdd1
Clvs2
Hey2
Stxbp5
Hint3
Trmt11
Katna1
Pcmt1
Lrp11
Mtres1
Sec63
Nr2e1
Snx3
Aig1
Adat2
Pex3
Fuca2
Cep57l1
Ltv1
Plagl1
Cd164
Utrn
Smpd2
Mical1
Zbtb24
Grm1
Wasf1
Gtf3c6
Slc16a10
Rev3l
Fyn
Tube1
Lama4
Prep
Tnfaip3
Perp
Arfgef3
Reps1
Fam184a
Asf1a
Gopc
Qrsl1
Rtn4ip1
Vta1
Smpdl3a
Tnfaip3
Arfgef3
Reps1
Fam184a
Asf1a
Gopc
Qrsl1
Rtn4ip1
Vta1
Smpdl3a
Reep3
Fabp7
Serinc1
Hsf2
Echdc1
Mgat4c
Ptprk
Nts
Dcbld1
Lrriq1
Slc6a15
Ccdc59
Lama2
Lin7a
Ppp1r12a
P4ha1
Septin10
Lims1
Zwint
Ube2d1
Slc17a8
Atp2b1
Rhobtb1
Arid5b
Actr6
Bltp3b
Poc1b
Dusp6
Tmpo
Kitl
Psen1
Sgk1
Cep290
Ikbip
Hbs1l
Epb41l2
Apaf1
Med23
Ahi1
Pde7b
Map7
Ccn2
Stx7
Iyd
Rmnd1
Vip
Mtrf1l
Rgs17
Hdac2
Rwdd1
Clvs2
Hey2
Stxbp5
Hint3
Trmt11
Katna1
Pcmt1
Lrp11
Mtres1
Sec63
Nr2e1
Snx3
Aig1
Adat2
Pex3
Fuca2
Cep57l1
Ltv1
Plagl1
Cd164
Utrn
Smpd2
Mical1
Zbtb24
Grm1
Wasf1
Gtf3c6
Slc16a10
Rev3l
Fyn
Tube1
Lama4
Popdc3
Prep
Tnfaip3
Arfgef3
Hebp2
Reps1
Fam184a
Asf1a
Gopc
Qrsl1
Rtn4ip1
Vta1
Smpdl3a
Reep3

Reep3	Moxd1	Fabp7
Fabp7	Pex7	Pkib
Pkib	Ifngr1	Serinc1
Serinc1	Cfap54	Hsf2
Hsf2	Cdk17	Rspo3
Rspo3	Ntn4	Echdc1
Echdc1	Fgd6	Mgat4c
Mgat4c	Ndufa12	Ptprk
Ptprk	Tmcc3	Dcbld1
Dcbld1	Cep83	Slc6a15
Lrriq1	Socs2	Ccdc59
Slc6a15	Nudt4	Lama2
Ccdc59	Nuak1	Lin7a
Lama2	Tcp11l2	Ppp1r12a
Lin7a	Rfx4	P4ha1
Ppp1r12a	Cry1	Septin10
P4ha1	Abtb3	Lims1
Septin10	Timp3	Zwint
Lims1	Hsp90b1	Ube2d1
Zwint	Ascl1	Dcn
Ube2d1	Washc3	Slc17a8
Dcn	Sirt1	Atp2b1
Atp2b1	Herc4	Rhobtb1
Rhobtb1	Hnrnph3	Arid5b
Arid5b	Rufy2	Actr6
Actr6	Ccar1	Bltp3b
Bltp3b	Ddx21	Poc1b
Poc1b	Ddx50	Dusp6
Dusp6	Vps26a	Tmpo
Tmpo	Supv3l1	Kitl
Kitl	Fam241b	Psen1
Psen1	Aifm2	Sgk1
Sgk1	Macroh2a2	Cep290
Cep290	Tysnd1	Ikbip
Ikbip	Sar1a	Hbs1l
Hbs1l	Ppa1	Epb41l2
Epb41l2	Npffr1	Apaf1
Apaf1	Eif4ebp2	Med23
Med23	Pald1	Ahi1
Ahi1	Sgpl1	Pde7b
Pde7b	Pcbd1	Map7
Map7	Unc5b	Stx7
Ccn2	Slc29a3	Moxd1
Stx7	Vsir	Pex7
Moxd1	Slc16a7	Ifngr1
Pex7	Lrig3	Cfap54
Ifngr1	Anapc16	Cdk17
Cfap54	Ddit4	Snrpf
Cdk17	Dnajb12	Ntn4
Snrpf	Micu1	Fgd6
Ntn4	Cand1	Ndufa12
Fgd6	Tbk1	Tmcc3
Ndufa12	Pno1	Cep83

Tmcc3	Plek	Socs2
Cep83	Srgap1	Nudt4
Socs2	Egfr	Nuak1
Nudt4	Usp15	Tcp11l2
Nuak1	Vps54	Rfx4
Tcp11l2	Tbc1d15	Cry1
Rfx4	Rab21	Abtb3
Cry1	2310011J03Rik	Timp3
Abtb3	Peli1	Hsp90b1
Timp3	Apc2	Ascl1
Hsp90b1	Thap2	Igf1
Ascl1	Slc1a4	Washc3
Igf1	Dock2	Sirt1
Washc3	Rab1a	Herc4
Mybpc1	Gamt	Hnrnph3
Sirt1	Ptprr	Rufy2
Herc4	Actr2	Ccar1
Hnrnph3	Ndufs7	Ddx21
Rufy2	Ptprb	Ddx50
Ccar1	Pwwp3a	Srgn
Ddx21	Meis1	Vps26a
Ddx50	Uqcr11	Supv3l1
Srgn	Cnot2	Hkdc1
Vps26a	Tcf3	Fam241b
Supv3l1	Frs2	Aifm2
Hkdc1	Yeats4	Macroh2a2
Fam241b	Cobl	Tysnd1
Aifm2	Rab36	Sar1a
Macroh2a2	Grb10	Ppa1
Tysnd1	Adora2a	Npffr1
Sar1a	Snrpd3	Eif4ebp2
Ppa1	Nav3	Pald1
Eif4ebp2	Ddc	Tbata
Pald1	Cpm	Sgpl1
Tbata	Mdm2	Pcbd1
Sgpl1	Csrp2	Unc5b
Pcbd1	Osbpl8	Slc29a3
Unc5b	Mknk2	Vsir
Slc29a3	Cabin1	Slc16a7
Vsir	Ap3d1	Lrig3
Slc16a7	Phlda1	Anapc16
Anapc16	Sf3a2	Ddit4
Ddit4	Mdm1	Dnajb12
Dnajb12	Timm13	Micu1
Micu1	Vps13d	Cand1
Cand1	Llph	Tbk1
Tbk1	Tmbim4	Pno1
Pno1	Helb	Plek
Plek	Prmt2	Srgap1
Srgap1	Dip2a	Egfr
Egfr	Hmg20b	Usp15
Usp15	Fzr1	Vps54
Vps54	Ncln	Tbc1d15

Tbc1d15	Col6a2	Rab21
Pcsk4	Hcfc2	2310011J03Rik
Rab21	Nfyb	Peli1
2310011J03Rik	Txnrd1	Apc2
Peli1	Ppm1m	Thap2
Apc2	Nopchap1	Lgr5
Thap2	Aldh1l2	Slc1a4
Slc1a4	Wdr82	Dock2
Dock2	Glyctk	Rab1a
Rab1a	Pofut2	Gamt
Gamt	Slc36a1	Ptprr
Ptprr	Adarb1	Actr2
Actr2	Appl2	Ndufs7
Ndufs7	Sumo3	Ptprb
Ptprb	Hint1	Pwwp3a
Pwwp3a	Lyrm7	Uqcr11
Uqcr11	Fbxw11	Cnot2
Cnot2	Stk10	Tcf3
Tcf3	Papolg	Best3
Frs2	Pfkl	Frs2
Yeats4	Pus10	Yeats4
Cobl	Rhbdf1	Cobl
Rab36	Pex13	Rab36
Grb10	Cfap410	Grb10
Adora2a	Mpg	Snrpd3
Snrpd3	Ahsa2	Nav3
Nav3	Nprl3	Ddc
Ddc	Xpo1	Cpm
Cpm	Nsg2	Mdm2
Mdm2	Cpeb4	Csrp2
Csrp2	Asb3	Osbpl8
Osbpl8	Cdc34	Mknk2
Mknk2	Tpgs1	Cabin1
Cabin1	Erlec1	Ap3d1
Ap3d1	Shc2	Phlda1
Phlda1	Sptbn1	Sf3a2
Sf3a2	Wdpcp	Mdm1
Mdm1	Mdh1	Timm13
Jsrp1	Ccng1	Vps13d
Timm13	Nudcd2	Llph
Vps13d	Polrmt	Tmbim4
Llph	Hcn2	Helb
Tmbim4	Acsl6	Prmt2
Helb	Slc22a4	Dip2a
Prmt2	Cyfip2	Hmg20b
Dip2a	Mgat1	Fzr1
Hmg20b	Ppp2ca	Ncln
Fzr1	Sgcd	Col6a2
Ncln	Flt4	Hcfc2
Col6a2	Hnrnpab	Nfyb
Hcfc2	Phykpl	Txnrd1
Nfyb	Hspa4	Glt8d2
Txnrd1	Cnot6	Ppm1m

Glt8d2	Gfpt2	Nopchap1
Ppm1m	Zfp354a	Aldh1l2
Nopchap1	Mapk9	Wdr82
Wdr82	Canx	Glyctk
Glyctk	Rack1	Pofut2
Pofut2	Rasgef1c	Slc36a1
Slc36a1	Rufy1	Adarb1
Adarb1	Rnf130	Appl2
Appl2	Rad50	Sumo3
Sumo3	Mrnip	Hint1
Hint1	Clk4	Lyrm7
Lyrm7	Sar1b	Fbxw11
Fbxw11	Jade2	Stk10
Stk10	Pdlim4	Papolg
Papolg	Cdkl3	Rel
Pfkl	Ube2b	Pfkl
Pus10	Cdkn2aipnl	Pus10
Rhbdf1	Kremen1	Rhbdf1
Pex13	Nefh	Pex13
Cfap410	Med7	Cfap410
Mpg	Tnip1	Mpg
Ahsa2	Vdac1	Ahsa2
Nprl3	Slu7	Nprl3
Xpo1	Ascc2	Xpo1
Nsg2	Hus1	Nsg2
Cpeb4	Tns3	Cpeb4
Asb3	Btg2	Stc2
Cdc34	Castor1	Asb3
Tpgs1	Igfbp3	Cdc34
Chac2	Pes1	Tpgs1
Erlec1	Adcy1	Chac2
Shc2	Tcn2	Erlec1
Sptbn1	Osbp2	Shc2
Wdpcp	Gabrg2	Sptbn1
Mdh1	Smtn	Wdpcp
Ccng1	Arf5	Mdh1
Fgf22	2310033P09Rik	Ccng1
Nudcd2	Guk1	Nudcd2
Polrmt	Rnf185	Polrmt
Hcn2	Limk2	Hcn2
Acsl6	Patz1	Acsl6
Slc22a4	Eif4enif1	Slc22a4
Zfp354b	Trim11	Zfp354b
Cyfip2	Ogdh	Cyfip2
Mgat1	Drg1	Mgat1
Ppp2ca	Rtn4	Ppp2ca
Sgcd	Mtif2	Sgcd
Flt4	Rps27a	Flt4
Hnrnpab	Cfap36	Hnrnpab
Phykpl	Ppp4r3b	Phykpl
Hspa4	Pnpt1	Hspa4
Cnot6	Pold2	Cnot6
Gfpt2	Zkscan17	Gfpt2

Zfp354a	Aebp1	Zfp354a
Mapk9	Polm	Mapk9
Canx	Dbnl	Canx
Rack1	Mrps24	Rack1
Rasgef1c	Ccdc117	Rufy1
Rufy1	Dynll2	Rnf130
Rnf130	Xbp1	Rad50
Rad50	Supt4a	Mrnip
Mrnip	Septin4	Clk4
Clk4	2810021J22Rik	Sar1b
Sar1b	Ska2	Jade2
Jade2	Smg8	Pdlim4
Pdlim4	Rnf187	Cdkl3
Cdkl3	Mrpl22	Ube2b
Ube2b	Cnot8	Cdkn2aipnl
Cdkn2aipnl	Rps6kb1	Kremen1
Kremen1	Sap30l	Nefh
Nefh	Galnt10	Med7
Med7	Rnft1	Tnip1
Havcr2	Mfap3	Vdac1
Tnip1	Fam114a2	Upp1
Vdac1	Gria1	Slu7
Slu7	Ppm1d	Nipal4
Ascc2	Znhit3	Ascc2
Hus1	Prpsap2	Hus1
Tns3	Ggnbp2	Zfp607a
Btg2	Acaca	Tns3
Igfbp3	Llgl1	Btg2
Pes1	Drg2	Igfbp3
Adcy1	Srebf1	Pes1
Tcn2	Tom1l1	Adcy1
Osbp2	Cox11	Tcn2
Gabrg2	Stxbp4	Osbp2
Smtn	Bzw2	Gabrg2
Arf5	Elac2	Smtn
2310033P09Rik	Polr1f	Arf5
Guk1	Atxn7l1	2310033P09Rik
Rnf185	Atp6v1c2	Guk1
Limk2	Sypl1	Rnf185
Patz1	Pdia6	Limk2
Eif4enif1	Nampt	Patz1
Trim11	Nbas	Eif4enif1
Ogdh	Tspan13	Trim11
Drg1	Rock2	Ogdh
Rtn4	Laptm4a	Drg1
Mtif2	Cyria	Rtn4
Rps27a	Snx13	Mtif2
Cfap36	Ntsr2	Rps27a
Ppp4r3b	Lpin1	Cfap36
Pnpt1	Pum2	Ppp4r3b
Pold2	Nrcam	Pnpt1
Zkscan17	Rgs9	Efemp1
Aebp1	Trib2	Pold2

Polm	Arsg	Zkscan17
Pgam2	Hs1bp3	Aebp1
Dbnl	Lratd1	Polm
Mrps24	Smc6	Pgam2
Ccdc117	Amz2	Dbnl
Dynll2	Gna13	Mrps24
Xbp1	Prkar1a	Ccdc117
Supt4a	Fam20a	Dynll2
Septin4	Abca8b	Xbp1
2810021J22Rik	Rdh14	Supt4a
Ska2	Map2k6	Septin4
Smg8	Klhl29	2810021J22Rik
Rnf187	Trappc12	Ska2
Tubd1	Adi1	Smg8
Mrpl22	Rnaseh1	Rnf187
Cnot8	Ubxn2a	Tubd1
Rps6kb1	Cmpk2	Mrpl22
Sap30l	Pfn4	Cnot8
Galnt10	Itsn2	Rps6kb1
Rnft1	Rnf144a	Sap30l
Mfap3	Id2	Galnt10
Fam114a2	Mboat2	Rnft1
Gria1	Ncoa1	Mfap3
Ppm1d	Bcap29	Fam114a2
Znhit3	Cenpo	Gria1
Myo19	Klf11	Ppm1d
Prpsap2	Adcy3	Znhit3
Ggnbp2	Grhl1	Myo19
Acaca	Dnajc27	Prpsap2
Llgl1	Efr3b	Ggnbp2
Drg2	Cbll1	Acaca
Srebf1	Pomc	Shmt1
Tom1l1	Dnmt3a	Llgl1
Cox11	Dld	Drg2
Stxbp4	Kif3c	Srebf1
Bzw2	Sh3yl1	Tom1l1
Elac2	Rab10	Cox11
Polr1f	Sntg2	Stxbp4
Atxn7l1	Pxdn	Bzw2
Sypl1	Ddx52	Elac2
Pdia6	Taf15	Polr1f
Nampt	Ace	Atxn7l1
Pik3cg	Rasl10b	Atp6v1c2
Nbas	Cdc27	Sypl1
Tspan13	Mettl2	Pdia6
Rock2	Nle1	Nampt
Laptm4a	Tlk2	Pik3cg
Cyria	Mrc2	Nbas
Snx13	Rffl	Tspan13
Ntsr2	Lig3	Rock2
Lpin1	Map3k3	Laptm4a
Pum2	Tmem132e	Cyria
Nrcam	Asic2	Snx13

Rgs9	Ddx42	Ntsr2
Trib2	Ftsj3	Lpin1
Arsg	Psmc5	Pum2
Hs1bp3	Ern1	Nrcam
Lratd1	Nf1	Rgs9
Smc6	Pecam1	Trib2
Amz2	Ddx5	Arsg
Gna13	Psmd12	Hs1bp3
Prkar1a	Helz	Lratd1
Fam20a	Cacng4	Smc6
Abca8b	Cep112	Amz2
Rdh14	Nherf1	Gna13
Map2k6	Grin2c	Prkar1a
Klhl29	Nt5c	Fam20a
Trappc12	Jpt1	Abca8b
Adi1	Sumo2	Rdh14
Rnaseh1	Nup85	Map2k6
Ubxn2a	Gga3	Klhl29
Fkbp1b	Cluh	Trappc12
Cmpk2	Mif4gd	Adi1
Pfn4	Slc25a19	Rnaseh1
Itsn2	Pafah1b1	Ubxn2a
Rnf144a	Tmem94	Fkbp1b
Id2	Recql5	Cmpk2
Mboat2	Sap30bp	Pfn4
Ncoa1	Itgb4	Itsn2
Dus4l	Galk1	Rnf144a
Bcap29	Unk	Id2
Slc26a4	Aspa	Mboat2
Cenpo	Mrpl38	Ncoa1
Klf11	Fbf1	Dus4l
Adcy3	Acox1	Bcap29
Grhl1	Ten1	Slc26a4
Dnajc27	Srp68	Cenpo
Efr3b	Tsen54	Klf11
Cbll1	Ncbp3	Adcy3
Pomc	Camkk1	Grhl1
Dnmt3a	Atp2a3	Dnajc27
Dld	Ankfy1	Efr3b
Kif3c	Exoc7	Cbll1
Sh3yl1	Ube2g1	Dnmt3a
Rab10	Tekt1	Dld
Sntg2	Med31	Kif3c
Pxdn	Ube2o	Sh3yl1
Ddx52	Txndc17	Rab10
Taf15	Slc13a5	Sntg2
Ace	4933427D14Rik	Pxdn
Mmp28	Cygb	Ddx52
Rasl10b	Wscd1	Taf15
Cdc27	Snhg16	Ace
Itgb3	Mxra7	Mmp28
Mettl2	Rabep1	Rasl10b
Nle1	Mfsd11	Cdc27

Tlk2	Kif1c	Itgb3
Mrc2	Sec14l1	Mettl2
Rffl	Mink1	Nle1
Lig3	Pld2	Tlk2
Map3k3	Slc46a1	Mrc2
Tmem132e	0610010K14Rik	Rffl
Asic2	Eral1	Lig3
Ddx42	Dhrs13	Map3k3
Ftsj3	Blmh	Tmem132e
Rnf135	Cpd	Asic2
Psmc5	Timm22	Ddx42
Adap2	Nxn	Ftsj3
Ern1	Rflnb	Rnf135
Nf1	Rph3al	Psmc5
Pecam1	Doc2b	Adap2
Polg2	Ywhae	Ern1
Ddx5	Prpf8	Nf1
Psmd12	Nme2	Pecam1
Helz	Spag9	Polg2
Cacng4	Luc7l3	Ddx5
Cep112	Ankrd40	Psmd12
Nherf1	Cacna1g	Helz
Grin2c	Xylt2	Cacng4
Nt5c	Lrrc59	Cep112
Jpt1	Slc35b1	Rab37
Sumo2	Snx11	Nherf1
Nup85	Scrn2	Grin2c
Gga3	Cacnb1	Nt5c
Cluh	Fbxl20	Jpt1
Mif4gd	Dlg4	Sumo2
Slc25a19	Dvl2	Nup85
Pafah1b1	Nr1d1	Gga3
Tmem94	Aloxe3	Cluh
Recql5	Per1	Mif4gd
Sap30bp	Vamp2	Slc25a19
Itgb4	Tmem107	Pafah1b1
Galk1	Ctc1	Tmem94
Unk	Pfas	Recql5
Trim47	Myh10	Sap30bp
Aspa	Ntn1	Itgb4
Mrpl38	Stx8	Galk1
Fbf1	Cfap52	Unk
Acox1	Adprm	Trim47
Ten1	Acly	Aspa
Srp68	Kat2a	Mrpl38
Tsen54	Stat5b	Fbf1
Ncbp3	Tmem101	Acox1
Camkk1	Lsm12	Ten1
Atp2a3	Ubtf	Srp68
Ankfy1	Ccdc43	Tsen54
Exoc7	Adam11	Ncbp3
Ube2g1	Eftud2	Camkk1
Med31	Ccdc103	Atp2a3

Ube2o	Gfap	Ankfy1
Txndc17	Dcakd	Exoc7
Slc13a5	Nmt1	Ube2g1
4933427D14Rik	Plcd3	Med31
Cygb	Map3k14	Ube2o
Wscd1	Gosr2	Txndc17
Snhg16	Klhl28	Slc13a5
Mxra7	Fkbp3	4933427D14Rik
Rabep1	Foxg1	Cygb
Mfsd11	Scfd1	Wscd1
Kif1c	Coch	Snhg16
Sec14l1	Strn3	Mxra7
Mink1	Ap4s1	Rabep1
Pld2	Dtd2	Mfsd11
Slc46a1	Ston2	Kif1c
0610010K14Rik	Gtf2a1	Sec14l1
Eral1	Sel1l	Mink1
Dhrs13	Dnaaf2	Pld2
Coro6	Klhdc2	Slc46a1
Blmh	Nemf	0610010K14Rik
Cpd	Sec23a	Eral1
Timm22	L2hgdh	Dhrs13
Nxn	Cdkl1	Coro6
Rflnb	Trappc6b	Blmh
Doc2b	Pnn	Cpd
Ywhae	Mia2	Timm22
Prpf8	Galc	Nxn
Nme2	Spata7	Rflnb
Spag9	Ptpn21	Doc2b
Luc7l3	Npas3	Ywhae
Ankrd40	Zc3h14	Prpf8
Cacna1g	Ttc8	Nme2
Xylt2	Polr2h	Spag9
Lrrc59	Ppp2r3c	Luc7l3
Slc35b1	Psma6	Ankrd40
Snx11	Nfkbia	Cacna1g
Scrn2	Ralgapa1	Xylt2
Cacnb1	Mbip	Lrrc59
Fbxl20	Ngb	Slc35b1
Dlg4	Gstz1	Snx11
Dvl2	Sptlc2	Scrn2
Nr1d1	Ahsa1	Cacnb1
Aloxe3	Vipas39	Fbxl20
Per1	Snw1	Dlg4
Vamp2	Slirp	Dvl2
Tmem107	Adck1	Nr1d1
Ctc1	Nova1	Alox8
Pfas	Mthfd1	Aloxe3
Myh10	Ppp2r5e	Per1
Pik3r5	Sgpp1	Vamp2
Ntn1	Akap5	Tmem107
Stx8	Sptb	Ctc1
Usp43	Rab15	Pfas

Myh3	Fut8	Myh10
Adprm	Atl1	Ntn1
Acly	Sav1	Stx8
Kat2a	Nin	Adprm
Stat5b	Pygl	Acly
Tmem101	Trim9	Kat2a
Lsm12	Tmx1	Stat5b
Ubtf	Actr10	Tmem101
Ccdc43	Timm9	Lsm12
Adam11	Rtn1	Ubtf
Eftud2	Lrrc9	Ccdc43
Ccdc103	Serpina3n	Adam11
Gfap	Dhrs7	Eftud2
Dcakd	Ppm1a	Gfap
Nmt1	Clmn	Dcakd
Plcd3	Glrx5	Nmt1
Map3k14	Mnat1	Plcd3
Gosr2	Prkch	Map3k14
Klhl28	Hif1a	Gosr2
Fkbp3	Papola	Klhl28
Foxg1	Pals1	Fkbp3
Scfd1	Snapc1	Foxg1
Coch	Atp6v1d	Scfd1
Strn3	Vrk1	Coch
Ap4s1	Eif2s1	Strn3
Dtd2	Pigh	Ap4s1
Ston2	Vti1b	Dtd2
Gtf2a1	Zfp36l1	Ston2
Sel1l	Galnt16	Gtf2a1
Dnaaf2	Erh	Sel1l
Klhdc2	Susd6	Dnaaf2
Nemf	Srsf5	Klhdc2
Sec23a	Smoc1	Nemf
L2hgdh	S2bpcox16	Sec23a
Cdkl1	Pcnx1	L2hgdh
Trappc6b	Pacs2	Cdkl1
Pnn	Mta1	Trappc6b
Mia2	Wdr37	Pnn
Galc	Gtpbp4	Mia2
Spata7	Zmynd11	Galc
Ptpn21	Esyt2	Spata7
Npas3	Tdp1	Ptpn21
Zc3h14	Psmc1	Npas3
Ttc8	Nrde2	Zc3h14
Polr2h	Rps6ka5	Ttc8
Ppp2r3c	Ccdc88c	Polr2h
Prorp	Dglucy	Ppp2r3c
Psma6	Fbln5	Prorp
Nfkbia	Trip11	Psma6
Ralgapa1	Atxn3	Nfkbia
Mbip	Lgmn	Ralgapa1
Ngb	Golga5	Mbip
Gstz1	Pitrm1	Ngb

Sptlc2	Chga	Gstz1
Ahsa1	Pfkp	Sptlc2
Vipas39	Unc79	Ahsa1
Snw1	Otub2	Vipas39
Slirp	Ppp4r4	Snw1
Adck1	Net1	Slirp
Nova1	Tshz3	Adck1
Mthfd1	Gdi2	Nova1
Ppp2r5e	Rgs6	Mthfd1
Sgpp1	Dcaf4	Ppp2r5e
Akap5	Papln	Sgpp1
Sptb	Numb	Akap5
Rab15	Acot2	Sptb
Fut8	Fam161b	Rab15
Atl1	Coq6	Fut8
Sav1	Entpd5	Atl1
Nin	Aldh6a1	Sav1
Pygl	Abcd4	Nin
Trim9	Isca2	Pygl
Tmx1	Npc2	Trim9
Actr10	Fcf1	Tmx1
Timm9	Ylpm1	Actr10
Rtn1	Mlh3	Timm9
Lrrc9	Tmed10	Rtn1
Serpina3n	Fos	Serpina3n
Dhrs7	Erg28	Dhrs7
Ppm1a	Tgfb3	Ppm1a
Clmn	Gpatch2l	Clmn
4930447C04Rik	Vash1	Glrx5
Glrx5	Angel1	Mnat1
Mnat1	Ccnk	Prkch
Prkch	Cyp46a1	Hif1a
Hif1a	Hhipl1	Papola
Papola	Evl	Pals1
Pals1	Yy1	Snapc1
Snapc1	Slc25a29	Atp6v1d
Atp6v1d	Wars1	Vrk1
Vrk1	Meg3	Eif2s1
Eif2s1	Hsp90aa1	Pigh
Pigh	Zfp839	Vti1b
Vti1b	Fdft1	Zfp36l1
Zfp36l1	Tecpr2	Galnt16
Galnt16	Cinp	Erh
Erh	Traf3	Susd6
Susd6	Amn	Srsf5
Srsf5	Cdc42bpb	Smoc1
Smoc1	Eif5	S2bpcox16
S2bpcox16	Ppp1r13b	Pcnx1
Pcnx1	Zfyve21	Pacs2
Pacs2	Xrcc3	Mta1
Mta1	Klc1	Wdr37
Wdr37	Atp5mj	Gtpbp4
Gtpbp4	Kif26a	Zmynd11

Zmynd11	Hecw1	Esyt2
Esyt2	Ggps1	Tdp1
Tdp1	Gng4	Psmc1
Psmc1	Gpr137b	Nrde2
Nrde2	Mtr	Rps6ka5
Rps6ka5	Ryr2	Ccdc88c
Ccdc88c	Amph	Dglucy
Dglucy	Gli3	Fbln5
Trip11	Trim27	Trip11
Atxn3	Zkscan3	Atxn3
Lgmn	Carmil1	Lgmn
Golga5	Mrs2	Golga5
Pitrm1	Gpld1	Pitrm1
Chga	Exoc2	Chga
Pfkp	Gcnt2	Pfkp
Unc79	Tmem14c	Unc79
Asb2	Mak	Otub2
Otub2	Elovl2	Ppp4r4
Ppp4r4	Nedd9	Net1
Net1	Hivep1	Tshz3
Tshz3	Tbc1d7	Gdi2
Gdi2	Mcur1	Rgs6
Rgs6	Cap2	Dpf3
Dpf3	Nup153	Dcaf4
Dcaf4	Kif13a	Numb
Papln	Tpmt	Acot2
Numb	Dek	Acot3
Acot2	Id4	Fam161b
Fam161b	Ippk	Coq6
Coq6	Nol8	Entpd5
Entpd5	Spin1	Aldh6a1
Aldh6a1	Wrnip1	Abcd4
Abcd4	Ripk1	Isca2
Isca2	Pxdc1	Npc2
Npc2	Prpf4b	Fcf1
Fcf1	Eci2	Ylpm1
Ylpm1	Fars2	Mlh3
Mlh3	Ssr1	Tmed10
Tmed10	Riok1	Fos
Fos	Snrnp48	Erg28
Erg28	Slc35b3	Tgfb3
Tgfb3	Shc3	Gpatch2l
Gpatch2l	Sema4d	Esrrb
Vash1	Gadd45g	Vash1
Angel1	Syk	Angel1
Ccnk	Aopep	Ccnk
Cyp46a1	Auh	Cyp46a1
Hhipl1	Fancc	Hhipl1
Evl	Ptch1	Evl
Yy1	Sptlc1	Yy1
Slc25a29	Ercc6l2	Slc25a29
Wars1	Sfxn1	Wars1
Meg3	Habp4	Meg3

Hsp90aa1	Ctsl	Hsp90aa1
Zfp839	Drd1	Zfp839
Fdft1	Zfp346	Fdft1
Tecpr2	Prxl2c	Tecpr2
Cinp	Cdk20	Cinp
Traf3	Lman2	Traf3
Amn	Prelid1	Cdc42bpb
Cdc42bpb	Nsd1	Exoc3l4
Eif5	Pdlim7	Eif5
Ppp1r13b	Ddx41	Ppp1r13b
Zfyve21	Fam193b	Zfyve21
Xrcc3	Pcbd2	Xrcc3
Klc1	Txndc15	Klc1
Atp5mj	Ddx46	Atp5mj
Kif26a	Caml	Kif26a
Hecw1	B4galt7	Hecw1
Ggps1	Cxcl14	Ggps1
Gng4	Zfp729a	Gng4
Gpr137b	Zfp369	Gpr137b
Mtr	Ptdss1	Mtr
Ryr2	Mterf3	Ryr2
Amph	Uqcrb	Amph
Gli3	Fastkd3	Gli3
Trim27	Cfap90	Trim27
Zkscan3	Adcy2	Zkscan3
Carmil1	Cetn3	Carmil1
Mrs2	Smad5	Mrs2
Gpld1	Trpc7	Gpld1
Exoc2	Hnrnpk	Exoc2
Gcnt2	Ccnh	Gcnt2
Tmem14c	Rasa1	Tmem14c
Mak	Qng1	Elovl2
Elovl2	Gkap1	Nedd9
Nedd9	Naa35	Hivep1
Hivep1	Golm1	Tbc1d7
Tbc1d7	Agtpbp1	Mcur1
Mcur1	Dapk1	Cap2
Cap2	Nkd2	Nup153
Nup153	Tppp	Kif13a
Kif13a	Pdcd6	Tpmt
Tpmt	Sdha	Dek
Dek	Ccdc127	Id4
Id4	Erap1	Ippk
Ippk	Cast	Ogn
Nol8	Pcsk1	Nol8
Spin1	Rhobtb3	Spin1
Wrnip1	Glrx	Wrnip1
Ripk1	Arsk	Ripk1
Pxdc1	Srd5a1	Pxdc1
Prpf4b	Nsun2	Prpf4b
Eci2	Mctp1	Eci2
Rpp40	Slf1	Rpp40
Fars2	Med10	Fars2

Ly86	Ndufs6	Ly86
Ssr1	Mrpl36	Ssr1
Riok1	Lpcat1	Riok1
Snrnp48	Clptm1l	Snrnp48
Slc35b3	Tert	Slc35b3
Shc3	Hapln1	Shc3
Sema4d	Vcan	Sema4d
Gadd45g	Xrcc4	Gadd45g
Syk	Zcchc9	Syk
Aopep	Slc30a5	Aopep
Auh	Rad17	Auh
Fancc	Ocln	Fancc
Ptch1	Gtf2h2	Ptch1
Sptlc1	Smn1	Sptlc1
Ercc6l2	Mccc2	Ercc6l2
Sfxn1	Cartpt	Sfxn1
Habp4	Ptcd2	Habp4
Ctsl	Btf3	Ctsl
Drd1	Ankra2	Drd1
Zfp346	Arhgef28	Zfp346
Prxl2c	Hexb	Prxl2c
Cdk20	Gfm2	Cdk20
Lman2	Polk	Lman2
Prelid1	Cert1	Prelid1
Nsd1	Hmgcr	Nsd1
Pdlim7	Poc5	Pdlim7
Ddx41	Iqgap2	Ddx41
Fam193b	Crhbp	Fam193b
Pcbd2	Aggf1	Pcbd2
Txndc15	Pde8b	Txndc15
Ddx46	Ap3b1	Ddx46
Caml	Scamp1	Caml
B4galt7	Jmy	B4galt7
Cxcl14	Dimt1	Cxcl14
Zfp729a	Kif2a	Zfp729a
Zfp369	Ercc8	Zfp369
Ptdss1	Elovl7	Ptdss1
Mterf3	Pde4d	Mterf3
Uqcrb	Rab3c	Uqcrb
Fastkd3	Plk2	Fastkd3
Cfap90	Thbs4	Cfap90
Adcy2	Serinc5	Adcy2
Cetn3	Mtx3	Cetn3
Smad5	Zfyve16	Smad5
Trpc7	Rasgrf2	Trpc7
Hnrnpk	Erbin	Hnrnpk
Ccnh	Nln	Ccnh
Rasa1	Trappc13	Rasa1
Qng1	Trim23	Qng1
Gkap1	Ppwd1	Gkap1
Naa35	Cwc27	Naa35
Golm1	Srek1ip1	Golm1
Agtpbp1	Rgs7bp	Agtpbp1

Dapk1	Rnf180	Dapk1
Tppp	Htr1a	Nkd2
Pdcd6	Parp8	Tppp
Sdha	Emb	Pdcd6
Ccdc127	Hcn1	Sdha
Erap1	Mrps30	Ccdc127
Cast	Slc4a7	Erap1
Pcsk1	Psmd6	Cast
Rhobtb3	Atxn7	Pcsk1
Glrx	Pdhb	Rhobtb3
Srd5a1	Fam107a	Glrx
Nsun2	Kctd6	Arsk
Mctp1	Map3k1	Srd5a1
Slf1	Il6st	Nsun2
Med10	Plpp1	Mctp1
Ndufs6	Cspg4b	Slf1
Mrpl36	Ndufs4	Med10
Lpcat1	Kat6b	Ndufs6
Clptm1l	Samd8	Mrpl36
Tert	Vdac2	Lpcat1
Hapln1	Nkiras1	Clptm1l
Vcan	Comtd1	Tert
Xrcc4	Ube2e1	Hapln1
Atg10	Nr1d2	Vcan
Zcchc9	Thrb	Xrcc4
Slc30a5	Dlg5	Atg10
Rad17	Ngly1	Zcchc9
Ocln	Oxsm	Slc30a5
Gtf2h2	Prxl2a	Rad17
Serf1	Glud1	Ocln
Smn1	Bmpr1a	Gtf2h2
Mccc2	Nid2	Smn1
Cartpt	Rtraf	Mccc2
Ptcd2	Nudt13	Ptcd2
Btf3	Ecd	Btf3
Ankra2	Dnajc9	Ankra2
Arhgef28	Anxa7	Arhgef28
Hexb	Ppp3cb	Hexb
Gfm2	Zswim8	Gfm2
Polk	Camk2g	Polk
Cert1	Vcl	Cert1
Hmgcr	Ap3m1	Hmgcr
Poc5	Txndc16	Poc5
Iqgap2	Ero1a	Iqgap2
Crhbp	Psmc6	Crhbp
Aggf1	Samd4	Aggf1
Pde8b	Mapk1ip1l	Pde8b
Ap3b1	Ktn1	Ap3b1
Scamp1	Peli2	Scamp1
Jmy	Otx2	Jmy
Dimt1	Slc35f4	Dimt1
Kif2a	Anxa11	Kif2a
Ercc8	Ppif	Ercc8

Elovl7	Slmap	Elovl7
Pde4d	Rnase4	Pde4d
Rab3c	Arf4	Rab3c
Plk2	Dnah12	Plk2
Serinc5	Hacl1	Serinc5
Mtx3	Eaf1	Mtx3
Zfyve16	Mettl6	Zfyve16
Rasgrf2	Sh3bp5	Dhfr
Erbin	Capn7	Rasgrf2
Nln	Arhgef3	Erbin
Trappc13	Btd	Nln
Trim23	Bap1	Trappc13
Ppwd1	Galnt15	Trim23
Cwc27	Sema3g	Ppwd1
Srek1ip1	Dph3	Cwc27
Rgs7bp	Oxnad1	Srek1ip1
Rnf180	Nisch	Rgs7bp
Htr1a	Parg	Rnf180
Parp8	Ogdhl	Htr1a
Emb	Glt8d1	Parp8
Hcn1	Spcs1	Emb
Mrps30	Nek4	Hcn1
Fgf10	Chat	Mrps30
Slc4a7	Ebpl	Fgf10
Psmd6	Kpna3	Slc4a7
Atxn7	Spryd7	Psmd6
Fezf2	Rnaseh2b	Atxn7
Cfap20dc	Mapk8	Fezf2
Pdhb	Pspc1	Cfap20dc
Fam107a	Ctsb	Pdhb
Kctd6	Gdf10	Fam107a
Map3k1	Zmym2	Kctd6
Il6st	Cryl1	Map3k1
Plpp1	Prkcd	Il6st
Ndufs4	Eef1akmt1	Plpp1
Kat6b	Xpo4	Ndufs4
Samd8	Tkt	Kat6b
Vdac2	Lats2	Samd8
Nkiras1	Dcp1a	Vdac2
Comtd1	Sap18	Nkiras1
Ube2e1	Mrpl57	Comtd1
Nr1d2	Zdhhc20	Ube2e1
Thrb	Hmbox1	Nr1d2
Dlg5	Micu2	Thrb
Ngly1	Fgf9	Dlg5
Oxsm	Ints9	Ngly1
Prxl2a	Extl3	Oxsm
Glud1	Cab39l	Prxl2a
Bmpr1a	Cdadc1	Glud1
Cdhr1	Atp8a2	Bmpr1a
Nid2	Amer2	Ldb3
Rtraf	Mtmr6	Nid2
Nudt13	Spata13	Rtraf

Ecd	Cacna2d3	Nudt13
Dnajc9	Mipep	Ecd
Anxa7	Wnt5a	Dnajc9
Ppp3cb	Esd	Anxa7
Zswim8	Lcp1	Ppp3cb
Camk2g	Zc3h13	Zswim8
Plau	Slc25a30	Camk2g
Vcl	Gpalpp1	Vcl
Ap3m1	Nufip1	Ap3m1
Txndc16	Tsc22d1	Txndc16
Ero1a	Enox1	Ero1a
Psmc6	Dnajc15	Psmc6
Samd4	Akap11	Samd4
Mapk1ip1l	Rgcc	Mapk1ip1l
Ktn1	Tdrd3	Ktn1
Peli2	Naa16	Peli2
Slc35f4	Wbp4	Slc35f4
Anxa11	Sugt1	Anxa11
Ppif	Elp3	Ppif
Slmap	Ccdc25	Slmap
Gm49342	Clu	Rnase4
Rnase4	Ephx2	Arf4
Arf4	Trim35	Hacl1
Hacl1	Stmn4	Eaf1
Eaf1	Dpysl2	Mettl6
Mettl6	Bnip3l	Sh3bp5
Sh3bp5	Ppp2r2a	Capn7
Capn7	Nefm	Arhgef3
Arhgef3	Nefl	Btd
Btd	Pibf1	Bap1
Bap1	Entpd4b	Phf7
Phf7	Rhobtb2	Sema3g
Sema3g	Klhl1	Dph3
Dph3	Bin3	Oxnad1
Oxnad1	Pdlim2	Nisch
Nisch	Sorbs3	Parg
Parg	Ppp3cc	Ogdhl
Ogdhl	Slc39a14	Glt8d1
Glt8d1	Fhip2b	Spcs1
Spcs1	Hr	Nek4
Nek4	Bmp1	Ebpl
Ebpl	Dmtn	Kpna3
Kpna3	Xpo7	Spryd7
Spryd7	Gfra2	Rnaseh2b
Rnaseh2b	Rb1	Mapk8
Mapk8	Rcbtb2	Pspc1
Pspc1	Itm2b	Ctsb
Ctsb	Med4	Gdf10
Zmym2	Sucla2	Zmym2
Cryl1	Uchl3	Cryl1
Prkcd	Gpc5	Prkcd
Eef1akmt1	Spry2	Eef1akmt1
Xpo4	Rbm26	Xpo4

Tkt	Obi1	Tkt
Pinx1	Ednrb	Pinx1
Lats2	Fbxl3	Lats2
Dcp1a	Cln5	Dcp1a
Sap18	Gpr180	Sap18
Mrpl57	Cldn10	Mrpl57
Zdhhc20	Dnajc3	Zdhhc20
Hmbox1	Mbnl2	Hmbox1
Micu2	Nipbl	Micu2
Fgf9	Nup155	Fgf9
Ints9	Fyb1	Ints9
Extl3	Dab2	Extl3
Cab39l	Ttc33	Cab39l
Cdadc1	Rab2b	Cdadc1
Atp8a2	Mettl3	Atp8a2
Amer2	Dad1	Amer2
Mtmr6	Lrp10	Mtmr6
Spata13	Rem2	Spata13
Cacna2d3	Haus4	Cacna2d3
Mipep	4931414P19Rik	Mipep
Wnt5a	Slc7a8	Wnt5a
Esd	Fbxo4	Esd
Lcp1	Acin1	Lcp1
Zc3h13	Oxct1	Zc3h13
Slc25a30	Drosha	Slc25a30
Gpalpp1	Psmb5	Gpalpp1
Nufip1	Pabpn1	Nufip1
Tsc22d1	6030458C11Rik	Tsc22d1
Enox1	Pdzd2	Enox1
Dnajc15	Slc22a17	Dnajc15
Akap11	Golph3	Akap11
Rgcc	Zfr	Rgcc
Tdrd3	Efs	Tdrd3
Diaph3	Ngdn	Naa16
Mtrf1	Sub1	Mtrf1
Wbp4	Jph4	Wbp4
Sugt1	Dhrs4	Sugt1
Elp3	Carmil3	Elp3
Ccdc25	Cpne6	Ccdc25
Clu	Dcaf11	Clu
Ephx2	Psme1	Ephx2
Trim35	Emc9	Trim35
Stmn4	Sdr39u1	Stmn4
Dpysl2	Zscan26	Dpysl2
Bnip3l	Sema5a	Bnip3l
Ppp2r2a	Cct5	Ppp2r2a
Nefm	Cmbl	Nefm
Nefl	Ankrd33b	Nefl
Pibf1	Ctnnd2	Nkx3-1
Entpd4b	Tars1	Pibf1
Rhobtb2	Amacr	Entpd4b
Bin3	Rai14	Rhobtb2
Pdlim2	Brix1	Bin3

Sorbs3	Rad1	Pdlim2
Ppp3cc	Nadk2	Sorbs3
Slc39a14	Mtdh	Ppp3cc
Fhip2b	Laptm4b	Slc39a14
Hr	Sdc2	Fhip2b
Bmp1	Dnah5	Hr
Dmtn	Trio	Bmp1
Xpo7	Ank	Dmtn
Gfra2	Marchf11	Xpo7
Rb1	Retreg1	Gfra2
Rcbtb2	Myo10	Rb1
Itm2b	Rnf19a	Rcbtb2
Med4	Pabpc1	Itm2b
Sucla2	Ywhaz	Med4
Uchl3	Rrm2b	Sucla2
Gpc5	Atp6v1c1	Uchl3
Spry2	Baalc	Gpc5
Rbm26	Fzd6	Spry2
Obi1	Slc25a32	Rbm26
Ednrb	Dcaf13	Obi1
Fbxl3	Lrp12	Ednrb
Cln5	Zfpm2	Fbxl3
Tgds	Oxr1	Cln5
Gpr180	Csmd3	Tgds
Cldn10	Eif3h	Gpr180
Dnajc3	Utp23	Cldn10
Mbnl2	Rad21	Dnajc3
Nipbl	Cdh10	Mbnl2
Nup155	Rida	Nipbl
Fyb1	Matn2	Nup155
Dab2	Pop1	Fyb1
Ttc33	Stk3	Dab2
Rab2b	Khdrbs3	Ttc33
Mettl3	Zfat	Rab2b
Dad1	Eif3e	Mettl3
Lrp10	Emc2	Dad1
Rem2	Eny2	Lrp10
Haus4	Ebag9	Rem2
4931414P19Rik	Sybu	Haus4
Slc7a8	Kcnv1	4931414P19Rik
Fbxo4	Myc	Slc7a8
Acin1	Washc5	Fbxo4
Oxct1	Sqle	Acin1
Drosha	Mtss1	Oxct1
Psmb5	Ndufb9	Drosha
Pabpn1	Fbxo32	Psmb5
6030458C11Rik	Ntaq1	Pabpn1
Pdzd2	Atad2	6030458C11Rik
Slc22a17	Zhx1	Pdzd2
Golph3	Tbc1d31	Slc22a17
Zfr	Derl1	Golph3
Efs	Mrpl13	Zfr
Ngdn	Sla	Efs

Sub1	Adcy8	Ngdn
Npr3	Asap1	Sub1
Jph4	Cyrib	Npr3
Dhrs4	Wnt7b	Jph4
Carmil3	Ppara	Dhrs4
Cpne6	Trmu	Carmil3
Dcaf11	Brd1	Cpne6
Psme1	Tef	Dcaf11
Emc9	Zc3h7b	Psme1
Cideb	Rangap1	Emc9
Sdr39u1	L3mbtl2	Sdr39u1
Zscan26	Rbx1	Zscan26
Sema5a	Xpnpep3	Sema5a
Cct5	St13	Cct5
Cmbl	Slc25a17	Cmbl
Ankrd33b	Adsl	Ankrd33b
Ctnnd2	Mief1	Ctnnd2
Tars1	Tab1	Tars1
Amacr	Syngr1	Amacr
Rai14	Cacna1i	Rai14
Brix1	Deptor	Brix1
Rad1	Dnal4	Rad1
Nadk2	Nptxr	Nadk2
Mtdh	Enpp2	Mtdh
Laptm4b	Josd1	Laptm4b
Sdc2	Tomm22	Sdc2
Dnah5	Cby1	Dnah5
Trio	Csnk1e	Trio
Ank	Fam118a	Ank
Marchf11	Sh3bp1	Marchf11
Retreg1	Samm50	Retreg1
Myo10	Parvb	Myo10
Rnf19a	Parvg	Rnf19a
Pabpc1	Efcab6	Pabpc1
Ywhaz	Ttll1	Ywhaz
Rrm2b	Myh9	Rrm2b
Atp6v1c1	Adamts20	Atp6v1c1
Baalc	Ndufa6	Baalc
Fzd6	Twf1	Fzd6
Slc25a32	Smdt1	Slc25a32
Dcaf13	Naga	Dcaf13
Lrp12	Nell2	Lrp12
Zfpm2	Septin3	Zfpm2
Oxr1	Slc38a2	Oxr1
Angpt1	Srebf2	Angpt1
Csmd3	Rpap3	Csmd3
Eif3h	Rapgef3	Eif3h
Utp23	Xrcc6	Utp23
Rad21	Desi1	Rad21
Cdh10	Pmm1	Cdh10
Rida	Hdac7	Rida
Matn2	Polr3h	Matn2
Pop1	Aco2	Pop1

Stk3	Nckap1l	Stk3
Khdrbs3	Pde1b	Khdrbs3
Zfat	Ppp1r1a	Zfat
Eif3e	Shisa9	Eif3e
Emc2	Txndc11	Emc2
Eny2	Litaf	Eny2
Ebag9	Nubp1	Ebag9
Sybu	Emp2	Sybu
Kcnv1	Hapstr1	Kcnv1
Myc	Bcl6	Myc
Washc5	Il1rap	Washc5
Sqle	Anks3	Sqle
Mtss1	Nudt16l1	Mtss1
Ndufb9	Mgrn1	Ndufb9
Fbxo32	Srl	Fbxo32
Ntaq1	Crebbp	Ntaq1
Atad2	Fgf12	Atad2
Zhx1	Plaat1	Zhx1
Tbc1d31	Zfp251	Tbc1d31
Derl1	Hes1	Derl1
Mrpl13	Zfp263	Mrpl13
Sla	Atp13a3	Sla
Dnaaf11	Glyr1	Adcy8
Adcy8	Tmem44	Asap1
Asap1	Lsg1	Cyrib
Cyrib	Rogdi	Wnt7b
Wnt7b	Dnaaf8	Ppara
Trmu	Ercc4	Trmu
Brd1	Gpt	Brd1
Tef	Apod	Tef
Zc3h7b	Adck5	Zc3h7b
Rangap1	Cyc1	Rangap1
L3mbtl2	Sharpin	L3mbtl2
Rbx1	Maf1	Rbx1
Xpnpep3	Hgh1	Xpnpep3
St13	Dgat1	St13
Slc25a17	Hsf1	Slc25a17
Adsl	Bop1	Adsl
Mief1	Mroh1	Mief1
Tab1	Fbxl6	Tab1
Syngr1	Slc52a2	Syngr1
Cacna1i	Gpaa1	Cacna1i
Deptor	Oplah	Deptor
Dnal4	Grina	Dnal4
Nptxr	Plec	Nptxr
Enpp2	Scrib	Enpp2
Josd1	Gfus	Josd1
Tomm22	Pycr3	Tomm22
Cby1	Naprt	Cby1
Csnk1e	Ly6h	Csnk1e
Fam118a	Rhpn1	Fam118a
Sh3bp1	Ly6e	Sh3bp1
Samm50	Lynx1	Samm50

Parvb	Zbtb11	Parvb
Parvg	Arc	Parvg
C1qtnf6	Cep97	C1qtnf6
Efcab6	Ptk2	Ttll1
Ttll1	Lmf2	Myh9
Myh9	Chkb	Adamts20
Adamts20	Mapk8ip2	Ndufa6
Ndufa6	Arsa	Twf1
Twf1	Rabl2	Smdt1
Smdt1	Shank3	Naga
Naga	Kif21a	Nell2
Nell2	Yaf2	Septin3
Septin3	Zcrb1	Slc38a2
Slc38a2	Alcam	Srebf2
Srebf2	Cblb	Rpap3
Rpap3	Dubr	Rapgef3
Rapgef3	Bbx	Xrcc6
Xrcc6	Nectin3	Desi1
Desi1	Tagln3	Pmm1
Pmm1	Cd200	Hdac7
Hdac7	Atg3	Polr3h
Polr3h	Slc35a5	Aco2
Aco2	Ccdc80	Nckap1l
Nckap1l	Gtpbp8	Pde1b
Pde1b	Mzt2	Ppp1r1a
Ppp1r1a	Prkdc	Shisa9
Shisa9	Mcm4	Txndc11
Txndc11	Ube2v2	Litaf
Litaf	Cep20	Nubp1
Nubp1	Nde1	Emp2
Emp2	Mpv17l	Hapstr1
Hapstr1	Pdxdc1	Bcl6
Bcl6	Ntan1	Il1rap
Il1rap	Rrn3	Anks3
Anks3	Bfar	Nudt16l1
Nudt16l1	Parn	Mgrn1
Mgrn1	Boc	Srl
Srl	Sidt1	Crebbp
Crebbp	Naa50	Fgf12
Fgf12	Ccdc191	Plaat1
Plaat1	Hira	Zfp251
Zfp251	Qtrt2	Hes1
Hes1	Mrpl40	Zfp263
Zfp263	Gbe1	Atp13a3
Atp13a3	Zbtb20	Glyr1
Glyr1	Usp7	Tmem44
Tmem44	Pmm2	Lsg1
Lsg1	Dgcr8	Rogdi
Rogdi	Trmt2a	Dnaaf8
Dnaaf8	Arl6	Eef2kmt
Eef2kmt	Crybg3	Ercc4
Ercc4	Riox2	Gpt
Gpt	Cpox	Apod

Apod	Cldnd1	Adck5
Adck5	St3gal6	Cyc1
Cyc1	Cmss1	Sharpin
Sharpin	Tbc1d23	Maf1
Maf1	Klhl22	Hgh1
Hgh1	Nit2	Dgat1
Dgat1	Tomm70a	Hsf1
Hsf1	Slc7a4	Bop1
Bop1	Tfg	Mroh1
Mroh1	Lrrc74b	Fbxl6
Fbxl6	Thap7	Slc52a2
Slc52a2	Lztr1	Gpaa1
Gpaa1	Ncam2	Oplah
Oplah	Aifm3	Grina
Grina	Snap29	Plec
Plec	Sdf2l1	Scrib
Scrib	Dlg1	Gfus
Gfus	Ppil2	Pycr3
Pycr3	Senp5	Naprt
Naprt	Ypel1	Ly6h
Ly6h	Ncbp2	Rhpn1
Rhpn1	Top3b	Ly6e
Ly6e	Pak2	Lynx1
Lynx1	Spag6l	Zbtb11
Zbtb11	Wdr53	Arc
Arc	Fgd4	Cep97
Cep97	Dnm1l	Ptk2
Ptk2	Igsf11	Mapk12
Mapk12	Tnk2	Lmf2
Lmf2	Yars2	Tymp
Tymp	B4galt4	Chkb
Chkb	Tfrc	Mapk8ip2
Mapk8ip2	Arhgap31	Arsa
Arsa	Fyttd1	Rabl2
Rabl2	Lrch3	Shank3
Shank3	Lmln	Kif21a
Kif21a	Cfap91	Yaf2
Yaf2	Osbpl11	Zcrb1
Zcrb1	Snx4	Alcam
Alcam	Zfp148	Cblb
Cblb	Gsk3b	Dubr
Dubr	Umps	Bbx
Bbx	Fstl1	Nectin3
Nectin3	Itgb5	Tagln3
Tagln3	Ndufb4	Cd200
Cd200	Abcc5	Atg3
Atg3	Rabl3	Slc35a5
Slc35a5	Gtf2e1	Ccdc80
Ccdc80	Stxbp5l	Gtpbp8
Gtpbp8	Hcls1	Mzt2
Mzt2	Mylk	Prkdc
Prkdc	Iqcb1	Mcm4
Mcm4	Adcy5	Ube2v2

Ube2v2	Ap2m1	Cep20
Cep20	Ece2	Nde1
Nde1	Clcn2	Mpv17l
Mpv17l	Slc49a4	Pdxdc1
Pdxdc1	Hspbap1	Ntan1
Ntan1	Ehhadh	Rrn3
Rrn3	Senp2	Bfar
Bfar	Tmem41a	Parn
Parn	Tra2b	Boc
Boc	Chodl	Sidt1
Sidt1	Dgkg	Naa50
Naa50	Btg3	Ccdc191
Ccdc191	D16Ertd472e	Hira
Hira	Cxadr	Qtrt2
Qtrt2	Usp25	Mrpl40
Mrpl40	Robo1	Gbe1
Gbe1	Eif4a2	Zbtb20
Zbtb20	St6gal1	Usp7
Usp7	Masp1	Pmm2
Pmm2	Mrpl39	Tmem114
Dgcr8	Atp5pf	Dgcr8
Trmt2a	App	Trmt2a
Arl6	Adamts1	Arl6
Crybg3	Ets2	Crybg3
Riox2	Dyrk1a	Riox2
Cpox	Vps26c	Cpox
Cldnd1	Slc15a2	Cldnd1
St3gal6	Kpna1	St3gal6
Cmss1	Arl13b	Cmss1
Tbc1d23	Pros1	Tbc1d23
Klhl22	Psmg1	Klhl22
Nit2	Brwd1	Nit2
Tomm70a	Grik1	Tomm70a
Slc7a4	Pigp	Slc7a4
Tfg	Dop1b	Tfg
Thap7	Cbr3	Thap7
Lztr1	Setd4	Lztr1
Ncam2	Clic6	Ncam2
Aifm3	Rcan1	Aifm3
Snap29	Atp5po	Snap29
Sdf2l1	Itsn1	Sdf2l1
Dlg1	Donson	Dlg1
Ppil2	Son	Ppil2
Senp5	Gart	Senp5
Ypel1	Tmem50b	Ypel1
Ncbp2	Ifngr2	Ncbp2
Top3b	Ifnar1	Top3b
Pak2	Ifnar2	Pak2
Spag6l	Cfap298	Spag6l
Wdr53	Synj1	Wdr53
Fgd4	Paxbp1	Fgd4
Dnm1l	Sod1	Dnm1l
Igsf11	Scaf4	Igsf11

Tnk2	Zfp641	Tnk2
Yars2	Kansl2	Yars2
B4galt4	Adcy6	B4galt4
Tfrc	Enah	Tfrc
Arhgap31	Lmbr1l	Arhgap31
Fyttd1	Tuba1b	Fyttd1
Lrch3	Prpf40b	Lrch3
Lmln	Fmnl3	Lmln
Cfap91	Nckap5l	Osbpl11
Osbpl11	Tmbim6	Snx4
Snx4	Faim2	Zfp148
Zfp148	Racgap1	Gsk3b
Gsk3b	Asic1	Umps
Umps	Smarcd1	Fstl1
Fstl1	Gpd1	Itgb5
Itgb5	Cox14	Ndufb4
Ndufb4	Cers5	Abcc5
Abcc5	Lima1	Rabl3
Rabl3	Larp4	Gtf2e1
Gtf2e1	Dip2b	Stxbp5l
Stxbp5l	Atf1	Hcls1
Hcls1	Slc11a2	Mylk
Mylk	Slc4a8	Iqcb1
Iqcb1	Scn8a	Adcy5
Adcy5	Nr4a1	Ap2m1
Ap2m1	Pcdhgc4	Ece2
Ece2	Csad	Clcn2
Clcn2	Igfbp6	Pdia5
Pdia5	Prr13	Thpo
Thpo	Map3k12	Slc49a4
Slc49a4	Tarbp2	Hspbap1
Hspbap1	Calcoco1	Ehhadh
Ehhadh	Rttn	Senp2
Senp2	Cdkn1a	Tmem41a
Tmem41a	Nus1	Tra2b
Tra2b	Cep89	Dgkg
Dgkg	Mospd1	Btg3
Btg3	Akirin1	D16Ertd472e
D16Ertd472e	Gtf2ird1	Cxadr
Cxadr	Noct	Usp25
Usp25	Abcc1	Robo1
Rfc4	Ndufa5	Eif4a2
Robo1	Fhl1	St6gal1
Eif4a2	Msrb2	Masp1
St6gal1	Rfc2	Mrpl39
Masp1	Denr	Atp5pf
Mrpl39	Prmt5	App
Atp5pf	Sympk	Adamts1
App	Nagpa	Ets2
Adamts1	Get1	Dyrk1a
Ets2	Ivns1abp	Vps26c
Dyrk1a	Rpp14	Slc15a2
Vps26c	Slc38a1	Kpna1

Slc15a2	Gps2	Arl13b
Kpna1	Bsg	Pros1
Arl13b	Vwa5a	Psmg1
Pros1	P3h3	Brwd1
Psmg1	Grm2	Grik1
Brwd1	Ccl25	Pigp
Grik1	Scg5	Dop1b
Pigp	Slc26a6	Cbr3
Dop1b	Acy1	Setd4
Cbr3	Frs3	Rcan1
Setd4	Creld2	Atp5po
Rcan1	Twf2	Itsn1
Atp5po	Zfp605	Donson
Itsn1	Ube2j2	Son
Donson	Marchf5	Gart
Son	Ache	Tmem50b
Gart	Wfdc1	Ifngr2
Tmem50b	Trip6	Ifnar1
Ifngr2	Agap3	Il10rb
Ifnar1	Tmem176a	Ifnar2
Il10rb	Dlx2	Cfap298
Ifnar2	Pisd	Synj1
Cfap298	Tpi1	Paxbp1
Synj1	Rab12	Mis18a
Paxbp1	Celsr3	Sod1
Mis18a	Pcbp4	Scaf4
Sod1	C1qtnf12	Zfp641
Scaf4	Ccndbp1	Kansl2
Zfp641	Ogfod2	Adcy6
Kansl2	Mrps23	Enah
Adcy6	Sfi1	Lmbr1l
Enah	Pigx	Tuba1b
Lmbr1l	Tiam2	Prpf40b
Tuba1b	Synj2	Fmnl3
Prpf40b	Rsph3b	Nckap5l
Fmnl3	Rps6ka2	Tmbim6
Nckap5l	Prkn	Faim2
Tmbim6	Agpat4	Racgap1
Faim2	Igf2r	Asic1
Racgap1	Acat2	Smarcd1
Asic1	Lnpep	Gpd1
Smarcd1	Chd1	Cox14
Gpd1	Mpc1	Cers5
Cox14	Pde10a	Lima1
Cers5	Phf10	Larp4
Lima1	Thbs2	Dip2b
Larp4	Hcfc1r1	Atf1
Dip2b	Tnfrsf12a	Slc11a2
Atf1	Paqr4	Slc4a8
Slc11a2	Flywch2	Scn8a
Slc4a8	Slc25a27	Nr4a1
Scn8a	Pla2g7	Pcdhgc4
Nr4a1	Tnfrsf21	Csad

Pcdhgc4	Mmut	Igfbp6
Csad	Tbc1d5	Prr13
Igfbp6	Satb1	Map3k12
Prr13	Cdc5l	Tarbp2
Map3k12	Aars2	Calcoco1
Tarbp2	Mrpl14	Rttn
Calcoco1	Slc29a1	Cdkn1a
Rttn	Hsp90ab1	Nus1
Cdkn1a	Slc5a7	Cep89
Nus1	Vegfa	Mospd1
Cep89	Gtpbp2	Akirin1
Mospd1	Polh	Gtf2ird1
Akirin1	Clic5	Noct
Gtf2ird1	Enpp5	Abcc1
Noct	Enpp4	Ndufa5
Abcc1	Calcr	Fhl1
Ndufa5	Fbxl17	Msrb2
Fhl1	Rsph9	Rfc2
Msrb2	Mrps18a	Denr
Rfc2	Rrp36	Prmt5
Denr	Ptk7	Sympk
Prmt5	Cnpy3	Nagpa
Sympk	Ubr2	Get1
Nagpa	Taf8	Ivns1abp
Get1	Gm20517	Rpp14
Ivns1abp	Bysl	Slc38a1
Rpp14	Tfeb	Gps2
Slc38a1	Foxp4	Bsg
Gps2	Trem2	Vwa5a
Bsg	Nfya	P3h3
Vwa5a	Kif6	Grm2
P3h3	Brd4	Il15ra
Grm2	Stk38	Serping1
Il15ra	Ppil1	Serinc2
Serinc2	Cpne5	Ccl25
Ccl25	Pi16	Scg5
Scg5	Mtch1	Parp3
Parp3	Fgd2	Slc26a6
Slc26a6	Ccdc167	Acy1
Acy1	Cmtr1	Frs3
Frs3	Glo1	Creld2
Creld2	Glp1r	Twf2
Twf2	Abcg1	Zfp605
Zfp605	Rsph1	Ube2j2
Ube2j2	Slc37a1	Marchf5
Marchf5	Wdr4	Ache
Ache	Ndufv3	Dtwd1
Dtwd1	Cbs	Wfdc1
Wfdc1	Sik1	Poc1a
Poc1a	Epb41l3	Trip6
Trip6	Akap8	Agap3
Agap3	Myl12a	Tmem176a
Tmem176a	Wiz	Dlx2

Dlx2	Lpin2	Pisd
Pisd	Emilin2	Tpi1
Tpi1	Smchd1	Rab12
Rab12	Cyp4f13	Celsr3
Celsr3	Clip4	Pcbp4
Pcbp4	Lbh	C1qtnf12
C1qtnf12	Galnt14	Ccndbp1
Ccndbp1	Ehd3	Ogfod2
Ogfod2	Xdh	Mrps23
Mrps23	Dpy30	Rhebl1
Rhebl1	Spast	Sfi1
Sfi1	Slc30a6	Pigx
Pigx	Prkd3	Pisd-ps2
Pisd-ps2	Yipf4	Tiam2
Tiam2	Birc6	Synj2
Synj2	Crim1	Rsph3b
Rsph3b	Vit	Rps6ka2
Rps6ka2	Strn	Prkn
Prkn	Ttc27	Agpat4
Agpat4	Cebpz	Igf2r
Slc22a3	Ndufaf7	Acat2
Igf2r	Pja2	Lnpep
Acat2	Qpct	Chd1
Lnpep	Man2a1	Mpc1
Chd1	Vapa	Pde10a
Mpc1	Hnrnpll	Phf10
Pde10a	Ralbp1	Thbs2
Phf10	Srsf7	Smoc2
Thbs2	Twsg1	Hcfc1r1
Smoc2	Ndufv2	Tnfrsf12a
Zfp51	Washc1	Paqr4
Hcfc1r1	Washc2	Flywch2
Tnfrsf12a	Nrxn1	Slc25a27
Pkmyt1	Cacna1h	Pla2g7
Paqr4	Tedc2	Tnfrsf21
Flywch2	Lrpprc	Adgrf4
Slc25a27	Atp6v0c	Mmut
Pla2g7	Pdpk1	Tbc1d5
Tnfrsf21	Prepl	Satb1
Cenpq	Abca3	Cdc5l
Mmut	Eci1	Spats1
Tbc1d5	Srbd1	Aars2
Satb1	E4f1	Mrpl14
Cdc5l	Epas1	Slc29a1
Spats1	Mlst8	Hsp90ab1
Aars2	Rhoq	Nfkbie
Mrpl14	Cript	Vegfa
Slc29a1	Mcfd2	Gtpbp2
Hsp90ab1	Msh2	Polh
Nfkbie	Hagh	Clic5
Tcte1	Spsb3	Enpp5
Vegfa	Mapk8ip3	Enpp4
Gtpbp2	C3	Fbxl17

Polh	Jpt2	Rsph9
Clic5	Tmem204	Mrps18a
Enpp5	Ift140	Rrp36
Enpp4	Telo2	Ptk7
Fbxl17	St6gal2	Cnpy3
Rsph9	Tekt4	Ubr2
Mrps18a	Sox8	Taf8
Rrp36	Pgap6	Gm20517
Ptk7	Mrpl28	Bysl
Cnpy3	Axin1	Tfeb
Ubr2	Rgs11	Foxp4
Taf8	Fam234a	Trem2
Gm20517	Luc7l	Nfya
Bysl	Dusp1	Kif6
Tfeb	Bnip1	Brd4
Foxp4	Phf1	Stk38
Trem2	Cuta	Ppil1
Nfya	Plin3	Cpne5
Kif6	Kdm4b	Mtch1
Brd4	Rfx2	Fgd2
Stk38	Uqcc2	Pim1
Ppil1	Grm8	Ccdc167
Cpne5	Mllt1	Cmtr1
Mtch1	Nudt3	Glo1
Fgd2	Snrpc	Glp1r
Pim1	Taf11	Abcg1
Ccdc167	Anks1	Rsph1
Cmtr1	Zfp523	Slc37a1
Glo1	Fkbp5	Wdr4
Glp1r	Nudt12	Ndufv3
Abcg1	Cul2	Cbs
Rsph1	Mtpap	Sik1
Slc37a1	Svil	Epb41l3
Wdr4	Zeb1	Akap8
Ndufv3	Epc1	Myl12a
Cbs	Sos1	Myom1
Sik1	Map4k3	Wiz
Epb41l3	Tmem178	Lpin2
Akap8	Thumpd2	Smchd1
Myl12a	Pkdcc	Cyp4f13
Myom1	Cox7a2l	Clip4
Wiz	Thada	Lbh
Lpin2	Dync2li1	Galnt14
Emilin2	Polr2d	Ehd3
Smchd1	Slc25a46	Xdh
Cyp4f13	Sap130	Dpy30
Clip4	Syt4	Spast
Lbh	Celf4	Slc30a6
Galnt14	Tpgs2	Prkd3
Ehd3	Slc39a6	Yipf4
Xdh	Elp2	Birc6
Dpy30	2700062C07Rik	Crim1
Spast	Zfp397	Strn

Slc30a6	Mapre2	Ttc27
Prkd3	Wac	Eif2ak2
Yipf4	Ccny	Cebpz
Birc6	Thoc1	Ndufaf7
Crim1	Rock1	Pja2
Strn	Cyp4f14	Qpct
Ttc27	Esco1	Man2a1
Cebpz	Mib1	Cyp1b1
Ndufaf7	Zfp871	Vapa
Pja2	Adamts10	Hnrnpll
Qpct	Kifc5b	Ralbp1
Man2a1	Dtna	Srsf7
Vapa	Cdh2	Twsg1
Hnrnpll	Tapbp	Ndufv2
Ralbp1	Pfdn6	Washc1
Srsf7	Wdr46	Washc2
Twsg1	Rnf138	Nrxn1
Ndufv2	Vps52	Cacna1h
Washc1	Ring1	Tedc2
Washc2	Slc39a7	Lrpprc
Nrxn1	Col11a2	Atp6v0c
Cacna1h	Brd2	Pdpk1
Tedc2	Tap2	Prepl
Lrpprc	Pfdn1	Abca3
Atp6v0c	Psd2	Eci1
Pdpk1	Dnajc18	Srbd1
Prepl	Sil1	E4f1
Abca3	Hspa9	Epas1
Eci1	Etf1	Mlst8
Srbd1	Nelfe	Rhoq
E4f1	Cdc23	Pigf
Epas1	Epb41l4a	Cript
Mlst8	Stard4	Mcfd2
Rhoq	Bin1	Msh2
Pigf	Ercc3	Hagh
Cript	Map3k2	Spsb3
Mcfd2	Iws1	Mapk8ip3
Msh2	Csnk2b	Jpt2
Hagh	Bag6	Tmem204
Spsb3	Prrc2a	Ift140
Mapk8ip3	Lims2	Telo2
Jpt2	Wdr33	St6gal2
Tmem204	Atp6v1g2	Pot1b
Ift140	Riok3	Sox8
Telo2	Rmc1	Pgap6
St6gal2	Aqp4	Mrpl28
Sox8	Npc1	Axin1
Pgap6	Mrpl27	Rgs11
Mrpl28	Zfp521	Fam234a
Axin1	Lama3	Luc7l
Rgs11	Dhx16	Dusp1
Fam234a	Impact	Bnip1
Luc7l	Ttc39c	Phf1

Dusp1	Ndfip1	Cuta
Bnip1	Atat1	Plin3
Phf1	Spry4	Kdm4b
Cuta	Gnl1	Rfx2
Plin3	Nr3c1	Uqcc2
Kdm4b	Mrps18b	Grm8
Rfx2	Gm8615	Mllt1
Uqcc2	Dele1	Nudt3
Grm8	Rpp21	Snrpc
Mllt1	Arap3	Taf11
Nudt3	Hdac3	Anks1
Snrpc	Diaph1	Zfp523
Taf11	Trim26	Fkbp5
Anks1	Gabbr1	Nudt12
Zfp523	Dcp2	Cul2
Fkbp5	Ik	Bambi
Pdzph1	Pggt1b	Mtpap
Nudt12	Mal2	Svil
Cul2	Ap3s1	Zeb1
Mtpap	Ankhd1	Epc1
Svil	Hbegf	Sos1
Zeb1	Yipf5	Map4k3
Epc1	Rbm27	Tmem178
Sos1	Lars1	Thumpd2
Map4k3	Tcerg1	Pkdcc
Tmem178	Ppp2r2b	Cox7a2l
Thumpd2	Dpysl3	Thada
Pkdcc	Jakmip2	Dync2li1
Cox7a2l	Hsd17b4	Adcyap1
Thada	Rab27b	Polr2d
Dync2li1	Mbd2	Slc25a46
Adcyap1	Smad4	Sap130
Polr2d	Sec11c	Syt4
Slc25a46	Pmaip1	Celf4
Sap130	Gnal	Tpgs2
Syt4	Impa2	Slc39a6
Celf4	Afg3l2	Elp2
Tpgs2	Srfbp1	2700062C07Rik
Slc39a6	Prelid3a	Zfp397
Elp2	Spire1	Mapre2
2700062C07Rik	Sncaip	Wac
Zfp397	Snx24	Ccny
Mapre2	Psmg2	Thoc1
Wac	Ptpn2	Rock1
Ccny	Cep192	Cyp4f14
Thoc1	Ldlrad4	Esco1
Rock1	Setbp1	Mib1
Cyp4f14	Galr1	Zfp871
Esco1	Me2	Adamts10
Mib1	Mapk4	Myo1f
Zfp871	Cxxc1	Kifc5b
Adamts10	Mbd1	Dtna
Myo1f	Smad2	Cdh2

Kifc5b
Dtna
Cdh2
Tapbp
Pfdn6
Wdr46
Rnf138
Vps52
Ring1
Slc39a7
Col11a2
Brd2
Psmb8
Tap2
Pfdn1
Psd2
Sting1
Dnajc18
Spata24
Sil1
Hspa9
Etf1
Nelfe
Cdc23
Epb41l4a
Stard4
Bin1
Ercc3
Map3k2
Iws1
Csnk2b
Bag6
Prrc2a
Lims2
Aif1
Wdr33
Atp6v1g2
Riok3
Rmc1
Aqp4
Npc1
Mrpl27
Zfp521
Dhx16
Impact
Ttc39c
Ndfip1
Atat1
Spry4
Gnl1
Nr3c1
Mrps18b
Gm8615
Sall3
Atp9b
Rbfa
Naa12
Csnk1a1
Pcyox1l
Grpel2
Napg
Txnl1
Nars1
Fech
Nedd4l
Lmnb1
C330018D20Rik
Megf10
Prrc1
Slc12a2
Fbn2
Isoc1
Dctn4
Rbm22
Rps14
Tcof1
Tmx3
Camk2a
Pdgfrb
Csf1r
Hmgxb3
Gnaq
Psat1
Tle4
Cndp2
Timm21
Cyb5a
Cbln2
Asrgl1
Incenp
Fth1
Rab3il1
Fads3
Fads2
Tmem138
Tmem216
Sdhaf2
Mrpl16
Osbp
Zfp91
Rfk
Pcsk5
Ostf1
Carnmt1
Ccdc86
Prpf19
Tapbp
Pfdn6
Wdr46
Rnf138
Vps52
Ring1
Slc39a7
Col11a2
Brd2
Psmb8
Tap2
Pfdn1
Psd2
Sting1
Dnajc18
Spata24
Sil1
Hspa9
Etf1
Nelfe
Cdc23
Epb41l4a
Stard4
Bin1
Ercc3
Map3k2
Iws1
Csnk2b
Bag6
Prrc2a
Lims2
Aif1
Wdr33
Atp6v1g2
Riok3
Rmc1
Aqp4
Npc1
Mrpl27
Zfp521
Lama3
Dhx16
Impact
Ttc39c
Ndfip1
Atat1
Spry4
Gnl1
Nr3c1
Mrps18b
Gm8615
Dele1
Rpp21

Dele1	Tmem132a	Arap3
Rpp21	Ddb1	Hdac3
Arap3	Fen1	Diaph1
Hdac3	Syt7	Trim26
Diaph1	Zfand5	Gabbr1
Trim26	Cemip2	Dcp2
Gabbr1	Rtn3	Ik
Dcp2	Atl3	Pggt1b
Ik	Naa40	Mal2
Pggt1b	Lipo3	Ap3s1
Mal2	Otub1	Ankhd1
Ap3s1	Cdc42bpg	Hbegf
Ankhd1	Ehd1	Yipf5
Hbegf	Atg2a	Rbm27
Yipf5	Stambpl1	Lars1
Rbm27	Ppp2r5b	Tcerg1
Lars1	Cdc37l1	Ppp2r2b
Tcerg1	Lipa	Dpysl3
Ppp2r2b	Ak3	Jakmip2
Dpysl3	Rcl1	Dtwd2
Jakmip2	Snx15	Hsd17b4
Dtwd2	Jak2	Rab27b
Hsd17b4	Sac3d1	Mbd2
Rab27b	Zfpl1	Smad4
Mbd2	Vps51	Sec11c
Smad4	Htr7	Grp
Sec11c	Tm7sf2	Pmaip1
Grp	Rpp30	Gnal
Pmaip1	Pcgf5	Impa2
Gnal	Syvn1	Cidea
Impa2	Il33	Afg3l2
Cidea	Tnks2	Srfbp1
Afg3l2	Tjp2	Prelid3a
Srfbp1	Frmd8	Spire1
Prelid3a	Uhrf2	Sncaip
Spire1	Rad9a	Snx24
Sncaip	Dpf2	Psmg2
Snx24	Gldc	Ppic
Psmg2	Mrpl21	Ptpn2
Ptpn2	Rps6kb2	Cep192
Cep192	Ighmbp2	Ldlrad4
Ldlrad4	Pola2	Setbp1
Setbp1	Coro1b	Me2
Me2	Eif1ad	Mapk4
Mapk4	Chka	Cxxc1
Cxxc1	Banf1	Mbd1
Mbd1	Tmem134	Smad2
Smad2	Aip	Sall3
Sall3	Pitpnm1	Atp9b
Atp9b	Sf3b2	Rbfa
Rbfa	Pold4	Naa12
Naa12	Pacs1	Csnk1a1
Csnk1a1	Cdk2ap2	Pcyox1l

Pcyox1l	Grk2	Grpel2
Grpel2	Klc2	Napg
Napg	Pip5k1b	Txnl1
Txnl1	Rab1b	Nars1
Nars1	Cnih2	Fech
Fech	Yif1a	Nedd4l
Nedd4l	Zng1	Lmnb1
Lmnb1	Rin1	C330018D20Rik
C330018D20Rik	Aldh3b1	Megf10
Megf10	Asah2	Prrc1
Prrc1	Rce1	Slc12a2
Slc12a2	Slc29a2	Fbn2
Fbn2	Pcx	Isoc1
Isoc1	Minpp1	Dctn4
Dctn4	Apba1	Rbm22
Rbm22	Papss2	Rps14
Rps14	Cpt1a	Tcof1
Tcof1	Peli3	Tmx3
Tmx3	Mrpl11	Camk2a
Camk2a	Mus81	Pdgfrb
Pdgfrb	Gal	Csf1r
Csf1r	Ppp6r3	Hmgxb3
Hmgxb3	Efemp2	Gnaq
Gnaq	Fibp	Psat1
Psat1	Lrp5	Tle4
Tle4	Drap1	Cndp2
Cndp2	Smarca2	Timm21
Timm21	Vldlr	Cyb5a
Cyb5a	Rnaseh2c	Cbln2
Cbln2	Kat5	Asrgl1
Asrgl1	Rela	Incenp
Incenp	Slc1a1	Fth1
Fth1	Ehbp1l1	Rab3il1
Rab3il1	Fam89b	Fads3
Fads3	Ltbp3	Fads2
Fads2	Scyl1	Tmem138
Tmem138	Capn1	Tmem216
Tmem216	Smc5	Sdhaf2
Sdhaf2	Arl2	Mrpl16
Cd6	Men1	Osbp
Mrpl16	Map4k2	Zfp91
Osbp	Sf1	Rfk
Fam111a	Prdx5	Pcsk5
Zfp91	Esrra	Ostf1
Rfk	Gpr137	Carnmt1
Pcsk5	Bad	Ccdc86
Ostf1	Plcb3	Prpf19
Carnmt1	Vegfb	Tmem132a
Ccdc86	Dnajc4	Slc15a3
Prpf19	Fermt3	Ddb1
Tmem132a	Stip1	Fen1
Slc15a3	Rcor2	Syt7
Ddb1	Mark2	Zfand5

Fen1	Spindoc	Cemip2
Syt7	Smc3	Rtn3
Zfand5	Pdcd4	Atl3
Cemip2	Shoc2	Naa40
Rtn3	Gpam	Lipo3
Atl3	Acsl5	Otub1
Naa40	Zdhhc6	Cdc42bpg
Lipo3	Vti1a	Ehd1
Otub1	Tcf7l2	Atg2a
Cdc42bpg	Rbp4	Stambpl1
Ehd1	Eif3a	Ppp2r5b
Atg2a	Dennd10	Cdc37l1
Stambpl1	Prdx3	Lipa
Ppp2r5b	Plce1	Ak3
Cdc37l1	Noc3l	Rcl1
Lipa	Sorbs1	Snx15
Ak3	Aldh18a1	Jak2
Rcl1	Tctn3	Sac3d1
Snx15	Ccnj	Zfpl1
Jak2	Tm9sf3	Tnfrsf25
Sac3d1	Lcor	Vps51
Zfpl1	Slit1	Htr7
Vps51	Smndc1	Tm7sf2
Htr7	Mxi1	Rpp30
Tm7sf2	Add3	Pcgf5
Rpp30	Xpnpep1	Syvn1
Pcgf5	Trim8	Il33
Syvn1	Arl3	Tnks2
Il33	Sfxn2	Tjp2
Tnks2	Maoa	Frmd8
Tjp2	Fundc1	Uhrf2
Frmd8	Nt5c2	Rad9a
Uhrf2	Pdcd11	Dpf2
Rad9a	Taf5	Gldc
Dpf2	Pcgf6	Mrpl21
Gldc	Gk	Rps6kb2
Mrpl21	Slk	Ighmbp2
Rps6kb2	Sfr1	Pola2
Ighmbp2	Gsto1	Coro1b
Pola2	Casp7	Eif1ad
Coro1b	Dclre1a	Chka
Eif1ad	Nhlrc2	Banf1
Chka	Afap1l2	Tmem134
Banf1	Ablim1	Aip
Tmem134	Trub1	Pitpnm1
Cst6	Gfra1	Sf3b2
Aip	Hspa12a	Pold4
Pitpnm1	Slc18a2	Pacs1
Sf3b2	3110040N11Rik	Cdk2ap2
Pold4	Btbd1	Grk2
Pacs1	Hdgfl3	Klc2
Cdk2ap2	P4hb	Pip5k1b
Grk2	Arhgdia	Rab1b

Klc2	Ints4	Cnih2
Pip5k1b	Alyref	Yif1a
Rab1b	Anapc11	Zng1
Cnih2	Pcyt2	Rin1
Yif1a	Sirt7	Aldh3b1
Zng1	Tollip	Asah2
Rin1	Aspscr1	Rce1
Aldh3b1	Cenpx	Slc29a2
Asah2	Lrrc45	Pcx
Rce1	Mob2	Minpp1
Slc29a2	Maged1	Apba1
Pcx	Fasn	Papss2
Minpp1	Arhgap19	Cpt1a
Apba1	Dus1l	Peli3
Papss2	Gps1	Mrpl11
Cpt1a	Zdhhc16	Mus81
Peli3	Rfng	Ppp6r3
Mrpl11	Mms19	Efemp2
Mus81	Csnk1d	Fibp
Ppp6r3	Ogfod3	Lrp5
Efemp2	Rab40b	Drap1
Fibp	Ubtd1	Smarca2
Lrp5	Wdr45b	Vldlr
Drap1	Fn3k	Rnaseh2c
Smarca2	Pi4k2a	Kat5
Vldlr	R3hcc1l	Rela
Rnaseh2c	Hps1	Slc1a1
Kat5	Cnnm1	Ehbp1l1
Rela	Got1	Fam89b
Slc1a1	Entpd7	Ltbp3
Ehbp1l1	Cutc	Scyl1
Fam89b	Dnmbp	Capn1
Ltbp3	Erlin1	Smc5
Scyl1	Chuk	Arl2
Capn1	Cwf19l1	Men1
Smc5	Scd2	Map4k2
Arl2	Ndufb8	Sf1
Men1	Sema4g	Prdx5
Map4k2	Mrpl43	Esrra
Sf1	Twnk	Kcnk4
Prdx5	Sfxn3	Gpr137
Esrra	Btrc	Bad
Kcnk4	Poll	Plcb3
Gpr137	Oga	Vegfb
Bad	Kcnip2	Dnajc4
Plcb3	Ldb1	Fermt3
Vegfb	Gbf1	Stip1
Dnajc4	Nfkb2	Rcor2
Fermt3	Fbxl15	Mark2
Stip1	Mfsd13a	Spindoc
Rcor2	Actr1a	Smc3
Mark2	Sufu	Pdcd4
Spindoc	Hexa	Shoc2

Smc3
Pdcd4
Shoc2
Gpam
Acsl5
Zdhhc6
Vti1a
Tcf7l2
Rbp4
Eif3a
Dennd10
Prdx3
Plce1
Noc3l
Sorbs1
Aldh18a1
Tctn3
Ccnj
Tll2
Tm9sf3
Pik3ap1
Lcor
Slit1
Smndc1
Mxi1
Add3
Xpnpep1
Trim8
Arl3
Maoa
Fundc1
Nt5c2
Pdcd11
Taf5
Pcgf6
Gk
Slk
Sfr1
Gsto1
Casp7
Dclre1a
Nhlrc2
Afap1l2
Ablim1
Trub1
Gfra1
Hspa12a
3110040N11Rik
Btbd1
Hdgfl3
Bhlhe22
P4hb
Arhgdia

Arih1
Bbs4
Adpgk
Parp6
Limd1
Sacm1l
Fyco1
Lztfl1
Tbl1x
Zfhx4
Hsd17b10
Huwe1
Fam120c
Tsr2
Fgd1
Gnl3l
Maged2
Apex2
Alas2
Tro
Abhd6
Flnb
Polr3a
Sat1
Acot9
Prdx4
Rps24
Ptprj
Banp
Jph3
Itgb8
Sp4
Atp10a
Ube3a
Kdm5c
Sbds
Rabgef1
Itga7
Rdh5
Cd63
Gdf11
Dnajc14
Dgka
Rps26
Pa2g4
Esyt1
Smarcc2
Cdh9
Chmp6
Baiap2
Rnf41
Nabp2
Aatk

Gpam
Acsl5
Zdhhc6
Vti1a
Tcf7l2
Eif3a
Dennd10
Prdx3
Plce1
Noc3l
Sorbs1
Aldh18a1
Tctn3
Ccnj
Tm9sf3
Pik3ap1
Lcor
Slit1
Smndc1
Mxi1
Add3
Xpnpep1
Trim8
Arl3
Sfxn2
Maoa
Fundc1
Nt5c2
Pdcd11
Taf5
Pcgf6
Gk
Slk
Sfr1
Gsto1
Dclre1a
Nhlrc2
Afap1l2
Ablim1
Trub1
Gfra1
Hspa12a
Slc18a2
3110040N11Rik
Btbd1
Hdgfl3
Bhlhe22
P4hb
Arhgdia
Ints4
Alyref
Anapc11
Pcyt2

Ints4
Alyref
Anapc11
Pcyt2
Sirt7
Tollip
Aspscr1
Cenpx
Lrrc45
Mob2
Maged1
Fasn
Arhgap19
Dus1l
Gps1
Zdhhc16
Rfng
Mms19
Slc16a3
Csnk1d
Ogfod3
Rab40b
Ubtd1
Wdr45b
Fn3k
Pi4k2a
R3hcc1l
Hps1
Cnnm1
Got1
Entpd7
Cutc
Dnmbp
Erlin1
Chuk
Cwf19l1
Scd3
Scd2
Ndufb8
Sema4g
Mrpl43
Twnk
Sfxn3
Kazald1
Btrc
Poll
Oga
Kcnip2
Ldb1
Gbf1
Fbxl15
Mfsd13a
Actr1a
Tepsin
Cnpy2
Faap100
Atp5f1b
Tac2
Nab2
Shmt2
R3hdm2
Gli1
Ddit3
Mbd6
Dctn2
Ttc4
Pip4k2c
Katnal2
Hdhd2
Agap2
Pias2
St8sia5
Ark2c
Atp5f1a
Pstpip2
Atp23
Usp33
Clns1a
Paip1
Nnt
Paox
Echs1
Fuom
Caly
Zfp511
Tubgcp2
Adgra1
Inpp5a
Dpysl4
Bet1l
Ric8a
Sirt3
Psmd13
Ifitm3
Ptdss2
Hras
Taldo1
Tmem80
Rplp2
Pnpla2
Cd151
Tspan4
Chid1
Tmem192
Zfp711
Chm
Sirt7
Tollip
Myadml2
Aspscr1
Cenpx
Lrrc45
Mob2
Maged1
Fasn
Arhgap19
Dus1l
Gps1
Zdhhc16
Rfng
Mms19
Csnk1d
Ogfod3
Rab40b
Ubtd1
Wdr45b
Fn3k
Pi4k2a
R3hcc1l
Hps1
Cnnm1
Got1
Entpd7
Cutc
Dnmbp
Erlin1
Chuk
Cwf19l1
Scd2
Ndufb8
Sema4g
Mrpl43
Twnk
Sfxn3
Kazald1
Btrc
Poll
Oga
Kcnip2
Ldb1
Gbf1
Nfkb2
Fbxl15
Mfsd13a
Actr1a
Sufu
Hexa
Arih1
Bbs4

Sufu	Crcp	Adpgk
Hexa	Asl	Parp6
Arih1	Gusb	Limd1
Bbs4	Phkg1	Sacm1l
Adpgk	Sumf2	Fyco1
Parp6	Tm9sf2	Lztfl1
Limd1	Clybl	Tbl1x
Sacm1l	Fgf14	Zfhx4
Fyco1	Farp1	Hsd17b10
Lztfl1	Dock9	Huwe1
Tbl1x	Tnrc6c	Fam120c
Zfhx4	Tmc6	Tsr2
Hsd17b10	Cant1	Fgd1
Huwe1	Rbfox3	Gnl3l
Fam120c	Cbx2	Maged2
Tsr2	Cbx8	Apex2
Fgd1	Gaa	Alas2
Gnl3l	Eif4a3	Tro
Maged2	Nptx1	Abhd6
Apex2	Rptor	Flnb
Alas2	Pde8a	Polr3a
Tro	Cpeb1	Sat1
Abhd6	Tma16	Acot9
Flnb	Klhl4	Prdx4
Polr3a	Zfp202	Rps24
Sat1	Copg2	Ptprj
Acot9	Podxl	Banp
Prdx4	Mkln1	Jph3
Rps24	Bach1	Itgb8
Ptprj	Cct8	Sp4
Banp	Usp16	Atp10a
Jph3	Phf6	Ube3a
Itgb8	Hprt1	Kdm5c
Sp4	Ccdc51	Sbds
Atp10a	Atrip	Rabgef1
Ube3a	Shisa5	Itga7
Kdm5c	Pfkfb4	Rdh5
Sbds	Uqcrc1	Cd63
Rabgef1	Arhgef9	Gdf11
Itga7	Cnksr2	Ormdl2
Rdh5	Rps6ka6	Dnajc14
Cd63	Tmem47	Dgka
Gdf11	Marchf8	Rps26
Ormdl2	Wdr73	Pa2g4
Dnajc14	Nmb	Esyt1
Dgka	Sec11a	Smarcc2
Rps26	Pigq	Cdh9
Pa2g4	Rab40c	Chmp6
Esyt1	Mettl26	Baiap2
Smarcc2	Mcrip2	Rnf41
Cdh9	Rhot2	Nabp2
Chmp6	Rhbdl1	Aatk
Baiap2	Jmjd8	Tepsin

Rnf41	Wdr24	Cnpy2
Nabp2	Fbxl16	Faap100
Aatk	Gng13	Atp5f1b
Tepsin	Prps2	Prim1
Cnpy2	Sdc3	Nab2
Faap100	Hadha	Shmt2
Atp5f1b	Hspa4l	R3hdm2
Prim1	Mfsd8	Ddit3
Tac2	Larp1b	Mbd6
Nab2	Jade1	Dctn2
Shmt2	D3Ertd751e	Ttc4
R3hdm2	Gdap1	Pip4k2c
Gli1	Itih5	Katnal2
Ddit3	Atp5f1c	Hdhd2
Mbd6	Taf3	Agap2
Dctn2	Exosc7	Pias2
Ttc4	Zdhhc3	St8sia5
Pip4k2c	St8sia2	Ark2c
Katnal2	Slco3a1	Atp5f1a
Hdhd2	Pgm1	Pstpip2
Agap2	Slc25a10	Atp23
Pias2	Hgs	Usp33
St8sia5	Rpl14	Clns1a
Ark2c	Rassf3	Paip1
Atp5f1a	Itgb1	Nnt
Pstpip2	Nrp1	Paox
Atp23	Pard3	Echs1
Usp33	Homer2	Fuom
Clns1a	Dhtkd1	Caly
Paip1	Sec61a2	Zfp511
Nnt	Nudt5	Adam8
Paox	Zfp282	Tubgcp2
Echs1	Pdia4	Adgra1
Fuom	Iscu	Inpp5a
Caly	Fam20c	Dpysl4
Zfp511	Prkar1b	Bet1l
Tubgcp2	Pdgfa	Ric8a
Adgra1	Dnaaf5	Sirt3
Inpp5a	Get4	Psmd13
Dpysl4	Xiap	Ifitm3
Bet1l	Stag2	Ptdss2
Ric8a	Cplx2	Hras
Sirt3	Higd2a	Taldo1
Psmd13	Nop16	Eps8l2
Ifitm3	Arl10	Tmem80
Ptdss2	4833439L19Rik	Rplp2
Hras	Thoc3	Pnpla2
Taldo1	Faf2	Cd151
Tmem80	Tspan17	Tspan4
Rplp2	Unc5a	Chid1
Pnpla2	Uimc1	Tmem192
Cd151	Smad7	Apool
Tspan4	Myo5b	Zfp711

Chid1	Snca	Chm
Tmem192	Gria4	Crcp
Apool	Kbtbd3	Asl
Zfp711	Aasdhppt	Gusb
Chm	Cwf19l2	Phkg1
Crcp	Alkbh8	Sumf2
Asl	Sox17	Tm9sf2
Gusb	Lypla1	Clybl
Phkg1	Oprk1	Fgf14
Sumf2	Rb1cc1	Farp1
Tm9sf2	Sntg1	Dock9
Clybl	Adhfe1	Tnrc6c
Fgf14	Sgk3	Tmc6
Farp1	Cops5	Cant1
Dock9	Stau2	Rbfox3
Tnrc6c	Rdh10	Cbx2
Tmc6	Terf1	Cbx8
Cant1	Paqr8	Gaa
Rbfox3	Eya1	Eif4a3
Cbx2	Tmem14a	Nptx1
Cbx8	Tram1	Rptor
Gaa	Lactb2	Pde8a
Eif4a3	Ube2w	Cpeb1
Nptx1	Tmem70	Tma16
Rptor	Pikfyve	Klhl4
Pde8a	Idh1	Zfp202
Cpeb1	Mettl21a	Copg2
Tma16	Creb1	Podxl
Klhl4	Klf7	Mkln1
Zfp202	Fastkd2	Bach1
Copg2	Adam23	Cct8
Podxl	Eef1b2	Usp16
Mkln1	Ndufs1	Phf6
Bach1	Nrp2	Hprt1
Cct8	Maip1	Ccdc51
Usp16	Rftn2	Atrip
Phf6	Mob4	Shisa5
Hprt1	Hspd1	Pfkfb4
Ccdc51	Coq10b	Uqcrc1
Atrip	Sf3b1	Arhgef9
Shisa5	Slc39a10	Cnksr2
Pfkfb4	Slc40a1	Rps6ka6
Uqcrc1	Wdr75	Tmem47
Arhgef9	Ikzf2	Marchf8
Cnksr2	Lancl1	Wdr73
Rps6ka6	Acadl	Nmb
Tmem47	Kansl1l	Sec11a
Marchf8	Rpe	Pigq
Wdr73	Raph1	Rab40c
Sec11a	Carf	Mettl26
Pigq	Ica1l	Mcrip2
Rab40c	Wdr12	Rhot2
Mettl26	Nop58	Rhbdl1

Mcrip2	Sumo1	Jmjd8
Rhot2	Als2	Wdr24
Rhbdl1	Stradb	Fbxl16
Jmjd8	Trak2	Gng13
Wdr24	Cflar	Prps2
Fbxl16	Ndufb3	Sdc3
Gng13	Clk1	Hadha
Prps2	Ppil3	Tyms
Sdc3	Nif3l1	Hspa4l
Hadha	Orc2	Plk4
Hspa4l	Col5a2	Mfsd8
Plk4	Col3a1	Larp1b
Mfsd8	Poglut2	Jade1
Larp1b	Ercc5	D3Ertd751e
Jade1	Khdrbs2	Crispld1
D3Ertd751e	Ptp4a1	Gdap1
Crispld1	Il1r1	Itih5
Gdap1	Map4k4	Atp5f1c
Itih5	Npas2	Taf3
Atp5f1c	Pdcl3	Exosc7
Taf3	Chst10	Zdhhc3
Exosc7	Rev1	Slco3a1
Zdhhc3	Eif5b	Pgm1
St8sia2	Mrpl30	Slc25a10
Slco3a1	Cracdl	Hgs
Pgm1	Asnsd1	Rpl14
Slc25a10	Osgepl1	Rassf3
Hgs	Pms1	Itgb1
Rpl14	Inpp1	Nrp1
Rassf3	Gls	Pard3
Itgb1	Stat1	Homer2
Nrp1	Nabp1	Dhtkd1
Pard3	Tmeff2	Sec61a2
Homer2	Mgat4a	Nudt5
Dhtkd1	Unc50	Zfp282
Sec61a2	Coa5	Pdia4
Nudt5	Inpp4a	Iscu
Zfp282	Tmem131	Fam20c
Pdia4	Sema4c	Prkar1b
Iscu	Plekhb2	Pdgfa
Fam20c	Imp4	Dnaaf5
Prkar1b	Dst	Get4
Pdgfa	Prim2	Xiap
Dnaaf5	Zfp142	Stag2
Get4	Rhbdd1	Cplx2
Xiap	Mff	Higd2a
Stag2	Fam135a	Nop16
Cplx2	Sdhaf4	Arl10
Higd2a	Smap1	4833439L19Rik
Nop16	B3gat2	Thoc3
Arl10	Ogfrl1	Faf2
4833439L19Rik	Agfg1	Tspan17
Thoc3	Sphkap	Unc5a

Faf2	Rnf25	Uimc1
Tspan17	Bcs1l	Smad7
Unc5a	Plcd4	Myo5b
Uimc1	Cnot9	Snca
Smad7	Ctdsp1	Gria4
Myo5b	Pnkd	Kbtbd3
Snca	Ppm1f	Aasdhppt
Gria4	Igfbp5	Cwf19l2
Kbtbd3	Xrcc5	Alkbh8
Aasdhppt	Tmem169	Sox17
Cwf19l2	Atic	Lypla1
Alkbh8	Fn1	Rb1cc1
Sox17	Zfand2b	Sntg1
Lypla1	Abcb6	Adhfe1
Oprk1	Ankzf1	Sgk3
Rb1cc1	Glb1l	Cops5
Sntg1	Stk16	Stau2
Adhfe1	Tuba4a	Rdh10
Sgk3	Dnajb2	Terf1
Cops5	Ptprn	Paqr8
Stau2	Speg	Eya1
Rdh10	Dnpep	Tmem14a
Terf1	Obsl1	Tram1
Msc	Stk11ip	Lactb2
Paqr8	Trip12	Slco5a1
Eya1	Slc16a14	Ube2w
Tmem14a	Itm2c	Tmem70
Tram1	4933407L21Rik	Pikfyve
Lactb2	2810459M11Rik	Idh1
Ube2w	Psmd1	Mettl21a
Tmem70	Ncl	Creb1
Pikfyve	Epha4	Klf7
Idh1	Ptma	Fastkd2
Mettl21a	Pde6d	Adam23
Creb1	Cops7b	Eef1b2
Klf7	Farsb	Ndufs1
Fastkd2	Ecel1	Nrp2
Adam23	Mrpl44	Maip1
Eef1b2	Serpine2	Rftn2
Ndufs1	Eif4e2	Mob4
Nrp2	Efhd1	Hspd1
Maip1	Ngef	Coq10b
Rftn2	Ndufa10	Sf3b1
Mob4	Rnpepl1	Slc39a10
Hspd1	Capn10	Slc40a1
Coq10b	Mterf4	Wdr75
Sf3b1	Pask	Ikzf2
Slc39a10	Ppp1r7	Lancl1
Slc40a1	Septin2	Acadl
Wdr75	Stk25	Kansl1l
Lancl1	Bok	Rpe
Acadl	Thap4	Raph1
Kansl1l	Atg4b	Carf

Rpe	Dtymk	Ica1l
Raph1	Ing5	Wdr12
Carf	Inpp5d	Nop58
Ica1l	Atg16l1	Sumo1
Wdr12	Iqca1	Als2
Nop58	Mlph	Stradb
Sumo1	Lrrfip1	Trak2
Als2	Scly	Cflar
Stradb	Ilkap	Ndufb3
Trak2	Asb1	Clk1
Cflar	Cdh7	Ppil3
Ndufb3	Hdac4	Nif3l1
Clk1	Cln8	Orc2
Ppil3	Relch	Col5a2
Nif3l1	Tnfrsf11a	Col3a1
Orc2	Htr4	Poglut2
Col3a1	Pam	Ercc5
Poglut2	Ccdc93	Tex30
Ercc5	Actr3	Khdrbs2
Tex30	Slc35f5	Slc9a2
Khdrbs2	Lypd1	Ptp4a1
Ptp4a1	Tmem163	Slc9a4
Map4k4	Ccnt2	Il1r1
Npas2	Ubxn4	Map4k4
Pdcl3	Mcm6	Npas2
Chst10	Dars1	Pdcl3
Rev1	Rgs2	Chst10
Eif5b	Cdc73	Rev1
Mrpl30	Cfh	Eif5b
Mitd1	Tsn	Mrpl30
Cracdl	Nifk	Cracdl
Stk17b	Tfcp2l1	Stk17b
Asnsd1	Epb41l5	Asnsd1
Osgepl1	Ptpn4	Osgepl1
Ormdl1	Dbi	Ormdl1
Pms1	Steap3	Pms1
Inpp1	Nek7	Inpp1
Gls	Ddx59	Gls
Stat1	Pfkfb2	Stat1
Nabp1	Tmem9	Nabp1
Tmeff2	Csrp1	Tmeff2
Mgat4a	Gpr37l1	Mgat4a
Unc50	Srgap2	Unc50
Coa5	Arl8a	Coa5
Inpp4a	Eif2d	Inpp4a
Tmem131	Rassf5	Tmem131
Sema4c	Nucks1	Zap70
Plekhb2	Elk4	Sema4c
Imp4	Cdk18	Plekhb2
Dst	Rbbp5	Imp4
Prim2	Nfasc	Dst
Zfp142	Lrrn2	Prim2
Col19a1	Pik3c2b	Zfp142

Rhbdd1	Syt2	Col19a1
Mff	Klhl12	Rhbdd1
Fam135a	Cyb5r1	Mff
Sdhaf4	Adipor1	Fam135a
Smap1	Ppfia4	Sdhaf4
B3gat2	Atp2b4	Smap1
Ogfrl1	Tor1aip1	B3gat2
Agfg1	Xpr1	Ogfrl1
Sphkap	Stx6	Agfg1
Wnt10a	Mr1	Sphkap
Rnf25	Glul	Wnt10a
Bcs1l	Rgs16	Rnf25
Plcd4	Lamc1	Bcs1l
Cnot9	Rgl1	Plcd4
Ctdsp1	Rnf2	Cnot9
Pnkd	Coq8a	Ctdsp1
Ppm1f	Cdc42bpa	Pnkd
Igfbp5	Ahctf1	Ppm1f
Xrcc5	Tfb2m	Igfbp5
Tmem169	Kif26b	Xrcc5
Pecr	Efcab2	Tmem169
Atic	Parp1	Pecr
Fn1	Acbd3	Atic
Zfand2b	Cox20	Fn1
Abcb6	Desi2	Zfand2b
Ankzf1	Sdccag8	Abcb6
Glb1l	Capn2	Ankzf1
Stk16	Trp53bp2	Glb1l
Tuba4a	Srp9	Stk16
Dnajb2	Cnih3	Tuba4a
Ptprn	Nvl	Dnajb2
Speg	Tmem63a	Ptprn
Dnpep	Pycr2	Speg
Obsl1	Fh1	Dnpep
Stk11ip	Rgs7	Obsl1
Trip12	Cfap45	Stk11ip
Slc16a14	Tagln2	Trip12
Itm2c	Copa	Slc16a14
2810459M11Rik	Dcaf8	Itm2c
Psmd1	Vangl2	2810459M11Rik
Ncl	Uck2	Psmd1
Epha4	Tada1	Ncl
Ptma	Pou2f1	Epha4
Pde6d	Mpzl1	Ptma
Cops7b	Mpc2	Pde6d
Nppc	Dcaf6	Cops7b
Farsb	Nme7	Farsb
Ecel1	Atp1b1	Ecel1
Mrpl44	Blzf1	Mrpl44
Serpine2	Ccdc181	Serpine2
Eif4e2	Scyl3	Eif4e2
Efhd1	Kifap3	Efhd1
Ngef	Prrx1	Ngef

Ndufa10	Astn1	Ndufa10
Rnpepl1	Ralgps2	Rnpepl1
Capn10	Abl2	Capn10
Mterf4	Soat1	Mterf4
Pask	Smyd2	Pask
Ppp1r7	Ptpn14	Ppp1r7
Septin2	Kctd3	Septin2
Stk25	Esrrg	Stk25
Bok	Slc30a10	Bok
Thap4	Eprs1	Thap4
Atg4b	Bpnt1	Atg4b
Dtymk	Iars2	Dtymk
Ing5	Mark1	Ing5
Inpp5d	Lpgat1	Inpp5d
Atg16l1	Ppp2r5a	Atg16l1
Lrrfip1	Pacc1	Lrrfip1
Scly	Tatdn3	Scly
Ilkap	Angel2	Ilkap
Asb1	Plxna2	Asb1
Cdh7	Usf1	Cdh7
Hdac4	Nmt2	Hdac4
Serpinb8	Dclre1c	Serpinb8
Cln8	Cfap126	Cln8
Relch	Fam107b	Relch
Tnfrsf11a	Frmd4a	Tnfrsf11a
Htr4	Dusp12	Htr4
Gin1	Sephs1	Gin1
Pam	Atf6	Pam
Ccdc93	Phyh	Ccdc93
Actr3	Uhmk1	Actr3
Slc35f5	Uap1	Slc35f5
Lypd1	Optn	Lypd1
Tmem163	Ddr2	Tmem163
Ccnt2	Hsd17b7	Ccnt2
Ubxn4	Rgs5	Ubxn4
Mcm6	Enkur	Lct
Dars1	Aldh9a1	Mcm6
Rgs2	Mgst3	Dars1
Cdc73	Mettl13	Rgs2
Cfh	Vamp4	Cdc73
Tsn	Pigc	Cfh
Nifk	Prdx6	Tsn
Tfcp2l1	Klhl20	Nifk
Epb41l5	Dars2	Tfcp2l1
Ptpn4	Mrc1	Epb41l5
Dbi	Stam	Ptpn4
Steap3	Rabgap1l	Dbi
Nek7	Rsu1	Steap3
Cd55	Vim	Nek7
Ddx59	Pip4k2a	Cd55
Pfkfb2	Bmi1	Ddx59
Tmem9	Dnajc1	Pfkfb2
Tnnt2	Mllt10	Tmem9

Csrp1	Plxdc2	Csrp1
Gpr37l1	Nek6	Gpr37l1
Srgap2	Psmb7	Srgap2
Arl8a	Ppp6c	Arl8a
Eif2d	Golga1	Eif2d
Rassf5	Arpc5l	Rassf5
Rab29	Orc4	Rab29
Nucks1	Kif5c	Nucks1
Elk4	Lypd6b	Elk4
Cdk18	Mmadhc	Cdk18
Rbbp5	Mindy3	Rbbp5
Nfasc	Spopl	Nfasc
Lrrn2	Pfkfb3	Lrrn2
Pik3c2b	Yme1l1	Pik3c2b
Syt2	Prkcq	Syt2
Klhl12	Acbd5	Klhl12
Cyb5r1	Abi2	Cyb5r1
Adipor1	Gad2	Adipor1
Ppfia4	Zbtb43	Ppfia4
Atp2b4	Odf2	Atp2b4
Tor1aip1	Slc2a8	Tor1aip1
Xpr1	Lrsam1	Xpr1
Stx6	Niban2	Stx6
Mr1	Stxbp1	Mr1
Glul	Coq4	Glul
Rgs16	Med27	Rgs16
Lamc1	Ttf1	Lamc1
Lamc2	Ddx31	Lamc2
Rgl1	Dpm2	Rgl1
Rnf2	St6galnac6	Rnf2
Coq8a	Tsc1	Coq8a
Cdc42bpa	Eng	Cdc42bpa
Ahctf1	Gtf3c5	Ahctf1
Tfb2m	Ak1	Tfb2m
Kif26b	Slc25a25	Kif26b
Efcab2	Ptges2	Efcab2
Parp1	Ralgds	Parp1
Acbd3	Kcnj3	Acbd3
Cox20	Dnm1	Cox20
Desi2	Gpd2	Desi2
Sdccag8	Ermn	Sdccag8
Capn2	Pierce1	Capn2
Trp53bp2	Olfm1	Trp53bp2
Srp9	Acvr1c	Srp9
Cnih3	Acvr1	Cnih3
Nvl	Col5a1	Nvl
Tmem63a	Upp2	Tmem63a
Pycr2	Lamc3	Pycr2
Opn3	Fibcd1	Fh1
Fh1	Abl1	Rgs7
Rgs7	Fubp3	Dusp23
Dusp23	Tor1b	Tagln2
Cfap45	Tor1a	Copa

Tagln2	BC005624	Dcaf8
Copa	Crat	Vangl2
Dcaf8	Usp20	Uck2
Vangl2	Dolpp1	Tada1
Uck2	Ntmt1	Pou2f1
Tada1	Miga2	Mpzl1
Pou2f1	Sh3glb2	Mpc2
Mpzl1	Hspa5	Dcaf6
Mpc2	Gapvd1	Nme7
Dcaf6	Psmd5	Atp1b1
Nme7	Zeb2	Blzf1
Atp1b1	Rab14	Ccdc181
Blzf1	Gsn	Scyl3
Ccdc181	Stom	Kifap3
Scyl3	Dab2ip	Prrx1
Kifap3	Ttll11	Astn1
Prrx1	Mrrf	Ralgps2
Astn1	Grb14	Abl2
Ralgps2	Rbm18	Soat1
Abl2	Lhx6	Smyd2
Soat1	Gca	Ptpn14
Smyd2	Ndufa8	Kctd3
Kctd3	Slc4a10	Esrrg
Esrrg	Psmd14	Slc30a10
Slc30a10	Strbp	Eprs1
Eprs1	Wdr5	Bpnt1
Bpnt1	Brd3	Iars2
Iars2	Egfl7	Mark1
Mark1	Notch1	Lpgat1
Lpgat1	Sec16a	Ppp2r5a
Ppp2r5a	Inpp5e	Pacc1
Pacc1	Pmpca	Tatdn3
Tatdn3	Entr1	Angel2
Angel2	Gpsm1	Plxna2
Plxna2	Nacc2	Usf1
Usf1	Camsap1	Nmt2
Nmt2	Tmem141	Suv39h2
Dclre1c	Traf2	Dclre1c
Fam107b	Abca2	Fam107b
Frmd4a	Uap1l1	Frmd4a
Dusp12	Dpp7	Dusp12
Sephs1	Grin1	Sephs1
Atf6	Arl6ip6	Atf6
Phyh	Anapc2	Phyh
Uhmk1	Ssna1	Uhmk1
Uap1	Rbms1	Uap1
Optn	Arrdc1	Optn
Hsd17b7	Zmynd19	Ddr2
Ccdc3	Dph7	Hsd17b7
Rgs5	Marchf7	Ccdc3
Aldh9a1	Hnmt	Rgs5
Mgst3	Baz2b	Aldh9a1
Mettl13	Wdsub1	Mgst3

Vamp4	Pkp4	Mettl13
Pigc	Nup35	Vamp4
Prdx6	Dusp19	Myoc
Klhl20	Nckap1	Pigc
Nsun6	Dnajc10	Prdx6
Dars2	Itprid2	Klhl20
Mrc1	Slc25a12	Nsun6
Stam	Ube2e3	Dars2
Rabgap1l	Dync1i2	Mrc1
Trdmt1	Cwc22	Stam
Rsu1	Zfp385b	Rabgap1l
Vim	Hat1	Trdmt1
Pip4k2a	Stk39	Rsu1
Bmi1	Cers6	Vim
Dnajc1	Ssrp1	Pip4k2a
Mllt10	Timm10	Bmi1
Plxdc2	Ube2l6	Dnajc1
Nek6	Clp1	Mllt10
Psmb7	Med19	Plxdc2
Ppp6c	Fastkd1	Nek6
Golga1	Itgav	Psmb7
Arpc5l	Phospho2	Ppp6c
Orc4	Zc3h15	Golga1
Kif5c	Mtx2	Arpc5l
Lypd6b	Atf2	Orc4
Mmadhc	Ola1	Kif5c
Mindy3	Sp3	Lypd6b
Itga8	Itga6	Mmadhc
Spopl	Arl14ep	Mindy3
Pfkfb3	Slc12a6	Itga8
Yme1l1	Emc4	Spopl
Prkcq	Katnbl1	Pfkfb3
Acbd5	Nop10	Yme1l1
Abi2	Lpcat4	Prkcq
Pdss1	Ccdc34	Acbd5
Apbb1ip	Lin7c	Abi2
Gad2	Commd9	Pdss1
Zbtb43	Traf6	Apbb1ip
Odf2	Iftap	Gad2
Slc2a8	Dnajc24	Zbtb43
Lrsam1	Elp4	Odf2
Niban2	Pax6	Slc2a8
Stxbp1	Eif3m	Lrsam1
Coq4	Tcp11l1	Niban2
Med27	Cstf3	Stxbp1
Ttf1	Hipk3	Coq4
Ddx31	Fbxo3	Med27
Dpm2	Caprin1	Ttf1
St6galnac6	Nat10	Ddx31
Tsc1	Cat	Dpm2
Eng	Pamr1	St6galnac6
Gtf3c5	Trim44	Tsc1
Ak1	Api5	Eng

Slc25a25	Ttc17	Gtf3c5
Ptges2	Hsd17b12	Ak1
Ralgds	Ext2	Slc25a25
Kcnj3	Gatm	Ptges2
Dnm1	Sema6d	Ralgds
Nr4a2	Myef2	Kcnj3
Gpd2	Dut	Dnm1
Ermn	Fbn1	Nr4a2
Olfm1	Cops2	Gpd2
Acvr1c	Galk2	Ermn
Acvr1	Meis2	Olfm1
Col5a1	Cd82	Acvr1c
Upp2	Tspan18	Acvr1
Lamc3	Syt13	Col5a1
Fibcd1	Chst1	Upp2
Abl1	Pex16	Lamc3
Fubp3	Mapk8ip1	Fibcd1
Tor1b	Sord	Abl1
Tor1a	Creb3l1	Fubp3
BC005624	Eif3j1	Tor1b
Crat	Frmd5	Tor1a
Usp20	Mdk	BC005624
Dolpp1	Harbi1	Crat
Ntmt1	Atg13	Usp20
Miga2	Hypk	Dolpp1
Sh3glb2	Arhgap1	Ntmt1
Hspa5	Pdia3	Miga2
Gapvd1	Lrp4	Sh3glb2
Psmd5	Map1a	Hspa5
Zeb2	Arfgap2	Gapvd1
Rab14	Pacsin3	Psmd5
Gsn	Adal	Zeb2
Stom	Tubgcp4	Rab14
Dab2ip	Lamp5	Gsn
Ttll11	Ubr1	Stom
Mrrf	Snap25	Dab2ip
Grb14	Mkks	Ttll11
Rbm18	Jag1	Mrrf
Lhx6	Slx4ip	Grb14
Gca	Mtch2	Rbm18
Ndufa8	Cdan1	Lhx6
Ifih1	Haus2	Gca
Slc4a10	Lrrc57	Ndufa8
Psmd14	Snap23	Slc4a10
Strbp	Zfp106	Psmd14
Wdr5	Vps39	Strbp
Brd3	Ehd4	Wdr5
Egfl7	Itpka	Brd3
Notch1	Tyro3	Egfl7
Sec16a	Ubox5	Notch1
Inpp5e	Ptpra	Sec16a
Pmpca	Rtf1	Inpp5e
Entr1	Ndufaf1	Pmpca

Gpsm1	Dnaaf9	Entr1
Nacc2	Atrn	Gpsm1
Camsap1	Chac1	Nacc2
Tmem141	Dll4	Camsap1
Traf2	Spint1	Tmem141
Abca2	Gfra4	Traf2
Uap1l1	Rpusd2	Abca2
Dpp7	Adissp	Neb
Grin1	Spef1	Uap1l1
Arl6ip6	Cdc25b	Dpp7
Anapc2	Ivd	Grin1
Ssna1	Smox	Arl6ip6
Rbms1	Rassf2	Anapc2
Arrdc1	Slc23a2	Ssna1
Zmynd19	Tmem230	Rbms1
Dph7	Pcna	Arrdc1
Marchf7	Gpcpd1	Zmynd19
Hnmt	Rasgrp1	Dph7
Baz2b	Fam98b	Marchf7
Wdsub1	Chgb	Hnmt
Pkp4	Spred1	Baz2b
Nup35	Crls1	Wdsub1
Dusp19	Bmp2	Pkp4
Nckap1	Slc27a2	Galnt3
Frzb	Hdc	Nup35
Dnajc10	Gabpb1	Dusp19
Itprid2	Usp8	Nckap1
Itga4	Trpm7	Frzb
Slc25a12	Sppl2a	Dnajc10
Ube2e3	Stard7	Itprid2
Dync1i2	Fahd2a	Itga4
Cwc22	Mrps5	Slc25a12
Zfp385b	Mal	Ube2e3
Hat1	Nphp1	Dync1i2
Stk39	Ndufaf5	Cwc22
Cers6	Fbln7	Cybrd1
Ssrp1	Ttl	Zfp385b
Timm10	Polr1b	Hat1
Ube2l6	Slc20a1	Stk39
Clp1	Pdyn	Cers6
Med19	Snrpb	Ssrp1
Fastkd1	Nop56	Timm10
Itgav	Idh3b	Ube2l6
Phospho2	Cpxm1	Clp1
Zc3h15	Vps16	Med19
Mtx2	Pcsk2	Fastkd1
Atf2	Rrbp1	Itgav
Ola1	Snx5	Phospho2
Sp3	Mgme1	Zc3h15
Itga6	Kat14	Mtx2
Arl14ep	Polr3f	Atf2
Slc12a6	Rbbp9	Ola1
Emc4	Sec23b	Sp3

Katnbl1	Dtd1	Itga6
Nop10	Xrn2	Arl14ep
Lpcat4	Nkx2-2	Slc12a6
Ccdc34	Cd93	Emc4
Lin7c	Napb	Katnbl1
Commd9	Gzf1	Nop10
Traf6	Cst3	Lpcat4
Iftap	Acss1	Ccdc34
Dnajc24	Nsfl1c	Lin7c
Elp4	Snph	Commd9
Pax6	Fam110a	Traf6
Eif3m	Tbc1d20	Iftap
Tcp11l1	Rbck1	Dnajc24
Cstf3	Pdrg1	Elp4
Hipk3	Kif3b	Pax6
Fbxo3	Mapre1	Eif3m
Caprin1	Cdk5rap1	Tcp11l1
Nat10	Snta1	Cstf3
Cat	Necab3	Hipk3
Pamr1	Fam210b	Fbxo3
Trim44	Cstf1	Caprin1
Api5	Pkia	Nat10
Ttc17	Stmn2	Cat
Hsd17b12	Rtf2	Pamr1
Ext2	Tpd52	Trim44
Gatm	Pag1	Api5
Sema6d	Rae1	Ttc17
Myef2	Rab22a	Hsd17b12
Dut	Zdbf2	Ext2
Fbn1	Stx16	Gatm
Cops2	Gnas	Sema6d
Galk2	Phactr3	Myef2
Meis2	Impa1	Dut
Cd82	Fabp5	Fbn1
Tspan18	Snx16	Cops2
Syt13	Ptpn1	Galk2
Chst1	Nfatc2	Meis2
Pex16	Atp9a	Cd82
Mapk8ip1	Lrrcc1	Tspan18
Sord	Zfp64	Syt13
Creb3l1	E2f5	Chst1
Eif3j1	Dok5	Pex16
Frmd5	Car2	Mapk8ip1
Mdk	Psma7	Sord
Wdr76	Ntsr1	Creb3l1
Harbi1	Mrgbp	Eif3j1
Atg13	Col9a3	Frmd5
Hypk	Gid8	Mdk
Arhgap1	Nkain4	Wdr76
Pdia3	Arfgap1	Harbi1
Lrp4	Chrna4	Atg13
Map1a	Helz2	Hypk
Arfgap2	Stmn3	Arhgap1

Pacsin3	Zgpat	Pdia3
Adal	Zbtb46	Lrp4
Tubgcp4	Oprl1	Map1a
Lamp5	Pcmtd2	Arfgap2
Ubr1	Raly	Pacsin3
Snap25	Ahcy	Adal
Mkks	Itch	Tubgcp4
Jag1	Armc1	Lamp5
Slx4ip	Mtfr1	Ubr1
Mtch2	Map1lc3a	Snap25
Cdan1	Ggt7	Mkks
Haus2	Acss2	Jag1
Lrrc57	Gss	Slx4ip
Snap23	Mmp24	Mtch2
Zfp106	Eif6	Cdan1
Vps39	Hps3	Haus2
Ehd4	Nfs1	Lrrc57
Itpka	Rbm39	Snap23
Ltk	Epb41l1	Zfp106
Tyro3	Aar2	Vps39
Ubox5	Tbl1xr1	Ehd4
Ptpra	Ndrg3	Itpka
Rtf1	Rab5if	Ltk
Ndufaf1	Samhd1	Tyro3
Dnaaf9	Rpn2	Ubox5
Atrn	Src	Ptpra
Chac1	Ctnnbl1	Rtf1
Dll4	Tti1	Ndufaf1
Gfra4	Rprd1b	Dnaaf9
Rpusd2	Ralgapb	Atrn
Adissp	Fam83d	Chac1
Spef1	Dhx35	Dll4
Cdc25b	Skil	Gfra4
Ivd	Zmat3	Rpusd2
Smox	Pik3ca	Adissp
Adra1d	Zfp639	Spef1
Rassf2	Mfn1	Cdc25b
Slc23a2	Gnb4	Ivd
Tmem230	Actl6a	Smox
Pcna	Ndufb5	Adra1d
Gpcpd1	Pex5l	Rassf2
Rasgrp1	Ccdc39	Slc23a2
Fam98b	Ttc14	Tmem230
Chgb	Ncoa3	Pcna
Spred1	Dnajc19	Gpcpd1
Mcm8	Fxr1	Rasgrp1
Crls1	Tnik	Fam98b
Bmp2	Gm8325	Chgb
Slc27a2	Pld1	Spred1
Gabpb1	Nceh1	Mcm8
Usp8	Sec62	Fermt1
Trpm7	Dcun1d1	Crls1
Sppl2a	Mccc1	Slc27a2

Stard7	Acad9	Gabpb1
Fahd2a	Anxa5	Usp8
Mrps5	Exosc9	Trpm7
Mal	Trpc3	Sppl2a
Nphp1	Afg2a	Stard7
Bcl2l11	Slc7a11	Fahd2a
Ndufaf5	Rab33b	Mrps5
Fbln7	Cog6	Mal
Zc3h8	Ufm1	Nphp1
Ttl	Trpc4	Bcl2l11
Polr1b	Supt20	Ndufaf5
Slc20a1	Exosc8	Zc3h8
Pdyn	Mbnl1	Ttl
Tgm3	P2ry1	Polr1b
Snrpb	Dhx36	Slc20a1
Nop56	Gfm1	Pdyn
Idh3b	Mfsd1	Snrpb
Cpxm1	Schip1	Nop56
Vps16	Ift80	Idh3b
Pcsk2	Kpna4	Cpxm1
Rrbp1	Ppm1l	Vps16
Snx5	Nmd3	Pcsk2
Mgme1	Smad9	Rrbp1
Kat14	Dclk1	Snx5
Polr3f	Nbea	Mgme1
Rbbp9	Tm4sf1	Kat14
Sec23b	Wwtr1	Polr3f
Dtd1	Ppid	Rbbp9
Xrn2	Pfn2	Sec23b
Nkx2-2	Tsc22d2	Dtd1
Cd93	Serp1	Xrn2
Napb	Etfdh	Nkx2-2
Gzf1	Eif2a	Cd93
Cst3	Mme	Napb
Acss1	Slc33a1	Gzf1
Nsfl1c	Gmps	Cst3
Snph	Kcnab1	Acss1
Fam110a	Ssr3	Nsfl1c
Tbc1d20	Ccnl1	Snph
Rbck1	Serpini1	Fam110a
Tpx2	Pdcd10	Tbc1d20
Pdrg1	Dclre1b	Rbck1
Kif3b	Olfml3	Pdrg1
Mapre1	Syt6	Kif3b
Cdk5rap1	Nras	Mapre1
Snta1	Sike1	Cdk5rap1
Necab3	Tspan2	Snta1
E2f1	Vangl1	Necab3
Fam210b	Ptgfrn	E2f1
Cstf1	Gdap2	Fam210b
Pkia	Hmgcs2	Cstf1
Stmn2	Notch2	Pkia
Rtf2	Sec22b	Stmn2

Tpd52	Prpf38b	Rtf2
Pag1	Stxbp3	Tpd52
Rae1	Gpsm2	Pag1
Rab22a	Clcc1	Rae1
Zdbf2	Ampd2	Rab22a
Stx16	Gstm4	Zdbf2
Gnas	Ahcyl1	Stx16
Edn3	Slc6a17	Gnas
Phactr3	Kcnc4	Edn3
Impa1	Dram2	Phactr3
Fabp5	Ddx20	Impa1
Snx16	Slc27a3	Fabp5
Ptpn1	Ints3	Snx16
Nfatc2	Crtc2	Ptpn1
Atp9a	Jtb	Nfatc2
Lrrcc1	Tpm3	Atp9a
Zfp64	4933434E20Rik	Lrrcc1
E2f5	Hax1	Zfp64
Dok5	Il6ra	E2f5
Car2	Chrnb2	Dok5
Psma7	Adar	Car2
Ntsr1	Pmvk	Psma7
Mrgbp	Slc50a1	Ntsr1
Col9a3	Tmem144	Mrgbp
Gid8	Slc35a3	Col9a3
Nkain4	Vcam1	Gid8
Arfgap1	Extl2	Nkain4
Chrna4	Olfm3	Arfgap1
Stmn3	Col11a1	Chrna4
Zgpat	Larp7	Helz2
Zbtb46	Ndst4	Stmn3
Oprl1	Ndst3	Zgpat
Pcmtd2	Rnpc3	Zbtb46
Raly	Cyp2u1	Oprl1
Ahcy	Hadh	Pcmtd2
Itch	Lef1	Raly
Armc1	Trim2	Ahcy
Mtfr1	Plrg1	Itch
Map1lc3a	Pla2g12a	Armc1
Ggt7	Npy2r	Mtfr1
Acss2	Gucy1b1	Map1lc3a
Gss	Snx7	Ggt7
Mmp24	Gar1	Acss2
Eif6	Ppa2	Gss
Hps3	Ctso	Mmp24
Nfs1	Ints12	Eif6
Rbm39	Gstcd	Hps3
Epb41l1	Pdgfc	Nfs1
Aar2	Glrb	Rbm39
Tbl1xr1	Aimp1	Epb41l1
Ndrg3	Tbck	Aar2
Dsn1	Papss1	Tbl1xr1
Rab5if	Kcnq5	Ndrg3

Samhd1	Fubp1	Rab5if
Rpn2	Dnajb4	Samhd1
Src	Efna3	Rpn2
Ctnnbl1	Adam15	Src
Tti1	Zbtb7b	Ctnnbl1
Rprd1b	Thbs3	Tti1
Ralgapb	Gba1	Rprd1b
Dhx35	Scamp3	Ralgapb
Skil	Hcn3	Dhx35
Zmat3	Ash1l	Skil
Pik3ca	Rit1	Zmat3
Zfp639	Arhgef2	Pik3ca
Mfn1	Khdc4	Zfp639
Gnb4	Lamtor2	Mfn1
Actl6a	Lmna	Gnb4
Ndufb5	Sema4a	Actl6a
Pex5l	Pmf1	Ndufb5
Ccdc39	Gpatch4	Pex5l
Ttc14	Naxe	Ccdc39
Ncoa3	Ntrk1	Ttc14
Dnajc19	Dclk2	Ncoa3
Fxr1	Lrba	Dnajc19
Tnik	Rps3a1	Fxr1
Gm8325	Sh3d19	Tnik
Pld1	Gatb	Gm8325
Nceh1	Fbxw7	Pld1
Sec62	Fmo5	Nceh1
Dcun1d1	Chd1l	Sec62
Mccc1	Acp6	Dcun1d1
Acad9	Gpr89	Mccc1
Anxa5	Rnf115	Acad9
Exosc9	Polr3c	Anxa5
Trpc3	Nudt17	Exosc9
Afg2a	Pias3	Trpc3
Slc7a11	Pex11b	Afg2a
Rab33b	Polr3gl	Slc7a11
Cog6	Rprd2	Rab33b
Ufm1	Tars2	Cog6
Trpc4	Mettl14	Ufm1
Supt20	Bcar3	Trpc4
Exosc8	Gclm	Supt20
Mbnl1	Pip5k1a	Exosc8
P2ry1	Abcd3	Mbnl1
Dhx36	F3	P2ry1
Gfm1	Tlcd4	Dhx36
Mfsd1	Rwdd3	Gfm1
Schip1	Ptbp2	Mfsd1
Ift80	Snx27	Schip1
Kpna4	Celf3	Ift80
Ppm1l	Adh5	Kpna4
Nmd3	Mrpl9	Ppm1l
Smad9	Them4	Nmd3
Dclk1	Rap1gds1	Bche

Nbea	Tspan5	Smad9
Tm4sf1	Eif4e	Dclk1
Wwtr1	Mttp	Nbea
Ppid	Ppp3ca	Tm4sf1
Pfn2	Nfkb1	Wwtr1
Tsc22d2	Manba	Ppid
Serp1	Cisd2	Pfn2
Etfdh	Tacr3	Tsc22d2
Eif2a	Wls	Serp1
Mme	Lrrc7	Etfdh
Slc33a1	Cth	Eif2a
Gmps	Zranb2	Mme
Kcnab1	Adgrl2	Slc33a1
Ssr3	Rpf1	Gmps
Ccnl1	Ctbs	Kcnab1
Serpini1	Bcl10	Ssr3
Pdcd10	Ddah1	Ccnl1
Dclre1b	Ccn1	Shox2
Olfml3	Cryz	Serpini1
Syt6	Asph	Pdcd10
Nras	Trp53inp1	Dclre1b
Sike1	Cibar1	Olfml3
Tspan2	Pip4p2	Syt6
Vangl1	Calb1	Nras
Ptgfrn	Decr1	Sike1
Gdap2	Nbn	Tspan2
Hmgcs2	Mmp16	Ngf
Notch2	Cpne3	Vangl1
Sec22b	Tmem68	Ptgfrn
Prpf38b	Tgs1	Gdap2
Stxbp3	Rps20	Hmgcs2
Gpsm2	Ubxn2b	Notch2
Clcc1	Nsmaf	Sec22b
Ampd2	Faxc	Prpf38b
Gstm4	Coq3	Stxbp3
Ahcyl1	Pnisr	Gpsm2
Slc6a17	Sdcbp	Clcc1
Kcnc4	Tstd3	Sypl2
Dram2	Ccnc	Ampd2
Ddx20	Odf2l	Gstm4
Slc27a3	Ndufaf4	Ahcyl1
Ints3	Lmo4	Slc6a17
Rab13	Gtf2b	Kcnc4
Crtc2	Pdlim5	Dram2
Jtb	Rngtt	Ddx20
Tpm3	Ube2j1	Npr1
4933434E20Rik	Rragd	Slc27a3
Hax1	Casp8ap2	Ints3
Il6ra	Map3k7	Rab13
Chrnb2	Epha7	Crtc2
Adar	Akirin2	Jtb
Pmvk	Rars2	Tpm3
Slc50a1	Slc35a1	4933434E20Rik

Efna1	Cfap206	Hax1
Gask1b	C9orf72	Il6ra
Tmem144	Rnf20	Chrnb2
Slc35a3	Smc2	Adar
Sass6	Exosc3	Pmvk
Vcam1	Tmod1	Slc50a1
Extl2	Xpa	Efna1
Olfm3	Ncbp1	Gask1b
Col11a1	Trmo	Tmem144
Larp7	Anp32b	Slc35a3
Ndst3	Nans	Sass6
Prss12	Coro2a	Vcam1
Rnpc3	Col15a1	Extl2
Cyp2u1	Nr4a3	Olfm3
Hadh	Erp44	Col11a1
Lef1	Invs	Larp7
Trim2	Tex10	Ndst4
Plrg1	Tmeff1	Ndst3
Pla2g12a	Brinp1	Prss12
Npy2r	Fmn2	Rnpc3
Gucy1b1	Zfp618	Cyp2u1
Snx7	Slc44a5	Hadh
Gar1	Tnc	Lef1
Ppa2	Txn1	Trim2
Ctso	Astn2	Mcub
Ints12	Ugcg	Plrg1
Gstcd	Ptbp3	Pla2g12a
Pdgfc	Hsdl2	Npy2r
Glrb	Snx30	Gucy1b1
Aimp1	Zfp37	Snx7
Tbck	Wdr31	Gar1
Papss1	Alad	Tdo2
Kcnq5	Pole3	Ppa2
Fubp1	Kdm4c	Ctso
Dnajb4	Dmac1	Ints12
Efna3	Ptprd	Gstcd
Adam15	Mpdz	Pdgfc
Zbtb7b	Zdhhc21	Glrb
Thbs3	Aco1	Alpk1
Gba1	Smu1	Aimp1
Scamp3	Dnaja1	Tbck
Hcn3	Aptx	Papss1
Ash1l	Slc44a1	Kcnq5
Rit1	B4galt1	Fubp1
Arhgef2	Fktn	Dnajb4
Khdc4	Bag1	Efna3
Lamtor2	Chmp5	Adam15
Lmna	Tmem38b	Zbtb7b
Sema4a	Nfx1	Thbs3
Pmf1	Rad23b	Gba1
Gpatch4	Nol6	Scamp3
Naxe	Elp1	Hcn3
Dclk2	Ubap2	Ash1l

Lrba
Rps3a1
Sh3d19
Gatb
Fbxw7
Chd1l
Acp6
Gpr89
Rnf115
Polr3c
Nudt17
Pias3
Pex11b
Polr3gl
Rprd2
Tars2
Ecm1
Ctsk
Mettl14
Bcar3
Gclm
Pip5k1a
Abcd3
F3
Tlcd4
Rwdd3
Ptbp2
Snx27
Celf3
Adh5
Mrpl9
Them4
Rap1gds1
Tspan5
Eif4e
Mttp
Ppp3ca
Nfkb1
Manba
Cisd2
Wls
Lrrc7
Cth
Zranb2
Adgrl2
Rpf1
Ctbs
Bcl10
Ddah1
Ccn1
Cryz
Asph
Trp53inp1
Epb41l4b
Dcaf12
Ubap1
Fam219a
Spmip6
Nudt2
Cntfr
Enho
Dctn3
Vcp
Fancg
Pigo
Stoml2
Unc13b
Tesk1
Ccdc107
Tln1
Creb3
Gba2
Rgp1
Npr2
Hint2
Reck
Clta
Gne
Glipr2
Snapc3
Psip1
Sh3gl2
Plin2
Rps6
Mllt3
Usp24
Plpp3
Prkaa2
Dab1
Slc35d1
Mier1
Sgip1
Pde4b
Ak4
Dnajc6
Jak1
Cachd1
St3gal3
Dph2
B4galt2
Slc6a9
Bend5
Elavl4
Atg4c
Eps15
Ttc39a
Rit1
Arhgef2
Khdc4
Lamtor2
Lmna
Sema4a
Pmf1
Gpatch4
Naxe
Pear1
Dclk2
Lrba
Rps3a1
Sh3d19
Gatb
Fbxw7
Chd1l
Acp6
Gpr89
Rnf115
Polr3c
Nudt17
Pias3
Pex11b
Polr3gl
Rprd2
Tars2
Mettl14
Bcar3
Gclm
Pip5k1a
Abcd3
F3
Tlcd4
Rwdd3
Ptbp2
Snx27
Celf3
Adh5
Mrpl9
Them4
Rap1gds1
Tspan5
Eif4e
Mttp
Ppp3ca
Nfkb1
Manba
Cisd2
Wls
Lrrc7
Zranb2
Adgrl2

Ccne2	Dock7	Rpf1
Cibar1	Rnf11	Ctbs
Pip4p2	Osbpl9	Bcl10
Calb1	Usp1	Ddah1
Decr1	Tm2d1	Cryz
Nbn	Nfia	Asph
Mmp16	Txndc12	Trp53inp1
Cpne3	Btf3l4	Cibar1
Rmdn1	Hook1	Pip4p2
Tmem68	Ift74	Calb1
Tgs1	Plaa	Decr1
Rps20	Caap1	Nbn
Ubxn2b	Pum1	Mmp16
Nsmaf	Laptm5	Cpne3
Faxc	Cc2d1b	Rmdn1
Coq3	Pdpn	Tmem68
Pnisr	Tnfrsf1b	Tgs1
Sdcbp	Echdc2	Rps20
Tstd3	Scp2	Ubxn2b
Ccnc	Cpt2	Nsmaf
Odf2l	Czib	Faxc
Ndufaf4	Magoh	Coq3
Lmo4	Lrp8	Pnisr
Gtf2b	Ndc1	Sdcbp
Pdlim5	Lrrc42	Tstd3
Rngtt	Tmem59	Ccnc
Ube2j1	Tceanc2	Odf2l
Rragd	Cyb5rl	Ndufaf4
Casp8ap2	Mrpl37	Lmo4
Map3k7	Col9a2	Gtf2b
Epha7	Exo5	Pdlim5
Akirin2	Dyrk2	Rngtt
Rars2	Ctps1	Ube2j1
Slc35a1	Hivep3	Rragd
Smim8	Ppcs	Casp8ap2
C9orf72	Ccdc30	Map3k7
Rnf20	Ybx1	Epha7
Smc2	P3h1	Akirin2
Polr1e	Svbp	Rars2
Exosc3	Slc2a1	Slc35a1
Tmod1	Rragc	Smim8
Xpa	Mycbp	C9orf72
Ncbp1	Ndufs5	Rnf20
Trmo	Macf1	Smc2
Anp32b	Ppie	Polr1e
Nans	Trit1	Exosc3
Coro2a	Mycl	Tmod1
Nr4a3	Mfsd2a	Xpa
Erp44	Cap1	Ncbp1
Invs	Ppt1	Trmo
Tex10	Epha8	Anp32b
Tmeff1	Ephb2	Nans
Brinp1	Eloa	Coro2a

Fmn2	Pithd1	Col15a1
Zfp618	Lypla2	Nr4a3
Slc44a5	Gale	Erp44
Tnc	Hmgcl	Invs
Txn1	Fuca1	Tex10
Astn2	Pnrc2	Tmeff1
Ugcg	Srsf10	Brinp1
Ptbp3	Rnf220	Fmn2
Hsdl2	Plk3	Zfp618
Snx30	Ptch2	Slc44a5
Zfp37	Eif2b3	Tnc
Wdr31	Urod	Txn1
Alad	Toe1	Astn2
Pole3	Mmachc	Ptgr1
Kdm4c	Prdx1	Ugcg
Dmac1	Akr1a1	Ptbp3
Ptprd	Nasp	Hsdl2
Mpdz	Ipp	Snx30
Zdhhc21	Pik3r3	Zfp37
Aco1	Pomgnt1	Wdr31
Smu1	Lurap1	Alad
Dnaja1	Lrrc41	Pole3
Aptx	Nsun4	Kdm4c
Slc44a1	Mknk1	Dmac1
B4galt1	Mob3c	Ptprd
Fktn	Atpaf1	Mpdz
Bag1	Cmpk1	Zdhhc21
Chmp5	Ebna1bp2	Aco1
Tmem38b	Cfap57	Smu1
Nfx1	Aldh4a1	Dnaja1
Rad23b	Mrto4	Aptx
Nol6	Akr7a5	Slc44a1
Elp1	Slc66a1	B4galt1
Ubap2	Capzb	Fktn
Epb41l4b	Vwa5b1	Bag1
Dcaf12	Pink1	Chmp5
Ubap1	Ddost	Tmem38b
Fam219a	Kif17	Nfx1
Nudt2	Hp1bp3	Rad23b
Cntfr	Eif4g3	Nol6
Enho	Hspg2	Elp1
Dctn3	Alpl	Ubap2
Vcp	Ptpn12	Epb41l4b
Fancg	Zcchc17	Dcaf12
Pigo	Fabp3	Ubap1
Stoml2	Tinagl1	Fam219a
Unc13b	Pef1	Nudt2
Tesk1	Sema3c	Cntfr
Ccdc107	Adgrb2	Enho
Tpm2	Hpca	Dctn3
Tln1	Ptp4a2	Vcp
Creb3	Azin2	Fancg
Gba2	Khdrbs1	Pigo

Rgp1	Ak2	Stoml2
Npr2	Rnf19b	Unc13b
Hint2	Ccdc28b	Tesk1
Reck	Phc2	Ccdc107
Clta	Tmem234	Tpm2
Gne	Eif3i	Tln1
Glipr2	Zfp362	Creb3
Snapc3	Hdac1	Gba2
Psip1	Nipal3	Rgp1
Sh3gl2	Csmd2	Npr2
Plin2	Srrm1	Hint2
Rps6	Yars1	Reck
Mllt3	Sfpq	Clta
Usp24	Syf2	Gne
Plpp3	Tmem50a	Snapc3
Prkaa2	Maco1	Psip1
Dab1	AU040320	Sh3gl2
Slc35d1	Stmn1	Plin2
Mier1	Ncdn	Rps6
Sgip1	Psmb2	Mllt3
Pde4b	Extl1	Hacd4
Ak4	Zfp593	Usp24
Dnajc6	Ago3	Plpp3
Jak1	Sh3bgrl3	Prkaa2
Cachd1	Tekt2	Dab1
St3gal3	Trappc3	Slc35d1
Dph2	Gpn2	Mier1
B4galt2	Map7d1	Sgip1
Slc6a9	Gpatch3	Pde4b
Bend5	Nudc	Ak4
Elavl4	Slc9a1	Dnajc6
Itgb3bp	Tmem222	Jak1
Atg4c	Mrps15	Cachd1
Eps15	Map3k6	St3gal3
Dock7	Meaf6	Dph2
Rnf11	Wasf2	B4galt2
Osbpl9	Gnl2	Slc6a9
Usp1	Rspo1	Bend5
Tm2d1	Epha10	Elavl4
Nfia	Fam76a	Itgb3bp
Txndc12	Stx12	Atg4c
Btf3l4	Ppp1r8	Eps15
Hook1	Sema3a	Dock7
Ift74	Rpa2	Rnf11
Plaa	Eya3	Osbpl9
Caap1	Yrdc	Usp1
Pum1	Mtf1	Tm2d1
Laptm5	Sesn2	Nfia
Cc2d1b	Inpp5b	Txndc12
Pdpn	Rcc1	Btf3l4
Lrrc38	Trnau1ap	Hook1
Tnfrsf1b	Taf12	Ift74
Podn	Gmeb1	Plaa

Echdc2	Sf3a3	Caap1
Scp2	Epb41	Pum1
Cpt2	Utp11	Laptm5
Czib	Ptpru	Cc2d1b
Magoh	Mecr	Pdpn
Lrp8	Srsf4	Tnfrsf1b
Ndc1	Casp9	Echdc2
Lrrc42	Plekhm2	Scp2
Tmem59	Arhgef19	Cpt2
Tceanc2	Fbxo42	Czib
Cyb5rl	Necap2	Magoh
Mrpl37	Cdk14	Lrp8
Col9a2	Padi2	Ndc1
Exo5	Kcnab2	Lrrc42
Dyrk2	Psmc2	Tmem59
Kcnq4	Xrcc2	Tceanc2
Ctps1	Rpl22	Cyb5rl
Hivep3	Acot7	Mrpl37
Ppcs	Prkag2	Col9a2
Ccdc30	Rheb	Exo5
Ybx1	Nol9	Dyrk2
P3h1	Smarcd3	Kcnq4
Svbp	Zbtb48	Ctps1
Slc2a1	Abcf2	Hivep3
Rragc	Nub1	Ppcs
Mycbp	Vamp3	Ccdc30
Ndufs5	Per3	Ybx1
Macf1	Tmub1	P3h1
Ppie	Fastk	Svbp
Trit1	Ube4b	Slc2a1
Mycl	Pgd	Rragc
Mfsd2a	Slc4a2	Mycbp
Cap1	Park7	Ndufs5
Ppt1	Errfi1	Macf1
Epha8	Cdk5	Ppie
Ephb2	Abcb8	Trit1
Eloa	Dffa	Mycl
Pithd1	Pex14	Mfsd2a
Lypla2	Casz1	Cap1
Gale	Nos3	Ppt1
Hmgcl	H6pd	Epha8
Fuca1	Slc25a33	Ephb2
Pnrc2	Klhl7	Eloa
Srsf10	Ctnnbip1	Pithd1
Rnf220	Lzic	Lypla2
Plk3	Mtor	Gale
Eif2b3	Nmnat1	Hmgcl
Urod	Hycc1	Fuca1
Toe1	Tomm7	Pnrc2
Mmachc	Rint1	Srsf10
Prdx1	Fbxo44	Rnf220
Akr1a1	Mad2l2	Plk3
Nasp	Kmt2e	Eif2b3

Ipp	Agtrap	Urod
Pik3r3	Mthfr	Toe1
Pomgnt1	Orc5	Mmachc
Lurap1	Dnajc2	Prdx1
Lrrc41	Clcn6	Akr1a1
Nsun4	Pmpcb	Nasp
Mknk1	Mfn2	Ipp
Mob3c	Miip	Pik3r3
Atpaf1	Trp73	Pomgnt1
Cmpk1	Dffb	Lurap1
Ebna1bp2	Lrrc47	Lrrc41
Aldh4a1	Wrap73	Nsun4
Mrto4	Tprg1l	Mknk1
Akr7a5	Acap3	Mob3c
Slc66a1	Ints11	Atpaf1
Capzb	Atad3a	Cmpk1
Htr6	Ssu72	Ebna1bp2
Vwa5b1	Pex10	Aldh4a1
Pink1	Rer1	Mrto4
Ddost	Morn1	Akr7a5
Kif17	Ski	Slc66a1
Hp1bp3	Prkcz	Capzb
Eif4g3	Gabrd	Htr6
Hspg2	Plch2	Pla2g2f
Alpl	Pank4	Vwa5b1
Ptpn12	Prxl2b	Pink1
Zcchc17	Mib2	Ddost
Fabp3	Cdk11b	Kif17
Tinagl1	Nadk	Hp1bp3
Pef1	Gnb1	Eif4g3
Sema3c	Mrpl20	Hspg2
Adgrb2	Ccnl2	Alpl
Hpca	Mxra8	Ptpn12
Ptp4a2	Dvl1	Zcchc17
Azin2	Cptp	Fabp3
Khdrbs1	Sdf4	Tinagl1
Ak2	Cd38	Hcrtr1
Rnf19b	Prom1	Pef1
Ccdc28b	Kcnip4	Sema3c
Phc2	5730480H06Rik	Adgrb2
Tmem234	Adgra3	Hpca
Eif3i	Sorcs2	Ptp4a2
Zfp362	Afap1	Azin2
Hdac1	Ablim2	Khdrbs1
Nipal3	Htra3	Ak2
Csmd2	Trmt44	Rnf19b
Zbtb8a	Acox3	Ccdc28b
Srrm1	Rgs12	Phc2
Yars1	Lrpap1	Tmem234
CK137956	Htt	Eif3i
Sfpq	Add1	Zfp362
Syf2	Pcdh7	Hdac1
Tmem50a	Rnf4	Nipal3

Maco1	Nelfa	Csmd2
AU040320	Man2b2	Zbtb8a
Stmn1	Ppp2r2c	Srrm1
Ncdn	Crmp1	Yars1
Psmb2	Evc	Sfpq
Extl1	Stk32b	Syf2
Zfp593	Stx18	Tmem50a
Ago3	Nsg1	Maco1
Sh3bgrl3	Zbtb49	AU040320
Trappc3	Rab28	Stmn1
Gpn2	Rnf32	Ncdn
Map7d1	Dnajb6	Psmb2
Gpatch3	Fosl2	Extl1
Nudc	Slc4a1ap	Zfp593
Slc9a1	Eif2b4	Ago3
Tmem222	Snx17	Sh3bgrl3
Csf3r	Ppm1g	Trappc3
Mrps15	Nrbp1	Gpn2
Meaf6	Ociad1	Map7d1
Hgf	Ociad2	Gpatch3
Wasf2	Sgcb	Nudc
Gnl2	Cgref1	Slc9a1
Epha10	Khk	Tmem222
Fam76a	Agbl5	Csf3r
Stx12	Mapre3	Mrps15
Ppp1r8	Ppargc1a	Meaf6
Sema3a	Dpysl5	Wasf2
Rpa2	Dhx15	Gnl2
Eya3	Pgm2	Rspo1
Yrdc	Sepsecs	Epha10
Mtf1	Tbc1d1	Fam76a
Sesn2	Slc35f6	Stx12
Inpp5b	Anapc4	Ppp1r8
Rcc1	Klf3	Rpa2
Trnau1ap	Zcchc4	Eya3
Taf12	Fam114a1	Yrdc
Gmeb1	Sel1l3	Mtf1
Sf3a3	D5Ertd579e	Sesn2
Epb41	Rfc1	Inpp5b
Utp11	Tbc1d14	Rcc1
Ptpru	Tada2b	Trnau1ap
Mecr	Grpel1	Taf12
Srsf4	Lias	Gmeb1
Casp9	Ugdh	Sf3a3
Plekhm2	Pds5a	Epb41
Arhgef19	Ube2k	Utp11
Fbxo42	Nsun7	Ptpru
Necap2	Apbb2	Mecr
Cdk14	Guf1	Srsf4
Padi2	Gnpda2	Casp9
Kcnab2	Gabra4	Plekhm2
Psmc2	Gabrb1	Arhgef19
Xrcc2	Commd8	Fbxo42

Rpl22
Acot7
Prkag2
Rheb
Nol9
Smarcd3
Zbtb48
Abcf2
Nub1
Vamp3
Per3
Tmub1
Fastk
Ube4b
Pgd
Slc4a2
Park7
Errfi1
Cdk5
Cort
Abcb8
Dffa
Pex14
Slc2a5
Casz1
Nos3
H6pd
Slc25a33
Klhl7
Ctnnbip1
Lzic
Mtor
Nmnat1
Hycc1
Tomm7
Rint1
Fbxo44
Mad2l2
Kmt2e
Agtrap
Mthfr
Orc5
Dnajc2
Clcn6
Pmpcb
Mfn2
Miip
Dffb
Lrrc47
Wrap73
Tprg1l
Acap3
Ints11
Slc10a4
Slc30a9
Uchl1
Fip1l1
Lnx1
Chic2
Pdgfra
Srd5a3
Tmem165
Clock
Epha5
Ppat
Paics
Rest
Polr2b
Cenpc1
Pigg
Dr1
Mtf2
Dipk1a
Glmn
Brdt
Cdc7
Tgfbr3
Zfp326
Rufy3
Sparcl1
Nudt9
Hsd17b11
Klhl8
Aff1
Gpat3
Coq2
Enoph1
Hnrnpdl
Cds1
Prkg2
Crybb1
Tpst2
Tfip11
Srrd
Asphd2
Tesc
Nos1
Wsb2
Dck
Mthfd2l
Shroom3
Ccng2
Gtf2h3
Eif2b1
Ddx55
Tmed2
Necap2
Cdk14
Padi2
Kcnab2
Psmc2
Xrcc2
Rpl22
Acot7
Prkag2
Rheb
Nol9
Smarcd3
Zbtb48
Abcf2
Nub1
Vamp3
Per3
Tmub1
Fastk
Ube4b
Pgd
Slc4a2
Park7
Errfi1
Cdk5
Abcb1b
Abcb8
Dffa
Pex14
Slc2a5
Nos3
H6pd
Slc25a33
Klhl7
Ctnnbip1
Lzic
Mtor
Nmnat1
Hycc1
Tomm7
Rint1
Fbxo44
Mad2l2
Kmt2e
Agtrap
Mthfr
Orc5
Dnajc2
Clcn6
Pmpcb
Mfn2
Miip
Trp73

Atad3a	Rilpl1	Dffb
Ssu72	Cdk2ap1	Lrrc47
Pex10	Rchy1	Wrap73
Rer1	Rilpl2	Tprg1l
Morn1	Snrnp35	Acap3
Ski	Cdkl2	Ints11
Prkcz	Arl6ip4	Atad3a
Gabrd	G3bp2	Ssu72
Plch2	Pitpnm2	Pex10
Pank4	Uso1	Rer1
Prxl2b	Abcb9	Morn1
Mib2	Naaa	Ski
Cdk11b	Sdad1	Prkcz
Nadk	Slc15a4	Gabrd
Gnb1	Ajm1	Plch2
Mrpl20	Rimbp2	Pank4
Ccnl2	Rsrc2	Prxl2b
Mxra8	Scarb2	Mib2
Dvl1	Zcchc8	Cdk11b
Cptp	Stx2	Nadk
Sdf4	Ran	Gnb1
Prom1	Nipsnap2	Mrpl20
Kcnip4	Diablo	Ccnl2
5730480H06Rik	Vps33a	Mxra8
Adgra3	Mmp17	Dvl1
Sorcs2	Bcl7a	Cptp
Afap1	Sfswap	Sdf4
Ablim2	Psmd9	Cd38
Htra3	Cfap251	Prom1
Trmt44	Psph	Kcnip4
Acox3	Cct6a	5730480H06Rik
Rgs12	Rhof	Adgra3
Lrpap1	Mapkapk5	Sorcs2
Htt	Aldh2	Afap1
Add1	Acad10	Ablim2
Pcdh7	Brap	Htra3
Rnf4	Fam168a	Trmt44
Nelfa	Vps29	Acox3
Man2b2	Fam216a	Rgs12
Ppp2r2c	Gpn3	Lrpap1
Crmp1	Arpc3	Htt
Evc	Anapc7	Add1
Stx18	Atp2a2	Pcdh7
Nsg1	P2rx7	Rnf4
Zbtb49	Ift81	Nelfa
Rab28	P2rx4	Man2b2
Rnf32	Camkk2	Ppp2r2c
Dnajb6	Anapc5	Crmp1
Fosl2	Rnf34	Evc
Slc4a1ap	Kdm2b	Stk32b
Eif2b4	Ncor2	Stx18
Snx17	Dhx37	Nsg1
Ppm1g	Aacs	Zbtb49

Nrbp1	Anxa3	Rab28
Slc30a3	Mrpl1	Rnf32
Ociad1	Pgam5	Dnajb6
Ociad2	Ankle2	Fosl2
Sgcb	Golga3	Slc4a1ap
Cgref1	Ddx51	Eif2b4
Khk	Ep400	Snx17
Agbl5	Pus1	Ppm1g
Mapre3	Ulk1	Nrbp1
Ppargc1a	Prkab1	Slc30a3
Dpysl5	Cit	Ociad1
Dhx15	Rab35	Ociad2
Pgm2	Sirt4	Sgcb
Sepsecs	Pxn	Cgref1
Tbc1d1	St7	Khk
Slc35f6	Triap1	Agbl5
Anapc4	Gatc	Mapre3
Klf3	Srsf9	Ppargc1a
Zcchc4	Cabp1	Dpysl5
Fam114a1	Acads	Dhx15
Pi4k2b	Ints1	Pgm2
Sel1l3	Sppl3	Sepsecs
D5Ertd579e	Psmg3	Tbc1d1
Rfc1	Mad1l1	Slc35f6
Tbc1d14	Mrm2	Anapc4
Tada2b	2210016L21Rik	Klf3
Grpel1	Snx8	Zcchc4
Lias	Foxp2	Fam114a1
Ugdh	Tmem168	Sel1l3
Pds5a	Lfng	D5Ertd579e
Ube2k	Tmem106b	Rfc1
Apbb2	Mmab	Tbc1d14
Guf1	Radil	Tada2b
Gnpda2	Ube3b	Grpel1
Gabra4	Wipi2	Lias
Gabrb1	Actb	Ugdh
Commd8	Fscn1	Pds5a
Slc30a9	Zfp12	Ube2k
Uchl1	Usp30	Nsun7
Fip1l1	Rbm19	Apbb2
Lnx1	Plbd2	Guf1
Chic2	Ddx54	Gnpda2
Pdgfra	Rita1	Gabra4
Srd5a3	Rasal1	Gabrb1
Tmem165	Dtx1	Commd8
Clock	Rph3a	Slc30a9
Epha5	Aimp2	Uchl1
Ppat	Eif2ak1	Fip1l1
Paics	Rpl6	Lnx1
Rest	Erp29	Chic2
Polr2b	Ccz1	Pdgfra
Cenpc1	Arpc1a	Srd5a3
Pigg	Arpc1b	Tmem165

Dr1	Pdap1	Clock
Mtf2	Ptcd1	Epha5
Dipk1a	Cpsf4	Ppat
Glmn	Zkscan14	Paics
Brdt	Phf14	Rest
Cdc7	Ndufa4	Polr2b
Tgfbr3	Rnf6	Cenpc1
Zfp326	Cdk8	Pigg
Rufy3	Wasf3	Dr1
Dmp1	Glcci1	Mtf2
Sparcl1	Usp12	Dipk1a
Nudt9	Polr1d	Glmn
Hsd17b11	Pan3	Brdt
Klhl8	Flt1	Cdc7
Aff1	Pomp	Tgfbr3
Gpat3	Mtus2	Zfp326
Coq2	N4bp2l2	Rufy3
Enoph1	Hsph1	Sparcl1
Hnrnpdl	Col1a2	Nudt9
Cds1	Tspan12	Hsd17b11
Prkg2	Ing3	Klhl8
Bmp3	Fam3c	Aff1
Fgf5	Auts2	Gpat3
Crybb1	Limk1	Coq2
Tpst2	Eln	Enoph1
Tfip11	Pot1a	Hnrnpdl
Srrd	Bcl7b	Cds1
Asphd2	Wasl	Prkg2
Tesc	Cul1	Bmp3
Nos1	Ezh2	Fgf5
Rfc5	Aass	Tpst2
Wsb2	Rbm28	Tfip11
Dck	Lrwd1	Srrd
Mthfd2l	Cux1	Asphd2
Shroom3	Gcc1	Tesc
Ccng2	Ephb4	Nos1
Gtf2h3	Actl6b	Rfc5
Eif2b1	Gnb2	Wsb2
Ddx55	Gigyf1	Dck
Tmed2	Pop7	Mthfd2l
Rilpl1	Tfr2	Shroom3
Cdk2ap1	Gm20605	Ccng2
Rchy1	Agfg2	Gtf2h3
Rilpl2	Tsc22d4	Eif2b1
Snrnp35	Ppp1r35	Ddx55
Cdkl2	Mepce	Tmed2
Arl6ip4	Zkscan1	Rilpl1
G3bp2	Mcm7	Cdk2ap1
Pitpnm2	Asns	Rchy1
Uso1	Dlx6	Rilpl2
Abcb9	Dlx5	Snrnp35
Naaa	Dync1i1	Cdkl2
Sdad1	Cald1	Arl6ip4

Slc15a4	Exoc4	G3bp2
Ajm1	Plxna4	Pitpnm2
Rimbp2	Calu	Uso1
Rsrc2	Ccdc136	Abcb9
Scarb2	Ahcyl2	Naaa
Zcchc8	Klhdc10	Sdad1
Stx2	Hibadh	Slc15a4
Ran	Gars1	Ajm1
Nipsnap2	Adcyap1r1	Rimbp2
Diablo	Nt5c3	Rsrc2
Vps33a	Fkbp9	Scarb2
Mmp17	Tmem209	Zcchc8
Bcl7a	Avl9	Stx2
Sfswap	Cep41	Ran
Psmd9	Herc6	Nipsnap2
Cfap251	Abcg2	Diablo
Psph	Herc3	Vps33a
Cct6a	Tmem176b	Mmp17
Rhof	Malsu1	Bcl7a
Tmem116	Tra2a	Sfswap
Mapkapk5	Npy	Psmd9
Aldh2	Gsdme	Psph
Acad10	Osbpl3	Cct6a
Brap	Luc7l2	Rhof
Fam168a	Zc3hav1	Mapkapk5
Vps29	Trim24	Aldh2
Fam216a	Cbx3	Acad10
Gpn3	Ptn	Brap
Arpc3	Mtpn	Fam168a
Anapc7	Zyx	Vps29
Atp2a2	Fam131b	Fam216a
P2rx7	Casp2	Gpn3
Ift81	Gstk1	Arpc3
P2rx4	Ephb6	Anapc7
Camkk2	Ccdc184	Atp2a2
Anapc5	Dbpht2	P2rx7
Rnf34	Mad2l1	Ift81
Kdm2b	Ssbp1	P2rx4
Ncor2	Prdm5	Camkk2
Dhx37	Agk	Anapc5
Aacs	Mrps33	Rnf34
Anxa3	Smarcad1	Kdm2b
Mrpl1	Mkrn1	Ncor2
Pgam5	Slc37a3	Dhx37
Ankle2	Gfpt1	Aacs
Golga3	Nfu1	Anxa3
Ddx51	Pcyox1	Mrpl1
Ep400	Tgfa	Pgam5
Pus1	Add2	Ankle2
Ulk1	Dusp11	Golga3
Prkab1	Cct7	Ddx51
Cit	Zfp638	Ep400
Rab35	Fbxl14	Pus1

Sirt4	Prickle2	Gpc2
Pxn	Adamts9	Ulk1
St7	Lrig1	Prkab1
Triap1	1700003E16Rik	Cit
Gatc	Wdr54	Rab35
Srsf9	Ino80b	Sirt4
Cabp1	Wbp1	Pxn
Acads	Mogs	St7
Ints1	Mrpl53	Triap1
Sppl3	Pole4	Gatc
Psmg3	Tacr1	Srsf9
Mad1l1	Mrpl19	Cabp1
Mrm2	Aplf	Acads
2210016L21Rik	Rab43	Ints1
Snx8	Isy1	Sppl3
Foxp2	Cnbp	Psmg3
Tmem168	Copg1	Mad1l1
Lfng	Tmf1	Mrm2
Tmem106b	Hmces	2210016L21Rik
Mmab	Uba3	Snx8
Radil	Rpn1	Foxp2
Ube3b	Frmd4b	Tmem168
Wipi2	Foxp1	Lfng
Actb	Cntn3	Tmem106b
Fscn1	Chl1	Mmab
Zfp12	Ruvbl1	Radil
Ung	Sec61a1	Ube3b
Usp30	Abtb1	Wipi2
Rbm19	Plxna1	Actb
Plbd2	Chchd6	Fscn1
Ddx54	Klf15	Zfp12
Rita1	Aldh1l1	Usp30
Rasal1	Slc41a3	Rbm19
Dtx1	Nup210	Plbd2
Rph3a	Cntn6	Ddx54
Aimp2	Wnt7a	Rita1
Eif2ak1	Xpc	Rasal1
Rpl6	Tmem43	Dtx1
Erp29	Slc6a6	Rph3a
Ccz1	Grip2	Aimp2
Arpc1a	Sumf1	Eif2ak1
Arpc1b	Itpr1	Rpl6
Pdap1	Bhlhe40	Erp29
Ptcd1	Edem1	Ccz1
Cpsf4	Arl8b	Arpc1a
Zkscan14	Ret	Arpc1b
Phf14	Mlf2	Pdap1
Ndufa4	Ptms	Ptcd1
Rnf6	Plxnd1	Cpsf4
Cdk8	Lrrc23	Zkscan14
Wasf3	Tmcc1	Phf14
Glcci1	Cops7a	Ndufa4
Usp12	Rasgef1a	Rnf6

Polr1d	Bms1	Cdk8
Pan3	Zfp248	Wasf3
Flt1	Gabarapl1	Glcci1
Pomp	Rad52	Usp12
Slc46a3	Adipor2	Rasl11a
Mtus2	Erc1	Polr1d
N4bp2l2	Ccdc77	Pan3
Hsph1	Kdm5a	Flt1
Medag	Ybx3	Pomp
Col1a2	Etv6	Slc46a3
Tspan12	Lrp6	Mtus2
Ing3	Dusp16	N4bp2l2
Fam3c	Ddx47	Hsph1
Auts2	Fam234b	Medag
Limk1	Grin2b	Col1a2
Eln	Atf7ip	Tspan12
Pot1a	Wbp11	Ing3
Bcl7b	Arhgdib	Fam3c
Wasl	Rerg	Auts2
Cul1	Ptpro	Limk1
Ezh2	Strap	Eln
Aass	Lmo3	Pot1a
Rbm28	Plekha5	Bcl7b
Lrwd1	Aebp2	Wasl
Cux1	Slco1c1	Cul1
Gcc1	Slco1a4	Ezh2
Ephb4	Recql	Aass
Actl6b	Golt1b	Rbm28
Gnb2	Ldhb	Lrwd1
Gigyf1	Rad18	Cux1
Pop7	Sspn	Gcc1
Tfr2	Bhlhe41	Ephb4
Pcolce	Srgap3	Actl6b
Gm20605	Rassf8	Gnb2
Agfg2	Thumpd3	Gigyf1
Tsc22d4	Kras	Pop7
Ppp1r35	Bcat1	Tfr2
Mepce	Mtmr14	Pcolce
Zkscan1	Ogg1	Gm20605
Mcm7	Camk1	Agfg2
Asns	Etnk1	Tsc22d4
Dlx6	Ttll3	Ppp1r35
Dlx5	C2cd5	Mepce
Dync1i1	Il17rc	Zkscan1
Cald1	Cmas	Mcm7
Exoc4	St8sia1	Asns
Plxna4	Creld1	Dync1i1
Calu	Emc3	Cald1
Ccdc136	Itpr2	Exoc4
Irf5	Med21	Plxna4
Ahcyl2	Sec13	Calu
Klhdc10	Ccdc91	Ccdc136
Hibadh	Atp2b2	Irf5

Gars1	Far2	Ahcyl2
Adcyap1r1	Ergic2	Klhdc10
Nt5c3	Tmtc1	Hibadh
Fkbp9	Slc6a11	Gars1
Tmem209	Caprin2	Adcyap1r1
Avl9	Slc6a1	Nt5c3
Cep41	Dennd5b	Fkbp9
Herc6	Atg7	Tmem209
Abcg2	Vgll4	Avl9
Herc3	Tamm41	Cep41
Tmem176b	Timp4	Herc6
Malsu1	Cand2	Abcg2
Tra2a	Efcab12	Herc3
Npy	Mbd4	Tmem176b
Gsdme	Ift122	Malsu1
Osbpl3	Necap1	Gpnmb
Luc7l2	Pianp	Tra2a
Nfe2l3	Ing4	Npy
Trim24	Mrpl51	Gsdme
Cbx3	Vamp1	Osbpl3
Ptn	Ltbr	Luc7l2
Mtpn	Scnn1a	Zc3hav1
Slc13a4	Tnfrsf1a	Nfe2l3
Zyx	Cd9	Trim24
Fam131b	D6Wsu163e	Cbx3
Clcn1	Prmt8	Ptn
Casp2	Tspan9	Mtpn
Gstk1	Fkbp4	Slc13a4
Trpv6	Strn4	Zyx
Ephb6	Slc8a2	Fam131b
Ccdc184	Mzf1	Casp2
Dbpht2	Zfp606	Ephb6
Mad2l1	Zik1	Ccdc184
Ssbp1	Mark4	Dbpht2
Prdm5	Ercc2	Ssbp1
Agk	Rtn2	Prdm5
Mrps33	Vasp	Agk
Hpgds	Qpctl	Mrps33
Smarcad1	Dmpk	Hpgds
Mkrn1	Dmwd	Smarcad1
Slc37a3	Nova2	Mkrn1
Gfpt1	Pglyrp1	Slc37a3
Nfu1	Pdcd5	Gfpt1
Pcyox1	Uri1	Nfu1
Tgfa	Pop4	Anxa4
Add2	Ttyh1	Pcyox1
Dusp11	Rpl28	Tgfa
Cct7	U2af2	Add2
Pradc1	Zfp273	Dusp11
Zfp638	Cyfip1	Cct7
Fbxl14	Herc2	Pradc1
Prickle2	Nipa2	Zfp638
Lrig1	Psd3	Fbxl14

1700003E16Rik	Zfp719	Prickle2
Kbtbd8	Zdhhc13	Adamts9
Wdr54	Zfp108	Lrig1
Ino80b	Rhpn2	1700003E16Rik
Wbp1	Slc7a10	Kbtbd8
Mogs	Gas2	Wdr54
Mrpl53	Kctd15	Ino80b
Pole4	Slc17a6	Wbp1
Tacr1	Prmt3	Mogs
Mrpl19	Asb7	Mrpl53
Arhgap25	Snrpa1	Pole4
Aplf	Pcsk6	Mrpl19
Rab43	Tars3	Arhgap25
Isy1	Tjp1	Aplf
Cnbp	Entrep2	Rab43
Copg1	Apba2	Isy1
Tmf1	Mphosph10	Cnbp
Hmces	Mtmr10	Copg1
Uba3	Chrna7	Tmf1
Rpn1	Crtc3	Hmces
Frmd4b	Furin	Uba3
Foxp1	Hddc3	Rpn1
Gm20696	Unc45a	Frmd4b
Cntn3	Vps33b	Foxp1
Chl1	Iqgap1	Gm20696
Ruvbl1	Cib1	Cntn3
Sec61a1	Sema4b	Chl1
Abtb1	Idh2	Ruvbl1
Plxna1	Pex11a	Sec61a1
Chchd6	Nr2f2	Abtb1
Klf15	Synm	Plxna1
Aldh1l1	Ttc23	Chchd6
Slc41a3	Lrrc28	Klf15
Nup210	Mef2a	Aldh1l1
Cntn6	Ctsc	Slc41a3
Wnt7a	Tyrobp	Nup210
Xpc	Sipa1l3	Cntn6
Tmem43	Dpf1	Wnt7a
Slc6a6	Yif1b	Xpc
Grip2	Fam98c	Tmem43
Sumf1	Psmd8	Slc6a6
Itpr1	Ryr1	Grip2
Bhlhe40	Nfkbib	Sumf1
Edem1	Lrfn1	Itpr1
Arl8b	Pak4	Bhlhe40
Slc6a13	Psmc4	Edem1
Ret	Zfp626	Arl8b
A2m	Mfge8	Slc6a13
Mlf2	Acan	Ret
Ptms	Aen	A2m
Plxnd1	Det1	Mlf2
Lag3	Mrps11	Ptms
Tmcc1	Mrpl46	Plxnd1

Cops7a	Ccdc90b	Lag3
Rasgef1a	Tmem126b	Tmcc1
Tuba8	Tmem126a	Cops7a
Bms1	Sytl2	Rasgef1a
Zfp248	Eed	Tuba8
Clec2d	Me3	Bms1
Gabarapl1	Zfand6	Zfp248
Rad52	Fah	Clec2d
Adipor2	Sh3gl3	Gabarapl1
Erc1	Rab30	Rad52
Ccdc77	Ndufc2	Adipor2
Kdm5a	Anapc15	Wnt5b
Magohb	Coq7	Erc1
Ybx3	Arl6ip1	Ccdc77
Etv6	Smg1	Kdm5a
Lrp6	Xylt1	Magohb
Dusp16	Nucb2	Ybx3
Ddx47	Pik3c2a	Etv6
Fam234b	Ipo5	Lrp6
Grin2b	1110004F10Rik	Dusp16
Atf7ip	Pde3b	Ddx47
Wbp11	Maz	Fam234b
Arhgdib	Mvp	Grin2b
Rerg	Cdipt	Atf7ip
Ptpro	Sez6l2	Wbp11
Strap	Kctd13	Rerg
Lmo3	Stard10	Ptpro
Plekha5	Ino80e	Strap
Aebp2	Fchsd2	Lmo3
Slco1c1	Aldoa	Plekha5
Slco1a4	Ppp4c	Aebp2
Recql	Plekhb1	Slco1c1
Golt1b	Rab6a	Slco1a4
Ldhb	Mrpl48	Recql
Kcnj8	Coro1a	Golt1b
Abcc9	Sult1a1	Ldhb
Rad18	Sgf29	Abcc9
Sspn	Ppme1	Rad18
Bhlhe41	Cln3	Sspn
Srgap3	Pold3	Bhlhe41
Rassf8	Rabep2	Srgap3
Thumpd3	Pgm2l1	Rassf8
Kras	Syt3	Thumpd3
Bcat1	Sh2b1	Kras
Mtmr14	Slco2b1	Bcat1
Cpne9	Eif3c	Mtmr14
Ogg1	Myh14	Cpne9
Camk1	Spns1	Ogg1
Etnk1	Rps3	Camk1
Ttll3	Dgat2	Etnk1
C2cd5	Il4ra	Ttll3
Il17rc	Nsmce1	C2cd5
Cmas	Psma1	Il17rc

St8sia1	Thap12	Cmas
Creld1	Copb1	St8sia1
Emc3	Zkscan2	Creld1
Itpr2	Far1	Emc3
Med21	Acer3	Itpr2
Sec13	Myo7a	Med21
Ccdc91	Lcmt1	Sec13
Atp2b2	Arhgap17	Ccdc91
Far2	Disp1	Atp2b2
Ergic2	Parva	Far2
Tmtc1	Dkk3	Ergic2
Slc6a11	Pak1	Tmtc1
Caprin2	Rbbp6	Slc6a11
Slc6a1	Rusf1	Caprin2
Dennd5b	Tgfb1i1	Slc6a1
Atg7	Itgam	Dennd5b
Vgll4	Rnf141	Atg7
Tamm41	Fus	Vgll4
Timp4	Kat8	Tamm41
Cand2	Bckdk	Timp4
Efcab12	Stx4a	Cand2
Mbd4	Stx1b	Efcab12
Ift122	Fbxl19	Mbd4
Necap1	Bcl7c	Ift122
Pianp	Phkg2	Necap1
Ing4	Rnf40	Pianp
Mrpl51	Prr14	Ing4
Vamp1	Zfp747l1	Mrpl51
Ltbr	Nucb1	Vamp1
Tnfrsf1a	Bcat2	Ltbr
Cd9	Nomo1	Scnn1a
D6Wsu163e	Sergef	Tnfrsf1a
Prmt8	Lamtor1	Cd9
Tspan9	Rgs10	D6Wsu163e
Fkbp4	Tial1	Prmt8
Strn4	Bag3	Tspan9
Slc8a2	Fgfr2	Fkbp4
Mzf1	Ate1	Strn4
Zfp606	Tacc2	Slc8a2
Zik1	Ptpn5	Mzf1
Mark4	Acadsb	Zfp606
Ercc2	Dctn5	Zik1
Rtn2	Ndufab1	Mark4
Vasp	Ubfd1	Ercc2
Qpctl	Ears2	Rtn2
Dmpk	Gga2	Vasp
Dmwd	Mettl9	Qpctl
Nova2	Cdr2	Dmpk
Pdcd5	Mrpl17	Dmwd
Uri1	Polr3e	Nova2
Pop4	Arfip2	Pdcd5
Zfp939	Uqcrc2	Uri1
Ttyh1	Rrp8	Pop4

Rpl28	Vwa3a	Zfp939
U2af2	Ilk	Ttyh1
Zfp583	Tpp1	Rpl28
Zfp273	Cckbr	U2af2
Cyfip1	Crym	Zfp583
Herc2	Ldaf1	Zfp273
Nipa2	Eri2	Cyfip1
Psd3	Chst15	Herc2
Zfp719	Oat	Nipa2
Zdhhc13	Thumpd1	Psd3
Zfp108	Lhpp	Zfp719
Faap24	Fam53b	Zdhhc13
Rhpn2	Eef1akmt2	Zfp108
Slc7a10	Abraxas2	Faap24
Gas2	Trim21	Rhpn2
Kctd15	Zranb1	Slc7a10
Slc17a6	Ctbp2	Gas2
Prmt3	Rrm1	Kctd15
Asb7	Uros	Slc17a6
Snrpa1	Knop1	Prmt3
Pcsk6	Vps35l	Asb7
Tars3	Bccip	Snrpa1
Tjp1	Dhx32	Pcsk6
Entrep2	Stim1	Tars3
Apba2	Pgap2	Tjp1
Mphosph10	D7Ertd443e	Entrep2
Mtmr10	Mki67	Apba2
Chrna7	Atp6ap2	Mphosph10
Crtc3	Usp9x	Mtmr10
Blm	Cask	Chrna7
Furin	Swap70	Crtc3
Hddc3	Wee1	Blm
Unc45a	Tmem9b	Furin
Vps33b	Dennd2b	Hddc3
Iqgap1	Trim66	Unc45a
Cib1	Tub	Vps33b
Sema4b	Eif3f	Iqgap1
Idh2	Ndufb11	Cib1
Pex11a	Rbm10	Sema4b
Nr2f2	Cdk16	Idh2
Synm	Usp11	Pex11a
Lrrc28	Glrx3	Nr2f2
Mef2a	LTO1	Synm
Ctsc	Ano1	Lrrc28
Tyrobp	Fadd	Mef2a
Sipa1l3	Cttn	Ctsc
Dpf1	Nadsyn1	Tyrobp
Yif1b	Dock11	Sipa1l3
Fam98c	Cul4b	Dpf1
Psmd8	Smarca1	Yif1b
Ryr1	Rab33a	Fam98c
Nfkbib	Slc25a14	Psmd8
Fbxo17	Enox2	Ryr1

Lrfn1	Igsf1	Nfkbib
Pak4	Stk26	Lrfn1
Psmc4	Gpc4	Pak4
Zfp626	Slc9a9	Psmc4
Mfge8	Arhgef6	Zfp626
Hapln3	Rbmx	Mfge8
Aen	Fgf13	Hapln3
Det1	Ccdc22	Acan
Mrps11	Syp	Aen
Mrpl46	Gpkow	Det1
Ccdc90b	Praf2	Mrps11
Tmem126b	Ccdc120	Mrpl46
Tmem126a	Gripap1	Ccdc90b
Sytl2	Otud5	Tmem126b
Eed	Pim2	Tmem126a
Me3	Slc35a2	Sytl2
Zfand6	Pqbp1	Eed
Fah	Timm17b	Me3
Sh3gl3	Hdac6	Zfand6
Rab30	Wdr13	Fah
Ndufc2	Rbm3	Sh3gl3
Anapc15	Ebp	Rab30
Coq7	Porcn	Ndufc2
Arl6ip1	Ftsj1	Anapc15
Smg1	Rpgr	Coq7
Xylt1	Dynlt3	Arl6ip1
Nucb2	Aff2	Smg1
Pik3c2a	Vbp1	Xylt1
Ipo5	Fundc2	Nucb2
1110004F10Rik	Brcc3	Pik3c2a
Pde3b	Rab39b	Ipo5
Maz	Msn	1110004F10Rik
Mvp	Heph	Pde3b
Cdipt	Gpr165	Maz
Sez6l2	Ophn1	Mvp
Kctd13	Stard8	Cdipt
Stard10	Efnb1	Sez6l2
Ino80e	Igbp1	Kctd13
Fchsd2	Magee2	Stard10
Aldoa	Pbdc1	Ino80e
Ppp4c	Magee1	Fchsd2
Plekhb1	Atrx	Aldoa
Rab6a	Cox7b	Ppp4c
Mrpl48	Magt1	Plekhb1
Coro1a	Itm2a	Rab6a
Sgf29	Chmp1b2	Mrpl48
Ppme1	Sh3bgrl	Coro1a
Cln3	Sytl4	Sult1a1
Nfatc2ip	Cstf2	Sgf29
Lipt2	Gla	Ppme1
Pold3	Col4a5	Cln3
Rabep2	Acsl4	Nfatc2ip
Pgm2l1	Pak3	Lipt2

Syt3	Dcx	Pold3
Sh2b1	Lrch2	Rabep2
Slco2b1	Cdkl5	Pgm2l1
Eif3c	Phka2	Atp2a1
Myh14	Slc7a3	Syt3
Spns1	Pdha1	Sh2b1
Rps3	Nlgn3	Slco2b1
Dgat2	Rps6ka3	Eif3c
Il4ra	Zmym3	Myh14
Nsmce1	Nono	Spns1
Psma1	Taf1	Rps3
Kdm8	Rps4x	Dgat2
Thap12	Chic1	Il4ra
Copb1	Flna	Nsmce1
Zkscan2	Abcb7	Psma1
Far1	Mtm1	Kdm8
Acer3	Gabre	Thap12
Lcmt1	Gpm6b	Copb1
Arhgap17	Gabra3	Zkscan2
Disp1	Gabrq	Far1
Parva	Cetn2	Acer3
Dkk3	Nsdhl	Myo7a
Pak1	Hccs	Lcmt1
Rbbp6	Rbbp7	Arhgap17
Rusf1	Arhgap6	Disp1
Tgfb1i1	Syap1	Parva
Itgam	Msl3	Dkk3
Rnf141	Ctps2	Pak1
Dkkl1	Zfp275	Rbbp6
Fus	Ap1s2	Rusf1
Tead2	Zrsr2	Tgfb1i1
Cd37	Haus7	Itgam
Kat8	Zfp92	Rnf141
Bckdk	Bgn	Fus
Stx4a	Atp2b3	Tead2
Stx1b	Abcd1	Cd37
Fbxl19	Pir	Kat8
Bcl7c	Piga	Bckdk
Phkg2	Plxnb3	Stx4a
Rnf40	Hcfc1	Stx1b
Prr14	Naa10	Fbxl19
Zfp747l1	L1cam	Bcl7c
Nucb1	Irak1	Phkg2
Bcat2	Mecp2	Rnf40
Nomo1	Plxna3	Prr14
Sergef	Fam3a	Zfp747l1
Lamtor1	G6pdx	Nucb1
Rgs10	Mpp1	Bcat2
Tial1	Dkc1	Nomo1
Bag3	Tceal6	Sergef
Fgfr2	Morf4l2	Lamtor1
Ate1	Plp1	Rgs10
Tacc2	Zcchc18	Tial1

Ptpn5	Psmd10	Bag3
Acadsb	Tsc22d3	Fgfr2
Cpxm2	Prps1	Ate1
Dctn5	Morc4	Tacc2
Ndufab1	Rnf128	Ptpn5
Ubfd1	Atp11a	Acadsb
Ears2	Mcf2l	Cpxm2
Gga2	Cul4a	Dctn5
Mettl9	Lamp1	Ndufab1
Cdr2	Gas6	Ubfd1
Mrpl17	Rasa3	Ears2
Polr3e	Coprs	Gga2
Arfip2	Agpat5	Mettl9
Uqcrc2	Vps36	Cdr2
Rrp8	Slc25a15	Mrpl17
Vwa3a	Erlin2	Polr3e
Ilk	Plpbp	Arfip2
Tpp1	Adgra2	Dnhd1
Cckbr	Brf2	Uqcrc2
Crym	Col4a1	Rrp8
Ldaf1	Col4a2	Vwa3a
Lyrm1	Naxd	Ilk
Eri2	Ankrd10	Tpp1
Chst15	Arhgef7	Cckbr
Oat	Leprotl1	Crym
Thumpd1	Dctn6	Ldaf1
Lhpp	Gpm6a	Lyrm1
Fam53b	Aga	Eri2
Eef1akmt2	Dlc1	Chst15
Abraxas2	Eri1	Oat
Trim21	Tnks	Thumpd1
Zranb1	Dusp4	Lhpp
Ctbp2	Saraf	Fam53b
Rrm1	Mrps31	Eef1akmt2
Uros	Smim19	Abraxas2
Knop1	Polb	Trim21
Vps35l	Ikbkb	Zranb1
Bccip	Plat	Ctbp2
Dhx32	Ap3m2	Rrm1
Stim1	Kat6a	Uros
Pgap2	Ank1	Knop1
Atp6ap2	Gpat4	Vps35l
Usp9x	Gins4	Bccip
Cask	Sfrp1	Dhx32
Swap70	Adam9	Stim1
Wee1	Tm2d2	Pgap2
Tmem9b	Plekha2	D7Ertd443e
Dennd2b	Slit2	Atp6ap2
Trim66	Tenm3	Usp9x
Tub	Dctd	Cask
Eif3f	Wwc2	Swap70
Ndufb11	Fgfr1	Wee1
Rbm10	Rwdd4a	Tmem9b

Cdk16	Plpp5	Dennd2b
Usp11	Ash2l	Trim66
Glrx3	Tti2	Tub
LTO1	Mak16	Eif3f
Fadd	Wrn	Ndufb11
Cttn	Gsr	Rbm10
Nadsyn1	Gtf2e2	Cdk16
Dock11	Frg1	Usp11
Cul4b	Asah1	Glrx3
Smarca1	Pcm1	LTO1
Rab33a	Slc7a2	Ano1
Slc25a14	Vps37a	Fadd
Rbmx2	Cnot7	Cttn
Enox2	Msmo1	Nadsyn1
Stk26	Klhl2	Dock11
Gpc4	Galnt7	Cul4b
Slc9a9	Sap30	Smarca1
Arhgef6	Scrg1	Rab33a
Rbmx	Tmem184c	Slc25a14
Fgf13	Nr3c2	Rbmx2
Mcf2	Sin3b	Enox2
Ccdc22	Sorbs2	Igsf1
Syp	Irf2	Stk26
Plp2	Casp3	Gpc4
Gpkow	Cfap97	Slc9a9
Praf2	Slc25a4	Arhgef6
Ccdc120	Ufsp2	Rbmx
Gripap1	Tlr3	Fgf13
Otud5	Cbr4	Mcf2
Pim2	Sh3rf1	Ccdc22
Slc35a2	Nek1	Syp
Pqbp1	Mfap3l	Gpkow
Timm17b	N4bp1	Praf2
Hdac6	Cbln1	Ccdc120
Wdr13	Heatr3	Gripap1
Rbm3	Adcy7	Otud5
Ebp	Brd7	Pim2
Porcn	Nkd1	Slc35a2
Slc38a5	Sall1	Pqbp1
Ftsj1	Rbl2	Timm17b
Rpgr	Aktip	Hdac6
Dynlt3	Eif2ak3	Wdr13
Aff2	Setd6	Rbm3
Vbp1	Got2	Ebp
Fundc2	Cdh11	Porcn
Mtcp1	Smad1	Slc38a5
Brcc3	Lsm6	Ftsj1
Rab39b	Slc10a7	Rpgr
Msn	Tnpo2	Dynlt3
Gpr165	Vps35	Aff2
Ophn1	Orc6	Vbp1
Stard8	Gpt2	Fundc2
Efnb1	Dnaja2	Mtcp1

Igbp1	Itfg1	Brcc3
Magee2	Rfx1	Rab39b
Pbdc1	Tecr	Msn
Magee1	Tbc1d9	Heph
Atrx	Zfp330	Gpr165
Cox7b	Gab1	Ophn1
Magt1	Smarca5	Stard8
Itm2a	Txnl4b	Efnb1
Chmp1b2	Zfp821	Igbp1
Sh3bgrl	Ist1	Magee2
Cstf2	Dhodh	Pbdc1
Gla	Ap1g1	Magee1
Col4a5	Phlpp2	Atrx
Acsl4	Mmp2	Cox7b
Chrdl1	Gnao1	Magt1
Pak3	St3gal2	Itm2a
Dcx	Il34	Chmp1b2
Lrch2	Amfr	Hmgn5
Cdkl5	Cog4	Sh3bgrl
Phka2	Nudt21	Sytl4
Slc7a3	Bbs2	Cstf2
Pdha1	Cdyl2	Gla
Nlgn3	Mt3	Col4a5
Rps6ka3	Mt2	Acsl4
Zmym3	Mt1	Chrdl1
Nono	Nudt7	Pak3
Taf1	Herpud1	Dcx
Rps4x	Cntnap4	Lrch2
Dmrtc1a	Psme3ip1	Cdkl5
Chic1	Pllp	Phka2
Flna	Arl2bp	Slc7a3
Abcb7	Cx3cl1	Pdha1
Mtm1	Ciapin1	Nlgn3
Gpm6b	Coq9	Rps6ka3
Gabra3	Polr2c	Zmym3
Cetn2	Adgrg1	Nono
Nsdhl	Drc7	Taf1
Hccs	Katnb1	Rps4x
Rbbp7	Kifc3	Dmrtc1a
Arhgap6	Cngb1	Chic1
Syap1	Mmp15	Flna
Msl3	Tmem38a	Abcb7
Ctps2	Usb1	Mtm1
Zfp275	Cfap20	Gpm6b
Ap1s2	Tpm4	Gabra3
Zrsr2	Jak3	Cetn2
Haus7	Pgls	Nsdhl
Zfp92	Slc27a1	Hccs
Bgn	Map1lc3b	Rbbp7
Atp2b3	Mvb12a	Arhgap6
Abcd1	Mthfsd	Syap1
Pir	Cox4i1	Msl3
Vegfd	Emc8	Ctps2

Piga	Babam1	Zfp275
Plxnb3	Gse1	Ap1s2
Hcfc1	Zdhhc7	Zrsr2
Renbp	6430548M08Rik	Haus7
Naa10	Usp10	Car5b
Arhgap4	Cotl1	Zfp92
L1cam	Klhl36	Bgn
Irak1	Taf1c	Atp2b3
Mecp2	Mast3	Abcd1
Plxna3	Pik3r2	Pir
Fam3a	Mbtps1	Piga
G6pdx	Necab2	Plxnb3
Mpp1	Hsbp1	Hcfc1
Dkc1	Rab3a	Renbp
Tceal6	Cdh13	Naa10
Morf4l2	Mphosph6	Arhgap4
Plp1	1700030J22Rik	L1cam
Zcchc18	Lsm4	Irak1
Psmd10	Ntpcr	Mecp2
Tsc22d3	Mau2	Plxna3
Prps1	Pbx4	Fam3a
Rbm41	Atp13a1	G6pdx
Morc4	Ints10	Mpp1
Rnf128	Dctn1	Dkc1
Atp11a	Pgr	Tceal6
Mcf2l	Cdh5	Morf4l2
Cul4a	Bean1	Plp1
Lamp1	Cmtm3	Zcchc18
Gas6	Nae1	Psmd10
Rasa3	Ciao2b	Tsc22d3
Coprs	Rrad	Prps1
Agpat5	Cbfb	Rbm41
Nek3	Phaf1	Morc4
Vps36	Psmb10	Rnf128
Thsd1	Dus2	Atp11a
Slc25a15	Nfatc3	Mcf2l
Erlin2	Pla2g15	Cul4a
Plpbp	Slc7a6	Lamp1
Adgra2	Smpd3	Gas6
Brf2	Zfp90	Rasa3
Eif4ebp1	Vps4a	Coprs
Col4a1	Cog8	Agpat5
Col4a2	Nip7	Nek3
Naxd	Mtmr2	Vps36
Ankrd10	Terf2	Thsd1
Arhgef7	Cep57	Slc25a15
Leprotl1	Cyb5b	Erlin2
Dctn6	Maml2	Plpbp
Gpm6a	Mre11a	Adgra2
Vegfc	Wwp2	Brf2
Aga	Ankrd49	Col4a1
Dlc1	Gpr83	Col4a2
Eri1	Panx1	Naxd

Tnks	Med17	Ankrd10
Dusp4	Vstm5	Arhgef7
Saraf	4931406C07Rik	Leprotl1
Mrps31	Taf1d	Dctn6
Smim19	Kars1	Gpm6a
Polb	Adat1	Aga
Ikbkb	Gabarapl2	Dlc1
Plat	Tmem231	Eri1
Ap3m2	Tmem170	Tnks
Kat6a	Cfdp1	Dusp4
Ank1	Bcar1	Saraf
Gpat4	Ldhd	Mrps31
Gins4	Wdr59	Smim19
Sfrp1	Aars1	Polb
Adam9	Bmper	Ikbkb
Tm2d2	Afg3l1	Plat
Plekha2	Acad8	Ap3m2
Slit2	Dbndd1	Kat6a
Tenm3	Ccsap	Ank1
Dctd	Abcb10	Gpat4
Wwc2	Urb2	Gins4
Fgfr1	Cog2	Sfrp1
Rwdd4a	Agt	Adam9
Plpp5	Arv1	Tm2d2
Ash2l	2310022B05Rik	Plekha2
Tti2	2810004N23Rik	Slit2
Mak16	Gnpat	Tenm3
Wrn	Sprtn	Dctd
Gsr	Egln1	Wwc2
Gtf2e2	Vps26b	Fgfr1
Frg1	Jam3	Rwdd4a
Asah1	Snx19	Plpp5
Pcm1	Aplp2	Ash2l
Slc7a2	Trpc6	Tti2
Vps37a	Dcun1d5	Mak16
Cnot7	Sesn3	Wrn
Msmo1	Usp2	Gsr
Klhl2	Thy1	Gtf2e2
Galnt7	Nectin1	Frg1
Sap30	Oaf	Asah1
Scrg1	Grik4	Pcm1
Ednra	Sc5d	Slc7a2
Tmem184c	Ubash3b	Vps37a
Nr3c2	Clmp	Cnot7
Sin3b	Rexo2	Msmo1
Sorbs2	Cul5	Klhl2
Irf2	Kcnj5	Galnt7
Casp3	Ets1	Sap30
Cfap97	Kirrel3	Scrg1
Slc25a4	St3gal4	Ednra
Ufsp2	Dcps	Tmem184c
Tlr3	Srpra	Nr3c2
Cbr4	Rpusd4	Sin3b

Sh3rf1	Abhd12	Sorbs2
Nek1	Acat1	Irf2
Mfap3l	Rdx	Casp3
N4bp1	Fdx1	Cfap97
Cbln1	Ppp2r1b	Slc25a4
Heatr3	Alg9	Ufsp2
Adcy7	Cryab	Tlr3
Brd7	Dixdc1	Cbr4
Nkd1	Pts	Sh3rf1
Sall1	Cadm1	Nek1
Rbl2	Bud13	Mfap3l
Aktip	Zpr1	N4bp1
Eif2ak3	Tagln	Cbln1
Setd6	Bace1	Heatr3
Got2	Dscaml1	Adcy7
Cdh11	Il10ra	Brd7
Smad1	Arcn1	Nkd1
Lsm6	Ddx6	Sall1
Slc10a7	Ddx25	Rbl2
Tnpo2	Pus3	Aktip
Vps35	Nlrx1	Eif2ak3
Orc6	Trappc4	Gins3
Mylk3	Slc37a4	Setd6
Gpt2	Hyou1	Got2
Dnaja2	Stt3a	Cdh11
Itfg1	Fez1	Smad1
Rfx1	Hinfp	Lsm6
Tecr	C2cd2l	Slc10a7
Tbc1d9	Tmem218	Tnpo2
Zfp330	Dpagt1	Vps35
Gab1	Robo4	Orc6
Smarca5	Hmbs	Gpt2
Txnl4b	Vps11	Dnaja2
Zfp821	Abcg4	Itfg1
Ist1	Mcam	Rfx1
Dhodh	Pin1	Tecr
Ap1g1	Olfm2	Tbc1d9
Phlpp2	Icam5	Zfp330
Mmp2	Tyk2	Gab1
Gnao1	Pde4a	Smarca5
St3gal2	Ilf3	Txnl4b
Il34	Tmed1	Zfp821
Amfr	Scg3	Ist1
Cog4	Yipf2	Dhodh
Nudt21	Lysmd2	Ap1g1
Bbs2	Carm1	Phlpp2
Cdyl2	Tmod2	Mmp2
Mt3	Smarca4	Gnao1
Mt2	Gnb5	St3gal2
Mt1	Ldlr	Il34
Herpud1	Kank2	Amfr
Cntnap4	Dock6	Cog4
Psme3ip1	Polr2m	Nudt21

Pllp	Rab27a	Bbs2
Arl2bp	Sltm	Cdyl2
Cx3cl1	Rsl24d1	Mt3
Ciapin1	Nedd4	Mt2
Coq9	Rnf111	Mt1
Polr2c	Myo1e	Herpud1
Adgrg1	Mns1	Cntnap4
Katnb1	Fam81a	Psme3ip1
Kifc3	Tcf12	Pllp
Mmp15	Anxa2	Arl2bp
Tmem38a	Cgnl1	Cx3cl1
Usb1	Ice2	Ciapin1
Cfap20	Rora	Coq9
Tpm4	Rp9	Polr2c
Jak3	Fem1b	Adgrg1
Pgls	Cln6	Katnb1
Slc27a1	Anp32a	Kifc3
Map1lc3b	Irak1bp1	Mmp15
Mvb12a	Glce	Tmem38a
Mthfsd	Phip	Usb1
Cox4i1	Lca5	Cfap20
Emc8	Drd2	Tpm4
Babam1	Sh3bgrl2	Jak3
Gse1	Elovl4	Pgls
Zdhhc7	Bckdhb	Slc27a1
6430548M08Rik	Zw10	Map1lc3b
Usp10	Tent5a	Mvb12a
Cotl1	Usp28	Mthfsd
Klhl36	Idh3a	Cox4i1
Taf1c	Tle3	Emc8
Mast3	Acsbg1	Babam1
Pik3r2	Dnaja4	Gse1
Mbtps1	Imp3	Zdhhc7
Necab2	Thsd4	6430548M08Rik
Ifi30	Ptpn9	Crispld2
Hsbp1	Crabp1	Usp10
Rab3a	Ireb2	Cotl1
Cdh13	Pkm	Klhl36
Mphosph6	Man2c1	Taf1c
1700030J22Rik	Celf6	Mast3
Lsm4	Neil1	Pik3r2
Ntpcr	Commd4	Mbtps1
Map3k21	1700017B05Rik	Necab2
Mau2	Psma4	Hsbp1
Pbx4	Fam219b	Rab3a
Atp13a1	Mpi	Cdh13
Ints10	Ube2q2	Mphosph6
Dctn1	Ulk3	1700030J22Rik
Pgr	Fbxo22	Lsm4
Cdh5	Csk	Ntpcr
Bean1	Tmem266	Mau2
Cmtm3	Etfa	Pbx4
Nae1	Clk3	Atp13a1

Ciao2b	Rcn2	Ints10
Rrad	Tspan3	Dctn1
Car7	Tmem30a	Pgr
Cbfb	Hmg20a	Cdh5
Tradd	Cox7a2	Bean1
Phaf1	Col12a1	Cmtm3
Psmb10	Stoml1	Nae1
Dus2	Loxl1	Ciao2b
Nfatc3	Nptn	Car7
Pla2g15	Hcn4	Cbfb
Slc7a6	Neo1	Tradd
Smpd3	Mto1	Phaf1
Zfp90	Gsta4	Psmb10
Vps4a	Elovl5	Dus2
Cog8	Gclc	Nfatc3
Nip7	Lrrc1	Pla2g15
Mtmr2	Tmed3	Slc7a6
Terf2	Mlip	Smpd3
Cep57	Rasgrf1	Zfp90
Cyb5b	Ctsh	Vps4a
Maml2	Tpm1	Cog8
Mre11a	Zic1	Nip7
Wwp2	Lactb	Mtmr2
Ankrd49	Car12	Terf2
Gpr83	Plod2	Cep57
Panx1	Aph1b	Cyb5b
Med17	Usp3	Maml2
Vstm5	Ciao2a	Mre11a
4931406C07Rik	Snx1	Wwp2
Taf1d	Ppib	Ankrd49
Kars1	Csnk1g1	Gpr83
Adat1	Trip4	Panx1
Gabarapl2	Spg21	Med17
Tmem231	Dpp8	Vstm5
Tmem170	Dis3l	4931406C07Rik
Cfdp1	Tipin	Taf1d
Bcar1	Snapc5	Kars1
Ldhd	Rpl4	Adat1
Wdr59	Smad3	Gabarapl2
Aars1	Pias1	Tmem231
Bmper	U2surp	Tmem170
Afg3l1	Atr	Cfdp1
Acad8	Xrn1	Bcar1
Dbndd1	Tfdp2	Ldhd
Ccsap	Atp1b3	Wdr59
Acta1	Rasa2	Aars1
Abcb10	Ube3d	Bmper
Urb2	Rwdd2a	Afg3l1
Cog2	Me1	Acad8
Agt	Nt5e	Dbndd1
Arv1	Snx14	Ccsap
2310022B05Rik	Syncrip	Abcb10
2810004N23Rik	Zfp949	Urb2

Gnpat	Crtap	Cog2
Sprtn	Cmtm6	Agt
Egln1	Dync1li1	Arv1
Vps26b	Stt3b	2310022B05Rik
Jam3	Tgfbr2	2810004N23Rik
Snx19	Slc25a36	Gnpat
Adamts8	Clstn2	Sprtn
Aplp2	Copb2	Egln1
Trpc6	Mrps22	Vps26b
Birc3	Pik3cb	Jam3
Dcun1d5	Faim	Snx19
Sesn3	Armc8	Adamts8
Usp2	Dbr1	Aplp2
Thy1	Mras	Trpc6
Nectin1	Nck1	Dcun1d5
Oaf	Cdc25a	Pdgfd
Grik4	Nme6	Sesn3
Sc5d	Map4	Usp2
Ubash3b	Dhx30	Thy1
Clmp	Smarcc1	Nectin1
Rexo2	Cspg5	Oaf
Cul5	Scap	Grik4
Barx2	Kif9	Sc5d
Ets1	Pth1r	Ubash3b
Kirrel3	Lrrfip2	Clmp
St3gal4	Mlh1	Rexo2
Dcps	Dclk3	Cul5
Srpra	Trib1	Ets1
Rpusd4	Stac	Kirrel3
Abhd12	Arpp21	St3gal4
Acat1	Pdcd6ip	Dcps
Rdx	Fbxl2	Srpra
Fdx1	Scn5a	Rpusd4
Ppp2r1b	Wdr48	Abhd12
Alg9	Gorasp1	Acat1
Cryab	Ttc21a	Rdx
Dixdc1	Csrnp1	Fdx1
Pts	Mobp	Ppp2r1b
Cadm1	Rpsa	Alg9
Bud13	Slc25a38	Cryab
Zpr1	Hhatl	Dixdc1
Tagln	Nktr	Pts
Bace1	Ss18l2	Cadm1
Dscaml1	Pccb	Bud13
Il10ra	Amotl2	Zpr1
Arcn1	Cck	Tagln
Ddx6	Cep63	Bace1
Ddx25	Trak1	Dscaml1
Pus3	Ephb1	Il10ra
Nlrx1	Abhd5	Arcn1
Trappc4	Ryk	Ddx6
Slc37a4	Rab6b	Ddx25
Hyou1	1110059G10Rik	Pus3

Stt3a	Srprb	Nlrx1
Fez1	Trf	Trappc4
Hinfp	Topbp1	Slc37a4
C2cd2l	Uba5	Hyou1
Tmem218	Nphp3	Stt3a
Dpagt1	Dnajc13	Fez1
Robo4	Gnai2	Hinfp
Hmbs	Mrpl3	C2cd2l
Vps11	Cpne4	Tmem218
Robo3	Nudt16	Dpagt1
Abcg4	Aste1	Robo4
Mcam	Atp2c1	Hmbs
Pin1	Pik3r4	Vps11
Olfm2	Manf	Robo3
Icam5	Cish	Abcg4
Tyk2	Hemk1	Mcam
Pde4a	Rbm5	Pin1
Ilf3	Rbm6	Olfm2
Tmed1	Mon1a	Icam5
Scg3	Traip	Tyk2
Yipf2	Bsn	Pde4a
Lysmd2	Apeh	Ilf3
Carm1	Ip6k1	Tmed1
Tmod2	Cdhr4	Scg3
Smarca4	Nckipsd	Yipf2
Gnb5	Ip6k2	Lysmd2
Ldlr	Prkar2a	Carm1
Kank2	Slc25a20	Tmod2
Dock6	Qars1	Smarca4
Polr2m	Nicn1	Gnb5
Rab27a	Amt	Ldlr
Sltm	Klhdc8b	Kank2
Rsl24d1	Usp4	Dock6
Nedd4	Nt5m	Polr2m
Rnf111	Srek1	Rab27a
Myo1e	Eml4	Sltm
Mns1	Thsd7a	Rsl24d1
Fam81a	Flcn	Nedd4
Tcf12	Atxn2l	Rnf111
Anxa2	Chsy1	Myo1e
Cgnl1	Gpr19	Mns1
Ice2	Pygm	Fam81a
Rora	Colgalt2	Tcf12
Rp9	Crebl2	Anxa2
Fem1b	Marchf3	Cgnl1
Cln6	Entrep3	Ice2
Anp32a	1700025G04Rik	Rora
Irak1bp1	Pon2	Rp9
Glce	Cd59a	Itga11
Phip	6820408C15Rik	Fem1b
Lca5	Malt1	Cln6
Drd2	Lmo2	Anp32a
Sh3bgrl2	Kank1	Irak1bp1

Elovl4	Exd2	Glce
Bckdhb	Resf1	Phip
Zw10	Syde1	Lca5
Tent5a	Abtb2	Drd2
Usp28	Mier3	Sh3bgrl2
Htr3a	Snx33	Elovl4
Idh3a	Ablim3	Bckdhb
Tle3	Inppl1	Zw10
Acsbg1	Ccdc88a	Tent5a
Dnaja4	Tpcn1	Usp28
Imp3	Katnip	Htr3a
Thsd4	Heyl	Idh3a
Ptpn9	Gpbp1	Tle3
Ireb2	Slc8b1	Acsbg1
Pkm	Bet1	Dnaja4
Man2c1	Ilvbl	Imp3
Celf6	Gng11	Thsd4
Neil1	Chrm1	Ptpn9
Commd4	Gtf3c1	Ireb2
1700017B05Rik	Cntrob	Pkm
Psma4	Alas1	Man2c1
Fam219b	Pdxk	Celf6
Mpi	Lama1	Neil1
Ube2q2	Srxn1	Commd4
Ulk3	Cdv3	1700017B05Rik
Fbxo22	Arap1	Psma4
Csk	Igdcc4	Fam219b
Tmem266	Ank2	Mpi
Etfa	Ppp1r9a	Ube2q2
Clk3	Pwp2	Ulk3
Rcn2	Trpc1	Fbxo22
Tspan3	Spring1	Csk
Tmem30a	Prr5l	Tmem266
Hmg20a	Abcc10	Etfa
Cox7a2	Zswim6	Clk3
Col12a1	Abcc4	Rcn2
Stoml1	Rnft2	Pstpip1
Loxl1	Ugt8a	Tspan3
Nptn	Pkd1	Stra6
Hcn4	Fbxw8	Tmem30a
Neo1	Psmf1	Hmg20a
Mto1	Smap2	Cox7a2
Gsta4	Cyb5r4	Col12a1
Elovl5	Arhgef17	Stoml1
Gclc	Ccdc85a	Loxl1
Lrrc1	Acsl3	Nptn
Tmed3	Rims3	Hcn4
Mlip	Rangrf	Neo1
Rasgrf1	Nfyc	Mto1
Ctsh	Fbxo21	Gsta4
Tpm1	Slc16a1	Elovl5
Zic1	Atg12	Gclc
Lactb	Sgpp2	Lrrc1

Car12	Cspg4	Tmed3
Plod2	Lrig2	Mlip
Aph1b	Hspa13	Rasgrf1
Usp3	Camkv	Ctsh
Ciao2a	Nup93	Tpm1
Snx1	Ucp3	Zic1
Ppib	Rasgrp2	Lactb
Csnk1g1	Ap4b1	Car12
Trip4	Pebp1	Plod2
Spg21	Ift57	Aph1b
Parp16	Fkbp1a	Usp3
Dpp8	Inha	Ciao2a
Dis3l	Slx9	Snx1
Tipin	Chpf	Ppib
Snapc5	Foxj3	Csnk1g1
Rpl4	Mycbp2	Trip4
Smad3	Sox10	Spg21
2300009A05Rik	Asic4	Parp16
Pias1	Ogfod1	Dpp8
U2surp	Trim33	Igdcc3
Atr	Nfatc1	Dis3l
Xrn1	Polr2f	Tipin
Tfdp2	Gmppa	Snapc5
Atp1b3	Cdo1	Rpl4
Rasa2	1700088E04Rik	Smad3
Ube3d	Afap1l1	2300009A05Rik
Rwdd2a	Micall1	Pias1
Me1	Eif3l	U2surp
Snx14	Npat	Atr
Syncrip	Ankrd54	Xrn1
Zfp949	Pygb	Tfdp2
Crtap	Lmo7	Atp1b3
Cmtm6	Resp18	Rasa2
Dync1li1	Cntnap3	Ube3d
Stt3b	Pfkm	Rwdd2a
Tgfbr2	Gas7	Me1
Zcwpw2	Entpd6	Nt5e
Slc25a36	Senp1	Snx14
Clstn2	Tbc1d4	Syncrip
Nmnat3	Triobp	Zfp949
Copb2	Apmap	Crtap
Mrps22	Nol12	Cmtm6
Pik3cb	Cdc14b	Dync1li1
Faim	Lss	Stt3b
Armc8	Slc7a6os	Tgfbr2
Dbr1	Cep15	Slc25a36
Mras	Atg9a	Clstn2
Nck1	Ybey	Copb2
Cdc25a	Gga1	Mrps22
Nme6	Slc22a15	Pik3cb
Map4	Phldb2	Faim
Dhx30	Podxl2	Armc8
Smarcc1	Abhd10	Dbr1

Cspg5
Scap
Ptgs2
Kif9
Pth1r
Lrrfip2
Mlh1
Dclk3
Trib1
Arpp21
Pdcd6ip
Fbxl2
Myd88
Wdr48
Gorasp1
Csrnp1
Mobp
Rpsa
Slc25a38
Hhatl
Nktr
Ss18l2
Pccb
Vipr1
Amotl2
Cck
Cep63
Trak1
Ephb1
Abhd5
Ryk
Rab6b
1110059G10Rik
Srprb
Trf
Topbp1
Uba5
Nphp3
Dnajc13
Gnai2
Mrpl3
Cpne4
Nudt16
Aste1
Atp2c1
Pik3r4
Manf
Cish
Hemk1
Rbm5
Rbm6
Mon1a
Traip
Cnppd1
Atp1a1
Dis3
Card10
Mgll
Tmed7
Mzt1
Tie1
Lpcat2
Mamdc2
S100b
Ttc28
Slitrk5
Eefsec
Scaf11
Trim45
Arid2
Hsf4
Szt2
Shf
Ttll4
Slc35a4
Stk36
Ptprm
Rpgrip1l
Wdr3
Kctd17
Noc4l
Ptprf
Lpp
Mif
Dpyd
Galnt9
Fem1c
Ctdp1
Kdm4a
Tnxb
Dnm2
Plppr5
Chst2
Map2k4
Prrg3
Usp37
Ipo13
Fntb
Palmd
Atp6v0b
Trappc8
Arhgap44
Clasp2
Spg11
Agl
Nudt15
Mras
Nck1
Cdc25a
Nme6
Map4
Dhx30
Smarcc1
Cspg5
Scap
Ptgs2
Pth1r
Lrrfip2
Mlh1
Dclk3
Trib1
Arpp21
Pdcd6ip
Fbxl2
Myd88
Wdr48
Gorasp1
Csrnp1
Mobp
Rpsa
Slc25a38
Hhatl
Nktr
Ss18l2
Pccb
Vipr1
Amotl2
Cck
Cep63
Trak1
Ephb1
Abhd5
Ryk
Slco2a1
Rab6b
1110059G10Rik
Srprb
Trf
Topbp1
Uba5
Nphp3
Dnajc13
Gnai2
Mrpl3
Cpne4
Nudt16
Aste1
Atp2c1
Pik3r4

Bsn	Ctdspl2	Manf
Apeh	Gucd1	Cish
Ip6k1	Cacul1	Hemk1
Nckipsd	Snap91	Rbm5
Ip6k2	Antxr1	Rbm6
Prkar2a	Eri3	Mon1a
Slc25a20	Mcee	Traip
Qars1	Terf2ip	Bsn
Nicn1	Gtpbp6	Apeh
Amt	Armcx2	Ip6k1
Klhdc8b	Trmt13	Uba7
Usp4	Specc1l	Nckipsd
Nt5m	Tagap	Ip6k2
Srek1	Adamts15	Prkar2a
Eml4	Zbtb1	Slc25a20
Thsd7a	Fan1	Qars1
Flcn	Armcx1	Nicn1
Atxn2l	Crlf2	Amt
Chsy1	Tomm6	Klhdc8b
Gpr19	Fhip2a	Usp4
Pygm	Fndc3a	Nt5m
Colgalt2	Cryzl2	Srek1
Crebl2	Larp4b	Eml4
Marchf3	Cdc14a	Thsd7a
Entrep3	Otud7a	Flcn
1700025G04Rik	Ppip5k1	Atxn2l
Pon2	Ttc7b	Chsy1
Cd59a	Idua	Gpr19
Malt1	Gtf2a2	Pygm
Lmo2	Znrf1	Colgalt2
Kank1	Dph5	Crebl2
Exd2	Fam20b	Marchf3
Resf1	Rbfox2	Entrep3
Syde1	Adgrb3	1700025G04Rik
Mansc1	Myo6	Pon2
Abtb2	Tmem35a	Cd59a
Mier3	Fa2h	Malt1
Snx33	Ndn	Lmo2
Ablim3	Spata2l	Kank1
Inppl1	Lgi3	Exd2
Ccdc88a	Rfwd3	Resf1
Tpcn1	Caskin1	Syde1
Katnip	Pank1	Mansc1
Heyl	Cplx1	Abtb2
Gpbp1	Map3k13	Mier3
Slc8b1	Pcgf3	Snx33
Bet1	Pdpr	Ablim3
Ilvbl	Pik3c3	Inppl1
Gng11	Hacd3	Ccdc88a
Chrm1	AW554918	Tpcn1
Gtf3c1	Vps8	Katnip
Cntrob	Ddx19b	Heyl
Alas1	Zfp7	Gpbp1

Pdxk
Srxn1
Cdv3
Slc10a3
Alox12b
Arap1
Igdcc4
Ank2
Ppp1r9a
Pwp2
Trpc1
Spring1
Prr5l
Abcc10
Zswim6
Abcc4
Rnft2
Ugt8a
Pkd1
Fbxw8
Psmf1
Smap2
Cyb5r4
Arhgef17
Ccdc85a
Acsl3
Rims3
Rangrf
Nfyc
Fbxo21
Slc16a1
Atg12
Sgpp2
Cspg4
Lrig2
Hspa13
Camkv
Nup93
Rbm11
Ucp3
Rasgrp2
Ap4b1
Pebp1
Ift57
Fkbp1a
Inha
Slx9
Chpf
Foxj3
Mycbp2
Sox10
Asic4
Ogfod1
Cep350
Gabrb3
Qsox1
Ucp2
Arhgap39
Acbd6
Fcsk
Stard9
Smyd5
Lrrc24
Ccar2
Foxn3
Adra2a
Sfxn5
Vav3
BC034090
Lrrc14
Egr3
Sf3b3
Spr
Fkbpl
St18
Gadd45gip1
Rbm4b
Mtss2
Tmem131l
Nrxn2
Exoc6b
Rpap2
Asb13
Tubgcp5
Atp7a
Atp6v1h
Tasor2
Ephx4
Tmem87a
Alg3
Tcea1
Ppp1r16a
Mrpl15
B3galt2
Kcnk10
Ston1
Engase
Cdk10
Klf9
Ppargc1b
Lgals3bp
Zfp267
Pxk
Map9
Mapkbp1
Ccp110
Slc8b1
Bet1
Ilvbl
Gng11
Chrm1
Mctp2
Gtf3c1
Cntrob
Alas1
Pdxk
Lama1
Srxn1
Cdv3
Arap1
Igdcc4
Ank2
Ppp1r9a
Pwp2
Trpc1
Spring1
Prr5l
Abcc10
Zswim6
Abcc4
Rnft2
Ugt8a
Pkd1
Fbxw8
Psmf1
Smap2
Cyb5r4
Arhgef17
Ccdc85a
Acsl3
Rims3
Rangrf
Nfyc
Fbxo21
Slc16a1
Atg12
Sgpp2
Cspg4
Lrig2
Itgbl1
Hspa13
Camkv
Nup93
Rbm11
Ucp3
Rasgrp2
Ap4b1
Pebp1
Ift57

Trim33	Zdhhc15	Fkbp1a
Nfatc1	Usp36	Inha
Polr2f	Gucy1a1	Slx9
Gmppa	Chmp2a	Chpf
Cdo1	Gde1	Foxj3
1700088E04Rik	Parl	Mycbp2
Afap1l1	Rbm34	Sox10
Calhm2	Vhl	Asic4
Micall1	Ndufb7	Ogfod1
Eif3l	Brk1	Trim33
Npat	Mga	Nfatc1
Ankrd54	Zswim5	Polr2f
Pygb	Trim36	Gmppa
Lmo7	Ppp3r1	Cdo1
Resp18	Tnks1bp1	Afap1l1
Cntnap3	Jcad	Calhm2
Pfkm	Zfp446	Micall1
Gas7	Zbtb41	Eif3l
Entpd6	Slc16a2	Npat
Senp1	Cdkl4	Ankrd54
Tbc1d4	Rfc3	Pygb
Triobp	Gria2	Lmo7
Apmap	Coil	Resp18
Nol12	Tesk2	Cntnap3
Cdc14b	Skic3	Pfkm
Lss	Kcnk1	Gas7
Slc7a6os	Neu4	Entpd6
Cep15	Slc66a2	Senp1
Atg9a	Scaper	Clec1a
Ybey	Pds5b	Tbc1d4
Gga1	Cpsf1	Triobp
Slc22a15	Cct2	Apmap
Phldb2	Rpap1	Nol12
Podxl2	Fgd5	Cdc14b
Abhd10	Galnt17	Lss
Cnppd1	Gpbp1l1	Slc7a6os
Atp1a1	Phka1	Cep15
Dis3	Ypel4	Atg9a
Card10	Poglut1	Ybey
Mgll	Farp2	Gga1
Tmprss7	Zfp551	Slc22a15
Tmed7	Zdhhc5	Phldb2
Mzt1	Ccdc174	Podxl2
Tie1	Hdlbp	Abhd10
Lpcat2	Fstl5	Cnppd1
Mamdc2	Ctnnd1	Atp1a1
S100b	Meak7	Dis3
Ttc28	Ccs	Card10
Slitrk5	Golim4	Mgll
Eefsec	Kctd7	Tmed7
Ttf2	Tmed8	Mzt1
Scaf11	Tpst1	Tie1
Trim45	Srsf2	Lpcat2

Arid2	Mks1	Mamdc2
Hsf4	Pomt2	S100b
Szt2	Sik3	Ttc28
Shf	Tmem63c	Slitrk5
Ttll4	Exoc3	Eefsec
Slc35a4	Ino80	Ttf2
Stk36	Tspoap1	Scaf11
Ptprm	Cipc	Trim45
Rpgrip1l	Lrrc58	Arid2
Wdr3	Ogt	Hsf4
Kctd17	Scx	Szt2
Noc4l	Zfc3h1	Shf
Ptprf	Emid1	Ttll4
Lpp	Ccnd3	Slc35a4
Mif	Irf2bpl	Stk36
Dpyd	Faah	Ptprm
Galnt9	Zbed5	Rpgrip1l
Gstt2	Rhbdd3	Wdr3
Fem1c	Tmem275	Kctd17
Ctdp1	Nsf	Noc4l
Kdm4a	Hsdl1	Ptprf
Dnm2	Chmp7	Lpp
Plppr5	Lsm3	Mif
Chst2	R3hcc1	Dpyd
Map2k4	Gas2l1	Galnt9
Pus7l	Chchd4	Fem1c
Prrg3	Efcab14	Ctdp1
Usp37	Mrps17	Kdm4a
Ipo13	Vps18	Tnxb
Fntb	Atm	Dnm2
Palmd	Gpc1	Plppr5
Atp6v0b	Rhov	Chst2
Trappc8	Foxj1	Map2k4
Arhgap44	Usp54	Pus7l
Clasp2	Golgb1	Prrg3
Spg11	Hdac11	Usp37
Agl	Plekhm1	Ipo13
Nudt15	Slc25a37	Fntb
Ctdspl2	Senp6	Palmd
Gucd1	Agpat1	Atp6v0b
Cacul1	Arhgap27	Trappc8
Snap91	Exosc4	Frrs1
Antxr1	Ints14	Arhgap44
Eri3	Zdhhc14	Clasp2
Mcee	Setd5	Spg11
Terf2ip	Jdp2	Agl
Gtpbp6	Thoc5	Nudt15
Armcx2	Igsf9b	Ctdspl2
Trmt13	Dnajc17	Gucd1
Specc1l	Evpl	Cacul1
Tagap	Nipsnap1	Snap91
Adamts15	Nek9	Antxr1
Zbtb1	Traf3ip1	Eri3

Fan1	Fhod3	Mcee
Armcx1	Med13	Terf2ip
Crlf2	Fam53c	Gtpbp6
Tomm6	Sdr42e1	Armcx2
Fhip2a	Tmem132d	Trmt13
Catsper2	Iqsec1	Specc1l
Fndc3a	Slc26a2	Tagap
Cryzl2	Exosc1	Adamts15
Prss35	Tmem132c	Zbtb1
Larp4b	Kctd9	Fan1
Otud7a	Plcg2	Armcx1
Ppip5k1	Zbed4	Crlf2
Ttc7b	Ina	Tomm6
Idua	Wbp2	Fhip2a
Gtf2a2	Cbl	Catsper2
Znrf1	Ube2f	Fndc3a
Dph5	Gtf2h5	Cryzl2
Fam20b	Smc4	Prss35
Rbfox2	Ramp1	Larp4b
Adgrb3	Mtmr3	Cdc14a
Myo6	Cpne2	Otud7a
Tmem35a	Tkfc	Ppip5k1
Fa2h	Tulp4	Ttc7b
Ndn	Wdr5b	Idua
Spata2l	Cmip	Gtf2a2
Lgi3	Spata6	Znrf1
Rfwd3	Kcnh5	Dph5
Caskin1	Pja1	Fam20b
Pank1	Tbc1d10a	Rbfox2
Cplx1	Neurl1b	Adgrb3
Map3k13	Gcsh	Myo6
Pcgf3	Zfp707	Tmem35a
Pdpr	Zxdc	Fa2h
Pik3c3	Cops8	Ndn
Hacd3	Trmt5	Spata2l
AW554918	Cyb561a3	Lgi3
Vps8	Polr3b	Rfwd3
Ddx19b	Pkd2	Caskin1
Zfp7	Scara3	Pank1
Cep350	Caskin2	Cplx1
Gabrb3	Rasd2	Map3k13
Qsox1	Sec22a	Pcgf3
Ucp2	Diaph2	Pdpr
Arhgap39	Snx2	Pik3c3
Acbd6	Uaca	Hacd3
Fcsk	Poglut3	AW554918
Stard9	Edil3	Vps8
Smyd5	Pcnx4	Ddx19b
Lrrc24	Mad2l1bp	Zfp7
Ccar2	Hmgxb4	Cep350
Foxn3	Zfp395	Gabrb3
Adra2a	Ice1	Qsox1
Sfxn5	Fbxo16	Ucp2

Vav3	Zfp418	Arhgap39
BC034090	Morc2a	Acbd6
Emx1	Rsrc1	Fcsk
Lrrc14	Zfyve9	Stard9
Egr3	Washc4	Smyd5
Sf3b3	Ccpg1	Lrrc24
Spr	Atp5pd	Ccar2
Fkbpl	Inpp5j	Foxn3
Gadd45gip1	Ptpn13	Adra2a
Rbm4b	Daam1	Sfxn5
Mtss2	Tent4a	Vav3
Tmem131l	Pla2g3	BC034090
Nrxn2	Hid1	Emx1
Exoc6b	Slc41a2	Lrrc14
Rpap2	Myo5a	Egr3
Asb13	Ppp1r18	Sf3b3
Dysf	2700049A03Rik	Spr
Tubgcp5	Mon2	Fkbpl
Atp7a	Tut4	St18
Atp6v1h	Chst11	Gadd45gip1
Tasor2	Ppm1h	Rbm4b
Ephx4	Pik3ip1	Mtss2
Tmem87a	Ssh3	Tmem131l
Alg3	Mtrr	Nrxn2
Tcea1	Rxylt1	Exoc6b
Ppp1r16a	Gpatch8	Rpap2
Dnah8	Zyg11b	Asb13
Mrpl15	Setmar	Dysf
B3galt2	Tiparp	Tubgcp5
Kcnk10	Ankrd12	Atp7a
Engase	Lrrn1	Atp6v1h
Cdk10	Ythdc2	Tasor2
Klf9	Cacna1a	Ephx4
Ppargc1b	Tmem109	Tmem87a
Lgals3bp	Bmp2k	Alg3
Zfp267	Xpot	Tcea1
Pxk	Pbx2	Ppp1r16a
Map9	Tdg	Dnah8
Mapkbp1	Dbn1	Mrpl15
Ccp110	Rnps1	B3galt2
Zdhhc15	Sema3f	Kcnk10
Usp36	Fam171a2	Ston1
Gucy1a1	Prr7	Engase
Chmp2a	Fras1	Cdk10
Gde1	Dnai2	Klf9
Parl	Gns	Ppargc1b
Rbm34	Grn	Lgals3bp
Vhl	Ppp1r21	Zfp267
Ndufb7	Ttyh2	Pxk
Brk1	Tmx4	Map9
Mga	Cnot6l	Mapkbp1
Zswim5	Mrps10	Ccp110
Trim36	Adgrb1	Zdhhc15

Ppp3r1	Dgkh	Usp36
Tnks1bp1	Nagk	Gucy1a1
Jcad	Sirt6	Chmp2a
Zfp446	Mast4	Gde1
Zbtb41	Pcdh11x	Parl
Slc16a2	Tmub2	Rbm34
Cdkl4	Map4k5	Vhl
Rfc3	Dusp5	Ndufb7
Zfp944	Tle2	Brk1
Gria2	B3galt1	Mga
Coil	Gna11	Zswim5
Tesk2	Rab24	Trim36
Skic3	G6pc3	Ppp3r1
Kcnk1	Cpne7	Tnks1bp1
Neu4	Unc13a	Jcad
Slc66a2	Zfp661	Zfp446
Scaper	Sos2	Zbtb41
Rxfp1	Colgalt1	Slc16a2
Pds5b	Grip1	Cdkl4
Cpsf1	Celf5	Rfc3
Fancd2	Cpsf7	Zfp944
Cct2	Nrip3	Gria2
Rpap1	Nup54	Coil
Fgd5	Tet3	Tesk2
Galnt17	Larp6	Skic3
Gpbp1l1	Plvap	Kcnk1
Phka1	Ttc21b	Neu4
Ypel4	Tmem127	Slc66a2
Poglut1	Acot11	Scaper
Farp2	Mfsd12	Pds5b
Zfp551	Atosa	Cpsf1
Zdhhc5	Ano8	Cct2
Ccdc174	Ankrd27	Rpap1
Hdlbp	Myl12b	Fgd5
Fstl5	Nudt19	Galnt17
Ctnnd1	Mrpl34	Gpbp1l1
Meak7	Cactin	Phka1
Ccs	Sncb	Ypel4
Golim4	Rps29	Poglut1
Kctd7	Cog3	Farp2
Tmed8	Filip1	Zfp551
Vav1	Pip5k1c	Zdhhc5
Tpst1	Cobll1	Ccdc174
Srsf2	Sidt2	Hdlbp
Mks1	Pygo1	Fstl5
Pomt2	Mdga2	Ctnnd1
Sik3	Dhcr24	Meak7
Tmem63c	Rnf44	Ccs
Exoc3	Rtkn	Golim4
Ino80	Dhx8	Kctd7
Tspoap1	Mrpl54	Tmed8
Cipc	Arl4d	Vav1
Lrrc58	Synrg	Tpst1

Ogt	Zfr2	Srsf2
Scx	Cog7	Mks1
Zfc3h1	Cebpa	Pomt2
Emid1	Atcay	Sik3
Ccnd3	Dop1a	Tmem63c
Irf2bpl	Dapk3	Exoc3
Faah	Parm1	Ino80
Zbed5	Vat1	Tspoap1
Rhbdd3	Eef2	Cipc
Rnf43	Htr2a	Lrrc58
Nsf	Foxn2	Ogt
Hsdl1	Rundc1	Zfc3h1
Chmp7	Zbtb7a	Emid1
Lsm3	Ncapd3	Ccnd3
R3hcc1	Map2k2	Irf2bpl
Gas2l1	Zc3h12b	Faah
Chchd4	Kri1	Zbed5
Rasl10a	Anapc13	Rhbdd3
Efcab14	Rrp12	Rnf43
Mrps17	Dhx57	Nsf
Vps18	Zc4h2	Hsdl1
Atm	Eef2k	Chmp7
Gpc1	Xkr6	Lsm3
Rhov	Oma1	R3hcc1
Foxj1	Mtmr9	Gas2l1
Usp54	Becn1	Chchd4
Golgb1	Secisbp2l	Rasl10a
Hdac11	Fam167a	Efcab14
Plekhm1	Eva1a	Mrps17
Slc25a37	Egln3	Vps18
Senp6	Dcbld2	Atm
Agpat1	Shc4	Gpc1
Arhgap27	Gcfc2	Rhov
Exosc4	Dnai4	Foxj1
Ints14	Brinp3	Usp54
Zdhhc14	Arhgap5	Golgb1
Setd5	Secisbp2	Hdac11
Jdp2	Nubpl	Plekhm1
Thoc5	Eif2s3x	Slc25a37
Igsf9b	Elmod2	Senp6
Dnajc17	Ap2b1	Agpat1
Evpl	Mitf	Arhgap27
Nipsnap1	Ints6	Exosc4
Nek9	Zc3h12c	Ints14
Traf3ip1	Tanc1	Zdhhc14
Fhod3	1110059E24Rik	Setd5
Med13	Plekhh3	Jdp2
Fam53c	Ccdc186	Thoc5
Sdr42e1	Saxo4	Igsf9b
Tmem132d	Heatr5a	Dnajc17
Iqsec1	Fam124a	Evpl
Trim59	Ano4	Nipsnap1
Slc26a2	Tubg1	Nek9

Exosc1	Arl6ip5	Traf3ip1
Tmem132c	Lars2	Fhod3
Kctd9	Epn1	Med13
Plcg2	Sppl2b	Fam53c
Zbed4	Leprot	Sdr42e1
Fam151b	Lsm7	Tmem132d
Ina	Rims4	Iqsec1
Wbp2	Spcs2	Trim59
Cbl	Ccdc106	Slc26a2
Ube2f	Pdk3	Exosc1
Gtf2h5	Trim13	Tmem132c
Smc4	Scai	Kctd9
Ramp1	Lcat	Plcg2
Mtmr3	Oaz1	Zbed4
Cpne2	Eogt	Ina
Tkfc	Pcyt1b	Wbp2
Tulp4	Hectd1	Cbl
Wdr5b	Tut7	Ube2f
Cmip	Helq	Gtf2h5
Kcnh5	Pkig	Smc4
Pja1	Tpbg	Ramp1
Tbc1d10a	Raver2	Mtmr3
Neurl1b	Arx	Cpne2
Gcsh	Plekhj1	Tkfc
Zfp707	Adrb1	Tulp4
Zxdc	Vps13c	Wdr5b
Cops8	Nat14	Cmip
Trmt5	G2e3	Kcnh5
Cyb561a3	Cops4	Pja1
Dhrs11	Mid1	Tbc1d10a
Polr3b	Gnptab	Neurl1b
Pkd2	Gdpd5	Gcsh
Scara3	Sec31a	Zfp707
Caskin2	Fbxo33	Zxdc
Rasd2	Lzts2	Cops8
Sec22a	Nup37	Trmt5
Diaph2	Uvrag	Cyb561a3
Snx2	Kcnh4	Dhrs11
Uaca	Nfkbiz	Polr3b
Poglut3	Pdzrn3	Pkd2
Edil3	Rmi1	Scara3
Pcnx4	Gm49322	Caskin2
Mad2l1bp	1810055G02Rik	Rasd2
Hmgxb4	Hacd2	Sec22a
Gjc1	Shq1	Diaph2
Zfp395	Pcsk7	Snx2
Ice1	Brsk1	Uaca
Fbxo16	Dennd1a	Poglut3
Zfp418	Klf16	Edil3
Morc2a	Oser1	Pcnx4
Rsrc1	Emsy	Mad2l1bp
Zfyve9	Crb2	Hmgxb4
Washc4	Kank4	Gjc1

Ccpg1	Tmem98	Zfp395
Atp5pd	Sstr1	Ice1
Inpp5j	Rabgap1	Fbxo16
Ptpn13	Haus8	Zfp418
Daam1	Myo1d	Morc2a
Tent4a	Thyn1	Rsrc1
Exph5	Rcbtb1	Hdx
Hid1	Tab3	Zfyve9
8430429K09Rik	Mbd3	Washc4
Slc41a2	Plk5	Ccpg1
Myo5a	Tstd2	Atp5pd
Ppp1r18	Reep6	Inpp5j
2700049A03Rik	Cox18	Ptpn13
Mon2	Fbxl21	Daam1
Tut4	Ntng2	Tent4a
Chst11	Tdrd7	Exph5
Ppm1h	Gnptg	Hid1
Pik3ip1	Prdm4	8430429K09Rik
Ssh3	Eif1	Slc41a2
Mtrr	Ccdc180	Myo5a
Rxylt1	Leng8	Ppp1r18
Gpatch8	Capn5	2700049A03Rik
Zyg11b	Igfbpl1	Mon2
Setmar	Mpv17l2	Tut4
Tiparp	Aldh1b1	Chst11
Ankrd12	Pcdh17	Ppm1h
Lrrn1	Ankrd11	Pik3ip1
Ythdc2	Dcaf10	Ssh3
Cacna1a	Utp6	Mtrr
Tmem109	L3mbtl1	Rxylt1
Bmp2k	Iqcg	Gpatch8
Xpot	Tsen34	Zyg11b
Pbx2	Mboat7	Setmar
Tdg	Prpf39	Tiparp
Dbn1	Trmt10b	Ankrd12
Rnps1	Ky	Lrrn1
Sema3f	Togaram1	Ythdc2
Fam171a2	Frmpd1	Cacna1a
Prr7	Ric8b	Tmem109
Fras1	Midn	Bmp2k
Neurod1	Rsf1	Xpot
Gns	Rubcn	Pbx2
Grn	Cnot3	Tdg
Ppp1r21	Grhpr	Dbn1
Ttyh2	Cbarp	Rnps1
Tmx4	Aamdc	Fam171a2
Cnot6l	Zcchc7	Prr7
Mrps10	Lrfn5	Fras1
Adgrb1	Gtf3c4	Neurod1
Dgkh	Zswim4	Gns
Pabpc5	Sbno2	Grn
Nagk	Ndufa3	Ppp1r21
Sirt6	Kcnc2	Ttyh2

Mast4	Thrsp	Tmx4
Pcdh11x	Rnf38	Cnot6l
Tmub2	Arhgap45	Mrps10
Tle6	Alg8	Adgrb1
Map4k5	Dok3	Dgkh
Dusp5	Usp35	Pabpc5
Asb16	Abca7	Nagk
Tle2	Prkx	Sirt6
B3galt1	Supt16	Mast4
Gna11	Dagla	Pcdh11x
Rab24	Wdr18	Tmub2
Gna15	Selenoo	Map4k5
G6pc3	Bbs10	Dusp5
Cpne7	Tmem161b	Asb16
Unc13a	Fbxo45	Tle2
Zfp661	Dym	B3galt1
Sos2	Xylb	Gna11
Colgalt1	Dync1li2	Rab24
Scn7a	Mrps2	Gna15
Grip1	Kiss1r	G6pc3
Celf5	Cd99l2	Cpne7
Cpsf7	R3hdm4	Unc13a
Nrip3	Acta2	Zfp661
Nup54	Cep19	Sos2
Tet3	Zdhhc17	Colgalt1
Larp6	Mlc1	Scn7a
Plvap	Tk2	Grip1
Ttc21b	Pim3	Celf5
Tmem127	Ppp1r26	Cpsf7
Acot11	Plppr3	Nrip3
Mfsd12	Lysmd3	Nup54
Atosa	Alg12	Tet3
Ano8	Ids	Larp6
Ankrd27	Krt222	Plvap
Myl12b	Ythdc1	Ttc21b
Nudt19	Palm	Tmem127
Mrpl34	Syt1	Acot11
Cactin	Zfp983	Mfsd12
Sncb	Zhx3	Atosa
Rps29	Hykk	Ano8
Cog3	Cox8a	Ankrd27
Filip1	Rnf126	Myl12b
Pip5k1c	Cerk	Gipc3
Cobll1	Uba6	Nudt19
Sidt2	Gramd4	Mrpl34
Pygo1	Dennd5a	Cactin
Ushbp1	Dcdc2a	Sncb
Mdga2	Cd276	Rps29
Dhcr24	Bbs9	Cog3
Rnf44	Cog5	Filip1
Rtkn	Pknox2	Pip5k1c
Dhx8	Aldh5a1	Cobll1
Mrpl54	Ibtk	Sidt2

Arl4d	Ttc38	Pygo1
Synrg	Acss3	Ushbp1
Zfr2	Fbxw2	Mdga2
Cog7	Pip4p1	Tjp3
Cebpa	Dock4	Dhcr24
Atcay	Tdp2	Rnf44
Rubcnl	Apex1	Rtkn
Dop1a	Tmem59l	Dhx8
Dapk3	Ints6l	Mrpl54
Parm1	Rusc2	Arl4d
Hrh2	Nme5	Synrg
Vat1	Fnip1	Zfr2
Eef2	Atosb	Cog7
Htr2a	Ripor2	Cebpa
Foxn2	Mettl25	Atcay
Rundc1	Tmtc2	Dop1a
Zbtb7a	Pabir2	Dapk3
Ncapd3	Parp2	Parm1
Map2k2	Tmem63b	Hrh2
Tbr1	Prtg	Vat1
Zc3h12b	Zfp57	Eef2
Kri1	Adamtsl2	Htr2a
Anapc13	5031439G07Rik	Foxn2
Rrp12	Dnajb5	Rundc1
Dhx57	Fmnl2	Zbtb7a
Zc4h2	Sugp2	Ncapd3
Eef2k	Ptpn23	Map2k2
Xkr6	Smug1	Tbr1
Oma1	Phf24	Zc3h12b
Mtmr9	Slc2a6	Kri1
Becn1	Galt	Anapc13
Secisbp2l	Sigmar1	Rrp12
Eva1a	Zranb3	Dhx57
Egln3	Slain2	Zc4h2
Dcbld2	Arl5a	Eef2k
Shc4	Dgkb	Xkr6
Gcfc2	Slf2	Oma1
Dnai4	Myrf	Mtmr9
Brinp3	Vezt	Becn1
Arhgap5	Colec12	Secisbp2l
Secisbp2	Rab3gap1	Eva1a
Nubpl	Prr5	Egln3
Eif2s3x	Lmo1	Dcbld2
Elmod2	Metap2	Shc4
Ap2b1	Rpp25l	Wnk4
Mitf	Rfxank	Neil2
Ints6	Acaa1a	Gcfc2
Zc3h12c	Mgat5	Dnai4
Tanc1	Prickle1	Brinp3
1110059E24Rik	Surf6	Arhgap5
Plekhh3	Pphln1	Secisbp2
Ccdc186	Gatad2a	Nubpl
Heatr5a	H1f2	Eif2s3x

Fam124a	Dipk1b	Elmod2
Ano4	Ankmy2	Ap2b1
Tubg1	Rorb	Mitf
Arl6ip5	Gxylt1	Ints6
Lars2	Arhgap36	Zc3h12c
Epn1	Ndufa13	Tanc1
Sppl2b	Rif1	1110059E24Rik
Leprot	Sh3bp4	Plekhh3
Lsm7	Nepro	Ccdc186
Rims4	Pdzrn4	Heatr5a
Spcs2	Kctd1	Fam124a
Ccdc106	Ube2r2	Ano4
Pdk3	Armh4	Tubg1
Abraxas1	Gmip	Arl6ip5
Trim13	Rbm43	Lars2
Scai	Igfbp7	Epn1
Lcat	Pnpla8	Sppl2b
Neu3	Fstl4	Leprot
Oaz1	Edc4	Lsm7
Eogt	Lrrk2	Rims4
Pcyt1b	9530068E07Rik	Spcs2
Hectd1	Macrod1	Ccdc106
Tut7	Snapc4	Pdk3
Abi3bp	Naa30	Abraxas1
Helq	Noa1	Trim13
Pkig	Ap5m1	Scai
Tpbg	Lrrn3	Lcat
Raver2	Slc2a13	Neu3
Arx	BC031181	Oaz1
Plekhj1	Zdhhc23	Eogt
Adrb1	Lzts1	Pcyt1b
Vps13c	Skp1	Hectd1
Nat14	Polr1h	Tut7
G2e3	Srp72	Helq
Cops4	Qsox2	Pkig
Mid1	Kidins220	Tpbg
Gnptab	Igsf10	Raver2
Gdpd5	Tmem260	Arx
Sec31a	Ubac1	Plekhj1
Fbxo33	Csgalnact1	Adrb1
Lzts2	Gpr101	Vps13c
Nup37	Rmdn2	Nat14
Uvrag	Serbp1	G2e3
Kcnh4	Tmem258	Cops4
Nfkbiz	Abt1	Mid1
Pdzrn3	Cracd	Ror1
Rmi1	Gadd45a	Gnptab
Gm49322	Sec24a	Gdpd5
1810055G02Rik	Ppp1r11	Sec31a
Hacd2	Gng12	Fbxo33
Shq1	Cep135	Lzts2
Pcsk7	Matcap2	Nup37
Brsk1	Pcdh8	Uvrag

Dennd1a	Gpi1	Kcnh4
Klf16	Tbcc	Nfkbiz
Oser1	Siah2	Pdzrn3
Emsy	Exoc1	Rmi1
Crb2	Npy1r	Gm49322
Kank4	Calm2	1810055G02Rik
Tmem98	Thap11	Hacd2
Sstr1	Hif1an	Shq1
Rabgap1	Arhgap26	Pcsk7
Haus8	Wtip	Brsk1
Myo1d	Elf1	Dennd1a
Thyn1	Megf11	Klf16
Prdm8	Marchf1	Oser1
Rcbtb1	Tbc1d24	Emsy
Tab3	Btg1	Crb2
Mbd3	Eea1	Kank4
Plk5	Fam13b	Tmem98
Tstd2	Tmem255a	Sstr1
Reep6	Rnf13	Rabgap1
Cox18	Phpt1	Haus8
Fbxl21	Cdh8	Myo1d
Ntng2	Commd2	Thyn1
Tdrd7	Ppfibp2	Prdm8
Gnptg	Sbf1	Rcbtb1
Prdm4	Cdc42ep3	Tab3
Eif1	Slc38a7	Mbd3
Leng8	Rnf113a1	Plk5
Capn5	Adamts2	Tgfbi
Mpv17l2	Cnot1	Tstd2
Aldh1b1	Ermard	Reep6
Pcdh17	Iqce	Cox18
Ankrd11	Lgi4	Slc12a8
Dcaf10	Ppp6r2	Fbxl21
Utp6	Ndrg4	Ntng2
L3mbtl1	Ttyh3	Tdrd7
Iqcg	Bicral	Gnptg
Tsen34	Fxyd1	Prdm4
Chrna5	Upf3b	Eif1
Fam174c	Fxyd7	Leng8
Mboat7	Spart	Capn5
Prpf39	Fgf1	Igfbpl1
Trmt10b	Arhgap21	Mpv17l2
Togaram1	Chst12	Aldh1b1
Frmpd1	Plxnb2	Pcdh17
Ric8b	Eepd1	Ankrd11
Midn	Eipr1	Dcaf10
Rsf1	Rfxap	Utp6
Rubcn	Etl4	L3mbtl1
Cnot3	Mgat4b	Tsen34
Grhpr	Atp13a2	Mboat7
Cbarp	Alg5	Prpf39
Aamdc	Mag	Trmt10b
Zcchc7	Clcn7	Ky

Lrfn5	Nudt1	Togaram1
Gtf3c4	Ccdc148	Frmpd1
Zswim4	Tbc1d9b	Ric8b
Sbno2	Man1b1	Midn
Ndufa3	Dennd3	Rsf1
Kcnc2	Tcaf1	Rubcn
Thrsp	Cenpt	Cnot3
Rnf38	Tmtc3	Grhpr
Arhgap45	Aaas	Cbarp
Alg8	Cc2d1a	Aamdc
Dok3	Nop14	Zcchc7
Usp35	Ago2	Lrfn5
Abca7	Zcchc12	Gtf3c4
Prkx	Cab39	Zswim4
Supt16	Cyld	Sbno2
Dagla	Micall2	Ndufa3
Wdr18	Zscan12	Kcnc2
Selenoo	Rbm42	Thrsp
Bbs10	Oxsr1	Rnf38
Tmem161b	Ttll7	Arhgap45
Fbxo45	Cuedc2	Alg8
Dym	Cox6b1	Dok3
Xylb	Tubb4b	Usp35
Dync1li2	Kcnk9	Abca7
Mrps2	Dnajc12	Prkx
Kiss1r	Dner	Supt16
Cd99l2	Wdr44	Dagla
R3hdm4	Decr2	Wdr18
Cep19	Anln	Selenoo
Zdhhc17	Tent4b	Bbs10
Mlc1	Klhl13	Tmem161b
Tk2	Slitrk2	Fbxo45
Pim3	Mbd5	Dym
Ppp1r26	Fam135b	Xylb
Polr3g	Cnep1r1	Dync1li2
Plppr3	Slc6a20a	Mrps2
Lysmd3	Dpp10	Kiss1r
Alg12	Sun1	Cd99l2
Ids	Jmjd4	R3hdm4
Krt222	Amdhd2	Acta2
Ythdc1	Topors	Cep19
Palm	Ssx2ip	Zdhhc17
Syt1	Pnpla7	Twist1
Zfp983	Plch1	Mlc1
Zhx3	Psenen	Tk2
Hykk	Siah1a	Pim3
Cox8a	Lin37	Ppp1r26
Rnf126	Mrpl41	Polr3g
Cerk	Wnt4	Plppr3
Uba6	Mrpl55	Lysmd3
Gramd4	Dchs1	Alg12
Dennd5a	Syde2	Ids
Dcdc2a	Proser3	Krt222

Cd276	2410004B18Rik	Ythdc1
Bbs9	Phkb	Palm
Cog5	Acaa2	Syt1
Pknox2	Arhgap33	Zfp983
Aldh5a1	Arhgef26	Zhx3
Ibtk	C1qa	Hykk
Ttc38	Gtdc1	Cox8a
Fbxw2	Ehmt1	Rnf126
Pip4p1	Rap2b	Cerk
Dock4	C1qc	Uba6
Tdp2	Zfp157	Gramd4
Apex1	Neto2	Dennd5a
Tmem59l	Fzd8	Dcdc2a
Ints6l	C1qb	Cd276
Rusc2	C1ql2	Bbs9
Nme5	Unc93b1	H2-Q4
Fnip1	Trim67	Cog5
Atosb	Kirrel2	Pknox2
Ripor2	Zfp280c	Aldh5a1
Mettl25	Ttc7	Ibtk
Tmtc2	Stox1	Ttc38
Pabir2	Aifm1	Fbxw2
Parp2	4921524J17Rik	Pip4p1
Tmem63b	Kdm1a	Dock4
Zfp57	Elac1	Tdp2
Adamtsl2	Rab8b	Apex1
5031439G07Rik	Trappc14	Tmem59l
Dnajb5	Slc39a12	Ints6l
Fmnl2	Kifbp	Rusc2
Sugp2	Lrfn3	Nme5
Ptpn23	Bcorl1	Fnip1
Smug1	Trim17	Atosb
Phf24	Spryd3	Ripor2
Slc2a6	Cnpy4	Mettl25
Galt	Zic4	Tmtc2
Sigmar1	Tmem177	Pabir2
Zranb3	Anapc10	Parp2
Slain2	Taf6	Tmem63b
Arl5a	Zdhhc9	Prtg
Dgkb	Pml	Zfp57
Slf2	Trim3	Adamtsl2
Myrf	Otud4	5031439G07Rik
Vezt	Asap3	Dnajb5
Colec12	Zfp39	Fmnl2
Rab3gap1	Tns2	Sugp2
Prr5	Zfp113	Ptpn23
Lmo1	Apln	Smug1
Metap2	Hk1	Phf24
Rpp25l	Ss18	Slc2a6
Rfxank	Sstr4	Galt
Acaa1a	Frem2	Sigmar1
Mgat5	Zscan21	Zranb3
Prickle1	Mmaa	Slain2

Surf6	Zfp146	Arl5a
Pphln1	Tspan15	Dgkb
Gatad2a	Apbb1	Slf2
H1f2	Inhbb	Myrf
Dipk1b	Smpd1	Vezt
Ankmy2	Paip2	Colec12
Rorb	Cavin3	Rab3gap1
Slc26a8	Sh3glb1	Prr5
Gxylt1	Rbmxl1	Lmo1
Ndufa13	Scd1	Metap2
Rif1	Selenof	Rpp25l
Sh3bp4	Rnf139	Rfxank
Nepro	Trmt12	Acaa1a
Polr1has	Slc35b2	Mgat5
Pdzrn4	Rab11fip3	Prickle1
Kctd1	Dcaf15	Surf6
Ube2r2	Socs5	Pphln1
Armh4	Zcwpw1	Gatad2a
Gmip	Ralgapa2	H1f2
Rbm43	Setd7	Dipk1b
Igfbp7	Sik2	Ankmy2
Pnpla8	Fam91a1	Rorb
Fstl4	Psd	Slc26a8
Edc4	Prmt9	Gxylt1
Lrrk2	Aff3	Ndufa13
9530068E07Rik	Cfap61	Rif1
Macrod1	Arhgap10	Sh3bp4
Snapc4	Ddx1	Nepro
Naa30	Lrrc20	Polr1has
Noa1	Ndufc1	Pdzrn4
Ap5m1	Ppp1r14a	Kctd1
Lrrn3	Mycn	Ube2r2
Slc2a13	Dennd11	Armh4
BC031181	Elf2	Gmip
Zdhhc23	Cyb561d2	Rbm43
Lzts1	Pacrg	Igfbp7
Skp1	Rbm17	Pnpla8
Polr1h	Atg101	Fstl4
Srp72	Islr	Edc4
Qsox2	Fam193a	Lrrk2
Kidins220	Spry1	9530068E07Rik
Tmem260	Thap1	Macrod1
Ubac1	Lipt1	Snapc4
P2ry12	Syn1	Naa30
Csgalnact1	Mospd3	Noa1
P2ry13	Zfyve28	Ap5m1
Rmdn2	Fgf2	Lrrn3
Serbp1	Hook3	Slc2a13
Tmem258	Mxd4	BC031181
Abt1	Matr3	Zdhhc23
Cracd	Spred3	Lzts1
Gadd45a	Clic4	Skp1
Sec24a	Zfp692	Polr1h

Ppp1r11	Pomk	Srp72
Gng12	Mex3c	Qsox2
Cep135	Aagab	Kidins220
Matcap2	Dzank1	Igsf10
Pcdh8	Hgsnat	Tmem260
Gpi1	Kin	Ubac1
Tbcc	Rsrp1	P2ry12
Siah2	Bltp1	Csgalnact1
Exoc1	Gemin5	P2ry13
Npy1r	Tmem97	Rmdn2
Calm2	Galnt6	Serbp1
Thap11	Stag1	Tmem258
Hif1an	Tbcel	Abt1
Arhgap26	Ldlrap1	Cracd
Wtip	Lsm1	Gadd45a
Elf1	Ttc13	Sec24a
Megf11	Man1c1	Ppp1r11
Marchf1	Tacc3	Gng12
Tbc1d24	Jade3	Cep135
Btg1	Bag4	Matcap2
Rnf39	Bbs7	Pcdh8
Eea1	Capn15	Gpi1
Fam13b	Larp1	Tbcc
Tmem255a	Fam53a	Siah2
Rnf13	Slc9a7	Exoc1
Phpt1	Taf2	Npy1r
Cdh8	Slc12a9	Calm2
Commd2	Paqr7	Thap11
Ppfibp2	Nudt22	Lum
Sbf1	Actr1b	Hif1an
Cdc42ep3	Letmd1	Arhgap26
Slc38a7	Uvssa	Wtip
Rnf113a1	Sf3b6	Elf1
Adamts2	Ccn3	Megf11
Cnot1	Letm2	Marchf1
Ermard	Srrt	Tbc1d24
Iqce	Pafah2	Btg1
Lgi4	Kdm6a	Rnf39
Ppp6r2	Enpp1	Eea1
Ndrg4	Ctbp1	Fam13b
Ttyh3	Trmt6	Tmem255a
Bicral	Rims2	Rnf13
Fxyd1	Rcor3	Phpt1
Upf3b	Atp11b	Cdh8
Fxyd7	Cnnm4	Commd2
Spart	Tbc1d2b	Ppfibp2
Fgf1	Serpine1	Sbf1
Arhgap21	Ranbp10	Cdc42ep3
Chst12	Dmxl1	Slc38a7
Plxnb2	Endod1	Rnf113a1
Eepd1	Depdc5	Adamts2
Eipr1	Vgf	Cnot1
Rfxap	Slc30a1	Ermard

Etl4	Cep85	Sh3tc1
Mgat4b	Arid5a	Iqce
Atp13a2	Slc18b1	Lgi4
Alg5	Azin1	Ppp6r2
Mag	Ints7	Ndrg4
Clcn7	Fbxo27	Ttyh3
Nudt1	Klf10	Bicral
Ccdc148	Uggt1	Fxyd1
Tbc1d9b	Thoc2	Upf3b
Man1b1	Asxl2	Fxyd7
Dennd3	Ubr5	Spart
Tcaf1	Slc2a12	Fgf1
Cenpt	Zmat4	Arhgap21
Tmtc3	Cib2	Chst12
Aaas	Nenf	Plxnb2
Cc2d1a	Fam168b	Eepd1
Nop14	Arhgef4	Eipr1
Ago2	Pank2	Rfxap
Zcchc12	Ppfia1	Etl4
Cab39	Mavs	Mgat4b
Cyld	Bcdin3d	Atp13a2
Micall2	Atg14	Alg5
Zscan12	Mrpl47	Mag
Rbm42	Rapgef6	Clcn7
Oxsr1	Fbxo34	Nudt1
Ttll7	Shank2	Ccdc148
Cuedc2	Plekhg2	Tbc1d9b
Cox6b1	Zdhhc18	Man1b1
Tubb4b	Rps16	Dennd3
Kcnk9	Mcrs1	Tcaf1
Dnajc12	Tob1	Cenpt
Dner	Kcnh3	Tmtc3
Wdr44	Clba1	Aaas
Decr2	Nme1	Cc2d1a
Anln	Adgrl3	Nop14
Tent4b	Osbpl5	Ago2
Rps27l	Bclaf1	Zcchc12
Klhl13	Spag1	Cab39
Slitrk2	Wdtc1	Cyld
Mbd5	Kcnk2	Micall2
Fam135b	Cldn11	Zscan12
Cnep1r1	Rgs22	Rbm42
Slc6a20a	Zfp60	Oxsr1
Dpp10	Prkci	Ttll7
Sun1	Vps13b	Cuedc2
Jmjd4	H2-DMa	Cox6b1
Amdhd2	Phc3	Tubb4b
Topors	Kctd8	Kcnk9
Ssx2ip	Slc20a2	Dnajc12
Pnpla7	Cdkn1c	Dner
Plch1	Ldah	Wdr44
Psenen	Rfx7	Decr2
Siah1a	Inf2	Anln

Lin37	Esyt3	Tent4b
Mrpl41	Armc3	Rps27l
Wnt4	Atp8a1	Klhl13
Mrpl55	Ahdc1	Slitrk2
Dchs1	Ddhd1	Mbd5
Syde2	Lzts3	Fam135b
Proser3	Cd81	Cnep1r1
2410004B18Rik	Fam13a	Slc6a20a
Phkb	Cisd1	Dpp10
Acaa2	Fermt2	Sun1
Arhgap33	Tmem33	Jmjd4
Arhgef26	Gnpnat1	Amdhd2
C1qa	Mynn	Topors
Gtdc1	2810032G03Rik	Ssx2ip
Ehmt1	Limch1	Pnpla7
Rap2b	Mrps26	Plch1
C1qc	Eef1a1	Psenen
Zfp157	Phyhipl	Siah1a
Neto2	Fam222b	Lin37
Fzd8	Xkr8	Mrpl41
C1qb	Ppp1r16b	Wnt4
C1ql2	Actr5	Mrpl55
Unc93b1	Slc32a1	Dchs1
Trim67	Mrpl23	Syde2
Kirrel2	Pced1a	Proser3
Zfp280c	Dzip1l	2410004B18Rik
Ttc7	Coa8	Phkb
Stox1	Vopp1	Acaa2
Nfkbid	Phf12	Arhgap33
Aifm1	N4bp2	Arhgef26
4921524J17Rik	Rpl10a	C1qa
Kdm1a	Fam76b	Gtdc1
Elac1	D630003M21Rik	Ehmt1
Rab8b	Ctnna1	Rap2b
Trappc14	Fbxw17	C1qc
Slc39a12	Abhd18	Zfp157
Kifbp	Tgm2	Neto2
Lrfn3	Smim14	Fzd8
Bcorl1	Tspan14	C1qb
Trim17	Ppm1k	C1ql2
Spryd3	Vstm2l	Unc93b1
Cnpy4	Fdxacb1	Trim67
Tmem177	Rtkn2	Kirrel2
Anapc10	Iars1	Zfp280c
Taf6	Cpe	Ttc7
Tfb1m	Zfp365	Stox1
Zdhhc9	Nufip2	Aifm1
Pml	Egr2	4921524J17Rik
Trim3	Ackr1	Kdm1a
Otud4	Jmjd1c	Elac1
Nxt1	Stk35	Rab8b
Asap3	Dusp8	Trappc14
Zfp39	Wdr19	Slc39a12

Tns2	Pcdh18	Kifbp
Zfp113	H2az1	Lrfn3
Apln	Rcor1	Bcorl1
Hk1	Sirpa	Trim17
Ss18	Ankrd9	Spryd3
Sstr4	Bri3bp	Cnpy4
Zscan21	Ankrd13b	Zic4
Mmaa	Ndufv1	Tmem177
Zfp146	Ssh2	Anapc10
Tspan15	Bicd2	Taf6
Apbb1	Smarce1	Tfb1m
Inhbb	Scarb1	Zdhhc9
Smpd1	Chchd5	Pml
Paip2	Inpp4b	Trim3
Cavin3	Fgd3	Otud4
Sh3glb1	Ano10	Nxt1
Rbmxl1	Wdr20	Asap3
Scd1	Nsrp1	Zfp39
Selenof	Card19	Tns2
Rnf139	Zc3h7a	Zfp113
Trmt12	Ninj1	Apln
Slc35b2	Cfap68	Hk1
Rab11fip3	Snn	Ss18
Dcaf15	6430571L13Rik	Sstr4
Socs5	Ccdc92	Frem2
Zcwpw1	Wnk2	Zscan21
Ralgapa2	Sh3rf3	Mmaa
Setd7	Rara	Zfp146
Sik2	Dhx38	Tspan15
Fam91a1	Igsf9	Apbb1
Psd	Slc24a2	Inhbb
Prmt9	Parp11	Smpd1
Aff3	Arap2	Paip2
Cfap61	Acd	Cavin3
Arhgap10	Cramp1	Sh3glb1
Ddx1	Hpf1	Rbmxl1
Lrrc20	Acer2	Scd1
Ndufc1	Dnah10	Selenof
Ppp1r14a	Wipf2	Rnf139
Mycn	Fam120a	Trmt12
Dennd11	Rapgefl1	Slc35b2
Elf2	Mindy4	Rab11fip3
Krt80	Atp6v0a2	Dcaf15
Cyb561d2	Dennd4c	Socs5
Pacrg	Phf2	Zcwpw1
Rbm17	Kcnj9	Ralgapa2
Atg101	Tigar	Setd7
Islr	Igsf8	Sik2
Fam193a	Gcc2	Fam91a1
Spry1	Ptpdc1	Psd
Thap1	Mrm3	Prmt9
Lipt1	Haus6	Aff3
Syn1	Cntnap5c	Arhgap10

Mospd3
Zfyve28
Fgf2
Hook3
Mxd4
Matr3
Spred3
Clic4
Zfp692
Pomk
Mex3c
Aagab
Dzank1
Hgsnat
Kin
Rsrp1
Bltp1
Gemin5
Tmem97
Ovol2
Galnt6
Stag1
Tbcel
Lsm1
Ttc13
Man1c1
Tacc3
Jade3
Bag4
Tap1
Bbs7
Capn15
Larp1
Fam53a
Slc9a7
Taf2
Slc12a9
Chst7
Paqr7
Nudt22
Actr1b
Letmd1
Uvssa
Sf3b6
Ccn3
Letm2
Srrt
Pafah2
Kdm6a
Ctbp1
Trmt6
Rims2
Rcor3
Dexi
Kmt2c
Nod1
Smim3
Dlec1
Mturn
Rnf144b
Cdkn2aip
Cntln
Galnt11
Fkbp14
Kcna6
Tmem237
Kdm1b
Opa1
Atp13a4
Sbno1
Trappc11
AW551984
Ano2
Phf20
Cdon
Fam210a
Tbc1d32
Mphosph9
Ccdc50
Camk4
Rbm24
Tmem181a
Stox2
Snrk
Notch3
Ormdl3
Spon1
Atg5
Pde4dip
Ttc39b
Enpp6
Hycc2
Mylip
Slc43a2
Chpf2
Btbd10
Prkab2
Fbxo8
Pgap3
Mfsd14b
Tapbpl
Bend3
Cep44
Primpol
Gask1a
F11r
Ddx1
Lrrc20
Ndufc1
Ppp1r14a
Mycn
Dennd11
Elf2
Cyb561d2
Pacrg
Rbm17
Atg101
Islr
Fam193a
Spry1
Thap1
Lipt1
Syn1
Mospd3
Zfyve28
Fgf2
Hook3
Mxd4
Matr3
Spred3
Clic4
Zfp692
Pomk
Mex3c
Aagab
Dzank1
Hgsnat
Kin
Rsrp1
Bltp1
Gemin5
Tmem97
Galnt6
Stag1
Tbcel
Ldlrap1
Lsm1
Ttc13
Man1c1
Tacc3
Jade3
Bag4
Tap1
Bbs7
Capn15
Larp1
Fam53a
Slc9a7
Taf2

Atp11b	Pdss2	Slc12a9
Cnnm4	Cep250	Chst7
Tbc1d2b	Mical2	Paqr7
Ranbp10	Sobp	Nudt22
Dmxl1	Usp38	Actr1b
Endod1	Ncapd2	Letmd1
Depdc5	Bcl9	Uvssa
Vgf	Glra3	Sf3b6
Slc30a1	Trpm4	Ccn3
Cep85	Sema7a	Letm2
Arid5a	Slc22a23	Srrt
Slc18b1	Ovca2	Pafah2
Azin1	Iffo1	Kdm6a
Ints7	Fau	Enpp1
Fbxo27	Nop2	Ctbp1
Klf10	Ostm1	Trmt6
Tedc1	Bphl	Rims2
Uggt1	Smg6	Rcor3
Dtl	Snx25	Atp11b
Thoc2	Atg9b	Icam1
Asxl2	Galnt18	Htra4
Ubr5	Wdr36	Cnnm4
Slc2a12	Snx10	Tbc1d2b
Zmat4	Afg1l	Ranbp10
Cib2	Spats2l	Dmxl1
Nenf	Edem2	Endod1
Fam168b	Kcnh2	Depdc5
Arhgef4	1700066M21Rik	Vgf
Pank2	Trpc4ap	Slc30a1
Ppfia1	Sesn1	Cep85
Mavs	Tsr1	Arid5a
Bcdin3d	Mlxip	Slc18b1
Atg14	Txlng	Azin1
Mrpl47	Zfp384	Ints7
Rapgef6	Plcl1	Fbxo27
Fbxo34	Sgsm2	Klf10
Shank2	Ankrd35	Uggt1
Plekhg2	Fbxo25	Thoc2
Zdhhc18	Lasp1	Asxl2
Rps16	Focad	Ubr5
Vash2	Ncoa6	Slc2a12
Mcrs1	Pcp4l1	Zmat4
Wdhd1	Sbf2	Cib2
Tob1	Gmds	Nenf
Kcnh3	Rbm8a	Fam168b
Rskr	Trp53inp2	Arhgef4
Clba1	Pigu	Pank2
Nme1	Setd1b	Ppfia1
Adgrl3	Rras	Mavs
Osbpl5	Pals2	Bcdin3d
Bclaf1	Gpr162	Atg14
Kcnmb2	Txnip	Mrpl47
Spag1	Upf3a	Rapgef6

Wdtc1	Pmepa1	Fbxo34
Kcnk2	Scaf1	Shank2
Cldn11	Higd1a	Plekhg2
Zfp60	Cdc16	Zdhhc18
Prkci	Fig4	Rps16
Vps13b	Egr1	Vash2
H2-DMa	Hdhd3	Mcrs1
Phc3	Poli	Wdhd1
Kctd8	Usp5	Tob1
Slc20a2	Mllt6	Kcnh3
Cdkn1c	Cdc40	Rskr
Ldah	Srcin1	Clba1
Rfx7	Dennd2a	Nme1
Inf2	Abhd17c	Adgrl3
Atp8a1	Uqcrfs1	Osbpl5
Ahdc1	Chmp4b	Bclaf1
Ddhd1	Nos1ap	Kcnmb2
Lzts3	Cdk19	Spag1
Cd81	Tfdp1	Wdtc1
Spag6	Socs7	Kcnk2
Fam13a	Sv2a	Cldn11
Cisd1	Polr2l	Zfp60
Fermt2	Otud7b	Prkci
Tmem33	Tmco3	Vps13b
Gnpnat1	Prr3	H2-DMa
Mynn	Ptov1	Phc3
2810032G03Rik	Mesd	Kctd8
Limch1	Dcun1d2	Slc20a2
Mrps26	Parp12	Cdkn1c
Eef1a1	Rpf2	Ldah
Phyhipl	Grtp1	Rfx7
Fam222b	Tbkbp1	Inf2
Xkr8	Jarid2	Atp8a1
Ppp1r16b	Tbc1d17	Aspg
Actr5	Fchsd1	Ahdc1
Slc32a1	Armc10	Ddhd1
Mrpl23	Car14	Lzts3
Pced1a	Rgs4	Cd81
Dzip1l	Cbfa2t2	Spag6
Coa8	Osbpl7	Fam13a
Vopp1	Zfp280d	Cisd1
Phf12	Ubn2	Fermt2
N4bp2	Atf5	Tmem33
Rpl10a	Pcid2	Gnpnat1
Fam76b	Inip	Mynn
D630003M21Rik	Cul7	2810032G03Rik
Ctnna1	Ranbp9	Limch1
Fbxw17	Ciart	Mrps26
Abhd18	Fndc4	Eef1a1
Tgm2	Reep2	Phyhipl
Smim14	Efl1	Fam222b
Tspan14	Ift172	Xkr8
Ppm1k	Rad9b	Ppp1r16b

Sh2d4b	Susd4	Actr5
Vstm2l	Susd1	Slc32a1
Fdxacb1	Pptc7	Mrpl23
Rtkn2	Akap12	Pced1a
Nmrk1	Tctn1	Dzip1l
Iars1	Cep85l	Coa8
Cpe	Slc35f1	Vopp1
Zfp365	Ripor1	Phf12
Nufip2	Samd10	N4bp2
Egr2	Gng10	Rpl10a
Ackr1	Dock10	Fam76b
Jmjd1c	Phrf1	D630003M21Rik
Stk35	Mcl1	Ctnna1
Dusp8	Nfe2l1	Fbxw17
Wdr19	Ensa	Abhd18
Pcdh18	Med30	Tgm2
H2az1	Tm6sf1	Smim14
Rcor1	Polr3k	Tspan14
Sirpa	Zkscan16	Ppm1k
Ankrd9	Degs1	Vstm2l
Bri3bp	Lrrc56	Fdxacb1
Ankrd13b	Ctss	Rtkn2
Ndufv1	Pold1	Nmrk1
Ssh2	Ramac	Iars1
Bicd2	Creb3l2	Cpe
Smarce1	Rnh1	Zfp365
Scarb1	Ric1	Nufip2
Chchd5	Herc1	Ackr1
Inpp4b	Dgki	Jmjd1c
Fgd3	Lpar1	Stk35
Ano10	Arfrp1	Dusp8
Wdr20	Scube3	Wdr19
Nsrp1	Trps1	Pcdh18
Card19	Pak1ip1	H2az1
Zc3h7a	Rtel1	Rcor1
Ninj1	Atp5mf	Sirpa
Cfap68	Josd2	Ankrd9
Snn	Mapkap1	Bri3bp
Itprid1	Taf5l	Ankrd13b
6430571L13Rik	Dsel	Ndufv1
Ccdc92	Gmeb2	Ssh2
Neurod6	Golga4	Bicd2
Wnk2	Mindy1	Smarce1
Sh3rf3	Atp5mg	Scarb1
Rara	Pbx3	Chchd5
Dhx38	Bud31	Inpp4b
Slc9b2	Pakap	Fgd3
Igsf9	Wdr26	Ano10
Slc24a2	Nudcd1	Wdr20
Parp11	Shank1	Nsrp1
Arap2	Mvb12b	Card19
Acd	Angptl6	Zc3h7a
Cramp1	Nup205	Ninj1

Hpf1	Trhr	Cfap68
Acer2	Abcf1	Snn
Ccdc138	Ptpn3	6430571L13Rik
Wipf2	Gabpb2	Ccdc92
Fam120a	Kdm3b	Neurod6
Rapgefl1	Ascc3	Wnk2
Mindy4	Ephx1	Sh3rf3
Atp6v0a2	Sema6c	Rara
Dennd4c	Smurf1	Dhx38
Phf2	Cnot4	Igsf9
Kcnj9	Zscan2	Slc24a2
Tigar	Ost4	Parp11
Igsf8	Six3	Arap2
Gcc2	Sde2	Acd
Ptpdc1	Rap1gap2	Cramp1
Mrm3	Trmt112	Hpf1
Haus6	Ctnnal1	Acer2
Cntnap5c	Hace1	Ccdc138
Dexi	Abitram	Wipf2
Kmt2c	Tmem214	Fam120a
Nod1	Ralgps1	Rapgefl1
Smim3	Agbl3	Mindy4
Dlec1	Vars2	Atp6v0a2
Mturn	Gcnt1	Dennd4c
Rnf144b	Kif16b	Phf2
Cdkn2aip	Phb1	Kcnj9
Cntln	Ythdf1	Tigar
Galnt11	Itpkb	Igsf8
Fkbp14	Garnl3	Gcc2
Kcna6	Pi4kb	Ptpdc1
Tmem237	Zcchc2	Mrm3
Kdm1b	Bpgm	Haus6
Opa1	Zfhx3	Cntnap5c
Cnbd2	Rnf146	Dexi
Atp13a4	Nipal2	Kmt2c
Sbno1	Mrps34	Nod1
Trappc11	Shfl	Smim3
AW551984	Man2a2	Cldn22
Phf20	Ctu1	Mturn
Cdon	Fam117a	Rnf144b
Fam210a	Irs2	Cdkn2aip
Tbc1d32	Zfp653	Cntln
Mphosph9	Rpl12	Galnt11
Ccdc50	Pogz	Fkbp14
Camk4	Kat7	Kcna6
Rbm24	Plcl2	Tmem237
Tmem181a	Dido1	Kdm1b
Stox2	Mtcl3	Opa1
Snrk	Rccd1	Cnbd2
Notch3	Sccpdh	Atp13a4
Ormdl3	Cnst	Sbno1
Spon1	Supt3	Trappc11
Atg5	Edc3	Ano2

P3h2	Ube2l3	Phf20
Pde4dip	Pdk2	Cdon
Ttc39b	Lmtk2	Fam210a
Enpp6	Rabggtb	Tbc1d32
Hycc2	Ppp1r9b	Mphosph9
Mylip	Bloc1s5	Ccdc50
Slc43a2	Tspyl5	Camk4
Spag4	Cables2	Rbm24
Chpf2	Txndc5	Tmem181a
Btbd10	Ube3c	Stox2
Prkab2	Rps21	Snrk
Fbxo8	Bmp6	Notch3
Pgap3	Cpq	Ormdl3
Mfsd14b	Timm8b	Spon1
Tapbpl	Mtg1	Atg5
Bend3	Arhgap18	P3h2
Cep44	Tasp1	Pde4dip
Primpol	St6galnac5	Ttc39b
Gask1a	Adrm1	Enpp6
F11r	Arpin	Hycc2
Pdss2	Usp6nl	Mylip
Cep250	Pigk	Slc43a2
Mical2	Foxred1	Chpf2
Sobp	Osbpl2	Btbd10
Usp38	Myo16	Prkab2
Ncapd2	Ak5	Fbxo8
Neurod2	Hrh3	Pgap3
Bcl9	Atpsckmt	Mfsd14b
Glra3	Psmd7	Tapbpl
Trpm4	Zzz3	Bend3
Sema7a	Mtg2	Cep44
Slc22a23	Zfp503	Primpol
Ovca2	Ss18l1	Gask1a
Iffo1	Rreb1	F11r
Fau	L3mbtl3	Pdss2
Nop2	Rsad1	Cep250
Ostm1	Marchf6	Mical2
Bphl	Atp6v1g1	Sobp
Smg6	Htr5a	Usp38
Snx25	Lsm14b	Ncapd2
Kash5	Mycbpap	Neurod2
Atg9b	Nrn1	Bcl9
Galnt18	Itga9	Glra3
Wdr36	Taf4	Trpm4
Snx10	Prune2	Sema7a
Afg1l	Cdc123	Slc22a23
Spats2l	Zc3hc1	Ovca2
Edem2	Whrn	Iffo1
Kcnh2	Camk1d	Fau
1700066M21Rik	Sart1	Nop2
Trpc4ap	Shd	Ostm1
Satb2	Stim2	Bphl
Sesn1	Eeig1	Smg6

Tsr1	Akna	Snx25
Mlxip	Ube2h	Kash5
Txlng	Cmc1	Atg9b
Zfp384	Naif1	Galnt18
Tcte2	Akap7	Wdr36
Plcl1	Adgrl4	Snx10
Sgsm2	Polg	Afg1l
Ankrd35	Tbc1d19	Spats2l
Fbxo25	Tekt5	Edem2
Lasp1	AW209491	Kcnh2
Focad	Nubp2	1700066M21Rik
Ncoa6	Rbpj	Trpc4ap
Pcp4l1	Rlbp1	Sesn1
Sbf2	Bbln	Tsr1
Gmds	Adk	Mlxip
Rbm8a	Zdhhc1	Txlng
Trp53inp2	Tbc1d25	Zfp384
Pigu	Abhd2	Tcte2
Setd1b	Ciz1	Plcl1
Rras	Daglb	Sgsm2
Pals2	Metrnl	Ankrd35
Gpr162	Gpatch2	Fbxo25
Txnip	Il18	Lasp1
Upf3a	Srrm2	Focad
Pmepa1	Arid4b	Ncoa6
Foxf2	Ppp1r10	Pcp4l1
Scaf1	Rpl22l1	Sbf2
Higd1a	Tbcd	Gmds
Foxq1	Suv39h1	Rbm8a
Cdc16	Tbce	Trp53inp2
Fig4	Sec24d	Pigu
Egr1	Tgfb2	Setd1b
Hdhd3	B3galnt2	Rras
Poli	Ints15	Pals2
Usp5	Lyplal1	Gpr162
Mllt6	Lgi2	Txnip
Cdc40	Fn3krp	Upf3a
Srcin1	Pomt1	Pmepa1
Dennd2a	Vstm2b	Foxf2
Tmem255b	Prrc2b	Scaf1
Abhd17c	Npepl1	Higd1a
Uqcrfs1	Megf9	Foxq1
Olfml2b	Foxk2	Cdc16
Chmp4b	Pcsk1n	Fig4
Nos1ap	Azi2	Egr1
Cdk19	Fndc3b	Hdhd3
Tfdp1	Cybc1	Poli
Socs7	Cdk5rap2	Usp5
Sv2a	Hexd	Mllt6
Polr2l	Ndst2	Cdc40
Otud7b	Minar1	Spsb2
Tmco3	Rftn1	Srcin1
Prr3	Rab3gap2	Dennd2a

Ptov1	Igfbp2	Abhd17c
Mesd	Rnf122	Uqcrfs1
Dcun1d2	Mettl22	Olfml2b
Parp12	Atp6v0e2	Chmp4b
Rpf2	C130074G19Rik	Nos1ap
Grtp1	Smarcal1	Cdk19
Tbkbp1	Exosc2	Tfdp1
Jarid2	Fut11	Socs7
Tbc1d17	Picalm	Sv2a
Fchsd1	Sec24c	Polr2l
Armc10	Marchf4	Otud7b
Car14	Wdr17	Tmco3
Rgs4	Wdr45	Prr3
Cbfa2t2	Dusp10	Ptov1
Osbpl7	Cdh6	Mesd
Zfp280d	Prss23	Dcun1d2
Ubn2	Prdm16	Parp12
Atf5	Heatr5b	Rpf2
Pcid2	Cntnap2	Grtp1
Inip	Alg1	Tbkbp1
Cul7	Tmem135	Jarid2
Ranbp9	Mtmr7	Tbc1d17
Ciart	Prpf18	Fchsd1
Fndc4	Dcxr	Armc10
Reep2	Snx22	Car14
Efl1	Morc3	Rgs4
Ift172	Mtmr12	Cbfa2t2
Rad9b	Tcta	Osbpl7
Saxo2	Slc9a8	Zfp280d
Susd4	Zdhhc2	Ubn2
Susd1	Ubn1	Atf5
Pptc7	Wfs1	Pcid2
Akap12	Tnrc18	Inip
Tctn1	Micu3	Cul7
Cep85l	Asb6	Ranbp9
Slc35f1	Tspyl4	Ciart
Ripor1	Cntn5	Fndc4
Samd10	Znfx1	Reep2
Gng10	Nup133	Efl1
Dock10	Bltp3a	Ift172
Phrf1	Ptpa	Rad9b
Mcl1	Cyp7b1	Susd4
Nfe2l1	Cep104	Susd1
Ensa	Zup1	Pptc7
Med30	Mmd2	Akap12
Tm6sf1	Stau1	Tctn1
Polr3k	Sgcz	Cep85l
Zkscan16	Ncam1	Slc35f1
Degs1	Ajap1	Ripor1
Lrrc56	Rsph4a	Samd10
Ctss	Ppp1r3f	Gng10
Pold1	Ubald1	Dock10
Ramac	Nphp4	Phrf1

Creb3l2
Rnh1
Ric1
Herc1
Dgki
Lpar1
Arfrp1
Trps1
Pak1ip1
Rtel1
Atp5mf
Josd2
Mapkap1
Taf5l
Dsel
Gmeb2
Golga4
Mindy1
Atp5mg
Pbx3
Bud31
Pakap
Wdr26
Nudcd1
Shank1
Mvb12b
Angptl6
Nup205
Trhr
Abcf1
Ptpn3
Gabpb2
Kdm3b
Ascc3
Ephx1
Sema6c
Smurf1
Cnot4
Zscan2
Ost4
Sde2
Rap1gap2
Trmt112
Ctnnal1
Hace1
Abitram
Tmem214
Ralgps1
Agbl3
Vars2
Kif16b
Phb1
Ythdf1
Ccser1
Grin3a
Myo9a
Fam149b
Rcan2
Rbms3
Pgap4
Stub1
Trmt9b
Prex1
Ap5z1
Strip2
Hnrnpu
Odad3
Lonrf1
Zfp189
Coro7
Mrpl12
Vasn
Kyat1
Cpeb3
Rxrb
Spout1
Dusp26
Icmt
Oxld1
Zmynd8
Tbc1d13
Mrps6
Lap3
Sdk1
Zer1
Msantd3
Ncoa7
Usp53
Nploc4
Lmbrd2
Ldb2
Plekhg5
Dync2i2
Dock3
Ralyl
Trp53rka
Fnbp1l
Prkrip1
Slx4
Alg2
Bahcc1
Htatip2
Orai2
Fbxl5
Alkbh4
Dnttip2
Mcl1
Nfe2l1
Ensa
Med30
Tm6sf1
Polr3k
Zkscan16
Degs1
Lrrc56
Ctss
Pold1
Ramac
Creb3l2
Rnh1
Ric1
Herc1
Dgki
Lpar1
Arfrp1
Trps1
Pak1ip1
Rtel1
Atp5mf
Josd2
Mapkap1
Taf5l
Dsel
Gmeb2
Golga4
Mindy1
Atp5mg
Pbx3
Bud31
Pakap
Wdr26
Nudcd1
Shank1
Mvb12b
Angptl6
Nup205
Trhr
Abcf1
Ptpn3
Gabpb2
Kdm3b
Ascc3
Ephx1
Sema6c
Smurf1
Cnot4
Zscan2
Ost4
Sde2

Itpkb	Thap3	Rap1gap2
Garnl3	Dnajc28	Trmt112
Pi4kb	Cc2d2a	Ctnnal1
Zcchc2	Dnajc11	Hace1
Bpgm	Ypel5	Abitram
Zfhx3	Polr2j	Tmem214
Rnf146	Cep131	Ralgps1
Nipal2	Cpeb2	Agbl3
Mrps34	Cercam	Vars2
Shfl	Zfp597	Gcnt1
Man2a2	Zfand1	Kif16b
Ctu1	Cplane1	Phb1
Fam117a	Ncoa5	Ythdf1
Irs2	Gabbr2	Itpkb
Zfp653	Zc3h10	Garnl3
Rpl12	Myl6b	Pi4kb
Pogz	Trub2	Zcchc2
Kat7	Wdr70	Bpgm
Plcl2	Olig2	Zfhx3
Dido1	Arhgap29	Rnf146
Mtcl3	Zfp335	Nipal2
Rccd1	Nhsl1	Mrps34
Sccpdh	Slc45a1	Shfl
Cnst	Epg5	Man2a2
Supt3	Zfp800	Ctu1
Edc3	Mcph1	Fam117a
Ube2l3	Rapgef1	Irs2
Pdk2	Pcif1	Zfp653
Lmtk2	Endov	Rpl12
Rabggtb	Rere	Pogz
Ppp1r9b	Srrm3	Kat7
Bloc1s5	Heca	Plcl2
Tspyl5	Tmem120a	Dido1
Cables2	Alg14	Mtcl3
Txndc5	Armh3	Rccd1
Ube3c	Gpr37	Sccpdh
Rps21	Slc26a11	Cnst
Bmp6	Cited2	Supt3
Cpq	Spsb1	Edc3
Timm8b	Pak5	Ube2l3
Mtg1	Coq10a	Pdk2
Arhgap18	Rhbdd2	Lmtk2
Tasp1	Urb1	Rabggtb
St6galnac5	Gsap	Ppp1r9b
Adrm1	Pik3cd	Bloc1s5
Arpin	Plcb4	Tspyl5
Usp6nl	Dag1	Cables2
Pigk	Clstn1	Txndc5
Foxred1	Stk32a	Ube3c
Osbpl2	Hip1	Rps21
Myo16	Rhou	Bmp6
Ak5	Ccdc40	Cpq
Hrh3	Zfp292	Timm8b

Anpep	Rsbn1l	Mtg1
Atpsckmt	Tbc1d16	Arhgap18
Psmd7	Dtx4	Tasp1
Zzz3	Ccdc32	St6galnac5
Mtg2	Phtf2	Adrm1
Trhr2	Ankrd13c	Arpin
Ss18l1	Cbx4	Usp6nl
Rreb1	Edrf1	Pigk
L3mbtl3	Timeless	Foxred1
Rsad1	Magi2	Osbpl2
Marchf6	Ginm1	Myo16
Atp6v1g1	Bahd1	Ak5
Htr5a	Gnaz	Hrh3
Lsm14b	Slc7a5	Atpsckmt
Nrn1	Cox15	Psmd7
Itga9	Lats1	Zzz3
Adgrg6	Rab11fip2	Mtg2
Taf4	Ythdf2	Zfp503
Prune2	Elavl1	Ss18l1
Cdc123	Ipo8	Rreb1
Zc3hc1	Stat2	L3mbtl3
Whrn	Disp2	Rsad1
Camk1d	Negr1	Marchf6
Sart1	Ift88	Atp6v1g1
Runx2	Rbms2	Htr5a
Shd	Orc3	Lsm14b
Stim2	Ndufb10	F13a1
Eeig1	Baz2a	Nrn1
Akna	Gjb6	Itga9
Ube2h	Flywch1	Taf4
Cmc1	Klhl42	Prune2
Naif1	Plpp6	Cdc123
Akap7	Gramd1b	Zc3hc1
Adgrl4	Mrps35	9330171B17Rik
Polg	Cacna2d1	Whrn
Tbc1d19	Zmym5	Camk1d
Tekt5	Gorab	Sart1
AW209491	Gpr26	Runx2
Nubp2	Pnrc1	Shd
Rbpj	Gpr176	Stim2
Rlbp1	Abcc8	Eeig1
Bbln	Rgl3	Akna
Adk	Maob	Ube2h
Zdhhc1	Hs2st1	Cmc1
Tbc1d25	Thbs1	Naif1
Abhd2	Tax1bp3	Akap7
Ciz1	Kcns1	Adgrl4
Daglb	Ikzf5	Dap
Metrnl	Alkbh3	Polg
Gpatch2	2310057M21Rik	Tbc1d19
Il18	Fmo1	Tekt5
Srrm2	Ankrd6	AW209491
Arid4b	Scamp2	Nubp2

Ppp1r10	Odad1	Rbpj
Rpl22l1	Nemp1	Rlbp1
Tbcd	Zfp704	Bbln
Suv39h1	Kyat3	Adk
Tbce	Ttc12	Zdhhc1
Sec24d	Gas8	Tbc1d25
Tgfb2	Prrc2c	Abhd2
B3galnt2	Tm7sf3	Ciz1
Ints15	Trappc5	Daglb
Lyplal1	Fgfr1op2	Metrnl
Lgi2	Lrp1	Gpatch2
Fn3krp	Ints13	Il18
Pomt1	Sema3d	Srrm2
Vstm2b	Daam2	Arid4b
Prrc2b	Klhdc4	Ppp1r10
Npepl1	Dnm3	Rpl22l1
Megf9	Plekha1	Tbcd
Foxk2	Mrps28	Suv39h1
Pcsk1n	Bach2	Tbce
Azi2	Cdk6	Sec24d
Fndc3b	Pacsin1	Tgfb2
Cybc1	Cdin1	B3galnt2
Cdk5rap2	Hey1	Ints15
Hexd	Suco	Lyplal1
Ndst2	Rbm48	Lgi2
Minar1	Zfp770	Fn3krp
Rftn1	Dcaf1	Pomt1
Rab3gap2	Cul9	Vstm2b
Igfbp2	Nsmce4a	Prrc2b
Rnf122	Eeig2	Npepl1
Mettl22	Ankib1	Megf9
Atp6v0e2	Mars1	Foxk2
C130074G19Rik	Skic2	Pcsk1n
Smarcal1	Ufl1	Azi2
Exosc2	Bcor	Fndc3b
Fut11	Trim41	Cybc1
Drd5	Etfrf1	Cdk5rap2
Picalm	Gpr63	Hexd
Sec24c	Cacng5	Ndst2
Marchf4	Pex2	Minar1
Wdr17	Aqr	Rftn1
Wdr45	Ppp1ca	Rab3gap2
Dusp10	Klhl32	Igfbp2
Cdh6	Wdr47	Rnf122
Mreg	Map3k10	Mettl22
Prss23	Abhd13	Atp6v0e2
Prdm16	Akap9	C130074G19Rik
Heatr5b	Fbxl4	Smarcal1
Cntnap2	Elapor1	Exosc2
Alg1	Slc25a28	Fut11
Tmem135	Dtx3	Drd5
Mtmr7	Cdh18	Picalm
Prpf18	Rc3h1	Sec24c

Dcxr
Snx22
Morc3
Mtmr12
Tcta
Slc9a8
Zdhhc2
Ubn1
Wfs1
Tnrc18
Micu3
Asb6
Tspyl4
Cntn5
Dse
Znfx1
Nup133
Bltp3a
Ptpa
Cyp7b1
Cep104
Zup1
Mmd2
Stau1
Sgcz
Ncam1
Ajap1
Rsph4a
Ppp1r3f
Ubald1
Nphp4
Ccser1
Grin3a
Myo9a
Fam149b
Rcan2
Rbms3
Pgap4
Stub1
Mocos
Trmt9b
Prex1
Ap5z1
Strip2
Hnrnpu
Odad3
Lonrf1
Zfp189
Coro7
Mrpl12
Vasn
Kyat1
Cpeb3
Pitpnc1
Zbtb38
Ppp1r15a
Rprd1a
Spns2
Sgms1
Cdh12
Usp45
Arglu1
Os9
Mybbp1a
Gtpbp10
Blvrb
Rabggta
Cfap69
Dgkz
Bptf
Dxo
Ltbp4
Lrfn2
Chrm4
Marchf9
Ambra1
Manea
Tsfm
Zfp609
Abhd11
Necab1
Adam22
Pitpnm3
Tex2
Ckap5
Otud6b
Mettl27
Wdr7
Plppr2
Btaf1
Rundc3b
Abcb1a
Ofd1
Cstpp1
Ranbp17
Pogk
Mis12
Bace2
Kazn
Ildr2
Tmem51
Pck2
Dhx33
Gemin8
Plekhg1
Dok4
Marchf4
Wdr17
Wdr45
Dusp10
Cdh6
Mreg
Prss23
Prdm16
Heatr5b
Cntnap2
Alg1
Tmem135
Mtmr7
Prpf18
Dcxr
Snx22
Morc3
Mtmr12
Tcta
Slc9a8
Zdhhc2
Ubn1
Wfs1
Tnrc18
Micu3
Asb6
Tspyl4
Cntn5
Dse
Znfx1
Nup133
Bltp3a
Ptpa
Cyp7b1
Cep104
Zup1
Mmd2
Stau1
Ncam1
Ajap1
Ppp1r3f
Ubald1
Nphp4
Ccser1
Grin3a
Myo9a
Fam149b
Rcan2
Rbms3
Pgap4
Stub1
Mocos
Trmt9b

Rxrb	Erc2	Prex1
Spout1	Ppip5k2	Ap5z1
Dusp26	Rimklb	Strip2
Icmt	Tasor	Hnrnpu
Oxld1	Oaz2	Odad3
Zmynd8	Ppp1r14c	Lonrf1
Tbc1d13	Efhd2	Zfp189
Mrps6	Rad54l2	Coro7
Lap3	Nup88	Mrpl12
Sdk1	Phc1	Vasn
Zer1	Mthfd1l	Kyat1
Msantd3	Hmgn1	Cpeb3
Ncoa7	Madd	Rxrb
Usp53	Tbl3	Spout1
Nploc4	Col16a1	Dusp26
Lmbrd2	Dnajc16	Icmt
Ldb2	Limd2	Oxld1
Plekhg5	Cyp2s1	Zmynd8
Cplx3	St8sia4	Tbc1d13
Dync2i2	Sh3pxd2b	Mrps6
Dock3	Camta2	Lap3
Ralyl	Creg1	Sdk1
Trp53rka	Klc3	Zer1
Fnbp1l	Il17rd	Msantd3
Prkrip1	Virma	Ncoa7
Slx4	Zfhx2	Usp53
Alg2	Scamp5	Nploc4
Bahcc1	Rcsd1	Lmbrd2
Htatip2	Kcna2	Ldb2
Orai2	Hnrnpul1	Plekhg5
Fbxl5	Cep126	Dync2i2
Alkbh4	Eif4h	Dock3
Dnttip2	Ppp1r13l	Ralyl
Thap3	Ints8	Trp53rka
Dnajc28	Slc25a34	Fnbp1l
Cc2d2a	Rnf167	Prkrip1
Dnajc11	Siah1b	Slx4
Ypel5	Myh6	Alg2
Polr2j	Cmtm5	Bahcc1
Cep131	Appl1	Htatip2
Cpeb2	Spen	Orai2
Cercam	Snrnp25	Fbxl5
Zfp597	Oard1	Alkbh4
Zfand1	Cept1	Dnttip2
Cplane1	Cop1	Thap3
Ncoa5	Ttc3	Dnajc28
Gabbr2	C1qtnf4	Cc2d2a
Zc3h10	Iqcc	Dnajc11
Tbc1d2	Iqsec3	Ypel5
Myl6b	Eml2	Polr2j
Trub2	Tex264	Cep131
Wdr70	Dennd6a	Cpeb2
Olig2	Hlcs	Cercam

Arhgap29
Zfp335
Nhsl1
Slc45a1
Epg5
Zfp800
Mcph1
Rapgef1
Pcif1
Endov
Rere
Srrm3
Neurl2
Heca
Tmem120a
Alg14
Armh3
Eva1c
Gpr37
Slc26a11
Cited2
Spsb1
Pak5
Coq10a
Rhbdd2
Urb1
Gsap
Pik3cd
Plcb4
Dag1
Clstn1
Etfbkmt
Hip1
Rhou
Zfp292
Rsbn1l
Tbc1d16
Dtx4
Ccdc32
Phtf2
Ankrd13c
Cbx4
Edrf1
Timeless
Magi2
Ginm1
Bahd1
Gnaz
Slc7a5
Ptger3
Cox15
Lats1
Rab11fip2
1700123O20Rik
Snrpd2
Gpr161
Szrd1
Tiprl
Sft2d2
Psme4
Plekhh2
Reps2
Dlk1
Erf
Bsdc1
Crocc
Ino80d
Begain
Osbpl10
Wdr25
Tmem205
Gfer
Kcnd3
Gm21988
Atp1a3
Fbxw4
Slc19a2
S100pbp
Rfx3
Ulk4
Slc16a11
Arhgef1
Tet2
Rcc2
Rps19
Cables1
Arhgef10l
Igsf21
Gm11992
Sh3kbp1
Abhd4
Npnt
Trim62
Nrg3
Map7d2
Iffo2
Ghitm
Dpcd
Irgq
Fam117b
Ramp3
Wdr43
Wwp1
Nacad
Fzd7
Grid1
Zfp597
Zfand1
Cplane1
Ncoa5
Gabbr2
Zc3h10
Tbc1d2
Myl6b
Trub2
Wdr70
Olig2
Arhgap29
Zfp335
Nhsl1
Slc45a1
Epg5
Zfp800
Mcph1
Rapgef1
Pcif1
Endov
Rere
Srrm3
Heca
Tmem120a
Alg14
Fgl2
Armh3
Gpr37
Slc26a11
Cited2
Spsb1
Pak5
Coq10a
Rhbdd2
Urb1
Gsap
Pik3cd
Plcb4
Dag1
Clstn1
Stk32a
Hip1
Rhou
Zfp292
Rsbn1l
Tbc1d16
Dtx4
Ccdc32
Phtf2
Ankrd13c
Cbx4
Edrf1

Ythdf2	Rwdd2b	Timeless
Elavl1	Ostc	Ifi203
Ipo8	Tspyl2	Magi2
Stat2	Elmo1	Ginm1
Nup43	Iqsec2	Bahd1
Disp2	Ccdc8	Gnaz
Negr1	Pde9a	Slc7a5
Ift88	Nbl1	Ptger3
Rbms2	Msantd4	Cox15
Orc3	H2az2	Lats1
Ndufb10	Zfp598	Rab11fip2
Baz2a	N4bp2l1	Ythdf2
Gjb6	Smc1a	Elavl1
Flywch1	Pnma8a	Ipo8
Klhl42	Dnah7b	Stat2
Plpp6	Brca2	Disp2
Gramd1b	Osgin2	Negr1
Mrps35	Zmiz2	Ift88
Cacna2d1	Lonp1	Rbms2
Zmym5	Hectd2	Orc3
Gorab	Prkd2	Ndufb10
Gpr26	Rpusd1	Baz2a
Pnrc1	Trir	Gjb6
Gpr176	Map6d1	Flywch1
Abcc8	Yeats2	Klhl42
Ndp	Clvs1	Plpp6
Rgl3	Elovl6	Gramd1b
Maob	Arhgap12	Mrps35
Hs2st1	Phf8	Cacna2d1
Tax1bp3	Ublcp1	Zmym5
Kcns1	Chd7	Gorab
Ikzf5	Vps41	Gpr26
Alkbh3	Rbbp8	Pnrc1
2310057M21Rik	Mul1	Gpr176
Fmo1	Wnk3	Abcc8
Ankrd6	Zfp236	Ndp
Bmal2	Car8	9430038I01Rik
Scamp2	Rusc1	Rgl3
Nemp1	Uspl1	Maob
Zfp704	Dmxl2	Hs2st1
Kyat3	Tox	Thbs1
Ttc12	Ttc1	Tax1bp3
Gas8	Cdk13	Kcns1
Prrc2c	Katnal1	Ikzf5
Gpr34	Gtf3c3	Alkbh3
Tm7sf3	Sntb2	2310057M21Rik
Trappc5	Nkx6-2	Fmo1
Fgfr1op2	Slc7a1	Ankrd6
Lrp1	Thoc6	Bmal2
Ints13	Ak7	Scamp2
Gbp7	Inhba	Nemp1
Sema3d	Pcf11	Zfp704
Nxph4	Atp1b2	Kyat3

Daam2
Klhdc4
Dnm3
Plekha1
Mrps28
Bach2
Accs
Cdk6
Pacsin1
Cdin1
Hey1
Suco
Rbm48
Cchcr1
Zfp770
Dcaf1
Cul9
Nsmce4a
Eeig2
Ankib1
Mars1
Skic2
Ufl1
Bcor
Trim41
Etfrf1
Gpr63
Cacng5
Pex2
Aqr
Ppp1ca
Klhl32
Wdr47
Map3k10
Abhd13
Akap9
Fbxl4
Elapor1
Slc25a28
Dtx3
Cdh18
Rc3h1
Mterf1a
Pitpnc1
Zbtb38
Ppp1r15a
Rprd1a
Spns2
Sgms1
Cdh12
Usp45
Arglu1
Os9
Atg2b
Ankrd42
Wrap53
Rap1gap
Vcf2
Rgl2
Ssr2
Pum3
Shtn1
Ccdc9
Cldn5
Htr2c
Mettl18
Wapl
Dicer1
Pik3r1
Meis3
Paqr6
Hibch
Nthl1
Utp4
Mfsd6
Arhgap32
Rpl21
Tardbp
Gpr12
Shld2
Dcp1b
Zfp281
Stx3
Cep78
Rrp9
Rnf123
Ago1
Sox5
Disp3
Hspb8
Ptchd1
Fbxo2
Nop53
Camsap2
Selenow
Prelp
Sdk2
Tmtc4
Cdc42ep4
Mbp
Entpd3
Bicdl1
Ccdc74a
Mtnap1
Gucy1a2
Ggact
Gas8
Prrc2c
Gpr34
Tm7sf3
Trappc5
Fgfr1op2
Lrp1
Ints13
Gbp7
Sema3d
Daam2
Klhdc4
Dnm3
Plekha1
Mrps28
Bach2
Accs
Cdk6
Pacsin1
Cdin1
Hey1
Rigi
Suco
Rbm48
Cchcr1
Zfp770
Dcaf1
Cul9
Nsmce4a
Eeig2
Ankib1
Mars1
Skic2
Ufl1
Bcor
Sec1
Trim41
Etfrf1
Gpr63
Cacng5
Pex2
Aqr
Ppp1ca
Klhl32
Wdr47
Map3k10
Abhd13
Akap9
Fbxl4
Elapor1
Slc25a28
Dtx3
Cdh18

Mybbp1a	Vcf1	Rc3h1
Gtpbp10	Mrps27	Mterf1a
Blvrb	Gcn1	Pitpnc1
Rabggta	Kif21b	Zbtb38
Cfap69	Ddx24	Ppp1r15a
Dgkz	Pcca	Slc26a10
Bptf	Slc39a11	Rprd1a
Dxo	Rragb	Spns2
Ltbp4	Bbox1	Sgms1
Lrfn2	Prima1	Cdh12
Chrm4	Rims1	Usp45
Marchf9	Pyroxd1	Arglu1
Ambra1	Fbxl9	Os9
Pvr	Bivm	Mybbp1a
Manea	Fcho2	Gtpbp10
Tsfm	Amot	Blvrb
Zfp609	Kcnj2	Rabggta
Abhd11	Cox6a1	Cfap69
Necab1	Btbd7	Dgkz
Adam22	Zic5	Bptf
Pitpnm3	Mpped1	Dxo
Tex2	Trpc5	Ltbp4
Ckap5	Ubr7	Lrfn2
Otud6b	Gm20604	Chrm4
Mettl27	Pi4ka	Marchf9
Wdr7	Coro2b	Ambra1
Plppr2	Pgm5	Pvr
Btaf1	Coq5	Manea
Rundc3b	Kirrel1	Tsfm
Abcb1a	Rnf10	Zfp609
Ofd1	Pde3a	Abhd11
Cstpp1	Utp15	Necab1
Ranbp17	Plekha6	Adam22
Pogk	Gpr155	Pitpnm3
Mis12	Tpp2	Tex2
Kazn	Ubac2	Ckap5
Ildr2	Ppp2r2d	Otud6b
Tmem51	Slc24a4	Mettl27
Pck2	Enc1	Wdr7
Dhx33	Ydjc	Plppr2
Gemin8	Mapk1ip1	Btaf1
Plekhg1	Cir1	Rundc3b
Dok4	Cpsf2	Abcb1a
Erc2	Myrip	Ofd1
Ppip5k2	Abca9	Cstpp1
Rimklb	Gck	Ranbp17
Tasor	Phlda3	Pogk
Oaz2	Poldip3	Mis12
Ppp1r14c	Fam169a	Kazn
Dnph1	Abca8a	Ildr2
Efhd2	Ptpre	Tmem51
Rad54l2	Pdcd7	Pck2
Nup88	Rpl37	Dhx33

Phc1
Mthfd1l
Hmgn1
Madd
Tbl3
Col16a1
Slco4c1
Dnajc16
Limd2
Ap1g2
St8sia4
Sh3pxd2b
Camta2
Creg1
Klc3
Il17rd
Virma
Zfhx2
Scamp5
Kcna2
Hnrnpul1
Cep126
Eif4h
Erg
Ppp1r13l
Ints8
Slc25a34
Rnf167
Cd53
Siah1b
Cmtm5
Appl1
Spen
Snrnp25
Oard1
Cept1
Cop1
Ttc3
C1qtnf4
Iqcc
Iqsec3
Eml2
Tex264
Dennd6a
Hlcs
1700123O20Rik
Snrpd2
Gpr161
Six5
Szrd1
Tiprl
Sft2d2
Psme4
Fhdc1
Ppp4r3a
Tcf20
Mcm3
Ankrd13a
Ipo9
Ndufa7
Shisa4
Git2
Lman1
Wipi1
Gpr45
Dlx1
Tdrkh
Ammecr1l
Slc16a6
Metap1d
Nol4
Rnpep
Fam222a
Rimoc1
Agrn
Mvk
Mfsd9
Tango6
Pkp2
Pigs
Znrf3
Dcaf17
Mettl8
Arhgef11
Elmod1
Rapgef5
Zbed3
Tlk1
Acacb
Wdr41
Mat2b
Igsf3
Csgalnact2
Tbca
Dstyk
Dync2i1
Wdr11
Zfp386
Tmcc2
Abhd14b
Svop
Hnrnpf
Arsb
Zfp239
Kank3
Uggt2
Gemin8
Plekhg1
Dok4
Nrl
Erc2
Ppip5k2
Rimklb
Tasor
Oaz2
Ppp1r14c
Dnph1
Efhd2
Rad54l2
Nup88
Phc1
Mthfd1l
Hmgn1
Madd
Tbl3
Col16a1
Slco4c1
Dnajc16
Limd2
Ap1g2
St8sia4
Sh3pxd2b
Camta2
Creg1
Klc3
Il17rd
Virma
Zfhx2
Scamp5
Rcsd1
Kcna2
Hnrnpul1
Cep126
Eif4h
Erg
Ppp1r13l
Ints8
Slc25a34
Rnf167
Cd53
Siah1b
Cmtm5
Appl1
Spen
Snrnp25
Oard1
Cept1
Cop1
Ttc3

Plekhh2	Inpp5f	C1qtnf4
Reps2	Csdc2	Iqcc
Erf	Ccdc115	Iqsec3
Bsdc1	Klhdc8a	Eml2
Crocc	Vwa1	Tex264
Ino80d	Ssh1	Dennd6a
Begain	Rassf4	Hlcs
Osbpl10	Ppig	1700123O20Rik
Wdr25	Msantd2	Snrpd2
Tmem205	Cox10	Gpr161
Gfer	Klhl23	Szrd1
Kcnd3	Dzip1	Tiprl
Gm21988	Tent2	Sft2d2
Atp1a3	Armc5	Psme4
Fbxw4	Bend6	Plekhh2
Slc19a2	Nfrkb	Reps2
S100pbp	Zfp451	Erf
Rfx3	Chchd7	Bsdc1
Ulk4	Slc35e2	Crocc
Slc16a11	Tbc1d22b	Ino80d
Arhgef1	Kdm5b	Begain
Tet2	Sanbr	Osbpl10
Rcc2	Abhd14a	Wdr25
Rps19	Fbxo38	Tmem205
Cables1	Zfand4	Gfer
Arhgef10l	Bag2	Kcnd3
Igsf21	Sgsm1	Gm21988
Sh3kbp1	Lyn	Atp1a3
Abhd4	Rabif	Fbxw4
Npnt	Tmc7	Slc19a2
Trim62	Grk3	S100pbp
Nrg3	Ptchd4	Rfx3
Map7d2	Isl1	Ulk4
Iffo2	Nxt2	Slc16a11
Ghitm	Sestd1	Arhgef1
Dpcd	Pelo	Tet2
Irgq	Itga1	Rcc2
Fam117b	Stab1	Rps19
Ramp3	Mrtfa	Cables1
Wdr43	Gm5617	Arhgef10l
Wwp1	Ttc19	Igsf21
Nacad	Ehbp1	Sh3kbp1
Fzd7	Sgsm3	Abhd4
Grid1	Tmem183a	Npnt
Rwdd2b	Setd1a	Trim62
Ostc	S100a13	Nrg3
Tspyl2	Pbrm1	Map7d2
Elmo1	Hps4	Iffo2
Iqsec2	Specc1	Ghitm
Pde9a	Arl15	Dpcd
Nbl1	Arel1	Irgq
Msantd4	Frem3	Fam117b
H2az2	Gnl3	Ramp3

Zfp598
N4bp2l1
Smc1a
Cyyr1
Ripk2
Pnma8a
Dnah7b
Brca2
Osgin2
Otud3
Zmiz2
Lonp1
Hectd2
Prkd2
Pla2g5
Rpusd1
Trir
Map6d1
Yeats2
Clvs1
Arhgap11a
Elovl6
Arhgap12
Phf8
Ublcp1
Chd7
Vps41
Rbbp8
Mul1
Wnk3
Zfp236
Car8
Rusc1
Uspl1
Dmxl2
Tox
Ttc1
Cdk13
Katnal1
Gtf3c3
Sntb2
Nkx6-2
Slc7a1
Thoc6
Inhba
Pcf11
Atp1b2
Atg2b
Ankrd42
Wrap53
Rap1gap
Vcf2
Rgl2
Osbpl6
Lgalsl
Snx18
Rbm45
Smim12
Dlgap3
Tsen2
Gatad2b
Rbm7
Crtac1
Dennd4b
Atf4
Zmym6
Agps
Nfkbil1
Fbrs
Frmpd3
Dhx29
Mgat3
Adora1
Zfp532
Mindy2
Zmym4
Mios
Reln
C1galt1
Dctpp1
Zfp410
Tbc1d8b
Abcb4
Leo1
Tbc1d10b
Prdm10
Ago4
Cd2bp2
Usp22
Mideas
Dmtf1
AA986860
Pwwp3b
Ubap2l
Dnal1
Sun2
Kcnj12
Golga7b
Gtpbp1
Sem1
Asxl1
Sin3a
Adprs
Fam227a
Nek10
Dhrs7b
Wdr43
Wwp1
Nacad
Fzd7
Grid1
Rwdd2b
Ostc
Tspyl2
Elmo1
Iqsec2
Ccdc8
Pde9a
Nbl1
Msantd4
H2az2
Zfp598
N4bp2l1
Smc1a
Cyyr1
Ripk2
Pnma8a
Dnah7b
Brca2
Osgin2
Otud3
Zmiz2
Lonp1
Hectd2
Prkd2
Rpusd1
Trir
Map6d1
Yeats2
Clvs1
Elovl6
Arhgap12
Phf8
Ublcp1
Chd7
Vps41
Rbbp8
Mul1
Wnk3
Zfp236
Car8
Rusc1
Uspl1
Dmxl2
Tox
Ttc1
Cdk13
Katnal1
Gtf3c3

Ssr2
Pum3
Myzap
Shtn1
Ccdc9
Cldn5
Htr2c
Mettl18
Wapl
Dicer1
Pik3r1
Meis3
Paqr6
Hibch
Nthl1
Utp4
Mfsd6
Gk5
Arhgap32
Mmrn2
Rpl21
Tardbp
Gpr12
Shld2
Dcp1b
Zfp281
Stx3
Cep78
Rrp9
Irf8
Rnf123
Ago1
Sox5
Disp3
Ptchd1
Fbxo2
Fmod
Nop53
L3mbtl4
Camsap2
Selenow
Prelp
Sdk2
Tmtc4
Cdc42ep4
Inava
Mbp
Entpd3
Bicdl1
Ccdc74a
Mtnap1
Gucy1a2
Ggact
Mier2
Ube2q1
4632404H12Rik
Thsd7b
Cux2
Ipo11
Sh2b3
Fam199x
Kdm7a
Kcna4
Atxn2
Hirip3
Asb4
Stk40
Pbxip1
Oscp1
Maff
Safb2
Shc1
Zfyve1
Xkr7
Pla2g6
Flad1
Itpr3
Alkbh5
Arrdc4
Wdr55
Dusp15
Immp1l
Rgs8
Ypel3
Garem1
Selenok
Jph1
Mapk6
Stn1
Dhx9
Sipa1l1
Commd10
Dnali1
Atpaf2
Tceal9
Naa25
Map3k9
Trafd1
Wdr74
Ttc9
Dpm3
Bmt2
Sgtb
Hectd4
Id1
Krtcap2
Sntb2
Nkx6-2
Slc7a1
Thoc6
Inhba
Pcf11
Atp1b2
Atg2b
Ankrd42
Wrap53
Rap1gap
Vcf2
Rgl2
Ssr2
Pum3
Shtn1
Ccdc9
Cldn5
Htr2c
Mettl18
Wapl
Dicer1
Pik3r1
Meis3
Paqr6
Hibch
Nthl1
Utp4
Mfsd6
Gk5
Arhgap32
Mmrn2
Rpl21
Tardbp
Cacna2d4
Gpr12
Shld2
Dcp1b
Zfp281
Stx3
Cep78
Rrp9
Irf8
Rnf123
Ago1
Sox5
Disp3
Hspb8
Ptchd1
Fbxo2
Fmod
Nop53
Camsap2

Vcf1
Mrps27
Gcn1
Kif21b
Ddx24
Klf8
Pcca
Pnpla3
Slc39a11
Rragb
Rims1
Pyroxd1
Fbxl9
Bivm
Fcho2
Amot
Kcnj2
Rasl12
Cox6a1
Btbd7
Mpped1
Trpc5
Ubr7
Gm20604
Alg13
Pi4ka
Coro2b
Pgm5
Coq5
Kirrel1
Rnf10
Utp15
Plekha6
Gpr155
Tpp2
Ubac2
Ppp2r2d
Slc24a4
Enc1
Ydjc
Mapk1ip1
Cir1
Cpsf2
Myrip
Abca9
Phlda3
Poldip3
Fam169a
Abca8a
Ptpre
Pdcd7
Haus1
Rpl37
Bex2
Nmnat2
Tmem108
Mrap2
Maneal
Trim46
Hebp1
Smg7
Exog
Cimip2b
Rnf214
Aqp11
Gpr153
Hecw2
Krba1
Fgf11
Alkbh6
Nrep
Lrrtm3
Zc3h6
Trp53rkb
Gm9776
Tom1
Lhfpl4
Aida
Foxo4
Greb1l
Sbk1
Borcs5
Nhlrc3
Gng2
Nemp2
Edem3
Dnai3
Zfp513
Tmem18
Spice1
Dpy19l1
Synpo
Zfp866
Tuba1c
Mob1a
Tmem186
Crppa
Ppp2r3a
Pyurf
Tmem212
Simc1
Rfesd
Zmpste24
Upf2
Tmem64
Pigv
Selenow
Prelp
Sdk2
Tmtc4
Nlrp5-ps
Cdc42ep4
Inava
Mbp
Entpd3
Bicdl1
Ccdc74a
Mtnap1
Gucy1a2
Ggact
Vcf1
Mrps27
Kctd12b
Gcn1
Kif21b
Ddx24
Klf8
Pcca
Slc39a11
Rragb
Rims1
Pyroxd1
Fbxl9
Bivm
Fcho2
Amot
Kcnj2
Rasl12
Cox6a1
Btbd7
Mpped1
Trpc5
Ubr7
Gm20604
Alg13
Pi4ka
Coro2b
Pgm5
Coq5
Kirrel1
Rnf10
Pde3a
Utp15
Plekha6
Gpr155
Tpp2
Ubac2
Ppp2r2d
Slc24a4

Fhdc1	Fam13c	Enc1
Ppp4r3a	Uevld	Ydjc
Tcf20	Trim56	Mapk1ip1
Ankrd13a	Tmem11	Cir1
Ipo9	Zfp784	Cpsf2
Ndufa7	Smco3	Myrip
Shisa4	B3galnt1	Abca9
Git2	Kcnj6	Gck
Lman1	D17H6S53E	Phlda3
Wipi1	Pcdhb19	Poldip3
Gpr45	Fbrsl1	Fam169a
Dlx1	Hexim2	Abca8a
Tdrkh	Gprasp1	Ptpre
Ammecr1l	Tmem130	Pdcd7
Slc16a6	2510009E07Rik	Haus1
Metap1d	Gpr135	Rpl37
Nol4	Usp48	Fhdc1
Rnpep	Otud1	Ppp4r3a
Fam222a	Rnf227	Card6
Rimoc1	Eif3j2	Tcf20
Agrn	Leng9	Mcm3
Mvk	Epop	Ankrd13a
Mfsd9	Gpr149	Ipo9
Tango6	Pgp	Ndufa7
Pkp2	Gjc2	Shisa4
Pigs	Zfp536	Git2
S100a10	Pcdhb12	Lman1
Znrf3	Elfn2	Wipi1
Dcaf17	Rab9b	Gpr45
Mettl8	Zbtb37	Dlx1
Arhgef11	Acot6	Tdrkh
Elmod1	Tril	Ammecr1l
Rapgef5	Rai2	Slc16a6
Zbed3	Sorcs1	Metap1d
Tlk1	Setx	Nol4
Wdr41	Dnai7	Rnpep
Ncapg2	Zc2hc1a	Fam222a
Mat2b	Mdga1	Rimoc1
Igsf3	Pars2	Agrn
Csgalnact2	Pxylp1	Mvk
Tbca	Zfp3	Mfsd9
Dstyk	Vps37d	Tango6
Dync2i1	Ubxn10	Pkp2
Wdr11	1700019D03Rik	Pigs
Zfp386	Adamts3	S100a10
Tmcc2	Rbm20	Znrf3
Abhd14b	Pld6	Dcaf17
Svop	Tox3	Spidr
Hnrnpf	Diras1	Mettl8
Arsb	Dpy19l3	Arhgef11
Zfp239	Fem1a	Elmod1
Kank3	1190005I06Rik	Rapgef5
Uggt2	Pde12	Zbed3

Inpp5f	Rpl7	Tlk1
Inka1	Ptpn11	Acacb
Csdc2	1700048O20Rik	Wdr41
Ccdc115	Oaz1-ps	Ncapg2
Klhdc8a	Lysmd4	Mat2b
Vwa1	Tmem145	Igsf3
Ssh1	Mgat5b	Csgalnact2
Rassf4	Taf10	Tbca
Ppig	Zmym1	Dstyk
Msantd2	Kbtbd7	Dync2i1
Cox10	Slc36a4	Wdr11
Klhl23	Zfp469	Zfp386
Dzip1	Trp53bp1	Tmcc2
Tent2	Cenatac	Abhd14b
Armc5	Klhl15	Svop
Bend6	Wdfy3	Hnrnpf
Nfrkb	Thrap3	Arsb
Cmklr1	Orai3	Zfp239
Slc35f2	Emx2	Kank3
Zfp451	Cep164	Uggt2
Chchd7	Pura	Inpp5f
Slc35e2	2900052L18Rik	Inka1
Tbc1d22b	Mgat2	Csdc2
Kdm5b	Gpr75	Ccdc115
Sanbr	Gls2	Klhdc8a
Abhd14a	Mrpl50	Vwa1
Fbxo38	Rell2	Ssh1
Zfand4	Irf2bp1	Rassf4
Bag2	Ccdc141	Ppig
Sgsm1	Als2cl	Msantd2
Lyn	Fmn1	Cox10
Rabif	Pcdhb14	Klhl23
Tmc7	Cep68	Dzip1
Grk3	Gpr22	Tent2
Ptchd4	Zrsr2-ps1	Armc5
Nxt2	Tafa2	Bend6
Sestd1	Eml6	Nfrkb
Pelo	S100a1	Cmklr1
Stab1	Rsbn1	Slc35f2
Mrtfa	Bmerb1	Zfp451
Gm5617	Pheta1	Chchd7
Ttc19	Arf6	Slc35e2
Ehbp1	1810030O07Rik	Tbc1d22b
Sgsm3	Nkrf	Kdm5b
Tmem183a	Lsm8	Sanbr
Setd1a	Rnf182	Abhd14a
S100a13	Foxo1	Fbxo38
Pbrm1	Spink10	Zfand4
Hps4	Gpr146	Bag2
Specc1	Kcnj4	Sgsm1
Arl15	Grsf1	Ammecr1
Arel1	Dnajc21	Lyn
Gnl3	Nxpe4	Rabif

Osbpl6	Nhlrc1	Tmc7
Lgalsl	Pced1b	Grk3
Snx18	Osbpl1a	Ptchd4
Rbm45	Pcsk9	Nxt2
Smim12	Ctla2a	Sestd1
Dlgap3	Ubb-ps	Pelo
Tsen2	Cnr1	Itga1
Gatad2b	Zfp879	Stab1
Rbm7	Ubr3	Hsd3b7
Crtac1	Trp53i13	Mrtfa
Dennd4b	Ackr3	Gm5617
Atf4	Phlpp1	Ttc19
Zmym6	Marveld1	Ehbp1
Agps	Slc25a53	Sgsm3
Nfkbil1	Snhg11	Tmem183a
Fbrs	Sowaha	Setd1a
Frmpd3	BC024139	S100a13
Dhx29	Tmem74b	Pbrm1
Mgat3	Cxxc4	Hps4
Adora1	Slc16a13	Specc1
Zfp532	Tigd3	Arl15
Mindy2	Sptssa	Arel1
Zmym4	Camsap3	Gnl3
Mios	N6amt1	Osbpl6
Reln	Dock5	Lgalsl
C1galt1	Zfp507	Snx18
Dctpp1	Rin3	Rbm45
Zfp410	Fhip1b	Smim12
Tbc1d8b	Tent5c	Dlgap3
Abcb4	Tnfaip8l1	Tsen2
Leo1	Lncpint	Gatad2b
Tbc1d10b	Ascc1	Rbm7
Prdm10	Zfand3	Crtac1
Ago4	2510039O18Rik	Dennd4b
Cd2bp2	Hs3st5	Atf4
Sdhaf3	Bod1	Zmym6
Usp22	Zfp488	Agps
Mideas	Tram1l1	Ccno
Dmtf1	Rps2	Nfkbil1
AA986860	Dact1	Fbrs
Ubap2l	Tceal3	Frmpd3
Dnal1	Rasip1	Dhx29
Sun2	Cage1	Mgat3
Kcnj12	Acp1	Adora1
Golga7b	Garem2	Mfap4
Gtpbp1	Smim7	Zfp532
Sem1	Zbtb39	Mindy2
Asxl1	Swi5	Zmym4
Sin3a	Rnf208	Mios
Adprs	Cnrip1	Reln
Fam227a	Csrnp2	C1galt1
Nek10	Pard6b	Dctpp1
Dhrs7b	Gm7334	Zfp410

Mier2	Zbtb7c	Tbc1d8b
Ube2q1	Csrnp3	Abcb4
4632404H12Rik	Plppr4	Leo1
Thsd7b	Fzd1	Tbc1d10b
Cux2	Zfp612	Prdm10
Ipo11	Tmem201	Ago4
Sh2b3	Kcnj10	Cd2bp2
Fam199x	Gemin7	Sdhaf3
Kdm7a	Gskip	Usp22
Kcna4	Dok7	Mideas
Atxn2	Airim	Dmtf1
Hirip3	Trmt10c	AA986860
Stk40	Macir	Pwwp3b
Pbxip1	Scml4	Ubap2l
Oscp1	Hjurp	Dnal1
Safb2	Zfp36	Sun2
Shc1	Fads6	Kcnj12
Zfyve1	Setd2	Golga7b
Xkr7	Isca1	Gtpbp1
Pla2g6	Cyb5d1	Acot5
Flad1	Zfp354c	Sem1
Acad12	Shb	Asxl1
Alkbh5	Ankrd45	Sin3a
Arrdc4	Lsm11	Adprs
Wdr55	Lemd2	Fam227a
Dusp15	Ankrd50	Nek10
Immp1l	Zfp444	Dhrs7b
Rgs8	Coa4	Mier2
Ypel3	Uqcrq	Ube2q1
Garem1	4930503L19Rik	4632404H12Rik
Selenok	Syt16	Thsd7b
Npl	Rassf9	Cux2
Jph1	H1f10	Ipo11
Mapk6	Sstr3	Sh2b3
Stn1	Zfp367	Fam199x
Dhx9	Cfap43	Kdm7a
Sipa1l1	Ubtd2	Kcna4
Commd10	Pwwp2a	Atxn2
Atpaf2	Kctd21	Hirip3
Tceal9	Napepld	Stk40
Naa25	Sft2d3	Pbxip1
Map3k9	Tst	Oscp1
Trafd1	Shld1	Safb2
Wdr74	Fzd5	Shc1
Ttc9	Tubg2	Zfyve1
Dpm3	Prrt3	Xkr7
Bmt2	Ankrd34b	Pla2g6
Sgtb	Prkce	Flad1
Hectd4	Megf8	Itpr3
Id1	Lrfn4	Alkbh5
Krtcap2	Kcng3	Arrdc4
Bex2	Pcdhb7	Wdr55
Nmnat2	Zc2hc1c	Dusp15

Tmem108	E130308A19Rik	Immp1l
Mrap2	Rnf216	Rgs8
Maneal	Lingo2	Ypel3
Trim46	S1pr5	Garem1
Hebp1	S1pr1	Selenok
Smg7	Arhgef37	Jph1
Exog	Magi1	Mapk6
Rnf214	Kmt5b	Stn1
Aqp11	Slc25a26	Dhx9
Gpr153	Dmd	Sipa1l1
Hecw2	Saysd1	Commd10
Krba1	Prrt2	Shcbp1l
Mcts2	Rpl18a	Atpaf2
Flt3	Tubb2b	Tceal9
Fgf11	Bola3	Naa25
Alkbh6	AI467606	Map3k9
Nrep	Amer3	Trafd1
Lrrtm3	Borcs6	Wdr74
Zc3h6	Sox3	Ttc9
Trp53rkb	Shroom2	Dpm3
Gm9776	Cirbp	Bmt2
Tom1	Lrrc3b	Sgtb
Lhfpl4	Dpy19l4	Hectd4
Aida	Vcpip1	Id1
Foxo4	Nudt18	Krtcap2
Mamstr	Asxl3	Bex2
Greb1l	Hs6st1	Nmnat2
Sbk1	Eola1	Tmem108
Notum	Med26	Mrap2
Borcs5	Zfp688	Maneal
Nhlrc3	Zfp574	Trim46
Gng2	Morn2	Hebp1
Nemp2	Klhdc9	Smg7
Edem3	Zfp691	Exog
Zfp513	Dcaf12l1	Rnf214
Tmem18	Rtn4rl1	Lgr6
Spice1	Insig1	Aqp11
Dpy19l1	Preb	Gpr153
Synpo	Lhfpl2	Hecw2
Zfp866	Fahd1	Krba1
Tuba1c	Adra2c	Mcts2
Hic1	Zfp423	Flt3
Mob1a	Nyap1	Fgf11
Tmem186	Sh2d5	Alkbh6
Crppa	Wdr81	Nrep
Ppp2r3a	Kcnk13	Lrrtm3
Pyurf	Trim39	Zc3h6
Loricrin	2410002F23Rik	Trp53rkb
Simc1	Dipk2a	Gm9776
Rfesd	Hnrnph2	Tom1
Gpi-ps	Tmem60	Lhfpl4
Zmpste24	Cox19	Aida
Upf2	Gprin3	Foxo4

Tmem64	Zfp956	Mamstr
Pigv	Trrap	Greb1l
Fam13c	Pcdhb3	Sbk1
Uevld	Sys1	Notum
Trim56	Pou3f3	Borcs5
Tmem11	Zfp560	Nhlrc3
Zfp784	C1ql1	Gng2
B3galnt1	Kcna5	Onecut1
Kcnj6	Ddx28	Nemp2
D17H6S53E	Penk	Edem3
Pcdhb19	St7l	Disc1
Fbrsl1	Frrs1l	Zfp513
Filip1l	Glb1	Tmem18
Gm6741	Zfp553	Spice1
Hexim2	Dbx2	Dpy19l1
Gprasp1	Chrm2	Synpo
Tmem130	Esf1	Zfp866
2510009E07Rik	Pigz	Tuba1c
Gpr135	Sh3tc2	Hic1
Usp48	Mtus1	Lrrn4
Otud1	Zfp629	Mob1a
Rnf227	Vwc2l	Tmem186
Hilpda	Pcdhb10	Crppa
Eif3j2	Pid1	Ppp2r3a
Leng9	Plekha7	Hpdl
Epop	Cdc42ep2	Pyurf
Pgp	Mfsd5	Simc1
Gjc2	Spred2	Rfesd
Zfp536	Col27a1	Gpi-ps
Pcdhb12	Wdr89	Zmpste24
Elfn2	Thtpa	Upf2
Rab9b	Pnoc	Tmem64
Zbtb37	Sprn	Pigv
Acot6	Tssc4	Fam13c
Tril	Togaram2	Uevld
Hscb	Basp1	Trim56
Rai2	B230219D22Rik	Tmem11
Sorcs1	Ifitm10	Zfp784
Setx	Ccdc149	Smco3
Zc2hc1a	Whamm	B3galnt1
Mdga1	Gm9801	Kcnj6
Pars2	Zfp36l2	D17H6S53E
Pxylp1	Zswim3	Pcdhb19
Zfp3	Serpinb9	Fbrsl1
Vps37d	Ccdc9b	Hexim2
1700019D03Rik	Lyrm2	Gprasp1
Adamts3	Cradd	Tmem130
Rbm20	Adra1a	2510009E07Rik
0610009L18Rik	Paip2b	Gpr135
Tox3	Npas4	Usp48
Diras1	C2cd4c	Otud1
Dpy19l3	Tmem268	Rnf227
Kcns3	Ifit2	Eif3j2

Fem1a	Mtmr11	Leng9
1190005I06Rik	Mrps12	Epop
Pde12	Cavin2	Pgp
Rpl7	Wnk1	Gjc2
Ptpn11	Gpr158	Zfp536
Gm12481	Ing1	Pcdhb12
1700048O20Rik	Slc25a51	Elfn2
Oaz1-ps	C2cd2	Rab9b
Rtn4r	Tmem104	Zbtb37
Adamtsl5	Eif4g1	Acot6
Lysmd4	Onecut2	Tril
2900005J15Rik	B3gat1	Hscb
Tmem145	Zfp830	Rai2
Mgat5b	Pofut1	Sorcs1
Taf10	Stard5	Setx
Zmym1	Snx12	Zc2hc1a
Kbtbd7	Otulin	Fbxl7
Slc36a4	Eid2	Mdga1
Trp53bp1	Ppp1r15b	Pars2
Cenatac	Lrrc8d	Pxylp1
Klhl15	Hpcal4	Zfp3
Wdfy3	Mosmo	Vps37d
Thrap3	Cep295	1700019D03Rik
Orai3	Brd10	Ecm2
Emx2	Patl1	Adamts3
Cep164	Fut10	Rbm20
Pura	Tmem229b	0610009L18Rik
2900052L18Rik	Chrm3	Tox3
Mgat2	Olig1	Diras1
Gpr75	Gldn	Dpy19l3
Gls2	Adamts6	Kcns3
Npy5r	Nxph1	Fem1a
Adgrd1	Gsg1l	1190005I06Rik
Mrpl50	Zfp84	Pde12
Pcdhb21	Cd109	Rpl7
Rell2	Pcdhb20	Ptpn11
Irf2bp1	Iqub	1700048O20Rik
Ccdc141	Scaf8	Oaz1-ps
Als2cl	Pnma2	Rtn4r
Fmn1	Rprml	Adamtsl5
Pcdhb14	Scand1	Lysmd4
Cep68	Vps13a	2900005J15Rik
Gpr22	Hepacam	Tmem145
Zrsr2-ps1	Usp27x	Mgat5b
Tafa2	Pnma3	Taf10
Eml6	Zfp62	Zmym1
S100a1	Myorg	Kbtbd7
Rsbn1	Stxbp6	Slc36a4
Bmerb1	Hs3st2	Zfp469
Proca1	Ermp1	Trp53bp1
Pheta1	Slc25a23	Cenatac
Arf6	Rpl37a	Klhl15
1810030O07Rik	Zfp322a	Wdfy3

Nkrf	Gjb2	Thrap3
Bclaf3	Rpl27a	Orai3
Lsm8	Asphd1	Emx2
Rnf182	Jrk	Cep164
Foxo1	Pcdhb17	Pura
Spink10	Rbp1	2900052L18Rik
Gpr146	Yod1	Mgat2
Gm7887	Kcnk6	Gpr75
Kcnj4	Bex3	Gls2
Grsf1	Hnrnpa1	Npy5r
Dnajc21	Cmtr2	Mrpl50
Nxpe4	Ppm1e	Pcdhb21
Nhlrc1	Camk2n1	Rell2
Bhlha9	Nexmif	Irf2bp1
Pced1b	Sox18	Ccdc141
Osbpl1a	Scn4b	Als2cl
Ubb-ps	Cox17	Fmn1
Cnr1	Golph3l	Pcdhb14
Zfp879	Kctd4	Cep68
Ubr3	Ar	Gpr22
Gpr4	Zfp526	Zrsr2-ps1
1700001O22Rik	Fam43a	Tafa2
Trp53i13	Spin2c	Eml6
Ackr3	Zfp319	S100a1
Alkbh2	Unc119b	Rsbn1
Phlpp1	Rlig1	Bmerb1
Marveld1	Zfp518b	Pheta1
Slc25a53	Lyrm4	Arf6
Snhg11	Prr12	1810030O07Rik
Lacc1	Tmem215	Nkrf
Sowaha	Bdh1	Bclaf3
Tmem74b	Tcaim	Lsm8
Cxxc4	B3gntl1	Rnf182
Slc16a13	Hrk	Foxo1
Tigd3	Vwa5b2	Spink10
Sptssa	Ttc34	Gpr146
Camsap3	Zfp316	Kcnj4
N6amt1	Rbm12b1	Grsf1
Dock5	Cxxc5	Dnajc21
Zfp507	Mtfr1l	Nxpe4
Rin3	Lyset	Nhlrc1
Shisa2	C87436	Bhlha9
Fhip1b	Chtf8	Il20rb
Tent5c	Slitrk4	Pced1b
Tnfaip8l1	Csnk2a2	Osbpl1a
Lncpint	Mapk10	Ubb-ps
Ascc1	Hmga1	Cnr1
Zfand3	Cdc42se1	Zfp879
2510039O18Rik	Cystm1	Ubr3
Hs3st5	Fat4	Gpr4
Zfp758	Ccdc66	1700001O22Rik
Bod1	Mrps7	Trp53i13
Tram1l1	Fam83h	Ackr3

Rps2	Rhoj	Phlpp1
Ackr2	Epm2aip1	Marveld1
Dact1	Riox1	Slc25a53
Tceal3	Zfp787	Snhg11
Rasip1	Gpr61	Lacc1
Cage1	Cldn12	Sowaha
Acp1	Mpeg1	Tmem74b
Garem2	Cyren	Cxxc4
Smim7	Lrrc75b	Slc16a13
Zbtb39	Atp10d	Tigd3
Swi5	Slc39a3	Dsg2
Rnf208	Brox	Sptssa
Cnrip1	Ckap4	Camsap3
Csrnp2	Vat1l	N6amt1
Pard6b	Hectd3	Dock5
Gm7334	Pramel12	Zfp507
Zbtb7c	Fbl	Shisa2
Csrnp3	Mbtps2	Fhip1b
Plppr4	Atxn1	Tent5c
Fzd1	Zfp740	Tnfaip8l1
Zfp612	Tefm	Lncpint
Tmem201	Gpr6	Ascc1
Ccnjl	Adck2	Zfand3
Kcnj10	Nqo2	2510039O18Rik
Gemin7	Zbtb21	Hs3st5
Slc38a6	Tshz1	Zfp758
Gskip	Tapt1	Bod1
Dok7	Mars2	Lingo4
Airim	Spsb4	Zfp488
Serpinb1a	Zfp41	Tram1l1
Trmt10c	Fbxo41	Rps2
Macir	Cfap65	Dact1
Scml4	Mipol1	Tceal3
Hjurp	Spata2	Rasip1
Zfp36	Pcdhb15	Cage1
Fads6	Zfp445	Acp1
Setd2	Nipa1	Garem2
Isca1	D030056L22Rik	Smim7
Cyb5d1	Tmem164	Zbtb39
Zfp354c	Dusp28	Swi5
Shb	Ngrn	Rnf208
D630023F18Rik	Lrrc4b	Cnrip1
Ankrd45	Tmem198b	Csrnp2
Lsm11	Rnf31	Pard6b
Lemd2	Ticam1	Gm7334
Ankrd50	Cltc	Zbtb7c
Zfp444	Cd24a	Csrnp3
Coa4	Zfp654	Plppr4
Uqcrq	Tet1	Fzd1
4930503L19Rik	Khnyn	Zfp612
Syt16	Cyp4x1	Tmem201
H1f10	Samd14	Ccnjl
Sstr3	Rab2a	Kcnj10

Zfp367
Ubtd2
Pwwp2a
Kctd21
Napepld
Sft2d3
Tst
Shld1
Tubg2
Prrt3
Cetn4
Ankrd34b
Prkce
Megf8
Lrfn4
Kcng3
Pcdhb7
Zc2hc1c
E130308A19Rik
Rnf216
Lingo2
S1pr5
S1pr1
Magi1
Kmt5b
Slc25a26
Dmd
Ldhb-ps
Saysd1
Prrt2
Rpl18a
Tubb2b
Pigw
Bola3
Amer3
Borcs6
Shroom2
Cirbp
Lrrc3b
Dpy19l4
Vcpip1
Nudt18
Asxl3
Hs6st1
Eola1
Kcng4
Med26
Zfp688
Zfp574
Morn2
Klhdc9
Zfp691
Dcaf12l1
Dync2h1
Dusp18
Ythdf3
Rpl9
Cdh19
Fam185a
Mageh1
Taf9b
H2bc6
C2cd3
Ptgs1
Emc6
Gap43
Zfp358
Neurl4
Zfp286
Lancl3
Gm973
Abhd17b
Zfp768
B4gat1
Atmin
Ctdspl
Zbtb44
Flrt2
Gpr68
Rexo1
AI837181
Dlk2
Xxylt1
Arl4a
Gphn
Dynlrb1
Ark2n
Zfp30
Dlgap2
Rnf152
Mroh7
Baiap3
Tspyl1
Fbxo28
Cltb
Tmem41b
Lxn
Tyw3
Zfp235
Frat2
Ankrd34c
A430005L14Rik
Paxx
Mtrfr
Fbxo30
Polr1g
Gemin7
Slc38a6
Gskip
Dok7
Erich5
Airim
Serpinb1a
Trmt10c
Macir
Scml4
Hjurp
Zfp36
Fads6
Setd2
Isca1
Cyb5d1
Zfp354c
Shb
D630023F18Rik
Ankrd45
Lsm11
Lemd2
Ankrd50
Zfp444
Coa4
Uqcrq
4930503L19Rik
Syt16
H1f10
Sstr3
Zfp367
Ubtd2
Pwwp2a
Kctd21
Napepld
Sft2d3
Tst
Shld1
Tubg2
Prrt3
Cetn4
Ankrd34b
Prkce
Megf8
Lrfn4
Kcng3
Pcdhb7
E130308A19Rik
Rnf216
Lingo2
S1pr5
S1pr1
Magi1

Rtn4rl1	Trpt1	Kmt5b
Insig1	Gal3st3	Slc25a26
Preb	Msl3l2	Dmd
Lhfpl2	Rps8	Ldhb-ps
Sowahb	Yipf6	Saysd1
Fahd1	Champ1	Prrt2
Adra2c	Ust	Rpl18a
Zfp423	Ppp1r2	Tubb2b
Nyap1	Ubiad1	Pigw
Sh2d5	Bola2	Bola3
Wdr81	Wbp1l	Amer3
Tmem88	Rnf150	Borcs6
Kcnk13	Zc3hav1l	Sox3
Trim39	Lrrc49	Shroom2
Akr1e1	Atg16l2	Cirbp
2410002F23Rik	Ankfn1	Lrrc3b
Dipk2a	Phf13	Dpy19l4
Hnrnph2	Lix1	Vcpip1
Tmem60	Flrt1	Nudt18
Cox19	Slc38a9	Asxl3
Gprin3	Sned1	Hs6st1
Zfp956	Gjb1	Eola1
Trrap	Akap10	Kcng4
Pcdhb3	Pygo2	Med26
Sys1	Cdca4	Zfp688
Gpr150	Diras2	Zfp574
Pou3f3	Bri3	Morn2
Zfp560	Bex4	Klhdc9
C1ql1	Lonp2	Zfp691
Kcna5	Usp14	Tmem86b
Ddx28	Rell1	Dcaf12l1
Mettl24	Tnrc6b	Rtn4rl1
Penk	Sstr2	Insig1
St7l	Tshz2	Preb
Frrs1l	Ankrd16	Lhfpl2
Glb1	Pcdhb16	Sowahb
Zfp553	Trappc9	Fahd1
Dbx2	Sox1ot	Adra2c
Chrm2	Marcksl1	Zfp423
Esf1	Kcna3	Nyap1
Pigz	Kcna1	Sh2d5
Sh3tc2	Ino80c	Wdr81
Mtus1	Dipk1c	Cxcr4
Zfp629	Gigyf2	Kcnk13
Vwc2l	Hes5	Trim39
Pcdhb10	Tmem196	Akr1e1
Pid1	Timm8a1	2410002F23Rik
Plekha7	Tmem229a	Dipk2a
Cdc42ep2	Rgmb	Hnrnph2
Mfsd5	Eno4	Tmem60
Spred2	Ccdc187	Cox19
Col27a1	Isg20l2	Gprin3
Pcdhb4	Arxes2	2810002D19Rik

Wdr89	Zbtb33	Zfp956
Thtpa	Ldlrad3	Trrap
Prr15	Arf1	Pcdhb3
Adrb2	Tenm4	Sys1
Pnoc	Taf13	Gpr150
Sprn	4632415L05Rik	Pou3f3
Mms22l	Rbm15	Zfp560
Tssc4	Arid4a	C1ql1
Zfp764	Entpd1	Ddx28
Basp1	Col6a3	Mettl24
B230219D22Rik	Nat8l	Penk
Ifitm10	Nwd1	St7l
Ccdc149	Kmt2d	Frrs1l
Whamm	Selplg	Glb1
Gm9801	Mcmbp	Zfp553
Zfp36l2	Asb8	Dbx2
Zswim3	Gpr85	Chrm2
Serpinb9	Amigo2	Esf1
Ccdc9b	Mfap1b	Pigz
Lyrm2	Fbxo10	Sh3tc2
Cradd	Rnf149	Mtus1
Adra1a	Gng7	Zfp629
Pcdhb8	Crebrf	Vwc2l
Paip2b	Bcl11b	Pcdhb10
Npas4	Dip2c	Pid1
C2cd4c	Rbm33	Plekha7
Tmem268	Syngr2	Cdc42ep2
Ifit2	Sacs	Mfsd5
Mtmr11	Zfp738	Smtnl2
Mrps12	Dleu7	Spred2
Cavin2	Frmd6	Col27a1
Wnk1	Slitrk3	Pcdhb4
Gpr158	Ankrd46	Wdr89
Teddm2	Pskh1	Thtpa
Ing1	Ric3	Pnoc
Slc25a51	Lhfpl6	Sprn
C2cd2	Pcdhb18	Tssc4
Tmem104	Coa7	Zfp764
Eif4g1	Arxes1	Basp1
B3gat1	Pdp2	B230219D22Rik
Polr2k	F2r	Ifitm10
Zfp830	Socs4	Ccdc149
Pofut1	Scrt1	Whamm
Stard5	Fam171b	Gm9801
Snx12	Gli2	Zfp36l2
Otulin	Zfp407	Zswim3
Eid2	Timm29	Serpinb9
Ppp1r15b	Nup42	Ccdc9b
Lrrc8d	Ccdc57	Lyrm2
Hpcal4	Inka2	Cradd
Mosmo	Spata33	Slitrk6
AB041806	Mypop	Adra1a
Cep295	Zdhhc22	Pcdhb8

Brd10
Patl1
Fut10
Tmem229b
Chrm3
Olig1
Nxph1
Gsg1l
Zfp84
Pcdhb20
Scaf8
Pnma2
Pik3r6
Rprml
Scand1
Vps13a
Hepacam
Usp27x
Pnma3
Zfp62
Myorg
Stxbp6
Ccbe1
Hs3st2
Ermp1
Slc25a23
Rpl37a
Gm11223
Zfp322a
Gjb2
Rpl27a
Asphd1
Jrk
Pcdhb17
Rbp1
Yod1
Kcnk6
Lrrc75a
Bex3
Hnrnpa1
Cmtr2
Ppm1e
Camk2n1
Nexmif
Sox18
Scn4b
Cox17
Golph3l
Kctd4
Ar
Zfp526
Fam43a
Spin2c
Zbtb8b
Fitm2
Nrip1
Mmgt2
Tlcd5
Phldb1
Tob2
Thnsl1
Sp8
Mlec
Igf2
Myof
Nog
Ctxn1
Lemd3
Rhno1
Mex3d
Ccdc6
Lurap1l
Tprn
Tbc1d12
Fndc9
Klhl11
Mcat
Foxo3
Rpl29
Dcun1d3
Cfap100
Cyb561d1
Cep120
Slc35e4
Lonrf2
Dact2
Vps37c
Slc39a9
Vstm2a
Phf3
Hexim1
Cdk5r1
Zfp710
Rimkla
Bnip5
Rnf24
Efna5
Fkrp
Zfp689
Tada3
Atp13a5
Gm6206
Prex2
Mrgpre
Yjefn3
C1galt1c1
Paip2b
Npas4
C2cd4c
Tmem268
Clec14a
Ifit2
Mtmr11
Mrps12
Cavin2
Wnk1
Gpr158
Ing1
Slc25a51
C2cd2
Tmem104
Eif4g1
Onecut2
B3gat1
Polr2k
Zfp830
Pofut1
Stard5
Snx12
Otulin
Eid2
Ppp1r15b
Lrrc8d
Hpcal4
Mosmo
Cep295
Brd10
Patl1
Fut10
Tmem229b
Chrm3
Olig1
Adamts6
Nxph1
Gsg1l
Zfp84
Cd109
Pcdhb20
Scaf8
Pnma2
Rprml
Scand1
Vps13a
Hepacam
Usp27x
She
Pnma3
Zfp62
Myorg

Zfp319
Arsj
Unc119b
Rlig1
Zfp518b
Lyrm4
Prr12
Bdh1
Tcaim
B3gntl1
Hrk
Vwa5b2
Ttc34
Zfp316
Rbm12b1
Cxxc5
Mtfr1l
Lyset
C87436
Tifa
Chtf8
Slitrk4
Csnk2a2
Mapk10
Hmga1
Nxph3
Cdc42se1
Cystm1
Fat4
Ccdc66
Mrps7
Fam83h
Epm2aip1
Riox1
Zfp787
Gpr61
Cldn12
Mpeg1
Cyren
Lrrc75b
Ddit4l
Slc39a3
Brox
Ckap4
Vat1l
Pip5kl1
Hectd3
Pramel12
Fbl
Mbtps2
Atxn1
Irgm1
Zfp740
Nrsn1
Elfn1
Atxn7l2
Ndnf
Tmem121
Mterf2
Rapgef4
Armcx3
Acap2
Bmyc
Ptgr3
Sephs2
Gpr137c
Ankrd34a
Pcdh10
Dcaf5
Oxtr
Fam110b
Frmd3
Plcxd3
Cfap144
Zfp518a
Frmpd4
Purg
Rtl5
Pdp1
Tigd2
Lrp1b
Kcnk3
Scn3b
Mblac1
Iba57
Lix1l
Zfp219
Trappc1
Prmt6
Syt12
Ccdc71
Sorl1
Zfp2
Smcr8
Kmt5a
Tenm2
Retreg2
Dcaf7
Gemin4
Ogfr
Tmem241
Tmem200a
Zfp260
Chchd10
Cyp20a1
Aff4
Stxbp6
Ccbe1
Ermp1
Slc25a23
Rpl37a
Zfp322a
Gjb2
Rpl27a
Asphd1
Jrk
Pcdhb17
Rbp1
Yod1
Lrrc75a
Bex3
Hnrnpa1
Cmtr2
Ppm1e
Camk2n1
Nexmif
Sox18
Scn4b
Tafa4
Cox17
Golph3l
Kctd4
Ar
Zfp526
Fam43a
Spin2c
Zfp319
Arsj
Unc119b
Rlig1
Zfp518b
Lyrm4
Prr12
Tmem215
Bdh1
Tcaim
B3gntl1
Hrk
Vwa5b2
Ttc34
Zfp316
Rbm12b1
Cxxc5
Mtfr1l
Lyset
C87436
Tifa
Chtf8
Slitrk4

Tefm	Ctu2	Csnk2a2
Adck2	Tmem67	Mapk10
Nqo2	Ccnq	Hmga1
Zbtb21	Pls1	Nxph3
Tshz1	Proser1	Cdc42se1
Tapt1	Htr1b	Cystm1
Mars2	Spty2d1	Fat4
Spsb4	Rps23	Ccdc66
1110032F04Rik	Cdc42ep1	Mrps7
Zfp41	Sall2	Fam83h
Fbxo41	Tceal1	Rhoj
Mipol1	Adamts16	Epm2aip1
Spata2	H1f1	Riox1
Pcdhb15	Clip1	Zfp787
Zfp445	Fzd9	Gpr61
Nipa1	Polr1a	Cldn12
D030056L22Rik	Tmie	Mpeg1
Tmem164	Lingo1	Cyren
Dusp28	Ap5b1	Lrrc75b
Ngrn	Cfap46	Atp10d
Lrrc4b	Zfpm1	Ddit4l
Tmem198b	Tsku	Slc39a3
Rnf31	Grm5	Krt1
Ticam1	Zbtb45	Brox
Cltc	Zfp644	Ckap4
Cd24a	Omg	Vat1l
Zfp654	Slc17a5	Hectd3
Dmrta2	C1ql3	Pramel12
Tet1	2310022A10Rik	Fbl
Khnyn	Zbtb5	Mbtps2
Cyp4x1	Bdp1	Atxn1
Samd14	Aftph	Irgm1
Rab2a	Morn4	Zfp740
Dync2h1	Zbtb14	Tefm
Dusp18	Urgcp	Adck2
Ythdf3	Orai1	Nqo2
Rpl9	Nckap5	Zbtb21
Cdh19	Lig4	Tshz1
Fam185a	Gal3st1	Tapt1
Gm527	Zfp668	Mars2
Mageh1	Trex1	Spsb4
Taf9b	Zfp646	1110032F04Rik
H2bc6	Rpl36al	Zfp41
C2cd3	Zfp672	Fbxo41
Ptgs1	Micos13	Mipol1
Mc4r	Zfp280b	Spata2
Emc6	Tmsb4x	Pcdhb15
Gap43	Fzd4	Zfp445
Zfp358	Bag5	Nipa1
Neurl4	Crh	D030056L22Rik
Pcdhb13	Sertad2	Tmem164
Zfp286	Armcx4	Dusp28
Lancl3	Arhgap23	Ngrn

Gm973
Abhd17b
Zfp768
B4gat1
Atmin
Ctdspl
Zbtb44
Flrt2
Gpr68
Rexo1
AI837181
Dlk2
Xxylt1
Arl4a
Gphn
Dynlrb1
Ark2n
Zfp30
Dlgap2
Rnf152
Mroh7
Baiap3
Tspyl1
Fbxo28
Cltb
Tmem41b
Lxn
Tyw3
Zfp235
Frat2
Ankrd34c
A430005L14Rik
Paxx
Fgfbp3
Mtrfr
Fbxo30
Polr1g
Trpt1
Gal3st3
Msl3l2
Rps8
Yipf6
Champ1
Ust
Ppp1r2
Ubiad1
Bola2
Wbp1l
Rnf150
Zc3hav1l
Lrrc49
Atg16l2
Ankfn1
Fam161a
Zbtb12
Suox
Arl4c
Calhm5
Rlf
2810025M15Rik
Vcpkmt
Rasd1
Tmem17
Rasl11b
Slc35c1
H2ax
Lrrc4
Pgrmc2
BC030500
Rpp38
Ccdc137
Mrps16
Plekhf2
Glt1d1
Ankrd55
Ppp1r3d
Idnk
Pitpnb
Amz1
Rap2c
Tmx2
Tdrp
Zfp697
Grem2
Bex1
Rspry1
1600012H06Rik
Opalin
Sarm1
Kcnk12
Slc25a44
Ubqln2
Mchr1
Eif5a2
Lgr4
Pla2g4e
Snip1
Il17d
Pigm
Hic2
Heatr1
Evc2
Prag1
Dscam
Fzd2
Rictor
Lrrc4b
Tmem198b
Rnf31
Ticam1
Cltc
Cd24a
Zfp654
Dmrta2
Tet1
Khnyn
Cyp4x1
Chst9
Samd14
Rab2a
Dync2h1
Dusp18
Ythdf3
Rpl9
Cdh19
Fam185a
A2ml1
Mageh1
Taf9b
H2bc6
C2cd3
Ptgs1
Emc6
Gap43
Zfp358
Neurl4
Pcdhb13
Zfp286
Lancl3
Gm973
Abhd17b
Zfp768
B4gat1
Atmin
Ctdspl
Zbtb44
Flrt2
Gpr68
Rexo1
Cmya5
AI837181
Dlk2
Xxylt1
Arl4a
Gphn
Dynlrb1
Ark2n
Zfp30
Dlgap2

Phf13	Nsun3	Rnf152
Lix1	Synpo2	Baiap3
Flrt1	Neto1	Tspyl1
Slc38a9	Ndufaf6	Fbxo28
Gjb1	Amer1	Cltb
Akap10	Mir9-2hg	Tmem41b
Ccdc88b	Carmil2	Lxn
Pygo2	Snx21	Tyw3
Cdca4	Septin6	Zfp235
Diras2	Nhsl3	Frat2
Bri3	Armcx6	Ankrd34c
Bex4	Ppp1r3g	A430005L14Rik
Lonp2	Fbxo46	Paxx
Gpr157	Lypd6	Fgfbp3
Usp14	Fam118b	Mtrfr
Rell1	Slc35d3	Fbxo30
Tnrc6b	Pcdh20	Polr1g
Ripply2	Oprd1	Trpt1
Sstr2	Fam171a1	Gal3st3
Tshz2	B230217C12Rik	Msl3l2
Ankrd16	Adra1b	Rps8
Pcdhb16	Fam228b	Rtl3
Trappc9	Lamtor4	Yipf6
Sox1ot	Hyls1	Champ1
Marcksl1	Kcnb1	Ust
Kcna3	Tor1aip2	Ppp1r2
Kcna1	Maml1	Ubiad1
Palm3	Lrrc4c	Bola2
Ino80c	Fam78a	Wbp1l
Dipk1c	Zfp831	Rnf150
Prrg1	Zfp61	Zc3hav1l
Gigyf2	Micos10	Lrrc49
Hes5	Zscan29	Atg16l2
Tmem196	Gpd1l	Phf13
Timm8a1	Ubald2	Lix1
Tmem229a	Tmem150c	Flrt1
Rgmb	Trhde	Slc38a9
Isg20l2	Vstm4	Sned1
Arxes2	Gpatch11	Gjb1
Zbtb33	Ccdc96	Akap10
Ldlrad3	Prkaa1	Ccdc88b
Arf1	2310061I04Rik	Pygo2
Tenm4	Ftl1	Cdca4
Taf13	Scg2	Diras2
4632415L05Rik	Zbtb26	Bri3
Rbm15	Plekho2	Lonp2
Arid4a	Arhgap42	Gpr157
Entpd1	Vamp8	Usp14
Col6a3	Ptges	Rell1
Dmrt2	Pgbd5	Tnrc6b
Nat8l	Htr1f	Ripply2
Nwd1	Ccdc126	Sstr2
Kmt2d	B3galt6	Tshz2

Selplg	Ecpas	Ankrd16
Mcmbp	Fam131a	Pcdhb16
Tmem81	Slc29a4	Trappc9
Asb8	Sstr5	Sox1ot
Bend7	Vwc2	Marcksl1
Gpr85	Cdh20	Gp5
Amigo2	Zfp623	Kcna3
Mfap1b	Tmem125	Kcna1
Fbxo10	Zfp940	Palm3
Rnf149	Atp5me	Ino80c
Gng7	Phospho1	Dipk1c
Crebrf	Minar2	Gigyf2
Bcl11b	Pdik1l	Hes5
Dip2c	Tatdn1	Tmem196
Rbm33	Tvp23a	Timm8a1
Syngr2	Cdr2l	Tmem229a
Sacs	Tmem123	Rgmb
Zfp738	Ankrd37	Isg20l2
Dleu7	Map10	Arxes2
Frmd6	Sgms2	Zbtb33
Slitrk3	Gm42743	Ldlrad3
Ankrd46	Zfp438	Arf1
Pskh1	Amigo1	Tenm4
Ric3	Gja1	Taf13
Lhfpl6	Zfp169	4632415L05Rik
Pcdhb18	Kcns2	Rbm15
Coa7	Prkca	Arid4a
Arxes1	Creg2	Entpd1
Pdp2	Gdpgp1	Nat8l
F2r	Selenon	Nwd1
Socs4	Gatd1	Kmt2d
Scrt1	Hs3st1	Selplg
Fam171b	Zfp11	Mcmbp
Zfp407	Olfml1	Tmem81
Ranbp3l	P4ha3	Asb8
Timm29	Fbll1	Gpr85
Nup42	Mb21d2	Amigo2
Ccdc57	Lingo3	Mfap1b
Inka2	Mblac2	Fbxo10
Spata33	Sv2c	Rnf149
Mypop	Garin5a	Gng7
Bdnf	Camk2n2	Crebrf
Zdhhc22	Adnp	Bcl11b
Zbtb8b	Commd3	Dip2c
Fitm2	Eml5	Rbm33
Nrip1	Rpusd3	Syngr2
Tyw5	Plcb1	Sacs
Mmgt2	Zfp524	Zfp738
Tlcd5	Fam174a	Frmd6
Phldb1	Bzw1	Slitrk3
Tob2	Tmem199	Ankrd46
Thnsl1	Rnf7	Pskh1
Mlec	Swsap1	Ric3

E130311K13Rik	Pcdhb9	Lhfpl6
Igf2	Islr2	Pcdhb18
Gm9828	Msantd1	Coa7
Myof	Jagn1	Arxes1
Nog	Pcmtd1	Pdp2
Ctxn1	Usp42	F2r
Lemd3	Taf7	Socs4
Rhno1	Mtln	Scrt1
Mex3d	Pcdh19	Osr1
Ccdc6	Nup160	Fam171b
Lurap1l	Cacna1c	Gli2
Tprn	Gfod1	Zfp407
Tbc1d12	2900026A02Rik	Ranbp3l
Zfp454	Rab11fip5	Timm29
Klhl11	Plekhm3	Nup42
Mcat	Spryd4	Cyp4f16
Foxo3	Zfp46	Ccdc57
Rpl29	Commd1	Msx1
Tmem53	Ncald	Inka2
Insc	Plpp7	Spata33
Dcun1d3	Pcdh1	Mypop
Cyb561d1	Flrt3	Bdnf
Cep120	Zbtb22	Zdhhc22
Slc35e4	Ywhag	Zbtb8b
Lonrf2	Msantd7	Fitm2
Dact2	Kctd16	Nrip1
Vps37c	Ppp1r37	Tyw5
Slc39a9	Plagl2	Mmgt2
Vstm2a	Ccdc157	Tlcd5
Slc25a47	Fhad1	Phldb1
Arhgap30	Bbs12	Nhlh2
Phf3	Crebzf	Tob2
Hexim1	Zfp24	Thnsl1
Cdk5r1	Cbr1	Mlec
Zfp710	Pcdhb11	E130311K13Rik
Rimkla	Irf2bp2	Igf2
Bnip5	Kcnj16	Morc2b
Rnf24	Zfp786	Gm9828
Efna5	Ufsp1	Myof
Fkrp	Mafg	Nog
Zfp689	Fam181b	Ctxn1
Tada3	Rps19bp1	Lemd3
Atp13a5	Usp29	Rhno1
Gm6206	Gm5124	Tpcn2
Prex2	Zfp579	Mex3d
Mrgpre	Pusl1	Ccdc6
Yjefn3	Tceal8	Lurap1l
C1galt1c1	Mical3	Tprn
Nrsn1	Map3k19	Tbc1d12
Elfn1	Rap2a	Zfp454
Atxn7l2	H1f4	Klhl11
Ndnf	B3gnt2	Mcat
Tmem121	Lrrc3	Foxo3

Mterf2	Coa6	Rpl29
Rapgef4	Dcun1d4	Tmem53
Armcx3	Trim32	P2ry6
Acap2	Pcbp1	Insc
Bmyc	Tmem198	Dcun1d3
Ptgr3	Senp8	Cyb561d1
Sephs2	Wdcp	Cep120
Gpr137c	Kcnf1	Slc35e4
Ankrd34a	Mettl5	Lonrf2
Pcdh10	Xrcc1	Dact2
Dcaf5	Tubgcp6	Pkd1l3
Oxtr	Nlgn2	Vps37c
Fam110b	Sox12	Slc39a9
Frmd3	Rtl8c	Vstm2a
Plcxd3	Arf3	Arhgap30
Zfp518a	Mest	Phf3
Frmpd4	Tbc1d22a	Hexim1
Purg	Klhdc1	Cdk5r1
Rtl5	Sox6	Zfp710
Pdp1	Spats2	Rimkla
Myef2l	B3glct	Bnip5
Tigd2	Xkr4	Rnf24
Lrp1b	Erich1	Efna5
Kcnk3	Smim11	Fkrp
Scn3b	Slc6a7	Zfp689
Mblac1	Tagap1	Tada3
Iba57	Pfdn4	Atp13a5
Lix1l	Klf13	Gm6206
Zfp219	Zfp217	Prex2
Trappc1	Pard3b	Mrgpre
Prmt6	Dock8	Yjefn3
Syt12	Rgs14	C1galt1c1
Ccdc71	Gnpda1	Nrsn1
Sorl1	Mtcl1	Elfn1
Zfp2	Sema5b	Atxn7l2
Smcr8	Foxo6	Ndnf
Kmt5a	Rbm12b2	Tmem121
Tenm2	Babam2	Mterf2
Retreg2	Ppp4r2	Rapgef4
Dcaf7	Rps10	Armcx3
Gemin4	Plpp2	Acap2
Ogfr	Acvr2a	Bmyc
Tmem241	Pld4	Ptgr3
Tmem200a	Opa3	Sephs2
Zfp260	Ppp1r36	Gpr137c
Chchd10	Gpr17	Ankrd34a
Cyp20a1	Zfp622	Pcdh10
Aff4	Dnah3	Dcaf5
Ctu2	5330438D12Rik	Ntf3
Tmem67	Taf9	Oxtr
Ccnq	Ppp6r1	Fam110b
Pls1	Cdc42se2	Frmd3
Dtx3l	Ltn1	Plcxd3

Proser1	Doc2a	Zfp518a
Htr1b	Tbc1d30	Frmpd4
Spty2d1	Hbb-bs	Purg
Rps23	Slc39a1	Rtl5
Cdc42ep1	Ankrd44	Pdp1
Sall2	Cx3cr1	Myef2l
Tceal1	Immt	Tigd2
Adamts16	Cemip	Lrp1b
H1f1	B630019K06Rik	Kcnk3
Clip1	Tmem106c	Scn3b
Fzd9	Il1rapl1	Mblac1
Polr1a	Mpp3	Iba57
Tmie	Actn2	Lix1l
Lingo1	Nrros	Zfp219
Ap5b1	Trpm3	Trappc1
Cfap46	Rft1	Prmt6
Zfpm1	Ezr	Syt12
Tsku	Rexo4	Ccdc71
Grm5	Ccdc171	Sorl1
Zbtb45	Smim30	Zfp2
Zfp644	B4galt3	Smcr8
Omg	Tmco1	Kmt5a
Slc17a5	Bmpr1b	Tenm2
Tifab	Zfp961	Retreg2
C1ql3	Get3	Dcaf7
2310022A10Rik	Atp6v1a	Gemin4
Gpr3	Cherp	Ogfr
Zbtb5	Epha3	Tmem241
Bdp1	Nav2	Tmem200a
Aftph	Robo2	Zfp260
Morn4	Nup188	Chchd10
Zbtb14	Pbx1	Cyp20a1
Urgcp	Magi3	Aff4
Orai1	St6galnac3	Ctu2
Nckap5	Adarb2	Tmem67
Lig4	Gan	Ccnq
Gal3st1	Cpne8	Pls1
Zfp668	H1f3	Dtx3l
Trex1	Hook2	Proser1
Zfp646	Dlg2	Htr1b
Arhgap15	Lrrtm4	Spty2d1
Rpl36al	Serp2	Rps23
Zfp672	Adam17	Cdc42ep1
Micos13	Plekhg3	Sall2
Zfp280b	Pcdh15	Tceal1
Tmsb4x	Asap2	H1f1
Fzd4	Rnf103	Clip1
Bag5	Zfp112	Fzd9
Crh	Zmat1	Polr1a
Sertad2	Rap1b	Tmie
Armcx4	Jun	Lingo1
Arhgap23	Tln2	Ap5b1
Fam161a	Tnrc6a	Cfap46

Zbtb12	BC004004	Zfpm1
Suox	Zfp608	Grm5
Arl4c	Kcnt2	Zbtb45
Calhm5	Map1b	Zfp644
Rlf	Suclg1	Omg
2810025M15Rik	Swt1	Slc17a5
Vcpkmt	Repin1	Tifab
Rasd1	Traf7	C1ql3
Tmem17	Zfp212	2310022A10Rik
Rasl11b	Grk4	Gpr3
Slc35c1	1700030K09Rik	Zbtb5
H2ax	Nup107	Bdp1
Lrrc4	Atad2b	Aftph
Pgrmc2	Sae1	Morn4
BC030500	Junb	Zbtb14
Rpp38	C130026L21Rik	Urgcp
Ccdc137	Reep1	Orai1
Mrps16	Dnah6	Nckap5
Plekhf2	Prkcb	Lig4
Glt1d1	Ubxn8	Gal3st1
Ankrd55	Lamb2	Zfp668
Ppp1r3d	Cyp2j6	Trex1
Idnk	Msl1	Zfp646
Pitpnb	Senp7	Arhgap15
Amz1	Prkg1	Rpl36al
Rap2c	Arhgef15	Zfp672
Tmx2	Rnaseh2a	Micos13
Tdrp	Ctif	Zfp280b
Zfp697	Fbxo31	Tmsb4x
Grem2	Glis3	Fzd4
Bex1	Rnf157	Bag5
Rspry1	Gas1	Sertad2
1600012H06Rik	Mrpl35	Armcx4
Opalin	Ube2ql1	Arhgap23
Sarm1	Uba2	Fam161a
Kcnk12	Hrh1	Zbtb12
Slc25a44	Creb5	Suox
Ubqln2	Krcc1	Arl4c
Mchr1	Cntn2	Calhm5
Lsm10	Sv2b	Rlf
Eif5a2	Aph1c	2810025M15Rik
Lgr4	Brsk2	Vcpkmt
Pla2g4e	Jam2	Rasd1
Snip1	Cfap300	Tmem17
Il17d	Zfta	Rasl11b
Pigm	Lins1	Slc35c1
Gja4	Myh7	Lpar4
Hic2	Tmem248	H2ax
Heatr1	Yap1	Lrrc4
Evc2	Fank1	Pgrmc2
Prag1	Chmp3	BC030500
Dscam	Rnf26	Rpp38
Fzd2	Supt7l	Ccdc137

Rictor	Mapk11	Mrps16
Nsun3	Ptprt	Plekhf2
Synpo2	Spag16	Glt1d1
Neto1	Cdh22	Ankrd55
Ndufaf6	Mllt11	Ppp1r3d
Amer1	Arhgap20	Idnk
Mir9-2hg	Styx	Pitpnb
Carmil2	Gm9899	Amz1
Snx21	Dand5	Rap2c
Septin6	Ndfip2	Tmx2
Kif7	Aldh1a1	Tdrp
Nhsl3	Trmt1l	Zfp697
Armcx6	Ddx10	Grem2
Tcf19	Rab4b	Bex1
Ppp1r3g	Pom121	Rspry1
Fbxo46	AI854703	1600012H06Rik
Lypd6	Nrgn	Agmo
Fam118b	Sec61b	Opalin
Pcdh20	Gatd3a	Vwa3b
Oprd1	Gas5	Sarm1
Fam171a1	Dis3l2	Kcnk12
B230217C12Rik	Ficd	Slc25a44
Adra1b	Zfp952	Ubqln2
Fam228b	Cacng8	Mchr1
Fam241a	Phgdh	Lsm10
Gm11868	Cbx7	Eif5a2
Lamtor4	Hunk	Lgr4
Hyls1	Mapk14	Pla2g4e
Kcnb1	Thoc7	Snip1
Tor1aip2	Ggcx	Il17d
Maml1	Hs6st3	Pigm
Lrrc4c	Kdm3a	Hic2
Fam78a	Tcf4	Heatr1
Zfp831	Usp21	Evc2
Zfp61	Nrdc	Prag1
Micos10	Kcnip1	Dscam
Zscan29	Cstf2t	Fzd2
Gpd1l	Shisa7	Foxc1
Ubald2	Ebf4	Rictor
Tmem150c	3110082I17Rik	Nsun3
Trhde	Ier2	Synpo2
Vstm4	Eif3k	Neto1
Gpatch11	Tanc2	Ndufaf6
Prkaa1	Zfand2a	Amer1
2310061I04Rik	Rpia	Mir9-2hg
Ftl1	Sh3pxd2a	Carmil2
Scg2	Dennd4a	Snx21
Zbtb26	Aldh7a1	Septin6
Plekho2	Plxnb1	Kif7
Arhgap42	Kics2	Nhsl3
Vamp8	Mast1	Armcx6
Ptges	Nebl	Ppp1r3g
Pgbd5	Dusp7	Fbxo46

Htr1f	Tmem39b	Lypd6
Ccdc126	Ptrh1	Fam118b
B3galt6	Chd8	Pcdh20
Ecpas	Chchd3	Oprd1
Fam131a	Lysmd1	Fam171a1
Slc29a4	Ubxn7	B230217C12Rik
Vwc2	Exoc6	Adra1b
Cdh20	Grwd1	Fam228b
Zfp623	Camk2d	Fam241a
Tmem125	Ppfia2	Gm11868
Zfp940	Nudcd3	Lamtor4
Atp5me	Txlna	Hyls1
Phospho1	Fpgt	Kcnb1
Minar2	Srcap	Tor1aip2
Pdik1l	Ech1	Maml1
Tatdn1	Mat2a	Lrrc4c
Rtn4rl2	Nanp	Fam78a
Tvp23a	Zftraf1	Zfp831
Cdr2l	Shisa6	Zfp61
Tmem123	Cnn3	Micos10
Ankrd37	Adnp2	Zscan29
Dcaf12l2	Stum	Catsperz
Map10	Zfp14	Gpd1l
Gm42743	Tusc1	Ubald2
Efcab5	Ndst1	Tmem150c
Zfp438	Tmem179	Trhde
Amigo1	Sirt5	Vstm4
Gja1	Nt5dc3	Gpatch11
Zfp169	Tceal5	Prkaa1
Kcns2	Ercc6	2310061I04Rik
Prkca	Skida1	Ftl1
Creg2	Utp18	Scg2
Gdpgp1	1810037I17Rik	Zbtb26
Selenon	Slc25a40	Plekho2
Fhip1a	Skp2	Arhgap42
Gatd1	Syne3	Vamp8
Hs3st1	Spock3	Ptges
Zfp11	Gon4l	Pgbd5
Olfml1	Tprkb	Htr1f
Gprc5c	Fra10ac1	Ccdc126
P4ha3	Fgfr3	B3galt6
Fbll1	Msi1	Ecpas
Mb21d2	Lifr	Fam131a
Lingo3	Arfgap3	Slc29a4
Mblac2	Prr14l	Vwc2
Sv2c	Eapp	Cdh20
Garin5a	Cpsf3	Zfp623
Camk2n2	Mrps21	Tmem125
Adnp	Taf4b	Zfp940
Commd3	Rhob	Atp5me
Eml5	Zfp747	Phospho1
Rpusd3	Pnma1	Minar2
Plcb1	Mdm4	Pdik1l

Zfp524	Cklf	Tatdn1
Fam174a	Dnajc8	Rtn4rl2
Bzw1	Spcs3	Tvp23a
Tmem199	Tmem74	Cdr2l
Rnf7	Slc30a7	Tmem123
Msrb3	Cadps	Ankrd37
Swsap1	A930005H10Rik	Zfp366
Pcdhb9	Atp5if1	Map10
Islr2	Tmem120b	Gm42743
Msantd1	Tle5	Efcab5
Gm6563	Sytl5	Zfp438
Jagn1	Vapb	Amigo1
Pcmtd1	Vsnl1	Gja1
Usp42	Lclat1	Zfp169
Taf7	Kcnn2	Kcns2
Mtln	Tmem62	Prkca
Pcdh19	Dedd2	Creg2
Nup160	Parp4	Gdpgp1
Cacna1c	Trim65	Selenon
Gfod1	Zfp867	Fhip1a
2900026A02Rik	Sh3bp2	Gatd1
Zfp52	Adam12	Hs3st1
Rab11fip5	Cggbp1	Zfp11
Plekhm3	Kdm2a	Olfml1
Spryd4	Tmt1a	Gprc5c
Zfp46	Slc8a1	P4ha3
Commd1	Zfp869	Fbll1
Ncald	Irs4	Mb21d2
Plpp7	Tmem119	Lingo3
Pcdh1	1600014C10Rik	Mblac2
Flrt3	Srsf12	Sv2c
Zbtb22	Adam10	Garin5a
Ywhag	Ankrd24	Ghsr
Msantd7	Zscan22	Camk2n2
Kctd16	Zfp771	Adnp
Ppp1r37	Hmgb2	Commd3
Plagl2	Lrrc8c	Eml5
Ccdc157	Vmac	Rpusd3
Fhad1	Phactr1	Plcb1
Bbs12	Msra	Zfp524
Crebzf	Fsd1l	Fam174a
Zfp24	Set	Bzw1
Cbr1	Kctd18	Tmem199
Pcdhb11	Klhl18	Rnf7
Irf2bp2	Cadm4	Msrb3
Kcnj16	Actn4	Swsap1
Zfp786	Usp46	Pcdhb9
Ufsp1	Nsd3	Islr2
Siglech	Elp6	Msantd1
Mafg	Atrnl1	Nhlh1
Fam181b	Smim10l2a	Jagn1
Rps19bp1	Rnd1	Zgrf1
Usp29	Tafa5	Pcmtd1

Gm5124
Zfp579
Pusl1
Tceal8
Mical3
Map3k19
Pcdhb2
Rap2a
Krt9
H1f4
B3gnt2
Lrrc3
Coa6
Dcun1d4
Trim32
Pcdhb6
Pcbp1
Tmem198
Senp8
Wdcp
Kcnf1
Mettl5
Fam229b
Xrcc1
Tubgcp6
Nlgn2
Sox12
Rtl8c
Arf3
Mest
Tbc1d22a
Klhdc1
Sox6
Rspo2
Spats2
B3glct
Xkr4
Erich1
Igfn1
Smim11
Slc6a7
Tagap1
Pfdn4
Klf13
Pard3b
Dock8
Rgs14
Gnpda1
Mtcl1
F730043M19Rik
Sema5b
Foxo6
Rbm12b2
Tmem158
Pcnx3
Zfp667
Dmac2l
Klhl5
Zkscan4
Kcnmb4
Zfp174
Miga1
Nt5c1a
Zfp647
Nyap2
Lrtm2
Agap1
Cntn1
Ep300
Gabrg3
Commd5
Nfic
Ddx17
Smyd3
Gabra5
Bmal1
Cgrrf1
Klf2
Zfp811
Ankrd17
Pld5
Kcmf1
Zfp101
Ntrk2
Tmem245
Mrfap1
Zfp93
Sec23ip
Tead1
Tns1
Snupn
Stam2
Fut9
Fbxo6
Map6
Nell1
Pcdh9
Nap1l5
Maf
Srsf11
Cd47
Alk
Zfp458
Mtcl2
Pprc1
Epm2a
Usp42
Taf7
Mtln
Pcdh19
Nup160
Cacna1c
Gfod1
2900026A02Rik
Zfp52
Rab11fip5
Plekhm3
Spryd4
Zfp46
Commd1
Ncald
Plpp7
Pcdh1
Flrt3
Zbtb22
Ywhag
Msantd7
Kctd16
Ppp1r37
Plagl2
Ccdc157
Bbs12
Crebzf
Zfp24
Cbr1
Pcdhb11
Irf2bp2
Kcnj16
Zfp786
Ufsp1
Siglech
Mafg
Fam181b
Rps19bp1
Usp29
Gm5124
Zfp579
Pusl1
Tceal8
Mical3
Map3k19
Pcdhb2
Rap2a
Krt9
H1f4
B3gnt2
Lrrc3
Coa6
Dcun1d4

Babam2	Cpsf6	Trim32
Rasal3	Zcchc24	Pcdhb6
Ppp4r2	Epha6	Pcbp1
Rps10	Kxd1	Tmem198
Plpp2	Unc80	Senp8
Acvr2a	Cdca7	Wdcp
Pld4	B4galnt4	Kcnf1
Opa3	Zfp580	Mettl5
Gpr17	Dach1	Xrcc1
Zeb2os	Klhl25	Iqcj
Zfp622	Gpc3	Tubgcp6
5330438D12Rik	Mettl4	Nlgn2
Taf9	Zzef1	Sox12
Ppp6r1	Kbtbd11	Rtl8c
Cdc42se2	Cope	Arf3
Ltn1	Tmem191	Mest
Doc2a	Klhl26	Tbc1d22a
Tbc1d30	Slain1	Klhdc1
Hbb-bs	Ubl7	Sox6
Slc39a1	Rras2	Rspo2
Ankrd44	Paqr3	Spats2
Cx3cr1	Nap1l3	B3glct
Immt	Ghr	Xkr4
Cemip	Rtl6	Erich1
B630019K06Rik	Nkain3	Smim11
Tmem106c	Eef1d	Slc6a7
Il1rapl1	Abcd2	Tagap1
Mpp3	Fmnl1	Pfdn4
Actn2	Dnaaf3	Klf13
Nrros	Mta3	Zfp217
Trpm3	Zfp1	Pard3b
Rft1	Elob	Dock8
Ezr	Rnf181	Rgs14
Rexo4	Izumo4	Gnpda1
Ccdc171	Per2	Mtcl1
Smim30	Fancm	F730043M19Rik
B4galt3	Ppp4r1l-ps	Sema5b
Tmco1	Tmem69	Foxo6
Bmpr1b	Zfp277	Rbm12b2
Zfp961	Aasdh	Babam2
Get3	Fto	Rasal3
Atp6v1a	Emc7	Ppp4r2
A330008L17Rik	Prr18	Rps10
Cherp	Triqk	Plpp2
Epha3	Irs1	Acvr2a
Nav2	Zkscan5	Pld4
Robo2	Elapor2	Opa3
Nup188	A430033K04Rik	Gpr17
Pbx1	Zfp709	Zeb2os
Magi3	Mia3	Zfp622
St6galnac3	Otulinl	5330438D12Rik
Adarb2	Grik2	Taf9
Gan	Eif3b	Ppp6r1

Cpne8	St3gal5	Cdc42se2
D930048N14Rik	Fez2	Ltn1
H1f3	B4galt6	Doc2a
Hook2	Pgm3	Tbc1d30
Dlg2	AI504432	Hbb-bs
Lrrtm4	Socs6	Slc39a1
Serp2	Car10	Ankrd44
Adam17	Cnot10	Cx3cr1
Plekhg3	Col8a2	Immt
Pcdh15	Snx32	Cemip
Gm9885	Cfl1	B630019K06Rik
Asap2	Pgpep1	Tmem106c
Rnf103	Npm3	Il1rapl1
Zfp112	R3hdm1	Mpp3
Zmat1	Pard6g	Actn2
Rap1b	Lrguk	Nrros
Jun	Cebpg	Trpm3
Tln2	Spock1	Acot4
Tnrc6a	Cars2	Rft1
BC004004	Ncoa4	Ezr
Zfp608	Kcnq3	Rexo4
Kcnt2	Lrif1	Ccdc171
Map1b	Cep70	Tchh
Suclg1	Dennd1b	Smim30
Swt1	Synpr	B4galt3
Repin1	Usp39	Tmco1
Traf7	Sertm1	Bmpr1b
Gpr25	Tyw1	Zfp961
Zfp212	Usp34	Get3
Grk4	Actr3b	Atp6v1a
1700030K09Rik	AI987944	A330008L17Rik
Nup107	Lig1	Cherp
Atad2b	Adap1	Epha3
Sae1	Slit3	Nav2
Junb	Tgoln1	Robo2
C130026L21Rik	Mok	Nup188
Reep1	Zbtb25	Pbx1
Dnah6	Med12l	Magi3
Prkcb	Chn1	St6galnac3
Ubxn8	Adgrf5	Adarb2
Lamb2	Foxk1	Gan
Cyp2j6	Rab31	Cpne8
Msl1	Pign	H1f3
Senp7	Rlim	Hook2
Prkg1	Ptprn2	Dlg2
Arhgef15	Tug1	Lrrtm4
Rnaseh2a	Zfp175	Serp2
Ctif	Trnp1	Adam17
Fbxo31	Drc3	Isoc2b
Glis3	Fry	Plekhg3
Rnf157	Chd9	Pcdh15
Gas1	Ppp1r14b	Gm9885
Mrpl35	Fkbp2	Asap2

Ube2ql1
Uba2
Hrh1
Creb5
Krcc1
Cntn2
Sv2b
Aph1c
Brsk2
Jam2
Cfap300
Zfta
Lins1
Myh7
Tmem248
Yap1
Fank1
Socs3
Chmp3
Rnf26
Supt7l
Mapk11
Ptprt
Fes
Gpr21
Cdh22
Mterf1b
Mllt11
Arhgap20
Gm9899
Dand5
Ndfip2
Gm12592
Aldh1a1
Trmt1l
Ddx10
Rab4b
Pom121
AI854703
Nrgn
Sec61b
Gatd3a
Gas5
Dis3l2
Ficd
Zfp943
Zfp952
Cacng8
Phgdh
Adamts18
Cbx7
Hunk
Mapk14
Them6
Retsat
Gpr173
Ilrun
Elmod3
Ier5
Nbeal2
Nfil3
Dnah9
Grm7
Cspp1
Setd3
St8sia3
Tsnax
Ift56
Pcbp2
Pou3f4
Jakmip3
Gulp1
H2bc27
Usp13
Sipa1
Cep162
Acbd4
Commd7
Tatdn2
Jmjd6
Gjc3
Magel2
Ide
Slc35f3
Ero1b
Zfp607b
Ebf1
Zfp180
Nat8f1
Cntrl
Npm1
Txnl4a
Chd6
Ado
Dph6
Homez
Cfap119
Gsk3a
5730455P16Rik
Scn3a
Slc44a2
Armc7
Dmac2
Aak1
Rbbp4
Snrpg
Rnf103
Zfp112
Zmat1
Rap1b
Jun
Tln2
Tnrc6a
BC004004
Zfp608
Kcnt2
Map1b
Suclg1
Swt1
Repin1
Traf7
Gpr25
Zfp212
Grk4
1700030K09Rik
Nup107
Atad2b
Sae1
Junb
C130026L21Rik
Reep1
Prkcb
Ubxn8
Lamb2
Cyp2j6
Msl1
Senp7
Prkg1
Arhgef15
Rnaseh2a
Ctif
Fbxo31
Glis3
Rnf157
Gas1
Mrpl35
Ube2ql1
Uba2
Hrh1
Creb5
Krcc1
Cntn2
Sv2b
Aph1c
Brsk2
Jam2
Cfap300
Zfta
Lins1

Thoc7
Ggcx
Hs6st3
Kdm3a
Tcf4
Usp21
Nrdc
Kcnip1
Cstf2t
Shisa7
Ebf4
3110082I17Rik
Ier2
Eif3k
4930563E22Rik
Tanc2
Zfand2a
Zfp472
Rpia
Sh3pxd2a
Dennd4a
Aldh7a1
Plxnb1
Kics2
Mast1
Nebl
4732471J01Rik
Dusp7
Tmem39b
Ptrh1
Chd8
Chchd3
Lysmd1
Ubxn7
Exoc6
Grwd1
Camk2d
Ppfia2
Nudcd3
Txlna
Lipg
Fpgt
Srcap
Ech1
Mat2a
Kdm4d
Nanp
Zftraf1
Shisa6
Cnn3
Adnp2
Stum
Pde5a
Arhgap24
Rpl38
Bcl2
Cep170
Chst3
Sphk2
Gm17494
Uxs1
Birc2
Yipf1
Ryr3
Mrpl18
Nsd2
Zfp53
Antkmt
Las1l
Mpp7
Rit2
E2f6
Fam136a
Bloc1s2
Spop
Ece1
Dtnbp1
Pus7
Zfp317
Lgals8
Eif1a
Zbtb8os
Arl16
Gnai1
Prdm2
Brd9
Gapdh
Bloc1s3
Pkn1
Zfp746
Mex3b
A830018L16Rik
Tmem178b
Sptan1
Megf6
Ankrd29
Cyb5d2
Ddx49
Bak1
Rpl32
Fam204a
Rpl36
Abat
Zfp329
Zfp105
Camk2b
Tmem248
Yap1
Chmp3
Rnf26
Supt7l
Mapk11
Ptprt
Fes
Gpr21
Cdh22
Mterf1b
Mllt11
Arhgap20
Styx
Gm9899
Dand5
Ndfip2
Aldh1a1
Trmt1l
Ddx10
Rab4b
Pom121
AI854703
Nrgn
Sec61b
Gatd3a
Gas5
Dis3l2
Ficd
Zfp943
Zfp952
Cacng8
Phgdh
Adamts18
Cbx7
Hunk
Mapk14
Thoc7
Ggcx
Hs6st3
Kdm3a
Tnfaip6
Tcf4
Usp21
Nrdc
Kcnip1
Cstf2t
Shisa7
Ebf4
3110082I17Rik
Ier2
Eif3k
Tanc2

Zfp14	Cacnb2	Zfand2a
Tusc1	Itpk1	Zfp472
Ndst1	Zfp809	Rpia
Tmem179	Mdn1	Sh3pxd2a
Sirt5	Septin11	Tll1
Nt5dc3	Palld	Dennd4a
Tceal5	Eml1	Aldh7a1
Ercc6	Sdhc	Plxnb1
A930004D18Rik	Gstm1	Kics2
Skida1	Adamts17	Mast1
Utp18	Chsy3	Nebl
1810037I17Rik	Sez6l	4732471J01Rik
Slc25a40	Zfp846	Dusp7
Skp2	Arhgap35	Tmem39b
Spock3	Usf2	Ptrh1
Gon4l	Cryzl1	Chd8
Tprkb	Kcnh1	Chchd3
Fra10ac1	Tspan7	Lysmd1
Fgfr3	Idi1	Ubxn7
Msi1	Serpina9	Exoc6
Lifr	Mrps14	Grwd1
Arfgap3	Zfp68	Camk2d
Prr14l	Spock2	Ppfia2
Eapp	Mcm9	Nudcd3
Cpsf3	Upf1	Txlna
Mrps21	Ube2e2	Lipg
Taf4b	Phf21a	Fpgt
Rhob	Dock1	Srcap
Zfp747	Zfp85	Slc39a8
Pnma1	Abce1	Ech1
Mdm4	H2bc8	Mat2a
Cklf	Phtf1	Nanp
Slfn5	Rrp1b	Zftraf1
Dnajc8	Zfp420	Shisa6
Spcs3	Txndc9	Cnn3
Tmem74	Syt17	Adnp2
Slc30a7	Nrf1	Stum
Cadps	Panx2	Pde5a
A930005H10Rik	Map2k5	Zfp14
Atp5if1	Znrf2	Tusc1
Tmem120b	Dhcr7	Ndst1
Tle5	Wdr91	Tmem179
Sytl5	Kl	Sirt5
Vapb	Fam133b	Nt5dc3
9430021M05Rik	Rpl23a	Tceal5
Vsnl1	Rpl5	Ercc6
Lclat1	Tmed9	A930004D18Rik
Thnsl2	Gpc6	Skida1
Kcnn2	Serhl	Utp18
Tmem62	Tmod3	1810037I17Rik
Gm9947	Anks1b	Slc25a40
Dedd2	Fbh1	Skp2
Trim65	Rpl30	Syne3

Zfp867
Sh3bp2
Cggbp1
Kdm2a
Tmt1a
Slc8a1
Zfp869
Pm20d2
Tmem119
1600014C10Rik
Srsf12
Adam10
Ankrd24
Zscan22
Zfp771
Hmgb2
Lrrc8c
Vmac
Phactr1
Msra
Zfp182
Fsd1l
Set
Kctd18
Klhl18
Cadm4
Actn4
Usp46
Nsd3
Elp6
Atrnl1
Smim10l2a
Rnd1
Tafa5
Tmem158
Pcnx3
Zfp667
Dmac2l
4931415C17Rik
Klhl5
Zkscan4
Kcnmb4
Zfp174
Miga1
Nt5c1a
Zfp647
Nyap2
Lrtm2
Agap1
Cntn1
Ep300
Gabrg3
Commd5
Gda
Zfp110
Eif4b
Samd12
Tubb2a
Ccser2
Memo1
0610030E20Rik
Egln2
Kcnt1
Zfp958
Thra
Rnf169
H1f5
Cds2
Nap1l1
Rex1bd
Abi1
Zfp516
Deaf1
Col25a1
Tlcd3b
Kcnc1
Hdhd5
Vwa8
Grin2a
Hnrnpa3
Sh2d3c
Eno1b
Frem1
Rpl18
Nap1l4
Zfp945
Ntrk3
Mfsd4a
Pde1a
Skap2
Mtfmt
Tafa1
Il1rapl2
Hnrnpm
Ddn
Septin9
Usp47
Zc3h4
R74862
Naa38
Cdyl
Rpl11
Slc27a4
Tonsl
Hopx
Csf2ra
Spock3
Gon4l
Tprkb
Fra10ac1
Fgfr3
Msi1
Lifr
Arfgap3
Prr14l
Eapp
Cpsf3
Mrps21
Taf4b
Rhob
Zfp747
Pnma1
Mdm4
Cklf
Slfn5
Dnajc8
Spcs3
Tmem74
Slc30a7
Cadps
A930005H10Rik
Atp5if1
Tmem120b
Tle5
Sytl5
Vapb
Vsnl1
Lclat1
Thnsl2
Kcnn2
Tmem62
Dedd2
Parp4
Trim65
Zfp867
Sh3bp2
Adam12
Cggbp1
Kdm2a
Tmt1a
Slc8a1
Zfp869
Pm20d2
Tmem119
1600014C10Rik
Srsf12
Adam10
Ankrd24
Zscan22

Pdlim1	Eda	Zfp771
Nfic	Slc14a1	Lrrc8c
Ddx17	Wdr83os	Vmac
Smyd3	Nrsn2	Phactr1
Gabra5	Mamld1	Msra
Bmal1	Ppp2r5d	Zfp182
M5C1000I18Rik	Zfp933	Fsd1l
Cgrrf1	Max	Set
Klf2	Bcas3	Kctd18
Zfp78	Hadhb	Klhl18
Zfp811	Ptk2b	Cadm4
Ankrd17	Mbtd1	Actn4
Pld5	Zfp426	Usp46
Kcmf1	Cfap418	Nsd3
Zfp101	Kbtbd2	Elp6
Ntrk2	Nhs	Atrnl1
Tmem245	Arhgef12	Smim10l2a
Mrfap1	Znhit1	Rnd1
Zfp93	Uqcr10	Tafa5
Pgbd1	Tcea2	Tmem158
Sec23ip	Trp53	Pcnx3
Tead1	Ccdc28a	Dsp
Tns1	Nsmce2	Zfp667
Snupn	Syn3	Dmac2l
Stam2	1500035N22Rik	Klhl5
Fut9	Taf1b	Zkscan4
Fbxo6	Cdh24	Kcnmb4
Map6	Zfp637	Zfp174
Nell1	Akap17b	Miga1
Pcdh9	Rcan3	Nt5c1a
Nap1l5	Flot1	Zfp647
Maf	Ndufs8	Nyap2
Srsf11	Kcnh7	Lrtm2
Cd47	Fdps	Agap1
Gm7353	Slx1b	Cntn1
Alk	Eif4a1	Ep300
Zfp458	Rgs3	Gabrg3
Mtcl2	Atl2	Commd5
Pprc1	Nkapd1	Pdlim1
Epm2a	Dbp	Nfic
Gucy2g	Sclt1	Ddx17
Cpsf6	Zfp874b	Smyd3
Zcchc24	Zfp341	Gabra5
Epha6	Kmt5c	Bmal1
Kxd1	Kcng2	M5C1000I18Rik
Zfp459	Hydin	Cgrrf1
Unc80	Ntng1	Klf2
B4galnt4	Tnip2	Zfp78
Zfp580	Zfp422	Zfp811
Dach1	Ube4a	Ankrd17
Klhl25	Ptp4a3	Kcmf1
Gpc3	Zfp930	Zfp101
Mettl4	4930453N24Rik	Ntrk2

Zzef1
Kbtbd11
Cope
Tmem191
Klhl26
Slain1
Ubl7
Rras2
Paqr3
Nap1l3
Ghr
Rtl6
Gemin6
Nkain3
Eef1d
Abcd2
Tcf7l1
Fmnl1
Mta3
1700034H15Rik
Zfp1
Elob
Rnf181
Izumo4
Per2
Fancm
Ppp4r1l-ps
Tmem69
Tmem150a
Zfp277
Aasdh
Fto
Emc7
Prr18
Triqk
Irs1
Zkscan5
Elapor2
A430033K04Rik
Zfp709
Mia3
Otulinl
Grik2
Eif3b
St3gal5
Fez2
B4galt6
Pgm3
AI504432
Socs6
Car10
Cnot10
Snx32
Unc5c
Grb2
9430015G10Rik
Hspa2
Ntm
Zfp74
Taok2
Nptx2
Atxn7l3
Chpt1
Kif13b
H2aj
Rpl3
Dhps
Psma3
Rp2
H4c1
Prmt7
Gemin2
Tpt1
Gm10076
Serpinb6a
Pop5
Zdhhc8
Kif27
Slc35e3
Zfp462
Pcnx2
Arrb2
Golm2
Cend1
Scrt2
Pwwp2b
Gtf2i
Nrg2
Ap2a1
Ppih
2610008E11Rik
Zfp941
Caln1
Hnrnpc
Bckdha
C030014I23Rik
Zfp128
Chst8
Pantr1
Zfp868
Sntb1
Rnf14
Wtap
Irak2
Samd5
Zfp266
Tmem245
Mrfap1
Zfp93
Pgbd1
Sec23ip
Tead1
Tns1
Snupn
Stam2
Fut9
Fbxo6
Map6
Nell1
Pcdh9
Nap1l5
Maf
Srsf11
Cd47
Gm7353
Alk
Zfp458
Mtcl2
Pprc1
Epm2a
Gucy2g
Cpsf6
Zcchc24
Epha6
Kxd1
Unc80
B4galnt4
Zfp580
Dach1
Klhl25
Gpc3
Mettl4
Zzef1
Kbtbd11
Gm5069
Cope
Tmem191
Klhl26
Slain1
Ubl7
Rras2
Paqr3
Nap1l3
Ghr
Rtl6
Nkain3
Eef1d
Abcd2
Tcf7l1

Cfl1
Pgpep1
Npm3
R3hdm1
Pard6g
Lrguk
Cebpg
Spock1
Cars2
Ncoa4
Kcnq3
Lrif1
Cep70
Dennd1b
Synpr
Usp39
Sertm1
Tyw1
Usp34
Actr3b
AI987944
Lig1
Adap1
Slit3
Tgoln1
Mok
Zbtb25
Med12l
Cd248
Chn1
Adgrf5
Foxk1
Cebpb
Rab31
Pign
Rlim
Ptprn2
Tug1
Zfp175
Trnp1
Drc3
Fry
Chd9
Ppp1r14b
Fkbp2
Apol8
Them6
Retsat
Gpr173
Ilrun
Elmod3
Ier5
Nbeal2
Tor3a
A930017M01Rik
Dcc
Tmem219
Tnfrsf19
Fam78b
Eno3
Nr1h2
Rpl35a
H4c9
Marf1
Atp8b2
Plaat3
H4c3
Mrps9
Slc9a6
Cd302
Bloc1s4
Plekhh1
Ipmk
Rxfp3
Nsa2
H3f3a
Tsga10
Lrrtm1
Intu
B2m
Gstp1
Ube2s
Zbtb40
Kcnd2
Arl1
Csmd1
Tmem263
Rpl26
Trmt61a
Slc4a4
H4c8
Galnt13
Copz1
Mkx
Rrs1
Clasrp
Rrp1
Haghl
Lsamp
Mcrip1
Dnajc30
Prcp
Ppm1b
Prpf40a
Maml3
Fnip2
Fmnl1
Mta3
1700034H15Rik
Zfp1
Elob
Rnf181
Izumo4
Per2
Fancm
Ppp4r1l-ps
Tmem69
Tmem150a
Zfp277
Aasdh
Fto
Emc7
Prr18
Triqk
Irs1
Zkscan5
Elapor2
A430033K04Rik
Zfp709
Mia3
Otulinl
Grik2
Eif3b
St3gal5
Fez2
B4galt6
Pgm3
AI504432
Socs6
Car10
Cnot10
Snx32
Cfl1
Pgpep1
Npm3
R3hdm1
Pard6g
Lrguk
Cebpg
Spock1
Cars2
Ncoa4
Kcnq3
Lrif1
Cep70
Dennd1b
Synpr
Usp39
Sertm1

Nfil3
C330011M18Rik
Grm7
Cspp1
Setd3
Gm10010
St8sia3
Tsnax
Ift56
Pcbp2
Pou3f4
Jakmip3
H2bc27
Usp13
Sipa1
Cep162
Acbd4
Commd7
Tatdn2
Jmjd6
Gjc3
Ide
Slc35f3
Ero1b
Zfp607b
Zfp180
Nat8f1
Cntrl
Npm1
Txnl4a
Chd6
Ado
Dph6
Homez
Cfap119
Gsk3a
5730455P16Rik
Scn3a
Slc44a2
Armc7
Dmac2
Aak1
Mettl15
Rbbp4
Bbof1
Snrpg
Arhgap24
Rpl38
Bcl2
Cep170
Sphk2
Gm17494
Uxs1
Sfmbt2
Stk19
H2-K1
Exoc5
Mmgt1
Exosc5
Taok3
Agbl4
Slc38a10
Ddhd2
Naca
Dnai1
Cxcl12
Phf5a
Zfp873
Fiz1
Acvr2b
Zcchc14
Nol4l
Hipk2
Tmem151a
Stx17
Nol10
Smim20
Obscn
Mrps36
Rps7
Snrpa
H4c4
Sox21
Cox5b
Zic2
Cep128
Sec22c
Skic8
Dpp6
Ksr2
Dot1l
Pclo
Akap6
Mdc1
U2af1
Med9
Cd2ap
Gdpd1
Dlgap4
Tmem91
Ppp1r1b
Gm2423
Ext1
Cyp2d22
Kalrn
Bod1l
Tyw1
Usp34
Actr3b
AI987944
Lig1
Adap1
Slit3
Tgoln1
Mok
Zbtb25
Med12l
Cd248
Chn1
Adgrf5
Foxk1
Cebpb
Rab31
Pign
Rlim
Ptprn2
Tug1
Zfp175
Trnp1
Drc3
Fry
Chd9
Ppp1r14b
Fkbp2
Apol8
Them6
Retsat
Gpr173
Ilrun
Elmod3
Ier5
Nbeal2
A930024E05Rik
Nfil3
Dnah9
C330011M18Rik
Grm7
Cspp1
Setd3
Gm10010
St8sia3
Tsnax
Ift56
Pcbp2
Pou3f4
Jakmip3
H2bc27
Usp13
Sipa1

Birc2	Akr1b10	Cep162
Yipf1	Armt1	Acbd4
Ryr3	Tac1	Commd7
Mrpl18	Mospd2	Tatdn2
Zfp759	Rps17	Jmjd6
Nsd2	Suclg2	Gjc3
Zfp53	Patj	Ide
Antkmt	Ssbp3	Slc35f3
Las1l	Zscan20	Ero1b
Mpp7	Rbak	Zfp607b
Rit2	Slc25a3	Ebf1
E2f6	Myt1l	Zfp180
Fam136a	Ppp4r1	Nat8f1
Bloc1s2	Rcc1l	Cntrl
Spop	Flot2	Npm1
Ece1	Rps12	Txnl4a
Dtnbp1	Rpl34	Chd6
Pus7	Zfp13	Ado
Zfp317	Gmfb	Dph6
Lgals8	Pgghg	Homez
Eif1a	Zfp27	Cfap119
Zbtb8os	Lmtk3	Gsk3a
Arl16	Iah1	5730455P16Rik
Lmcd1	Pgk1	Scn3a
Gnai1	Lmnb2	Slc44a2
Prdm2	Qki	Armc7
Brd9	Btbd3	Dmac2
Rps18-ps3	Scfd2	Aak1
Gapdh	Rai1	Mettl15
Bloc1s3	Zfp954	Rbbp4
Pkn1	Cttnbp2nl	Bbof1
Zfp746	Unc13c	Snrpg
Mex3b	Cnih4	Arhgap24
A830018L16Rik	Hs6st2	Rpl38
Tmem178b	Lancl2	Bcl2
Sptan1	2700097O09Rik	Cep170
Ankrd29	Btbd9	Chst3
Cyb5d2	Gspt1	Sphk2
Ddx49	Erbb4	Gm17494
Bak1	Rapgef2	Uxs1
Zfp119a	Gak	Birc2
Rpl32	Lhfpl5	Yipf1
Fam204a	Opcml	Ryr3
Rpl36	Morf4l1	Mrpl18
Abat	Trank1	Zfp759
Zfp329	Nectin2	Nsd2
Zfp105	Rpp25	Zfp53
Camk2b	Erbb2	Antkmt
Cacnb2	Rpl17	Las1l
Itpk1	Itgb1bp1	Mpp7
Zfp809	Gm15772	Rit2
Mdn1	Otof	E2f6
Septin11	Tmem65	Fam136a

Palld	Borcs7	Bloc1s2
Eml1	Tubb3	Spop
Sdhc	Dgkk	Ece1
Zfp729b	Zfp706	Dtnbp1
Tpm3-rs7	Arf2	Pus7
Gstm1	Ap3b2	Zfp317
Adamts17	Acat3	Lgals8
Chsy3	Zfp398	Eif1a
Sez6l	Mppe1	Zbtb8os
Zfp846	Syt9	Arl16
Arhgap35	Timm17a	Lmcd1
Usf2	Armc9	Gnai1
Cryzl1	Tubb4a	Prdm2
Kcnh1	Srpk2	Brd9
Tspan7	Mysm1	Gapdh
Idi1	Ganc	Bloc1s3
Mrps14	Rpl7a	Pkn1
Zfp68	Ncs1	Zfp746
Spock2	Atp5mc2	Mex3b
Mcm9	Cebpzos	A830018L16Rik
Upf1	H2bc12	Tmem178b
Ube2e2	Ppox	Sptan1
Phf21a	Smim29	Megf6
Dock1	Shisal1	Ankrd29
Zfp85	Zfp512	Cyb5d2
Uqcc5	Ei24	Ddx49
Abce1	Kcnc3	Bak1
H2bc8	Hikeshi	1600029O15Rik
Phtf1	4833420G17Rik	Zfp119a
Rrp1b	Actg1	Rpl32
Zfp420	Zfp28	Fam204a
Txndc9	Phactr2	Rpl36
Syt17	Impdh2	Abat
Nrf1	Klhl24	Zfp329
Panx2	Hdac10	Zfp105
Map2k5	Acadm	Camk2b
Znrf2	Cfl2	Cacnb2
Dhcr7	Zfp938	Itpk1
Wdr91	Gm10123	Sema3b
Kl	Mtap	Zfp809
Fam133b	Zfp696	Mdn1
Rpl23a	Atp11c	Septin11
Rpl5	Kdr	Palld
Tmed9	Ccdc177	Eml1
Gpc6	Ufc1	Sdhc
Serhl	Mrpl42	Zfp729b
Tmod3	Nrg1	Tpm3-rs7
Anks1b	Ica1	Gstm1
Fbh1	Rpl35	Adamts17
Rpl30	Ccni	Chsy3
Gda	2010204K13Rik	Sez6l
Zfp110	Manbal	Smco4
Eif4b	H2ac15	Zfp846

Samd12	Zfp780b	Arhgap35
Tubb2a	Ing2	Usf2
Ccser2	Lrrc40	Cryzl1
Memo1	Ctnna2	Kcnh1
0610030E20Rik	Mapk3	Tspan7
Egln2	Kif1b	Idi1
Lin9	Zeb1os1	Mrps14
Kcnt1	Zfp26	Zfp68
Zfp958	Kcnma1	Spock2
Thra	Bbs5	Mcm9
Rnf169	Clip2	Upf1
H1f5	Numbl	Ube2e2
Cds2	Rps4l	Phf21a
Nap1l1	Pstk	Dock1
Rex1bd	Nol7	Zfp85
Abi1	Ldha	Uqcc5
Rps27a-ps2	Ptpmt1	Abce1
Zfp516	1110038F14Rik	H2bc8
Deaf1	Grm4	Phtf1
Col25a1	Scoc	Rrp1b
Gm10052	Syt10	Zfp420
Tlcd3b	Naa15	Txndc9
Kcnc1	Zfp35	Syt17
Hdhd5	Tmem117	Nrf1
Vwa8	Luzp2	Panx2
Grin2a	Rpl27	Map2k5
Hnrnpa3	Usp31	Znrf2
Sh2d3c	Krr1	Dhcr7
Eno1b	Mapk1	Wdr91
Rpl18	Alg11	Kl
Nap1l4	Bcl9l	Fam133b
Zfp945	Tmed5	Rpl23a
Ntrk3	Stk24	Rpl5
Mfsd4a	Cyp26b1	Tmed9
Pde1a	Ddo	Gpc6
Skap2	Wscd2	Serhl
Mtfmt	Sorcs3	Tmod3
Tafa1	B9d2	Anks1b
Gm12715	Nmral1	Fbh1
Il1rapl2	Plppr1	Rpl30
Hnrnpm	Syne2	Gda
Ddn	D630045J12Rik	Zfp110
Septin9	Rps15	Eif4b
Usp47	Snu13	Samd12
Zc3h4	Zkscan7	Tubb2a
R74862	Arhgap22	Ccser2
Naa38	Snrnp70	Memo1
Cdyl	Eno1	0610030E20Rik
Rpl11	Sema3e	Egln2
Slc27a4	Nup98	Lin9
Tonsl	Aox1	Kcnt1
Hopx	Col23a1	Kcnj14
Csf2ra	Jazf1	Zfp958

Eda	Klhdc3	Thra
Slc14a1	Egfem1	Rnf169
Wdr83os	Unc5d	H1f5
Nrsn2	Sox11	Cds2
Fxn	Jakmip1	Nap1l1
Mamld1	Zbtb18	Rex1bd
Ppp2r5d	Brwd3	Abi1
Zfp933	Crb1	Zfp516
Max	Pcdhb5	Deaf1
Bcas3	Cycs	Col25a1
Hadhb	Sfxn4	Gm10052
Ptk2b	Mapk15	Tlcd3b
Mbtd1	Rnf217	Kcnc1
Zfp426	Chadl	Hdhd5
Cfap418	Utp14a	Vwa8
Kbtbd2	Chchd1	Grin2a
Arhgef12	Slc22a8	Hnrnpa3
Fcgr3	Prpf38a	Sh2d3c
Znhit1	Ap3s2	Eno1b
Uqcr10	Hspbp1	Frem1
Tcea2	Lin28b	Rpl18
Trp53	Gpatch1	Nap1l4
Ccdc28a	Alms1	Zfp945
Tor4a	Ppcdc	Ntrk3
Nsmce2	Gpx1	Mfsd4a
Calcrl	Chd4	Pde1a
Syn3	Slc24a3	Skap2
Taf1b	Uqcrh	Mtfmt
Cdh24	Ptcd3	Tafa1
Zfp637	Nlgn1	Gm12715
Akap17b	Rpl7l1	Hnrnpm
Rcan3	Crem	Ddn
Flot1	Zkscan8	Septin9
Ndufs8	Dpp3	Usp47
Kcnh7	Srrm4	Zc3h4
Fdps	Pepd	R74862
Slx1b	Brpf3	Naa38
Eif4a1	Nr6a1	Cdyl
Rgs3	Pym1	Rpl11
Atl2	Mro	Slc27a4
Nkapd1	Gpn1	Tonsl
Dbp	Trerf1	Hopx
Sclt1	Dzip3	Csf2ra
Zfp874b	Ipcef1	Eda
Zfp341	Mtx1	Slc14a1
Kmt5c	Fbln2	Wdr83os
Kcng2	Cnnm2	Nrsn2
Ntng1	Cadm2	Fxn
Tnip2	Mocs1	Mamld1
Zfp422	Prr36	Ppp2r5d
Ube4a	Med14	Zfp933
Ptp4a3	Arb2a	Max
Zfp930	Arih2	Bcas3

Tmem40	Rab3ip	Hadhb
4930453N24Rik	Gm4735	Ptk2b
Unc5c	Ano6	Mbtd1
Grb2	Ifi27	Zfp426
9430015G10Rik	H2ac18	Cfap418
Hspa2	Paqr9	Kbtbd2
Ntm	Chi3l1	Nhs
Zfp74	Plcxd1	Arhgef12
Taok2	Ethe1	Znhit1
Nptx2	Zfp428	Uqcr10
Atxn7l3	Ccdc146	Tcea2
Chpt1	Cdpf1	Trp53
Kif13b	H4c12	Ccdc28a
H2aj	Tank	Tor4a
Rpl3	Cntn4	Nsmce2
Dhps	Clasp1	Calcrl
Psma3	Hhip	Syn3
Rp2	Siva1	Taf1b
H4c1	Scn1a	Cdh24
Prmt7	Selenop	Zfp637
Gemin2	Gm26448	Akap17b
Tpt1	Gm26447	Rcan3
Gm10076	Snora73a	Flot1
Serpinb6a	Snora23	Ndufs8
Pop5	Gm25129	Kcnh7
Zdhhc8	Snora44	Fdps
Kif27	Gm25788	Slx1b
Slc35e3	Gm23442	Eif4a1
Zfp462	Rny3	Rgs3
Pcnx2	Snord15b	Atl2
Arrb2	Gm22422	Nkapd1
Pyroxd2	Snord22	Dbp
Golm2	Rnu12	Sclt1
Cend1	Snora73b	Zfp874b
Alyref2	Snora74a	Zfp341
Scrt2	Rny1	Kmt5c
Pwwp2b	Gm22154	Kcng2
Gtf2i	Tacc1	Ntng1
Nrg2	Cpped1	Tnip2
Ap2a1	Aurkaip1	Zfp422
Ppih	Dhrs3	Ube4a
2610008E11Rik	Ubr4	Ptp4a3
Zfp941	Hnrnpr	Zfp930
Zfp937	Med18	4930453N24Rik
Caln1	Phactr4	Unc5c
Hnrnpc	Gm1976	Grb2
Bckdha	Adamtsl1	9430015G10Rik
C030014I23Rik	Kndc1	Hspa2
Zfp128	Prpf4	Ntm
Chst8	Cdc26	Zfp74
Pantr1	Slc31a1	Taok2
Zfp868	Fkbp15	Nptx2
Rnf14	Slc31a2	Atxn7l3

Wtap	E230001N04Rik	Chpt1
Irak2	Cacng3	Kif13b
Samd5	Anks6	H2aj
Zfp266	Ipo7	Rpl3
0610040J01Rik	Tmem42	Dhps
Tor3a	Pomgnt2	Psma3
A930017M01Rik	Vps37b	Rp2
Dcc	Numa1	H4c1
Tmem219	Bpnt2	Prmt7
Tnfrsf19	Wdr6	Gemin2
Fam78b	Nrxn3	Tpt1
Fhit	Akap13	Gm10076
Layn	Msl2	Serpinb6a
Eno3	Plekhd1	BC002059
Nr1h2	Zfyve26	Pop5
Rpl35a	Rdh11	Zdhhc8
H4c9	Hmgn3	Slc35e3
Marf1	Ankdd1a	Zfp462
Atp8b2	Hmgb1	Pcnx2
Plaat3	Gm6969	Arrb2
H4c3	Lsm14a	Pyroxd2
Mrps9	Garre1	Golm2
Slc9a6	Flvcr1	Cend1
Cd302	Insyn1	Alyref2
Bloc1s4	Zfp932	Scrt2
Plekhh1	Tecpr1	Pwwp2b
Ipmk	Fbxl18	Gtf2i
Nsa2	Wdr35	Nrg2
H3f3a	Gm5113	Ap2a1
Tsga10	Zbtb16	Ppih
Lrrtm1	Fxyd6	2610008E11Rik
Intu	Zfp575	Zfp941
B2m	Vkorc1l1	Zfp937
Gstp1	Zbtb6	Caln1
Ube2s	Zfp810	Hnrnpc
Zbtb40	Zfp772	Bckdha
Kcnd2	Ecsit	C030014I23Rik
Arl1	Nck2	Zfp128
Acyp2	Zfp617	Chst8
Csmd1	Fbxl12	Pantr1
Tmem263	Suds3	Zfp868
Rpl26	Bub3	Sntb1
Trmt61a	Cntnap5b	Rnf14
Slc4a4	Hes6	Wtap
H4c8	Polr1c	Irak2
Galnt13	Xpo5	Samd5
Copz1	Eif1ax	Zfp266
Mkx	Frat1	0610040J01Rik
Rrs1	Usp51	Tor3a
Clasrp	Lgi1	A930017M01Rik
Rrp1	Rplp0	Dcc
Haghl	Ppp1r3c	Tmem219
Zfp143	Rps28	Tnfrsf19

Lsamp	Bmpr2	Fam78b
Myl4	Tmem128	Fhit
Mcrip1	Lyar	Eno3
Dnajc30	Trmt2b	Nr1h2
Prcp	B3galt4	Rpl35a
Ppm1b	Tspan6	H4c9
Blnk	Zfp563	Marf1
Prpf40a	Zfp763	Atp8b2
Maml3	H4c11	Plaat3
Fnip2	Hdac8	H4c3
Sfmbt2	Cbln4	Mrps9
Stk19	S1pr3	Slc9a6
H2-K1	Krt77	Cd302
Exoc5	Syngap1	Bloc1s4
Mmgt1	Prkag1	Plekhh1
Exosc5	Uqcc4	Ipmk
Taok3	Gm10222	Rxfp3
Agbl4	Nnat	Nsa2
Slc38a10	Blcap	H3f3a
Ddhd2	Myl9	Tsga10
Naca	Pex26	Lrrtm1
Dnai1	Romo1	Intu
Cxcl12	Arfgef1	B2m
Phf5a	Zic3	Gstp1
Zfp873	Htatsf1	Ube2s
Fiz1	Vxn	Zbtb40
Acvr2b	Sptbn2	Kcnd2
Zcchc14	Rtl8b	Arl1
Nol4l	Rtl8a	Acyp2
Hipk2	Zfp760	Csmd1
Tmem151a	Zfp948	Tmem263
Stx17	Zfp160	Rpl26
Nol10	Gtf2f2	Trmt61a
Smim20	Lrch1	Slc4a4
Mrps36	Afdn	H4c8
Rps7	Tcp1	Tdrd5
Snrpa	Tm9sf4	Galnt13
H4c4	Smim45	Copz1
Sox21	Ccdc134	Mkx
Cox5b	Ninl	Rrs1
Zic2	Zfp120	Clasrp
Cep128	Insm1	Rrp1
Sec22c	Macrod2	Haghl
Skic8	Pick1	Zfp143
Dpp6	Lgals1	Lsamp
Ksr2	Amn1	Mcrip1
Dot1l	Efcc1	Dnajc30
Pclo	Ap5s1	Prcp
Akap6	Cenpb	Ppm1b
Mdc1	Shroom4	Blnk
U2af1	Usf3	Prpf40a
Med9	Ddrgk1	Maml3
Cd2ap	Nat8f4	Fnip2

Gdpd1	Aup1	Sfmbt2
Dlgap4	Htra2	Stk19
Tmem91	Dok1	H2-K1
Ppp1r1b	D430041D05Rik	Exoc5
Gm2423	Chrac1	Mmgt1
Ext1	Cep152	Exosc5
Cyp2d22	Rpl34-ps1	Taok3
Kalrn	Mfap1a	Agbl4
Bod1l	Gng5	Slc38a10
Akr1b10	Zfp467	Ddhd2
Armt1	Myadm	Naca
Tac1	Zfyve19	Cxcl12
Mospd2	Gjd2	Phf5a
Rps17	Clxn	Zfp873
Myl1	Clec16a	Fiz1
Suclg2	Gpr88	Acvr2b
Patj	Flnc	Zcchc14
Ssbp3	Tmem167b	Nol4l
Zscan20	Trp53i11	Hipk2
Gm14292	Sars1	Tmem151a
Rbak	Celsr2	Stx17
Slc25a3	Cry2	Nol10
Myt1l	Sort1	Smim20
Ppp4r1	Ptprz1	Obscn
Rcc1l	Psma5	Mrps36
Flot2	Rap1a	Rps7
Rps12	Csde1	Snrpa
Rpl34	H2bc21	H4c4
Zfp13	H2ac20	Sox21
Gmfb	Sf3b4	Cox5b
Pgghg	Sp9	Zic2
Zfp27	Selenbp1	Cep128
Lmtk3	Cgn	Sec22c
Iah1	Ssb	Zfp229
Pgk1	Clk2	Skic8
Lmnb2	Dap3	Dpp6
Qki	Msto1	Ksr2
Btbd3	Syt11	Dot1l
Scfd2	Zfp619	Pclo
Rai1	Zbtb34	Akap6
Zfp954	Urm1	Mdc1
Cttnbp2nl	Slc7a14	U2af1
Unc13c	Cdk7	Med9
Cnih4	Pde7a	Cd2ap
Hs6st2	Zbtb10	Gdpd1
Lancl2	Rps18-ps6	Dlgap4
2700097O09Rik	Cep43	Ppp1r1b
Btbd9	Adgrv1	Gm2423
Gspt1	Nr2f1	Ext1
Erbb4	Zfp72	Cyp2d22
Tnfaip8	Gprin1	Kalrn
Rapgef2	Fam8a1	Bod1l
Gak	Dusp22	Akr1b10

Lhfpl5
Opcml
Morf4l1
Trank1
Nectin2
Rpp25
Erbb2
Gm10115
Rpl17
Itgb1bp1
Gm15772
Otof
Tmem65
Borcs7
Tubb3
Ftl1-ps1
Zfp706
Arf2
Ap3b2
Acat3
Ifit3b
Zfp398
Mppe1
Syt9
Gm12751
Gm10118
Cys1
Timm17a
Armc9
Tubb4a
Srpk2
2310039H08Rik
Mysm1
Ganc
Rpl7a
Ncs1
Atp5mc2
Cebpzos
Ppox
Smim29
Shisal1
Zfp512
Ei24
Kcnc3
Zfp599
Hikeshi
4833420G17Rik
Actg1
Zfp28
Phactr2
Impdh2
Klhl24
Hdac10
H3c1
H4c2
H2ac6
H3c6
H4c6
H2ac12
H4c18
H4c17
Epc2
Tmem19
Scyl2
Dazap1
Ank3
Strada
Marcks
Nkain2
Pcgf1
Arid1b
Psmb3
Tmem100
Msi2
Cacng7
Tlcd3a
Ccdc92b
Ahnak
Sat2
Sco1
Atxn1l
Insyn2b
Hba-a2
Hba-a1
B3gnt9
Fcho1
Ell
Ssbp4
Fam149a
Fat1
Mfhas1
Ndufaf3
Gmppb
Slc35g2
Scn2b
Ccdc153
4930581F22Rik
Eif3g
Rnf213
Ccnd1
Capza1
Gm20634
Tmem256
Zscan25
Rnf121
Serpinh1
Armt1
Mospd2
Rps17
Suclg2
Patj
Ccdc62
Ssbp3
Zscan20
Rbak
Slc25a3
Myt1l
Ppp4r1
Rcc1l
Flot2
Rps12
Rpl34
Zfp13
Gmfb
Pgghg
Zfp27
Lmtk3
Iah1
Pgk1
Lmnb2
Qki
Btbd3
Zfp119b
Scfd2
Rai1
Zfp954
Cttnbp2nl
Unc13c
Cnih4
Hs6st2
Lancl2
2700097O09Rik
Btbd9
Gspt1
Erbb4
Tnfaip8
Rapgef2
Gak
Lhfpl5
Opcml
Morf4l1
Trank1
Nectin2
Rpp25
Rpl17
Itgb1bp1
Gm15772
Otof
Tmem65

Acadm	9230112E08Rik	Borcs7
Cfl2	Tlnrd1	Tubb3
Zfp938	Fam217b	Zfp706
Gm10123	Chchd2	Arf2
Mtap	Tmem132b	Ap3b2
Zfp696	Rgma	Fbxl12os
Atp11c	Nsmce3	Acat3
Kdr	Ccdc190	Ifit3b
Ccdc177	Top1	Zfp398
Ufc1	Rasal2	Mppe1
Mrpl42	Slc17a7	Syt9
Nrg1	Mn1	Cys1
Ica1	Fv1	Timm17a
Rpl35	Lrrc8b	Armc9
Ccni	Sox13	Tubb4a
2010204K13Rik	Etnk2	Srpk2
Manbal	Htr1d	2310039H08Rik
H2ac15	Cntnap5a	Mysm1
Zfp780b	Utp3	Ganc
Ing2	Sars2	Rpl7a
Lrrc40	Eid2b	Ncs1
Ctnna2	Zfp974	Atp5mc2
Mapk3	Hmgn2-ps	Cebpzos
Kif1b	Rmdn3	Ppox
Zeb1os1	Fryl	Smim29
Zfp26	Dgkd	Shisal1
Kcnma1	Pnma8b	Zfp512
Bbs5	Cited4	Ei24
Clip2	Bicra	Kcnc3
Numbl	Zswim9	Zfp599
Rps4l	Zscan18	Hikeshi
Ift25	Nicol1	4833420G17Rik
Pstk	Zfp804a	Actg1
Nol7	Ccnyl1	Zfp28
Ldha	Gad1	Phactr2
Ptpmt1	Klhl9	Impdh2
1110038F14Rik	Rraga	Klhl24
Grm4	Tgfbrap1	Hdac10
Scoc	Rabepk	Acadm
Syt10	Ndufb6	Cfl2
Naa15	Camkmt	Zfp938
Hacd1	Rasgrp3	Gm10123
Zfp35	Safb	Mtap
Tmem117	Zfp827	Zfp696
Luzp2	Ptges3	Atp11c
Rpl27	Lrrc73	Kdr
Usp31	Yipf3	Ccdc177
Uqcc6	Jund	Ufc1
Krr1	Nr2c2ap	Mrpl42
Mapk1	Srsf3	Nrg1
Alg11	Arhgef10	Ica1
Bcl9l	Smim15	Rpl35
Tmed5	Cecr2	Ccni

Stk24	Syndig1l	2010204K13Rik
Cyp26b1	2210408I21Rik	Manbal
Ddo	Slc25a16	H2ac15
Wscd2	Zfp213	Zfp780b
Sorcs3	1700086L19Rik	Ing2
B9d2	Zfp942	Lrrc40
Nmral1	Zfp65	Ctnna2
Plppr1	Zfp1006	Mapk3
Syne2	Armc2	Kif1b
D630045J12Rik	Tia1	Zeb1os1
Rps15	Egr4	Zfp26
Snu13	Setdb2	Kcnma1
Zkscan7	Tbpl1	Bbs5
Arhgap22	Map3k5	Clip2
Snrnp70	Hpcal1	Numbl
Eno1	2410004P03Rik	Rps4l
Sema3e	Rpl23	Ift25
Zfp773	Grid2	Pstk
Nup98	Dtnb	Nol7
Aox1	1110002L01Rik	Ldha
Col23a1	Zfp777	Ptpmt1
Jazf1	H2ac13	1110038F14Rik
Klhdc3	Atp5mk	Grm4
Egfem1	Gprin2	Scoc
Unc5d	Pcnp	Syt10
Sox11	Nt5dc2	Parp10
Jakmip1	Cfap44	Naa15
Zbtb18	Entrep1	Hacd1
Brwd3	2510002D24Rik	Zfp35
Pcdhb5	Rimbp3	Tmem117
H2ac21	Cebpd	Luzp2
Cycs	Eef1g	Rpl27
Sfxn4	Tut1	Usp31
Sp3os	Mta2	Uqcc6
Rnf217	Eml3	Krr1
Chadl	Rom1	Mapk1
Utp14a	B3gat3	Alg11
Chchd1	Ganab	3300002I08Rik
Slc22a8	Ints5	Bcl9l
Prpf38a	1810009A15Rik	Tmed5
Ap3s2	Uqcc3	Stk24
Hspbp1	Ubxn1	Cyp26b1
Lin28b	Bscl2	Ddo
Gpatch1	Gng3	Wscd2
Alms1	Hnrnpul2	Sorcs3
Ppcdc	Ttc9c	B9d2
Gpx1	Zbtb3	Nmral1
Chd4	Polr2g	Plppr1
Slc24a3	Snx29	Syne2
Uqcrh	Sms	D630045J12Rik
Ptcd3	Mpst	Rps15
Nlgn1	Nalf2	Snu13
Rpl7l1	Gspt2	Zkscan7

Crem	Cdr1os	Arhgap22
Zkscan8	Zhx2	Snrnp70
Gm7964	2610005L07Rik	Eno1
Dpp3	6820431F20Rik	Sema3e
Srrm4	Apcdd1	Zfp773
Pepd	Ccdc112	Nup98
Brpf3	Mcc	Aox1
Amd2	Lrrtm2	Col23a1
Nr6a1	Ppia	Jazf1
Pym1	Fndc1	Klhdc3
Mro	Septin5	Egfem1
Gpn1	Tuba1a	Unc5d
Trerf1	Taf1a	Sox11
Dzip3	Klf12	Jakmip1
Ipcef1	Nanos1	Zbtb18
Mtx1	Ppp1r3e	Krt73
Fbln2	Phf20l1	Brwd3
Vrk2	Lratd2	Pcdhb5
Cnnm2	Ptrh2	Cycs
Cadm2	Zfp9	Sfxn4
Mocs1	Lyrm9	Sp3os
Prr36	Adam1a	Rnf217
Med14	Zfp783	Chadl
Arb2a	Spef2	Utp14a
Arih2	Gm57857	Chchd1
Rab3ip	Smim10l1	Slc22a8
Gm4735	Gm10419	Prpf38a
Spata6l	Grcc10	Ap3s2
Ano6	Cep170b	Hspbp1
Ifi27	Rybp	Gpatch1
H2ac18	Gpr27	Alms1
Paqr9	Nfxl1	Ppcdc
Chi3l1	4933439C10Rik	Gpx1
Plcxd1	Gm12258	Chd4
Ethe1	Sod3	Slc24a3
Zfp428	Ptgr2	Uqcrh
Cdpf1	Acot1	Ptcd3
H4c12	Bhlhb9	Nlgn1
Tank	Gprasp2	Rpl7l1
Cntn4	Armcx5	Crem
Clasp1	Uprt	Zkscan8
Hhip	Zxda	Gm7964
Siva1	Zxdb	Dpp3
Scn1a	Srp54a	Srrm4
Selenop	Lrrc61	Pepd
Gm26448	Drc1	Brpf3
Snora73a	Vma21	Amd2
Gm25813	Tmem185a	Nr6a1
Rny3	5031425E22Rik	Klk8
Rnu12	Stmp1	Pym1
Snora73b	Zfp449	Mro
Snora74a	Nudt10	Gpn1
Rny1	Nudt11	Trerf1

Tacc1	H2-D1	Dzip3
Cpped1	C4b	Ipcef1
Aurkaip1	Hsd17b8	Mtx1
Dhrs3	Zfp414	Fbln2
Ubr4	Cyp4f15	Cnnm2
Hnrnpr	Gm4924	Cadm2
Med18	Arhgdig	Mocs1
Phactr4	Wdr90	Prr36
Gm1976	Nme3	Med14
Adamtsl1	Eme2	Arb2a
Kndc1	D330041H03Rik	Arih2
Prpf4	Sft2d1	Tnnt1
Cdc26	Rsph3a	Rab3ip
Slc31a1	Mtarc2	Gm4735
Fkbp15	Dok6	Krt2
Slc31a2	Cep76	Spata6l
Cacng3	Ppp1r12b	Ano6
Anks6	Csnk1g3	Ifi27
Ipo7	Prr16	H2ac18
Tmem42	Pcdhb22	Paqr9
Pomgnt2	Prob1	Chi3l1
Vps37b	D2hgdh	Plcxd1
Numa1	Cops9	Ethe1
Bpnt2	C130036L24Rik	Zfp428
Wdr6	Rab18	Cdpf1
Nrxn3	Wdfy1	H4c12
Akap13	Nbeal1	Tank
Msl2	Hspe1	Cntn4
Plekhd1	Pgap1	Clasp1
Zfyve26	Tmem88b	Hhip
Rdh11	Faap20	Siva1
Mthfs	Klhl21	Scn1a
Hmgn3	Rpl31	Selenop
Hmgb1	Lmbrd1	Gm26448
Gm6969	5730409E04Rik	Gm26447
Lsm14a	Btbd19	Snora73a
Garre1	Kti12	Snora23
Flvcr1	Lrp8os2	Gm23442
Insyn1	Alg6	Rny3
Zfp932	Insyn2a	Snord15b
Tecpr1	Tufm	Snord22
Fbxl18	Iqck	Rnu12
Wdr35	AA474408	Snora73b
Gm5113	Il11ra3	Snora74a
Zbtb16	Ccl27al	Rny1
Fxyd6	Ccl27a	Gm22154
Zfp575	Il11ra1	Tacc1
Vkorc1l1	Mob3b	Cpped1
Psg16	Hbb-bt	Aurkaip1
Zbtb6	Trim68	Dhrs3
Rnasel	Rhog	Ubr4
Zfp810	Ggh	Hnrnpr
Zfp772	4632427E13Rik	Med18

Ecsit	Exoc8	Phactr4
Nck2	Mlycd	Gm1976
Zfp617	Snrnp40	Adamtsl1
Fbxl12	Svip	Kndc1
Suds3	Rbm15b	Prpf4
Bub3	Rpl13a	Cdc26
Cntnap5b	Zfp788	Slc31a1
Hes6	Plekhf1	Fkbp15
Polr1c	Znhit6	Slc31a2
Xpo5	Sdhaf1	E230001N04Rik
Eif1ax	Dnajb14	Cacng3
Frat1	Zfp382	Anks6
Lgi1	Zfp568	Ipo7
Rplp0	Spint2	Tmem42
Ppp1r3c	Ap1ar	Pomgnt2
Rps28	Dda1	Vps37b
Bmpr2	Gramd2a	Numa1
Tmem128	Amy1	Bpnt2
Lyar	Zfp94	Wdr6
Trmt2b	Zfp109	Nrxn3
B3galt4	Peak1	Akap13
Tspan6	Tnfaip8l3	Msl2
Zfp563	Ccdc61	Plekhd1
Zfp763	Ehd2	Zfyve26
H4c11	AI429214	Rdh11
Hdac8	H3c13	Mthfs
Cbln4	Zfp865	Hmgn3
S1pr3	Zfp628	Ankdd1a
Krt77	Mir100hg	Hmgb1
Pcna-ps2	S100a16	Gm6969
Syngap1	Gm10699	Lsm14a
Prkag1	Mex3a	Garre1
Uqcc4	Fat3	Flvcr1
Nnat	Arfip1	Insyn1
Blcap	Gm10710	Zfp932
Myl9	Zfp971	Tecpr1
Pex26	Gm14296	Fbxl18
Romo1	Kcng1	Wdr35
Arfgef1	Mocs3	Gm5113
Zic3	Zfas1	Zbtb16
Ccdc87	Lekr1	Fxyd6
Htatsf1	Arfgef2	Zfp575
Vxn	Ankub1	Vkorc1l1
Sptbn2	Tox2	Psg16
Rtl8b	Mafb	Zbtb6
Rtl8a	Tmem267	Rnasel
Zfp760	Sox2	Zfp810
Zfp948	Cpne1	Zfp772
Zfp160	Mmp24os1	Ecsit
Gtf2f2	Myh7b	Hmcn1
Lrch1	Eif2s2	Nck2
Afdn	Kif5a	Zfp617
Mas1	A830092H15Rik	Fbxl12

Tcp1	Tspyl3	Vsig10
Tm9sf4	Zcchc3	Suds3
Smim45	Csnk2a1	Bub3
Ccdc134	Zfp950	Cntnap5b
Ninl	Syndig1	Hes6
Zfp442	Fndc10	Asb18
Zfp120	Thbd	Polr1c
Insm1	Pdzd8	Xpo5
Ndufaf2	Atxn7l3b	Eif1ax
Col8a1	Kiz	Frat1
Macrod2	Smim26	Lgi1
Pick1	Ankef1	Rplp0
Lgals1	Ube2n	Ppp1r3c
Amn1	Plxnc1	Rps28
Efcc1	Hspa12b	Bmpr2
Ap5s1	Arrdc3	Tmem128
Cenpb	Slc4a11	Lyar
Shroom4	Itpa	Trmt2b
Usf3	Gm10762	B3galt4
Ddrgk1	Hps6	Tspan6
Aup1	Pdzd7	Zfp563
Htra2	Ctxn2	Zfp763
D430041D05Rik	Serf2	H4c11
Chrac1	Grk6	Hdac8
Cep152	Lcmt2	Cbln4
Rpl34-ps1	B3galt5	S1pr3
Gm10240	Ranbp6	Krt77
Mfap1a	Inafm2	Syngap1
Gng5	Pabir1	Prkag1
Zfp467	Pak6	Uqcc4
Myadm	Ptar1	Gm10222
Zfyve19	Chrm5	Nnat
Gjd2	Ano3	Blcap
Clxn	Qser1	Myl9
Clec16a	Nrbf2	Pex26
Gpr88	Fjx1	Romo1
Tmem167b	Prdm11	Arfgef1
Trp53i11	H2bc3	Zic3
Sars1	Nxpe3	Ccdc87
Celsr2	Zfp408	Htatsf1
Cry2	Yae1d1	Vxn
Psrc1	Lrrc55	Sptbn2
Sort1	Ccdc162	Rtl8b
Ptprz1	Znhit2	Rtl8a
Psma5	Amd1	Zfp760
Il3ra	Heg1	Zfp948
Rap1a	Pde11a	Zfp160
Csde1	Ift70a1	Gtf2f2
H2bc21	Ift70b	Lrch1
H2ac20	Wipf1	Afdn
Sf3b4	Carns1	Mas1
Sp9	Aldh3b2	Tcp1
Selenbp1	Scn9a	Tm9sf4

Cgn	Scn2a	Smim45
Ssb	Fign	Ccdc134
Clk2	Zbtb2	Ninl
Dap3	Rprm	Zfp442
Msto1	Rc3h2	Zfp120
Syt11	Reno1	Insm1
Zfp619	Fnbp1	Ndufaf2
Zfp114	Dolk	Macrod2
Zbtb34	Dnlz	Pick1
Gm5537	Alg10b	Lgals1
Gm5641	Slitrk1	Amn1
Urm1	Commd6	Efcc1
Slc7a14	Gm13375	Ap5s1
Gm10259	Aarsd1	Cenpb
Cdk7	Rsph10b	Shroom4
Pde7a	6330403L08Rik	Usf3
Zbtb10	Nrbp2	Ddrgk1
Rps18-ps6	Nynrin	Nat8f4
Cep43	Zfp652	Aup1
Adgrv1	Zc3h3	Htra2
Nr2f1	Ly6a	Dok1
Zfp72	Selenot	D430041D05Rik
Zfp874a	Selenos	Chrac1
Zfp825	Selenom	Cep152
Gprin1	Selenoi	Rpl34-ps1
Fam8a1	Txnrd2	Gmnc
Dusp22	Msrb1	Mfap1a
H3c1	Gpx4	Aard
H4c2	Sox4	Gng5
H3c6	Ywhaq	Zfp467
H4c6	Acsf2	Myadm
H2ac12	Selenoh	Zfyve19
H4c18	Mog	Gjd2
H4c17	Ass1	Clxn
Epc2	Gm24119	Clec16a
Lyz2	Gm26079	Flnc
Tmem19	Rab11b	Tmem167b
Scyl2	Snord17	Trp53i11
Dazap1	Gm16485	Sars1
Ank3	Ankrd63	Celsr2
Strada	Erich3	Cry2
Marcks	Chml	Psrc1
Nkain2	Tmem203	Sort1
Pcgf1	Nrarp	Ptprz1
Zfp975	Klhdc7a	Psma5
Arid1b	Fam43b	Rap1a
Psmb3	AI593442	Csde1
Tmem100	F8a	H2bc21
Msi2	Sf3b5	H2ac20
Cacng7	AW011738	Sf3b4
Tlcd3a	Smim1	Sp9
Ccdc92b	Sarnp	Selenbp1
Ahnak	Ctdsp2	Ssb

Sat2	AU041133	Clk2
Sco1	Dohh	Dap3
Atxn1l	Plekhn1	Msto1
Hba-a2	Cfap74	Syt11
Hba-a1	Ddi2	Zfp619
B3gnt9	Emc1	Zfp114
Fcho1	Nkain1	Zbtb34
Ell	Bnip3	Gm5537
Ssbp4	1110065P20Rik	Gm5641
Fam149a	Ndufaf8	Urm1
Fat1	Ube2d3	Slc7a14
Mfhas1	Zfp764l1	Gm10259
3000002C10Rik	AU022252	Cdk7
Ndufaf3	Hs3st4	Pde7a
Gmppb	Smarcd2	Zbtb10
Slc35g2	Ccdc47	Rps18-ps6
Scn2b	Gm7336	Cep43
Mpzl3	Gm11627	Adgrv1
4930581F22Rik	Psme3	Nr2f1
Eif3g	Vps25	Zfp72
Rnf213	Fam174b	Zfp874a
Gm10288	Chd2	Zfp825
Ccnd1	Casc3	Gprin1
Prss36	Tm2d3	Fam8a1
Capza1	5830417I10Rik	Dusp22
Gm20634	Cisd3	H4c2
Tmem256	D030028A08Rik	H3c6
Zscan25	Tomm5	H4c6
Rnf121	Tmem8b	H4c18
Il18bp	Gm20878	Epc2
Serpinh1	U2af1l4	Lyz2
9230112E08Rik	Zfp566	Tmem19
Tlnrd1	Evi2a	Scyl2
Adamtsl3	Zfp59	Dazap1
Fam217b	Rbis	Ank3
Chchd2	Actmap	Strada
Tmem132b	Dph1	Marcks
Rgma	Dact3	Nkain2
Nsmce3	Eif5a	Pcgf1
Ccdc190	Leng1	Zfp975
Top1	Prkcg	Arid1b
Ntn5	H2ac25	Psmb3
Rasal2	Zfp931	Tmem100
Slc17a7	Gm14326	Msi2
Mn1	Gm14410	Cacng7
Fv1	Gm14305	Tlcd3a
Zfp992	Zfp973	Ccdc92b
Lrrc8b	Gm4724	Ahnak
Sox13	Gm4631	Sat2
Etnk2	Gm14391	Sco1
Cntnap5a	Mon1b	Atxn1l
Utp3	Dpm1	Hba-a2
Sars2	Ube2v1	Hba-a1

Eid2b	Pdf	B3gnt9
Zfp974	Ak6	Fcho1
Hmgn2-ps	Dnaaf10	Ell
Rmdn3	Sec61g	Ssbp4
Fryl	Samd1	Fam149a
Dgkd	Ly6c1	Fat1
Pnma8b	Slc45a4	Mfhas1
Cited4	Alkbh1	3000002C10Rik
Bicra	Prnp	Ndufaf3
Zswim9	Zfp781a	Gmppb
Zscan18	Fastkd5	Slc35g2
Nicol1	Slc8a3	Scn2b
Zfp804a	Kcnip3	Mpzl3
Trabd2b	Cyp4v3	4930581F22Rik
Ccnyl1	Jrkl	Eif3g
Gad1	Ccdc82	Rnf213
Klhl9	Prps1l3	Ccnd1
Rraga	Srp54c	Plpp4
Tgfbrap1	Pms2	Prss36
Rabepk	Kdelr2	Capza1
Dnajc25	A130010J15Rik	Gm20634
Ccl19	Nalf1	Tmem256
Ndufb6	Rab10os	Zscan25
Camkmt	Mphosph8	Rnf121
Rasgrp3	Psme2	Il18bp
Safb	Zfp664	Serpinh1
Zfp827	Ccdc13	9230112E08Rik
Ptges3	Tmppe	Tlnrd1
Lrrc73	2310009B15Rik	Adamtsl3
Yipf3	Rab9	Fam217b
Jund	Trappc2	Chchd2
Nr2c2ap	Naa80	Tmem132b
Srsf3	Arpc4	Rgma
Arhgef10	Mthfsl	Nsmce3
Smim15	Rpl36a	Ccdc190
3830408C21Rik	St6galnac4	Top1
Cecr2	Gm21981	Ntn5
Syndig1l	Pigb	Rasal2
2210408I21Rik	Utp14b	Slc17a7
Slc25a16	Rab7	Mn1
Zfp213	Znrd2	Fv1
1700086L19Rik	Pin4	Zfp992
Zfp946	Nhsl2	Lrrc8b
Zfp942	Phyhd1	Sox13
Zfp65	Med12	Etnk2
Zfp58	6530402F18Rik	Cntnap5a
Zfp1006	Apoo	Utp3
Armc2	Zfx	Sars2
Tia1	Tmsb10	Eid2b
Egr4	Marchf2	Zfp974
Setdb2	Maea	Hmgn2-ps
Tbpl1	C1qtnf5	Rmdn3
Map3k5	Clec2l	Fryl

Hpcal1	Zbtb9	Dgkd
Rpl23	Ankrd39	Pnma8b
Grid2	Seh1l	Bicra
Psmg4	Rpl39	Zswim9
Dtnb	Prrt4	Zscan18
1110002L01Rik	Rab26	Nicol1
Zfp777	Eloc	Zfp804a
H2ac13	Fdx2	Ccnyl1
Atp5mk	Dynlt2a2	Gad1
Pcnp	Gm11175	Klhl9
Cfap44	Atp6v0c-ps2	Rraga
Entrep1	Brms1	Tgfbrap1
2510002D24Rik	Spaca6	Rabepk
Rimbp3	Gm25776	Dnajc25
Eef1g	Snord94	Ccl19
Tut1	B230307C23Rik	Ndufb6
Mta2	Ap2m1-ps	Camkmt
Eml3	Slc48a1	Rasgrp3
Rom1	Gm5898	Safb
B3gat3	Nap1l2	Zfp827
Ganab	Pisd-ps1	Ptges3
Ints5	Gm13456	Lrrc73
1810009A15Rik	Ctsf	Yipf3
Uqcc3	AA414768	Jund
Ubxn1	Vcp-rs	Nr2c2ap
Bscl2	Ndufb4c	Srsf3
Gng3	Gm15725	Arhgef10
Hnrnpul2	Gm12892	Smim15
Ttc9c	Gm13394	3830408C21Rik
Zbtb3	Gm14584	Cecr2
Polr2g	Ube2d-ps	Syndig1l
Snx29	Rpl3-ps1	2210408I21Rik
Sms	Ubl5	Slc25a16
Mpst	Pabpc1l2b	Zfp213
Nalf2	Tmem240	1700086L19Rik
Gspt2	Tomm6os	Zfp946
Cdr1os	Ccdc85c	Zfp942
Zhx2	Bbip1	Zfp65
2610005L07Rik	Crocc2	Zfp58
6820431F20Rik	Slc2a4rg-ps	Zfp1006
Apcdd1	Prdm16os	Bves
Ccdc112	4930570G19Rik	Armc2
Mcc	B930095G15Rik	Tia1
Lrrtm2	Mir22hg	Egr4
Ppia	Brip1os	Setdb2
Ccnf	Gm15663	Tbpl1
Septin5	Gm12940	Map3k5
Tuba1a	Snhg17	Hpcal1
Taf1a	Firre	Rpl23
Klf12	C530005A16Rik	Grid2
Nanos1	Oip5os1	Psmg4
Ppp1r3e	Trmt61b	Dtnb
Phf20l1	A730017L22Rik	1110002L01Rik

Lratd2	Rtl9	Zfp777
Ptrh2	Gm11613	Atp5mk
Zfp9	5430405H02Rik	Gprin2
Lyrm9	Lin52	Pcnp
Adam1a	Zfp703	Nt5dc2
Zfp783	Gm12905	Cfap44
Gm57857	Rtl1	Entrep1
Smim10l1	Wipf3	2510002D24Rik
Gm10419	Gm2415	Rimbp3
Grcc10	Pierce2	Cebpd
9330160F10Rik	Snhg12	Eef1g
Cep170b	Nbdy	Tut1
G530011O06Rikx	Ftx	Mta2
A530017D24Rik	9330162012Rik	Eml3
Rybp	Gm14204	Rom1
Gpr27	B130055M24Rik	B3gat3
Nfxl1	Gm15500	Ganab
4933439C10Rik	Susd5	Ints5
Gm12258	Isoc2a	1810009A15Rik
Sod3	2410006H16Rik	Uqcc3
Ptgr2	Snhg20	Ubxn1
Acot1	4933431E20Rik	Bscl2
Bhlhb9	Gm13889	Gng3
Gprasp2	A230103J11Rik	Hnrnpul2
Armcx5	2900079G21Rik	Ttc9c
Uprt	Lbhd2	Zbtb3
Zxda	Gm16638	Polr2g
Zxdb	Plcxd2	Snx29
Srp54a	A830082K12Rik	Sms
Lrrc61	5530601H04Rik	Mpst
Drc1	E130307A14Rik	Csf2rb2
Vma21	2610035D17Rik	Nalf2
Tmem185a	Lamtor5	Gspt2
5031425E22Rik	Tmem170b	Cdr1os
Stmp1	2210408F21Rik	Zhx2
Zfp449	Frg2f1	2610005L07Rik
Ccdc160	4933407K13Rik	6820431F20Rik
2500004C02Rik	Kantr	Apcdd1
Nudt10	Cers1	Ccdc112
Nudt11	2810001G20Rik	Mcc
H2-D1	C030037D09Rik	Lrrtm2
Lst1	Epb41l4aos	Ppia
Gm10501	Zfp111	Fndc1
C4b	5330434G04Rik	Ccnf
Hsd17b8	1500009L16Rik	Septin5
Zfp414	Tmem250	Tuba1a
Cyp4f15	Pet100	Taf1a
Gm4924	Gm22442	Klf12
Arhgdig	Gm25394	Psmb11
Wdr90	Gm25395	Nanos1
Nme3	Gm25492	Ppp1r3e
Eme2	Rprl3	Phf20l1
D330041H03Rik	Rmrp	Lratd2

Sft2d1	Scarna2	Ptrh2
Rsph3a	Gm23547	Zfp9
D730003I15Rik	Gm23262	Lyrm9
Mtarc2	Snora78	Adam1a
Dok6	Scarna6	Zfp783
Gm7276	Gm22009	Spef2
Cep76	Gm25835	Gm57857
Ppp1r12b	Scarna10	Smim10l1
Csnk1g3	Mia	Gm10419
Prr16	Bcl2l2	Grcc10
Pcdhb22	Galnt2	9330160F10Rik
Prob1	Cbx6	Cep170b
D2hgdh	Gm20431	A530017D24Rik
Cops9	Ier5l	Rybp
C130036L24Rik	Slc5a3	Gpr27
Rab18	Rasgef1b	Nfxl1
Wdfy1	Gm16536	4933439C10Rik
Gm10557	Rbm12	Gm12258
Nbeal1	Shkbp1	Sod3
Hspe1	Timm10b	Ptgr2
Pgap1	Zfp882	Acot1
Tmem88b	Umad1	Bhlhb9
Faap20	Rps6kc1	Gprasp2
Klhl21	Mfsd14a	Armcx5
Rpl31	Uckl1	Uprt
Cenps	Gm43517	Zxda
Lmbrd1	Pakap	Zxdb
5730409E04Rik	Cfap92	Srp54a
Btbd19	Ier3ip1	Lrrc61
Kti12	Gm15446	Drc1
Gm10575	4732440D04Rik	Vma21
Alg6	Galnt4	Tmem185a
Insyn2a	Pakap	5031425E22Rik
Tufm	Nwd2	Stmp1
Iqck	Dlx6os1	Zfp449
AA474408	E430024P14Rik	2500004C02Rik
Il11ra3	Cdk5r2	Nudt10
Ccl27al	Lime1	Nudt11
Ccl27a	Rnf8	C030034I22Rik
Il11ra1	AI480526	H2-D1
Mob3b	Ttbk2	Gm10501
Hbb-bt	Cmc4	C4b
Trim68	Shprh	Hsd17b8
Rhog	Usp49	Zfp414
Ggh	Kcne5	Cyp4f15
Klhl40	Pou3f1	Gm4924
4632427E13Rik	Uba52	Arhgdig
Exoc8	Acad11	Wdr90
Mlycd	Peds1	Nme3
Snrnp40	Pcp4	Eme2
Svip	Bloc1s1	D330041H03Rik
Rbm15b	Churc1	Sft2d1
Rpl13a	Mettl23	Rsph3a

Zfp788	Tarbp1	D730003I15Rik
AW146154	Lrrc10b	Mtarc2
Znhit6	9130221H12Rik	Dok6
Zfp791	Mir99ahg	Cep76
Sdhaf1	Snrpe	Ppp1r12b
Dnajb14	Tex9	Csnk1g3
Zfp382	Gm5067	Prr16
Zfp568	Apold1	Pcdhb22
Spint2	Rps27	Prob1
Ap1ar	Klhl33	D2hgdh
Dda1	Myl6	Cops9
Gramd2a	Rps13	C130036L24Rik
Amy1	Hspa1b	Rab18
Zfp94	Synj2bp	Wdfy1
Zfp109	Ccdc71l	Nbeal1
Peak1	Tcerg1l	Hspe1
Tnfaip8l3	Nme1nme2	Pgap1
Ccdc61	Smim13	Tmem88b
Ehd2	Eid1	Faap20
AI429214	Col6a5	Klhl21
H3c13	H4c14	Rpl31
Zfp865	2610021A01Rik	Lmbrd1
Zfp628	Cerox1	5730409E04Rik
Mir100hg	Catspere2	Btbd19
S100a16	Lamtor3	Kti12
Gm10699	Tma7	Alg6
Mex3a	Gm17167	Insyn2a
Fat3	Cyp4f17	Tufm
Arfip1	Sec14l5	Iqck
Zfp971	Siah3	AA474408
Gm14296	Gpr62	Il11ra3
Kcng1	Zfp964	Ccl27al
Mocs3	Sco2	Ccl27a
Zfas1	Cox16	Il11ra1
Lekr1	Inafm1	Mob3b
Arfgef2	A830073O21Rik	Hbb-bt
Tox2	Ube2d2a	Trim68
Mafb	Rps2-ps10	Rhog
Tmem267	Hspa1a	Ggh
3110070M22Rik	E130317F20Rik	Klhl40
Sox2	Peg10	4632427E13Rik
Cpne1	Bend4	Exoc8
Mmp24os1	Dynlt1a	Mlycd
Myh7b	Kcnb2	Snrnp40
Eif2s2	Fancf	Svip
Kif5a	1110038B12Rik	Rbm15b
A830092H15Rik	Neat1	Rpl13a
Tspyl3	Malat1	Zfp976
Zcchc3	Zfp141	Zfp788
Csnk2a1	Gpank1	AW146154
Ccl28	Med20	Gm10638
Zfp950	Scnm1	Znhit6
Syndig1	Rn7s6	Zfp791

Fndc10	Gm26461	Sdhaf1
Thbd	Rpph1	Dnajb14
Pdzd8	Lrch4	Gm10643
Atxn7l3b	Zfhx2os	Zfp382
Kiz	Six3os1	Zfp568
Smim26	Smim17	Spint2
Ism1	Gm20683	Gm10649
Ankef1	Eif4e3	Ap1ar
Ube2n	Rpl41	Dda1
4833422C13Rik	Gm20716	Gramd2a
Plxnc1	Ppp2r3d	Amy1
Hspa12b	Tmem181c-ps	Zfp94
Arrdc3	Tomm20	Zfp109
Itpa	Zfp853	Peak1
Gm10762	Hmgcs1	Tnfaip8l3
Hps6	Rnasek	Kcnd3os
Pdzd7	Silc1	Ccdc61
Itpripl1	Zbed6	Ehd2
2010315B03Rik	Zfp955a	AI429214
BC025920	Purb	H3c13
Platr25	Tmem121b	Zfp865
Ctxn2	Gm10182	Zfp628
Serf2	Rnaset2b	Mir100hg
Grk6	H2ac24	S100a16
Lcmt2	Zfp131	Gm10699
B3galt5	D430019H16Rik	Mex3a
Ranbp6	Rbm4	Fat3
Inafm2		Arfip1
Pabir1	Ccdc85b	Zfp971
Pak6	Itpripl2	Gm14296
Ptar1	Gm21781	Kcng1
Ano3	Pou3f2	Mocs3
Qser1	Zfp967	Zfas1
Nrbf2	Zfp799	Lekr1
Fjx1	Zfp870	Arfgef2
Mir670hg	Gm21984	Tox2
Prdm11	Gm14325	Mafb
H2bc3	Tmem200c	Tmem267
Nxpe3	Zfp748	3110070M22Rik
Zfp408	Fignl2	Sox2
Yae1d1	Entpd4	Cpne1
Lrrc55	Gm10243	Mmp24os1
Znhit2	Noc2l	Myh7b
Mix23	Fam177a	Eif2s2
Amd1	Rps7-ps3	Kif5a
Heg1	Dynlt1f	A830092H15Rik
Pde11a	Rnaset2a	Tspyl3
Ift70a1	Nim1k	Zcchc3
Ift70b	H4c16	Csnk2a1
Wipf1	Sox1	Zfp950
Carns1	Syne1	Syndig1
Aldh3b2	Dnah7a	Fndc10
Scn2a	Vkorc1	Thbd

Zbtb2	Kcnj11	Pdzd8
Rprm	Cmtm4	Atxn7l3b
Rc3h2	Ptrhd1	Kiz
Glo1-ps	H1f0	Smim26
Prcd	Lhx8	Ism1
Fnbp1	Dynlt1b	Ube2n
Dolk	Samd11	4833422C13Rik
Dnlz	Gm21992	Plxnc1
Alg10b	Rnps1-ps	Hspa12b
Slitrk1	Zfp994	Arrdc3
Commd6	Moap1	Itpa
1700001L05Rik	Cdkn2d	Gm10762
Gm13375	Tpbgl	Hps6
Aarsd1	Mfsd4b4	2010315B03Rik
Gm10855	Lbhd1	BC025920
Rsph10b	Erdr1y	Platr25
6330403L08Rik	Tmem181b-ps	Ctxn2
Nrbp2	Tmem151b	Serf2
Nynrin	Gm10778	Grk6
Gal3st4	Zfp955b	Lcmt2
Zfp652	Galntl6	B3galt5
Zc3h3	Zfp850	Ranbp6
Ly6a	A330023F24Rik	Chst14
Selenot	2810029C07Rik	Inafm2
Selenos	Mir9-3hg	Pabir1
Selenom	Pvt1	Pak6
Selenoi	1600020E01Rik	Ptar1
Txnrd2	Gm4419	Ano3
Msrb1	9330151L19Rik	Qser1
Gpx4	B230354K17Rik	Nrbf2
Dio3	D130020L05Rik	Fjx1
Sox4	Gm10524	Mir670hg
Ywhaq	Gm3764	Prdm11
Acsf2	Cep83os	H2bc3
Selenoh	Snhg5	Nxpe3
Mog	4932441J04Rik	Zfp408
Ass1	D130017N08Rik	Yae1d1
Trbc2	1300002E11Rik	Lrrc55
Ighm	Gm26871	Znhit2
Gm24119	Tmem147os	Mix23
Rab11b	Tnfsf12	Amd1
Snord17	Zfp87	Heg1
Gm16485	4732491K20Rik	Cerkl
Ankrd63	6720427I07Rik	Pde11a
Fem1al	Mirg	Ift70a1
Erich3	Thoc2l	Ift70b
Chml	AW047730	Wipf1
Tmem203	Gm6277	Carns1
Nrarp	Rian	Scn9a
Klhdc7a	2610037D02Rik	Scn2a
Fam43b	Mir124a-1hg	Fign
AI593442	Jpx	Zbtb2
F8a	6430590A07Rik	Rprm

Sf3b5	Dleu2	Rc3h2
AW011738	Gm10561	Reno1
Smim1	Gpr137b-ps	Glo1-ps
Sarnp	2310015A10Rik	Prcd
Ctdsp2	9530059O14Rik	Fnbp1
AU041133	Gm6225	Dolk
Dohh	Gm4673	Dnlz
Ppil6	Miat	Alg10b
Abracl	5430416N02Rik	Slitrk1
Mrpl48-ps	B230217O12Rik	Commd6
Plekhn1	Sgms1os1	1700001L05Rik
Cfap74	Ctxnd1	Gm13375
Ddi2	Gm26777	Aarsd1
Emc1	E530011L22Rik	Gm10855
Nkain1	5031434O11Rik	6330403L08Rik
Zfp995	4933404O12Rik	Nrbp2
Bnip3	A330009N23Rik	Nynrin
1110065P20Rik	Tunar	Zfp652
Ndufaf8	A130014A01Rik	Zc3h3
Ube2d3	A330074K22Rik	Ly6a
Zfp764l1	Gm27000	Selenot
AU022252	Gm26982	Selenos
Hs3st4	Gm27032	Selenom
Smarcd2	Rassf10	Selenoi
Ccdc47	Rnf113a2	Txnrd2
Marchf10	Ccdc166	Msrb1
Gm7336	Sowahc	Gpx4
Aoc2	Snhg6	Sox4
Psme3	Rpl24	Ywhaq
Vps25	Pigbos1	Acsf2
Fam174b	Gm28043	Selenoh
Chd2	Kctd12	Mog
Casc3	1500015A07Rik	Ass1
Tm2d3	Tmem185b	Ighm
5830417I10Rik	Gm28035	Gm24119
Cisd3	Atf7	Gm26079
D030028A08Rik	Xndc1	Rab11b
Tomm5	H3c4	Snord17
Tmem8b	1700052K11Rik	Gm16485
Gm20878	Zfp383	Fem1al
Haus5	2900052N01Rik	Erich3
U2af1l4	2810013P06Rik	Chml
Zfp566	Gm28539	Tmem203
Evi2a	2810402E24Rik	Nrarp
Zfp59	2410022M11Rik	Klhdc7a
Rbis	Gm28439	Fam43b
Actmap	2610306M01Rik	AI593442
Dph1	Mir124-2hg	F8a
Dact3	Gm29170	Sf3b5
Eif5a	2010320M18Rik	AW011738
Leng1	Gm15459	Smim1
Prkcg	Snhg14	Sarnp
H2ac25	Atp6-ps	Ctdsp2

Zfp931	A630072M18Rik	AU041133
Gm14326	Gm28437	Dohh
Gm14322	H3c10	Abracl
Zfp970	Gm19412	Mrpl48-ps
Gm14410	Kcnq1ot1	Plekhn1
Gm14295	Gm29609	Cfap74
Gm14305	9130401M01Rik	Ddi2
Zfp973	Chaserr	Emc1
Gm4724	H3c11	Nkain1
Gm4631	Gm28661	Zfp995
Gm14391	Zbtb11os1	Bnip3
Mon1b	Pcdha11	1110065P20Rik
Dpm1	Pcdhga10	Ndufaf8
Ube2v1	Gm37885	Ube2d3
Pdf	Snrpn	Zfp764l1
Ak6	Pcdha3	AU022252
Atp6ap1l	Gm37628	Hs3st4
Dnaaf10	Gm19938	Smarcd2
Sec61g	Iqschfp	Ccdc47
Samd1	Pcdhga12	Gm7336
Ly6c1	Pcdhga9	Aoc2
Slc45a4	Gm38198	Psme3
Alkbh1	Pcdhgc5	Vps25
Prnp	6430511E19Rik	Fam174b
Zfp781a	A930004J17Rik	Chd2
Fastkd5	Snurf	Casc3
Slc8a3	Gm37899	Tm2d3
Kcnip3	Dchs2	5830417I10Rik
Cyp4v3	Pcdhac2	Cisd3
Jrkl	Pcdhga11	D030028A08Rik
Ccdc82	Pcdhgb2	Tomm5
Prps1l3	Gm7694	Tmem8b
Srp54c	Norad	Gm20878
Pms2	Pcdhgc3	Haus5
Capn3	Zc3h11a	U2af1l4
Kdelr2	Gm56350	Zfp566
Gm4204	Pcdhgb1	Evi2a
A130010J15Rik	Gm37305	Zfp59
Nalf1	Pcdhgb6	Rbis
Rab10os	Pcdha5	Actmap
Mphosph8	4833445I07Rik	Dph1
Psme2	Pcdhga1	Dact3
Zfp664	Pcdhac1	Eif5a
Ccdc13	Pcdha12	Leng1
Tmppe	Pcdhga2	Prkcg
2310009B15Rik	Pcdhga7	H2ac25
Rab9	9330121J05Rik	Zfp931
Trappc2	Gm38372	Gm14326
Lemd1	Pcdhga5	Zfp970
Naa80	Pcdhgb4	Gm14412
Gm3411	Pcdhga4	Gm14410
Atg4a	Pcdha6	Gm14295
Arpc4	4933431K14Rik	Gm14305

Mthfsl	Pcdhgb5	Zfp973
Neu2	Pcdha9	Gm2026
Rpl36a	Pcdhga6	Gm6710
St6galnac4	Pcdha8	Gm4724
Gm21981	Pcdhga8	Gm4631
Pigb	Gm30238	Gm14391
Utp14b	Tigd5	Mon1b
Rab7	Gm37895	Dpm1
Znrd2	Pcdhgb7	Ube2v1
Pin4	Gm36952	Pdf
Nhsl2	Pcdha2	Ak6
Phyhd1	Gm9916	Dnaaf10
Med12	Gm7292	Sec61g
H2-T10	Pcdha4	Samd1
6530402F18Rik	Pcdha7	Ly6c1
Apoo	Pcdhga3	Slc45a4
Zfx	Gm37334	Alkbh1
Tmsb10	B230334C09Rik	Prnp
Haus3	Gm42692	Zfp781a
Marchf2	Snhg8	Fastkd5
Maea	Gm43668	Prox1os
Rbpj-ps3	Gm42427	Slc8a3
C1qtnf5	Sox2ot	Kcnip3
Clec2l	Gm43305	Cyp4v3
Zbtb9	H2bc18	Jrkl
Ankrd39	Tmem217rt	Ccdc82
Seh1l	Gm42829	Prps1l3
Rpl39	Gm43175	Srp54c
Prrt4	Gm43318	Pms2
Rab26	Lhfpl3	Capn3
Eloc	E330034L11Rik	Kdelr2
Tmem243	Gm42847	Gm4204
Fdx2	Gm42583	A130010J15Rik
Dynlt2a2	Peg13	Nalf1
Gm11175	Gtf3c2	Rab10os
Atp6v0c-ps2	Mrpl33	Mphosph8
Brms1	Dancr	Psme2
Spaca6	5930430L01Rik	Zfp664
Snord94	Gm45495	Ccdc13
B230307C23Rik	Gm43481	Tmppe
Ndufa12-ps	Ncbp2as2	2310009B15Rik
Ap2m1-ps	Gm42715	Rab9
Rps6-ps4	Gm42742	Trappc2
Slc48a1	Gm7993	Naa80
Rps2-ps13	Gm43597	Atg4a
Gm5898	Mpv17	Arpc4
Gm12435	Gm42732	Mthfsl
Gm715	Gm4366	Rpl36a
Nap1l2	Gm3375	St6galnac4
Pisd-ps1	Zfp862-ps	Gm21981
Gm13456	Gm44433	Pigb
Gm4875	Gm2011	Utp14b
Ctsf	9530085L11Rik	Rab7

AA414768	4933427D14Rik	Znrd2
Vcp-rs	9330102E08Rik	Pin4
Llph-ps2	Gm44435	Nhsl2
Ndufb4c	Gm44168	Phyhd1
Gm15725	Gm43872	Med12
Gm12892	C030015A19Rik	H2-T10
Gm13394	6430584L05Rik	6530402F18Rik
Gm14584	Pnma8c	Apoo
Ube2d-ps	Gm44950	Zfx
Gm12346	Gm44509	Tmsb10
Gm13378	Snhg1	Haus3
Rpl3-ps1	AV356131	Marchf2
Ubl5	Particl	Maea
Pabpc1l2b	Gm35040	Rbpj-ps3
Tmem240	Gm33989	C1qtnf5
Tomm6os	Gm49388	Clec2l
Ccdc85c	Frmpd2	Zbtb9
Gm12371	B230209E15Rik	Ankrd39
Gm14827	A230103L15Rik	Seh1l
C630043F03Rik	Gm45159	Rpl39
C230037L18Rik	Gm44798	Prrt4
Dlx1as	4930431P19Rik	Rab26
Gm5577	Gm45176	Eloc
Bbip1	A330076H08Rik	Tmem243
Smim43	Prmt1	Fdx2
Slc2a4rg-ps	Samd4b	Dynlt2a2
2810455O05Rik	Gm19410	Gm11175
Gm15631	Gm49396	Atp6v0c-ps2
Prdm16os	A230057D06Rik	Brms1
4930570G19Rik	Zfp582	Spaca6
B930095G15Rik	Nup62	Gm25776
Mir22hg	Gdf1	Snord94
1110018N20Rik	9330162G02Rik	B230307C23Rik
Brip1os	B230206I08Rik	Ndufa12-ps
Gm15663	Gm45606	H3c15
Gm12940	Hspa14	Rpl19-ps11
2310058D17Rik	Gm28756	Ap2m1-ps
Snhg17	Chmp1b	Rps6-ps4
Firre	Brd3os	Slc48a1
C530005A16Rik	Gm6145	Gm12216
Gm16001	Smim32	Gm5898
Zfp335os	Igip	Gm12312
Oip5os1	Pde2a	Gm715
1110046J04Rik	Gm45799	Nap1l2
Trmt61b	C130073E24Rik	Pisd-ps1
4930412C18Rik	Gm19935	Gm13456
A730017L22Rik	Smim36	Gm14421
Gm11613	A030001D20Rik	Ctsf
3110056K07Rik	Zbed5-ps	AA414768
Tdg-ps2	Gm10033	Vcp-rs
Gm14267	C230057M02Rik	Ndufb4c
4921531C22Rik	Gm45779	Gm15725
5430405H02Rik	C78859	Gm12892

Lin52	Gpx4-ps2	Gm13394
Zfp703	Gm9856	Gm14584
Kcnmb4os2	Nudt8	Gm15712
Gm12905	Gm47204	Ube2d-ps
Gm15912	Gm20300	Rpl3-ps1
Gm15411	Gm49338	Ubl5
Gm15706	Gm48604	1700056N10Rik
Rad51ap2	Btbd8	Pabpc1l2b
Wipf3	Gm49759	Tmem240
Gm15728	Gm47483	Tomm6os
Gm2415	Gm47163	Ccdc85c
Pierce2	Rmst	Gm14827
Pcsk2os1	A830082N09Rik	C630043F03Rik
C230034O21Rik	Gm48226	C230037L18Rik
Snhg12	Sfta3-ps	Dlx1as
D030055H07Rik	Srp54b	Gm5577
Nbdy	Gm48623	Bbip1
B230369F24Rik	Rps18-ps5	Slc2a4rg-ps
A330102I10Rik	Gm49654	2810455O05Rik
Ftx	Gm46430	Prdm16os
Gm15559	Gm40841	4930570G19Rik
Gt(ROSA)26Sor	Gm47260	B930095G15Rik
9330162012Rik	Gm32699	Mir22hg
Gm57488	2900060N12Rik	Brip1os
Gm14204	Gm49602	6330418K02Rik
B130055M24Rik	Ndufb1	Gm15663
Gm13446	Scamp4	Gm12940
Gm15500	Gm47155	2310058D17Rik
Susd5	Btf3l4b	Snhg17
Gm13205	Gm48692	Firre
Gm15743	Gm47694	C530005A16Rik
A630052C17Rik	Gm49336	Gm16001
Isoc2a	Gm49069	Oip5os1
2410006H16Rik	Gm35823	1110046J04Rik
Snhg20	Pnp	Trmt61b
A230072C01Rik	Rmrp	4930412C18Rik
Rtl10	Gm56450	A730017L22Rik
4933431E20Rik	Bc1	Gm11613
Gm15941	Vps28	3110056K07Rik
Gm13889	C030006K11Rik	Tdg-ps2
9130024F11Rik	Pdxp	4921531C22Rik
A230103J11Rik	Gm49492	5430405H02Rik
2900079G21Rik	Nnt	Gm15624
Gm11611	Lncppara	Lin52
Gm16638	Riok2	Zfp703
Plcxd2	Gm49601	Kcnmb4os2
A830082K12Rik	Magef1	Gm12905
2010001A14Rik	Gm1604a	Gm15912
5530601H04Rik	Gm49797	Syna
E130307A14Rik	Morf4l1-ps1	Gm15411
E230016M11Rik	Gm49599	Gm15706
2610035D17Rik	Ermardl2	Wipf3
Lamtor5	Gm21926	Gm15728

Gm13629
Tmem170b
2210408F21Rik
Frg2f1
4933407K13Rik
Kantr
Cers1
Gm16323
4933406I18Rik
Gm16835
2810001G20Rik
Zmiz1os1
C030037D09Rik
Epb41l4aos
Zfp111
5330434G04Rik
1500009L16Rik
Gm11762
Tmem250
Pet100
Gjd2os
Gm22442
Gm25394
Gm25395
Gm25492
Rprl3
Rmrp
Scarna2
Gm23547
Scarna6
Gm22009
Scarna10
Fcor
Bcl2l2
Galnt2
B230216N24Rik
Cbx6
Gm20431
Ier5l
Slc5a3
Gm3555
Rasgef1b
Gm16536
Rbm12
Shkbp1
Npcd
Timm10b
Zfp882
Umad1
Rps6kc1
Mfsd14a
Uckl1
Gm43517
Gm49909
C230013L11Rik
A230051N06Rik
Gm36684
Gm1043
Ptp4a1
Ntn3
Gm50010
Apbb3
BC037704
Gm50240
Gm50462
Gm50388
Gm50431
Snhg4
Gm54798
Tmem223
Gm41760
Gm46620
Gm54728
Gm50387
Kctd16
Smim10l3
Gpr52
Gm2415
Pierce2
Pcsk2os1
C230034O21Rik
Mkln1os
Snhg12
Gm15513
Nbdy
B230369F24Rik
Gm13830
Ftx
Gt(ROSA)26Sor
9330162012Rik
Gm57488
Gm14204
B130055M24Rik
Gm13446
Gm15500
Susd5
A630052C17Rik
Isoc2a
2410006H16Rik
Snhg20
A230072C01Rik
4732414G09Rik
4933431E20Rik
Gm15941
Gm13889
2900079G21Rik
Gm11611
Gm16638
Emx2os
Plcxd2
A830082K12Rik
2010001A14Rik
5530601H04Rik
E130307A14Rik
2610035D17Rik
Lamtor5
Tmem170b
2210408F21Rik
Frg2f1
4933407K13Rik
Kantr
Cers1
4933406I18Rik
Gm16835
2810001G20Rik
C030037D09Rik
Epb41l4aos
Zfp111
5330434G04Rik
1500009L16Rik

Pakap
Ier3ip1
Gm15446
4732440D04Rik
Galnt4
Pakap
Nwd2
Dlx6os1
1110002E22Rik
E430024P14Rik
Cdk5r2
Lime1
Rnf8
AI480526
Ttbk2
Cmc4
Shprh
Usp49
Pou3f1
Uba52
Acad11
Peds1
Pcp4
Car15
Bloc1s1
Churc1
Mettl23
Tarbp1
9130221H12Rik
Gm17081
Mir99ahg
Gypc
Snrpe
A930033H14Rik
Tex9
Zfp712
Zfp493
Gm5067
Apold1
Rps27
Klhl33
Myl6
Rps13
Hspa1b
Synj2bp
Ccdc71l
Gm20458
Tcerg1l
9330020H09Rik
Smim13
Gm17641
Eid1
Gcnt4
Tmem250
Pet100
Gm22442
Gm25395
Rprl3
Rmrp
Scarna2
Gm23547
Gm23262
Snora78
Scarna6
Gm22009
Gm25835
Scarna10
Fcor
Bcl2l2
Galnt2
Cbx6
Gm15688
Gm20431
Zfp966
Ier5l
Slc5a3
Rasgef1b
Gm16536
Rbm12
Shkbp1
Npcd
Timm10b
Zfp882
Umad1
Rps6kc1
Mfsd14a
Uckl1
Gm43517
Pakap
Ier3ip1
Gm15446
4732440D04Rik
Galnt4
Pakap
Nwd2
Dlx6os1
1110002E22Rik
E430024P14Rik
Cdk5r2
Lime1
Rnf8
AI480526
Gm14399
Ttbk2
Cmc4
Shprh

H4c14
Gm4202
2610021A01Rik
Cerox1
Catspere2
Gm17066
Mtag2
Lamtor3
Gm17484
Tma7
Gm17167
Akain1
Sec14l5
Siah3
Gpr62
Zfp964
Sco2
Cox16
Inafm1
A830073O21Rik
Ube2d2a
Tnnc1
Gon7
C2cd4b
Rps2-ps10
Hspa1a
Gm17354
E130317F20Rik
Peg10
Bend4
Dynlt1a
Kcnb2
Zfp804b
Fancf
1110038B12Rik
Gm19345
Zfp963
Neat1
Malat1
BC023719
A930015D03Rik
A730060N03Rik
Zfp141
Gpank1
Gm20491
Med20
BC051226
Scnm1
Rn7s6
Gm26461
Rpph1
Gm20646
Lrch4
Usp49
Kcne5
Pou3f1
Or10ad1
Uba52
Acad11
Peds1
Pcp4
Car15
Bloc1s1
Churc1
Mettl23
Tarbp1
Lrrc10b
9130221H12Rik
Gm17081
Mir99ahg
Snrpe
Tex9
Zfp712
Zfp493
Gm5067
Apold1
Rps27
Klhl33
Myl6
Rps13
Hspa1b
Synj2bp
Ccdc71l
Tcerg1l
9330020H09Rik
Gm5141
Nme1nme2
Vgll3
Smim13
Eid1
Gcnt4
H4c14
Gm4202
2610021A01Rik
Cerox1
Catspere2
Gm17066
Lamtor3
Gm17484
Tma7
Gm17167
Cyp4f17
Akain1
Sec14l5
Siah3
Gpr62

Zfhx2os
AA465934
Smim17
Gm20633
Gm20683
Eif4e3
Rpl41
Gm20716
Ppp2r3d
Tmem181c-ps
Tomm20
Zfp853
Hmgcs1
Rnasek
Silc1
Spata31f1b
Zbed6
Zfp955a
Purb
Smim18
Tmem121b
Rnaset2b
H2ac24
Zfp131
D430019H16Rik
Rbm4

Ccdc85b
Itpripl2
Gm21781
Pou3f2
Gm20939
Zfp967
Zfp799
Gm2163
Ccl19-ps5
Zfp870
Gm21984
Gm3892
Gm14325
Tmem200c
Zfp748
Entpd4
Gm10243
Erdr1x
Noc2l
Fam177a
Rps7-ps3
Dynlt1f
Rnaset2a
Nim1k
Gm21596
H4c16

Zfp964
Sco2
Cox16
Inafm1
A830073O21Rik
Ube2d2a
Gon7
C2cd4b
Rps2-ps10
Hspa1a
Gm17354
E130317F20Rik
Peg10
Bend4
Dynlt1a
Kcnb2
Fancf
1110038B12Rik
Gm19345
Zfp963
Neat1
Malat1
BC023719
A930015D03Rik
A730060N03Rik
Zfp141
Gpank1
Gm20518
Med20
BC051226
Scnm1
Rn7s6
Gm26461
Rpph1
Lrch4
Zfhx2os
AA465934
Smim17
Gm20633
Gm20683
Eif4e3
Rpl41
Gm20716
Ppp2r3d
Tmem181c-ps
Tomm20
Zfp853
Hmgcs1
Rnasek
Silc1
Zbed6
Zfp955a
Purb

Sox1
Syne1
Vkorc1
Kcnj11
Cmtm4
Ptrhd1
H1f0
1500002C15Rik
Dynlt1b
4933409K07Rik
Samd11
Gm21992
Rnps1-ps
Zfp994
Moap1
Cdkn2d
Tpbgl
Mfsd4b4
Zfp781b
Lbhd1
Gm21985
Erdr1y
Tmem181b-ps
Zfp433
Tmem151b
Gm10778
Shisa8
Zfp955b
Galntl6
Zfp850
A330023F24Rik
Gm26881
2810029C07Rik
Mir9-3hg
Pvt1
2610316D01Rik
1600020E01Rik
Gm4419
9330151L19Rik
D930019O06Rik
B230354K17Rik
D130020L05Rik
Gm10524
Gm3764
Cep83os
A830009L08Rik
Snhg5
AU022754
4932441J04Rik
Gm27029
5033417F24Rik
Gm26805
2900076A07Rik

Tmem121b
Gm10182
Rnaset2b
H2ac24
Zfp131
D430019H16Rik
Rbm4

Ccdc85b
Itpripl2
Gm21781
Pou3f2
Zfp967
Zfp799
Zfp870
Gm21984
Gm14325
Tmem200c
Zfp748
Entpd4
Gm10243
Noc2l
Rps7-ps3
Dynlt1f
Rnaset2a
Nim1k
H4c16
Sox1
Syne1
Vkorc1
Kcnj11
Cmtm4
Ptrhd1
H1f0
4933409K07Rik
Gm21992
Rnps1-ps
Zfp994
Moap1
Cdkn2d
Tpbgl
Mfsd4b4
Zfp960
Zfp781b
Lbhd1
Gm21985
Erdr1y
Tmem181b-ps
Tmem151b
Gm10778
Zfp955b
Galntl6
Zfp850

D130017N08Rik
1300002E11Rik
Gm26871
Tmem147os
Tnfsf12
Zfp87
9030407P20Rik
4732491K20Rik
6720427I07Rik
Mirg
Thoc2l
B130046B21Rik
AW047730
Gm6277
Rian
Gm2824
9930014A18Rik
B230322F03Rik
2610037D02Rik
Mir124a-1hg
Jpx
G730003C15Rik
6430590A07Rik
E230029C05Rik
4930578M01Rik
Dleu2
Gm17322
A930012L18Rik
Gm2061
4921504A21Rik
Gm10561
1700101I11Rik
Gpr137b-ps
2310015A10Rik
9530059O14Rik
AI115009
Gm6225
Gm4673
A730056A06Rik
Miat
5430416N02Rik
B230217O12Rik
Sgms1os1
Ctxnd1
Gm26777
Panct2
E530011L22Rik
9330104G04Rik
5031434O11Rik
4933404O12Rik
A330009N23Rik
A730020E08Rik
Tunar
A330023F24Rik
2810029C07Rik
Gm26793
Mir9-3hg
Pvt1
Gm17491
1600020E01Rik
Gm4419
9330151L19Rik
1700086O06Rik
D930019O06Rik
B230354K17Rik
D130020L05Rik
Gm10524
Gm3764
Cep83os
2310002F09Rik
Snhg5
Gm17690
4932441J04Rik
Gm27029
5033417F24Rik
2900076A07Rik
D130017N08Rik
1300002E11Rik
Gm26871
Gm10516
Tmem147os
Tnfsf12
Zfp87
9030407P20Rik
4732491K20Rik
6720427I07Rik
Mirg
Thoc2l
1810014B01Rik
B130046B21Rik
AW047730
Gm6277
Rian
Gm2824
9930014A18Rik
2610037D02Rik
Mir124a-1hg
Jpx
G730003C15Rik
6430590A07Rik
4930578M01Rik
Dleu2
Gm17322
Gm2061
4921504A21Rik
Gm20033

A130014A01Rik
A330074K22Rik
Gm27000
Gm10605
Gm26982
Gm27032
Bin2
Gm3693
Rassf10
Rnf113a2
Ccdc166
Sowahc
B830012L14Rik
Snhg6
Rpl24
Pigbos1
Gm28043
Kctd12
1500015A07Rik
1500004A13Rik
Tmem185b
Gm28036
Gm28035
Atf7
Xndc1
H3c4
2900093K20Rik
1700052K11Rik
Zfp383
2900052N01Rik
Gm28800
2810013P06Rik
Gm28539
2810402E24Rik
2410022M11Rik
2610306M01Rik
Gm28151
Mir124-2hg
Gm29170
A230077H06Rik
2010320M18Rik
Gm15459
Snhg14
Atp6-ps
Gm10138
Gm29100
A630072M18Rik
Gm28424
Gm28437
1700008J07Rik
Gm12663
H3c10
Csnk2a1-ps3

Gm10561
Gm26644
Gpr137b-ps
2310015A10Rik
9530059O14Rik
AI115009
Gm6225
Gm4673
Miat
5430416N02Rik
B230217O12Rik
Sgms1os1
Ctxnd1
Gm26777
Panct2
E530011L22Rik
9330104G04Rik
5031434O11Rik
4933404O12Rik
A330009N23Rik
A730020E08Rik
Tunar
A130014A01Rik
A330074K22Rik
Gm27000
Gm10605
Gm26982
Gm27032
Bin2
Rassf10
Rnf113a2
Ccdc166
Sowahc
B830012L14Rik
Snhg6
Rpl24
Pigbos1
Gm28043
Kctd12
Commd1b
1500015A07Rik
1500004A13Rik
Tmem185b
Gm28036
Gm28035
Atf7
Xndc1
H3c4
Gm29325
1700052K11Rik
Zfp383
2900052N01Rik
2810013P06Rik

Kcnq1ot1
Gm28635
Gm29609
9130401M01Rik
Gm28438
Chaserr
H3c11
Gm28661
Zbtb11os1
Pcdha11
C230085N15Rik
Gm2464
Pcdhga10
Gm37885
Gm37717
Gm38260
Snrpn
Gm38190
Pcdha3
Gm37628
Gm19938
Gm37233
Gm36936
Gm36939
Pcdhga12
Pcdhga9
Gm37166
Gm38198
Gm19445
Gm21955
Gm38085
Gm37783
Gm37226
Pcdhgc5
6430511E19Rik
Gm7265
A930004J17Rik
Snurf
Gm37899
Gm37077
Dchs2
Pcdhac2
Gm32444
Pcdhga11
Gm37602
Pcdhgb2
Gm7694
Gm37711
Gm37814
Gm38036
C130023A14Rik
Norad
Gm37811
Gm28539
Gm28320
2810402E24Rik
2410022M11Rik
Gm28439
2610306M01Rik
Mir124-2hg
Gm29170
A230077H06Rik
2010320M18Rik
Gm15459
Snhg14
Atp6-ps
Gm29100
A630072M18Rik
Gm28424
Gm28437
Gm12663
H3c10
2810405F17Rik
Csnk2a1-ps3
Kcnq1ot1
Gm28635
Gm29609
9130401M01Rik
Gm28438
Chaserr
H3c11
Gm28661
Zbtb11os1
Gm37107
Gm38056
Pcdha11
Pcdhga10
Gm37885
Gm38260
Snrpn
Gm38190
Pcdha3
Gm37628
Gm19938
Gm37233
9430053O09Rik
Gm36936
Gm36939
Iqschfp
Pcdhga12
Pcdhga9
Gm37166
Gm38198
Gm36989
Gm21955
Gm38085

Pcdhgc3
Gm37551
Zc3h11a
Gm56350
Pcdhgb1
Gm37305
Pcdhgb8
Pcdhgb6
Pcdha5
Gm37558
4833445I07Rik
Pcdhga1
AA914427
Gm37331
Gm38286
Gm37728
Pcdhac1
Pcdha12
Gm38357
Pcdhga2
Gm37238
Gm37180
Gm15853
Gm37534
Gm36995
Pcdha4b
Gm37247
Pcdhga7
Gm38372
Gm10048
8030445P17Rik
Pcdhga5
Pcdhgb4
Gm37352
Gm37706
Pcdhga4
Gm37530
Pcdha6
Gm2136
4933431K14Rik
Pcdhgb5
Gm6934
Pcdha9
Pcdhga6
Pcdha8
Gm37608
9630010A21Rik
Gm37008
Pcdhga8
Gm30238
Gm37499
Tigd5
Gm38073
Gm37783
Gm37226
Pcdhgc5
6430511E19Rik
Gm7265
A930004J17Rik
Snurf
Gm19863
Gm37899
Gm37077
Dchs2
Pcdhac2
Gm32444
Pcdhga11
Gm37602
Pcdhgb2
Gm7694
Gm37056
Gm37711
Gm37814
Gm38034
C130023A14Rik
Gm37084
Norad
Pcdhgc3
Zc3h11a
Gm17244
Gm56350
Gm8797
Pcdhgb1
Gm37305
Pcdhgb8
Pcdhgb6
Pcdha5
Gm37558
4833445I07Rik
Pcdhga1
AA914427
Gm38148
Gm38286
Gm37728
Gm37731
Pcdhac1
Gm38235
Pcdha12
Gm38357
Pcdhga2
Gm10723
Gm37238
Gm37180
Gm15853
Gm37534
Gm36995

Gm37895
Gm37211
5330406M23Rik
Gm37773
Pcdhgb7
Gm37880
Gm37296
Gm36952
Pcdha2
Gm9916
Gm7292
Gm36858
Pcdha4
Gm38300
Pcdha7
Gm38077
Pcdhga3
A430027H14Rik
Gm37334
B230334C09Rik
Gm43317
Gm42768
3110039M20Rik
Nwd2os
Gm6560
Gm42766
Snhg8
Gm43437
Gm43445
Gm43668
Gm42433
Gm42427
Sox2ot
Gm43751
Gm43316
BC030343
Gm42972
Gm43462
Gm43606
Gm43737
Gm42772
Gm43305
Gm42973
2900064K03Rik
H2bc18
Tmem217rt
Gm42829
Gm42769
Gm43175
Gm43789
Gm43679
Gm43318
Gm42770
Gm37247
Pcdhga7
9330121J05Rik
Gm38372
Gm10048
Pcdhga5
Pcdhgb4
Gm37352
Gm38366
Gm37206
Pcdhga4
Gm37529
Gm37530
Pcdha6
4933431K14Rik
2700078F05Rik
Pcdhgb5
Pcdha9
Gm37522
Pcdhga6
Pcdha8
Gm37608
Gm37607
Gm19026
Gm37606
9630010A21Rik
Gm37420
Gm37008
Pcdhga8
Gm30238
Gm37940
Gm37499
Tigd5
Gm37895
Gm37677
Gm20045
Gm38029
5330406M23Rik
Gm37773
Pcdhgb7
Gm37880
Gm37296
Gm36952
D130019J16Rik
Pcdha2
Gm9916
Gm37984
Gm7292
Gm36858
Pcdha4
Gm37174
Gm10862
Gm38043

Gm42616
Gm43672
Gm42664
Gm43571
Snord3a
C230096K16Rik
Gm43046
Lhfpl3
E330034L11Rik
Gm42446
Gm30382
Gm42771
Gm43750
Gm42608
Gm43034
Gm42847
Gm42583
Gm42656
Peg13
Gtf3c2
Gm43681
Gm20186
Gm42791
Mrpl33
Dancr
Gm43843
5930430L01Rik
Gm45495
Gm42547
Gm43481
Ncbp2as2
Gm43682
Gm42715
Gm42742
Gm7993
Gm43597
Gm43268
Mpv17
Gm42732
Gm4366
Gm3375
Zfp862-ps
Ccdc142
Gm44433
Gm38910
Gm2011
9530085L11Rik
Gm43879
4933427D14Rik
9330102E08Rik
Gm44230
Gm44235
Gm44438
Pcdha7
Gm38077
Pcdhga3
Gm37254
Gm37069
Gm37334
4921539H07Rik
B230334C09Rik
Gm43317
Gm42768
Gm42946
Gm42979
Nwd2os
Gm42692
Gm6560
Gm42766
2610011E03Rik
4833413G10Rik
Snhg8
Gm43437
Gm43445
Gm43668
C230031I18Rik
Gm42433
Gm42427
Sox2ot
Gm42843
Gm43751
Gm43316
BC030343
Gm43099
Gm42972
C130013H08Rik
E430021H15Rik
Gm43829
Gm43462
Gm43300
Gm43398
Gm43187
Gm43606
Gm43737
Gm42772
Gm43305
Gm42973
Gm42748
2900064K03Rik
Gm43341
Gm43654
H2bc18
Gm42683
Tmem217rt
Gm42829
Gm42769

Gm44027
D830050J10Rik
Gm44435
Gm43869
Gm44168
Gm43872
6330415B21Rik
Gm44210
Gm43889
C030015A19Rik
6430584L05Rik
Pnma8c
Gm44950
Gm44509
Snhg1
Gm44510
Gm39041
AV356131
Particl
Gm35040
Gm33989
Gm45153
Gm49388
Gm45177
Gm44560
Gm44678
Gm44846
B230209E15Rik
Gm45201
Gm44644
A230103L15Rik
Gm45159
Gm44798
4930431P19Rik
2900027M19Rik
Gm44676
Gm45176
A330076H08Rik
Prmt1
Samd4b
Gm44797
Gm44675
Gm19410
Gm49396
Gm45182
A230057D06Rik
Zfp582
Cpeb1os1
A930037H05Rik
Gm44800
Gm45073
Gm44677
Nup62
Gm43175
Gm43679
Gm43318
Gm42770
Gm42993
Gm42616
Gm43672
Gm43019
Gm42664
Gm43571
Snord3a
Gm43430
C230096K16Rik
Gm42842
Gm19409
Gm43046
Gm43174
Gm42992
Gm42607
Lhfpl3
E330034L11Rik
Gm42991
Gm43320
Gm42446
Gm9403
Gm42771
Gm42822
Gm43750
Gm42608
Gm43034
Gm42847
Gm42583
Gm42656
Peg13
Gtf3c2
Gm43681
Gm20186
Gm42791
Mrpl33
Gm42930
Dancr
Gm43843
5930430L01Rik
Gm45495
Gm42547
Gm43481
Ncbp2as2
Gm2762
Gm43682
Gm42715
Gm42742
Gm7993
Gm43597

Gdf1
Gm44801
9330162G02Rik
B230206I08Rik
Gm44831
3110080E11Rik
Gm35021
Gm45606
Gm45605
Hspa14
Gm28756
Chmp1b
Brd3os
Gm45629
Gm45441
Gm7328
Gm6145
Smim32
Igip
Pde2a
Gm45799
9330121K16Rik
Gm45258
Gm45871
Gm45250
Smim36
A030001D20Rik
Gm45457
Zbed5-ps
Gm10033
C230057M02Rik
Gm45779
Gm18860
Gm32856
Gm20163
Gm39822
C78859
Gm47079
Gm46218
Gm39469
Gpx4-ps2
Gm49628
Gm9856
Nudt8
Gm47204
Gm20300
Gm49338
Gm48604
Btbd8
Gm49759
Gm49337
Gm47483
Gm38431
Gm42528
Gm43268
Gm42738
Mpv17
Gm42549
Gm42732
Gm4366
4732416N19Rik
Gm3375
Zfp862-ps
Gm44240
Ccdc142
Gm44433
Gm45062
Gm38910
Gm2011
9530085L11Rik
Gm44257
Gm44242
Gm43879
4933427D14Rik
9330102E08Rik
Gm44230
Gm44235
Gm44438
D830050J10Rik
Gm44228
Gm44435
Gm44168
Gm43872
Gm44210
Gm44229
Gm43864
C030015A19Rik
6430584L05Rik
Gm20371
Gm44167
Pnma8c
Gm44950
Gm44509
Gm45477
Snhg1
Gm44510
AV356131
Particl
Gm35040
Gm33989
Gm45177
Gm49493
Gm44560
Gm44847
Gm45179
Gm44846

Gm3365
Gm49318
Gm47624
Gm47163
Gm47033
Gm48015
Rmst
Gm47591
BC024063
A830082N09Rik
Gm48226
Gm33869
Srp54b
4933412E12Rik
Gm47477
Gm47572
Gm48623
E430024I08Rik
D430020J02Rik
Rps18-ps5
Gm35161
Gm47863
Gm49654
Gm46430
Gm40841
Gm47260
Zfp935
Gm32699
Gm49327
2900060N12Rik
Gm49749
Gm7049
Gm35558
B230303A05Rik
Snhg10
Gm49602
Ndufb1
Scamp4
Gm47155
Gm34220
Btf3l4b
Gm48692
5330431K02Rik
F630042J09Rik
C630044B11Rik
C730002L08Rik
Gm49336
Gm49069
Gm49284
5830448L01Rik
Gm49032
Gm49685
Gm49747
B230209E15Rik
Gm45201
Gm44680
A230103L15Rik
Gm45159
Gm44798
4930431P19Rik
2900027M19Rik
Wdr97
Gm44676
Gm45176
Gm45220
A330076H08Rik
Prmt1
Samd4b
Gm44797
Gm44675
Gm19410
Gm49396
Gm44518
Gm45182
Gm45718
A230057D06Rik
Zfp582
Cpeb1os1
A930037H05Rik
Gm44800
Gm45223
Gm45130
Gm44677
Nup62
Gdf1
Gm44801
9330162G02Rik
Gm45178
B230206I08Rik
Gm44831
3110080E11Rik
Gm45809
Gm35021
Gm45606
Gm18194
Gm45605
Hspa14
Gm28756
Chmp1b
Brd3os
Gm45441
Gm7328
Gm6145
Smim32
Gm42067
Igip

Gm49037
Pnp
Rmrp
Gm56450
C230086J09Rik
Gm5468
Bc1
Gm17753
Vps28
Gm49593
Gm35853
C030006K11Rik
Pdxp
Gm49492
Nnt
Gm49502
4833412C15Rik
Lncppara
Gm49463
Gm30371
Riok2
Gm49601
Gm49678
Magef1
Fam246a
Gm1604a
Gm5165
A630036G19Rik
Gm49797
Gm49677
Morf4l1-ps1
Gm49674
Gm49599
Ermardl2
Gm21926
Gm30531
2700054A10Rik
Gm49949
Gm49909
Gm49883
C230013L11Rik
A230051N06Rik
Gm20008
Gm7818
Gm36684
Gm7072
Gm1043
Ptp4a1
Gm46633
Ntn3
Gm49980
Gm50010
Apbb3
Pde2a
Gm45799
C130073E24Rik
9330121K16Rik
Gm45871
Gm45263
Smim36
A030001D20Rik
Gm45457
Zbed5-ps
Gm45804
Gm10033
C230057M02Rik
Gm45743
Gm45779
Gm32856
Gm20163
C78859
Gm46218
Gpx4-ps2
Gm9856
Nudt8
Gm47204
Gm20300
Gm49338
5330432J10Rik
E030022I16Rik
Gm48604
Btbd8
Gm49759
Gm46123
Gm47483
Gm38431
Gm3365
Gm47163
Gm4864
Gm47801
Rmst
Gm47022
9530018F02Rik
BC024063
A830082N09Rik
Gm48226
Srp54b
Gm47590
4933412E12Rik
Gm47477
Gm47572
Gm48623
Gm47493
E430024I08Rik
Rps18-ps5
Gm48870

Snhg4
BC037704
Gm50240
Gm5242
Gm50462
Gm50388
Gm50431
Snhg4
Gm54798
Tmem223
Gm50403
Gm41760
Gm46620
Gm54728
Gm50387
Kctd16
Gm29695
Gm50107
Smim10l3
Gm30541
Gpr52
Gm50470
Gm13301

Gm48261
Gm48482
Gm49654
Gm46430
Gm40841
Gm48551
Gm47494
Gm48408
Gm47260
Zfp935
Gm32699
2900060N12Rik
Gm49749
Gm7049
Gm35558
Gm10432
B230303A05Rik
Snhg10
Gm49602
Gm48381
Ndufb1
Scamp4
Gm47155
Gm34220
Btf3l4b
5330431K02Rik
F630042J09Rik
Gm48582
Gm47246
Gm47694
Gm49336
Gm49069
Gm49284
5830448L01Rik
Gm48998
Gm49032
Gm49685
Gm20555
Gm49747
Pnp
Rmrp
Gm56450
C230086J09Rik
Gm9247
Bc1
Gm49526
Vps28
Gm49593
C030006K11Rik
Pdxp
Gm49492
Nnt
4833412C15Rik

Lncppara
Gm49463
Riok2
Gm49601
Magef1
Fam246a
A630089N07Rik
Gm1604a
Gm5165
A630036G19Rik
Gm49797
Gm49677
Morf4l1-ps1
Gm49674
Gm49599
Ermardl2
Gm21926
E130008D07Rik
Gm49909
Gm55377
C230013L11Rik
A230051N06Rik
Gm7818
Gm34567
Gm36684
Gm7072
Gm1043
Ptp4a1
Ntn3
Gm49980
Gm50010
Apbb3
BC037704
Gm50240
Gm50462
Gm50388
Gm50431
Gm50253
Snhg4
Gm54798
Tmem223
Gm50403
Itprip
Gm41760
Gm46620
Gm54728
Gm31356
Gm50387
Kctd16
Gm50107
Gm50268
Smim10l3
Gpr52

Gm50470
Gm13301

Supplementary Table 4. GO term enrichment for genes discordantly regulated between sexes in NAc and BNST (FuMd = female up, male down; MuFd = male up, female down)

region	overlap	ONTOLOGY	ID	Description	GeneRatio	BgRatio	pvalue
BNST	FuMd	BP	GO:0044057	regulation of system process	112/2029	402/11192	6.75258E-07
BNST	FuMd	BP	GO:0035418	protein localization to synapse	35/2029	93/11192	6.70054E-06
BNST	FuMd	BP	GO:0048167	regulation of synaptic plasticity	67/2029	223/11192	8.43942E-06
BNST	FuMd	BP	GO:0042752	regulation of circadian rhythm	34/2029	92/11192	1.42389E-05
BNST	FuMd	BP	GO:1903539	protein localization to postsynapt	26/2029	64/11192	2.12192E-05
BNST	FuMd	BP	GO:0003341	cilium movement	42/2029	125/11192	2.28073E-05
BNST	FuMd	BP	GO:0062237	protein localization to postsynaps	26/2029	65/11192	2.93257E-05
BNST	FuMd	BP	GO:0023061	signal release	108/2029	415/11192	2.99044E-05
BNST	FuMd	BP	GO:0050808	synapse organization	125/2029	494/11192	2.99135E-05
BNST	FuMd	BP	GO:2000463	positive regulation of excitatory p	18/2029	38/11192	3.43314E-05
BNST	FuMd	BP	GO:1990778	protein localization to cell periphe	90/2029	335/11192	3.87293E-05
BNST	FuMd	BP	GO:0071417	cellular response to organonitrog	113/2029	441/11192	4.05723E-05
BNST	FuMd	BP	GO:1901699	cellular response to nitrogen com	118/2029	469/11192	6.48457E-05
BNST	FuMd	BP	GO:0098815	modulation of excitatory postsyna	23/2029	57/11192	7.10671E-05
BNST	FuMd	BP	GO:0048511	rhythmic process	63/2029	220/11192	7.72163E-05
BNST	FuMd	BP	GO:0031347	regulation of defense response	93/2029	358/11192	0.000112702
BNST	FuMd	BP	GO:0099177	regulation of trans-synaptic signa	121/2029	490/11192	0.000124127
BNST	FuMd	BP	GO:0060294	cilium movement involved in cell	32/2029	93/11192	0.00012503
BNST	FuMd	BP	GO:0007623	circadian rhythm	48/2029	160/11192	0.000159789
BNST	FuMd	BP	GO:0050804	modulation of chemical synaptic	120/2029	489/11192	0.000174455
BNST	FuMd	BP	GO:0050807	regulation of synapse organizatic	73/2029	271/11192	0.000185284
BNST	FuMd	BP	GO:0097120	receptor localization to synapse	29/2029	83/11192	0.000188527
BNST	FuMd	BP	GO:0050803	regulation of synapse structure o	74/2029	276/11192	0.000196355
BNST	FuMd	BP	GO:0001539	cilium or flagellum-dependent ce	33/2029	99/11192	0.000196563
BNST	FuMd	BP	GO:0060285	cilium-dependent cell motility	33/2029	99/11192	0.000196563
BNST	FuMd	BP	GO:0001774	microglial cell activation	13/2029	26/11192	0.000216082
BNST	FuMd	BP	GO:0060291	long-term synaptic potentiation	35/2029	108/11192	0.000238089
BNST	FuMd	BP	GO:0106027	neuron projection organization	35/2029	108/11192	0.000238089
BNST	FuMd	BP	GO:0007018	microtubule-based movement	80/2029	306/11192	0.000266617
BNST	FuMd	BP	GO:0031644	regulation of nervous system pro	38/2029	121/11192	0.000271248
BNST	FuMd	BP	GO:0098876	vesicle-mediated transport to the	42/2029	138/11192	0.000284627
BNST	FuMd	BP	GO:0060079	excitatory postsynaptic potential	28/2029	81/11192	0.00029638
BNST	FuMd	BP	GO:0002269	leukocyte activation involved in ir	13/2029	27/11192	0.000347952
BNST	FuMd	BP	GO:0021801	cerebral cortex radial glia-guided	13/2029	27/11192	0.000347952
BNST	FuMd	BP	GO:0022030	telencephalon glial cell migration	13/2029	27/11192	0.000347952
BNST	FuMd	BP	GO:0061001	regulation of dendritic spine mor	21/2029	55/11192	0.000360237
BNST	FuMd	BP	GO:0030317	flagellated sperm motility	26/2029	75/11192	0.00045707
BNST	FuMd	BP	GO:0061669	spontaneous neurotransmitter se	7/2029	10/11192	0.000457607
BNST	FuMd	BP	GO:0099633	protein localization to postsynapt	16/2029	38/11192	0.000501499
BNST	FuMd	BP	GO:0099645	neurotransmitter receptor localiz	16/2029	38/11192	0.000501499
BNST	FuMd	BP	GO:0007015	actin filament organization	87/2029	345/11192	0.000530035
BNST	FuMd	BP	GO:0035082	axoneme assembly	23/2029	64/11192	0.000535086
BNST	FuMd	BP	GO:1902414	protein localization to cell junctio	37/2029	121/11192	0.000577033
BNST	FuMd	BP	GO:1903530	regulation of secretion by cell	112/2029	465/11192	0.000600337
BNST	FuMd	BP	GO:0044782	cilium organization	82/2029	324/11192	0.000672478

BNST	FuMd	BP	GO:0090090	negative regulation of canonical \	32/2029	101/11192	0.000673495
BNST	FuMd	BP	GO:0097722	sperm motility	26/2029	77/11192	0.000722911
BNST	FuMd	BP	GO:0051046	regulation of secretion	116/2029	487/11192	0.000751655
BNST	FuMd	BP	GO:0032922	circadian regulation of gene expr	22/2029	62/11192	0.000854613
BNST	FuMd	BP	GO:0007159	leukocyte cell-cell adhesion	52/2029	189/11192	0.000879771
BNST	FuMd	BP	GO:0150076	neuroinflammatory response	18/2029	47/11192	0.000893439
BNST	FuMd	BP	GO:0030031	cell projection assembly	112/2029	470/11192	0.000905622
BNST	FuMd	BP	GO:1903829	positive regulation of protein loca	100/2029	413/11192	0.000958867
BNST	FuMd	BP	GO:0099565	chemical synaptic transmission,	31/2029	99/11192	0.001009122
BNST	FuMd	BP	GO:0061900	glial cell activation	14/2029	33/11192	0.001019882
BNST	FuMd	BP	GO:0120031	plasma membrane bounded cell	110/2029	462/11192	0.001034812
BNST	FuMd	BP	GO:0006892	post-Golgi vesicle-mediated tran:	29/2029	91/11192	0.001062419
BNST	FuMd	CC	GO:0099572	postsynaptic specialization	119/2045	399/11259	5.25326E-09
BNST	FuMd	CC	GO:0032279	asymmetric synapse	112/2045	385/11259	6.59728E-08
BNST	FuMd	CC	GO:0098984	neuron to neuron synapse	119/2045	419/11259	1.09212E-07
BNST	FuMd	CC	GO:0014069	postsynaptic density	106/2045	363/11259	1.2089E-07
BNST	FuMd	CC	GO:0099091	postsynaptic specialization, intra	19/2045	46/11259	0.000212558
BNST	FuMd	CC	GO:0097060	synaptic membrane	109/2045	441/11259	0.000276052
BNST	FuMd	CC	GO:0044309	neuron spine	54/2045	195/11259	0.000627309
BNST	FuMd	CC	GO:0032838	plasma membrane bounded cell	48/2045	169/11259	0.00067014
BNST	FuMd	CC	GO:0005858	axonemal dynein complex	10/2045	19/11259	0.000712048
BNST	FuMd	CC	GO:0005930	axoneme	37/2045	122/11259	0.000712147
BNST	FuMd	CC	GO:0045211	postsynaptic membrane	77/2045	302/11259	0.000813973
BNST	FuMd	CC	GO:0097014	ciliary plasm	37/2045	123/11259	0.000843607
BNST	FuMd	CC	GO:0043197	dendritic spine	52/2045	189/11259	0.000920203
BNST	FuMd	CC	GO:0030863	cortical cytoskeleton	29/2045	92/11259	0.001327682
BNST	FuMd	CC	GO:0099634	postsynaptic specialization mem	39/2045	135/11259	0.001451893
BNST	FuMd	CC	GO:0099568	cytoplasmic region	55/2045	207/11259	0.001608775
BNST	FuMd	CC	GO:0031514	motile cilium	45/2045	165/11259	0.002367929
BNST	FuMd	MF	GO:0003779	actin binding	82/2016	301/11081	5.67015E-05
		BP	GO:0019646	aerobic electron transport chain	31/2728	48/11192	3.99907E-09
BNST	MuFd	BP	GO:0046390	ribose phosphate biosynthetic pr	76/2728	179/11192	6.51865E-08
BNST	MuFd	BP	GO:0046034	ATP metabolic process	53/2728	111/11192	6.67025E-08
BNST	MuFd	BP	GO:0009260	ribonucleotide biosynthetic proce	74/2728	173/11192	6.70606E-08
BNST	MuFd	BP	GO:0009201	ribonucleoside triphosphate bios	48/2728	97/11192	6.8525E-08
BNST	MuFd	BP	GO:0042773	ATP synthesis coupled electron t	33/2728	58/11192	1.13639E-07
BNST	MuFd	BP	GO:0042775	mitochondrial ATP synthesis cou	33/2728	58/11192	1.13639E-07
BNST	MuFd	BP	GO:0009199	ribonucleoside triphosphate meta	62/2728	139/11192	1.25707E-07
BNST	MuFd	BP	GO:0006754	ATP biosynthetic process	42/2728	83/11192	2.02756E-07
BNST	MuFd	BP	GO:0009205	purine ribonucleoside triphospha	59/2728	132/11192	2.30733E-07
BNST	MuFd	BP	GO:0009142	nucleoside triphosphate biosynth	49/2728	103/11192	2.38626E-07
BNST	MuFd	BP	GO:0006120	mitochondrial electron transport,	17/2728	22/11192	2.61866E-07
BNST	MuFd	BP	GO:0009206	purine ribonucleoside triphospha	45/2728	92/11192	2.69025E-07
BNST	MuFd	BP	GO:0009152	purine ribonucleotide biosyntheti	69/2728	163/11192	2.98369E-07
BNST	MuFd	BP	GO:0015986	proton motive force-driven ATP s	33/2728	60/11192	3.37173E-07
BNST	MuFd	BP	GO:0015980	energy derivation by oxidation of	99/2728	259/11192	3.8563E-07
BNST	MuFd	BP	GO:0009145	purine nucleoside triphosphate b	45/2728	93/11192	3.99183E-07
BNST	MuFd	BP	GO:0006164	purine nucleotide biosynthetic pr	75/2728	183/11192	4.39992E-07
BNST	MuFd	BP	GO:0006091	generation of precursor metaboli	127/2728	353/11192	4.90499E-07
BNST	MuFd	BP	GO:0019693	ribose phosphate metabolic proc	105/2728	280/11192	5.12555E-07
BNST	MuFd	BP	GO:0009144	purine nucleoside triphosphate m	60/2728	139/11192	7.91382E-07
BNST	MuFd	BP	GO:0009259	ribonucleotide metabolic process	102/2728	273/11192	9.0747E-07
BNST	MuFd	BP	GO:1901605	alpha-amino acid metabolic proc	54/2728	122/11192	1.08027E-06

BNST	MuFd	BP	GO:0009165	nucleotide biosynthetic process	81/2728	206/11192	1.17351E-06
BNST	MuFd	BP	GO:1901293	nucleoside phosphate biosynthet	81/2728	207/11192	1.47396E-06
BNST	MuFd	BP	GO:0072522	purine-containing compound bios	75/2728	188/11192	1.51976E-06
BNST	MuFd	BP	GO:0009141	nucleoside triphosphate metabol	64/2728	154/11192	1.71331E-06
BNST	MuFd	BP	GO:0006119	oxidative phosphorylation	50/2728	112/11192	1.97011E-06
BNST	MuFd	BP	GO:0009060	aerobic respiration	63/2728	152/11192	2.29433E-06
BNST	MuFd	BP	GO:0042776	proton motive force-driven mitocl	30/2728	56/11192	2.40767E-06
BNST	MuFd	BP	GO:0009150	purine ribonucleotide metabolic p	96/2728	259/11192	2.8233E-06
BNST	MuFd	BP	GO:0045333	cellular respiration	74/2728	189/11192	4.03092E-06
BNST	MuFd	BP	GO:0022900	electron transport chain	40/2728	85/11192	4.19902E-06
BNST	MuFd	BP	GO:0006520	amino acid metabolic process	70/2728	182/11192	1.48372E-05
BNST	MuFd	BP	GO:0042255	ribosome assembly	30/2728	60/11192	1.49198E-05
BNST	MuFd	BP	GO:0022904	respiratory electron transport cha	36/2728	77/11192	1.50275E-05
BNST	MuFd	BP	GO:0009117	nucleotide metabolic process	123/2728	361/11192	1.61744E-05
BNST	MuFd	BP	GO:0055086	nucleobase-containing small mol	137/2728	411/11192	1.90093E-05
BNST	MuFd	BP	GO:0006163	purine nucleotide metabolic proc	112/2728	325/11192	2.19072E-05
BNST	MuFd	BP	GO:0006753	nucleoside phosphate metabolic	124/2728	368/11192	2.68706E-05
BNST	MuFd	BP	GO:0002181	cytoplasmic translation	53/2728	131/11192	3.18064E-05
BNST	MuFd	BP	GO:0042273	ribosomal large subunit biogenes	33/2728	72/11192	5.54804E-05
BNST	MuFd	BP	GO:0009064	glutamine family amino acid met	25/2728	50/11192	7.58988E-05
BNST	MuFd	BP	GO:0070202	regulation of establishment of pro	9/2728	11/11192	0.000100992
BNST	MuFd	BP	GO:0070203	regulation of establishment of pro	9/2728	11/11192	0.000100992
BNST	MuFd	BP	GO:0070199	establishment of protein localizat	14/2728	22/11192	0.000105397
BNST	MuFd	BP	GO:0072521	purine-containing compound met	114/2728	343/11192	0.000106935
BNST	MuFd	BP	GO:1901137	carbohydrate derivative biosynth	152/2728	479/11192	0.000115604
BNST	MuFd	BP	GO:0070200	establishment of protein localizat	11/2728	16/11192	0.000221431
BNST	MuFd	BP	GO:1904851	positive regulation of establishme	8/2728	10/11192	0.000342109
BNST	MuFd	BP	GO:0006541	glutamine metabolic process	12/2728	19/11192	0.000371762
BNST	MuFd	BP	GO:0000027	ribosomal large subunit assembl	14/2728	24/11192	0.000389346
BNST	MuFd	BP	GO:1904814	regulation of protein localization	10/2728	15/11192	0.000634142
BNST	MuFd	BP	GO:0009063	amino acid catabolic process	26/2728	59/11192	0.000703731
BNST	MuFd	BP	GO:0042254	ribosome biogenesis	96/2728	295/11192	0.000808225
BNST	MuFd	BP	GO:0090407	organophosphate biosynthetic pr	132/2728	427/11192	0.001032885
BNST	MuFd	BP	GO:0033108	mitochondrial respiratory chain c	33/2728	82/11192	0.001042196
BNST	MuFd	CC	GO:0098800	inner mitochondrial membrane p	60/2756	121/11259	1.74658E-09
BNST	MuFd	CC	GO:0005746	mitochondrial respirasome	39/2756	69/11259	1.18264E-08
BNST	MuFd	CC	GO:0005743	mitochondrial inner membrane	162/2756	451/11259	2.00147E-08
BNST	MuFd	CC	GO:0098803	respiratory chain complex	38/2756	68/11259	2.77393E-08
BNST	MuFd	CC	GO:0070469	respirasome	40/2756	74/11259	4.23664E-08
BNST	MuFd	CC	GO:0098798	mitochondrial protein-containing	102/2756	262/11259	1.12748E-07
BNST	MuFd	CC	GO:0019866	organelle inner membrane	166/2756	478/11259	1.82396E-07
BNST	MuFd	CC	GO:1990204	oxidoreductase complex	45/2756	98/11259	2.81807E-06
BNST	MuFd	CC	GO:0005747	mitochondrial respiratory chain c	24/2756	42/11259	5.99286E-06
BNST	MuFd	CC	GO:0030964	NADH dehydrogenase complex	24/2756	42/11259	5.99286E-06
BNST	MuFd	CC	GO:0045271	respiratory chain complex I	24/2756	42/11259	5.99286E-06
BNST	MuFd	CC	GO:0005840	ribosome	84/2756	228/11259	1.79061E-05
BNST	MuFd	CC	GO:0044391	ribosomal subunit	70/2756	185/11259	3.24187E-05
BNST	MuFd	CC	GO:0022626	cytosolic ribosome	46/2756	114/11259	0.000123905
BNST	MuFd	CC	GO:0033177	proton-transporting two-sector A	12/2756	18/11259	0.000183883
BNST	MuFd	CC	GO:0016469	proton-transporting two-sector A	21/2756	41/11259	0.000194599
BNST	MuFd	CC	GO:0015934	large ribosomal subunit	44/2756	112/11259	0.000348645
BNST	MuFd	CC	GO:0022625	cytosolic large ribosomal subunit	25/2756	55/11259	0.000539
BNST	MuFd	CC	GO:0005741	mitochondrial outer membrane	61/2756	171/11259	0.000640239

BNST	MuFd	CC	GO:1902495	transmembrane transporter com|	90/2756	271/11259	0.000650528
BNST	MuFd	CC	GO:0019867	outer membrane	64/2756	183/11259	0.000868466
BNST	MuFd	CC	GO:0031968	organelle outer membrane	64/2756	183/11259	0.000868466
BNST	MuFd	CC	GO:0101031	protein folding chaperone comple	16/2756	32/11259	0.0015553
BNST	MuFd	CC	GO:1990351	transporter complex	93/2756	291/11259	0.002085577
BNST	MuFd	CC	GO:0005753	mitochondrial proton-transporting	10/2756	17/11259	0.002599339
BNST	MuFd	CC	GO:0070069	cytochrome complex	13/2756	25/11259	0.00272841
BNST	MuFd	CC	GO:1905369	endopeptidase complex	28/2756	70/11259	0.002875651
BNST	MuFd	CC	GO:0000502	proteasome complex	24/2756	58/11259	0.003321043
BNST	MuFd	MF	GO:0015453	oxidoreduction-driven active tran	14/2706	19/11081	8.42098E-06
BNST	MuFd	MF	GO:0009055	electron transfer activity	19/2706	31/11081	1.36491E-05
BNST	MuFd	MF	GO:0003735	structural constituent of ribosome	59/2706	153/11081	6.55006E-05
BNST	MuFd	MF	GO:0008137	NADH dehydrogenase (ubiquinoi	9/2706	11/11081	0.000102592
BNST	MuFd	MF	GO:0050136	NADH dehydrogenase (quinone)	9/2706	11/11081	0.000102592
BNST	MuFd	MF	GO:0016491	oxidoreductase activity	138/2706	432/11081	0.000184872
NAc	MuFd	BP	GO:0002181	cytoplasmic translation	72/1028	118/6304	1.65243E-28
NAc	MuFd	BP	GO:0140236	translation at presynapse	36/1028	46/6304	2.01261E-20
NAc	MuFd	BP	GO:0140241	translation at synapse	36/1028	47/6304	7.27496E-20
NAc	MuFd	BP	GO:0140242	translation at postsynapse	36/1028	47/6304	7.27496E-20
NAc	MuFd	BP	GO:0006119	oxidative phosphorylation	49/1028	91/6304	1.37957E-16
NAc	MuFd	BP	GO:0043043	peptide biosynthetic process	126/1028	395/6304	1.56759E-15
NAc	MuFd	BP	GO:0006412	translation	124/1028	387/6304	1.84908E-15
NAc	MuFd	BP	GO:0009060	aerobic respiration	57/1028	124/6304	4.97913E-15
NAc	MuFd	BP	GO:0015986	proton motive force-driven ATP s	35/1028	56/6304	6.66873E-15
NAc	MuFd	BP	GO:0042776	proton motive force-driven mitocl	33/1028	52/6304	2.10141E-14
NAc	MuFd	BP	GO:0006518	peptide metabolic process	137/1028	459/6304	3.26462E-14
NAc	MuFd	BP	GO:0043604	amide biosynthetic process	135/1028	452/6304	4.89081E-14
NAc	MuFd	BP	GO:0045333	cellular respiration	59/1028	145/6304	1.20161E-12
NAc	MuFd	BP	GO:0015980	energy derivation by oxidation of	68/1028	183/6304	3.65853E-12
NAc	MuFd	BP	GO:0009206	purine ribonucleoside triphospha	37/1028	74/6304	1.49232E-11
NAc	MuFd	BP	GO:0009201	ribonucleoside triphosphate bios	38/1028	78/6304	2.17167E-11
NAc	MuFd	BP	GO:0009145	purine nucleoside triphosphate b	37/1028	75/6304	2.49181E-11
NAc	MuFd	BP	GO:0006754	ATP biosynthetic process	35/1028	70/6304	5.34713E-11
NAc	MuFd	BP	GO:0009142	nucleoside triphosphate biosynth	38/1028	81/6304	9.09091E-11
NAc	MuFd	BP	GO:0046034	ATP metabolic process	39/1028	87/6304	2.82483E-10
NAc	MuFd	BP	GO:0033108	mitochondrial respiratory chain c	26/1028	46/6304	5.09222E-10
NAc	MuFd	BP	GO:0042773	ATP synthesis coupled electron t	27/1028	50/6304	9.47546E-10
NAc	MuFd	BP	GO:0042775	mitochondrial ATP synthesis cou	27/1028	50/6304	9.47546E-10
NAc	MuFd	BP	GO:0006091	generation of precursor metaboli	76/1028	240/6304	1.41071E-09
NAc	MuFd	BP	GO:0009144	purine nucleoside triphosphate m	42/1028	103/6304	2.10721E-09
NAc	MuFd	BP	GO:0009205	purine ribonucleoside triphospha	41/1028	100/6304	2.70369E-09
NAc	MuFd	BP	GO:0006164	purine nucleotide biosynthetic pr	49/1028	132/6304	4.28123E-09
NAc	MuFd	BP	GO:0072522	purine-containing compound bios	49/1028	132/6304	4.28123E-09
NAc	MuFd	BP	GO:0042254	ribosome biogenesis	51/1028	140/6304	4.37364E-09
NAc	MuFd	BP	GO:0009165	nucleotide biosynthetic process	52/1028	144/6304	4.37661E-09
NAc	MuFd	BP	GO:1901293	nucleoside phosphate biosynthet	52/1028	144/6304	4.37661E-09
NAc	MuFd	BP	GO:0010257	NADH dehydrogenase complex a	21/1028	35/6304	5.53065E-09
NAc	MuFd	BP	GO:0032981	mitochondrial respiratory chain c	21/1028	35/6304	5.53065E-09
NAc	MuFd	BP	GO:0009199	ribonucleoside triphosphate meta	42/1028	106/6304	5.90743E-09
NAc	MuFd	BP	GO:0009152	purine ribonucleotide biosyntheti	46/1028	122/6304	7.24241E-09
NAc	MuFd	BP	GO:0009141	nucleoside triphosphate metabol	43/1028	111/6304	8.73047E-09
NAc	MuFd	BP	GO:0022613	ribonucleoprotein complex bioge	68/1028	214/6304	9.18797E-09
NAc	MuFd	BP	GO:0046390	ribose phosphate biosynthetic pr	48/1028	131/6304	1.01046E-08

NAc	MuFd	BP	GO:0009260	ribonucleotide biosynthetic proce	47/1028	128/6304	1.33454E-08
NAc	MuFd	BP	GO:0022900	electron transport chain	30/1028	65/6304	1.433E-08
NAc	MuFd	BP	GO:0042274	ribosomal small subunit biogene:	25/1028	49/6304	1.85415E-08
NAc	MuFd	BP	GO:0022904	respiratory electron transport cha	29/1028	63/6304	2.69274E-08
NAc	MuFd	BP	GO:0019646	aerobic electron transport chain	22/1028	44/6304	2.13097E-07
NAc	MuFd	BP	GO:0009150	purine ribonucleotide metabolic p	55/1028	179/6304	8.44357E-07
NAc	MuFd	BP	GO:0019693	ribose phosphate metabolic proc	57/1028	188/6304	8.66356E-07
NAc	MuFd	BP	GO:0009259	ribonucleotide metabolic process	56/1028	185/6304	1.14587E-06
NAc	MuFd	BP	GO:0006364	rRNA processing	32/1028	86/6304	2.01163E-06
NAc	MuFd	BP	GO:0007005	mitochondrion organization	78/1028	292/6304	2.58514E-06
NAc	MuFd	BP	GO:0009117	nucleotide metabolic process	65/1028	232/6304	3.24532E-06
NAc	MuFd	BP	GO:0042273	ribosomal large subunit biogenes	19/1028	40/6304	3.88185E-06
NAc	MuFd	BP	GO:0006753	nucleoside phosphate metabolic	65/1028	235/6304	5.21404E-06
NAc	MuFd	BP	GO:0055086	nucleobase-containing small mol	69/1028	254/6304	5.30916E-06
NAc	MuFd	BP	GO:0006163	purine nucleotide metabolic proc	60/1028	216/6304	1.04385E-05
NAc	MuFd	BP	GO:0006626	protein targeting to mitochondrio	20/1028	47/6304	1.71498E-05
NAc	MuFd	BP	GO:0072655	establishment of protein localizat	22/1028	55/6304	2.14655E-05
NAc	MuFd	BP	GO:0006396	RNA processing	104/1028	440/6304	2.40107E-05
NAc	MuFd	BP	GO:0072521	purine-containing compound met	60/1028	222/6304	2.59778E-05
NAc	MuFd	BP	GO:0006605	protein targeting	52/1028	186/6304	3.34891E-05
NAc	MuFd	BP	GO:0016072	rRNA metabolic process	33/1028	104/6304	6.5723E-05
NAc	MuFd	BP	GO:0006457	protein folding	32/1028	100/6304	7.03539E-05
NAc	MuFd	BP	GO:0034470	ncRNA processing	43/1028	149/6304	7.12738E-05
NAc	MuFd	BP	GO:0045454	cell redox homeostasis	11/1028	20/6304	8.28391E-05
NAc	MuFd	BP	GO:0070585	protein localization to mitochondi	22/1028	60/6304	0.000102672
NAc	MuFd	BP	GO:0006120	mitochondrial electron transport,	11/1028	21/6304	0.000148468
NAc	MuFd	BP	GO:0002183	cytoplasmic translational initiatio	10/1028	18/6304	0.000158182
NAc	MuFd	BP	GO:1902600	proton transmembrane transport	22/1028	62/6304	0.000179275
NAc	MuFd	BP	GO:0006123	mitochondrial electron transport,	7/1028	10/6304	0.000228861
NAc	MuFd	BP	GO:0022618	protein-RNA complex assembly	32/1028	106/6304	0.000239489
NAc	MuFd	BP	GO:0051604	protein maturation	54/1028	212/6304	0.000345769
NAc	MuFd	BP	GO:0098754	detoxification	11/1028	23/6304	0.00041483
NAc	MuFd	BP	GO:0000470	maturation of LSU-rRNA	9/1028	17/6304	0.000550564
NAc	MuFd	BP	GO:0000028	ribosomal small subunit assembl	8/1028	14/6304	0.000580362
NAc	MuFd	BP	GO:0071826	protein-RNA complex organizatic	32/1028	111/6304	0.00059504
NAc	MuFd	BP	GO:1901137	carbohydrate derivative biosynth	67/1028	285/6304	0.000818318
NAc	MuFd	BP	GO:0045116	protein neddylation	8/1028	15/6304	0.001066808
NAc	MuFd	BP	GO:0042744	hydrogen peroxide catabolic proc	7/1028	12/6304	0.001115619
NAc	MuFd	BP	GO:0042255	ribosome assembly	14/1028	37/6304	0.001296985
NAc	MuFd	BP	GO:1902229	regulation of intrinsic apoptotic si	9/1028	19/6304	0.00153474
NAc	MuFd	BP	GO:0006413	translational initiation	23/1028	76/6304	0.001672204
NAc	MuFd	BP	GO:0006839	mitochondrial transport	24/1028	81/6304	0.001845418
NAc	MuFd	BP	GO:0090407	organophosphate biosynthetic pr	65/1028	284/6304	0.002006522
NAc	MuFd	CC	GO:0022626	cytosolic ribosome	67/1046	106/6344	1.31564E-27
NAc	MuFd	CC	GO:0044391	ribosomal subunit	69/1046	117/6344	8.66421E-26
NAc	MuFd	CC	GO:0005840	ribosome	80/1046	151/6344	1.88676E-25
NAc	MuFd	CC	GO:0022625	cytosolic large ribosomal subunit	39/1046	53/6344	3.63393E-20
NAc	MuFd	CC	GO:0098800	inner mitochondrial membrane p	55/1046	102/6344	2.78872E-18
NAc	MuFd	CC	GO:0015934	large ribosomal subunit	42/1046	71/6344	3.32305E-16
NAc	MuFd	CC	GO:0098798	mitochondrial protein-containing	70/1046	163/6344	5.48588E-16
NAc	MuFd	CC	GO:0005746	mitochondrial respirasome	37/1046	61/6344	6.45464E-15
NAc	MuFd	CC	GO:0098803	respiratory chain complex	37/1046	61/6344	6.45464E-15
NAc	MuFd	CC	GO:0070469	respirasome	37/1046	63/6344	2.76422E-14

NAc	MuFd	CC	GO:1990904	ribonucleoprotein complex	124/1046	408/6344	3.54124E-13
NAc	MuFd	CC	GO:0005740	mitochondrial envelope	117/1046	381/6344	8.15592E-13
NAc	MuFd	CC	GO:0019866	organelle inner membrane	91/1046	269/6344	8.66539E-13
NAc	MuFd	CC	GO:0005743	mitochondrial inner membrane	87/1046	255/6344	1.67015E-12
NAc	MuFd	CC	GO:0005747	mitochondrial respiratory chain c	25/1046	37/6344	5.1611E-12
NAc	MuFd	CC	GO:0030964	NADH dehydrogenase complex	25/1046	37/6344	5.1611E-12
NAc	MuFd	CC	GO:0045271	respiratory chain complex I	25/1046	37/6344	5.1611E-12
NAc	MuFd	CC	GO:0031966	mitochondrial membrane	111/1046	364/6344	5.55892E-12
NAc	MuFd	CC	GO:0022627	cytosolic small ribosomal subunit	25/1046	38/6344	1.27465E-11
NAc	MuFd	CC	GO:0015935	small ribosomal subunit	28/1046	50/6344	1.91381E-10
NAc	MuFd	CC	GO:1990204	oxidoreductase complex	36/1046	78/6344	6.97587E-10
NAc	MuFd	CC	GO:0042788	polysomal ribosome	20/1046	30/6344	1.0638E-09
NAc	MuFd	CC	GO:0005844	polysome	29/1046	59/6344	5.31383E-09
NAc	MuFd	CC	GO:0043209	myelin sheath	56/1046	186/6344	1.9929E-06
NAc	MuFd	CC	GO:0005839	proteasome core complex	10/1046	13/6344	2.52903E-06
NAc	MuFd	CC	GO:0032040	small-subunit processome	19/1046	40/6344	4.59989E-06
NAc	MuFd	CC	GO:0016469	proton-transporting two-sector A	17/1046	34/6344	6.11271E-06
NAc	MuFd	CC	GO:0005751	mitochondrial respiratory chain c	10/1046	14/6344	7.54076E-06
NAc	MuFd	CC	GO:0045277	respiratory chain complex IV	10/1046	14/6344	7.54076E-06
NAc	MuFd	CC	GO:0005753	mitochondrial proton-transporting	11/1046	17/6344	1.09819E-05
NAc	MuFd	CC	GO:0045259	proton-transporting ATP synthas	11/1046	17/6344	1.09819E-05
NAc	MuFd	CC	GO:0030684	preribosome	23/1046	57/6344	1.42225E-05
NAc	MuFd	CC	GO:0000276	mitochondrial proton-transporting	8/1046	10/6344	1.75498E-05
NAc	MuFd	CC	GO:0045263	proton-transporting ATP synthas	8/1046	10/6344	1.75498E-05
NAc	MuFd	CC	GO:0070069	cytochrome complex	12/1046	21/6344	2.60228E-05
NAc	MuFd	CC	GO:1905368	peptidase complex	25/1046	73/6344	0.000153945
NAc	MuFd	CC	GO:0140535	intracellular protein-containing cc	110/1046	486/6344	0.000157258
NAc	MuFd	CC	GO:0033177	proton-transporting two-sector A	9/1046	16/6344	0.000330032
NAc	MuFd	CC	GO:0000502	proteasome complex	18/1046	49/6344	0.000483433
NAc	MuFd	CC	GO:0031970	organelle envelope lumen	15/1046	41/6344	0.001476992
NAc	MuFd	CC	GO:0005758	mitochondrial intermembrane spa	13/1046	34/6344	0.001905076
NAc	MuFd	CC	GO:1905369	endopeptidase complex	18/1046	55/6344	0.00225469
NAc	MuFd	CC	GO:0033178	proton-transporting two-sector A	7/1046	14/6344	0.003816787
NAc	MuFd	CC	GO:1904949	ATPase complex	28/1046	103/6344	0.003898925
NAc	MuFd	CC	GO:0030127	COPII vesicle coat	6/1046	11/6344	0.004322111
NAc	MuFd	CC	GO:0046540	U4/U6 x U5 tri-snRNP complex	8/1046	19/6344	0.007294948
NAc	MuFd	CC	GO:0097526	spliceosomal tri-snRNP complex	8/1046	19/6344	0.007294948
NAc	MuFd	CC	GO:0005759	mitochondrial matrix	31/1046	122/6344	0.007323463
NAc	MuFd	CC	GO:0005681	spliceosomal complex	29/1046	113/6344	0.008050631
NAc	MuFd	MF	GO:0003735	structural constituent of ribosome	65/1006	103/6225	2.76409E-27
NAc	MuFd	MF	GO:0005198	structural molecule activity	91/1006	272/6225	5.22084E-13
NAc	MuFd	MF	GO:0019843	rRNA binding	23/1006	37/6225	3.07301E-10
NAc	MuFd	MF	GO:0016491	oxidoreductase activity	63/1006	222/6225	2.09981E-06
NAc	MuFd	MF	GO:0015453	oxidoreduction-driven active tran	11/1006	16/6225	3.71072E-06
NAc	MuFd	MF	GO:0015078	proton transmembrane transporte	23/1006	59/6225	1.97675E-05
NAc	MuFd	MF	GO:0015252	proton channel activity	9/1006	13/6225	2.80051E-05
NAc	MuFd	MF	GO:0046933	proton-transporting ATP synthas	9/1006	13/6225	2.80051E-05
NAc	MuFd	MF	GO:0009055	electron transfer activity	12/1006	22/6225	3.95735E-05
NAc	MuFd	MF	GO:0043021	ribonucleoprotein complex bindin	33/1006	109/6225	0.000151674
NAc	MuFd	MF	GO:0051082	unfolded protein binding	20/1006	54/6225	0.000157874
NAc	MuFd	MF	GO:0016655	oxidoreductase activity, acting or	8/1006	13/6225	0.000271776
NAc	MuFd	MF	GO:0008137	NADH dehydrogenase (ubiquinoi	7/1006	11/6225	0.000510498
NAc	MuFd	MF	GO:0050136	NADH dehydrogenase (quinone)	7/1006	11/6225	0.000510498

NAc	MuFd	MF	GO:0015399	primary active transmembrane tr	22/1006	67/6225	0.000549527
NAc	MuFd	MF	GO:0016209	antioxidant activity	11/1006	25/6225	0.000922535
NAc	MuFd	MF	GO:0003955	NAD(P)H dehydrogenase (quino	7/1006	12/6225	0.001054727
NAc	MuFd	MF	GO:0045182	translation regulator activity	25/1006	84/6225	0.001222061
NAc	MuFd	MF	GO:0003954	NADH dehydrogenase activity	7/1006	13/6225	0.00196817
NAc	MuFd	MF	GO:0008135	translation factor activity, RNA bi	18/1006	56/6225	0.002243811
NAc	MuFd	MF	GO:0090079	translation regulator activity, nucl	20/1006	65/6225	0.002362582
NAc	MuFd	MF	GO:0048027	mRNA 5'-UTR binding	8/1006	17/6225	0.002798442
NAc	MuFd	MF	GO:0030544	Hsp70 protein binding	12/1006	32/6225	0.002871054
NAc	MuFd	MF	GO:0031625	ubiquitin protein ligase binding	47/1006	198/6225	0.003220251
NAc	MuFd	MF	GO:0016651	oxidoreductase activity, acting or	9/1006	21/6225	0.003365963
NAc	MuFd	MF	GO:0003746	translation elongation factor activ	7/1006	14/6225	0.003391754
NAc	MuFd	MF	GO:0031386	protein tag activity	6/1006	11/6225	0.003892337
NAc	MuFd	MF	GO:0141047	molecular tag activity	6/1006	11/6225	0.003892337
NAc	FuMd	BP	GO:0050808	synapse organization	147/1608	403/6304	3.05346E-07
NAc	FuMd	BP	GO:0120039	plasma membrane bounded cell	174/1608	495/6304	4.26699E-07
NAc	FuMd	BP	GO:0048812	neuron projection morphogenesis	171/1608	486/6304	5.05117E-07
NAc	FuMd	BP	GO:0048858	cell projection morphogenesis	174/1608	497/6304	5.79119E-07
NAc	FuMd	BP	GO:0042391	regulation of membrane potentia	100/1608	261/6304	2.21848E-06
NAc	FuMd	BP	GO:0007409	axonogenesis	116/1608	319/6304	6.85713E-06
NAc	FuMd	BP	GO:0048667	cell morphogenesis involved in n	150/1608	434/6304	8.44554E-06
NAc	FuMd	BP	GO:0061564	axon development	124/1608	347/6304	8.85136E-06
NAc	FuMd	BP	GO:0050770	regulation of axonogenesis	57/1608	138/6304	2.98415E-05
NAc	FuMd	BP	GO:0050804	modulation of chemical synaptic	138/1608	405/6304	4.39596E-05
NAc	FuMd	BP	GO:0099177	regulation of trans-synaptic signa	138/1608	406/6304	5.03213E-05
NAc	FuMd	BP	GO:0050905	neuromuscular process	47/1608	111/6304	7.07163E-05
NAc	FuMd	BP	GO:0009101	glycoprotein biosynthetic process	48/1608	116/6304	0.000118162
NAc	FuMd	BP	GO:0030001	metal ion transport	131/1608	390/6304	0.00014358
NAc	FuMd	BP	GO:0001508	action potential	37/1608	84/6304	0.000155047
NAc	FuMd	BP	GO:0007219	Notch signaling pathway	30/1608	65/6304	0.000236241
NAc	FuMd	BP	GO:1990138	neuron projection extension	56/1608	145/6304	0.000300571
NAc	FuMd	BP	GO:0044090	positive regulation of vacuole org	11/1608	16/6304	0.00033908
NAc	FuMd	BP	GO:0010975	regulation of neuron projection de	124/1608	374/6304	0.00039627
NAc	FuMd	BP	GO:2000050	regulation of non-canonical Wnt	8/1608	10/6304	0.0004775
NAc	FuMd	CC	GO:0097060	synaptic membrane	128/1619	358/6344	6.30261E-06
NAc	FuMd	CC	GO:0043025	neuronal cell body	158/1619	466/6344	1.68558E-05
NAc	FuMd	CC	GO:0044304	main axon	33/1619	67/6344	2.29327E-05
NAc	FuMd	CC	GO:0042734	presynaptic membrane	63/1619	161/6344	8.38731E-05
NAc	FuMd	CC	GO:0045211	postsynaptic membrane	89/1619	245/6344	8.71212E-05
NAc	FuMd	CC	GO:0098839	postsynaptic density membrane	41/1619	94/6344	9.23243E-05
NAc	FuMd	CC	GO:0043197	dendritic spine	65/1619	168/6344	9.68409E-05
NAc	FuMd	CC	GO:0150034	distal axon	96/1619	269/6344	0.000100742
NAc	FuMd	CC	GO:0044309	neuron spine	66/1619	173/6344	0.000140112
NAc	FuMd	CC	GO:0044298	cell body membrane	14/1619	22/6344	0.000177742
NAc	FuMd	CC	GO:0034702	monoatomic ion channel comple	57/1619	150/6344	0.000439864
NAc	FuMd	CC	GO:0032809	neuronal cell body membrane	13/1619	21/6344	0.00045185
NAc	FuMd	CC	GO:0098984	neuron to neuron synapse	124/1619	375/6344	0.000458015
NAc	FuMd	CC	GO:0099634	postsynaptic specialization mem	45/1619	115/6344	0.000835165
NAc	FuMd	CC	GO:0032589	neuron projection membrane	24/1619	52/6344	0.000980585
NAc	FuMd	CC	GO:0032279	asymmetric synapse	114/1619	348/6344	0.001132401
NAc	FuMd	CC	GO:0034703	cation channel complex	39/1619	98/6344	0.001246698
NAc	FuMd	CC	GO:0048786	presynaptic active zone	38/1619	95/6344	0.00128006
NAc	FuMd	CC	GO:0031256	leading edge membrane	41/1619	105/6344	0.001464473

NAc	FuMd	CC	GO:0031252	cell leading edge	89/1619	264/6344	0.00148607
NAc	FuMd	CC	GO:0014069	postsynaptic density	108/1619	330/6344	0.001574004
NAc	FuMd	CC	GO:0098797	plasma membrane protein comp	78/1619	232/6344	0.003084174
NAc	FuMd	CC	GO:0044224	juxtaparanode region of axon	8/1619	12/6344	0.003175156
NAc	FuMd	CC	GO:0031594	neuromuscular junction	28/1619	68/6344	0.00326579
NAc	FuMd	CC	GO:0030427	site of polarized growth	58/1619	165/6344	0.00339832
NAc	FuMd	CC	GO:0034704	calcium channel complex	17/1619	36/6344	0.003914823
NAc	FuMd	CC	GO:0030426	growth cone	56/1619	160/6344	0.004395385
NAc	FuMd	MF	GO:0046873	metal ion transmembrane transp	80/1609	190/6225	5.35724E-07
NAc	FuMd	MF	GO:0022843	voltage-gated monoatomic catior	36/1609	70/6225	3.74778E-06
NAc	FuMd	MF	GO:0005261	monoatomic cation channel activ	59/1609	136/6225	5.44217E-06
NAc	FuMd	MF	GO:0015085	calcium ion transmembrane tran:	35/1609	70/6225	1.15136E-05
NAc	FuMd	MF	GO:0005244	voltage-gated monoatomic ion cl	44/1609	97/6225	2.22185E-05
NAc	FuMd	MF	GO:0022832	voltage-gated channel activity	44/1609	97/6225	2.22185E-05
NAc	FuMd	MF	GO:0005262	calcium channel activity	30/1609	59/6225	3.19392E-05
NAc	FuMd	MF	GO:1901702	salt transmembrane transporter a	85/1609	226/6225	4.88555E-05
NAc	FuMd	MF	GO:0022839	monoatomic ion gated channel a	56/1609	138/6225	9.33865E-05
NAc	FuMd	MF	GO:0022836	gated channel activity	56/1609	141/6225	0.00018478
NAc	FuMd	MF	GO:0008324	monoatomic cation transmembra	87/1609	244/6225	0.000345508
NAc	FuMd	MF	GO:0005216	monoatomic ion channel activity	67/1609	179/6225	0.000354174
NAc	FuMd	MF	GO:0022890	inorganic cation transmembrane	86/1609	241/6225	0.000361918
NAc	FuMd	MF	GO:0015267	channel activity	70/1609	192/6225	0.000632189
NAc	FuMd	MF	GO:0022803	passive transmembrane transpo	70/1609	192/6225	0.000632189
NAc	FuMd	MF	GO:0099094	ligand-gated monoatomic cation	17/1609	33/6225	0.001381255
NAc	FuMd	MF	GO:0030695	GTPase regulator activity	88/1609	260/6225	0.00206694
NAc	FuMd	MF	GO:0060589	nucleoside-triphosphatase regula	88/1609	260/6225	0.00206694
NAc	FuMd	MF	GO:0015079	potassium ion transmembrane tr	29/1609	69/6225	0.002364243
NAc	FuMd	MF	GO:0015318	inorganic molecular entity transm	92/1609	275/6225	0.002459628
NAc	FuMd	MF	GO:0005245	voltage-gated calcium channel a	15/1609	29/6225	0.002494821
NAc	FuMd	MF	GO:0005249	voltage-gated potassium channe	19/1609	40/6225	0.002513062
NAc	FuMd	MF	GO:0016772	transferase activity, transferring p	131/1609	412/6225	0.003009034
NAc	FuMd	MF	GO:0016773	phosphotransferase activity, alco	108/1609	332/6225	0.003080482

p.adjust	qvalue	geneID	Count
0.003433689	0.003290285	Srf/Anxa6/Akap1/Scn1b/Atp2	112
0.014304822	0.0137074	Cacng2/Asic2/Grin2c/Dlg1/G	35
0.014304822	0.0137074	Prrt1/Srf/Psen1/Eif4ebp2/Adc	67
0.016901101	0.016195249	Cry1/Fbxw11/Adcy1/Rock2/Ic	34
0.016901101	0.016195249	Cacng2/Grin2c/Dlg1/Gsk3b/C	26
0.016901101	0.016195249	Pltp/Pfn4/Nherf1/Tekt1/Dnah	42
0.016901101	0.016195249	Cacng2/Grin2c/Dlg1/Gsk3b/C	26
0.016901101	0.016195249	Rhot1/Slc25a22/Psen1/Cry1/	108
0.016901101	0.016195249	Prrt1/Cntnap1/Cacng2/Wasf1	125
0.017192527	0.016474503	Grin2c/Shank3/Igsf11/Gsk3b	18
0.017192527	0.016474503	Cacng2/Map7/Stx7/Sptbn1/R	90
0.017192527	0.016474503	Castor2/Myo1c/Pip4k2b/Akt3	113
0.025364638	0.024305316	Castor2/Myo1c/Pip4k2b/Akt3	118
0.025812576	0.024734547	Grin2c/Shank3/Igsf11/Gsk3b	23
0.026176325	0.025083104	Cry1/Fbxw11/Adcy1/Nampt/F	63
0.035320884	0.033845753	Ttbk1/C1qbp/Peli1/Appl2/Rtn	93
0.035320884	0.033845753	Prrt1/Srf/Rhot1/Cacng2/Pser	121
0.035320884	0.033845753	Pltp/Pfn4/Tekt1/Dnah5/Rabl2	32
0.039980954	0.038311201	Cry1/Fbxw11/Adcy1/Nampt/F	48
0.039980954	0.038311201	Prrt1/Srf/Rhot1/Cacng2/Pser	120
0.039980954	0.038311201	Cntnap1/Psen1/Rock2/Asic2	73
0.039980954	0.038311201	Cacng2/Stx7/Cacng4/Dlg1/G	29
0.039980954	0.038311201	Cntnap1/Psen1/Rock2/Asic2	74
0.039980954	0.038311201	Pltp/Pfn4/Tekt1/Dnah5/Rabl2	33
0.039980954	0.038311201	Pltp/Pfn4/Tekt1/Dnah5/Rabl2	33
0.042260609	0.04049565	Ttbk1/Nampt/App/Ifngr2/Jak2	13
0.043238629	0.041432824	Prrt1/Psen1/Adcy1/Grin2c/Gt	35
0.043238629	0.041432824	Psen1/Pafah1b1/Shank3/Gsl	35
0.045976463	0.044056316	Ift27/Rhot1/Pltp/Wasf1/Rab1	80
0.045976463	0.044056316	Rock2/Cacng4/Grin2c/Hnrnp	38
0.04668801	0.044738146	Steap2/Vps53/Rab11fip4/Spt	42
0.047096692	0.04512976	Grin2c/Shank3/Igsf11/Gsk3b	28
0.050552421	0.048441165	Ttbk1/Nampt/App/Ifngr2/Jak2	13
0.050552421	0.048441165	Rtn4/Pafah1b1/Foxg1/Gli3/S	13
0.050552421	0.048441165	Rtn4/Pafah1b1/Foxg1/Gli3/S	13
0.050883527	0.048758443	Pafah1b1/Shank3/Baiap2/Ng	21
0.061235001	0.058677601	Pltp/Pfn4/Dnah5/Rabl2/Cfap4	26
0.061235001	0.058677601	Doc2b/Prkn/Rph3a/Stx1b/Pp	7
0.063753112	0.061090546	Cacng2/Dlg1/Gsk3b/Camk2a	16
0.063753112	0.061090546	Cacng2/Dlg1/Gsk3b/Camk2a	16
0.064783628	0.062078024	Srf/Eps8/Rhot1/Myo1c/Wasf1	87
0.064783628	0.062078024	Dnah5/Rsph1/Cfap206/Dcx/N	23
0.068237559	0.065387706	Cacng2/Asic2/Grin2c/Dlg1/G	37
0.069379854	0.066482294	Rhot1/Slc25a22/Cry1/Rtn4/L	112
0.074450494	0.071341165	Ift27/Pfn4/Nherf1/Tekt1/Fam	82

0.074450494	0.071341165	Stk4/Kremen1/Nherf1/Ccdc8	32
0.078212788	0.074946332	Pltp/Pfn4/Dnah5/Rabl2/Cfap4	26
0.07962842	0.076302842	Rhot1/Slc25a22/Cry1/Rtn4/L	116
0.08855935	0.084860783	Cry1/Nampt/Id2/Per1/Csnk1ε	22
0.08855935	0.084860783	Peli1/Stk10/Gli3/Ap3b1/Cyrib	52
0.08855935	0.084860783	Ttbk1/Psen1/Adcy1/Nampt/A	18
0.08855935	0.084860783	Srf/Eps8/Ift27/Cobl/Cyfip2/Pf	112
0.091997001	0.088154865	Myo1c/Cacng2/Psen1/Sptbn	100
0.093964637	0.090040326	Grin2c/Shank3/Igsf11/Gsk3b	31
0.093964637	0.090040326	Ttbk1/Psen1/Nampt/App/Ifng	14
0.093964637	0.090040326	Srf/Eps8/Ift27/Cobl/Pfn4/Nhe	110
0.094778962	0.090820642	Steap2/Sptbn1/Llgl1/Rab10/(	29
3.26227E-06	2.95841E-06	Prrt1/Eps8/Akap1/Cacng2/Sp	119
1.87682E-05	1.70201E-05	Prrt1/Eps8/Cacng2/Sptbn1/A	112
1.87682E-05	1.70201E-05	Prrt1/Eps8/Cacng2/Sptbn1/A	119
1.87682E-05	1.70201E-05	Prrt1/Eps8/Cacng2/Sptbn1/A	106
0.026399747	0.023940782	Akap1/Shank3/Dlg1/Baiap2/S	19
0.028571431	0.025910188	Prrt1/Akap1/Cacng2/Slc6a8/	109
0.043656688	0.039590352	Atp2b1/Asic2/Myh10/Numb/H	54
0.043656688	0.039590352	Eif4ebp2/Hnrnpab/Cfap36/Ki	48
0.043656688	0.039590352	Dnah5/Dnhd1/Dnai2/Dnai4/D	10
0.043656688	0.039590352	Cfap36/Tekt1/Gli3/Dnah5/Rs	37
0.043656688	0.039590352	Prrt1/Akap1/Cacng2/Slc6a8/	77
0.043656688	0.039590352	Cfap36/Tekt1/Gli3/Dnah5/Rs	37
0.043957412	0.039863066	Atp2b1/Asic2/Myh10/Numb/H	52
0.058892177	0.053406754	Cobl/Sptbn1/Llgl1/Sptb/Myh9	29
0.060108371	0.054509668	Prrt1/Cacng2/Adcy1/Rtn4/Ca	39
0.062440565	0.056624633	Eif4ebp2/Hnrnpab/Cfap36/Ki	55
0.086499036	0.078442214	Ift27/Cfap36/Nherf1/Pafah1b	45
0.053582913	0.052463805	Eps8/Myo1c/Anxa6/Wasf1/C	82
2.04753E-05	2.04753E-05	Ndufv2/Ndufs3/Cyc1/Uqcrh/(	31
7.01696E-05	7.01696E-05	Ndufv2/Atp5pd/Ppargc1a/Nd	76
7.01696E-05	7.01696E-05	Ndufv2/Atp5pd/Ppargc1a/Nd	53
7.01696E-05	7.01696E-05	Ndufv2/Atp5pd/Ppargc1a/Nd	74
7.01696E-05	7.01696E-05	Ndufv2/Atp5pd/Ppargc1a/Nd	48
8.04522E-05	8.04522E-05	Ndufv2/Ndufs3/Cyc1/Uqcrh/(	33
8.04522E-05	8.04522E-05	Ndufv2/Ndufs3/Cyc1/Uqcrh/(	33
8.04522E-05	8.04522E-05	Ndufv2/Atp5pd/Ppargc1a/Nd	62
0.000105954	0.000105954	Ndufv2/Atp5pd/Ppargc1a/Nd	42
0.000105954	0.000105954	Ndufv2/Atp5pd/Ppargc1a/Nd	59
0.000105954	0.000105954	Ndufv2/Atp5pd/Ppargc1a/Nd	49
0.000105954	0.000105954	Ndufv2/Ndufs3/Coq9/Ndufs2/	17
0.000105954	0.000105954	Ndufv2/Atp5pd/Ppargc1a/Nd	45
0.000109118	0.000109118	Ndufv2/Atp5pd/Ppargc1a/Nd	69
0.000115088	0.000115088	Ndufv2/Atp5pd/Ndufs3/Atp5p	33
0.000120225	0.000120225	Acadm/Ndufv2/Atp5pd/Pparg	99
0.000120225	0.000120225	Ndufv2/Atp5pd/Ppargc1a/Nd	45
0.000125153	0.000125153	Ndufv2/Atp5pd/Ppargc1a/Nd	75
0.000131214	0.000131214	Acadm/Ndufv2/Atp5pd/Pparg	127
0.000131214	0.000131214	Ndufv2/Atp5pd/Ppargc1a/Nd	105
0.000192946	0.000192946	Ndufv2/Atp5pd/Ppargc1a/Nd	60
0.000211193	0.000211193	Ndufv2/Atp5pd/Ppargc1a/Nd	102
0.000240478	0.000240478	Adss1/Fpgs/Sardh/Apip/Qdpr	54

0.000250348	0.000250348	Ndufv2/Atp5pd/Ppargc1a/Nd	81
0.000299276	0.000299276	Ndufv2/Atp5pd/Ppargc1a/Nd	81
0.000299276	0.000299276	Ndufv2/Atp5pd/Ppargc1a/Nd	75
0.000324895	0.000324895	Ndufv2/Atp5pd/Ppargc1a/Nd	64
0.000360248	0.000360248	Ndufv2/Atp5pd/Ndufs3/Cyc1/	50
0.000405069	0.000405069	Ndufv2/Atp5pd/Ndufs3/Cyc1/	63
0.00041091	0.00041091	Ndufv2/Atp5pd/Ndufs3/Atp5p	30
0.0004663	0.0004663	Ndufv2/Atp5pd/Ppargc1a/Nd	96
0.000644947	0.000644947	Ndufv2/Atp5pd/Ppargc1a/Nd	74
0.000651484	0.000651484	Ndufv2/Ppargc1a/Ndufs3/Cy	40
0.00213725	0.00213725	Gars/Adss1/Fpgs/Sardh/Car	70
0.00213725	0.00213725	Rpl10/Rps5/Rrp7a/Nle1/Eif5/	30
0.00213725	0.00213725	Ndufv2/Ppargc1a/Ndufs3/Cy	36
0.002238183	0.002238183	Ndufv2/Atp5pd/Ppargc1a/Nd	123
0.002561258	0.002561258	Ndufv2/Atp5pd/Ppargc1a/Nd	137
0.00287602	0.00287602	Ndufv2/Atp5pd/Ppargc1a/Nd	112
0.003439434	0.003439434	Ndufv2/Atp5pd/Ppargc1a/Nd	124
0.003971923	0.003971923	Rpl37a/Eif3m/Mcts1/Rpl8/Sl	53
0.00676332	0.00676332	Ddx18/Nhp2/Rpl10/Znhit3/Nl	33
0.009037248	0.009037248	Fpgs/Pfas/Glud1/Pycr3/Nit2/	25
0.01149062	0.01149062	Cct8/Cct4/Cct5/Terf1/Cct6a/	9
0.01149062	0.01149062	Cct8/Cct4/Cct5/Terf1/Cct6a/	9
0.011649043	0.011649043	Cct8/Ruvbl2/Cct4/Macroh2a1	14
0.011649043	0.011649043	Ndufv2/Atp5pd/Ppargc1a/Nd	114
0.012331051	0.012331051	Ndufv2/Atp5pd/Ppargc1a/Nd	152
0.023137299	0.023137299	Cct8/Cct4/Cct5/Terf1/Cct6a/	11
0.035032004	0.035032004	Cct8/Cct4/Cct5/Cct6a/Cct7/	8
0.037322001	0.037322001	Pfas/Glud1/Nit2/Glul/Gmps/	12
0.038335594	0.038335594	Rpl10/Nle1/Mrto4/Mrpl20/Mr	14
0.061260544	0.061260544	Cct8/Cct4/Macroh2a1/Cct5/T	10
0.066724085	0.066724085	Sardh/Qdpr/Tha1/Dld/Gstz1/	26
0.075238402	0.075238402	Ddx18/Bud23/Nhp2/Nob1/En	96
0.093614834	0.093614834	Ndufv2/Atp5pd/Ppargc1a/Nd	132
0.093614834	0.093614834	Ndufs3/Ndufa11/Tfam/Uqcc1	33
1.12829E-06	1.08104E-06	Ndufv2/Atp5pd/Ndufs3/Cyc1/	60
3.81991E-06	3.65995E-06	Ndufv2/Ndufs3/Cyc1/Uqcrh/	39
4.30984E-06	4.12936E-06	Mrpl12/Ndufv2/Atp5pd/Ndufs	162
4.47989E-06	4.29229E-06	Ndufv2/Ndufs3/Cyc1/Uqcrh/	38
5.47374E-06	5.24451E-06	Ndufv2/Ndufs3/Cyc1/Uqcrh/	40
1.21392E-05	1.16308E-05	Mrpl12/Ndufv2/Atp5pd/Ndufs	102
1.68326E-05	1.61277E-05	Mrpl12/Ndufv2/Atp5pd/Ndufs	166
0.000227559	0.00021803	Ndufv2/Ndufs3/Cyc1/Uqcrh/	45
0.000351945	0.000337206	Ndufv2/Ndufs3/Ndufa11/Ndu	24
0.000351945	0.000337206	Ndufv2/Ndufs3/Ndufa11/Ndu	24
0.000351945	0.000337206	Ndufv2/Ndufs3/Ndufa11/Ndu	24
0.000963943	0.000923575	Mrpl12/Ppargc1a/Ubb/Rpl37	84
0.001610958	0.001543495	Mrpl12/Ubb/Rpl37a/Mrpl34/	70
0.005717349	0.005477923	Ppargc1a/Ubb/Rpl37a/Mcts1	46
0.007856921	0.007527896	Atp5pd/Atp5pb/Atp5mc1/Atp	12
0.007856921	0.007527896	Atp5pd/Atp5pb/Atp5mj/Atp5f	21
0.013248524	0.012693713	Mrpl12/Rpl37a/Mrpl34/Mrpl5	44
0.019344107	0.018534031	Rpl37a/Nhp2/Rpl8/Rpl10/Rpl	25
0.021012061	0.020132136	Ubc/Ubb/Coasy/Cnp/Bcl2l1/	61

0.021012061	0.020132136	Ndufv2/Ndufs3/Cyc1/Uqcrh/C	90
0.02550132	0.024433398	Ubc/Ubb/Coasy/Emd/Cnp/Bc	64
0.02550132	0.024433398	Ubc/Ubb/Coasy/Emd/Cnp/Bc	64
0.043683644	0.041854298	Cct8/Sdf2/Ppp5c/Ruvbl2/Cct	16
0.056136793	0.053785944	Ndufv2/Ndufs3/Cyc1/Uqcrh/C	93
0.067166922	0.064354164	Atp5pd/Atp5pb/Atp5mj/Atp5f	10
0.067790507	0.064951634	Cyc1/Uqcrh/Cox5a/Cox7c/Uc	13
0.068802614	0.065921357	Psmd11/Psmc6/Capns1/Rad	28
0.076621214	0.073412537	Psmd11/Psmc6/Rad23a/Psn	24
0.006606167	0.006515652	Ndufv2/Ndufs3/Cyc1/Uqcrh/C	14
0.006606167	0.006515652	Ndufv2/Ndufs3/Cyc1/Uqcrh/C	19
0.019861808	0.01958967	Mrpl12/Ubb/Rpl37a/Mrpl34/M	59
0.019861808	0.01958967	Ndufv2/Ndufs3/Ndufs2/Ndufa	9
0.019861808	0.01958967	Ndufv2/Ndufs3/Ndufs2/Ndufa	9
0.029825972	0.029417308	Acadm/Ndufv2/Ndufs3/Cyc1/	138
6.32714E-25	6.3001E-25	Rpl11/Rps14/Rpl36/Rps23/R	72
3.85315E-17	3.83668E-17	Rps14/Rpl36/Rpl23/Rpl38/Rp	36
6.96395E-17	6.93418E-17	Rps14/Rpl36/Rpl23/Rpl38/Rp	36
6.96395E-17	6.93418E-17	Rps14/Rpl36/Rpl23/Rpl38/Rp	36
1.05647E-13	1.05196E-13	Ndufa2/Cox7c/Ndufb3/Atp5p	49
1.00038E-12	9.96106E-13	Rpl11/Rps14/Rpl36/Rps23/R	126
1.01145E-12	1.00712E-12	Rpl11/Rps14/Rpl36/Rps23/R	124
2.38313E-12	2.37295E-12	Ndufa2/Cox7c/Ndufb3/Atp5p	57
2.83717E-12	2.82504E-12	Ndufa2/Ndufb3/Atp5pd/Ndufs	35
8.04629E-12	8.01189E-12	Ndufa2/Ndufb3/Atp5pd/Ndufs	33
1.13639E-11	1.13153E-11	Rpl11/Rps14/Rpl36/Rps23/R	137
1.56058E-11	1.55391E-11	Rpl11/Rps14/Rpl36/Rps23/R	135
3.53921E-10	3.52408E-10	Ndufa2/Cox7c/Ndufb3/Atp5p	59
1.00061E-09	9.96331E-10	Ndufa2/Cox7c/Ppp1cb/Ndufb	68
3.80939E-09	3.7931E-09	Nme1/Ndufa2/Ndufb3/Atp5p	37
5.19707E-09	5.17486E-09	Nme1/Ndufa2/Ndufb3/Atp5p	38
5.61244E-09	5.58845E-09	Nme1/Ndufa2/Ndufb3/Atp5p	37
1.13745E-08	1.13259E-08	Ndufa2/Ndufb3/Atp5pd/Ndufs	35
1.83206E-08	1.82422E-08	Nme1/Ndufa2/Ndufb3/Atp5p	38
5.40813E-08	5.38501E-08	Ndufa2/Ndufb3/Atp5pd/Ndufs	39
9.28482E-08	9.24513E-08	Ndufa2/Ndufb3/Ndufs6/Ndufa	26
1.57746E-07	1.57071E-07	Cox7c/Ndufs6/Ndufv2/Ndufc2	27
1.57746E-07	1.57071E-07	Cox7c/Ndufs6/Ndufv2/Ndufc2	27
2.25067E-07	2.24105E-07	Ndufa2/Cox7c/Ppp1cb/Ndufb	76
3.22741E-07	3.21361E-07	Nme1/Ndufa2/Ndufb3/Atp5p	42
3.98171E-07	3.96468E-07	Nme1/Ndufa2/Ndufb3/Atp5p	41
5.40582E-07	5.38271E-07	Nme1/Ndufa2/Ndufb3/Atp5p	49
5.40582E-07	5.38271E-07	Nme1/Ndufa2/Ndufb3/Atp5p	49
5.40582E-07	5.38271E-07	Rpl11/Rps14/Rps23/Rpl38/R	51
5.40582E-07	5.38271E-07	Nme1/Ndufa2/Ndufb3/Atp5p	52
5.40582E-07	5.38271E-07	Nme1/Ndufa2/Ndufb3/Atp5p	52
6.41723E-07	6.3898E-07	Ndufa2/Ndufb3/Ndufs6/Ndufa	21
6.41723E-07	6.3898E-07	Ndufa2/Ndufb3/Ndufs6/Ndufa	21
6.65281E-07	6.62437E-07	Nme1/Ndufa2/Ndufb3/Atp5p	42
7.92319E-07	7.88932E-07	Nme1/Ndufa2/Ndufb3/Atp5p	46
9.28583E-07	9.24613E-07	Nme1/Ndufa2/Ndufb3/Atp5p	43
9.50831E-07	9.46766E-07	Rpl11/Rps14/Rps23/Rpl38/P	68
1.01817E-06	1.01382E-06	Nme1/Ndufa2/Ndufb3/Atp5p	48

1.31025E-06	1.30465E-06	Nme1/Ndufa2/Ndufb3/Atp5p(	47
1.37174E-06	1.36587E-06	Cox7c/Ndufb3/Ndufs6/Ndufa:	30
1.7316E-06	1.7242E-06	Rps14/Rps23/Rpl38/Rps6/Rp	25
2.45488E-06	2.44438E-06	Cox7c/Ndufs6/Ndufa5/Ndufv:	29
1.89756E-05	1.88945E-05	Cox7c/Ndufs6/Ndufv2/Ndufc:	22
7.34783E-05	7.31642E-05	Nme1/Ndufa2/Hint1/Ndufb3/	55
7.37173E-05	7.34022E-05	Nme1/Ndufa2/Hint1/Ndufb3/	57
9.53814E-05	9.49736E-05	Nme1/Ndufa2/Hint1/Ndufb3/	56
0.000163884	0.000163183	Rpl11/Rps6/Gtf2h5/Rpl35/Rp	32
0.000206218	0.000205337	Ndufa2/Gabarap/Ndufb3/Ndu	78
0.000253599	0.000252515	Nme1/Ndufa2/Hint1/Ndufb3/	65
0.000297272	0.000296002	Rpl11/Rpl38/Rpl24/Rpl23a/R	19
0.000390938	0.000389267	Nme1/Ndufa2/Hint1/Ndufb3/	65
0.000390938	0.000389267	Nme1/Ndufa2/Hint1/Ndufb3/	69
0.00075413	0.000750906	Nme1/Ndufa2/Hint1/Ndufb3/	60
0.001216047	0.001210848	Srebf1/Hspd1/Mtch1/Chchd4	20
0.001494388	0.001488	Dnaja1/Srebf1/Hspd1/Mtch1/	22
0.00164173	0.001634712	Rpl11/Prpf31/Rps6/Lsm4/Gtf	104
0.001745068	0.001737609	Nme1/Ndufa2/Hint1/Ndufb3/	60
0.002210859	0.002201408	Rpl11/Rabgef1/Zdhhc2/Sreb	52
0.004265311	0.004247077	Rpl11/Rps6/Gtf2h5/Rpl35/Rp	33
0.00447389	0.004454764	Dnaja1/Pfdn2/Ero1a/Hspd1/D	32
0.00447389	0.004454764	Rpl11/Rps6/Gtf2h5/Rpl35/Rp	43
0.005115984	0.005094114	Prdx5/Ero1a/Gclc/Apex1/Txn	11
0.006240204	0.006213529	Dnaja1/Srebf1/Hspd1/Mtch1/	22
0.008882534	0.008844563	Ndufs6/Ndufv2/Ndufc2/Nduft	11
0.009318163	0.009278329	Eif3m/Eif3d/Impact/Rpl13a/E	10
0.010400661	0.010356199	Atp5pd/Atp5mf/Atp6v1f/Atp6	22
0.013079229	0.013023317	Cox7c/Cox4i1/Cox7b/Cox7a2	7
0.01348536	0.013427712	Rpl11/Rps14/Rpl38/Prpf31/R	32
0.019187668	0.019105644	Dnaja1/Pfdn2/Ero1a/Hspd1/D	54
0.022691193	0.022594192	Mt3/Txn1/Gsr/Akr1a1/Slc30a	11
0.029691696	0.029564768	Rpl35/Rpl7/Nhp2/Rsl1d1/Rpl	9
0.030863952	0.030732013	Rps14/Rpl38/Rps6/Fau/Rps2	8
0.031211096	0.031077673	Rpl11/Rps14/Rpl38/Prpf31/R	32
0.042342435	0.042161427	Nme1/Ndufa2/Ndufb3/Atp5p(	67
0.054464082	0.054231256	Rpl11/Cops9/Rpl5/Dcun1d4/I	8
0.056206651	0.055966376	Prdx5/Snca/Mt3/Prdx3/Prdx1	7
0.064495522	0.064219813	Rpl11/Rps14/Rpl38/Rps6/Rp	14
0.075339975	0.075017907	Rps3/Cdkn2d/Tpt1/Mtch2/Rp	9
0.081048973	0.0807025	Eif3m/Eif3k/Eif3d/Gigyf2/Eif1	23
0.088326333	0.087948751	Srebf1/Hspd1/Mtch1/Chchd4	24
0.094851527	0.094446051	Nme1/Ndufa2/Ndufb3/Atp5p(	65
6.84131E-25	6.27351E-25	Rpl11/Rps14/Rpl36/Rps23/R	67
2.25269E-23	2.06573E-23	Rpl11/Rps14/Rpl36/Rps23/R	69
3.27038E-23	2.99895E-23	Rpl11/Rps14/Rpl36/Rps23/R	80
4.7241E-18	4.33202E-18	Rpl11/Rpl36/Rpl23/Rpl18a/R	39
2.90027E-16	2.65956E-16	Atp5mk/Ndufa2/Cox7c/Nduft	55
2.87998E-14	2.64095E-14	Rpl11/Rpl36/Rpl23/Rpl18a/R	42
4.07523E-14	3.737E-14	Atp5mk/Ndufa2/Cox7c/Nduft	70
3.72935E-13	3.41983E-13	Ndufa2/Cox7c/Ndufb3/Ndufs	37
3.72935E-13	3.41983E-13	Ndufa2/Cox7c/Ndufb3/Ndufs	37
1.4374E-12	1.3181E-12	Ndufa2/Cox7c/Ndufb3/Ndufs	37

1.67404E-11	1.5351E-11	Rpl11/Rps14/Rpl36/Rps23/R	124
3.46615E-11	3.17848E-11	Nme1/Atp5mk/Ndufa2/Cox7c	117
3.46615E-11	3.17848E-11	Atp5mk/Ndufa2/Cox7c/Ndufb	91
6.2034E-11	5.68855E-11	Atp5mk/Ndufa2/Cox7c/Ndufb	87
1.57869E-10	1.44767E-10	Ndufa2/Ndufb3/Ndufs6/Nduf	25
1.57869E-10	1.44767E-10	Ndufa2/Ndufb3/Ndufs6/Nduf	25
1.57869E-10	1.44767E-10	Ndufa2/Ndufb3/Ndufs6/Nduf	25
1.60591E-10	1.47263E-10	Nme1/Atp5mk/Ndufa2/Cox7c	111
3.48851E-10	3.19898E-10	Rps14/Rps23/Rps4x/Rps6/R	25
4.9759E-09	4.56292E-09	Rps14/Rps23/Rps4x/Rps6/R	28
1.72736E-08	1.58399E-08	Ndufa2/Ndufb3/Ndufs6/Nduf	36
2.51445E-08	2.30576E-08	Rpl11/Rpl36/Rps23/Rpl18a/R	20
1.20139E-07	1.10168E-07	Rpl11/Rpl36/Rps23/Rps4x/R	29
4.31796E-05	3.95959E-05	Nme1/Phgdh/Rps27a/Atp5pd	56
5.26038E-05	4.82379E-05	Psma3/Psmb7/Psmb1/Psmb	10
9.19979E-05	8.43624E-05	Rps14/Rps23/Rps4x/Rps6/R	19
0.000117726	0.000107956	Atp5mk/Atp5pd/Atp5mf/Atp6	17
0.000135214	0.000123991	Cox7c/Cox4i1/Cox7b/Cox7a2	10
0.000135214	0.000123991	Cox7c/Cox4i1/Cox7b/Cox7a2	10
0.000184212	0.000168924	Atp5mk/Atp5pd/Atp5mf/Atp5	11
0.000184212	0.000168924	Atp5mk/Atp5pd/Atp5mf/Atp5	11
0.000231116	0.000211934	Rps14/Rps23/Rps4x/Rps6/R	23
0.000268408	0.000246131	Atp5pd/Atp5mf/Atp5mg/Atp5	8
0.000268408	0.000246131	Atp5pd/Atp5mf/Atp5mg/Atp5	8
0.000386625	0.000354536	Cox7c/Uqcrb/Cox4i1/Cox7b/C	12
0.002210119	0.002026688	Psma3/Psmb7/Psmb1/Psmb	25
0.002210119	0.002026688	Skp1/Psma3/Psmb7/Asb1/Ps	110
0.004516225	0.004141397	Atp5pd/Atp5mf/Atp5mg/Atp5	9
0.006445768	0.005910796	Psma3/Psmb7/Psmb1/Psmb	18
0.019200902	0.017607305	Bloc1s1/Chchd4/Ndufb7/Chc	15
0.024161943	0.022156599	Bloc1s1/Chchd4/Ndufb7/Chc	13
0.027915215	0.025598365	Psma3/Psmb7/Psmb1/Psmb	18
0.04607821	0.042253905	Atp6v1f/Atp6v1d/Atp5f1e/Atp	7
0.04607821	0.042253905	Atp5mk/Atp5pd/Atp5mf/Atp6	28
0.049944396	0.045799213	Sar1a/Sec13/Sec23a/Pdcd6/	6
0.079337516	0.072752823	Prpf31/Lsm4/Snrpd3/Snrpn/L	8
0.079337516	0.072752823	Prpf31/Lsm4/Snrpd3/Snrpn/L	8
0.079337516	0.072752823	Glud1/Rps3/Pdha1/Bloc1s1/I	31
0.085435271	0.07834449	Prpf31/Lsm4/Snrpd3/Hnrnpa	29
1.88511E-24	1.78357E-24	Rpl11/Rps14/Rpl36/Rps23/R	65
1.78031E-10	1.68441E-10	Rpl11/Rps14/Rpl36/Rps23/R	91
6.98598E-08	6.60967E-08	Rpl11/Rpl23/Rps4x/Rpl23a/R	23
0.000358018	0.000338733	Phgdh/Ndufa2/Glud1/Prdx5/N	63
0.000506142	0.000478878	Ndufa2/Ndufv2/Ndufb7/Ndufs	11
0.00224691	0.002125877	Atp5pd/Atp5mf/Atp6v1f/Atp6	23
0.002387437	0.002258835	Atp5pd/Atp5mf/Atp5f1e/Atp5	9
0.002387437	0.002258835	Atp5pd/Atp5mf/Atp5f1e/Atp5	9
0.002998789	0.002837255	Ndufa2/Ndufv2/Ndufb7/Ndufs	12
0.009788166	0.009260913	Nme1/Prpf31/Rpl35/Snrpd3/I	33
0.009788166	0.009260913	Dnaja1/Pfdn2/Naca/Dnajb6/S	20
0.015445955	0.014613938	Ndufa2/Ndufv2/Ndufb7/Cbr1/	8
0.02486856	0.023528982	Ndufa2/Ndufv2/Ndufb7/Ndufs	7
0.02486856	0.023528982	Ndufa2/Ndufv2/Ndufb7/Ndufs	7

0.024985163	0.023639304	Ndufa2/Ndufv2/Atp6v1f/Atp6	22
0.039323075	0.037204885	Prdx5/Selenof/Gsr/Prdx3/Soc	11
0.042313179	0.040033923	Ndufa2/Ndufv2/Ndufb7/Ndufs	7
0.046302545	0.043808396	Rps14/Rps9/Eef1b2/Eef1d/E	25
0.07064695	0.066841458	Ndufa2/Ndufv2/Ndufb7/Ndufs	7
0.076513961	0.072392434	Eef1b2/Eef1d/Eif3m/Eif3k/Eif	18
0.076727664	0.072594626	Eef1b2/Eef1d/Eif3m/Eif3k/Eif	20
0.085132985	0.080547183	Rps14/Rps3a1/Rpl5/Rsl1d1/I	8
0.085132985	0.080547183	Dnaja1/Rps3/Dnajb6/Snca/S	12
0.088968309	0.084175912	Rpl11/Psma3/Rpl23/Rps27a/	47
0.088968309	0.084175912	Ndufa2/Ndufv2/Ndufb7/Cbr1/	9
0.088968309	0.084175912	Eef1b2/Eef1d/Eif5a/Eef1g/Gt	7
0.09480622	0.089699357	Rps27a/Fau/Nedd8/Sumo3/S	6
0.09480622	0.089699357	Rps27a/Fau/Nedd8/Sumo3/S	6
0.000583897	0.000547268	Cttn/Snap91/Nrcam/Ezr/Pafa	147
0.000583897	0.000547268	Cttn/Snap91/Slc23a2/Camk2	174
0.000583897	0.000547268	Cttn/Snap91/Slc23a2/Camk2	171
0.000583897	0.000547268	Cttn/Snap91/Slc23a2/Camk2	174
0.001789428	0.001677172	Got1/Stx1b/Nrcam/Cacnb4/A	100
0.004462191	0.004182267	Cttn/Snap91/Nrcam/Plppr4/P	116
0.004462191	0.004182267	Cttn/Snap91/Camk2a/Nrcam	150
0.004462191	0.004182267	Cttn/Snap91/Epb41l3/Nrcam	124
0.013372313	0.012533434	Snap91/Pafah1b1/Cdh2/Nefl	57
0.017728926	0.016616747	Camk2a/Stx1b/Camk4/Plppr	138
0.018449626	0.017292236	Camk2a/Stx1b/Camk4/Plppr	138
0.023766566	0.022275631	Pafah1b1/Adora2a/Nefl/Pex5	47
0.036657441	0.03435783	Galnt11/Ramp1/Ube2j1/St6g	48
0.041361171	0.038766483	Slc23a2/Camk2a/Cacnb4/Ry	131
0.041687021	0.039071892	Nrcam/Cacnb4/Cacna2d1/Kc	37
0.059547574	0.055812009	Got1/Galnt11/Fbxw7/Kcna5/I	30
0.07130611	0.066832902	Cttn/Slc23a2/Pafah1b1/Syt2/	56
0.075972678	0.071206725	Rab3gap2/Snx18/Rab3gap1/	11
0.084113546	0.078836897	Snap91/Nrcam/Pafah1b1/Ap	124
0.096287889	0.090247513	Daam2/Abl2/Mllt3/Csnk1e/Ar	8
0.003233238	0.002998714	Snap91/Dlg3/Stx1b/Nrcam/P	128
0.003921494	0.003637047	Snap91/Kif3a/Camk2a/Mast1	158
0.003921494	0.003637047	Epb41l3/Nrcam/Apbb1/Adora	33
0.006460099	0.005991515	Snap91/Stx1b/Apbb1/Adora2	63
0.006460099	0.005991515	Snap91/Dlg3/Nrcam/Plppr4/	89
0.006460099	0.005991515	Dlg3/Nrcam/Plppr4/Epha7/St	41
0.006460099	0.005991515	Camk2a/Apbb1/Ophn1/Cdk5	65
0.006460099	0.005991515	Got1/Cttn/Snap91/Dlg3/Pafal	96
0.007986371	0.007407079	Camk2a/Apbb1/Ophn1/Cdk5	66
0.009118159	0.008456772	Hpca/Rgs8/Insr/Gria1/Kcna2	14
0.018073993	0.016762994	Cttn/Dlg3/Cacnb4/Ryr1/Cacn	57
0.018073993	0.016762994	Hpca/Rgs8/Insr/Gria1/Kcna2	13
0.018073993	0.016762994	Snap91/Camk2a/Epb41l3/Dl	124
0.030602846	0.028383064	Dlg3/Nrcam/Plppr4/Cdh2/Ep	45
0.033536005	0.031103466	Epb41l3/Adora2a/Hcn1/Hpca	24
0.03630762	0.033674041	Snap91/Camk2a/Epb41l3/Dl	114
0.036481722	0.033835515	Cttn/Cacnb4/Ryr1/Cacna2d1	39
0.036481722	0.033835515	Snap91/Stx1b/Adora2a/Napb	38
0.038117692	0.03535282	Epb41l3/Adora2a/Psd2/Psd/I	41

0.038117692	0.03535282	Cttn/Cyth3/Epb41l3/Sh2b1/E	89
0.038450671	0.035661646	Snap91/Camk2a/Epb41l3/Dl(	108
0.069733529	0.064675398	Cttn/Snap91/Dlg3/Cacnb4/R	78
0.069733529	0.064675398	Epb41l3/Dlg4/Lgi3/Kcna2/Cn	8
0.069733529	0.064675398	Dlg3/Stx1b/Trip4/Apbb1/Syt2	28
0.069733529	0.064675398	Cttn/Dlg3/Pafah1b1/Apbb1/S	58
0.07724246	0.071639667	Cacnb4/Ryr1/Cacna2d1/Mcu	17
0.083512324	0.077454746	Cttn/Dlg3/Pafah1b1/Apbb1/S	56
0.000381971	0.000358653	Slc23a2/Slc12a9/Cacnb4/Ry	80
0.001293424	0.001214464	Cacnb4/Ryr1/Kcnj9/Cacna2d	36
0.001293424	0.001214464	Cacnb4/Ryr1/Kcnj9/Cacna2d	59
0.002052291	0.001927005	Cacnb4/Ryr1/Cacna2d1/Atp2	35
0.002640294	0.002479113	Cacnb4/Ryr1/Kcnj9/Cacna2d	44
0.002640294	0.002479113	Cacnb4/Ryr1/Kcnj9/Cacna2d	44
0.003253235	0.003054635	Cacnb4/Ryr1/Cacna2d1/Mcu	30
0.004354247	0.004088435	Slc23a2/Slc12a9/Cacnb4/Ry	85
0.007398284	0.006946643	Cacnb4/Ryr1/Kcnj9/Cacna2d	56
0.013174788	0.01237051	Cacnb4/Ryr1/Kcnj9/Cacna2d	56
0.019849837	0.018638069	Slc23a2/Slc12a9/Cacnb4/Ry	87
0.019849837	0.018638069	Cacnb4/Ryr1/Kcnj9/Cacna2d	67
0.019849837	0.018638069	Slc23a2/Slc12a9/Cacnb4/Ry	86
0.030050062	0.028215605	Cacnb4/Ryr1/Kcnj9/Cacna2d	70
0.030050062	0.028215605	Cacnb4/Ryr1/Kcnj9/Cacna2d	70
0.061552193	0.057794633	Ryr1/Kcnj9/Itpr1/Hcn1/Kcnj4/	17
0.081446068	0.076474052	Tns3/Cyth3/Arhgap20/Arfgap	88
0.081446068	0.076474052	Tns3/Cyth3/Arhgap20/Arfgap	88
0.081446068	0.076474052	Slc12a9/Kcnj9/Kcna5/Hcn1/K	29
0.081446068	0.076474052	Slc23a2/Slc12a9/Cacnb4/Ry	92
0.081446068	0.076474052	Cacnb4/Ryr1/Cacna2d1/Rim	15
0.081446068	0.076474052	Kcnj9/Kcna5/Hcn1/Kcnj4/Kcr	19
0.091516001	0.085929248	Limk2/Pfkp/Nadk2/Camk2a/(	131
0.091516001	0.085929248	Limk2/Pfkp/Nadk2/Camk2a/N	108
